# The *Cryptosporidium Parvum* Transcriptome during In Vitro Development

**DOI:** 10.1371/journal.pone.0031715

**Published:** 2012-03-15

**Authors:** Mary J. Mauzy, Shinichiro Enomoto, Cheryl A. Lancto, Mitchell S. Abrahamsen, Mark S. Rutherford

**Affiliations:** Department of Veterinary and Biomedical Sciences, College of Veterinary Medicine, University of Minnesota, St. Paul, Minnesota, United States of America; Technion-Israel Institute of Technology Haifa 32000 Israel, Israel

## Abstract

Cryptosporidiosis is caused by an obligate intracellular parasite that has eluded global transcriptional or proteomic analysis of the intracellular developmental stages. The transcript abundance for 3,302 genes (87%) of the *Cryptosporidium parvum* protein coding genome was elucidated over a 72 hr infection within HCT8 cells using Real Time-PCR. The parasite had detectable transcription of all genes *in vitro* within at least one time point tested, and adjacent genes were not co-regulated. Five genes were not detected within the first 24 hr of infection, one containing two AP2 domains. The fewest genes detected were at 2 hr post infection, while 30% (985) of the genes have their highest expression at 48 and/or 72 hr. Nine expression clusters were formed over the entire 72 hr time course and indicate patterns of transcriptional increases at each of the 7 time points collected except 36 hr, including genes paralleling parasite 18S rRNA transcript levels. Clustering within only the first 24 hr of infection indicates spikes in expression at each of the 4 time points, a group paralleling 18S rRNA transcript levels, and a cluster with peaks at both 6 and 24 hr. All genes were classified into 18 functional categories, which were unequally distributed across clusters. Expression of metabolic, ribosomal and proteasome proteins did not parallel 18S rRNA levels indicating distinct biochemical profiles during developmental stage progression. Proteins involved in translation are over-represented at 6 hr, while structural proteins are over-represented at 12 hr. Standardization methods identified 107 genes with <80% at a single of its total expression at a single time point over 72 hr. This comprehensive transcriptome of the intracellular stages of *C. parvum* provides insight for understanding its complex development following parasitization of intestinal epithelial cells.

## Introduction

*Cryptosporidium* species are global contaminants of surface water and are the second leading cause of human gastrointestinal illness in the United States. Reported incidence is highest in children, yet seroprevalence is significant in all age categories [1], [2]. Due to its resistance to standard water chlorine disinfection, Cryptosporidium is a public health concern and a potential water-borne bioterrorism agent due to its low infectious dose (as low as 10 oocysts) and its ability to be stably delivered to the human population en masse [3]. Illness varies from profuse, self-limiting diarrhea to life threatening malabsorption and dehydration depending on immune status. Effective therapeutics have not been formulated because the eukaryotic parasite has a condensed genome lacking many of the traditional drug targets [4]. Most of the remaining genes have remained functionally uncharacterized, thereby limiting pharmacological targets [5], [6].

Apicomplexa are parasitic eukaryotes noted for undergoing both asexual and sexual replicative stages during their life cycle. *Cryptosporidium* spp. complete their life cycle within a single host utilizing only epithelial cells. Ingestion of an oocyst results in excystation of four sporozoites in the gastrointestinal tract. Following attachment to the host epithelial cell, the parasite resides within an intracellular but extracytoplasmic parasitophorous vacuole derived from the host cell membrane. Therein, sporozoites mature into trophozoites which then progress through asexual replication (3–4 rounds of mitosis) in ∼24 hr to form type 1 meronts that release 6–8 merozoites. These merozoites infect new epithelial cells to either repeat asexual replication generating more type 1 meronts, or through an unknown, presumably environmental trigger progress through sexual development resulting in type 2 meronts. Type 2 meronts release 4 merozoites that develop either into micro- or macro-gamonts that continue through sporogony to produce infectious oocysts that are excreted in high numbers in the feces. Sexual development of *Cryptosporidium* has been morphologically described *in vitro*, indicating the environmental switch is present, yet monolayer cultures are unable to maintain continuous propagation [7], [8].

Little is known regarding how *Cryptosporidium* regulates developmental stage progression or the different cellular requirements required by each stage. The intricate enclosure of the parasite in a parasitophorous vacuole on the host cell surface has limited high-throughput analyses of the transcriptional or proteomic repertoire of *Cryptosporidium* to the sporozoite stage [9]–[12]. Morphological characterization of the parasite is also limited because many stages are macroscopically identical even though the parasite utilizes both asexual and sexual developmental progression. To gain insight into *Cryptosporidium* biology and development, we completed a genome-wide transcriptome analysis over a 72 hr *in vitro* infection of human epithelial cells using the zoonotic species, *C. parvum*. Real time-PCR (RT-PCR) for 3,302 genes (87% of the protein coding genes) indicated that each gene has detectable transcription in at least one time point assessed. Further characterization of gene expression indicates 9 clusters over the entire 72 hr time course, while the initial asexual replication cycle (2–24 hr) produced 6 clusters with both clustering techniques distinguishing genes with coordinate patterns of expression. Additionally, we identified genes for which mRNA levels spiked at single time points, suggestive of fluctuations in transcript density dependent on the parasitic developmental stage present. Herein we describe the first comprehensive temporal transcriptome analysis of *C. parvum* during in vitro development in epithelial cells.

## Materials and Methods

### C. *parvum* parasites

*C. parvum* oocysts (Iowa strain) were purchased (Bunch Grass Farm, Drury, ID) and stored in antibiotics at 4°C for less than 2 months prior to use. Before infecting the cells, oocysts were surface sterilized by treatment with a 33% bleach (sodium hypochlorite) solution (1 ml/3×10^7^ oocysts) on ice for 7 minutes, washed profusely with Hank's buffered saline solution (HBSS), and stored in HBSS at 4°C overnight.

### *C. parvum* infection model

Human ileocecal adenocarcinoma (HCT8, ATCC CCL-244; American Type Culture Collection, Rockville, MD.) cells were cultured in a humidified incubator at 37°C in an atmosphere containing 5% CO_2_ on 22 mm glass coverslips contained in 6 well plates or 10 cm^2^ dishes until confluency was reached in 6 days. Culture media [RPMI 1640 medium supplemented with 10% fetal bovine serum (FBS), 1 mM sodium pyruvate, 50 U/ml penicillin G, 50 U/ml streptomycin, and 0.25 µg/ml amphotericin B (Fungizone) (pH 7.4)] was changed every 24–48 hours as required. After six days the culture media was replaced with Cp-Up media [RPMI 1640 media containing 10% fetal bovine serum, 15 mM HEPES, 50 mM glucose, 10 µg/ml bovine insulin, 35 µg/ml ascorbic acid, 1.0 µg/ml folic acid, 4.0 µg/ml 4-aminobenzoic acid, 2.0 µg/ml calcium pantothenate, 50 U/ml penicillin G, 50 U/ml streptomycin, and 0.25 µg/ml amphotericin B (Fungizone) (pH 7.4)] and cultured for an additional four days with media refreshment as necessary [13], [14]. Prior to infection, oocysts were warmed to room temperature and inoculated onto culture monolayers at 1.5×10^6^ oocysts/well for coverslips or 2−2.5×10^7^ oocysts/10 cm^2^ dish as previously described [14]. Following a 2 hr excystation period, the unexcysted oocysts and free sporozoites were washed from monolayers with warm HBSS and cultures were incubated in Cp-Up media for the specified time points at 37°C [7], [14]. Infection rate was 80%–90% at 24 hr depending on the batch and storage period of oocysts. Cells without infection were used as mock controls. At the specified time periods, monolayers were washed once briefly in warm HBSS and the coverslips were fixed in PBS containing 4% paraformaldehyde for 15 minutes followed by four washings in PBS and stored at 4°C. The 10 cm^2^ dishes were rinsed once in PBS then lysed in TRIzol (Invitrogen) and stored at −80°C until RNA isolation.

### Indirect immunofluorescence

*C. parvum* infected, paraformaldehyde fixed HCT8 cell coverslips were permeabilized by treating with 0.15% Triton-X 100 in PBS for 10 min. Coverslips were washed and nonspecific binding sites were blocked for 40 min by using 2.5% fetal calf serum +2.5% goat serum. Coverslips were incubated for 1 hr with either Cp-65.10, a pan monoclonal antibody that recognizes all *C. parvum* life-stages, or a secondary control antibody. Following washing, the coverslips were incubated with AlexaFluor 568 (Invitrogen). The coverslips were washed, counterstained with DAPI and mounted to slides. Photomicrographs were captured at 40X using a Nikon microscope equipped with a high resolution Zeiss Axiovert 2000, with an Axiocam attachment.

#### RNA isolation

RNA was isolated from samples stored in TRIzol following the manufacturers protocol. In brief, 0.2 ml chloroform was added per 1 ml of TRIzol used, mixed briefly and incubated for 3 minutes at room temperature. The aqueous layer was recovered after separation via centrifugation at 10,000 rpm for 20 minutes. Five hundred µl of isopropanol was added per 1 ml TRIzol used, incubated at room temperature for 10 minutes and removed by centrifugation at 10,000 rpm for 20 minutes. The resulting pellet was washed first in 75% ethanol (1 ml per 1 ml TRIzol used), then 70% ethanol (1 ml per 1 ml TRIzol used), with pelleting of RNA at 10,000 rpm after each wash. After removal of the final wash, RNA was resuspended in molecular grade water at a concentration of 1–2 µg/µl. RNA recovery and integrity was verified on a formaldehyde gel prior to use.

#### DNase treatment

DNA contamination was degraded utilizing the Turbo DNA-*free* kit (Applied Biosystems) following the manufacturers recommendations. 50 µl reactions were constructed with 30 µg of RNA and 5 µl of 10× buffer. 2 µl of Turbo DNase was incubated with the sample at 37°C for 30 minutes, followed by a 2 minute room temperature incubation with 10 µl of inactivating reagent. The inactivating reagent was removed via centrifugation at 10,000× g for 90 seconds. The RNA was collected and quantified for cDNA synthesis.

#### cDNA synthesis

To obtain enough cDNA for the whole genome transcriptome and to reduce qRT-PCR variability inherent to cDNA synthesis [15], [16], the cDNAs for each time point and replicate were made in multiple 20 µl reaction volumes, and then replicate cDNA reactions for each time point were individually pooled for each of the four separate time courses. Aliquots were stored at −80°C until used in the qPCR reactions. cDNA synthesis was accomplished using Superscript III cDNA synthesis kit (Invitrogen), with the following modifications from the manufacturers protocol. Two micrograms of DNased RNA was utilized, with 200 ng of random hexamer primers. Denaturing was done at 65°C for 5 minutes, followed by the synthesis reaction with incubations at 25°C for 10 minutes, 50°C for 60 minutes with the reaction being terminated at 85°C for 20 minutes. 1 U of recombinant RNase H (New England BioLabs) was incubated with each sample for 20 minutes at 37°C to remove template RNA. Each cDNA synthesis reaction included a negative control lacking reverse transcriptase to verify proper DNase treatment. cDNA synthesis, removal of genomic DNA contaminants (Figure S1), and DNA degradation was verified using *C. parvum* 18S ribosomal RNA primers (F-CTCCACCAACTAAGAACGGCC, R-TAGAGATTGGAGGTTGTTCCT).

#### Real Time PCR (RT-PCR)

Twenty microliter reactions were constructed using a 4 µl of template from a 1∶100 dilution of synthesized cDNA (2 µg RNA starting concentration), 0.1 µM primer pairs, and 2× AccuQuant SYBR Green SuperMix, with Low Rox (Quanta BioSciences, Inc.). RT-PCR was run on Stratagene Mx3000P or Mx3005P thermocyclers, with cycle conditions of: 1 cycle of initial denaturation (95°C for 1 minute), 45 PCR cycles (95°C for 1 minute, 58°C for 30 sec. and ramping to 60°C for 30 sec.). Amplification was followed by melt curve generation via 1 minute at 95°C ramping down to 57°C in 2 degree intervals followed by ramping back up to 95°C for 30 seconds. All primers (Table S2) were designed using Primer3(http://frodo.wi.mit.edu/primer3/) and tested on 1) *C. parvum* genomic DNA to confirm amplification and product size, and 2) HCT8 cDNA to disqualify primers amplifying host transcripts indistinguishable from parasite products. The *C. parvum* genome has few gene paralogues, and few *Cryptosporidium* genes have introns [12], [17], which allowed primer testing on *Cryptosporidium* genomic DNA. Successful primer pairs (3,302) were used to query the HCT8/*Cryptosporidium* cDNA constructed from total RNA isolated at 2, 6, 12, 24, 36, 48 and 72 hours (hr) post addition of oocysts to the cell culture. Confirmation of successful 18S rRNA amplification utilizing both the amplification and disassociation curves for each reaction allowed for inclusion of the data for subsequent analysis. For each gene of interest, the amplification and disassociation curves were also verified for consistency, and wells were individually disqualified from analysis if the C_t_ was >40, the amplification curve slope was skewed from average, or the product amplified did not have the correct melt temperature. The unprocessed RT-PCR experimental data are available on cryptogenome.umn.edu, on cryptoDB (cryptodb.org), and will also be available in the NCBI repository.

The raw fluorescence data was curve-fitted to calculate the concentration of template in each sample.

The fluorescence (F(c) as a function of the PCR cycle (c) was fitted to a four point logistic curve:
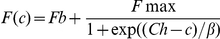
where the four parameters represent, Fb; fluorescence base, Fmax; fluorescence maximum, Ch; cycle at midpoint of the rise, and β; slope. The initial fluorescence (I0) was calculated by extrapolating back to cycle  = 0. This results in a value of transcript per 18S rRNA concentration per 2 µg of cDNA.

Hence the relative DNA concentration is obtained by normalizing the I0 of a gene to that of 18S ribosomal RNA (Table S1) as previously validated for RT-PCR [8], [18], [19]. The median gene expression was calculated using 4 independent infection time courses and standardization to the total transcript detected over the entire time course per gene.
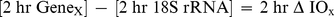


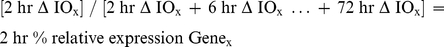



#### Functional Annotation

As described in Jensen et al [20], all genes were assigned to one of 18 functional categories based on gene annotation comparison between cryptogenome.umn.edu and cryptodb.org as well as literature review. The 18 functional categories used were (categories modified for this analysis are explained in parenthesis): DNA-associated, Interacting, Metabolism, Oxidative Stress, Phosphorylation, Protease related, Protein folding, Protein transport/modification, RNA-associated, Transcription, Translation, Transporter, Other, Hypothetical, Conserved Hypothetical, Apicomplexan conserved (genes with or without known function that are conserved across Aplicomplexa), and *Cryptosporidium* (genes of interest within *Cryptosporidium*, such as mucins, COWPs, TSPs, TRAPs and MEDLE family).

#### Computational analysis

Cluster analysis was done using the DIvisive ANAlysis Clustering (DIANA) algorithm available in the ‘cluster’ package of R [21], [22]. Data was analyzed as the percent relative expression for each gene's time course as described above using the absolute cosine angle distance metric [23]. Heatmaps were visualized using the R package ‘gplots’ [24].

## Results

Our understanding of *Cryptosporidium* infection has remained quite limited due to the parasite's tight association with the host intestinal epithelial cell. We used Real Time (RT)-PCR to describe the transcriptional profile of 3,302 *C. parvum* genes during a 72 hr *in vitro* infection time course using the epithelial cell line, HCT8, resulting in the first comprehensive transcriptome for this obligate intracellular parasite. Four thousand thirty-three primer pairs were tested, and 731 were rejected based on irregularity in amplification efficiency or because they amplified host cell cDNA. Four independent in vitro infections were prepared and assessed by RT- PCR. Gene expression ranged from 10^−10^ to 0.5 transcripts relative to *C. parvum* 18S rRNA, while the median expression relative to rRNA across the time course did not change (Table 1). Approximately two-thirds of the transcripts identified were expressed at all time points, albeit at different relative transcript levels.

**Table 1:** Transcriptome Summary Statistics.

	Time point						
	2 hr	6 hr	12 hr	24 hr	36 hr	48 hr	72 hr
Number genes detected	2532	2868	3229	3294	3300	3301	3301
% genome (% dataset) detected	65.2 (76.7)	73.8 (86.9)	83.1 (97.8)	84.8 (99.8)	84.9 (99.94)	84.9 (99.97)	84.9 (99.97)
18S ribosomal average C_t_ (SD)	18.9 (1.2)	17.4 (1.1)	16.6 (1.2)	14.8 (1.0)	14.6 (1.0)	15.0 (1.1)	16.0 (1.2)
Sum Log_10_ Expression	−14843.69	−16539.95	−18794.9	−18786.91	−18375.61	−18196.81	−19070.31
Mean Log_10_ (SD)	−5.82 (1.62)	−5.77 (1.65)	−5.82 (1.61)	−5.70 (1.51)	−5.57 (1.38)	−5.51 (1.28)	−5.78 (1.30)
Median Log_10_	−6	−6	−6	−5.7	−5.7	−5.7	−6
Minimum Log_10_ Expression	−9.97	−10.93	−11.95	−10.75	−10.22	−9.52	−9.22
Maximum Log_10_ Expression	−1.25	−0.47	−1.52	−1.19	−0.98	−1.03	−0.86
Genes with >80% expression	0	2	31	1	1	33	39

For all Log_10_ calculations zero values were removed.

### Morphological description of C. parvum developmental stage progression

**Figure 1:**
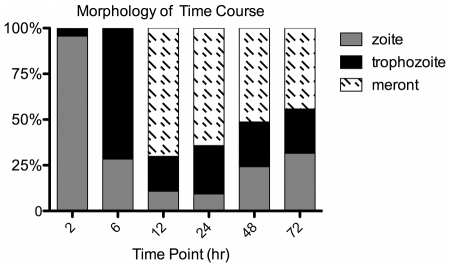
Quantification of Parasite Stage Progression. HCT-8 coverslips were infected and stained with pan-specific antibody to quantify *C. parvum* infection. >300 parasites were counted on 15–30 frames for each of four replicates for each time point. The average for each of the 3 stages of parasite determined was converted into a percentage of the total parasite detected per time point. zoite  =  sporozoite/merozoite, trophozoite  =  all trophozoites (size of stained parasite was > zoite but <merozoite), meront  =  all parasites staining > trophozoite.

In agreement with prior data [25], removal of unexcysted oocysts after 2 hr incubation with the host cell culture layer results in relatively homogenous parasite morphology during the first 24 hr of infection (Figure 1). At 2 hr sporozoites have attached and appear as small, dense structures atop the host cell. By 6 hr the sporozoites have expanded into trophozoites, which are medium sized spheres atop the host cell. Thereafter, immature (12 hr) and mature (24 hr) meront development is observed. These appear as larger staining spheres, with merozoites observed within more mature meronts. However, once a full cycle of asexual replication has been completed (between 24–36 hr p.i.) and merozoite reinfection has begun, the relative homogeneity previously observed is diminished as the various parasitic stages progress at different time scales. Morphological description is also complicated by the occurrence of sexual replication within type 2 meronts, which are not distinguishable from asexually derived (type 1) meronts by light microscopy.

### Gene Expression is not Correlated to Chromosomal Location

All 3,302 genes analyzed were detectable within at least one time point of the 72 hr time course. The transcriptional density across each time point follows a normal distribution, with an increase in detectable genes (non-zero values) occurring from 6, 12, and 24 hr, coinciding with a decrease in *C. parvum* 18S rRNA C_t_ values (Table 1). *C. parvum* 18S rRNA C_t_ values increase slightly after 36 hr. However, there is no decline in transcript abundance measured as either total number of detectable genes or total mRNA content at 72 hr (Table 1, Figure S2).

**Figure 2:**
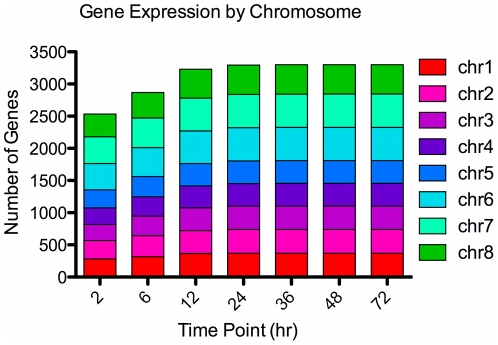
Expression by Chromosome. For each time point, the number of genes with detectable expression (non-zero) was quantified by chromosomal location.

**Figure 3:**
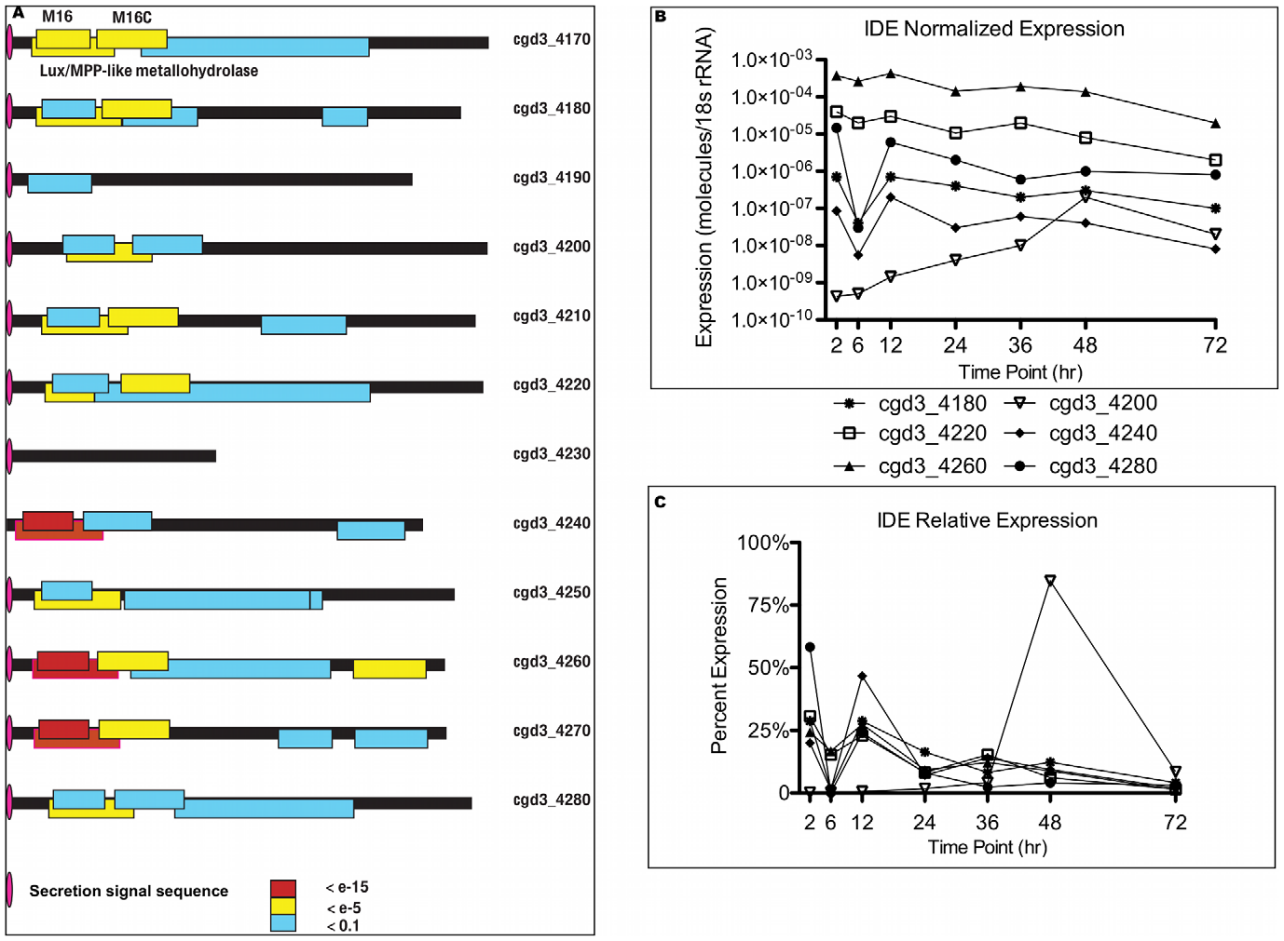
Chromosome 3 Peptidase Loci are not Co-expressed. Twelve genes, including 11 peptidase genes are present in tandem on chromosome 3. (**A**) The protease motifs were identified and color coded based on the conservation level. (**B**) RT-PCR for each gene was normalized to *C. parvum* 18S rRNA and the median for four biological replicates for the 7 time point time course is shown. (**C**) The normalized expression was then averaged by each gene's total expression for all 7 time points.

Previous work had suggested a transcriptional cascade of gene regulation [26], but our data suggests *C. parvum* utilizes a complex transcriptional regulatory system. First, a single chromosome exhibiting prominent transcription is not observed at any time point regardless of transcript quantity (Figure 2). Second, by examining the quantity of transcript, tandem genes and divergent genes do not have correlated expression as a norm (data not shown). Third, genes within a cluster (see below) do not map to the same chromosomal location. Complex transcriptional regulation was further verified by examining the tandem cluster of peptidases found on chromosome 3 (Figure 3). *C. parvum* has a large number of M16 family proteases and their kin. Four genes contain a full protease catalytic domain and 15 degenerate genes appear to be related to M16 enzymes. The most notable are the tandem Insulin Degrading Enzymes (IDEs) found near the telomere of chromosome 3. Using relaxed criteria to identify the metallohydrolase domain, the cluster contained 12 consecutive protease genes, cgd3_4170 through cgd3_4280, spanning a 57 kb region. This grouping of genes appears to be formed by gene duplication. All of these IDE genes were expressed, but at vastly different levels (Figure 3B) and with different expression patterns (Figure 3C). For example, cgd3_4240 was expressed at low levels early in the infection with a sharp peak in transcript levels at 48 hr, while all others showed greater expression throughout the time course. In contrast, cgd3_4260 was constitutively expressed at much higher levels throughout the examined time course. The 12 IDE genes sorted into five different expression pattern clusters, further confirming that *C. parvum* gene expression is independent of chromosome location, even for co-localized gene families.

### Expression profiling of an in vitro 72 hour time course

**Figure 4:**
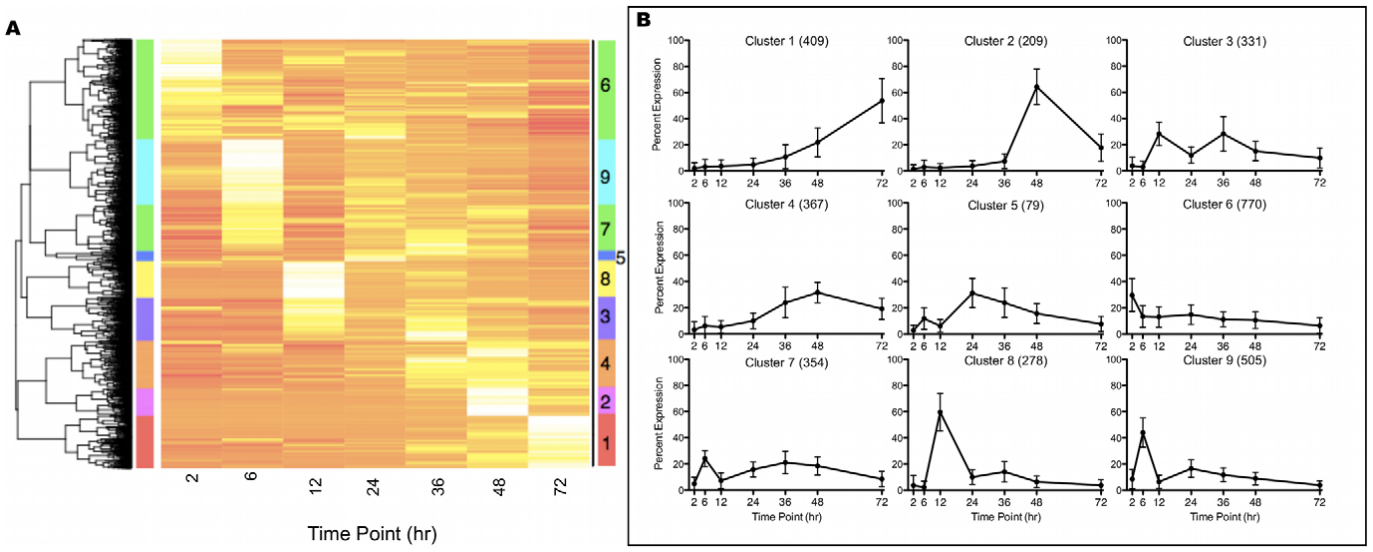
*C. parvum* Transcriptome 2–72 hr Relative Expression Cluster Analysis. The DIANA algorithm was used to cluster each gene's relative expression using the absolute cosign distance metric. For the heatmap each gene is represented in one row and each column represents the percent expression at one time point. (**A**) The relative expression (%) was determined for each gene's median normalized value over the 7 time point, 72 hr time course (3302 genes). Nine clusters (1–9) are present with the dendrogram distance cutoff of 100 as indicated with the colored bar to the right of the heatmap. (**B**) For the 72 hr time course, each clusters' average expression (+/− SD) is graphed, with the number of genes found in each cluster within the parentheses.

**Figure 5:**
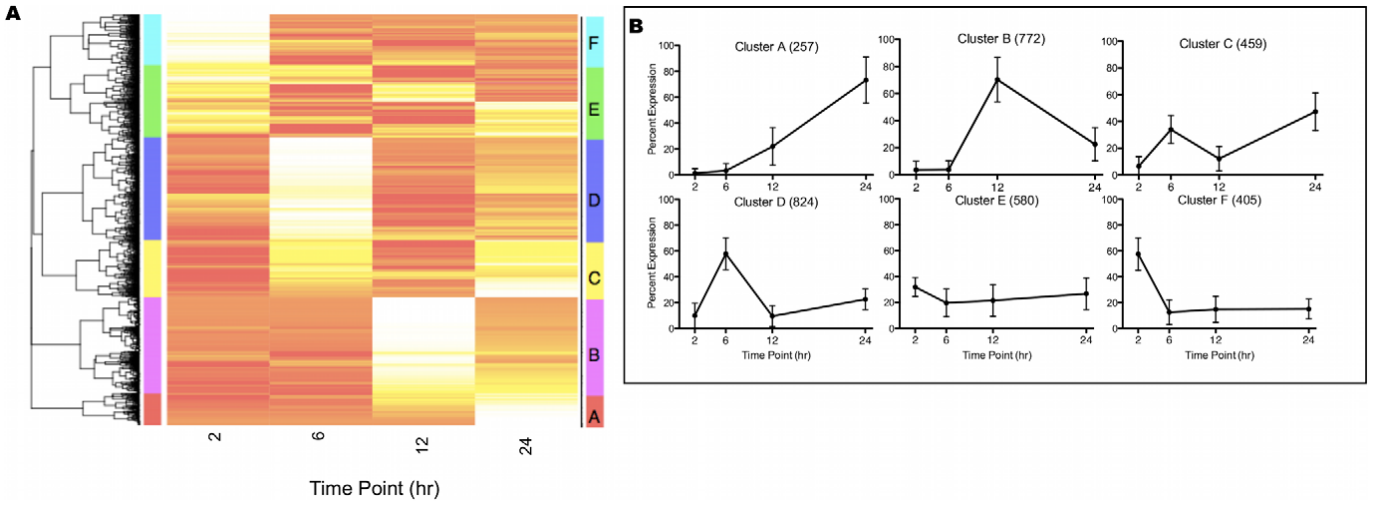
Distribution of Genes Across Clusters. The percentage of each of 13 functional categories was determined across all clusters. Five categories were omitted: Apicomplexan, *Cryptosporidium*, Oxidative Stress, Hypothetical, and Conserved Hypothetical. Chi-square testing was used to determine any significance in the distribution of the number of genes across clusters using the null hypothesis that the functional classifications would be equally distributed across clusters. Data was using the 2–72 hr (A) and 2–24 hr (B) data sets. Chi-square significance: * = <0.05, ** = <0.01, *** = <0.001, **** = <0.0001.

RT-PCR provides a quantitative evaluation of each gene transcript present, but to delineate patterns of gene expression we focused on the relative change of gene expression rather than the absolute transcript abundance during the 72 hr time period examined. To determine which genes have similar patterns of expression, we normalized to *C. parvum* 18S rRNA and computed a relative expression for each gene by dividing its expression at each time point by the sum of gene expression for all 7 time points. The expression profile is a vector of 7 relative percentage values, and all genes were clustered to identify shared patterns of expression using the Divisive Analysis (DIANA) algorithm (Figure 4) [21], [22], [27]. Utilizing the distance cutoff of 100, 9 clusters were defined for genes expressed over the 72 hr infection period. Based on putative functional annotations and protein domain similarities, we classified each gene into 1 of 18 categories [20]. Examination of the distribution of functional categories was assessed between clusters (Table 2, Figure 5A). All functional categories except the 2–24 hr clustering ‘Other’ were unequally distributed between clusters at a significance level <0.05 (Figure 5A). In addition, genes annotated as apicomplexan, *Cryptosporidium*, hypothetical or conserved hypothetical proteins were randomly distributed across all clusters (data not shown).

**Table 2:** Functional Categories Distribution across 2-72 hr.

Cluster	1	2	3	4	5	6	7	8	9	Total
**Apicomplexan**	13	5	5	3	0	17	10	13	9	75
***Cryptosporidium***	11	14	7	2	0	13	5	10	4	66
**DNA-Associated**	24	6	12	49	9	44	30	6	33	213
**RNA-Associated**	12	4	5	6	1	70	15	6	35	154
**Transcription**	2	1	2	5	3	27	7	2	16	65
**Translation**	2	2	5	3	4	48	9	2	94	169
**Protease Related**	14	4	10	22	2	26	29	8	22	137
**Protein Folding**	2	1	0	0	1	6	5	1	12	28
**Protein Transport/Modification**	18	8	11	13	6	19	40	9	28	152
**Transporter**	10	7	12	11	3	45	12	2	15	117
**Structural**	3	0	14	9	3	9	1	8	3	50
**Interacting**	29	8	28	29	6	73	29	16	36	254
**Other**	26	14	22	25	2	43	24	9	22	187
**Metabolism**	23	11	9	23	2	32	18	13	28	159
**Oxidative Stress**	2	1	0	0	0	3	0	0	3	9
**Phosphorylation**	22	12	15	20	3	29	14	15	8	138
**Conserved Hypothetical**	17	9	9	9	4	47	10	7	19	131
**Hypothetical**	179	102	165	138	30	219	96	151	118	1198
**total**	409	209	331	367	79	770	354	278	505	3302

Cluster 7 represented an expression pattern following the normalizing gene's expression (*C. parvum* 18S rRNA). Cluster 7 had a high proportion of genes associated with protein transport or modification, but also contained multiple genes for DNA-associated proteins involved in replication, including 5 termination factors and 4 histone-modifying enzymes. Genes with a high expression within this cluster were ethanolamine-phosphatecytidyl transferase and NADP-dependent alcohol dehydrogenase.

Five clusters contained genes with a defined peak in expression at a single time point. Cluster 6 contained the largest number of genes, and expression peaked at 2 hr followed by a lower expression at the remaining time points (Figure 4B). The largest functional category expressed in this cluster was that of RNA-associated genes. Notably, 22 helicases, including Dbp8p-like helicase, Sgs1, Has1p, Rok1p-family helicase, DRS1, Mtr4p-like, and 8 involved in ribosome biogenesis, and 11 spliceosome proteins, including PRP22, splicing factor 3b, FBP21, and U5snRNP had their highest expression at 2 hr. Many transcription-related genes were found in this cluster, including 11 polymerase-associated proteins. Additionally, 19 ribosome and 13 tRNA-associated transcripts were present. We also observed high expression at 2 hr for 6 H^+^ transporters, 3 mitosis-associated genes (XPMC2, DIM1 and Ard1) and 8 DNA replication-associated genes (including Separin/esp1, SMC1, UVR8-like protein with RCC1 domains, and Cbf5p). Within this cluster thymidine kinase, phosphatidate cytidylyltransferase, initiation factor 3 subunit 8, and 60S ribosomal subunit L7A had the highest magnitude of expression.

As shown in Figure 4B, genes in cluster 9 had a defined peak in expression at 6 hr with a small rise again at 24 hr. The largest functional category of genes with this expression pattern was translation-associated transcripts that included 56 ribosome and 17 tRNA associated genes. Genes with high expression within this cluster included a mucin, Rad23, 40S ribosomal proteins S3 and S19, 60S ribosomal protein L8, and elongation factor 1-gamma.

Clusters 3 and 8 both contained genes with spikes in expression at 12 hr, with cluster 8 having a higher average percentage of expression at 12 hr and little expression thereafter. In contrast, genes in cluster 3 showed almost equal peaks of expression at 12 and 36 hr. For both clusters, the functional class most highly represented are structural genes, in particular cytoskeletal components that include two unique myosins, four actin, three kinesin, and two tubulin gene transcripts. However, cluster 8 contained many genes associated with metabolism, whereas cluster 3 contained transcripts for a variety of predicted transporter genes.

Although there was not a cluster containing genes with peak expression exclusively at 24 hr, genes in cluster 5 showed a bimodal expression pattern that included a small peak at 6 hr and a larger peak at 24 hr. This cluster contained the fewest genes overall, and the largest functional categories represented were structural protein, transcription-related, and DNA-associated genes. The replication factors minichromosome maintenance complex component (MCM) 6 and MCM 7 were found in this cluster along with the gene for Structural Maintenance of Chromosomes (SMC) 2 and the DNA replication-related chl12 gene, which has one of the highest transcript copy numbers within this cluster.

Three clusters (1, 2, and 4) indicate a pattern of increased expression within the last three time points, which correspond to time periods that sexual replication stages are observed. Cluster 2 represented genes with a tightly defined spike in transcript levels at 48 hr, while cluster 1 contained genes in which transcript levels peaked at 72 hr. Cluster 4 contained genes with little or no expression until 36 hr. Afterwards, expression of these genes increased at 48 hr and remained high with a slight drop off at 72 hr. The largest functional category within cluster 4 was DNA-associated genes, including ten DNA repair genes and eleven replication-related genes. Genes encoding enzymes associated with metabolism were the highest represented functional group within clusters 1 and 2, with cluster 2 containing transcripts for three amino acid transporters and the DNA-associated genes ARD1 and topoisomerase II, as well as four COWP genes. Two genes in this cluster, COWP1 and CpCCP2, exhibited high transcript numbers. A spike in expression for DNA-associated genes including DMC1, SMC1 and SMC3 was observed at 72 hr in cluster 1. A similar spike was observed for three cyclin homologues and the remaining four COWP genes, with COWP8 having a highly detected copy number.

### Expression profiling of the asexual replication cycle (2–24 hr)

**Figure 6:**
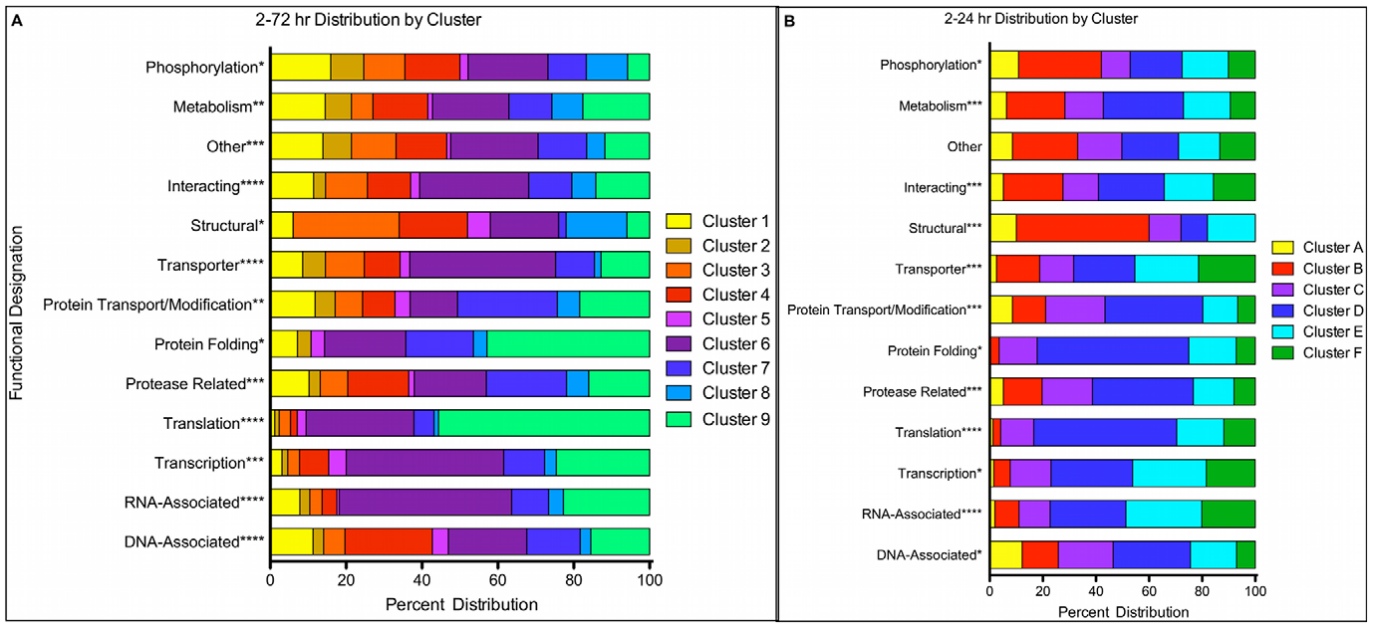
*C. parvum* Transcriptome 2–24 hr Relative Expression Cluster Analysis. The DIANA algorithm was used to cluster each gene's relative expression using the absolute cosign distance metric. For the heatmap each gene is represented in one row and each column represents the percent expression at one time point. (**A**)The relative expression (%) was determined for each gene's median normalized value over the first 24 hr of the time course, which includes 4 time points (3297 genes). Six clusters (A–F) of genes are present with a dendrogram distance cutoff of 1, as depicted with the color bar to the right of the heatmap. (**B**) The average gene expression per cluster (+/− SD) using the first 4 time points (24 hr) is plotted with the number of genes found in each cluster within the ().

Morphological homogeneity is highest (Figure 1) in the first 24 hr of infection because sexual replication stages do not appear in culture until after 24 hr [28]. We further focused analyses on the first 24 hr to identify genes expressed by asexual developmental stages. The DIANA algorithm distinguished six clusters (divisive coefficient  = 0.98) using the dendrogram distance cutoff of 1 (Figure 6) [24], [27]. One cluster (cluster E) contained genes mirroring 18S rRNA transcript levels and likely represented housekeeping functions for all asexual stages. Interestingly, 30% of the genes in cluster E were hypothetical proteins (Table 3), suggesting we know little of even the basic biochemistry for this pathogen. Four clusters represented genes with a spike in expression at each of the four time points, while cluster C contained genes with a bimodal expression pattern at 6 and 24 hr. Across the 6 clusters, almost 50% of the genes were assigned between cluster D (6 hr spiked expression pattern) and cluster B (12 hr spiked expression), while cluster A (24 hr maximum expression) had the fewest genes. Five genes were not included in this cluster analysis because they had no detectable expression within the first 24 hr (Table 4), including cgd4_1110, which is a hypothetical gene with a predicted AP2 domain at both the N- and C-termini.

**Table 3:** Functional Categories Distribution across 2-24 hr.

Cluster	A	B	C	D	E	F	Total
**Apicomplexan**	9	20	8	16	12	10	75
**Cryptosporidium**	8	17	5	7	15	12	64
**DNA-Associated**	26	30	45	61	37	16	215
**RNA-Associated**	3	14	17	43	43	29	149
**Transcription**	1	3	9	20	18	10	61
**Translation**	2	5	21	92	30	21	171
**Protease Related**	7	20	26	52	21	11	137
**Protein Folding**	0	1	4	16	5	2	28
**Protein Transport/Modification**	13	19	34	56	20	10	152
**Transporter**	3	19	15	27	28	25	117
**Structural**	5	25	6	5	9	0	50
**Interacting**	13	57	34	63	48	40	255
**Other**	16	46	31	40	29	27	189
**Metabolism**	10	35	23	49	28	15	160
**Oxidative Stress**	0	0	0	4	4	1	9
**Phosphorylation**	15	43	15	27	24	14	138
**Conserved Hypothetical**	12	18	21	30	27	24	132
**Hypothetical**	114	400	145	216	182	138	1195
**total**	257	772	459	824	580	405	3297

**Table 4:** Genes with no Detectable Expression in the first 24 hr.

Gene ID	2–24 hr expression	48 hr (72 hr) expression	Annotation
cgd3_90	none	45% (54%)	Hypothetical protein with signal peptide
cgd3_720	none	93% (7%)	Very large probable mucin, 11700 aa long protein with signal peptide and pronounced Thr repeat (308 aa long)
cgd7_5140	none	73% (27%)	A surface protein with 2 conserved cysteines
cgd4_1110	none	37% (62%)	Uncharacterized protein
cgd3_1540	none	9% (90%)	Large protein with signal peptide. cysteine-rich, threonine-rich, possible mucin [CpPOWP3 (29)

This focused analysis revealed additional genes that exhibited high expression at 2 hr within the first 24 hr, but showed increased expression at later time points 36, 48 or 72 hr when analyzing the entire time course. Using this approach we found 96 genes differing within the 2 hr clusters, of which 48% are annotated as hypothetical proteins. At 2 hr post-infection, transcripts may represent newly transcribed genes or may have been carried forward from sporozoite packaging within the oocyst. Further, disregarding expression data collected at 36–72 hr and focusing on the first round of asexual development (2–24 hr) resulted in differing gene expression patterns compared to analysis with the entire time course (2–72 hr). With this asexual specific analysis (Figure 5B), the 2 hr time point now showed the highest relative expression of genes CpCCp1, COWP1, COWP2, glutathioneperoxidase, triosephosphate isomerase, Cyclin A, PRP2, and RNA polymerase II B6 and B10 subunits.

Similarly, at 6 hr we found a 153 gene difference compared to the 72 hr clustering, with only 35% being hypothetical. Transcripts for two mucins, the DNA polymerase epsilon subunit, MUS81 endonuclease, CDC45, a chromatin protein similar to BDF1, 13 metabolism related genes, HSP40, RAB11, PRP5, and a FLX1-like transporter all peaked at 6 hr, which corresponds to the time point when a high percentage of trophozoites were present.

An additional 223 genes show an expression peak at 12 hr, half of which were annotated as hypothetical proteins. Three *Cryptosporidium* specific genes, TRAPC1, TSP1, and a mucin, were all highly expressed. Additionally, DMC1, topoisomerase VIA, MCM10, endonulcease III, histone H4, and 14 additional metabolism related genes had increased expression at this time point.

At 24 hr we observed 44 genes varying from the previous 72 hr cluster scheme, 39% of which were annotated as hypothetical. Genes with a predicted function included TSP6, MCM6 and 7, SMC2 and 4, RNA polymerase II CTD/NL1 interacting protein, and translation initiation factor if-2B beta.

The largest difference found was in the genes within this analysis that have a bimodal expression at 6 and 24 hr. Four hundred-two genes found within cluster C are not found in cluster 5, with 35% being hypothetical. This group of genes, found in cluster C (Figure 5), included eight Apicomplexan genes, including lipin2, ORC subunit 5, and cgd7_4100 (membrane protein), and five *Cryptosporidium* related genes: TSP10, TSP4, mucin and a COWP. The largest category represented was that of DNA-associated genes, such as repC1, DNA polymerase theta, delta 1 and 2, MCM2 and 3, bub1, ORC1 and 2, PDNA, HDAC2, topoisomerase I, and DNAse 1. In addition, cluster C included CHC1 clathrin heavy chain, as well as the Adaptin AP complex subunits alpha, beta, mu and gamma.

### Spikes in Gene Expression

**Figure 7:**
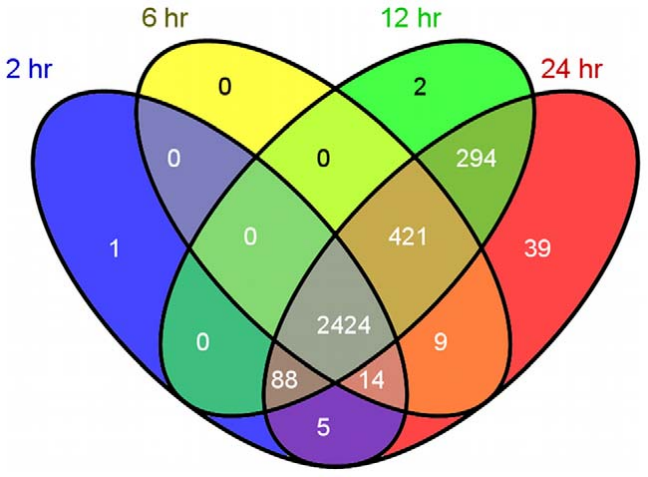
Unique Asexual Stage Gene Expression. Gene transcription fluctuates during the infection time course. Most genes are detected at all 4 time points within the first 24 hours of infection (asexual replication). Gene lists were constructed containing the gene IDs for all genes with detectable transcription (non-zero) at 2, 6, 12, and 24 hr time points. These lists were compared to show the number of genes with overlapping expression at all combination of the four time points used. Blue = 2 hr, yellow = 6 hr, green = 12 hr, and red = 24 hr [72].

Seventy-four percent (2,424) of the genes detected during the first 24 hr of infection have detectable expression at all four time points (Figure 7). Of the genes with variable detection, 42 were identified at only one time point: one at 2 hr, two at 12 hr, and thirty-nine at 24 hr post-infection (Figure 7). This patterning further suggests that the number of transcripts detected is dependent on the parasite stage present and changes as the parasite develops. To ascertain large shifts in transcription across the entire time course used, we identified transcripts with >80% of its total expression within a single time point over the 72 hr time course, indicative of at least a 4-fold induction of transcription. This analysis resulted in 107 time-point specific genes, 72 of which show >80% of their total expression at either the 48 or 72 hr time points only (Table S3). The residual 20% expression for these 72 genes fell primarily in either the 48 or 72 hr time point, suggesting they are expressed by sexual developmental stages only.

An unexpectedly large number (59%) of the 107 genes with >80% expression at one time point were annotated as hypothetical genes (Table S3). The highest number of remaining genes were categorized as *Cryptosporidium*-specific, interacting, and phosphorylation-related genes (Table S3). Genes having a functional annotation included the Rab GDP dissociation inhibitor (cgd7_4740) spiking at 36 hr and phosphoglucomutase and two myosins, which spiked at 12 hr. At 48 hr we observed a spike in expression of seven *Cryptosporidium* related genes, including five mucins, CpCCP3, and COWP6. Also of note at 48 hr were genes encoding translation initiation factor 3 subunit 10 and a predicted oocyst wall protein (CpPOWP) 4 [29]. Genes represented at 72 hr included COWP7, CpPOWP3 [29], DMC1, pyruvate decarboxylase, and cyclin A.

## Discussion

We applied RT-PCR and cluster analyses to describe changes in the *C. parvum* transcriptome during the first 72 hr *in vitr*o infection of intestinal epithelial cells. The time points assayed (2, 6, 12, 24, 36, 48, and 72 hr) were predicted to be integral transitional states during *in vitro* parasite development based on our morphological assessment [25] (Figure 1) and other reports [30]. Transcripts were measured using four independent culture replicates. We detected transcripts for all 3,302 genes tested (Table 1). This detection rate was surprising considering that the intensely studied genome of *Sachromyces cerevisiae* contained 12.3% “dubious” ORFs with no transcripts detected [31]. When transcript abundance for each gene was standardized as a percentage of its total transcript abundance across all time points, we found 107 genes with spikes in expression of >80% at a single time point.

The dataset produced 9 clusters based on relative level of expression in which the 18 functional categories were distributed unequally. The representation of functional categories of genes at each time point suggested a choreographed developmental program for this parasite during infection. At 2 hr post infection, sporozoites expressed proportionately more RNA-associated, transcription and transporter genes. At 6 hr trophozoites expressed more translation-related genes, at 12 hr meronts exhibited more transcripts for structural protein-related genes, transporters, and enzymes required for metabolism, while the 24 hr meronts had an over-representation of DNA-associated genes. At 36 hr merozoites have reinfected new epithelial cells for another round of replication, and DNA-associated genes were again overrepresented. However, by 48 and 72 hr post-infection, metabolic enzymes were the overrepresented functional category. Comprehensively, our transcriptome suggests a cascade of gene expression consistent with unique biologies for each developmental stage.

### Sporozoites: preparation for parasitism and transcription

At 2 hr, the sporozoite has attached to the host cell and appears to be ramping up transcription in preparation for cellular parasitization [32]. In our analysis, transcripts for 2,532 genes had detectable expression at 2 hr, representing the smallest population out of the 7 time points examined, as might be predicted based on the assumption that sporozoites are quiescent within the oocyst. Previously published EST data [9]–[12], although not quantitative, indicates <1,000 non-repetitive transcripts which suggests that host attachment triggers a robust gene expression profile as the parasite begins to capture nutrients from the host cell. The freshly excysted sporozoite is a transient state that is primed for translation, based on the high amount of ribosomal proteins found in both EST and proteomic data [9]–[11]. Excystation itself increases expression of ribosomal protein genes, yet our data showed that additional translational machinery is not highly transcribed until after 12 hr [33], even though many transcripts for previously undetected genes appear at 2 hr. This illustrates that *Cryptosporidium* sporozoites are packaged with all components needed to attach, commence parasitophorous vacuole formation, and initiate transcription following attachment to the host cell. This rapid increase in transcription following attachment is similar to the ring stage of *Plasmodium* and tachyzoite replication in *Toxoplasma* [34]–[36]. These events have not been captured in prior *Cryptosporidium* EST and proteome analyses and represent the power in examining a global transcriptome approach.

The majority (96%) of the genes detected in the first 24 hr were expressed at all four time points (Figure 7), and 16% had elevated expression at 2 hr. However, transcripts for cgd8_730, a possible Apicomplexan protein, were only detected at 2 hr within the first 24 hr. The *P. falciparum* homologue for cgd8_730, PFD0910w, shows highest expression during gametogenesis which would suggest that cgd8_730 in sporozoites is a remnant transcript that was expressed during sexual development [37]. Toward this end, we observed very low cgd8_730 transcripts levels at 2 hr, but transcripts increased >1,000-fold at 48 and 72 hr, consistent with expression during gametogenesis. The 2 hr transcriptome included many transporters, DNA-associated proteins, and transcription-related genes, which peaked at 2 hr and were turned off or greatly down-regulated in proceeding time points. This is surprising since many of these genes might be expected to show constitutive expression over the course of an infection. The possibility that the observed down-regulation is an artifact due to normalization must be considered. However, normalization using parasite 18S rRNA gene quantity had been previously established under these culture conditions to correlate with parasite density [7], [8]. Even so, we observed that ribosomal components decreased in expression at later time points (72 hr) despite a lack of corresponding decrease in total number of *C. parvum* transcripts or parasite numbers detected (Table 1). Because individual parasite stages cannot be readily purified away from the parasitized host cell, the measured RNA concentration used to initiate cDNA synthesis is largely derived from the host cell RNA and thus this phenomena may also be related to host cell growth or demise during infection.

The *Cryptosporidium* genome lacks genes for energy synthesis beyond glycolysis. Therefore, expression of transporters was expected to initiate early following attachment. Fewer than half of the annotated transporters had been detected previously in freshly excysted sporozoites [4], [6], [9], [12]. Our analyses found that almost 20% of the genes annotated as transporters, including most of the sugar and nucleotide-like transporters, had high expression at 2 hr. Additionally, two putative amino acid transporters (cgd3_470 and cgd5_1630) had 50% of their relative expression at this time point. This is consistent with the parasite needing quick access to the host cell contents for nutrients necessary for developmental stage progression.

The *Cryptosporidium* specific genes TSP8 (cgd6_780) and CpCCP1(cgd7_1730) were expressed at high levels at 2 hr. TSP8 proteins are abundant in sporozoites and type 1 merozoites [38]. Our data indicated that 79% of TSP8's relative expression was at 2 hr, suggesting the protein is made during the first round of asexual development following sporozoite invasion and either was not required for later asexual divisions or represents a very stable protein. In apparent contrast to prior work showing that the CpCCP1 protein was abundant in sporozoites and was embedded throughout the circumference of the parasitophorous vacuole during intracellular development [39], we found CpCCP1 had >90% relative transcript expression in the combined 48 and 72 hr time points. This apparent conflict can be explained if the CpCCP1 protein, as opposed to new gene transcription, was packaged in oocysts and carried forward rather than induced de novo gene expression following excystation. It may also suggest that CpCCP1 is more of a generic protein important for parasitophorous vacuole integrity throughout the entirety of the infection rather than being used only by sporozoites during initial host cell invasion.

### Trophozoites: Protein synthesis and degradation

The largest number of genes had peak expression at 6 hr. Trophozoites are the major morphological stage present at this time and our data showed an expression increase of genes required for protein translation. In addition, we observed increased transcription for genes encoding proteins required for folding, modification and transport of the newly translated polypeptides. This was supported with the detection of 15 chaperone proteins, including many heat shock proteins (HSPs). Additionally, the trophozoite is transcribing almost the entire complement of proteasome genes which could be used to recycle either proteins no longer needed by the sporozoite or newly acquired host proteins to facilitate new protein synthesis (Figure S3).

In conjunction with parasite preparation for new protein synthesis, trophozoites are enlarging in preparation for mitosis and division into merozoites through schizogony [30], [40]. Nutrient availability, protein synthesis, and ribosome biogenesis are major factors needed for mitosis in eukaryotic organisms [41]–[43]. Our analyses showed that all but three of the parasite ribosomal and tRNA synthesis genes peaked in expression at 6 hr (Figure S3). The exceptions, 60S ribosome L7A (cgd3_760), tRNA related adenyl cyclase associated protein (cgd5_440), and L35 (cgd3_830), had peaks at 12 hr or bimodally at 12 and 36 hr, respectively. Schizogony involves multiple rounds of DNA division followed by daughter cell budding. Our data clearly supported that the trophozoite stage was preparing for chromosomal duplication and separation with the expression of transcripts for CDC45, all replication licensing factors (MCM), replication factors C2, C5 and repA1, replication protein A1 large subunit, origin recognition complex (ORC)1 and ORC2, and the spindle checkpoint gene bub1. *Plasmodium* lacks orthologs for CDC45 and Bub1, but ORC1 transcription is highest within the schizont stage, with protein detected in the trophozoite [44]–[46]. ORC proteins have not been detected in the *Cryptosporidium* sporozoite proteomes, but we observe increased expression of all ORCs at 24 hr (Figure S3). This suggests the merozoite may be prepackaging the transcripts necessary to proceed with DNA-replication following attachment.

### Immature (12 hr) Meronts: Dividing nuclei

It has been previously reported in other Apicomplexa that nuclear division occurs within the immature type 1 meront and is followed by cytokinesis, resulting in the formation of the mature type 1 meront [47]. The 12 hr transcriptome supports a similar division process for *C. parvum.* Transcripts for the H2B, H3 and H4 histones were highly expressed at 12 hr. Acetyltransferase GCN5, which activates transcription of genes required for DNA replication and movement from G1/S based on nutrient availability, MCM10, which regulates DNA replication initiation in fission and budding yeast [48], and PMS1, which functions in DNA repair, also showed maximum expression at 12 hr [49].

The immature *C. parvum* meront transcriptome also included genes for structural components found to precede zoite formation in other Apicomplexa, such as the apical complex proteins that include components of the microneme and rhoptry, as well as the formation of the Inner Membrane Complex [50]-[52]. These complexes are composed of a variety of membrane skeletal proteins, such as actins, myosins, tubulins, and kinesins. Within this 12 hr cluster were five *Cryptosporidium*-specific genes, TSP3, TSP11, TSP12, TRAP-C1, and Cp15/60, confirming previous transcription data [14], [53]. TSP3, TSP11, and TRAP-C1 have been documented as microneme-associated proteins, further suggesting the parasite is preparing for the future separation of daughter cells [9]. Cp15/60 (cgd7_4540) and cgd3_1570 are previously reported sporozoite antigens, but their high transcript levels at 12 hr in our culture system suggested they are also components of merozoites.

**Figure 8:**
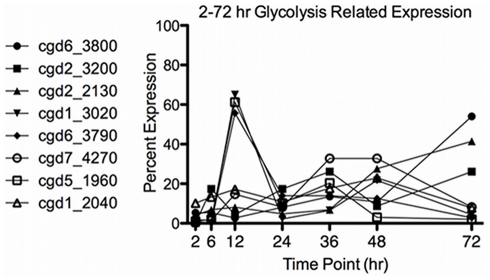
Glycolysis Related Transcripts. The relative expression using 2-72 hr data is graphed for the annotated enzymes involved in glycolysis.

Metabolic enzymes required for energy conversion and potential storage of sugars were expressed at their highest level at 12 hr in the developing merozoites and may be used to complete development or store energy for the traversal to the next host cell. Overall, 8 of the 9 genes used in glycolysis were present in our data set (Figure 8). Three of these genes were constitutively expressed (cgd6_3800 [hexokinase], cgd2_2130 [pyrophosphate-dependent phosphofructokinase], and cgd1_2040 [pyruvate kinase]), whereas cgd2_3200 [glucose-6-phosphate isomerase] spiked in expression at 6 hr. Transcripts for the remaining enzymes cgd1_3020 [fructose-1 disphosphate aldolase], cgd6_3790 [glyceraldehyde 3-phosphate dehydrogenase], cgd7_4270 [phosphoglycerate mutase], and cgd5_1960 [enolase] all spiked at 12 hr. It is interesting to note that these same four proteins are found in the sporozoite proteome [10], suggesting that the type I meront/merozoite also has a high need for energy production. Non-constitutive expression of these enzymes is not surprising since *Toxoplasma* also demonstrates stage-specific expression of enolase and pyruvate kinase [54]–[56]. In addition, cgd3_3100, a putative sugar transporter, was highly expressed at this time point providing further evidence for an increase in metabolism in type I meronts. Interestingly, most of the host cell glycolytic pathway genes have been analyzed during a very similar infection time course [57]. Combining data generated in this study indicates that while the parasite is increasing cgd1_3020, cgd6_3790, and cgd5_1960 expression at 12 hr, host cell expression of orthologs is decreasing [58] suggesting that the host cell is decreasing its own glucose usage in response to parasite invasion.

### Mature Meronts/Merozoites: cell cycle completion and DNA repair

The 24 hr time point contained the highest fraction of mature type I meronts with 6–8 merozoites that have completed cytokineses (Figure 1). Although both sporozoites and merozoites infect epithelial cells within the same host, their different expression profiles and distribution of functional categories between 2 hr and 24 hr indicated that sporozoites are biochemically distinct from merozoites. Most notably DNA-associated genes involved in replication and mitosis were specifically elevated at 24 hr. Surprisingly, transcripts for two core components for the condesin complex, SMC2 and SMC4 [59], [60], topoisomerase II, DNA ligase I, and RAD45 were elevated after cytokinesis. Since merozoite cytokinesis is largely complete by 24 hr, this suggests that either these proteins are produced and packaged for subsequent infection by merozoites or the transcripts have a very long half-life. The pre-packaging of proteins needed in the next round of DNA synthesis within merozoites was illustrated further by the high expression of CDC6, MCM6, and MCM7 at 24 hr. In eukaryotes, the MCM2-7 complex assembles on ORC at early G1 to form a prereplicative complex [61]. Activation of the replicative origin by CDC6 prevents cells from entering S phase when environmental conditions are unfavorable. Here, *Cryptosporidium* merozoites appear poised to initiate the next cell cycle upon reinfection. Other MCMs were more highly expressed at earlier time points. Taken together, the transcriptome is consistent with a mechanism in which the sporozoites infect with replication complexes that are missing a few subunits, and that DNA replication ensues only after translation of the missing factors. Alternatively, the asynchrony of MCM synthesis could also indicate that *Cryptosporidium*, like *Plasmodium*, may use a different helicase formation that is developmentally regulated during asexual development, similar to the expression and use of the MCM2/6/7 complex in *Plasmodium* [62].

### Sexual stage-associated transcripts

The previous discussion was based on the clustering of the first 24 hr because of the relatively synchronous developmental stage progression permits us to look specifically at the transcripts required for one round of asexual replication. When taking the relative expression utilizing the entire 72 hr time course, several replication-associated transcripts spiked in expression at later time points, appearing to mute expression within the first 24 hr. In later time points, additional sexual stages are developing, and certain expression patterns may be diluted by simultaneous and asynchronous progression of both asexual and sexual developmental programs. For example, gametogenesis occurs after 24 hr and requires rapid DNA replication. This would explain the large increase in DNA replication genes within the later time points (Figure S3). Additionally, it is highly plausible that like other Apicomplexans, *Cryptosporidium* sexual replication may not use schizogony, thus changing the transcriptional requirements for sexual development [50].

At 36 hr reinfection of epithelial cells by merozoites has occurred, which coincides with an overrepresentation of DNA-associated genes. Genes in cluster 3 had similar peaks in expression at 12 and 36 hr, whereas cluster 8 genes are much more highly expressed at 12 hr. The cyclical expression of some genes between 12 hr and 36 hr (cluster 3) suggests a reinfection of asexually replicating merozoites had a similar gene expression pattern to that of the first round of asexual replication. However, having a separate subset of genes that does not follow this cyclical patterning (cluster 8) also suggests that there is a difference in the cultures at these time points. We noted that only cgd7_4740, Rab GDP disassociation inhibitor, has a high (84%) relative expression at 36 hr, suggesting it is specific to a new stage that is present at this time. Interestingly, cgd4_3110, a putative nucleotide-sugar transporter and cgd2_800, a Major facilitator super family transporter, also had the majority of their expression at 36 hr, which may indicate a change in the nutritional requirements of this new developing parasite stage.

The culture at 48 hr has increased morphological complexity, with over half of the organisms appearing to be of meront size or greater. Despite this developmental stage heterogeneity, the appearance of genes that peaked at 48 hr (clusters 2 and 4) indicated the presence of new developmental stages. Metabolic enzymes and transporters were the most overrepresented functional categories expressed at this time point. Metabolic enzymes such as fatty acid synthetase, S-adenosylhomocysteinase, and pyruvate:ferredoxin oxidoreductase showed increased expression as the time course ensues. This is surprising considering that the streamlined metabolic pathways in *Cryptosporidium* might predict a more constitutive expression pattern rather than the striking peak observed here. Increased expression of these enzymes could reflect a shift in the metabolic need of the sexual stages developing at this time or may be due to nutrient depletion of the host cells forcing the parasite to shift its energy sources. Relative expression of the amino acid transporters cgd3_2730, cgd3_2050, and cgd3_4350 was highest at 48 hr and further emphasizes a shift in metabolic requirements. A large number of genes at this time point appeared to encode extracellular proteins, including 5 mucin-like genes, one of which (cgd3_720), showed no detectable expression until 48 hr. Three protein kinases and one phosphatase were highly expressed and may be key regulators in the life cycle progression of this parasite.

Clearly the 72 hr transcriptome indicates significant changes within the culture, and 37 of the 107 putative developmental stage-specific genes (>80% expression at one time point) peaked at 72 hr. Metabolic enzymes are again the overrepresented category at 72 hr, with 82% of pyruvate decarboxylase total transcripts observed at this time. Relative expression of cgd7_4800 (amino acid transporter) and cgd2_590 (nucleotide-sugar transporter) also peak at this time point, which is consistent with a change in the metabolic requirements of the parasite. There are eighteen AP2 domain containing genes in our dataset out of the nineteen predicted in *Cryptosporidium* [63]. The hypothetical protein, cgd4_1110, an AP2 domain containing protein, had no detectable expression in the first 24 hr, but has over 95% of its expression between 48 and 72 hr [63]–[65]. In addition, another AP2 domain containing gene (cgd2_3490) had the majority of its expression during the 48 and 72 hr time points, whereas the AP2 domain containing proteins, cgd4_2950 and cgd8_3130 had >60% expression at 12 hr. This expression patterning suggests that like the other Apicomplexa, stage-specific utilization of AP2 domain containing transcription factors by *C. parvum* controls developmental stage-associated gene expression.

*Cryptosporidium* sporozoites infect as diploid organisms and meiosis is required to proceed through zygote formation. Genes known to be integral for meiosis in many organisms were highly expressed specifically at 48 and 72 hr in our culture. Dmc1, CDK (cgd5_2510) and Cyclin A (cgd3_4050) all had their highest expression at 72 hr, while Hop1 peaked at 48 hr. ORC4 had over 50% of its expression at 48 hr alone, while ORC2 and ORC5 both demonstrate cyclic expression that peaked at 6, 24, and 48 hr. When analyzing all 7 time points, MCM 2, 3, and 5–7 were more constitutively expressed, although MCM 2, 3 and 10 had higher relative expression at 48 hr. We found ORC1 and CDC6 expression to follow similar patterns, until 72 hr where CDC6 remained high while ORC1 expression plummeted (Figure S3). This suggests that ORC1, CDC6, and perhaps OCR4 may form a replication initiation complex during the sexual stages present at later time points, whereas ORC2 and ORC5 have more universal roles in DNA replication and differs from the model in which ORC1-ORC6 and CDC6 function as a stoichiometric complex for DNA origin initiation [66]. However, recent mapping of ORC binding has identified many sites that are not consistent with origin of replications [67], [68]. It is likely that due to the streamlined genome of *Cryptosporidium* many proteins possess multiple functions, in this case perhaps an additional chromatin-associated role for ORC1.

### Conclusions

We observed transcripts for all 3,200+ genes assessed within at least one time point over 72 hr in vitro infection of HCT-8 cells. This 72 hr time course covered the known length of time parasite can be propagated *in vitro* without a large decrease in parasite numbers detected through 18S rRNA C_t_ values. Following the removal of unexcysted oocysts at 2 hr incubation, the first 24 hr of the time course remained highly homogenous despite clear life cycle progression. Thus, transcripts specific to the predominant parasitic stage at specific time points were discovered and transcriptome differences between sporozoites and merozoites were discernible. Over 75% of the genome had detectable transcription throughout the 72 hr time course, yet the identification of genes with large spikes in expression at a single time point was particularly intriguing as they may encode developmental stage-specific proteins [53], [69]–[71]. It is even more interesting that the vast majority of our putative developmental stage-specific genes are of unknown function and lack any recognizable protein motif. These putative stage specific genes may reveal biology that is unique to a specific developmental stage and may be useful for purifying individual stages from the heterogeneous cultures or as chemotherapeutic targets.

## Supporting Information

Figure S1 **cDNA Synthesis Control.** Lack of genomic contamination was confirmed for the cDNA time courses with RT-PCR of replicate reactions of samples with (black) and without (red) reverse transcription. All the reactions without reverse transcriptase had a Ct value >35 with amplicon melt temperatures ranging from 73–75°C. Due to the nature of ribosomal primers, anything >35 with a melt temperature <83–85°C is deemed as no product detected.
(TIFF)

Figure S2 **Categorization of Gene Expression.** RT-PCR was run on 3302 genes in the *C. parvum* genome and normalized to *C. parvum* 18S rRNA expression. Each gene's median expression from four biological replicates was categorized for each time point as being: zero (not detected), high (log_10_ normalized value >2 standard deviations (SD) from mean), moderate (log_10_ normalized value within 2 SD from mean), or low (log_10_ normalized value <2 SD from mean) expression at each time point.
(TIF)

Figure S3 **Relative Expression for DNA Replication Associated Proteins, Proteasome Subunits, Ribosome related and tRNA synthesis.** The relative expression for 2–72 hr data is graphed for the (A) ORC, (B) MCM and (C) Meiosis related genes, (D) tRNA synthesis, (E) ribosomal proteins, (F) alpha and (G) beta proteasome subunits.
(TIF)

Table S1 ***C. parvum*** **18S rRNA-normalized transcript abundance for each of the four infection time courses.**
(PDF)

Table S2 **Primers used for C. parvum RT-PCR.**
(PDF)

Table S3 ***C. parvum*** **genes with >80% of total expression at a single time point.**
(PDF)
