# Supplementary figures and images for: The Cryptosporidium Parvum Transcriptome during In Vitro Development
Source: PLoS One. 2012 Mar 15;7(3):e31715. doi: 10.1371/journal.pone.0031715 (PMC3305300; doi:10.1371/journal.pone.0031715)

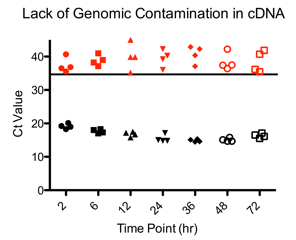

Supplement: Figure S1 — cDNA Synthesis Control. Lack of genomic contamination was confirmed for the cDNA time courses with RT-PCR of replicate reactions of samples with (black) and without (red) reverse transcription. All the reactions without reverse transcriptase had a Ct value >35 with amplicon melt temperatures ranging from 73–75°C. Due to the nature of ribosomal primers, anything >35 with a melt temperature <83–85°C is deemed as no product detected. (TIFF) [file pone.0031715.s001.tiff]

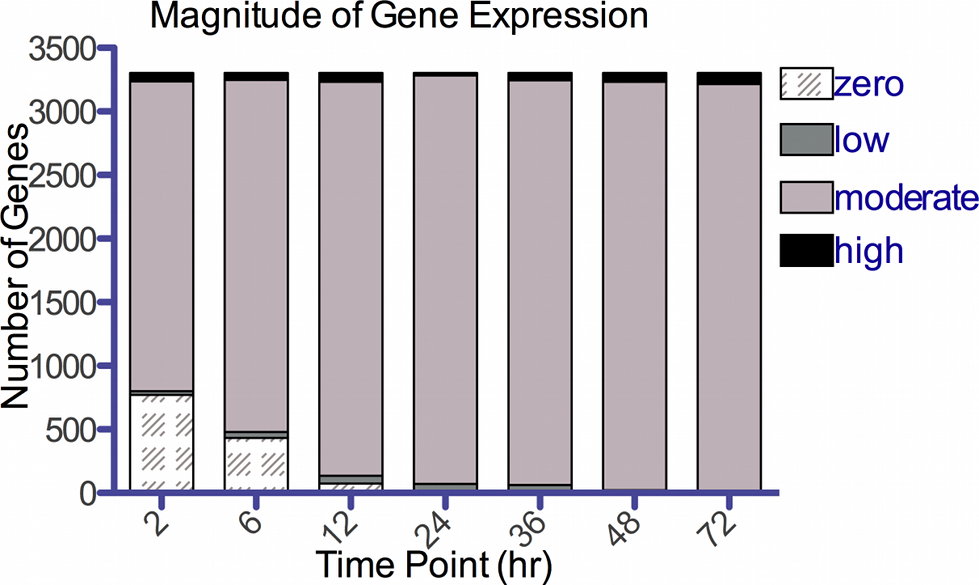

Supplement: Figure S2 — Categorization of Gene Expression. RT-PCR was run on 3302 genes in the C. parvum genome and normalized to C. parvum 18S rRNA expression. Each gene's median expression from four biological replicates was categorized for each time point as being: zero (not detected), high (log10 normalized value >2 standard deviations (SD) from mean), moderate (log10 normalized value within 2 SD from mean), or low (log10 normalized value <2 SD from mean) expression at each time point. (TIF) [file pone.0031715.s002.tif]

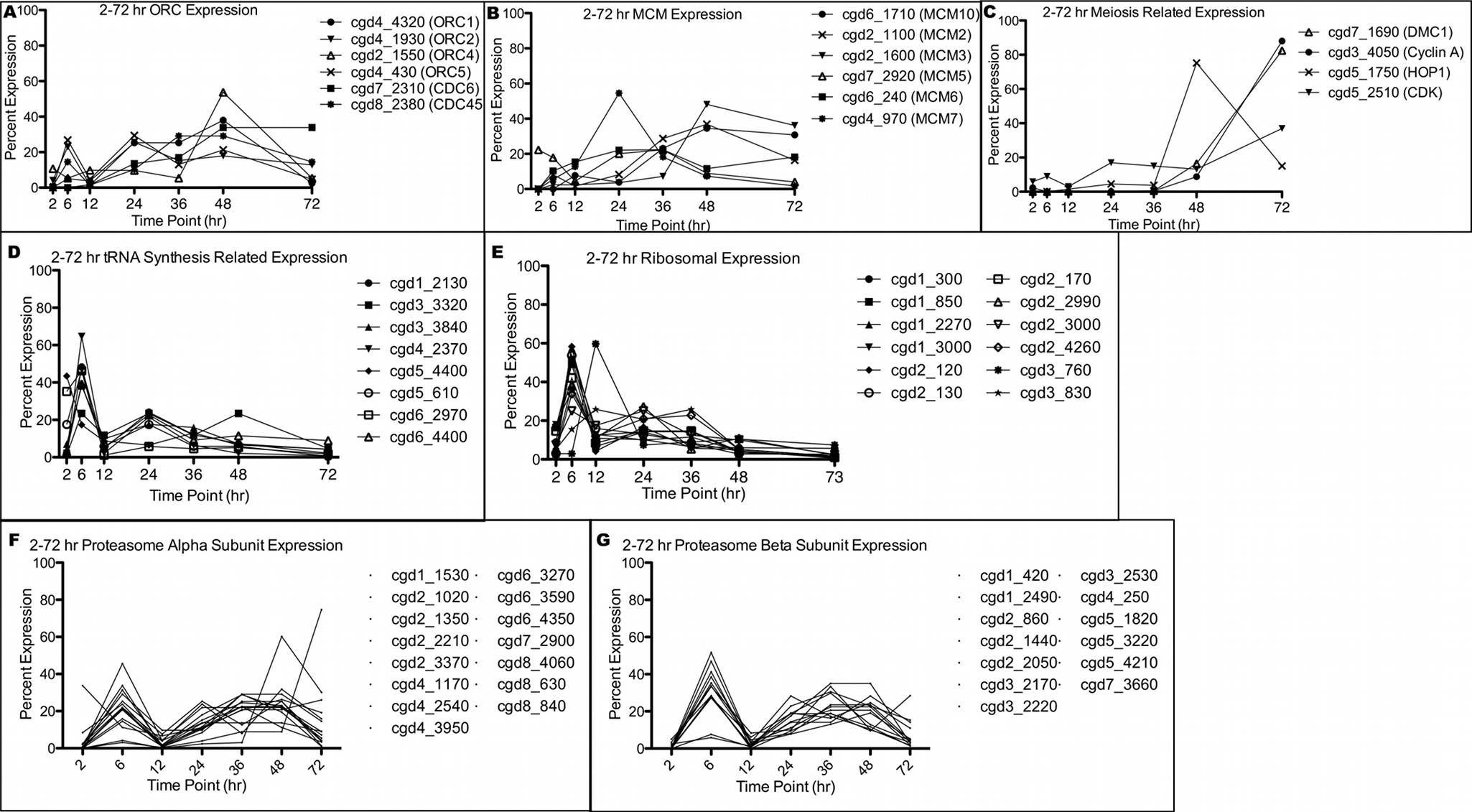

Supplement: Figure S3 — Relative Expression for DNA Replication Associated Proteins, Proteasome Subunits, Ribosome related and tRNA synthesis. The relative expression for 2–72 hr data is graphed for the (A) ORC, (B) MCM and (C) Meiosis related genes, (D) tRNA synthesis, (E) ribosomal proteins, (F) alpha and (G) beta proteasome subunits. (TIF) [file pone.0031715.s003.tif]
