# Supplementary material for: The Cryptosporidium Parvum Transcriptome during In Vitro Development
Source: PLoS One. 2012 Mar 15;7(3):e31715. doi: 10.1371/journal.pone.0031715 (PMC3305300; doi:10.1371/journal.pone.0031715)
Supplement: Table S1 — C. parvum 18S rRNA-normalized transcript abundance for each of the four infection time courses. (PDF) [file pone.0031715.s004.pdf]

Time point	Time Course 1	Time Course 2	Time Course 3	Time Course 4
	cgd2_1400			
**2h**	0.00E+00	5.01E-08	0.00E+00	1.02E-07
**6h**	2.01E-09	1.98E-08	5.85E-08	1.42E-06
**12h**	7.70E-10	2.02E-08	2.16E-08	1.77E-08
**24h**	2.57E-09	1.22E-08	2.19E-08	1.43E-07
**36h**	1.23E-08	1.10E-08	9.35E-09	6.18E-08
**48h**	1.12E-06	1.17E-07	9.78E-08	3.57E-06
**72h**	1.53E-07	4.09E-06	2.81E-08	1.11E-06
	cgd3_3330			
**2h**	9.34E-07	6.72E-06	6.10E-08	9.62E-07
**6h**	3.35E-08	4.10E-07	5.05E-08	1.13E-08
**12h**	2.05E-07	1.17E-06	1.15E-08	6.87E-08
**24h**	1.11E-07	7.36E-07	8.05E-08	2.19E-08
**36h**	1.33E-06	9.56E-08	1.85E-07	1.27E-07
**48h**	6.29E-07	3.73E-07	2.51E-07	1.16E-06
**72h**	2.38E-07	6.43E-08	3.53E-07	1.33E-07
	cgd3_2710			
**2h**	3.91E-09	0.00E+00	0.00E+00	2.27E-09
**6h**	5.45E-09	4.85E-08	2.04E-09	1.19E-08
**12h**	4.74E-09	2.29E-08	5.72E-09	8.78E-09
**24h**	1.87E-09	9.08E-09	4.15E-09	4.44E-09
**36h**	9.49E-08	8.11E-09	8.85E-09	1.12E-08
**48h**	2.04E-07	6.68E-08	3.16E-08	7.38E-07
**72h**	8.55E-08	8.34E-08	1.01E-07	1.99E-07
	cgd3_2730			
**2h**	2.44E-08	1.08E-07	0.00E+00	3.60E-09
**6h**	2.29E-08	3.11E-07	7.12E-10	1.36E-08
**12h**	1.81E-07	2.56E-07	2.77E-09	3.77E-08
**24h**	4.27E-08	1.17E-07	4.87E-08	1.50E-08
**36h**	1.07E-06	7.95E-08	1.10E-07	4.49E-08
**48h**	8.53E-07	2.21E-07	1.54E-07	1.38E-06
**72h**	1.16E-07	5.21E-08	4.82E-07	5.87E-08
	cgd3_3340			
**2h**	0.00E+00	0.00E+00	0.00E+00	1.07E-05
**6h**	3.68E-07	7.88E-07	8.29E-08	2.53E-07
**12h**	2.45E-06	1.57E-05	7.76E-07	7.59E-07
**24h**	6.33E-07	2.32E-06	1.50E-07	5.33E-07
**36h**	2.19E-05	1.49E-07	8.39E-08	2.61E-07
**48h**	2.51E-06	1.36E-06	0.00E+00	4.78E-06
**72h**	6.04E-07	3.51E-07	1.01E-06	1.25E-06
	cgd3_3140			
**2h**	9.20E-08	0.00E+00	0.00E+00	0.00E+00
**6h**	0.00E+00	0.00E+00	0.00E+00	0.00E+00
**12h**	1.81E-06	5.70E-06	0.00E+00	5.87E-08
**24h**	5.36E-08	1.18E-06	1.34E-08	1.01E-09
**36h**	5.39E-06	2.80E-06	2.89E-07	5.78E-07
**48h**	4.32E-06	9.25E-06	3.46E-07	8.71E-06
**72h**	1.40E-06	3.20E-06	4.09E-06	3.78E-06
	cgd3_3200			
**2h**	4.60E-10	1.33E-08	0.00E+00	8.06E-10
**6h**	7.77E-10	0.00E+00	0.00E+00	1.32E-10

**12h**	7.33E-07	4.95E-07	1.31E-07	0.00E+00
**24h**	5.25E-08	1.32E-07	4.66E-08	2.25E-08
**36h**	7.98E-07	1.97E-07	1.55E-07	1.56E-07
**48h**	2.22E-07	2.47E-07	4.45E-08	6.96E-07
**72h**	6.32E-08	9.10E-08	2.18E-07	8.61E-08
	cgd3_3210			
**2h**	6.69E-10	0.00E+00	0.00E+00	1.32E-09
**6h**	2.18E-08	2.92E-07	1.04E-08	6.66E-08
**12h**	7.83E-08	2.34E-07	7.11E-09	1.19E-08
**24h**	2.96E-08	2.17E-07	3.97E-08	1.29E-08
**36h**	2.06E-06	1.91E-07	2.09E-07	4.17E-07
**48h**	2.45E-07	4.67E-07	4.21E-08	1.35E-06
**72h**	1.74E-07	1.28E-07	7.46E-07	2.69E-07
	cgd3_3240			
**2h**	1.65E-08	8.72E-08	3.04E-09	2.09E-08
**6h**	1.18E-09	5.44E-10	2.04E-10	6.46E-09
**12h**	5.59E-09	1.73E-08	5.05E-09	2.14E-09
**24h**	4.16E-09	6.79E-09	3.69E-09	3.96E-09
**36h**	3.42E-08	2.25E-09	2.28E-08	4.37E-09
**48h**	2.04E-08	3.16E-08	2.02E-08	3.77E-08
**72h**	2.82E-08	1.51E-08	6.79E-08	2.22E-08
	cgd3_3270			
**2h**	2.00E-08	6.05E-08	6.47E-08	5.10E-08
**6h**	9.58E-09	2.64E-09	1.55E-08	4.43E-09
**12h**	4.54E-08	2.91E-08	5.69E-08	5.47E-08
**24h**	9.56E-09	9.41E-09	1.87E-08	1.41E-08
**36h**	4.64E-09	5.18E-09	9.14E-09	1.40E-08
**48h**	1.53E-08	1.13E-08	2.91E-08	1.49E-08
**72h**	5.57E-09	2.96E-09	7.64E-09	1.07E-08
	cgd3_3320			
**2h**	0.00E+00	5.99E-08	0.00E+00	1.13E-08
**6h**	2.02E-07	1.33E-07	9.86E-08	7.02E-08
**12h**	4.51E-08	1.66E-07	5.98E-08	3.95E-08
**24h**	7.13E-08	2.82E-07	1.64E-07	6.61E-08
**36h**	9.78E-08	3.75E-08	5.79E-08	4.87E-08
**48h**	1.43E-07	6.06E-08	8.62E-08	2.53E-07
**72h**	1.98E-08	1.11E-08	3.68E-08	1.24E-08
	cgd3_3130			
**2h**	0.00E+00	7.63E-09	3.90E-09	2.69E-10
**6h**	0.00E+00	0.00E+00	0.00E+00	0.00E+00
**12h**	2.23E-10	6.95E-10	7.57E-10	8.51E-10
**24h**	2.21E-10	9.58E-10	2.94E-10	2.08E-10
**36h**	5.68E-09	9.40E-10	4.68E-10	5.66E-11
**48h**	2.32E-08	1.26E-08	6.50E-09	2.83E-08
**72h**	1.93E-08	5.25E-08	1.07E-07	1.16E-08
	cgd1_3020			
**2h**	7.07E-10	1.66E-09	0.00E+00	0.00E+00
**6h**	2.09E-08	4.64E-09	1.22E-07	4.91E-10
**12h**	6.48E-06	4.53E-06	5.33E-07	7.91E-08
**24h**	2.72E-07	1.84E-07	9.98E-08	1.87E-08
**36h**	9.76E-07	3.98E-07	1.12E-07	6.40E-08
**48h**	2.02E-06	3.16E-06	8.54E-07	4.50E-07
**72h**	3.23E-07	1.95E-07	8.65E-08	2.12E-07

	cgd1_3090			
**2h**	4.33E-09	2.00E-08	3.44E-07	5.18E-08
**6h**	2.23E-07	1.84E-07	1.44E-06	1.18E-08
**12h**	1.30E-07	6.62E-08	6.84E-07	2.16E-08
**24h**	1.00E-07	4.62E-08	6.19E-07	2.09E-08
**36h**	1.11E-07	7.87E-08	7.02E-07	5.85E-08
**48h**	9.40E-08	1.28E-07	1.27E-06	9.59E-07
**72h**	2.74E-08	8.34E-08	2.51E-07	3.41E-07
	cgd1_3080			
**2h**	1.29E-07	0.00E+00	2.69E-06	2.50E-07
**6h**	1.95E-06	2.51E-06	6.47E-06	3.06E-07
**12h**	1.49E-06	7.40E-07	1.66E-06	9.50E-07
**24h**	2.01E-06	1.86E-07	7.52E-06	3.48E-07
**36h**	1.25E-06	1.14E-06	1.85E-05	7.46E-07
**48h**	1.27E-06	2.18E-06	1.23E-05	5.85E-06
**72h**	7.71E-07	9.36E-07	2.07E-06	6.27E-06
	cgd1_3070			
**2h**	2.12E-04	3.13E-04	1.86E-03	1.83E-04
**6h**	2.77E-05	2.88E-05	1.38E-05	2.57E-06
**12h**	4.20E-04	3.57E-04	5.76E-04	8.50E-05
**24h**	7.90E-05	2.32E-05	2.18E-04	2.48E-05
**36h**	2.89E-05	1.02E-05	4.40E-05	1.03E-05
**48h**	4.97E-04	8.57E-05	5.97E-04	6.84E-04
**72h**	4.21E-05	4.60E-05	1.21E-04	2.75E-04
	cgd1_3060			
**2h**	1.53E-07	3.79E-07	1.57E-06	1.04E-07
**6h**	8.43E-08	5.39E-08	3.48E-07	3.51E-08
**12h**	9.92E-08	5.51E-08	4.12E-07	8.14E-08
**24h**	6.81E-08	4.54E-08	2.09E-07	2.82E-08
**36h**	5.58E-08	2.30E-07	1.02E-07	4.71E-08
**48h**	5.45E-08	5.32E-08	3.06E-07	4.30E-07
**72h**	2.08E-08	1.04E-08	9.13E-08	1.67E-07
	cgd1_3050			
**2h**	5.30E-07	3.90E-06	7.77E-06	7.99E-09
**6h**	4.47E-07	7.35E-07	2.03E-06	8.34E-07
**12h**	1.61E-07	6.81E-07	1.03E-06	1.51E-08
**24h**	5.06E-07	1.24E-07	1.07E-06	1.39E-07
**36h**	5.83E-07	8.60E-07	1.73E-06	8.83E-07
**48h**	8.56E-07	1.37E-06	5.04E-06	6.18E-06
**72h**	5.98E-07	1.52E-07	9.22E-07	5.86E-05
	cgd1_3040			
**2h**	2.23E-07	7.24E-07	7.46E-07	6.90E-09
**6h**	6.77E-08	1.53E-08	1.22E-07	1.23E-09
**12h**	1.77E-07	4.99E-07	4.69E-07	1.84E-07
**24h**	1.63E-07	1.87E-07	2.48E-07	1.33E-08
**36h**	3.95E-06	1.21E-06	4.92E-06	5.33E-07
**48h**	1.94E-06	2.80E-06	5.10E-06	9.77E-06
**72h**	4.52E-07	3.10E-07	1.36E-06	9.82E-06
	cgd1_3030			
**2h**	7.99E-09	9.24E-09	6.80E-08	4.23E-09
**6h**	1.51E-07	2.16E-07	9.35E-07	1.29E-07
**12h**	1.32E-08	1.30E-08	3.14E-08	0.00E+00
**24h**	1.23E-07	5.69E-08	3.95E-07	5.80E-09

**36h**	1.32E-07	7.66E-08	4.36E-07	4.70E-08
**48h**	8.24E-08	1.11E-07	5.24E-07	3.42E-07
**72h**	9.25E-08	1.24E-07	8.35E-07	1.28E-06
	cgd2_1370			
**2h**	8.45E-08	7.99E-08	6.70E-08	2.01E-08
**6h**	1.58E-07	2.80E-07	1.15E-07	9.20E-08
**12h**	2.99E-08	9.26E-08	2.20E-08	1.66E-08
**24h**	1.09E-07	2.71E-08	1.60E-07	1.06E-08
**36h**	4.16E-08	3.25E-07	5.90E-08	2.45E-08
**48h**	5.26E-08	5.16E-08	1.47E-07	2.74E-07
**72h**	1.68E-08	1.32E-08	2.80E-08	1.40E-07
	cgd2_1350			
**2h**	3.18E-08	2.05E-08	7.09E-08	0.00E+00
**6h**	2.93E-07	4.80E-07	1.14E-06	2.13E-07
**12h**	9.54E-09	2.13E-08	8.86E-09	1.27E-09
**24h**	1.28E-07	7.20E-08	1.99E-07	2.87E-08
**36h**	8.45E-08	4.94E-07	4.44E-07	7.60E-08
**48h**	9.64E-08	1.99E-07	4.30E-07	3.38E-07
**72h**	2.92E-08	3.35E-08	6.48E-08	4.50E-07
	cgd2_1310			
**2h**	4.95E-06	3.46E-05	9.56E-06	3.13E-05
**6h**	6.00E-06	1.32E-04	3.48E-06	1.99E-05
**12h**	2.51E-06	3.67E-05	9.63E-06	5.45E-05
**24h**	2.29E-06	6.93E-06	6.65E-06	4.51E-06
**36h**	1.37E-06	3.30E-05	6.09E-06	5.82E-06
**48h**	1.87E-06	1.78E-05	2.79E-06	6.02E-05
**72h**	4.06E-07	3.30E-06	1.67E-06	1.70E-05
	cgd2_1450			
**2h**	1.09E-09	2.16E-10	0.00E+00	0.00E+00
**6h**	2.47E-07	5.90E-07	2.37E-06	1.47E-08
**12h**	4.62E-09	1.34E-08	2.12E-08	1.78E-08
**24h**	9.05E-08	5.86E-08	2.81E-07	1.49E-08
**36h**	1.12E-07	3.07E-07	3.06E-07	4.36E-08
**48h**	6.35E-08	1.09E-07	4.96E-07	6.03E-07
**72h**	4.25E-08	3.10E-08	7.34E-08	7.84E-07
	cgd2_1470			
**2h**	2.75E-08	5.07E-08	3.94E-08	3.14E-08
**6h**	5.20E-08	7.85E-08	1.97E-07	1.21E-09
**12h**	2.09E-08	7.58E-08	3.18E-08	2.15E-08
**24h**	2.93E-08	2.93E-08	5.98E-08	1.06E-08
**36h**	6.67E-09	1.00E-07	2.07E-08	9.48E-09
**48h**	1.45E-08	5.68E-08	7.44E-08	2.06E-07
**72h**	1.30E-08	7.27E-09	3.13E-08	1.36E-07
	cgd2_1480			
**2h**	0.00E+00	2.36E-07	2.98E-07	5.80E-07
**6h**	1.44E-07	5.36E-08	2.44E-08	1.35E-06
**12h**	0.00E+00	1.31E-07	1.01E-07	4.84E-06
**24h**	5.63E-08	2.15E-08	4.69E-08	7.73E-07
**36h**	9.45E-08	2.88E-07	2.07E-07	0.00E+00
**48h**	4.46E-07	1.32E-07	1.26E-08	1.84E-06
**72h**	9.63E-08	3.22E-08	1.81E-08	5.68E-06
	cgd2_1490			
**2h**	1.84E-07	1.73E-08	3.55E-09	0.00E+00

**6h**	4.16E-07	1.07E-08	4.46E-10	3.17E-10
**12h**	1.20E-07	5.84E-09	1.21E-09	5.67E-10
**24h**	2.50E-07	7.20E-10	1.04E-09	8.54E-11
**36h**	6.48E-08	3.46E-08	8.77E-10	7.73E-10
**48h**	4.88E-08	3.74E-08	3.46E-08	1.61E-07
**72h**	1.52E-08	6.89E-09	1.58E-08	6.71E-08
	cgd2_1510			
**2h**	5.90E-07	1.12E-07	2.23E-07	4.71E-09
**6h**	5.28E-08	1.46E-07	1.04E-07	4.82E-09
**12h**	2.84E-08	1.82E-07	4.84E-08	3.16E-08
**24h**	1.05E-08	8.79E-09	4.65E-08	9.41E-09
**36h**	1.96E-08	2.20E-07	2.84E-08	5.28E-09
**48h**	1.09E-08	7.34E-08	3.80E-08	1.78E-07
**72h**	2.02E-08	1.81E-08	2.68E-08	4.84E-08
	cgd2_1520			
**2h**	3.29E-06	9.41E-06	5.61E-06	1.25E-05
**6h**	5.96E-07	3.29E-06	3.05E-06	2.64E-07
**12h**	1.89E-06	2.65E-05	5.20E-06	6.26E-06
**24h**	5.51E-07	1.16E-06	3.14E-06	3.57E-07
**36h**	6.28E-07	8.43E-06	9.48E-07	2.42E-06
**48h**	2.31E-07	1.60E-06	2.48E-06	2.00E-05
**72h**	2.80E-07	3.06E-07	3.44E-07	1.45E-05
	cgd2_1530			
**2h**	1.02E-07	8.71E-08	1.57E-07	1.63E-08
**6h**	4.15E-08	8.99E-08	1.20E-07	8.85E-09
**12h**	2.59E-08	1.08E-08	5.57E-08	1.22E-08
**24h**	7.89E-09	1.58E-08	3.20E-08	5.72E-09
**36h**	1.77E-08	5.29E-08	3.26E-08	5.17E-09
**48h**	9.51E-09	4.36E-08	6.67E-08	1.96E-07
**72h**	2.40E-08	3.60E-09	6.06E-08	3.95E-08
	cgd2_1600			
**2h**	0.00E+00	0.00E+00	0.00E+00	0.00E+00
**6h**	0.00E+00	0.00E+00	4.10E-09	4.41E-09
**12h**	3.90E-09	1.21E-09	1.55E-09	1.55E-09
**24h**	1.25E-09	2.84E-10	5.47E-09	1.28E-08
**36h**	7.85E-09	4.51E-09	2.94E-09	1.58E-07
**48h**	1.68E-07	1.38E-08	3.03E-08	4.67E-08
**72h**	4.60E-08	7.92E-10	3.86E-08	2.61E-08
	cgd2_1610			
**2h**	0.00E+00	0.00E+00	0.00E+00	0.00E+00
**6h**	2.19E-09	1.26E-09	7.28E-09	8.96E-09
**12h**	4.16E-10	4.00E-10	6.47E-11	2.31E-10
**24h**	1.03E-08	4.00E-09	2.30E-09	3.13E-09
**36h**	7.60E-09	2.53E-09	1.19E-10	7.09E-09
**48h**	4.81E-08	1.39E-07	3.90E-08	2.53E-08
**72h**	1.32E-08	2.75E-08	4.96E-09	1.38E-08
	cgd2_1060			
**2h**	4.36E-06	1.99E-05	1.71E-06	2.13E-05
**6h**	1.95E-06	7.02E-06	2.23E-06	5.43E-05
**12h**	1.79E-06	1.08E-05	1.19E-06	4.55E-05
**24h**	1.46E-06	3.33E-06	3.14E-06	2.30E-05
**36h**	1.13E-06	6.94E-06	9.44E-07	7.70E-06
**48h**	1.13E-06	8.28E-06	3.35E-06	2.80E-05

**72h**	4.27E-07	1.19E-06	7.14E-07	3.72E-06
	cgd2_1100			
**2h**	0.00E+00	0.00E+00	0.00E+00	1.66E-08
**6h**	1.58E-08	6.78E-08	3.43E-07	5.30E-07
**12h**	6.78E-09	5.44E-08	3.30E-08	1.94E-07
**24h**	9.68E-08	2.48E-07	7.11E-08	1.01E-06
**36h**	5.38E-07	8.46E-07	3.76E-07	1.08E-06
**48h**	1.66E-07	1.40E-06	6.44E-07	1.10E-06
**72h**	1.93E-07	1.88E-07	5.69E-07	2.02E-06
	cgd2_1150			
**2h**	3.17E-07	1.43E-07	3.51E-07	2.34E-07
**6h**	6.93E-07	8.93E-07	1.86E-06	1.38E-06
**12h**	2.65E-07	1.91E-07	5.73E-07	3.54E-07
**24h**	3.45E-07	2.07E-07	9.34E-07	9.26E-07
**36h**	1.81E-07	3.68E-07	6.21E-07	3.95E-07
**48h**	1.65E-07	3.52E-07	9.68E-07	6.97E-07
**72h**	8.32E-08	1.12E-08	1.85E-07	2.40E-07
	cgd2_1160			
**2h**	3.91E-05	6.38E-04	2.88E-05	1.06E-04
**6h**	6.42E-05	5.15E-03	1.72E-04	1.24E-03
**12h**	5.47E-05	4.13E-04	3.83E-05	9.73E-04
**24h**	3.62E-05	4.13E-04	3.32E-05	6.35E-04
**36h**	5.14E-05	6.91E-04	4.40E-05	4.95E-04
**48h**	5.22E-05	2.85E-04	2.91E-05	2.05E-04
**72h**	5.73E-06	1.94E-05	9.93E-06	1.41E-04
	cgd2_1170			
**2h**	2.12E-07	5.60E-08	7.85E-08	1.86E-08
**6h**	1.32E-07	6.22E-08	2.62E-08	1.69E-08
**12h**	1.66E-07	1.15E-07	2.53E-08	1.49E-08
**24h**	7.21E-08	3.19E-08	3.95E-08	1.06E-08
**36h**	3.00E-08	4.36E-08	1.28E-08	1.41E-08
**48h**	6.63E-08	7.08E-08	8.26E-08	2.58E-08
**72h**	2.48E-08	2.57E-09	5.32E-08	4.64E-08
	cgd2_1190			
**2h**	7.77E-08	1.89E-08	1.02E-07	4.37E-08
**6h**	4.20E-09	1.10E-08	1.15E-09	8.78E-09
**12h**	9.80E-08	5.70E-08	5.12E-08	3.64E-08
**24h**	3.89E-09	4.90E-09	2.32E-08	1.50E-08
**36h**	1.28E-08	1.56E-08	4.70E-09	8.92E-09
**48h**	4.00E-08	2.00E-08	4.25E-08	3.18E-08
**72h**	3.17E-08	2.48E-09	3.77E-08	6.46E-08
	cgd2_1200			
**2h**	0.00E+00	1.80E-08	4.95E-10	6.13E-09
**6h**	2.91E-07	6.30E-06	7.94E-07	3.03E-06
**12h**	1.78E-07	8.69E-07	2.43E-07	8.65E-07
**24h**	2.94E-07	1.67E-06	2.68E-07	1.69E-06
**36h**	9.02E-07	1.02E-06	3.81E-07	5.21E-07
**48h**	5.47E-07	3.12E-06	2.94E-07	1.93E-06
**72h**	7.17E-07	1.71E-07	4.98E-07	8.68E-07
	cgd2_1220			
**2h**	1.60E-07	8.03E-08	3.43E-08	2.88E-08
**6h**	2.36E-09	4.21E-09	8.02E-09	3.50E-09
**12h**	3.75E-08	9.63E-09	2.29E-08	9.65E-09

**24h**	0.00E+00	9.12E-10	3.94E-09	6.49E-09
**36h**	1.11E-08	1.01E-08	5.23E-09	9.25E-09
**48h**	3.86E-08	3.05E-08	3.38E-08	2.61E-08
**72h**	2.37E-08	1.58E-08	4.12E-08	3.91E-08
	cgd2_1270			
**2h**	0.00E+00	0.00E+00	0.00E+00	1.16E-08
**6h**	6.26E-09	4.90E-09	2.56E-09	2.29E-08
**12h**	9.00E-10	2.24E-12	0.00E+00	0.00E+00
**24h**	1.28E-09	1.51E-10	1.51E-09	1.62E-08
**36h**	4.50E-09	3.87E-09	5.16E-09	2.33E-08
**48h**	3.16E-08	4.62E-08	5.53E-08	8.01E-08
**72h**	2.21E-08	1.32E-09	9.22E-08	1.54E-07
	cgd2_1290			
**2h**	0.00E+00	0.00E+00	2.74E-08	0.00E+00
**6h**	0.00E+00	0.00E+00	0.00E+00	1.19E-08
**12h**	1.09E-07	1.40E-06	5.09E-08	3.08E-07
**24h**	3.75E-07	1.03E-07	1.11E-07	8.13E-07
**36h**	6.44E-07	2.94E-06	6.49E-07	2.58E-06
**48h**	7.67E-07	3.56E-06	5.92E-07	1.77E-06
**72h**	9.43E-08	1.97E-07	4.33E-07	1.34E-06
	cgd2_1300			
**2h**	0.00E+00	0.00E+00	0.00E+00	0.00E+00
**6h**	0.00E+00	0.00E+00	0.00E+00	0.00E+00
**12h**	2.25E-09	1.44E-07	1.71E-08	3.50E-08
**24h**	5.67E-10	2.37E-09	9.00E-10	1.56E-08
**36h**	9.65E-09	2.53E-08	1.99E-09	1.30E-07
**48h**	1.08E-06	1.04E-06	9.31E-08	2.82E-06
**72h**	5.45E-08	1.49E-07	1.22E-08	1.50E-03
	cgd2_1110			
**2h**	2.03E-10	0.00E+00	0.00E+00	0.00E+00
**6h**	3.84E-10	4.12E-09	7.16E-09	1.62E-08
**12h**	1.81E-10	7.15E-11	4.30E-10	5.95E-09
**24h**	4.58E-09	9.00E-10	3.36E-09	1.60E-08
**36h**	5.33E-09	4.40E-09	5.03E-09	1.47E-08
**48h**	7.39E-09	3.43E-09	2.40E-08	8.66E-09
**72h**	3.89E-09	2.34E-09	6.81E-09	1.91E-08
	cgd2_1620			
**2h**	5.53E-08	1.81E-07	1.00E-08	9.39E-08
**6h**	3.49E-07	1.47E-07	1.27E-08	9.78E-08
**12h**	7.00E-08	8.99E-08	4.26E-09	7.88E-08
**24h**	3.78E-07	1.81E-07	1.60E-08	6.02E-08
**36h**	3.00E-07	2.44E-07	1.06E-08	4.51E-08
**48h**	3.93E-07	1.47E-06	4.43E-08	1.85E-07
**72h**	2.08E-07	3.61E-07	1.92E-08	5.26E-08
	cgd2_1650			
**2h**	9.77E-10	4.69E-09	8.40E-10	1.05E-08
**6h**	9.60E-09	4.96E-08	7.53E-08	1.88E-07
**12h**	1.65E-09	1.24E-09	1.06E-09	6.16E-09
**24h**	1.54E-08	3.76E-09	4.17E-08	9.16E-08
**36h**	1.43E-08	1.45E-08	3.81E-08	3.11E-08
**48h**	1.17E-07	1.63E-08	3.70E-08	6.76E-08
**72h**	9.47E-09	1.10E-09	6.52E-09	1.28E-08
	cgd2_1660			

**2h**	1.33E-08	5.42E-09	1.53E-08	3.28E-08
**6h**	7.90E-09	1.29E-08	7.38E-08	2.82E-08
**12h**	6.44E-10	5.22E-10	1.69E-09	4.98E-09
**24h**	1.10E-08	3.39E-09	1.75E-08	4.30E-08
**36h**	1.00E-08	3.53E-09	1.77E-08	3.66E-08
**48h**	8.18E-08	1.12E-08	1.11E-08	2.40E-08
**72h**	1.59E-08	2.90E-09	5.86E-09	9.93E-09
	cgd2_1740			
**2h**	1.56E-07	1.04E-07	1.95E-08	7.02E-08
**6h**	1.84E-07	1.77E-07	1.40E-07	3.18E-07
**12h**	5.47E-08	2.94E-08	1.17E-08	7.07E-08
**24h**	1.62E-07	4.37E-08	8.89E-08	1.61E-07
**36h**	1.86E-07	1.65E-07	1.13E-08	5.37E-08
**48h**	2.73E-07	3.67E-07	6.46E-08	1.43E-07
**72h**	8.27E-08	1.64E-07	9.94E-09	5.00E-08
	cgd2_1760			
**2h**	3.12E-07	8.51E-07	7.65E-08	6.11E-07
**6h**	3.04E-07	2.36E-07	8.21E-08	3.30E-07
**12h**	2.45E-07	1.41E-07	9.66E-08	4.18E-07
**24h**	4.35E-07	1.55E-07	1.43E-07	4.31E-07
**36h**	2.54E-07	2.10E-07	1.84E-08	6.11E-08
**48h**	5.20E-07	4.27E-07	8.55E-08	2.67E-07
**72h**	8.48E-08	1.73E-07	1.50E-08	7.94E-08
	cgd2_1800			
**2h**	7.15E-08	5.88E-08	2.72E-08	5.95E-08
**6h**	3.11E-07	4.13E-07	1.70E-08	1.18E-07
**12h**	4.89E-08	8.10E-08	1.25E-08	7.19E-08
**24h**	2.55E-07	9.74E-08	1.48E-08	6.54E-08
**36h**	2.26E-07	1.12E-07	3.99E-09	2.33E-08
**48h**	9.41E-08	1.23E-07	5.00E-09	3.73E-08
**72h**	4.09E-08	1.38E-08	7.65E-09	3.25E-08
	cgd2_1830			
**2h**	8.94E-06	1.15E-05	5.12E-07	1.92E-05
**6h**	2.31E-06	3.45E-06	1.46E-07	3.84E-06
**12h**	3.86E-07	8.04E-06	3.35E-08	2.37E-07
**24h**	1.25E-06	1.70E-07	8.49E-08	1.08E-06
**36h**	2.14E-06	1.32E-06	3.55E-09	8.75E-07
**48h**	1.23E-06	1.20E-06	5.66E-08	1.01E-06
**72h**	2.76E-06	3.35E-06	5.21E-08	2.31E-06
	cgd2_1850			
**2h**	1.60E-07	8.57E-08	1.21E-07	7.93E-08
**6h**	3.13E-06	4.19E-07	2.19E-07	5.28E-07
**12h**	8.35E-08	1.11E-07	5.25E-08	5.65E-08
**24h**	4.23E-07	3.34E-07	8.91E-08	1.75E-07
**36h**	5.20E-07	6.62E-07	1.26E-06	1.43E-07
**48h**	2.46E-07	1.25E-07	2.14E-08	9.60E-08
**72h**	3.78E-08	2.40E-08	5.20E-09	1.30E-08
	cgd2_1910			
**2h**	0.00E+00	0.00E+00	0.00E+00	0.00E+00
**6h**	2.76E-10	0.00E+00	3.60E-09	0.00E+00
**12h**	5.03E-08	3.58E-07	9.06E-09	1.39E-08
**24h**	2.00E-07	5.33E-08	4.83E-08	5.73E-08
**36h**	7.08E-07	1.90E-06	1.53E-08	3.61E-07

**48h**	1.67E-06	2.80E-06	1.45E-07	9.74E-07
**72h**	1.42E-05	1.25E-06	3.35E-07	1.13E-06
	cgd2_1920			
**2h**	1.30E-07	0.00E+00	0.00E+00	0.00E+00
**6h**	9.75E-06	8.94E-06	1.15E-06	1.82E-05
**12h**	5.55E-08	4.44E-07	2.18E-08	3.16E-07
**24h**	3.03E-06	2.77E-06	3.55E-07	6.09E-06
**36h**	1.75E-06	3.53E-06	1.51E-07	3.39E-06
**48h**	6.37E-06	7.98E-05	2.50E-07	5.63E-06
**72h**	6.24E-06	7.41E-06	2.15E-07	6.05E-06
	cgd2_1960			
**2h**	0.00E+00	0.00E+00	0.00E+00	0.00E+00
**6h**	0.00E+00	0.00E+00	1.46E-08	2.49E-08
**12h**	7.41E-08	1.49E-08	4.09E-08	9.21E-08
**24h**	1.25E-08	4.20E-09	2.45E-08	5.35E-08
**36h**	5.11E-08	9.73E-09	6.35E-08	1.88E-07
**48h**	6.51E-07	3.05E-08	5.89E-08	1.70E-07
**72h**	2.60E-08	1.75E-09	9.14E-09	4.81E-08
	cgd2_1330			
**2h**	9.49E-07	8.48E-08	3.29E-06	5.55E-08
**6h**	2.11E-07	8.05E-08	1.77E-05	8.59E-08
**12h**	6.56E-08	1.40E-07	2.34E-06	8.55E-08
**24h**	2.06E-07	7.96E-08	6.48E-06	1.16E-07
**36h**	1.09E-07	9.66E-08	7.99E-07	5.32E-08
**48h**	4.09E-08	1.95E-07	4.21E-06	7.35E-07
**72h**	2.70E-07	5.83E-08	5.09E-07	2.57E-07
	cgd2_1340			
**2h**	2.87E-06	6.13E-07	2.79E-06	1.07E-07
**6h**	7.08E-06	1.82E-06	4.41E-06	2.32E-08
**12h**	1.64E-07	1.70E-06	4.05E-06	4.22E-07
**24h**	2.66E-06	2.18E-06	5.88E-06	1.19E-07
**36h**	2.08E-06	3.55E-06	1.12E-06	4.80E-07
**48h**	1.78E-06	1.26E-06	1.71E-06	1.13E-05
**72h**	1.68E-06	6.55E-07	5.90E-07	4.61E-06
	cgd2_1380			
**2h**	0.00E+00	7.96E-09	0.00E+00	1.36E-09
**6h**	5.07E-09	2.92E-09	5.60E-09	6.06E-11
**12h**	0.00E+00	5.97E-10	1.94E-09	1.44E-10
**24h**	1.31E-08	3.20E-09	1.33E-08	8.32E-11
**36h**	3.59E-08	3.86E-08	1.33E-09	1.49E-09
**48h**	5.29E-08	5.80E-08	4.95E-08	1.97E-07
**72h**	7.62E-07	2.97E-07	3.08E-08	4.63E-07
	cgd2_1410			
**2h**	1.30E-02	3.34E-04	6.08E-04	3.18E-04
**6h**	6.18E-04	2.86E-04	7.13E-04	4.44E-04
**12h**	2.76E-04	3.45E-04	3.60E-04	2.54E-04
**24h**	3.42E-04	1.97E-04	4.08E-04	3.50E-04
**36h**	3.41E-04	4.02E-04	3.95E-04	4.20E-04
**48h**	4.91E-04	2.01E-04	1.89E-04	3.83E-04
**72h**	9.83E-04	1.31E-04	1.75E-04	1.91E-04
	cgd2_1420			
**2h**	6.19E-07	7.38E-08	4.61E-08	2.71E-08
**6h**	5.09E-08	1.86E-08	5.01E-08	1.51E-08

**12h**	9.10E-08	9.88E-08	8.12E-08	3.38E-08
**24h**	5.57E-08	1.89E-08	2.27E-08	2.99E-08
**36h**	8.23E-08	1.04E-07	3.93E-08	2.15E-08
**48h**	7.54E-08	2.49E-08	3.69E-08	2.26E-07
**72h**	3.27E-07	7.75E-08	1.25E-08	1.58E-07
	cgd2_1430			
**2h**	0.00E+00	0.00E+00	0.00E+00	0.00E+00
**6h**	1.19E-06	6.48E-08	6.85E-07	4.90E-07
**12h**	4.33E-08	8.78E-08	2.38E-07	0.00E+00
**24h**	9.18E-07	2.36E-07	5.26E-06	2.45E-07
**36h**	1.26E-06	6.40E-07	6.15E-07	6.96E-07
**48h**	1.48E-06	5.27E-07	1.67E-06	6.24E-06
**72h**	4.51E-06	6.12E-07	2.48E-06	5.41E-06
	cgd2_1440			
**2h**	0.00E+00	6.67E-07	2.94E-10	0.00E+00
**6h**	2.31E-07	1.65E-07	2.83E-07	2.32E-07
**12h**	5.82E-09	7.79E-09	7.11E-09	3.79E-09
**24h**	4.65E-07	8.25E-08	1.25E-07	1.35E-07
**36h**	1.25E-07	1.41E-07	1.16E-07	1.60E-07
**48h**	1.92E-07	1.98E-07	1.38E-07	4.16E-07
**72h**	2.17E-07	6.85E-08	3.14E-08	1.73E-07
	cgd2_1500			
**2h**	2.72E-06	5.02E-07	1.13E-07	4.32E-08
**6h**	1.71E-06	8.48E-07	5.45E-07	1.01E-07
**12h**	3.98E-07	7.46E-06	4.83E-07	1.63E-07
**24h**	2.48E-06	1.48E-06	3.84E-07	9.59E-08
**36h**	4.83E-07	1.56E-06	7.20E-08	1.50E-07
**48h**	4.64E-07	3.58E-07	1.70E-07	7.67E-07
**72h**	2.63E-07	8.80E-08	4.36E-08	8.55E-08
	cgd2_1590			
**2h**	2.78E-08	4.43E-09	1.78E-10	0.00E+00
**6h**	0.00E+00	3.15E-09	9.35E-11	2.58E-10
**12h**	0.00E+00	3.24E-10	2.00E-11	0.00E+00
**24h**	1.89E-09	3.10E-10	8.28E-11	4.76E-11
**36h**	3.31E-09	3.91E-09	3.40E-10	3.49E-10
**48h**	2.16E-08	6.38E-08	2.05E-08	1.19E-07
**72h**	2.84E-08	7.37E-09	4.60E-09	2.25E-08
	cgd2_1680			
**2h**	1.38E-06	5.02E-08	2.45E-07	3.08E-08
**6h**	3.56E-08	4.50E-08	2.20E-07	1.69E-08
**12h**	1.01E-08	2.03E-07	4.32E-07	4.31E-08
**24h**	1.17E-07	7.70E-08	1.32E-07	2.54E-08
**36h**	2.39E-07	2.11E-07	9.08E-08	1.52E-07
**48h**	1.01E-07	1.05E-07	1.72E-07	1.17E-06
**72h**	1.16E-07	2.00E-05	9.58E-08	8.14E-07
	cgd2_1690			
**2h**	2.69E-08	2.30E-09	5.48E-09	1.62E-09
**6h**	7.75E-07	1.55E-06	1.90E-06	8.29E-07
**12h**	4.72E-09	2.20E-08	1.93E-08	2.36E-08
**24h**	4.34E-07	3.35E-07	8.35E-07	3.67E-07
**36h**	3.65E-07	4.75E-07	3.81E-07	4.44E-07
**48h**	6.39E-08	3.02E-07	3.45E-07	6.46E-07
**72h**	1.20E-07	2.11E-08	3.71E-08	6.29E-08

	cgd2_1700			
**2h**	3.23E-08	2.97E-09	0.00E+00	0.00E+00
**6h**	8.20E-09	1.52E-09	1.01E-09	9.27E-10
**12h**	6.88E-09	2.47E-08	2.33E-08	7.10E-09
**24h**	9.27E-09	8.07E-09	3.97E-09	2.13E-09
**36h**	1.56E-07	1.63E-07	5.39E-08	4.91E-08
**48h**	2.12E-08	8.68E-08	5.66E-08	3.34E-07
**72h**	1.28E-07	5.84E-08	4.54E-08	3.69E-07
	cgd2_1710			
**2h**	2.07E-07	2.06E-04	1.96E-06	0.00E+00
**6h**	2.16E-07	1.20E-05	4.11E-05	3.76E-05
**12h**	8.77E-07	6.99E-05	1.56E-05	1.23E-05
**24h**	4.12E-06	1.31E-04	9.42E-06	9.00E-06
**36h**	2.59E-05	1.58E-04	5.25E-05	6.35E-05
**48h**	3.84E-04	1.02E-03	3.74E-04	1.31E-03
**72h**	5.67E-06	1.73E-04	3.47E-05	4.12E-04
	cgd2_1750			
**2h**	4.51E-08	3.55E-07	5.20E-07	8.68E-08
**6h**	6.22E-08	4.82E-07	3.20E-07	1.56E-07
**12h**	0.00E+00	5.40E-07	2.56E-07	1.50E-07
**24h**	2.60E-07	1.24E-06	3.25E-07	3.95E-07
**36h**	1.35E-07	2.61E-07	9.73E-08	1.56E-07
**48h**	7.67E-08	3.04E-07	2.50E-07	7.76E-07
**72h**	2.20E-08	2.30E-07	6.12E-08	8.36E-07
	cgd2_1810			
**2h**	5.56E-09	1.02E-08	5.74E-09	8.59E-09
**6h**	8.14E-08	2.38E-07	1.31E-07	1.44E-07
**12h**	0.00E+00	8.69E-09	6.11E-09	2.14E-09
**24h**	1.50E-07	1.06E-07	6.54E-08	3.12E-08
**36h**	7.67E-08	4.70E-08	2.89E-08	4.41E-08
**48h**	4.57E-08	9.48E-08	3.46E-08	1.91E-07
**72h**	2.25E-08	1.10E-08	9.60E-09	7.54E-08
	cgd2_1870			
**2h**	5.34E-07	3.15E-06	8.90E-07	3.08E-07
**6h**	2.88E-06	4.76E-06	9.13E-06	1.07E-06
**12h**	6.72E-07	2.56E-06	4.40E-06	6.31E-07
**24h**	5.37E-06	1.76E-06	5.89E-06	5.40E-07
**36h**	2.63E-06	4.32E-06	1.28E-06	6.99E-07
**48h**	8.53E-07	1.79E-06	9.08E-07	4.40E-06
**72h**	1.49E-07	1.96E-07	5.05E-08	6.56E-07
	cgd2_1880			
**2h**	6.25E-09	7.69E-08	2.20E-08	8.10E-09
**6h**	6.45E-09	2.63E-09	4.83E-09	3.61E-09
**12h**	5.66E-08	2.45E-08	7.36E-09	5.85E-09
**24h**	1.86E-08	3.12E-08	5.10E-09	3.79E-09
**36h**	3.08E-08	6.73E-08	9.70E-09	1.82E-08
**48h**	4.54E-08	2.67E-07	4.72E-08	3.38E-07
**72h**	7.64E-08	6.91E-08	4.26E-08	3.09E-07
	cgd2_1900			
**2h**	1.08E-08	9.34E-09	7.23E-08	1.24E-08
**6h**	2.06E-08	1.38E-07	1.60E-07	2.80E-08
**12h**	3.99E-09	1.60E-07	3.17E-08	8.94E-09
**24h**	8.32E-08	2.19E-07	8.18E-08	3.50E-08

**36h**	1.86E-07	6.36E-07	1.02E-07	2.00E-07
**48h**	1.50E-07	1.29E-06	2.68E-07	2.84E-06
**72h**	4.03E-07	3.96E-07	3.31E-07	1.58E-06
	cgd2_1950			
**2h**	9.15E-09	1.86E-06	3.65E-07	1.21E-07
**6h**	1.29E-07	2.03E-06	1.49E-06	1.76E-07
**12h**	7.12E-06	8.88E-07	2.92E-07	9.58E-08
**24h**	7.11E-07	1.31E-06	5.89E-07	3.00E-07
**36h**	3.09E-07	1.33E-06	8.55E-08	2.60E-07
**48h**	1.52E-07	9.14E-07	2.49E-07	1.93E-06
**72h**	6.96E-08	2.56E-07	5.51E-08	9.48E-07
	cgd2_1940			
**2h**	0.00E+00	1.38E-09	1.71E-09	0.00E+00
**6h**	5.21E-08	8.61E-08	2.30E-07	5.36E-08
**12h**	4.91E-07	1.42E-08	4.57E-09	4.92E-10
**24h**	5.76E-08	8.23E-08	4.07E-08	2.69E-08
**36h**	4.01E-08	1.18E-07	3.62E-08	5.30E-08
**48h**	2.68E-08	5.08E-08	3.67E-08	1.58E-07
**72h**	2.59E-08	2.24E-08	1.05E-08	1.18E-07
	cgd2_1980			
**2h**	9.27E-08	1.27E-08	5.76E-09	1.25E-09
**6h**	2.73E-09	3.38E-08	1.25E-08	9.17E-09
**12h**	4.17E-08	4.31E-08	6.60E-09	8.85E-11
**24h**	1.42E-08	5.10E-08	8.76E-09	2.07E-09
**36h**	4.65E-08	9.91E-08	1.79E-08	3.71E-08
**48h**	1.53E-08	5.28E-08	5.16E-08	3.09E-07
**72h**	3.33E-08	8.60E-08	1.29E-08	1.80E-07
	cgd2_2000			
**2h**	1.36E-07	1.11E-05	3.38E-08	0.00E+00
**6h**	2.30E-06	2.49E-05	1.29E-05	4.53E-05
**12h**	2.56E-06	1.67E-04	2.45E-05	1.43E-05
**24h**	5.12E-06	6.34E-05	1.30E-05	2.28E-05
**36h**	3.55E-05	1.30E-04	4.61E-05	1.64E-04
**48h**	1.52E-05	1.10E-04	4.01E-05	2.21E-04
**72h**	3.42E-05	1.92E-04	1.57E-05	1.01E-04
	cgd2_2020			
**2h**	5.83E-08	3.87E-09	8.21E-10	0.00E+00
**6h**	1.06E-07	2.95E-07	1.97E-07	7.17E-08
**12h**	7.87E-08	2.52E-08	2.69E-09	1.15E-08
**24h**	1.38E-07	7.78E-07	9.11E-08	1.03E-07
**36h**	7.43E-08	3.40E-07	2.88E-08	6.53E-08
**48h**	1.85E-08	3.18E-07	4.18E-08	2.80E-07
**72h**	1.29E-08	2.87E-08	1.13E-08	9.71E-08
	cgd2_1730			
**2h**	6.80E-08	1.08E-06	3.21E-09	3.34E-08
**6h**	1.40E-07	1.58E-06	1.24E-06	3.09E-07
**12h**	1.54E-08	3.12E-07	4.30E-08	2.00E-08
**24h**	5.30E-07	1.36E-06	4.66E-07	3.57E-07
**36h**	6.24E-07	3.14E-06	8.77E-07	1.86E-06
**48h**	4.55E-07	1.36E-06	3.56E-07	2.28E-06
**72h**	1.85E-07	1.44E-06	3.73E-07	4.54E-06
	cgd2_2060			
**2h**	2.07E-07	1.72E-05	7.70E-07	4.21E-08

**6h**	2.16E-07	2.48E-05	1.32E-06	8.28E-07
**12h**	8.77E-07	2.43E-06	3.14E-07	4.83E-08
**24h**	4.12E-06	4.17E-06	1.13E-06	3.10E-07
**36h**	2.59E-05	6.10E-06	2.25E-07	2.00E-07
**48h**	3.84E-04	4.27E-06	9.05E-07	2.89E-06
**72h**	5.67E-06	9.48E-07	2.35E-07	7.99E-07
	cgd2_2080			
**2h**	4.07E-09	3.00E-08	5.87E-08	1.35E-07
**6h**	1.28E-09	1.14E-08	5.03E-08	6.31E-08
**12h**	0.00E+00	3.94E-09	9.57E-09	2.96E-08
**24h**	8.95E-09	3.36E-09	1.53E-08	6.34E-08
**36h**	4.37E-09	1.81E-09	5.72E-09	2.82E-08
**48h**	8.49E-08	1.03E-08	2.70E-08	9.65E-08
**72h**	8.01E-09	1.06E-08	1.99E-08	3.55E-07
	cgd2_1550			
**2h**	5.56E-09	3.50E-05	2.83E-06	7.10E-08
**6h**	8.14E-08	2.40E-05	9.91E-07	9.07E-09
**12h**	0.00E+00	2.29E-05	1.76E-06	5.60E-08
**24h**	1.50E-07	9.30E-06	1.45E-06	2.80E-07
**36h**	7.67E-08	2.55E-05	8.72E-07	1.74E-07
**48h**	4.57E-08	1.62E-05	2.95E-06	7.10E-06
**72h**	2.25E-08	3.28E-06	1.63E-07	8.51E-07
	cgd2_1780			
**2h**	0.00E+00	0.00E+00	0.00E+00	0.00E+00
**6h**	2.88E-06	9.13E-04	6.04E-05	4.50E-06
**12h**	6.72E-07	2.06E-05	8.18E-07	0.00E+00
**24h**	5.37E-06	3.71E-05	2.32E-05	5.47E-06
**36h**	2.63E-06	2.66E-04	1.70E-04	3.62E-05
**48h**	8.53E-07	4.49E-05	1.02E-05	1.30E-05
**72h**	1.49E-07	6.48E-06	7.54E-07	3.59E-06
	cgd2_1820			
**2h**	6.25E-09	1.07E-07	4.00E-07	4.76E-08
**6h**	6.45E-09	5.24E-08	8.76E-08	1.93E-08
**12h**	5.66E-08	1.80E-07	1.80E-07	9.33E-08
**24h**	1.86E-08	4.98E-08	8.33E-08	3.20E-08
**36h**	3.08E-08	1.07E-07	1.18E-07	2.89E-08
**48h**	4.54E-08	8.28E-08	1.10E-07	1.79E-07
**72h**	7.64E-08	4.08E-08	1.39E-08	7.03E-08
	cgd2_1840			
**2h**	1.08E-08	1.09E-03	1.84E-04	1.85E-05
**6h**	2.06E-08	3.55E-04	1.62E-05	2.29E-06
**12h**	3.99E-09	8.97E-04	1.95E-04	8.23E-06
**24h**	8.32E-08	3.52E-04	7.27E-05	3.60E-06
**36h**	1.86E-07	1.84E-04	7.48E-05	7.34E-06
**48h**	1.50E-07	1.84E-04	1.42E-04	3.66E-04
**72h**	4.03E-07	5.93E-05	1.75E-05	4.64E-05
	cgd2_1280			
**2h**	0.00E+00	1.34E-09	0.00E+00	0.00E+00
**6h**	1.29E-07	7.10E-08	1.54E-08	1.04E-08
**12h**	0.00E+00	1.16E-08	2.70E-09	6.95E-08
**24h**	7.11E-07	4.53E-08	2.07E-08	3.75E-09
**36h**	3.09E-07	8.87E-08	4.38E-07	3.41E-09
**48h**	1.52E-07	8.51E-08	3.65E-08	1.14E-07

**72h**	6.96E-08	1.48E-08	1.10E-08	8.09E-08
	cgd2_2100			
**2h**	0.00E+00	0.00E+00	0.00E+00	0.00E+00
**6h**	0.00E+00	0.00E+00	0.00E+00	0.00E+00
**12h**	4.91E-07	4.09E-10	0.00E+00	0.00E+00
**24h**	5.76E-08	8.63E-11	0.00E+00	0.00E+00
**36h**	4.01E-08	1.58E-09	1.66E-09	2.97E-10
**48h**	2.68E-08	5.01E-08	1.57E-07	1.37E-07
**72h**	2.59E-08	6.37E-07	1.63E-06	2.48E-06
	cgd2_2130			
**2h**	9.27E-08	3.46E-08	9.62E-09	3.37E-07
**6h**	2.73E-09	1.03E-07	9.74E-08	5.64E-06
**12h**	4.17E-08	2.21E-06	1.94E-07	0.00E+00
**24h**	1.42E-08	2.54E-07	1.21E-07	1.56E-09
**36h**	4.65E-08	1.16E-06	1.60E-07	3.68E-08
**48h**	1.53E-08	5.00E-07	3.46E-07	1.51E-06
**72h**	3.33E-08	6.57E-07	6.06E-07	1.41E-06
	cgd2_2140			
**2h**	0.00E+00	1.46E-05	0.00E+00	0.00E+00
**6h**	0.00E+00	5.17E-07	0.00E+00	0.00E+00
**12h**	2.56E-06	5.72E-05	8.82E-06	0.00E+00
**24h**	5.12E-06	2.02E-06	3.56E-06	0.00E+00
**36h**	3.55E-05	8.65E-05	2.56E-05	5.18E-05
**48h**	1.52E-05	1.02E-04	4.91E-05	2.37E-04
**72h**	3.42E-05	1.53E-05	1.34E-04	7.36E-05
	cgd2_2160			
**2h**	5.83E-08	6.72E-08	8.52E-09	0.00E+00
**6h**	1.06E-07	3.51E-06	1.83E-06	1.09E-07
**12h**	7.87E-08	1.41E-07	1.62E-08	0.00E+00
**24h**	1.38E-07	1.52E-06	8.82E-07	9.25E-08
**36h**	7.43E-08	2.18E-06	2.04E-07	1.32E-07
**48h**	1.85E-08	1.50E-06	2.51E-07	2.34E-06
**72h**	1.29E-08	1.02E-07	6.47E-08	5.36E-07
	cgd2_2040			
**2h**	6.80E-08	5.14E-04	7.06E-05	1.60E-05
**6h**	1.40E-07	1.35E-04	8.12E-06	1.25E-06
**12h**	1.54E-08	4.85E-05	5.15E-06	3.35E-06
**24h**	5.30E-07	3.78E-05	4.02E-06	3.43E-07
**36h**	6.24E-07	2.15E-04	1.28E-04	9.53E-07
**48h**	4.55E-07	6.61E-05	1.63E-06	5.67E-06
**72h**	1.85E-07	1.14E-04	9.44E-06	1.86E-05
	cgd2_2210			
**2h**	1.08E-07	1.18E-07	5.87E-08	1.30E-06
**6h**	1.54E-06	2.84E-06	2.40E-06	2.05E-06
**12h**	4.25E-09	1.36E-06	1.35E-07	8.16E-07
**24h**	3.55E-06	2.09E-06	6.99E-07	3.76E-06
**36h**	1.04E-06	3.46E-06	1.21E-07	1.79E-06
**48h**	1.93E-06	5.85E-06	1.84E-06	6.79E-06
**72h**	1.95E-06	2.00E-06	4.30E-07	4.80E-06
	cgd2_2220			
**2h**	6.50E-08	2.82E-06	1.41E-05	1.30E-06
**6h**	1.05E-05	5.60E-05	2.24E-05	4.75E-05
**12h**	7.40E-07	8.54E-06	2.03E-07	2.89E-06

**24h**	7.70E-05	4.27E-05	6.60E-06	3.60E-05
**36h**	6.07E-06	2.50E-05	2.16E-06	1.96E-05
**48h**	3.56E-06	1.60E-05	4.57E-06	2.05E-05
**72h**	3.36E-06	1.55E-05	7.98E-07	2.36E-05
	cgd2_2230			
**2h**	1.54E-06	2.45E-06	4.34E-07	1.97E-07
**6h**	3.01E-07	3.65E-07	1.87E-07	3.02E-08
**12h**	4.35E-07	2.76E-06	3.15E-07	9.36E-08
**24h**	1.20E-06	3.80E-07	6.75E-08	9.34E-08
**36h**	3.88E-07	9.38E-07	2.90E-08	5.65E-08
**48h**	8.79E-07	3.06E-07	9.67E-08	8.45E-07
**72h**	2.38E-07	6.19E-08	3.77E-08	2.61E-07
	cgd2_2260			
**2h**	1.11E-05	3.65E-04	8.55E-05	3.76E-05
**6h**	4.46E-06	1.29E-05	7.33E-07	2.98E-06
**12h**	1.65E-05	2.54E-04	3.77E-05	5.36E-05
**24h**	7.63E-05	1.77E-05	4.20E-06	1.34E-05
**36h**	2.27E-06	5.73E-05	3.00E-06	1.05E-05
**48h**	6.77E-06	6.80E-06	2.00E-06	3.36E-05
**72h**	2.27E-06	1.03E-05	6.05E-07	2.45E-05
	cgd2_2270			
**2h**	8.24E-08	4.49E-08	2.07E-07	1.09E-08
**6h**	1.17E-07	3.75E-08	4.34E-08	1.85E-08
**12h**	1.76E-08	4.75E-08	9.75E-09	9.03E-09
**24h**	2.29E-07	6.47E-08	1.94E-08	2.72E-08
**36h**	5.32E-08	1.98E-07	2.35E-08	4.91E-08
**48h**	2.38E-07	5.63E-08	2.17E-08	2.40E-07
**72h**	6.15E-08	3.06E-08	6.84E-09	1.28E-07
	cgd2_2280			
**2h**	1.29E-06	9.41E-07	6.71E-07	0.00E+00
**6h**	2.93E-09	7.50E-08	3.13E-08	0.00E+00
**12h**	1.09E-06	3.13E-05	4.45E-07	1.16E-06
**24h**	2.23E-05	8.92E-06	9.70E-07	7.33E-07
**36h**	2.42E-06	4.06E-05	2.91E-07	3.70E-06
**48h**	5.90E-06	4.51E-05	3.12E-06	5.19E-05
**72h**	9.82E-07	4.57E-06	4.07E-07	3.16E-05
	cgd2_2290			
**2h**	1.11E-07	1.93E-07	9.07E-08	3.24E-08
**6h**	6.77E-08	1.66E-07	6.00E-08	2.21E-08
**12h**	5.66E-09	3.57E-07	7.35E-08	2.01E-08
**24h**	5.79E-07	2.85E-07	4.19E-08	3.89E-08
**36h**	1.10E-07	3.31E-07	9.10E-09	3.10E-08
**48h**	1.23E-07	8.80E-08	4.83E-08	5.08E-07
**72h**	5.50E-08	6.86E-08	1.24E-08	6.74E-07
	cgd2_2320			
**2h**	1.41E-09	1.28E-08	2.08E-08	0.00E+00
**6h**	2.13E-08	3.85E-09	6.06E-07	0.00E+00
**12h**	0.00E+00	3.90E-08	5.63E-09	1.19E-09
**24h**	2.23E-08	5.14E-09	3.66E-09	1.53E-10
**36h**	2.69E-08	1.71E-07	1.31E-08	5.18E-09
**48h**	4.48E-08	3.06E-08	7.19E-09	5.09E-08
**72h**	3.64E-08	1.38E-08	7.86E-09	1.22E-07
	cgd2_2340			

**2h**	0.00E+00	0.00E+00	5.78E-08	3.92E-09
**6h**	0.00E+00	2.59E-09	1.21E-08	9.12E-11
**12h**	4.21E-08	2.10E-07	1.91E-08	5.68E-08
**24h**	8.35E-08	1.71E-08	9.33E-09	3.96E-09
**36h**	6.76E-08	3.96E-07	1.84E-08	1.42E-07
**48h**	1.62E-07	9.92E-08	9.69E-08	5.85E-07
**72h**	4.87E-07	2.79E-07	3.90E-08	1.40E-06
	cgd2_2350			
**2h**	1.82E-07	7.96E-08	1.31E-03	0.00E+00
**6h**	8.21E-08	7.11E-08	4.49E-05	2.28E-08
**12h**	3.99E-08	7.30E-07	3.78E-06	3.27E-08
**24h**	4.84E-06	8.25E-08	2.26E-07	5.01E-09
**36h**	1.42E-07	3.84E-07	8.18E-05	1.78E-08
**48h**	1.12E-07	1.53E-07	6.67E-08	2.23E-07
**72h**	1.34E-07	1.01E-07	1.31E-06	5.20E-07
	cgd2_2360			
**2h**	1.07E-08	8.03E-08	1.34E-06	0.00E+00
**6h**	1.61E-06	2.74E-06	6.62E-07	6.83E-08
**12h**	5.89E-09	2.16E-06	3.54E-08	1.86E-08
**24h**	5.30E-06	1.47E-06	5.11E-07	6.52E-08
**36h**	6.86E-06	6.12E-06	3.60E-08	1.25E-07
**48h**	4.79E-07	1.33E-06	3.15E-07	2.62E-06
**72h**	7.45E-07	5.49E-07	4.77E-08	1.77E-06
	cgd2_2380			
**2h**	6.82E-08	0.00E+00	0.00E+00	0.00E+00
**6h**	1.16E-06	1.57E-05	8.95E-05	9.58E-06
**12h**	7.95E-06	1.36E-04	1.04E-05	1.08E-05
**24h**	1.67E-04	5.50E-05	2.11E-05	5.44E-05
**36h**	1.22E-05	1.06E-04	2.20E-05	1.04E-05
**48h**	4.55E-06	1.20E-04	1.94E-05	1.96E-04
**72h**	6.32E-06	2.68E-05	3.26E-06	8.80E-05
	cgd2_2390			
**2h**	2.99E-08	1.81E-08	7.01E-08	1.51E-08
**6h**	6.33E-08	6.91E-08	1.01E-07	9.72E-08
**12h**	1.42E-08	1.11E-08	1.84E-08	3.74E-08
**24h**	4.02E-08	4.22E-08	5.12E-08	8.90E-08
**36h**	4.14E-08	5.35E-08	4.70E-08	6.71E-08
**48h**	9.56E-08	4.34E-08	5.28E-08	1.05E-07
**72h**	2.51E-08	5.34E-09	1.12E-08	5.30E-08
	cgd2_2410			
**2h**	4.85E-07	1.15E-06	1.36E-06	3.23E-08
**6h**	8.29E-07	3.33E-07	3.39E-07	2.40E-08
**12h**	1.68E-07	1.19E-06	3.43E-07	6.44E-08
**24h**	8.92E-07	2.63E-07	5.77E-07	1.00E-07
**36h**	9.13E-07	1.22E-06	2.22E-07	1.77E-07
**48h**	7.45E-07	4.97E-07	3.20E-07	2.51E-06
**72h**	2.30E-07	1.80E-07	1.21E-07	7.35E-07
	cgd2_2430			
**2h**	9.81E-07	1.67E-06	2.00E-07	2.29E-08
**6h**	3.24E-06	1.47E-06	5.25E-07	1.26E-08
**12h**	3.23E-07	1.21E-06	8.69E-07	1.44E-07
**24h**	3.65E-06	1.03E-06	6.79E-07	3.71E-08
**36h**	3.56E-06	3.45E-06	2.84E-07	4.33E-07

**48h**	2.73E-06	1.46E-06	3.81E-07	3.63E-06
**72h**	1.29E-06	4.31E-07	1.52E-07	8.47E-07
	cgd2_2450			
**2h**	6.03E-07	1.09E-06	1.90E-07	2.16E-08
**6h**	2.05E-07	8.35E-08	3.20E-08	3.59E-09
**12h**	4.39E-07	3.99E-07	1.37E-07	1.27E-07
**24h**	5.01E-07	5.95E-08	6.78E-08	9.50E-09
**36h**	4.87E-07	1.02E-06	8.33E-08	8.18E-08
**48h**	1.05E-07	1.92E-07	1.26E-07	3.42E-07
**72h**	8.99E-07	6.43E-08	5.91E-08	3.78E-07
	cgd2_2460			
**2h**	1.13E-07	9.03E-08	5.69E-08	1.51E-08
**6h**	1.05E-07	8.29E-08	6.18E-08	1.32E-07
**12h**	1.85E-07	4.46E-08	1.43E-07	1.72E-07
**24h**	6.71E-08	5.07E-08	1.05E-07	7.51E-08
**36h**	1.32E-07	2.03E-07	1.67E-07	1.24E-07
**48h**	5.26E-08	7.50E-08	9.03E-08	2.00E-07
**72h**	8.61E-08	5.10E-08	3.62E-08	1.77E-07
	cgd2_2480			
**2h**	1.25E-04	5.50E-06	9.89E-05	0.00E+00
**6h**	1.20E-04	2.73E-04	1.44E-04	2.07E-04
**12h**	5.64E-04	2.39E-05	1.68E-04	1.66E-04
**24h**	1.37E-04	4.40E-05	3.42E-04	2.07E-05
**36h**	1.18E-04	4.62E-05	1.38E-04	3.57E-05
**48h**	4.23E-05	2.98E-05	2.49E-05	5.73E-05
**72h**	4.67E-05	8.18E-06	6.56E-05	3.90E-05
	cgd2_2490			
**2h**	0.00E+00	0.00E+00	0.00E+00	0.00E+00
**6h**	0.00E+00	0.00E+00	2.60E-08	0.00E+00
**12h**	0.00E+00	8.43E-10	0.00E+00	0.00E+00
**24h**	5.62E-09	1.92E-09	4.30E-09	9.83E-09
**36h**	2.68E-09	4.24E-09	1.04E-08	3.91E-08
**48h**	4.51E-09	2.30E-08	2.26E-08	4.77E-08
**72h**	2.72E-08	6.74E-08	7.89E-09	2.22E-07
	cgd2_2500			
**2h**	0.00E+00	0.00E+00	0.00E+00	0.00E+00
**6h**	1.55E-04	2.64E-05	2.04E-05	1.42E-06
**12h**	0.00E+00	5.28E-06	2.97E-06	0.00E+00
**24h**	1.47E-04	1.96E-05	1.19E-05	4.32E-06
**36h**	1.34E-04	4.51E-04	2.59E-05	7.87E-05
**48h**	5.65E-05	1.79E-04	1.17E-05	1.08E-04
**72h**	1.20E-04	1.63E-04	2.61E-05	1.27E-04
	cgd2_2510			
**2h**	3.08E-07	0.00E+00	0.00E+00	0.00E+00
**6h**	0.00E+00	4.50E-07	2.46E-07	0.00E+00
**12h**	0.00E+00	6.79E-08	0.00E+00	0.00E+00
**24h**	3.44E-07	1.57E-07	6.15E-08	1.12E-07
**36h**	2.94E-06	7.04E-07	1.54E-07	1.25E-07
**48h**	8.23E-05	1.41E-04	5.58E-05	7.60E-05
**72h**	6.48E-06	4.83E-06	1.91E-06	7.43E-06
	cgd2_2530			
**2h**	4.02E-03	1.15E-03	1.82E-04	9.62E-06
**6h**	4.94E-04	3.84E-04	7.35E-05	1.13E-05

**12h**	7.08E-03	6.06E-04	7.58E-04	7.92E-04
**24h**	1.23E-03	7.46E-05	4.58E-04	1.15E-04
**36h**	3.03E-03	2.33E-03	1.61E-03	6.97E-04
**48h**	2.59E-03	1.16E-03	8.82E-04	2.74E-03
**72h**	4.30E-04	7.97E-04	6.46E-05	8.78E-05
	cgd2_2540			
**2h**	5.80E-07	1.80E-06	6.00E-07	1.36E-07
**6h**	9.71E-08	9.66E-08	4.28E-08	7.20E-08
**12h**	5.06E-06	5.68E-06	4.74E-06	2.67E-06
**24h**	7.31E-07	8.43E-07	6.27E-07	2.73E-07
**36h**	1.10E-06	1.66E-06	4.32E-07	2.93E-07
**48h**	7.62E-07	1.40E-06	3.40E-07	1.33E-06
**72h**	1.55E-07	1.65E-07	1.93E-07	3.39E-07
	cgd2_2580			
**2h**	1.85E-04	4.94E-04	9.33E-05	6.91E-07
**6h**	2.05E-04	6.45E-05	7.70E-07	7.23E-06
**12h**	1.10E-03	2.50E-04	1.34E-04	2.84E-05
**24h**	1.92E-04	8.92E-05	1.31E-04	2.54E-06
**36h**	4.76E-04	1.15E-03	5.73E-04	1.55E-04
**48h**	8.37E-04	3.44E-04	2.12E-04	7.27E-04
**72h**	8.73E-04	1.91E-04	2.39E-04	0.00E+00
	cgd2_2590			
**2h**	1.36E-07	1.50E-07	4.07E-08	3.69E-09
**6h**	7.60E-09	1.46E-08	4.56E-09	0.00E+00
**12h**	1.27E-07	7.92E-07	9.63E-08	1.93E-08
**24h**	2.53E-07	4.96E-08	3.20E-08	2.84E-09
**36h**	8.66E-07	7.16E-07	5.02E-08	3.80E-08
**48h**	9.63E-07	4.45E-07	1.56E-07	1.71E-06
**72h**	1.94E-07	7.85E-08	4.92E-08	7.18E-07
	cgd2_2610			
**2h**	4.44E-07	2.13E-06	0.00E+00	2.14E-08
**6h**	5.32E-08	9.75E-07	5.79E-08	0.00E+00
**12h**	1.32E-07	4.51E-07	2.25E-09	0.00E+00
**24h**	3.69E-06	5.35E-07	1.97E-07	2.07E-07
**36h**	1.44E-06	1.17E-06	4.33E-06	4.15E-08
**48h**	6.69E-06	9.02E-06	2.54E-06	9.41E-06
**72h**	5.43E-06	1.29E-06	4.34E-07	8.36E-06
	cgd2_2620			
**2h**	9.21E-08	2.20E-07	3.43E-08	2.18E-09
**6h**	5.08E-08	8.06E-08	7.30E-08	9.55E-10
**12h**	2.09E-09	1.39E-07	1.84E-08	6.74E-10
**24h**	1.83E-07	4.05E-08	3.10E-08	2.61E-08
**36h**	9.03E-08	1.44E-07	2.41E-08	2.45E-08
**48h**	1.20E-07	1.06E-07	7.52E-08	2.79E-07
**72h**	3.92E-08	1.65E-08	4.11E-09	1.63E-07
	cgd2_2630			
**2h**	9.57E-04	1.65E-03	2.10E-03	1.47E-04
**6h**	6.74E-04	4.03E-04	2.78E-04	3.52E-05
**12h**	1.06E-03	1.77E-03	3.65E-04	3.16E-04
**24h**	7.82E-04	7.90E-06	2.60E-04	1.27E-04
**36h**	1.25E-03	5.75E-04	2.88E-04	5.89E-05
**48h**	2.08E-03	7.89E-04	7.96E-04	2.09E-05
**72h**	1.02E-03	1.95E-04	3.14E-04	4.91E-04

	cgd2_2650			
**2h**	3.89E-07	3.05E-07	3.81E-07	1.00E-07
**6h**	1.17E-07	1.48E-07	8.16E-09	1.58E-08
**12h**	1.85E-07	5.36E-07	1.63E-07	4.41E-08
**24h**	1.28E-06	1.88E-07	7.31E-08	1.43E-07
**36h**	3.52E-07	1.91E-07	2.97E-08	4.37E-08
**48h**	1.62E-07	1.15E-07	7.69E-08	3.07E-07
**72h**	7.56E-07	1.62E-07	6.22E-08	7.66E-07
	cgd2_2660			
**2h**	1.81E-05	2.21E-05	4.01E-06	6.13E-07
**6h**	2.19E-06	2.32E-06	9.02E-07	1.18E-07
**12h**	8.32E-06	2.10E-05	6.81E-06	1.80E-06
**24h**	9.48E-06	3.54E-06	1.96E-06	4.23E-07
**36h**	6.53E-06	5.95E-06	4.23E-07	3.38E-07
**48h**	3.13E-06	2.10E-06	1.12E-06	7.14E-06
**72h**	2.16E-06	5.00E-07	2.53E-07	2.04E-06
	cgd2_2670			
**2h**	1.13E-06	1.32E-06	4.08E-07	6.18E-08
**6h**	1.16E-07	1.41E-07	1.31E-07	4.44E-08
**12h**	3.55E-08	4.91E-07	1.83E-07	1.30E-08
**24h**	8.04E-07	4.19E-07	2.08E-07	1.63E-07
**36h**	8.68E-07	4.73E-07	1.09E-07	2.48E-07
**48h**	5.13E-07	2.74E-07	3.04E-07	1.42E-06
**72h**	3.93E-07	2.18E-07	7.49E-08	8.30E-07
	cgd2_2680			
**2h**	3.05E-06	1.84E-06	7.74E-07	2.38E-08
**6h**	4.62E-07	4.26E-07	4.31E-07	2.61E-08
**12h**	1.10E-07	2.18E-06	7.13E-07	2.79E-07
**24h**	1.12E-06	2.03E-07	1.19E-07	5.03E-08
**36h**	2.51E-06	6.43E-07	4.37E-08	6.05E-08
**48h**	2.51E-07	1.20E-07	2.45E-07	8.51E-07
**72h**	3.35E-07	1.07E-07	8.40E-08	1.21E-06
	cgd2_2690			
**2h**	3.39E-04	8.54E-04	7.77E-04	6.99E-04
**6h**	1.62E-04	1.77E-04	2.02E-04	3.64E-04
**12h**	1.03E-04	1.20E-04	1.85E-04	3.38E-04
**24h**	5.76E-05	3.25E-05	1.35E-04	8.85E-05
**36h**	2.11E-04	1.98E-04	1.54E-04	2.50E-05
**48h**	4.55E-05	3.90E-05	4.65E-05	5.52E-05
**72h**	1.15E-04	4.80E-05	3.54E-05	5.57E-05
	cgd2_2700			
**2h**	8.71E-09	2.03E-08	1.62E-08	0.00E+00
**6h**	1.56E-06	1.05E-06	2.99E-06	1.65E-08
**12h**	4.88E-08	1.45E-07	3.58E-08	8.79E-09
**24h**	2.96E-06	3.62E-07	1.69E-06	8.24E-08
**36h**	8.24E-07	5.29E-07	4.05E-07	1.97E-07
**48h**	8.20E-07	4.10E-07	6.18E-07	8.11E-07
**72h**	1.30E-07	9.56E-08	4.40E-08	8.40E-07
	cgd2_2710			
**2h**	3.02E-04	6.05E-04	8.78E-05	2.22E-01
**6h**	2.76E-04	7.03E-05	1.37E-05	0.00E+00
**12h**	6.40E-04	3.61E-05	1.55E-04	5.86E-02
**24h**	3.95E-05	6.03E-05	3.61E-05	8.13E-02

**36h**	1.58E-04	1.63E-04	3.23E-05	2.93E-03
**48h**	5.53E-05	1.53E-05	3.40E-05	6.29E-03
**72h**	1.12E-04	4.32E-05	7.10E-05	4.48E-03
	cgd2_2720			
**2h**	7.81E-07	2.03E-07	6.65E-08	7.75E-09
**6h**	1.05E-09	1.15E-08	8.66E-09	0.00E+00
**12h**	1.03E-07	1.20E-06	6.47E-08	4.09E-09
**24h**	1.24E-07	3.98E-08	2.51E-08	3.63E-09
**36h**	3.17E-07	4.63E-07	2.88E-08	4.60E-08
**48h**	2.47E-07	2.45E-07	8.44E-08	3.77E-07
**72h**	3.03E-07	2.03E-07	7.62E-08	2.02E-06
	cgd2_2730			
**2h**	3.45E-06	4.56E-06	1.19E-06	4.36E-05
**6h**	2.27E-06	3.60E-06	1.34E-06	4.06E-05
**12h**	1.96E-06	9.55E-06	8.42E-07	4.84E-03
**24h**	3.78E-06	1.29E-06	7.47E-07	0.00E+00
**36h**	5.74E-06	2.94E-06	6.22E-08	9.69E-05
**48h**	4.07E-06	1.06E-06	5.30E-07	2.86E-05
**72h**	7.58E-07	9.34E-08	1.46E-07	7.17E-05
	cgd2_2750			
**2h**	8.93E-06	1.05E-05	1.52E-05	0.00E+00
**6h**	2.00E-06	5.99E-06	2.35E-06	2.34E-03
**12h**	1.36E-06	1.42E-05	2.90E-06	0.00E+00
**24h**	5.06E-06	3.34E-06	2.30E-06	0.00E+00
**36h**	7.34E-06	1.14E-05	7.53E-07	3.49E-03
**48h**	5.91E-06	6.53E-06	3.80E-06	0.00E+00
**72h**	5.55E-05	1.03E-05	1.66E-05	0.00E+00
	cgd2_2760			
**2h**	0.00E+00	0.00E+00	0.00E+00	0.00E+00
**6h**	2.15E-08	2.28E-08	1.64E-07	0.00E+00
**12h**	1.88E-09	1.46E-07	2.58E-08	1.41E-03
**24h**	1.24E-07	5.99E-08	5.28E-08	0.00E+00
**36h**	3.02E-07	6.58E-07	2.50E-07	4.05E-04
**48h**	3.88E-07	3.11E-07	4.93E-07	1.42E-04
**72h**	2.49E-07	1.77E-07	1.76E-07	4.65E-05
	cgd2_2770			
**2h**	2.94E-09	5.11E-08	3.35E-08	0.00E+00
**6h**	2.74E-07	8.08E-07	3.98E-07	0.00E+00
**12h**	1.70E-08	2.29E-07	1.90E-08	0.00E+00
**24h**	3.81E-07	8.51E-08	8.75E-08	0.00E+00
**36h**	3.42E-07	7.78E-07	6.66E-08	2.46E-03
**48h**	2.93E-07	1.85E-07	8.40E-08	0.00E+00
**72h**	4.08E-07	8.18E-08	4.49E-08	0.00E+00
	cgd2_2780			
**2h**	0.00E+00	1.05E-06	3.69E-06	0.00E+00
**6h**	7.16E-06	7.37E-06	2.86E-05	0.00E+00
**12h**	0.00E+00	2.53E-06	5.56E-07	0.00E+00
**24h**	2.37E-05	1.40E-05	4.43E-06	0.00E+00
**36h**	1.82E-05	1.37E-05	4.90E-06	0.00E+00
**48h**	7.80E-06	1.69E-05	6.69E-06	1.97E-03
**72h**	5.45E-05	6.34E-06	1.88E-05	0.00E+00
	cgd2_2790			
**2h**	4.60E-07	7.77E-08	2.24E-08	6.28E-06

**6h**	1.07E-07	1.84E-07	3.79E-08	5.99E-06
**12h**	9.08E-08	2.38E-07	1.44E-07	3.00E-06
**24h**	1.83E-07	1.16E-07	7.23E-08	6.14E-06
**36h**	7.33E-08	9.48E-08	5.25E-09	4.90E-06
**48h**	4.74E-08	3.96E-08	1.59E-08	0.00E+00
**72h**	6.98E-08	1.47E-08	3.40E-09	4.68E-05
	cgd2_2800			
**2h**	2.05E-07	5.36E-08	5.01E-08	0.00E+00
**6h**	8.59E-08	7.52E-08	1.10E-07	2.58E-02
**12h**	1.45E-08	1.84E-07	6.52E-08	1.68E-02
**24h**	1.16E-07	3.20E-08	4.75E-08	2.41E-05
**36h**	5.33E-08	1.01E-07	1.06E-07	3.35E-06
**48h**	5.32E-08	1.34E-07	3.26E-08	2.34E-05
**72h**	3.94E-08	2.63E-08	1.24E-08	5.72E-06
	cgd2_2810			
**2h**	3.36E-05	1.20E-04	1.90E-05	1.06E-03
**6h**	6.36E-04	1.98E-04	6.64E-05	1.75E-03
**12h**	2.35E-05	1.75E-04	2.47E-05	0.00E+00
**24h**	1.30E-04	5.09E-05	2.63E-05	3.79E-04
**36h**	4.12E-05	1.49E-04	9.21E-06	1.10E-03
**48h**	4.33E-05	1.68E-05	1.18E-05	1.74E-03
**72h**	4.32E-05	4.23E-05	1.48E-05	1.07E-04
	cgd2_2840			
**2h**	1.67E-08	3.12E-08	2.00E-09	2.39E-04
**6h**	1.17E-07	1.30E-07	5.81E-08	0.00E+00
**12h**	7.39E-08	9.21E-07	7.82E-08	0.00E+00
**24h**	2.67E-07	1.26E-07	1.01E-07	6.96E-05
**36h**	2.87E-07	5.09E-07	2.79E-08	7.78E-05
**48h**	4.00E-07	1.45E-07	1.38E-07	7.31E-04
**72h**	2.82E-07	1.50E-07	4.60E-08	2.12E-05
	cgd2_2860			
**2h**	1.90E-08	5.75E-08	1.93E-08	0.00E+00
**6h**	3.04E-09	1.20E-09	1.23E-08	4.71E-06
**12h**	3.74E-08	1.43E-07	1.12E-07	1.23E-03
**24h**	3.63E-08	2.84E-08	1.69E-08	6.97E-05
**36h**	5.62E-08	6.68E-08	5.42E-08	1.33E-05
**48h**	5.59E-08	3.21E-08	1.21E-07	1.36E-05
**72h**	5.35E-08	7.92E-08	1.91E-08	3.14E-05
	cgd2_2880			
**2h**	1.31E-05	4.00E-06	9.94E-07	0.00E+00
**6h**	2.64E-06	9.36E-07	3.41E-06	0.00E+00
**12h**	2.52E-07	1.19E-06	1.39E-07	0.00E+00
**24h**	5.71E-06	3.99E-06	3.47E-06	0.00E+00
**36h**	5.65E-06	8.49E-06	1.17E-05	2.10E-03
**48h**	9.03E-06	4.85E-06	3.27E-05	3.91E-03
**72h**	0.00E+00	1.50E-05	3.08E-05	0.00E+00
	cgd2_2890			
**2h**	1.69E-08	1.74E-08	6.85E-09	2.72E-05
**6h**	4.97E-07	1.26E-06	1.76E-06	3.17E-02
**12h**	1.81E-08	3.82E-07	8.33E-08	2.90E-04
**24h**	1.23E-06	1.82E-07	4.00E-07	2.14E-04
**36h**	5.82E-07	7.83E-07	6.24E-07	2.26E-04
**48h**	2.41E-06	1.81E-07	7.95E-07	9.31E-05

**72h**	4.16E-07	1.42E-07	5.66E-07	1.28E-03
	cgd5_1910			
**2h**	2.45E-07	1.20E-07	6.36E-08	8.19E-08
**6h**	4.58E-09	0.00E+00	7.53E-10	5.38E-08
**12h**	2.61E-07	6.03E-07	2.82E-07	5.99E-07
**24h**	1.34E-07	2.46E-07	2.29E-07	1.75E-07
**36h**	1.41E-06	2.34E-07	3.20E-07	6.07E-07
**48h**	3.39E-07	2.81E-07	2.09E-07	1.12E-07
**72h**	2.28E-07	9.91E-08	1.23E-07	1.27E-07
	cgd5_1920			
**2h**	4.98E-08	0.00E+00	5.78E-09	9.27E-09
**6h**	6.09E-08	4.96E-08	3.26E-08	4.73E-08
**12h**	3.09E-07	2.99E-06	9.82E-07	3.46E-07
**24h**	2.50E-07	3.62E-07	3.50E-07	6.31E-07
**36h**	3.10E-06	1.36E-06	5.61E-07	7.49E-07
**48h**	4.65E-07	1.50E-06	6.48E-07	1.06E-07
**72h**	6.72E-07	1.19E-06	6.09E-07	1.21E-07
	cgd5_1820			
**2h**	5.73E-06	2.04E-06	3.18E-07	5.50E-07
**6h**	3.86E-04	5.25E-05	5.37E-05	1.69E-04
**12h**	2.01E-05	6.84E-06	2.78E-06	1.57E-05
**24h**	1.56E-04	2.75E-05	2.19E-05	1.36E-04
**36h**	8.19E-05	3.29E-05	2.69E-05	6.06E-05
**48h**	9.34E-05	2.64E-05	1.39E-05	3.02E-05
**72h**	6.01E-06	5.92E-06	2.66E-06	3.29E-06
	cgd5_1850			
**2h**	1.51E-04	3.54E-05	3.65E-05	2.50E-04
**6h**	2.20E-04	4.41E-05	2.14E-05	1.10E-04
**12h**	9.40E-05	5.12E-05	4.82E-06	1.13E-04
**24h**	1.44E-04	6.64E-05	4.65E-05	1.52E-04
**36h**	1.71E-04	4.87E-05	3.72E-05	1.06E-04
**48h**	3.16E-04	2.25E-05	1.13E-05	1.88E-05
**72h**	4.21E-05	4.53E-06	4.72E-06	6.37E-06
	cgd5_1860			
**2h**	1.98E-07	1.05E-06	8.04E-07	1.08E-06
**6h**	4.49E-08	3.86E-07	1.02E-07	2.08E-07
**12h**	4.22E-07	4.20E-07	5.39E-08	8.23E-07
**24h**	2.10E-07	8.81E-07	9.27E-07	4.64E-07
**36h**	1.76E-07	4.93E-07	3.86E-07	3.47E-07
**48h**	5.22E-07	1.76E-07	1.17E-07	8.84E-08
**72h**	1.08E-08	1.18E-07	4.46E-08	3.12E-08
	cgd5_1870			
**2h**	1.21E-08	6.64E-09	2.56E-09	2.79E-08
**6h**	1.55E-07	2.18E-07	1.20E-07	1.80E-07
**12h**	2.81E-08	3.59E-08	1.01E-08	4.95E-08
**24h**	3.89E-07	5.82E-08	1.56E-07	4.25E-07
**36h**	2.13E-07	5.27E-07	1.88E-07	2.85E-07
**48h**	2.37E-07	1.51E-07	9.06E-08	1.63E-07
**72h**	1.60E-08	7.82E-08	2.25E-08	3.43E-08
	cgd5_1880			
**2h**	5.31E-07	2.64E-06	1.66E-06	3.10E-06
**6h**	5.82E-05	6.77E-05	2.17E-05	4.82E-05
**12h**	4.87E-06	1.62E-06	1.47E-06	8.27E-06

**24h**	3.48E-05	4.56E-06	8.45E-06	5.42E-05
**36h**	1.90E-05	9.84E-06	1.38E-05	9.70E-06
**48h**	1.08E-05	6.47E-06	7.56E-06	1.26E-05
**72h**	4.89E-07	2.30E-06	3.72E-07	4.51E-07
	cgd5_1890			
**2h**	2.84E-04	8.17E-05	6.76E-05	4.00E-04
**6h**	7.90E-04	1.22E-04	9.49E-05	1.28E-03
**12h**	2.72E-04	1.71E-05	3.61E-06	2.30E-04
**24h**	9.29E-04	4.58E-05	7.78E-05	1.16E-03
**36h**	8.20E-04	9.35E-05	1.08E-04	3.24E-04
**48h**	6.10E-04	4.95E-05	7.20E-05	1.38E-04
**72h**	6.63E-05	2.84E-05	1.10E-05	2.38E-05
	cgd5_1900			
**2h**	5.63E-07	1.30E-06	3.54E-06	2.28E-06
**6h**	8.03E-07	8.91E-07	1.02E-06	9.97E-07
**12h**	7.41E-07	3.31E-06	1.21E-06	2.05E-06
**24h**	1.17E-06	1.12E-06	2.95E-06	2.03E-06
**36h**	1.72E-06	6.73E-07	5.66E-07	5.70E-07
**48h**	1.50E-06	7.04E-07	4.24E-07	3.45E-07
**72h**	3.45E-07	2.06E-07	1.77E-07	7.20E-08
	cgd5_1780			
**2h**	5.29E-07	9.94E-07	4.17E-07	1.06E-06
**6h**	4.20E-07	2.86E-07	3.73E-07	7.98E-07
**12h**	3.40E-06	1.06E-05	2.32E-06	4.02E-06
**24h**	9.34E-07	5.43E-06	3.64E-06	2.69E-06
**36h**	3.32E-06	1.55E-06	1.61E-06	4.21E-06
**48h**	1.21E-06	2.60E-06	4.02E-07	3.30E-07
**72h**	1.07E-07	3.09E-07	2.00E-07	1.73E-07
	cgd5_1800			
**2h**	7.19E-06	2.05E-05	1.24E-05	4.41E-05
**6h**	6.56E-05	1.53E-05	1.94E-05	4.78E-05
**12h**	1.11E-04	3.54E-05	1.50E-05	1.07E-04
**24h**	2.76E-04	5.24E-05	7.47E-05	3.19E-04
**36h**	2.34E-04	2.41E-05	3.23E-05	1.28E-04
**48h**	2.72E-04	6.22E-04	4.49E-05	1.28E-04
**72h**	2.11E-04	2.85E-05	2.59E-05	6.05E-05
	cgd5_1810			
**2h**	9.11E-08	1.75E-07	1.01E-07	2.88E-07
**6h**	7.21E-07	9.60E-07	5.21E-07	4.59E-07
**12h**	1.04E-07	1.32E-07	9.53E-08	4.16E-07
**24h**	1.09E-06	9.80E-07	5.73E-07	1.09E-06
**36h**	9.04E-07	4.50E-07	1.39E-06	8.61E-07
**48h**	6.49E-06	8.80E-06	2.40E-06	1.18E-06
**72h**	4.32E-07	8.34E-07	3.50E-07	1.93E-07
	cgd1_1920			
**2h**	8.96E-09	0.00E+00	0.00E+00	2.63E-08
**6h**	0.00E+00	3.57E-09	4.45E-08	8.68E-09
**12h**	4.80E-07	2.80E-07	1.47E-07	1.65E-07
**24h**	1.60E-07	7.87E-08	1.41E-07	5.91E-08
**36h**	1.02E-06	5.59E-07	1.66E-06	7.45E-07
**48h**	2.48E-06	1.34E-06	4.09E-06	1.49E-06
**72h**	1.20E-06	2.71E-07	4.10E-06	7.59E-07
	cgd1_1930			

**2h**	6.14E-09	0.00E+00	8.96E-09	5.64E-09
**6h**	1.77E-07	5.23E-08	4.47E-07	1.46E-07
**12h**	3.67E-08	1.12E-08	1.17E-07	5.18E-08
**24h**	1.38E-07	1.80E-08	2.20E-07	1.31E-07
**36h**	1.27E-07	3.93E-08	3.88E-07	1.00E-07
**48h**	1.54E-07	1.55E-07	7.02E-07	1.51E-07
**72h**	4.76E-08	3.03E-08	5.80E-07	1.86E-07
	cgd1_1940			
**2h**	1.13E-06	1.06E-07	2.10E-06	1.71E-06
**6h**	2.59E-05	2.72E-05	5.33E-05	2.55E-05
**12h**	7.53E-06	5.74E-06	2.02E-05	7.76E-06
**24h**	2.08E-05	1.14E-05	3.87E-05	2.87E-05
**36h**	3.27E-05	2.59E-05	5.32E-05	2.63E-05
**48h**	6.48E-05	4.59E-05	9.81E-05	4.28E-05
**72h**	1.05E-05	2.46E-06	4.74E-05	2.41E-05
	cgd1_1950			
**2h**	2.69E-06	3.17E-06	3.09E-06	5.35E-07
**6h**	3.82E-06	2.69E-06	6.11E-06	9.67E-07
**12h**	1.53E-06	1.65E-06	5.34E-07	9.29E-07
**24h**	1.36E-06	8.74E-07	1.36E-06	1.03E-06
**36h**	3.05E-06	1.69E-06	2.09E-06	9.08E-07
**48h**	2.07E-06	3.94E-06	4.03E-06	6.89E-07
**72h**	7.14E-07	9.94E-07	1.63E-06	4.52E-07
	cgd1_1960			
**2h**	7.54E-08	7.90E-08	2.26E-07	9.23E-08
**6h**	1.96E-07	9.30E-08	2.81E-07	3.39E-07
**12h**	3.35E-08	8.54E-09	4.41E-08	3.18E-08
**24h**	3.94E-08	1.45E-08	7.64E-08	1.04E-07
**36h**	6.71E-08	1.79E-08	8.38E-08	5.25E-08
**48h**	1.44E-07	2.69E-07	2.68E-07	1.08E-07
**72h**	2.36E-08	1.37E-08	1.06E-07	2.99E-08
	cgd1_1970			
**2h**	1.10E-08	5.70E-08	1.35E-08	9.15E-10
**6h**	4.32E-08	7.25E-09	2.23E-07	8.86E-09
**12h**	1.26E-06	6.17E-07	1.26E-06	1.47E-07
**24h**	1.12E-06	2.02E-07	2.81E-07	2.94E-07
**36h**	2.16E-06	9.84E-07	2.87E-06	9.47E-07
**48h**	1.50E-06	2.41E-06	3.47E-06	4.89E-07
**72h**	3.97E-07	1.18E-07	7.81E-07	1.90E-07
	cgd1_1990			
**2h**	4.47E-04	2.09E-04	5.79E-04	2.31E-04
**6h**	2.94E-04	2.85E-04	4.16E-04	3.85E-04
**12h**	6.82E-05	3.21E-05	2.83E-04	1.18E-04
**24h**	1.21E-03	3.18E-05	2.48E-04	1.64E-04
**36h**	6.01E-05	5.34E-05	1.59E-04	8.27E-05
**48h**	9.60E-05	7.55E-05	3.20E-04	1.48E-04
**72h**	1.60E-05	4.55E-06	5.50E-05	2.26E-05
	cgd1_2000			
**2h**	1.40E-03	1.33E-03	1.54E-03	2.02E-03
**6h**	1.27E-04	2.15E-04	5.36E-04	6.19E-04
**12h**	1.40E-03	9.41E-04	3.98E-03	1.25E-03
**24h**	2.82E-04	4.57E-05	6.82E-04	3.01E-04
**36h**	2.06E-04	1.54E-04	4.07E-04	3.17E-04

48h	4.35E-04	2.60E-04	5.97E-04	1.51E-04
72h	1.16E-04	6.09E-05	4.10E-04	3.81E-04
	cgd1_2020			
2h	0.00E+00	0.00E+00	0.00E+00	6.75E-10
6h	1.39E-09	4.66E-10	4.74E-09	7.48E-10
12h	7.01E-11	5.45E-11	1.30E-10	1.92E-10
24h	8.91E-10	3.49E-10	1.57E-09	1.83E-09
36h	2.99E-09	3.35E-09	4.03E-09	4.51E-09
48h	4.68E-08	4.90E-08	1.35E-07	4.98E-08
72h	5.59E-08	1.78E-08	1.36E-07	6.10E-08
	cgd1_2030			
2h	1.85E-05	5.23E-05	3.13E-06	3.28E-06
6h	9.72E-06	6.09E-06	2.83E-06	2.69E-07
12h	9.55E-06	1.49E-05	4.66E-06	8.57E-06
24h	1.50E-05	3.39E-06	3.29E-06	1.92E-06
36h	1.62E-05	3.46E-05	1.39E-05	1.20E-05
48h	7.47E-05	2.75E-05	1.99E-05	1.01E-05
72h	1.54E-05	6.18E-06	1.53E-05	5.97E-06
	cgd1_2060			
2h	2.14E-06	0.00E+00	0.00E+00	0.00E+00
6h	8.58E-08	7.09E-08	1.35E-06	2.21E-07
12h	4.92E-08	0.00E+00	0.00E+00	1.31E-08
24h	1.28E-06	2.81E-07	7.64E-07	1.02E-06
36h	1.28E-06	9.50E-07	9.38E-07	5.84E-07
48h	1.30E-05	6.21E-06	1.20E-05	3.17E-06
72h	1.20E-05	1.26E-06	1.04E-05	1.28E-05
	cgd1_2070			
2h	3.74E-07	3.20E-07	4.68E-07	8.91E-08
6h	3.47E-07	9.44E-08	4.88E-08	3.35E-08
12h	3.93E-08	1.44E-08	2.40E-08	1.10E-08
24h	5.44E-08	9.58E-09	1.23E-07	5.00E-08
36h	3.93E-07	1.77E-07	4.51E-07	1.17E-07
48h	5.66E-07	2.47E-07	3.32E-07	1.61E-07
72h	1.41E-07	1.88E-08	3.75E-07	1.55E-07
	cgd1_2080			
2h	1.38E-03	2.36E-05	0.00E+00	5.10E-03
6h	3.41E-03	1.89E-04	5.08E-05	4.18E-03
12h	7.98E-04	1.75E-04	4.15E-05	3.35E-03
24h	1.24E-03	4.15E-04	8.90E-05	2.62E-03
36h	5.06E-04	4.24E-04	1.37E-04	1.46E-03
48h	4.25E-04	1.18E-04	3.57E-05	9.84E-04
72h	8.84E-05	3.07E-05	1.84E-05	2.80E-04
	cgd1_2090			
2h	1.22E-02	5.31E-05	0.00E+00	6.91E-03
6h	6.26E-03	2.88E-04	1.23E-04	2.34E-02
12h	5.21E-04	2.99E-04	2.98E-05	8.48E-03
24h	3.76E-03	1.57E-03	2.44E-04	1.01E-02
36h	7.62E-04	7.04E-04	1.33E-04	2.92E-03
48h	1.04E-03	2.04E-04	1.72E-05	2.93E-03
72h	2.39E-04	1.15E-05	3.22E-05	1.19E-03
	cgd1_2100			
2h	1.01E-04	9.14E-05	3.01E-05	3.73E-04
6h	1.27E-04	1.80E-04	4.75E-05	3.75E-04

**12h**	9.36E-04	9.00E-05	3.45E-05	1.49E-04
**24h**	3.75E-05	8.94E-04	5.40E-05	6.73E-04
**36h**	1.07E-04	3.16E-04	2.81E-04	2.60E-04
**48h**	5.84E-05	3.25E-04	8.43E-05	2.52E-04
**72h**	2.39E-06	5.24E-05	4.78E-05	4.00E-05
	cgd1_2110			
**2h**	3.35E-04	3.16E-05	3.70E-05	9.21E-05
**6h**	7.01E-03	1.94E-04	7.87E-05	2.91E-03
**12h**	2.12E-05	7.54E-05	1.62E-04	5.96E-04
**24h**	9.06E-05	1.83E-03	2.99E-04	1.94E-03
**36h**	5.01E-04	4.61E-04	2.74E-04	9.96E-04
**48h**	1.30E-04	7.87E-04	4.08E-05	4.43E-04
**72h**	6.65E-05	5.60E-05	3.27E-05	8.96E-05
	cgd1_2120			
**2h**	5.52E-06	3.67E-05	5.44E-06	5.94E-07
**6h**	2.99E-05	2.92E-04	1.65E-04	8.67E-05
**12h**	3.23E-06	1.36E-04	7.42E-06	6.37E-06
**24h**	5.93E-06	8.54E-04	3.67E-05	9.91E-05
**36h**	3.31E-05	6.23E-04	3.98E-04	4.90E-05
**48h**	1.35E-05	8.43E-04	1.74E-04	2.82E-05
**72h**	3.49E-06	1.72E-05	1.99E-05	2.48E-06
	cgd1_2130			
**2h**	2.11E-06	3.46E-07	7.20E-07	1.22E-07
**6h**	1.60E-05	6.18E-05	1.65E-05	1.47E-05
**12h**	4.84E-07	1.03E-06	4.27E-06	2.58E-06
**24h**	1.87E-06	4.41E-05	7.13E-06	1.37E-05
**36h**	3.38E-06	5.11E-05	5.94E-06	3.47E-06
**48h**	1.81E-06	7.86E-06	2.38E-06	2.79E-06
**72h**	4.31E-07	1.62E-06	1.28E-06	1.96E-07
	cgd1_2140			
**2h**	1.21E-05	2.33E-05	4.78E-05	1.36E-06
**6h**	7.87E-07	2.71E-04	2.99E-05	1.47E-05
**12h**	2.52E-06	5.27E-05	5.79E-05	6.03E-06
**24h**	4.07E-06	2.54E-04	5.69E-05	8.80E-06
**36h**	7.40E-06	3.89E-04	2.16E-04	1.21E-05
**48h**	1.44E-05	8.41E-04	1.20E-04	4.15E-05
**72h**	4.58E-06	2.80E-05	2.01E-05	2.43E-06
	cgd1_2150			
**2h**	1.54E-05	4.15E-05	5.36E-05	3.66E-06
**6h**	1.56E-05	3.40E-05	6.63E-06	3.05E-05
**12h**	4.30E-06	3.61E-05	8.85E-05	1.39E-05
**24h**	6.60E-06	1.02E-04	6.15E-05	1.98E-05
**36h**	3.83E-06	1.69E-04	6.73E-05	8.69E-06
**48h**	1.05E-05	1.46E-04	1.06E-04	2.47E-05
**72h**	4.52E-06	3.53E-05	1.98E-05	8.71E-06
	cgd1_2160			
**2h**	3.03E-06	6.62E-06	0.00E+00	4.50E-07
**6h**	5.50E-07	2.07E-06	1.89E-07	2.16E-06
**12h**	2.64E-07	9.31E-06	0.00E+00	3.37E-06
**24h**	1.41E-06	3.49E-06	3.94E-06	2.53E-06
**36h**	1.78E-07	2.44E-06	2.26E-06	2.88E-07
**48h**	1.33E-06	1.04E-05	8.11E-07	2.78E-06
**72h**	6.10E-07	9.45E-07	1.07E-05	1.18E-06

	cgd1_2180			
**2h**	1.75E-03	1.67E-04	1.01E-04	3.26E-03
**6h**	1.79E-04	3.25E-04	4.24E-05	8.83E-04
**12h**	1.85E-05	1.19E-04	2.59E-04	6.88E-04
**24h**	8.85E-05	1.40E-03	8.27E-05	1.55E-03
**36h**	1.30E-04	1.48E-04	9.93E-05	8.77E-04
**48h**	8.43E-05	3.16E-04	6.56E-05	2.67E-03
**72h**	1.86E-04	2.69E-05	0.00E+00	1.24E-03
	cgd1_2190			
**2h**	5.39E-06	4.56E-07	2.36E-05	7.06E-05
**6h**	5.67E-07	2.82E-06	2.58E-05	7.33E-05
**12h**	3.59E-08	0.00E+00	3.02E-05	3.52E-05
**24h**	9.48E-07	4.16E-07	2.18E-05	2.47E-05
**36h**	8.69E-07	6.89E-07	6.17E-05	6.03E-05
**48h**	1.78E-06	4.31E-07	2.97E-05	2.23E-05
**72h**	2.03E-07	5.65E-07	6.82E-06	5.44E-06
	cgd1_2200			
**2h**	2.40E-08	7.53E-09	1.24E-06	1.61E-07
**6h**	3.52E-09	7.31E-08	1.16E-07	0.00E+00
**12h**	1.67E-09	5.26E-09	1.51E-07	7.99E-08
**24h**	7.75E-07	3.01E-07	8.50E-08	2.90E-06
**36h**	5.33E-07	2.20E-07	1.48E-07	5.95E-06
**48h**	3.49E-06	1.12E-06	6.81E-07	1.30E-05
**72h**	3.99E-07	1.54E-07	1.50E-07	1.59E-06
	cgd1_2210			
**2h**	2.30E-07	7.33E-08	4.46E-07	2.68E-07
**6h**	2.88E-06	7.49E-07	5.49E-07	7.59E-07
**12h**	6.92E-08	4.49E-08	4.92E-08	4.39E-08
**24h**	3.59E-07	2.41E-07	3.79E-07	2.04E-07
**36h**	2.26E-07	3.65E-07	1.21E-07	1.98E-07
**48h**	6.64E-07	9.20E-07	1.01E-06	7.57E-07
**72h**	5.09E-07	1.96E-07	2.95E-07	2.01E-07
	cgd1_2230			
**2h**	1.31E-05	1.81E-06	1.93E-06	1.46E-05
**6h**	1.88E-05	3.13E-05	5.53E-05	3.77E-05
**12h**	7.29E-06	3.60E-06	6.54E-06	3.96E-06
**24h**	3.78E-05	2.42E-05	5.58E-05	2.27E-05
**36h**	2.55E-05	1.47E-05	2.65E-05	1.21E-05
**48h**	1.82E-05	2.14E-05	5.34E-05	6.58E-06
**72h**	2.46E-05	1.71E-05	2.80E-05	1.10E-05
	cgd1_2240			
**2h**	2.32E-09	1.39E-09	0.00E+00	4.17E-09
**6h**	3.05E-08	6.78E-08	4.84E-08	5.22E-08
**12h**	1.60E-09	3.57E-09	4.05E-10	3.66E-10
**24h**	4.80E-08	1.91E-08	6.00E-08	6.15E-08
**36h**	6.62E-08	4.52E-08	5.13E-08	3.63E-08
**48h**	1.07E-07	9.99E-08	2.60E-07	4.37E-08
**72h**	7.49E-08	2.67E-08	1.30E-07	4.63E-08
	cgd1_2270			
**2h**	7.19E-06	2.74E-06	1.51E-06	2.88E-07
**6h**	4.99E-05	6.11E-05	2.28E-05	1.09E-06
**12h**	1.96E-05	1.23E-05	5.56E-06	1.68E-06
**24h**	1.62E-05	8.69E-06	5.55E-06	9.68E-07

**36h**	1.12E-05	1.53E-05	3.99E-06	5.44E-07
**48h**	2.96E-06	5.85E-06	4.01E-06	2.99E-07
**72h**	7.11E-07	6.86E-07	2.64E-07	2.30E-07
	cgd1_2310			
**2h**	2.87E-07	5.86E-07	3.08E-07	5.25E-07
**6h**	2.15E-07	2.24E-07	2.31E-07	4.67E-07
**12h**	7.21E-07	2.27E-06	2.44E-06	7.02E-07
**24h**	4.61E-07	2.53E-07	5.48E-07	5.65E-07
**36h**	5.54E-07	3.45E-07	3.42E-07	2.76E-07
**48h**	4.97E-07	2.19E-07	6.55E-07	1.66E-07
**72h**	2.69E-07	9.77E-08	2.24E-07	8.44E-08
	cgd1_2380			
**2h**	5.82E-07	8.66E-08	1.47E-07	1.36E-07
**6h**	2.68E-07	1.15E-07	2.56E-07	3.56E-07
**12h**	4.20E-08	8.03E-08	2.02E-07	4.10E-08
**24h**	2.10E-07	5.37E-08	1.83E-07	2.48E-07
**36h**	3.35E-07	8.04E-08	2.53E-07	1.26E-07
**48h**	2.04E-07	1.81E-07	1.04E-06	2.42E-07
**72h**	2.80E-07	7.78E-08	2.05E-07	1.74E-07
	cgd1_2390			
**2h**	4.84E-07	8.19E-04	2.87E-07	2.73E-07
**6h**	1.79E-07	1.67E-07	1.82E-07	2.24E-07
**12h**	1.74E-07	4.02E-07	2.68E-07	1.05E-07
**24h**	2.72E-07	1.49E-07	1.18E-07	1.11E-07
**36h**	3.66E-07	1.68E-07	2.26E-07	7.56E-08
**48h**	2.48E-07	1.73E-07	3.29E-07	8.25E-08
**72h**	1.23E-07	8.15E-08	1.08E-07	9.95E-08
	cgd1_2410			
**2h**	4.51E-07	2.25E-07	1.18E-07	5.03E-08
**6h**	2.56E-07	1.18E-07	2.79E-07	4.12E-07
**12h**	3.57E-08	3.31E-08	3.48E-08	3.73E-08
**24h**	1.85E-07	1.75E-08	7.70E-08	1.93E-07
**36h**	2.02E-07	1.12E-07	2.41E-07	9.89E-08
**48h**	1.81E-07	1.24E-07	2.73E-07	9.27E-08
**72h**	1.26E-07	7.55E-08	7.86E-08	8.15E-08
	cgd1_2440			
**2h**	1.24E-06	1.03E-06	1.90E-06	4.16E-06
**6h**	1.79E-07	1.33E-07	4.08E-07	4.92E-07
**12h**	3.65E-07	1.18E-07	1.22E-06	5.25E-07
**24h**	1.05E-07	6.36E-08	2.03E-07	1.26E-07
**36h**	1.88E-07	1.46E-07	2.04E-07	1.05E-07
**48h**	9.48E-08	1.18E-07	3.38E-07	1.19E-07
**72h**	1.21E-07	4.34E-08	1.98E-07	9.81E-08
	cgd1_2450			
**2h**	6.53E-06	3.59E-06	3.29E-06	3.28E-06
**6h**	2.23E-06	9.61E-07	1.96E-07	8.60E-07
**12h**	2.35E-06	3.55E-06	2.20E-06	7.33E-07
**24h**	1.51E-06	3.08E-07	1.57E-06	3.63E-07
**36h**	1.97E-06	1.14E-06	1.01E-06	1.23E-07
**48h**	1.50E-06	1.13E-06	9.87E-07	2.97E-07
**72h**	9.23E-07	3.08E-07	5.46E-07	2.47E-07
	cgd1_2480			
**2h**	1.44E-03	4.96E-04	1.80E-04	5.06E-04

**6h**	6.57E-04	4.35E-04	7.90E-04	7.15E-04
**12h**	1.01E-04	7.15E-05	1.63E-04	1.29E-04
**24h**	4.39E-04	9.58E-05	3.55E-04	2.98E-04
**36h**	3.79E-04	1.04E-04	5.22E-04	1.29E-04
**48h**	1.25E-04	1.61E-04	4.93E-04	2.96E-04
**72h**	1.37E-04	9.69E-05	1.45E-04	3.39E-04
	cgd1_2500			
**2h**	4.56E-08	3.51E-08	1.46E-07	3.84E-09
**6h**	8.68E-06	2.21E-06	2.12E-06	2.86E-06
**12h**	1.28E-07	2.84E-07	5.86E-07	2.70E-07
**24h**	5.31E-07	1.52E-07	1.48E-06	9.71E-07
**36h**	1.55E-06	6.62E-07	1.70E-06	6.96E-07
**48h**	2.09E-07	1.75E-06	6.64E-07	2.42E-07
**72h**	1.96E-07	1.27E-07	1.82E-07	8.66E-08
	cgd1_2520			
**2h**	0.00E+00	0.00E+00	0.00E+00	0.00E+00
**6h**	0.00E+00	0.00E+00	0.00E+00	0.00E+00
**12h**	1.89E-05	1.28E-05	5.96E-06	3.90E-06
**24h**	8.06E-06	1.54E-06	1.26E-05	3.27E-06
**36h**	2.10E-05	1.46E-05	4.93E-05	1.16E-05
**48h**	6.05E-05	3.45E-05	1.29E-04	4.28E-05
**72h**	6.49E-05	9.25E-05	2.05E-04	3.57E-05
	cgd1_2550			
**2h**	0.00E+00	2.79E-09	0.00E+00	0.00E+00
**6h**	2.76E-07	4.33E-08	4.36E-08	2.88E-08
**12h**	4.19E-09	3.84E-08	0.00E+00	1.38E-08
**24h**	3.77E-07	2.14E-07	1.18E-07	1.61E-07
**36h**	2.35E-07	1.56E-07	1.26E-07	1.00E-07
**48h**	1.54E-06	9.51E-07	9.81E-07	3.54E-07
**72h**	3.74E-07	1.51E-07	1.95E-07	1.61E-07
	cgd1_2560			
**2h**	1.69E-05	6.50E-06	2.09E-04	1.96E-05
**6h**	7.01E-04	5.94E-05	2.88E-04	5.08E-04
**12h**	1.80E-05	7.21E-05	7.51E-05	7.16E-05
**24h**	1.04E-04	7.42E-06	6.97E-05	1.53E-04
**36h**	1.18E-04	9.20E-05	6.16E-05	6.16E-05
**48h**	3.22E-05	5.01E-05	1.02E-04	5.44E-05
**72h**	6.34E-05	5.24E-05	5.91E-05	6.68E-05
	cgd1_2570			
**2h**	5.22E-08	3.39E-08	5.24E-09	0.00E+00
**6h**	5.11E-07	6.44E-08	6.96E-07	5.37E-07
**12h**	8.24E-07	4.77E-07	1.90E-07	1.78E-07
**24h**	1.02E-06	6.77E-08	3.93E-07	2.87E-07
**36h**	2.06E-06	1.02E-06	1.71E-06	6.80E-07
**48h**	7.56E-07	4.04E-07	1.05E-06	2.57E-07
**72h**	3.57E-07	2.20E-07	2.60E-07	1.22E-07
	cgd1_2610			
**2h**	2.41E-08	3.03E-09	3.76E-08	2.62E-08
**6h**	6.23E-08	1.19E-08	1.61E-08	1.22E-08
**12h**	5.34E-09	4.95E-09	1.73E-08	3.50E-09
**24h**	2.04E-08	5.96E-09	1.58E-08	3.25E-08
**36h**	1.55E-08	9.97E-09	2.30E-08	9.06E-09
**48h**	1.50E-08	6.36E-09	6.56E-08	7.89E-09

**72h**	1.22E-08	6.75E-09	1.91E-08	8.42E-09
	cgd1_2620			
**2h**	5.38E-05	7.33E-05	3.39E-07	1.57E-06
**6h**	1.06E-06	7.88E-07	9.05E-08	7.96E-08
**12h**	1.73E-05	6.27E-06	1.84E-06	9.85E-07
**24h**	2.76E-06	1.30E-06	4.62E-07	6.16E-07
**36h**	3.03E-06	1.23E-06	4.25E-07	1.44E-07
**48h**	4.63E-06	3.60E-06	2.11E-06	4.63E-07
**72h**	1.65E-06	1.28E-06	1.13E-06	9.79E-07
	cgd1_2650			
**2h**	6.89E-04	1.73E-03	1.26E-03	2.92E-03
**6h**	3.90E-04	1.31E-04	1.03E-03	3.58E-04
**12h**	6.23E-04	4.72E-04	1.19E-04	4.22E-04
**24h**	7.88E-05	3.26E-04	1.21E-04	3.40E-04
**36h**	1.77E-04	2.25E-04	2.32E-04	1.18E-04
**48h**	1.25E-04	5.98E-05	1.83E-05	1.31E-05
**72h**	2.00E-04	1.91E-04	3.62E-04	6.99E-05
	cgd1_2670			
**2h**	1.18E-03	2.59E-04	5.82E-04	4.26E-04
**6h**	4.48E-04	6.27E-05	9.47E-04	5.13E-04
**12h**	7.42E-04	6.61E-04	1.08E-03	1.45E-03
**24h**	2.72E-04	8.58E-05	2.57E-04	6.29E-04
**36h**	2.43E-04	6.59E-05	2.41E-04	4.33E-05
**48h**	8.01E-05	2.18E-04	3.40E-04	9.15E-05
**72h**	1.10E-04	6.32E-05	1.61E-04	4.82E-05
	cgd1_2680			
**2h**	2.94E-08	1.75E-08	1.27E-08	5.34E-09
**6h**	4.52E-09	2.12E-09	2.81E-09	0.00E+00
**12h**	2.92E-09	5.04E-09	5.03E-08	2.48E-08
**24h**	5.60E-10	1.95E-09	8.01E-09	1.06E-08
**36h**	4.20E-09	5.10E-09	7.67E-09	1.45E-09
**48h**	1.18E-08	2.26E-08	4.29E-08	6.10E-09
**72h**	2.66E-08	7.38E-09	3.35E-08	2.05E-08
	cgd1_2710			
**2h**	2.71E-07	2.96E-07	3.47E-07	4.42E-07
**6h**	1.13E-06	2.96E-07	1.01E-06	2.60E-07
**12h**	5.15E-07	2.89E-07	1.86E-08	2.14E-07
**24h**	4.58E-07	3.23E-07	4.60E-07	4.32E-07
**36h**	4.73E-07	3.68E-07	5.14E-07	1.28E-07
**48h**	4.28E-07	3.50E-07	7.09E-07	1.36E-07
**72h**	2.28E-07	8.69E-08	1.88E-07	1.26E-07
	cgd1_2720			
**2h**	0.00E+00	1.55E-07	4.87E-08	3.18E-08
**6h**	2.13E-07	6.86E-08	1.27E-06	1.41E-07
**12h**	1.56E-07	1.89E-07	8.47E-08	2.86E-08
**24h**	2.81E-07	1.42E-07	7.18E-07	5.27E-07
**36h**	9.68E-07	4.61E-07	2.96E-06	1.16E-06
**48h**	1.33E-06	1.74E-06	6.74E-06	1.25E-06
**72h**	7.62E-07	9.62E-07	2.45E-06	1.42E-06
	cgd1_2730			
**2h**	3.20E-04	2.40E-04	7.44E-04	4.39E-04
**6h**	1.41E-04	6.20E-05	5.63E-04	2.94E-04
**12h**	2.55E-04	1.16E-04	1.88E-04	2.28E-04

**24h**	6.08E-05	6.92E-05	2.24E-04	1.97E-04
**36h**	2.18E-04	1.10E-04	3.80E-04	1.47E-04
**48h**	6.43E-05	7.41E-05	2.24E-04	8.06E-05
**72h**	1.80E-05	1.97E-05	1.16E-04	4.02E-05
	cgd1_2740			
**2h**	1.33E-03	5.83E-04	8.27E-04	7.82E-04
**6h**	8.00E-04	1.25E-03	6.72E-04	6.51E-03
**12h**	4.54E-05	1.66E-04	6.00E-05	8.35E-04
**24h**	5.57E-05	1.31E-04	4.42E-04	1.27E-03
**36h**	3.00E-04	2.54E-04	2.58E-04	8.25E-04
**48h**	1.82E-04	8.73E-05	1.71E-04	5.89E-04
**72h**	5.69E-05	3.88E-05	3.66E-05	1.50E-04
	cgd1_2770			
**2h**	6.72E-04	3.67E-04	2.95E-04	2.50E-04
**6h**	7.44E-05	2.08E-04	1.15E-04	2.48E-04
**12h**	1.49E-04	4.90E-05	4.47E-05	5.31E-04
**24h**	1.63E-04	1.56E-04	1.46E-04	3.10E-04
**36h**	8.09E-05	1.87E-05	1.86E-05	8.10E-05
**48h**	3.64E-05	1.04E-05	2.17E-05	3.34E-05
**72h**	6.43E-06	3.11E-06	4.71E-06	8.62E-06
	cgd1_2780			
**2h**	7.69E-06	1.69E-06	2.93E-06	1.36E-06
**6h**	1.50E-07	5.93E-07	7.04E-07	6.62E-07
**12h**	7.98E-07	3.02E-07	9.95E-08	2.76E-06
**24h**	1.52E-06	1.46E-06	1.26E-06	4.02E-06
**36h**	2.06E-07	2.43E-07	1.76E-07	8.63E-07
**48h**	4.28E-07	1.86E-07	2.20E-07	9.73E-07
**72h**	1.72E-08	3.10E-08	6.65E-08	5.04E-08
	cgd1_2790			
**2h**	0.00E+00	2.31E-08	0.00E+00	0.00E+00
**6h**	1.67E-07	1.59E-07	2.36E-07	1.09E-06
**12h**	2.11E-08	2.98E-09	7.44E-10	3.39E-08
**24h**	1.53E-08	9.22E-09	1.07E-07	2.47E-07
**36h**	4.98E-08	4.13E-08	1.91E-08	3.90E-08
**48h**	1.22E-07	2.75E-08	1.82E-08	1.44E-07
**72h**	1.64E-08	1.46E-08	1.04E-08	8.74E-09
	cgd1_2800			
**2h**	4.54E-05	1.17E-04	6.56E-06	0.00E+00
**6h**	5.61E-05	7.51E-05	6.04E-05	1.33E-04
**12h**	1.87E-06	1.77E-06	1.78E-06	5.09E-05
**24h**	4.85E-06	7.62E-06	1.41E-05	2.05E-04
**36h**	2.84E-05	1.50E-05	4.57E-06	3.88E-05
**48h**	2.90E-05	4.70E-06	2.38E-06	1.53E-05
**72h**	5.46E-06	6.98E-06	2.39E-06	9.48E-05
	cgd1_2810			
**2h**	2.41E-07	9.68E-08	5.54E-06	0.00E+00
**6h**	7.72E-06	1.47E-06	2.06E-06	5.13E-06
**12h**	1.82E-08	0.00E+00	2.04E-06	8.18E-07
**24h**	1.72E-07	1.84E-07	6.08E-07	4.25E-06
**36h**	2.72E-07	3.46E-07	1.56E-06	2.34E-06
**48h**	2.77E-07	8.28E-08	9.45E-07	5.57E-07
**72h**	1.02E-07	8.97E-08	1.87E-07	6.85E-08
	cgd1_2830			

**2h**	0.00E+00	0.00E+00	1.39E-07	0.00E+00
**6h**	2.69E-06	2.64E-06	1.16E-06	1.77E-06
**12h**	2.46E-07	4.03E-07	2.52E-07	3.10E-06
**24h**	5.29E-07	1.10E-06	2.46E-06	1.19E-05
**36h**	1.42E-06	2.75E-06	3.76E-06	4.22E-06
**48h**	1.80E-06	1.62E-06	4.10E-06	5.02E-06
**72h**	1.43E-06	7.16E-07	5.44E-07	1.17E-06
	cgd1_2840			
**2h**	6.98E-05	2.36E-05	3.15E-04	0.00E+00
**6h**	7.47E-05	4.61E-05	1.37E-03	4.41E-04
**12h**	1.24E-07	0.00E+00	1.62E-04	1.80E-04
**24h**	1.74E-05	1.33E-05	1.42E-04	1.66E-04
**36h**	2.53E-05	1.42E-05	2.26E-04	6.46E-05
**48h**	2.72E-05	2.16E-05	3.71E-04	1.39E-04
**72h**	4.62E-06	4.97E-06	9.23E-05	1.51E-05
	cgd1_2850			
**2h**	0.00E+00	0.00E+00	0.00E+00	0.00E+00
**6h**	0.00E+00	2.71E-07	1.97E-05	0.00E+00
**12h**	3.20E-04	3.94E-04	5.15E-04	3.77E-04
**24h**	6.11E-05	1.49E-04	1.08E-04	1.41E-04
**36h**	3.69E-04	4.32E-04	3.41E-04	2.63E-04
**48h**	1.38E-04	6.47E-05	1.24E-03	5.45E-05
**72h**	8.30E-05	6.55E-05	4.68E-04	3.96E-05
	cgd1_2860			
**2h**	4.54E-08	3.00E-08	1.88E-08	0.00E+00
**6h**	0.00E+00	3.82E-09	2.51E-09	1.05E-06
**12h**	0.00E+00	1.30E-08	1.26E-07	9.84E-08
**24h**	6.81E-09	1.51E-08	4.31E-07	1.80E-06
**36h**	2.13E-07	2.33E-07	3.87E-07	5.29E-06
**48h**	6.96E-08	8.91E-08	2.01E-06	2.29E-06
**72h**	5.01E-07	1.79E-07	1.21E-06	1.56E-06
	cgd1_2880			
**2h**	2.18E-04	3.02E-04	8.63E-04	1.82E-03
**6h**	1.90E-04	1.74E-04	2.52E-04	1.52E-03
**12h**	6.64E-04	5.62E-04	5.79E-04	2.43E-03
**24h**	1.38E-04	1.59E-04	2.15E-04	2.33E-03
**36h**	2.01E-04	1.91E-04	4.81E-04	1.23E-03
**48h**	1.06E-04	7.07E-05	3.26E-04	6.59E-04
**72h**	4.27E-05	3.01E-05	1.25E-04	1.83E-04
	cgd2_2940			
**2h**	1.48E-04	1.53E-04	7.44E-05	5.19E-06
**6h**	8.64E-05	1.04E-04	6.36E-05	8.81E-07
**12h**	8.40E-05	1.81E-05	2.83E-05	8.58E-07
**24h**	3.40E-05	9.35E-05	1.15E-05	1.42E-06
**36h**	2.76E-05	4.61E-05	3.33E-06	1.53E-06
**48h**	2.67E-05	5.25E-05	1.49E-05	1.00E-04
**72h**	5.55E-06	2.65E-05	4.08E-06	2.47E-05
	cgd2_2960			
**2h**	1.37E-06	1.03E-06	3.30E-07	2.25E-09
**6h**	9.99E-07	8.91E-07	4.35E-07	3.74E-08
**12h**	4.30E-07	3.25E-08	3.08E-07	7.30E-09
**24h**	4.52E-07	1.66E-06	1.83E-07	1.61E-08
**36h**	6.75E-07	1.22E-06	6.19E-08	5.97E-08

**48h**	7.51E-08	4.13E-07	1.08E-07	5.68E-07
**72h**	1.08E-07	4.50E-07	1.09E-07	1.09E-06
	cgd2_2970			
**2h**	2.26E-03	4.66E-05	3.91E-06	1.89E-04
**6h**	2.00E-05	1.59E-04	1.16E-06	1.51E-04
**12h**	2.75E-04	2.28E-04	1.53E-07	3.61E-05
**24h**	1.10E-04	4.90E-05	2.26E-06	2.57E-05
**36h**	2.10E-04	2.43E-04	1.19E-07	6.60E-05
**48h**	1.33E-05	7.19E-05	1.37E-05	1.33E-04
**72h**	3.51E-05	2.86E-04	2.91E-06	1.75E-04
	cgd2_2980			
**2h**	1.56E-05	4.26E-06	2.47E-07	2.78E-06
**6h**	0.00E+00	0.00E+00	3.93E-08	0.00E+00
**12h**	0.00E+00	1.13E-07	0.00E+00	0.00E+00
**24h**	1.44E-05	1.77E-06	5.73E-08	1.92E-06
**36h**	7.56E-06	4.80E-07	1.85E-06	3.78E-05
**48h**	1.82E-05	2.91E-06	1.03E-05	3.03E-05
**72h**	5.58E-05	4.19E-06	6.27E-05	3.80E-05
	cgd2_2990			
**2h**	1.00E-06	1.36E-06	1.95E-06	3.65E-07
**6h**	4.14E-06	9.66E-06	1.14E-05	1.78E-06
**12h**	1.26E-06	3.05E-06	5.30E-06	1.18E-06
**24h**	2.60E-06	1.02E-05	6.63E-06	2.31E-06
**36h**	4.89E-07	2.16E-06	1.49E-06	1.13E-06
**48h**	4.97E-07	1.79E-06	2.31E-06	3.43E-06
**72h**	1.09E-07	6.75E-07	1.89E-07	2.32E-06
	cgd2_3000			
**2h**	2.73E-06	2.70E-06	3.57E-06	2.53E-06
**6h**	2.95E-05	1.62E-05	8.93E-06	1.12E-05
**12h**	7.89E-06	4.89E-06	6.14E-06	8.70E-06
**24h**	1.20E-05	2.27E-05	1.06E-05	8.99E-06
**36h**	3.43E-06	8.59E-06	7.34E-06	2.07E-06
**48h**	4.33E-06	4.03E-06	3.01E-06	6.98E-06
**72h**	5.04E-07	2.53E-06	3.66E-07	2.10E-06
	cgd2_3010			
**2h**	1.41E-07	1.99E-07	3.06E-08	1.14E-08
**6h**	3.47E-07	3.48E-07	1.99E-07	6.17E-08
**12h**	1.31E-07	9.87E-08	5.27E-08	2.51E-09
**24h**	1.93E-07	9.83E-07	1.38E-07	1.02E-08
**36h**	2.91E-07	8.54E-07	4.74E-08	7.81E-08
**48h**	2.21E-07	5.00E-07	2.12E-07	6.55E-07
**72h**	2.04E-04	1.30E-06	1.25E-07	7.62E-07
	cgd2_2930			
**2h**	1.50E-08	7.21E-08	2.17E-08	0.00E+00
**6h**	0.00E+00	0.00E+00	1.56E-10	0.00E+00
**12h**	2.96E-07	5.45E-08	2.76E-08	0.00E+00
**24h**	1.62E-08	4.87E-08	1.83E-08	8.37E-10
**36h**	1.48E-07	9.24E-08	7.17E-09	8.61E-09
**48h**	3.89E-08	8.53E-08	1.16E-08	1.15E-07
**72h**	5.46E-08	3.98E-07	4.61E-08	6.68E-07
	cgd2_3020			
**2h**	3.47E-05	3.27E-05	0.00E+00	3.28E-05
**6h**	6.94E-05	1.07E-04	5.37E-05	8.72E-05

**12h**	6.92E-05	1.53E-05	1.51E-05	5.13E-05
**24h**	3.32E-05	2.89E-04	7.15E-05	5.07E-05
**36h**	7.67E-05	4.89E-05	1.79E-05	5.98E-05
**48h**	3.15E-05	1.79E-04	4.74E-05	3.89E-05
**72h**	7.33E-05	1.22E-04	2.68E-05	1.84E-04
	cgd2_3030			
**2h**	1.70E-09	0.00E+00	0.00E+00	0.00E+00
**6h**	7.06E-10	8.43E-10	0.00E+00	0.00E+00
**12h**	9.30E-09	7.40E-09	6.53E-09	1.49E-09
**24h**	3.77E-09	1.67E-08	4.28E-09	0.00E+00
**36h**	4.36E-08	2.60E-08	1.21E-08	2.10E-08
**48h**	2.91E-08	4.65E-08	2.00E-08	7.10E-08
**72h**	7.02E-08	5.01E-08	2.65E-08	1.51E-07
	cgd2_3040			
**2h**	2.94E-08	3.13E-08	3.71E-08	0.00E+00
**6h**	0.00E+00	0.00E+00	0.00E+00	0.00E+00
**12h**	9.94E-09	0.00E+00	7.93E-10	0.00E+00
**24h**	5.54E-09	6.79E-09	7.69E-09	1.64E-09
**36h**	1.17E-08	7.53E-09	2.68E-09	7.12E-10
**48h**	5.42E-08	1.52E-07	1.87E-07	2.39E-07
**72h**	1.08E-06	1.85E-06	6.29E-07	0.00E+00
	cgd2_3050			
**2h**	1.00E-05	5.50E-06	2.82E-06	1.95E-07
**6h**	1.76E-08	4.48E-08	8.55E-09	0.00E+00
**12h**	3.21E-06	1.22E-06	6.50E-07	2.52E-07
**24h**	1.35E-07	3.96E-07	1.24E-07	8.43E-09
**36h**	5.03E-07	7.75E-07	6.26E-08	1.51E-07
**48h**	3.13E-07	1.04E-06	9.96E-07	3.56E-06
**72h**	1.86E-06	3.43E-06	9.78E-07	3.64E-06
	cgd2_3060			
**2h**	3.05E-06	4.70E-07	2.56E-08	9.33E-07
**6h**	3.76E-08	1.71E-09	1.15E-08	5.90E-09
**12h**	5.90E-07	1.30E-07	3.21E-08	4.96E-08
**24h**	3.43E-08	3.89E-08	1.42E-08	1.03E-07
**36h**	1.35E-07	1.88E-08	9.35E-09	1.47E-07
**48h**	1.32E-07	9.69E-08	3.20E-07	1.54E-07
**72h**	9.24E-08	1.98E-07	1.12E-06	5.43E-07
	cgd2_3070			
**2h**	4.39E-04	1.02E-04	2.46E-05	2.33E-04
**6h**	8.41E-06	9.74E-07	8.50E-07	5.41E-07
**12h**	1.80E-04	8.55E-05	4.44E-05	7.82E-05
**24h**	2.34E-05	1.81E-05	1.28E-05	4.72E-05
**36h**	2.73E-05	1.71E-05	1.17E-05	2.09E-05
**48h**	1.46E-05	1.14E-05	5.51E-05	1.45E-05
**72h**	4.99E-06	1.63E-05	7.29E-05	2.26E-05
	cgd2_3080			
**2h**	3.85E-08	0.00E+00	0.00E+00	0.00E+00
**6h**	4.50E-08	3.92E-08	3.58E-08	4.57E-08
**12h**	1.96E-07	8.27E-09	0.00E+00	7.58E-09
**24h**	1.05E-07	6.94E-08	3.37E-08	1.45E-07
**36h**	2.84E-07	7.87E-08	6.64E-08	2.38E-07
**48h**	2.38E-07	2.29E-07	1.49E-06	2.19E-07
**72h**	1.07E-07	1.01E-07	1.47E-06	2.49E-07

	cgd2_3090			
**2h**	1.22E-07	2.74E-09	3.18E-09	2.79E-08
**6h**	1.37E-06	7.67E-07	1.21E-07	3.75E-07
**12h**	1.49E-06	2.41E-07	1.04E-07	1.34E-07
**24h**	9.79E-07	7.12E-07	1.88E-07	7.55E-07
**36h**	1.63E-06	1.12E-06	1.46E-06	1.45E-06
**48h**	8.66E-07	8.69E-07	5.40E-06	9.52E-07
**72h**	1.19E-07	1.53E-07	1.36E-06	4.78E-07
	cgd2_3140			
**2h**	0.00E+00	0.00E+00	0.00E+00	0.00E+00
**6h**	8.21E-09	8.54E-10	0.00E+00	7.47E-09
**12h**	7.23E-09	0.00E+00	0.00E+00	0.00E+00
**24h**	1.17E-09	2.42E-09	3.90E-10	4.32E-08
**36h**	3.20E-08	5.63E-10	4.24E-10	3.42E-08
**48h**	6.15E-07	4.36E-07	1.95E-06	1.23E-06
**72h**	1.93E-07	1.44E-06	9.43E-06	4.53E-06
	cgd2_3170			
**2h**	0.00E+00	0.00E+00	0.00E+00	0.00E+00
**6h**	4.61E-06	8.71E-07	5.75E-07	1.21E-05
**12h**	1.51E-05	6.77E-07	0.00E+00	0.00E+00
**24h**	1.72E-05	1.96E-06	8.01E-08	9.83E-05
**36h**	4.25E-05	2.32E-06	2.03E-06	3.57E-04
**48h**	2.58E-05	8.51E-06	1.38E-04	6.63E-05
**72h**	1.16E-05	2.70E-05	3.86E-04	7.66E-05
	cgd2_3180			
**2h**	4.77E-09	0.00E+00	0.00E+00	0.00E+00
**6h**	1.96E-06	9.33E-07	9.51E-08	9.43E-07
**12h**	5.04E-07	2.74E-08	0.00E+00	6.67E-09
**24h**	1.95E-06	3.98E-07	2.38E-07	2.09E-06
**36h**	1.11E-06	1.58E-07	2.63E-07	1.69E-06
**48h**	4.89E-07	4.33E-07	2.51E-06	9.82E-07
**72h**	1.99E-07	4.12E-07	2.10E-06	1.06E-06
	cgd2_3190			
**2h**	1.90E-09	0.00E+00	0.00E+00	0.00E+00
**6h**	0.00E+00	1.17E-08	0.00E+00	0.00E+00
**12h**	1.45E-05	5.04E-07	9.99E-08	2.61E-07
**24h**	3.06E-07	4.47E-08	3.93E-09	6.66E-07
**36h**	1.23E-06	9.72E-08	1.41E-07	9.67E-07
**48h**	2.25E-07	2.09E-07	1.29E-06	5.17E-07
**72h**	3.28E-07	9.42E-07	4.56E-06	1.62E-06
	cgd2_3200			
**2h**	1.36E-08	0.00E+00	0.00E+00	0.00E+00
**6h**	1.54E-06	2.60E-06	2.52E-06	6.36E-08
**12h**	8.17E-07	1.52E-07	1.41E-06	2.80E-08
**24h**	1.63E-06	1.45E-06	4.55E-08	2.72E-06
**36h**	3.07E-06	1.28E-06	2.91E-06	2.35E-06
**48h**	8.64E-07	1.71E-06	4.59E-06	8.95E-07
**72h**	3.31E-06	7.65E-07	1.33E-05	2.46E-06
	cgd2_3210			
**2h**	3.47E-06	4.52E-07	1.93E-06	1.39E-06
**6h**	8.06E-07	5.65E-08	1.38E-08	1.53E-07
**12h**	9.97E-06	1.80E-06	1.96E-06	7.66E-07
**24h**	3.00E-06	1.28E-06	7.46E-06	1.59E-06

**36h**	2.45E-05	1.01E-05	2.07E-05	1.05E-05
**48h**	1.18E-05	5.64E-06	2.20E-05	2.11E-06
**72h**	9.87E-06	1.18E-05	1.52E-05	9.58E-06
	cgd2_3220			
**2h**	7.83E-07	2.38E-07	2.89E-09	8.13E-07
**6h**	3.40E-06	4.29E-07	1.70E-07	1.82E-06
**12h**	1.71E-06	2.73E-07	7.13E-08	4.20E-07
**24h**	8.62E-07	5.62E-07	1.11E-07	2.83E-06
**36h**	7.81E-07	1.09E-07	1.15E-07	8.06E-07
**48h**	2.71E-07	2.17E-07	1.30E-06	5.90E-07
**72h**	2.91E-08	5.05E-08	7.39E-07	2.35E-07
	cgd2_3230			
**2h**	2.40E-06	2.61E-07	2.35E-08	1.01E-06
**6h**	1.37E-06	1.00E-06	1.25E-07	8.14E-07
**12h**	4.42E-07	1.38E-07	2.59E-08	3.12E-08
**24h**	6.73E-07	3.28E-07	7.85E-08	1.64E-06
**36h**	1.68E-06	3.70E-07	9.57E-07	1.75E-06
**48h**	4.49E-07	4.85E-07	2.67E-06	7.50E-07
**72h**	8.21E-07	6.16E-07	9.81E-06	3.55E-06
	cgd2_3130			
**2h**	0.00E+00	0.00E+00	0.00E+00	0.00E+00
**6h**	0.00E+00	2.79E-10	3.24E-10	0.00E+00
**12h**	0.00E+00	2.16E-09	3.05E-10	0.00E+00
**24h**	0.00E+00	3.34E-10	8.28E-11	0.00E+00
**36h**	2.77E-09	4.77E-09	9.71E-10	3.12E-10
**48h**	2.29E-07	1.29E-07	1.54E-07	4.74E-07
**72h**	7.86E-07	1.57E-07	2.00E-07	1.28E-06
	cgd2_3240			
**2h**	1.40E-08	1.01E-08	2.98E-09	0.00E+00
**6h**	8.89E-09	1.09E-08	2.40E-08	4.65E-09
**12h**	4.88E-09	2.71E-08	7.37E-09	2.61E-09
**24h**	5.66E-08	2.04E-08	2.79E-08	8.23E-09
**36h**	2.27E-07	6.37E-08	3.27E-09	3.59E-08
**48h**	2.46E-07	1.18E-07	6.98E-08	2.55E-07
**72h**	8.00E-06	9.58E-07	1.26E-06	3.85E-06
	cgd2_3250			
**2h**	3.25E-08	3.87E-08	4.57E-08	2.25E-09
**6h**	4.01E-08	1.49E-08	1.55E-08	4.37E-09
**12h**	1.29E-08	2.87E-08	1.74E-08	5.05E-09
**24h**	4.20E-08	1.58E-08	3.11E-08	7.81E-09
**36h**	7.35E-08	8.60E-08	1.85E-09	6.88E-09
**48h**	2.05E-07	5.63E-08	7.71E-08	2.01E-07
**72h**	3.88E-06	8.73E-07	8.61E-07	6.46E-06
	cgd2_3260			
**2h**	4.06E-08	2.71E-07	1.03E-07	1.03E-09
**6h**	3.62E-08	4.13E-08	6.64E-08	4.73E-09
**12h**	1.46E-08	1.34E-07	1.93E-08	5.75E-09
**24h**	3.71E-07	7.66E-08	5.68E-08	1.65E-08
**36h**	1.35E-06	1.16E-06	1.15E-07	1.43E-07
**48h**	1.31E-06	1.03E-06	4.09E-07	6.02E-06
**72h**	1.11E-06	2.04E-07	2.11E-07	2.56E-06
	cgd2_3330			
**2h**	1.15E-07	6.94E-07	2.26E-08	0.00E+00

**6h**	2.37E-05	1.67E-05	2.37E-05	9.89E-07
**12h**	3.14E-07	2.95E-06	2.26E-07	0.00E+00
**24h**	5.49E-05	1.59E-05	1.02E-05	1.05E-06
**36h**	2.61E-05	3.98E-05	2.08E-06	2.03E-06
**48h**	8.41E-06	4.34E-06	3.31E-06	2.07E-05
**72h**	4.79E-06	1.13E-06	4.79E-07	2.32E-06
	cgd2_3340			
**2h**	2.12E-08	4.38E-08	1.83E-07	5.54E-08
**6h**	4.13E-07	1.03E-06	1.14E-06	4.56E-08
**12h**	3.26E-09	1.08E-07	6.29E-08	1.13E-08
**24h**	1.24E-06	1.04E-06	5.52E-07	4.52E-08
**36h**	7.24E-07	8.16E-07	9.87E-08	8.59E-08
**48h**	3.99E-07	2.40E-07	1.39E-07	6.63E-07
**72h**	1.36E-07	4.37E-08	1.46E-08	2.54E-07
	cgd2_3360			
**2h**	0.00E+00	0.00E+00	0.00E+00	0.00E+00
**6h**	0.00E+00	0.00E+00	0.00E+00	0.00E+00
**12h**	0.00E+00	8.00E-06	1.68E-05	2.26E-05
**24h**	4.87E-06	2.15E-06	1.52E-06	4.44E-07
**36h**	9.67E-06	9.37E-06	3.25E-05	1.26E-05
**48h**	1.55E-05	1.04E-05	3.14E-05	1.61E-05
**72h**	5.06E-05	3.35E-05	3.35E-06	1.35E-05
	cgd2_3370			
**2h**	0.00E+00	0.00E+00	0.00E+00	0.00E+00
**6h**	3.85E-07	3.27E-07	2.25E-06	5.75E-06
**12h**	1.01E-07	5.01E-08	3.37E-08	8.18E-08
**24h**	1.03E-06	4.13E-07	6.04E-07	4.49E-06
**36h**	6.90E-07	2.04E-06	1.97E-06	9.87E-06
**48h**	1.45E-06	2.28E-06	8.73E-07	4.45E-06
**72h**	1.33E-06	9.53E-07	1.06E-07	8.66E-06
	cgd2_3400			
**2h**	1.62E-05	9.05E-06	3.59E-07	0.00E+00
**6h**	1.27E-05	1.45E-05	1.63E-05	3.82E-06
**12h**	6.98E-06	9.65E-07	1.02E-07	0.00E+00
**24h**	3.41E-05	1.12E-05	3.23E-05	2.12E-06
**36h**	2.47E-05	2.97E-05	8.27E-07	9.64E-06
**48h**	4.54E-05	7.23E-05	1.01E-05	8.07E-05
**72h**	7.43E-05	4.06E-05	4.85E-05	2.72E-04
	cgd2_3430			
**2h**	5.58E-07	5.88E-07	3.27E-07	4.83E-08
**6h**	2.97E-07	1.31E-07	8.98E-08	1.54E-08
**12h**	1.82E-07	1.67E-06	1.63E-07	4.59E-08
**24h**	7.65E-07	1.36E-07	1.46E-07	1.19E-08
**36h**	4.14E-07	8.95E-07	3.24E-08	4.77E-08
**48h**	2.21E-07	1.40E-07	1.13E-07	9.18E-07
**72h**	2.60E-07	2.48E-07	7.58E-08	1.31E-06
	cgd2_3440			
**2h**	3.60E-07	1.83E-07	4.93E-07	6.26E-08
**6h**	1.90E-07	1.33E-07	1.84E-07	5.73E-08
**12h**	2.27E-07	4.20E-07	2.04E-07	7.92E-08
**24h**	4.50E-07	6.41E-08	1.51E-07	2.56E-08
**36h**	1.24E-07	2.66E-07	2.60E-08	4.51E-08
**48h**	2.80E-07	1.72E-07	8.50E-08	5.12E-07

**72h**	2.24E-07	6.78E-08	5.66E-08	4.26E-07
	cgd2_3450			
**2h**	0.00E+00	1.73E-08	1.45E-08	1.12E-08
**6h**	1.98E-07	2.81E-07	2.50E-07	5.00E-08
**12h**	3.31E-07	7.44E-07	9.14E-08	1.56E-07
**24h**	1.66E-06	3.77E-07	1.78E-07	6.68E-08
**36h**	2.80E-06	2.41E-06	1.05E-07	2.51E-06
**48h**	2.30E-06	7.15E-07	4.87E-07	1.59E-06
**72h**	1.14E-05	5.11E-06	9.83E-07	1.15E-05
	cgd2_3460			
**2h**	0.00E+00	0.00E+00	0.00E+00	7.21E-08
**6h**	1.32E-07	1.17E-07	6.75E-08	6.74E-09
**12h**	2.77E-08	5.88E-08	5.27E-09	7.10E-10
**24h**	1.46E-07	2.29E-08	2.54E-08	3.39E-09
**36h**	4.71E-08	1.07E-07	9.41E-09	4.23E-09
**48h**	1.98E-07	9.50E-08	1.09E-07	2.68E-07
**72h**	9.07E-07	4.53E-07	2.42E-07	7.11E-07
	cgd2_3480			
**2h**	0.00E+00	0.00E+00	0.00E+00	0.00E+00
**6h**	0.00E+00	6.86E-09	1.28E-06	0.00E+00
**12h**	2.72E-08	3.54E-05	1.05E-06	1.70E-07
**24h**	2.96E-05	1.03E-05	3.74E-06	2.08E-08
**36h**	9.50E-05	1.16E-04	1.43E-06	3.82E-06
**48h**	9.17E-05	5.08E-05	2.52E-05	9.66E-05
**72h**	9.31E-04	1.44E-04	1.15E-04	2.54E-04
	cgd2_3510			
**2h**	8.93E-09	6.69E-09	3.43E-09	9.56E-09
**6h**	4.66E-09	3.84E-09	1.07E-08	1.07E-09
**12h**	0.00E+00	2.83E-09	2.01E-09	4.39E-10
**24h**	2.79E-09	3.17E-09	6.91E-09	2.27E-09
**36h**	6.53E-09	1.75E-08	7.77E-10	2.10E-09
**48h**	1.52E-08	1.59E-08	3.72E-09	2.24E-08
**72h**	1.61E-08	1.50E-08	3.44E-09	3.23E-08
	cgd2_3530			
**2h**	0.00E+00	2.27E-09	2.65E-10	9.87E-09
**6h**	0.00E+00	2.45E-10	2.52E-09	4.92E-10
**12h**	0.00E+00	3.05E-09	6.85E-10	0.00E+00
**24h**	3.69E-09	4.31E-09	9.94E-10	5.15E-10
**36h**	2.03E-08	5.68E-08	7.37E-09	1.52E-09
**48h**	5.37E-08	3.54E-08	9.47E-09	4.28E-08
**72h**	2.66E-08	6.90E-09	4.67E-09	7.12E-08
	cgd2_3540			
**2h**	0.00E+00	0.00E+00	0.00E+00	4.71E-08
**6h**	3.44E-07	2.55E-07	5.52E-07	2.29E-08
**12h**	0.00E+00	1.25E-07	1.48E-08	0.00E+00
**24h**	3.72E-06	6.51E-07	3.00E-07	1.80E-08
**36h**	7.07E-07	5.46E-06	1.29E-08	7.48E-08
**48h**	5.81E-07	7.33E-07	1.54E-07	2.61E-06
**72h**	1.24E-06	3.45E-07	4.20E-07	6.11E-06
	cgd2_3550			
**2h**	1.07E-07	1.98E-07	3.18E-07	3.19E-07
**6h**	6.74E-07	2.52E-07	1.01E-06	9.11E-08
**12h**	1.39E-07	5.31E-07	3.10E-07	3.27E-08

**24h**	8.20E-07	1.58E-07	6.08E-07	1.07E-07
**36h**	5.29E-07	8.81E-07	1.39E-07	1.24E-07
**48h**	4.60E-07	2.42E-07	1.81E-07	5.66E-07
**72h**	1.85E-07	5.25E-08	1.29E-08	3.54E-07
	cgd2_3580			
**2h**	0.00E+00	0.00E+00	0.00E+00	4.15E-04
**6h**	3.68E-04	2.16E-04	1.62E-04	8.07E-05
**12h**	1.67E-04	2.64E-04	5.99E-05	5.73E-05
**24h**	3.14E-04	6.51E-05	4.51E-05	1.31E-05
**36h**	2.76E-04	2.84E-04	1.12E-04	1.43E-05
**48h**	2.02E-04	5.35E-05	3.74E-05	4.95E-05
**72h**	2.20E-04	1.24E-04	8.88E-05	7.83E-05
	cgd2_3590			
**2h**	0.00E+00	2.59E-08	3.06E-08	2.89E-08
**6h**	8.29E-10	5.20E-09	1.08E-09	7.77E-10
**12h**	6.28E-09	2.02E-07	1.88E-09	5.26E-10
**24h**	1.50E-07	1.50E-08	1.32E-08	8.97E-11
**36h**	2.82E-07	7.55E-07	1.71E-08	2.25E-08
**48h**	1.34E-07	1.96E-07	6.79E-08	4.64E-07
**72h**	7.53E-07	1.77E-07	3.31E-07	1.80E-06
	cgd2_3600			
**2h**	0.00E+00	0.00E+00	0.00E+00	0.00E+00
**6h**	9.49E-09	1.44E-08	8.80E-08	1.12E-09
**12h**	0.00E+00	2.98E-08	6.52E-09	1.92E-09
**24h**	8.22E-08	5.16E-08	3.50E-08	5.53E-09
**36h**	1.96E-07	4.22E-07	1.52E-08	2.70E-08
**48h**	6.09E-07	2.78E-07	2.84E-07	6.47E-07
**72h**	9.76E-06	1.93E-06	5.58E-06	1.18E-05
	cgd2_3610			
**2h**	0.00E+00	0.00E+00	0.00E+00	0.00E+00
**6h**	0.00E+00	0.00E+00	0.00E+00	0.00E+00
**12h**	5.07E-09	3.34E-10	1.69E-10	0.00E+00
**24h**	7.46E-10	2.29E-10	1.77E-10	0.00E+00
**36h**	2.82E-09	1.11E-08	1.32E-10	3.22E-10
**48h**	2.98E-08	1.73E-08	6.13E-08	3.66E-08
**72h**	1.10E-06	2.74E-07	2.02E-07	1.22E-06
	cgd2_3620			
**2h**	0.00E+00	6.13E-08	3.84E-07	0.00E+00
**6h**	0.00E+00	0.00E+00	0.00E+00	0.00E+00
**12h**	0.00E+00	6.12E-09	0.00E+00	0.00E+00
**24h**	4.99E-08	3.84E-09	6.43E-08	0.00E+00
**36h**	1.01E-06	2.03E-08	0.00E+00	0.00E+00
**48h**	2.93E-05	1.50E-05	3.53E-05	1.71E-06
**72h**	1.04E-03	1.58E-04	2.53E-04	9.69E-05
	cgd2_3630			
**2h**	4.44E-06	2.37E-05	8.60E-05	2.62E-04
**6h**	5.30E-05	8.47E-05	2.44E-04	3.42E-05
**12h**	5.36E-06	1.00E-04	3.89E-05	6.64E-07
**24h**	1.91E-04	5.72E-05	2.09E-04	2.63E-05
**36h**	2.33E-04	3.01E-04	5.70E-05	7.99E-05
**48h**	1.72E-04	6.61E-05	9.57E-05	1.05E-04
**72h**	5.04E-04	6.76E-05	1.02E-04	1.25E-04
	cgd2_3640			

**2h**	6.45E-07	2.21E-06	4.11E-07	1.11E-07
**6h**	1.36E-07	3.89E-07	9.30E-08	4.63E-08
**12h**	3.41E-07	9.96E-07	4.03E-07	3.84E-08
**24h**	1.56E-06	1.15E-06	3.31E-07	6.42E-08
**36h**	5.25E-07	7.60E-07	4.67E-08	3.03E-08
**48h**	4.30E-07	8.10E-07	1.24E-07	5.23E-08
**72h**	7.32E-07	4.76E-07	8.02E-08	2.96E-07
	cgd2_3650			
**2h**	1.37E-06	1.64E-06	1.21E-06	8.04E-08
**6h**	3.41E-07	1.38E-07	1.82E-07	5.96E-08
**12h**	1.51E-06	2.03E-06	9.61E-07	2.80E-08
**24h**	3.30E-06	7.19E-07	1.13E-06	2.89E-08
**36h**	1.37E-06	1.35E-06	2.15E-07	9.02E-08
**48h**	1.20E-06	8.57E-07	5.60E-07	1.11E-07
**72h**	5.05E-06	1.03E-06	1.83E-06	6.57E-07
	cgd2_3660			
**2h**	0.00E+00	0.00E+00	0.00E+00	0.00E+00
**6h**	0.00E+00	0.00E+00	0.00E+00	0.00E+00
**12h**	0.00E+00	1.30E-09	0.00E+00	0.00E+00
**24h**	1.16E-10	0.00E+00	9.48E-11	0.00E+00
**36h**	5.84E-11	3.00E-10	0.00E+00	6.92E-11
**48h**	9.90E-09	2.48E-08	1.32E-08	9.60E-10
**72h**	5.56E-08	6.13E-08	8.35E-08	1.82E-08
	cgd2_3670			
**2h**	0.00E+00	0.00E+00	0.00E+00	0.00E+00
**6h**	1.74E-08	0.00E+00	1.62E-08	3.84E-08
**12h**	0.00E+00	1.26E-07	1.17E-07	0.00E+00
**24h**	4.13E-07	1.71E-08	4.71E-08	0.00E+00
**36h**	2.20E-06	1.95E-06	2.34E-08	2.00E-08
**48h**	5.27E-07	1.90E-06	4.66E-07	2.07E-08
**72h**	1.41E-06	5.40E-06	2.77E-06	4.16E-07
	cgd2_3680			
**2h**	9.98E-07	8.66E-07	1.31E-06	5.16E-08
**6h**	1.22E-07	1.21E-07	5.00E-08	1.75E-09
**12h**	3.13E-07	7.94E-07	3.41E-07	4.22E-08
**24h**	2.60E-07	2.12E-07	1.84E-07	3.01E-08
**36h**	4.44E-07	9.09E-07	6.10E-08	3.05E-08
**48h**	2.79E-07	4.09E-07	1.64E-07	7.19E-08
**72h**	3.46E-08	4.38E-08	4.74E-08	3.52E-08
	cgd2_3690			
**2h**	6.08E-07	3.51E-07	5.34E-07	0.00E+00
**6h**	8.00E-08	1.01E-07	1.44E-07	0.00E+00
**12h**	2.27E-08	1.82E-08	7.78E-09	4.79E-09
**24h**	4.37E-07	7.19E-08	1.19E-07	5.53E-09
**36h**	3.58E-07	3.09E-07	3.58E-08	5.31E-09
**48h**	9.92E-08	1.92E-07	1.06E-07	1.28E-08
**72h**	2.19E-07	1.12E-07	1.77E-07	7.59E-08
	cgd2_3750			
**2h**	1.05E-07	3.59E-07	3.73E-07	3.09E-08
**6h**	0.00E+00	2.53E-09	0.00E+00	0.00E+00
**12h**	2.14E-07	5.70E-07	2.08E-07	0.00E+00
**24h**	8.02E-07	1.07E-07	2.95E-08	1.49E-09
**36h**	9.21E-07	7.90E-07	2.43E-08	1.87E-08

**48h**	7.45E-07	4.50E-07	1.88E-07	5.88E-08
**72h**	1.36E-06	1.26E-06	1.05E-06	5.32E-07
	cgd2_3770			
**2h**	0.00E+00	6.38E-09	0.00E+00	0.00E+00
**6h**	6.59E-09	3.88E-09	2.06E-08	7.40E-10
**12h**	0.00E+00	4.92E-09	7.43E-10	0.00E+00
**24h**	7.17E-08	1.26E-08	1.40E-08	9.05E-09
**36h**	1.37E-07	6.06E-08	2.45E-09	1.25E-08
**48h**	1.45E-07	3.02E-07	9.26E-08	6.79E-08
**72h**	3.44E-07	4.45E-07	1.82E-07	2.57E-07
	cgd2_3780			
**2h**	2.00E-05	2.04E-05	2.41E-05	6.73E-07
**6h**	1.28E-05	7.33E-06	5.87E-06	5.78E-07
**12h**	6.73E-06	1.17E-05	2.86E-05	5.50E-07
**24h**	1.77E-05	4.67E-06	4.63E-06	4.51E-07
**36h**	1.12E-05	1.63E-05	3.02E-06	6.57E-07
**48h**	1.82E-05	1.52E-05	4.48E-06	2.10E-06
**72h**	3.70E-06	1.53E-06	3.03E-07	1.09E-06
	cgd2_3810			
**2h**	0.00E+00	0.00E+00	0.00E+00	0.00E+00
**6h**	0.00E+00	0.00E+00	0.00E+00	0.00E+00
**12h**	0.00E+00	1.96E-09	0.00E+00	0.00E+00
**24h**	3.04E-10	0.00E+00	1.07E-10	3.61E-10
**36h**	2.63E-10	1.16E-09	1.45E-10	5.93E-10
**48h**	3.81E-08	1.68E-07	1.88E-07	4.74E-08
**72h**	2.36E-07	4.01E-07	3.90E-07	4.25E-07
	cgd2_3850			
**2h**	0.00E+00	0.00E+00	3.92E-07	0.00E+00
**6h**	0.00E+00	0.00E+00	6.13E-07	0.00E+00
**12h**	2.32E-06	1.55E-05	2.82E-06	0.00E+00
**24h**	1.10E-04	1.32E-05	1.99E-05	1.16E-06
**36h**	2.18E-04	1.17E-04	2.42E-05	5.94E-06
**48h**	2.23E-04	1.53E-04	4.56E-05	2.36E-05
**72h**	2.46E-04	1.81E-04	1.55E-03	1.39E-04
	cgd2_3860			
**2h**	0.00E+00	2.23E-08	0.00E+00	1.66E-08
**6h**	0.00E+00	2.44E-09	0.00E+00	0.00E+00
**12h**	7.74E-09	4.93E-08	2.57E-07	2.56E-09
**24h**	7.96E-08	9.27E-09	2.67E-09	2.53E-10
**36h**	6.44E-07	2.96E-07	6.98E-08	4.31E-08
**48h**	2.08E-07	1.84E-07	2.53E-07	1.11E-07
**72h**	2.21E-07	4.73E-07	3.61E-07	4.84E-07
	cgd2_3870			
**2h**	5.43E-05	0.00E+00	5.17E-04	0.00E+00
**6h**	3.34E-04	2.94E-03	3.68E-03	0.00E+00
**12h**	3.75E-05	4.50E-04	3.43E-05	1.29E-05
**24h**	6.80E-04	5.91E-04	8.13E-04	1.69E-06
**36h**	4.95E-04	8.53E-03	3.17E-04	2.70E-06
**48h**	2.22E-03	1.36E-02	1.52E-03	2.46E-05
**72h**	2.65E-03	2.15E-03	1.11E-03	1.67E-04
	cgd2_3900			
**2h**	0.00E+00	0.00E+00	0.00E+00	0.00E+00
**6h**	0.00E+00	4.81E-10	0.00E+00	0.00E+00

**12h**	2.83E-08	6.07E-08	3.95E-09	0.00E+00
**24h**	1.85E-08	1.11E-08	1.20E-08	0.00E+00
**36h**	1.08E-07	1.76E-07	1.85E-08	7.18E-07
**48h**	1.89E-07	2.82E-07	4.51E-08	1.08E-05
**72h**	3.44E-07	5.80E-08	6.62E-08	2.80E-08
	cgd2_3930			
**2h**	4.68E-06	5.39E-06	1.25E-05	3.04E-06
**6h**	4.27E-06	4.34E-06	4.38E-06	2.17E-06
**12h**	2.80E-06	6.38E-06	1.41E-05	1.17E-06
**24h**	8.16E-06	9.98E-06	9.28E-06	2.34E-06
**36h**	1.26E-05	2.13E-05	2.14E-05	3.02E-06
**48h**	3.32E-05	8.71E-05	3.29E-05	4.95E-06
**72h**	4.55E-05	2.50E-05	6.89E-06	1.10E-05
	cgd2_3950			
**2h**	5.03E-04	5.46E-04	1.89E-03	2.28E-04
**6h**	1.05E-02	3.44E-03	5.21E-03	2.08E-02
**12h**	1.33E-03	9.59E-04	2.75E-04	8.68E-05
**24h**	4.85E-03	5.20E-03	3.73E-03	3.34E-03
**36h**	2.65E-03	2.94E-03	2.84E-03	7.12E-03
**48h**	1.14E-03	2.88E-03	2.79E-04	1.18E-03
**72h**	1.06E-03	5.47E-04	4.39E-04	1.60E-04
	cgd2_3960			
**2h**	1.28E-04	8.46E-05	1.18E-04	1.51E-05
**6h**	1.93E-04	6.51E-05	1.04E-04	2.45E-05
**12h**	1.13E-04	1.35E-04	6.08E-05	2.12E-05
**24h**	5.43E-04	7.31E-05	1.23E-04	9.25E-06
**36h**	1.39E-04	1.89E-04	3.31E-05	9.17E-06
**48h**	9.83E-05	9.72E-05	5.48E-05	1.54E-05
**72h**	9.65E-05	3.05E-05	6.21E-06	2.41E-05
	cgd2_3980			
**2h**	3.14E-08	7.32E-08	1.00E-07	0.00E+00
**6h**	5.75E-09	1.33E-09	4.82E-09	5.13E-09
**12h**	8.17E-07	2.32E-06	8.32E-07	4.26E-07
**24h**	3.68E-07	1.17E-07	1.02E-07	1.53E-08
**36h**	5.15E-07	8.13E-07	2.16E-07	2.96E-08
**48h**	6.26E-07	1.10E-06	6.02E-07	1.38E-07
**72h**	9.86E-07	3.70E-07	3.39E-07	4.94E-07
	cgd2_3990			
**2h**	3.00E-07	1.34E-08	4.25E-06	0.00E+00
**6h**	8.01E-05	2.00E-05	1.14E-04	5.88E-06
**12h**	1.64E-06	4.00E-06	1.15E-06	2.44E-08
**24h**	1.99E-04	4.89E-05	3.90E-05	6.11E-07
**36h**	5.70E-05	6.18E-05	7.37E-05	1.35E-05
**48h**	7.90E-05	9.82E-05	3.18E-05	4.39E-06
**72h**	3.22E-05	8.84E-06	2.96E-06	1.89E-06
	cgd2_4000			
**2h**	2.93E-08	5.44E-08	1.01E-08	0.00E+00
**6h**	1.06E-08	1.82E-08	4.32E-09	0.00E+00
**12h**	1.05E-09	1.25E-08	1.10E-08	0.00E+00
**24h**	3.45E-08	9.42E-09	2.51E-08	0.00E+00
**36h**	4.17E-08	4.56E-08	3.52E-08	1.31E-06
**48h**	6.21E-08	2.32E-08	2.03E-08	1.63E-07
**72h**	3.58E-07	1.56E-08	3.16E-08	0.00E+00

	cgd2_4030			
**2h**	0.00E+00	0.00E+00	0.00E+00	0.00E+00
**6h**	0.00E+00	0.00E+00	0.00E+00	0.00E+00
**12h**	7.62E-09	3.43E-08	5.52E-09	0.00E+00
**24h**	1.99E-08	4.88E-09	9.82E-09	2.29E-09
**36h**	2.61E-08	3.89E-08	1.90E-08	1.14E-08
**48h**	3.65E-08	3.49E-08	7.93E-09	2.92E-09
**72h**	3.21E-07	2.32E-07	1.61E-07	5.53E-08
	cgd2_4050			
**2h**	0.00E+00	1.50E-07	0.00E+00	0.00E+00
**6h**	7.49E-08	1.36E-07	8.47E-08	0.00E+00
**12h**	8.65E-07	1.72E-07	3.41E-08	0.00E+00
**24h**	2.30E-07	1.01E-07	1.53E-07	3.59E-05
**36h**	1.80E-06	1.75E-06	7.61E-07	0.00E+00
**48h**	3.04E-05	2.48E-05	1.25E-05	8.20E-07
**72h**	2.10E-04	6.54E-05	1.13E-04	9.53E-06
	cgd2_4060			
**2h**	1.35E-07	6.04E-08	3.11E-08	0.00E+00
**6h**	4.22E-08	6.47E-09	5.21E-08	7.55E-10
**12h**	7.82E-09	2.04E-08	2.98E-09	0.00E+00
**24h**	5.17E-07	6.81E-08	5.94E-08	1.69E-08
**36h**	7.10E-07	3.23E-07	1.83E-07	2.61E-09
**48h**	1.55E-06	7.72E-07	3.64E-07	5.38E-08
**72h**	4.41E-06	1.75E-06	2.14E-06	5.55E-07
	cgd2_4070			
**2h**	5.53E-08	6.70E-09	3.02E-09	0.00E+00
**6h**	1.01E-07	4.38E-08	5.21E-08	1.24E-08
**12h**	3.15E-09	1.85E-08	2.81E-09	0.00E+00
**24h**	1.20E-07	9.11E-08	4.81E-08	9.63E-10
**36h**	1.16E-07	2.97E-07	4.36E-08	1.51E-08
**48h**	1.84E-07	1.25E-07	4.61E-08	1.03E-08
**72h**	4.62E-07	5.45E-07	0.00E+00	2.04E-07
	cgd2_4090			
**2h**	1.13E-07	2.28E-07	1.59E-07	4.65E-08
**6h**	5.80E-08	5.58E-08	3.71E-08	1.92E-09
**12h**	4.63E-08	2.88E-07	4.83E-08	0.00E+00
**24h**	7.62E-08	6.72E-08	7.72E-08	2.68E-08
**36h**	2.49E-08	1.20E-07	1.20E-08	5.60E-09
**48h**	7.13E-08	1.58E-07	4.78E-08	5.30E-08
**72h**	6.48E-08	6.71E-08	4.84E-08	3.19E-07
	cgd2_4110			
**2h**	3.92E-09	2.93E-09	6.48E-09	0.00E+00
**6h**	5.69E-07	1.84E-07	1.31E-07	1.34E-09
**12h**	1.96E-08	6.29E-08	2.29E-09	6.43E-10
**24h**	3.42E-07	3.02E-07	6.42E-08	1.88E-09
**36h**	3.38E-07	6.27E-07	2.31E-08	7.81E-09
**48h**	3.68E-07	8.34E-07	1.15E-07	3.38E-08
**72h**	4.70E-07	1.70E-07	4.63E-08	1.18E-07
	cgd2_4130			
**2h**	4.17E-10	0.00E+00	0.00E+00	0.00E+00
**6h**	0.00E+00	3.41E-11	0.00E+00	0.00E+00
**12h**	3.62E-09	4.11E-08	4.16E-09	3.11E-10
**24h**	1.11E-08	1.47E-08	1.89E-09	0.00E+00

**36h**	6.32E-08	1.81E-07	5.52E-09	1.34E-09
**48h**	2.77E-07	3.88E-07	5.92E-08	2.38E-08
**72h**	2.14E-07	8.89E-08	4.86E-08	7.12E-08
	cgd2_4150			
**2h**	2.90E-07	2.23E-08	0.00E+00	0.00E+00
**6h**	0.00E+00	0.00E+00	3.53E-08	0.00E+00
**12h**	1.31E-05	1.30E-05	6.30E-06	3.22E-07
**24h**	1.76E-06	2.44E-06	1.46E-06	8.86E-08
**36h**	1.55E-05	1.61E-05	6.55E-06	1.79E-06
**48h**	6.17E-06	8.19E-06	6.22E-06	1.63E-06
**72h**	3.59E-06	3.29E-06	8.05E-07	2.54E-06
	cgd2_4160			
**2h**	1.91E-05	2.58E-05	8.14E-06	8.44E-07
**6h**	2.62E-07	5.63E-07	4.53E-06	6.33E-07
**12h**	1.02E-05	5.80E-05	2.43E-05	3.31E-06
**24h**	6.62E-06	8.54E-06	3.72E-06	2.06E-06
**36h**	8.24E-06	7.89E-06	1.57E-06	1.90E-06
**48h**	1.12E-06	3.03E-06	2.65E-06	2.82E-06
**72h**	4.12E-06	2.12E-06	4.48E-06	2.99E-06
	cgd2_4180			
**2h**	0.00E+00	4.92E-08	2.62E-09	6.28E-09
**6h**	0.00E+00	3.23E-09	1.64E-09	3.84E-09
**12h**	4.08E-07	1.20E-06	1.31E-06	8.56E-08
**24h**	5.27E-08	9.72E-08	6.91E-08	1.92E-08
**36h**	3.40E-07	4.73E-07	9.53E-07	4.61E-07
**48h**	4.33E-07	4.06E-07	6.39E-07	2.24E-07
**72h**	6.68E-08	1.93E-08	2.79E-08	3.64E-08
	cgd2_4190			
**2h**	1.41E-09	6.28E-10	0.00E+00	0.00E+00
**6h**	1.82E-09	0.00E+00	2.50E-09	0.00E+00
**12h**	0.00E+00	1.69E-09	1.70E-10	0.00E+00
**24h**	1.67E-08	7.51E-09	1.10E-08	2.74E-10
**36h**	1.25E-08	2.31E-08	3.01E-10	1.24E-10
**48h**	1.00E-08	2.51E-08	4.44E-09	8.88E-10
**72h**	1.88E-08	8.91E-09	3.71E-09	5.59E-09
	cgd2_4200			
**2h**	1.12E-05	9.41E-06	3.12E-06	1.07E-06
**6h**	1.33E-05	6.27E-06	2.66E-06	4.37E-07
**12h**	2.98E-06	1.85E-05	7.05E-06	3.61E-07
**24h**	2.03E-05	8.72E-06	2.58E-06	2.95E-07
**36h**	2.12E-05	1.86E-05	8.63E-07	8.26E-07
**48h**	1.95E-05	2.97E-05	7.39E-06	2.70E-06
**72h**	1.58E-05	1.61E-05	4.62E-06	9.26E-06
	cgd2_4220			
**2h**	0.00E+00	0.00E+00	0.00E+00	0.00E+00
**6h**	0.00E+00	0.00E+00	0.00E+00	0.00E+00
**12h**	2.02E-08	2.00E-09	5.01E-10	0.00E+00
**24h**	1.03E-10	2.08E-09	7.46E-10	0.00E+00
**36h**	6.13E-09	5.80E-09	2.39E-09	4.88E-10
**48h**	5.02E-09	3.56E-09	5.77E-09	6.17E-10
**72h**	8.08E-10	7.77E-09	5.84E-09	1.27E-08
	cgd2_4240			
**2h**	1.38E-08	9.59E-08	2.20E-07	3.35E-08

**6h**	1.39E-07	1.24E-07	7.68E-08	6.72E-10
**12h**	8.63E-08	7.41E-08	4.85E-08	0.00E+00
**24h**	1.33E-07	5.95E-08	9.83E-08	4.92E-09
**36h**	4.72E-08	9.39E-08	2.14E-08	1.32E-09
**48h**	8.85E-08	1.61E-07	1.33E-07	1.10E-08
**72h**	4.81E-08	1.27E-08	3.06E-08	3.07E-08
	cgd2_4250			
**2h**	0.00E+00	1.03E-06	0.00E+00	0.00E+00
**6h**	1.96E-08	6.79E-09	7.67E-08	4.37E-09
**12h**	0.00E+00	2.15E-09	3.86E-09	0.00E+00
**24h**	3.21E-08	3.05E-08	5.71E-09	7.34E-10
**36h**	4.75E-08	6.99E-08	2.07E-09	3.23E-09
**48h**	2.00E-08	5.07E-08	1.12E-07	7.55E-11
**72h**	4.77E-08	2.43E-08	1.31E-08	1.32E-08
	cgd2_4260			
**2h**	2.97E-05	1.54E-05	5.05E-05	7.95E-07
**6h**	2.58E-04	2.05E-04	1.89E-04	3.77E-06
**12h**	6.39E-05	7.54E-05	1.46E-04	3.75E-06
**24h**	1.19E-04	1.25E-04	1.40E-04	8.90E-06
**36h**	3.51E-04	1.77E-04	8.79E-05	9.79E-06
**48h**	1.91E-05	3.49E-05	3.72E-05	5.35E-06
**72h**	1.34E-05	1.69E-05	9.85E-06	6.11E-06
	cgd2_4270			
**2h**	0.00E+00	0.00E+00	1.34E-09	0.00E+00
**6h**	0.00E+00	8.73E-10	0.00E+00	0.00E+00
**12h**	7.71E-08	6.80E-07	3.62E-08	2.78E-09
**24h**	7.70E-08	1.02E-08	7.52E-09	2.50E-09
**36h**	2.97E-08	2.42E-08	1.31E-09	2.88E-09
**48h**	1.93E-08	2.61E-08	4.27E-09	2.43E-09
**72h**	9.34E-09	5.97E-09	5.83E-10	6.55E-09
	cgd2_4280			
**2h**	0.00E+00	1.28E-07	4.94E-09	0.00E+00
**6h**	1.21E-06	9.19E-07	7.12E-08	3.00E-08
**12h**	0.00E+00	3.59E-07	5.16E-08	0.00E+00
**24h**	3.78E-06	1.44E-06	5.30E-07	5.10E-08
**36h**	7.09E-07	5.74E-07	4.20E-08	4.77E-08
**48h**	1.48E-06	8.43E-07	2.43E-07	1.09E-08
**72h**	5.77E-07	3.53E-07	1.07E-08	2.87E-07
	cgd2_4300			
**2h**	4.42E-07	8.05E-07	4.20E-07	1.02E-07
**6h**	4.03E-07	2.25E-07	7.90E-08	5.94E-08
**12h**	2.69E-07	9.19E-07	2.71E-07	1.04E-07
**24h**	2.17E-07	8.10E-08	8.45E-08	5.55E-08
**36h**	1.33E-07	1.24E-07	1.83E-08	3.00E-08
**48h**	9.03E-08	1.57E-07	5.18E-08	3.56E-08
**72h**	1.29E-07	1.93E-08	2.49E-08	2.68E-08
	cgd2_4320			
**2h**	4.09E-07	7.15E-07	1.65E-07	2.39E-08
**6h**	4.44E-07	2.33E-07	1.39E-07	1.54E-08
**12h**	6.21E-08	7.61E-07	2.19E-07	1.76E-08
**24h**	4.22E-07	1.65E-07	1.46E-07	1.31E-08
**36h**	1.70E-07	1.69E-07	1.55E-08	1.18E-08
**48h**	7.70E-08	2.40E-07	7.40E-08	5.52E-09

**72h**	6.28E-08	6.85E-08	2.73E-08	3.74E-08
	cgd2_4330			
**2h**	0.00E+00	1.79E-08	2.27E-08	7.88E-08
**6h**	1.46E-07	2.59E-07	6.96E-08	8.38E-08
**12h**	0.00E+00	1.86E-09	1.62E-09	6.52E-09
**24h**	1.33E-07	8.60E-08	3.93E-08	1.29E-07
**36h**	3.59E-08	4.18E-08	3.40E-09	4.01E-08
**48h**	1.17E-08	2.17E-08	4.55E-09	1.76E-08
**72h**	4.20E-08	5.01E-08	2.52E-08	5.38E-08
	cgd2_4340			
**2h**	0.00E+00	0.00E+00	3.77E-08	0.00E+00
**6h**	0.00E+00	0.00E+00	8.58E-09	0.00E+00
**12h**	4.23E-06	6.59E-06	5.50E-07	4.60E-07
**24h**	5.45E-07	5.65E-07	8.79E-08	1.35E-07
**36h**	8.87E-07	1.11E-06	1.62E-07	7.69E-08
**48h**	4.16E-06	3.37E-06	1.13E-06	2.67E-07
**72h**	1.42E-05	1.46E-05	2.51E-06	8.11E-06
	cgd2_4360			
**2h**	3.20E-09	6.90E-07	3.39E-08	0.00E+00
**6h**	0.00E+00	3.22E-09	0.00E+00	0.00E+00
**12h**	2.11E-06	1.41E-05	2.01E-06	2.14E-07
**24h**	3.48E-06	1.24E-06	2.92E-07	1.43E-07
**36h**	4.76E-06	3.38E-06	4.58E-07	8.85E-07
**48h**	1.41E-06	3.18E-06	6.69E-07	3.45E-07
**72h**	1.13E-06	2.25E-06	4.00E-07	2.01E-06
	cgd2_4380			
**2h**	3.18E-06	5.10E-06	8.92E-07	7.51E-07
**6h**	3.02E-05	1.85E-05	1.39E-05	3.55E-06
**12h**	5.49E-06	1.14E-05	1.92E-06	2.75E-07
**24h**	1.23E-05	7.48E-06	4.65E-06	1.01E-06
**36h**	3.60E-06	3.19E-06	2.44E-07	2.89E-07
**48h**	4.24E-06	6.69E-06	6.45E-07	4.62E-07
**72h**	3.26E-07	7.06E-07	3.61E-08	2.59E-07
	cgd3_30			
**2h**	3.60E-06	3.31E-06	7.51E-07	1.60E-06
**6h**	1.01E-05	9.26E-06	5.01E-06	2.23E-05
**12h**	5.99E-07	3.07E-06	3.39E-06	2.10E-06
**24h**	6.73E-06	3.57E-06	1.11E-05	1.03E-05
**36h**	3.26E-06	5.64E-06	2.76E-06	2.70E-06
**48h**	5.40E-06	1.41E-05	2.13E-05	3.75E-06
**72h**	1.67E-06	1.63E-05	2.49E-06	2.28E-06
	cgd3_40			
**2h**	0.00E+00	0.00E+00	0.00E+00	0.00E+00
**6h**	7.78E-05	2.31E-06	5.83E-07	1.25E-03
**12h**	2.33E-05	2.63E-06	1.52E-06	3.02E-04
**24h**	1.20E-05	9.80E-07	6.41E-06	1.93E-03
**36h**	8.40E-05	2.14E-05	3.96E-06	0.00E+00
**48h**	3.77E-04	1.16E-06	4.24E-07	1.63E-03
**72h**	4.70E-05	2.32E-06	7.14E-07	7.72E-04
	cgd3_50			
**2h**	5.76E-08	6.09E-07	3.32E-08	0.00E+00
**6h**	0.00E+00	0.00E+00	0.00E+00	0.00E+00
**12h**	0.00E+00	9.46E-06	5.43E-07	0.00E+00

**24h**	2.92E-07	4.54E-07	2.10E-07	4.22E-09
**36h**	3.10E-06	7.05E-06	2.92E-07	1.64E-07
**48h**	3.90E-06	1.17E-05	9.28E-06	1.12E-07
**72h**	4.14E-07	1.90E-06	7.67E-07	1.41E-06
	cgd3_70			
**2h**	2.50E-05	3.16E-04	9.62E-05	1.39E-04
**6h**	3.30E-06	2.94E-05	0.00E+00	1.46E-04
**12h**	7.49E-06	8.86E-04	1.89E-04	6.07E-05
**24h**	1.54E-06	1.58E-04	1.18E-05	5.98E-06
**36h**	1.88E-06	4.21E-05	1.22E-05	2.42E-06
**48h**	1.01E-06	2.43E-05	3.54E-05	3.19E-05
**72h**	3.48E-06	6.48E-05	7.38E-05	1.89E-05
	cgd3_80			
**2h**	9.45E-07	0.00E+00	0.00E+00	0.00E+00
**6h**	0.00E+00	0.00E+00	0.00E+00	0.00E+00
**12h**	0.00E+00	0.00E+00	0.00E+00	0.00E+00
**24h**	2.62E-07	5.97E-08	4.90E-08	0.00E+00
**36h**	1.06E-07	7.55E-07	2.34E-08	1.91E-10
**48h**	3.40E-05	5.64E-05	1.39E-05	6.14E-07
**72h**	2.74E-05	5.26E-06	5.55E-06	8.45E-06
	cgd3_90			
**2h**	0.00E+00	0.00E+00	0.00E+00	0.00E+00
**6h**	0.00E+00	0.00E+00	0.00E+00	0.00E+00
**12h**	0.00E+00	0.00E+00	3.53E-10	0.00E+00
**24h**	0.00E+00	4.19E-11	0.00E+00	0.00E+00
**36h**	2.06E-10	1.26E-09	2.09E-10	1.39E-10
**48h**	5.58E-09	7.76E-09	4.08E-09	6.55E-10
**72h**	5.24E-09	1.61E-09	6.40E-09	7.83E-09
	cgd3_100			
**2h**	0.00E+00	0.00E+00	0.00E+00	0.00E+00
**6h**	0.00E+00	0.00E+00	0.00E+00	0.00E+00
**12h**	2.77E-07	7.26E-08	2.27E-07	1.20E-07
**24h**	3.32E-08	2.96E-08	2.25E-08	2.34E-08
**36h**	2.83E-07	9.63E-08	2.88E-07	3.41E-07
**48h**	2.90E-07	4.96E-08	1.12E-07	2.04E-07
**72h**	1.13E-07	4.56E-08	3.99E-08	5.32E-08
	cgd3_110			
**2h**	2.67E-05	4.44E-06	7.56E-08	5.52E-07
**6h**	4.55E-06	1.95E-06	7.99E-07	2.16E-06
**12h**	2.59E-08	9.36E-10	8.32E-07	3.89E-07
**24h**	7.98E-07	1.95E-07	7.68E-07	6.26E-07
**36h**	3.40E-06	3.60E-07	6.28E-07	5.60E-07
**48h**	4.75E-06	5.69E-07	2.82E-07	7.18E-07
**72h**	9.91E-07	1.36E-07	6.38E-07	1.73E-07
	cgd3_140			
**2h**	8.66E-07	0.00E+00	0.00E+00	0.00E+00
**6h**	2.25E-04	6.00E-06	1.90E-04	6.17E-07
**12h**	0.00E+00	8.70E-07	4.36E-07	0.00E+00
**24h**	3.90E-04	2.78E-06	2.46E-05	2.83E-07
**36h**	1.78E-04	5.22E-05	7.95E-06	1.25E-06
**48h**	3.90E-04	6.70E-05	7.15E-05	5.05E-06
**72h**	3.16E-04	8.72E-06	5.21E-05	1.06E-04
	cgd3_160			

**2h**	6.93E-08	5.12E-09	0.00E+00	2.55E-09
**6h**	1.33E-08	3.68E-10	2.04E-08	0.00E+00
**12h**	2.92E-09	1.68E-08	1.36E-08	0.00E+00
**24h**	1.96E-08	6.18E-10	1.73E-08	1.60E-09
**36h**	1.99E-08	4.42E-08	1.72E-08	8.13E-10
**48h**	4.66E-08	5.39E-08	4.48E-08	3.96E-09
**72h**	3.48E-08	2.28E-08	3.81E-08	3.12E-08
	cgd3_190			
**2h**	0.00E+00	0.00E+00	1.22E-09	0.00E+00
**6h**	0.00E+00	0.00E+00	0.00E+00	0.00E+00
**12h**	0.00E+00	0.00E+00	1.09E+10	0.00E+00
**24h**	0.00E+00	7.29E-11	5.16E-10	0.00E+00
**36h**	2.01E-08	6.67E-09	5.82E-10	0.00E+00
**48h**	5.40E-07	1.66E-07	4.76E-07	1.03E-08
**72h**	5.93E-05	2.51E-06	2.96E-06	4.41E-06
	cgd3_210			
**2h**	6.20E-06	0.00E+00	6.85E-07	0.00E+00
**6h**	6.00E-05	1.18E-06	6.51E-05	2.32E-06
**12h**	0.00E+00	2.73E-07	1.47E-06	0.00E+00
**24h**	7.76E-05	2.87E-06	2.37E-05	1.15E-06
**36h**	2.81E-05	8.24E-05	1.51E-05	1.74E-07
**48h**	1.37E-05	1.46E-05	3.80E-05	1.18E-06
**72h**	2.33E-05	1.37E-05	2.87E-05	3.44E-06
	cgd3_220			
**2h**	0.00E+00	0.00E+00	0.00E+00	0.00E+00
**6h**	2.70E-08	2.47E-09	8.56E-10	0.00E+00
**12h**	4.93E-09	1.12E-09	8.00E-10	0.00E+00
**24h**	2.15E-08	1.65E-09	4.29E-09	5.57E-10
**36h**	3.45E-08	4.99E-08	7.89E-08	5.03E-10
**48h**	4.24E-06	1.53E-06	1.54E-06	2.26E-07
**72h**	1.57E-06	3.44E-07	2.50E-07	2.87E-07
	cgd3_250			
**2h**	2.03E-08	0.00E+00	3.33E-09	0.00E+00
**6h**	7.14E-07	5.24E-08	1.96E-07	4.15E-09
**12h**	1.59E-08	1.61E-08	6.66E-08	0.00E+00
**24h**	1.25E-06	5.77E-08	1.47E-07	4.97E-09
**36h**	5.46E-07	5.96E-07	2.51E-08	2.77E-08
**48h**	2.33E-06	3.70E-07	2.31E-07	1.81E-08
**72h**	3.21E-06	5.69E-07	5.98E-07	2.44E-07
	cgd3_260			
**2h**	3.54E-04	2.07E-04	1.51E-05	6.48E-04
**6h**	5.99E-04	1.04E-04	1.12E-04	5.06E-06
**12h**	0.00E+00	6.93E-05	2.14E-04	0.00E+00
**24h**	2.78E-04	2.05E-05	1.99E-04	7.60E-07
**36h**	5.50E-05	1.79E-04	6.22E-05	1.62E-05
**48h**	2.05E-04	9.12E-05	4.49E-05	3.48E-05
**72h**	2.74E-05	4.71E-05	2.18E-04	4.51E-05
	cgd3_270			
**2h**	0.00E+00	0.00E+00	0.00E+00	7.96E-08
**6h**	0.00E+00	0.00E+00	0.00E+00	0.00E+00
**12h**	8.81E-05	1.76E-04	3.84E-04	1.53E-04
**24h**	0.00E+00	6.58E-05	5.56E-05	3.60E-05
**36h**	3.04E-05	1.67E-04	7.89E-04	2.98E-04

**48h**	3.43E-05	7.78E-05	3.55E-04	6.14E-05
**72h**	2.52E-05	5.53E-05	8.70E-05	1.56E-05
	cgd3_280			
**2h**	7.23E-08	4.28E-07	5.31E-08	0.00E+00
**6h**	3.45E-05	8.05E-05	1.01E-04	4.22E-05
**12h**	3.63E-07	2.23E-06	9.50E-07	2.85E-06
**24h**	5.35E-06	1.09E-04	6.26E-05	2.07E-05
**36h**	0.00E+00	3.75E-05	4.04E-05	2.15E-05
**48h**	1.27E-04	4.84E-05	4.07E-05	4.25E-05
**72h**	9.05E-06	2.08E-05	2.83E-05	8.21E-06
	cgd3_300			
**2h**	2.12E-06	1.75E-03	8.87E-05	7.46E-04
**6h**	1.43E-02	1.08E-02	9.39E-03	6.71E-02
**12h**	2.90E-04	1.24E-04	1.18E-03	5.23E-03
**24h**	2.49E-03	1.16E-03	5.38E-03	2.39E-02
**36h**	2.34E-04	8.87E-04	6.05E-04	3.18E-06
**48h**	3.65E-03	4.12E-04	2.49E-04	6.08E-07
**72h**	1.75E-03	1.21E-03	7.94E-04	1.24E-03
	cgd3_310			
**2h**	0.00E+00	0.00E+00	0.00E+00	0.00E+00
**6h**	0.00E+00	0.00E+00	0.00E+00	0.00E+00
**12h**	1.52E-09	1.05E-09	2.89E-09	0.00E+00
**24h**	6.62E-09	3.36E-11	1.77E-10	2.93E-10
**36h**	2.05E-08	5.75E-09	0.00E+00	1.80E-10
**48h**	1.18E-08	6.25E-09	3.06E-09	0.00E+00
**72h**	6.24E-08	1.74E-08	2.35E-08	8.02E-09
	cgd3_330			
**2h**	5.99E-05	4.94E-05	3.26E-03	2.52E-04
**6h**	1.48E-03	2.15E-05	1.48E-04	1.25E-04
**12h**	5.33E-04	1.13E-04	1.16E-05	7.10E-04
**24h**	6.17E-05	1.21E-04	1.50E-05	5.25E-04
**36h**	6.53E-05	1.69E-04	1.72E-06	2.65E-04
**48h**	3.64E-05	8.20E-05	4.40E-06	2.34E-04
**72h**	3.41E-05	6.01E-05	1.28E-06	1.02E-04
	cgd3_340			
**2h**	1.42E-08	0.00E+00	0.00E+00	0.00E+00
**6h**	0.00E+00	0.00E+00	0.00E+00	0.00E+00
**12h**	7.20E-09	3.27E-09	2.40E-10	0.00E+00
**24h**	6.96E-09	1.16E-09	1.25E-09	8.07E-11
**36h**	3.64E-08	1.50E-08	3.57E-10	2.36E-09
**48h**	2.23E-08	2.04E-08	5.99E-09	5.00E-09
**72h**	3.95E-08	3.40E-08	3.94E-08	3.69E-08
	cgd3_350			
**2h**	1.49E-04	1.04E-04	4.52E-04	5.72E-05
**6h**	2.00E-04	1.23E-04	1.01E-04	3.45E-05
**12h**	5.50E-05	3.85E-05	7.03E-05	9.46E-06
**24h**	3.53E-04	1.27E-04	1.49E-04	1.03E-04
**36h**	8.36E-05	1.31E-04	4.20E-05	3.95E-05
**48h**	2.40E-04	7.55E-05	1.58E-04	5.95E-05
**72h**	8.10E-04	3.60E-04	2.93E-04	2.59E-04
	cgd3_380			
**2h**	4.29E-07	0.00E+00	1.99E-07	0.00E+00
**6h**	4.09E-06	7.37E-07	2.29E-06	7.33E-08

**12h**	1.36E-07	1.43E-07	3.04E-07	1.50E-08
**24h**	8.72E-06	2.61E-07	3.11E-06	4.79E-09
**36h**	6.01E-06	5.54E-06	1.76E-07	8.05E-08
**48h**	3.57E-06	1.47E-06	5.43E-07	9.33E-08
**72h**	1.96E-06	1.61E-06	1.25E-06	2.03E-06
	cgd3_390			
**2h**	3.45E-05	0.00E+00	4.76E-05	0.00E+00
**6h**	1.08E-05	2.66E-07	2.64E-06	0.00E+00
**12h**	7.47E-05	4.60E-06	2.58E-06	0.00E+00
**24h**	4.01E-05	2.33E-06	5.62E-06	8.28E-08
**36h**	6.18E-05	9.08E-06	1.28E-06	1.07E-06
**48h**	2.48E-05	1.13E-05	7.56E-06	4.35E-07
**72h**	1.27E-04	4.47E-06	1.78E-06	8.00E-06
	cgd3_400			
**2h**	2.40E-06	1.08E-05	5.76E-06	4.73E-05
**6h**	1.02E-06	4.28E-06	2.03E-06	5.27E-06
**12h**	1.64E-07	4.95E-06	2.37E-06	3.03E-06
**24h**	8.85E-07	3.05E-06	3.19E-06	1.98E-05
**36h**	1.58E-07	1.11E-06	8.12E-07	8.41E-06
**48h**	1.47E-06	4.93E-06	7.21E-06	1.02E-05
**72h**	8.97E-07	2.20E-06	2.94E-06	6.29E-06
	cgd3_410			
**2h**	3.22E-03	3.95E-04	1.90E-05	1.69E-03
**6h**	5.29E-04	1.17E-04	6.64E-06	2.88E-04
**12h**	8.49E-04	7.52E-04	2.81E-02	5.18E-04
**24h**	5.62E-04	1.09E-04	5.56E-06	1.56E-03
**36h**	1.98E-04	5.56E-05	3.59E-04	3.12E-04
**48h**	5.42E-04	2.05E-04	3.39E-06	9.59E-04
**72h**	2.70E-05	3.44E-05	1.83E-05	1.02E-04
	cgd3_420			
**2h**	1.02E-08	0.00E+00	0.00E+00	0.00E+00
**6h**	7.19E-08	5.28E-10	7.61E-09	4.52E-10
**12h**	2.12E-09	7.82E-10	9.20E-10	1.56E-09
**24h**	4.94E-08	1.45E-09	1.76E-09	0.00E+00
**36h**	1.99E-08	5.54E-09	3.03E-10	2.40E-10
**48h**	2.22E-08	1.13E-08	5.43E-09	1.27E-10
**72h**	8.82E-08	1.21E-08	5.35E-09	1.46E-08
	cgd3_430			
**2h**	0.00E+00	0.00E+00	0.00E+00	6.03E-09
**6h**	2.89E-08	9.28E-07	5.85E-07	1.67E-06
**12h**	1.24E-08	2.55E-07	6.81E-07	2.29E-06
**24h**	2.01E-08	1.11E-06	5.38E-07	2.97E-06
**36h**	1.60E-07	1.60E-05	8.82E-06	4.25E-06
**48h**	3.42E-07	3.29E-06	5.40E-06	4.86E-06
**72h**	6.92E-07	3.55E-06	5.53E-06	9.49E-06
	cgd3_440			
**2h**	3.19E-07	2.12E-08	1.34E-08	0.00E+00
**6h**	0.00E+00	0.00E+00	0.00E+00	0.00E+00
**12h**	4.54E-09	4.49E-09	1.41E-10	0.00E+00
**24h**	5.67E-08	3.16E-10	2.65E-09	0.00E+00
**36h**	2.22E-08	4.51E-08	7.99E-10	0.00E+00
**48h**	1.11E-06	2.47E-07	1.51E-07	3.49E-09
**72h**	1.85E-07	1.23E-07	2.08E-08	8.63E-08

	cgd3_450			
**2h**	2.43E-07	5.56E-09	3.03E-08	1.16E-08
**6h**	5.52E-08	5.80E-09	5.21E-09	0.00E+00
**12h**	8.34E-09	1.30E-08	7.70E-09	0.00E+00
**24h**	1.05E-07	6.91E-09	1.79E-08	2.26E-09
**36h**	5.89E-08	4.13E-08	2.90E-09	2.63E-09
**48h**	8.62E-08	4.95E-08	1.48E-08	6.81E-09
**72h**	6.59E-08	1.34E-08	7.49E-09	1.92E-08
	cgd3_460			
**2h**	4.38E-05	8.65E-06	5.07E-06	3.36E-06
**6h**	2.46E-06	1.17E-06	2.87E-07	1.84E-06
**12h**	1.73E-06	2.41E-06	4.08E-07	3.40E-07
**24h**	5.88E-06	9.62E-07	4.76E-07	6.82E-07
**36h**	2.89E-05	5.91E-06	9.92E-07	7.34E-07
**48h**	1.13E-05	5.35E-06	2.76E-06	1.16E-06
**72h**	5.56E-05	2.66E-05	1.20E-05	8.77E-06
	cgd3_470			
**2h**	2.09E-03	7.44E-04	2.42E-03	2.25E-04
**6h**	1.94E-05	1.99E-05	2.40E-05	1.14E-05
**12h**	2.96E-04	2.38E-04	5.00E-05	1.52E-05
**24h**	2.10E-04	1.44E-05	1.86E-05	4.18E-05
**36h**	4.05E-04	1.37E-04	3.72E-05	2.84E-05
**48h**	2.45E-04	7.28E-05	4.31E-05	1.47E-05
**72h**	7.88E-04	2.70E-04	2.17E-04	1.51E-04
	cgd3_480			
**2h**	0.00E+00	1.82E-06	9.01E-06	3.75E-07
**6h**	0.00E+00	0.00E+00	0.00E+00	0.00E+00
**12h**	1.95E-08	2.86E-07	1.75E-08	0.00E+00
**24h**	4.21E-07	2.46E-07	9.71E-08	0.00E+00
**36h**	9.71E-07	1.04E-06	3.04E-07	1.13E-07
**48h**	1.56E-06	9.42E-07	6.89E-07	2.73E-08
**72h**	3.85E-06	1.04E-06	2.33E-07	1.08E-06
	cgd3_500			
**2h**	2.21E-06	8.42E-08	3.38E-07	1.06E-07
**6h**	1.90E-07	5.28E-10	1.10E-08	1.51E-08
**12h**	4.38E-07	2.06E-07	6.86E-08	4.49E-08
**24h**	1.27E-06	9.81E-08	3.85E-08	1.29E-08
**36h**	5.84E-06	1.23E-06	3.59E-07	5.91E-08
**48h**	2.20E-06	2.20E-06	4.05E-07	2.68E-07
**72h**	3.20E-06	5.18E-07	1.97E-07	3.85E-07
	cgd3_510			
**2h**	7.31E-03	9.28E-04	5.83E-03	7.67E-04
**6h**	1.07E-02	8.90E-04	9.53E-04	3.71E-04
**12h**	1.72E-03	1.37E-03	3.91E-03	3.95E-04
**24h**	7.00E-03	8.24E-04	1.74E-03	9.86E-04
**36h**	2.86E-03	1.54E-03	9.48E-05	1.08E-04
**48h**	4.15E-03	3.94E-03	8.37E-04	1.73E-04
**72h**	3.12E-04	1.82E-04	1.21E-04	3.92E-04
	cgd3_520			
**2h**	6.10E-08	2.53E-09	8.57E-09	0.00E+00
**6h**	1.33E-08	0.00E+00	0.00E+00	0.00E+00
**12h**	6.19E-09	1.22E-08	5.11E-09	0.00E+00
**24h**	1.42E-08	5.26E-10	5.38E-09	2.87E-10

**36h**	6.37E-08	1.57E-08	4.23E-09	2.68E-10
**48h**	4.94E-07	2.71E-07	1.29E-07	8.76E-09
**72h**	3.70E-07	8.87E-08	9.75E-09	8.04E-08
	cgd3_530			
**2h**	0.00E+00	0.00E+00	0.00E+00	0.00E+00
**6h**	0.00E+00	0.00E+00	0.00E+00	0.00E+00
**12h**	1.60E-06	8.95E-07	2.74E-06	4.55E-08
**24h**	5.68E-06	8.44E-08	4.28E-07	4.30E-09
**36h**	2.72E-05	8.77E-06	4.24E-07	1.10E-07
**48h**	8.20E-06	4.48E-06	2.39E-06	2.33E-07
**72h**	3.47E-06	1.54E-06	2.73E-07	4.11E-06
	cgd3_560			
**2h**	0.00E+00	0.00E+00	0.00E+00	0.00E+00
**6h**	0.00E+00	4.35E-09	0.00E+00	0.00E+00
**12h**	1.22E-05	1.12E-05	6.00E-06	4.30E-06
**24h**	1.69E-04	1.37E-06	9.59E-07	6.70E-08
**36h**	4.76E-04	4.02E-05	1.19E-05	7.95E-06
**48h**	2.23E-05	2.68E-05	3.10E-05	1.26E-06
**72h**	1.58E-05	7.96E-06	1.17E-06	4.88E-06
	cgd3_570			
**2h**	0.00E+00	0.00E+00	0.00E+00	0.00E+00
**6h**	2.51E-05	2.44E-06	2.30E-06	1.11E-05
**12h**	8.20E-06	6.41E-06	7.50E-06	4.11E-05
**24h**	3.79E-06	2.84E-06	5.69E-07	1.33E-05
**36h**	5.94E-05	8.76E-06	1.30E-05	2.25E-06
**48h**	1.71E-04	5.74E-06	3.08E-06	2.34E-06
**72h**	6.00E-05	1.82E-06	1.36E-06	7.29E-06
	cgd3_580			
**2h**	0.00E+00	0.00E+00	0.00E+00	0.00E+00
**6h**	0.00E+00	0.00E+00	0.00E+00	0.00E+00
**12h**	5.72E-05	6.92E-06	4.88E-06	0.00E+00
**24h**	1.06E-04	7.26E-07	1.20E-07	0.00E+00
**36h**	8.58E-05	1.36E-05	6.25E-07	1.20E-06
**48h**	1.50E-04	1.82E-06	2.39E-08	1.38E-07
**72h**	3.72E-04	9.77E-07	1.77E-07	4.94E-07
	cgd3_590			
**2h**	1.87E-08	0.00E+00	0.00E+00	0.00E+00
**6h**	1.10E-06	7.59E-08	1.91E-07	1.18E-08
**12h**	6.03E-08	1.18E-07	2.73E-07	0.00E+00
**24h**	1.86E-06	1.39E-07	6.83E-07	0.00E+00
**36h**	1.17E-06	3.26E-06	5.94E-08	7.19E-09
**48h**	2.23E-06	1.95E-06	1.03E-06	1.06E-07
**72h**	1.49E-06	7.62E-07	8.15E-07	4.44E-07
	cgd3_600			
**2h**	4.34E-09	0.00E+00	0.00E+00	0.00E+00
**6h**	0.00E+00	0.00E+00	0.00E+00	0.00E+00
**12h**	4.68E-08	1.40E-08	1.60E-08	8.37E-09
**24h**	9.52E-08	8.14E-09	1.84E-08	0.00E+00
**36h**	3.21E-07	4.43E-07	2.54E-09	3.29E-10
**48h**	2.15E-07	1.90E-07	3.84E-08	5.34E-09
**72h**	5.49E-08	2.43E-08	1.51E-08	1.05E-08
	cgd3_610			
**2h**	4.65E-06	1.98E-06	6.81E-07	9.43E-07

**6h**	3.44E-07	1.94E-07	2.02E-07	8.64E-08
**12h**	2.29E-07	9.16E-07	1.93E-06	4.41E-07
**24h**	7.33E-07	4.48E-07	2.62E-07	2.47E-07
**36h**	6.53E-07	9.32E-07	2.35E-07	4.02E-07
**48h**	3.92E-07	3.79E-07	1.28E-07	1.89E-07
**72h**	9.24E-08	4.12E-08	2.11E-08	5.80E-08
	cgd3_620			
**2h**	0.00E+00	0.00E+00	0.00E+00	0.00E+00
**6h**	1.74E-06	0.00E+00	0.00E+00	0.00E+00
**12h**	6.67E-05	1.11E-05	1.94E-05	0.00E+00
**24h**	1.34E-04	1.41E-06	1.12E-06	0.00E+00
**36h**	2.88E-04	2.74E-04	5.83E-07	1.18E-06
**48h**	1.96E-04	1.15E-04	1.75E-05	2.66E-06
**72h**	3.53E-04	6.93E-06	2.56E-05	1.75E-05
	cgd3_630			
**2h**	5.58E-06	0.00E+00	0.00E+00	0.00E+00
**6h**	0.00E+00	0.00E+00	0.00E+00	0.00E+00
**12h**	8.26E-06	5.32E-05	5.40E-05	0.00E+00
**24h**	2.37E-04	7.82E-06	1.89E-05	0.00E+00
**36h**	1.32E-03	5.50E-04	2.27E-05	5.17E-06
**48h**	5.91E-04	3.21E-04	2.05E-04	8.33E-07
**72h**	5.15E-04	3.19E-05	3.29E-05	5.37E-05
	cgd3_640			
**2h**	3.10E-07	4.00E-08	1.48E-07	2.16E-08
**6h**	1.92E-07	2.77E-08	1.56E-08	2.06E-08
**12h**	6.87E-08	4.79E-08	4.28E-08	1.59E-08
**24h**	1.73E-07	4.21E-08	5.82E-08	1.60E-08
**36h**	1.26E-07	1.83E-07	6.56E-08	6.97E-08
**48h**	2.09E-07	1.25E-07	3.20E-08	3.39E-08
**72h**	1.94E-07	1.28E-07	3.06E-08	3.01E-07
	cgd3_660			
**2h**	4.95E-08	0.00E+00	2.53E-06	3.29E-08
**6h**	1.12E-07	8.32E-09	1.71E-08	7.76E-08
**12h**	2.96E-08	4.09E-08	1.13E-07	0.00E+00
**24h**	7.79E-07	1.13E-07	1.07E-07	2.03E-09
**36h**	2.03E-06	2.04E-06	1.47E-07	8.28E-07
**48h**	1.66E-06	1.50E-06	7.19E-07	1.40E-07
**72h**	1.20E-06	4.74E-07	2.22E-07	1.15E-06
	cgd3_670			
**2h**	4.01E-07	6.13E-08	3.54E-07	0.00E+00
**6h**	3.02E-06	3.64E-07	3.92E-07	1.08E-07
**12h**	5.96E-08	7.59E-08	1.73E-07	1.23E-08
**24h**	2.25E-06	2.80E-07	8.06E-07	2.29E-07
**36h**	9.74E-07	9.86E-07	6.26E-08	4.63E-08
**48h**	2.49E-06	3.71E-06	5.94E-07	1.16E-07
**72h**	4.31E-07	4.06E-07	1.10E-07	6.05E-07
	cgd3_680			
**2h**	1.14E-08	0.00E+00	0.00E+00	0.00E+00
**6h**	1.27E-09	2.04E-10	0.00E+00	0.00E+00
**12h**	0.00E+00	0.00E+00	6.25E-10	0.00E+00
**24h**	6.59E-09	1.43E-10	1.86E-09	0.00E+00
**36h**	1.69E-08	5.39E-09	8.48E-11	0.00E+00
**48h**	7.45E-08	2.11E-07	1.09E-08	6.64E-10

**72h**	5.95E-07	1.51E-07	5.85E-08	1.45E-07
	cgd3_710			
**2h**	0.00E+00	0.00E+00	0.00E+00	0.00E+00
**6h**	0.00E+00	0.00E+00	0.00E+00	0.00E+00
**12h**	1.21E-07	2.03E-08	9.69E-09	0.00E+00
**24h**	2.03E-08	1.68E-10	2.22E-09	5.66E-10
**36h**	1.25E-07	3.57E-08	1.56E-09	2.48E-10
**48h**	3.33E-07	1.05E-08	7.82E-09	3.18E-09
**72h**	3.80E-08	3.43E-09	0.00E+00	5.84E-09
	cgd3_720			
**2h**	0.00E+00	0.00E+00	0.00E+00	0.00E+00
**6h**	0.00E+00	0.00E+00	0.00E+00	0.00E+00
**12h**	0.00E+00	0.00E+00	0.00E+00	0.00E+00
**24h**	5.54E-10	0.00E+00	0.00E+00	0.00E+00
**36h**	0.00E+00	6.43E-09	0.00E+00	0.00E+00
**48h**	7.27E-06	5.81E-06	1.34E-06	3.49E-08
**72h**	1.84E-06	6.93E-08	1.90E-07	5.10E-07
	cgd3_730			
**2h**	3.49E-04	8.84E-06	2.84E-05	1.90E-04
**6h**	7.26E-04	7.60E-06	1.44E-05	1.33E-04
**12h**	1.04E-05	6.12E-05	1.87E-05	0.00E+00
**24h**	3.86E-04	1.61E-05	1.75E-04	3.53E-05
**36h**	1.78E-04	7.43E-05	5.61E-05	9.29E-05
**48h**	3.50E-04	2.81E-04	1.72E-05	1.77E-04
**72h**	5.28E-04	1.01E-04	2.43E-05	7.78E-05
	cgd3_740			
**2h**	0.00E+00	1.23E-08	0.00E+00	0.00E+00
**6h**	0.00E+00	0.00E+00	0.00E+00	0.00E+00
**12h**	6.06E-09	2.88E-08	3.63E-08	5.05E-09
**24h**	3.12E-08	1.23E-08	1.23E-08	6.53E-09
**36h**	2.66E-08	2.15E-08	1.80E-08	5.19E-08
**48h**	9.85E-08	8.67E-08	4.14E-08	6.80E-08
**72h**	2.85E-08	3.18E-08	1.36E-08	6.89E-08
	cgd3_750			
**2h**	7.24E-07	2.67E-08	1.07E-07	1.89E-08
**6h**	7.37E-08	2.65E-08	4.82E-08	2.32E-08
**12h**	5.72E-07	6.08E-08	8.90E-08	6.17E-08
**24h**	2.54E-07	9.82E-08	9.22E-08	1.76E-07
**36h**	2.96E-07	1.96E-07	3.12E-08	1.78E-07
**48h**	5.78E-07	2.07E-07	1.34E-07	8.50E-08
**72h**	1.96E-07	8.79E-08	1.71E-08	3.65E-07
	cgd3_760			
**2h**	9.28E-09	6.13E-09	6.34E-08	2.36E-08
**6h**	1.74E-07	3.38E-08	1.35E-08	2.19E-09
**12h**	7.99E-07	1.41E-08	1.83E-08	1.18E-05
**24h**	2.69E-07	2.71E-08	6.35E-08	1.23E-08
**36h**	1.89E-07	8.47E-08	2.61E-08	2.02E-08
**48h**	1.65E-07	1.10E-07	2.82E-08	9.20E-09
**72h**	8.12E-08	2.71E-08	1.33E-08	1.39E-07
	cgd3_770			
**2h**	0.00E+00	0.00E+00	1.63E-09	0.00E+00
**6h**	2.40E-08	1.94E-08	1.92E-08	2.71E-09
**12h**	0.00E+00	5.18E-10	7.21E-10	0.00E+00

**24h**	7.20E-08	3.52E-09	2.16E-08	0.00E+00
**36h**	7.57E-08	3.46E-08	2.81E-09	3.32E-09
**48h**	7.88E-08	1.96E-08	2.11E-08	1.71E-09
**72h**	2.12E-08	1.19E-08	2.24E-09	3.83E-08
	cgd3_790			
**2h**	1.13E-05	0.00E+00	2.56E-05	0.00E+00
**6h**	6.71E-05	1.37E-06	3.36E-06	2.22E-05
**12h**	4.50E-06	3.37E-07	4.63E-06	0.00E+00
**24h**	7.20E-05	2.21E-06	3.69E-06	0.00E+00
**36h**	2.34E-05	2.24E-05	9.15E-07	2.85E-06
**48h**	6.57E-05	1.05E-05	1.10E-06	3.14E-06
**72h**	2.73E-05	1.08E-05	4.20E-06	1.19E-05
	cgd3_800			
**2h**	9.60E-08	7.83E-08	2.26E-07	2.36E-08
**6h**	2.69E-07	1.23E-08	2.85E-08	1.60E-08
**12h**	4.03E-07	1.95E-07	1.96E-07	2.14E-08
**24h**	4.08E-07	4.88E-08	1.03E-07	2.04E-08
**36h**	6.02E-07	4.39E-07	1.16E-07	5.13E-08
**48h**	6.97E-07	3.15E-07	8.76E-08	3.71E-08
**72h**	4.38E-07	3.74E-07	1.30E-07	6.45E-07
	cgd3_810			
**2h**	0.00E+00	0.00E+00	0.00E+00	0.00E+00
**6h**	0.00E+00	0.00E+00	3.97E-09	0.00E+00
**12h**	1.56E-07	6.23E-08	2.27E-07	0.00E+00
**24h**	2.64E-07	3.27E-09	4.40E-08	1.00E-09
**36h**	2.66E-07	2.71E-07	1.65E-08	5.18E-10
**48h**	3.37E-07	7.66E-08	5.55E-08	3.88E-08
**72h**	2.73E-07	2.42E-08	6.10E-08	4.53E-08
	cgd3_850			
**2h**	0.00E+00	1.40E-08	6.11E-10	0.00E+00
**6h**	5.08E-09	1.96E-09	3.12E-09	1.69E-09
**12h**	4.33E-08	1.90E-08	5.41E-09	2.48E-08
**24h**	1.12E-08	2.00E-09	4.79E-09	1.11E-08
**36h**	4.21E-08	2.78E-08	1.44E-08	3.30E-08
**48h**	2.88E-08	7.88E-09	1.12E-08	1.45E-08
**72h**	9.67E-08	2.93E-08	1.18E-08	5.65E-08
	cgd3_860			
**2h**	0.00E+00	0.00E+00	0.00E+00	0.00E+00
**6h**	1.23E-08	1.91E-08	1.12E-07	1.25E-08
**12h**	2.33E-08	5.08E-09	1.99E-09	0.00E+00
**24h**	4.60E-08	9.72E-09	9.59E-08	4.14E-08
**36h**	1.12E-07	1.54E-07	8.68E-08	1.10E-07
**48h**	1.33E-07	1.52E-07	8.37E-08	2.75E-08
**72h**	1.35E-07	6.45E-08	1.68E-08	8.99E-08
	cgd3_880			
**2h**	1.25E-08	5.41E-09	0.00E+00	0.00E+00
**6h**	0.00E+00	8.75E-10	8.87E-10	1.08E-09
**12h**	4.20E-08	1.34E-07	6.25E-08	1.04E-07
**24h**	1.23E-08	8.24E-09	1.24E-08	3.26E-08
**36h**	4.83E-08	4.07E-08	4.40E-08	6.04E-08
**48h**	2.02E-08	2.20E-08	2.95E-08	3.00E-08
**72h**	2.39E-08	3.33E-08	2.62E-08	1.66E-08
	cgd3_890			

**2h**	2.88E-06	3.85E-06	1.04E-05	1.10E-06
**6h**	2.87E-07	3.19E-08	2.17E-07	1.16E-07
**12h**	9.78E-08	2.02E-06	8.77E-07	7.40E-07
**24h**	5.20E-06	6.66E-07	2.97E-06	5.73E-07
**36h**	5.56E-06	3.93E-06	4.99E-07	9.59E-07
**48h**	5.26E-06	6.33E-06	1.38E-06	2.20E-06
**72h**	3.93E-06	3.65E-06	3.55E-07	4.02E-06
	cgd3_930			
**2h**	2.75E-07	0.00E+00	2.26E-06	2.67E-08
**6h**	4.35E-08	1.05E-07	1.33E-07	2.74E-08
**12h**	7.72E-07	3.85E-07	3.60E-07	5.36E-08
**24h**	5.85E-07	5.70E-08	1.55E-07	1.07E-08
**36h**	8.40E-07	2.59E-06	2.27E-08	2.22E-08
**48h**	7.05E-07	1.28E-06	1.65E-06	1.00E-07
**72h**	5.80E-05	1.85E-05	1.43E-05	3.56E-05
	cgd3_950			
**2h**	0.00E+00	0.00E+00	0.00E+00	0.00E+00
**6h**	7.65E-09	0.00E+00	0.00E+00	0.00E+00
**12h**	8.18E-09	4.06E-10	8.06E-10	0.00E+00
**24h**	2.05E-07	8.92E-10	3.13E-09	2.55E-10
**36h**	1.99E-07	1.25E-07	1.47E-09	5.50E-09
**48h**	1.34E-06	2.43E-07	9.40E-08	1.34E-08
**72h**	3.21E-06	5.60E-07	6.86E-07	2.90E-07
	cgd3_960			
**2h**	0.00E+00	0.00E+00	0.00E+00	0.00E+00
**6h**	2.24E-09	0.00E+00	7.48E-10	6.41E-10
**12h**	5.05E-09	2.88E-09	1.22E-09	0.00E+00
**24h**	2.75E-08	1.79E-09	7.85E-09	2.12E-09
**36h**	8.96E-08	3.94E-08	7.17E-09	3.21E-09
**48h**	1.05E-07	3.91E-08	1.44E-08	1.47E-08
**72h**	1.32E-06	9.25E-08	1.26E-07	1.59E-07
	cgd3_970			
**2h**	9.25E-09	2.16E-09	0.00E+00	0.00E+00
**6h**	2.27E-08	0.00E+00	0.00E+00	0.00E+00
**12h**	3.70E-08	6.72E-09	5.60E-09	0.00E+00
**24h**	5.52E-08	2.05E-09	8.04E-09	6.13E-10
**36h**	2.15E-07	1.22E-07	1.65E-08	7.84E-09
**48h**	1.48E-07	8.95E-08	2.88E-08	2.16E-08
**72h**	1.00E-06	7.10E-08	1.71E-07	1.33E-07
	cgd3_980			
**2h**	5.86E-07	1.28E-07	5.23E-07	4.25E-07
**6h**	2.63E-07	8.11E-08	6.78E-08	6.16E-08
**12h**	1.02E-08	1.77E-07	1.81E-07	3.79E-07
**24h**	2.46E-07	3.30E-08	9.69E-08	4.45E-08
**36h**	1.74E-07	1.42E-07	1.15E-07	1.32E-07
**48h**	3.08E-07	2.25E-07	1.00E-07	9.84E-08
**72h**	9.86E-07	6.05E-08	1.46E-07	2.57E-07
	cgd3_1010			
**2h**	0.00E+00	0.00E+00	0.00E+00	0.00E+00
**6h**	9.36E-08	1.95E-07	1.38E-07	8.88E-08
**12h**	0.00E+00	0.00E+00	8.40E-09	1.21E-07
**24h**	2.89E-07	5.22E-07	1.65E-06	1.32E-06
**36h**	5.09E-08	2.28E-07	5.22E-07	1.04E-06

**48h**	8.29E-08	7.02E-07	1.82E-07	8.80E-08
**72h**	5.24E-08	6.84E-07	1.58E-07	6.60E-07
	cgd3_1030			
**2h**	7.80E-08	0.00E+00	3.82E-07	0.00E+00
**6h**	7.99E-07	1.45E-07	5.44E-07	3.23E-08
**12h**	0.00E+00	2.71E-08	3.80E-08	0.00E+00
**24h**	7.75E-07	7.56E-08	8.59E-07	9.19E-09
**36h**	5.71E-07	6.24E-07	1.08E-07	1.45E-08
**48h**	9.85E-07	1.19E-06	2.20E-07	3.48E-08
**72h**	2.07E-06	5.32E-07	4.08E-07	4.85E-07
	cgd3_1060			
**2h**	0.00E+00	1.30E-08	0.00E+00	0.00E+00
**6h**	2.23E-08	3.77E-08	1.26E-08	1.45E-08
**12h**	9.75E-09	6.44E-10	0.00E+00	0.00E+00
**24h**	3.98E-08	1.81E-09	2.28E-08	1.88E-09
**36h**	1.85E-08	1.87E-08	5.29E-09	1.73E-08
**48h**	4.01E-08	3.59E-08	5.80E-09	4.08E-09
**72h**	7.66E-08	2.35E-08	2.24E-08	4.32E-08
	cgd3_1070			
**2h**	1.88E-08	1.95E-09	0.00E+00	0.00E+00
**6h**	1.59E-08	7.88E-09	1.79E-08	9.76E-09
**12h**	1.55E-08	3.35E-09	4.54E-10	3.83E-09
**24h**	2.08E-08	3.12E-09	1.04E-08	6.22E-09
**36h**	7.50E-09	3.20E-09	2.24E-09	2.16E-09
**48h**	2.36E-08	7.84E-09	5.96E-09	1.62E-08
**72h**	5.39E-08	4.74E-09	1.14E-08	1.42E-08
	cgd3_1160			
**2h**	1.57E-07	9.32E-09	7.37E-08	6.60E-08
**6h**	0.00E+00	1.17E-08	0.00E+00	0.00E+00
**12h**	1.89E-07	2.40E-07	2.09E-07	6.25E-08
**24h**	6.13E-08	1.68E-08	7.35E-09	6.90E-10
**36h**	3.16E-08	3.89E-08	1.84E-08	1.81E-08
**48h**	3.50E-08	1.56E-08	2.28E-08	1.08E-08
**72h**	2.11E-08	1.67E-08	9.29E-09	2.69E-08
	cgd3_1200			
**2h**	5.82E-08	1.97E-08	3.59E-08	1.83E-08
**6h**	2.98E-08	8.18E-08	6.86E-08	7.77E-08
**12h**	1.33E-08	4.02E-08	7.63E-10	3.29E-08
**24h**	1.31E-08	2.34E-08	3.42E-08	1.29E-07
**36h**	3.19E-08	1.01E-08	6.63E-08	5.27E-08
**48h**	3.57E-08	2.40E-08	1.76E-08	3.42E-08
**72h**	2.15E-08	1.17E-08	1.69E-08	1.59E-08
	cgd3_1210			
**2h**	0.00E+00	0.00E+00	4.23E-07	0.00E+00
**6h**	1.20E-07	2.64E-08	2.41E-08	8.81E-09
**12h**	1.16E-07	0.00E+00	8.13E-09	0.00E+00
**24h**	8.31E-08	6.61E-09	1.58E-08	4.91E-08
**36h**	9.84E-08	3.24E-08	3.98E-09	1.28E-08
**48h**	7.19E-08	3.88E-08	1.11E-08	1.04E-08
**72h**	5.02E-08	2.69E-08	2.96E-08	3.23E-08
	cgd3_1220			
**2h**	5.25E-07	2.58E-07	5.69E-07	1.79E-07
**6h**	7.03E-06	8.47E-07	1.29E-06	8.12E-07

**12h**	1.79E-06	8.31E-07	5.72E-07	5.66E-07
**24h**	3.64E-06	5.50E-07	9.87E-07	5.92E-07
**36h**	1.69E-06	8.21E-07	2.51E-07	8.39E-07
**48h**	1.34E-06	4.54E-07	1.30E-07	3.58E-07
**72h**	1.18E-06	8.78E-08	3.76E-08	1.56E-07
	cgd3_1230			
**2h**	1.27E-05	1.82E-06	6.94E-06	1.50E-05
**6h**	9.73E-05	7.74E-06	8.76E-06	2.00E-05
**12h**	4.97E-06	5.12E-06	2.54E-06	7.02E-07
**24h**	1.07E-04	7.08E-06	1.12E-05	2.49E-06
**36h**	1.18E-05	9.94E-06	7.36E-07	1.55E-06
**48h**	4.34E-05	1.27E-05	7.45E-06	2.64E-06
**72h**	1.91E-05	4.48E-06	2.88E-06	5.40E-06
	cgd3_1250			
**2h**	3.37E-03	1.09E-03	3.31E-04	2.26E-03
**6h**	1.43E-02	1.72E-03	9.39E-04	5.37E-03
**12h**	1.86E-03	6.93E-04	4.40E-04	8.76E-03
**24h**	9.34E-03	3.42E-03	3.06E-03	7.22E-03
**36h**	2.23E-03	1.67E-04	4.79E-04	9.72E-04
**48h**	4.35E-04	6.69E-04	2.68E-04	6.10E-04
**72h**	6.33E-04	1.07E-04	9.50E-05	2.73E-04
	cgd3_1270			
**2h**	0.00E+00	5.96E-07	0.00E+00	0.00E+00
**6h**	2.56E-05	8.74E-07	2.04E-06	4.04E-07
**12h**	1.22E-06	7.25E-09	6.82E-08	0.00E+00
**24h**	4.75E-06	3.93E-07	6.15E-07	5.04E-07
**36h**	6.34E-06	2.50E-06	1.97E-07	2.14E-06
**48h**	8.60E-06	9.55E-07	4.08E-06	3.05E-07
**72h**	7.93E-06	1.21E-06	2.53E-07	1.32E-06
	cgd3_1290			
**2h**	0.00E+00	0.00E+00	0.00E+00	0.00E+00
**6h**	1.31E-06	6.91E-07	1.04E-06	1.61E-06
**12h**	3.56E-08	6.67E-08	3.70E-08	3.94E-09
**24h**	4.52E-06	4.38E-07	7.97E-07	1.04E-06
**36h**	1.84E-06	7.64E-07	1.65E-06	1.33E-06
**48h**	2.07E-06	9.28E-07	1.62E-06	6.93E-07
**72h**	1.45E-07	1.26E-07	5.46E-08	2.04E-07
	cgd3_1300			
**2h**	1.02E-05	1.20E-06	1.27E-06	1.13E-06
**6h**	9.19E-05	2.12E-05	2.24E-06	4.12E-06
**12h**	7.28E-06	2.64E-06	1.87E-06	1.79E-06
**24h**	5.66E-05	3.26E-06	3.96E-06	4.53E-06
**36h**	1.23E-05	2.44E-06	2.47E-06	1.11E-06
**48h**	1.21E-05	2.80E-06	1.28E-06	1.04E-06
**72h**	1.10E-06	4.71E-07	1.57E-07	4.42E-07
	cgd3_1310			
**2h**	0.00E+00	0.00E+00	5.52E-08	0.00E+00
**6h**	1.61E-06	3.90E-07	1.50E-07	1.05E-06
**12h**	2.09E-06	6.79E-08	5.70E-08	0.00E+00
**24h**	2.50E-05	1.79E-07	3.03E-06	1.36E-06
**36h**	1.27E-05	2.30E-06	1.38E-06	5.03E-07
**48h**	6.02E-06	3.42E-06	2.44E-06	1.27E-06
**72h**	4.33E-06	3.52E-07	7.27E-07	7.18E-07

	cgd3_1320			
**2h**	0.00E+00	2.18E-09	0.00E+00	0.00E+00
**6h**	2.06E-08	2.87E-09	5.79E-09	4.64E-09
**12h**	0.00E+00	3.70E-09	1.65E-09	0.00E+00
**24h**	6.33E-08	9.65E-09	8.06E-09	7.21E-09
**36h**	8.06E-08	1.52E-08	3.29E-09	4.94E-09
**48h**	2.73E-08	1.88E-08	6.68E-09	6.77E-09
**72h**	3.63E-07	6.55E-09	5.08E-09	1.67E-08
	cgd3_1330			
**2h**	0.00E+00	0.00E+00	0.00E+00	0.00E+00
**6h**	0.00E+00	0.00E+00	0.00E+00	0.00E+00
**12h**	2.05E-08	4.72E-09	1.23E-09	0.00E+00
**24h**	1.40E-09	2.97E-10	2.98E-09	6.15E-10
**36h**	1.05E-08	1.08E-08	1.72E-10	8.72E-10
**48h**	6.56E-09	3.61E-09	1.27E-09	7.44E-10
**72h**	1.95E-09	2.27E-09	5.58E-09	3.31E-09
	cgd3_1340			
**2h**	8.07E-05	1.74E-06	1.56E-05	1.02E-07
**6h**	1.13E-05	4.81E-08	2.32E-07	7.05E-08
**12h**	1.19E-05	3.72E-06	5.79E-06	2.26E-07
**24h**	1.83E-05	6.98E-07	5.25E-06	1.19E-07
**36h**	1.50E-05	3.61E-06	2.18E-07	2.83E-07
**48h**	1.48E-05	2.50E-06	1.26E-06	3.78E-07
**72h**	5.80E-06	4.73E-07	2.99E-07	4.18E-07
	cgd3_1350			
**2h**	0.00E+00	0.00E+00	2.10E-08	0.00E+00
**6h**	1.00E-03	4.67E-08	1.48E-07	4.79E-09
**12h**	1.48E-07	1.47E-09	2.12E-08	0.00E+00
**24h**	1.27E-06	1.39E-08	3.11E-07	5.15E-09
**36h**	4.51E-07	2.31E-07	2.29E-08	1.11E-09
**48h**	8.12E-07	2.16E-07	9.32E-08	1.08E-08
**72h**	2.13E-07	2.73E-08	2.83E-08	3.31E-08
	cgd3_1360			
**2h**	0.00E+00	5.64E-07	0.00E+00	0.00E+00
**6h**	1.23E-05	1.72E-06	2.12E-06	4.60E-06
**12h**	3.41E-04	2.44E-06	1.10E-05	0.00E+00
**24h**	2.39E-04	1.72E-06	7.71E-06	1.37E-07
**36h**	7.76E-04	7.29E-05	1.00E-05	3.30E-05
**48h**	9.70E-04	6.45E-05	3.43E-05	6.04E-06
**72h**	5.20E-04	1.37E-04	1.14E-05	2.04E-04
	cgd3_1370			
**2h**	0.00E+00	0.00E+00	0.00E+00	0.00E+00
**6h**	0.00E+00	0.00E+00	0.00E+00	0.00E+00
**12h**	1.84E-06	1.21E-07	1.39E-07	0.00E+00
**24h**	1.26E-07	7.75E-08	7.47E-08	2.80E-08
**36h**	3.06E-07	8.39E-07	7.96E-08	2.15E-07
**48h**	6.42E-06	9.64E-06	6.06E-06	7.42E-07
**72h**	1.89E-07	8.30E-07	1.21E-06	8.53E-07
	cgd3_1380			
**2h**	0.00E+00	0.00E+00	5.48E-09	0.00E+00
**6h**	9.11E-08	2.13E-08	1.93E-09	2.26E-08
**12h**	1.23E-08	1.21E-08	5.83E-09	1.89E-08
**24h**	2.58E-07	1.52E-08	4.54E-08	1.08E-08

**36h**	1.06E-07	6.42E-08	1.62E-08	1.40E-08
**48h**	8.57E-08	4.34E-08	3.65E-08	1.28E-08
**72h**	1.18E-07	5.56E-08	7.58E-09	1.39E-08
	cgd3_1390			
**2h**	2.45E-07	3.12E-08	3.27E-07	1.02E-08
**6h**	1.26E-06	6.93E-08	2.20E-07	2.93E-08
**12h**	1.15E-07	3.47E-08	8.48E-08	1.02E-09
**24h**	1.35E-06	1.96E-08	9.05E-08	2.06E-08
**36h**	4.60E-07	4.50E-07	2.94E-08	2.17E-08
**48h**	2.23E-07	1.74E-07	8.35E-08	1.25E-08
**72h**	1.44E-07	6.80E-08	4.10E-08	7.46E-08
	cgd3_1400			
**2h**	1.21E-08	0.00E+00	0.00E+00	0.00E+00
**6h**	0.00E+00	0.00E+00	3.10E-10	0.00E+00
**12h**	1.39E-08	1.30E-08	3.06E-08	0.00E+00
**24h**	7.04E-09	7.09E-10	1.14E-09	1.53E-09
**36h**	3.40E-08	3.74E-08	7.37E-10	3.25E-10
**48h**	4.05E-08	3.61E-08	7.38E-08	3.34E-10
**72h**	1.93E-07	2.29E-07	4.36E-08	3.68E-08
	cgd3_1420			
**2h**	0.00E+00	0.00E+00	2.87E-09	0.00E+00
**6h**	1.09E-08	2.32E-08	1.01E-08	4.09E-08
**12h**	1.30E-08	2.96E-09	4.98E-09	0.00E+00
**24h**	2.92E-08	5.35E-09	6.16E-09	4.18E-08
**36h**	3.07E-08	2.27E-08	5.62E-09	1.76E-08
**48h**	8.93E-08	7.16E-08	3.32E-08	1.86E-08
**72h**	3.22E-07	7.70E-08	3.26E-08	1.20E-07
	cgd3_1440			
**2h**	4.43E-07	2.01E-07	5.87E-08	1.61E-08
**6h**	1.90E-07	4.36E-08	2.67E-08	2.35E-09
**12h**	2.07E-08	2.02E-08	2.25E-08	1.77E-09
**24h**	1.38E-07	1.41E-08	1.85E-08	2.72E-09
**36h**	3.41E-07	9.16E-08	0.00E+00	1.24E-09
**48h**	3.08E-07	1.14E-07	5.77E-08	5.00E-09
**72h**	2.65E-07	1.46E-07	7.85E-08	1.68E-07
	cgd3_1490			
**2h**	0.00E+00	0.00E+00	0.00E+00	0.00E+00
**6h**	4.26E-07	0.00E+00	1.85E-07	0.00E+00
**12h**	0.00E+00	2.31E-08	6.91E-09	0.00E+00
**24h**	2.49E-06	2.18E-07	1.68E-07	6.17E-07
**36h**	7.30E-07	1.68E-06	3.28E-07	2.05E-07
**48h**	2.04E-06	4.46E-06	7.38E-07	3.92E-07
**72h**	1.53E-06	1.17E-06	3.47E-07	1.40E-06
	cgd3_1510			
**2h**	1.98E-08	3.67E-09	5.08E-09	0.00E+00
**6h**	2.15E-07	4.48E-09	1.98E-08	1.03E-08
**12h**	1.03E-08	0.00E+00	1.63E-09	0.00E+00
**24h**	2.92E-08	4.82E-09	1.93E-08	6.86E-09
**36h**	3.38E-08	2.45E-08	1.31E-09	3.65E-09
**48h**	2.78E-08	1.84E-08	1.58E-08	8.65E-10
**72h**	4.86E-08	1.84E-08	1.47E-08	1.95E-08
	cgd3_1520			
**2h**	7.70E-08	1.94E-07	1.77E-07	3.07E-08

**6h**	0.00E+00	0.00E+00	0.00E+00	0.00E+00
**12h**	0.00E+00	2.04E-09	1.72E-09	0.00E+00
**24h**	2.81E-08	1.11E-09	3.46E-10	1.44E-09
**36h**	4.16E-09	1.65E-09	0.00E+00	0.00E+00
**48h**	1.50E-08	2.77E-08	1.79E-08	2.17E-09
**72h**	6.83E-07	7.45E-07	1.59E-06	1.97E-04
	cgd3_1540			
**2h**	6.16E-08	0.00E+00	0.00E+00	0.00E+00
**6h**	0.00E+00	0.00E+00	0.00E+00	0.00E+00
**12h**	0.00E+00	1.74E-09	0.00E+00	0.00E+00
**24h**	7.42E-09	0.00E+00	0.00E+00	0.00E+00
**36h**	5.65E-09	5.45E-09	0.00E+00	0.00E+00
**48h**	5.25E-06	1.53E-06	1.18E-06	1.83E-08
**72h**	3.43E-05	8.75E-06	1.07E-05	4.09E-06
	cgd3_1560			
**2h**	2.80E-05	2.34E-06	4.66E-06	4.82E-07
**6h**	6.49E-06	8.72E-07	1.80E-06	4.55E-07
**12h**	5.12E-06	1.02E-06	3.13E-06	4.41E-07
**24h**	1.65E-05	1.71E-06	6.19E-06	1.57E-06
**36h**	1.27E-05	4.67E-06	5.99E-07	1.31E-06
**48h**	2.62E-06	7.79E-06	2.98E-06	2.08E-06
**72h**	2.52E-06	4.79E-07	1.03E-06	1.12E-06
	cgd3_1570			
**2h**	4.59E-06	3.99E-06	9.53E-07	3.08E-06
**6h**	2.41E-07	2.57E-07	8.15E-08	1.46E-07
**12h**	7.03E-05	5.51E-05	2.74E-05	5.03E-05
**24h**	3.64E-06	4.59E-06	2.97E-06	4.20E-06
**36h**	9.12E-06	6.06E-06	4.85E-06	7.66E-06
**48h**	3.15E-06	3.94E-06	1.49E-06	5.34E-06
**72h**	3.87E-07	1.09E-06	2.79E-07	9.01E-07
	cgd3_1590			
**2h**	2.27E-06	1.53E-07	7.22E-07	9.17E-08
**6h**	6.32E-07	4.60E-09	4.65E-09	7.80E-09
**12h**	3.02E-07	3.30E-07	5.57E-07	1.18E-08
**24h**	1.30E-06	2.98E-08	2.02E-07	4.28E-08
**36h**	2.60E-07	1.07E-07	9.56E-09	2.25E-08
**48h**	1.40E-07	7.53E-08	4.50E-08	4.88E-08
**72h**	1.39E-07	5.61E-08	2.66E-08	9.44E-08
	cgd3_1650			
**2h**	2.22E-05	2.94E-06	2.57E-05	2.48E-07
**6h**	1.10E-04	2.80E-05	3.86E-05	5.87E-06
**12h**	9.25E-06	1.94E-06	2.36E-05	3.18E-06
**24h**	2.41E-04	7.43E-06	6.01E-05	5.10E-06
**36h**	3.50E-05	2.13E-05	2.20E-06	2.23E-06
**48h**	2.14E-05	5.36E-06	5.47E-06	8.35E-07
**72h**	9.17E-06	3.93E-06	6.97E-07	3.77E-06
	cgd3_1660			
**2h**	0.00E+00	2.67E-09	0.00E+00	0.00E+00
**6h**	3.13E-09	7.01E-10	1.48E-09	7.88E-10
**12h**	5.47E-09	9.68E-10	7.37E-10	5.01E-09
**24h**	3.86E-08	6.27E-10	6.22E-10	4.07E-09
**36h**	3.87E-08	8.33E-09	9.73E-09	5.26E-09
**48h**	5.56E-08	1.94E-08	8.72E-09	1.55E-08

**72h**	2.92E-07	2.17E-08	1.44E-08	5.63E-08
	cgd3_1690			
**2h**	0.00E+00	0.00E+00	1.69E-07	0.00E+00
**6h**	0.00E+00	0.00E+00	0.00E+00	0.00E+00
**12h**	1.57E-05	8.41E-06	3.95E-05	1.15E-07
**24h**	4.00E-05	1.78E-07	1.51E-06	3.62E-07
**36h**	9.03E-05	2.35E-05	3.69E-06	2.22E-06
**48h**	4.65E-05	9.22E-06	9.30E-06	1.79E-06
**72h**	2.35E-05	4.58E-06	2.32E-06	6.04E-06
	cgd3_1710			
**2h**	1.57E-05	0.00E+00	0.00E+00	0.00E+00
**6h**	0.00E+00	0.00E+00	0.00E+00	0.00E+00
**12h**	2.34E-03	2.37E-04	2.36E-04	1.15E-04
**24h**	4.01E-04	2.32E-05	3.40E-05	1.34E-04
**36h**	6.07E-04	7.11E-05	1.34E-04	1.23E-04
**48h**	1.25E-04	1.17E-04	2.85E-05	1.09E-04
**72h**	1.59E-04	1.52E-05	2.54E-05	2.86E-05
	cgd3_1730			
**2h**	0.00E+00	0.00E+00	3.84E-09	0.00E+00
**6h**	2.29E-09	2.21E-09	2.62E-09	0.00E+00
**12h**	1.30E-07	2.76E-07	2.85E-07	1.68E-07
**24h**	3.14E-07	1.50E-08	4.52E-08	2.16E-08
**36h**	3.96E-07	1.95E-07	3.33E-08	7.45E-08
**48h**	1.12E-07	5.70E-08	3.32E-08	2.21E-08
**72h**	1.94E-07	8.19E-08	2.01E-08	3.33E-08
	cgd3_1740			
**2h**	2.70E-04	7.15E-07	4.00E-07	0.00E+00
**6h**	0.00E+00	0.00E+00	0.00E+00	0.00E+00
**12h**	1.10E-05	4.52E-05	4.01E-04	0.00E+00
**24h**	1.66E-04	1.66E-06	2.52E-05	1.02E-07
**36h**	1.18E-04	8.60E-05	1.13E-05	1.48E-06
**48h**	2.08E-04	5.52E-05	2.45E-05	6.24E-07
**72h**	1.59E-05	6.93E-06	1.39E-06	4.39E-06
	cgd3_1750			
**2h**	1.60E-06	3.06E-08	4.85E-07	0.00E+00
**6h**	0.00E+00	0.00E+00	0.00E+00	0.00E+00
**12h**	2.30E-07	6.19E-07	3.33E-06	1.10E-08
**24h**	5.73E-07	1.74E-08	1.23E-07	6.30E-09
**36h**	2.26E-06	1.24E-06	5.27E-07	1.18E-07
**48h**	6.56E-07	2.82E-07	1.18E-07	3.99E-08
**72h**	5.96E-06	1.93E-07	5.65E-08	1.44E-07
	cgd3_1780			
**2h**	0.00E+00	0.00E+00	0.00E+00	0.00E+00
**6h**	0.00E+00	0.00E+00	0.00E+00	0.00E+00
**12h**	1.65E-07	1.09E-07	6.65E-08	2.50E-10
**24h**	1.10E-07	8.06E-11	2.67E-09	8.63E-10
**36h**	5.73E-08	2.81E-08	1.73E-08	4.88E-09
**48h**	4.19E-08	2.15E-08	1.26E-08	6.02E-10
**72h**	3.05E-07	4.16E-08	7.12E-09	5.30E-08
	cgd3_1790			
**2h**	5.44E-08	0.00E+00	4.74E-08	3.09E-09
**6h**	2.47E-07	2.74E-08	4.11E-07	4.56E-10
**12h**	5.02E-09	6.24E-09	2.37E-08	0.00E+00

**24h**	1.04E-06	1.55E-08	1.14E-07	1.49E-09
**36h**	5.27E-07	5.22E-07	1.54E-08	7.02E-09
**48h**	3.73E-07	1.64E-07	2.03E-07	1.26E-08
**72h**	4.15E-07	2.62E-07	8.29E-08	3.59E-07
	cgd3_1810			
**2h**	7.58E-07	6.42E-08	2.53E-07	8.62E-09
**6h**	7.45E-07	1.54E-07	4.15E-07	7.69E-08
**12h**	4.48E-08	3.30E-08	1.79E-07	1.01E-08
**24h**	5.15E-07	5.66E-08	4.68E-07	6.43E-08
**36h**	7.48E-07	5.46E-07	1.14E-07	6.52E-08
**48h**	6.05E-07	5.37E-07	4.50E-07	1.04E-07
**72h**	1.50E-07	2.14E-07	8.16E-08	1.90E-07
	cgd3_1830			
**2h**	5.58E-09	5.77E-09	9.79E-09	0.00E+00
**6h**	1.89E-09	0.00E+00	1.91E-09	0.00E+00
**12h**	3.67E-09	0.00E+00	1.38E-08	0.00E+00
**24h**	2.08E-08	0.00E+00	3.99E-09	0.00E+00
**36h**	8.28E-08	7.42E-08	0.00E+00	8.02E-10
**48h**	1.83E-07	4.56E-08	3.68E-08	1.87E-09
**72h**	1.73E-06	2.02E-07	3.56E-08	3.18E-07
	cgd3_1840			
**2h**	1.03E-08	4.34E-09	1.53E-07	1.03E-08
**6h**	4.41E-08	4.29E-08	3.50E-07	2.86E-08
**12h**	2.73E-09	3.24E-08	2.78E-08	0.00E+00
**24h**	2.31E-09	5.07E-08	5.10E-07	1.73E-08
**36h**	3.82E-09	5.11E-07	1.16E-08	5.42E-09
**48h**	8.47E-07	6.16E-07	2.55E-07	3.32E-08
**72h**	5.41E-08	3.75E-07	1.72E-07	7.34E-07
	cgd3_1850			
**2h**	1.21E-05	2.06E-05	4.32E-05	6.31E-05
**6h**	2.17E-06	2.76E-05	8.13E-06	4.77E-06
**12h**	2.53E-05	1.14E-05	1.07E-05	1.71E-05
**24h**	2.05E-06	1.44E-06	1.99E-05	8.60E-06
**36h**	2.51E-06	5.77E-06	1.37E-06	7.53E-06
**48h**	1.50E-05	7.87E-06	3.73E-06	3.74E-06
**72h**	2.30E-06	5.36E-06	1.49E-06	1.09E-05
	cgd3_1860			
**2h**	0.00E+00	7.52E-09	0.00E+00	0.00E+00
**6h**	0.00E+00	7.67E-09	0.00E+00	0.00E+00
**12h**	0.00E+00	0.00E+00	1.22E-10	0.00E+00
**24h**	0.00E+00	2.88E-10	4.25E-10	0.00E+00
**36h**	0.00E+00	6.28E-09	1.27E-10	0.00E+00
**48h**	1.36E-07	2.18E-08	2.18E-08	2.38E-09
**72h**	2.15E-08	3.46E-08	2.46E-08	7.10E-08
	cgd3_1870			
**2h**	2.92E-08	2.16E-08	6.58E-08	0.00E+00
**6h**	3.42E-08	5.11E-08	9.92E-08	4.09E-08
**12h**	3.39E-08	1.45E-08	2.39E-08	4.06E-08
**24h**	1.47E-08	1.42E-08	8.51E-08	1.45E-08
**36h**	3.42E-08	7.35E-08	4.42E-08	7.87E-08
**48h**	2.37E-07	7.97E-08	7.69E-08	2.39E-08
**72h**	7.02E-08	7.11E-08	4.35E-08	3.65E-07
	cgd3_1880			

**2h**	0.00E+00	0.00E+00	3.42E-09	0.00E+00
**6h**	4.83E-10	0.00E+00	9.64E-10	5.93E-09
**12h**	0.00E+00	0.00E+00	0.00E+00	0.00E+00
**24h**	4.70E-10	1.03E-10	1.56E-09	0.00E+00
**36h**	3.26E-09	8.81E-09	1.77E-09	8.63E-10
**48h**	4.01E-08	2.64E-08	1.03E-08	7.04E-09
**72h**	1.40E-08	1.97E-08	1.78E-08	6.50E-08
	cgd3_1890			
**2h**	0.00E+00	0.00E+00	1.20E-09	0.00E+00
**6h**	9.73E-09	0.00E+00	3.15E-08	2.06E-09
**12h**	1.50E-09	2.00E-09	6.03E-09	0.00E+00
**24h**	6.33E-10	8.35E-10	1.27E-08	2.65E-09
**36h**	1.18E-08	2.63E-08	4.83E-09	8.63E-09
**48h**	2.01E-07	3.22E-08	2.03E-08	3.79E-09
**72h**	6.21E-08	8.45E-08	2.82E-08	2.25E-07
	cgd3_1900			
**2h**	0.00E+00	0.00E+00	4.29E-06	3.83E-06
**6h**	2.13E-07	2.21E-05	2.85E-05	0.00E+00
**12h**	0.00E+00	6.14E-07	1.08E-06	1.40E-06
**24h**	8.35E-07	2.88E-06	1.73E-05	2.60E-06
**36h**	4.01E-06	5.71E-05	1.23E-06	1.24E-05
**48h**	7.28E-05	4.44E-05	2.60E-05	2.87E-06
**72h**	5.03E-06	3.13E-05	1.65E-05	1.80E-04
	cgd3_1910			
**2h**	1.60E-08	0.00E+00	3.82E-08	6.81E-07
**6h**	1.40E-09	8.86E-07	5.68E-07	1.57E-07
**12h**	0.00E+00	2.50E-08	2.81E-07	0.00E+00
**24h**	1.25E-09	1.20E-07	4.36E-07	5.85E-09
**36h**	2.16E-07	4.60E-06	2.80E-07	5.07E-07
**48h**	6.54E-07	2.38E-07	1.65E-07	1.16E-07
**72h**	2.07E-07	2.81E-07	1.65E-07	1.31E-06
	cgd3_1920			
**2h**	0.00E+00	0.00E+00	0.00E+00	0.00E+00
**6h**	1.96E-08	1.06E-08	4.24E-09	6.37E-08
**12h**	1.11E-08	1.01E-09	1.38E-09	3.59E-09
**24h**	4.42E-08	1.65E-08	1.18E-08	7.01E-08
**36h**	4.39E-08	2.30E-08	3.60E-08	1.02E-07
**48h**	2.25E-08	1.07E-08	8.41E-09	1.50E-08
**72h**	2.29E-08	2.80E-09	1.08E-08	3.42E-08
	cgd3_1940			
**2h**	4.06E-06	9.91E-06	3.40E-06	8.33E-06
**6h**	5.98E-06	7.55E-06	2.75E-06	1.46E-05
**12h**	9.82E-06	6.43E-06	4.02E-06	1.36E-05
**24h**	5.66E-06	4.14E-06	3.08E-06	1.32E-05
**36h**	2.71E-06	4.64E-06	2.44E-06	4.31E-06
**48h**	3.17E-06	1.24E-06	1.02E-06	4.94E-06
**72h**	4.30E-07	3.77E-07	1.12E-07	5.96E-07
	cgd3_1950			
**2h**	0.00E+00	0.00E+00	0.00E+00	0.00E+00
**6h**	0.00E+00	0.00E+00	0.00E+00	1.30E-07
**12h**	7.43E-07	0.00E+00	0.00E+00	0.00E+00
**24h**	6.37E-08	2.92E-07	4.39E-07	0.00E+00
**36h**	2.67E-06	4.59E-06	6.33E-07	4.04E-06

**48h**	1.34E-05	1.49E-06	5.16E-07	4.58E-06
**72h**	2.05E-06	2.65E-06	4.48E-07	4.25E-06
	cgd3_1960			
**2h**	0.00E+00	0.00E+00	2.98E-09	0.00E+00
**6h**	8.21E-10	8.77E-10	4.78E-08	9.94E-09
**12h**	1.54E-09	2.95E-09	4.37E-09	0.00E+00
**24h**	3.99E-09	0.00E+00	9.12E-09	2.26E-09
**36h**	2.21E-08	6.42E-08	1.14E-08	1.55E-08
**48h**	5.47E-08	1.61E-08	1.38E-08	3.33E-09
**72h**	2.82E-08	1.19E-08	6.77E-09	4.60E-08
	cgd3_1980			
**2h**	0.00E+00	0.00E+00	0.00E+00	0.00E+00
**6h**	1.94E-05	4.61E-06	4.16E-04	2.62E-07
**12h**	4.02E-07	5.56E-07	6.66E-06	0.00E+00
**24h**	3.87E-06	3.71E-06	7.14E-05	3.29E-06
**36h**	1.03E-06	8.23E-05	2.96E-06	1.52E-06
**48h**	1.30E-04	7.71E-05	3.52E-05	1.70E-05
**72h**	9.37E-06	3.73E-06	1.39E-06	1.15E-05
	cgd3_2000			
**2h**	0.00E+00	0.00E+00	2.26E-05	0.00E+00
**6h**	0.00E+00	0.00E+00	1.81E-04	1.37E-05
**12h**	0.00E+00	0.00E+00	1.56E-05	9.97E-06
**24h**	2.50E-06	1.19E-06	6.79E-05	8.09E-06
**36h**	3.92E-05	6.76E-06	2.52E-05	1.44E-05
**48h**	3.42E-05	4.48E-06	1.87E-05	2.63E-06
**72h**	7.20E-05	4.17E-06	4.13E-05	1.24E-05
	cgd3_2010			
**2h**	2.41E-08	0.00E+00	2.40E-07	0.00E+00
**6h**	3.96E-09	2.09E-09	1.68E-09	1.55E-08
**12h**	1.75E-08	2.82E-08	3.59E-07	0.00E+00
**24h**	2.09E-09	1.21E-08	4.12E-08	1.08E-08
**36h**	2.35E-08	4.62E-07	3.21E-08	3.46E-08
**48h**	3.32E-07	8.43E-08	3.97E-08	3.65E-08
**72h**	2.99E-08	5.52E-08	5.20E-08	1.28E-07
	cgd3_2030			
**2h**	0.00E+00	0.00E+00	3.03E-09	0.00E+00
**6h**	0.00E+00	7.67E-09	0.00E+00	0.00E+00
**12h**	0.00E+00	0.00E+00	0.00E+00	0.00E+00
**24h**	4.10E-09	0.00E+00	1.19E-08	3.50E-08
**36h**	6.57E-09	2.17E-08	1.19E-08	2.45E-08
**48h**	5.05E-08	8.60E-09	6.68E-09	8.79E-09
**72h**	1.12E-08	1.66E-08	3.44E-09	5.08E-08
	cgd3_2040			
**2h**	0.00E+00	0.00E+00	8.58E-09	0.00E+00
**6h**	5.06E-09	2.37E-09	9.82E-08	6.70E-08
**12h**	0.00E+00	2.38E-09	1.47E-08	0.00E+00
**24h**	8.58E-09	1.48E-08	3.17E-08	1.79E-08
**36h**	7.99E-09	1.25E-07	1.81E-08	1.03E-08
**48h**	3.25E-07	8.93E-08	4.99E-08	1.45E-08
**72h**	1.34E-08	3.80E-08	4.55E-09	1.92E-08
	cgd3_2050			
**2h**	0.00E+00	2.90E-08	7.15E-08	0.00E+00
**6h**	1.43E-08	6.38E-08	6.51E-07	1.10E-07

**12h**	0.00E+00	3.36E-09	0.00E+00	0.00E+00
**24h**	3.39E-08	2.34E-07	2.31E-07	1.37E-08
**36h**	1.41E-07	2.59E-06	2.25E-07	2.55E-08
**48h**	2.89E-06	1.65E-06	3.19E-07	1.25E-07
**72h**	7.66E-08	2.48E-07	5.45E-08	4.64E-07
	cgd3_2060			
**2h**	0.00E+00	0.00E+00	5.45E-09	0.00E+00
**6h**	2.25E-08	5.16E-08	1.73E-07	6.66E-08
**12h**	1.15E-09	7.73E-10	4.52E-08	0.00E+00
**24h**	1.63E-09	1.90E-08	1.90E-07	2.33E-08
**36h**	1.79E-08	3.95E-07	1.83E-08	1.70E-08
**48h**	1.18E-07	9.24E-08	9.52E-08	9.29E-09
**72h**	6.27E-09	8.31E-09	6.95E-09	1.63E-08
	cgd3_2070			
**2h**	0.00E+00	1.16E-08	0.00E+00	3.20E-08
**6h**	1.21E-08	1.59E-09	7.77E-09	5.11E-10
**12h**	6.53E-09	1.28E-09	2.34E-09	0.00E+00
**24h**	2.50E-08	4.60E-09	1.02E-08	1.44E-09
**36h**	1.35E-08	7.46E-09	1.78E-08	4.39E-09
**48h**	7.69E-09	4.57E-09	1.04E-09	1.10E-10
**72h**	1.27E-08	1.56E-09	6.29E-10	4.53E-09
	cgd3_2090			
**2h**	2.73E-06	8.38E-07	2.76E-06	3.27E-06
**6h**	3.50E-05	1.19E-05	4.14E-06	1.09E-05
**12h**	7.44E-06	2.02E-06	3.45E-06	1.02E-05
**24h**	1.66E-05	4.35E-06	1.49E-05	1.55E-05
**36h**	1.15E-05	3.26E-06	3.87E-06	6.22E-06
**48h**	2.22E-06	6.19E-07	7.12E-07	1.54E-06
**72h**	1.03E-06	1.27E-07	2.54E-07	6.00E-07
	cgd3_2100			
**2h**	1.61E-08	2.43E-08	4.16E-07	4.41E-08
**6h**	1.00E-09	4.83E-09	9.93E-08	2.35E-08
**12h**	6.09E-09	3.49E-08	1.68E-07	4.14E-08
**24h**	7.88E-09	3.31E-09	2.39E-08	4.43E-09
**36h**	1.32E-08	3.98E-08	1.87E-08	1.12E-08
**48h**	6.82E-08	3.38E-08	5.73E-08	5.14E-10
**72h**	3.21E-08	2.37E-08	1.27E-08	7.98E-08
	cgd3_2120			
**2h**	0.00E+00	2.34E-09	0.00E+00	0.00E+00
**6h**	0.00E+00	0.00E+00	0.00E+00	0.00E+00
**12h**	9.97E-10	8.86E-09	2.36E-09	3.89E-09
**24h**	9.25E-10	0.00E+00	6.83E-09	0.00E+00
**36h**	2.59E-10	3.88E-08	2.10E-09	5.64E-10
**48h**	2.86E-08	8.78E-09	1.89E-08	0.00E+00
**72h**	1.26E-08	1.64E-08	1.36E-08	3.48E-08
	cgd3_2130			
**2h**	2.61E-05	6.22E-05	3.86E-04	1.00E-04
**6h**	1.77E-04	1.78E-04	1.34E-03	3.72E-04
**12h**	1.88E-05	1.30E-04	6.82E-04	1.34E-04
**24h**	5.40E-05	3.18E-04	4.95E-04	3.76E-04
**36h**	2.78E-04	1.77E-04	3.35E-05	9.07E-04
**48h**	2.32E-04	2.75E-04	3.19E-04	9.74E-05
**72h**	8.45E-05	1.32E-04	9.03E-05	6.41E-04

	cgd3_2140			
**2h**	0.00E+00	7.56E-10	1.08E-08	0.00E+00
**6h**	1.14E-07	4.24E-08	2.46E-07	7.81E-08
**12h**	3.36E-09	4.35E-09	6.01E-09	1.93E-09
**24h**	0.00E+00	8.55E-09	1.11E-07	2.30E-08
**36h**	4.93E-08	9.74E-08	2.15E-08	4.74E-08
**48h**	2.88E-07	2.16E-07	9.40E-08	6.63E-08
**72h**	2.27E-08	4.82E-08	3.87E-08	3.24E-08
	cgd3_2160			
**2h**	2.93E-08	3.26E-09	0.00E+00	3.33E-09
**6h**	7.77E-07	3.60E-07	3.87E-07	2.69E-07
**12h**	5.13E-09	8.12E-09	1.21E-08	1.84E-08
**24h**	4.15E-07	2.31E-07	1.42E-07	4.12E-07
**36h**	8.30E-08	6.53E-08	1.62E-07	2.31E-07
**48h**	8.67E-08	1.25E-07	5.14E-08	1.48E-07
**72h**	1.96E-08	2.32E-08	1.34E-08	5.57E-08
	cgd3_2170			
**2h**	0.00E+00	0.00E+00	0.00E+00	0.00E+00
**6h**	1.08E-07	1.20E-07	2.90E-07	9.80E-08
**12h**	5.08E-09	6.44E-09	6.08E-09	1.46E-09
**24h**	4.92E-08	1.40E-07	8.88E-08	3.90E-08
**36h**	3.07E-08	7.22E-08	8.98E-08	4.75E-08
**48h**	7.84E-08	1.72E-07	9.47E-08	2.83E-08
**72h**	2.45E-08	3.91E-08	4.14E-08	3.27E-08
	cgd3_2200			
**2h**	0.00E+00	0.00E+00	0.00E+00	0.00E+00
**6h**	2.08E-07	8.57E-08	3.11E-07	2.81E-07
**12h**	0.00E+00	5.17E-09	2.00E-09	3.66E-09
**24h**	7.92E-08	4.97E-08	2.00E-07	1.12E-07
**36h**	9.64E-08	3.82E-07	1.38E-07	1.97E-07
**48h**	1.58E-07	1.75E-07	7.17E-08	6.01E-08
**72h**	1.07E-08	5.54E-08	1.31E-08	3.01E-08
	cgd3_2210			
**2h**	0.00E+00	0.00E+00	2.84E-09	0.00E+00
**6h**	4.51E-09	1.36E-09	3.08E-08	5.54E-09
**12h**	1.41E-09	1.96E-09	4.88E-09	1.96E-09
**24h**	2.14E-09	3.47E-09	3.20E-08	7.14E-09
**36h**	1.50E-08	6.97E-08	2.56E-08	2.80E-08
**48h**	6.64E-08	7.59E-08	1.73E-08	1.15E-08
**72h**	3.18E-08	6.70E-08	8.46E-09	8.77E-08
	cgd3_1800			
**2h**	0.00E+00	0.00E+00	0.00E+00	0.00E+00
**6h**	0.00E+00	0.00E+00	0.00E+00	0.00E+00
**12h**	8.87E-10	4.15E-09	4.31E-10	0.00E+00
**24h**	6.68E-10	3.51E-10	1.29E-09	3.02E-09
**36h**	1.01E-09	6.59E-09	4.26E-10	2.75E-09
**48h**	2.14E-08	2.12E-08	7.98E-09	6.98E-09
**72h**	1.82E-08	3.14E-08	2.16E-08	2.63E-08
	cgd3_2250			
**2h**	2.78E-06	2.68E-07	2.23E-07	1.40E-06
**6h**	2.85E-06	1.59E-06	1.22E-06	2.34E-06
**12h**	3.28E-06	5.88E-07	3.85E-07	3.20E-06
**24h**	1.47E-06	1.01E-06	1.35E-06	4.22E-06

**36h**	1.17E-06	5.01E-07	2.88E-07	1.06E-06
**48h**	9.14E-07	1.75E-06	2.91E-07	3.22E-06
**72h**	2.04E-07	2.76E-07	1.22E-07	2.23E-07
	cgd3_2280			
**2h**	0.00E+00	9.32E-07	1.78E-08	0.00E+00
**6h**	0.00E+00	3.05E-07	0.00E+00	0.00E+00
**12h**	2.82E-08	5.98E-07	2.79E-08	0.00E+00
**24h**	1.13E-09	1.50E-07	4.19E-08	2.16E-10
**36h**	2.55E-09	1.72E-06	7.34E-09	6.02E-09
**48h**	7.28E-08	5.85E-07	4.35E-08	5.86E-09
**72h**	4.38E-08	6.73E-07	3.43E-08	5.42E-08
	cgd3_2300			
**2h**	1.64E-07	1.23E-06	6.38E-07	4.44E-06
**6h**	1.74E-07	6.12E-07	2.65E-07	6.98E-07
**12h**	2.62E-07	8.73E-07	5.10E-07	2.17E-06
**24h**	5.95E-08	1.27E-06	2.25E-07	9.43E-07
**36h**	3.30E-07	2.30E-07	2.18E-07	4.71E-06
**48h**	1.30E-06	4.47E-07	1.44E-07	3.52E-06
**72h**	1.81E-07	1.84E-07	7.70E-07	9.32E-07
	cgd3_2310			
**2h**	3.63E-07	2.50E-07	2.27E-07	7.02E-07
**6h**	2.45E-06	6.37E-07	6.51E-07	4.61E-07
**12h**	9.97E-08	4.54E-07	3.78E-07	1.32E-06
**24h**	4.30E-07	2.57E-07	7.26E-07	6.53E-07
**36h**	4.88E-08	4.70E-07	1.63E-07	2.67E-07
**48h**	2.38E-07	7.44E-07	2.67E-07	4.73E-07
**72h**	9.56E-08	1.78E-07	5.72E-08	1.67E-07
	cgd3_2320			
**2h**	3.24E-08	3.21E-09	9.78E-08	1.67E-08
**6h**	4.94E-09	8.30E-09	1.40E-08	7.18E-09
**12h**	1.47E-09	6.21E-09	1.56E-08	4.77E-09
**24h**	1.36E-09	2.21E-09	9.60E-09	3.73E-09
**36h**	9.41E-09	4.47E-08	3.53E-09	6.48E-09
**48h**	5.87E-08	3.18E-08	1.59E-08	8.69E-09
**72h**	5.35E-09	2.46E-08	5.47E-09	2.93E-08
	cgd3_2330			
**2h**	0.00E+00	0.00E+00	0.00E+00	0.00E+00
**6h**	7.64E-09	0.00E+00	2.74E-08	0.00E+00
**12h**	1.88E-07	5.71E-07	1.81E-06	1.23E-07
**24h**	8.89E-09	1.36E-08	5.19E-07	1.33E-08
**36h**	1.19E-08	0.00E+00	2.31E-08	6.99E-09
**48h**	2.81E-07	3.67E-07	2.65E-07	5.10E-08
**72h**	4.33E-08	1.27E-07	9.53E-08	1.52E-07
	cgd3_2340			
**2h**	7.27E-05	1.22E-05	9.34E-06	4.30E-05
**6h**	1.18E-04	3.89E-05	2.01E-05	1.37E-04
**12h**	2.83E-05	1.18E-05	1.56E-05	1.58E-04
**24h**	8.86E-05	2.87E-05	4.58E-05	1.69E-04
**36h**	2.21E-05	1.81E-05	2.17E-05	3.88E-05
**48h**	4.41E-05	1.23E-05	9.69E-06	3.44E-05
**72h**	1.25E-05	7.69E-06	2.50E-06	2.55E-05
	cgd3_2360			
**2h**	0.00E+00	0.00E+00	3.55E-08	2.56E-08

**6h**	3.13E-09	7.28E-08	9.18E-07	0.00E+00
**12h**	2.32E-09	5.21E-08	5.01E-08	0.00E+00
**24h**	9.33E-09	6.19E-09	4.28E-07	2.34E-09
**36h**	2.11E-08	9.27E-07	2.94E-08	1.01E-08
**48h**	6.70E-07	2.41E-07	2.20E-07	2.17E-08
**72h**	1.92E-08	5.15E-08	1.99E-08	1.88E-07
	cgd3_2370			
**2h**	4.20E-09	9.24E-09	2.48E-09	0.00E+00
**6h**	1.05E-08	9.70E-09	2.81E-07	1.99E-08
**12h**	0.00E+00	3.03E-09	2.22E-08	0.00E+00
**24h**	3.78E-09	1.26E-09	1.20E-07	5.52E-09
**36h**	2.44E-08	1.01E-07	1.27E-08	1.71E-08
**48h**	2.65E-07	5.10E-08	5.44E-08	4.11E-08
**72h**	3.31E-08	2.65E-08	7.55E-09	5.02E-08
	cgd3_2400			
**2h**	1.67E-08	6.03E-08	1.83E-07	5.52E-09
**6h**	5.29E-09	3.08E-08	8.84E-08	1.48E-08
**12h**	2.09E-09	1.08E-08	1.59E-08	1.63E-09
**24h**	1.02E-08	5.20E-09	8.33E-08	1.96E-08
**36h**	2.27E-08	8.55E-08	1.90E-08	2.18E-08
**48h**	6.60E-08	2.43E-08	3.56E-08	1.54E-08
**72h**	3.25E-08	3.43E-08	2.38E-08	5.71E-08
	cgd3_2430			
**2h**	5.77E-09	0.00E+00	7.14E-09	1.17E-08
**6h**	0.00E+00	0.00E+00	8.50E-10	0.00E+00
**12h**	0.00E+00	8.22E-10	7.38E-10	0.00E+00
**24h**	7.70E-10	0.00E+00	3.97E-09	2.78E-10
**36h**	1.72E-09	5.18E-09	5.75E-10	3.09E-09
**48h**	3.47E-08	0.00E+00	1.01E-08	5.13E-09
**72h**	3.26E-08	2.48E-08	1.11E-08	1.73E-08
	cgd3_2440			
**2h**	1.32E-05	5.65E-06	7.44E-05	1.50E-05
**6h**	7.52E-05	6.10E-05	1.74E-04	5.87E-05
**12h**	3.26E-05	3.28E-05	1.23E-04	6.03E-05
**24h**	7.99E-05	5.70E-05	1.46E-04	1.19E-04
**36h**	2.91E-05	4.94E-05	3.86E-05	4.09E-05
**48h**	1.03E-04	3.60E-05	5.02E-05	7.92E-05
**72h**	4.84E-06	1.32E-06	1.49E-06	6.77E-06
	cgd3_2450			
**2h**	0.00E+00	0.00E+00	1.62E-08	2.27E-09
**6h**	0.00E+00	0.00E+00	7.82E-10	0.00E+00
**12h**	0.00E+00	6.45E-10	4.76E-09	8.21E-09
**24h**	4.47E-10	5.32E-11	1.55E-09	0.00E+00
**36h**	2.01E-09	1.85E-08	2.85E-09	3.16E-09
**48h**	2.36E-08	1.67E-08	5.99E-09	1.16E-10
**72h**	3.48E-09	2.63E-09	2.22E-09	9.71E-09
	cgd3_2460			
**2h**	3.91E-07	9.96E-07	1.54E-06	4.70E-07
**6h**	2.72E-07	5.79E-07	1.01E-06	4.21E-07
**12h**	3.74E-07	5.70E-07	8.62E-07	1.66E-06
**24h**	1.51E-07	1.84E-07	6.03E-07	2.99E-07
**36h**	6.92E-07	6.93E-07	1.18E-07	7.10E-07
**48h**	1.47E-06	4.73E-07	2.79E-07	6.63E-07

**72h**	2.57E-07	3.72E-07	1.12E-07	4.06E-07
	cgd3_2470			
**2h**	6.05E-06	1.32E-05	5.78E-06	4.62E-06
**6h**	2.28E-06	6.67E-06	3.00E-06	2.22E-07
**12h**	2.47E-06	6.16E-06	5.50E-06	7.37E-06
**24h**	2.84E-06	1.46E-06	1.16E-05	3.61E-06
**36h**	4.27E-06	2.10E-06	7.91E-07	4.06E-06
**48h**	6.91E-06	1.45E-06	8.61E-07	2.23E-06
**72h**	5.49E-07	5.16E-07	8.76E-07	1.21E-06
	cgd3_2520			
**2h**	9.99E-10	8.50E-09	1.70E-08	1.64E-08
**6h**	8.84E-09	8.95E-09	4.62E-08	4.23E-08
**12h**	0.00E+00	8.66E-10	2.84E-09	0.00E+00
**24h**	2.33E-09	5.27E-09	1.35E-08	2.06E-09
**36h**	9.87E-09	2.84E-08	1.16E-08	1.44E-08
**48h**	1.17E-07	1.64E-08	1.31E-08	2.67E-08
**72h**	1.40E-08	1.99E-08	4.53E-09	3.34E-08
	cgd3_2540			
**2h**	8.28E-07	8.51E-07	2.16E-07	1.38E-06
**6h**	4.68E-06	1.08E-06	4.86E-07	5.09E-06
**12h**	1.49E-06	1.49E-06	6.28E-07	2.90E-06
**24h**	2.03E-06	5.90E-07	2.86E-07	2.70E-06
**36h**	2.62E-06	1.57E-06	1.73E-06	6.32E-06
**48h**	2.51E-06	1.69E-06	6.03E-07	4.43E-06
**72h**	3.99E-07	3.72E-07	1.52E-07	1.34E-06
	cgd3_2560			
**2h**	0.00E+00	0.00E+00	3.67E-08	0.00E+00
**6h**	1.04E-04	2.42E-06	1.30E-06	3.57E-06
**12h**	0.00E+00	6.33E-08	1.69E-07	0.00E+00
**24h**	1.98E-06	2.52E-07	2.58E-06	2.39E-06
**36h**	2.47E-06	2.89E-06	3.90E-06	6.93E-06
**48h**	4.23E-06	1.15E-06	4.15E-07	7.64E-07
**72h**	1.09E-06	2.19E-06	1.27E-06	5.67E-06
	cgd3_2570			
**2h**	1.01E-08	1.44E-08	1.07E-08	0.00E+00
**6h**	7.78E-08	2.27E-08	3.25E-08	7.38E-08
**12h**	8.14E-09	9.22E-09	1.38E-08	0.00E+00
**24h**	5.55E-08	3.65E-08	7.92E-08	1.45E-08
**36h**	2.26E-08	7.65E-08	2.60E-08	9.84E-09
**48h**	2.22E-07	1.56E-07	6.57E-08	1.73E-08
**72h**	4.93E-08	5.95E-08	6.33E-08	1.04E-07
	cgd3_2650			
**2h**	0.00E+00	0.00E+00	1.08E-09	3.74E-08
**6h**	3.82E-10	3.41E-09	2.49E-08	0.00E+00
**12h**	1.02E-09	6.31E-10	9.86E-09	0.00E+00
**24h**	1.76E-09	6.82E-09	7.18E-08	2.31E-09
**36h**	1.84E-09	4.55E-08	3.12E-09	7.05E-10
**48h**	8.46E-08	4.52E-08	1.66E-08	1.29E-09
**72h**	9.51E-09	8.00E-09	3.04E-09	4.24E-09
	cgd3_2670			
**2h**	5.99E-08	2.46E-08	2.82E-08	0.00E+00
**6h**	1.04E-07	2.67E-08	6.48E-09	8.86E-08
**12h**	4.40E-09	1.06E-08	6.06E-08	1.82E-07

**24h**	2.92E-08	1.47E-08	4.21E-08	4.22E-08
**36h**	1.41E-08	8.01E-08	3.74E-09	5.06E-08
**48h**	6.10E-08	8.78E-08	1.79E-08	4.93E-09
**72h**	2.25E-08	3.31E-08	1.50E-08	7.66E-08
	cgd3_2690			
**2h**	3.70E-09	0.00E+00	0.00E+00	0.00E+00
**6h**	2.30E-08	2.79E-08	1.33E-07	5.32E-09
**12h**	2.03E-09	2.61E-09	9.41E-09	2.01E-09
**24h**	8.26E-09	1.98E-08	5.48E-08	1.31E-08
**36h**	8.95E-09	1.86E-07	3.19E-08	9.54E-09
**48h**	1.07E-07	3.00E-08	1.88E-08	2.19E-09
**72h**	6.36E-09	2.23E-08	3.19E-08	2.25E-08
	cgd3_2770			
**2h**	8.37E-09	1.53E-08	3.64E-08	1.55E-08
**6h**	3.76E-08	5.85E-08	2.14E-07	4.18E-08
**12h**	1.40E-09	2.45E-09	1.45E-08	1.30E-08
**24h**	6.84E-09	1.76E-08	1.05E-07	2.11E-08
**36h**	1.01E-08	1.54E-07	9.30E-09	1.51E-08
**48h**	1.60E-07	1.02E-07	6.53E-08	1.41E-08
**72h**	9.70E-09	3.38E-08	9.06E-09	5.31E-08
	cgd3_2780			
**2h**	0.00E+00	0.00E+00	1.21E-08	7.01E-09
**6h**	1.38E-08	6.88E-09	6.50E-08	3.28E-08
**12h**	1.37E-09	0.00E+00	1.85E-08	0.00E+00
**24h**	8.35E-09	1.08E-08	5.23E-08	3.24E-09
**36h**	6.16E-09	5.14E-08	3.78E-09	6.10E-09
**48h**	6.22E-08	3.42E-08	2.77E-08	5.32E-09
**72h**	6.26E-09	9.90E-09	6.88E-09	1.56E-08
	cgd3_2790			
**2h**	0.00E+00	0.00E+00	0.00E+00	0.00E+00
**6h**	3.00E-07	1.23E-06	1.25E-06	5.27E-06
**12h**	0.00E+00	3.43E-08	4.73E-08	0.00E+00
**24h**	3.72E-07	5.67E-07	1.39E-06	2.48E-07
**36h**	4.51E-08	7.38E-07	1.55E-07	8.66E-08
**48h**	1.93E-06	1.16E-06	1.01E-06	2.81E-08
**72h**	2.23E-08	5.44E-08	1.59E-08	2.17E-08
	cgd3_2820			
**2h**	1.05E-08	1.16E-08	3.51E-09	8.45E-09
**6h**	3.45E-09	1.85E-09	5.72E-09	1.65E-09
**12h**	3.39E-08	8.18E-09	1.14E-08	6.39E-10
**24h**	1.66E-09	7.09E-10	4.12E-09	2.87E-09
**36h**	6.09E-09	1.63E-08	1.38E-09	2.33E-09
**48h**	1.06E-08	3.75E-09	1.58E-09	1.39E-09
**72h**	5.93E-09	2.10E-09	1.87E-09	1.83E-09
	cgd3_2850			
**2h**	1.26E-07	7.35E-08	4.00E-09	2.13E-07
**6h**	5.35E-09	6.61E-10	5.78E-09	4.31E-09
**12h**	7.35E-09	9.21E-09	2.42E-08	5.09E-09
**24h**	2.01E-09	2.46E-09	1.97E-09	6.50E-09
**36h**	1.09E-08	1.48E-08	1.82E-09	9.23E-09
**48h**	6.09E-08	4.98E-08	1.34E-08	1.34E-08
**72h**	3.11E-08	6.31E-08	3.39E-09	2.80E-08
	cgd3_2860			

**2h**	1.18E-08	0.00E+00	2.50E-09	0.00E+00
**6h**	5.24E-10	2.14E-09	9.36E-10	5.62E-10
**12h**	3.92E-09	1.11E-08	1.38E-08	0.00E+00
**24h**	9.69E-10	6.92E-10	1.80E-09	2.29E-10
**36h**	2.06E-09	9.51E-09	1.67E-09	4.69E-10
**48h**	2.03E-07	7.15E-08	6.96E-08	4.41E-08
**72h**	1.02E-08	8.79E-09	4.16E-08	2.07E-08
	cgd3_2890			
**2h**	1.61E-08	1.47E-08	1.93E-07	4.48E-08
**6h**	5.10E-10	2.28E-09	5.61E-08	0.00E+00
**12h**	1.31E-09	1.16E-08	1.00E-07	0.00E+00
**24h**	5.50E-10	5.16E-09	6.15E-08	3.29E-09
**36h**	1.60E-09	3.08E-07	4.60E-09	1.83E-09
**48h**	3.83E-07	1.12E-07	8.91E-08	4.38E-09
**72h**	5.10E-09	1.54E-08	1.23E-08	1.18E-08
	cgd3_2900			
**2h**	1.36E-08	0.00E+00	1.53E-09	0.00E+00
**6h**	8.61E-09	2.75E-10	6.77E-09	1.38E-08
**12h**	2.57E-09	1.31E-09	7.10E-10	0.00E+00
**24h**	6.11E-09	1.01E-10	1.31E-08	1.35E-08
**36h**	1.35E-08	3.95E-08	4.29E-09	1.65E-08
**48h**	2.98E-08	1.15E-08	3.83E-09	8.68E-09
**72h**	6.04E-09	1.69E-08	4.31E-09	2.98E-08
	cgd3_2910			
**2h**	0.00E+00	4.66E-09	1.58E-08	5.37E-09
**6h**	1.26E-07	5.68E-08	8.39E-08	8.91E-08
**12h**	0.00E+00	5.66E-09	1.59E-09	8.37E-09
**24h**	6.43E-08	3.60E-08	2.92E-08	6.60E-08
**36h**	2.81E-08	1.64E-08	2.57E-08	2.83E-08
**48h**	1.69E-08	3.67E-08	2.31E-08	4.08E-08
**72h**	1.72E-08	6.98E-09	7.52E-09	1.16E-08
	cgd3_2930			
**2h**	6.65E-09	1.03E-07	3.48E-08	7.47E-08
**6h**	6.66E-08	1.07E-08	1.15E-08	6.99E-08
**12h**	3.50E-08	5.48E-09	2.47E-08	1.55E-08
**24h**	2.36E-08	8.34E-09	1.24E-08	2.83E-08
**36h**	3.44E-08	7.42E-09	7.95E-08	2.10E-08
**48h**	5.71E-09	4.02E-08	3.37E-08	2.29E-08
**72h**	3.88E-08	1.78E-08	1.64E-08	2.37E-08
	cgd3_2940			
**2h**	2.02E-08	1.42E-09	2.36E-08	9.09E-08
**6h**	4.64E-09	6.53E-09	1.22E-08	3.83E-08
**12h**	0.00E+00	9.98E-09	3.77E-08	6.44E-08
**24h**	1.33E-08	3.36E-09	5.85E-09	3.96E-08
**36h**	1.08E-08	1.63E-09	5.17E-08	5.26E-09
**48h**	7.54E-09	2.89E-08	1.85E-08	1.96E-08
**72h**	2.97E-08	1.29E-08	1.67E-08	3.32E-08
	cgd3_2980			
**2h**	0.00E+00	0.00E+00	0.00E+00	1.27E-08
**6h**	2.27E-09	5.43E-08	0.00E+00	8.90E-08
**12h**	0.00E+00	0.00E+00	6.33E-08	1.18E-09
**24h**	1.15E-09	3.60E-09	5.51E-08	1.05E-07
**36h**	6.37E-08	1.01E-08	2.23E-07	3.29E-08

**48h**	1.57E-08	3.74E-07	1.95E-07	1.52E-07
**72h**	2.95E-07	5.72E-08	3.84E-08	1.34E-07
	cgd3_2990			
**2h**	0.00E+00	9.55E-09	0.00E+00	9.92E-09
**6h**	4.11E-09	1.88E-08	1.58E-09	9.94E-09
**12h**	0.00E+00	0.00E+00	1.25E-10	9.46E-09
**24h**	1.84E-09	1.89E-09	3.15E-09	2.17E-08
**36h**	1.69E-09	3.39E-09	3.27E-08	5.66E-09
**48h**	3.54E-09	3.93E-08	2.41E-08	3.60E-08
**72h**	2.63E-08	2.51E-08	1.95E-08	7.43E-09
	cgd3_3010			
**2h**	0.00E+00	1.45E-09	3.25E-08	1.39E-07
**6h**	2.53E-07	2.30E-07	1.70E-07	1.35E-06
**12h**	1.62E-07	2.30E-07	1.08E-06	8.65E-06
**24h**	4.70E-07	2.23E-07	2.12E-07	5.68E-06
**36h**	3.20E-07	2.01E-07	3.49E-06	6.41E-07
**48h**	1.05E-07	3.09E-06	2.06E-06	8.13E-06
**72h**	4.87E-07	3.26E-07	1.01E-07	5.53E-08
	cgd3_3020			
**2h**	0.00E+00	4.54E-07	1.32E-06	0.00E+00
**6h**	8.98E-05	2.56E-06	1.28E-04	4.64E-04
**12h**	0.00E+00	1.08E-05	3.54E-05	6.15E-05
**24h**	2.62E-05	8.67E-06	7.21E-05	1.40E-04
**36h**	7.47E-05	4.04E-06	1.37E-04	6.42E-05
**48h**	2.77E-05	2.59E-04	2.79E-04	3.07E-04
**72h**	5.54E-05	1.62E-05	2.15E-05	6.12E-05
	cgd3_3030			
**2h**	0.00E+00	0.00E+00	0.00E+00	1.44E-08
**6h**	3.76E-09	1.00E-09	3.19E-09	1.33E-08
**12h**	5.42E-08	2.23E-08	4.17E-08	9.32E-08
**24h**	5.57E-09	4.58E-09	5.65E-09	3.91E-08
**36h**	2.92E-08	1.93E-08	4.58E-07	6.68E-08
**48h**	9.71E-09	1.11E-07	1.03E-07	1.93E-07
**72h**	1.07E-07	3.89E-08	8.36E-08	1.05E-07
	cgd3_3040			
**2h**	0.00E+00	0.00E+00	0.00E+00	1.18E-09
**6h**	6.24E-09	2.56E-09	9.12E-10	3.02E-09
**12h**	5.37E-07	8.01E-07	8.45E-07	0.00E+00
**24h**	9.42E-08	2.34E-08	5.81E-08	1.26E-07
**36h**	1.45E-07	1.45E-07	2.07E-06	1.78E-07
**48h**	1.27E-07	3.46E-07	1.61E-07	1.48E-07
**72h**	6.77E-08	2.84E-08	2.01E-08	5.09E-08
	cgd3_3080			
**2h**	0.00E+00	5.40E-09	8.97E-09	9.98E-09
**6h**	1.05E-08	4.21E-10	7.33E-09	4.60E-09
**12h**	0.00E+00	0.00E+00	4.15E-10	1.49E-09
**24h**	4.19E-09	5.64E-10	1.47E-09	1.18E-08
**36h**	2.77E-09	1.86E-09	2.22E-08	1.62E-09
**48h**	3.00E-09	3.07E-08	8.24E-09	1.54E-08
**72h**	4.95E-09	4.54E-09	7.78E-09	8.33E-09
	cgd3_3100			
**2h**	0.00E+00	0.00E+00	0.00E+00	7.48E-10
**6h**	0.00E+00	0.00E+00	0.00E+00	4.24E-10

**12h**	5.93E-08	8.70E-08	3.93E-07	1.32E-07
**24h**	5.50E-09	1.79E-09	1.26E-08	1.41E-08
**36h**	1.17E-08	1.76E-08	3.08E-07	1.48E-08
**48h**	3.06E-08	1.51E-07	1.09E-07	5.13E-08
**72h**	2.33E-08	2.27E-08	2.36E-08	8.62E-09
	cgd3_3110			
**2h**	0.00E+00	0.00E+00	3.72E-08	3.37E-09
**6h**	7.48E-08	2.04E-07	9.39E-08	3.63E-07
**12h**	1.84E-07	3.57E-08	3.85E-07	1.24E-07
**24h**	1.56E-07	1.21E-07	1.30E-07	9.39E-07
**36h**	2.33E-07	1.19E-07	1.05E-06	4.69E-08
**48h**	2.90E-07	5.99E-07	9.85E-07	1.23E-06
**72h**	1.18E-06	9.71E-07	9.22E-07	2.53E-07
	cgd3_3350			
**2h**	1.28E-06	1.19E-05	3.11E-06	1.02E-05
**6h**	6.41E-07	3.21E-07	1.94E-06	1.91E-06
**12h**	3.46E-07	8.09E-07	9.49E-07	1.87E-06
**24h**	3.55E-07	5.07E-07	1.93E-06	1.04E-06
**36h**	1.71E-06	5.55E-06	2.87E-07	3.59E-06
**48h**	4.13E-06	4.51E-06	1.43E-06	1.33E-06
**72h**	1.39E-06	1.31E-06	4.44E-07	2.86E-06
	cgd3_3360			
**2h**	1.30E-08	6.48E-07	1.06E-06	1.03E-07
**6h**	2.73E-08	8.66E-08	2.82E-07	6.32E-08
**12h**	1.73E-08	1.40E-07	2.90E-07	3.03E-08
**24h**	1.16E-08	1.35E-07	2.91E-07	6.40E-08
**36h**	4.03E-08	4.39E-07	2.27E-08	5.41E-08
**48h**	7.23E-07	2.31E-07	2.59E-07	4.63E-08
**72h**	9.27E-08	1.44E-07	8.06E-08	2.18E-07
	cgd3_3370			
**2h**	0.00E+00	0.00E+00	0.00E+00	0.00E+00
**6h**	0.00E+00	0.00E+00	0.00E+00	0.00E+00
**12h**	2.22E-04	3.94E-04	9.90E-04	3.38E-05
**24h**	4.86E-06	1.58E-05	3.89E-05	4.50E-06
**36h**	3.56E-05	5.65E-04	1.15E-05	1.97E-05
**48h**	2.16E-04	5.52E-05	5.05E-05	1.43E-05
**72h**	6.20E-06	9.30E-06	1.16E-05	1.12E-05
	cgd3_3380			
**2h**	1.00E-07	2.35E-07	5.45E-07	4.18E-08
**6h**	2.78E-09	1.35E-08	1.12E-08	1.03E-08
**12h**	1.29E-07	1.91E-07	9.34E-07	2.11E-07
**24h**	3.01E-08	3.10E-08	1.41E-07	2.17E-08
**36h**	4.84E-08	3.14E-07	2.54E-08	2.63E-08
**48h**	3.08E-07	6.38E-08	8.99E-08	4.86E-08
**72h**	3.16E-08	3.28E-08	2.24E-08	8.21E-08
	cgd3_3390			
**2h**	0.00E+00	3.62E-09	0.00E+00	1.26E-03
**6h**	2.17E-09	2.72E-08	2.93E-08	0.00E+00
**12h**	0.00E+00	4.96E-09	8.80E-09	1.53E-05
**24h**	5.45E-09	9.27E-09	1.81E-08	7.27E-04
**36h**	1.14E-08	7.35E-08	6.37E-09	0.00E+00
**48h**	9.52E-08	2.57E-08	4.59E-09	1.37E-04
**72h**	1.48E-08	1.63E-08	1.75E-08	1.21E-04

	cgd3_3400			
**2h**	0.00E+00	0.00E+00	0.00E+00	0.00E+00
**6h**	3.25E-07	7.00E-08	9.81E-09	1.84E-07
**12h**	1.09E-08	1.36E-08	2.58E-08	6.90E-08
**24h**	3.25E-07	9.23E-08	5.58E-08	1.53E-07
**36h**	1.48E-07	2.09E-07	3.74E-08	9.43E-08
**48h**	1.02E-06	9.50E-07	0.00E+00	1.10E-06
**72h**	9.79E-08	2.32E-07	2.57E-08	5.83E-07
	cgd3_3410			
**2h**	0.00E+00	0.00E+00	5.73E-09	0.00E+00
**6h**	7.49E-08	1.14E-07	5.23E-07	1.53E-07
**12h**	3.25E-09	8.35E-09	4.39E-08	5.24E-09
**24h**	4.23E-08	5.87E-08	5.58E-07	3.11E-08
**36h**	2.89E-08	1.45E-06	2.53E-08	1.55E-08
**48h**	8.50E-07	2.64E-07	2.81E-07	3.00E-08
**72h**	1.51E-08	1.50E-08	7.76E-09	2.20E-08
	cgd3_3420			
**2h**	0.00E+00	2.13E-09	2.99E-09	0.00E+00
**6h**	2.85E-08	1.06E-08	7.73E-08	6.79E-08
**12h**	2.49E-09	3.63E-09	1.32E-08	3.88E-09
**24h**	3.19E-08	1.04E-08	1.64E-07	1.89E-08
**36h**	4.24E-08	2.53E-07	6.44E-09	2.68E-08
**48h**	2.03E-07	1.19E-07	4.73E-08	2.38E-08
**72h**	1.36E-08	2.68E-08	8.39E-09	4.90E-08
	cgd3_3440			
**2h**	1.23E-08	7.11E-08	3.41E-07	0.00E+00
**6h**	2.14E-09	2.30E-08	3.83E-08	2.61E-08
**12h**	2.20E-09	1.69E-08	6.51E-08	1.73E-08
**24h**	9.50E-09	8.69E-09	6.95E-08	1.85E-08
**36h**	5.44E-07	1.40E-07	1.14E-08	1.65E-08
**48h**	1.78E-07	3.88E-08	1.87E-08	4.10E-09
**72h**	2.37E-08	2.00E-08	1.50E-08	1.83E-08
	cgd3_3470			
**2h**	4.32E-07	0.00E+00	0.00E+00	0.00E+00
**6h**	1.61E-06	5.21E-07	2.87E-06	1.40E-05
**12h**	6.27E-07	5.38E-07	2.64E-06	0.00E+00
**24h**	1.05E-05	1.07E-06	2.63E-06	3.70E-06
**36h**	1.27E-05	3.58E-06	3.81E-06	6.50E-06
**48h**	5.64E-05	8.85E-06	4.51E-06	8.33E-06
**72h**	1.00E-05	2.56E-06	8.96E-07	3.41E-06
	cgd3_3480			
**2h**	0.00E+00	0.00E+00	0.00E+00	0.00E+00
**6h**	0.00E+00	0.00E+00	7.00E-10	1.36E-08
**12h**	3.92E-09	6.29E-09	1.32E-08	8.39E-09
**24h**	4.91E-09	1.35E-09	5.44E-09	2.85E-10
**36h**	2.64E-08	1.20E-07	1.01E-08	1.45E-08
**48h**	2.28E-07	4.26E-08	2.93E-08	6.98E-09
**72h**	1.32E-08	2.21E-08	1.60E-08	2.27E-08
	cgd3_3540			
**2h**	0.00E+00	1.04E-07	1.99E-07	0.00E+00
**6h**	5.14E-10	3.26E-09	2.41E-08	1.92E-09
**12h**	1.09E-09	1.19E-08	7.43E-08	0.00E+00
**24h**	3.65E-09	4.12E-09	5.11E-08	5.88E-11

**36h**	1.15E-08	1.24E-07	2.79E-09	2.35E-10
**48h**	1.70E-07	1.35E-08	2.39E-08	2.64E-10
**72h**	7.25E-08	2.01E-07	9.05E-08	0.00E+00
	cgd1_3100			
**2h**	8.03E-04	6.69E-05	1.36E-03	1.35E-04
**6h**	3.16E-04	5.93E-05	8.71E-04	9.59E-05
**12h**	7.44E-05	1.16E-04	3.84E-04	2.89E-06
**24h**	5.23E-05	1.41E-04	1.19E-04	1.00E-05
**36h**	9.30E-05	3.13E-05	1.96E-04	1.54E-05
**48h**	1.39E-04	6.74E-05	6.73E-05	8.70E-05
**72h**	9.66E-05	7.86E-05	5.70E-06	5.82E-05
	cgd1_3110			
**2h**	7.40E-08	0.00E+00	0.00E+00	0.00E+00
**6h**	1.99E-08	0.00E+00	1.46E-07	7.45E-08
**12h**	3.71E-08	8.69E-08	4.53E-07	0.00E+00
**24h**	3.07E-07	3.69E-09	1.13E-06	2.15E-08
**36h**	4.47E-06	8.25E-07	1.90E-06	8.07E-07
**48h**	1.77E-06	3.49E-06	1.81E-05	3.16E-05
**72h**	5.81E-06	5.93E-06	1.99E-05	1.01E-05
	cgd1_3120			
**2h**	0.00E+00	0.00E+00	0.00E+00	0.00E+00
**6h**	0.00E+00	0.00E+00	0.00E+00	0.00E+00
**12h**	1.39E-11	1.39E-10	1.58E-09	0.00E+00
**24h**	3.60E-11	0.00E+00	2.12E-09	0.00E+00
**36h**	9.13E-10	4.74E-10	5.83E-10	1.68E-10
**48h**	3.78E-08	7.87E-08	3.30E-07	2.36E-07
**72h**	2.83E-07	1.06E-07	1.25E-06	2.76E-06
	cgd1_3140			
**2h**	1.98E-03	7.94E-03	2.10E-02	9.46E-05
**6h**	2.03E-03	8.61E-04	2.07E-03	3.66E-05
**12h**	2.33E-03	2.56E-03	6.21E-03	1.67E-04
**24h**	6.35E-04	6.05E-04	1.29E-03	1.07E-04
**36h**	7.93E-04	2.37E-04	2.16E-03	2.31E-04
**48h**	7.81E-04	8.63E-04	1.99E-03	3.25E-04
**72h**	2.13E-04	1.15E-06	1.74E-03	7.23E-05
	cgd1_3150			
**2h**	0.00E+00	0.00E+00	0.00E+00	0.00E+00
**6h**	0.00E+00	0.00E+00	0.00E+00	0.00E+00
**12h**	0.00E+00	0.00E+00	1.21E-08	1.30E-09
**24h**	3.65E-09	3.38E-09	0.00E+00	4.53E-10
**36h**	1.30E-08	6.47E-08	2.76E-09	1.36E-09
**48h**	3.24E-07	1.56E-07	1.10E-07	5.33E-08
**72h**	1.10E-07	8.23E-07	4.60E-07	1.32E-07
	cgd1_3160			
**2h**	1.07E-05	2.85E-04	1.68E-05	1.23E-04
**6h**	2.33E-05	7.01E-04	7.69E-05	6.23E-04
**12h**	6.86E-06	6.17E-04	2.28E-05	1.40E-04
**24h**	3.30E-05	1.28E-04	7.41E-05	1.00E-03
**36h**	5.70E-06	7.12E-06	1.20E-05	1.77E-04
**48h**	3.38E-05	1.48E-04	4.93E-05	2.73E-04
**72h**	1.02E-05	1.25E-04	1.48E-05	1.49E-04
	cgd1_3180			
**2h**	0.00E+00	1.07E-06	1.69E-07	0.00E+00

**6h**	0.00E+00	0.00E+00	0.00E+00	0.00E+00
**12h**	1.08E-06	1.93E-05	6.64E-06	9.29E-07
**24h**	1.13E-06	4.24E-06	1.50E-06	6.86E-07
**36h**	2.90E-06	1.09E-04	6.77E-06	1.52E-06
**48h**	6.84E-06	9.73E-05	4.42E-05	5.45E-06
**72h**	3.70E-06	4.38E-05	1.47E-05	3.88E-06
	cgd1_3190			
**2h**	2.15E-07	9.12E-08	2.16E-08	2.16E-07
**6h**	4.78E-07	5.45E-07	3.14E-06	2.84E-06
**12h**	2.94E-08	1.23E-08	9.30E-07	1.61E-07
**24h**	3.35E-07	1.97E-07	5.59E-06	4.43E-07
**36h**	1.03E-06	1.59E-06	1.61E-06	2.40E-07
**48h**	1.75E-07	6.24E-07	1.06E-06	2.09E-07
**72h**	2.76E-07	4.85E-07	1.27E-06	1.98E-07
	cgd1_3200			
**2h**	7.83E-08	6.76E-07	6.13E-08	3.59E-07
**6h**	7.95E-08	7.30E-07	1.24E-06	3.73E-07
**12h**	8.14E-08	3.81E-08	5.72E-07	1.23E-07
**24h**	4.94E-07	4.01E-07	1.05E-06	1.40E-07
**36h**	3.95E-07	9.26E-07	1.00E-05	1.76E-07
**48h**	1.28E-06	4.87E-07	7.22E-07	2.23E-07
**72h**	5.59E-08	1.80E-07	4.80E-08	4.13E-08
	cgd1_3210			
**2h**	0.00E+00	0.00E+00	0.00E+00	0.00E+00
**6h**	0.00E+00	0.00E+00	0.00E+00	0.00E+00
**12h**	6.96E-07	5.82E-07	5.43E-06	1.24E-06
**24h**	3.09E-06	3.73E-06	3.54E-06	4.29E-07
**36h**	2.04E-06	4.01E-06	4.70E-06	2.01E-06
**48h**	4.69E-06	3.40E-06	4.39E-06	8.05E-07
**72h**	8.02E-07	1.47E-06	6.30E-07	1.17E-06
	cgd1_3220			
**2h**	0.00E+00	0.00E+00	0.00E+00	0.00E+00
**6h**	0.00E+00	0.00E+00	0.00E+00	0.00E+00
**12h**	2.14E-06	4.01E-06	1.01E-04	2.96E-06
**24h**	3.33E-06	1.03E-05	6.01E-05	1.58E-06
**36h**	1.96E-06	1.87E-05	7.42E-05	4.56E-06
**48h**	8.83E-06	1.28E-05	6.62E-05	2.05E-06
**72h**	1.59E-06	2.31E-05	2.78E-06	1.27E-06
	cgd1_3230			
**2h**	3.01E-08	0.00E+00	0.00E+00	0.00E+00
**6h**	1.16E-08	0.00E+00	4.48E-09	1.25E-08
**12h**	8.39E-08	7.21E-07	2.92E-06	4.65E-07
**24h**	1.48E-07	7.15E-07	7.48E-07	5.58E-08
**36h**	3.72E-07	1.43E-06	2.27E-06	6.21E-07
**48h**	5.31E-07	1.25E-06	2.10E-06	1.80E-07
**72h**	8.95E-07	1.53E-06	1.39E-06	8.38E-07
	cgd1_3240			
**2h**	3.94E-04	7.42E-05	1.80E-05	9.57E-04
**6h**	1.19E-02	1.33E-04	1.66E-04	3.47E-03
**12h**	6.82E-04	3.39E-05	9.73E-05	1.27E-03
**24h**	1.05E-03	7.80E-05	4.67E-04	1.14E-03
**36h**	1.35E-03	7.82E-05	1.41E-04	6.73E-04
**48h**	2.72E-03	5.68E-05	2.17E-04	6.75E-04

**72h**	1.72E-03	9.13E-05	5.64E-05	2.25E-03
	cgd1_3250			
**2h**	1.93E-02	1.30E-02	1.73E-03	1.71E-02
**6h**	9.20E-03	6.55E-03	3.81E-03	6.78E-03
**12h**	8.30E-03	2.27E-03	1.97E-03	1.60E-02
**24h**	8.08E-03	3.31E-03	1.53E-03	2.85E-03
**36h**	7.38E-03	3.78E-03	1.01E-03	2.85E-03
**48h**	4.34E-03	2.10E-03	5.47E-04	1.99E-03
**72h**	3.98E-03	1.78E-03	6.93E-05	9.23E-04
	cgd1_3260			
**2h**	1.87E-05	1.84E-05	0.00E+00	2.04E-05
**6h**	7.57E-06	1.41E-05	1.58E-05	4.02E-06
**12h**	1.31E-05	1.13E-05	8.35E-05	1.15E-05
**24h**	6.65E-06	4.77E-05	5.86E-05	4.22E-06
**36h**	6.78E-06	3.27E-05	3.05E-05	5.50E-06
**48h**	5.11E-06	1.16E-05	2.09E-05	2.98E-06
**72h**	3.53E-06	4.38E-06	2.00E-06	1.20E-06
	cgd1_3290			
**2h**	5.35E-06	1.06E-05	7.08E-06	3.65E-05
**6h**	5.93E-06	8.68E-06	4.62E-06	4.60E-06
**12h**	5.82E-07	3.89E-07	1.70E-06	3.02E-06
**24h**	2.10E-06	1.66E-06	5.44E-06	3.94E-06
**36h**	3.24E-06	4.68E-06	1.62E-06	1.36E-06
**48h**	8.55E-06	1.73E-06	3.20E-06	5.31E-06
**72h**	3.37E-06	1.38E-06	3.00E-07	3.38E-07
	cgd3_3560			
**2h**	2.78E-08	1.49E-09	6.82E-09	2.07E-07
**6h**	1.70E-09	0.00E+00	0.00E+00	0.00E+00
**12h**	3.77E-08	2.64E-09	5.31E-10	4.36E-08
**24h**	2.83E-09	1.57E-09	7.81E-09	7.56E-09
**36h**	3.51E-08	1.27E-07	4.74E-09	3.46E-07
**48h**	3.39E-07	2.11E-07	9.63E-08	5.34E-07
**72h**	3.10E-07	2.02E-07	7.83E-08	7.26E-07
	cgd3_3570			
**2h**	1.52E-04	0.00E+00	1.67E-05	6.01E-03
**6h**	1.89E-04	2.06E-05	3.06E-05	3.30E-03
**12h**	9.22E-05	3.82E-06	1.68E-05	9.89E-04
**24h**	1.69E-05	1.60E-05	2.42E-05	1.91E-03
**36h**	4.86E-05	4.67E-05	1.15E-05	1.32E-03
**48h**	2.04E-05	2.53E-05	4.84E-05	4.04E-04
**72h**	1.63E-04	4.81E-05	6.52E-06	2.08E-03
	cgd3_3580			
**2h**	5.58E-08	7.71E-09	6.06E-08	1.27E-07
**6h**	9.51E-09	1.07E-08	5.01E-09	2.46E-07
**12h**	7.47E-08	2.70E-08	7.98E-08	4.64E-07
**24h**	3.22E-08	5.42E-08	9.11E-08	2.73E-07
**36h**	1.92E-08	1.12E-07	6.75E-09	3.42E-07
**48h**	1.22E-07	4.68E-08	4.66E-08	2.50E-07
**72h**	3.02E-08	1.05E-07	1.99E-08	3.92E-07
	cgd3_3590			
**2h**	0.00E+00	0.00E+00	0.00E+00	0.00E+00
**6h**	1.06E-09	2.38E-10	4.20E-09	1.55E-08
**12h**	0.00E+00	2.38E-09	7.50E-10	0.00E+00

**24h**	4.25E-09	2.73E-09	6.35E-09	2.12E-08
**36h**	2.34E-08	1.62E-08	1.03E-08	2.60E-08
**48h**	3.24E-08	1.99E-08	1.24E-08	3.81E-08
**72h**	8.39E-08	6.33E-08	1.64E-08	1.24E-07
	cgd3_3600			
**2h**	0.00E+00	0.00E+00	0.00E+00	0.00E+00
**6h**	4.38E-10	0.00E+00	1.58E-07	4.63E-07
**12h**	2.30E-07	9.06E-08	5.48E-08	0.00E+00
**24h**	3.24E-08	2.31E-08	9.03E-08	4.11E-07
**36h**	2.65E-07	1.30E-07	7.86E-08	1.24E-06
**48h**	1.59E-06	7.03E-07	3.46E-07	2.04E-06
**72h**	2.51E-07	7.96E-07	3.68E-07	1.11E-06
	cgd3_3610			
**2h**	0.00E+00	0.00E+00	0.00E+00	0.00E+00
**6h**	1.94E-09	0.00E+00	4.96E-09	2.67E-07
**12h**	3.99E-09	3.75E-09	5.22E-09	0.00E+00
**24h**	7.15E-09	1.62E-08	1.58E-08	3.67E-08
**36h**	1.52E-08	1.81E-07	1.71E-08	4.02E-07
**48h**	7.16E-08	3.72E-06	3.99E-08	1.12E-07
**72h**	1.38E-08	4.52E-07	5.02E-09	1.05E-07
	cgd3_3680			
**2h**	4.50E-08	2.31E-08	1.35E-07	8.16E-07
**6h**	2.95E-08	1.39E-08	1.34E-07	3.73E-07
**12h**	5.39E-08	5.52E-08	2.55E-07	1.61E-06
**24h**	1.41E-08	4.74E-08	1.57E-07	1.27E-07
**36h**	2.05E-08	1.22E-07	6.30E-09	3.55E-07
**48h**	3.76E-07	7.53E-08	6.05E-08	3.99E-07
**72h**	4.38E-08	3.27E-08	1.07E-08	2.22E-07
	cgd3_3700			
**2h**	0.00E+00	0.00E+00	3.28E-06	0.00E+00
**6h**	0.00E+00	2.00E-09	0.00E+00	0.00E+00
**12h**	0.00E+00	1.63E-09	1.82E-07	0.00E+00
**24h**	2.49E-09	6.83E-09	0.00E+00	0.00E+00
**36h**	0.00E+00	2.96E-08	2.44E-08	1.42E-06
**48h**	7.70E-06	3.08E-08	1.44E-06	7.96E-07
**72h**	6.63E-08	1.09E-08	2.24E-06	1.35E-06
	cgd3_3710			
**2h**	0.00E+00	0.00E+00	0.00E+00	0.00E+00
**6h**	5.63E-10	2.71E-09	1.28E-07	3.35E-07
**12h**	9.35E-09	1.14E-09	5.13E-09	7.66E-08
**24h**	9.76E-09	3.48E-08	5.54E-08	2.40E-07
**36h**	1.84E-08	9.04E-08	2.28E-08	9.95E-07
**48h**	1.75E-07	7.86E-08	7.04E-08	4.52E-07
**72h**	4.77E-09	2.98E-08	1.73E-08	4.89E-07
	cgd3_3740			
**2h**	0.00E+00	0.00E+00	4.28E-09	0.00E+00
**6h**	0.00E+00	0.00E+00	0.00E+00	0.00E+00
**12h**	0.00E+00	0.00E+00	4.69E-10	0.00E+00
**24h**	3.26E-10	0.00E+00	7.20E-10	0.00E+00
**36h**	1.18E-09	5.79E-09	0.00E+00	5.29E-09
**48h**	6.14E-07	2.80E-07	2.46E-07	5.21E-07
**72h**	9.13E-08	1.48E-06	6.71E-07	1.51E-06
	cgd3_3770			

**2h**	1.89E-06	8.30E-07	2.11E-06	3.71E-04
**6h**	3.40E-07	1.03E-06	6.48E-06	3.30E-05
**12h**	1.94E-07	1.85E-07	5.90E-07	1.06E-05
**24h**	2.74E-07	1.18E-06	6.95E-06	3.13E-05
**36h**	7.41E-07	8.66E-06	5.13E-07	2.67E-05
**48h**	6.85E-06	1.43E-06	1.48E-06	9.07E-06
**72h**	6.71E-07	4.02E-07	2.86E-07	6.23E-06
	cgd3_3800			
**2h**	4.06E-09	0.00E+00	5.50E-09	0.00E+00
**6h**	3.12E-08	1.29E-09	1.62E-08	0.00E+00
**12h**	1.91E-09	3.59E-08	5.00E-09	2.41E-07
**24h**	1.54E-08	8.96E-09	4.91E-08	9.62E-08
**36h**	3.35E-08	7.99E-08	3.53E-09	1.65E-07
**48h**	9.42E-08	1.67E-08	8.42E-09	3.63E-08
**72h**	0.00E+00	1.28E-07	5.11E-08	1.52E-06
	cgd3_3840			
**2h**	2.12E-06	1.93E-04	2.29E-04	8.43E-03
**6h**	1.76E-04	6.94E-04	1.60E-03	3.01E-03
**12h**	3.98E-05	1.52E-04	4.45E-04	9.59E-04
**24h**	8.62E-04	2.97E-04	7.63E-04	1.96E-04
**36h**	5.04E-04	6.59E-04	4.59E-04	6.70E-05
**48h**	2.76E-03	1.79E-04	2.57E-04	4.40E-05
**72h**	5.90E-05	1.39E-04	3.24E-04	1.13E-04
	cgd3_3860			
**2h**	0.00E+00	1.20E-09	0.00E+00	0.00E+00
**6h**	6.85E-10	0.00E+00	1.49E-09	7.33E-09
**12h**	6.54E-10	2.09E-10	1.89E-09	3.39E-09
**24h**	4.34E-10	1.91E-09	2.79E-09	1.50E-08
**36h**	8.08E-09	1.23E-07	2.17E-08	6.75E-08
**48h**	1.47E-07	2.95E-08	2.98E-08	3.97E-08
**72h**	9.70E-09	9.78E-08	4.99E-08	5.59E-08
	cgd3_3880			
**2h**	3.45E-10	7.77E-08	1.08E-07	1.45E-06
**6h**	2.93E-09	4.24E-09	2.23E-08	6.49E-08
**12h**	1.23E-08	5.98E-08	1.66E-07	1.05E-07
**24h**	3.66E-09	1.13E-08	3.87E-08	7.22E-08
**36h**	2.70E-09	2.69E-07	1.01E-08	1.35E-07
**48h**	1.01E-07	8.35E-08	3.20E-08	8.40E-08
**72h**	1.49E-08	6.00E-08	5.09E-08	1.44E-07
	cgd3_3890			
**2h**	3.02E-06	7.40E-06	7.49E-06	2.64E-05
**6h**	3.73E-05	9.44E-06	5.49E-06	1.52E-05
**12h**	1.02E-05	1.16E-05	1.88E-05	1.32E-05
**24h**	3.99E-05	8.01E-06	1.04E-05	5.38E-06
**36h**	7.78E-06	8.13E-06	5.13E-06	8.62E-06
**48h**	7.82E-06	3.79E-06	1.39E-06	2.08E-06
**72h**	4.26E-07	9.28E-07	3.42E-07	6.99E-07
	cgd3_3900			
**2h**	0.00E+00	0.00E+00	0.00E+00	0.00E+00
**6h**	0.00E+00	0.00E+00	0.00E+00	7.99E-10
**12h**	0.00E+00	1.65E-10	2.06E-08	1.29E-09
**24h**	1.08E-10	1.14E-09	8.88E-10	1.63E-08
**36h**	3.76E-09	8.30E-08	9.61E-11	1.02E-07

**48h**	7.23E-08	4.40E-08	6.52E-08	5.79E-08
**72h**	4.73E-08	3.11E-08	4.54E-08	2.36E-07
	cgd3_3910			
**2h**	0.00E+00	8.24E-09	3.68E-08	3.66E-07
**6h**	2.46E-09	4.32E-08	1.34E-07	6.83E-07
**12h**	7.19E-09	3.54E-08	3.24E-07	7.01E-08
**24h**	1.10E-08	5.20E-08	5.28E-07	5.85E-07
**36h**	1.89E-08	9.73E-07	7.49E-08	2.96E-06
**48h**	1.51E-06	1.73E-06	2.22E-06	1.14E-06
**72h**	3.93E-08	1.25E-07	2.53E-07	3.22E-07
	cgd3_3920			
**2h**	7.16E-07	2.40E-06	1.00E-05	4.68E-05
**6h**	5.49E-08	1.35E-07	9.84E-08	1.07E-07
**12h**	1.78E-07	2.59E-06	2.44E-06	6.66E-06
**24h**	1.06E-07	3.80E-07	6.71E-07	4.36E-07
**36h**	8.09E-08	8.03E-07	2.39E-07	5.70E-07
**48h**	1.18E-06	8.97E-07	7.03E-07	7.96E-07
**72h**	6.64E-08	2.27E-07	3.40E-07	4.76E-07
	cgd3_3930			
**2h**	6.71E-07	8.94E-07	1.57E-06	2.72E-06
**6h**	8.30E-06	5.44E-06	3.01E-06	5.50E-06
**12h**	2.20E-06	2.01E-06	2.70E-06	4.79E-06
**24h**	5.53E-06	4.99E-06	7.18E-06	4.20E-06
**36h**	2.17E-06	1.06E-06	2.39E-06	2.15E-06
**48h**	1.12E-06	1.57E-06	1.44E-06	1.78E-06
**72h**	3.16E-07	1.22E-07	2.03E-07	3.26E-07
	cgd3_3950			
**2h**	0.00E+00	0.00E+00	1.67E-08	5.77E-07
**6h**	1.69E-09	3.90E-08	3.59E-08	3.81E-08
**12h**	1.32E-08	2.97E-09	1.20E-09	3.36E-09
**24h**	4.80E-09	1.71E-08	3.85E-08	3.52E-08
**36h**	1.14E-08	3.19E-08	3.21E-08	3.79E-08
**48h**	5.46E-08	5.96E-08	4.85E-08	9.02E-08
**72h**	1.04E-08	9.24E-09	5.72E-09	3.14E-08
	cgd3_3960			
**2h**	0.00E+00	0.00E+00	0.00E+00	0.00E+00
**6h**	5.59E-10	0.00E+00	2.51E-08	4.93E-08
**12h**	6.97E-10	1.46E-10	7.84E-09	7.85E-10
**24h**	4.10E-09	5.51E-09	1.37E-08	4.99E-08
**36h**	4.80E-09	3.30E-08	8.67E-09	7.44E-08
**48h**	1.07E-07	3.91E-08	4.94E-08	1.13E-07
**72h**	3.91E-09	3.66E-08	2.21E-09	2.36E-08
	cgd3_3970			
**2h**	8.00E-08	1.41E-08	6.64E-08	6.99E-08
**6h**	1.63E-08	7.26E-09	9.09E-10	3.77E-08
**12h**	6.77E-08	4.11E-09	8.67E-09	2.43E-08
**24h**	8.35E-09	8.85E-09	3.13E-08	4.10E-08
**36h**	1.91E-08	3.99E-08	2.72E-08	5.79E-08
**48h**	1.06E-07	4.12E-08	6.39E-08	1.55E-07
**72h**	2.33E-08	4.45E-08	2.06E-08	3.10E-08
	cgd3_3980			
**2h**	0.00E+00	2.60E-08	2.79E-07	5.63E-06
**6h**	9.06E-10	2.56E-08	1.35E-06	9.74E-07

**12h**	4.12E-08	6.61E-08	4.86E-08	2.24E-07
**24h**	3.85E-08	5.90E-08	8.31E-07	1.45E-06
**36h**	4.17E-08	1.43E-05	2.82E-07	2.67E-06
**48h**	1.21E-06	1.61E-06	1.53E-06	1.96E-06
**72h**	2.41E-07	1.66E-06	5.81E-07	2.73E-06
	cgd3_4010			
**2h**	5.74E-08	6.34E-07	4.11E-07	3.41E-06
**6h**	2.08E-04	2.21E-06	2.20E-06	2.97E-05
**12h**	5.52E-06	1.12E-06	2.13E-06	1.55E-06
**24h**	1.07E-04	6.36E-06	3.96E-05	1.66E-05
**36h**	4.44E-05	1.72E-05	2.66E-06	1.34E-05
**48h**	1.02E-04	1.90E-05	3.26E-06	1.10E-05
**72h**	2.09E-05	1.15E-05	1.54E-06	1.09E-05
	cgd3_4020			
**2h**	2.00E-07	9.60E-08	2.75E-07	1.91E-07
**6h**	4.02E-06	6.12E-07	1.72E-07	5.06E-07
**12h**	1.44E-06	2.35E-07	1.68E-07	4.07E-07
**24h**	4.81E-06	1.76E-07	4.39E-07	1.18E-06
**36h**	1.09E-06	3.05E-07	7.58E-07	4.15E-07
**48h**	6.30E-07	1.10E-07	5.37E-08	1.72E-07
**72h**	2.52E-07	1.76E-07	3.81E-08	2.90E-07
	cgd3_4050			
**2h**	0.00E+00	0.00E+00	5.37E-08	1.45E-07
**6h**	0.00E+00	0.00E+00	5.52E-10	9.21E-09
**12h**	0.00E+00	1.13E-10	5.61E-09	1.06E-09
**24h**	2.73E-10	5.34E-10	4.44E-09	5.74E-08
**36h**	3.63E-10	2.19E-08	1.94E-09	1.02E-08
**48h**	4.11E-07	8.31E-08	5.35E-08	1.18E-07
**72h**	8.34E-07	1.05E-06	1.04E-06	1.90E-06
	cgd3_4060			
**2h**	1.55E-07	2.18E-08	1.12E-08	4.76E-06
**6h**	6.50E-08	1.60E-07	8.21E-07	6.70E-06
**12h**	1.58E-08	1.82E-07	9.03E-08	6.27E-07
**24h**	9.15E-07	8.92E-07	5.03E-06	1.69E-05
**36h**	1.04E-06	9.83E-06	7.43E-07	4.42E-06
**48h**	1.52E-05	6.66E-06	3.70E-06	3.48E-06
**72h**	2.35E-05	7.91E-06	1.62E-05	2.79E-05
	cgd3_4070			
**2h**	9.38E-08	1.44E-07	2.04E-07	1.60E-06
**6h**	1.73E-07	1.79E-07	9.88E-08	4.23E-07
**12h**	1.09E-07	1.47E-07	3.80E-07	3.31E-07
**24h**	1.73E-07	2.51E-07	2.90E-07	8.38E-07
**36h**	7.82E-08	3.56E-07	1.97E-07	1.54E-07
**48h**	5.31E-07	2.79E-07	8.19E-08	3.09E-07
**72h**	1.75E-08	4.94E-08	3.27E-08	7.30E-08
	cgd3_4090			
**2h**	0.00E+00	0.00E+00	0.00E+00	3.06E-07
**6h**	4.91E-08	4.55E-06	4.33E-06	1.65E-05
**12h**	2.44E-06	4.00E-08	1.79E-07	2.38E-07
**24h**	5.03E-07	2.14E-06	3.92E-06	2.09E-05
**36h**	3.57E-07	1.50E-05	2.29E-07	5.55E-06
**48h**	2.41E-05	7.05E-06	7.17E-06	1.16E-05
**72h**	1.46E-06	1.53E-06	1.13E-06	7.52E-06

	cgd3_4180			
**2h**	1.54E-07	2.77E-07	1.17E-06	5.49E-06
**6h**	8.18E-09	1.62E-08	6.61E-08	1.67E-07
**12h**	3.00E-07	4.36E-07	2.08E-06	9.39E-07
**24h**	2.29E-07	2.40E-07	6.13E-07	1.91E-06
**36h**	4.71E-08	8.11E-07	9.10E-08	3.78E-07
**48h**	1.01E-06	2.12E-07	2.43E-07	4.17E-07
**72h**	1.10E-07	1.24E-07	2.38E-08	1.26E-07
	cgd3_4200			
**2h**	0.00E+00	8.47E-10	0.00E+00	5.96E-09
**6h**	6.94E-10	2.77E-10	3.45E-10	6.95E-09
**12h**	0.00E+00	1.15E-09	5.47E-09	1.68E-09
**24h**	3.74E-09	1.16E-09	3.72E-09	1.32E-08
**36h**	1.55E-09	1.83E-08	8.69E-09	2.05E-08
**48h**	4.79E-07	2.11E-07	1.01E-07	2.20E-07
**72h**	1.42E-08	2.40E-08	8.74E-09	1.77E-08
	cgd3_4250			
**2h**	5.53E-08	1.92E-07	1.40E-06	2.26E-06
**6h**	1.36E-08	4.32E-08	3.35E-08	1.40E-07
**12h**	1.92E-07	2.38E-07	6.56E-07	3.32E-07
**24h**	5.81E-08	1.16E-07	4.94E-07	7.67E-07
**36h**	5.03E-08	5.61E-07	3.78E-08	1.31E-07
**48h**	7.48E-07	2.63E-07	1.67E-07	2.23E-07
**72h**	3.70E-08	2.56E-08	2.29E-08	2.37E-08
	cgd3_4270			
**2h**	1.67E-08	9.35E-08	4.91E-07	1.66E-06
**6h**	3.84E-09	1.66E-08	4.70E-08	5.40E-08
**12h**	2.81E-08	1.42E-07	2.95E-07	2.05E-07
**24h**	2.68E-08	4.04E-08	2.94E-07	1.58E-07
**36h**	9.17E-09	1.15E-07	1.26E-08	1.48E-08
**48h**	1.49E-07	7.11E-08	1.03E-07	6.19E-08
**72h**	4.90E-08	3.81E-08	2.06E-08	6.26E-08
	cgd3_4280			
**2h**	2.62E-06	8.27E-06	2.09E-05	1.15E-04
**6h**	2.39E-09	4.19E-08	2.51E-08	5.03E-08
**12h**	8.89E-07	7.17E-06	1.53E-05	4.84E-06
**24h**	1.16E-07	7.00E-07	4.46E-06	2.55E-06
**36h**	8.02E-08	3.13E-06	1.97E-07	9.68E-07
**48h**	1.55E-06	1.52E-06	1.18E-06	1.35E-06
**72h**	2.02E-07	6.19E-07	1.40E-06	9.90E-07
	cgd3_4290			
**2h**	4.90E-09	0.00E+00	1.39E-09	6.46E-08
**6h**	1.14E-10	3.95E-10	6.69E-09	2.52E-08
**12h**	0.00E+00	1.60E-09	1.72E-08	8.62E-09
**24h**	3.82E-10	2.84E-09	8.68E-09	3.36E-08
**36h**	1.06E-09	4.34E-08	1.21E-09	3.86E-08
**48h**	4.53E-08	2.40E-08	3.47E-08	2.22E-08
**72h**	3.00E-08	1.44E-08	9.17E-09	6.65E-08
	cgd3_4330			
**2h**	1.05E-09	1.95E-09	4.45E-09	1.23E-07
**6h**	5.13E-09	1.83E-08	9.33E-09	8.58E-08
**12h**	8.69E-09	3.49E-09	3.91E-08	2.95E-08
**24h**	7.87E-09	1.09E-08	2.61E-08	1.22E-07

**36h**	7.06E-09	3.38E-08	9.37E-09	4.58E-08
**48h**	9.64E-08	5.23E-08	2.11E-08	6.70E-08
**72h**	7.97E-09	1.39E-08	4.84E-08	1.32E-08
	cgd3_4340			
**2h**	6.50E-10	6.29E-09	0.00E+00	2.22E-09
**6h**	2.07E-09	1.96E-09	9.99E-09	1.10E-08
**12h**	1.70E-08	4.92E-09	1.60E-08	2.82E-09
**24h**	2.79E-08	2.39E-08	4.59E-08	2.29E-08
**36h**	4.91E-08	4.96E-08	8.95E-08	7.10E-08
**48h**	3.59E-07	7.22E-08	4.40E-08	1.27E-07
**72h**	1.23E-07	7.37E-08	3.33E-07	9.72E-08
	cgd3_4350			
**2h**	2.49E-09	0.00E+00	0.00E+00	0.00E+00
**6h**	2.87E-09	5.25E-09	1.49E-08	3.46E-08
**12h**	8.48E-10	4.27E-09	2.60E-08	3.00E-09
**24h**	3.89E-09	6.00E-09	1.95E-08	4.34E-08
**36h**	2.53E-09	1.50E-08	1.13E-08	7.16E-08
**48h**	1.75E-06	3.68E-07	1.10E-07	3.53E-07
**72h**	8.06E-08	7.32E-08	4.85E-09	1.08E-07
	cgd4_10			
**2h**	1.89E-07	0.00E+00	0.00E+00	1.20E-06
**6h**	6.00E-07	1.10E-07	0.00E+00	1.14E-06
**12h**	0.00E+00	0.00E+00	1.56E-07	4.32E-07
**24h**	8.30E-08	7.19E-07	1.58E-06	1.84E-06
**36h**	1.72E-07	5.75E-07	6.87E-07	9.77E-07
**48h**	2.91E-07	7.58E-08	2.86E-07	1.30E-07
**72h**	1.90E-08	0.00E+00	0.00E+00	1.50E-07
	cgd4_30			
**2h**	0.00E+00	2.16E-09	0.00E+00	1.18E-09
**6h**	7.51E-10	8.09E-10	1.41E-09	1.77E-10
**12h**	9.60E-10	5.25E-09	2.10E-08	1.73E-08
**24h**	6.85E-09	6.37E-09	1.84E-08	2.08E-08
**36h**	9.92E-09	5.26E-08	1.24E-08	8.08E-08
**48h**	1.12E-07	4.83E-08	3.52E-08	4.46E-08
**72h**	6.25E-09	1.32E-09	1.13E-08	9.93E-09
	cgd4_40			
**2h**	1.08E-09	0.00E+00	0.00E+00	0.00E+00
**6h**	0.00E+00	0.00E+00	0.00E+00	0.00E+00
**12h**	4.77E-06	1.05E-05	1.35E-05	3.54E-05
**24h**	1.21E-06	3.49E-06	4.52E-07	1.32E-06
**36h**	3.05E-07	7.73E-07	6.50E-07	9.42E-07
**48h**	6.40E-07	2.11E-06	1.46E-07	2.71E-07
**72h**	4.78E-07	8.61E-07	1.66E-07	2.76E-07
	cgd4_60			
**2h**	0.00E+00	0.00E+00	0.00E+00	0.00E+00
**6h**	0.00E+00	0.00E+00	0.00E+00	0.00E+00
**12h**	2.73E-09	3.85E-09	1.23E-09	9.86E-10
**24h**	2.43E-09	2.73E-10	3.13E-09	1.21E-09
**36h**	3.15E-09	5.27E-09	1.63E-09	1.35E-08
**48h**	1.53E-08	5.90E-09	2.58E-09	7.42E-09
**72h**	4.21E-08	1.63E-08	1.29E-08	3.77E-08
	cgd4_70			
**2h**	1.19E-06	3.68E-06	5.62E-05	6.59E-05

**6h**	1.10E-07	1.17E-06	7.08E-06	6.59E-06
**12h**	1.44E-08	5.69E-06	1.20E-05	4.14E-05
**24h**	2.35E-06	1.38E-06	1.18E-05	1.37E-05
**36h**	2.50E-06	3.20E-05	3.52E-06	6.43E-06
**48h**	1.46E-05	9.72E-07	3.75E-06	8.04E-06
**72h**	5.51E-07	1.18E-06	5.50E-07	4.94E-06
	cgd4_80			
**2h**	5.00E-09	4.37E-08	5.79E-08	7.26E-08
**6h**	0.00E+00	0.00E+00	0.00E+00	0.00E+00
**12h**	6.21E-08	1.33E-07	9.33E-07	2.71E-07
**24h**	9.19E-09	2.27E-08	5.67E-08	7.97E-08
**36h**	6.23E-08	4.91E-07	2.47E-08	4.80E-07
**48h**	4.74E-06	7.85E-08	6.04E-07	6.11E-08
**72h**	5.95E-08	4.01E-08	9.98E-09	9.16E-08
	cgd4_100			
**2h**	1.78E-07	3.01E-09	0.00E+00	4.59E-09
**6h**	6.09E-09	1.82E-09	1.31E-09	6.80E-09
**12h**	1.72E-09	1.04E-09	1.20E-08	1.89E-08
**24h**	1.54E-08	3.06E-09	4.08E-08	2.17E-08
**36h**	6.01E-08	4.52E-08	2.95E-08	5.14E-08
**48h**	2.14E-07	2.17E-08	6.13E-08	3.45E-08
**72h**	3.10E-08	1.64E-08	5.71E-08	6.49E-09
	cgd4_110			
**2h**	1.45E-05	1.58E-06	1.31E-04	1.61E-04
**6h**	0.00E+00	0.00E+00	1.38E-06	2.70E-06
**12h**	2.91E-06	2.68E-06	9.73E-06	1.44E-05
**24h**	1.17E-05	5.83E-07	3.86E-05	5.86E-05
**36h**	9.47E-06	1.80E-05	0.00E+00	6.36E-05
**48h**	6.06E-05	4.29E-06	3.23E-06	2.34E-05
**72h**	9.72E-06	4.67E-06	2.46E-06	1.70E-05
	cgd4_120			
**2h**	0.00E+00	4.89E-08	0.00E+00	0.00E+00
**6h**	4.30E-08	1.39E-07	5.21E-08	2.81E-07
**12h**	0.00E+00	1.22E-08	6.98E-09	1.75E-08
**24h**	1.66E-07	2.33E-08	3.27E-08	4.30E-08
**36h**	1.73E-07	1.02E-07	2.56E-08	4.81E-08
**48h**	5.09E-08	1.44E-08	1.65E-08	2.07E-08
**72h**	1.50E-08	6.15E-09	1.38E-08	3.04E-09
	cgd1_3300			
**2h**	1.59E-05	8.53E-06	2.50E-05	4.05E-07
**6h**	2.36E-05	4.25E-05	6.36E-05	3.01E-06
**12h**	3.05E-06	2.46E-05	5.41E-07	1.84E-07
**24h**	7.27E-06	3.85E-06	3.76E-04	9.52E-06
**36h**	6.76E-06	4.93E-06	1.12E-05	2.88E-06
**48h**	1.11E-05	1.20E-05	3.31E-05	1.59E-05
**72h**	2.47E-06	3.69E-06	4.03E-06	9.46E-06
	cgd1_3310			
**2h**	2.70E-08	3.48E-09	4.22E-08	4.16E-08
**6h**	3.55E-08	5.12E-08	4.38E-08	4.18E-08
**12h**	5.21E-09	8.76E-09	1.71E-10	0.00E+00
**24h**	3.55E-08	1.19E-08	5.09E-08	2.09E-08
**36h**	6.73E-08	2.50E-08	1.88E-08	3.10E-08
**48h**	2.09E-08	8.82E-09	8.28E-08	3.44E-08

**72h**	2.16E-08	7.93E-09	5.18E-09	1.52E-08
	cgd1_3320			
**2h**	1.35E-10	1.29E-09	2.03E-08	0.00E+00
**6h**	6.18E-08	1.00E-07	2.47E-07	8.06E-08
**12h**	2.45E-09	3.18E-09	4.17E-10	0.00E+00
**24h**	3.40E-08	1.93E-08	5.14E-08	2.83E-08
**36h**	4.59E-08	2.97E-08	6.19E-08	9.59E-08
**48h**	7.55E-08	4.34E-08	1.39E-07	7.02E-08
**72h**	3.45E-08	1.21E-08	2.64E-08	7.23E-08
	cgd1_3330			
**2h**	0.00E+00	2.56E-08	0.00E+00	0.00E+00
**6h**	6.79E-09	2.63E-08	8.12E-08	2.95E-08
**12h**	1.98E-09	5.65E-09	2.33E-10	4.97E-09
**24h**	6.27E-08	3.54E-09	2.38E-08	2.00E-08
**36h**	2.16E-08	9.10E-09	3.62E-08	4.91E-08
**48h**	1.00E-08	9.43E-09	1.00E-07	6.71E-08
**72h**	2.19E-08	2.70E-08	2.66E-08	9.57E-08
	cgd1_3340			
**2h**	4.82E-07	3.51E-07	5.23E-07	4.73E-07
**6h**	1.04E-07	2.38E-07	4.77E-07	5.12E-07
**12h**	2.94E-07	8.63E-07	7.32E-08	1.70E-06
**24h**	1.99E-07	8.25E-08	3.37E-07	5.20E-07
**36h**	2.41E-07	1.52E-07	4.53E-07	5.49E-07
**48h**	1.61E-07	1.71E-07	8.58E-07	6.42E-07
**72h**	1.73E-07	1.17E-07	2.03E-07	2.92E-07
	cgd1_3360			
**2h**	3.69E-08	6.80E-09	7.20E-08	5.18E-08
**6h**	2.40E-08	8.88E-08	5.10E-08	1.87E-07
**12h**	3.25E-08	1.57E-08	3.45E-09	1.32E-08
**24h**	5.36E-08	8.61E-09	4.51E-08	4.44E-08
**36h**	2.99E-08	1.27E-08	2.95E-08	2.51E-08
**48h**	3.58E-08	4.01E-08	4.03E-07	1.58E-07
**72h**	3.62E-07	1.38E-07	9.08E-07	2.00E-06
	cgd1_3400			
**2h**	3.84E-04	1.94E-04	4.79E-04	5.52E-06
**6h**	2.83E-04	7.37E-04	4.94E-04	1.03E-03
**12h**	6.32E-04	8.42E-04	2.40E-05	1.02E-03
**24h**	1.01E-03	5.73E-04	5.84E-04	2.27E-03
**36h**	2.33E-04	2.94E-04	8.62E-05	6.36E-04
**48h**	2.13E-04	1.03E-04	2.33E-03	2.36E-04
**72h**	4.71E-04	9.92E-05	5.45E-04	2.77E-04
	cgd1_3410			
**2h**	0.00E+00	0.00E+00	0.00E+00	0.00E+00
**6h**	1.49E-08	1.36E-08	3.94E-07	5.80E-07
**12h**	5.39E-09	4.25E-08	8.65E-10	0.00E+00
**24h**	3.62E-07	6.64E-08	1.28E-07	2.00E-07
**36h**	5.96E-07	2.16E-07	5.23E-07	7.73E-07
**48h**	2.20E-07	1.67E-07	9.52E-07	6.58E-07
**72h**	2.96E-07	1.69E-07	3.32E-07	2.00E-07
	cgd1_3420			
**2h**	6.63E-09	1.95E-08	3.51E-08	0.00E+00
**6h**	1.99E-08	3.73E-08	8.98E-08	3.08E-10
**12h**	4.71E-09	5.82E-09	3.62E-10	6.12E-09

**24h**	1.22E-08	4.94E-09	7.96E-09	2.13E-08
**36h**	4.67E-08	3.10E-08	5.23E-08	6.13E-08
**48h**	5.25E-08	3.91E-08	6.93E-08	4.33E-08
**72h**	4.17E-08	3.39E-08	8.32E-08	4.06E-08
	cgd1_3450			
**2h**	4.96E-08	1.95E-08	3.81E-08	9.50E-08
**6h**	8.28E-10	0.00E+00	7.25E-09	0.00E+00
**12h**	1.15E-07	1.44E-07	1.03E-08	8.98E-08
**24h**	1.19E-07	8.57E-08	1.51E-08	1.38E-08
**36h**	9.95E-08	3.84E-08	1.88E-07	9.08E-08
**48h**	5.14E-08	4.89E-08	2.40E-07	1.67E-07
**72h**	2.58E-07	4.54E-08	1.72E-07	8.44E-08
	cgd1_3470			
**2h**	0.00E+00	0.00E+00	2.21E-09	0.00E+00
**6h**	6.84E-09	2.72E-08	8.05E-08	3.10E-08
**12h**	1.35E-08	0.00E+00	0.00E+00	0.00E+00
**24h**	7.96E-08	1.93E-08	1.15E-07	6.89E-08
**36h**	5.02E-08	1.20E-07	3.79E-07	4.25E-07
**48h**	4.15E-07	1.30E-07	6.33E-07	3.08E-07
**72h**	3.42E-07	2.57E-07	7.22E-07	5.44E-07
	cgd1_3500			
**2h**	3.90E-09	4.61E-10	5.60E-09	0.00E+00
**6h**	1.48E-09	2.52E-10	8.65E-08	0.00E+00
**12h**	1.47E-07	1.11E-07	3.43E-09	8.44E-08
**24h**	3.63E-07	1.93E-08	1.31E-07	1.59E-08
**36h**	1.87E-06	4.93E-07	2.73E-06	2.39E-06
**48h**	5.42E-07	7.19E-07	2.06E-06	1.10E-06
**72h**	2.34E-06	5.81E-07	2.42E-06	1.53E-06
	cgd1_3510			
**2h**	0.00E+00	0.00E+00	0.00E+00	0.00E+00
**6h**	0.00E+00	0.00E+00	0.00E+00	0.00E+00
**12h**	9.62E-05	6.26E-05	3.64E-04	9.64E-05
**24h**	3.79E-05	8.67E-05	2.28E-04	1.27E-05
**36h**	6.26E-05	1.81E-04	9.05E-04	9.59E-05
**48h**	1.03E-04	1.45E-04	7.61E-04	3.97E-05
**72h**	2.65E-04	2.11E-04	1.67E-03	9.49E-05
	cgd1_3530			
**2h**	9.16E-03	4.87E-03	1.45E-02	6.09E-03
**6h**	1.83E-02	2.95E-02	7.53E-02	7.90E-03
**12h**	2.84E-03	8.89E-03	1.32E-02	7.43E-04
**24h**	3.60E-03	9.43E-03	3.67E-02	1.30E-03
**36h**	3.28E-03	5.35E-03	1.75E-02	2.86E-03
**48h**	8.41E-04	9.47E-04	7.02E-03	4.39E-04
**72h**	7.45E-04	2.25E-04	1.35E-03	2.19E-04
	cgd1_3540			
**2h**	1.16E-03	9.77E-04	5.15E-03	2.00E-03
**6h**	2.93E-03	1.38E-03	1.12E-02	6.22E-04
**12h**	3.37E-04	1.66E-03	3.96E-03	1.18E-04
**24h**	2.64E-04	7.64E-04	8.23E-03	2.63E-04
**36h**	3.46E-04	6.73E-04	2.59E-03	5.61E-04
**48h**	9.59E-05	1.74E-04	1.41E-03	1.24E-04
**72h**	1.90E-04	1.81E-04	1.23E-03	2.00E-04
	cgd1_3560			

**2h**	0.00E+00	0.00E+00	0.00E+00	0.00E+00
**6h**	0.00E+00	0.00E+00	0.00E+00	0.00E+00
**12h**	1.69E-09	0.00E+00	0.00E+00	0.00E+00
**24h**	6.51E-09	2.56E-11	6.83E-11	0.00E+00
**36h**	1.05E-07	5.93E-17	2.24E-10	2.06E-07
**48h**	4.87E-06	2.58E-07	1.41E-06	3.95E-06
**72h**	1.90E-06	3.83E-10	6.77E-07	3.58E-06
	cgd1_3570			
**2h**	6.23E-08	1.69E-08	3.56E-06	6.93E-08
**6h**	0.00E+00	0.00E+00	0.00E+00	4.71E-08
**12h**	1.99E-03	8.32E-04	2.21E-03	1.26E-03
**24h**	5.47E-05	1.73E-05	1.87E-04	4.10E-05
**36h**	3.50E-05	1.56E-04	5.52E-04	2.26E-05
**48h**	1.07E-05	2.87E-05	1.40E-04	2.09E-05
**72h**	6.70E-06	3.21E-06	1.41E-05	1.55E-06
	cgd1_3580			
**2h**	0.00E+00	0.00E+00	0.00E+00	0.00E+00
**6h**	0.00E+00	0.00E+00	4.10E-08	0.00E+00
**12h**	6.21E-05	7.74E-05	1.08E-04	5.38E-05
**24h**	1.43E-05	1.25E-05	3.69E-05	9.80E-06
**36h**	1.71E-05	6.47E-05	2.65E-04	1.47E-05
**48h**	1.04E-05	1.64E-05	7.58E-05	4.41E-06
**72h**	6.92E-07	7.67E-07	7.38E-06	2.64E-07
	cgd1_3590			
**2h**	0.00E+00	0.00E+00	5.62E-10	0.00E+00
**6h**	0.00E+00	0.00E+00	0.00E+00	0.00E+00
**12h**	6.64E-07	8.89E-07	2.38E-07	3.48E-07
**24h**	1.71E-07	1.86E-07	3.81E-07	1.86E-07
**36h**	7.12E-07	1.46E-06	1.72E-06	3.55E-07
**48h**	7.19E-07	1.49E-06	4.97E-07	3.82E-07
**72h**	1.28E-07	6.69E-08	1.74E-07	7.16E-08
	cgd1_3600			
**2h**	8.68E-07	1.14E-08	2.59E-07	1.60E-07
**6h**	6.96E-08	0.00E+00	1.29E-08	4.82E-09
**12h**	1.09E-06	2.48E-07	3.81E-07	3.89E-07
**24h**	6.07E-07	8.55E-07	5.20E-07	3.65E-07
**36h**	6.79E-07	7.47E-07	1.10E-06	2.25E-07
**48h**	1.37E-06	1.66E-06	1.58E-06	3.57E-07
**72h**	8.40E-07	4.63E-07	2.23E-06	4.39E-07
	cgd1_3610			
**2h**	6.54E-05	4.38E-05	2.31E-04	4.94E-05
**6h**	2.52E-05	2.18E-05	8.43E-05	2.72E-05
**12h**	3.24E-05	5.94E-05	6.16E-05	1.16E-05
**24h**	3.76E-05	7.17E-05	6.20E-05	1.11E-05
**36h**	1.89E-05	2.18E-05	8.43E-05	9.67E-06
**48h**	1.44E-05	2.87E-05	1.04E-04	4.08E-06
**72h**	2.88E-06	4.03E-06	1.38E-05	2.23E-06
	cgd1_3620			
**2h**	2.23E-02	1.23E-02	3.90E-04	5.74E-03
**6h**	2.13E-03	6.57E-03	2.04E-03	2.92E-03
**12h**	2.53E-04	2.46E-03	4.53E-04	1.02E-05
**24h**	6.58E-04	2.47E-03	2.92E-04	7.54E-04
**36h**	2.49E-03	1.04E-03	4.25E-04	1.19E-03

**48h**	1.39E-03	1.28E-03	2.19E-04	3.28E-04
**72h**	4.86E-04	1.22E-03	1.26E-05	7.06E-04
	cgd1_3630			
**2h**	2.51E-06	5.10E-07	1.25E-06	3.13E-07
**6h**	2.23E-07	8.99E-07	1.97E-06	1.94E-06
**12h**	6.24E-06	3.21E-06	1.27E-05	3.85E-06
**24h**	4.81E-06	8.30E-07	1.22E-05	1.30E-05
**36h**	6.72E-06	5.91E-06	3.55E-05	4.24E-05
**48h**	1.02E-05	2.02E-06	8.60E-06	1.80E-05
**72h**	2.02E-06	1.01E-06	2.36E-06	2.76E-06
	cgd1_3640			
**2h**	4.94E-03	9.34E-03	2.45E-04	3.95E-04
**6h**	1.60E-02	3.49E-03	4.63E-04	8.89E-04
**12h**	4.17E-02	5.20E-02	8.94E-04	7.38E-04
**24h**	7.15E-03	5.38E-03	2.90E-04	2.59E-04
**36h**	1.79E-02	4.57E-03	2.91E-04	8.88E-04
**48h**	1.10E-03	2.82E-03	1.60E-04	1.71E-04
**72h**	3.91E-03	8.41E-04	3.49E-05	1.72E-04
	cgd4_130			
**2h**	0.00E+00	0.00E+00	0.00E+00	0.00E+00
**6h**	0.00E+00	0.00E+00	0.00E+00	0.00E+00
**12h**	0.00E+00	5.20E-08	2.94E-08	0.00E+00
**24h**	0.00E+00	2.00E-09	0.00E+00	1.26E-08
**36h**	2.31E-09	9.36E-07	0.00E+00	5.28E-07
**48h**	2.15E-05	2.41E-05	1.99E-05	3.58E-05
**72h**	2.66E-07	1.65E-06	1.74E-06	1.46E-06
	cgd4_150			
**2h**	0.00E+00	5.03E-09	0.00E+00	8.73E-09
**6h**	2.38E-09	0.00E+00	0.00E+00	8.48E-09
**12h**	6.58E-09	1.00E-10	2.57E-09	4.02E-09
**24h**	9.36E-10	9.62E-10	7.32E-09	1.29E-08
**36h**	1.02E-08	9.77E-09	6.36E-09	1.06E-08
**48h**	5.03E-08	9.71E-09	7.88E-09	3.12E-09
**72h**	2.76E-09	7.15E-09	1.15E-08	9.58E-09
	cgd4_160			
**2h**	5.09E-08	2.55E-06	4.31E-07	1.10E-05
**6h**	3.64E-09	2.46E-07	7.57E-08	2.10E-07
**12h**	4.11E-08	7.65E-07	1.35E-06	1.28E-06
**24h**	3.41E-08	1.65E-07	1.14E-07	4.74E-07
**36h**	7.61E-08	2.58E-07	4.05E-08	2.92E-07
**48h**	2.07E-06	2.46E-07	3.52E-07	5.24E-07
**72h**	3.08E-08	9.70E-08	5.37E-08	1.57E-07
	cgd4_190			
**2h**	0.00E+00	0.00E+00	0.00E+00	0.00E+00
**6h**	1.82E-05	7.93E-06	4.19E-05	5.44E-06
**12h**	4.81E-06	6.41E-06	9.35E-06	7.27E-06
**24h**	1.73E-06	2.12E-06	6.22E-05	5.98E-06
**36h**	1.64E-05	2.12E-05	1.41E-04	3.19E-05
**48h**	2.13E-05	1.23E-05	2.61E-05	4.29E-06
**72h**	2.55E-05	1.28E-05	7.99E-05	2.26E-06
	cgd4_210			
**2h**	0.00E+00	0.00E+00	0.00E+00	0.00E+00
**6h**	2.29E-08	5.33E-08	8.73E-08	4.66E-07

**12h**	0.00E+00	3.50E-09	3.19E-10	5.37E-09
**24h**	5.14E-09	3.95E-08	1.32E-07	2.14E-07
**36h**	1.45E-07	1.41E-07	7.75E-08	6.16E-07
**48h**	1.97E-06	5.73E-07	3.37E-07	3.34E-07
**72h**	7.06E-09	2.55E-08	3.52E-08	1.61E-07
	cgd4_230			
**2h**	0.00E+00	0.00E+00	0.00E+00	0.00E+00
**6h**	0.00E+00	0.00E+00	0.00E+00	0.00E+00
**12h**	5.44E-09	4.84E-09	6.02E-10	2.63E-08
**24h**	6.58E-10	9.48E-10	5.13E-09	2.18E-09
**36h**	4.01E-09	1.31E-08	4.04E-09	1.64E-07
**48h**	3.79E-07	3.48E-08	1.69E-08	4.30E-08
**72h**	1.00E-09	2.46E-09	1.34E-09	2.74E-08
	cgd4_240			
**2h**	0.00E+00	0.00E+00	0.00E+00	0.00E+00
**6h**	0.00E+00	5.68E-08	0.00E+00	0.00E+00
**12h**	4.69E-07	1.23E-06	1.50E-06	9.36E-07
**24h**	2.51E-07	4.08E-07	5.16E-07	3.51E-07
**36h**	3.70E-07	2.61E-07	1.31E-06	9.81E-07
**48h**	3.99E-07	5.51E-07	3.28E-07	8.35E-07
**72h**	1.53E-08	2.39E-08	3.24E-08	1.04E-08
	cgd4_250			
**2h**	0.00E+00	2.82E-09	8.07E-08	1.15E-07
**6h**	1.19E-08	1.84E-07	9.51E-08	2.04E-06
**12h**	2.79E-08	7.19E-09	2.60E-09	4.73E-08
**24h**	1.24E-07	1.22E-07	5.73E-07	1.62E-06
**36h**	5.30E-07	6.96E-07	2.79E-07	1.96E-06
**48h**	4.36E-06	4.64E-07	2.31E-07	6.62E-07
**72h**	4.21E-08	5.21E-08	3.88E-08	1.33E-07
	cgd4_260			
**2h**	0.00E+00	0.00E+00	0.00E+00	2.69E-06
**6h**	5.93E-07	2.82E-05	3.47E-04	1.46E-04
**12h**	1.25E-06	2.90E-06	1.71E-05	5.86E-06
**24h**	5.83E-07	2.40E-05	1.86E-04	1.08E-04
**36h**	4.76E-06	4.55E-04	3.81E-05	1.29E-04
**48h**	5.90E-05	6.51E-05	1.15E-04	1.12E-04
**72h**	8.13E-06	1.03E-05	2.55E-05	2.97E-05
	cgd4_280			
**2h**	1.81E-05	2.71E-06	2.14E-06	3.10E-06
**6h**	4.70E-08	3.18E-07	1.49E-05	1.24E-05
**12h**	1.71E-07	6.99E-07	6.30E-06	1.21E-06
**24h**	1.13E-06	1.80E-05	3.54E-05	1.89E-05
**36h**	3.84E-07	8.26E-06	9.95E-07	7.08E-06
**48h**	6.22E-05	3.09E-05	1.00E-05	1.95E-05
**72h**	1.36E-06	3.20E-06	2.54E-06	6.05E-06
	cgd4_290			
**2h**	2.07E-08	1.06E-08	0.00E+00	1.51E-07
**6h**	2.07E-08	1.39E-07	9.39E-08	7.58E-07
**12h**	1.66E-07	1.04E-07	1.04E-07	5.73E-07
**24h**	5.06E-08	1.39E-08	4.91E-08	4.30E-07
**36h**	1.44E-07	1.16E-07	1.21E-07	1.33E-06
**48h**	1.71E-07	9.28E-08	6.28E-08	2.26E-06
**72h**	3.96E-08	3.92E-08	4.57E-08	9.77E-08

	cgd4_300			
**2h**	0.00E+00	1.42E-06	0.00E+00	1.22E-05
**6h**	1.88E-07	1.07E-06	2.64E-07	2.25E-06
**12h**	1.25E-07	5.66E-07	4.68E-07	1.34E-05
**24h**	4.00E-07	2.52E-06	4.42E-07	1.50E-06
**36h**	6.32E-07	3.89E-06	2.32E-06	4.89E-06
**48h**	1.95E-06	1.33E-06	2.28E-07	3.01E-06
**72h**	1.70E-06	3.24E-06	8.64E-07	1.06E-05
	cgd4_310			
**2h**	0.00E+00	4.14E-07	1.01E-06	0.00E+00
**6h**	8.39E-07	9.06E-07	1.55E-06	4.32E-06
**12h**	0.00E+00	1.21E-07	3.50E-07	4.43E-07
**24h**	1.64E-07	1.37E-06	4.40E-07	5.13E-06
**36h**	4.55E-07	8.04E-06	4.42E-06	6.76E-06
**48h**	6.23E-06	2.94E-06	3.13E-06	3.87E-06
**72h**	4.92E-07	1.25E-06	1.50E-06	1.95E-06
	cgd4_340			
**2h**	1.59E-08	3.54E-07	0.00E+00	1.00E-05
**6h**	0.00E+00	3.11E-09	2.94E-07	1.27E-05
**12h**	0.00E+00	2.50E-07	1.18E-07	1.81E-06
**24h**	1.49E-07	8.45E-07	1.53E-06	4.58E-06
**36h**	3.91E-07	6.14E-06	6.46E-06	6.11E-06
**48h**	1.16E-05	3.67E-06	1.83E-06	2.19E-06
**72h**	1.22E-07	1.25E-06	1.88E-06	1.16E-06
	cgd4_360			
**2h**	0.00E+00	1.53E-08	0.00E+00	2.28E-07
**6h**	8.34E-08	2.20E-07	6.83E-08	3.71E-07
**12h**	0.00E+00	1.03E-08	6.90E-10	2.32E-08
**24h**	3.28E-08	7.51E-08	8.28E-09	4.70E-07
**36h**	5.86E-08	4.89E-07	4.45E-07	8.29E-07
**48h**	1.38E-06	6.93E-07	5.43E-07	5.62E-07
**72h**	2.39E-07	1.97E-07	3.56E-07	2.74E-07
	cgd4_370			
**2h**	4.11E-08	9.38E-09	0.00E+00	2.72E-07
**6h**	2.62E-08	1.17E-08	4.33E-09	1.36E-07
**12h**	0.00E+00	2.65E-09	9.24E-09	7.24E-09
**24h**	2.32E-09	2.52E-08	1.10E-08	2.38E-07
**36h**	1.00E-08	1.60E-07	2.93E-07	5.63E-07
**48h**	7.75E-07	4.26E-07	2.71E-07	4.20E-07
**72h**	4.44E-08	3.35E-07	2.85E-07	3.76E-07
	cgd4_390			
**2h**	0.00E+00	0.00E+00	0.00E+00	7.15E-11
**6h**	3.29E-08	4.34E-08	2.93E-08	5.15E-08
**12h**	7.80E-09	1.51E-10	3.67E-10	4.83E-09
**24h**	7.77E-09	9.82E-09	2.28E-08	6.32E-08
**36h**	5.09E-08	2.60E-08	2.01E-08	4.25E-08
**48h**	4.65E-08	2.28E-08	5.31E-08	7.17E-08
**72h**	1.85E-08	3.13E-08	8.07E-09	5.40E-09
	cgd4_420			
**2h**	2.55E-04	2.85E-04	2.60E-04	8.28E-04
**6h**	1.70E-04	1.76E-04	2.61E-04	4.69E-04
**12h**	2.77E-05	1.26E-04	1.72E-04	2.27E-04
**24h**	1.63E-04	3.02E-06	4.44E-04	4.10E-04

**36h**	9.55E-05	6.59E-05	3.92E-05	3.63E-04
**48h**	1.45E-04	2.65E-05	6.96E-05	2.69E-04
**72h**	6.55E-05	1.31E-05	1.82E-04	2.46E-04
	cgd4_440			
**2h**	1.81E-09	2.58E-09	1.03E-09	9.69E-08
**6h**	7.87E-09	1.11E-09	1.24E-08	1.20E-07
**12h**	1.80E-08	1.41E-09	9.08E-10	6.35E-09
**24h**	1.06E-08	3.14E-09	2.18E-09	1.15E-07
**36h**	6.53E-09	6.75E-08	1.94E-07	5.74E-08
**48h**	2.33E-07	6.70E-08	4.93E-08	6.27E-08
**72h**	4.78E-08	1.54E-08	8.77E-08	6.52E-08
	cgd4_450			
**2h**	1.66E-06	0.00E+00	2.40E-05	1.36E-05
**6h**	7.20E-06	5.18E-05	2.46E-05	5.29E-05
**12h**	5.47E-06	3.80E-05	5.75E-06	6.65E-05
**24h**	2.17E-05	1.38E-05	9.77E-06	3.42E-05
**36h**	3.52E-05	3.93E-04	3.34E-04	2.30E-04
**48h**	2.94E-04	3.44E-04	7.82E-05	8.33E-05
**72h**	4.35E-04	2.75E-04	3.79E-04	4.86E-04
	cgd4_460			
**2h**	1.54E-08	9.75E-10	1.29E-08	9.39E-09
**6h**	6.75E-08	3.16E-08	1.38E-08	7.55E-08
**12h**	1.69E-09	8.06E-09	3.02E-09	2.39E-08
**24h**	4.67E-08	6.18E-09	7.25E-09	8.92E-08
**36h**	3.60E-08	1.74E-08	1.03E-07	1.18E-07
**48h**	7.03E-08	1.46E-08	2.74E-08	2.12E-08
**72h**	5.15E-09	4.02E-09	4.02E-09	7.93E-09
	cgd4_470			
**2h**	7.59E-08	8.70E-09	1.16E-07	9.01E-08
**6h**	1.21E-06	3.53E-07	4.49E-07	2.60E-07
**12h**	1.11E-06	8.17E-08	2.18E-07	9.94E-08
**24h**	2.84E-06	3.33E-07	2.59E-07	4.12E-07
**36h**	1.01E-06	1.61E-07	1.33E-07	1.63E-07
**48h**	1.58E-07	1.00E-07	2.67E-07	5.55E-08
**72h**	1.33E-07	3.81E-08	1.54E-08	3.56E-08
	cgd4_480			
**2h**	0.00E+00	0.00E+00	2.44E-08	1.58E-08
**6h**	3.96E-07	8.90E-09	4.43E-09	2.09E-07
**12h**	0.00E+00	2.80E-09	6.28E-10	9.92E-08
**24h**	1.22E-08	3.73E-08	1.44E-08	1.89E-07
**36h**	2.70E-08	2.35E-07	1.22E-06	1.17E-06
**48h**	4.93E-07	2.19E-07	4.09E-07	2.44E-07
**72h**	3.86E-07	2.09E-07	4.09E-07	2.42E-07
	cgd4_490			
**2h**	5.52E-08	2.51E-07	1.13E-08	4.21E-06
**6h**	4.32E-08	1.21E-08	5.75E-09	6.17E-07
**12h**	1.53E-07	4.30E-08	1.06E-07	5.97E-07
**24h**	1.26E-08	4.07E-08	8.31E-08	6.10E-07
**36h**	1.24E-07	2.66E-06	2.32E-06	3.06E-06
**48h**	4.60E-07	4.44E-07	1.60E-06	5.31E-07
**72h**	3.31E-07	6.47E-07	5.07E-07	1.51E-06
	cgd4_500			
**2h**	0.00E+00	1.87E-08	0.00E+00	0.00E+00

**6h**	0.00E+00	0.00E+00	0.00E+00	0.00E+00
**12h**	0.00E+00	0.00E+00	0.00E+00	9.06E-08
**24h**	1.57E-10	0.00E+00	5.29E-09	2.62E-07
**36h**	3.48E-09	2.84E-08	0.00E+00	2.10E-07
**48h**	5.45E-07	6.17E-08	2.65E-07	1.20E-05
**72h**	6.10E-07	6.41E-07	5.84E-06	1.18E-05
	cgd4_510			
**2h**	0.00E+00	0.00E+00	1.61E-09	0.00E+00
**6h**	2.44E-09	5.64E-09	1.25E-07	9.75E-07
**12h**	0.00E+00	1.87E-09	5.92E-09	4.21E-08
**24h**	5.98E-09	6.17E-09	6.07E-08	2.00E-07
**36h**	9.21E-09	6.96E-08	3.73E-09	4.31E-07
**48h**	1.58E-07	2.72E-08	4.41E-08	5.58E-08
**72h**	3.89E-08	1.45E-08	6.72E-09	5.42E-08
	cgd4_540			
**2h**	3.58E-08	1.40E-07	2.36E-07	1.07E-05
**6h**	1.75E-08	1.18E-07	9.12E-08	7.13E-07
**12h**	4.05E-08	3.21E-08	1.15E-07	8.14E-07
**24h**	4.23E-08	4.22E-08	1.66E-07	3.97E-07
**36h**	4.98E-08	4.21E-07	3.71E-08	9.04E-07
**48h**	5.23E-07	1.48E-07	7.45E-08	1.16E-07
**72h**	2.55E-08	1.20E-08	8.72E-09	7.26E-08
	cgd4_550			
**2h**	0.00E+00	0.00E+00	0.00E+00	0.00E+00
**6h**	0.00E+00	0.00E+00	0.00E+00	0.00E+00
**12h**	9.33E-10	5.11E-10	0.00E+00	2.10E-10
**24h**	0.00E+00	1.61E-10	9.53E-11	1.71E-10
**36h**	2.94E-11	8.72E-10	7.40E-11	2.80E-09
**48h**	4.18E-09	2.87E-09	2.25E-10	1.30E-09
**72h**	2.66E-09	3.07E-09	2.34E-10	3.64E-09
	cgd4_560			
**2h**	0.00E+00	0.00E+00	2.95E-05	0.00E+00
**6h**	0.00E+00	0.00E+00	0.00E+00	0.00E+00
**12h**	1.89E-05	4.15E-08	5.16E-05	4.98E-06
**24h**	3.28E-06	5.91E-07	6.42E-05	4.03E-06
**36h**	5.78E-05	4.13E-06	1.63E-05	1.56E-05
**48h**	3.57E-05	2.43E-05	2.18E-05	2.38E-05
**72h**	4.32E-04	1.88E-05	4.08E-05	1.19E-04
	cgd4_590			
**2h**	2.52E-08	1.76E-08	2.59E-08	4.06E-07
**6h**	5.97E-07	2.92E-07	1.12E-07	5.69E-07
**12h**	1.46E-07	1.64E-07	9.99E-08	3.81E-07
**24h**	7.07E-07	1.38E-07	2.88E-07	1.51E-07
**36h**	3.37E-07	2.67E-07	3.99E-07	1.41E-06
**48h**	1.32E-06	5.36E-07	2.27E-07	3.36E-07
**72h**	1.70E-07	7.01E-08	9.53E-08	1.08E-07
	cgd4_600			
**2h**	0.00E+00	4.02E-10	0.00E+00	4.97E-10
**6h**	0.00E+00	0.00E+00	0.00E+00	0.00E+00
**12h**	3.37E-08	6.89E-08	2.24E-07	8.38E-08
**24h**	2.87E-09	6.40E-09	2.92E-08	1.60E-08
**36h**	6.72E-08	1.49E-07	1.12E-08	1.20E-07
**48h**	5.10E-07	1.16E-07	2.78E-08	1.86E-07

**72h**	1.95E-07	8.79E-08	3.94E-08	1.39E-07
	cgd4_610			
**2h**	0.00E+00	4.81E-07	3.16E-06	2.99E-05
**6h**	1.59E-07	5.27E-07	1.55E-06	4.65E-06
**12h**	7.87E-07	1.43E-07	3.40E-06	1.38E-05
**24h**	1.06E-06	1.30E-07	5.86E-06	9.47E-06
**36h**	3.71E-07	1.65E-06	2.45E-07	1.65E-05
**48h**	1.48E-05	7.92E-07	3.06E-06	5.92E-06
**72h**	1.85E-06	9.57E-07	1.83E-06	3.99E-06
	cgd4_640			
**2h**	0.00E+00	0.00E+00	0.00E+00	0.00E+00
**6h**	0.00E+00	0.00E+00	0.00E+00	6.81E-11
**12h**	0.00E+00	1.70E-10	5.16E-10	9.31E-11
**24h**	0.00E+00	3.59E-11	0.00E+00	1.09E-10
**36h**	0.00E+00	6.03E-10	0.00E+00	5.96E-09
**48h**	2.93E-08	4.73E-09	2.05E-09	1.68E-08
**72h**	7.63E-09	8.52E-09	2.09E-08	4.57E-08
	cgd4_660			
**2h**	0.00E+00	3.34E-08	1.23E-08	7.75E-08
**6h**	2.04E-09	7.74E-09	1.28E-08	6.48E-09
**12h**	3.66E-08	1.10E-08	2.49E-08	9.48E-08
**24h**	6.67E-08	7.42E-09	3.15E-08	1.18E-07
**36h**	7.05E-09	3.69E-08	5.45E-09	1.29E-08
**48h**	3.16E-07	2.80E-08	1.00E-07	1.90E-07
**72h**	1.79E-07	3.85E-08	5.77E-08	8.63E-08
	cgd4_670			
**2h**	0.00E+00	0.00E+00	0.00E+00	0.00E+00
**6h**	0.00E+00	0.00E+00	0.00E+00	4.13E-10
**12h**	0.00E+00	8.01E-10	0.00E+00	0.00E+00
**24h**	2.23E-09	4.95E-10	0.00E+00	5.90E-09
**36h**	1.39E-08	4.78E-09	0.00E+00	2.39E-08
**48h**	3.98E-06	2.12E-07	2.83E-06	2.35E-06
**72h**	5.32E-06	1.24E-06	1.21E-05	1.28E-05
	cgd4_680			
**2h**	0.00E+00	0.00E+00	0.00E+00	0.00E+00
**6h**	0.00E+00	0.00E+00	0.00E+00	0.00E+00
**12h**	0.00E+00	1.34E-08	2.61E-07	5.89E-07
**24h**	2.33E-09	9.09E-09	5.04E-08	8.58E-08
**36h**	4.50E-08	3.69E-07	1.60E-07	4.04E-06
**48h**	9.54E-07	4.91E-07	2.69E-07	7.91E-07
**72h**	3.27E-06	3.35E-06	5.97E-06	1.09E-05
	cgd4_690			
**2h**	0.00E+00	2.44E-06	5.35E-09	1.24E-06
**6h**	5.23E-07	4.63E-06	0.00E+00	4.21E-06
**12h**	2.60E-06	5.95E-07	2.07E-08	2.18E-07
**24h**	2.80E-06	1.03E-06	7.01E-07	4.19E-06
**36h**	9.28E-06	2.09E-05	3.66E-07	1.12E-05
**48h**	6.20E-04	9.28E-05	3.35E-05	4.21E-05
**72h**	2.75E-04	7.28E-05	1.92E-05	5.10E-05
	cgd4_700			
**2h**	0.00E+00	0.00E+00	0.00E+00	4.25E-08
**6h**	3.51E-06	2.77E-06	6.58E-08	2.02E-06
**12h**	1.20E-06	4.33E-05	3.34E-07	5.20E-07

**24h**	3.79E-06	1.22E-05	2.19E-07	8.08E-07
**36h**	6.40E-06	1.38E-04	1.53E-06	8.34E-06
**48h**	1.30E-05	9.68E-05	2.61E-06	3.67E-06
**72h**	1.68E-05	1.33E-04	4.88E-06	7.78E-06
	cgd4_720			
**2h**	8.94E-08	7.86E-08	1.65E-08	1.25E-07
**6h**	4.83E-08	2.32E-08	2.64E-09	5.18E-08
**12h**	1.72E-08	2.13E-07	2.21E-08	5.60E-08
**24h**	1.07E-08	6.89E-08	2.05E-08	5.19E-08
**36h**	3.24E-08	1.25E-07	9.29E-09	3.76E-08
**48h**	3.03E-09	6.48E-08	4.55E-09	1.13E-08
**72h**	5.16E-08	1.78E-07	8.75E-09	2.54E-08
	cgd4_730			
**2h**	0.00E+00	0.00E+00	0.00E+00	3.65E-09
**6h**	0.00E+00	0.00E+00	0.00E+00	0.00E+00
**12h**	0.00E+00	8.21E-07	0.00E+00	3.75E-10
**24h**	0.00E+00	0.00E+00	0.00E+00	1.94E-10
**36h**	0.00E+00	7.29E-08	0.00E+00	3.01E-09
**48h**	9.48E-09	1.04E-07	2.10E-09	1.40E-08
**72h**	5.75E-08	0.00E+00	4.97E-08	6.32E-08
	cgd4_740			
**2h**	0.00E+00	8.05E-08	0.00E+00	2.69E-08
**6h**	6.44E-05	5.03E-05	1.52E-05	3.89E-05
**12h**	8.14E-07	6.07E-06	4.94E-07	7.51E-07
**24h**	8.71E-05	1.54E-05	2.70E-05	3.82E-05
**36h**	8.28E-05	1.66E-04	3.69E-05	4.24E-05
**48h**	1.63E-04	5.23E-05	4.44E-05	1.85E-05
**72h**	5.60E-06	9.52E-06	9.31E-07	2.75E-06
	cgd4_750			
**2h**	0.00E+00	0.00E+00	2.69E-09	6.59E-08
**6h**	0.00E+00	0.00E+00	2.60E-09	1.52E-08
**12h**	2.97E-09	2.24E-09	9.01E-09	6.68E-09
**24h**	3.54E-10	2.69E-09	2.71E-09	1.97E-08
**36h**	8.93E-09	9.96E-08	2.50E-09	3.31E-08
**48h**	1.14E-07	1.76E-07	3.45E-08	5.11E-07
**72h**	1.15E-07	2.95E-08	3.49E-08	1.68E-07
	cgd4_760			
**2h**	8.36E-09	3.76E-06	1.89E-08	3.78E-08
**6h**	8.58E-08	2.19E-07	1.63E-08	3.18E-09
**12h**	2.15E-08	4.17E-08	3.70E-09	6.80E-09
**24h**	1.29E-07	1.95E-07	1.29E-08	3.91E-08
**36h**	1.48E-07	2.29E-07	8.81E-08	2.85E-08
**48h**	7.69E-08	5.91E-07	1.66E-08	5.97E-08
**72h**	5.77E-08	1.22E-07	1.13E-08	2.59E-08
	cgd4_770			
**2h**	0.00E+00	0.00E+00	0.00E+00	0.00E+00
**6h**	2.24E-04	3.28E-07	4.30E-05	1.20E-05
**12h**	0.00E+00	2.67E-07	1.26E-06	2.14E-07
**24h**	3.38E-06	5.74E-07	1.40E-06	1.08E-05
**36h**	1.21E-06	1.41E-05	1.02E-06	1.42E-05
**48h**	2.24E-05	1.26E-06	4.34E-06	1.81E-05
**72h**	5.46E-06	2.28E-07	1.52E-06	2.42E-06
	cgd4_780			

**2h**	0.00E+00	0.00E+00	0.00E+00	0.00E+00
**6h**	0.00E+00	2.17E-06	1.03E-09	9.16E-08
**12h**	3.94E-08	2.11E-06	2.39E-08	5.46E-08
**24h**	4.42E-09	9.65E-07	2.56E-08	2.75E-06
**36h**	7.94E-08	4.04E-05	1.50E-08	9.51E-07
**48h**	3.57E-06	1.08E-05	2.42E-07	6.76E-07
**72h**	2.28E-06	2.77E-05	7.97E-08	4.52E-07
	cgd4_790			
**2h**	9.77E-09	0.00E+00	2.56E-08	0.00E+00
**6h**	3.28E-08	1.53E-06	1.61E-07	2.01E-07
**12h**	9.65E-09	1.36E-07	8.36E-10	2.35E-08
**24h**	3.70E-08	7.19E-07	1.68E-07	2.77E-07
**36h**	3.33E-08	6.21E-06	1.30E-08	4.90E-07
**48h**	1.87E-06	1.90E-05	2.42E-07	5.35E-07
**72h**	3.02E-07	1.44E-06	2.14E-08	2.23E-07
	cgd4_820			
**2h**	2.12E-05	0.00E+00	3.70E-08	1.16E-07
**6h**	4.00E-06	7.12E-06	3.71E-08	5.31E-07
**12h**	9.99E-06	1.23E-06	3.90E-07	5.97E-06
**24h**	1.96E-06	6.19E-07	2.49E-07	8.64E-07
**36h**	4.29E-05	6.99E-06	3.55E-08	8.60E-07
**48h**	2.12E-05	1.86E-06	1.03E-07	3.50E-07
**72h**	1.36E-05	1.01E-06	5.85E-08	5.75E-07
	cgd4_850			
**2h**	0.00E+00	0.00E+00	0.00E+00	0.00E+00
**6h**	0.00E+00	0.00E+00	0.00E+00	0.00E+00
**12h**	4.92E-09	3.46E-09	1.51E-08	1.14E-08
**24h**	3.19E-11	7.57E-11	9.62E-10	2.63E-09
**36h**	1.10E-09	3.06E-07	1.64E-10	1.17E-07
**48h**	1.91E-08	5.58E-08	1.23E-08	1.75E-08
**72h**	1.09E-07	1.00E-06	1.15E-07	1.69E-07
	cgd4_910			
**2h**	0.00E+00	5.12E-07	2.28E-07	0.00E+00
**6h**	0.00E+00	0.00E+00	0.00E+00	1.12E-05
**12h**	0.00E+00	0.00E+00	6.09E-07	4.04E-06
**24h**	0.00E+00	1.23E-08	8.32E-08	8.25E-07
**36h**	2.41E-07	8.91E-08	8.10E-08	4.61E-07
**48h**	1.83E-05	1.98E-05	1.28E-04	1.42E-04
**72h**	1.92E-05	7.61E-06	1.59E-05	4.53E-05
	cgd4_920			
**2h**	3.61E-06	0.00E+00	2.63E-05	3.87E-05
**6h**	1.54E-05	1.47E-04	2.95E-03	1.09E-03
**12h**	3.06E-05	1.05E-04	1.26E-04	4.51E-04
**24h**	2.66E-06	8.14E-06	5.72E-04	5.40E-05
**36h**	7.87E-06	4.27E-05	2.14E-04	4.08E-05
**48h**	9.70E-06	6.67E-06	2.03E-04	1.10E-04
**72h**	6.90E-06	3.56E-05	5.83E-05	1.24E-04
	cgd4_930			
**2h**	9.70E-09	1.02E-08	3.51E-07	5.65E-07
**6h**	0.00E+00	2.85E-08	4.10E-08	4.04E-06
**12h**	5.93E-08	5.32E-08	2.83E-07	2.12E-07
**24h**	7.05E-08	4.32E-09	8.71E-08	1.32E-07
**36h**	8.78E-08	2.34E-08	1.66E-08	1.90E-07

**48h**	3.63E-06	6.55E-08	1.23E-07	5.38E-07
**72h**	7.43E-07	2.27E-08	9.26E-08	1.26E-06
	cgd4_940			
**2h**	5.18E-08	5.41E-09	3.19E-08	2.61E-08
**6h**	4.26E-08	2.78E-08	9.30E-09	3.23E-07
**12h**	4.82E-08	2.50E-08	1.04E-07	2.12E-07
**24h**	2.88E-08	5.57E-09	4.46E-08	1.01E-07
**36h**	1.52E-08	4.22E-09	1.98E-08	6.68E-08
**48h**	1.10E-07	1.87E-08	9.08E-09	2.64E-07
**72h**	1.98E-08	1.13E-09	6.50E-09	1.22E-07
	cgd4_950			
**2h**	0.00E+00	0.00E+00	0.00E+00	0.00E+00
**6h**	2.22E-10	3.46E-09	1.12E-09	1.06E-08
**12h**	0.00E+00	0.00E+00	9.95E-10	0.00E+00
**24h**	4.84E-10	2.48E-10	3.45E-09	1.33E-09
**36h**	6.49E-10	2.32E-10	1.25E-09	1.27E-09
**48h**	1.01E-08	6.90E-10	1.09E-09	7.47E-09
**72h**	2.82E-09	2.00E-10	1.47E-09	2.96E-09
	cgd4_960			
**2h**	0.00E+00	0.00E+00	0.00E+00	0.00E+00
**6h**	1.32E-09	2.66E-10	2.07E-09	0.00E+00
**12h**	0.00E+00	0.00E+00	1.51E-09	0.00E+00
**24h**	1.28E-09	5.22E-11	2.36E-10	1.48E-10
**36h**	7.06E-11	5.03E-11	2.22E-10	1.92E-09
**48h**	5.08E-09	7.71E-10	7.07E-10	1.16E-09
**72h**	4.73E-09	8.71E-10	7.37E-10	3.44E-09
	cgd4_980			
**2h**	0.00E+00	0.00E+00	0.00E+00	0.00E+00
**6h**	0.00E+00	6.41E-11	1.10E-09	7.35E-10
**12h**	1.85E-09	1.59E-09	3.47E-09	1.33E-09
**24h**	5.47E-10	3.05E-10	9.01E-10	1.08E-09
**36h**	6.75E-09	4.66E-10	2.72E-09	2.41E-09
**48h**	3.80E-07	4.41E-08	5.93E-08	7.45E-08
**72h**	6.91E-08	1.04E-08	1.20E-08	5.56E-08
	cgd4_990			
**2h**	0.00E+00	0.00E+00	0.00E+00	0.00E+00
**6h**	0.00E+00	0.00E+00	0.00E+00	0.00E+00
**12h**	7.52E-05	1.09E-04	2.61E-05	4.22E-05
**24h**	3.69E-06	5.84E-07	1.25E-06	5.25E-06
**36h**	2.14E-07	3.98E-06	7.80E-08	1.74E-05
**48h**	3.55E-05	1.14E-05	6.79E-06	2.25E-05
**72h**	8.44E-06	3.33E-07	6.67E-08	5.03E-05
	cgd4_1050			
**2h**	0.00E+00	0.00E+00	0.00E+00	0.00E+00
**6h**	0.00E+00	0.00E+00	0.00E+00	0.00E+00
**12h**	0.00E+00	2.56E-11	0.00E+00	1.98E-09
**24h**	0.00E+00	0.00E+00	1.78E-10	0.00E+00
**36h**	5.14E-11	5.95E-11	2.50E-10	1.22E-11
**48h**	5.13E-10	1.39E-10	7.77E-11	4.88E-10
**72h**	7.19E-09	3.66E-09	2.76E-09	5.69E-09
	cgd4_1100			
**2h**	1.09E-05	2.77E-06	1.09E-07	3.15E-05
**6h**	0.00E+00	0.00E+00	0.00E+00	4.92E-06

**12h**	0.00E+00	4.83E-07	5.54E-08	0.00E+00
**24h**	4.64E-06	4.83E-08	2.05E-07	2.03E-06
**36h**	2.05E-05	7.57E-08	0.00E+00	6.79E-07
**48h**	7.15E-05	1.07E-04	2.23E-05	5.07E-04
**72h**	5.33E-04	7.29E-05	1.47E-05	5.64E-04
	cgd4_1110			
**2h**	0.00E+00	0.00E+00	0.00E+00	0.00E+00
**6h**	0.00E+00	0.00E+00	0.00E+00	0.00E+00
**12h**	0.00E+00	0.00E+00	0.00E+00	0.00E+00
**24h**	0.00E+00	0.00E+00	1.77E-09	0.00E+00
**36h**	8.76E-10	1.15E-10	2.77E-10	1.06E-10
**48h**	3.73E-06	3.49E-07	2.10E-07	1.51E-07
**72h**	7.27E-07	5.61E-08	2.29E-07	1.16E-06
	cgd4_1130			
**2h**	2.19E-09	0.00E+00	0.00E+00	0.00E+00
**6h**	5.08E-10	2.50E-09	3.35E-09	2.93E-09
**12h**	0.00E+00	9.75E-10	3.95E-09	1.32E-09
**24h**	7.64E-09	6.30E-10	5.23E-09	8.25E-09
**36h**	3.97E-08	4.44E-09	1.03E-08	3.41E-08
**48h**	6.13E-08	8.76E-09	3.75E-09	1.20E-08
**72h**	1.79E-07	9.27E-08	1.20E-08	3.16E-07
	cgd4_1140			
**2h**	0.00E+00	3.56E-10	0.00E+00	0.00E+00
**6h**	3.39E-09	4.17E-09	0.00E+00	4.73E-09
**12h**	0.00E+00	1.47E-09	6.39E-10	9.20E-10
**24h**	5.53E-09	1.57E-09	6.82E-09	7.54E-09
**36h**	1.04E-09	3.83E-09	1.92E-09	3.50E-08
**48h**	3.37E-08	1.29E-08	4.37E-08	2.00E-07
**72h**	5.82E-08	2.57E-08	8.09E-08	1.73E-07
	cgd4_1150			
**2h**	4.50E-07	5.03E-06	1.69E-04	7.78E-06
**6h**	6.54E-06	3.52E-05	7.72E-05	2.60E-05
**12h**	3.44E-07	7.11E-06	3.99E-05	4.56E-07
**24h**	3.10E-06	2.44E-06	1.31E-04	1.44E-06
**36h**	4.11E-06	4.09E-06	1.77E-05	1.56E-06
**48h**	1.02E-04	2.28E-05	6.85E-05	2.63E-05
**72h**	1.70E-04	9.83E-05	3.46E-04	2.56E-05
	cgd4_1160			
**2h**	1.49E-08	4.84E-08	4.13E-07	1.95E-07
**6h**	2.17E-08	2.76E-08	3.53E-08	1.43E-07
**12h**	5.06E-08	1.80E-08	2.58E-08	3.15E-07
**24h**	1.32E-08	5.16E-09	4.29E-08	4.42E-08
**36h**	2.09E-08	1.19E-08	3.81E-08	6.03E-08
**48h**	2.51E-07	2.30E-08	1.94E-07	2.22E-07
**72h**	1.52E-07	6.04E-08	2.33E-07	2.66E-07
	cgd4_1170			
**2h**	0.00E+00	0.00E+00	0.00E+00	0.00E+00
**6h**	2.40E-07	1.09E-06	2.73E-07	1.22E-06
**12h**	0.00E+00	8.50E-09	4.06E-07	8.47E-09
**24h**	6.73E-07	1.36E-07	1.75E-06	5.55E-08
**36h**	1.70E-06	6.32E-07	1.29E-07	4.21E-07
**48h**	2.67E-05	4.87E-06	2.02E-05	1.42E-06
**72h**	7.79E-06	2.98E-06	2.01E-05	8.35E-07

	cgd4_1210			
**2h**	2.06E-10	6.06E-09	1.01E-07	7.08E-08
**6h**	2.08E-09	4.13E-08	1.07E-08	1.28E-07
**12h**	0.00E+00	1.10E-08	7.03E-09	2.10E-08
**24h**	2.92E-09	9.18E-09	1.91E-07	2.56E-08
**36h**	4.94E-09	3.83E-09	5.69E-09	2.93E-08
**48h**	1.69E-07	7.70E-08	4.72E-08	1.74E-07
**72h**	6.03E-08	2.33E-08	7.98E-08	7.24E-08
	cgd4_1220			
**2h**	3.25E-05	2.53E-05	4.07E-05	4.43E-05
**6h**	6.29E-06	4.22E-06	3.96E-07	5.01E-07
**12h**	6.45E-05	2.68E-05	3.02E-05	8.04E-06
**24h**	5.96E-06	1.44E-06	2.99E-06	1.84E-06
**36h**	6.63E-06	2.40E-06	1.87E-06	3.77E-07
**48h**	1.73E-05	3.46E-06	8.08E-06	6.66E-06
**72h**	5.81E-06	5.94E-06	3.53E-06	3.54E-07
	cgd4_1240			
**2h**	2.23E-08	3.16E-08	1.78E-06	1.93E-07
**6h**	0.00E+00	4.24E-09	1.42E-07	3.87E-08
**12h**	2.48E-08	3.00E-08	1.79E-07	2.68E-09
**24h**	7.13E-09	4.12E-09	3.28E-07	1.28E-08
**36h**	2.38E-07	1.04E-07	3.06E-07	5.65E-08
**48h**	4.15E-06	3.32E-07	1.68E-06	7.24E-07
**72h**	8.86E-07	2.99E-07	1.53E-06	2.55E-07
	cgd4_1270			
**2h**	6.27E-09	7.51E-10	1.96E-07	9.72E-08
**6h**	0.00E+00	4.03E-09	0.00E+00	4.05E-08
**12h**	1.03E-08	2.47E-09	5.54E-09	1.02E-08
**24h**	5.09E-09	6.44E-09	6.30E-09	1.18E-08
**36h**	3.33E-09	3.37E-10	2.29E-09	5.18E-09
**48h**	1.14E-07	1.03E-08	1.32E-08	4.00E-08
**72h**	2.19E-08	2.12E-09	1.96E-11	6.05E-09
	cgd4_1280			
**2h**	1.58E-08	1.38E-08	6.27E-08	2.81E-08
**6h**	0.00E+00	9.89E-10	4.70E-09	1.44E-09
**12h**	0.00E+00	2.28E-09	2.25E-09	4.48E-09
**24h**	9.51E-10	1.07E-09	1.59E-10	2.28E-09
**36h**	3.27E-10	9.49E-10	7.75E-10	9.30E-10
**48h**	1.98E-08	1.17E-08	1.68E-08	1.35E-08
**72h**	8.96E-09	9.25E-09	1.75E-08	4.55E-09
	cgd4_1300			
**2h**	0.00E+00	4.80E-11	0.00E+00	0.00E+00
**6h**	0.00E+00	0.00E+00	0.00E+00	0.00E+00
**12h**	0.00E+00	6.97E-11	2.61E-10	0.00E+00
**24h**	1.75E-10	1.32E-10	1.19E-09	0.00E+00
**36h**	4.30E-11	2.77E-11	2.60E-10	3.90E-10
**48h**	4.28E-07	5.90E-08	2.38E-07	2.79E-07
**72h**	2.68E-09	2.13E-09	4.22E-09	8.36E-09
	cgd4_1310			
**2h**	0.00E+00	0.00E+00	0.00E+00	0.00E+00
**6h**	0.00E+00	0.00E+00	0.00E+00	0.00E+00
**12h**	0.00E+00	0.00E+00	0.00E+00	0.00E+00
**24h**	1.17E-10	0.00E+00	0.00E+00	1.92E-10

**36h**	0.00E+00	7.21E-11	0.00E+00	0.00E+00
**48h**	7.88E-09	3.19E-10	1.60E-09	1.37E-09
**72h**	7.87E-08	2.44E-08	2.22E-07	7.60E-08
	cgd4_180			
**2h**	0.00E+00	0.00E+00	0.00E+00	0.00E+00
**6h**	3.15E-05	1.98E-05	3.76E-05	2.75E-05
**12h**	1.06E-07	7.56E-06	2.43E-05	2.19E-06
**24h**	1.75E-06	1.27E-05	3.49E-06	5.92E-05
**36h**	5.88E-06	8.99E-06	9.91E-06	6.16E-05
**48h**	5.33E-06	1.41E-05	2.19E-06	7.05E-05
**72h**	2.30E-05	5.51E-05	7.07E-05	1.69E-05
	cgd2_1040			
**2h**	0.00E+00	5.11E-09	2.45E-07	1.65E-07
**6h**	2.80E-08	1.03E-07	1.45E-07	3.25E-07
**12h**	3.55E-08	8.29E-09	7.80E-08	1.04E-07
**24h**	1.92E-08	1.07E-08	1.39E-07	4.82E-08
**36h**	1.71E-08	4.64E-09	6.67E-09	4.54E-08
**48h**	7.88E-08	3.11E-08	2.26E-08	5.53E-08
**72h**	9.74E-09	3.23E-09	6.14E-09	1.74E-08
	cgd2_1080			
**2h**	1.25E-08	1.44E-08	1.81E-08	1.20E-08
**6h**	1.02E-08	3.48E-08	2.57E-09	5.49E-08
**12h**	7.54E-10	7.29E-09	1.87E-08	2.38E-08
**24h**	7.06E-09	1.22E-08	2.96E-08	1.29E-08
**36h**	4.81E-09	4.16E-09	3.91E-09	2.65E-08
**48h**	5.44E-08	9.79E-09	1.79E-08	1.24E-07
**72h**	2.42E-08	3.15E-09	7.30E-09	7.58E-08
	cgd2_1090			
**2h**	0.00E+00	0.00E+00	0.00E+00	0.00E+00
**6h**	4.91E-10	2.53E-09	1.08E-08	3.27E-08
**12h**	7.56E-10	1.95E-09	3.75E-09	3.46E-09
**24h**	7.14E-10	4.24E-10	1.09E-08	3.22E-09
**36h**	5.96E-09	5.93E-10	7.48E-09	6.88E-09
**48h**	9.32E-08	1.06E-08	9.64E-09	6.82E-08
**72h**	9.55E-09	1.70E-09	6.54E-09	3.66E-08
	cgd2_1120			
**2h**	3.03E-08	2.99E-08	6.25E-08	2.98E-07
**6h**	2.35E-08	1.84E-08	1.16E-08	1.01E-07
**12h**	5.80E-08	2.42E-08	6.43E-08	1.62E-07
**24h**	4.81E-08	8.65E-09	3.01E-08	1.07E-07
**36h**	2.22E-08	9.78E-09	8.46E-09	6.26E-08
**48h**	9.05E-08	3.26E-08	7.85E-09	3.19E-07
**72h**	2.82E-08	7.28E-09	3.59E-09	2.75E-08
	cgd2_1130			
**2h**	1.62E-09	0.00E+00	4.43E-09	9.82E-09
**6h**	6.36E-09	1.32E-08	1.74E-08	3.88E-08
**12h**	1.62E-08	2.24E-09	1.85E-09	1.11E-08
**24h**	8.81E-09	4.43E-09	7.28E-09	4.11E-08
**36h**	1.31E-08	1.38E-08	3.23E-09	1.46E-08
**48h**	6.81E-08	1.62E-08	5.79E-09	3.23E-07
**72h**	2.05E-08	9.55E-10	2.44E-10	6.18E-08
	cgd2_1230			
**2h**	0.00E+00	7.57E-09	1.11E-08	4.43E-09

**6h**	0.00E+00	3.52E-10	2.47E-09	0.00E+00
**12h**	0.00E+00	5.34E-10	0.00E+00	0.00E+00
**24h**	0.00E+00	2.97E-11	9.92E-10	2.03E-09
**36h**	7.26E-10	7.40E-10	1.52E-10	1.60E-09
**48h**	4.29E-07	8.08E-08	6.63E-08	1.41E-06
**72h**	1.20E-08	5.83E-08	7.80E-09	5.09E-07
	cgd2_1240			
**2h**	0.00E+00	0.00E+00	0.00E+00	0.00E+00
**6h**	0.00E+00	1.25E-06	4.59E-07	1.25E-05
**12h**	3.46E-08	6.67E-06	1.38E-08	7.36E-06
**24h**	9.37E-07	4.68E-06	4.89E-07	3.58E-06
**36h**	4.62E-06	2.14E-05	4.98E-07	1.01E-05
**48h**	4.12E-05	2.19E-05	1.40E-05	1.81E-04
**72h**	1.75E-05	2.64E-05	3.80E-07	1.19E-04
	cgd2_1260			
**2h**	0.00E+00	0.00E+00	0.00E+00	0.00E+00
**6h**	1.65E-08	1.40E-07	3.87E-08	1.05E-06
**12h**	3.02E-09	2.21E-10	0.00E+00	0.00E+00
**24h**	2.78E-08	1.69E-08	3.13E-08	1.05E-07
**36h**	4.12E-08	1.46E-08	4.75E-09	4.54E-07
**48h**	1.50E-07	2.92E-07	6.72E-09	2.87E-06
**72h**	1.17E-08	5.45E-09	3.34E-09	3.82E-07
	cgd2_1460			
**2h**	0.00E+00	1.22E-07	3.86E-08	1.19E-07
**6h**	4.06E-08	8.44E-08	6.33E-08	7.01E-07
**12h**	1.74E-09	1.15E-08	8.75E-09	1.61E-07
**24h**	2.20E-08	1.05E-08	9.71E-08	7.02E-08
**36h**	3.15E-08	1.67E-08	1.17E-08	1.90E-07
**48h**	2.30E-07	3.40E-08	3.63E-08	3.29E-07
**72h**	2.09E-08	2.05E-08	8.84E-09	3.88E-07
	cgd2_1570			
**2h**	7.73E-08	2.90E-09	8.92E-09	8.79E-09
**6h**	2.13E-08	1.99E-08	9.84E-08	8.48E-08
**12h**	1.51E-09	5.45E-09	4.28E-09	5.51E-09
**24h**	2.34E-08	9.39E-09	3.14E-08	5.71E-08
**36h**	2.76E-08	3.83E-09	6.43E-09	4.53E-08
**48h**	5.04E-08	3.47E-08	4.11E-08	4.67E-08
**72h**	2.37E-08	5.11E-09	1.21E-08	3.29E-08
	cgd2_1580			
**2h**	1.72E-07	3.33E-08	1.06E-06	1.31E-06
**6h**	1.19E-06	4.01E-08	8.92E-08	2.02E-07
**12h**	1.09E-07	1.04E-07	5.14E-08	1.59E-07
**24h**	3.35E-07	6.46E-08	2.05E-07	1.83E-07
**36h**	2.32E-07	4.50E-08	1.85E-08	1.25E-07
**48h**	2.60E-07	1.22E-07	1.11E-07	1.84E-07
**72h**	2.78E-07	9.71E-09	7.80E-08	3.03E-07
	cgd2_1720			
**2h**	2.04E-08	2.82E-09	3.60E-09	4.47E-08
**6h**	1.34E-08	3.89E-09	1.56E-08	2.56E-08
**12h**	6.44E-09	5.37E-10	1.02E-09	8.42E-09
**24h**	3.46E-09	1.12E-09	3.36E-09	1.41E-08
**36h**	2.21E-08	2.31E-09	1.05E-08	4.92E-08
**48h**	2.14E-07	2.22E-08	1.74E-08	1.15E-07

**72h**	4.35E-08	3.24E-09	1.28E-08	1.74E-08
	cgd2_1770			
**2h**	0.00E+00	1.08E-09	0.00E+00	4.16E-09
**6h**	2.77E-08	2.07E-08	1.61E-07	2.40E-07
**12h**	9.97E-09	9.04E-10	4.06E-09	4.48E-09
**24h**	4.24E-08	3.24E-09	1.71E-08	1.47E-07
**36h**	4.33E-08	8.44E-09	5.34E-08	7.19E-08
**48h**	8.00E-08	1.63E-08	1.46E-08	6.40E-08
**72h**	2.22E-08	3.14E-09	7.03E-09	2.39E-08
	cgd2_1930			
**2h**	7.78E-09	0.00E+00	2.36E-07	0.00E+00
**6h**	3.41E-07	3.45E-07	7.78E-07	3.18E-03
**12h**	7.34E-09	5.12E-08	4.72E-08	1.86E-04
**24h**	4.36E-08	1.65E-07	6.25E-07	3.11E-03
**36h**	2.66E-08	4.75E-07	8.02E-07	1.85E-03
**48h**	6.22E-08	1.99E-07	5.08E-07	2.87E-04
**72h**	8.76E-09	5.94E-07	3.36E-07	4.60E-04
	cgd2_2010			
**2h**	0.00E+00	6.43E-09	4.31E-08	1.61E-08
**6h**	2.20E-08	3.82E-09	8.45E-09	2.53E-08
**12h**	1.73E-08	8.67E-09	1.33E-08	1.29E-08
**24h**	2.92E-08	4.17E-09	1.76E-08	6.27E-08
**36h**	4.89E-08	1.51E-08	3.59E-08	9.52E-08
**48h**	1.27E-07	4.14E-08	2.51E-08	9.79E-08
**72h**	1.78E-08	1.18E-09	7.25E-09	1.63E-08
	cgd2_2050			
**2h**	1.02E-07	1.63E-07	3.39E-07	3.27E-08
**6h**	8.01E-06	5.53E-06	2.88E-05	2.34E-05
**12h**	4.01E-07	1.60E-07	4.39E-07	8.53E-07
**24h**	5.82E-06	2.17E-06	7.76E-06	1.60E-05
**36h**	2.46E-05	9.62E-07	1.38E-05	1.36E-05
**48h**	2.39E-05	2.45E-06	1.46E-05	1.52E-05
**72h**	1.14E-06	1.82E-07	7.28E-07	2.10E-06
	cgd2_2070			
**2h**	1.23E-08	4.70E-09	6.39E-07	1.10E-07
**6h**	1.35E-08	7.15E-09	1.32E-08	2.28E-08
**12h**	3.30E-09	1.78E-09	1.94E-08	5.33E-08
**24h**	7.67E-09	1.23E-09	1.48E-08	4.10E-08
**36h**	2.41E-08	1.91E-09	3.95E-09	6.34E-08
**48h**	4.88E-08	7.29E-09	8.17E-09	3.42E-08
**72h**	1.21E-08	1.19E-09	4.54E-08	4.68E-08
	cgd2_2110			
**2h**	0.00E+00	0.00E+00	0.00E+00	2.96E-03
**6h**	0.00E+00	0.00E+00	0.00E+00	0.00E+00
**12h**	0.00E+00	0.00E+00	4.36E-08	2.50E-07
**24h**	0.00E+00	1.48E-08	1.69E-09	0.00E+00
**36h**	3.42E-10	1.44E-09	0.00E+00	7.51E-09
**48h**	3.81E-07	2.33E-07	2.68E-07	7.15E-07
**72h**	2.80E-05	1.71E-05	1.13E-05	4.15E-05
	cgd2_2240			
**2h**	5.81E-09	2.73E-08	1.05E-07	3.63E-07
**6h**	3.61E-09	7.91E-09	7.05E-09	3.83E-08
**12h**	1.16E-08	3.89E-08	4.56E-08	8.97E-08

**24h**	5.57E-08	3.71E-09	3.13E-08	6.69E-08
**36h**	2.36E-08	6.15E-09	1.11E-08	6.43E-08
**48h**	2.91E-07	5.64E-08	1.02E-07	1.38E-07
**72h**	2.39E-08	1.53E-08	8.70E-08	6.45E-08
	cgd2_2550			
**2h**	0.00E+00	0.00E+00	7.18E-07	0.00E+00
**6h**	1.24E-07	4.11E-08	2.47E-07	5.56E-07
**12h**	1.36E-05	1.28E-07	1.73E-06	4.20E-06
**24h**	6.01E-08	7.74E-08	1.10E-06	5.09E-06
**36h**	1.12E-06	1.10E-06	2.48E-06	1.13E-04
**48h**	2.12E-05	4.33E-06	2.02E-05	3.14E-05
**72h**	7.03E-05	4.36E-07	6.39E-06	1.96E-05
	cgd2_2910			
**2h**	4.03E-08	4.55E-07	1.20E-06	6.28E-06
**6h**	1.37E-08	4.00E-08	2.35E-08	9.44E-08
**12h**	1.57E-07	2.61E-07	1.02E-06	1.81E-06
**24h**	1.08E-08	2.47E-08	2.41E-07	2.54E-07
**36h**	6.11E-08	6.23E-08	9.28E-08	7.69E-07
**48h**	4.27E-07	1.28E-07	1.92E-07	5.65E-07
**72h**	2.93E-07	4.17E-08	1.96E-07	5.93E-07
	cgd2_2920			
**2h**	1.37E-09	5.32E-09	1.79E-08	2.22E-09
**6h**	0.00E+00	0.00E+00	0.00E+00	2.20E-10
**12h**	4.18E-09	3.80E-09	8.21E-09	1.98E-08
**24h**	9.50E-10	5.29E-11	3.86E-09	4.84E-09
**36h**	1.75E-09	8.86E-10	3.55E-09	2.31E-08
**48h**	1.22E-07	6.42E-08	1.35E-07	1.81E-07
**72h**	8.93E-07	2.26E-07	1.02E-06	1.76E-06
	cgd2_3100			
**2h**	8.46E-09	7.80E-09	4.32E-08	5.43E-08
**6h**	2.25E-08	1.30E-08	8.49E-08	1.13E-07
**12h**	6.18E-09	3.29E-09	6.48E-09	1.66E-08
**24h**	7.46E-09	7.41E-09	2.95E-08	4.66E-08
**36h**	2.72E-08	6.13E-09	1.81E-08	1.13E-07
**48h**	6.91E-08	2.70E-08	5.51E-08	1.58E-07
**72h**	5.69E-07	1.56E-07	7.56E-07	7.75E-07
	cgd2_3110			
**2h**	3.64E-06	2.14E-04	7.85E-04	1.49E-03
**6h**	2.40E-06	4.34E-05	4.41E-04	6.75E-04
**12h**	3.00E-06	2.94E-05	1.17E-03	8.16E-04
**24h**	2.92E-06	6.62E-06	3.04E-04	3.54E-04
**36h**	1.21E-05	1.19E-05	2.18E-05	4.33E-04
**48h**	4.56E-04	8.52E-05	6.19E-04	4.58E-04
**72h**	8.48E-06	1.59E-06	2.03E-05	4.12E-05
	cgd2_3270			
**2h**	2.16E-06	8.03E-09	4.94E-08	1.98E-07
**6h**	1.72E-04	1.35E-07	1.40E-07	3.45E-07
**12h**	0.00E+00	2.27E-04	1.51E-03	1.43E-03
**24h**	0.00E+00	1.54E-05	4.30E-07	4.20E-07
**36h**	2.50E-07	9.76E-08	3.00E-07	2.31E-06
**48h**	3.94E-06	9.52E-07	1.63E-06	2.52E-06
**72h**	1.26E-07	2.60E-08	3.48E-07	3.34E-07
	cgd2_3290			

**2h**	2.67E-06	3.65E-05	1.75E-04	3.41E-04
**6h**	3.42E-06	2.77E-05	3.79E-05	2.02E-04
**12h**	6.12E-06	2.15E-05	8.46E-05	1.96E-04
**24h**	6.59E-06	8.15E-06	1.39E-04	2.89E-04
**36h**	1.11E-05	6.10E-06	4.10E-06	4.12E-05
**48h**	5.91E-05	3.30E-05	4.15E-05	6.96E-05
**72h**	6.48E-04	3.90E-04	7.40E-05	2.12E-05
	cgd2_3310			
**2h**	0.00E+00	0.00E+00	0.00E+00	0.00E+00
**6h**	7.41E-05	0.00E+00	9.06E-08	0.00E+00
**12h**	0.00E+00	2.86E-05	0.00E+00	3.22E-04
**24h**	6.20E-07	1.48E-11	1.14E-04	1.61E-08
**36h**	1.81E-09	1.14E-09	1.07E-09	3.46E-09
**48h**	4.84E-08	2.73E-05	7.07E-07	3.20E-07
**72h**	1.02E-06	4.19E-06	5.97E-08	5.73E-08
	cgd2_3320			
**2h**	0.00E+00	0.00E+00	2.19E-08	8.15E-08
**6h**	0.00E+00	1.36E-06	2.73E-06	2.77E-06
**12h**	0.00E+00	2.18E-09	9.55E-08	6.14E-08
**24h**	9.47E-09	4.00E-09	1.35E-06	1.66E-06
**36h**	7.37E-09	4.50E-09	1.99E-08	1.89E-06
**48h**	2.89E-06	3.07E-07	4.09E-06	1.71E-06
**72h**	2.71E-07	6.09E-08	2.23E-06	2.17E-06
	cgd2_3350			
**2h**	0.00E+00	0.00E+00	0.00E+00	0.00E+00
**6h**	0.00E+00	0.00E+00	2.66E-08	0.00E+00
**12h**	9.30E-08	1.25E-07	2.96E-07	1.65E-07
**24h**	9.93E-08	7.97E-08	5.64E-07	2.67E-07
**36h**	5.01E-07	4.33E-07	6.07E-07	2.66E-06
**48h**	2.46E-06	4.09E-07	1.08E-06	2.14E-06
**72h**	1.40E-07	3.39E-08	1.01E-07	4.55E-07
	cgd2_3380			
**2h**	1.49E-07	5.11E-06	7.54E-05	3.61E-04
**6h**	5.73E-07	2.56E-05	3.89E-05	1.15E-04
**12h**	5.01E-08	2.88E-06	6.01E-06	1.84E-05
**24h**	6.55E-07	2.76E-06	5.87E-05	3.56E-05
**36h**	1.06E-06	3.60E-06	5.01E-06	4.68E-05
**48h**	4.62E-05	6.99E-06	2.97E-05	2.80E-05
**72h**	4.68E-06	9.14E-07	1.66E-05	2.02E-05
	cgd2_3420			
**2h**	8.44E-06	8.48E-06	8.48E-06	2.77E-05
**6h**	8.80E-07	1.10E-06	1.91E-07	6.79E-07
**12h**	1.98E-06	3.72E-06	2.46E-06	4.23E-06
**24h**	5.70E-07	1.10E-06	1.69E-06	1.07E-06
**36h**	6.66E-07	8.18E-07	5.63E-07	1.54E-06
**48h**	1.02E-06	2.25E-07	5.20E-07	6.02E-07
**72h**	4.91E-07	8.56E-08	7.49E-07	1.48E-06
	cgd2_3490			
**2h**	0.00E+00	0.00E+00	2.30E-06	6.07E-07
**6h**	1.11E-06	1.37E-07	5.47E-06	5.41E-06
**12h**	0.00E+00	1.44E-07	7.59E-07	5.50E-07
**24h**	1.69E-08	1.84E-07	2.58E-06	1.06E-06
**36h**	5.46E-08	1.71E-07	1.25E-07	8.04E-06

**48h**	1.33E-04	7.80E-06	6.98E-05	6.11E-05
**72h**	3.91E-05	2.26E-06	1.59E-04	1.35E-04
	cgd2_3700			
**2h**	0.00E+00	0.00E+00	0.00E+00	7.56E-09
**6h**	1.54E-08	3.28E-09	1.01E-07	3.41E-08
**12h**	0.00E+00	4.96E-10	1.29E-09	8.78E-09
**24h**	1.88E-10	8.60E-11	3.58E-08	7.89E-09
**36h**	1.22E-08	1.17E-08	1.44E-09	1.19E-07
**48h**	5.01E-08	1.52E-08	8.96E-09	4.87E-08
**72h**	4.80E-09	2.53E-09	1.01E-08	3.05E-08
	cgd2_3710			
**2h**	0.00E+00	0.00E+00	0.00E+00	0.00E+00
**6h**	0.00E+00	0.00E+00	0.00E+00	0.00E+00
**12h**	8.07E-09	4.90E-09	4.15E-09	4.79E-09
**24h**	3.11E-10	8.62E-11	5.73E-09	1.49E-09
**36h**	5.24E-07	2.48E-09	3.24E-09	2.29E-08
**48h**	1.46E-08	1.97E-09	5.77E-09	1.70E-08
**72h**	1.35E-08	9.00E-10	3.83E-08	8.20E-08
	cgd2_3730			
**2h**	3.28E-04	1.61E-03	0.00E+00	2.14E-03
**6h**	1.21E-04	6.99E-04	3.61E-04	6.88E-04
**12h**	1.69E-04	1.23E-03	1.05E-03	1.53E-03
**24h**	9.99E-05	1.57E-04	4.50E-04	3.16E-04
**36h**	3.55E-04	2.74E-04	2.51E-04	6.77E-04
**48h**	2.72E-04	1.92E-04	7.62E-04	6.29E-04
**72h**	5.97E-05	2.64E-05	4.14E-05	1.82E-04
	cgd2_3920			
**2h**	0.00E+00	5.37E-08	2.89E-07	1.80E-07
**6h**	6.00E-08	1.18E-06	4.65E-05	2.24E-05
**12h**	0.00E+00	4.30E-08	2.55E-06	6.72E-07
**24h**	1.40E-07	6.15E-08	3.85E-05	1.26E-05
**36h**	2.97E-08	2.50E-07	1.24E-07	7.09E-06
**48h**	9.61E-06	8.70E-07	9.69E-06	4.82E-06
**72h**	2.29E-07	3.79E-08	1.41E-06	1.69E-06
	cgd2_4020			
**2h**	6.66E-05	1.95E-04	4.75E-04	3.96E-04
**6h**	1.95E-05	1.96E-05	2.70E-05	6.75E-05
**12h**	9.80E-05	1.08E-04	2.08E-05	1.25E-04
**24h**	4.13E-05	6.93E-05	7.98E-05	1.36E-04
**36h**	2.61E-05	4.46E-05	2.81E-05	6.57E-05
**48h**	1.57E-04	4.26E-05	4.39E-05	3.06E-04
**72h**	6.00E-06	1.03E-05	2.44E-06	1.82E-05
	cgd2_4040			
**2h**	1.66E-06	9.03E-06	1.85E-05	1.30E-05
**6h**	0.00E+00	1.91E-07	1.68E-08	1.52E-07
**12h**	2.91E-07	2.07E-06	2.04E-05	1.46E-05
**24h**	9.04E-08	6.07E-07	8.94E-06	7.25E-06
**36h**	4.19E-06	1.67E-06	3.52E-06	3.27E-05
**48h**	8.76E-06	4.22E-06	7.33E-06	4.16E-05
**72h**	8.39E-06	5.27E-06	1.71E-05	4.17E-05
	cgd2_4080			
**2h**	0.00E+00	1.55E-08	0.00E+00	0.00E+00
**6h**	6.23E-09	1.44E-08	1.35E-07	1.50E-07

**12h**	1.52E-08	3.90E-10	2.18E-09	2.37E-09
**24h**	6.27E-10	1.59E-09	6.37E-08	6.32E-08
**36h**	5.31E-09	1.01E-08	5.83E-09	2.14E-07
**48h**	1.33E-07	4.01E-08	1.21E-07	2.75E-07
**72h**	3.88E-08	1.23E-08	2.59E-07	1.99E-07
	cgd2_4370			
**2h**	1.36E-05	3.55E-06	1.23E-05	9.00E-05
**6h**	5.55E-07	1.25E-06	1.05E-07	3.00E-06
**12h**	3.31E-06	1.22E-05	8.76E-06	2.39E-05
**24h**	8.12E-07	1.76E-07	2.40E-06	4.72E-06
**36h**	2.47E-06	8.12E-07	1.57E-06	3.70E-06
**48h**	9.52E-06	1.69E-06	4.04E-06	1.05E-05
**72h**	9.33E-07	2.23E-07	5.11E-07	2.59E-06
	cgd3_120			
**2h**	1.48E-08	0.00E+00	4.64E-09	3.41E-09
**6h**	0.00E+00	4.68E-09	4.15E-09	1.07E-08
**12h**	0.00E+00	3.21E-10	1.64E-09	5.82E-10
**24h**	1.17E-08	6.93E-10	3.85E-09	9.55E-09
**36h**	1.22E-09	1.04E-09	9.72E-09	9.15E-09
**48h**	2.79E-08	1.53E-08	8.83E-09	2.52E-08
**72h**	9.93E-09	1.35E-09	1.08E-08	1.98E-08
	cgd3_130			
**2h**	0.00E+00	1.52E-09	5.87E-08	1.44E-07
**6h**	0.00E+00	2.39E-08	3.27E-08	1.93E-07
**12h**	0.00E+00	3.43E-10	4.64E-08	3.49E-08
**24h**	6.46E-09	1.56E-09	1.85E-08	2.74E-08
**36h**	3.26E-09	3.42E-09	1.43E-09	3.76E-08
**48h**	9.77E-08	1.87E-08	3.09E-08	2.38E-08
**72h**	2.06E-08	3.18E-09	1.19E-08	5.95E-08
	cgd3_170			
**2h**	2.74E-08	3.00E-08	7.34E-08	7.07E-08
**6h**	5.74E-08	5.77E-09	6.23E-09	2.03E-08
**12h**	6.91E-09	1.18E-08	2.25E-08	7.46E-08
**24h**	1.04E-08	5.02E-09	6.62E-09	1.58E-08
**36h**	1.06E-08	3.63E-09	1.77E-08	1.93E-08
**48h**	2.40E-08	4.23E-09	6.71E-09	1.15E-08
**72h**	5.11E-09	2.03E-09	2.48E-08	2.42E-08
	cgd3_200			
**2h**	3.14E-08	5.88E-08	2.24E-06	4.42E-06
**6h**	5.55E-08	1.86E-07	8.54E-07	9.21E-07
**12h**	3.17E-08	5.15E-08	1.85E-06	1.42E-06
**24h**	5.08E-08	3.65E-08	8.24E-07	4.95E-07
**36h**	5.22E-08	4.88E-08	7.29E-08	7.04E-07
**48h**	8.86E-07	5.74E-08	5.27E-07	3.74E-07
**72h**	7.56E-08	2.71E-08	1.85E-07	3.66E-07
	cgd3_230			
**2h**	0.00E+00	0.00E+00	0.00E+00	0.00E+00
**6h**	0.00E+00	0.00E+00	0.00E+00	0.00E+00
**12h**	5.02E-08	6.93E-08	3.29E-07	8.08E-07
**24h**	2.35E-09	8.30E-10	1.71E-07	5.12E-07
**36h**	9.05E-07	2.20E-07	6.57E-07	1.41E-05
**48h**	1.44E-05	2.06E-06	9.07E-06	1.10E-05
**72h**	1.37E-05	5.25E-06	5.18E-05	6.59E-05

	cgd3_320			
**2h**	0.00E+00	0.00E+00	0.00E+00	0.00E+00
**6h**	5.81E-06	1.06E-05	2.46E-05	5.01E-04
**12h**	6.65E-08	1.06E-06	2.02E-06	4.50E-06
**24h**	1.42E-05	4.95E-06	8.94E-06	7.89E-05
**36h**	5.61E-05	1.88E-05	6.39E-05	1.05E-04
**48h**	2.28E-05	8.09E-06	6.40E-05	4.24E-05
**72h**	5.95E-06	3.75E-06	2.47E-05	4.26E-05
	cgd3_490			
**2h**	6.61E-09	2.23E-08	2.44E-07	1.88E-07
**6h**	5.27E-10	1.13E-09	9.27E-09	3.95E-09
**12h**	1.75E-07	2.26E-07	2.51E-07	2.82E-07
**24h**	8.26E-08	7.19E-08	1.79E-07	1.02E-07
**36h**	1.04E-06	5.55E-07	8.15E-07	1.76E-06
**48h**	1.14E-06	3.13E-07	7.20E-07	9.21E-07
**72h**	3.61E-08	2.09E-08	1.23E-08	8.95E-08
	cgd3_540			
**2h**	3.65E-09	1.78E-08	2.56E-08	1.13E-07
**6h**	3.76E-09	3.88E-09	7.73E-09	3.40E-08
**12h**	5.51E-09	2.02E-08	5.18E-08	3.98E-08
**24h**	2.22E-09	3.50E-09	3.43E-08	2.57E-08
**36h**	8.98E-09	1.22E-08	1.57E-08	2.72E-08
**48h**	7.32E-08	1.11E-08	4.73E-08	6.13E-08
**72h**	9.25E-10	1.87E-09	3.57E-09	9.71E-09
	cgd3_820			
**2h**	0.00E+00	0.00E+00	0.00E+00	0.00E+00
**6h**	8.74E-10	5.80E-10	5.49E-09	8.40E-10
**12h**	1.79E-08	2.08E-08	1.07E-08	2.11E-08
**24h**	3.05E-08	1.45E-08	9.39E-09	8.05E-09
**36h**	1.15E-07	6.69E-08	5.10E-08	1.19E-07
**48h**	3.53E-08	1.03E-08	1.60E-08	1.23E-07
**72h**	5.10E-09	8.89E-09	1.44E-09	7.45E-09
	cgd3_830			
**2h**	2.43E-07	1.74E-07	1.22E-07	8.28E-07
**6h**	1.71E-06	3.55E-07	5.46E-07	7.29E-07
**12h**	4.52E-06	8.28E-07	1.24E-07	1.52E-06
**24h**	1.03E-06	2.55E-07	7.29E-07	8.33E-07
**36h**	2.57E-06	1.27E-06	4.14E-07	7.40E-07
**48h**	5.34E-07	1.37E-07	1.84E-07	3.03E-07
**72h**	1.45E-07	5.73E-08	4.07E-08	5.28E-08
	cgd3_840			
**2h**	0.00E+00	2.63E-08	2.59E-08	1.24E-07
**6h**	6.21E-08	7.02E-08	1.43E-07	2.31E-07
**12h**	1.90E-09	7.15E-09	3.89E-08	7.45E-08
**24h**	5.77E-08	2.00E-08	1.36E-07	1.13E-07
**36h**	1.74E-08	1.58E-08	2.03E-08	2.13E-07
**48h**	3.17E-07	6.63E-08	1.92E-07	9.46E-08
**72h**	2.92E-08	2.18E-09	9.00E-09	6.60E-08
	cgd3_870			
**2h**	0.00E+00	0.00E+00	3.68E-08	1.17E-06
**6h**	1.85E-07	8.25E-08	1.23E-06	8.30E-07
**12h**	0.00E+00	1.14E-08	2.74E-07	1.01E-08
**24h**	2.99E-08	2.67E-07	6.76E-07	3.37E-06

**36h**	3.85E-07	8.33E-08	1.79E-07	3.40E-06
**48h**	8.85E-06	5.59E-07	1.86E-06	1.60E-06
**72h**	9.27E-08	1.12E-07	2.05E-06	3.39E-06
	cgd3_910			
**2h**	0.00E+00	0.00E+00	0.00E+00	0.00E+00
**6h**	0.00E+00	0.00E+00	0.00E+00	0.00E+00
**12h**	2.02E-06	4.16E-06	2.32E-06	2.88E-06
**24h**	2.55E-07	2.22E-07	4.12E-07	4.70E-07
**36h**	2.95E-06	5.62E-07	2.97E-06	7.49E-06
**48h**	5.17E-06	2.33E-07	9.86E-07	1.23E-06
**72h**	3.53E-07	2.81E-07	3.39E-07	8.35E-07
	cgd3_920			
**2h**	2.33E-09	1.20E-09	0.00E+00	0.00E+00
**6h**	0.00E+00	4.00E-10	0.00E+00	0.00E+00
**12h**	2.78E-07	5.24E-07	1.43E-06	2.25E-06
**24h**	1.72E-08	5.16E-09	1.34E-07	7.27E-08
**36h**	4.70E-08	6.71E-08	2.43E-08	5.07E-07
**48h**	2.19E-07	3.77E-08	2.41E-07	2.17E-07
**72h**	1.61E-07	3.85E-08	1.59E-07	3.62E-07
	cgd3_990			
**2h**	6.88E-09	3.73E-09	1.88E-08	1.96E-08
**6h**	8.38E-09	2.94E-08	2.59E-07	1.41E-07
**12h**	5.24E-08	1.92E-09	6.60E-09	0.00E+00
**24h**	3.85E-09	5.26E-09	7.74E-08	4.38E-08
**36h**	1.85E-08	1.79E-08	2.21E-08	1.06E-07
**48h**	6.00E-08	1.36E-08	3.31E-08	4.51E-08
**72h**	1.96E-08	4.51E-09	2.02E-08	5.47E-08
	cgd3_1040			
**2h**	0.00E+00	0.00E+00	0.00E+00	0.00E+00
**6h**	0.00E+00	0.00E+00	0.00E+00	0.00E+00
**12h**	6.36E-09	6.66E-09	3.41E-09	3.85E-10
**24h**	5.63E-10	6.54E-10	6.45E-10	5.85E-10
**36h**	2.94E-09	2.77E-09	1.84E-09	1.05E-08
**48h**	4.94E-09	7.19E-10	7.81E-10	4.38E-09
**72h**	2.13E-08	8.29E-09	5.89E-08	3.34E-08
	cgd3_1110			
**2h**	0.00E+00	1.68E-09	2.14E-09	1.47E-09
**6h**	1.13E-09	0.00E+00	2.22E-09	0.00E+00
**12h**	4.49E-06	4.08E-08	1.91E-07	2.19E-07
**24h**	9.83E-08	4.91E-08	3.45E-07	2.43E-06
**36h**	7.03E-07	4.55E-07	9.87E-07	6.28E-07
**48h**	2.00E-06	1.78E-07	3.78E-07	1.17E-06
**72h**	9.02E-07	3.81E-07	8.21E-07	7.73E-07
	cgd3_1050			
**2h**	0.00E+00	0.00E+00	0.00E+00	0.00E+00
**6h**	0.00E+00	1.71E-08	4.25E-07	2.34E-07
**12h**	0.00E+00	1.21E-10	2.60E-09	1.29E-10
**24h**	4.43E-06	2.94E-09	1.51E-07	1.60E-07
**36h**	9.63E-09	1.09E-08	2.46E-08	4.47E-07
**48h**	2.73E-07	4.51E-08	7.66E-08	3.05E-07
**72h**	1.51E-07	3.50E-08	3.32E-07	1.61E-07
	cgd3_1450			
**2h**	0.00E+00	1.63E-09	4.69E-09	8.07E-08

**6h**	2.12E-08	2.92E-08	1.08E-07	9.03E-08
**12h**	0.00E+00	7.77E-09	2.82E-09	4.30E-09
**24h**	5.10E-08	8.50E-09	1.51E-08	1.12E-07
**36h**	7.69E-08	3.38E-08	2.68E-08	1.22E-07
**48h**	1.39E-07	4.66E-08	4.30E-08	1.03E-07
**72h**	3.74E-08	3.94E-09	4.59E-08	2.67E-08
	cgd3_1460			
**2h**	1.49E-07	1.27E-07	3.08E-07	1.06E-06
**6h**	9.28E-09	2.27E-08	2.51E-07	3.00E-07
**12h**	1.77E-08	3.09E-08	1.29E-07	1.06E-07
**24h**	4.94E-08	3.60E-08	1.07E-07	2.99E-07
**36h**	3.70E-08	2.48E-08	4.07E-08	2.63E-07
**48h**	8.45E-07	1.99E-07	4.57E-07	6.82E-07
**72h**	1.45E-07	4.15E-08	5.34E-07	1.85E-07
	cgd3_1470			
**2h**	0.00E+00	4.58E-09	3.27E-09	6.32E-08
**6h**	7.21E-09	8.30E-09	4.71E-08	5.99E-08
**12h**	6.72E-09	6.41E-09	1.47E-08	3.46E-08
**24h**	6.05E-09	6.54E-09	2.98E-08	4.24E-08
**36h**	2.40E-08	1.52E-08	2.82E-08	7.95E-08
**48h**	2.96E-07	6.54E-08	6.86E-08	1.32E-07
**72h**	4.75E-08	7.92E-09	1.37E-07	5.61E-08
	cgd3_1480			
**2h**	0.00E+00	2.26E-09	0.00E+00	0.00E+00
**6h**	0.00E+00	0.00E+00	1.34E-08	2.50E-08
**12h**	0.00E+00	1.77E-09	2.99E-09	2.27E-09
**24h**	1.29E-09	5.93E-10	6.87E-09	1.40E-08
**36h**	1.62E-08	6.99E-09	6.02E-08	1.20E-07
**48h**	1.62E-07	3.49E-08	9.56E-08	7.60E-08
**72h**	4.16E-08	1.00E-08	6.21E-08	6.29E-08
	cgd3_1500			
**2h**	6.89E-09	3.60E-09	3.74E-08	1.19E-07
**6h**	6.04E-08	2.64E-08	1.57E-07	4.08E-07
**12h**	7.12E-09	2.71E-09	6.38E-08	6.45E-08
**24h**	1.12E-08	7.00E-09	1.20E-07	7.23E-08
**36h**	9.47E-09	5.99E-09	2.56E-08	2.07E-07
**48h**	4.59E-07	5.07E-08	6.26E-08	1.05E-07
**72h**	2.74E-08	8.34E-10	8.04E-08	7.57E-08
	cgd3_1550			
**2h**	4.32E-08	1.47E-07	4.61E-08	2.39E-07
**6h**	6.31E-09	5.36E-08	5.65E-08	2.67E-07
**12h**	3.32E-09	1.61E-08	4.98E-08	1.29E-07
**24h**	3.64E-08	7.36E-08	7.43E-08	8.05E-08
**36h**	3.95E-08	2.47E-08	1.16E-07	2.93E-07
**48h**	6.15E-07	1.43E-07	2.61E-07	5.08E-07
**72h**	9.67E-07	1.63E-07	4.09E-06	3.87E-06
	cgd3_1580			
**2h**	0.00E+00	0.00E+00	0.00E+00	0.00E+00
**6h**	0.00E+00	2.16E-05	5.12E-05	8.28E-05
**12h**	6.06E-05	7.25E-06	2.83E-05	8.33E-06
**24h**	2.49E-05	7.99E-06	9.07E-05	6.10E-05
**36h**	7.42E-06	1.37E-05	7.79E-05	3.28E-05
**48h**	1.07E-04	5.41E-05	5.21E-04	1.67E-04

**72h**	2.60E-04	2.68E-05	1.58E-04	2.30E-04
	cgd3_1600			
**2h**	1.53E-08	3.14E-08	2.05E-07	2.27E-07
**6h**	9.76E-08	1.23E-06	1.12E-06	2.75E-06
**12h**	6.08E-09	1.52E-08	2.14E-07	2.54E-06
**24h**	1.14E-07	1.42E-07	9.74E-07	1.11E-07
**36h**	3.08E-07	1.05E-07	6.51E-07	2.49E-06
**48h**	1.60E-06	3.10E-07	1.31E-06	7.96E-07
**72h**	2.58E-07	2.15E-08	2.30E-07	5.51E-07
	cgd3_1610			
**2h**	1.21E-07	2.73E-08	3.55E-07	2.24E-06
**6h**	7.25E-10	4.54E-08	1.99E-08	4.31E-08
**12h**	9.02E-09	2.78E-08	3.26E-07	8.43E-08
**24h**	1.08E-08	9.64E-09	7.24E-08	1.46E-07
**36h**	2.98E-08	7.11E-09	2.45E-08	1.05E-07
**48h**	1.93E-07	1.87E-08	1.15E-07	1.39E-07
**72h**	6.38E-08	1.10E-08	3.32E-08	7.19E-08
	cgd3_1620			
**2h**	6.89E-07	5.69E-07	6.24E-06	7.96E-06
**6h**	5.21E-08	5.02E-07	7.31E-07	6.92E-07
**12h**	9.68E-09	2.35E-07	1.71E-06	1.01E-06
**24h**	1.05E-07	1.25E-07	9.15E-07	1.46E-06
**36h**	2.93E-07	1.01E-07	1.30E-06	1.20E-06
**48h**	1.75E-06	1.49E-07	1.17E-06	1.77E-06
**72h**	2.60E-06	3.30E-07	1.38E-06	1.81E-06
	cgd3_1630			
**2h**	3.20E-07	1.38E-07	3.60E-07	1.15E-06
**6h**	3.18E-07	4.74E-07	1.47E-07	2.66E-07
**12h**	2.37E-07	7.87E-08	1.29E-07	3.43E-07
**24h**	2.05E-07	1.13E-07	2.40E-07	2.53E-07
**36h**	4.18E-07	1.19E-07	2.18E-07	3.19E-07
**48h**	1.82E-07	4.25E-08	2.93E-07	4.44E-07
**72h**	3.07E-07	1.39E-07	3.13E-07	2.34E-07
	cgd3_1640			
**2h**	2.04E-06	1.01E-05	1.26E-05	6.11E-05
**6h**	3.34E-07	6.94E-06	4.70E-06	2.53E-05
**12h**	3.29E-06	5.76E-06	6.41E-06	1.52E-05
**24h**	1.05E-06	2.32E-06	1.51E-05	5.95E-06
**36h**	1.23E-05	6.33E-06	6.42E-06	4.09E-05
**48h**	4.32E-05	4.12E-06	9.95E-06	4.38E-05
**72h**	9.83E-06	1.94E-06	3.33E-06	1.55E-05
	cgd3_1670			
**2h**	6.19E-05	1.79E-05	1.64E-05	1.91E-04
**6h**	3.89E-06	2.10E-06	1.08E-06	3.23E-06
**12h**	3.60E-07	2.65E-07	3.99E-07	7.62E-09
**24h**	1.06E-08	5.07E-09	8.08E-09	8.60E-07
**36h**	4.23E-09	2.40E-09	3.45E-09	3.29E-09
**48h**	3.71E-06	1.79E-06	4.70E-06	1.52E-05
**72h**	3.01E-05	1.91E-05	7.59E-06	1.38E-05
	cgd3_1700			
**2h**	0.00E+00	0.00E+00	0.00E+00	0.00E+00
**6h**	0.00E+00	0.00E+00	0.00E+00	0.00E+00
**12h**	2.44E-10	1.74E-09	5.49E-09	8.30E-10

**24h**	0.00E+00	3.85E-10	9.75E-09	1.39E-08
**36h**	3.65E-09	2.28E-09	2.10E-09	1.38E-08
**48h**	2.14E-08	5.89E-09	3.13E-08	1.87E-08
**72h**	1.05E-08	2.26E-09	4.11E-08	1.96E-08
	cgd3_1760			
**2h**	1.76E-08	1.25E-08	2.52E-08	2.00E-07
**6h**	0.00E+00	9.40E-11	7.73E-10	0.00E+00
**12h**	2.23E-07	8.21E-08	5.59E-08	1.70E-08
**24h**	1.58E-08	1.37E-08	5.08E-09	1.17E-07
**36h**	2.07E-07	9.57E-08	9.33E-08	1.55E-07
**48h**	4.37E-07	9.73E-08	6.62E-08	2.18E-07
**72h**	1.17E-07	4.55E-08	4.95E-08	1.14E-07
	cgd3_1770			
**2h**	1.91E-09	0.00E+00	0.00E+00	1.04E-08
**6h**	0.00E+00	1.79E-10	1.37E-10	1.20E-10
**12h**	4.30E-07	6.47E-07	8.23E-07	1.35E-07
**24h**	1.04E-07	3.24E-08	1.22E-07	7.04E-07
**36h**	1.18E-07	3.59E-08	8.03E-08	3.16E-07
**48h**	2.42E-07	1.98E-08	7.42E-08	4.23E-08
**72h**	4.15E-08	3.17E-09	4.24E-08	7.51E-08
	cgd3_1820			
**2h**	8.16E-09	1.12E-07	8.42E-08	2.89E-07
**6h**	1.46E-06	1.20E-05	9.84E-06	1.02E-05
**12h**	4.43E-08	8.83E-08	3.60E-07	2.37E-06
**24h**	5.08E-07	4.67E-06	1.72E-06	4.40E-06
**36h**	2.25E-07	8.69E-07	3.35E-06	8.37E-06
**48h**	1.06E-06	6.59E-06	1.60E-06	3.95E-06
**72h**	2.01E-07	4.33E-07	1.49E-06	2.32E-06
	cgd3_1990			
**2h**	7.69E-09	3.39E-08	7.94E-08	4.20E-07
**6h**	5.26E-08	5.41E-07	6.41E-07	1.35E-06
**12h**	1.92E-09	4.04E-08	7.75E-07	2.68E-08
**24h**	8.88E-10	1.92E-07	1.16E-07	
**36h**	3.86E-09	2.27E-09	7.60E-08	6.08E-07
**48h**	2.21E-08	2.18E-07	1.31E-07	1.31E-07
**72h**	1.06E-08	2.89E-08	1.07E-07	2.87E-07
	cgd3_2020			
**2h**	8.09E-08	0.00E+00	0.00E+00	0.00E+00
**6h**	3.78E-07	5.28E-06	3.14E-06	1.08E-05
**12h**	4.13E-08	1.63E-07	2.10E-07	2.41E-06
**24h**	1.06E-07	3.82E-06	1.21E-06	1.86E-06
**36h**	3.24E-07	5.22E-07	4.43E-06	1.67E-05
**48h**	1.43E-06	4.18E-06	1.40E-06	3.70E-06
**72h**	9.07E-08	7.09E-07	1.14E-06	8.12E-07
	cgd3_2080			
**2h**	8.93E-07	2.43E-05	3.89E-06	1.53E-04
**6h**	4.22E-08	5.00E-07	1.74E-06	2.94E-06
**12h**	2.65E-06	4.26E-06	2.00E-07	5.30E-05
**24h**	2.55E-07	8.86E-06	5.02E-06	3.09E-05
**36h**	2.28E-06	1.84E-05	3.37E-05	2.52E-04
**48h**	1.33E-05	4.62E-05	2.77E-05	7.61E-05
**72h**	1.04E-06	2.11E-05	4.50E-05	3.72E-05
	cgd3_2110			

**2h**	2.19E-06	3.30E-06	1.51E-05	4.28E-05
**6h**	1.55E-06	2.36E-06	2.75E-06	2.37E-05
**12h**	2.58E-06	6.82E-06	7.74E-06	4.30E-05
**24h**	3.68E-07	5.84E-06	3.14E-06	4.17E-05
**36h**	1.39E-06	3.33E-07	2.16E-06	2.91E-05
**48h**	1.30E-06	2.74E-06	2.71E-06	1.08E-05
**72h**	1.90E-07	9.88E-07	1.29E-06	4.97E-06
	cgd3_2150			
**2h**	0.00E+00	0.00E+00	0.00E+00	7.00E-10
**6h**	2.81E-07	1.63E-06	1.12E-06	1.91E-06
**12h**	1.84E-08	5.54E-09	3.27E-09	1.60E-07
**24h**	1.38E-07	6.11E-07	6.98E-07	3.26E-07
**36h**	2.53E-07	4.11E-07	8.56E-07	1.61E-06
**48h**	1.72E-07	2.91E-07	5.38E-07	6.62E-07
**72h**	1.80E-08	1.74E-07	2.11E-07	7.49E-08
	cgd3_2180			
**2h**	0.00E+00	0.00E+00	4.06E-09	0.00E+00
**6h**	4.04E-10	3.00E-08	1.75E-08	2.87E-09
**12h**	8.64E-11	4.40E-09	1.29E-10	1.71E-08
**24h**	0.00E+00	6.91E-10	5.17E-09	1.81E-08
**36h**	1.70E-10	6.79E-11	7.88E-09	2.12E-07
**48h**	1.82E-07	1.48E-07	4.25E-07	1.76E-06
**72h**	2.68E-09	1.20E-07	1.14E-07	1.17E-07
	cgd3_2190			
**2h**	4.23E-09	1.24E-08	7.43E-07	2.37E-07
**6h**	1.38E-07	4.23E-07	4.29E-07	6.16E-07
**12h**	4.07E-08	6.85E-08	2.60E-07	2.07E-06
**24h**	3.11E-08	3.12E-07	3.36E-07	7.34E-07
**36h**	4.58E-08	3.51E-07	1.28E-06	2.89E-06
**48h**	4.54E-07	1.48E-06	1.06E-06	1.28E-06
**72h**	2.24E-08	2.46E-07	5.82E-07	2.44E-07
	cgd3_2220			
**2h**	5.28E-09	1.49E-08	4.04E-08	1.32E-07
**6h**	1.14E-07	3.12E-07	2.14E-07	3.80E-07
**12h**	1.04E-08	1.07E-08	4.10E-08	5.72E-08
**24h**	1.21E-07	1.21E-07	1.48E-07	2.17E-07
**36h**	1.09E-07	2.20E-07	2.85E-07	2.30E-07
**48h**	1.54E-07	1.23E-07	2.84E-07	4.68E-07
**72h**	3.31E-09	4.89E-08	6.14E-08	1.45E-08
	cgd3_2230			
**2h**	1.23E-09	4.02E-10	5.37E-08	2.78E-07
**6h**	3.63E-08	1.41E-07	2.51E-07	1.03E-06
**12h**	1.12E-09	9.44E-09	1.19E-08	4.96E-08
**24h**	1.58E-08	1.10E-07	8.37E-08	2.15E-07
**36h**	1.20E-08	2.46E-08	1.31E-07	3.48E-07
**48h**	8.00E-08	1.68E-07	2.87E-07	3.31E-07
**72h**	4.16E-09	4.11E-08	5.06E-08	3.27E-08
	cgd3_2240			
**2h**	4.22E-09	4.33E-09	1.16E-08	1.60E-08
**6h**	2.65E-07	4.10E-07	1.00E-06	2.35E-06
**12h**	1.21E-08	4.48E-09	5.86E-09	1.14E-07
**24h**	9.69E-08	2.07E-07	1.54E-07	2.88E-07
**36h**	2.61E-07	6.77E-07	6.60E-07	6.44E-07

**48h**	2.44E-07	2.66E-07	5.52E-07	5.00E-07
**72h**	6.93E-09	6.47E-08	1.33E-07	4.50E-08
	cgd3_2410			
**2h**	2.10E-06	1.04E-05	9.22E-06	9.56E-05
**6h**	1.45E-05	8.79E-05	4.66E-05	5.81E-04
**12h**	4.34E-07	9.21E-06	3.94E-06	2.03E-05
**24h**	2.09E-06	7.28E-05	5.85E-05	1.34E-04
**36h**	5.93E-06	2.55E-05	2.61E-05	3.77E-04
**48h**	1.01E-05	4.21E-05	2.21E-05	2.02E-04
**72h**	4.22E-06	9.54E-06	2.77E-05	3.04E-05
	cgd3_2490			
**2h**	6.90E-08	1.23E-05	1.50E-05	2.21E-05
**6h**	9.61E-08	0.00E+00	7.08E-06	1.88E-06
**12h**	9.61E-09	0.00E+00	2.48E-07	3.49E-08
**24h**	3.80E-08	7.85E-07	2.26E-06	1.47E-06
**36h**	1.52E-06	4.65E-06	3.80E-05	1.68E-06
**48h**	2.40E-05	1.11E-04	4.53E-04	1.67E-04
**72h**	7.75E-07	1.39E-05	3.39E-05	9.97E-06
	cgd3_2500			
**2h**	8.66E-05	3.36E-04	9.68E-04	1.16E-04
**6h**	2.54E-05	2.61E-04	2.60E-04	9.67E-05
**12h**	7.31E-06	3.96E-06	1.28E-04	4.05E-05
**24h**	1.59E-05	1.51E-04	3.98E-04	9.80E-05
**36h**	6.49E-06	9.95E-06	4.02E-05	1.21E-05
**48h**	2.81E-05	3.62E-05	1.53E-04	5.74E-05
**72h**	2.76E-06	3.57E-05	0.00E+00	5.15E-06
	cgd3_2390			
**2h**	1.20E-06	4.65E-06	2.63E-05	2.20E-05
**6h**	1.82E-05	5.97E-05	1.36E-04	2.83E-04
**12h**	1.17E-06	1.44E-06	6.41E-05	4.92E-05
**24h**	9.70E-06	1.67E-05	5.17E-05	4.18E-05
**36h**	3.07E-06	2.92E-06	6.33E-05	1.25E-05
**48h**	2.24E-06	1.85E-06	1.73E-05	8.64E-06
**72h**	4.61E-07	1.01E-06	1.46E-05	1.58E-06
	cgd3_2510			
**2h**	1.02E-08	1.04E-08	9.67E-08	2.57E-07
**6h**	5.01E-08	2.64E-08	2.51E-08	2.14E-07
**12h**	1.48E-08	5.52E-08	3.61E-07	3.72E-07
**24h**	7.86E-08	3.94E-08	9.32E-08	7.81E-08
**36h**	4.53E-08	4.00E-08	4.00E-07	1.08E-07
**48h**	3.76E-08	2.21E-08	1.35E-07	3.92E-08
**72h**	1.47E-08	3.76E-08	1.35E-07	2.56E-08
	cgd3_2530			
**2h**	4.07E-08	8.23E-08	8.97E-08	9.05E-08
**6h**	5.51E-07	6.56E-07	3.31E-06	3.40E-06
**12h**	4.98E-08	2.62E-07	7.27E-07	1.02E-06
**24h**	1.12E-07	1.16E-07	1.03E-06	2.09E-06
**36h**	5.18E-07	6.78E-07	3.87E-06	2.48E-06
**48h**	3.76E-07	3.15E-07	1.47E-06	8.64E-07
**72h**	1.22E-07	1.82E-07	3.30E-07	2.43E-07
	cgd3_2550			
**2h**	5.40E-07	4.44E-07	2.26E-06	4.41E-06
**6h**	5.20E-07	4.52E-07	3.13E-06	1.84E-06

**12h**	5.58E-07	1.04E-06	4.12E-06	8.95E-06
**24h**	8.30E-07	3.42E-07	2.61E-06	1.83E-06
**36h**	6.11E-07	7.16E-07	4.84E-06	3.95E-06
**48h**	1.59E-07	2.43E-07	1.79E-06	1.70E-06
**72h**	2.63E-07	5.03E-07	9.60E-07	4.62E-07
	cgd3_2580			
**2h**	4.90E-05	1.86E-05	1.06E-04	9.10E-05
**6h**	8.11E-05	9.58E-05	4.06E-04	1.38E-04
**12h**	6.33E-05	1.59E-04	4.25E-04	5.45E-04
**24h**	8.51E-05	5.37E-05	4.80E-04	3.43E-05
**36h**	2.05E-04	3.34E-04	8.15E-04	1.35E-04
**48h**	1.70E-04	3.73E-04	6.65E-04	2.46E-04
**72h**	5.39E-05	1.53E-04	6.08E-04	7.24E-05
	cgd3_2590			
**2h**	5.14E-08	6.19E-08	3.89E-07	6.60E-08
**6h**	1.75E-07	4.24E-07	2.64E-06	1.31E-06
**12h**	1.87E-08	5.28E-08	2.94E-07	5.07E-07
**24h**	3.45E-08	1.90E-08	9.65E-07	1.24E-06
**36h**	8.44E-07	2.95E-07	9.00E-07	0.00E+00
**48h**	1.10E-07	2.30E-07	3.01E-07	1.18E-06
**72h**	1.40E-07	1.17E-07	2.04E-07	9.55E-08
	cgd3_2600			
**2h**	1.43E-07	7.64E-08	8.72E-07	3.37E-07
**6h**	2.97E-07	7.17E-07	9.35E-06	1.43E-06
**12h**	0.00E+00	1.52E-08	5.56E-07	4.10E-06
**24h**	2.18E-06	1.66E-04	8.05E-04	0.00E+00
**36h**	2.81E-07	4.11E-07	5.01E-06	9.25E-04
**48h**	1.52E-05	2.54E-04	0.00E+00	0.00E+00
**72h**	0.00E+00	0.00E+00	0.00E+00	0.00E+00
	cgd3_2610			
**2h**	1.73E-06	1.08E-05	0.00E+00	1.50E-06
**6h**	8.12E-06	1.27E-05	6.19E-05	1.03E-03
**12h**	4.70E-07	1.78E-06	4.42E-06	2.99E-05
**24h**	2.14E-06	3.51E-06	8.67E-05	4.02E-05
**36h**	5.59E-06	5.07E-06	4.52E-05	7.20E-06
**48h**	2.84E-06	1.10E-05	3.40E-05	4.82E-05
**72h**	6.60E-07	1.59E-06	6.61E-06	1.90E-06
	cgd3_2620			
**2h**	0.00E+00	9.14E-05	6.35E-04	1.16E-04
**6h**	0.00E+00	1.73E-04	2.12E-03	1.62E-03
**12h**	0.00E+00	1.01E-05	5.46E-04	3.17E-04
**24h**	0.00E+00	1.69E-05	4.15E-04	5.59E-04
**36h**	0.00E+00	4.53E-05	2.41E-04	5.15E-05
**48h**	0.00E+00	2.54E-05	7.61E-05	3.72E-05
**72h**	0.00E+00	6.40E-06	2.75E-05	3.98E-06
	cgd3_2630			
**2h**	2.28E-05	1.40E-07	3.57E-07	1.42E-06
**6h**	4.24E-05	9.14E-08	2.15E-06	8.68E-07
**12h**	4.24E-06	1.02E-08	2.08E-07	3.29E-07
**24h**	3.62E-05	6.47E-08	4.64E-07	4.83E-07
**36h**	6.46E-05	9.78E-08	2.19E-07	1.60E-07
**48h**	6.52E-06	8.95E-08	3.60E-07	1.98E-07
**72h**	1.38E-06	4.45E-08	3.74E-08	9.41E-09

	cgd1_3660			
**2h**	0.00E+00	3.03E-06	1.13E-05	4.51E-05
**6h**	1.74E-03	2.65E-04	8.72E-05	6.84E-05
**12h**	7.98E-04	3.76E-05	3.50E-05	2.36E-05
**24h**	7.45E-04	6.69E-05	2.98E-04	7.51E-05
**36h**	1.11E-03	1.65E-04	2.06E-05	7.31E-05
**48h**	1.09E-03	1.03E-04	2.65E-05	6.43E-05
**72h**	4.88E-04	2.78E-05	2.41E-05	4.74E-05
	cgd1_3680			
**2h**	0.00E+00	0.00E+00	0.00E+00	0.00E+00
**6h**	0.00E+00	0.00E+00	0.00E+00	0.00E+00
**12h**	4.80E-08	1.58E-08	1.44E-07	6.70E-08
**24h**	8.81E-09	2.30E-08	9.19E-08	8.56E-07
**36h**	5.58E-07	2.29E-07	1.03E-06	1.36E-06
**48h**	2.71E-07	1.45E-07	1.16E-06	5.05E-07
**72h**	1.99E-06	3.36E-07	2.39E-06	1.46E-06
	cgd1_3690			
**2h**	6.67E-07	3.78E-08	0.00E+00	2.25E-05
**6h**	3.41E-04	8.57E-05	1.93E-05	1.28E-03
**12h**	7.35E-03	1.48E-03	9.12E-04	1.69E-03
**24h**	1.46E-03	5.70E-04	5.40E-04	1.38E-03
**36h**	2.21E-03	4.40E-04	1.02E-03	4.83E-03
**48h**	1.07E-03	2.20E-04	3.08E-04	1.04E-03
**72h**	1.71E-04	1.73E-05	1.67E-04	1.38E-04
	cgd1_3700			
**2h**	1.45E-07	7.42E-08	0.00E+00	0.00E+00
**6h**	6.66E-05	2.51E-05	1.89E-05	2.76E-05
**12h**	7.81E-05	6.58E-05	7.60E-05	5.52E-05
**24h**	1.68E-05	1.23E-05	6.28E-05	8.18E-05
**36h**	4.13E-05	1.51E-05	3.97E-04	8.95E-05
**48h**	7.22E-05	4.13E-05	1.49E-04	1.46E-04
**72h**	1.46E-05	2.86E-06	2.55E-05	1.08E-05
	cgd1_3710			
**2h**	5.94E-05	0.00E+00	0.00E+00	1.13E-07
**6h**	8.12E-03	1.72E-03	2.65E-04	1.56E-03
**12h**	8.80E-04	4.72E-05	8.02E-05	1.60E-04
**24h**	6.39E-03	7.53E-04	6.01E-04	5.18E-04
**36h**	2.70E-03	6.00E-04	3.06E-04	5.14E-04
**48h**	6.78E-03	1.30E-03	2.00E-04	3.25E-04
**72h**	1.74E-03	1.53E-04	5.85E-05	1.38E-04
	cgd1_3720			
**2h**	0.00E+00	0.00E+00	0.00E+00	0.00E+00
**6h**	7.95E-04	4.73E-05	2.93E-04	5.99E-04
**12h**	5.32E-05	4.29E-05	3.38E-04	2.06E-04
**24h**	1.23E-04	1.73E-05	2.84E-04	3.14E-04
**36h**	1.56E-04	1.54E-05	1.77E-04	1.09E-03
**48h**	1.32E-04	2.76E-05	1.58E-04	1.96E-04
**72h**	8.43E-05	4.66E-06	6.68E-05	1.65E-04
	cgd1_3730			
**2h**	0.00E+00	0.00E+00	0.00E+00	0.00E+00
**6h**	0.00E+00	0.00E+00	0.00E+00	0.00E+00
**12h**	0.00E+00	1.65E-07	1.60E-06	7.54E-08
**24h**	1.34E-07	8.45E-08	1.68E-06	4.19E-07

**36h**	5.10E-07	1.01E-06	2.60E-05	1.22E-05
**48h**	1.31E-05	2.30E-06	5.12E-05	5.67E-05
**72h**	2.22E-05	2.62E-06	8.10E-05	6.15E-05
	cgd1_3740			
**2h**	0.00E+00	0.00E+00	0.00E+00	0.00E+00
**6h**	0.00E+00	4.15E-06	2.10E-04	5.96E-06
**12h**	1.09E-05	2.11E-07	3.73E-05	8.55E-05
**24h**	4.27E-05	7.85E-06	2.52E-04	1.75E-04
**36h**	3.27E-04	6.61E-05	8.57E-05	7.20E-05
**48h**	1.42E-04	2.73E-05	5.17E-05	3.18E-04
**72h**	4.51E-04	7.06E-05	1.17E-04	1.59E-05
	cgd1_3760			
**2h**	3.94E-07	0.00E+00	0.00E+00	0.00E+00
**6h**	1.35E-04	1.62E-04	4.47E-04	1.63E-04
**12h**	2.33E-06	3.37E-06	1.82E-05	7.26E-06
**24h**	1.94E-05	1.48E-04	5.39E-04	4.80E-05
**36h**	1.87E-05	1.45E-04	1.35E-04	1.23E-05
**48h**	3.94E-05	1.68E-04	3.81E-05	1.50E-05
**72h**	6.42E-06	1.55E-04	5.11E-05	3.74E-06
	cgd1_3770			
**2h**	0.00E+00	0.00E+00	0.00E+00	0.00E+00
**6h**	0.00E+00	0.00E+00	0.00E+00	0.00E+00
**12h**	8.03E-09	4.48E-09	3.67E-08	2.72E-08
**24h**	2.77E-08	9.16E-08	2.38E-08	3.28E-09
**36h**	4.58E-08	8.27E-08	1.86E-07	6.56E-08
**48h**	5.88E-08	2.67E-07	2.33E-08	1.37E-08
**72h**	2.91E-08	9.55E-08	4.52E-07	4.65E-08
	cgd1_3780			
**2h**	1.03E-07	5.52E-08	0.00E+00	5.70E-08
**6h**	7.24E-08	7.34E-08	1.41E-08	3.22E-08
**12h**	7.69E-05	5.53E-05	4.26E-05	5.53E-05
**24h**	2.96E-05	2.45E-05	1.12E-05	2.75E-06
**36h**	8.74E-06	1.33E-05	4.74E-05	4.82E-05
**48h**	1.65E-05	6.23E-06	6.71E-06	3.27E-06
**72h**	9.91E-07	1.10E-06	6.38E-07	1.35E-06
	cgd1_3800			
**2h**	4.09E-08	7.58E-09	1.99E-09	4.77E-09
**6h**	7.29E-08	0.00E+00	1.38E-09	2.17E-08
**12h**	5.92E-06	7.89E-06	5.90E-06	3.86E-06
**24h**	5.89E-07	2.03E-06	4.53E-07	2.23E-07
**36h**	5.63E-07	1.21E-06	4.34E-06	2.78E-06
**48h**	4.74E-07	6.30E-07	1.84E-06	5.53E-07
**72h**	2.36E-07	1.26E-06	7.52E-07	4.50E-07
	cgd3_3060			
**2h**	3.09E-03	3.34E-03	4.15E-02	1.19E-02
**6h**	6.97E-03	8.02E-03	1.70E-02	2.39E-02
**12h**	1.12E-03	2.89E-04	2.75E-03	3.43E-02
**24h**	4.29E-04	7.57E-04	4.43E-03	4.71E-03
**36h**	2.16E-03	7.63E-04	3.09E-03	2.12E-03
**48h**	1.25E-03	8.45E-04	8.39E-04	4.95E-03
**72h**	1.45E-04	1.75E-04	1.34E-03	1.96E-04
	cgd3_3070			
**2h**	4.32E-08	3.52E-08	1.92E-07	1.66E-08

**6h**	3.91E-07	1.04E-07	1.43E-06	7.39E-07
**12h**	7.47E-09	3.60E-10	3.06E-08	2.14E-07
**24h**	1.90E-08	1.76E-08	3.53E-07	4.81E-07
**36h**	4.28E-08	6.79E-08	4.65E-07	1.07E-07
**48h**	1.39E-08	2.53E-08	1.39E-07	1.38E-07
**72h**	6.64E-09	4.71E-08	7.08E-08	1.49E-09
	cgd3_3090			
**2h**	0.00E+00	0.00E+00	0.00E+00	0.00E+00
**6h**	0.00E+00	0.00E+00	0.00E+00	1.04E-05
**12h**	5.26E-07	2.44E-06	2.13E-06	3.36E-05
**24h**	8.83E-07	1.86E-06	6.25E-05	2.60E-05
**36h**	1.61E-05	1.07E-05	6.09E-05	1.70E-05
**48h**	2.75E-06	7.60E-06	4.19E-05	2.58E-05
**72h**	2.90E-06	8.68E-06	4.00E-05	8.09E-06
	cgd3_3120			
**2h**	3.95E-05	1.16E-04	3.35E-04	1.21E-04
**6h**	2.78E-05	2.37E-05	1.85E-04	3.39E-04
**12h**	5.23E-05	1.57E-05	7.55E-05	2.41E-04
**24h**	3.86E-06	7.55E-06	4.16E-05	1.97E-05
**36h**	1.32E-05	9.50E-06	1.16E-04	8.90E-05
**48h**	5.72E-06	1.11E-05	1.19E-04	4.56E-05
**72h**	1.33E-05	1.17E-05	6.63E-05	1.14E-05
	cgd3_3150			
**2h**	7.93E-09	0.00E+00	0.00E+00	0.00E+00
**6h**	6.12E-07	2.99E-07	2.19E-06	6.32E-06
**12h**	6.58E-08	5.74E-08	7.59E-08	5.00E-07
**24h**	2.54E-08	3.71E-08	1.37E-06	7.65E-07
**36h**	4.21E-07	3.94E-07	2.49E-06	1.30E-06
**48h**	4.84E-08	5.13E-08	5.78E-07	3.62E-07
**72h**	8.04E-08	7.78E-08	1.14E-07	5.44E-08
	cgd3_3160			
**2h**	5.86E-07	4.73E-07	2.60E-06	1.33E-06
**6h**	2.61E-06	1.20E-06	2.91E-05	7.26E-05
**12h**	9.40E-08	2.32E-08	1.67E-06	3.37E-06
**24h**	3.97E-07	1.57E-07	5.87E-06	7.46E-06
**36h**	1.57E-06	1.17E-06	8.26E-06	2.84E-06
**48h**	3.40E-07	3.49E-07	3.16E-06	1.36E-06
**72h**	1.19E-07	1.88E-07	7.61E-07	2.81E-07
	cgd3_3170			
**2h**	1.37E-07	2.83E-07	1.52E-07	1.64E-06
**6h**	5.07E-05	1.94E-05	2.89E-04	3.83E-04
**12h**	1.30E-06	3.63E-07	6.17E-06	1.37E-05
**24h**	3.73E-06	2.62E-06	1.08E-04	5.03E-05
**36h**	1.39E-05	9.47E-06	8.44E-05	3.54E-05
**48h**	3.94E-06	2.64E-06	2.92E-05	1.01E-05
**72h**	1.44E-06	1.02E-06	6.82E-06	1.64E-06
	cgd3_3180			
**2h**	1.40E-07	7.79E-08	2.59E-07	2.05E-07
**6h**	7.53E-09	1.61E-09	1.36E-07	3.76E-08
**12h**	2.90E-06	1.13E-06	6.10E-06	1.39E-05
**24h**	2.97E-07	2.10E-07	5.65E-07	4.03E-07
**36h**	4.10E-07	3.33E-07	4.32E-06	3.12E-06
**48h**	2.36E-07	2.13E-07	1.43E-06	9.48E-07

**72h**	5.97E-08	4.49E-08	5.04E-07	1.48E-07
	cgd3_3190			
**2h**	1.52E-06	2.52E-06	2.15E-06	3.19E-06
**6h**	4.56E-07	3.03E-07	1.49E-06	5.80E-06
**12h**	3.86E-06	1.32E-06	7.61E-06	1.55E-05
**24h**	9.23E-07	5.71E-07	2.87E-06	2.45E-06
**36h**	6.05E-07	4.25E-07	2.97E-06	2.97E-06
**48h**	2.81E-07	2.98E-07	1.36E-06	1.23E-06
**72h**	4.54E-08	1.00E-07	2.03E-07	6.18E-08
	cgd3_3230			
**2h**	2.43E-06	4.54E-05	1.48E-04	1.09E-03
**6h**	5.65E-05	8.47E-05	4.65E-05	6.53E-04
**12h**	7.29E-06	1.79E-06	5.32E-05	3.49E-04
**24h**	1.96E-05	1.83E-05	8.31E-05	8.93E-05
**36h**	1.31E-05	2.65E-05	8.93E-05	3.02E-05
**48h**	1.86E-05	1.47E-05	3.46E-05	6.26E-05
**72h**	4.24E-06	8.69E-06	9.90E-06	9.98E-06
	cgd3_3290			
**2h**	0.00E+00	0.00E+00	0.00E+00	0.00E+00
**6h**	0.00E+00	0.00E+00	0.00E+00	0.00E+00
**12h**	2.00E-05	9.49E-06	5.50E-05	5.92E-04
**24h**	1.16E-06	1.30E-06	2.75E-06	1.18E-05
**36h**	3.65E-06	2.90E-06	4.85E-05	2.04E-05
**48h**	7.51E-07	2.02E-06	1.43E-05	1.49E-05
**72h**	7.15E-07	1.48E-06	3.68E-07	9.74E-07
	cgd3_3310			
**2h**	0.00E+00	0.00E+00	1.54E-08	0.00E+00
**6h**	3.44E-09	0.00E+00	6.76E-09	0.00E+00
**12h**	1.44E-09	2.45E-09	4.58E-09	5.65E-08
**24h**	6.83E-10	3.48E-09	6.53E-09	1.58E-08
**36h**	1.45E-08	1.02E-08	1.86E-08	5.35E-08
**48h**	8.70E-09	1.59E-08	2.29E-08	4.19E-08
**72h**	8.75E-09	9.77E-09	1.34E-08	1.47E-08
	cgd3_3450			
**2h**	2.72E-07	0.00E+00	4.78E-08	9.85E-08
**6h**	0.00E+00	0.00E+00	0.00E+00	0.00E+00
**12h**	0.00E+00	2.58E-08	1.53E-07	5.67E-08
**24h**	7.94E-10	2.10E-08	1.09E-06	8.77E-07
**36h**	1.80E-06	7.04E-07	5.42E-06	8.00E-07
**48h**	1.61E-05	1.96E-05	4.20E-05	9.44E-05
**72h**	1.99E-06	9.77E-07	7.05E-07	1.21E-06
	cgd3_3460			
**2h**	1.68E-07	5.97E-07	2.30E-06	1.94E-06
**6h**	1.51E-06	1.30E-06	5.46E-06	1.78E-05
**12h**	3.24E-07	2.53E-07	4.07E-06	3.51E-06
**24h**	6.93E-07	1.53E-06	6.14E-06	9.61E-06
**36h**	5.06E-07	3.55E-07	4.44E-06	1.91E-06
**48h**	2.96E-07	3.68E-07	1.43E-06	3.23E-06
**72h**	2.95E-07	1.67E-07	5.63E-07	5.78E-07
	cgd3_3490			
**2h**	2.49E-09	0.00E+00	4.11E-08	1.19E-07
**6h**	2.71E-09	5.68E-08	5.54E-07	2.27E-07
**12h**	8.35E-07	1.47E-06	3.12E-06	7.86E-06

**24h**	3.13E-08	1.26E-07	7.70E-07	5.87E-07
**36h**	2.35E-07	1.76E-07	1.96E-06	3.68E-06
**48h**	1.47E-07	2.50E-07	9.90E-07	1.25E-06
**72h**	5.17E-07	2.94E-07	1.35E-06	2.56E-06
	cgd3_3500			
**2h**	6.46E-07	3.57E-07	2.42E-06	7.01E-07
**6h**	1.36E-07	3.51E-07	1.60E-07	0.00E+00
**12h**	1.70E-07	6.51E-08	4.55E-06	2.01E-06
**24h**	3.78E-07	8.04E-05	3.59E-07	1.29E-06
**36h**	2.63E-07	3.65E-07	1.40E-06	8.55E-07
**48h**	7.87E-08	5.97E-08	1.79E-07	3.19E-07
**72h**	1.86E-07	1.28E-07	1.84E-07	1.85E-07
	cgd3_3520			
**2h**	1.25E-07	1.35E-07	4.09E-07	2.29E-07
**6h**	0.00E+00	1.04E-07	2.34E-07	8.32E-07
**12h**	3.32E-07	2.00E-07	2.56E-06	7.74E-07
**24h**	9.14E-08	8.85E-08	6.16E-07	1.20E-06
**36h**	3.10E-07	1.92E-07	1.40E-06	1.30E-06
**48h**	2.44E-07	1.52E-07	6.26E-07	1.13E-06
**72h**	4.47E-07	4.42E-07	1.65E-06	1.54E-06
	cgd3_3550			
**2h**	1.43E-07	2.86E-07	4.10E-06	2.92E-07
**6h**	7.42E-08	7.73E-08	4.91E-07	4.05E-07
**12h**	6.52E-08	4.26E-08	1.21E-06	1.02E-06
**24h**	3.25E-08	1.07E-07	5.41E-07	7.17E-07
**36h**	3.26E-07	2.43E-07	1.51E-06	1.05E-06
**48h**	3.47E-07	2.50E-07	1.27E-06	1.80E-06
**72h**	5.60E-07	5.36E-07	7.09E-07	9.79E-07
	cgd3_3620			
**2h**	3.19E-05	4.98E-06	1.59E-04	3.26E-05
**6h**	2.08E-04	3.51E-05	5.82E-04	9.48E-04
**12h**	3.41E-06	4.10E-07	3.84E-04	4.64E-04
**24h**	5.11E-05	1.98E-05	4.26E-04	1.22E-03
**36h**	5.41E-05	2.94E-05	3.74E-04	2.31E-04
**48h**	6.13E-05	2.04E-05	1.72E-04	3.42E-04
**72h**	1.38E-05	8.04E-06	2.74E-05	1.53E-05
	cgd3_3690			
**2h**	1.41E-07	1.17E-07	1.96E-06	3.09E-07
**6h**	3.00E-08	3.97E-08	2.16E-07	1.02E-07
**12h**	7.95E-08	7.59E-08	9.45E-07	3.72E-07
**24h**	2.74E-08	5.36E-08	3.73E-07	7.86E-08
**36h**	1.65E-07	1.06E-07	4.11E-07	5.91E-07
**48h**	2.93E-08	4.71E-08	1.64E-07	1.48E-07
**72h**	5.95E-08	7.14E-08	6.11E-08	1.79E-07
	cgd3_3830			
**2h**	0.00E+00	0.00E+00	0.00E+00	0.00E+00
**6h**	0.00E+00	0.00E+00	0.00E+00	4.73E-09
**12h**	2.35E-06	1.85E-06	2.99E-06	4.66E-06
**24h**	1.16E-07	7.84E-08	4.54E-07	5.54E-07
**36h**	2.18E-07	2.52E-07	9.92E-07	1.83E-06
**48h**	1.06E-07	1.86E-07	3.44E-07	4.53E-07
**72h**	6.79E-08	1.59E-07	7.48E-08	2.59E-07
	cgd3_3870			

**2h**	1.23E-04	1.06E-04	3.45E-02	6.17E-05
**6h**	7.81E-05	4.46E-05	2.57E-04	2.87E-04
**12h**	3.75E-05	6.84E-05	3.87E-04	7.60E-04
**24h**	5.47E-05	1.25E-04	5.14E-04	3.39E-04
**36h**	3.85E-05	4.16E-05	1.78E-04	2.94E-04
**48h**	1.97E-05	2.70E-05	6.35E-05	1.23E-04
**72h**	3.84E-06	1.50E-05	8.72E-06	2.35E-05
	cgd3_3940			
**2h**	4.70E-07	1.89E-07	1.12E-06	3.14E-07
**6h**	5.85E-08	3.83E-08	3.57E-07	1.21E-07
**12h**	9.27E-07	3.68E-07	1.83E-06	2.42E-06
**24h**	4.55E-07	2.04E-07	5.46E-07	2.83E-07
**36h**	3.52E-07	1.38E-06	6.94E-07	2.78E-07
**48h**	2.13E-07	4.01E-07	3.09E-07	4.83E-07
**72h**	1.15E-07	1.07E-07	1.06E-07	1.55E-07
	cgd3_4030			
**2h**	1.60E-05	4.76E-05	9.94E-06	3.27E-05
**6h**	1.55E-05	6.81E-06	7.22E-06	1.18E-05
**12h**	7.89E-06	1.80E-05	3.37E-05	3.49E-05
**24h**	1.76E-05	1.04E-05	3.69E-05	4.13E-05
**36h**	1.98E-05	1.86E-05	3.41E-05	5.08E-05
**48h**	9.93E-06	1.54E-05	1.32E-05	3.22E-05
**72h**	1.57E-05	1.72E-05	1.35E-05	8.89E-06
	cgd3_4040			
**2h**	3.52E-06	1.84E-06	7.98E-06	5.93E-06
**6h**	1.92E-05	1.50E-05	3.82E-05	1.02E-04
**12h**	3.56E-06	2.52E-06	3.03E-05	3.98E-05
**24h**	1.18E-05	4.63E-06	6.06E-05	2.37E-05
**36h**	5.24E-06	1.93E-06	2.19E-05	3.42E-05
**48h**	4.88E-06	8.33E-07	1.48E-04	6.18E-06
**72h**	4.63E-07	4.86E-07	2.59E-06	1.73E-06
	cgd3_4080			
**2h**	1.78E-08	9.64E-09	1.93E-07	1.68E-07
**6h**	4.10E-07	8.38E-07	6.00E-06	7.86E-06
**12h**	1.60E-07	1.46E-07	1.66E-06	2.74E-06
**24h**	2.36E-07	1.67E-07	3.46E-06	3.74E-06
**36h**	6.19E-08	5.37E-08	8.90E-07	2.32E-06
**48h**	1.19E-07	2.64E-08	8.34E-07	2.14E-07
**72h**	3.71E-08	1.91E-08	1.80E-07	4.97E-08
	cgd3_4120			
**2h**	5.25E-09	2.32E-08	2.79E-08	0.00E+00
**6h**	8.85E-09	3.62E-08	3.50E-08	1.25E-07
**12h**	1.38E-09	9.51E-10	1.46E-08	2.76E-08
**24h**	9.27E-09	6.10E-09	9.48E-08	1.06E-07
**36h**	4.09E-08	2.20E-08	6.02E-08	2.14E-07
**48h**	3.97E-08	9.40E-09	1.60E-07	5.58E-07
**72h**	2.02E-08	3.06E-08	5.81E-08	9.12E-08
	cgd3_4130			
**2h**	0.00E+00	3.71E-08	0.00E+00	0.00E+00
**6h**	2.53E-07	7.91E-07	2.14E-06	1.95E-06
**12h**	1.18E-06	5.44E-07	1.19E-05	8.10E-06
**24h**	1.08E-06	5.65E-07	4.74E-06	1.02E-05
**36h**	8.95E-06	3.58E-06	3.61E-05	4.35E-05

**48h**	7.36E-06	6.65E-07	1.93E-05	5.92E-06
**72h**	3.92E-06	6.58E-07	1.07E-05	1.04E-06
	cgd3_4140			
**2h**	3.07E-05	2.15E-05	7.73E-05	1.29E-04
**6h**	6.09E-05	2.11E-05	2.06E-04	3.70E-04
**12h**	2.11E-06	1.57E-06	8.06E-05	2.73E-04
**24h**	1.78E-05	4.80E-06	4.77E-05	7.33E-05
**36h**	1.43E-05	1.50E-05	7.69E-05	1.20E-04
**48h**	7.84E-06	2.06E-06	3.10E-05	1.12E-05
**72h**	3.00E-06	1.93E-06	9.00E-06	4.77E-06
	cgd3_4150			
**2h**	3.35E-05	6.09E-05	7.46E-05	2.36E-04
**6h**	1.67E-05	3.81E-06	4.06E-05	2.50E-05
**12h**	1.37E-05	1.88E-05	1.92E-03	3.32E-04
**24h**	2.20E-05	2.68E-03	4.33E-05	4.18E-05
**36h**	9.90E-06	8.47E-06	5.91E-05	8.70E-05
**48h**	3.19E-06	2.61E-06	2.60E-05	2.05E-05
**72h**	5.03E-06	2.03E-06	1.62E-05	4.23E-06
	cgd3_4190			
**2h**	1.68E-06	3.25E-06	1.58E-06	9.45E-06
**6h**	1.48E-06	3.47E-07	3.04E-06	2.15E-06
**12h**	8.82E-07	1.15E-06	3.53E-06	2.53E-05
**24h**	1.90E-06	7.61E-07	1.17E-06	6.03E-06
**36h**	1.16E-06	1.06E-06	2.98E-06	8.46E-06
**48h**	4.00E-07	3.92E-07	1.54E-06	1.58E-06
**72h**	8.01E-07	1.50E-07	1.23E-06	1.43E-07
	cgd3_4210			
**2h**	7.89E-07	1.65E-06	6.86E-07	3.51E-06
**6h**	5.64E-06	3.98E-06	7.56E-05	1.66E-05
**12h**	1.24E-06	4.38E-07	5.58E-06	7.08E-06
**24h**	2.80E-06	6.26E-07	7.88E-06	1.95E-05
**36h**	3.33E-06	1.24E-06	1.83E-05	1.30E-05
**48h**	1.26E-06	1.37E-06	7.75E-06	4.78E-06
**72h**	5.02E-07	3.79E-07	1.01E-06	9.33E-08
	cgd3_4230			
**2h**	2.81E-08	2.81E-08	3.20E-08	2.94E-08
**6h**	2.85E-09	3.61E-09	1.88E-09	2.64E-09
**12h**	6.76E-10	2.98E-10	2.45E-09	0.00E+00
**24h**	4.93E-10	7.40E-10	5.23E-09	5.00E-09
**36h**	2.27E-10	9.56E-09	4.45E-08	7.60E-08
**48h**	1.56E-07	2.17E-07	4.54E-07	7.27E-07
**72h**	9.81E-08	1.76E-08	5.00E-07	4.02E-08
	cgd3_4240			
**2h**	9.59E-08	7.48E-08	0.00E+00	2.43E-07
**6h**	3.58E-09	7.38E-09	3.25E-08	0.00E+00
**12h**	2.26E-08	1.46E-08	3.48E-07	3.67E-07
**24h**	2.15E-08	3.77E-08	2.23E-08	4.58E-07
**36h**	3.56E-08	8.78E-09	2.47E-07	8.08E-08
**48h**	2.45E-08	2.25E-08	7.47E-08	5.51E-08
**72h**	1.04E-08	5.02E-09	6.74E-08	5.67E-09
	cgd3_4260			
**2h**	1.87E-04	1.75E-04	5.33E-03	5.71E-04
**6h**	1.05E-04	9.21E-05	5.09E-04	4.19E-04

**12h**	1.60E-04	6.08E-05	6.98E-04	7.84E-04
**24h**	9.61E-05	5.96E-05	1.92E-04	2.93E-04
**36h**	1.26E-04	4.56E-05	1.10E-03	2.56E-04
**48h**	1.17E-04	5.66E-05	3.27E-04	1.59E-04
**72h**	2.42E-05	8.04E-06	9.12E-05	1.41E-05
	cgd4_400			
**2h**	0.00E+00	0.00E+00	0.00E+00	0.00E+00
**6h**	0.00E+00	0.00E+00	6.28E-09	0.00E+00
**12h**	6.69E-09	0.00E+00	8.07E-09	7.21E-09
**24h**	1.25E-09	2.24E-09	4.60E-09	2.75E-08
**36h**	5.68E-08	3.57E-08	7.40E-08	7.95E-08
**48h**	1.56E-07	9.88E-08	6.24E-07	3.68E-07
**72h**	6.83E-08	3.43E-08	7.15E-07	5.33E-08
	cgd4_800			
**2h**	0.00E+00	0.00E+00	0.00E+00	0.00E+00
**6h**	0.00E+00	2.87E-08	5.30E-08	3.20E-07
**12h**	3.75E-08	2.95E-08	1.31E-07	2.56E-07
**24h**	1.44E-08	8.45E-09	1.29E-07	2.06E-07
**36h**	1.71E-08	2.35E-08	4.60E-08	2.31E-07
**48h**	2.93E-08	3.94E-08	1.09E-07	1.04E-07
**72h**	1.52E-08	1.06E-08	3.20E-08	1.65E-08
	cgd4_870			
**2h**	0.00E+00	0.00E+00	0.00E+00	6.58E-08
**6h**	1.31E-08	2.80E-09	3.82E-08	0.00E+00
**12h**	6.00E-07	1.19E-07	7.57E-07	2.03E-06
**24h**	3.29E-07	2.17E-07	4.40E-07	4.74E-07
**36h**	2.38E-07	7.77E-07	2.79E-06	2.58E-06
**48h**	1.51E-07	5.64E-07	4.29E-07	1.64E-06
**72h**	9.25E-08	6.79E-07	4.03E-07	1.97E-07
	cgd4_970			
**2h**	0.00E+00	0.00E+00	0.00E+00	0.00E+00
**6h**	1.61E-08	3.21E-08	6.50E-07	3.02E-06
**12h**	1.40E-07	7.98E-08	1.18E-06	3.61E-06
**24h**	2.15E-07	7.46E-07	4.36E-06	7.46E-06
**36h**	3.95E-07	2.95E-07	1.82E-06	3.64E-06
**48h**	8.19E-08	3.53E-07	4.90E-07	4.03E-07
**72h**	7.06E-08	1.15E-07	1.25E-07	6.99E-07
	cgd3_2640			
**2h**	1.97E-04	8.22E-05	6.28E-05	5.75E-04
**6h**	3.41E-05	9.17E-05	1.24E-04	1.81E-04
**12h**	4.08E-05	8.73E-05	1.08E-03	1.62E-03
**24h**	9.50E-05	1.97E-04	1.34E-04	2.95E-04
**36h**	4.17E-05	8.54E-05	1.43E-04	3.23E-04
**48h**	2.40E-05	8.74E-05	1.42E-04	1.13E-04
**72h**	7.75E-06	5.03E-05	3.39E-05	2.30E-05
	cgd3_2740			
**2h**	5.50E-08	2.19E-08	2.68E-08	0.00E+00
**6h**	4.18E-08	3.56E-08	5.79E-08	3.31E-07
**12h**	4.41E-08	3.70E-08	4.27E-07	7.19E-07
**24h**	1.68E-08	1.84E-08	5.06E-08	2.34E-07
**36h**	1.03E-07	5.00E-08	7.98E-07	6.58E-07
**48h**	2.38E-08	5.74E-08	1.54E-07	1.35E-07
**72h**	2.94E-08	7.15E-08	1.07E-07	6.33E-08

	cgd3_2750			
**2h**	6.10E-08	4.88E-09	4.43E-09	0.00E+00
**6h**	2.34E-08	1.61E-08	0.00E+00	2.55E-08
**12h**	1.02E-09	5.60E-09	8.79E-09	4.20E-08
**24h**	2.51E-09	3.90E-09	3.07E-09	3.82E-09
**36h**	5.75E-09	1.24E-08	7.85E-08	1.96E-08
**48h**	2.94E-09	4.69E-09	2.11E-08	3.70E-08
**72h**	2.43E-08	3.40E-08	3.52E-08	2.25E-08
	cgd3_2870			
**2h**	2.70E-07	1.66E-08	7.29E-08	9.77E-07
**6h**	5.15E-08	1.02E-07	2.14E-07	4.15E-07
**12h**	2.42E-08	2.19E-08	2.88E-07	4.80E-07
**24h**	1.76E-08	3.12E-08	1.84E-07	4.97E-07
**36h**	3.70E-08	8.68E-08	3.41E-07	3.46E-07
**48h**	1.32E-07	2.90E-07	6.97E-07	4.45E-07
**72h**	2.42E-08	2.87E-08	5.06E-08	8.31E-08
	cgd3_2920			
**2h**	5.93E-07	3.87E-07	1.44E-08	5.00E-07
**6h**	3.58E-07	1.44E-06	1.69E-06	8.81E-06
**12h**	2.77E-08	7.06E-08	3.34E-07	1.68E-06
**24h**	1.25E-08	1.09E-07	8.28E-07	3.60E-06
**36h**	3.96E-07	2.99E-07	7.10E-07	2.02E-06
**48h**	1.79E-07	7.47E-07	4.10E-07	7.03E-07
**72h**	4.91E-08	3.20E-07	5.27E-07	7.24E-07
	cgd3_2950			
**2h**	2.82E-06	6.61E-07	7.62E-07	5.11E-06
**6h**	2.06E-07	8.31E-07	8.82E-07	1.74E-06
**12h**	5.27E-07	6.10E-07	3.40E-06	1.17E-05
**24h**	2.99E-07	3.16E-07	2.57E-06	3.29E-06
**36h**	4.78E-07	4.42E-07	9.84E-07	2.53E-06
**48h**	2.01E-07	3.25E-07	7.97E-07	1.59E-06
**72h**	4.68E-08	1.82E-07	1.46E-07	1.40E-07
	cgd3_2960			
**2h**	0.00E+00	0.00E+00	0.00E+00	0.00E+00
**6h**	1.73E-09	0.00E+00	1.39E-08	0.00E+00
**12h**	5.93E-09	2.36E-09	4.49E-08	9.50E-08
**24h**	6.44E-09	8.44E-09	1.18E-07	1.84E-07
**36h**	5.42E-08	6.10E-08	1.16E-07	3.53E-07
**48h**	1.29E-07	1.17E-07	1.57E-07	3.83E-07
**72h**	1.60E-09	8.69E-08	8.42E-09	9.96E-09
	cgd3_2970			
**2h**	1.06E-07	0.00E+00	0.00E+00	1.82E-08
**6h**	2.63E-08	0.00E+00	2.21E-09	1.87E-08
**12h**	0.00E+00	0.00E+00	2.64E-08	9.43E-09
**24h**	8.57E-09	2.71E-08	4.86E-08	1.30E-07
**36h**	1.23E-07	1.85E-07	4.62E-07	3.93E-07
**48h**	7.09E-07	1.54E-06	1.30E-06	2.16E-06
**72h**	5.39E-08	8.79E-07	8.28E-08	8.52E-08
	cgd3_3050			
**2h**	3.03E-08	2.90E-09	7.50E-08	6.90E-08
**6h**	4.37E-08	3.00E-08	7.62E-08	1.41E-07
**12h**	1.67E-07	6.82E-08	2.45E-07	3.59E-07
**24h**	7.76E-08	6.22E-08	1.76E-07	4.71E-07

**36h**	6.64E-08	1.68E-07	4.93E-07	6.14E-07
**48h**	2.70E-07	1.05E-06	7.24E-07	1.13E-06
**72h**	1.80E-08	1.35E-07	8.79E-08	6.89E-08
	cgd4_380			
**2h**	0.00E+00	7.20E-07	1.63E-06	7.37E-07
**6h**	1.68E-06	2.56E-06	5.85E-07	1.98E-06
**12h**	2.73E-07	3.49E-07	1.70E-06	8.85E-07
**24h**	1.87E-06	6.09E-07	2.20E-06	8.51E-07
**36h**	1.94E-07	1.50E-07	8.59E-07	6.29E-07
**48h**	2.57E-07	1.64E-07	4.79E-07	1.95E-07
**72h**	4.87E-08	3.66E-08	2.80E-07	1.07E-07
	cgd4_350			
**2h**	3.39E-08	5.64E-08	9.11E-08	1.04E-08
**6h**	7.92E-08	1.64E-07	1.39E-07	3.99E-08
**12h**	2.67E-09	4.57E-09	1.10E-07	2.06E-07
**24h**	1.35E-07	2.39E-08	7.21E-07	5.47E-07
**36h**	6.51E-08	6.10E-08	8.11E-07	4.84E-07
**48h**	8.63E-08	4.51E-08	1.50E-07	1.51E-07
**72h**	2.29E-08	5.09E-08	1.08E-07	3.78E-08
	cgd4_430			
**2h**	7.82E-08	4.60E-07	2.04E-07	1.09E-06
**6h**	2.96E-05	2.99E-05	1.96E-04	4.56E-04
**12h**	1.77E-06	1.03E-06	4.38E-05	5.59E-05
**24h**	6.91E-06	5.23E-06	2.42E-04	2.94E-04
**36h**	7.57E-06	1.23E-05	2.69E-04	9.78E-05
**48h**	2.82E-06	3.00E-06	1.77E-04	3.14E-04
**72h**	7.38E-06	9.31E-06	4.70E-05	2.09E-05
	cgd4_1320			
**2h**	0.00E+00	7.23E-09	0.00E+00	1.42E-07
**6h**	1.72E-08	2.58E-09	0.00E+00	3.65E-08
**12h**	1.76E-08	1.69E-08	4.56E-08	2.65E-07
**24h**	3.00E-08	4.26E-09	2.26E-07	5.14E-08
**36h**	1.71E-08	3.48E-08	3.76E-07	2.51E-07
**48h**	7.83E-08	2.98E-08	2.52E-07	1.84E-07
**72h**	1.24E-07	1.83E-07	1.13E-06	4.80E-07
	cgd4_1330			
**2h**	3.13E-08	4.39E-08	2.87E-07	4.36E-08
**6h**	1.69E-07	3.67E-08	5.79E-08	3.16E-06
**12h**	9.84E-09	2.75E-08	2.35E-07	2.98E-07
**24h**	1.71E-07	2.47E-08	1.93E-06	5.04E-07
**36h**	6.56E-08	1.36E-07	8.38E-07	6.34E-07
**48h**	1.41E-07	9.76E-08	6.46E-07	1.03E-06
**72h**	1.39E-07	1.85E-07	9.38E-07	5.52E-07
	cgd4_1340			
**2h**	7.02E-08	2.99E-07	1.21E-06	5.94E-08
**6h**	1.95E-07	6.98E-08	1.64E-07	6.47E-07
**12h**	2.12E-08	6.75E-09	3.10E-07	8.63E-07
**24h**	6.77E-08	2.31E-08	8.75E-07	1.13E-06
**36h**	8.45E-08	2.14E-07	1.96E-06	1.34E-06
**48h**	3.58E-08	8.67E-08	1.52E-06	1.04E-06
**72h**	5.80E-08	6.35E-08	1.85E-07	3.80E-08
	cgd4_1350			
**2h**	4.75E-08	1.15E-07	2.37E-06	1.74E-07

**6h**	6.11E-07	4.04E-07	1.56E-06	3.50E-06
**12h**	1.20E-07	7.29E-07	1.87E-06	4.70E-06
**24h**	6.58E-08	6.73E-08	2.14E-06	2.98E-06
**36h**	2.95E-07	5.95E-07	4.05E-06	2.84E-06
**48h**	5.85E-08	2.25E-07	2.73E-06	1.04E-06
**72h**	6.59E-08	4.82E-08	1.19E-07	8.59E-08
	cgd4_1370			
**2h**	8.48E-07	2.81E-06	1.33E-05	7.66E-06
**6h**	1.47E-06	1.17E-06	5.91E-06	4.32E-06
**12h**	1.25E-07	1.16E-07	2.41E-06	8.09E-06
**24h**	9.82E-07	5.13E-07	6.22E-06	3.75E-06
**36h**	3.47E-07	7.03E-07	2.59E-05	2.95E-06
**48h**	1.46E-07	3.23E-07	2.74E-06	1.88E-06
**72h**	4.15E-07	1.74E-07	1.20E-06	1.59E-07
	cgd4_1380			
**2h**	1.02E-05	2.11E-05	9.60E-05	1.18E-05
**6h**	1.19E-05	1.29E-05	2.30E-05	1.35E-04
**12h**	5.45E-07	2.54E-09	8.24E-05	8.48E-05
**24h**	6.29E-06	4.82E-06	5.85E-05	6.63E-05
**36h**	4.47E-06	6.79E-06	1.76E-04	2.18E-05
**48h**	2.96E-06	2.48E-06	2.01E-05	1.46E-05
**72h**	5.07E-07	1.86E-06	1.80E-06	2.06E-06
	cgd4_1390			
**2h**	3.62E-09	3.80E-09	1.22E-08	7.19E-08
**6h**	2.62E-07	5.15E-07	1.35E-06	2.37E-06
**12h**	1.00E-08	4.34E-09	3.11E-08	7.95E-08
**24h**	4.29E-08	7.03E-08	2.25E-06	2.52E-06
**36h**	9.00E-08	1.16E-07	5.25E-07	4.12E-07
**48h**	6.84E-08	9.18E-08	3.83E-07	4.68E-07
**72h**	3.61E-09	1.47E-09	6.57E-09	3.25E-09
	cgd4_1400			
**2h**	4.83E-07	3.21E-06	2.99E-06	8.05E-06
**6h**	7.38E-07	1.00E-06	5.62E-06	1.44E-05
**12h**	2.97E-07	2.48E-07	1.79E-06	3.97E-06
**24h**	5.87E-07	3.96E-07	4.92E-06	5.28E-06
**36h**	8.29E-07	6.74E-07	2.55E-06	1.94E-06
**48h**	2.49E-07	5.23E-07	1.44E-06	2.52E-06
**72h**	1.78E-07	4.89E-08	2.73E-07	1.27E-07
	cgd4_1420			
**2h**	2.17E-07	9.94E-07	1.25E-06	3.17E-06
**6h**	1.43E-07	6.11E-07	1.07E-06	3.13E-06
**12h**	3.11E-07	9.88E-08	7.14E-07	1.34E-06
**24h**	1.48E-07	2.10E-07	2.34E-06	1.25E-06
**36h**	3.43E-07	1.52E-07	5.37E-07	3.71E-07
**48h**	1.04E-07	1.30E-07	6.32E-07	2.98E-07
**72h**	7.09E-08	5.59E-08	1.84E-07	6.24E-08
	cgd4_1430			
**2h**	6.12E-08	1.85E-07	1.22E-07	4.29E-07
**6h**	3.17E-07	3.51E-07	1.09E-06	1.10E-06
**12h**	2.19E-09	5.96E-09	1.84E-07	3.77E-07
**24h**	8.08E-08	6.64E-08	3.27E-07	7.42E-07
**36h**	6.54E-08	1.23E-07	2.78E-07	8.04E-08
**48h**	1.03E-07	3.28E-08	3.46E-07	4.49E-07

**72h**	2.25E-08	5.58E-08	1.13E-07	2.22E-08
	cgd4_1440			
**2h**	4.80E-07	5.38E-07	2.20E-06	2.13E-06
**6h**	1.56E-06	2.80E-06	5.25E-06	1.12E-05
**12h**	1.42E-08	4.05E-09	5.99E-07	3.60E-06
**24h**	5.06E-07	7.18E-07	1.41E-05	9.87E-06
**36h**	1.04E-06	1.20E-06	6.63E-06	5.33E-06
**48h**	1.09E-06	5.58E-07	8.58E-06	1.16E-05
**72h**	5.84E-07	6.74E-07	1.00E-05	1.02E-06
	cgd4_1450			
**2h**	4.32E-09	0.00E+00	0.00E+00	2.03E-08
**6h**	1.54E-08	0.00E+00	9.39E-09	0.00E+00
**12h**	1.27E-09	7.77E-10	0.00E+00	3.75E-09
**24h**	2.25E-09	6.24E-10	5.90E-09	9.05E-09
**36h**	1.37E-08	1.61E-08	2.65E-08	2.53E-08
**48h**	3.80E-08	2.73E-08	9.00E-08	1.43E-07
**72h**	3.45E-08	2.33E-08	1.93E-07	8.56E-08
	cgd4_1470			
**2h**	6.61E-07	2.99E-06	5.14E-06	2.44E-06
**6h**	2.48E-06	1.01E-06	5.62E-06	6.37E-06
**12h**	1.80E-07	4.19E-07	1.42E-05	1.30E-05
**24h**	2.10E-06	1.09E-06	2.83E-06	3.80E-06
**36h**	9.53E-07	1.53E-06	9.33E-06	5.97E-06
**48h**	4.80E-07	3.51E-07	2.00E-06	8.65E-07
**72h**	1.01E-06	5.05E-07	2.43E-06	8.44E-07
	cgd4_1480			
**2h**	3.46E-04	1.97E-03	6.44E-03	2.11E-02
**6h**	2.83E-04	2.89E-04	1.67E-03	5.44E-03
**12h**	2.10E-04	2.29E-04	9.50E-03	2.69E-02
**24h**	4.94E-04	1.02E-03	6.40E-03	6.44E-03
**36h**	2.05E-04	3.25E-04	7.03E-03	7.19E-03
**48h**	3.72E-04	1.99E-04	2.32E-03	4.57E-03
**72h**	4.15E-05	6.51E-05	6.02E-04	5.36E-05
	cgd4_1500			
**2h**	1.40E-06	1.66E-05	1.28E-03	1.42E-04
**6h**	1.66E-05	3.07E-05	1.94E-03	1.31E-02
**12h**	3.11E-07	2.46E-06	1.83E-04	3.91E-04
**24h**	8.08E-06	2.76E-05	3.13E-04	4.93E-04
**36h**	7.20E-05	4.61E-05	4.86E-04	6.91E-04
**48h**	5.00E-05	5.66E-05	1.00E-03	1.05E-03
**72h**	1.83E-05	1.84E-05	5.77E-05	4.84E-05
	cgd4_1580			
**2h**	8.41E-06	1.94E-05	1.47E-04	1.60E-04
**6h**	1.05E-06	3.99E-06	3.76E-05	3.30E-05
**12h**	3.45E-06	4.14E-06	3.63E-04	2.02E-04
**24h**	9.11E-06	1.33E-05	5.53E-05	3.25E-05
**36h**	6.20E-06	5.79E-06	5.68E-05	3.95E-05
**48h**	2.03E-06	2.59E-06	2.88E-05	2.33E-05
**72h**	1.31E-06	7.17E-07	1.23E-05	1.54E-06
	cgd4_1600			
**2h**	0.00E+00	1.00E-08	2.30E-08	0.00E+00
**6h**	0.00E+00	1.29E-09	0.00E+00	0.00E+00
**12h**	2.44E-08	2.56E-08	3.66E-07	1.08E-06

**24h**	1.75E-08	7.95E-08	1.88E-07	2.87E-07
**36h**	0.00E+00	1.86E-07	2.27E-05	2.25E-06
**48h**	1.54E-07	4.46E-07	1.01E-06	1.21E-06
**72h**	1.36E-07	2.85E-08	2.12E-07	2.35E-07
	cgd4_1610			
**2h**	0.00E+00	2.23E-09	2.19E-08	0.00E+00
**6h**	0.00E+00	0.00E+00	0.00E+00	0.00E+00
**12h**	3.62E-09	3.62E-09	4.59E-08	7.25E-08
**24h**	3.87E-09	1.64E-08	3.45E-08	5.91E-08
**36h**	7.47E-08	2.73E-08	2.19E-07	3.31E-07
**48h**	5.54E-08	9.25E-08	5.19E-07	5.23E-07
**72h**	8.13E-08	1.29E-08	9.07E-08	1.01E-07
	cgd4_1620			
**2h**	1.91E-05	2.80E-05	6.33E-06	1.24E-04
**6h**	0.00E+00	1.33E-07	0.00E+00	0.00E+00
**12h**	0.00E+00	0.00E+00	2.89E-06	3.79E-05
**24h**	1.26E-07	3.17E-07	1.62E-05	6.74E-06
**36h**	1.49E-06	2.04E-06	9.38E-06	2.67E-06
**48h**	7.92E-05	3.39E-05	1.81E-04	3.07E-04
**72h**	3.44E-06	2.06E-05	3.01E-05	7.09E-05
	cgd4_1680			
**2h**	7.04E-08	1.95E-08	4.50E-08	2.76E-07
**6h**	3.72E-08	1.45E-07	2.21E-07	4.55E-07
**12h**	1.31E-08	1.48E-08	2.71E-07	4.11E-07
**24h**	3.92E-08	1.93E-08	1.45E-07	2.20E-07
**36h**	1.01E-07	3.45E-08	3.16E-07	2.28E-07
**48h**	1.09E-07	6.07E-08	1.33E-07	5.13E-07
**72h**	5.06E-08	5.27E-09	1.86E-08	8.77E-09
	cgd4_1690			
**2h**	0.00E+00	0.00E+00	0.00E+00	0.00E+00
**6h**	0.00E+00	0.00E+00	0.00E+00	0.00E+00
**12h**	9.94E-08	1.07E-06	9.08E-06	2.63E-05
**24h**	6.75E-07	9.20E-07	6.07E-06	1.31E-05
**36h**	9.44E-06	3.75E-06	4.06E-05	1.50E-05
**48h**	3.38E-06	1.61E-06	1.15E-05	3.15E-05
**72h**	4.59E-06	8.51E-06	7.01E-06	4.33E-06
	cgd4_1720			
**2h**	5.83E-07	2.53E-07	0.00E+00	3.22E-06
**6h**	1.44E-06	1.98E-06	1.84E-05	1.19E-05
**12h**	1.57E-07	3.50E-08	3.77E-06	4.54E-06
**24h**	1.41E-06	7.05E-07	2.75E-05	1.12E-05
**36h**	4.67E-07	2.97E-07	6.24E-06	3.52E-06
**48h**	1.02E-06	3.07E-07	1.00E-05	2.68E-06
**72h**	1.31E-07	2.23E-07	3.10E-06	1.08E-06
	cgd4_1730			
**2h**	2.51E-08	2.64E-08	8.78E-09	0.00E+00
**6h**	2.62E-07	2.93E-07	1.01E-06	7.04E-07
**12h**	8.08E-10	2.31E-09	7.74E-08	2.72E-07
**24h**	4.54E-08	3.07E-08	1.09E-06	4.31E-07
**36h**	8.10E-08	4.55E-08	2.08E-07	2.00E-07
**48h**	7.74E-08	8.55E-08	3.28E-07	1.34E-07
**72h**	3.61E-08	8.80E-08	2.09E-07	1.61E-07
	cgd4_1770			

**2h**	5.50E-08	1.32E-08	2.66E-07	7.90E-07
**6h**	2.83E-07	2.25E-07	1.14E-06	1.39E-06
**12h**	2.08E-08	2.69E-08	1.07E-07	5.28E-07
**24h**	4.66E-08	4.82E-08	8.23E-07	8.98E-07
**36h**	5.70E-08	1.10E-07	6.35E-07	1.30E-06
**48h**	5.17E-08	5.15E-08	2.20E-07	3.08E-07
**72h**	1.55E-08	5.59E-08	1.45E-07	4.44E-08
	cgd4_1780			
**2h**	0.00E+00	0.00E+00	0.00E+00	0.00E+00
**6h**	0.00E+00	0.00E+00	0.00E+00	0.00E+00
**12h**	4.59E-09	2.88E-09	7.52E-09	2.85E-08
**24h**	6.12E-10	1.72E-09	2.86E-08	4.20E-08
**36h**	2.29E-08	1.63E-08	1.41E-07	1.24E-07
**48h**	6.44E-09	3.30E-09	1.13E-08	4.64E-08
**72h**	2.04E-08	1.05E-08	4.21E-08	1.15E-08
	cgd4_1790			
**2h**	0.00E+00	0.00E+00	0.00E+00	0.00E+00
**6h**	6.42E-06	1.51E-07	4.87E-07	4.88E-06
**12h**	4.78E-06	9.41E-05	1.35E-04	1.34E-04
**24h**	2.99E-06	1.49E-06	7.70E-06	4.52E-05
**36h**	3.52E-06	4.08E-06	1.29E-04	6.70E-05
**48h**	3.85E-06	5.04E-06	6.25E-06	2.18E-05
**72h**	4.79E-06	1.66E-06	4.03E-06	3.95E-05
	cgd4_1800			
**2h**	0.00E+00	0.00E+00	0.00E+00	0.00E+00
**6h**	6.75E-06	5.65E-06	2.09E-05	2.43E-05
**12h**	4.72E-07	1.39E-06	5.06E-06	6.56E-06
**24h**	2.18E-06	9.62E-07	1.95E-05	1.40E-04
**36h**	9.11E-06	4.85E-06	8.22E-05	2.96E-05
**48h**	2.29E-05	1.34E-05	8.71E-05	1.09E-04
**72h**	3.32E-05	3.77E-05	1.71E-04	7.44E-05
	cgd4_1840			
**2h**	4.38E-07	1.00E-06	4.25E-06	3.24E-06
**6h**	2.25E-07	1.99E-07	7.65E-07	8.35E-07
**12h**	4.17E-07	5.37E-07	3.69E-06	6.97E-06
**24h**	2.47E-07	3.27E-07	8.98E-07	3.09E-06
**36h**	4.09E-07	5.21E-07	3.37E-06	1.75E-06
**48h**	1.23E-07	1.09E-07	6.85E-07	1.36E-06
**72h**	1.58E-07	1.62E-07	5.80E-07	1.19E-07
	cgd4_1850			
**2h**	1.21E-05	2.59E-06	1.16E-05	8.08E-07
**6h**	6.20E-06	2.50E-05	6.90E-04	1.10E-03
**12h**	1.69E-07	0.00E+00	8.45E-08	3.45E-06
**24h**	3.21E-06	5.35E-06	1.33E-04	2.63E-04
**36h**	4.28E-05	1.89E-05	6.48E-05	9.39E-05
**48h**	9.49E-06	1.76E-05	2.32E-04	1.83E-04
**72h**	9.10E-06	5.44E-06	5.93E-06	1.80E-06
	cgd4_1860			
**2h**	9.71E-09	5.40E-08	3.47E-08	4.11E-08
**6h**	1.08E-08	1.46E-08	4.00E-08	2.85E-07
**12h**	3.01E-08	3.05E-08	4.21E-07	4.19E-07
**24h**	1.50E-08	1.32E-08	1.27E-07	6.52E-07
**36h**	8.24E-08	1.02E-07	6.71E-07	3.40E-07

**48h**	5.90E-08	7.28E-08	1.73E-07	3.39E-07
**72h**	2.02E-08	8.85E-09	8.46E-08	1.33E-08
	cgd4_1870			
**2h**	3.01E-09	0.00E+00	0.00E+00	0.00E+00
**6h**	0.00E+00	0.00E+00	0.00E+00	0.00E+00
**12h**	0.00E+00	0.00E+00	8.76E-08	0.00E+00
**24h**	2.96E-09	2.20E-09	2.96E-08	6.51E-09
**36h**	3.98E-08	5.27E-09	1.35E-07	2.35E-08
**48h**	1.22E-07	6.52E-08	7.46E-07	1.53E-07
**72h**	1.36E-08	1.64E-08	2.31E-07	8.12E-09
	cgd4_1880			
**2h**	0.00E+00	3.32E-05	0.00E+00	1.47E-05
**6h**	4.40E-05	0.00E+00	0.00E+00	0.00E+00
**12h**	0.00E+00	0.00E+00	8.39E-05	0.00E+00
**24h**	4.05E-06	7.54E-06	0.00E+00	4.12E-06
**36h**	1.79E-05	3.86E-05	7.20E-05	2.37E-04
**48h**	4.46E-04	6.24E-04	9.23E-04	2.59E-03
**72h**	3.98E-04	3.42E-04	3.18E-03	7.26E-04
	cgd4_1890			
**2h**	6.00E-06	9.14E-06	5.13E-05	1.97E-05
**6h**	7.75E-07	5.21E-07	1.80E-06	4.03E-06
**12h**	5.61E-07	4.75E-07	6.28E-06	5.64E-06
**24h**	2.07E-06	1.46E-06	6.61E-06	8.25E-06
**36h**	1.34E-06	1.00E-06	5.49E-06	2.20E-06
**48h**	1.06E-06	1.27E-06	4.01E-06	1.87E-06
**72h**	6.33E-07	5.78E-07	2.47E-06	2.86E-06
	cgd4_1910			
**2h**	0.00E+00	0.00E+00	2.14E-06	4.07E-07
**6h**	0.00E+00	0.00E+00	4.54E-08	0.00E+00
**12h**	9.60E-08	2.15E-08	0.00E+00	3.24E-07
**24h**	6.24E-08	3.63E-07	2.71E-06	1.04E-06
**36h**	1.26E-06	5.05E-07	3.27E-05	4.26E-06
**48h**	1.28E-04	4.64E-05	1.69E-03	1.84E-03
**72h**	7.46E-05	1.18E-04	4.83E-04	1.77E-04
	cgd4_1920			
**2h**	4.05E-05	3.06E-05	1.47E-04	1.37E-04
**6h**	1.61E-04	1.23E-04	7.98E-04	9.95E-04
**12h**	4.17E-06	1.76E-06	2.47E-04	2.27E-04
**24h**	2.73E-05	2.16E-05	9.09E-04	8.03E-04
**36h**	3.05E-05	2.85E-05	2.93E-04	1.29E-04
**48h**	2.10E-05	1.23E-05	1.90E-04	9.37E-05
**72h**	4.12E-06	3.60E-06	1.83E-05	1.20E-05
	cgd4_1930			
**2h**	5.02E-09	0.00E+00	6.61E-08	2.75E-08
**6h**	1.95E-08	3.64E-08	1.45E-07	4.90E-07
**12h**	2.46E-08	0.00E+00	1.77E-09	9.08E-09
**24h**	1.84E-08	9.31E-09	2.20E-07	4.86E-07
**36h**	3.13E-08	5.02E-08	1.59E-07	7.79E-08
**48h**	4.39E-08	9.02E-09	1.22E-07	1.04E-07
**72h**	5.02E-08	3.16E-08	1.22E-07	4.78E-08
	cgd4_1950			
**2h**	6.50E-05	3.68E-05	1.60E-04	9.60E-05
**6h**	1.02E-04	2.79E-05	1.43E-04	3.28E-04

**12h**	2.63E-05	2.19E-05	1.93E-04	4.94E-04
**24h**	4.05E-05	1.17E-05	3.09E-04	2.72E-04
**36h**	6.45E-05	2.09E-05	4.05E-04	2.18E-04
**48h**	2.05E-05	1.58E-05	3.03E-04	1.63E-04
**72h**	2.27E-05	1.32E-05	2.75E-04	4.47E-05
	cgd4_1960			
**2h**	5.96E-08	1.30E-07	7.67E-08	3.52E-08
**6h**	1.98E-07	3.08E-08	4.96E-07	5.13E-07
**12h**	5.58E-08	2.91E-08	2.44E-07	2.60E-07
**24h**	6.28E-08	5.44E-08	3.91E-07	2.91E-07
**36h**	1.66E-07	1.33E-07	6.69E-07	6.55E-07
**48h**	5.58E-08	4.13E-08	1.79E-07	1.41E-07
**72h**	8.77E-08	4.83E-08	2.21E-07	1.39E-07
	cgd4_1970			
**2h**	2.85E-06	4.59E-07	5.20E-06	4.69E-07
**6h**	4.65E-06	1.68E-06	3.52E-05	1.59E-05
**12h**	4.80E-04	2.93E-04	2.43E-03	1.84E-03
**24h**	4.20E-05	2.99E-05	7.06E-05	3.24E-04
**36h**	3.88E-05	2.32E-05	9.33E-04	8.52E-04
**48h**	5.36E-05	1.99E-05	2.24E-04	1.22E-04
**72h**	1.18E-05	1.26E-05	7.03E-05	2.75E-05
	cgd4_1980			
**2h**	2.41E-08	4.58E-08	3.88E-07	1.36E-08
**6h**	1.92E-08	2.61E-08	5.30E-07	2.72E-07
**12h**	1.24E-06	1.99E-06	4.34E-05	3.35E-06
**24h**	4.60E-07	5.68E-07	9.25E-07	2.30E-06
**36h**	2.55E-06	1.09E-06	7.62E-05	4.83E-06
**48h**	7.62E-07	4.80E-07	3.15E-06	2.50E-06
**72h**	1.01E-06	4.44E-07	3.36E-06	2.76E-06
	cgd4_2000			
**2h**	0.00E+00	0.00E+00	0.00E+00	0.00E+00
**6h**	0.00E+00	4.07E-09	0.00E+00	0.00E+00
**12h**	9.46E-06	8.61E-06	5.99E-05	5.61E-05
**24h**	1.01E-06	2.47E-06	1.89E-05	4.16E-06
**36h**	2.23E-06	1.96E-06	1.00E-04	3.40E-05
**48h**	4.91E-07	8.34E-07	8.72E-06	4.49E-05
**72h**	5.84E-07	4.00E-07	4.89E-06	4.13E-06
	cgd4_2010			
**2h**	2.85E-07	3.19E-07	5.72E-07	1.25E-07
**6h**	6.26E-08	4.83E-08	4.27E-07	9.20E-08
**12h**	1.16E-07	7.96E-08	1.61E-07	6.55E-07
**24h**	1.77E-07	2.74E-07	2.08E-07	5.82E-07
**36h**	3.45E-07	9.04E-08	4.56E-07	8.43E-07
**48h**	1.45E-07	1.54E-07	5.49E-07	5.65E-07
**72h**	3.99E-08	1.81E-08	5.64E-07	1.90E-07
	cgd4_2030			
**2h**	3.42E-09	0.00E+00	1.53E-09	0.00E+00
**6h**	0.00E+00	0.00E+00	1.73E-08	2.28E-08
**12h**	1.76E-08	1.66E-08	6.77E-08	2.71E-08
**24h**	5.17E-09	1.70E-08	4.61E-08	5.29E-08
**36h**	2.84E-08	1.24E-08	1.03E-07	1.92E-07
**48h**	5.13E-08	2.49E-08	1.84E-07	6.28E-08
**72h**	7.12E-09	7.26E-09	3.55E-08	5.03E-08

	cgd4_2070			
**2h**	0.00E+00	0.00E+00	0.00E+00	0.00E+00
**6h**	1.38E-09	0.00E+00	0.00E+00	0.00E+00
**12h**	0.00E+00	0.00E+00	0.00E+00	0.00E+00
**24h**	4.61E-10	2.27E-09	3.47E-09	1.16E-09
**36h**	6.51E-09	8.04E-10	1.41E-08	6.31E-09
**48h**	1.24E-08	5.95E-09	4.13E-08	2.73E-08
**72h**	1.31E-08	6.13E-09	2.35E-07	1.79E-08
	cgd4_2080			
**2h**	0.00E+00	0.00E+00	0.00E+00	0.00E+00
**6h**	7.16E-08	3.64E-07	8.33E-08	1.09E-07
**12h**	2.99E-09	5.40E-08	6.74E-08	1.81E-07
**24h**	5.42E-08	6.29E-08	2.35E-06	3.22E-07
**36h**	1.65E-07	6.72E-08	8.26E-07	6.35E-07
**48h**	7.46E-08	4.38E-08	1.99E-07	1.81E-07
**72h**	7.49E-08	3.45E-08	5.47E-07	5.84E-08
	cgd4_2090			
**2h**	0.00E+00	0.00E+00	0.00E+00	0.00E+00
**6h**	1.55E-07	3.78E-07	2.20E-06	1.29E-06
**12h**	1.21E-06	7.77E-07	4.19E-06	3.24E-06
**24h**	3.24E-07	5.09E-07	1.93E-06	9.51E-07
**36h**	2.08E-07	7.30E-08	3.30E-06	1.53E-06
**48h**	2.75E-08	9.79E-08	1.01E-06	5.79E-07
**72h**	2.04E-08	2.63E-08	1.45E-07	4.95E-08
	cgd4_2100			
**2h**	0.00E+00	0.00E+00	0.00E+00	0.00E+00
**6h**	3.93E-08	3.50E-08	2.23E-07	1.15E-06
**12h**	6.87E-09	2.76E-10	7.89E-08	4.34E-08
**24h**	6.88E-09	1.50E-08	4.19E-07	3.22E-07
**36h**	5.42E-08	3.13E-08	4.58E-07	2.04E-07
**48h**	1.90E-08	3.52E-08	1.63E-07	9.71E-08
**72h**	5.42E-08	1.86E-08	1.48E-07	2.60E-06
	cgd4_2120			
**2h**	0.00E+00	0.00E+00	0.00E+00	0.00E+00
**6h**	8.19E-06	1.00E-05	4.48E-05	3.30E-05
**12h**	1.78E-06	8.28E-07	2.10E-05	1.03E-05
**24h**	1.05E-06	1.46E-06	1.81E-05	3.59E-05
**36h**	3.74E-06	3.06E-06	3.29E-05	1.92E-05
**48h**	9.01E-07	1.28E-06	9.62E-06	5.64E-06
**72h**	3.87E-07	1.28E-06	2.24E-05	5.11E-06
	cgd4_2130			
**2h**	1.40E-04	0.00E+00	0.00E+00	0.00E+00
**6h**	2.02E-06	1.18E-06	3.93E-05	1.86E-05
**12h**	5.19E-06	1.76E-06	5.83E-05	9.66E-06
**24h**	6.44E-07	1.11E-06	9.40E-06	1.17E-05
**36h**	6.03E-06	3.82E-06	1.95E-04	2.61E-05
**48h**	1.42E-06	1.87E-06	1.63E-04	7.59E-06
**72h**	1.66E-06	2.86E-06	3.61E-05	4.00E-06
	cgd4_2140			
**2h**	2.23E-08	4.43E-08	1.12E-09	3.05E-08
**6h**	1.65E-09	1.79E-08	4.92E-08	1.51E-07
**12h**	1.11E-06	4.19E-07	2.38E-06	4.99E-06
**24h**	5.44E-08	2.43E-08	1.44E-07	2.79E-07

**36h**	1.48E-07	9.82E-08	1.75E-06	2.09E-06
**48h**	3.57E-08	1.87E-08	1.69E-07	2.36E-07
**72h**	6.00E-08	5.25E-08	3.93E-07	1.05E-07
	cgd4_2150			
**2h**	1.80E-07	4.50E-08	1.64E-08	8.86E-09
**6h**	3.20E-08	6.76E-08	9.65E-08	3.37E-07
**12h**	1.42E-08	1.62E-08	1.25E-07	2.62E-07
**24h**	6.92E-09	6.45E-09	6.15E-08	3.11E-07
**36h**	8.26E-08	4.10E-08	5.85E-07	3.37E-07
**48h**	5.93E-08	4.87E-08	2.23E-07	1.67E-07
**72h**	2.85E-08	6.47E-08	1.37E-07	4.16E-08
	cgd4_2160			
**2h**	1.99E-07	1.14E-07	5.97E-08	3.67E-07
**6h**	1.46E-07	1.64E-07	6.24E-07	1.17E-06
**12h**	1.35E-08	6.48E-09	1.46E-07	2.19E-07
**24h**	2.70E-08	1.86E-08	1.44E-07	4.26E-07
**36h**	1.23E-07	1.02E-07	3.55E-07	2.15E-07
**48h**	4.70E-08	4.16E-08	1.96E-07	1.96E-07
**72h**	1.37E-08	3.30E-08	9.78E-08	3.54E-08
	cgd4_2170			
**2h**	1.84E-07	4.90E-08	2.11E-08	2.04E-07
**6h**	7.59E-08	5.39E-08	6.94E-07	4.48E-07
**12h**	2.17E-09	2.76E-09	1.06E-07	5.40E-08
**24h**	9.41E-09	9.16E-09	2.40E-07	3.06E-07
**36h**	6.48E-08	3.86E-08	7.88E-08	1.78E-07
**48h**	4.29E-08	3.12E-08	1.13E-07	7.01E-08
**72h**	1.08E-08	1.48E-08	1.25E-08	5.68E-09
	cgd4_2180			
**2h**	1.65E-07	1.90E-06	0.00E+00	1.71E-06
**6h**	2.63E-05	3.26E-05	1.21E-04	8.74E-05
**12h**	2.06E-07	4.55E-07	8.15E-06	3.02E-06
**24h**	5.96E-06	2.36E-06	7.68E-05	8.37E-05
**36h**	7.64E-06	8.40E-06	4.27E-05	1.71E-05
**48h**	7.41E-06	1.05E-05	2.71E-05	2.96E-05
**72h**	3.87E-07	1.58E-06	1.47E-05	1.28E-06
	cgd4_2200			
**2h**	0.00E+00	0.00E+00	0.00E+00	0.00E+00
**6h**	1.93E-08	2.70E-09	5.08E-09	1.60E-07
**12h**	8.19E-08	1.81E-08	7.15E-07	6.75E-07
**24h**	8.69E-08	5.37E-08	1.39E-06	2.85E-06
**36h**	2.14E-07	1.65E-07	2.29E-06	7.59E-07
**48h**	1.45E-07	7.87E-07	1.67E-06	1.74E-06
**72h**	1.61E-08	4.81E-08	2.02E-07	6.10E-08
	cgd4_2210			
**2h**	0.00E+00	0.00E+00	0.00E+00	0.00E+00
**6h**	1.58E-07	2.25E-07	1.30E-06	1.91E-06
**12h**	1.03E-08	1.92E-08	7.78E-08	3.82E-08
**24h**	4.35E-08	4.06E-08	1.58E-06	1.95E-06
**36h**	3.14E-07	3.98E-07	1.35E-06	1.09E-06
**48h**	2.29E-07	6.09E-07	1.19E-06	6.57E-06
**72h**	3.75E-08	1.46E-07	1.70E-07	1.57E-07
	cgd4_2220			
**2h**	1.54E-05	1.60E-03	3.09E-05	0.00E+00

**6h**	2.80E-03	4.86E-03	1.55E-02	1.25E-02
**12h**	1.13E-04	3.45E-05	1.59E-03	8.77E-04
**24h**	8.29E-04	5.77E-04	1.85E-03	7.52E-03
**36h**	1.71E-03	1.52E-03	2.06E-03	3.56E-03
**48h**	1.15E-03	1.24E-03	8.34E-04	2.25E-03
**72h**	5.86E-04	9.49E-05	1.04E-03	8.03E-04
	cgd4_2230			
**2h**	8.89E-08	1.79E-07	4.91E-08	5.08E-08
**6h**	2.38E-06	7.36E-07	3.83E-06	4.15E-06
**12h**	2.72E-08	1.25E-08	2.67E-07	2.18E-07
**24h**	4.63E-07	1.96E-07	3.35E-06	1.21E-06
**36h**	2.51E-07	4.41E-07	5.55E-07	2.98E-07
**48h**	2.88E-07	1.12E-07	3.40E-06	1.15E-06
**72h**	7.86E-08	1.14E-07	6.26E-07	6.68E-08
	cgd4_2240			
**2h**	2.99E-06	4.02E-06	1.40E-05	4.89E-06
**6h**	4.52E-05	1.59E-05	4.57E-04	1.41E-04
**12h**	3.52E-07	9.50E-07	2.70E-05	9.84E-06
**24h**	1.28E-05	5.13E-06	2.89E-04	7.81E-05
**36h**	8.94E-06	1.22E-05	7.65E-05	1.68E-05
**48h**	6.94E-06	3.11E-06	1.06E-04	2.17E-05
**72h**	3.09E-06	3.01E-06	2.36E-05	2.43E-06
	cgd4_2250			
**2h**	9.39E-08	1.57E-07	1.63E-07	1.25E-07
**6h**	3.43E-06	2.18E-06	6.58E-06	1.41E-05
**12h**	1.43E-08	1.72E-07	3.64E-07	1.42E-06
**24h**	8.10E-07	5.37E-07	1.76E-05	1.19E-05
**36h**	1.46E-06	2.46E-06	2.01E-05	3.96E-06
**48h**	1.27E-06	7.45E-07	8.76E-06	6.85E-06
**72h**	2.78E-07	5.71E-07	3.72E-07	9.13E-07
	cgd4_2280			
**2h**	1.20E-03	9.73E-04	4.43E-04	1.64E-03
**6h**	2.02E-04	6.48E-04	1.01E-03	7.49E-04
**12h**	9.56E-04	4.02E-03	2.61E-03	4.39E-03
**24h**	4.25E-04	6.43E-04	2.85E-03	2.21E-03
**36h**	2.26E-04	5.21E-04	5.81E-03	4.71E-03
**48h**	3.79E-04	2.70E-04	3.13E-03	1.85E-03
**72h**	2.06E-04	2.52E-04	5.92E-04	2.83E-04
	cgd4_2290			
**2h**	8.08E-08	7.82E-08	2.96E-07	2.93E-07
**6h**	2.11E-07	1.58E-07	1.77E-06	8.46E-07
**12h**	5.85E-08	3.00E-07	5.97E-07	8.00E-07
**24h**	6.59E-08	1.26E-07	6.33E-07	2.67E-07
**36h**	1.37E-07	3.52E-07	1.18E-06	6.12E-07
**48h**	3.96E-08	6.28E-08	5.16E-07	2.84E-07
**72h**	1.27E-07	1.73E-07	6.01E-07	2.32E-07
	cgd4_2310			
**2h**	0.00E+00	0.00E+00	9.29E-09	1.09E-08
**6h**	2.63E-08	4.97E-09	8.45E-08	1.19E-07
**12h**	1.42E-06	5.14E-06	6.27E-06	5.48E-06
**24h**	9.28E-08	1.24E-07	3.77E-07	4.56E-07
**36h**	4.16E-07	6.52E-07	6.37E-06	2.57E-06
**48h**	7.32E-08	1.43E-07	5.08E-07	5.37E-07

**72h**	1.34E-07	9.60E-08	4.16E-07	9.57E-08
	cgd4_2320			
**2h**	0.00E+00	0.00E+00	0.00E+00	0.00E+00
**6h**	3.18E-07	2.04E-07	3.58E-07	3.54E-07
**12h**	2.91E-07	2.17E-07	9.71E-07	1.01E-06
**24h**	9.81E-08	3.00E-07	6.48E-07	6.01E-07
**36h**	6.33E-07	4.74E-07	2.95E-06	2.46E-06
**48h**	2.55E-07	2.08E-07	1.08E-06	8.16E-07
**72h**	1.39E-07	8.20E-08	5.37E-07	1.46E-07
	cgd4_2330			
**2h**	0.00E+00	0.00E+00	0.00E+00	0.00E+00
**6h**	1.81E-08	3.55E-08	3.17E-06	3.51E-07
**12h**	2.61E-06	2.85E-06	1.17E-05	1.35E-05
**24h**	1.64E-07	3.32E-07	7.51E-07	1.58E-06
**36h**	3.16E-07	3.51E-07	5.14E-06	2.22E-06
**48h**	1.64E-07	2.66E-07	1.42E-06	6.84E-07
**72h**	2.83E-07	1.28E-07	7.94E-07	3.75E-07
	cgd4_2340			
**2h**	0.00E+00	0.00E+00	0.00E+00	0.00E+00
**6h**	0.00E+00	0.00E+00	0.00E+00	0.00E+00
**12h**	5.29E-06	1.12E-05	4.01E-05	6.98E-05
**24h**	9.04E-07	2.17E-06	2.41E-06	1.75E-06
**36h**	1.09E-06	6.06E-07	3.17E-05	5.30E-06
**48h**	5.62E-07	5.83E-07	5.69E-06	1.88E-06
**72h**	2.41E-07	2.59E-07	1.02E-06	9.00E-07
	cgd4_2360			
**2h**	7.25E-06	1.15E-05	8.47E-06	1.36E-05
**6h**	2.36E-05	2.69E-05	2.24E-04	1.62E-04
**12h**	3.87E-05	6.51E-05	2.61E-04	4.24E-04
**24h**	2.07E-05	3.37E-05	1.06E-04	1.58E-04
**36h**	9.96E-06	8.90E-06	7.50E-05	5.16E-05
**48h**	1.15E-05	1.13E-05	5.15E-05	4.23E-05
**72h**	1.33E-06	9.10E-07	6.69E-06	1.52E-06
	cgd4_2380			
**2h**	1.20E-07	6.17E-08	2.06E-07	5.58E-08
**6h**	6.92E-08	1.86E-07	1.15E-06	4.93E-07
**12h**	5.23E-08	6.48E-09	3.58E-07	2.22E-07
**24h**	6.59E-08	5.03E-08	3.09E-07	3.32E-07
**36h**	2.77E-07	1.73E-07	1.07E-06	2.14E-07
**48h**	1.31E-07	1.09E-07	7.09E-07	4.10E-07
**72h**	2.12E-08	3.81E-08	2.57E-08	1.75E-08
	cgd4_2390			
**2h**	9.69E-07	8.01E-07	1.61E-06	4.76E-07
**6h**	5.87E-07	3.15E-06	4.29E-06	2.00E-06
**12h**	8.17E-07	8.50E-07	3.11E-06	3.58E-06
**24h**	7.91E-07	1.45E-06	5.82E-06	2.72E-06
**36h**	1.49E-06	5.44E-06	5.33E-06	1.46E-06
**48h**	6.07E-07	1.65E-05	2.79E-06	1.26E-06
**72h**	1.90E-07	1.96E-04	9.36E-07	3.24E-07
	cgd4_2410			
**2h**	8.90E-08	8.32E-08	3.38E-08	1.81E-08
**6h**	6.21E-09	2.68E-08	1.67E-08	1.85E-09
**12h**	4.96E-09	3.23E-09	4.25E-08	1.66E-08

**24h**	4.10E-08	3.22E-08	4.23E-07	3.32E-07
**36h**	2.00E-07	9.75E-08	5.82E-07	4.68E-07
**48h**	2.58E-06	2.41E-06	1.46E-05	1.40E-05
**72h**	3.78E-07	4.19E-07	7.85E-07	2.53E-07
	cgd4_2420			
**2h**	0.00E+00	2.15E-09	8.84E-08	7.23E-08
**6h**	2.38E-08	3.67E-08	5.67E-07	2.66E-07
**12h**	4.13E-07	3.30E-07	4.45E-07	8.56E-07
**24h**	7.44E-08	1.49E-07	1.40E-06	1.64E-06
**36h**	1.01E-07	7.34E-08	7.53E-07	7.12E-07
**48h**	1.62E-07	2.23E-07	1.37E-06	1.28E-06
**72h**	1.32E-07	2.02E-07	4.02E-07	2.64E-07
	cgd4_2470			
**2h**	0.00E+00	0.00E+00	0.00E+00	0.00E+00
**6h**	0.00E+00	0.00E+00	0.00E+00	0.00E+00
**12h**	1.01E-05	4.48E-06	7.48E-06	1.43E-05
**24h**	2.00E-06	2.01E-06	7.76E-06	8.66E-06
**36h**	8.97E-06	3.61E-06	2.45E-05	3.59E-05
**48h**	5.17E-07	6.96E-07	5.49E-06	2.66E-06
**72h**	3.51E-07	1.92E-07	8.80E-07	4.26E-07
	cgd4_2480			
**2h**	0.00E+00	0.00E+00	0.00E+00	0.00E+00
**6h**	2.85E-07	2.96E-07	3.73E-07	3.26E-06
**12h**	6.20E-04	7.61E-04	2.59E-03	2.25E-03
**24h**	5.09E-05	3.86E-05	1.62E-04	2.58E-04
**36h**	5.03E-04	1.30E-04	3.42E-03	7.23E-03
**48h**	2.83E-05	3.86E-05	5.04E-05	1.04E-04
**72h**	6.63E-06	7.31E-06	8.21E-06	9.77E-06
	cgd4_2490			
**2h**	0.00E+00	0.00E+00	0.00E+00	0.00E+00
**6h**	0.00E+00	0.00E+00	0.00E+00	0.00E+00
**12h**	2.15E-08	3.78E-09	4.47E-09	1.14E-08
**24h**	4.05E-09	7.65E-09	2.14E-08	5.33E-09
**36h**	2.68E-08	5.48E-09	7.58E-08	1.26E-07
**48h**	8.65E-09	1.45E-08	6.74E-08	9.84E-08
**72h**	4.65E-08	4.03E-08	1.41E-07	2.67E-08
	cgd4_2500			
**2h**	0.00E+00	0.00E+00	2.12E-09	0.00E+00
**6h**	0.00E+00	2.13E-08	2.10E-08	0.00E+00
**12h**	2.53E-10	0.00E+00	0.00E+00	2.21E-09
**24h**	5.35E-10	3.36E-09	1.60E-08	7.08E-09
**36h**	3.24E-08	1.75E-08	4.24E-08	8.46E-08
**48h**	2.83E-08	3.12E-08	1.79E-07	2.24E-07
**72h**	1.32E-07	6.54E-08	3.44E-07	8.41E-08
	cgd4_2510			
**2h**	9.23E-07	6.37E-07	6.82E-06	4.37E-06
**6h**	1.45E-07	1.41E-07	7.53E-07	3.76E-07
**12h**	1.13E-05	6.09E-06	2.32E-05	0.00E+00
**24h**	9.17E-07	1.43E-06	1.90E-05	1.25E-05
**36h**	4.10E-06	1.83E-06	1.74E-05	4.13E-05
**48h**	9.37E-07	1.12E-06	4.46E-06	1.07E-05
**72h**	1.01E-06	2.52E-06	4.43E-06	2.31E-06
	cgd4_2520			

**2h**	4.12E-07	2.02E-07	1.20E-06	1.13E-06
**6h**	1.98E-07	3.59E-07	9.59E-07	4.08E-07
**12h**	5.03E-06	2.02E-06	3.49E-06	1.53E-05
**24h**	4.07E-07	3.04E-07	1.02E-06	1.88E-06
**36h**	5.66E-07	2.90E-07	2.64E-06	5.04E-06
**48h**	2.21E-07	1.17E-07	4.23E-07	1.12E-06
**72h**	1.22E-07	1.74E-07	5.84E-07	1.68E-07
	cgd4_2550			
**2h**	1.09E-06	4.93E-06	4.21E-06	5.40E-04
**6h**	7.78E-05	8.18E-05	2.52E-04	3.26E-03
**12h**	1.76E-05	1.23E-05	9.59E-05	2.43E-05
**24h**	1.95E-05	1.39E-05	2.13E-04	2.61E-04
**36h**	6.73E-05	2.71E-05	1.29E-04	1.37E-04
**48h**	3.57E-05	2.20E-05	4.89E-05	1.89E-04
**72h**	1.29E-05	1.84E-05	7.25E-05	1.93E-05
	cgd4_2560			
**2h**	1.79E-08	6.32E-09	3.63E-08	1.77E-07
**6h**	0.00E+00	1.02E-09	0.00E+00	0.00E+00
**12h**	1.89E-06	4.81E-07	3.34E-06	9.65E-06
**24h**	3.12E-07	1.63E-07	5.38E-07	1.01E-06
**36h**	3.24E-07	9.89E-08	2.45E-06	1.91E-06
**48h**	2.26E-07	9.00E-08	6.72E-07	1.94E-06
**72h**	1.33E-07	1.17E-07	5.02E-07	2.92E-07
	cgd4_2570			
**2h**	6.46E-06	4.33E-06	1.51E-05	3.10E-05
**6h**	4.98E-07	3.65E-07	1.76E-06	5.64E-07
**12h**	3.80E-06	2.48E-06	2.05E-05	3.68E-05
**24h**	2.11E-06	6.85E-07	3.76E-06	7.96E-06
**36h**	1.07E-06	1.65E-06	9.95E-06	5.65E-06
**48h**	8.37E-07	3.94E-07	1.70E-06	3.95E-06
**72h**	3.26E-07	4.92E-07	5.11E-06	4.99E-07
	cgd4_2580			
**2h**	1.18E-07	4.61E-08	4.18E-07	3.63E-07
**6h**	2.05E-07	7.02E-07	2.37E-06	2.09E-06
**12h**	5.96E-06	3.80E-08	1.19E-07	1.61E-07
**24h**	1.12E-07	2.28E-07	7.00E-07	8.57E-07
**36h**	2.73E-07	1.48E-07	1.03E-06	2.83E-07
**48h**	1.17E-07	3.63E-07	8.64E-07	6.35E-07
**72h**	4.23E-08	1.61E-08	0.00E+00	5.34E-08
	cgd4_2600			
**2h**	4.94E-06	6.29E-06	3.04E-05	3.34E-06
**6h**	1.26E-07	1.30E-06	1.95E-06	1.73E-07
**12h**	4.81E-04	4.22E-04	4.72E-04	9.56E-04
**24h**	7.59E-05	5.31E-05	1.61E-04	1.94E-04
**36h**	9.56E-05	5.63E-05	5.29E-04	2.72E-04
**48h**	6.88E-05	1.32E-04	1.89E-04	2.12E-04
**72h**	9.58E-06	4.99E-05	1.85E-05	2.12E-05
	cgd4_2610			
**2h**	6.64E-07	3.85E-08	8.73E-09	4.16E-07
**6h**	0.00E+00	0.00E+00	1.08E-07	3.53E-08
**12h**	1.48E-06	1.72E-06	3.03E-06	6.95E-06
**24h**	4.84E-07	3.79E-07	5.32E-07	4.32E-07
**36h**	3.94E-07	1.08E-06	4.08E-06	1.95E-06

**48h**	1.73E-07	4.41E-07	8.47E-07	1.11E-06
**72h**	0.00E+00	2.73E-07	3.71E-07	1.28E-07
	cgd4_2620			
**2h**	2.72E-05	2.19E-05	9.37E-05	1.22E-05
**6h**	2.14E-05	2.26E-05	4.75E-05	8.01E-05
**12h**	1.56E-05	3.58E-06	3.45E-05	7.51E-05
**24h**	1.41E-05	1.68E-05	1.79E-05	3.70E-05
**36h**	8.97E-06	1.18E-05	5.67E-05	2.91E-05
**48h**	5.32E-06	8.31E-06	1.43E-05	1.68E-05
**72h**	1.10E-04	7.74E-06	7.62E-06	3.33E-06
	cgd4_2630			
**2h**	1.53E-05	2.46E-06	1.78E-05	1.29E-05
**6h**	7.26E-07	1.35E-06	3.95E-06	4.56E-06
**12h**	2.26E-06	1.82E-07	1.10E-05	1.96E-05
**24h**	1.82E-06	1.87E-06	4.41E-06	4.55E-06
**36h**	1.89E-06	7.85E-04	6.50E-06	4.39E-06
**48h**	7.22E-07	6.16E-07	1.80E-06	2.47E-06
**72h**	2.40E-07	5.33E-07	6.80E-07	7.57E-07
	cgd4_2640			
**2h**	3.86E-07	4.50E-07	3.93E-06	5.67E-07
**6h**	2.46E-08	1.48E-07	1.06E-07	1.55E-07
**12h**	3.59E-07	2.86E-08	1.41E-06	2.35E-06
**24h**	1.91E-07	2.13E-07	2.90E-07	4.44E-07
**36h**	3.97E-07	2.06E-07	1.15E-06	5.92E-07
**48h**	2.06E-07	1.65E-07	3.33E-07	3.68E-07
**72h**	7.40E-08	1.26E-07	2.12E-07	2.14E-07
	cgd4_2650			
**2h**	7.09E-08	0.00E+00	2.36E-07	3.05E-07
**6h**	2.47E-08	6.91E-08	1.32E-07	6.86E-08
**12h**	1.62E-08	3.02E-09	1.24E-07	3.96E-07
**24h**	5.32E-08	4.69E-08	8.57E-08	1.88E-07
**36h**	7.49E-08	8.05E-08	3.87E-07	1.86E-07
**48h**	4.80E-08	3.04E-08	1.12E-07	1.31E-07
**72h**	1.39E-08	4.65E-08	4.80E-08	4.58E-08
	cgd4_2660			
**2h**	4.08E-07	3.58E-08	5.50E-07	3.58E-07
**6h**	5.40E-08	1.93E-07	5.08E-07	3.09E-07
**12h**	1.43E-09	0.00E+00	3.50E-08	2.78E-07
**24h**	3.07E-08	1.10E-08	1.05E-07	1.49E-07
**36h**	2.76E-08	4.95E-08	1.73E-07	8.23E-08
**48h**	3.08E-08	1.79E-08	1.20E-07	2.30E-07
**72h**	1.05E-08	2.60E-08	3.77E-08	4.22E-09
	cgd4_2670			
**2h**	4.88E-07	5.96E-07	1.23E-06	1.32E-07
**6h**	5.81E-07	6.05E-07	3.58E-06	2.59E-06
**12h**	2.72E-08	1.45E-08	3.15E-07	8.44E-07
**24h**	1.40E-07	4.80E-08	1.21E-06	2.11E-06
**36h**	1.94E-07	2.78E-07	1.19E-06	8.10E-07
**48h**	2.16E-07	2.25E-07	1.28E-06	1.51E-06
**72h**	2.09E-08	2.30E-07	4.22E-08	4.42E-08
	cgd4_2690			
**2h**	4.37E-06	0.00E+00	0.00E+00	0.00E+00
**6h**	5.39E-04	2.57E-03	3.04E-03	2.10E-03

**12h**	2.10E-05	2.18E-06	1.85E-04	8.70E-04
**24h**	1.30E-04	2.20E-04	1.03E-03	3.12E-03
**36h**	2.62E-04	3.21E-04	6.77E-04	4.72E-04
**48h**	1.97E-04	3.00E-04	3.76E-04	7.33E-04
**72h**	3.57E-05	1.50E-04	1.66E-05	3.21E-05
	cgd4_2700			
**2h**	4.58E-05	6.49E-04	8.65E-05	6.54E-06
**6h**	5.84E-05	3.08E-04	4.01E-04	2.68E-04
**12h**	6.59E-06	3.43E-06	5.01E-05	1.17E-04
**24h**	1.44E-05	2.45E-05	8.88E-05	2.34E-04
**36h**	4.58E-05	9.52E-05	1.28E-04	5.34E-05
**48h**	5.10E-05	1.31E-04	9.74E-05	1.40E-04
**72h**	7.05E-06	4.96E-05	1.96E-05	1.24E-05
	cgd4_2710			
**2h**	2.50E-05	1.03E-03	1.58E-04	4.51E-05
**6h**	8.82E-05	0.00E+00	6.48E-04	3.53E-04
**12h**	5.95E-06	0.00E+00	1.34E-04	1.48E-04
**24h**	2.09E-05	0.00E+00	1.91E-04	2.25E-04
**36h**	7.89E-05	0.00E+00	3.44E-04	1.02E-04
**48h**	9.71E-05	0.00E+00	1.38E-04	1.54E-04
**72h**	5.92E-05	0.00E+00	4.15E-04	7.33E-05
	cgd4_2720			
**2h**	2.30E-09	1.01E-08	1.65E-07	0.00E+00
**6h**	2.54E-09	3.97E-08	1.68E-08	1.29E-08
**12h**	4.31E-09	0.00E+00	1.13E-08	3.75E-08
**24h**	1.14E-08	1.66E-08	9.58E-08	3.08E-08
**36h**	6.81E-08	2.90E-08	3.09E-07	2.44E-07
**48h**	3.05E-06	2.92E-06	2.68E-06	4.65E-06
**72h**	1.16E-06	1.05E-06	9.17E-06	4.06E-06
	cgd4_2740			
**2h**	1.23E-06	4.27E-07	4.90E-06	3.67E-06
**6h**	2.20E-07	1.51E-07	9.71E-07	9.24E-07
**12h**	4.31E-07	3.24E-07	5.47E-07	3.38E-06
**24h**	7.55E-07	1.29E-06	1.57E-06	1.14E-06
**36h**	5.48E-07	1.92E-07	2.10E-06	1.63E-06
**48h**	6.74E-07	3.85E-07	4.20E-07	6.49E-07
**72h**	2.85E-07	3.07E-07	7.39E-07	2.69E-07
	cgd4_2750			
**2h**	6.79E-06	5.44E-06	4.47E-05	1.83E-05
**6h**	9.10E-06	9.52E-06	3.81E-06	4.36E-06
**12h**	2.81E-06	2.68E-06	1.56E-05	3.17E-05
**24h**	2.35E-05	1.56E-05	6.79E-06	5.14E-06
**36h**	1.73E-06	2.13E-06	1.58E-05	8.88E-06
**48h**	2.59E-06	1.82E-06	3.24E-06	2.07E-06
**72h**	9.92E-07	1.08E-06	2.29E-06	1.36E-06
	cgd4_2760			
**2h**	5.65E-08	1.90E-09	2.69E-07	1.98E-07
**6h**	0.00E+00	3.49E-08	3.32E-08	1.09E-07
**12h**	5.13E-10	6.68E-09	1.03E-08	1.15E-07
**24h**	3.22E-08	5.55E-08	7.91E-08	1.27E-07
**36h**	3.75E-08	5.91E-08	3.91E-07	4.14E-07
**48h**	7.16E-08	3.99E-08	2.38E-07	3.53E-07
**72h**	7.56E-08	2.65E-07	3.70E-07	1.26E-07

	cgd4_2770			
**2h**	1.81E-06	8.94E-07	2.59E-05	1.43E-05
**6h**	2.04E-06	2.64E-06	2.92E-06	3.21E-06
**12h**	8.61E-07	5.48E-07	9.40E-06	2.99E-05
**24h**	2.46E-06	2.90E-06	5.81E-06	5.06E-06
**36h**	1.43E-06	1.45E-06	4.63E-06	4.55E-06
**48h**	6.76E-07	4.37E-07	1.63E-06	1.85E-06
**72h**	3.98E-07	6.07E-07	7.27E-07	3.76E-07
	cgd4_2780			
**2h**	2.13E-05	1.38E-06	1.29E-05	1.02E-06
**6h**	5.57E-04	6.55E-04	1.27E-03	4.02E-03
**12h**	8.73E-05	2.16E-05	1.41E-04	4.44E-04
**24h**	1.73E-04	1.65E-04	7.31E-04	1.99E-03
**36h**	5.70E-04	5.62E-04	1.15E-03	1.95E-03
**48h**	1.73E-04	3.93E-04	7.51E-04	1.46E-03
**72h**	7.35E-05	1.13E-04	1.02E-04	6.52E-05
	cgd4_2790			
**2h**	2.91E-09	2.54E-09	8.03E-09	1.26E-08
**6h**	3.78E-07	3.83E-07	1.43E-06	1.97E-06
**12h**	6.80E-08	4.58E-08	1.06E-07	9.72E-07
**24h**	8.64E-08	6.67E-08	1.75E-06	2.36E-06
**36h**	4.32E-07	5.94E-07	1.66E-06	1.07E-06
**48h**	1.58E-07	3.61E-07	1.07E-06	1.10E-06
**72h**	9.74E-08	1.46E-07	7.19E-08	2.65E-08
	cgd4_2810			
**2h**	7.50E-05	8.38E-05	4.07E-04	8.66E-05
**6h**	3.34E-04	7.60E-04	1.89E-03	3.30E-03
**12h**	1.01E-05	1.57E-07	6.71E-05	2.99E-04
**24h**	9.36E-05	2.23E-04	6.78E-04	2.07E-03
**36h**	1.73E-04	1.09E-04	5.82E-04	8.24E-04
**48h**	1.66E-04	1.82E-04	2.94E-04	5.48E-04
**72h**	2.77E-05	3.28E-05	4.89E-05	7.89E-06
	cgd4_2820			
**2h**	0.00E+00	0.00E+00	0.00E+00	0.00E+00
**6h**	1.84E-08	2.24E-08	1.11E-08	5.95E-08
**12h**	2.95E-07	5.31E-07	5.40E-07	2.89E-06
**24h**	7.66E-08	3.68E-08	3.80E-07	2.08E-07
**36h**	5.16E-08	5.00E-08	6.39E-07	4.62E-07
**48h**	7.26E-08	6.63E-08	2.14E-07	3.97E-07
**72h**	1.48E-08	1.66E-08	1.35E-08	1.71E-08
	cgd4_2830			
**2h**	2.04E-07	2.70E-07	2.12E-06	1.08E-06
**6h**	2.92E-07	4.13E-07	1.35E-06	1.84E-06
**12h**	1.71E-07	1.69E-07	2.97E-07	8.55E-07
**24h**	1.20E-07	8.99E-08	1.42E-06	3.18E-06
**36h**	4.20E-07	2.10E-07	2.98E-07	2.62E-07
**48h**	3.34E-07	2.85E-07	3.61E-07	2.65E-07
**72h**	7.22E-08	4.31E-08	2.28E-08	2.51E-08
	cgd4_2840			
**2h**	3.20E-07	9.43E-08	1.37E-06	2.25E-07
**6h**	2.25E-07	1.01E-07	5.94E-07	1.11E-06
**12h**	1.38E-07	1.94E-07	3.25E-07	5.72E-07
**24h**	1.82E-07	2.74E-07	6.39E-07	1.08E-06

**36h**	4.34E-07	4.42E-07	5.88E-07	3.59E-07
**48h**	4.66E-07	5.14E-07	6.20E-07	8.68E-07
**72h**	1.73E-07	1.15E-07	1.48E-07	2.97E-08
	cgd4_2850			
**2h**	5.22E-08	8.60E-09	3.17E-07	4.20E-08
**6h**	2.55E-09	7.93E-09	4.73E-08	1.22E-08
**12h**	1.90E-08	2.95E-08	4.91E-08	5.83E-08
**24h**	2.29E-08	1.94E-08	1.10E-07	5.85E-08
**36h**	7.38E-08	6.09E-08	1.93E-07	6.76E-08
**48h**	1.08E-07	6.25E-08	9.99E-08	8.73E-08
**72h**	4.02E-08	8.82E-08	1.40E-07	7.35E-08
	cgd4_2860			
**2h**	5.63E-06	2.62E-06	9.55E-06	3.46E-05
**6h**	4.07E-06	1.97E-05	1.72E-05	4.65E-05
**12h**	1.37E-04	4.04E-04	3.49E-04	9.19E-04
**24h**	7.25E-05	1.59E-04	3.80E-04	6.67E-04
**36h**	1.07E-04	2.03E-04	7.31E-04	7.79E-04
**48h**	8.17E-05	1.77E-04	3.52E-04	1.13E-03
**72h**	2.13E-05	1.92E-05	5.94E-05	2.51E-05
	cgd4_2870			
**2h**	4.95E-07	4.05E-07	2.18E-06	6.02E-07
**6h**	9.28E-07	1.03E-06	1.31E-06	7.68E-07
**12h**	2.71E-07	1.27E-07	8.13E-07	2.62E-06
**24h**	3.96E-07	3.23E-07	5.17E-07	1.77E-06
**36h**	1.83E-07	1.72E-07	1.53E-06	8.19E-07
**48h**	1.03E-07	9.15E-08	3.64E-07	9.84E-07
**72h**	1.19E-07	1.81E-07	1.07E-06	1.26E-07
	cgd4_2880			
**2h**	3.90E-08	6.55E-08	7.43E-07	1.13E-07
**6h**	5.83E-07	2.33E-07	5.67E-07	2.77E-06
**12h**	9.98E-08	1.22E-07	5.59E-07	5.88E-07
**24h**	1.25E-07	3.23E-08	2.80E-07	8.95E-07
**36h**	6.86E-08	1.25E-07	1.17E-06	4.93E-07
**48h**	3.11E-08	1.81E-08	2.08E-07	2.92E-07
**72h**	2.42E-08	2.70E-08	1.98E-07	3.72E-08
	cgd4_2890			
**2h**	7.47E-05	2.66E-04	1.30E-03	7.14E-05
**6h**	1.37E-04	3.42E-04	1.22E-03	1.36E-03
**12h**	1.70E-04	1.76E-04	2.67E-04	8.73E-04
**24h**	3.47E-05	8.00E-05	3.65E-04	1.26E-03
**36h**	2.00E-04	1.87E-04	6.62E-04	1.01E-03
**48h**	4.43E-05	1.16E-04	6.38E-04	9.18E-04
**72h**	4.37E-05	1.09E-04	2.42E-04	1.47E-04
	cgd4_2920			
**2h**	0.00E+00	0.00E+00	1.23E-08	9.45E-09
**6h**	1.75E-08	2.16E-09	9.59E-10	9.69E-08
**12h**	7.49E-07	9.68E-06	3.79E-06	3.97E-06
**24h**	8.76E-08	1.09E-07	3.28E-07	5.84E-07
**36h**	5.88E-07	7.73E-07	7.93E-06	3.85E-06
**48h**	7.26E-08	1.45E-07	5.77E-07	8.05E-07
**72h**	1.16E-07	1.03E-07	7.41E-07	3.86E-07
	cgd4_2930			
**2h**	0.00E+00	0.00E+00	0.00E+00	0.00E+00

**6h**	0.00E+00	0.00E+00	0.00E+00	4.93E-09
**12h**	6.85E-08	1.10E-07	2.19E-07	2.14E-07
**24h**	1.06E-08	1.03E-08	3.48E-08	1.24E-07
**36h**	1.29E-07	1.16E-07	9.77E-07	8.31E-07
**48h**	1.25E-08	3.87E-08	1.06E-07	1.63E-07
**72h**	1.35E-08	4.10E-09	4.30E-08	7.95E-08
	cgd4_2940			
**2h**	5.54E-08	4.88E-08	1.54E-07	7.59E-08
**6h**	6.61E-08	4.18E-08	1.48E-07	7.71E-08
**12h**	8.99E-08	3.44E-08	7.46E-08	1.18E-07
**24h**	1.10E-08	1.93E-08	1.44E-07	1.09E-07
**36h**	7.93E-08	6.50E-08	2.74E-07	3.49E-07
**48h**	4.15E-08	4.92E-08	2.59E-07	2.22E-07
**72h**	2.01E-08	2.00E-08	3.44E-08	3.52E-08
	cgd4_2960			
**2h**	1.69E-07	8.02E-08	8.31E-07	4.27E-08
**6h**	9.64E-08	2.15E-07	8.87E-07	6.91E-07
**12h**	2.06E-07	2.35E-07	5.67E-07	2.41E-06
**24h**	7.33E-08	8.89E-08	3.79E-07	4.81E-07
**36h**	2.44E-07	1.49E-07	6.73E-07	8.24E-07
**48h**	9.78E-08	9.51E-08	4.93E-07	4.29E-07
**72h**	3.42E-08	2.56E-08	7.23E-08	9.43E-08
	cgd4_2980			
**2h**	1.79E-06	3.30E-06	5.90E-05	1.08E-05
**6h**	1.65E-06	1.58E-06	1.05E-05	9.97E-06
**12h**	1.97E-04	2.88E-04	5.22E-04	1.16E-03
**24h**	1.07E-05	3.91E-05	4.86E-05	6.85E-05
**36h**	2.01E-05	1.98E-05	1.29E-04	2.02E-04
**48h**	1.13E-05	7.09E-06	5.17E-05	9.96E-05
**72h**	2.48E-06	2.77E-06	1.04E-05	1.35E-05
	cgd4_3010			
**2h**	5.31E-06	4.64E-06	3.17E-05	2.05E-05
**6h**	2.19E-06	3.79E-06	1.37E-05	1.08E-05
**12h**	1.22E-05	1.40E-05	1.87E-05	8.73E-05
**24h**	3.95E-06	8.08E-06	2.24E-05	2.36E-05
**36h**	2.88E-06	2.33E-06	5.92E-06	1.08E-05
**48h**	1.99E-06	1.82E-06	8.40E-06	1.62E-05
**72h**	5.25E-07	3.39E-07	6.41E-07	8.38E-07
	cgd4_3030			
**2h**	7.62E-07	5.24E-07	3.41E-06	4.35E-06
**6h**	5.49E-07	1.66E-06	4.85E-06	2.20E-06
**12h**	4.06E-07	2.97E-07	9.66E-07	5.26E-06
**24h**	4.27E-07	4.26E-07	1.59E-06	5.29E-06
**36h**	5.62E-07	3.09E-07	7.91E-07	1.59E-06
**48h**	2.77E-07	2.47E-07	5.72E-07	1.95E-06
**72h**	1.41E-07	3.54E-08	2.23E-07	1.58E-07
	cgd4_3040			
**2h**	2.27E-04	1.12E-03	7.48E-04	6.18E-04
**6h**	1.38E-04	2.02E-03	2.25E-03	5.85E-04
**12h**	4.56E-05	4.04E-04	1.09E-03	3.11E-04
**24h**	8.07E-05	1.79E-04	8.02E-04	6.46E-04
**36h**	1.41E-04	3.50E-04	1.15E-03	6.60E-04
**48h**	5.03E-05	8.78E-05	5.33E-04	5.61E-04

**72h**	3.48E-05	8.43E-05	1.39E-03	1.12E-04
	cgd4_3050			
**2h**	1.99E-06	2.80E-06	3.44E-06	8.98E-06
**6h**	3.49E-06	1.71E-06	2.95E-06	5.18E-06
**12h**	1.95E-06	1.71E-06	4.19E-06	1.98E-05
**24h**	3.03E-06	4.11E-06	1.98E-05	6.38E-06
**36h**	1.00E-06	3.87E-07	1.90E-06	3.83E-06
**48h**	7.32E-07	2.74E-06	4.38E-06	1.02E-06
**72h**	2.55E-07	2.75E-07	9.02E-07	4.22E-07
	cgd4_3070			
**2h**	0.00E+00	0.00E+00	1.17E-07	0.00E+00
**6h**	6.42E-09	1.46E-08	1.42E-07	9.70E-07
**12h**	1.24E-05	1.91E-05	2.40E-05	1.00E-04
**24h**	2.46E-06	2.04E-06	1.39E-05	1.92E-05
**36h**	9.96E-06	3.36E-06	7.85E-05	8.03E-05
**48h**	6.11E-07	4.94E-06	2.22E-05	1.48E-05
**72h**	4.02E-06	1.93E-06	2.04E-05	3.61E-06
	cgd4_3090			
**2h**	1.50E-07	1.36E-08	1.13E-07	1.85E-07
**6h**	2.41E-08	5.98E-08	6.73E-08	1.37E-08
**12h**	9.83E-09	3.16E-09	8.89E-08	1.80E-07
**24h**	3.31E-08	3.57E-08	3.05E-07	1.45E-07
**36h**	2.41E-07	5.14E-08	3.72E-07	9.90E-07
**48h**	2.14E-06	6.66E-06	1.22E-05	7.38E-06
**72h**	1.27E-06	4.06E-07	2.13E-06	1.52E-06
	cgd4_3100			
**2h**	0.00E+00	0.00E+00	3.18E-09	0.00E+00
**6h**	1.56E-08	2.00E-08	5.88E-08	1.20E-07
**12h**	1.93E-07	6.35E-08	3.26E-07	1.48E-06
**24h**	1.31E-08	4.70E-08	4.26E-07	2.77E-07
**36h**	5.52E-07	3.87E-08	1.30E-06	1.43E-06
**48h**	2.77E-08	5.37E-08	2.89E-07	1.93E-07
**72h**	4.91E-08	1.74E-08	3.15E-07	8.05E-08
	cgd4_3110			
**2h**	7.40E-09	0.00E+00	8.68E-09	4.15E-08
**6h**	0.00E+00	2.57E-09	5.33E-09	1.89E-09
**12h**	2.29E-07	3.99E-08	4.75E-07	4.14E-06
**24h**	2.43E-08	2.65E-08	3.03E-07	1.84E-07
**36h**	2.32E-07	9.46E-08	1.53E-06	1.75E-06
**48h**	8.52E-08	7.94E-08	3.81E-07	6.02E-07
**72h**	3.39E-08	3.80E-08	3.38E-07	7.54E-08
	cgd4_3120			
**2h**	0.00E+00	3.44E-07	8.50E-07	0.00E+00
**6h**	3.51E-07	4.84E-07	1.76E-06	6.52E-06
**12h**	5.82E-07	1.96E-07	2.68E-06	1.70E-05
**24h**	3.03E-07	2.54E-07	3.87E-06	9.67E-06
**36h**	1.71E-06	7.25E-07	7.79E-06	1.02E-05
**48h**	8.08E-07	3.84E-06	7.66E-06	5.57E-06
**72h**	5.66E-07	8.63E-07	3.04E-06	1.26E-06
	cgd4_3150			
**2h**	5.50E-09	7.80E-09	1.75E-08	1.63E-07
**6h**	1.31E-07	6.35E-07	2.61E-06	2.37E-06
**12h**	3.21E-08	2.74E-08	7.02E-08	2.72E-07

**24h**	2.09E-08	2.27E-08	6.61E-07	1.53E-06
**36h**	4.10E-07	6.60E-08	1.11E-06	7.04E-07
**48h**	1.19E-07	1.42E-07	5.70E-07	1.17E-06
**72h**	1.02E-07	2.02E-07	9.95E-07	5.99E-08
	cgd4_3160			
**2h**	2.13E-07	1.87E-07	3.97E-07	1.02E-06
**6h**	9.56E-07	1.20E-06	2.29E-06	5.80E-06
**12h**	1.03E-07	6.62E-08	3.36E-07	1.05E-06
**24h**	1.49E-07	6.59E-08	7.92E-07	3.05E-06
**36h**	3.92E-07	2.30E-07	8.33E-07	9.66E-07
**48h**	3.02E-07	1.78E-07	5.64E-07	1.31E-06
**72h**	1.32E-07	3.74E-07	5.91E-07	1.50E-07
	cgd4_3180			
**2h**	1.27E-06	4.40E-07	1.07E-06	4.30E-06
**6h**	9.25E-08	6.62E-08	4.26E-07	3.68E-07
**12h**	5.07E-07	2.32E-07	7.29E-07	3.17E-06
**24h**	3.43E-07	2.60E-07	3.79E-07	1.83E-06
**36h**	4.28E-07	1.74E-07	1.01E-06	4.91E-07
**48h**	2.22E-07	1.69E-07	6.32E-07	8.88E-07
**72h**	6.76E-08	1.57E-07	7.74E-07	9.81E-08
	cgd4_3200			
**2h**	7.07E-06	3.41E-05	1.15E-05	5.62E-06
**6h**	2.90E-06	4.16E-06	4.94E-06	9.36E-06
**12h**	9.09E-07	3.75E-06	1.02E-05	9.92E+02
**24h**	2.41E-06	1.33E-06	1.20E-05	1.53E-05
**36h**	1.60E-06	1.94E-06	6.96E-06	2.53E-06
**48h**	2.46E-06	1.69E-06	8.20E-06	5.81E-06
**72h**	4.34E-07	2.06E-06	3.11E-06	3.15E-06
	cgd4_3210			
**2h**	2.62E-07	2.17E-09	7.97E-09	0.00E+00
**6h**	1.12E-08	2.22E-08	9.99E-08	5.95E-08
**12h**	7.98E-09	0.00E+00	3.23E-08	8.43E-08
**24h**	2.79E-08	1.04E-08	9.21E-08	1.18E-07
**36h**	4.43E-08	3.56E-07	2.46E-07	7.05E-08
**48h**	1.01E-07	8.25E-08	3.02E-07	2.24E-07
**72h**	8.08E-08	1.70E-07	5.74E-07	1.20E-07
	cgd4_3220			
**2h**	6.46E-07	1.96E-07	6.18E-07	1.27E-06
**6h**	2.51E-06	3.99E-06	4.33E-06	3.62E-05
**12h**	1.82E-04	1.14E-02	5.73E-04	6.71E-04
**24h**	4.20E-05	6.61E-05	1.30E-04	1.61E-04
**36h**	1.04E-04	9.58E-04	2.54E-03	2.62E-04
**48h**	8.89E-05	2.60E-02	1.13E-03	3.74E-04
**72h**	8.76E-05	0.00E+00	8.33E-04	2.32E-04
	cgd4_3230			
**2h**	1.12E-06	9.18E-07	0.00E+00	1.87E-06
**6h**	3.98E-07	1.39E-05	1.58E-05	5.59E-06
**12h**	9.87E-06	9.05E-06	9.50E-06	2.97E-05
**24h**	6.26E-06	4.04E-06	5.30E-06	1.25E-05
**36h**	1.91E-06	2.93E-06	8.82E-06	4.90E-06
**48h**	3.54E-06	3.11E-06	5.23E-06	7.81E-06
**72h**	1.00E-06	2.48E-06	3.68E-06	1.77E-06
	cgd4_3240			

**2h**	0.00E+00	0.00E+00	1.09E-06	0.00E+00
**6h**	0.00E+00	2.25E-07	9.18E-08	0.00E+00
**12h**	1.63E-06	2.25E-07	3.32E-06	6.19E-06
**24h**	2.49E-06	1.34E-06	1.51E-06	2.89E-06
**36h**	3.26E-06	1.55E-05	2.04E-05	1.37E-05
**48h**	9.96E-05	2.49E-04	1.08E-04	2.62E-04
**72h**	1.10E-05	1.35E-05	2.11E-05	3.03E-05
	cgd4_3250			
**2h**	1.07E-06	3.39E-07	2.76E-06	2.14E-06
**6h**	1.11E-06	2.94E-07	5.49E-06	2.04E-06
**12h**	2.75E-07	1.22E-07	2.83E-06	2.23E-06
**24h**	1.72E-06	4.70E-07	7.19E-07	4.25E-06
**36h**	1.37E-07	3.72E-07	1.36E-06	1.27E-06
**48h**	3.58E-07	7.32E-08	3.57E-07	4.99E-07
**72h**	1.29E-07	2.65E-07	2.83E-07	2.26E-07
	cgd4_3280			
**2h**	4.42E-07	1.80E-07	8.73E-07	1.28E-06
**6h**	1.07E-08	1.30E-08	9.73E-08	3.27E-08
**12h**	4.78E-06	1.12E-05	2.13E-05	2.25E-05
**24h**	1.71E-06	6.06E-07	7.28E-07	1.79E-06
**36h**	9.96E-07	1.90E-06	7.82E-06	6.35E-06
**48h**	7.06E-07	8.73E-07	1.89E-06	1.75E-06
**72h**	6.51E-07	7.77E-07	4.08E-06	1.18E-06
	cgd4_3290			
**2h**	2.94E-07	0.00E+00	1.58E-08	0.00E+00
**6h**	0.00E+00	0.00E+00	5.78E-07	1.49E-06
**12h**	7.91E-04	1.53E-03	5.89E-03	1.00E-02
**24h**	5.67E-05	2.22E-04	5.89E-05	7.94E-04
**36h**	9.09E-05	3.51E-04	1.72E-03	2.98E-03
**48h**	2.29E-05	9.87E-05	1.67E-04	2.50E-04
**72h**	1.51E-05	1.61E-05	3.26E-05	4.11E-05
	cgd4_3300			
**2h**	0.00E+00	0.00E+00	2.13E-09	0.00E+00
**6h**	1.02E-09	5.29E-09	7.08E-08	9.38E-08
**12h**	3.28E-06	7.06E-06	1.97E-05	1.88E-05
**24h**	3.56E-07	3.47E-07	2.26E-07	2.16E-06
**36h**	7.29E-07	1.49E-06	1.20E-05	1.26E-05
**48h**	1.35E-07	4.06E-07	1.55E-06	1.84E-06
**72h**	2.62E-07	6.89E-08	4.67E-07	4.40E-07
	cgd4_3310			
**2h**	0.00E+00	0.00E+00	8.68E-09	0.00E+00
**6h**	1.07E-07	6.30E-08	8.49E-07	1.43E-06
**12h**	2.65E-08	2.09E-09	7.46E-08	2.08E-07
**24h**	4.08E-08	8.31E-08	1.29E-07	7.92E-07
**36h**	3.49E-07	1.66E-07	1.05E-06	1.50E-06
**48h**	1.54E-07	1.87E-07	6.37E-07	9.66E-07
**72h**	3.69E-07	1.17E-07	6.37E-07	5.28E-07
	cgd4_3320			
**2h**	1.00E-03	5.27E-04	8.91E-03	2.50E-03
**6h**	5.06E-04	1.26E-04	3.41E-03	3.89E-03
**12h**	1.07E-03	2.66E-04	7.34E-03	4.36E-03
**24h**	6.82E-04	4.08E-04	5.40E-04	2.70E-03
**36h**	2.68E-04	2.51E-04	6.11E-04	1.02E-03

**48h**	3.44E-04	4.56E-05	6.70E-04	5.01E-04
**72h**	8.50E-04	1.41E-04	1.14E-03	2.56E-04
	cgd4_3330			
**2h**	1.04E-06	7.47E-07	3.40E-06	1.68E-06
**6h**	4.28E-09	1.41E-10	1.35E-07	4.36E-09
**12h**	8.74E-06	3.91E-06	3.54E-05	4.93E-05
**24h**	4.94E-07	1.01E-06	4.16E-07	1.10E-06
**36h**	7.60E-07	4.66E-07	5.68E-06	7.80E-06
**48h**	8.70E-07	1.29E-06	3.50E-06	3.64E-06
**72h**	6.00E-07	6.79E-08	2.28E-07	4.05E-07
	cgd4_3350			
**2h**	0.00E+00	0.00E+00	0.00E+00	0.00E+00
**6h**	0.00E+00	0.00E+00	0.00E+00	0.00E+00
**12h**	0.00E+00	0.00E+00	8.68E-09	0.00E+00
**24h**	6.39E-11	2.29E-11	7.36E-10	2.11E-09
**36h**	1.51E-09	5.45E-09	1.18E-08	9.49E-10
**48h**	3.24E-08	2.08E-08	6.48E-08	6.74E-08
**72h**	1.73E-08	6.57E-09	3.94E-08	1.25E-08
	cgd4_3360			
**2h**	8.95E-07	1.88E-06	9.87E-06	1.06E-05
**6h**	1.74E-07	7.38E-07	4.14E-06	2.30E-06
**12h**	3.26E-07	1.30E-07	6.79E-06	1.26E-05
**24h**	1.00E-06	1.17E-06	7.98E-07	6.72E-06
**36h**	7.75E-07	3.20E-07	2.31E-06	2.12E-06
**48h**	5.10E-07	2.69E-07	2.19E-06	3.51E-06
**72h**	2.74E-07	7.05E-08	5.18E-07	2.06E-07
	cgd4_3370			
**2h**	4.63E-06	1.00E-04	9.94E-06	9.34E-06
**6h**	6.12E-07	1.11E-04	7.29E-06	8.69E-06
**12h**	8.89E-08	4.69E-06	2.34E-07	6.43E-07
**24h**	1.28E-06	3.89E-05	2.62E-06	7.77E-06
**36h**	2.03E-06	4.03E-05	1.82E-05	2.76E-05
**48h**	3.09E-06	5.10E-05	6.95E-06	2.79E-05
**72h**	8.57E-06	1.06E-04	1.55E-04	1.71E-05
	cgd4_3380			
**2h**	0.00E+00	0.00E+00	0.00E+00	1.81E-08
**6h**	0.00E+00	3.34E-09	0.00E+00	0.00E+00
**12h**	2.49E-08	5.16E-08	1.11E-07	1.13E-06
**24h**	3.08E-08	6.55E-08	1.19E-07	2.74E-07
**36h**	2.80E-08	3.08E-08	2.79E-07	4.56E-07
**48h**	6.85E-08	6.23E-08	4.63E-08	3.83E-07
**72h**	3.04E-08	4.33E-08	1.75E-07	2.53E-08
	cgd4_3390			
**2h**	2.23E-05	7.35E-06	7.06E-05	3.90E-05
**6h**	5.70E-06	1.01E-05	3.95E-05	5.54E-05
**12h**	7.54E-06	1.74E-06	8.25E-06	8.26E-05
**24h**	1.21E-05	6.74E-06	1.31E-05	4.72E-05
**36h**	3.24E-06	1.21E-06	7.00E-06	5.35E-06
**48h**	3.72E-06	1.58E-06	3.08E-06	2.07E-05
**72h**	2.58E-06	1.24E-06	6.00E-06	1.14E-06
	cgd4_3400			
**2h**	1.84E-06	8.03E-07	8.23E-06	6.31E-06
**6h**	1.17E-06	3.06E-06	1.03E-05	2.03E-05

**12h**	3.55E-07	2.66E-07	3.29E-06	6.10E-06
**24h**	2.41E-06	1.04E-06	3.66E-06	1.55E-05
**36h**	7.69E-07	4.07E-07	3.16E-06	1.15E-06
**48h**	7.24E-07	2.41E-07	5.01E-07	3.22E-06
**72h**	8.72E-08	2.25E-07	9.85E-07	2.59E-07
	cgd4_3410			
**2h**	1.37E-06	6.93E-07	1.02E-05	1.42E-06
**6h**	1.68E-06	1.54E-06	1.17E-05	1.78E-05
**12h**	2.28E-06	1.71E-06	1.07E-05	1.09E-05
**24h**	4.18E-06	1.21E-06	4.27E-06	2.85E-05
**36h**	1.93E-06	4.05E-06	4.15E-05	1.80E-05
**48h**	1.56E-05	7.60E-07	5.62E-06	1.31E-05
**72h**	7.89E-07	1.10E-06	8.01E-06	1.83E-06
	cgd4_3420			
**2h**	3.64E-09	1.93E-08	6.86E-08	5.15E-08
**6h**	8.72E-08	1.86E-08	1.35E-07	2.58E-07
**12h**	5.59E-08	4.10E-08	1.50E-07	4.85E-07
**24h**	1.16E-07	3.30E-08	1.11E-07	5.84E-07
**36h**	2.11E-07	1.85E-07	8.20E-07	8.41E-07
**48h**	1.69E-07	8.35E-08	3.91E-07	7.95E-07
**72h**	3.24E-07	3.82E-07	1.68E-06	7.04E-07
	cgd4_3430			
**2h**	0.00E+00	2.71E-07	4.69E-06	1.05E-07
**6h**	0.00E+00	1.15E-08	0.00E+00	9.03E-08
**12h**	1.70E-05	1.07E-05	3.28E-05	1.02E-04
**24h**	3.55E-06	2.77E-06	2.96E-06	9.73E-06
**36h**	4.80E-06	8.72E-06	7.06E-05	9.74E-05
**48h**	6.84E-06	3.93E-06	2.10E-05	3.35E-05
**72h**	4.15E-05	4.45E-05	1.38E-04	7.96E-05
	cgd4_3440			
**2h**	0.00E+00	0.00E+00	0.00E+00	0.00E+00
**6h**	0.00E+00	0.00E+00	0.00E+00	0.00E+00
**12h**	2.40E-06	2.05E-06	3.02E-06	2.03E-05
**24h**	2.80E-07	2.62E-07	1.96E-07	1.06E-06
**36h**	2.19E-07	3.90E-07	4.43E-06	4.50E-06
**48h**	5.31E-07	4.76E-07	1.03E-06	3.45E-06
**72h**	1.13E-06	3.96E-07	1.80E-06	2.82E-06
	cgd4_3450			
**2h**	0.00E+00	0.00E+00	0.00E+00	0.00E+00
**6h**	0.00E+00	0.00E+00	0.00E+00	0.00E+00
**12h**	1.57E-06	3.45E-06	6.85E-06	1.73E-05
**24h**	3.57E-07	2.56E-07	4.63E-07	1.12E-06
**36h**	3.25E-07	5.69E-07	7.63E-06	8.76E-06
**48h**	1.09E-07	1.48E-07	8.05E-07	1.51E-06
**72h**	2.75E-07	1.25E-07	3.28E-07	4.13E-07
	cgd4_3460			
**2h**	1.40E-06	3.43E-08	0.00E+00	4.72E-08
**6h**	1.05E-07	1.29E-07	4.73E-07	3.12E-08
**12h**	1.47E-06	5.96E-06	9.88E-06	1.88E-05
**24h**	6.44E-07	5.80E-07	8.17E-07	3.36E-06
**36h**	2.17E-06	2.56E-06	1.64E-05	2.09E-05
**48h**	7.33E-07	1.36E-06	4.25E-06	5.22E-06
**72h**	1.33E-06	4.47E-07	2.57E-06	3.57E-06

	cgd4_3470			
**2h**	0.00E+00	0.00E+00	2.15E-08	0.00E+00
**6h**	0.00E+00	0.00E+00	8.67E-09	6.18E-09
**12h**	1.42E-05	2.17E-05	3.32E-05	1.85E-04
**24h**	1.10E-06	2.07E-06	5.92E-06	8.31E-06
**36h**	1.32E-06	1.75E-06	2.29E-05	3.45E-05
**48h**	5.37E-07	8.96E-07	3.88E-06	7.74E-06
**72h**	2.24E-07	1.36E-07	7.07E-07	8.15E-07
	cgd4_3520			
**2h**	4.66E-09	7.46E-09	5.81E-09	2.75E-08
**6h**	0.00E+00	5.05E-09	0.00E+00	0.00E+00
**12h**	1.33E-07	4.65E-07	5.66E-07	3.95E-06
**24h**	2.55E-08	1.30E-07	1.08E-07	9.05E-07
**36h**	2.60E-07	1.76E-07	2.09E-06	1.89E-06
**48h**	8.16E-08	1.17E-07	5.54E-07	5.90E-07
**72h**	5.30E-08	1.87E-08	2.49E-08	4.55E-08
	cgd4_3540			
**2h**	3.64E-07	1.46E-08	3.97E-08	1.82E-08
**6h**	5.04E-09	1.85E-07	6.96E-08	2.91E-08
**12h**	1.23E-06	6.64E-05	2.68E-06	1.48E-05
**24h**	1.92E-07	7.53E-07	9.26E-07	2.15E-06
**36h**	2.43E-06	8.25E-06	2.05E-05	3.44E-05
**48h**	3.16E-07	8.93E-06	1.42E-06	4.13E-06
**72h**	3.20E-07	5.51E-07	4.59E-07	6.32E-06
	cgd4_3560			
**2h**	2.10E-08	3.81E-09	1.03E-08	1.20E-08
**6h**	1.27E-08	2.61E-09	0.00E+00	5.80E-09
**12h**	8.80E-08	3.50E-08	3.08E-07	1.00E-06
**24h**	1.36E-07	1.70E-07	3.73E-07	5.17E-07
**36h**	1.35E-07	1.41E-07	1.80E-06	7.98E-07
**48h**	1.03E-07	4.16E-07	3.91E-07	2.88E-07
**72h**	2.26E-08	3.23E-08	1.08E-07	4.94E-08
	cgd4_3580			
**2h**	8.75E-07	1.96E-07	3.89E-06	1.43E-06
**6h**	1.20E-08	4.37E-08	3.34E-07	3.28E-08
**12h**	1.28E-06	5.30E-07	2.93E-06	4.41E-06
**24h**	9.27E-07	1.49E-06	9.11E-07	1.79E-06
**36h**	7.50E-07	3.09E-07	5.15E-06	5.11E-06
**48h**	5.82E-07	6.63E-07	1.05E-06	1.94E-06
**72h**	2.16E-07	2.85E-07	9.69E-07	3.74E-07
	cgd4_3590			
**2h**	1.73E-06	2.17E-07	5.59E-06	6.60E-06
**6h**	2.16E-07	2.63E-07	7.64E-07	1.17E-07
**12h**	2.51E-06	8.13E-07	4.58E-06	1.20E-05
**24h**	1.54E-06	1.97E-06	1.43E-06	2.81E-06
**36h**	4.71E-07	4.13E-07	4.15E-06	2.42E-06
**48h**	7.31E-07	1.05E-06	2.32E-06	2.38E-06
**72h**	1.27E-06	1.73E-06	4.86E-06	1.94E-06
	cgd4_3600			
**2h**	1.43E-06	6.45E-07	1.06E-05	5.45E-06
**6h**	1.68E-06	1.03E-06	3.42E-06	3.84E-06
**12h**	1.63E-06	5.26E-07	6.43E-06	9.89E-06
**24h**	9.87E-07	1.06E-06	1.45E-06	2.93E-06

**36h**	5.23E-07	3.88E-07	2.73E-06	2.96E-06
**48h**	1.40E-07	1.84E-07	4.80E-07	4.59E-07
**72h**	2.30E-07	1.18E-07	4.43E-07	1.54E-07
	cgd4_3610			
**2h**	5.17E-04	1.10E-04	7.32E-04	6.79E-04
**6h**	5.27E-05	2.36E-05	9.75E-05	5.03E-04
**12h**	6.15E-04	5.62E-04	2.74E-04	1.76E-03
**24h**	1.40E-04	1.55E-04	1.19E-04	5.85E-04
**36h**	9.64E-05	8.01E-05	1.85E-04	4.55E-04
**48h**	5.87E-05	1.31E-04	8.64E-05	2.35E-04
**72h**	4.13E-05	8.62E-05	9.09E-05	4.91E-05
	cgd4_3650			
**2h**	1.08E-06	3.61E-07	8.26E-06	7.27E-06
**6h**	1.66E-07	1.88E-07	1.25E-07	1.60E-07
**12h**	2.64E-05	4.92E-06	1.29E-05	1.56E-05
**24h**	1.40E-06	1.16E-06	9.52E-07	1.14E-06
**36h**	1.10E-06	8.05E-07	3.35E-06	2.83E-06
**48h**	3.05E-07	3.09E-07	9.53E-07	8.07E-07
**72h**	4.53E-07	5.25E-07	3.28E-07	1.44E-07
	cgd4_3680			
**2h**	0.00E+00	1.56E-08	1.48E-08	0.00E+00
**6h**	3.53E-09	8.32E-10	2.54E-08	9.33E-09
**12h**	2.19E-06	5.25E-07	9.83E-07	2.67E-06
**24h**	1.03E-07	2.08E-07	1.69E-07	3.39E-07
**36h**	9.83E-08	6.11E-08	7.68E-07	8.91E-07
**48h**	3.54E-08	5.01E-08	1.96E-07	2.62E-07
**72h**	7.11E-08	1.17E-07	5.66E-08	5.31E-08
	cgd4_3690			
**2h**	5.91E-06	5.12E-06	5.47E-05	3.36E-05
**6h**	3.37E-06	2.59E-06	2.05E-05	2.83E-05
**12h**	1.01E-06	1.25E-06	2.47E-05	1.00E-05
**24h**	5.78E-07	8.75E-07	6.68E-06	1.43E-05
**36h**	2.16E-06	1.81E-06	6.46E-06	4.73E-06
**48h**	1.10E-06	8.74E-07	6.78E-06	9.35E-06
**72h**	1.34E-06	1.30E-06	9.95E-07	3.36E-07
	cgd4_3700			
**2h**	0.00E+00	0.00E+00	0.00E+00	0.00E+00
**6h**	9.91E-08	2.29E-08	3.47E-07	2.66E-06
**12h**	4.76E-07	9.53E-07	6.19E-07	4.94E-07
**24h**	5.68E-07	5.21E-07	8.20E-07	8.55E-06
**36h**	4.86E-07	1.93E-06	2.06E-06	9.03E-06
**48h**	4.10E-05	4.71E-05	7.40E-05	1.82E-04
**72h**	5.62E-06	7.73E-06	4.52E-06	6.47E-06
	cgd4_3740			
**2h**	3.51E-05	2.78E-05	1.42E-05	7.22E-05
**6h**	9.01E-06	6.71E-05	5.20E-05	4.84E-04
**12h**	7.22E-08	4.43E-08	3.30E-06	9.34E-06
**24h**	1.32E-05	1.84E-05	9.19E-05	3.23E-04
**36h**	1.13E-05	2.74E-05	3.69E-05	3.26E-05
**48h**	1.20E-05	4.16E-05	3.52E-05	5.90E-05
**72h**	1.04E-05	3.01E-05	6.72E-06	1.30E-05
	cgd4_3750			
**2h**	1.08E-05	3.58E-06	2.65E-05	3.60E-05

**6h**	2.44E-06	2.10E-06	2.70E-05	2.71E-05
**12h**	1.33E-06	7.74E-07	5.42E-06	9.13E-06
**24h**	1.75E-06	2.38E-06	5.87E-06	2.09E-05
**36h**	2.43E-06	2.65E-06	6.29E-06	5.27E-06
**48h**	2.46E-06	2.69E-06	5.18E-06	7.55E-06
**72h**	2.16E-07	4.74E-07	3.65E-07	1.55E-07
	cgd4_3760			
**2h**	3.17E-07	1.36E-07	4.03E-07	1.28E-06
**6h**	1.96E-07	3.23E-07	2.08E-06	1.39E-06
**12h**	2.64E-08	1.37E-07	4.29E-08	1.79E-07
**24h**	7.22E-08	6.35E-08	4.30E-07	4.78E-07
**36h**	1.24E-07	8.54E-07	8.95E-08	2.78E-07
**48h**	5.21E-08	2.01E-07	1.75E-07	2.50E-07
**72h**	1.77E-08	2.98E-07	2.02E-07	2.73E-08
	cgd4_3770			
**2h**	1.43E-02	9.83E-03	1.66E-02	3.58E-02
**6h**	1.54E-02	3.36E-03	1.77E-02	6.89E-02
**12h**	1.76E-03	1.50E-03	8.40E-03	9.89E-03
**24h**	1.20E-03	6.05E-04	9.59E-03	8.04E-03
**36h**	9.82E-04	1.08E-03	6.40E-03	2.08E-03
**48h**	1.02E-03	3.94E-04	4.99E-03	3.68E-03
**72h**	1.59E-04	1.03E-04	5.02E-04	4.78E-04
	cgd4_3790			
**2h**	1.30E-07	6.04E-08	2.16E-08	1.24E-07
**6h**	1.88E-07	7.02E-08	4.49E-07	1.26E-06
**12h**	8.42E-09	1.41E-08	3.84E-08	5.07E-08
**24h**	5.35E-08	1.01E-08	2.88E-07	3.72E-07
**36h**	9.70E-08	1.72E-07	4.68E-07	3.88E-07
**48h**	3.66E-08	2.85E-08	2.04E-07	2.85E-07
**72h**	6.43E-08	3.92E-08	1.35E-07	2.19E-07
	cgd4_3810			
**2h**	1.16E-06	2.03E-06	2.17E-06	1.39E-06
**6h**	3.48E-06	7.83E-07	6.74E-06	5.30E-06
**12h**	5.47E-07	7.15E-07	1.75E-06	5.12E-06
**24h**	6.09E-07	4.17E-07	3.99E-06	3.14E-06
**36h**	9.75E-07	1.13E-06	3.41E-06	5.03E-06
**48h**	3.29E-07	2.22E-07	1.75E-06	1.54E-06
**72h**	3.53E-07	2.06E-07	1.20E-06	1.23E-06
	cgd4_3820			
**2h**	0.00E+00	0.00E+00	0.00E+00	0.00E+00
**6h**	0.00E+00	0.00E+00	0.00E+00	0.00E+00
**12h**	1.53E-07	1.09E-07	4.56E-07	1.71E-06
**24h**	1.84E-08	2.94E-08	6.53E-08	6.52E-08
**36h**	1.79E-07	1.26E-07	1.55E-06	1.52E-06
**48h**	2.21E-08	2.82E-08	3.37E-07	1.61E-07
**72h**	2.86E-08	4.78E-08	4.60E-07	2.40E-07
	cgd4_3830			
**2h**	0.00E+00	0.00E+00	0.00E+00	0.00E+00
**6h**	1.74E-05	4.28E-06	1.16E-05	2.65E-04
**12h**	7.26E-06	5.68E-08	2.18E-05	1.40E-04
**24h**	5.42E-06	1.10E-05	4.37E-05	2.36E-04
**36h**	7.03E-05	8.00E-05	1.06E-04	2.12E-04
**48h**	3.14E-05	3.19E-05	7.38E-05	1.47E-04

**72h**	4.46E-05	5.66E-05	1.91E-04	1.03E-04
	cgd4_3840			
**2h**	0.00E+00	0.00E+00	0.00E+00	0.00E+00
**6h**	0.00E+00	4.49E-09	1.94E-09	1.95E-08
**12h**	7.85E-08	6.79E-08	6.29E-07	7.64E-07
**24h**	3.70E-08	4.72E-08	1.59E-07	3.92E-07
**36h**	7.52E-07	4.71E-07	1.48E-06	1.86E-06
**48h**	1.53E-07	2.58E-07	7.98E-07	9.03E-07
**72h**	4.14E-07	3.56E-07	1.20E-06	8.70E-07
	cgd4_3850			
**2h**	9.82E-06	4.19E-06	5.50E-06	7.63E-07
**6h**	6.20E-05	8.13E-05	1.47E-04	4.49E-04
**12h**	1.43E-06	1.02E-06	2.91E-05	7.50E-05
**24h**	3.84E-06	1.81E-05	3.36E-05	1.56E-04
**36h**	2.71E-05	3.66E-05	5.74E-05	7.52E-05
**48h**	6.15E-06	1.50E-05	1.90E-05	3.61E-05
**72h**	7.80E-07	5.30E-07	5.69E-06	1.65E-06
	cgd4_3860			
**2h**	2.44E-08	7.21E-08	8.94E-08	4.71E-08
**6h**	8.55E-07	1.12E-06	1.50E-06	4.99E-06
**12h**	5.62E-08	4.58E-08	4.27E-07	5.96E-07
**24h**	6.05E-08	1.74E-07	5.69E-07	1.53E-06
**36h**	4.22E-07	2.60E-07	8.61E-07	1.19E-06
**48h**	2.05E-07	2.30E-07	5.14E-07	7.86E-07
**72h**	5.13E-08	2.18E-08	2.44E-07	6.63E-08
	cgd4_3870			
**2h**	0.00E+00	0.00E+00	0.00E+00	0.00E+00
**6h**	0.00E+00	2.04E-08	0.00E+00	0.00E+00
**12h**	9.34E-09	0.00E+00	2.83E-08	0.00E+00
**24h**	8.38E-10	1.32E-07	3.45E-08	3.70E-08
**36h**	2.04E-07	4.82E-07	1.83E-07	5.24E-07
**48h**	1.91E-06	2.45E-06	2.17E-06	4.16E-06
**72h**	4.63E-07	9.95E-07	1.49E-06	1.44E-06
	cgd4_3950			
**2h**	2.35E-07	3.43E-08	2.71E-07	9.08E-08
**6h**	1.40E-06	1.43E-06	3.29E-06	1.17E-05
**12h**	4.24E-07	2.43E-07	8.25E-07	1.62E-06
**24h**	3.88E-07	7.06E-07	2.50E-06	7.57E-06
**36h**	9.96E-07	8.65E-07	2.11E-06	1.80E-06
**48h**	8.50E-07	6.36E-07	2.38E-06	2.84E-06
**72h**	7.15E-08	7.34E-08	6.65E-07	1.66E-07
	cgd4_3970			
**2h**	3.19E-06	9.59E-07	2.43E-06	2.54E-06
**6h**	2.89E-07	3.75E-07	8.45E-07	2.50E-06
**12h**	3.63E-07	1.41E-07	8.81E-07	3.36E-06
**24h**	2.27E-07	3.51E-07	4.88E-07	1.26E-06
**36h**	1.57E-07	1.23E-07	9.96E-07	9.00E-07
**48h**	1.50E-07	1.07E-07	8.24E-07	1.04E-06
**72h**	3.77E-08	4.05E-08	2.23E-07	8.38E-08
	cgd4_3980			
**2h**	1.41E-08	3.95E-07	1.74E-08	1.54E-08
**6h**	3.83E-08	0.00E+00	1.94E-07	6.59E-08
**12h**	7.56E-09	3.99E-07	5.30E-08	8.83E-09

**24h**	3.17E-08	4.30E-06	3.34E-07	1.71E-07
**36h**	1.24E-07	1.24E-05	7.32E-07	3.19E-07
**48h**	3.47E-07	3.35E-05	1.31E-06	1.10E-06
**72h**	2.56E-07	3.97E-05	2.11E-06	1.47E-06
	cgd4_3990			
**2h**	2.03E-07	6.44E-08	2.87E-06	2.71E-08
**6h**	1.02E-07	4.03E-07	3.04E-06	1.13E-06
**12h**	2.30E-08	8.39E-08	2.19E-07	1.56E-06
**24h**	5.83E-08	2.38E-07	1.29E-06	4.06E-07
**36h**	2.29E-07	2.78E-07	8.50E-07	3.50E-07
**48h**	2.14E-07	4.09E-07	7.29E-07	5.00E-07
**72h**	1.21E-07	2.01E-07	3.91E-07	1.86E-07
	cgd4_4000			
**2h**	0.00E+00	0.00E+00	0.00E+00	0.00E+00
**6h**	9.21E-09	0.00E+00	0.00E+00	0.00E+00
**12h**	2.18E-08	0.00E+00	6.21E-09	0.00E+00
**24h**	1.80E-08	7.24E-09	1.44E-08	6.04E-08
**36h**	3.21E-08	2.18E-08	4.33E-08	1.29E-07
**48h**	2.19E-08	3.89E-05	1.69E-07	1.20E-07
**72h**	1.10E-07	1.04E-07	3.15E-07	9.30E-08
	cgd4_4010			
**2h**	0.00E+00	0.00E+00	0.00E+00	0.00E+00
**6h**	2.29E-07	2.75E-07	2.49E-04	5.33E-07
**12h**	3.70E-08	3.61E-08	1.78E-07	3.79E-09
**24h**	1.63E-07	1.39E-07	1.91E-06	1.43E-06
**36h**	2.87E-07	2.68E-07	1.03E-06	5.82E-07
**48h**	2.81E-07	2.43E-07	6.22E-07	5.97E-07
**72h**	2.46E-07	1.66E-07	9.52E-07	2.55E-07
	cgd4_4040			
**2h**	3.95E-08	9.20E-09	6.27E-08	1.19E-08
**6h**	5.07E-09	4.51E-10	1.76E-08	9.36E-08
**12h**	1.86E-08	2.52E-08	1.23E-07	1.74E-07
**24h**	3.07E-08	9.96E-09	5.01E-08	2.50E-08
**36h**	2.08E-08	2.43E-08	1.40E-07	9.29E-08
**48h**	8.85E-09	9.87E-09	2.35E-08	1.34E-08
**72h**	7.79E-09	2.10E-08	3.24E-08	5.19E-08
	cgd4_4050			
**2h**	0.00E+00	0.00E+00	4.69E-08	5.27E-09
**6h**	0.00E+00	0.00E+00	5.11E-08	0.00E+00
**12h**	5.23E-07	4.71E-07	2.03E-06	3.17E-06
**24h**	8.55E-08	1.02E-07	1.05E-07	1.20E-07
**36h**	1.75E-07	1.70E-07	1.84E-06	1.47E-06
**48h**	6.79E-08	8.98E-08	3.27E-07	1.99E-07
**72h**	8.42E-08	3.48E-07	2.73E-07	2.04E-07
	cgd4_4060			
**2h**	0.00E+00	3.46E-08	1.24E-08	0.00E+00
**6h**	0.00E+00	0.00E+00	0.00E+00	0.00E+00
**12h**	7.00E-07	1.12E-06	1.04E-05	1.07E-05
**24h**	7.18E-07	8.64E-07	6.62E-07	1.15E-06
**36h**	3.29E-06	2.59E-06	1.94E-05	6.36E-06
**48h**	1.55E-06	1.51E-06	1.44E-05	2.69E-06
**72h**	4.67E-07	9.60E-07	4.04E-06	5.14E-07
	cgd4_4080			

**2h**	0.00E+00	0.00E+00	0.00E+00	0.00E+00
**6h**	2.40E-09	2.41E-08	3.22E-08	1.58E-07
**12h**	1.73E-07	2.85E-07	4.37E-07	6.43E-07
**24h**	3.18E-08	2.14E-08	3.65E-07	1.57E-07
**36h**	3.06E-07	3.84E-07	1.36E-06	4.74E-07
**48h**	6.59E-08	8.25E-08	4.25E-07	3.57E-07
**72h**	2.92E-08	2.43E-07	1.92E-07	6.23E-08
	cgd4_4100			
**2h**	0.00E+00	2.46E-08	0.00E+00	0.00E+00
**6h**	1.66E-08	2.63E-08	4.52E-07	7.14E-07
**12h**	1.02E-08	2.32E-08	1.25E-07	8.78E-08
**24h**	1.13E-08	1.68E-08	5.22E-06	2.27E-07
**36h**	1.14E-07	1.08E-07	1.32E-06	4.54E-07
**48h**	7.65E-08	1.07E-07	4.63E-07	7.90E-07
**72h**	4.82E-08	3.90E-07	1.21E-07	2.69E-08
	cgd4_4110			
**2h**	1.70E-04	6.64E-05	1.27E-04	1.51E-04
**6h**	8.23E-06	1.67E-05	9.80E-05	1.70E-05
**12h**	3.87E-05	1.69E-05	1.93E-04	3.76E-04
**24h**	1.01E-04	3.41E-05	1.59E-04	1.36E-04
**36h**	3.19E-05	6.09E-05	7.93E-04	4.89E-05
**48h**	3.77E-05	2.39E-05	4.95E-05	4.83E-05
**72h**	7.24E-06	1.57E-05	2.63E-05	7.95E-06
	cgd4_4120			
**2h**	2.56E-09	8.87E-08	0.00E+00	0.00E+00
**6h**	6.05E-08	1.19E-07	1.49E-04	1.56E-06
**12h**	3.38E-07	1.23E-06	3.22E-06	2.70E-06
**24h**	2.52E-07	0.00E+00	2.87E-06	3.20E-06
**36h**	9.54E-07	7.80E-07	5.66E-06	1.81E-06
**48h**	7.29E-07	8.27E-07	2.90E-06	3.38E-06
**72h**	1.73E-07	1.75E-07	1.18E-06	1.86E-07
	cgd4_4140			
**2h**	9.05E-04	1.07E-03	1.48E-02	7.10E-03
**6h**	3.15E-03	4.51E-03	4.28E-02	4.31E-02
**12h**	5.78E-04	1.01E-04	8.17E-03	8.99E-03
**24h**	6.75E-04	2.75E-04	5.42E-03	8.53E-03
**36h**	8.77E-04	1.47E-03	4.58E-03	2.51E-03
**48h**	3.89E-04	8.58E-04	2.20E-03	2.43E-03
**72h**	1.77E-04	9.30E-05	1.22E-03	1.53E-04
	cgd4_4220			
**2h**	2.30E-04	7.17E-05	3.43E-04	3.81E-04
**6h**	1.31E-03	4.67E-05	8.65E-04	2.59E-05
**12h**	8.70E-06	2.84E-08	4.86E-05	3.89E-05
**24h**	2.70E-05	2.22E-05	9.31E-04	2.62E-05
**36h**	3.61E-05	3.84E-05	1.63E-04	3.31E-05
**48h**	4.88E-06	7.66E-06	1.30E-04	2.94E-05
**72h**	6.84E-06	1.29E-05	4.73E-05	8.78E-06
	cgd4_4230			
**2h**	1.05E-06	5.86E-07	2.56E-06	1.31E-06
**6h**	4.95E-08	2.07E-07	2.86E-06	3.73E-07
**12h**	1.36E-07	1.56E-07	9.76E-07	2.71E-06
**24h**	6.02E-07	4.28E-07	7.38E-06	1.57E-06
**36h**	2.04E-07	4.63E-07	1.32E-06	8.91E-07

**48h**	5.09E-08	8.07E-08	1.36E-06	3.12E-07
**72h**	1.66E-07	7.91E-08	8.78E-07	3.17E-07
	cgd4_4240			
**2h**	9.06E-07	7.27E-07	2.50E-06	2.53E-06
**6h**	6.95E-08	1.01E-07	1.88E-06	2.15E-07
**12h**	9.86E-08	3.42E-07	1.94E-06	3.91E-06
**24h**	3.20E-07	1.53E-07	2.97E-06	4.96E-07
**36h**	1.58E-07	3.07E-07	1.20E-06	5.53E-07
**48h**	3.28E-08	4.86E-08	6.72E-07	2.50E-07
**72h**	8.86E-08	1.74E-05	4.34E-04	4.68E-06
	cgd4_4260			
**2h**	0.00E+00	0.00E+00	0.00E+00	9.57E-09
**6h**	0.00E+00	0.00E+00	0.00E+00	0.00E+00
**12h**	0.00E+00	5.12E-08	0.00E+00	0.00E+00
**24h**	0.00E+00	3.48E-10	7.40E-10	6.98E-09
**36h**	2.04E-09	8.58E-10	4.14E-09	9.42E-09
**48h**	1.38E-08	1.52E-08	7.27E-08	8.05E-08
**72h**	6.79E-08	6.14E-08	5.09E-07	3.60E-07
	cgd4_4270			
**2h**	0.00E+00	0.00E+00	0.00E+00	0.00E+00
**6h**	0.00E+00	1.30E-09	0.00E+00	0.00E+00
**12h**	0.00E+00	2.63E-08	1.77E-08	0.00E+00
**24h**	1.80E-10	0.00E+00	1.11E-09	6.62E-10
**36h**	1.39E-08	9.80E-09	8.96E-09	1.31E-08
**48h**	1.41E-07	1.26E-07	9.37E-07	2.82E-07
**72h**	2.14E-08	2.73E-08	1.25E-07	8.40E-08
	cgd4_4280			
**2h**	0.00E+00	1.53E-07	0.00E+00	0.00E+00
**6h**	0.00E+00	1.76E-07	5.87E-07	2.39E-07
**12h**	1.62E-06	4.45E-06	1.20E-05	1.85E-05
**24h**	1.55E-07	6.04E-07	1.39E-05	7.23E-06
**36h**	2.10E-06	3.54E-06	4.93E-05	2.70E-05
**48h**	3.55E-06	4.48E-06	2.12E-05	9.35E-06
**72h**	1.25E-06	1.49E-06	2.26E-05	2.10E-06
	cgd4_4290			
**2h**	9.98E-08	2.95E-06	1.38E-07	0.00E+00
**6h**	2.28E-08	8.67E-09	1.19E-07	2.65E-06
**12h**	4.82E-06	1.86E-05	8.88E-05	2.73E-05
**24h**	6.40E-07	2.49E-06	1.47E-05	1.18E-05
**36h**	4.63E-06	7.27E-06	1.11E-04	3.05E-05
**48h**	4.69E-06	1.05E-05	7.05E-05	2.77E-05
**72h**	3.82E-06	1.58E-06	3.43E-05	5.38E-06
	cgd4_4300			
**2h**	3.30E-08	1.23E-08	3.14E-08	3.56E-07
**6h**	4.02E-08	1.44E-07	8.11E-07	2.06E-06
**12h**	3.02E-08	5.85E-08	2.26E-07	3.88E-07
**24h**	1.68E-08	6.28E-08	9.18E-07	1.16E-06
**36h**	1.96E-07	1.73E-07	8.80E-07	6.30E-07
**48h**	1.38E-07	1.12E-07	5.25E-07	4.02E-07
**72h**	2.47E-08	2.37E-08	2.86E-07	2.04E-08
	cgd4_4310			
**2h**	2.63E-06	8.19E-06	6.17E-06	4.54E-06
**6h**	3.39E-08	6.87E-08	7.49E-07	2.47E-07

**12h**	4.55E-07	7.00E-07	4.34E-06	4.39E-06
**24h**	6.07E-07	1.37E-06	1.46E-06	3.51E-06
**36h**	3.65E-07	3.31E-07	3.91E-06	9.48E-07
**48h**	1.47E-07	3.36E-07	1.52E-06	1.15E-06
**72h**	0.00E+00	5.44E-08	7.11E-07	1.02E-07
	cgd4_4320			
**2h**	4.47E-08	0.00E+00	0.00E+00	0.00E+00
**6h**	2.83E-08	4.57E-08	4.11E-07	1.30E-08
**12h**	1.71E-08	3.76E-08	1.73E-08	1.88E-07
**24h**	8.02E-09	4.10E-08	7.90E-07	4.04E-07
**36h**	5.64E-08	1.34E-07	1.10E-06	1.83E-07
**48h**	1.19E-07	1.05E-07	5.24E-07	3.82E-07
**72h**	1.07E-08	1.63E-08	1.30E-07	1.48E-08
	cgd4_4330			
**2h**	0.00E+00	1.21E-05	5.53E-07	5.69E-08
**6h**	2.27E-07	7.90E-07	3.91E-06	3.57E-06
**12h**	1.30E-05	2.98E-05	4.38E-04	8.51E-05
**24h**	2.75E-06	6.91E-06	5.55E-05	3.87E-05
**36h**	1.23E-06	3.16E-06	1.56E-04	2.23E-05
**48h**	3.34E-06	2.71E-06	4.27E-05	1.51E-05
**72h**	4.97E-07	4.17E-07	3.29E-05	2.18E-06
	cgd4_4340			
**2h**	0.00E+00	2.74E-07	0.00E+00	0.00E+00
**6h**	2.11E-06	2.01E-05	9.39E-05	8.75E-05
**12h**	2.20E-06	4.58E-06	2.85E-05	1.79E-05
**24h**	2.17E-06	4.16E-06	1.03E-04	4.58E-05
**36h**	6.78E-06	5.83E-06	1.71E-04	2.54E-05
**48h**	3.33E-06	5.06E-06	7.37E-05	2.18E-05
**72h**	2.13E-06	1.11E-06	1.90E-05	1.66E-06
	cgd4_4350			
**2h**	0.00E+00	0.00E+00	1.12E-05	1.85E-05
**6h**	2.90E-08	0.00E+00	0.00E+00	4.38E-05
**12h**	2.83E-06	0.00E+00	2.77E-04	2.58E-06
**24h**	1.19E-06	2.89E-05	2.61E-04	1.02E-05
**36h**	8.40E-06	1.52E-04	3.15E-04	6.50E-05
**48h**	1.64E-05	1.71E-04	8.24E-04	2.36E-05
**72h**	1.82E-05	8.65E-05	2.56E-04	7.36E-06
	cgd4_4360			
**2h**	0.00E+00	0.00E+00	0.00E+00	0.00E+00
**6h**	2.08E-09	1.43E-09	0.00E+00	2.23E-08
**12h**	1.69E-09	7.09E-10	9.67E-09	8.72E-09
**24h**	9.89E-09	6.17E-09	2.75E-07	5.05E-08
**36h**	2.21E-08	1.55E-08	2.40E-07	1.05E-07
**48h**	2.22E-08	1.20E-08	6.20E-08	7.86E-08
**72h**	6.33E-09	4.67E-09	6.15E-08	1.17E-08
	cgd4_4370			
**2h**	0.00E+00	0.00E+00	0.00E+00	0.00E+00
**6h**	2.15E-08	1.42E-08	1.30E-08	3.22E-07
**12h**	2.12E-09	2.46E-09	1.06E-08	1.20E-07
**24h**	1.49E-08	8.20E-09	1.74E-07	3.68E-07
**36h**	4.84E-08	2.82E-08	1.50E-07	1.55E-07
**48h**	2.15E-08	1.73E-08	2.83E-08	1.04E-07
**72h**	5.22E-09	1.82E-08	3.79E-09	1.58E-08

	cgd4_4390			
**2h**	1.24E-06	6.54E-06	1.55E-04	5.97E-05
**6h**	2.53E-06	9.08E-06	3.24E-06	2.67E-05
**12h**	7.72E-07	3.03E-07	3.34E-06	1.34E-05
**24h**	1.67E-06	3.17E-06	4.38E-05	3.34E-05
**36h**	2.52E-06	3.22E-06	4.95E-05	1.68E-05
**48h**	5.98E-06	2.39E-05	5.62E-05	2.82E-05
**72h**	1.02E-06	9.86E-07	1.43E-05	5.09E-06
	cgd4_4400			
**2h**	0.00E+00	0.00E+00	0.00E+00	0.00E+00
**6h**	0.00E+00	0.00E+00	1.61E-09	0.00E+00
**12h**	7.20E-09	3.35E-09	0.00E+00	4.17E-09
**24h**	1.05E-10	6.87E-10	2.65E-10	0.00E+00
**36h**	3.10E-10	4.96E-09	9.06E-09	1.47E-09
**48h**	4.80E-09	1.45E-08	2.91E-08	2.02E-08
**72h**	1.38E-08	2.31E-08	4.37E-08	3.06E-08
	cgd4_4410			
**2h**	9.22E-08	7.31E-09	4.41E-07	1.43E-08
**6h**	7.36E-08	1.20E-07	3.16E-07	2.51E-07
**12h**	1.98E-08	2.30E-08	1.46E-07	6.58E-07
**24h**	2.36E-09	1.06E-08	1.66E-07	4.16E-07
**36h**	8.77E-08	6.58E-08	1.14E-06	7.16E-07
**48h**	1.81E-08	1.63E-08	3.98E-07	4.03E-07
**72h**	1.67E-08	4.84E-08	1.25E-07	5.35E-08
	cgd4_4420			
**2h**	5.87E-07	6.92E-08	3.42E-06	4.79E-06
**6h**	5.84E-08	4.19E-08	3.82E-07	1.54E-07
**12h**	8.25E-08	8.72E-08	3.76E-07	6.31E-07
**24h**	3.98E-08	3.52E-08	2.81E-07	6.03E-07
**36h**	1.30E-07	1.64E-07	6.94E-07	4.38E-07
**48h**	3.32E-08	3.09E-08	3.38E-07	3.90E-07
**72h**	2.56E-08	3.31E-08	1.16E-07	3.04E-08
	cgd4_4440			
**2h**	0.00E+00	0.00E+00	0.00E+00	9.17E-09
**6h**	3.09E-09	2.87E-09	4.15E-09	3.88E-09
**12h**	0.00E+00	0.00E+00	7.21E-10	1.44E-08
**24h**	9.31E-10	5.06E-10	0.00E+00	5.75E-09
**36h**	1.12E-08	8.11E-09	6.67E-09	1.33E-08
**48h**	2.94E-09	3.73E-09	1.73E-08	2.55E-08
**72h**	4.72E-09	2.63E-09	1.17E-08	8.80E-10
	cgd4_4450			
**2h**	1.32E-08	0.00E+00	0.00E+00	5.14E-08
**6h**	0.00E+00	0.00E+00	1.17E-07	2.93E-08
**12h**	2.04E-06	1.43E-06	6.33E-06	8.27E-06
**24h**	3.63E-07	3.08E-07	5.84E-06	9.29E-06
**36h**	2.84E-06	2.81E-06	2.30E-05	1.70E-05
**48h**	2.55E-06	4.09E-06	1.06E-05	1.81E-05
**72h**	8.73E-07	9.20E-07	5.74E-06	3.98E-07
	cgd4_4460			
**2h**	4.68E-07	4.13E-08	1.42E-07	6.39E-07
**6h**	4.00E-07	5.33E-07	3.78E-06	5.08E-06
**12h**	1.46E-07	4.58E-07	3.90E-07	1.02E-06
**24h**	2.12E-07	1.38E-07	2.91E-06	5.46E-06

**36h**	9.81E-07	9.91E-07	2.22E-06	2.92E-06
**48h**	8.16E-07	9.99E-07	1.41E-06	2.79E-06
**72h**	6.80E-08	6.24E-08	1.71E-07	5.58E-08
	cgd4_4470			
**2h**	0.00E+00	0.00E+00	1.33E-08	3.43E-03
**6h**	1.22E-08	4.08E-09	3.96E-09	3.96E-06
**12h**	1.99E-08	7.63E-08	1.48E-08	1.22E-05
**24h**	2.81E-08	2.05E-08	2.62E-07	4.64E-05
**36h**	1.06E-07	7.11E-08	3.83E-07	1.05E-05
**48h**	7.50E-08	1.22E-07	2.17E-07	3.52E-05
**72h**	4.25E-09	2.60E-09	3.53E-08	2.79E-04
	cgd4_4480			
**2h**	0.00E+00	0.00E+00	0.00E+00	0.00E+00
**6h**	0.00E+00	0.00E+00	0.00E+00	0.00E+00
**12h**	4.69E-08	4.52E-08	1.75E-07	7.42E-08
**24h**	5.08E-09	7.23E-09	1.83E-08	4.23E-08
**36h**	1.11E-08	1.18E-08	9.20E-08	6.17E-08
**48h**	1.78E-09	5.18E-09	4.01E-09	1.92E-08
**72h**	2.13E-10	4.27E-10	8.66E-10	2.55E-09
	cgd4_4500			
**2h**	3.17E-05	4.68E-03	1.19E-04	1.13E-04
**6h**	7.35E-06	3.70E-04	6.11E-05	2.19E-05
**12h**	4.49E-05	1.52E-04	1.95E-04	9.54E-05
**24h**	1.39E-05	1.44E-05	1.04E-04	5.65E-05
**36h**	5.88E-06	1.26E-05	7.81E-05	2.83E-05
**48h**	3.03E-06	1.09E-05	4.11E-05	1.55E-05
**72h**	2.85E-06	2.77E-05	3.04E-05	4.05E-06
	cgd5_20			
**2h**	2.44E-09	0.00E+00	3.02E-09	1.68E-08
**6h**	2.86E-09	2.42E-09	1.09E-08	0.00E+00
**12h**	6.83E-07	2.43E-07	2.96E-06	2.61E-06
**24h**	2.06E-07	1.99E-07	7.03E-07	3.18E-07
**36h**	1.25E-07	7.43E-08	1.50E-06	5.85E-07
**48h**	3.38E-08	2.41E-08	1.28E-07	1.02E-07
**72h**	2.48E-08	2.58E-09	2.45E-08	1.09E-08
	cgd5_30			
**2h**	1.04E-06	1.10E-06	4.29E-06	3.63E-06
**6h**	4.52E-07	9.60E-07	1.56E-06	2.27E-06
**12h**	2.73E-06	7.83E-07	1.07E-05	7.72E-06
**24h**	1.95E-06	1.05E-06	1.69E-06	1.94E-06
**36h**	3.17E-07	4.71E-07	2.15E-06	1.30E-06
**48h**	5.86E-07	1.29E-07	4.55E-07	5.98E-07
**72h**	2.75E-07	1.19E-07	5.12E-07	1.51E-07
	cgd5_40			
**2h**	7.30E-07	5.56E-07	4.00E-06	3.67E-06
**6h**	8.00E-07	9.65E-07	1.11E-06	1.66E-06
**12h**	2.55E-06	5.84E-07	1.28E-05	1.28E-05
**24h**	2.61E-06	6.38E-07	1.55E-06	1.63E-06
**36h**	2.41E-07	4.67E-07	2.44E-06	2.79E-06
**48h**	4.49E-07	9.68E-08	7.50E-07	7.91E-07
**72h**	8.53E-08	2.60E-07	5.52E-07	5.33E-07
	cgd5_50			
**2h**	0.00E+00	0.00E+00	0.00E+00	0.00E+00

**6h**	1.51E-08	2.20E-09	1.52E-08	1.87E-08
**12h**	1.15E-07	7.95E-08	7.49E-07	5.26E-07
**24h**	9.26E-08	1.63E-08	7.04E-08	2.16E-07
**36h**	1.21E-07	1.73E-07	7.86E-07	5.92E-07
**48h**	1.75E-07	1.11E-07	3.68E-07	4.35E-07
**72h**	6.05E-08	9.10E-08	2.67E-07	3.28E-07
	cgd5_60			
**2h**	2.86E-07	2.08E-07	4.61E-08	0.00E+00
**6h**	8.71E-07	8.07E-07	7.44E-06	5.75E-06
**12h**	5.41E-06	4.01E-06	4.45E-05	2.93E-05
**24h**	1.42E-06	6.62E-07	3.73E-06	4.00E-06
**36h**	1.99E-06	5.26E-06	1.69E-05	1.30E-05
**48h**	1.58E-06	7.49E-07	3.84E-06	3.75E-06
**72h**	1.17E-06	1.23E-06	2.53E-06	1.23E-06
	cgd5_70			
**2h**	0.00E+00	1.97E-07	9.50E-08	2.84E-07
**6h**	0.00E+00	4.43E-08	2.17E-07	2.07E-08
**12h**	8.62E-07	7.54E-07	1.45E-05	8.59E-06
**24h**	3.56E-07	2.46E-07	6.18E-07	1.03E-06
**36h**	4.00E-07	6.27E-07	5.25E-06	6.13E-06
**48h**	4.31E-07	4.41E-07	1.21E-06	1.13E-06
**72h**	3.58E-07	1.28E-07	1.84E-07	2.60E-07
	cgd5_80			
**2h**	0.00E+00	0.00E+00	0.00E+00	0.00E+00
**6h**	0.00E+00	0.00E+00	0.00E+00	0.00E+00
**12h**	7.92E-09	1.92E-09	0.00E+00	1.73E-09
**24h**	4.98E-09	0.00E+00	9.98E-10	1.21E-09
**36h**	1.88E-08	3.11E-08	3.52E-08	1.40E-08
**48h**	6.81E-07	4.44E-07	7.95E-07	4.95E-07
**72h**	3.42E-08	1.17E-08	2.00E-09	3.96E-08
	cgd5_100			
**2h**	4.91E-07	6.07E-06	1.08E-05	5.80E-06
**6h**	1.95E-07	5.39E-08	1.12E-06	3.98E-07
**12h**	6.87E-07	4.13E-07	1.37E-05	1.30E-05
**24h**	8.93E-07	5.22E-07	1.27E-06	2.96E-06
**36h**	3.66E-07	3.06E-07	1.45E-06	1.55E-06
**48h**	1.80E-07	2.53E-07	1.07E-06	1.52E-06
**72h**	1.28E-07	6.00E-08	1.31E-07	9.16E-08
	cgd5_110			
**2h**	4.09E-05	2.11E-04	7.08E-06	2.10E-07
**6h**	1.97E-05	3.81E-05	4.35E-05	2.26E-05
**12h**	1.09E-06	2.94E-05	1.98E-06	4.55E-05
**24h**	2.10E-05	7.00E-05	7.12E-05	2.28E-05
**36h**	7.09E-05	1.08E-04	9.99E-06	7.20E-06
**48h**	1.72E-04	1.47E-04	3.98E-05	5.53E-05
**72h**	1.35E-05	1.01E-04	4.50E-06	7.95E-06
	cgd5_120			
**2h**	7.90E-09	3.60E-07	9.59E-08	3.93E-07
**6h**	3.07E-09	3.89E-09	3.77E-08	8.12E-08
**12h**	1.49E-08	1.66E-08	1.71E-07	6.17E-08
**24h**	6.20E-09	1.33E-08	3.48E-08	9.51E-08
**36h**	2.03E-08	1.52E-08	3.24E-08	1.10E-08
**48h**	2.45E-08	1.65E-08	3.93E-08	4.58E-08

**72h**	1.58E-08	6.88E-09	3.60E-08	7.19E-09
	cgd5_130			
**2h**	1.23E-08	3.82E-08	1.46E-08	4.65E-08
**6h**	3.70E-08	5.80E-08	1.75E-06	2.84E-07
**12h**	1.40E-08	1.27E-08	5.78E-07	8.15E-07
**24h**	1.40E-08	3.19E-08	9.16E-08	3.09E-07
**36h**	1.84E-07	5.51E-08	2.70E-07	1.54E-07
**48h**	2.56E-08	1.79E-08	1.16E-07	1.24E-07
**72h**	2.55E-08	4.06E-09	1.57E-08	1.77E-08
	cgd5_150			
**2h**	1.07E-07	2.09E-07	2.84E-07	7.00E-07
**6h**	1.73E-08	1.91E-07	1.57E-06	6.63E-07
**12h**	9.36E-09	3.64E-09	4.90E-07	2.88E-07
**24h**	2.20E-08	1.66E-07	7.07E-07	5.61E-07
**36h**	1.37E-07	1.31E-07	1.75E-06	4.27E-07
**48h**	7.58E-08	6.91E-08	1.21E-06	2.13E-07
**72h**	1.26E-07	7.78E-08	1.90E-06	8.40E-08
	cgd2_140			
**2h**	1.54E-07	1.97E-08	2.72E-07	5.15E-07
**6h**	2.37E-07	1.53E-07	8.64E-08	1.93E-06
**12h**	6.72E-08	2.70E-08	5.77E-08	5.64E-07
**24h**	1.37E-07	7.44E-08	1.17E-06	5.35E-07
**36h**	1.02E-07	7.43E-08	3.68E-07	2.50E-07
**48h**	8.01E-08	3.02E-08	8.38E-07	7.04E-07
**72h**	3.29E-08	2.39E-08	1.13E-07	7.74E-08
	cgd2_160			
**2h**	1.33E-08	1.21E-09	3.77E-07	1.17E-07
**6h**	1.05E-08	2.73E-08	2.04E-09	6.13E-09
**12h**	1.92E-08	4.71E-08	7.22E-08	3.25E-07
**24h**	5.18E-08	9.66E-08	4.10E-07	1.79E-07
**36h**	9.65E-06	3.87E-08	1.92E-07	8.03E-08
**48h**	7.93E-08	4.09E-08	4.91E-07	9.88E-08
**72h**	5.26E-08	3.84E-08	2.93E-07	5.54E-08
	cgd2_180			
**2h**	6.57E-06	3.16E-06	5.36E-05	2.21E-05
**6h**	4.45E-06	1.67E-05	5.08E-05	8.43E-05
**12h**	4.34E-07	3.06E-07	4.75E-06	2.12E-05
**24h**	3.58E-06	2.37E-06	1.51E-04	3.09E-05
**36h**	3.37E-06	3.41E-06	6.36E-05	1.74E-05
**48h**	1.33E-06	9.09E-07	9.00E-05	1.56E-05
**72h**	1.14E-06	1.17E-06	3.48E-05	4.47E-06
	cgd2_240			
**2h**	0.00E+00	0.00E+00	0.00E+00	0.00E+00
**6h**	0.00E+00	1.37E-09	3.12E-09	0.00E+00
**12h**	1.06E-06	3.02E-07	3.35E-07	2.73E-06
**24h**	5.12E-08	2.47E-08	6.44E-08	9.59E-08
**36h**	2.12E-07	8.99E-07	2.02E-06	1.61E-06
**48h**	2.62E-07	1.74E-07	1.37E-06	3.00E-07
**72h**	1.05E-06	1.67E-07	1.62E-06	7.75E-07
	cgd2_250			
**2h**	3.13E-06	2.94E-06	3.75E-06	6.46E-06
**6h**	1.54E-05	1.18E-06	2.74E-06	1.29E-05
**12h**	2.76E-06	1.61E-06	1.69E-06	9.75E-06

**24h**	9.04E-07	6.62E-07	5.51E-06	5.62E-05
**36h**	2.47E-06	9.10E-07	3.98E-06	4.67E-06
**48h**	3.94E-07	2.93E-07	2.53E-06	1.08E-06
**72h**	5.09E-07	8.50E-08	1.10E-06	4.96E-07
	cgd2_260			
**2h**	1.25E-07	4.65E-08	1.32E-06	4.22E-06
**6h**	0.00E+00	1.37E-08	1.65E-08	4.91E-08
**12h**	8.85E-07	7.44E-07	1.52E-06	4.32E-06
**24h**	9.37E-08	1.59E-07	7.95E-07	1.72E-07
**36h**	1.58E-07	1.38E-06	1.18E-06	5.21E-07
**48h**	3.67E-07	6.77E-08	8.69E-07	7.86E-07
**72h**	1.61E-07	6.52E-08	7.96E-07	2.54E-07
	cgd2_290			
**2h**	5.02E-08	0.00E+00	2.64E-08	1.37E-07
**6h**	7.21E-09	0.00E+00	3.85E-08	8.32E-09
**12h**	8.96E-08	2.48E-07	3.99E-07	3.15E-06
**24h**	2.62E-07	4.43E-07	2.32E-06	1.21E-06
**36h**	2.18E-06	1.46E-06	1.15E-05	6.90E-06
**48h**	2.19E-06	1.42E-06	7.30E-06	4.10E-06
**72h**	7.85E-07	6.17E-07	1.40E-05	1.19E-06
	cgd2_320			
**2h**	1.05E-04	4.52E-04	5.19E-05	2.76E-05
**6h**	1.36E-04	4.89E-05	1.26E-04	4.72E-04
**12h**	1.84E-05	2.75E-06	2.97E-05	3.50E-05
**24h**	2.39E-05	8.08E-05	1.24E-04	6.48E-05
**36h**	1.57E-05	7.30E-06	1.36E-04	3.75E-05
**48h**	2.75E-05	1.04E-05	8.15E-05	4.06E-05
**72h**	1.49E-05	1.51E-05	1.44E-04	1.74E-04
	cgd2_330			
**2h**	8.18E-07	2.75E-07	1.27E-06	2.42E-06
**6h**	1.98E-07	1.20E-07	3.10E-07	3.19E-07
**12h**	2.85E-07	5.35E-08	3.29E-07	3.25E-06
**24h**	2.87E-07	1.15E-04	5.97E-07	4.58E-07
**36h**	2.43E-07	8.71E-08	8.54E-07	5.31E-07
**48h**	2.21E-07	9.86E-08	4.66E-07	5.62E-07
**72h**	2.27E-08	0.00E+00	8.11E-07	4.88E-08
	cgd2_340			
**2h**	7.41E-07	1.26E-07	7.36E-07	2.16E-06
**6h**	0.00E+00	0.00E+00	2.83E-09	0.00E+00
**12h**	9.56E-07	3.02E-07	1.45E-06	5.83E-06
**24h**	5.10E-07	5.78E-07	3.41E-07	1.02E-06
**36h**	5.36E-08	4.34E-08	9.33E-07	5.03E-07
**48h**	4.90E-08	4.70E-08	3.31E-07	2.68E-07
**72h**	2.21E-09	5.42E-09	4.52E-07	2.49E-08
	cgd2_370			
**2h**	0.00E+00	0.00E+00	0.00E+00	2.46E-08
**6h**	0.00E+00	0.00E+00	0.00E+00	0.00E+00
**12h**	1.01E-05	4.12E-06	4.10E-06	1.71E-05
**24h**	1.17E-06	8.24E-07	8.68E-07	8.66E-07
**36h**	3.36E-07	2.08E-07	1.25E-05	2.55E-06
**48h**	2.28E-07	1.49E-07	1.60E-06	1.26E-06
**72h**	3.74E-08	8.70E-09	1.15E-06	2.78E-08
	cgd2_390			

**2h**	3.10E-08	6.76E-08	1.74E-07	7.63E-08
**6h**	1.42E-07	5.38E-08	1.01E-06	4.25E-07
**12h**	7.87E-07	3.12E-07	4.78E-06	2.15E-06
**24h**	1.60E-07	1.55E-07	9.12E-07	2.77E-07
**36h**	1.97E-07	1.01E-07	1.14E-05	1.15E-06
**48h**	3.64E-07	1.14E-07	4.25E-06	6.21E-07
**72h**	4.65E-08	2.76E-08	8.82E-07	9.59E-08
	cgd1_200			
**2h**	0.00E+00	0.00E+00	8.33E-08	0.00E+00
**6h**	0.00E+00	5.15E-09	0.00E+00	0.00E+00
**12h**	0.00E+00	6.32E-08	1.20E-07	5.89E-09
**24h**	4.85E-08	3.21E-08	2.03E-07	5.40E-08
**36h**	1.85E-07	4.42E-08	6.21E-07	3.36E-07
**48h**	1.41E-07	7.16E-08	6.00E-07	2.47E-07
**72h**	1.08E-07	1.13E-07	1.89E-07	2.14E-07
	cgd1_210			
**2h**	2.32E-04	3.59E-05	0.00E+00	0.00E+00
**6h**	1.96E-05	4.27E-05	3.91E-04	0.00E+00
**12h**	2.36E-04	1.25E-03	3.00E-04	7.67E-04
**24h**	5.51E-05	2.77E-04	6.48E-04	9.26E-04
**36h**	3.82E-04	5.09E-04	2.29E-03	2.66E-03
**48h**	2.22E-04	1.75E-04	9.60E-04	4.41E-04
**72h**	1.20E-04	1.70E-04	4.13E-04	5.09E-04
	cgd1_220			
**2h**	0.00E+00	0.00E+00	0.00E+00	0.00E+00
**6h**	3.18E-08	9.22E-08	1.35E-07	9.29E-07
**12h**	1.83E-08	2.55E-09	2.27E-09	1.85E-08
**24h**	2.73E-08	1.39E-08	1.92E-07	6.00E-07
**36h**	1.79E-07	5.07E-08	4.38E-07	5.06E-07
**48h**	6.67E-08	2.82E-08	1.50E-07	1.42E-07
**72h**	4.06E-08	4.22E-08	1.32E-07	8.75E-08
	cgd1_230			
**2h**	6.03E-09	2.50E-09	0.00E+00	0.00E+00
**6h**	3.38E-08	1.17E-09	7.79E-08	1.81E-07
**12h**	8.69E-09	6.25E-09	5.15E-09	5.00E-09
**24h**	9.09E-09	3.50E-09	3.87E-08	7.69E-08
**36h**	1.89E-07	5.44E-08	3.85E-07	2.12E-07
**48h**	2.47E-08	1.76E-08	7.19E-08	7.57E-08
**72h**	1.09E-07	8.49E-08	3.92E-07	3.11E-07
	cgd1_240			
**2h**	6.85E-06	6.82E-07	4.64E-05	0.00E+00
**6h**	1.52E-06	1.72E-05	8.15E-06	2.91E-06
**12h**	3.18E-05	3.39E-06	3.25E-05	3.58E-06
**24h**	1.06E-04	4.73E-06	6.24E-05	2.07E-04
**36h**	4.13E-05	3.14E-05	1.79E-04	1.34E-04
**48h**	5.82E-05	2.21E-05	8.82E-05	5.26E-05
**72h**	1.60E-04	1.26E-05	2.38E-05	6.58E-06
	cgd1_250			
**2h**	2.42E-07	4.79E-07	1.17E-06	4.49E-08
**6h**	0.00E+00	9.01E-09	4.58E-08	0.00E+00
**12h**	1.24E-07	2.09E-08	2.36E-07	1.86E-07
**24h**	1.48E-07	6.23E-08	9.09E-08	5.57E-07
**36h**	2.58E-07	2.34E-07	8.76E-07	7.16E-07

**48h**	2.84E-07	1.49E-07	3.42E-07	3.65E-07
**72h**	7.61E-08	2.11E-07	6.94E-07	5.80E-07
	cgd1_260			
**2h**	0.00E+00	0.00E+00	0.00E+00	0.00E+00
**6h**	0.00E+00	0.00E+00	3.34E-09	0.00E+00
**12h**	1.06E-09	4.28E-10	0.00E+00	0.00E+00
**24h**	1.94E-08	1.16E-09	2.02E-09	5.61E-08
**36h**	2.77E-08	3.61E-08	1.43E-07	8.05E-08
**48h**	1.62E-07	9.00E-08	6.97E-08	3.55E-07
**72h**	9.31E-08	6.48E-08	2.99E-07	4.54E-08
	cgd1_270			
**2h**	4.60E-07	2.69E-07	7.55E-07	1.28E-06
**6h**	3.74E-07	1.21E-07	4.89E-07	3.29E-06
**12h**	1.36E-08	5.01E-08	1.74E-07	2.15E-07
**24h**	6.12E-08	2.83E-08	3.42E-07	6.47E-06
**36h**	2.71E-07	1.60E-07	5.77E-07	5.09E-07
**48h**	4.06E-07	1.24E-07	5.44E-07	8.12E-07
**72h**	4.78E-08	5.26E-08	1.16E-07	6.06E-08
	cgd1_280			
**2h**	4.33E-08	4.93E-08	3.08E-07	0.00E+00
**6h**	1.32E-07	3.81E-08	5.80E-08	7.70E-07
**12h**	2.23E-07	7.81E-08	1.46E-07	1.52E-07
**24h**	1.18E-07	3.46E-08	4.10E-07	8.70E-07
**36h**	6.66E-07	2.72E-07	1.20E-06	7.33E-07
**48h**	1.64E-06	9.46E-07	1.53E-06	1.58E-06
**72h**	1.68E-07	1.07E-07	2.36E-07	1.74E-07
	cgd1_370			
**2h**	9.58E-04	3.52E-04	2.28E-03	3.24E-04
**6h**	4.58E-04	3.24E-04	7.45E-04	1.08E-02
**12h**	5.17E-04	1.82E-04	4.24E-04	8.84E-04
**24h**	1.46E-04	5.55E-04	1.15E-03	1.89E-03
**36h**	5.57E-04	3.86E-04	3.44E-03	1.63E-03
**48h**	4.32E-04	6.06E-04	1.39E-03	2.72E-03
**72h**	1.87E-04	4.51E-04	9.61E-04	1.32E-03
	cgd1_380			
**2h**	0.00E+00	0.00E+00	0.00E+00	0.00E+00
**6h**	0.00E+00	1.54E-09	0.00E+00	1.03E-06
**12h**	8.69E-08	1.22E-08	1.80E-08	9.43E-08
**24h**	1.93E-07	2.47E-07	7.96E-07	3.45E-06
**36h**	2.01E-06	3.19E-06	7.69E-06	3.02E-06
**48h**	4.49E-06	2.26E-06	7.65E-06	4.57E-06
**72h**	3.12E-06	5.85E-07	8.45E-06	2.11E-06
	cgd1_390			
**2h**	0.00E+00	0.00E+00	0.00E+00	0.00E+00
**6h**	0.00E+00	0.00E+00	0.00E+00	0.00E+00
**12h**	2.95E-06	9.43E-07	2.53E-06	7.78E-07
**24h**	3.70E-08	2.59E-07	1.72E-07	6.65E-07
**36h**	1.42E-06	1.91E-07	1.22E-05	6.80E-06
**48h**	3.59E-07	5.83E-07	2.56E-07	7.86E-07
**72h**	1.30E-07	5.43E-08	7.57E-08	7.91E-07
	cgd1_400			
**2h**	0.00E+00	0.00E+00	0.00E+00	0.00E+00
**6h**	0.00E+00	5.66E-08	3.33E-09	3.91E-09

**12h**	3.57E-08	4.58E-08	1.17E-07	2.99E-07
**24h**	1.39E-07	1.78E-08	5.97E-07	8.37E-08
**36h**	8.34E-08	8.28E-08	8.16E-07	3.99E-07
**48h**	2.24E-07	1.07E-07	4.78E-07	3.72E-07
**72h**	5.69E-08	5.85E-08	7.94E-07	1.09E-07
	cgd1_410			
**2h**	1.62E-08	0.00E+00	0.00E+00	0.00E+00
**6h**	2.68E-08	6.24E-07	3.91E-07	1.22E-06
**12h**	9.66E-08	2.87E-08	8.34E-08	2.57E-07
**24h**	6.22E-08	4.04E-08	1.01E-06	7.38E-07
**36h**	3.43E-07	8.96E-08	1.33E-06	5.77E-07
**48h**	1.10E-07	5.20E-08	3.07E-07	3.13E-07
**72h**	6.04E-08	5.08E-08	4.40E-07	1.12E-07
	cgd1_420			
**2h**	1.79E-08	3.30E-09	6.07E-08	0.00E+00
**6h**	1.92E-08	5.55E-08	6.17E-08	2.22E-07
**12h**	0.00E+00	0.00E+00	0.00E+00	3.97E-09
**24h**	6.18E-09	3.02E-09	2.90E-08	4.54E-08
**36h**	2.25E-08	7.92E-09	1.24E-07	5.86E-08
**48h**	2.10E-08	1.03E-08	2.11E-08	2.20E-08
**72h**	2.57E-08	3.48E-08	2.18E-07	8.91E-08
	cgd1_430			
**2h**	1.82E-07	1.14E-07	6.62E-06	9.31E-07
**6h**	5.16E-08	1.42E-08	2.62E-06	1.43E-06
**12h**	8.08E-07	5.19E-08	2.64E-07	4.83E-07
**24h**	2.46E-07	3.52E-07	2.34E-06	3.73E-06
**36h**	2.25E-06	1.46E-06	5.76E-06	4.61E-06
**48h**	1.83E-06	2.13E-06	1.16E-05	5.77E-06
**72h**	4.04E-06	2.72E-06	2.38E-05	6.75E-06
	cgd1_440			
**2h**	0.00E+00	0.00E+00	0.00E+00	0.00E+00
**6h**	0.00E+00	1.27E-09	6.11E-09	7.07E-09
**12h**	3.18E-07	1.31E-07	2.97E-07	9.52E-07
**24h**	1.06E-08	1.57E-08	2.40E-08	1.34E-08
**36h**	4.98E-08	2.18E-08	2.95E-07	3.86E-07
**48h**	1.62E-08	4.17E-09	3.02E-08	3.39E-08
**72h**	6.97E-09	2.98E-08	1.45E-07	2.35E-08
	cgd1_450			
**2h**	1.63E-07	0.00E+00	0.00E+00	1.17E-07
**6h**	0.00E+00	0.00E+00	0.00E+00	0.00E+00
**12h**	1.88E-04	7.61E-05	1.75E-03	1.08E-03
**24h**	6.54E-05	3.14E-05	1.03E-04	2.94E-05
**36h**	5.45E-05	2.32E-05	4.84E-04	4.29E-04
**48h**	1.46E-04	9.27E-06	4.37E-05	1.50E-05
**72h**	1.29E-06	5.45E-06	8.95E-06	2.38E-05
	cgd1_460			
**2h**	0.00E+00	0.00E+00	4.78E-08	0.00E+00
**6h**	6.89E-08	0.00E+00	2.92E-08	1.78E-07
**12h**	5.08E-08	8.97E-08	1.86E-06	1.41E-06
**24h**	3.50E-07	1.30E-07	8.22E-07	1.00E-06
**36h**	1.40E-06	1.30E-06	3.84E-06	4.17E-06
**48h**	7.49E-07	6.91E-07	3.22E-06	2.36E-06
**72h**	3.44E-07	4.27E-07	3.39E-06	5.53E-07

	cgd1_480			
**2h**	4.14E-05	1.54E-02	1.23E-03	2.89E-04
**6h**	6.21E-04	4.07E-04	2.41E-04	3.23E-04
**12h**	6.52E-04	4.53E-04	6.22E-04	5.49E-03
**24h**	2.14E-04	1.36E-04	7.54E-04	2.66E-03
**36h**	1.67E-04	1.35E-04	7.20E-04	7.73E-04
**48h**	5.20E-05	2.05E-04	1.18E-04	4.58E-04
**72h**	9.80E-05	1.42E-04	7.52E-04	4.09E-04
	cgd1_510			
**2h**	5.43E-08	7.10E-08	6.89E-07	2.23E-07
**6h**	1.74E-08	2.67E-08	7.26E-08	3.07E-07
**12h**	9.58E-08	2.36E-08	7.51E-07	5.51E-07
**24h**	6.43E-08	5.89E-08	2.65E-07	4.06E-07
**36h**	1.20E-07	1.18E-07	1.03E-06	7.10E-07
**48h**	3.62E-07	1.30E-07	4.33E-07	5.86E-07
**72h**	1.31E-07	2.08E-07	3.95E-07	2.84E-07
	cgd1_540			
**2h**	1.07E-06	1.02E-06	5.78E-06	1.87E-06
**6h**	4.78E-07	1.28E-06	6.08E-06	4.62E-05
**12h**	1.00E-06	8.59E-07	2.87E-06	7.19E-06
**24h**	1.10E-06	1.48E-06	4.61E-06	6.31E-06
**36h**	9.04E-07	9.72E-07	3.09E-06	1.31E-06
**48h**	7.56E-07	8.65E-07	2.13E-06	2.83E-06
**72h**	2.48E-07	1.90E-07	3.47E-07	1.90E-07
	cgd1_560			
**2h**	2.04E-06	6.17E-08	0.00E+00	0.00E+00
**6h**	2.70E-06	4.02E-06	6.51E-05	2.04E-07
**12h**	2.11E-06	2.06E-07	1.96E-05	8.81E-06
**24h**	3.96E-06	2.17E-06	3.85E-05	1.60E-05
**36h**	2.80E-05	1.81E-05	4.96E-04	4.06E-05
**48h**	2.96E-05	5.65E-06	1.46E-04	4.39E-05
**72h**	2.06E-05	1.90E-05	4.16E-04	1.82E-05
	cgd1_570			
**2h**	0.00E+00	3.44E-07	0.00E+00	0.00E+00
**6h**	0.00E+00	3.74E-06	6.07E-05	1.26E-05
**12h**	1.38E-03	6.43E-06	6.27E-05	1.72E-05
**24h**	1.97E-05	2.51E-06	1.17E-04	1.72E-05
**36h**	1.09E-03	2.34E-05	1.39E-04	3.37E-05
**48h**	2.16E-03	4.89E-06	1.11E-04	7.93E-06
**72h**	0.00E+00	1.41E-05	3.48E-04	5.32E-06
	cgd1_580			
**2h**	2.54E-08	0.00E+00	2.62E-08	7.15E-08
**6h**	1.63E-06	4.28E-06	3.81E-06	3.68E-06
**12h**	6.32E-07	2.28E-06	4.84E-06	1.13E-06
**24h**	5.00E-07	2.33E-06	2.77E-06	9.83E-07
**36h**	4.50E-07	5.20E-06	1.08E-06	3.71E-06
**48h**	5.97E-07	1.15E-06	5.71E-06	1.49E-06
**72h**	3.04E-07	1.18E-06	5.41E-07	1.14E-06
	cgd1_590			
**2h**	5.18E-09	1.80E-08	1.18E-08	0.00E+00
**6h**	0.00E+00	0.00E+00	1.65E-08	0.00E+00
**12h**	1.35E-06	1.07E-05	3.62E-06	2.24E-05
**24h**	6.95E-07	2.92E-06	4.58E-06	1.34E-06

**36h**	8.52E-07	3.29E-05	2.60E-06	2.77E-05
**48h**	8.82E-07	5.96E-06	6.90E-06	3.47E-06
**72h**	1.62E-06	1.02E-05	9.43E-06	4.07E-06
	cgd1_610			
**2h**	2.41E-07	1.75E-06	9.32E-07	5.29E-06
**6h**	3.13E-07	1.16E-06	1.16E-06	8.15E-07
**12h**	2.02E-07	1.62E-06	3.17E-07	8.95E-06
**24h**	1.85E-07	7.21E-07	2.17E-06	4.61E-07
**36h**	1.29E-07	1.89E-06	4.81E-07	2.02E-06
**48h**	6.88E-08	6.86E-07	4.80E-07	4.51E-07
**72h**	8.90E-08	2.08E-07	4.83E-07	1.59E-07
	cgd1_620			
**2h**	0.00E+00	5.86E-06	8.21E-07	0.00E+00
**6h**	0.00E+00	0.00E+00	0.00E+00	5.62E-06
**12h**	3.37E-04	1.70E-03	2.50E-03	2.86E-03
**24h**	2.88E-05	4.81E-05	2.48E-04	2.77E-04
**36h**	1.32E-04	1.77E-03	4.64E-04	1.41E-03
**48h**	6.58E-05	3.18E-04	3.20E-04	4.55E-04
**72h**	1.14E-05	3.13E-05	6.37E-05	9.11E-06
	cgd1_650			
**2h**	0.00E+00	0.00E+00	0.00E+00	0.00E+00
**6h**	0.00E+00	0.00E+00	0.00E+00	0.00E+00
**12h**	7.34E-07	4.77E-06	4.67E-06	1.83E-05
**24h**	1.25E-07	3.22E-07	4.59E-07	4.58E-07
**36h**	3.54E-07	6.07E-06	7.62E-07	7.10E-06
**48h**	5.80E-08	3.07E-07	3.34E-07	7.35E-07
**72h**	1.02E-07	7.49E-08	9.00E-07	1.58E-07
	cgd1_660			
**2h**	1.72E-09	0.00E+00	0.00E+00	0.00E+00
**6h**	3.61E-09	3.11E-08	2.89E-08	0.00E+00
**12h**	5.98E-07	3.07E-07	4.48E-07	1.12E-07
**24h**	7.58E-08	2.05E-08	2.90E-07	1.06E-07
**36h**	7.83E-07	3.50E-06	2.66E-06	4.70E-06
**48h**	1.78E-05	2.90E-05	4.34E-05	5.92E-05
**72h**	1.02E-05	1.42E-05	4.79E-05	8.88E-06
	cgd1_670			
**2h**	1.09E-06	2.13E-06	4.13E-06	4.42E-06
**6h**	3.53E-07	1.22E-06	4.74E-07	5.84E-07
**12h**	2.56E-07	1.01E-06	9.50E-07	3.78E-06
**24h**	1.42E-07	1.06E-06	7.06E-07	7.17E-07
**36h**	3.89E-07	1.60E-06	1.05E-06	1.04E-06
**48h**	4.83E-07	2.47E-06	1.04E-06	2.94E-06
**72h**	3.28E-07	7.82E-07	2.79E-06	1.49E-07
	cgd1_680			
**2h**	9.76E-08	6.94E-07	1.10E-06	1.86E-06
**6h**	1.47E-09	5.21E-09	5.13E-09	1.15E-08
**12h**	1.85E-06	5.39E-06	7.56E-06	8.74E-06
**24h**	9.40E-08	7.31E-07	9.06E-07	9.21E-07
**36h**	2.39E-07	3.33E-06	7.92E-07	2.16E-06
**48h**	9.82E-08	1.39E-06	4.41E-07	1.95E-06
**72h**	4.35E-08	1.45E-07	3.44E-07	1.45E-07
	cgd1_690			
**2h**	0.00E+00	8.31E-08	8.02E-08	2.92E-07

**6h**	0.00E+00	0.00E+00	0.00E+00	0.00E+00
**12h**	2.26E-09	3.91E-07	1.15E-07	1.65E-06
**24h**	1.33E-07	4.39E-07	1.43E-06	4.87E-07
**36h**	8.87E-07	1.88E-06	1.81E-06	3.46E-06
**48h**	2.43E-06	1.38E-05	1.28E-05	3.22E-05
**72h**	1.46E-06	4.72E-06	1.17E-05	3.01E-06
	cgd1_700			
**2h**	2.64E-03	1.21E-02	1.50E-02	3.14E-03
**6h**	1.35E-03	6.87E-04	4.87E-03	6.58E-03
**12h**	3.40E-04	2.13E-03	1.01E-03	3.56E-03
**24h**	3.83E-04	3.22E-03	2.47E-03	5.61E-04
**36h**	4.78E-04	5.12E-03	3.40E-03	1.32E-03
**48h**	1.38E-04	3.71E-03	2.13E-03	1.08E-03
**72h**	4.18E-04	1.43E-03	1.56E-03	5.88E-04
	cgd1_720			
**2h**	0.00E+00	0.00E+00	0.00E+00	0.00E+00
**6h**	0.00E+00	0.00E+00	0.00E+00	0.00E+00
**12h**	0.00E+00	0.00E+00	0.00E+00	0.00E+00
**24h**	6.27E-10	1.92E-08	4.24E-09	1.74E-08
**36h**	1.60E-08	8.40E-08	5.58E-08	4.55E-08
**48h**	6.18E-07	2.65E-06	4.54E-06	4.07E-06
**72h**	2.67E-07	8.33E-07	1.45E-06	4.94E-07
	cgd1_730			
**2h**	0.00E+00	0.00E+00	0.00E+00	0.00E+00
**6h**	2.91E-08	8.01E-08	7.26E-08	5.95E-07
**12h**	2.63E-09	1.22E-08	2.94E-08	8.88E-09
**24h**	8.21E-09	5.41E-08	4.42E-08	1.16E-07
**36h**	7.18E-08	1.33E-07	2.62E-07	1.19E-07
**48h**	7.81E-07	1.72E-06	1.30E-06	1.59E-06
**72h**	2.90E-07	1.31E-06	8.29E-07	1.16E-06
	cgd2_440			
**2h**	9.62E-06	1.95E-06	1.84E-05	8.90E-06
**6h**	3.79E-06	3.95E-06	1.82E-05	1.40E-05
**12h**	7.89E-06	1.57E-05	3.83E-05	2.11E-05
**24h**	4.01E-06	4.45E-06	9.07E-05	2.70E-05
**36h**	3.88E-06	4.32E-06	3.07E-05	9.88E-06
**48h**	2.29E-06	7.55E-06	3.88E-05	4.11E-05
**72h**	1.08E-06	3.62E-06	5.74E-06	2.18E-06
	cgd2_470			
**2h**	5.31E-08	1.84E-07	6.75E-07	6.81E-08
**6h**	2.77E-06	5.46E-06	3.53E-05	9.87E-06
**12h**	1.34E-05	1.57E-05	5.40E-05	2.24E-05
**24h**	1.71E-06	4.38E-06	5.70E-05	5.53E-05
**36h**	6.26E-06	7.70E-06	3.69E-05	1.36E-05
**48h**	6.00E-06	7.81E-06	4.81E-05	5.36E-05
**72h**	3.57E-05	5.19E-05	1.77E-04	3.65E-05
	cgd2_500			
**2h**	0.00E+00	0.00E+00	2.74E-09	1.01E-09
**6h**	1.70E-09	5.54E-10	1.09E-08	1.82E-08
**12h**	0.00E+00	4.91E-10	3.25E-09	2.64E-08
**24h**	2.27E-09	1.56E-09	4.13E-09	2.32E-08
**36h**	9.48E-09	7.86E-09	5.74E-08	2.48E-08
**48h**	1.36E-08	1.09E-08	3.81E-08	5.94E-08

**72h**	1.04E-08	5.58E-05	1.32E-07	2.79E-08
	cgd2_510			
**2h**	0.00E+00	0.00E+00	3.24E-08	0.00E+00
**6h**	0.00E+00	4.39E-09	1.72E-08	0.00E+00
**12h**	0.00E+00	0.00E+00	1.63E-09	5.77E-09
**24h**	2.06E-09	8.78E-10	1.80E-08	2.57E-08
**36h**	1.52E-08	2.65E-08	2.70E-08	3.75E-08
**48h**	5.10E-08	4.17E-08	1.56E-07	2.00E-07
**72h**	1.68E-07	2.75E-07	1.16E-06	4.65E-07
	cgd2_520			
**2h**	0.00E+00	6.26E-07	0.00E+00	7.70E-08
**6h**	5.75E-08	2.37E-07	2.81E-07	6.31E-07
**12h**	1.03E-08	0.00E+00	4.87E-07	1.14E-07
**24h**	1.81E-08	1.20E-07	3.73E-07	2.58E-07
**36h**	7.30E-08	7.29E-07	1.61E-07	2.80E-07
**48h**	9.54E-08	2.19E-07	2.74E-07	1.14E-07
**72h**	2.90E-08	1.25E-07	6.92E-07	2.67E-07
	cgd2_550			
**2h**	0.00E+00	0.00E+00	2.77E-09	0.00E+00
**6h**	0.00E+00	0.00E+00	0.00E+00	2.36E-08
**12h**	6.49E-07	1.19E-07	4.93E-07	1.36E-06
**24h**	9.17E-08	4.38E-08	2.79E-07	5.43E-07
**36h**	9.25E-07	4.28E-07	6.26E-06	3.54E-06
**48h**	3.57E-07	2.49E-07	1.32E-06	1.52E-06
**72h**	1.91E-07	2.26E-07	9.08E-07	5.51E-07
	cgd2_560			
**2h**	2.77E-04	1.36E-05	0.00E+00	0.00E+00
**6h**	2.39E-04	8.37E-05	2.16E-03	2.78E-03
**12h**	2.46E-04	7.74E-05	3.73E-04	9.02E-04
**24h**	1.05E-04	1.05E-04	6.27E-04	8.35E-04
**36h**	1.57E-04	1.19E-04	4.40E-04	1.08E-03
**48h**	9.26E-05	1.49E-04	4.67E-04	2.03E-03
**72h**	1.95E-04	4.79E-05	3.59E-04	1.73E-04
	cgd2_570			
**2h**	1.14E-08	3.99E-09	7.31E-07	1.59E-08
**6h**	8.59E-08	1.64E-07	1.17E-06	1.61E-06
**12h**	7.47E-08	1.06E-07	4.19E-07	1.39E-06
**24h**	8.69E-08	1.94E-07	6.21E-07	1.49E-06
**36h**	4.16E-07	4.23E-07	1.10E-06	4.18E-06
**48h**	4.56E-07	3.18E-07	1.13E-06	4.54E-06
**72h**	2.29E-07	4.83E-07	2.02E-06	9.23E-07
	cgd2_580			
**2h**	1.52E-09	0.00E+00	0.00E+00	0.00E+00
**6h**	8.12E-07	9.58E-07	4.75E-06	2.98E-06
**12h**	2.08E-07	3.78E-08	4.11E-07	7.97E-07
**24h**	3.91E-07	1.06E-07	2.70E-06	1.31E-05
**36h**	9.88E-07	1.10E-06	2.74E-06	7.70E-06
**48h**	8.01E-07	6.19E-07	2.38E-06	3.09E-06
**72h**	1.73E-08	9.29E-08	1.95E-07	7.87E-08
	cgd2_590			
**2h**	2.01E-09	0.00E+00	0.00E+00	0.00E+00
**6h**	1.07E-07	1.09E-06	3.60E-07	5.62E-07
**12h**	4.77E-06	3.64E-07	1.23E-05	7.24E-06

**24h**	1.43E-06	1.27E-06	6.68E-06	5.92E-06
**36h**	3.93E-06	2.54E-06	2.59E-05	9.02E-06
**48h**	1.73E-05	1.53E-05	2.26E-04	7.45E-05
**72h**	2.06E-05	2.36E-05	1.08E-04	2.04E-04
	cgd2_600			
**2h**	1.61E-06	3.19E-07	9.46E-07	1.64E-06
**6h**	3.79E-06	2.08E-06	3.98E-05	1.97E-05
**12h**	1.77E-07	1.35E-07	2.10E-06	2.14E-05
**24h**	7.81E-07	7.36E-07	9.81E-06	3.07E-05
**36h**	1.44E-06	2.20E-06	2.82E-06	4.41E-06
**48h**	9.03E-07	7.31E-07	1.20E-06	5.81E-06
**72h**	6.64E-08	1.57E-07	7.01E-07	9.52E-07
	cgd2_630			
**2h**	0.00E+00	0.00E+00	0.00E+00	0.00E+00
**6h**	0.00E+00	0.00E+00	0.00E+00	0.00E+00
**12h**	2.45E-07	2.70E-07	4.53E-07	3.48E-07
**24h**	4.04E-08	2.88E-08	4.82E-08	1.77E-07
**36h**	1.43E-07	3.51E-07	1.77E-07	3.13E-07
**48h**	5.12E-08	1.62E-07	5.93E-08	6.48E-07
**72h**	1.52E-07	1.39E-07	5.15E-07	1.22E-07
	cgd2_660			
**2h**	0.00E+00	0.00E+00	0.00E+00	1.47E-07
**6h**	0.00E+00	0.00E+00	0.00E+00	0.00E+00
**12h**	1.36E-05	2.31E-05	9.58E-05	9.67E-05
**24h**	8.37E-05	4.71E-06	1.97E-05	4.95E-06
**36h**	2.35E-05	1.80E-05	2.22E-04	5.81E-05
**48h**	9.93E-06	2.85E-05	6.74E-05	2.74E-05
**72h**	9.11E-06	6.51E-06	1.95E-05	3.19E-06
	cgd2_670			
**2h**	0.00E+00	1.12E-08	0.00E+00	0.00E+00
**6h**	3.37E-07	3.68E-08	1.84E-08	0.00E+00
**12h**	8.98E-07	5.70E-07	7.15E-06	3.25E-06
**24h**	1.19E-06	7.12E-07	1.42E-06	7.17E-07
**36h**	2.16E-06	1.09E-06	1.52E-05	2.85E-06
**48h**	2.07E-06	3.00E-06	1.24E-05	6.08E-06
**72h**	2.02E-06	2.08E-06	3.48E-06	1.53E-06
	cgd2_680			
**2h**	2.83E-05	7.52E-07	3.31E-05	4.62E-06
**6h**	4.95E-05	2.06E-05	1.11E-04	6.92E-05
**12h**	2.65E-07	7.52E-07	3.78E-05	2.10E-05
**24h**	3.36E-06	5.25E-06	5.05E-05	3.14E-05
**36h**	5.96E-06	3.40E-06	3.96E-05	8.28E-06
**48h**	1.31E-06	2.58E-06	1.65E-05	4.54E-06
**72h**	3.59E-07	7.25E-07	2.65E-06	8.58E-07
	cgd2_690			
**2h**	1.04E-05	1.64E-05	4.09E-05	9.86E-06
**6h**	3.37E-06	8.82E-06	1.07E-05	5.92E-06
**12h**	3.78E-06	1.62E-06	3.35E-05	5.24E-05
**24h**	3.61E-06	4.80E-06	9.17E-06	8.81E-06
**36h**	2.41E-06	1.60E-06	7.47E-06	4.04E-06
**48h**	1.34E-07	2.43E-07	7.54E-07	5.16E-07
**72h**	1.84E-07	1.82E-07	4.58E-07	3.18E-07
	cgd2_700			

**2h**	0.00E+00	9.35E-09	0.00E+00	0.00E+00
**6h**	1.28E-07	7.27E-08	2.66E-07	8.78E-07
**12h**	4.13E-07	1.53E-06	6.48E-07	1.79E-06
**24h**	1.21E-07	1.84E-07	2.05E-06	1.31E-06
**36h**	5.57E-07	5.46E-07	7.49E-06	3.88E-06
**48h**	2.06E-07	1.74E-07	1.98E-06	1.17E-06
**72h**	1.12E-06	5.92E-07	8.49E-07	1.11E-06
	cgd2_710			
**2h**	0.00E+00	1.38E-06	0.00E+00	0.00E+00
**6h**	0.00E+00	1.21E-07	0.00E+00	2.90E-06
**12h**	3.50E-06	8.12E-06	9.31E-06	2.82E-05
**24h**	1.85E-06	1.76E-06	6.03E-06	3.00E-06
**36h**	1.01E-05	3.89E-06	2.69E-05	1.74E-05
**48h**	3.55E-05	5.40E-05	4.30E-05	1.44E-05
**72h**	8.88E-06	1.13E-05	2.78E-05	1.60E-05
	cgd2_780			
**2h**	0.00E+00	0.00E+00	0.00E+00	0.00E+00
**6h**	0.00E+00	0.00E+00	0.00E+00	0.00E+00
**12h**	1.99E-04	2.43E-04	1.56E-03	1.26E-03
**24h**	2.10E-04	1.30E-04	1.43E-04	1.38E-04
**36h**	4.18E-05	5.80E-05	1.32E-03	3.06E-04
**48h**	5.25E-05	6.25E-05	2.68E-04	1.36E-04
**72h**	4.72E-05	4.57E-05	5.15E-04	5.01E-05
	cgd2_790			
**2h**	0.00E+00	4.54E-09	0.00E+00	0.00E+00
**6h**	0.00E+00	0.00E+00	0.00E+00	0.00E+00
**12h**	0.00E+00	0.00E+00	0.00E+00	4.01E-10
**24h**	6.00E-09	2.34E-09	1.61E-08	8.73E-09
**36h**	5.48E-09	1.62E-08	6.78E-08	1.25E-07
**48h**	2.10E-03	3.03E-06	2.54E-05	3.43E-05
**72h**	2.23E-06	3.61E-06	6.36E-06	7.11E-06
	cgd2_800			
**2h**	0.00E+00	6.96E-08	0.00E+00	0.00E+00
**6h**	0.00E+00	0.00E+00	0.00E+00	0.00E+00
**12h**	1.21E-07	3.57E-07	1.20E-06	0.00E+00
**24h**	1.09E-07	6.96E-08	2.26E-07	4.41E-07
**36h**	5.47E-07	5.55E-07	2.16E-06	3.54E-06
**48h**	1.52E-07	2.01E-07	4.07E-07	6.88E-07
**72h**	1.00E-07	7.43E-08	3.42E-07	2.26E-07
	cgd2_850			
**2h**	3.90E-08	0.00E+00	1.29E-08	5.36E-08
**6h**	0.00E+00	2.30E-08	0.00E+00	0.00E+00
**12h**	1.33E-09	1.11E-06	8.77E-09	3.64E-09
**24h**	1.89E-08	6.07E-09	2.26E-09	2.95E-08
**36h**	2.10E-08	2.04E-08	3.39E-08	2.67E-07
**48h**	2.01E-06	1.57E-06	9.56E-07	2.65E-06
**72h**	4.95E-07	3.84E-07	8.44E-07	5.32E-07
	cgd2_860			
**2h**	1.66E-08	0.00E+00	0.00E+00	0.00E+00
**6h**	7.33E-06	2.66E-06	5.63E-05	2.92E-05
**12h**	2.49E-07	1.18E-06	1.35E-06	2.78E-06
**24h**	6.39E-07	3.46E-07	1.54E-05	2.36E-05
**36h**	7.14E-06	4.05E-06	1.62E-05	9.38E-06

**48h**	8.94E-06	3.22E-06	4.23E-06	6.08E-06
**72h**	1.65E-07	1.87E-07	9.39E-07	1.37E-06
	cgd2_870			
**2h**	1.24E-08	9.17E-08	1.15E-06	4.85E-07
**6h**	0.00E+00	1.54E-07	3.46E-06	1.47E-06
**12h**	8.34E-05	2.76E-06	3.73E-06	1.79E-06
**24h**	3.51E-07	6.92E-07	1.60E-06	3.20E-06
**36h**	2.04E-06	4.85E-07	2.38E-06	1.90E-06
**48h**	5.47E-06	1.09E-07	6.75E-07	6.86E-07
**72h**	9.05E-07	7.57E-08	2.46E-06	1.34E-07
	cgd1_60			
**2h**	2.49E-08	0.00E+00	0.00E+00	0.00E+00
**6h**	0.00E+00	0.00E+00	2.38E-08	4.68E-10
**12h**	0.00E+00	1.98E-08	3.87E-09	4.54E-09
**24h**	8.87E-10	1.06E-10	3.79E-09	1.03E-08
**36h**	7.64E-07	1.52E-09	1.84E-08	3.76E-08
**48h**	2.56E-08	8.49E-08	4.45E-07	5.32E-06
**72h**	3.17E-08	8.75E-08	5.25E-08	5.24E-08
	cgd1_70			
**2h**	0.00E+00	0.00E+00	0.00E+00	0.00E+00
**6h**	0.00E+00	0.00E+00	1.16E-07	1.23E-08
**12h**	6.56E-09	0.00E+00	0.00E+00	4.42E-08
**24h**	0.00E+00	1.73E-09	5.82E-09	1.17E-07
**36h**	8.01E-09	1.38E-10	1.63E-08	4.60E-08
**48h**	1.76E-09	2.20E-10	1.84E-08	1.92E-08
**72h**	2.65E-09	8.24E-09	1.40E-09	0.00E+00
	cgd1_80			
**2h**	8.44E-08	0.00E+00	4.68E-05	6.08E-06
**6h**	6.52E-05	1.23E-04	8.42E-05	8.71E-06
**12h**	1.77E-04	2.77E-05	3.09E-04	1.55E-04
**24h**	1.02E-04	1.95E-05	2.30E-04	1.83E-04
**36h**	2.39E-05	4.91E-06	1.20E-04	1.20E-04
**48h**	1.53E-05	5.18E-06	1.20E-04	8.43E-05
**72h**	1.59E-06	1.14E-06	1.51E-06	4.39E-06
	cgd1_90			
**2h**	2.33E-07	6.26E-08	5.77E-07	1.92E-08
**6h**	1.15E-07	1.57E-07	7.66E-07	2.42E-07
**12h**	2.56E-08	3.19E-08	1.61E-07	2.07E-07
**24h**	1.59E-08	1.70E-08	4.16E-07	1.30E-06
**36h**	1.97E-07	5.27E-08	1.31E-06	3.36E-07
**48h**	1.92E-08	2.04E-08	2.06E-07	3.12E-07
**72h**	3.44E-08	6.72E-08	5.84E-08	1.79E-07
	cgd1_100			
**2h**	5.00E-05	0.00E+00	0.00E+00	0.00E+00
**6h**	1.02E-04	3.79E-06	9.18E-05	2.81E-05
**12h**	1.85E-05	0.00E+00	6.44E-05	8.68E-05
**24h**	6.65E-05	1.12E-05	3.69E-04	9.07E-04
**36h**	1.23E-04	1.85E-05	8.71E-04	5.75E-04
**48h**	3.53E-05	1.22E-05	5.07E-04	3.04E-04
**72h**	2.27E-05	8.07E-06	7.84E-05	5.03E-07
	cgd1_110			
**2h**	2.68E-06	6.02E-07	1.42E-06	3.33E-06
**6h**	1.33E-07	0.00E+00	4.00E-06	5.03E-08

**12h**	9.04E-05	1.72E-05	2.08E-04	1.95E-04
**24h**	8.06E-06	1.09E-06	3.71E-06	4.52E-06
**36h**	2.04E-06	5.11E-07	1.07E-05	1.30E-05
**48h**	4.64E-07	3.42E-07	7.22E-06	1.87E-06
**72h**	4.54E-07	2.04E-07	7.08E-07	9.92E-08
	cgd1_120			
**2h**	0.00E+00	0.00E+00	0.00E+00	1.16E-08
**6h**	0.00E+00	0.00E+00	0.00E+00	0.00E+00
**12h**	4.94E-07	5.99E-07	1.28E-06	1.10E-06
**24h**	4.28E-08	4.59E-08	6.77E-08	2.41E-08
**36h**	7.71E-09	2.29E-08	2.97E-07	1.82E-03
**48h**	3.96E-09	1.25E-08	3.87E-08	3.45E-08
**72h**	6.03E-09	4.54E-09	8.06E-09	4.87E-08
	cgd1_130			
**2h**	1.41E-04	5.60E-04	1.82E-07	0.00E+00
**6h**	5.46E-06	8.21E-06	2.90E-04	1.08E-05
**12h**	1.43E-03	8.13E-03	1.48E-03	9.41E-03
**24h**	2.17E-04	1.98E-04	2.45E-04	2.09E-04
**36h**	1.45E-04	2.01E-04	4.71E-04	1.32E-03
**48h**	1.05E-05	6.94E-05	4.05E-04	3.31E-04
**72h**	6.83E-05	1.62E-04	3.21E-04	3.02E-04
	cgd1_140			
**2h**	0.00E+00	2.42E-06	4.35E-08	1.07E-07
**6h**	0.00E+00	1.44E-08	0.00E+00	0.00E+00
**12h**	2.01E-06	1.32E-06	5.43E-06	9.81E-06
**24h**	7.91E-08	1.53E-07	4.34E-07	2.16E-07
**36h**	5.06E-08	4.08E-08	6.25E-07	2.11E-06
**48h**	1.80E-08	3.08E-08	2.55E-08	3.48E-07
**72h**	4.37E-09	5.81E-09	7.73E-08	1.31E-07
	cgd1_150			
**2h**	8.05E-06	9.28E-07	3.68E-06	2.99E-06
**6h**	0.00E+00	0.00E+00	0.00E+00	0.00E+00
**12h**	8.09E-07	2.92E-07	1.11E-06	5.14E-06
**24h**	8.43E-07	3.26E-07	5.08E-07	1.30E-06
**36h**	4.28E-07	4.47E-08	2.85E-07	7.89E-07
**48h**	9.86E-08	5.48E-08	1.70E-07	5.44E-07
**72h**	2.86E-08	1.84E-08	1.93E-08	2.07E-07
	cgd1_180			
**2h**	0.00E+00	0.00E+00	0.00E+00	0.00E+00
**6h**	0.00E+00	2.16E-07	8.71E-04	0.00E+00
**12h**	1.72E-06	8.73E-07	1.82E-06	9.31E-06
**24h**	3.14E-07	7.53E-08	3.41E-07	1.85E-06
**36h**	8.52E-08	1.37E-07	9.98E-07	3.84E-06
**48h**	5.39E-08	9.95E-08	2.18E-07	2.21E-06
**72h**	5.18E-09	1.43E-08	2.56E-07	1.80E-07
	cgd1_190			
**2h**	0.00E+00	2.62E-06	0.00E+00	0.00E+00
**6h**	4.86E-06	3.29E-06	5.92E-06	3.34E-07
**12h**	1.13E-03	2.79E-04	2.70E-04	2.22E-04
**24h**	3.39E-04	1.78E-05	3.29E-05	9.03E-05
**36h**	1.10E-04	2.50E-05	4.74E-04	8.29E-04
**48h**	1.92E-05	5.29E-06	1.01E-04	8.34E-05
**72h**	1.17E-04	3.00E-05	3.56E-04	4.06E-05

	cgd1_740			
**2h**	2.75E-08	0.00E+00	0.00E+00	0.00E+00
**6h**	2.57E-08	0.00E+00	0.00E+00	9.97E-06
**12h**	4.35E-08	8.94E-07	3.02E-06	2.91E-06
**24h**	1.19E-07	3.19E-06	4.15E-07	5.88E-05
**36h**	6.47E-07	4.80E-06	4.57E-06	3.92E-05
**48h**	2.94E-06	4.43E-05	1.03E-05	1.27E-04
**72h**	1.89E-06	2.12E-05	1.00E-05	6.12E-05
	cgd1_750			
**2h**	0.00E+00	0.00E+00	0.00E+00	0.00E+00
**6h**	4.74E-09	0.00E+00	0.00E+00	0.00E+00
**12h**	2.25E-08	5.95E-07	2.22E-07	4.48E-07
**24h**	1.63E-07	1.65E-06	1.33E-06	1.39E-06
**36h**	2.68E-07	3.08E-06	2.78E-06	3.23E-06
**48h**	3.78E-07	3.55E-06	1.41E-06	2.64E-06
**72h**	1.73E-07	3.20E-07	9.79E-07	3.78E-07
	cgd1_760			
**2h**	4.29E-07	2.89E-07	3.38E-06	1.57E-07
**6h**	7.25E-08	3.28E-06	2.88E-06	6.06E-05
**12h**	6.07E-06	1.38E-05	6.18E-05	1.46E-04
**24h**	3.03E-06	3.28E-05	2.33E-05	1.72E-04
**36h**	2.05E-06	4.56E-05	1.52E-05	3.22E-04
**48h**	2.74E-06	4.18E-05	1.90E-05	4.49E-04
**72h**	5.71E-06	3.13E-05	2.29E-05	2.64E-04
	cgd1_770			
**2h**	0.00E+00	1.83E-08	0.00E+00	0.00E+00
**6h**	3.03E-07	8.34E-07	5.14E-07	1.22E-05
**12h**	3.50E-07	4.45E-07	4.07E-07	6.72E-07
**24h**	1.29E-07	3.02E-06	6.09E-07	3.01E-05
**36h**	6.26E-07	5.26E-06	2.79E-06	3.11E-05
**48h**	2.27E-07	3.24E-06	3.34E-06	4.00E-05
**72h**	4.10E-07	1.68E-06	4.04E-06	1.37E-05
	cgd1_790			
**2h**	0.00E+00	0.00E+00	0.00E+00	0.00E+00
**6h**	0.00E+00	0.00E+00	0.00E+00	0.00E+00
**12h**	6.64E-08	2.88E-06	1.69E-06	2.94E-07
**24h**	6.39E-08	2.45E-07	7.51E-07	1.42E-06
**36h**	6.13E-08	2.48E-06	6.29E-07	4.93E-06
**48h**	1.05E-08	2.36E-07	2.60E-07	3.61E-07
**72h**	4.25E-07	2.42E-06	3.43E-06	3.00E-06
	cgd1_800			
**2h**	1.47E-08	0.00E+00	0.00E+00	3.88E-07
**6h**	1.32E-07	0.00E+00	1.27E-07	1.77E-07
**12h**	9.82E-08	4.71E-07	1.24E-06	3.75E-06
**24h**	2.03E-07	1.21E-06	8.94E-07	4.08E-05
**36h**	6.38E-07	1.33E-05	3.33E-06	1.40E-04
**48h**	1.15E-06	1.46E-05	4.00E-06	1.32E-04
**72h**	2.66E-06	1.07E-05	6.82E-06	2.11E-04
	cgd1_810			
**2h**	0.00E+00	0.00E+00	4.50E-09	0.00E+00
**6h**	0.00E+00	0.00E+00	0.00E+00	0.00E+00
**12h**	2.09E-08	1.67E-08	4.57E-09	1.13E-07
**24h**	9.34E-09	3.65E-08	8.14E-08	1.33E-07

**36h**	1.36E-08	5.95E-07	1.64E-07	1.91E-07
**48h**	6.21E-08	4.68E-07	2.36E-07	1.11E-06
**72h**	2.14E-07	2.80E-06	5.49E-07	2.69E-06
	cgd1_820			
**2h**	4.53E-08	5.56E-08	2.46E-07	0.00E+00
**6h**	0.00E+00	8.14E-08	0.00E+00	0.00E+00
**12h**	2.42E-07	2.03E-06	1.90E-06	3.91E-06
**24h**	1.20E-07	1.47E-06	6.91E-07	3.52E-06
**36h**	1.09E-06	1.50E-05	3.77E-06	2.10E-05
**48h**	9.29E-07	6.65E-06	4.44E-06	1.90E-05
**72h**	2.16E-06	1.49E-05	2.40E-06	1.34E-05
	cgd1_830			
**2h**	3.87E-07	6.94E-06	6.21E-06	7.50E-06
**6h**	6.68E-07	1.49E-05	9.30E-06	4.90E-06
**12h**	1.72E-06	1.32E-05	5.45E-06	2.23E-05
**24h**	7.63E-07	1.05E-05	9.90E-06	5.03E-05
**36h**	9.80E-07	2.71E-05	5.29E-06	1.00E-04
**48h**	1.42E-06	1.46E-05	8.07E-06	4.50E-05
**72h**	7.28E-07	4.52E-05	4.28E-06	3.12E-05
	cgd1_860			
**2h**	9.84E-08	2.02E-07	1.07E-06	1.41E-06
**6h**	3.00E-08	6.26E-07	2.30E-07	1.35E-08
**12h**	4.90E-09	1.33E-07	1.16E-07	6.30E-07
**24h**	3.66E-08	3.91E-07	2.58E-07	2.30E-07
**36h**	1.26E-07	7.62E-07	2.31E-07	2.37E-06
**48h**	3.13E-07	9.51E-07	6.45E-07	9.02E-05
**72h**	8.48E-08	6.87E-07	6.23E-08	5.72E-07
	cgd1_890			
**2h**	1.62E-05	3.14E-05	4.05E-05	3.03E-04
**6h**	3.39E-06	1.67E-05	3.08E-05	1.41E-05
**12h**	1.99E-06	1.72E-05	1.07E-05	7.40E-05
**24h**	5.04E-06	3.36E-05	3.67E-05	8.64E-05
**36h**	3.57E-06	5.48E-05	1.30E-05	1.43E-04
**48h**	6.61E-06	4.20E-05	1.72E-05	5.02E-04
**72h**	1.25E-06	1.85E-05	3.84E-06	4.61E-05
	cgd1_940			
**2h**	0.00E+00	0.00E+00	0.00E+00	0.00E+00
**6h**	0.00E+00	0.00E+00	0.00E+00	0.00E+00
**12h**	3.04E-07	8.52E-07	3.28E-06	1.05E-05
**24h**	4.83E-07	5.25E-06	3.56E-06	1.53E-05
**36h**	1.34E-06	1.87E-05	1.37E-05	2.71E-04
**48h**	2.29E-06	6.13E-06	3.55E-06	4.68E-05
**72h**	2.00E-06	2.34E-06	3.72E-06	5.67E-05
	cgd2_880			
**2h**	0.00E+00	0.00E+00	9.34E-07	0.00E+00
**6h**	2.02E-05	2.06E-05	1.64E-04	1.35E-04
**12h**	1.34E-06	2.65E-07	8.96E-06	5.57E-06
**24h**	5.12E-06	3.84E-06	8.06E-05	5.71E-05
**36h**	9.56E-06	2.79E-06	2.90E-05	9.21E-06
**48h**	8.96E-06	2.62E-06	1.12E-05	1.41E-05
**72h**	8.54E-07	1.76E-06	4.90E-06	6.77E-07
	cgd2_890			
**2h**	1.01E-08	0.00E+00	3.72E-08	0.00E+00

**6h**	2.32E-06	5.61E-07	2.44E-06	3.08E-06
**12h**	5.67E-08	2.38E-09	1.22E-07	9.51E-08
**24h**	3.47E-07	7.93E-08	2.54E-06	2.98E-06
**36h**	6.69E-07	3.12E-07	7.76E-07	2.94E-07
**48h**	8.29E-07	1.43E-07	7.00E-07	1.17E-06
**72h**	6.61E-07	1.98E-07	6.81E-07	1.83E-07
	cgd2_920			
**2h**	0.00E+00	0.00E+00	0.00E+00	0.00E+00
**6h**	0.00E+00	0.00E+00	0.00E+00	0.00E+00
**12h**	1.05E-06	3.59E-07	5.45E-06	1.85E-05
**24h**	5.09E-07	4.60E-07	1.51E-06	2.63E-06
**36h**	1.60E-06	9.55E-07	1.44E-05	1.09E-05
**48h**	9.75E-07	1.67E-07	2.59E-06	4.07E-06
**72h**	2.10E-06	3.43E-07	4.33E-06	2.17E-06
	cgd2_930			
**2h**	2.11E-09	0.00E+00	1.24E-08	0.00E+00
**6h**	3.58E-07	1.23E-07	1.25E-06	2.42E-06
**12h**	4.61E-08	6.81E-09	3.66E-07	1.62E-07
**24h**	8.17E-08	4.45E-08	3.65E-07	1.14E-06
**36h**	2.94E-07	3.31E-07	1.48E-06	1.09E-06
**48h**	1.96E-07	9.43E-08	2.08E-07	5.62E-07
**72h**	1.33E-07	7.46E-08	4.12E-07	4.90E-07
	cgd2_940			
**2h**	2.62E-03	1.17E-04	1.07E-02	3.60E-03
**6h**	4.08E-04	1.20E-04	1.73E-03	5.81E-04
**12h**	6.45E-04	1.61E-04	6.86E-03	5.45E-03
**24h**	8.57E-04	2.39E-04	1.80E-03	1.61E-03
**36h**	1.05E-03	3.04E-04	2.75E-03	1.18E-03
**48h**	5.48E-04	6.34E-05	4.99E-04	3.82E-04
**72h**	5.38E-04	6.80E-05	3.91E-04	2.56E-04
	cgd2_950			
**2h**	5.25E-06	2.71E-07	6.33E-05	3.92E-06
**6h**	3.07E-06	2.15E-06	3.94E-05	9.22E-06
**12h**	3.54E-07	6.67E-09	4.27E-06	4.52E-06
**24h**	5.39E-07	1.48E-07	3.87E-06	4.38E-06
**36h**	7.24E-07	8.95E-07	3.32E-06	2.39E-06
**48h**	4.20E-07	4.32E-07	2.17E-06	1.42E-06
**72h**	6.18E-07	2.72E-07	5.09E-07	8.11E-07
	cgd2_960			
**2h**	2.24E-06	9.10E-08	4.85E-06	1.17E-06
**6h**	1.88E-07	1.02E-06	2.88E-06	4.48E-06
**12h**	3.46E-07	1.58E-07	2.56E-06	2.34E-06
**24h**	3.20E-07	2.02E-07	2.08E-06	3.06E-06
**36h**	1.04E-06	1.04E-06	4.62E-06	2.67E-06
**48h**	8.15E-07	3.11E-07	1.45E-06	1.24E-05
**72h**	7.26E-07	2.16E-07	1.34E-06	5.52E-07
	cgd2_970			
**2h**	1.53E-08	5.68E-10	2.71E-08	1.13E-08
**6h**	7.70E-09	2.09E-08	6.54E-08	3.92E-08
**12h**	1.87E-09	0.00E+00	2.46E-08	3.18E-09
**24h**	1.46E-08	1.23E-08	1.17E-07	1.18E-07
**36h**	9.86E-08	1.12E-07	1.73E-07	3.24E-07
**48h**	5.33E-08	9.25E-08	3.87E-07	3.21E-07

**72h**	8.13E-08	1.99E-08	3.71E-08	1.13E-07
	cgd2_1020			
**2h**	2.55E-08	3.61E-09	1.29E-08	7.14E-08
**6h**	4.75E-07	2.75E-07	7.18E-07	1.23E-06
**12h**	7.06E-08	7.17E-11	5.09E-08	5.52E-08
**24h**	1.90E-08	8.88E-08	1.11E-06	9.06E-07
**36h**	1.04E-06	6.28E-07	6.56E-07	9.63E-07
**48h**	5.50E-07	5.82E-07	6.73E-07	1.18E-06
**72h**	1.10E-07	5.29E-08	2.03E-07	2.55E-07
	cgd1_10			
**2h**	4.28E-06	4.50E-07	2.85E-06	3.29E-05
**6h**	3.81E-06	2.95E-05	2.89E-05	2.88E-05
**12h**	1.53E-06	1.92E-07	1.29E-05	2.89E-06
**24h**	7.61E-06	1.09E-05	4.03E-05	3.55E-05
**36h**	1.68E-05	2.13E-05	8.89E-06	2.77E-05
**48h**	2.17E-05	9.07E-06	9.29E-06	4.96E-05
**72h**	2.88E-06	1.88E-06	2.78E-06	7.45E-06
	cgd1_20			
**2h**	1.08E-07	5.56E-08	8.21E-07	1.20E-06
**6h**	3.11E-07	4.39E-07	7.15E-07	4.28E-07
**12h**	1.68E-07	7.32E-09	6.63E-07	1.29E-06
**24h**	1.09E-07	1.63E-07	2.97E-07	6.78E-07
**36h**	4.04E-07	1.67E-07	4.40E-07	8.91E-07
**48h**	3.51E-07	2.39E-07	2.51E-07	7.25E-07
**72h**	2.58E-07	2.59E-08	7.79E-08	3.21E-08
	cgd1_30			
**2h**	2.04E-06	0.00E+00	0.00E+00	0.00E+00
**6h**	0.00E+00	0.00E+00	2.07E-06	2.84E-07
**12h**	2.18E-05	3.11E-05	5.71E-04	4.16E-05
**24h**	8.11E-06	2.15E-05	2.57E-05	5.47E-05
**36h**	7.82E-05	1.21E-04	4.76E-04	2.86E-04
**48h**	5.07E-05	1.28E-04	1.17E-04	1.96E-04
**72h**	8.34E-05	1.47E-04	2.25E-04	3.04E-05
	cgd5_170			
**2h**	7.67E-06	1.93E-05	3.16E-05	1.75E-06
**6h**	3.13E-05	9.46E-05	2.96E-05	4.66E-05
**12h**	6.96E-05	4.81E-04	9.01E-05	2.28E-04
**24h**	6.68E-05	1.45E-04	1.08E-04	6.76E-05
**36h**	3.64E-05	3.24E-04	7.03E-05	7.51E-05
**48h**	4.50E-05	7.54E-05	1.30E-04	3.73E-05
**72h**	5.49E-06	7.73E-06	9.07E-06	5.69E-06
	cgd5_210			
**2h**	6.72E-08	9.19E-08	1.71E-07	1.64E-08
**6h**	6.77E-07	2.44E-06	4.24E-06	2.13E-06
**12h**	6.42E-09	1.57E-07	5.99E-08	1.33E-07
**24h**	5.57E-08	8.26E-07	9.98E-07	6.81E-07
**36h**	1.15E-07	6.95E-07	2.56E-07	3.43E-07
**48h**	4.66E-08	4.82E-07	6.58E-07	1.14E-07
**72h**	5.01E-08	7.20E-08	6.63E-08	2.00E-08
	cgd5_220			
**2h**	6.34E-05	7.50E-04	2.20E-03	3.01E-04
**6h**	9.96E-04	8.23E-03	3.04E-03	2.62E-02
**12h**	9.03E-06	9.06E-04	2.63E-04	5.69E-03

**24h**	7.79E-04	1.06E-03	2.38E-03	7.38E-04
**36h**	8.25E-04	3.38E-03	5.35E-03	2.06E-03
**48h**	7.01E-05	4.74E-04	3.61E-03	1.71E-03
**72h**	2.45E-04	4.36E-04	1.27E-03	1.89E-04
	cgd5_230			
**2h**	0.00E+00	0.00E+00	1.15E-08	0.00E+00
**6h**	0.00E+00	0.00E+00	0.00E+00	3.94E-08
**12h**	3.08E-08	3.25E-09	3.95E-09	4.33E-08
**24h**	6.40E-09	3.32E-09	1.85E-09	4.57E-09
**36h**	1.01E-08	1.60E-08	2.26E-08	2.95E-08
**48h**	7.26E-09	1.24E-08	1.71E-08	1.25E-08
**72h**	5.16E-09	1.55E-08	7.69E-09	5.55E-09
	cgd5_240			
**2h**	6.79E-07	6.93E-06	1.29E-06	7.81E-06
**6h**	1.14E-07	5.60E-07	2.33E-07	2.87E-07
**12h**	8.72E-08	3.85E-06	6.28E-07	2.46E-06
**24h**	3.84E-07	1.15E-06	1.34E-06	9.55E-07
**36h**	7.80E-07	3.17E-06	1.72E-06	1.27E-06
**48h**	5.23E-07	2.34E-06	2.36E-06	1.25E-06
**72h**	6.50E-07	2.84E-07	7.13E-07	3.39E-07
	cgd5_300			
**2h**	1.96E-07	4.04E-07	7.76E-09	0.00E+00
**6h**	1.19E-09	1.06E-07	7.96E-07	4.68E-08
**12h**	2.31E-07	5.68E-06	5.10E-06	7.23E-06
**24h**	2.27E-07	3.48E-07	5.47E-07	6.25E-07
**36h**	6.17E-07	3.34E-06	9.07E-07	1.73E-06
**48h**	1.09E-06	8.21E-07	7.23E-07	4.44E-07
**72h**	6.05E-07	5.52E-08	8.33E-07	2.74E-07
	cgd5_310			
**2h**	6.01E-07	1.27E-06	1.98E-07	6.74E-07
**6h**	4.16E-08	7.78E-07	1.89E-07	1.41E-07
**12h**	3.77E-07	3.08E-06	7.77E-07	4.81E-06
**24h**	9.87E-08	3.73E-07	2.88E-07	2.38E-07
**36h**	1.08E-07	1.20E-06	2.23E-07	4.40E-07
**48h**	1.85E-07	4.80E-07	1.98E-07	3.00E-07
**72h**	2.72E-08	1.09E-08	8.14E-08	3.27E-08
	cgd5_320			
**2h**	5.47E-07	7.33E-07	4.44E-07	3.65E-07
**6h**	4.25E-08	3.97E-07	1.43E-07	4.01E-07
**12h**	2.78E-07	3.85E-06	1.22E-06	4.43E-06
**24h**	8.85E-08	1.98E-07	5.18E-07	3.26E-07
**36h**	1.33E-07	8.33E-07	3.60E-07	3.17E-07
**48h**	9.67E-08	6.90E-07	4.03E-07	4.45E-07
**72h**	3.40E-08	3.11E-08	3.92E-07	2.79E-08
	cgd2_30			
**2h**	7.51E-07	5.86E-09	6.88E-07	1.65E-07
**6h**	3.94E-06	2.83E-05	3.40E-06	3.21E-05
**12h**	4.70E-07	1.15E-06	2.18E-06	2.59E-06
**24h**	2.44E-06	1.85E-05	9.75E-07	3.69E-05
**36h**	1.26E-05	5.71E-05	2.51E-05	1.66E-05
**48h**	1.67E-05	4.83E-05	3.91E-05	2.11E-05
**72h**	4.88E-06	1.51E-05	2.56E-05	1.17E-05
	cgd2_60			

**2h**	2.42E-06	7.84E-07	3.86E-07	1.15E-06
**6h**	1.14E-06	9.47E-06	8.86E-07	9.55E-06
**12h**	1.96E-06	6.88E-06	5.04E-06	1.04E-05
**24h**	9.51E-07	8.82E-06	1.43E-06	1.20E-05
**36h**	7.83E-07	8.43E-06	2.34E-06	3.26E-06
**48h**	2.52E-06	1.59E-05	1.27E-05	1.17E-05
**72h**	2.17E-06	3.53E-06	1.68E-05	2.41E-06
	cgd2_80			
**2h**	1.11E-07	9.16E-07	5.62E-07	7.35E-07
**6h**	4.66E-07	1.60E-06	1.29E-07	4.96E-07
**12h**	6.15E-08	8.37E-07	1.80E-07	7.19E-07
**24h**	1.10E-07	4.08E-07	4.75E-07	4.37E-07
**36h**	1.29E-07	1.02E-06	5.59E-07	3.05E-07
**48h**	3.00E-07	6.01E-07	1.57E-06	5.12E-07
**72h**	3.39E-08	8.26E-08	1.36E-07	3.66E-08
	cgd2_100			
**2h**	0.00E+00	0.00E+00	0.00E+00	0.00E+00
**6h**	3.01E-07	3.77E-07	9.90E-08	8.62E-07
**12h**	9.92E-08	1.68E-07	4.15E-07	3.00E-08
**24h**	7.02E-08	5.86E-07	1.13E-07	6.11E-07
**36h**	6.49E-08	3.41E-07	8.80E-07	9.82E-08
**48h**	7.44E-07	1.51E-06	2.87E-06	7.87E-07
**72h**	6.05E-07	1.15E-06	4.57E-06	1.22E-06
	cgd1_2630			
**2h**	1.82E-07	3.13E-07	2.40E-07	3.36E-07
**6h**	8.66E-07	6.54E-08	2.52E-07	2.17E-06
**12h**	4.86E-07	6.00E-07	1.93E-07	1.89E-06
**24h**	4.18E-07	1.48E-07	4.02E-07	1.10E-06
**36h**	7.07E-07	5.74E-07	1.47E-06	3.18E-06
**48h**	8.40E-07	3.64E-07	3.61E-07	1.54E-06
**72h**	3.48E-07	1.78E-07	1.09E-07	3.24E-07
	cgd1_2640			
**2h**	1.96E-06	2.54E-06	1.12E-06	1.00E-05
**6h**	9.98E-05	9.69E-05	3.04E-05	2.52E-04
**12h**	4.87E-06	1.21E-05	2.77E-06	1.48E-05
**24h**	1.47E-05	8.14E-06	3.81E-05	1.03E-04
**36h**	1.65E-05	1.45E-05	2.34E-05	1.21E-04
**48h**	2.55E-05	7.56E-06	1.80E-05	7.58E-05
**72h**	1.38E-05	6.48E-06	6.98E-06	1.30E-05
	cgd1_2660			
**2h**	2.07E-06	3.36E-06	5.36E-06	1.60E-06
**6h**	3.95E-06	1.15E-06	6.84E-07	9.71E-06
**12h**	5.46E-07	1.01E-06	6.36E-07	3.35E-06
**24h**	7.39E-07	7.85E-07	1.11E-06	2.15E-06
**36h**	6.60E-07	5.73E-07	7.98E-07	2.28E-06
**48h**	4.75E-07	1.15E-07	1.97E-07	5.87E-07
**72h**	3.08E-07	1.60E-07	1.22E-07	2.03E-07
	cgd1_2690			
**2h**	6.84E-05	7.89E-06	1.93E-05	0.00E+00
**6h**	1.96E-04	7.09E-05	1.22E-04	5.43E-04
**12h**	3.94E-05	1.31E-05	5.30E-06	5.20E-05
**24h**	7.75E-06	1.17E-05	3.36E-05	2.91E-04
**36h**	4.06E-05	2.32E-05	1.70E-05	6.97E-05

**48h**	2.10E-05	3.47E-06	3.59E-05	1.26E-04
**72h**	1.19E-04	2.43E-05	4.23E-05	3.00E-05
	cgd1_2700			
**2h**	1.39E-08	3.68E-07	7.92E-08	8.50E-07
**6h**	2.62E-07	2.06E-07	1.16E-07	3.91E-07
**12h**	6.48E-08	1.76E-07	1.90E-08	3.35E-07
**24h**	1.11E-08	3.33E-08	8.66E-08	1.28E-07
**36h**	9.22E-08	1.26E-07	2.15E-07	3.03E-07
**48h**	5.96E-08	5.51E-08	1.01E-07	9.18E-08
**72h**	7.43E-08	1.11E-07	1.01E-07	4.45E-08
	cgd1_2750			
**2h**	3.79E-05	5.49E-05	3.88E-05	2.39E-05
**6h**	5.95E-05	1.01E-04	3.14E-05	3.42E-04
**12h**	2.62E-06	0.00E+00	2.29E-06	6.31E-05
**24h**	4.11E-06	4.50E-06	1.60E-05	4.79E-05
**36h**	7.61E-06	6.08E-06	4.58E-06	1.50E-05
**48h**	8.40E-06	3.39E-06	4.41E-06	1.17E-05
**72h**	6.82E-07	4.57E-06	2.95E-06	7.08E-06
	cgd1_2760			
**2h**	7.88E-06	5.00E-06	1.77E-06	5.96E-06
**6h**	2.03E-05	1.98E-04	4.24E-05	1.62E-04
**12h**	2.79E-06	2.51E-06	7.58E-07	1.82E-05
**24h**	3.57E-06	6.29E-06	3.45E-05	4.82E-05
**36h**	9.56E-06	2.94E-06	2.10E-06	1.20E-05
**48h**	3.25E-06	2.56E-06	3.34E-06	8.72E-06
**72h**	5.05E-07	2.57E-07	1.58E-07	4.42E-07
	cgd1_2870			
**2h**	6.57E-03	8.12E-03	3.25E-04	1.11E-04
**6h**	5.76E-03	1.95E-03	1.66E-04	1.55E-04
**12h**	4.26E-03	1.76E-03	9.84E-06	1.76E-05
**24h**	1.27E-03	1.37E-03	2.31E-05	5.99E-05
**36h**	1.23E-03	1.83E-03	7.90E-05	3.56E-05
**48h**	1.01E-03	3.65E-04	4.11E-05	1.72E-05
**72h**	5.20E-04	4.67E-04	3.35E-05	8.87E-06
	cgd1_2890			
**2h**	1.78E-04	1.13E-04	2.98E-04	7.15E-04
**6h**	2.72E-05	3.21E-05	8.73E-05	4.83E-04
**12h**	5.63E-05	5.00E-05	3.84E-04	2.80E-03
**24h**	5.54E-05	5.38E-05	7.37E-05	7.60E-04
**36h**	1.28E-05	1.84E-05	1.66E-04	3.90E-04
**48h**	2.76E-05	1.62E-05	1.98E-04	3.94E-04
**72h**	9.03E-06	6.46E-06	6.75E-05	5.87E-05
	cgd1_2900			
**2h**	1.14E-06	3.22E-06	3.27E-07	0.00E+00
**6h**	3.22E-06	7.73E-07	1.89E-06	6.18E-06
**12h**	1.39E-07	9.51E-09	1.05E-07	4.07E-07
**24h**	3.04E-07	7.83E-07	2.94E-06	1.89E-05
**36h**	6.66E-07	7.88E-07	4.95E-06	7.31E-06
**48h**	1.98E-06	8.55E-07	5.31E-06	1.14E-05
**72h**	3.18E-07	2.11E-07	1.42E-05	4.11E-06
	cgd1_2910			
**2h**	4.66E-04	1.29E-03	4.73E-06	0.00E+00
**6h**	2.09E-03	1.42E-03	2.49E-05	6.95E-04

**12h**	8.67E-05	1.34E-05	8.67E-05	4.37E-05
**24h**	1.78E-04	2.85E-04	2.49E-04	3.10E-04
**36h**	1.85E-04	1.92E-04	1.87E-04	8.27E-05
**48h**	2.04E-04	1.85E-04	1.01E-04	1.68E-05
**72h**	7.98E-05	9.57E-05	9.92E-05	2.47E-05
	cgd1_2920			
**2h**	0.00E+00	0.00E+00	3.83E-07	9.43E-06
**6h**	2.00E-07	2.72E-07	3.34E-07	7.29E-07
**12h**	0.00E+00	3.80E-10	2.19E-07	0.00E+00
**24h**	5.25E-08	3.07E-09	7.62E-07	2.04E-05
**36h**	2.72E-07	3.83E-07	3.14E-06	3.11E-05
**48h**	5.67E-07	9.97E-07	7.04E-06	1.30E-05
**72h**	3.22E-07	3.16E-07	2.22E-05	8.25E-06
	cgd1_2930			
**2h**	0.00E+00	0.00E+00	0.00E+00	0.00E+00
**6h**	0.00E+00	6.55E-09	0.00E+00	0.00E+00
**12h**	4.54E-09	8.19E-09	4.27E-10	0.00E+00
**24h**	8.27E-09	1.50E-08	4.86E-09	1.35E-08
**36h**	3.15E-08	3.06E-08	4.67E-08	2.69E-08
**48h**	9.84E-08	5.76E-08	3.77E-08	1.94E-07
**72h**	1.38E-07	1.12E-07	2.26E-07	2.55E-07
	cgd1_2940			
**2h**	0.00E+00	0.00E+00	0.00E+00	0.00E+00
**6h**	0.00E+00	0.00E+00	0.00E+00	0.00E+00
**12h**	1.16E-08	0.00E+00	2.39E-09	4.88E-09
**24h**	2.06E-08	6.18E-09	5.51E-09	5.51E-09
**36h**	4.60E-08	1.05E-07	1.86E-08	7.61E-09
**48h**	9.96E-08	7.87E-08	1.29E-07	4.50E-08
**72h**	1.92E-07	1.25E-07	2.44E-07	2.45E-07
	cgd1_2960			
**2h**	3.50E-03	2.35E-03	1.22E-03	2.76E-03
**6h**	1.46E-03	1.15E-04	1.42E-03	1.81E-03
**12h**	5.89E-04	8.59E-04	2.30E-03	1.23E-03
**24h**	4.09E-03	7.36E-04	3.60E-03	2.42E-03
**36h**	7.27E-04	8.01E-04	1.48E-03	6.43E-04
**48h**	5.41E-04	5.19E-04	1.46E-03	6.88E-04
**72h**	2.47E-04	2.62E-04	3.68E-04	1.94E-04
	cgd1_2970			
**2h**	1.32E-08	1.38E-09	1.30E-08	0.00E+00
**6h**	1.59E-08	4.96E-08	5.96E-08	4.96E-08
**12h**	5.42E-09	0.00E+00	2.64E-08	2.85E-08
**24h**	9.85E-09	3.84E-08	9.36E-08	2.24E-08
**36h**	6.68E-08	5.86E-08	5.72E-08	8.68E-08
**48h**	7.25E-08	1.82E-07	7.99E-08	5.57E-08
**72h**	4.27E-08	2.56E-07	2.36E-07	1.21E-07
	cgd1_2980			
**2h**	2.13E-08	2.36E-07	5.62E-07	5.98E-07
**6h**	7.10E-07	8.56E-07	1.91E-06	8.60E-07
**12h**	0.00E+00	4.36E-08	8.96E-07	1.74E-07
**24h**	1.10E-07	1.99E-07	8.81E-07	1.46E-07
**36h**	1.13E-06	1.42E-06	7.96E-07	9.10E-07
**48h**	5.11E-07	1.69E-06	3.78E-07	3.53E-07
**72h**	8.05E-07	1.13E-06	6.99E-07	5.89E-07

	cgd1_2990			
**2h**	0.00E+00	0.00E+00	3.66E-09	4.18E-09
**6h**	8.56E-10	2.54E-08	5.50E-09	2.76E-08
**12h**	0.00E+00	0.00E+00	1.04E-08	4.93E-09
**24h**	3.61E-09	4.94E-09	5.53E-09	6.28E-09
**36h**	6.04E-08	3.59E-08	4.25E-08	5.46E-08
**48h**	2.19E-08	2.44E-08	2.99E-08	1.16E-08
**72h**	7.54E-08	4.53E-08	1.01E-07	3.11E-08
	cgd1_3000			
**2h**	9.65E-06	6.34E-05	7.07E-06	1.09E-05
**6h**	2.42E-05	3.32E-05	1.74E-05	1.37E-05
**12h**	1.64E-06	3.36E-06	2.65E-05	1.01E-05
**24h**	7.25E-06	8.98E-06	8.76E-06	6.46E-06
**36h**	9.33E-06	9.14E-06	6.13E-06	3.39E-06
**48h**	1.93E-06	2.84E-06	2.20E-06	1.51E-06
**72h**	7.79E-07	1.04E-06	1.37E-06	7.17E-07
	cgd1_3010			
**2h**	5.04E-07	1.88E-06	0.00E+00	0.00E+00
**6h**	1.23E-05	2.67E-04	5.83E-05	3.34E-05
**12h**	4.23E-06	6.51E-06	8.49E-06	2.16E-06
**24h**	4.02E-06	8.38E-06	2.46E-05	1.71E-05
**36h**	1.69E-05	7.14E-05	2.61E-05	1.62E-05
**48h**	4.89E-06	1.77E-05	1.34E-05	5.89E-06
**72h**	1.95E-06	5.69E-06	2.15E-06	8.44E-07
	cgd1_3130			
**2h**	6.02E-05	2.79E-05	3.35E-05	6.25E-05
**6h**	6.82E-06	7.88E-06	5.22E-06	1.01E-05
**12h**	2.31E-05	1.94E-05	7.75E-05	3.21E-05
**24h**	3.57E-05	1.28E-05	2.36E-05	2.19E-05
**36h**	6.16E-06	1.32E-05	1.24E-05	9.69E-06
**48h**	1.42E-05	1.15E-05	1.98E-05	1.99E-05
**72h**	3.62E-06	6.36E-06	4.52E-06	2.02E-06
	cgd1_3170			
**2h**	0.00E+00	0.00E+00	0.00E+00	0.00E+00
**6h**	0.00E+00	0.00E+00	0.00E+00	0.00E+00
**12h**	1.86E-06	1.28E-05	5.43E-06	2.06E-06
**24h**	1.68E-06	2.28E-07	1.18E-06	9.53E-07
**36h**	3.09E-07	1.73E-06	1.87E-06	9.83E-07
**48h**	1.84E-06	1.16E-06	4.81E-06	2.90E-06
**72h**	3.36E-06	5.39E-06	3.07E-06	1.26E-06
	cgd1_3270			
**2h**	6.44E-09	1.58E-08	1.45E-08	8.58E-08
**6h**	2.92E-08	1.49E-08	6.12E-09	5.23E-09
**12h**	5.00E-08	1.96E-08	4.55E-08	3.06E-08
**24h**	5.33E-08	3.12E-08	2.73E-08	1.56E-08
**36h**	8.93E-08	2.87E-08	2.63E-08	2.17E-08
**48h**	1.39E-08	7.24E-08	1.47E-08	2.00E-08
**72h**	1.69E-08	3.65E-09	4.38E-09	5.67E-09
	cgd1_3350			
**2h**	0.00E+00	0.00E+00	0.00E+00	0.00E+00
**6h**	0.00E+00	0.00E+00	0.00E+00	0.00E+00
**12h**	7.44E-10	1.75E-09	9.03E-10	4.44E-09
**24h**	9.67E-09	5.28E-09	1.88E-09	5.58E-09

**36h**	3.59E-08	8.16E-08	1.58E-08	6.59E-09
**48h**	5.35E-07	3.61E-07	8.01E-07	8.82E-07
**72h**	1.55E-06	8.04E-07	2.22E-07	3.79E-07
	cgd1_3390			
**2h**	9.61E-07	1.09E-06	1.32E-07	8.67E-07
**6h**	1.21E-06	1.51E-06	1.74E-06	1.21E-06
**12h**	3.77E-07	2.75E-07	2.25E-06	7.65E-07
**24h**	7.26E-07	1.77E-06	4.06E-06	2.11E-06
**36h**	1.67E-06	2.75E-06	1.73E-06	5.41E-07
**48h**	2.64E-06	2.65E-06	6.45E-06	2.29E-06
**72h**	5.33E-06	4.98E-06	3.00E-06	8.07E-07
	cgd1_3440			
**2h**	1.47E-05	7.30E-06	0.00E+00	9.86E-06
**6h**	3.44E-07	2.72E-06	8.77E-06	2.92E-06
**12h**	9.28E-07	2.78E-07	5.46E-05	1.87E-06
**24h**	3.37E-06	1.49E-05	3.17E-05	1.26E-06
**36h**	3.23E-06	1.37E-05	3.65E-05	1.53E-06
**48h**	1.04E-06	2.84E-06	2.39E-05	6.16E-07
**72h**	6.24E-07	1.52E-06	1.74E-05	1.49E-06
	cgd5_1440			
**2h**	9.48E-05	7.84E-05	4.44E-04	4.00E-04
**6h**	2.14E-06	3.44E-05	3.45E-06	1.87E-06
**12h**	1.02E-03	2.77E-03	2.93E-04	1.54E-02
**24h**	4.60E-04	1.03E-03	5.76E-04	2.12E-03
**36h**	4.21E-04	1.72E-03	2.04E-03	4.64E-03
**48h**	4.37E-04	2.18E-04	6.62E-04	1.75E-03
**72h**	5.88E-05	2.09E-04	1.28E-04	1.45E-04
	cgd5_1450			
**2h**	1.21E-07	1.34E-07	2.57E-07	2.37E-07
**6h**	4.48E-08	1.09E-07	2.30E-07	3.36E-07
**12h**	3.32E-06	4.48E-06	1.15E-06	8.20E-06
**24h**	2.27E-06	7.18E-06	1.96E-06	1.88E-06
**36h**	1.23E-06	2.74E-06	5.07E-06	9.08E-06
**48h**	1.40E-06	2.19E-06	4.17E-06	2.24E-06
**72h**	6.12E-07	1.06E-06	1.10E-06	7.40E-07
	cgd5_1460			
**2h**	4.89E-05	2.73E-04	5.96E-05	3.85E-05
**6h**	2.42E-05	5.59E-04	1.24E-04	8.63E-04
**12h**	5.33E-04	1.35E-03	3.78E-04	3.41E-03
**24h**	1.14E-04	9.64E-04	3.70E-04	1.80E-03
**36h**	2.09E-04	1.22E-03	1.82E-03	1.30E-03
**48h**	2.32E-04	2.80E-04	4.54E-03	1.26E-03
**72h**	1.90E-04	1.98E-03	5.66E-04	8.73E-04
	cgd5_1480			
**2h**	1.92E-08	5.16E-10	1.63E-09	0.00E+00
**6h**	0.00E+00	1.47E-09	0.00E+00	0.00E+00
**12h**	1.82E-06	6.53E-07	3.12E-07	2.87E-06
**24h**	1.42E-07	7.87E-07	3.83E-07	4.75E-07
**36h**	7.04E-07	1.09E-06	2.04E-06	2.74E-06
**48h**	3.58E-07	2.36E-07	8.36E-06	3.18E-07
**72h**	6.61E-08	1.36E-07	6.84E-08	1.14E-07
	cgd5_1490			
**2h**	2.25E-06	0.00E+00	0.00E+00	0.00E+00

**6h**	2.94E-05	1.99E-06	1.31E-05	1.51E-03
**12h**	6.62E-05	2.09E-04	2.00E-05	1.27E-04
**24h**	1.42E-04	1.01E-05	5.95E-05	4.28E-04
**36h**	8.09E-04	2.77E-04	3.01E-04	4.08E-04
**48h**	4.60E-04	5.76E-05	1.11E-04	4.95E-04
**72h**	1.03E-04	3.98E-05	9.89E-05	5.29E-05
	cgd5_1500			
**2h**	2.84E-07	4.27E-08	9.33E-07	2.14E-06
**6h**	0.00E+00	3.25E-08	1.36E-08	4.13E-09
**12h**	1.52E-06	9.22E-07	1.19E-06	2.25E-06
**24h**	1.14E-07	4.24E-06	1.33E-06	5.40E-07
**36h**	1.92E-06	3.30E-06	8.42E-06	3.41E-06
**48h**	8.71E-07	2.61E-06	2.03E-06	2.64E-06
**72h**	6.79E-07	5.22E-07	1.92E-07	4.56E-07
	cgd5_1580			
**2h**	1.53E-03	7.32E-03	8.09E-03	1.04E-03
**6h**	2.56E-03	1.01E-02	1.36E-02	1.75E-02
**12h**	6.83E-04	2.17E-03	2.79E-03	7.52E-03
**24h**	7.58E-04	6.55E-03	7.15E-03	7.23E-03
**36h**	1.24E-03	4.25E-03	5.32E-03	1.95E-03
**48h**	3.46E-04	1.20E-03	1.40E-03	9.10E-04
**72h**	6.87E-05	3.94E-04	8.67E-04	3.57E-04
	cgd5_1590			
**2h**	7.45E-07	1.11E-07	2.22E-07	6.85E-08
**6h**	6.59E-08	8.13E-08	3.83E-08	1.56E-08
**12h**	2.38E-05	7.67E-06	1.74E-06	9.02E-06
**24h**	1.84E-06	2.25E-06	9.86E-07	1.20E-06
**36h**	1.54E-06	1.94E-06	2.76E-06	1.70E-06
**48h**	1.33E-06	6.69E-07	1.26E-06	8.20E-07
**72h**	5.56E-08	3.85E-07	5.12E-07	2.79E-07
	cgd5_1600			
**2h**	9.48E-07	3.50E-06	5.24E-06	1.08E-06
**6h**	2.21E-06	2.39E-06	4.34E-06	6.10E-06
**12h**	5.69E-06	7.57E-06	2.81E-06	1.38E-05
**24h**	1.82E-06	7.37E-06	1.30E-05	9.02E-06
**36h**	3.42E-06	1.80E-05	2.02E-05	8.02E-06
**48h**	3.50E-06	7.11E-06	9.40E-06	1.91E-06
**72h**	2.94E-07	2.93E-06	2.42E-06	2.73E-06
	cgd5_1610			
**2h**	2.48E-06	2.29E-06	1.84E-06	1.81E-06
**6h**	7.33E-07	7.90E-07	1.32E-06	4.95E-06
**12h**	2.77E-06	2.79E-06	8.85E-07	2.13E-06
**24h**	1.79E-06	2.59E-06	4.40E-06	5.37E-06
**36h**	1.05E-06	4.37E-06	4.10E-06	1.85E-06
**48h**	1.72E-06	1.54E-06	2.47E-06	2.20E-06
**72h**	1.12E-07	8.15E-07	1.23E-06	6.35E-07
	cgd5_1620			
**2h**	1.68E-06	6.97E-07	4.77E-07	8.23E-07
**6h**	3.00E-07	3.06E-07	3.36E-07	6.39E-07
**12h**	5.28E-07	5.40E-07	1.67E-07	7.09E-07
**24h**	7.04E-07	6.40E-07	8.33E-07	9.78E-07
**36h**	8.39E-07	1.50E-06	1.54E-06	4.71E-07
**48h**	1.04E-06	9.03E-07	1.05E-06	5.35E-07

**72h**	8.32E-08	2.54E-07	5.06E-07	3.26E-07
	cgd5_1630			
**2h**	6.72E-03	7.05E-04	0.00E+00	1.41E-02
**6h**	4.29E-04	5.66E-04	6.59E-04	1.08E-02
**12h**	4.98E-04	4.46E-04	1.28E-04	6.39E-03
**24h**	5.02E-04	1.60E-03	4.35E-04	5.41E-04
**36h**	5.51E-04	4.28E-04	3.78E-04	1.24E-03
**48h**	3.82E-04	3.81E-04	5.16E-04	2.33E-03
**72h**	3.52E-04	1.23E-04	3.79E-04	5.39E-04
	cgd5_2680			
**2h**	0.00E+00	0.00E+00	0.00E+00	0.00E+00
**6h**	1.35E-06	0.00E+00	3.36E-07	5.30E-06
**12h**	9.01E-08	7.71E-09	2.45E-07	1.19E-07
**24h**	3.59E-07	4.33E-07	1.14E-06	4.02E-06
**36h**	1.59E-07	3.03E-07	4.25E-06	3.72E-06
**48h**	1.59E-05	1.09E-05	2.58E-05	3.67E-05
**72h**	9.56E-06	3.60E-06	1.35E-05	1.09E-05
	cgd5_2690			
**2h**	2.99E-08	0.00E+00	0.00E+00	0.00E+00
**6h**	1.04E-07	3.15E-08	1.12E-08	7.02E-08
**12h**	2.40E-07	6.31E-08	5.35E-08	8.96E-08
**24h**	9.89E-08	3.01E-04	1.28E-07	1.64E-06
**36h**	3.47E-07	1.10E-07	1.43E-07	3.24E-07
**48h**	4.58E-06	3.30E-06	3.56E-06	1.20E-06
**72h**	7.52E-07	3.45E-07	3.29E-07	3.67E-07
	cgd5_2730			
**2h**	8.27E-09	0.00E+00	0.00E+00	1.82E-09
**6h**	7.89E-09	0.00E+00	0.00E+00	0.00E+00
**12h**	2.27E-06	5.27E-06	1.52E-06	1.31E-06
**24h**	9.74E-07	1.42E-06	3.61E-07	9.27E-07
**36h**	2.89E-07	6.05E-08	4.33E-07	4.54E-07
**48h**	3.46E-07	1.34E-07	5.22E-07	2.01E-07
**72h**	1.12E-06	4.46E-07	2.52E-07	2.63E-07
	cgd5_2740			
**2h**	1.80E-07	2.03E-08	0.00E+00	3.44E-09
**6h**	4.54E-07	4.66E-07	4.11E-07	2.85E-07
**12h**	5.51E-05	5.58E-05	4.60E-05	2.99E-05
**24h**	3.74E-06	2.63E-06	2.57E-06	4.17E-06
**36h**	1.53E-06	1.01E-06	1.03E-05	6.43E-06
**48h**	3.33E-07	1.74E-07	1.29E-06	1.57E-06
**72h**	1.02E-06	8.53E-07	9.58E-07	3.36E-07
	cgd5_2760			
**2h**	0.00E+00	0.00E+00	0.00E+00	0.00E+00
**6h**	0.00E+00	0.00E+00	0.00E+00	0.00E+00
**12h**	1.06E-04	1.28E-03	4.98E-05	1.87E-05
**24h**	4.26E-06	3.09E-06	4.65E-06	8.77E-06
**36h**	2.44E-06	1.92E-06	8.73E-06	1.49E-05
**48h**	4.58E-07	1.49E-07	1.35E-06	4.00E-07
**72h**	8.54E-07	3.61E-07	2.99E-07	3.37E-07
	cgd5_2770			
**2h**	1.05E-08	0.00E+00	0.00E+00	9.33E-10
**6h**	8.30E-08	5.23E-08	1.00E-08	2.50E-08
**12h**	8.28E-07	1.32E-06	6.34E-07	1.10E-06

**24h**	2.83E-07	2.05E-07	1.87E-07	3.26E-07
**36h**	5.45E-07	2.33E-07	7.17E-07	8.62E-07
**48h**	3.39E-07	4.50E-07	5.75E-07	3.38E-07
**72h**	1.46E-06	5.93E-07	3.79E-07	2.62E-07
	cgd5_2790			
**2h**	8.83E-08	3.03E-08	2.82E-07	1.61E-07
**6h**	0.00E+00	3.20E-07	2.03E-07	1.14E-07
**12h**	4.83E-06	5.32E-06	9.53E-06	6.37E-06
**24h**	1.23E-06	1.01E-06	6.74E-06	3.25E-06
**36h**	1.57E-06	3.94E-07	6.73E-06	2.08E-05
**48h**	7.44E-06	1.82E-06	1.64E-05	8.01E-06
**72h**	4.89E-06	1.75E-06	1.56E-05	2.57E-05
	cgd5_2810			
**2h**	2.06E-06	1.35E-06	8.95E-08	3.75E-06
**6h**	1.50E-06	3.35E-07	1.64E-08	1.88E-06
**12h**	1.26E-06	1.79E-06	6.59E-06	2.13E-06
**24h**	7.31E-07	4.16E-07	2.99E-06	2.13E-05
**36h**	3.63E-07	3.35E-07	1.29E-06	3.45E-06
**48h**	8.09E-07	4.56E-07	4.33E-06	2.80E-06
**72h**	4.60E-06	1.03E-06	5.81E-07	3.00E-06
	cgd5_2820			
**2h**	0.00E+00	0.00E+00	0.00E+00	5.90E-10
**6h**	0.00E+00	0.00E+00	0.00E+00	0.00E+00
**12h**	9.97E-07	6.22E-07	1.87E-07	1.42E-07
**24h**	1.86E-07	1.28E-07	7.58E-08	1.23E-07
**36h**	3.13E-07	6.29E-08	2.88E-07	4.36E-07
**48h**	1.39E-07	1.11E-07	1.21E-07	6.77E-08
**72h**	8.24E-08	1.42E-07	6.21E-08	5.76E-08
	cgd5_2840			
**2h**	0.00E+00	0.00E+00	0.00E+00	0.00E+00
**6h**	0.00E+00	2.69E-06	5.80E-06	1.66E-05
**12h**	1.06E-04	7.71E-05	1.47E-04	3.49E-05
**24h**	3.88E-05	4.26E-05	1.11E-04	2.79E-05
**36h**	9.92E-05	2.80E-05	7.86E-05	1.14E-04
**48h**	3.57E-03	1.93E-04	1.81E-04	2.35E-04
**72h**	9.05E-04	1.58E-03	1.28E-04	2.01E-04
	cgd5_2850			
**2h**	0.00E+00	0.00E+00	0.00E+00	0.00E+00
**6h**	9.08E-06	0.00E+00	1.60E-05	0.00E+00
**12h**	0.00E+00	0.00E+00	9.48E-06	7.31E-06
**24h**	5.79E-06	2.80E-06	2.17E-05	2.34E-05
**36h**	1.62E-05	1.40E-05	4.14E-06	6.05E-05
**48h**	3.70E-04	2.24E-04	3.04E-05	7.03E-05
**72h**	4.97E-04	1.37E-04	4.09E-05	2.42E-04
	cgd5_2860			
**2h**	0.00E+00	4.43E-06	0.00E+00	0.00E+00
**6h**	0.00E+00	5.89E-08	0.00E+00	0.00E+00
**12h**	6.90E-04	8.36E-04	1.83E-03	1.13E-03
**24h**	8.38E-05	7.02E-05	1.86E-04	9.08E-05
**36h**	4.63E-05	2.24E-05	5.92E-04	5.32E-04
**48h**	9.34E-05	1.26E-05	2.80E-04	5.55E-05
**72h**	1.13E-04	1.95E-05	5.54E-05	6.28E-05
	cgd5_3090			

**2h**	5.36E-06	4.36E-05	2.07E-06	2.97E-06
**6h**	5.28E-06	3.70E-05	3.45E-05	2.68E-05
**12h**	8.34E-08	2.09E-06	4.45E-07	1.73E-07
**24h**	4.04E-06	1.99E-05	1.72E-05	1.18E-05
**36h**	7.46E-06	2.07E-05	8.71E-06	5.66E-06
**48h**	1.18E-04	1.31E-04	2.70E-05	1.67E-05
**72h**	7.27E-06	2.56E-05	4.71E-06	1.61E-05
	cgd5_3130			
**2h**	4.00E-08	4.41E-08	0.00E+00	2.78E-08
**6h**	1.89E-08	0.00E+00	0.00E+00	1.01E-08
**12h**	4.31E-07	1.36E-06	7.00E-07	4.13E-07
**24h**	5.63E-07	5.37E-07	4.19E-07	5.44E-07
**36h**	4.59E-07	2.50E-06	1.52E-06	1.27E-06
**48h**	1.50E-05	5.92E-06	3.50E-06	5.73E-06
**72h**	3.32E-06	3.39E-06	1.80E-06	3.62E-06
	cgd5_3140			
**2h**	5.26E-07	9.49E-08	5.13E-08	6.49E-07
**6h**	2.35E-07	4.80E-08	3.46E-07	1.81E-07
**12h**	1.55E-05	7.02E-05	5.10E-05	2.98E-05
**24h**	1.69E-05	3.51E-05	5.00E-06	4.30E-06
**36h**	2.64E-06	1.47E-05	1.56E-05	1.44E-05
**48h**	1.07E-04	7.11E-05	1.53E-05	2.18E-05
**72h**	2.42E-05	2.95E-05	1.36E-05	2.03E-05
	cgd5_3190			
**2h**	0.00E+00	0.00E+00	0.00E+00	0.00E+00
**6h**	0.00E+00	0.00E+00	0.00E+00	0.00E+00
**12h**	0.00E+00	0.00E+00	0.00E+00	4.15E-08
**24h**	6.33E-09	6.34E-08	1.85E-07	1.76E-08
**36h**	8.10E-07	4.83E-07	5.41E-07	1.53E-07
**48h**	7.67E-07	3.02E-06	2.34E-07	2.19E-07
**72h**	8.68E-07	1.36E-06	4.45E-07	4.71E-07
	cgd5_3200			
**2h**	0.00E+00	8.42E-08	0.00E+00	0.00E+00
**6h**	3.22E-06	1.19E-06	2.34E-06	2.89E-06
**12h**	6.12E-07	2.71E-07	4.09E-07	1.38E-06
**24h**	6.16E-07	4.74E-07	2.35E-06	1.26E-06
**36h**	2.28E-06	3.98E-06	3.25E-06	8.76E-06
**48h**	2.59E-06	9.22E-07	8.13E-07	6.49E-07
**72h**	5.52E-06	1.84E-06	2.10E-06	5.43E-06
	cgd5_3230			
**2h**	0.00E+00	0.00E+00	0.00E+00	0.00E+00
**6h**	3.12E-06	2.21E-06	3.97E-06	4.04E-06
**12h**	2.27E-07	0.00E+00	7.17E-07	1.15E-07
**24h**	5.54E-07	5.50E-07	1.63E-06	1.36E-06
**36h**	1.21E-06	8.18E-07	8.60E-07	9.64E-07
**48h**	2.59E-06	4.64E-07	8.82E-07	4.27E-07
**72h**	4.42E-07	2.50E-07	1.41E-07	1.75E-07
	cgd5_3300			
**2h**	4.82E-06	2.73E-06	2.98E-06	1.02E-05
**6h**	8.24E-07	1.95E-06	2.35E-06	3.82E-06
**12h**	2.57E-06	3.47E-06	2.51E-05	3.02E-05
**24h**	3.01E-06	8.33E-06	4.42E-06	3.74E-06
**36h**	1.47E-06	3.42E-06	2.42E-06	8.15E-06

**48h**	1.42E-06	8.16E-07	2.46E-06	1.53E-06
**72h**	1.07E-06	5.91E-07	1.51E-07	7.30E-07
	cgd5_3310			
**2h**	9.27E-04	1.07E-03	8.68E-04	1.73E-03
**6h**	4.45E-04	7.63E-04	9.44E-04	1.28E-03
**12h**	8.96E-04	3.07E-03	3.46E-03	1.75E-03
**24h**	9.32E-04	2.10E-03	1.29E-03	1.24E-03
**36h**	5.02E-04	6.66E-04	7.08E-04	3.37E-04
**48h**	1.33E-03	5.14E-04	8.87E-04	1.92E-04
**72h**	2.32E-03	2.26E-04	1.41E-04	9.23E-05
	cgd5_3330			
**2h**	1.03E-06	2.94E-07	2.60E-07	5.08E-07
**6h**	1.54E-06	2.22E-07	6.05E-07	3.81E-07
**12h**	1.02E-07	1.00E-07	6.33E-07	1.34E-07
**24h**	2.10E-07	6.42E-08	5.71E-07	4.30E-07
**36h**	5.34E-07	9.18E-07	5.86E-07	5.24E-07
**48h**	9.82E-07	2.07E-07	7.53E-07	2.17E-07
**72h**	2.51E-07	1.59E-07	1.81E-07	3.00E-07
	cgd5_3360			
**2h**	4.22E-06	3.27E-06	2.32E-06	6.30E-06
**6h**	2.07E-05	3.32E-06	9.18E-06	6.99E-06
**12h**	5.93E-07	4.79E-07	2.92E-06	1.36E-06
**24h**	1.93E-06	1.54E-06	6.44E-06	4.65E-06
**36h**	4.01E-06	1.48E-06	2.10E-06	2.12E-06
**48h**	3.67E-06	1.05E-06	2.31E-06	1.62E-06
**72h**	7.76E-07	1.99E-07	1.91E-07	4.15E-07
	cgd5_3370			
**2h**	1.12E-05	1.49E-05	0.00E+00	4.23E-06
**6h**	3.36E-05	1.35E-04	4.87E-05	1.72E-05
**12h**	5.12E-07	8.92E-06	5.79E-07	2.65E-06
**24h**	2.97E-06	7.01E-06	2.90E-05	2.17E-05
**36h**	1.19E-05	8.98E-05	1.72E-05	9.56E-06
**48h**	1.64E-05	1.26E-05	1.04E-05	5.98E-06
**72h**	6.69E-06	4.99E-06	3.12E-06	3.60E-06
	cgd5_3390			
**2h**	1.54E-05	5.00E-05	7.78E-06	2.00E-06
**6h**	1.39E-05	2.54E-05	2.46E-05	3.39E-05
**12h**	3.19E-06	2.39E-05	4.14E-05	6.73E-06
**24h**	8.67E-06	1.56E-04	4.14E-05	1.25E-05
**36h**	1.92E-05	9.82E-05	7.06E-05	2.40E-05
**48h**	2.32E-05	4.12E-05	1.65E-04	1.19E-05
**72h**	3.04E-04	4.00E-04	1.50E-03	2.02E-04
	cgd5_3410			
**2h**	1.69E-04	0.00E+00	6.38E-06	0.00E+00
**6h**	0.00E+00	3.18E-07	5.60E-05	6.59E-06
**12h**	0.00E+00	0.00E+00	5.02E-05	0.00E+00
**24h**	2.58E-06	1.50E-06	4.29E-06	1.30E-05
**36h**	1.59E-05	6.02E-06	1.78E-05	4.11E-05
**48h**	8.14E-04	1.39E-04	5.67E-04	1.14E-04
**72h**	4.51E-04	2.22E-04	4.93E-04	5.20E-04
	cgd5_3470			
**2h**	3.68E-04	7.35E-05	5.63E-05	5.59E-04
**6h**	6.77E-05	4.80E-05	1.08E-04	3.56E-04

**12h**	8.69E-06	3.54E-05	1.52E-04	2.80E-04
**24h**	7.87E-05	3.31E-05	3.64E-04	9.67E-05
**36h**	5.95E-05	2.88E-05	3.01E-04	4.33E-04
**48h**	1.56E-04	2.50E-05	1.69E-04	3.74E-04
**72h**	6.08E-05	4.30E-05	1.07E-04	2.15E-04
	cgd5_3500			
**2h**	4.88E-03	1.55E-03	5.07E-05	1.30E-04
**6h**	6.50E-04	3.35E-04	3.96E-04	3.52E-05
**12h**	1.04E-03	1.05E-03	8.00E-04	5.61E-04
**24h**	2.75E-03	8.80E-04	9.60E-04	5.58E-04
**36h**	2.00E-03	5.18E-04	4.08E-04	5.59E-04
**48h**	3.87E-03	1.99E-03	3.83E-04	5.87E-04
**72h**	5.48E-04	4.73E-04	1.79E-04	6.74E-04
	cgd5_3510			
**2h**	2.98E-04	6.58E-05	6.06E-05	2.51E-04
**6h**	1.25E-04	4.57E-05	2.80E-04	5.52E-04
**12h**	1.86E-05	3.27E-05	4.80E-04	3.55E-04
**24h**	7.07E-05	4.79E-05	7.58E-04	6.55E-04
**36h**	2.77E-05	1.73E-05	1.72E-04	6.87E-04
**48h**	2.99E-05	8.23E-06	9.69E-05	3.12E-04
**72h**	1.50E-05	2.02E-05	8.76E-05	6.85E-05
	cgd5_3520			
**2h**	5.62E-06	1.43E-06	6.68E-07	6.10E-07
**6h**	4.02E-06	1.05E-06	3.81E-06	1.18E-06
**12h**	1.65E-06	1.52E-06	4.66E-06	2.17E-06
**24h**	2.45E-06	1.44E-06	8.98E-06	6.20E-06
**36h**	2.04E-06	1.21E-06	1.35E-06	6.38E-06
**48h**	4.09E-06	4.64E-07	1.74E-06	2.45E-06
**72h**	1.48E-06	4.21E-07	6.33E-07	1.67E-06
	cgd5_3540			
**2h**	2.79E-04	1.76E-04	1.21E-04	3.08E-04
**6h**	3.00E-03	2.96E-04	7.90E-04	6.28E-04
**12h**	1.58E-04	1.19E-04	9.30E-04	8.15E-04
**24h**	4.85E-04	1.49E-04	1.63E-03	3.40E-04
**36h**	3.00E-04	1.93E-04	5.11E-04	1.27E-03
**48h**	2.04E-04	7.39E-05	4.46E-04	4.50E-04
**72h**	3.07E-05	1.61E-05	1.09E-04	2.02E-04
	cgd5_3550			
**2h**	3.96E-08	1.20E-08	6.76E-09	1.04E-08
**6h**	1.36E-08	2.96E-09	2.31E-08	1.36E-08
**12h**	1.09E-06	1.16E-06	8.70E-07	5.80E-07
**24h**	2.05E-07	1.36E-07	2.10E-07	2.47E-08
**36h**	2.44E-08	6.42E-08	2.15E-07	7.91E-07
**48h**	4.81E-08	5.33E-08	7.23E-08	5.29E-08
**72h**	4.97E-08	4.36E-08	1.16E-08	1.04E-07
	cgd5_3560			
**2h**	9.41E-05	4.68E-05	4.81E-05	1.07E-04
**6h**	4.59E-05	4.09E-05	1.47E-04	2.12E-04
**12h**	3.81E-05	3.09E-05	1.54E-03	2.53E-04
**24h**	1.01E-04	6.28E-05	8.31E-04	2.27E-04
**36h**	2.61E-05	1.56E-05	1.97E-04	3.82E-04
**48h**	4.69E-05	1.48E-05	1.23E-04	1.18E-04
**72h**	0.00E+00	7.50E-06	2.96E-05	1.19E-04

	cgd5_3570			
**2h**	8.91E-08	7.85E-08	9.53E-09	1.31E-08
**6h**	1.43E-08	3.68E-09	2.03E-09	2.80E-09
**12h**	0.00E+00	0.00E+00	6.01E-08	1.56E-08
**24h**	3.75E-09	6.04E-09	2.95E-09	4.35E-09
**36h**	1.33E-07	1.06E-07	1.01E-08	1.45E-08
**48h**	6.06E-07	3.40E-07	2.57E-07	6.03E-07
**72h**	9.99E-08	4.17E-08	9.52E-09	1.81E-08
	cgd5_3590			
**2h**	3.33E-05	2.06E-05	6.71E-06	2.29E-05
**6h**	1.62E-04	1.64E-05	4.58E-05	3.71E-05
**12h**	2.45E-07	1.90E-06	1.14E-05	1.32E-06
**24h**	8.67E-06	3.62E-06	1.00E-05	6.63E-06
**36h**	2.87E-06	1.74E-06	6.01E-06	1.13E-05
**48h**	4.66E-06	9.32E-07	4.23E-06	6.54E-06
**72h**	4.16E-07	1.10E-07	7.73E-07	2.99E-06
	cgd5_3620			
**2h**	0.00E+00	0.00E+00	0.00E+00	0.00E+00
**6h**	0.00E+00	2.79E-06	0.00E+00	0.00E+00
**12h**	1.16E-05	2.11E-06	7.31E-05	7.00E-05
**24h**	1.26E-05	4.23E-06	5.77E-05	4.17E-05
**36h**	7.63E-06	1.12E-05	2.56E-05	1.12E-04
**48h**	3.05E-05	3.56E-06	3.69E-05	7.45E-05
**72h**	5.20E-06	4.35E-06	2.42E-05	4.78E-05
	cgd5_3630			
**2h**	0.00E+00	0.00E+00	0.00E+00	8.88E-09
**6h**	2.48E-08	2.90E-08	9.07E-08	1.01E-08
**12h**	4.12E-07	5.19E-07	3.86E-07	9.28E-08
**24h**	3.49E-07	2.10E-07	1.09E-06	6.19E-07
**36h**	9.01E-07	5.07E-07	1.16E-06	1.06E-06
**48h**	1.48E-06	4.88E-07	2.22E-06	4.27E-07
**72h**	4.95E-07	2.51E-07	5.94E-07	5.16E-07
	cgd5_3640			
**2h**	1.94E-08	4.12E-09	2.36E-08	1.30E-08
**6h**	8.78E-09	7.61E-09	8.15E-09	1.89E-08
**12h**	6.07E-07	1.85E-07	1.88E-07	3.57E-07
**24h**	3.13E-07	1.70E-07	1.28E-07	2.61E-07
**36h**	1.72E-07	2.09E-07	7.49E-07	4.40E-07
**48h**	1.83E-07	5.32E-08	2.27E-07	2.64E-07
**72h**	1.81E-07	6.70E-08	1.13E-07	7.97E-08
	cgd5_3650			
**2h**	3.61E-03	1.88E-03	1.64E-04	2.58E-03
**6h**	1.91E-05	1.20E-04	1.13E-04	1.25E-04
**12h**	3.17E-01	2.42E-02	7.89E-03	8.45E-03
**24h**	2.02E-02	8.31E-03	1.48E-03	8.67E-03
**36h**	9.92E-03	3.20E-03	6.88E-03	2.38E-02
**48h**	5.46E-03	1.51E-03	4.06E-04	8.22E-03
**72h**	3.10E-03	1.81E-04	1.69E-04	9.46E-04
	cgd5_3700			
**2h**	0.00E+00	0.00E+00	0.00E+00	5.87E-08
**6h**	2.45E-06	2.75E-06	5.00E-06	7.41E-06
**12h**	4.03E-07	9.11E-08	6.72E-07	3.07E-07
**24h**	8.63E-07	3.19E-06	1.03E-05	6.08E-06

**36h**	3.68E-06	2.33E-06	6.49E-06	1.60E-06
**48h**	1.38E-06	2.08E-06	3.13E-06	3.08E-06
**72h**	5.38E-07	3.90E-07	9.72E-07	1.48E-06
	cgd5_3730			
**2h**	2.51E-06	0.00E+00	0.00E+00	1.40E-06
**6h**	5.81E-07	4.33E-06	7.57E-05	6.98E-06
**12h**	0.00E+00	1.22E-08	6.15E-07	1.62E-06
**24h**	9.92E-06	1.38E-05	4.85E-04	3.50E-05
**36h**	1.74E-05	7.23E-05	2.00E-04	4.71E-05
**48h**	2.30E-05	2.93E-05	6.82E-05	2.15E-05
**72h**	9.99E-06	1.21E-05	1.18E-04	4.57E-06
	cgd5_3740			
**2h**	1.16E-05	1.17E-05	1.15E-05	1.41E-05
**6h**	4.52E-05	3.00E-05	2.93E-05	2.96E-05
**12h**	4.17E-06	1.00E-06	1.50E-05	8.92E-06
**24h**	8.44E-06	1.56E-05	8.67E-05	8.69E-06
**36h**	1.24E-05	9.27E-06	1.76E-05	8.54E-06
**48h**	4.61E-06	4.14E-06	6.62E-06	2.23E-06
**72h**	2.48E-06	1.45E-06	6.57E-06	1.09E-06
	cgd5_3750			
**2h**	0.00E+00	6.22E-05	1.06E-04	2.31E-04
**6h**	8.79E-05	2.93E-04	7.94E-04	6.36E-05
**12h**	1.60E-04	2.22E-05	1.36E-02	8.64E-05
**24h**	1.55E-04	1.48E-03	6.47E-04	3.42E-04
**36h**	4.42E-04	5.05E-04	9.22E-04	7.15E-04
**48h**	6.08E-04	4.28E-04	1.50E-04	5.18E-04
**72h**	7.12E-04	1.11E-04	3.27E-04	1.56E-04
	cgd5_3780			
**2h**	3.25E-07	3.69E-08	0.00E+00	1.66E-07
**6h**	3.10E-09	1.19E-09	0.00E+00	2.39E-09
**12h**	1.33E-07	2.14E-09	8.73E-08	4.22E-08
**24h**	4.16E-08	8.14E-08	2.16E-08	2.34E-08
**36h**	5.30E-08	2.59E-08	3.99E-08	9.34E-08
**48h**	4.36E-08	2.15E-08	1.97E-08	1.51E-08
**72h**	3.17E-08	8.58E-09	5.78E-08	3.43E-08
	cgd5_3790			
**2h**	2.77E-08	1.67E-07	3.15E-08	1.24E-07
**6h**	3.31E-07	2.55E-07	1.40E-07	1.32E-07
**12h**	1.98E-09	1.40E-08	1.79E-08	3.92E-09
**24h**	5.31E-08	8.00E-08	2.94E-07	6.77E-08
**36h**	2.00E-08	1.39E-07	4.21E-07	7.78E-08
**48h**	1.63E-07	3.94E-07	4.67E-07	9.58E-08
**72h**	8.19E-08	1.89E-08	2.20E-07	1.39E-08
	cgd5_3810			
**2h**	0.00E+00	0.00E+00	0.00E+00	0.00E+00
**6h**	0.00E+00	0.00E+00	0.00E+00	0.00E+00
**12h**	0.00E+00	0.00E+00	0.00E+00	0.00E+00
**24h**	7.43E-07	1.59E-05	3.32E-06	1.13E-06
**36h**	6.14E-06	1.91E-05	1.08E-05	1.03E-05
**48h**	4.52E-04	5.89E-05	9.41E-05	1.06E-03
**72h**	3.79E-05	2.53E-05	1.92E-05	3.82E-05
	cgd5_3850			
**2h**	0.00E+00	4.70E-06	0.00E+00	0.00E+00

**6h**	1.21E-04	7.01E-05	1.04E-04	6.63E-05
**12h**	4.39E-06	4.09E-06	2.74E-05	8.30E-06
**24h**	1.68E-05	7.23E-05	9.34E-04	8.71E-05
**36h**	1.87E-05	6.68E-05	2.15E-04	4.28E-05
**48h**	3.62E-05	4.04E-05	1.52E-04	4.10E-05
**72h**	6.54E-06	1.87E-05	1.16E-04	4.96E-06
	cgd5_3870			
**2h**	0.00E+00	0.00E+00	0.00E+00	0.00E+00
**6h**	6.90E-08	7.29E-08	1.87E-08	7.68E-08
**12h**	4.85E-08	0.00E+00	0.00E+00	3.27E-09
**24h**	4.97E-08	9.78E-08	2.27E-07	1.87E-07
**36h**	8.58E-08	2.09E-07	3.24E-07	1.55E-07
**48h**	3.17E-07	3.05E-07	5.92E-07	5.23E-07
**72h**	6.82E-08	1.74E-07	2.08E-07	8.72E-08
	cgd5_3920			
**2h**	0.00E+00	0.00E+00	0.00E+00	0.00E+00
**6h**	0.00E+00	0.00E+00	0.00E+00	0.00E+00
**12h**	2.15E-05	9.98E-06	4.14E-05	2.42E-05
**24h**	4.14E-06	4.48E-06	4.41E-05	5.87E-06
**36h**	1.91E-06	3.75E-06	3.53E-05	1.75E-05
**48h**	1.09E-06	1.16E-06	1.31E-06	2.18E-06
**72h**	2.11E-06	1.14E-06	4.85E-06	3.58E-06
	cgd5_3940			
**2h**	1.25E-04	5.45E-05	1.23E-05	8.38E-04
**6h**	2.57E-05	5.38E-05	2.44E-06	4.80E-04
**12h**	1.37E-04	5.25E-05	1.69E-05	1.69E-04
**24h**	1.76E-04	7.84E-05	4.97E-05	1.22E-03
**36h**	7.92E-05	5.19E-05	3.12E-05	1.13E-03
**48h**	1.51E-04	5.55E-05	6.98E-05	1.40E-04
**72h**	4.45E-05	1.42E-05	1.15E-05	3.97E-04
	cgd5_3950			
**2h**	9.00E-06	2.68E-06	5.68E-07	5.25E-06
**6h**	4.70E-05	3.64E-05	1.63E-05	4.82E-05
**12h**	4.48E-07	1.80E-06	2.87E-07	1.30E-06
**24h**	1.00E-05	1.11E-05	6.41E-06	6.91E-05
**36h**	4.07E-06	4.00E-06	3.47E-06	1.94E-05
**48h**	4.96E-06	2.94E-06	7.10E-07	1.20E-05
**72h**	1.09E-06	1.13E-06	2.27E-07	5.89E-06
	cgd5_3960			
**2h**	1.48E-02	2.23E-03	6.72E-04	1.66E-03
**6h**	1.03E-04	9.04E-05	2.83E-05	3.50E-04
**12h**	8.55E-03	1.38E-03	1.63E-04	5.53E-05
**24h**	2.94E-03	1.83E-03	3.49E-04	5.21E-04
**36h**	2.61E-04	4.02E-04	4.04E-04	1.02E-04
**48h**	3.20E-03	3.07E-04	1.11E-04	4.57E-05
**72h**	2.13E-04	6.78E-05	4.02E-05	1.49E-04
	cgd5_3970			
**2h**	0.00E+00	1.96E-07	8.81E-08	9.39E-06
**6h**	0.00E+00	1.89E-07	2.49E-07	1.36E-06
**12h**	4.66E-05	5.65E-05	1.19E-05	2.59E-05
**24h**	7.29E-06	1.05E-05	2.58E-06	9.48E-05
**36h**	5.48E-06	9.82E-06	6.24E-06	2.99E-04
**48h**	7.05E-06	5.99E-06	1.38E-06	7.96E-05

**72h**	7.94E-06	6.95E-06	1.53E-05	1.04E-05
	cgd5_3980			
**2h**	0.00E+00	0.00E+00	0.00E+00	0.00E+00
**6h**	0.00E+00	0.00E+00	0.00E+00	1.44E-09
**12h**	1.23E-08	9.56E-09	0.00E+00	8.74E-11
**24h**	5.50E-09	4.56E-10	1.29E-09	1.40E-09
**36h**	7.64E-08	1.38E-07	5.08E-09	3.96E-08
**48h**	8.37E-07	2.27E-07	1.62E-07	4.80E-07
**72h**	1.06E-07	1.99E-07	1.08E-07	1.44E-07
	cgd5_3990			
**2h**	0.00E+00	4.01E-06	1.62E-07	5.57E-05
**6h**	0.00E+00	4.17E-08	0.00E+00	1.57E-06
**12h**	5.95E-05	1.01E-04	4.97E-06	6.86E-06
**24h**	9.81E-06	6.97E-06	9.79E-07	3.55E-05
**36h**	1.03E-05	3.48E-06	1.24E-05	7.22E-05
**48h**	4.28E-05	3.55E-06	1.15E-06	1.01E-04
**72h**	1.67E-06	4.59E-06	3.27E-06	6.41E-05
	cgd5_4010			
**2h**	0.00E+00	0.00E+00	0.00E+00	0.00E+00
**6h**	0.00E+00	0.00E+00	0.00E+00	0.00E+00
**12h**	1.03E-05	8.41E-05	2.78E-06	3.88E-06
**24h**	3.33E-04	4.86E-05	1.80E-04	4.40E-05
**36h**	1.50E-05	6.39E-04	3.99E-04	1.05E-04
**48h**	7.09E-05	9.19E-04	2.90E-04	2.98E-05
**72h**	2.76E-04	5.83E-04	3.50E-04	3.59E-05
	cgd5_4040			
**2h**	0.00E+00	0.00E+00	0.00E+00	0.00E+00
**6h**	1.18E-07	4.94E-08	8.48E-08	8.54E-09
**12h**	3.52E-07	1.07E-07	3.97E-07	2.98E-07
**24h**	2.21E-07	1.61E-07	2.84E-07	9.97E-07
**36h**	1.26E-05	2.33E-06	1.46E-06	5.98E-06
**48h**	2.83E-06	7.96E-07	6.16E-07	9.12E-06
**72h**	4.38E-06	8.20E-07	6.31E-07	1.33E-06
	cgd5_4060			
**2h**	5.14E-04	6.94E-04	4.99E-04	2.44E-04
**6h**	8.50E-04	3.72E-04	1.06E-04	4.06E-04
**12h**	2.75E-04	6.52E-06	5.70E-05	2.34E-05
**24h**	3.70E-04	1.69E-04	2.30E-04	4.97E-04
**36h**	1.31E-04	2.14E-04	8.33E-04	3.85E-04
**48h**	2.28E-03	1.11E-04	1.26E-04	1.05E-05
**72h**	3.49E-04	4.24E-05	2.56E-05	9.10E-05
	cgd5_4070			
**2h**	0.00E+00	2.19E-08	1.23E-07	5.46E-06
**6h**	7.36E-07	1.64E-06	1.44E-06	3.85E-06
**12h**	7.45E-07	3.10E-07	6.90E-08	7.83E-07
**24h**	7.53E-07	7.74E-07	1.89E-06	1.34E-05
**36h**	9.74E-05	1.65E-06	1.01E-06	1.10E-05
**48h**	4.08E-06	3.00E-06	2.25E-06	6.10E-06
**72h**	1.39E-06	1.19E-06	3.00E-07	2.46E-06
	cgd5_4080			
**2h**	0.00E+00	0.00E+00	0.00E+00	3.62E-07
**6h**	0.00E+00	0.00E+00	0.00E+00	0.00E+00
**12h**	2.99E-07	1.65E-06	1.89E-07	1.09E-07

**24h**	3.91E-07	3.65E-07	2.93E-07	4.21E-07
**36h**	1.21E-06	1.72E-06	1.96E-06	3.13E-06
**48h**	2.48E-06	1.59E-06	1.11E-06	1.62E-06
**72h**	1.46E-06	1.99E-07	9.22E-07	1.04E-06
	cgd5_4100			
**2h**	2.20E-03	1.66E-03	3.43E-02	6.21E-05
**6h**	3.32E-03	2.02E-03	2.11E-04	9.62E-05
**12h**	2.20E-03	7.79E-04	3.09E-03	1.65E-05
**24h**	2.41E-03	1.99E-03	1.85E-03	7.99E-05
**36h**	8.33E-04	3.03E-03	1.04E-02	9.73E-05
**48h**	3.13E-03	1.55E-03	2.85E-03	8.28E-05
**72h**	9.99E-04	1.92E-03	3.91E-03	3.83E-06
	cgd5_4110			
**2h**	7.72E-05	4.51E-05	0.00E+00	1.73E-05
**6h**	0.00E+00	2.94E-05	0.00E+00	2.80E-05
**12h**	5.62E-02	2.04E-04	1.62E-03	5.32E-04
**24h**	1.14E-02	1.02E-03	5.08E-04	3.56E-04
**36h**	6.26E-03	7.87E-04	2.61E-04	6.63E-03
**48h**	3.17E-03	5.23E-04	5.74E-04	2.14E-04
**72h**	7.31E-04	9.12E-05	1.28E-04	3.81E-04
	cgd5_4130			
**2h**	0.00E+00	0.00E+00	4.85E-09	4.81E-09
**6h**	1.25E-09	1.03E-08	0.00E+00	2.20E-09
**12h**	3.48E-07	7.45E-08	1.48E-07	2.53E-07
**24h**	2.99E-07	3.32E-07	2.52E-07	2.59E-07
**36h**	3.32E-08	1.01E-07	3.94E-07	5.40E-07
**48h**	1.07E-07	1.73E-07	4.71E-07	4.00E-07
**72h**	1.42E-07	9.81E-08	1.56E-07	2.71E-07
	cgd5_4150			
**2h**	0.00E+00	0.00E+00	0.00E+00	0.00E+00
**6h**	0.00E+00	0.00E+00	0.00E+00	0.00E+00
**12h**	0.00E+00	0.00E+00	0.00E+00	5.70E-10
**24h**	1.46E-09	2.35E-08	1.61E-08	4.27E-09
**36h**	2.78E-08	7.78E-08	2.47E-08	2.87E-08
**48h**	3.58E-07	6.74E-07	4.11E-07	3.62E-07
**72h**	4.18E-07	4.03E-07	7.26E-07	4.45E-07
	cgd5_4170			
**2h**	1.08E-08	0.00E+00	0.00E+00	2.78E-09
**6h**	6.40E-08	3.97E-07	3.75E-07	1.69E-07
**12h**	6.12E-06	2.52E-06	1.46E-06	2.68E-06
**24h**	1.29E-06	3.92E-06	9.41E-06	4.81E-06
**36h**	2.75E-06	3.84E-06	7.91E-06	1.05E-05
**48h**	1.03E-06	7.05E-07	1.68E-06	1.44E-06
**72h**	1.06E-06	1.48E-06	2.48E-06	8.85E-07
	cgd5_4210			
**2h**	2.17E-07	0.00E+00	0.00E+00	0.00E+00
**6h**	2.60E-07	7.23E-07	1.93E-05	3.52E-05
**12h**	5.83E-07	1.00E-06	1.78E-06	5.83E-06
**24h**	2.12E-06	6.80E-06	6.77E-05	5.97E-05
**36h**	2.40E-06	4.87E-05	3.25E-05	6.43E-05
**48h**	2.84E-06	2.11E-05	5.29E-05	4.04E-05
**72h**	4.74E-06	1.34E-05	7.01E-05	2.68E-05
	cgd5_4220			

**2h**	0.00E+00	0.00E+00	0.00E+00	0.00E+00
**6h**	0.00E+00	0.00E+00	0.00E+00	0.00E+00
**12h**	7.37E-07	2.54E-08	4.05E-08	6.57E-08
**24h**	2.88E-08	1.34E-07	3.68E-08	1.96E-08
**36h**	1.47E-08	6.51E-08	1.26E-07	3.30E-07
**48h**	1.78E-08	1.28E-08	7.62E-08	1.71E-07
**72h**	2.49E-08	3.47E-08	2.70E-08	4.49E-08
	cgd5_4230			
**2h**	0.00E+00	0.00E+00	0.00E+00	0.00E+00
**6h**	0.00E+00	6.99E-08	5.15E-09	2.75E-07
**12h**	9.42E-06	2.35E-06	6.29E-06	9.70E-06
**24h**	6.42E-07	2.55E-05	7.46E-06	6.11E-06
**36h**	4.21E-06	2.15E-05	2.79E-05	6.16E-05
**48h**	5.47E-06	3.20E-05	8.13E-05	3.06E-05
**72h**	1.53E-05	3.44E-05	4.90E-05	5.74E-05
	cgd5_4240			
**2h**	0.00E+00	0.00E+00	0.00E+00	0.00E+00
**6h**	0.00E+00	0.00E+00	1.41E-08	7.15E-09
**12h**	1.12E-05	6.15E-06	1.64E-06	5.07E-06
**24h**	6.47E-07	4.14E-06	4.17E-06	3.08E-06
**36h**	3.90E-06	7.27E-06	3.98E-05	4.01E-05
**48h**	1.20E-06	2.69E-06	6.86E-06	8.51E-06
**72h**	2.13E-06	3.17E-06	7.37E-06	2.98E-06
	cgd5_4250			
**2h**	1.71E-07	0.00E+00	0.00E+00	0.00E+00
**6h**	9.54E-07	8.36E-06	7.80E-06	6.31E-06
**12h**	9.28E-07	9.37E-07	8.34E-07	8.66E-07
**24h**	4.03E-07	6.87E-06	1.88E-05	4.21E-05
**36h**	1.96E-06	3.29E-05	1.38E-05	2.98E-05
**48h**	3.15E-06	9.30E-06	2.11E-05	1.85E-05
**72h**	2.47E-06	8.49E-06	7.42E-06	7.91E-06
	cgd5_4260			
**2h**	4.30E-04	3.02E-04	1.96E-04	5.53E-04
**6h**	2.13E-05	3.61E-04	4.91E-04	2.73E-04
**12h**	2.13E-04	1.09E-04	1.50E-04	9.12E-05
**24h**	2.62E-04	1.82E-03	8.31E-04	9.81E-04
**36h**	6.32E-05	4.39E-04	2.02E-04	1.53E-03
**48h**	8.24E-05	5.33E-04	1.10E-04	1.62E-04
**72h**	1.75E-05	8.67E-05	1.18E-04	8.98E-05
	cgd5_4280			
**2h**	0.00E+00	0.00E+00	0.00E+00	0.00E+00
**6h**	3.44E-09	3.24E-08	6.12E-08	2.77E-09
**12h**	1.18E-08	2.80E-09	3.00E-09	7.87E-09
**24h**	5.38E-08	3.54E-08	2.76E-07	3.43E-07
**36h**	2.16E-07	1.79E-07	5.84E-08	1.62E-07
**48h**	3.60E-07	9.60E-08	1.83E-07	2.17E-07
**72h**	7.10E-08	9.68E-08	3.43E-08	1.13E-07
	cgd5_4290			
**2h**	4.69E-04	1.47E-04	3.56E-04	0.00E+00
**6h**	1.90E-05	1.10E-04	6.96E-04	0.00E+00
**12h**	5.01E-06	3.75E-05	7.58E-05	2.23E-04
**24h**	9.55E-05	5.02E-04	4.23E-05	1.80E-03
**36h**	2.83E-05	1.36E-04	7.82E-05	1.19E-04

**48h**	4.21E-05	3.14E-04	1.46E-04	1.05E-04
**72h**	2.24E-05	7.29E-06	8.53E-05	5.80E-05
	cgd5_4300			
**2h**	2.08E-07	9.62E-08	7.37E-09	7.61E-08
**6h**	0.00E+00	0.00E+00	2.79E-09	7.79E-09
**12h**	3.18E-08	4.09E-08	3.01E-07	6.68E-08
**24h**	7.98E-08	2.73E-08	7.49E-08	5.94E-08
**36h**	2.45E-07	2.19E-07	4.85E-07	6.44E-08
**48h**	7.06E-07	1.32E-07	7.25E-07	5.41E-07
**72h**	5.66E-07	9.52E-07	1.11E-06	3.38E-07
	cgd5_4320			
**2h**	5.65E-07	7.22E-07	7.95E-08	8.73E-07
**6h**	1.72E-06	8.62E-07	6.72E-07	2.89E-06
**12h**	2.94E-08	9.35E-08	4.06E-07	9.60E-08
**24h**	6.63E-07	2.28E-07	9.74E-07	1.56E-06
**36h**	3.73E-07	2.61E-07	2.96E-07	2.01E-07
**48h**	4.17E-07	3.60E-07	7.14E-07	9.86E-07
**72h**	3.39E-07	6.72E-07	7.74E-07	4.04E-07
	cgd5_4330			
**2h**	5.45E-05	5.01E-05	9.39E-05	7.42E-05
**6h**	4.66E-05	4.86E-05	1.85E-04	4.45E-05
**12h**	7.78E-06	3.39E-06	3.02E-04	4.78E-06
**24h**	1.56E-05	1.74E-05	1.48E-04	2.67E-05
**36h**	8.45E-06	4.80E-06	1.58E-04	9.09E-06
**48h**	6.30E-06	5.03E-06	1.97E-04	1.27E-05
**72h**	6.04E-06	5.65E-06	9.78E-05	5.97E-06
	cgd5_4350			
**2h**	3.68E-06	2.66E-06	0.00E+00	2.76E-06
**6h**	1.24E-06	1.51E-06	2.88E-06	9.78E-07
**12h**	9.94E-09	2.29E-07	2.57E-05	1.39E-06
**24h**	8.05E-07	4.89E-07	3.94E-06	1.03E-06
**36h**	1.11E-06	5.02E-07	9.66E-06	1.13E-06
**48h**	3.12E-07	5.74E-07	2.51E-06	2.20E-07
**72h**	4.04E-07	7.66E-07	2.76E-06	6.93E-07
	cgd5_4360			
**2h**	5.58E-05	6.15E-05	7.33E-05	8.56E-05
**6h**	3.27E-05	3.81E-05	1.72E-04	4.22E-05
**12h**	3.38E-05	4.37E-05	1.77E-04	2.74E-05
**24h**	1.86E-05	1.64E-05	1.12E-04	2.26E-05
**36h**	2.50E-05	1.59E-05	2.20E-04	3.22E-05
**48h**	6.52E-06	7.44E-06	7.90E-05	9.48E-06
**72h**	2.14E-03	3.26E-06	1.77E-05	5.27E-06
	cgd5_4370			
**2h**	1.48E-07	2.07E-07	1.37E-08	3.57E-08
**6h**	1.68E-06	2.10E-06	6.38E-07	1.05E-06
**12h**	1.15E-06	1.51E-06	7.14E-07	3.19E-07
**24h**	4.94E-07	5.88E-07	8.39E-07	1.23E-06
**36h**	3.47E-06	6.61E-07	4.34E-06	4.45E-06
**48h**	1.35E-06	1.23E-06	5.52E-07	5.45E-07
**72h**	2.54E-07	2.12E-07	8.39E-08	5.13E-07
	cgd5_4400			
**2h**	1.49E-06	2.32E-06	4.21E-08	1.83E-06
**6h**	1.37E-06	9.51E-07	3.18E-07	5.86E-07

**12h**	5.70E-07	3.30E-07	6.09E-07	3.04E-07
**24h**	2.73E-07	4.58E-07	1.86E-07	2.83E-07
**36h**	7.05E-07	1.84E-07	2.87E-06	4.47E-07
**48h**	3.03E-07	6.09E-07	1.99E-07	1.87E-07
**72h**	2.30E-07	1.28E-07	1.69E-07	2.94E-07
	cgd5_4420			
**2h**	9.49E-05	2.87E-04	9.42E-05	4.65E-04
**6h**	1.56E-04	3.57E-04	1.68E-05	1.80E-04
**12h**	4.45E-05	1.27E-05	6.45E-05	2.82E-04
**24h**	4.10E-04	1.82E-04	6.93E-06	6.40E-04
**36h**	2.03E-04	5.66E-05	4.52E-05	1.62E-04
**48h**	2.51E-04	1.12E-04	1.45E-05	8.05E-04
**72h**	9.19E-05	6.33E-05	2.74E-05	1.04E-04
	cgd5_4430			
**2h**	3.13E-02	1.02E-02	1.20E-04	1.94E-02
**6h**	1.20E-02	4.96E-03	8.34E-05	1.81E-02
**12h**	1.25E-02	3.92E-03	7.83E-04	9.77E-03
**24h**	9.19E-03	6.70E-03	1.96E-04	2.11E-03
**36h**	3.82E-03	2.35E-03	1.21E-04	6.35E-03
**48h**	4.62E-03	2.25E-03	1.63E-04	6.84E-03
**72h**	2.81E-03	1.92E-03	4.61E-05	2.71E-03
	cgd5_4440			
**2h**	2.96E-07	3.75E-07	2.91E-06	7.62E-07
**6h**	5.69E-07	5.72E-07	6.75E-06	1.10E-06
**12h**	2.42E-06	4.11E-07	1.05E-05	1.11E-06
**24h**	1.72E-06	1.19E-06	1.13E-05	3.41E-06
**36h**	3.02E-06	9.17E-07	4.33E-05	5.47E-06
**48h**	5.24E-06	1.92E-06	1.87E-05	4.86E-06
**72h**	1.21E-05	2.53E-06	1.50E-05	1.69E-06
	cgd5_4470			
**2h**	0.00E+00	0.00E+00	0.00E+00	0.00E+00
**6h**	4.98E-06	2.96E-05	2.02E-05	7.54E-07
**12h**	3.78E-06	6.94E-06	3.72E-05	6.74E-06
**24h**	8.39E-06	1.51E-05	7.58E-05	4.38E-05
**36h**	4.98E-05	8.18E-06	1.61E-04	8.27E-05
**48h**	2.23E-05	3.02E-05	4.16E-05	6.13E-05
**72h**	7.15E-06	4.33E-06	2.29E-05	6.82E-06
	cgd5_4480			
**2h**	2.03E-08	0.00E+00	0.00E+00	0.00E+00
**6h**	2.15E-06	6.66E-06	1.12E-05	5.11E-06
**12h**	8.19E-07	4.75E-07	6.08E-06	2.18E-06
**24h**	7.86E-07	7.16E-07	4.40E-05	2.06E-05
**36h**	1.69E-06	1.06E-06	4.68E-05	5.40E-06
**48h**	2.73E-06	3.00E-06	3.57E-05	1.04E-05
**72h**	5.42E-07	1.93E-06	7.09E-06	7.08E-07
	cgd1_950			
**2h**	1.32E-08	0.00E+00	0.00E+00	3.39E-09
**6h**	0.00E+00	0.00E+00	0.00E+00	3.51E-09
**12h**	1.29E-05	1.43E-05	1.98E-06	3.46E-06
**24h**	5.74E-06	2.03E-06	3.16E-06	1.47E-06
**36h**	1.68E-06	6.17E-07	1.56E-05	3.59E-06
**48h**	1.47E-06	2.38E-07	1.51E-06	1.71E-06
**72h**	1.82E-07	1.40E-07	6.15E-07	1.89E-07

	cgd1_960			
**2h**	0.00E+00	1.31E-09	0.00E+00	0.00E+00
**6h**	0.00E+00	0.00E+00	1.21E-08	2.89E-09
**12h**	6.36E-08	2.91E-07	2.74E-09	1.36E-08
**24h**	1.92E-07	1.05E-07	1.28E-07	1.23E-07
**36h**	1.96E-07	2.54E-07	5.15E-07	1.87E-07
**48h**	4.56E-07	1.45E-07	4.06E-07	4.45E-07
**72h**	1.03E-07	1.43E-07	1.35E-07	1.79E-07
	cgd1_970			
**2h**	0.00E+00	9.42E-09	0.00E+00	0.00E+00
**6h**	0.00E+00	0.00E+00	0.00E+00	0.00E+00
**12h**	2.36E-06	4.84E-06	4.40E-07	8.99E-07
**24h**	2.71E-06	6.77E-07	1.95E-06	9.70E-07
**36h**	5.92E-07	8.33E-07	4.41E-06	2.31E-06
**48h**	1.03E-06	3.85E-07	9.59E-07	3.67E-07
**72h**	1.40E-06	1.24E-06	2.43E-06	1.35E-06
	cgd1_980			
**2h**	1.09E-03	3.42E-04	1.20E-03	5.75E-04
**6h**	3.27E-04	1.27E-04	3.42E-03	8.62E-04
**12h**	3.74E-04	1.47E-04	3.24E-04	2.61E-04
**24h**	2.90E-04	1.09E-04	1.92E-03	6.43E-04
**36h**	2.18E-04	1.21E-04	1.54E-03	2.55E-04
**48h**	4.60E-04	1.04E-04	1.72E-03	3.49E-04
**72h**	1.27E-03	5.75E-04	3.35E-03	1.23E-03
	cgd1_990			
**2h**	3.97E-09	0.00E+00	0.00E+00	0.00E+00
**6h**	0.00E+00	0.00E+00	8.43E-09	1.94E-08
**12h**	4.93E-08	4.16E-08	1.45E-09	3.33E-08
**24h**	2.96E-08	1.96E-08	8.36E-08	3.71E-08
**36h**	1.51E-07	2.44E-07	1.62E-07	2.22E-07
**48h**	4.49E-06	1.45E-06	3.78E-06	1.73E-06
**72h**	1.35E-05	8.98E-06	1.22E-05	1.47E-05
	cgd1_1000			
**2h**	0.00E+00	0.00E+00	0.00E+00	2.31E-06
**6h**	7.64E-05	0.00E+00	1.05E-05	1.35E-04
**12h**	7.84E-04	2.12E-04	1.08E-06	5.72E-04
**24h**	8.73E-05	4.88E-05	1.35E-05	2.54E-04
**36h**	2.71E-04	2.22E-04	3.18E-05	1.30E-03
**48h**	9.23E-04	3.66E-04	2.46E-05	3.16E-04
**72h**	1.76E-03	1.18E-03	1.20E-04	2.10E-03
	cgd1_1010			
**2h**	0.00E+00	0.00E+00	0.00E+00	0.00E+00
**6h**	0.00E+00	0.00E+00	0.00E+00	0.00E+00
**12h**	1.52E-05	2.09E-06	5.25E-06	1.87E-05
**24h**	5.32E-06	2.27E-06	2.54E-05	2.14E-06
**36h**	1.09E-05	7.83E-06	2.61E-04	3.96E-05
**48h**	3.24E-06	5.25E-06	1.66E-04	7.53E-06
**72h**	1.50E-05	2.09E-06	1.30E-05	3.41E-06
	cgd1_1020			
**2h**	2.26E-06	4.52E-06	1.79E-04	0.00E+00
**6h**	1.58E-04	2.64E-05	3.71E-04	5.30E-04
**12h**	3.19E-07	2.26E-06	4.04E-07	2.94E-05
**24h**	9.58E-05	1.29E-05	1.23E-04	3.03E-04

**36h**	2.76E-04	3.99E-05	6.20E-05	1.85E-04
**48h**	1.94E-04	4.19E-05	2.32E-05	2.30E-04
**72h**	1.49E-04	4.60E-05	1.36E-04	3.78E-05
	cgd1_1030			
**2h**	4.46E-08	1.42E-08	0.00E+00	0.00E+00
**6h**	1.13E-07	7.81E-08	3.37E-07	2.14E-07
**12h**	1.36E-08	0.00E+00	1.96E-10	9.67E-09
**24h**	2.48E-08	3.16E-08	3.01E-07	1.33E-07
**36h**	1.76E-07	1.03E-07	1.62E-07	3.44E-07
**48h**	9.48E-08	1.22E-07	1.08E-07	1.07E-07
**72h**	1.41E-07	1.70E-08	9.06E-08	2.78E-08
	cgd1_1050			
**2h**	4.57E-06	4.81E-06	6.27E-05	4.81E-06
**6h**	4.36E-06	3.09E-06	1.84E-05	5.51E-06
**12h**	3.36E-06	9.35E-07	1.35E-06	2.86E-06
**24h**	2.82E-06	3.13E-06	2.07E-05	5.39E-06
**36h**	5.06E-06	1.01E-06	3.66E-05	5.10E-06
**48h**	3.67E-06	2.04E-06	8.35E-06	4.25E-06
**72h**	1.34E-06	8.47E-08	2.24E-06	3.79E-07
	cgd1_1060			
**2h**	4.91E-04	1.28E-04	1.00E-03	2.35E-04
**6h**	1.57E-04	1.69E-04	1.78E-03	1.38E-03
**12h**	8.03E-05	2.33E-06	1.30E-04	7.19E-05
**24h**	1.63E-04	6.48E-05	1.56E-03	4.84E-04
**36h**	1.42E-04	3.87E-05	4.33E-04	1.27E-04
**48h**	2.51E-04	2.06E-05	5.68E-04	8.16E-05
**72h**	1.84E-05	3.74E-06	9.51E-05	6.62E-06
	cgd1_1080			
**2h**	0.00E+00	4.99E-07	0.00E+00	0.00E+00
**6h**	1.11E-07	1.65E-08	1.01E-04	0.00E+00
**12h**	2.44E-04	6.89E-05	3.30E-05	7.12E-05
**24h**	1.15E-04	2.11E-05	8.07E-05	1.44E-04
**36h**	2.48E-04	4.04E-05	2.01E-04	2.48E-04
**48h**	1.83E-04	3.54E-05	1.08E-04	1.19E-04
**72h**	3.85E-05	2.38E-05	4.56E-05	6.81E-05
	cgd1_1090			
**2h**	2.05E-09	0.00E+00	0.00E+00	0.00E+00
**6h**	0.00E+00	0.00E+00	0.00E+00	0.00E+00
**12h**	7.37E-07	4.09E-06	3.23E-07	2.48E-08
**24h**	2.98E-07	5.31E-07	2.78E-07	2.66E-07
**36h**	1.57E-07	8.57E-08	5.19E-06	1.71E-06
**48h**	4.71E-08	1.94E-07	2.29E-07	2.63E-07
**72h**	4.08E-08	2.93E-08	8.02E-08	4.36E-08
	cgd1_1100			
**2h**	0.00E+00	0.00E+00	0.00E+00	0.00E+00
**6h**	0.00E+00	0.00E+00	0.00E+00	0.00E+00
**12h**	1.07E-09	4.45E-09	0.00E+00	4.52E-11
**24h**	2.13E-08	1.88E-08	6.20E-09	3.10E-08
**36h**	5.02E-08	1.24E-08	1.79E-07	6.09E-08
**48h**	1.59E-06	1.42E-06	1.53E-05	8.50E-06
**72h**	2.64E-06	2.44E-06	3.58E-06	5.29E-06
	cgd1_1110			
**2h**	8.13E-08	5.16E-08	0.00E+00	1.04E-09

**6h**	9.16E-07	1.59E-06	4.67E-07	4.05E-07
**12h**	8.67E-08	3.43E-07	1.37E-07	1.96E-08
**24h**	2.23E-07	2.87E-07	2.40E-06	1.03E-06
**36h**	7.40E-07	3.95E-07	1.67E-06	7.51E-07
**48h**	8.69E-07	8.66E-07	5.04E-06	2.64E-06
**72h**	1.88E-06	9.80E-07	2.18E-06	1.63E-06
	cgd1_1120			
**2h**	0.00E+00	0.00E+00	0.00E+00	0.00E+00
**6h**	5.18E-06	7.07E-06	1.49E-04	7.55E-05
**12h**	1.26E-05	5.40E-05	2.40E-04	1.41E-05
**24h**	3.21E-06	7.66E-06	6.78E-04	2.48E-04
**36h**	2.94E-05	2.36E-05	1.40E-03	4.34E-04
**48h**	4.67E-06	4.23E-06	3.95E-04	2.83E-04
**72h**	1.29E-05	1.62E-05	1.18E-04	2.00E-04
	cgd1_1130			
**2h**	0.00E+00	0.00E+00	0.00E+00	0.00E+00
**6h**	0.00E+00	0.00E+00	0.00E+00	2.56E-08
**12h**	4.72E-09	5.59E-09	2.17E-09	1.46E-10
**24h**	1.31E-08	3.64E-08	1.67E-08	4.55E-08
**36h**	1.13E-07	5.67E-08	2.75E-07	1.66E-07
**48h**	4.21E-08	1.41E-08	1.08E-07	6.39E-08
**72h**	9.32E-08	1.80E-07	2.32E-07	1.86E-07
	cgd1_1140			
**2h**	0.00E+00	1.74E-08	2.28E-08	6.94E-09
**6h**	1.31E-06	1.92E-06	1.37E-06	1.56E-06
**12h**	2.80E-07	5.40E-07	1.68E-07	3.75E-09
**24h**	2.94E-07	2.29E-07	3.04E-06	2.35E-06
**36h**	1.25E-06	7.65E-07	2.25E-06	1.24E-06
**48h**	4.49E-06	3.29E-06	8.12E-06	5.37E-06
**72h**	1.25E-06	2.14E-06	2.25E-06	2.23E-06
	cgd1_1150			
**2h**	2.77E-08	0.00E+00	2.43E-08	4.17E-09
**6h**	3.14E-08	0.00E+00	0.00E+00	7.04E-09
**12h**	5.46E-09	1.40E-07	1.86E-09	2.68E-09
**24h**	4.85E-08	5.59E-08	8.33E-08	6.70E-08
**36h**	1.01E-06	5.58E-07	9.44E-07	7.69E-07
**48h**	6.35E-06	3.48E-06	8.81E-06	4.21E-06
**72h**	2.11E-06	2.30E-06	1.41E-06	1.39E-06
	cgd1_1160			
**2h**	0.00E+00	0.00E+00	0.00E+00	0.00E+00
**6h**	0.00E+00	1.62E-07	0.00E+00	0.00E+00
**12h**	0.00E+00	0.00E+00	5.30E-05	3.86E-07
**24h**	4.24E-08	2.33E-06	2.19E-05	3.17E-05
**36h**	9.11E-06	3.08E-06	1.31E-04	5.70E-05
**48h**	8.46E-05	7.70E-05	2.71E-04	3.09E-04
**72h**	1.44E-04	4.55E-04	9.73E-04	7.91E-04
	cgd1_1170			
**2h**	0.00E+00	0.00E+00	0.00E+00	0.00E+00
**6h**	4.68E-07	0.00E+00	7.45E-10	5.13E-07
**12h**	6.66E-08	3.54E-07	7.97E-09	6.73E-09
**24h**	3.37E-08	7.45E-08	1.08E-07	3.04E-07
**36h**	1.38E-07	3.58E-07	8.80E-07	1.18E-06
**48h**	9.33E-07	8.07E-07	1.60E-06	1.60E-06

**72h**	1.24E-06	3.20E-06	8.98E-06	5.85E-06
	cgd1_1180			
**2h**	0.00E+00	1.07E-08	7.66E-09	0.00E+00
**6h**	0.00E+00	1.26E-08	0.00E+00	0.00E+00
**12h**	2.08E-07	2.15E-06	6.04E-08	3.76E-08
**24h**	1.91E-07	2.82E-07	3.72E-08	2.17E-07
**36h**	2.19E-07	2.45E-07	1.32E-06	7.89E-07
**48h**	5.34E-07	7.46E-07	2.60E-06	2.74E-06
**72h**	9.07E-07	2.10E-06	7.12E-06	2.82E-06
	cgd1_1190			
**2h**	0.00E+00	0.00E+00	0.00E+00	5.05E-06
**6h**	0.00E+00	1.79E-07	0.00E+00	1.64E-06
**12h**	1.62E-05	2.14E-04	3.42E-04	2.21E-05
**24h**	7.01E-06	2.46E-05	1.57E-04	8.82E-05
**36h**	5.16E-06	9.61E-06	4.84E-04	3.36E-04
**48h**	8.41E-06	6.95E-06	3.16E-04	2.15E-04
**72h**	5.08E-06	1.07E-05	7.32E-04	1.85E-04
	cgd1_1210			
**2h**	2.08E-05	3.40E-06	2.09E-03	5.57E-04
**6h**	4.88E-07	1.87E-07	3.89E-05	8.69E-06
**12h**	1.35E-04	5.36E-04	3.63E-03	2.42E-04
**24h**	9.14E-05	1.45E-04	4.48E-04	2.85E-04
**36h**	2.23E-05	3.40E-05	3.84E-03	1.32E-03
**48h**	5.65E-06	1.77E-05	2.32E-04	2.16E-04
**72h**	1.76E-04	6.07E-05	7.19E-04	4.42E-04
	cgd1_1220			
**2h**	3.82E-07	2.00E-09	0.00E+00	1.45E-07
**6h**	3.57E-08	1.60E-08	1.92E-07	3.20E-07
**12h**	2.17E-05	1.18E-05	4.71E-06	4.34E-06
**24h**	6.48E-06	3.48E-06	2.96E-06	1.31E-06
**36h**	1.66E-06	1.93E-06	4.68E-06	3.93E-06
**48h**	1.99E-06	9.23E-07	1.75E-06	1.08E-06
**72h**	9.06E-07	6.29E-07	2.72E-06	1.30E-06
	cgd1_1230			
**2h**	0.00E+00	0.00E+00	0.00E+00	0.00E+00
**6h**	0.00E+00	0.00E+00	0.00E+00	0.00E+00
**12h**	1.42E-05	7.90E-06	2.22E-05	2.49E-06
**24h**	3.51E-06	1.88E-05	9.78E-05	2.01E-06
**36h**	2.49E-06	2.23E-05	2.14E-05	3.94E-06
**48h**	1.17E-06	9.55E-06	2.40E-05	1.58E-06
**72h**	5.82E-07	2.50E-06	1.19E-06	4.89E-07
	cgd1_1240			
**2h**	5.41E-07	3.97E-08	0.00E+00	1.40E-08
**6h**	4.44E-07	4.84E-08	1.05E-07	1.12E-07
**12h**	9.84E-06	6.89E-06	2.04E-06	2.62E-06
**24h**	8.86E-07	2.17E-06	3.17E-06	1.37E-06
**36h**	6.85E-07	1.31E-06	2.88E-06	4.16E-06
**48h**	4.69E-07	9.98E-07	1.36E-06	1.50E-06
**72h**	3.42E-07	2.53E-06	2.18E-07	3.47E-07
	cgd1_1250			
**2h**	2.79E-07	1.64E-07	3.80E-07	5.46E-07
**6h**	2.09E-06	2.71E-06	2.66E-06	1.92E-06
**12h**	7.02E-06	1.30E-05	7.88E-06	7.37E-06

24h	8.97E-07	7.08E-06	1.11E-05	9.96E-07
36h	3.93E-06	5.83E-06	7.84E-06	7.56E-06
48h	9.57E-07	6.20E-06	5.90E-06	1.60E-06
72h	2.72E-06	3.32E-06	1.15E-05	2.71E-06
	cgd1_1270			
2h	2.84E-03	1.03E-05	1.35E-05	2.77E-05
6h	7.35E-06	9.11E-06	4.82E-05	1.80E-04
12h	1.11E-03	2.24E-04	1.34E-05	1.00E-03
24h	3.40E-04	1.42E-04	7.80E-05	1.38E-03
36h	3.65E-03	1.18E-03	2.04E-04	3.15E-03
48h	1.12E-03	7.30E-04	1.11E-04	2.46E-03
72h	7.63E-03	2.77E-04	1.88E-04	4.68E-03
	cgd1_1260			
2h	0.00E+00	6.22E-08	0.00E+00	4.44E-08
6h	0.00E+00	0.00E+00	0.00E+00	3.94E-08
12h	2.54E-07	5.03E-08	8.07E-09	4.37E-07
24h	1.57E-07	1.51E-06	9.81E-06	1.48E-07
36h	1.52E-06	1.09E-05	9.68E-06	1.73E-06
48h	4.62E-06	1.74E-05	5.27E-05	1.15E-06
72h	9.33E-06	1.53E-05	2.01E-05	3.77E-06
	cgd1_1280			
2h	6.46E-05	5.96E-05	7.29E-05	1.05E-04
6h	5.77E-05	7.49E-05	7.16E-05	5.89E-05
12h	1.09E-05	6.89E-06	1.50E-05	1.66E-05
24h	7.67E-06	5.41E-05	5.14E-04	2.93E-05
36h	1.44E-05	2.89E-05	1.86E-05	1.51E-05
48h	4.35E-06	1.07E-05	3.03E-05	6.21E-06
72h	1.93E-06	2.42E-06	4.01E-06	7.79E-07
	cgd1_1290			
2h	4.42E-05	2.85E-05	1.44E-05	2.24E-05
6h	1.65E-05	4.67E-05	6.71E-05	2.20E-05
12h	4.90E-06	2.04E-06	1.46E-05	6.45E-06
24h	3.16E-06	2.31E-05	1.62E-04	1.26E-05
36h	5.67E-06	2.58E-05	2.22E-05	6.89E-06
48h	5.33E-06	1.45E-05	4.75E-05	6.29E-06
72h	1.58E-06	4.29E-06	5.36E-06	8.41E-07
	cgd1_1300			
2h	6.71E-03	2.22E-04	3.23E-05	5.32E-03
6h	7.84E-03	3.93E-04	9.39E-05	5.19E-03
12h	1.31E-03	1.48E-04	1.81E-05	9.18E-04
24h	1.78E-03	1.82E-04	4.20E-04	4.06E-03
36h	5.73E-04	8.29E-05	2.07E-05	6.98E-04
48h	5.22E-04	9.85E-05	4.33E-05	1.35E-03
72h	7.94E-04	8.64E-05	4.62E-05	1.14E-03
	cgd1_1310			
2h	1.82E-05	3.60E-05	1.45E-05	2.42E-05
6h	3.76E-06	1.13E-05	5.76E-05	1.67E-05
12h	4.38E-06	2.59E-06	9.95E-06	7.01E-06
24h	9.27E-06	9.87E-06	1.86E-04	2.41E-05
36h	2.54E-06	2.78E-05	1.69E-05	4.80E-06
48h	3.25E-06	7.62E-06	2.50E-05	7.08E-06
72h	1.45E-06	9.29E-06	9.08E-06	7.35E-07
	cgd1_1330			

**2h**	1.75E-05	1.56E-04	3.19E-05	5.87E-05
**6h**	1.32E-03	2.80E-04	1.07E-04	1.70E-03
**12h**	3.22E-04	3.59E-06	2.75E-05	2.22E-04
**24h**	1.94E-04	1.55E-04	1.76E-04	1.47E-03
**36h**	1.16E-03	8.29E-04	3.61E-05	4.30E-04
**48h**	1.20E-03	1.89E-04	6.20E-05	7.43E-04
**72h**	7.74E-04	1.85E-04	1.49E-04	2.64E-04
	cgd1_1340			
**2h**	2.34E-03	1.98E-04	3.47E-05	6.60E-03
**6h**	2.04E-04	3.21E-04	1.06E-04	4.21E-04
**12h**	7.71E-05	8.62E-05	2.33E-05	5.41E-04
**24h**	1.92E-04	8.43E-05	1.26E-04	5.83E-04
**36h**	2.82E-04	1.82E-04	2.02E-05	4.81E-04
**48h**	1.94E-04	1.37E-04	1.53E-04	1.62E-04
**72h**	2.34E-04	2.35E-04	1.72E-05	2.11E-04
	cgd1_1350			
**2h**	6.42E-08	5.84E-07	0.00E+00	7.76E-07
**6h**	2.46E-06	1.05E-06	1.08E-06	1.36E-06
**12h**	2.21E-07	6.44E-07	8.30E-07	3.34E-07
**24h**	4.91E-07	1.98E-07	9.72E-07	1.29E-06
**36h**	1.68E-07	5.72E-07	7.78E-07	1.09E-06
**48h**	6.27E-07	1.38E-07	3.87E-07	1.23E-06
**72h**	1.85E-07	2.58E-07	2.86E-07	3.15E-07
	cgd1_1360			
**2h**	0.00E+00	0.00E+00	0.00E+00	0.00E+00
**6h**	0.00E+00	0.00E+00	0.00E+00	0.00E+00
**12h**	1.03E-04	1.14E-03	2.71E-04	1.20E-05
**24h**	9.58E-05	5.64E-05	5.49E-05	1.81E-05
**36h**	8.37E-05	1.06E-05	8.69E-05	6.04E-05
**48h**	4.37E-04	6.97E-05	1.75E-04	1.56E-05
**72h**	3.59E-04	1.44E-04	1.93E-04	4.05E-05
	cgd1_1370			
**2h**	0.00E+00	0.00E+00	0.00E+00	0.00E+00
**6h**	0.00E+00	0.00E+00	0.00E+00	0.00E+00
**12h**	5.75E-08	2.38E-07	2.22E-07	9.25E-08
**24h**	7.19E-08	5.74E-08	5.03E-08	6.15E-08
**36h**	4.41E-08	1.94E-07	4.67E-07	1.86E-07
**48h**	9.11E-08	9.74E-08	8.81E-08	1.54E-07
**72h**	1.18E-07	2.01E-07	1.79E-07	1.23E-07
	cgd1_1380			
**2h**	4.59E-08	2.77E-07	1.31E-07	1.29E-08
**6h**	6.89E-09	1.03E-07	3.97E-08	7.87E-08
**12h**	1.32E-06	1.22E-06	1.96E-06	1.32E-06
**24h**	4.22E-07	3.17E-07	2.78E-07	1.23E-06
**36h**	3.89E-07	1.44E-06	3.54E-06	3.21E-06
**48h**	6.09E-07	4.44E-07	4.88E-07	1.71E-06
**72h**	8.27E-07	7.08E-07	1.53E-06	7.55E-07
	cgd1_1390			
**2h**	3.07E-08	4.15E-07	2.13E-09	8.95E-08
**6h**	3.29E-07	7.36E-08	3.08E-07	2.02E-07
**12h**	1.02E-07	3.38E-07	3.14E-07	2.28E-07
**24h**	1.06E-07	8.74E-08	3.79E-07	9.72E-07
**36h**	1.97E-07	6.82E-07	1.06E-06	6.69E-07

**48h**	3.60E-07	3.43E-07	4.87E-07	9.14E-07
**72h**	1.63E-06	7.47E-07	1.13E-06	8.31E-07
	cgd1_1400			
**2h**	7.98E-08	2.84E-08	1.31E-07	1.69E-06
**6h**	0.00E+00	2.24E-07	2.52E-07	1.59E-07
**12h**	1.44E-07	2.00E-07	4.22E-07	8.15E-07
**24h**	1.32E-07	1.82E-07	5.49E-07	1.98E-06
**36h**	3.19E-07	8.73E-07	1.62E-06	4.47E-06
**48h**	1.60E-06	1.30E-06	1.38E-06	6.72E-06
**72h**	3.00E-06	1.87E-06	3.46E-06	8.54E-06
	cgd1_1410			
**2h**	3.02E-07	2.53E-06	8.42E-06	2.02E-05
**6h**	1.96E-06	4.27E-06	4.24E-06	9.25E-06
**12h**	8.19E-07	4.43E-07	1.81E-06	5.49E-06
**24h**	1.18E-06	1.37E-06	3.91E-06	6.26E-06
**36h**	1.36E-06	2.08E-06	2.06E-06	8.91E-06
**48h**	1.47E-06	1.24E-06	2.80E-06	7.97E-06
**72h**	7.71E-07	6.37E-07	6.18E-07	1.67E-06
	cgd1_1420			
**2h**	1.34E-07	3.46E-07	0.00E+00	1.45E-05
**6h**	6.91E-06	8.64E-06	4.97E-05	1.08E-04
**12h**	7.58E-07	0.00E+00	8.71E-07	1.63E-05
**24h**	1.20E-06	3.19E-06	3.14E-05	1.25E-04
**36h**	6.21E-06	7.73E-06	1.84E-05	2.91E-04
**48h**	2.63E-05	8.67E-06	1.48E-05	2.57E-04
**72h**	8.98E-05	2.22E-06	3.37E-06	6.86E-05
	cgd1_1430			
**2h**	1.20E-07	7.03E-07	8.07E-07	3.67E-07
**6h**	0.00E+00	1.78E-08	0.00E+00	6.48E-09
**12h**	2.79E-07	4.60E-07	1.56E-06	9.57E-07
**24h**	4.22E-07	3.84E-07	3.76E-07	3.81E-07
**36h**	3.14E-07	2.84E-07	1.14E-06	3.54E-06
**48h**	5.52E-07	2.31E-07	4.87E-07	1.09E-06
**72h**	1.70E-07	3.30E-08	9.03E-08	9.92E-08
	cgd1_1440			
**2h**	2.18E-07	1.44E-07	1.18E-07	6.51E-06
**6h**	0.00E+00	7.14E-09	0.00E+00	0.00E+00
**12h**	8.57E-07	1.56E-06	1.25E-06	2.08E-06
**24h**	3.03E-07	4.28E-07	7.13E-07	2.89E-07
**36h**	2.28E-07	3.19E-07	6.50E-07	4.14E-06
**48h**	1.57E-06	3.54E-07	4.11E-07	8.02E-07
**72h**	9.16E-08	2.94E-08	1.72E-08	7.05E-08
	cgd1_1450			
**2h**	5.87E-05	4.20E-04	5.88E-04	2.46E-03
**6h**	1.28E-05	1.33E-05	2.58E-05	1.13E-04
**12h**	1.21E-04	2.50E-05	8.66E-05	3.54E-04
**24h**	5.75E-05	4.41E-05	9.62E-05	3.54E-04
**36h**	1.22E-05	1.24E-05	2.43E-05	3.41E-04
**48h**	5.48E-05	9.51E-06	3.97E-05	6.40E-04
**72h**	1.23E-05	1.96E-06	2.30E-06	3.95E-05
	cgd1_1460			
**2h**	1.18E-07	2.07E-07	4.38E-07	1.14E-07
**6h**	6.73E-09	1.08E-07	8.90E-09	8.32E-09

**12h**	5.00E-07	2.25E-07	1.17E-07	7.66E-07
**24h**	3.15E-07	1.41E-07	1.03E-06	4.95E-07
**36h**	7.41E-07	6.33E-07	1.34E-06	2.55E-06
**48h**	1.77E-06	5.27E-07	1.01E-06	1.94E-06
**72h**	7.62E-07	5.25E-07	6.50E-07	0.00E+00
	cgd1_1480			
**2h**	2.41E-06	5.05E-07	2.45E-06	3.98E-07
**6h**	1.33E-06	3.59E-07	5.27E-07	7.61E-07
**12h**	6.47E-08	2.39E-07	1.68E-07	3.06E-07
**24h**	5.69E-07	3.44E-07	6.58E-07	1.32E-06
**36h**	1.28E-06	1.54E-07	2.86E-07	2.12E-07
**48h**	1.99E-07	3.94E-07	1.43E-06	6.94E-07
**72h**	8.16E-08	9.13E-08	9.30E-08	9.95E-08
	cgd1_1490			
**2h**	0.00E+00	0.00E+00	0.00E+00	0.00E+00
**6h**	1.06E-05	4.60E-07	6.04E-06	4.82E-06
**12h**	2.75E-05	3.47E-05	2.34E-04	1.66E-04
**24h**	2.94E-05	2.50E-05	4.39E-04	1.63E-04
**36h**	5.21E-05	2.67E-05	2.04E-04	1.27E-04
**48h**	5.86E-05	4.04E-05	2.65E-04	2.58E-04
**72h**	1.60E-04	9.14E-05	3.17E-04	4.03E-04
	cgd1_1500			
**2h**	4.05E-06	2.81E-07	1.78E-06	1.94E-06
**6h**	3.03E-06	6.34E-07	2.10E-06	1.49E-06
**12h**	4.32E-07	4.06E-07	1.36E-06	2.22E-06
**24h**	3.47E-07	7.65E-07	2.13E-06	3.22E-06
**36h**	7.89E-07	5.76E-07	1.31E-06	1.65E-06
**48h**	6.18E-07	5.71E-07	9.19E-07	1.96E-06
**72h**	3.69E-06	2.69E-06	4.21E-06	6.36E-06
	cgd1_1510			
**2h**	0.00E+00	0.00E+00	0.00E+00	0.00E+00
**6h**	0.00E+00	0.00E+00	0.00E+00	0.00E+00
**12h**	3.20E-08	4.04E-08	7.66E-08	9.99E-08
**24h**	1.97E-08	2.07E-08	0.00E+00	2.24E-08
**36h**	1.50E-08	3.40E-08	5.57E-08	1.51E-07
**48h**	2.57E-06	2.74E-06	4.56E-06	1.19E-05
**72h**	7.15E-06	3.10E-06	2.28E-06	9.29E-06
	cgd1_1520			
**2h**	0.00E+00	0.00E+00	0.00E+00	0.00E+00
**6h**	0.00E+00	0.00E+00	7.85E-10	0.00E+00
**12h**	4.19E-07	1.25E-07	1.63E-07	3.50E-07
**24h**	2.27E-08	5.84E-08	1.16E-07	6.13E-08
**36h**	2.07E-07	2.06E-07	1.01E-07	3.79E-07
**48h**	2.90E-08	3.43E-08	7.72E-08	7.35E-08
**72h**	6.02E-07	8.76E-07	2.22E-07	1.31E-06
	cgd1_1530			
**2h**	4.24E-05	1.53E-05	1.08E-05	2.41E-03
**6h**	1.64E-05	2.50E-05	9.94E-04	1.38E-04
**12h**	1.73E-06	6.48E-07	1.72E-04	1.05E-05
**24h**	1.54E-05	5.65E-06	1.59E-04	8.87E-05
**36h**	8.07E-05	2.76E-05	1.06E-04	8.74E-05
**48h**	5.47E-05	1.84E-05	1.03E-04	1.59E-04
**72h**	1.06E-05	1.20E-05	1.44E-04	1.36E-05

	cgd1_1550			
**2h**	2.54E-06	4.02E-07	4.42E-07	4.77E-07
**6h**	5.60E-06	8.84E-07	7.79E-07	9.23E-07
**12h**	2.79E-07	3.59E-08	1.64E-07	2.76E-07
**24h**	2.17E-07	1.65E-07	9.49E-07	4.96E-07
**36h**	5.98E-07	9.43E-07	5.35E-07	8.94E-07
**48h**	3.75E-07	1.94E-07	4.06E-07	5.91E-07
**72h**	3.23E-07	4.01E-07	1.90E-07	3.04E-07
	cgd1_1560			
**2h**	9.00E-05	2.20E-05	2.38E-05	1.64E-05
**6h**	1.80E-06	1.78E-06	2.54E-06	1.97E-06
**12h**	2.30E-05	1.03E-05	2.43E-05	3.40E-05
**24h**	1.55E-05	9.30E-06	1.72E-05	9.87E-06
**36h**	6.28E-06	1.45E-05	1.57E-05	9.61E-06
**48h**	1.06E-05	2.52E-06	1.77E-05	6.82E-06
**72h**	3.54E-06	4.38E-06	2.91E-06	1.98E-06
	cgd1_1570			
**2h**	9.45E-06	2.72E-06	4.92E-06	2.54E-06
**6h**	1.85E-06	1.28E-06	1.08E-06	1.03E-06
**12h**	8.81E-07	1.47E-06	7.79E-07	5.46E-06
**24h**	1.13E-06	8.62E-07	3.45E-06	2.27E-06
**36h**	1.46E-06	2.20E-06	1.89E-06	3.59E-06
**48h**	1.52E-06	1.09E-06	1.98E-06	2.45E-06
**72h**	5.25E-07	6.09E-07	4.95E-07	9.28E-07
	cgd1_1580			
**2h**	1.68E-04	1.01E-04	1.35E-03	5.54E-05
**6h**	4.54E-05	2.43E-05	3.52E-05	1.95E-05
**12h**	4.63E-05	3.32E-05	1.88E-04	1.20E-04
**24h**	1.49E-04	7.10E-05	3.87E-04	9.02E-05
**36h**	1.12E-05	3.45E-05	1.32E-04	7.56E-05
**48h**	2.19E-05	1.63E-05	8.81E-05	4.70E-05
**72h**	2.83E-06	6.09E-06	2.47E-05	6.72E-06
	cgd1_1610			
**2h**	4.11E-05	1.35E-05	6.29E-05	1.21E-05
**6h**	3.01E-06	9.41E-07	4.85E-05	0.00E+00
**12h**	0.00E+00	0.00E+00	8.96E-06	6.98E-07
**24h**	4.68E-06	3.80E-06	1.03E-04	1.55E-05
**36h**	2.67E-06	1.27E-05	1.64E-04	2.82E-05
**48h**	5.72E-05	4.17E-05	5.53E-04	1.91E-04
**72h**	3.69E-05	4.00E-05	1.64E-04	5.27E-04
	cgd1_1630			
**2h**	1.36E-05	1.01E-05	8.77E-06	8.71E-06
**6h**	4.17E-06	1.64E-06	1.22E-06	2.88E-06
**12h**	6.65E-07	6.03E-07	1.86E-06	4.63E-06
**24h**	1.09E-06	1.24E-06	1.96E-06	5.59E-06
**36h**	7.98E-07	2.37E-06	1.25E-06	2.27E-06
**48h**	6.02E-07	4.61E-07	5.90E-07	1.64E-06
**72h**	3.72E-07	2.33E-07	5.00E-07	4.55E-07
	cgd1_1640			
**2h**	1.20E-06	7.32E-06	7.98E-06	3.89E-08
**6h**	5.07E-06	4.95E-06	4.29E-06	2.47E-07
**12h**	1.76E-07	2.16E-07	3.07E-07	4.22E-07
**24h**	1.34E-06	6.66E-07	1.65E-06	2.02E-06

**36h**	2.91E-07	1.34E-06	9.77E-07	7.79E-07
**48h**	2.52E-06	1.33E-06	1.86E-06	4.01E-07
**72h**	2.04E-07	1.74E-07	2.12E-07	1.65E-07
	cgd1_1650			
**2h**	2.71E-06	4.96E-05	3.90E-05	8.81E-06
**6h**	2.45E-04	9.97E-05	4.29E-06	0.00E+00
**12h**	1.10E-05	4.21E-05	8.99E-04	1.92E-05
**24h**	1.41E-04	1.17E-05	6.72E-05	6.27E-06
**36h**	1.71E-04	6.86E-05	3.89E-04	8.45E-06
**48h**	2.78E-03	5.76E-05	1.39E-03	2.52E-05
**72h**	9.40E-04	1.46E-05	2.48E-03	1.15E-05
	cgd1_1670			
**2h**	2.49E-03	4.86E-05	3.12E-03	1.45E-05
**6h**	1.70E-03	2.63E-04	3.24E-04	9.97E-06
**12h**	4.72E-03	1.39E-04	5.57E-04	2.13E-05
**24h**	9.33E-04	1.90E-04	4.55E-05	3.40E-05
**36h**	1.37E-03	3.76E-05	8.72E-04	6.58E-06
**48h**	1.69E-03	3.11E-05	1.47E-04	8.12E-06
**72h**	2.38E-03	3.73E-05	7.56E-04	2.33E-05
	cgd1_1680			
**2h**	1.27E-08	0.00E+00	0.00E+00	0.00E+00
**6h**	0.00E+00	1.81E-09	2.43E-09	9.91E-10
**12h**	0.00E+00	1.35E-09	1.32E-09	0.00E+00
**24h**	6.30E-10	3.86E-09	5.81E-09	1.91E-08
**36h**	1.65E-08	2.44E-08	3.98E-08	4.38E-08
**48h**	5.51E-07	3.28E-07	5.43E-07	8.99E-07
**72h**	1.12E-06	6.59E-07	2.25E-06	5.35E-07
	cgd1_1690			
**2h**	0.00E+00	5.99E-09	0.00E+00	1.83E-09
**6h**	2.69E-09	0.00E+00	6.45E-10	0.00E+00
**12h**	1.54E-09	0.00E+00	0.00E+00	0.00E+00
**24h**	2.94E-10	7.17E-09	2.01E-09	4.02E-10
**36h**	1.24E-09	1.62E-08	2.53E-08	2.90E-09
**48h**	1.55E-08	1.33E-08	9.43E-09	1.22E-08
**72h**	1.28E-07	4.65E-08	3.43E-07	1.15E-07
	cgd1_1700			
**2h**	4.40E-07	7.40E-07	1.65E-07	3.23E-09
**6h**	2.83E-06	2.15E-05	7.18E-07	8.43E-07
**12h**	5.39E-06	5.43E-05	5.94E-06	8.47E-05
**24h**	8.55E-07	3.55E-05	2.25E-06	9.61E-05
**36h**	8.06E-06	4.10E-05	2.96E-05	3.07E-04
**48h**	9.56E-06	2.42E-05	7.46E-06	2.01E-04
**72h**	1.07E-05	3.20E-05	1.01E-05	8.59E-05
	cgd1_1710			
**2h**	4.23E-07	2.90E-07	2.80E-07	1.29E-07
**6h**	3.94E-07	1.40E-06	8.75E-07	5.71E-07
**12h**	7.52E-08	1.48E-07	4.68E-07	1.25E-06
**24h**	1.92E-07	1.37E-06	4.69E-07	3.20E-05
**36h**	1.63E-06	2.18E-06	3.23E-06	1.06E-05
**48h**	1.24E-06	1.06E-06	1.16E-06	1.23E-05
**72h**	7.80E-07	5.72E-07	1.22E-06	2.89E-06
	cgd1_1740			
**2h**	0.00E+00	0.00E+00	0.00E+00	1.49E-08

**6h**	4.48E-07	3.88E-07	2.73E-07	8.40E-08
**12h**	2.64E-07	1.37E-07	3.89E-07	1.31E-06
**24h**	1.09E-07	3.54E-07	9.64E-08	3.51E-06
**36h**	5.60E-07	7.04E-07	1.10E-06	2.09E-06
**48h**	6.75E-07	3.32E-07	4.64E-07	1.96E-06
**72h**	6.39E-07	1.04E-07	2.37E-07	2.35E-07
	cgd1_1750			
**2h**	1.09E-04	1.44E-04	1.25E-03	4.53E-05
**6h**	8.44E-05	6.31E-04	6.79E-04	4.05E-06
**12h**	9.43E-04	5.25E-04	3.64E-03	8.95E-05
**24h**	4.03E-04	4.42E-04	7.58E-04	4.29E-05
**36h**	2.14E-04	2.83E-04	2.71E-03	1.44E-05
**48h**	5.34E-04	9.14E-05	1.73E-03	2.27E-05
**72h**	7.83E-04	1.01E-04	9.49E-04	1.01E-05
	cgd1_1760			
**2h**	6.89E-07	2.19E-06	2.45E-07	6.17E-08
**6h**	1.02E-06	5.55E-07	3.62E-07	6.68E-08
**12h**	4.98E-07	2.64E-07	1.07E-06	1.63E-06
**24h**	3.52E-07	3.31E-07	1.30E-06	3.00E-06
**36h**	2.71E-07	6.12E-07	1.32E-06	8.85E-07
**48h**	7.78E-07	5.72E-07	1.33E-06	6.86E-07
**72h**	7.83E-08	1.38E-07	4.25E-08	2.26E-08
	cgd1_1780			
**2h**	8.01E-07	2.32E-07	4.75E-07	7.78E-08
**6h**	5.44E-07	6.91E-07	5.72E-07	1.19E-07
**12h**	6.81E-08	1.01E-08	3.34E-07	1.20E-06
**24h**	1.38E-07	1.42E-07	6.05E-07	2.28E-06
**36h**	1.89E-07	5.88E-07	3.79E-07	6.03E-07
**48h**	4.22E-07	3.19E-07	3.38E-07	3.02E-07
**72h**	1.09E-07	8.47E-08	4.75E-08	4.70E-08
	cgd1_1790			
**2h**	7.23E-06	1.68E-05	1.41E-04	1.62E-06
**6h**	9.02E-06	2.59E-05	9.30E-06	8.91E-06
**12h**	2.36E-06	1.68E-05	5.76E-05	6.75E-05
**24h**	6.75E-06	9.03E-06	1.50E-04	1.39E-04
**36h**	1.20E-05	2.22E-05	4.23E-05	1.67E-04
**48h**	7.86E-06	1.40E-05	1.19E-05	8.04E-05
**72h**	1.60E-06	3.19E-06	4.21E-06	6.44E-05
	cgd1_1800			
**2h**	4.87E-07	1.04E-06	1.67E-07	1.30E-07
**6h**	3.20E-06	1.26E-06	4.39E-06	3.75E-06
**12h**	2.75E-08	2.30E-07	1.67E-07	2.82E-07
**24h**	1.15E-06	2.17E-07	1.19E-06	4.45E-06
**36h**	5.07E-07	6.32E-07	4.56E-07	6.37E-07
**48h**	1.37E-06	1.02E-07	1.70E-07	5.07E-07
**72h**	3.38E-08	6.56E-08	6.35E-08	6.28E-08
	cgd1_1810			
**2h**	0.00E+00	0.00E+00	0.00E+00	0.00E+00
**6h**	0.00E+00	0.00E+00	0.00E+00	0.00E+00
**12h**	4.62E-08	5.77E-08	2.36E-08	8.48E-08
**24h**	5.53E-08	2.58E-08	1.06E-07	2.72E-07
**36h**	8.92E-08	1.59E-07	1.30E-07	3.77E-07
**48h**	1.69E-07	2.56E-08	7.63E-08	6.85E-08

**72h**	7.65E-09	6.31E-08	1.39E-08	3.47E-08
	cgd1_1820			
**2h**	2.45E-08	2.49E-09	5.57E-09	0.00E+00
**6h**	0.00E+00	5.36E-09	9.73E-09	0.00E+00
**12h**	4.87E-08	9.95E-08	1.59E-08	1.78E-07
**24h**	9.93E-08	5.03E-08	4.13E-08	4.43E-07
**36h**	9.38E-08	1.52E-07	1.65E-07	3.67E-07
**48h**	3.01E-07	7.72E-08	8.06E-08	1.87E-07
**72h**	1.23E-07	7.87E-08	2.05E-07	2.26E-07
	cgd1_1830			
**2h**	2.69E-02	8.05E-03	3.21E-03	2.09E-02
**6h**	5.45E-03	5.28E-03	2.94E-03	1.57E-02
**12h**	1.44E-03	2.99E-04	7.18E-04	3.85E-03
**24h**	2.55E-03	9.02E-04	1.67E-03	6.83E-03
**36h**	3.18E-03	7.17E-04	5.55E-04	9.66E-04
**48h**	3.47E-03	8.86E-04	8.32E-04	4.74E-03
**72h**	1.21E-03	3.63E-04	4.88E-04	8.73E-04
	cgd1_1840			
**2h**	1.82E-06	5.04E-06	2.67E-06	4.10E-07
**6h**	1.03E-06	8.98E-07	1.20E-06	2.42E-06
**12h**	1.49E-07	2.72E-07	1.26E-06	1.86E-06
**24h**	9.81E-07	6.03E-07	8.16E-07	1.71E-06
**36h**	2.24E-06	1.48E-06	1.41E-06	4.38E-06
**48h**	1.39E-06	3.96E-07	5.14E-07	1.22E-06
**72h**	3.90E-07	1.90E-07	2.65E-07	2.39E-07
	cgd1_1850			
**2h**	8.74E-07	0.00E+00	6.87E-08	1.28E-07
**6h**	5.03E-07	1.27E-06	1.94E-06	1.54E-05
**12h**	1.49E-06	1.41E-06	8.44E-07	3.87E-06
**24h**	4.29E-07	9.31E-07	1.46E-06	1.45E-05
**36h**	3.13E-06	2.51E-06	3.83E-06	8.03E-06
**48h**	2.61E-06	2.46E-06	1.53E-06	3.19E-05
**72h**	6.41E-07	1.08E-06	5.03E-07	1.91E-06
	cgd1_1860			
**2h**	1.23E-06	4.92E-07	5.97E-06	0.00E+00
**6h**	6.90E-05	4.37E-05	5.74E-05	1.68E-03
**12h**	7.17E-07	1.16E-05	1.63E-06	3.69E-07
**24h**	1.08E-05	8.66E-06	1.87E-05	4.48E-04
**36h**	4.98E-05	1.90E-05	1.53E-05	2.26E-04
**48h**	4.17E-04	8.37E-06	1.41E-05	1.47E-04
**72h**	8.96E-06	3.12E-06	5.62E-06	6.98E-05
	cgd1_1870			
**2h**	0.00E+00	2.44E-07	0.00E+00	0.00E+00
**6h**	0.00E+00	0.00E+00	5.78E-08	0.00E+00
**12h**	3.48E-04	1.58E-04	1.86E-05	3.92E-04
**24h**	1.25E-05	1.24E-05	6.75E-06	3.37E-05
**36h**	1.43E-05	6.30E-06	1.44E-05	1.40E-04
**48h**	9.09E-06	2.34E-06	4.06E-06	2.89E-05
**72h**	6.00E-05	3.00E-06	2.90E-06	6.33E-06
	cgd1_1880			
**2h**	0.00E+00	0.00E+00	8.61E-09	4.71E-09
**6h**	0.00E+00	0.00E+00	0.00E+00	0.00E+00
**12h**	0.00E+00	0.00E+00	7.97E-09	1.33E-07

**24h**	4.70E-09	5.22E-09	1.95E-09	1.84E-09
**36h**	4.80E-08	7.49E-09	2.23E-08	2.20E-07
**48h**	1.12E-07	8.95E-08	7.88E-08	4.48E-07
**72h**	5.78E-08	1.38E-08	1.59E-08	3.56E-08
	cgd1_1890			
**2h**	0.00E+00	0.00E+00	0.00E+00	0.00E+00
**6h**	1.15E-07	6.65E-08	7.31E-08	1.07E-07
**12h**	5.63E-08	1.26E-08	2.17E-08	2.22E-08
**24h**	3.61E-08	8.34E-08	2.84E-07	6.43E-07
**36h**	2.04E-07	1.43E-07	1.43E-07	2.72E-07
**48h**	4.05E-07	2.15E-07	2.52E-07	5.26E-07
**72h**	3.97E-08	2.64E-08	1.94E-08	5.41E-08
	cgd1_1910			
**2h**	1.12E-08	5.80E-08	0.00E+00	1.39E-08
**6h**	0.00E+00	3.84E-08	1.48E-09	9.09E-09
**12h**	2.26E-08	0.00E+00	9.16E-09	2.08E-08
**24h**	4.44E-08	3.21E-08	3.77E-08	1.03E-07
**36h**	3.96E-08	2.55E-08	4.68E-08	7.38E-08
**48h**	2.24E-06	5.27E-07	3.24E-07	2.04E-06
**72h**	2.84E-06	4.98E-07	4.94E-07	3.73E-07
	cgd1_1980			
**2h**	3.90E-05	3.83E-06	5.25E-05	8.48E-06
**6h**	2.93E-06	1.62E-05	9.24E-06	6.21E-06
**12h**	5.48E-07	4.40E-07	1.01E-06	3.97E-06
**24h**	1.62E-06	1.02E-06	2.88E-06	1.19E-05
**36h**	2.91E-06	1.07E-06	1.36E-06	4.61E-06
**48h**	1.68E-06	3.74E-07	7.65E-07	1.78E-06
**72h**	8.64E-07	3.38E-07	4.92E-07	1.25E-06
	cgd1_2010			
**2h**	7.88E-07	2.14E-06	0.00E+00	2.93E-06
**6h**	8.33E-06	2.80E-04	5.18E-05	4.05E-05
**12h**	1.18E-06	9.23E-06	5.06E-04	6.04E-06
**24h**	3.04E-06	1.74E-04	1.08E-03	2.18E-05
**36h**	6.75E-06	1.73E-04	2.01E-04	1.58E-05
**48h**	1.41E-05	5.67E-04	5.08E-04	2.80E-05
**72h**	4.53E-06	1.66E-05	1.33E-04	1.63E-05
	cgd1_2170			
**2h**	1.29E-02	1.70E-04	0.00E+00	8.84E-03
**6h**	3.39E-03	2.96E-04	6.07E-05	4.48E-03
**12h**	8.71E-05	6.97E-05	4.02E-05	1.41E-02
**24h**	8.49E-04	7.04E-05	6.12E-05	4.40E-03
**36h**	6.06E-04	1.26E-04	5.78E-05	8.40E-04
**48h**	2.40E-04	6.74E-05	5.45E-05	2.06E-03
**72h**	4.16E-04	1.23E-05	2.33E-05	9.69E-04
	cgd1_2220			
**2h**	2.47E-05	9.61E-06	1.02E-04	2.93E-04
**6h**	2.06E-05	8.66E-05	1.20E-04	3.57E-04
**12h**	3.92E-06	5.41E-06	2.32E-05	9.17E-05
**24h**	4.08E-05	5.13E-05	3.95E-05	3.91E-04
**36h**	2.05E-05	1.66E-05	2.82E-05	3.17E-04
**48h**	7.54E-05	4.84E-05	5.38E-05	1.98E-04
**72h**	1.58E-05	9.48E-06	1.75E-05	1.45E-04
	cgd1_2250			

**2h**	5.46E-04	1.55E-03	5.09E-03	1.14E-04
**6h**	1.14E-03	4.57E-03	6.78E-03	7.54E-05
**12h**	3.42E-04	2.28E-04	0.00E+00	1.85E-05
**24h**	8.24E-04	4.06E-04	2.99E-04	7.58E-05
**36h**	7.35E-04	1.45E-04	2.38E-04	2.95E-05
**48h**	4.85E-04	3.11E-04	1.40E-04	5.90E-05
**72h**	1.08E-04	8.16E-05	5.64E-04	9.60E-05
	cgd1_2260			
**2h**	2.35E-07	7.55E-07	5.14E-06	4.09E-07
**6h**	1.07E-06	1.02E-06	1.23E-06	1.33E-06
**12h**	2.36E-07	3.00E-07	2.24E-07	4.44E-07
**24h**	9.61E-07	2.26E-07	6.04E-07	3.60E-06
**36h**	5.05E-07	3.58E-07	5.16E-07	6.73E-07
**48h**	4.51E-07	7.55E-08	6.71E-08	4.39E-07
**72h**	2.81E-07	2.24E-07	4.13E-07	4.57E-07
	cgd1_2280			
**2h**	1.90E-05	0.00E+00	2.09E-05	0.00E+00
**6h**	1.84E-05	7.12E-06	2.15E-05	0.00E+00
**12h**	8.45E-06	2.57E-06	1.67E-05	0.00E+00
**24h**	1.61E-06	2.94E-06	1.12E-06	3.16E-05
**36h**	4.64E-06	2.67E-06	5.55E-06	1.01E-05
**48h**	2.35E-06	1.51E-06	1.47E-06	2.07E-05
**72h**	4.47E-06	1.87E-06	3.82E-06	1.13E-04
	cgd1_2290			
**2h**	5.57E-09	3.40E-08	4.09E-07	0.00E+00
**6h**	3.66E-07	3.93E-07	4.58E-07	4.27E-07
**12h**	2.21E-06	3.86E-06	1.48E-05	2.02E-06
**24h**	1.10E-06	6.07E-07	5.04E-07	3.36E-06
**36h**	2.49E-06	4.17E-06	3.22E-06	5.40E-06
**48h**	4.68E-07	1.08E-06	4.53E-07	2.04E-06
**72h**	7.52E-07	4.29E-07	2.02E-07	4.47E-07
	cgd1_2300			
**2h**	1.14E-06	2.07E-06	3.32E-06	5.21E-06
**6h**	4.24E-07	6.12E-07	5.55E-06	4.91E-07
**12h**	1.99E-06	5.25E-06	9.39E-06	3.34E-06
**24h**	1.38E-06	5.83E-07	6.14E-07	3.21E-06
**36h**	9.22E-07	1.99E-06	9.48E-07	3.09E-06
**48h**	2.62E-07	3.49E-07	2.71E-07	7.30E-07
**72h**	2.32E-07	1.69E-07	1.55E-07	3.51E-07
	cgd1_2320			
**2h**	9.45E-08	6.13E-08	6.48E-07	9.00E-09
**6h**	1.65E-08	2.00E-08	2.98E-08	0.00E+00
**12h**	1.29E-05	7.36E-06	6.00E-05	6.84E-06
**24h**	1.33E-06	7.12E-07	1.86E-06	6.91E-06
**36h**	1.69E-05	1.02E-05	2.41E-06	1.52E-05
**48h**	4.32E-06	4.42E-06	4.19E-06	9.14E-06
**72h**	1.21E-06	2.88E-07	1.93E-07	3.28E-07
	cgd1_2330			
**2h**	0.00E+00	1.08E-07	6.12E-07	1.09E-08
**6h**	1.16E-07	2.76E-07	1.91E-07	1.20E-07
**12h**	3.58E-08	2.93E-08	6.39E-07	3.76E-08
**24h**	4.89E-08	1.34E-07	4.65E-08	5.93E-07
**36h**	4.91E-07	3.52E-07	3.03E-07	3.73E-07

**48h**	3.37E-07	3.42E-07	3.02E-07	5.02E-07
**72h**	2.60E-07	9.47E-08	1.40E-07	1.62E-07
	cgd1_2350			
**2h**	2.46E-06	5.04E-06	6.78E-06	6.69E-06
**6h**	2.50E-06	2.21E-06	6.54E-06	2.70E-05
**12h**	2.71E-06	3.10E-06	1.29E-05	1.30E-05
**24h**	2.33E-06	4.23E-06	4.73E-06	6.27E-05
**36h**	3.68E-06	1.67E-06	1.48E-06	1.06E-05
**48h**	2.94E-06	3.56E-06	1.84E-06	1.73E-05
**72h**	6.77E-07	6.63E-07	4.27E-07	1.76E-06
	cgd1_2360			
**2h**	6.03E-07	7.00E-07	5.00E-06	4.98E-07
**6h**	6.17E-07	1.23E-06	4.48E-06	6.38E-07
**12h**	2.54E-07	3.08E-07	3.67E-07	1.39E-07
**24h**	6.08E-07	1.83E-06	3.26E-07	3.86E-06
**36h**	1.34E-06	5.28E-07	3.47E-07	5.89E-07
**48h**	1.30E-06	7.75E-07	6.17E-07	7.32E-07
**72h**	2.54E-07	6.94E-08	8.16E-08	7.63E-08
	cgd1_2370			
**2h**	7.56E-07	3.94E-07	2.64E-06	3.59E-08
**6h**	6.42E-07	6.95E-07	2.03E-05	7.34E-05
**12h**	2.49E-07	2.80E-07	0.00E+00	2.81E-06
**24h**	2.42E-06	2.27E-06	2.40E-06	8.30E-05
**36h**	6.93E-06	1.32E-06	1.86E-06	4.63E-05
**48h**	1.50E-05	2.76E-06	5.93E-05	4.85E-05
**72h**	1.69E-06	1.06E-06	6.43E-06	6.73E-05
	cgd1_2400			
**2h**	9.32E-04	7.48E-03	5.13E-03	4.28E-04
**6h**	1.75E-04	3.79E-04	2.51E-04	2.83E-04
**12h**	1.15E-04	5.75E-04	5.09E-04	9.10E-04
**24h**	8.36E-04	1.78E-03	9.29E-04	3.31E-04
**36h**	1.14E-04	7.71E-04	5.35E-04	1.42E-04
**48h**	2.63E-04	3.14E-04	3.41E-04	1.57E-04
**72h**	1.97E-05	1.15E-04	8.06E-05	3.25E-05
	cgd1_2420			
**2h**	8.34E-06	7.75E-06	3.16E-05	1.49E-05
**6h**	1.66E-05	8.51E-06	6.43E-06	2.88E-05
**12h**	1.65E-06	1.22E-05	2.77E-05	1.15E-05
**24h**	1.31E-05	2.90E-05	4.56E-05	2.18E-05
**36h**	3.39E-06	1.52E-05	1.72E-05	3.93E-06
**48h**	9.38E-06	1.04E-05	1.22E-05	7.06E-06
**72h**	2.15E-06	8.42E-06	1.16E-05	2.89E-06
	cgd1_2430			
**2h**	4.77E-07	5.33E-07	2.48E-06	2.40E-06
**6h**	8.17E-07	6.23E-07	1.06E-06	6.07E-06
**12h**	1.17E-07	3.88E-07	3.41E-07	1.63E-06
**24h**	1.48E-06	1.52E-06	2.07E-06	2.39E-06
**36h**	5.41E-07	9.57E-07	1.31E-06	8.77E-07
**48h**	1.21E-06	8.59E-07	5.37E-07	6.58E-07
**72h**	1.75E-07	3.77E-06	3.96E-07	6.69E-08
	cgd1_2460			
**2h**	5.69E-06	2.56E-06	2.22E-06	6.26E-06
**6h**	3.93E-06	1.16E-06	1.06E-06	2.93E-06

**12h**	1.10E-06	6.69E-06	1.70E-06	1.22E-05
**24h**	3.06E-06	3.30E-06	2.78E-06	3.15E-06
**36h**	1.43E-06	2.35E-06	2.32E-06	5.38E-06
**48h**	2.22E-06	1.29E-06	1.49E-06	2.36E-06
**72h**	6.90E-07	6.30E-07	6.56E-07	1.24E-06
	cgd1_2470			
**2h**	1.01E-06	2.23E-06	1.69E-06	1.33E-06
**6h**	6.99E-07	5.66E-07	1.62E-06	1.14E-06
**12h**	5.23E-08	6.80E-08	1.12E-07	1.07E-06
**24h**	5.69E-07	2.32E-06	1.62E-06	5.15E-07
**36h**	4.86E-07	9.73E-07	2.45E-06	1.86E-06
**48h**	5.34E-07	7.81E-07	9.77E-07	4.38E-07
**72h**	3.06E-07	6.80E-07	3.21E-06	3.43E-07
	cgd1_2490			
**2h**	4.68E-06	5.39E-05	2.65E-06	1.50E-07
**6h**	3.16E-05	2.63E-04	3.80E-04	1.15E-04
**12h**	3.27E-06	1.61E-04	6.26E-05	2.24E-05
**24h**	6.42E-06	1.96E-04	5.37E-04	6.35E-05
**36h**	2.87E-05	3.83E-04	3.17E-04	9.35E-05
**48h**	1.48E-05	1.55E-04	1.39E-04	3.78E-05
**72h**	7.62E-06	6.22E-05	6.18E-05	4.37E-06
	cgd1_2510			
**2h**	3.09E-07	1.27E-07	3.93E-07	3.22E-08
**6h**	5.75E-07	3.76E-07	1.61E-07	1.81E-06
**12h**	1.75E-07	5.68E-07	8.97E-07	3.46E-06
**24h**	2.99E-07	1.23E-06	9.73E-07	8.50E-07
**36h**	7.89E-07	9.11E-07	1.53E-06	2.56E-06
**48h**	1.19E-07	4.02E-07	5.45E-07	3.49E-07
**72h**	8.92E-07	5.37E-07	4.53E-07	3.98E-07
	cgd1_2530			
**2h**	3.12E-08	7.15E-08	7.45E-08	1.11E-07
**6h**	1.93E-07	1.88E-07	1.36E-07	2.59E-07
**12h**	1.59E-06	3.67E-06	5.58E-06	1.60E-05
**24h**	6.12E-07	4.15E-06	2.08E-06	1.92E-06
**36h**	2.71E-06	3.20E-06	9.17E-06	1.19E-05
**48h**	1.49E-06	2.08E-06	2.34E-06	2.53E-06
**72h**	2.80E-06	9.47E-07	2.01E-06	1.13E-06
	cgd1_2540			
**2h**	8.88E-09	6.87E-09	4.68E-08	0.00E+00
**6h**	1.13E-08	2.90E-08	7.01E-08	1.66E-07
**12h**	4.40E-07	4.64E-07	1.34E-06	5.47E-06
**24h**	1.17E-07	8.92E-07	8.46E-07	3.69E-07
**36h**	7.25E-07	3.03E-06	2.76E-06	2.45E-06
**48h**	5.25E-07	8.10E-07	1.01E-06	1.31E-06
**72h**	9.45E-07	7.82E-07	1.34E-06	8.70E-07
	cgd1_2580			
**2h**	6.81E-09	2.96E-08	1.20E-08	0.00E+00
**6h**	1.89E-08	7.92E-09	7.17E-09	3.98E-08
**12h**	1.52E-07	4.01E-08	1.96E-07	4.66E-07
**24h**	2.72E-08	6.43E-08	8.03E-08	1.41E-07
**36h**	1.38E-07	3.39E-07	4.00E-07	3.21E-07
**48h**	1.17E-07	1.39E-07	8.63E-08	1.63E-07
**72h**	1.08E-07	6.84E-08	7.87E-08	5.11E-09

	cgd1_2590			
**2h**	4.01E-07	1.31E-07	7.32E-07	1.14E-07
**6h**	5.02E-07	5.14E-07	6.17E-07	3.70E-06
**12h**	2.60E-07	2.56E-07	3.70E-07	2.36E-06
**24h**	3.67E-07	4.06E-07	1.32E-06	4.02E-06
**36h**	9.53E-07	8.27E-07	1.52E-06	1.41E-06
**48h**	8.97E-07	5.76E-07	7.74E-07	1.02E-06
**72h**	2.40E-07	2.41E-07	3.19E-07	1.85E-07
	cgd1_2600			
**2h**	7.30E-06	2.07E-05	1.09E-04	6.21E-06
**6h**	1.56E-06	5.21E-06	3.45E-06	1.88E-05
**12h**	2.13E-06	2.63E-06	8.96E-06	5.65E-06
**24h**	2.44E-06	9.75E-06	2.77E-05	2.15E-05
**36h**	6.52E-06	2.14E-05	2.19E-05	1.05E-05
**48h**	1.49E-06	8.22E-06	6.87E-06	3.17E-06
**72h**	3.92E-07	1.18E-05	4.95E-06	1.26E-06
	cgd1_3750			
**2h**	3.78E-07	1.55E-06	3.87E-07	1.75E-06
**6h**	5.99E-06	9.24E-06	1.86E-05	9.12E-06
**12h**	1.65E-08	3.67E-08	1.44E-06	1.54E-07
**24h**	1.19E-06	1.04E-05	1.83E-05	1.26E-06
**36h**	4.02E-07	2.52E-06	3.81E-06	3.70E-07
**48h**	1.57E-06	3.05E-06	5.54E-06	4.35E-07
**72h**	1.31E-07	7.82E-07	6.73E-07	5.86E-08
	cgd1_3790			
**2h**	1.48E-04	1.09E-04	7.01E-05	4.07E-06
**6h**	1.39E-05	3.80E-05	1.67E-05	4.01E-05
**12h**	1.54E-02	1.76E-03	5.38E-04	3.89E-03
**24h**	1.80E-03	9.02E-04	5.55E-04	2.74E-04
**36h**	1.42E-03	7.49E-04	9.81E-04	1.60E-03
**48h**	7.25E-04	3.08E-04	1.40E-04	2.93E-04
**72h**	2.20E-04	1.42E-04	2.80E-05	4.69E-05
	cgd1_3820			
**2h**	1.58E-06	1.20E-06	3.24E-06	1.38E-06
**6h**	5.24E-07	2.03E-06	6.09E-07	1.39E-06
**12h**	1.11E-06	1.17E-06	2.19E-05	4.06E-06
**24h**	1.82E-06	6.24E-06	2.06E-05	2.96E-06
**36h**	4.41E-06	1.08E-05	3.90E-05	1.76E-05
**48h**	2.80E-06	3.85E-06	1.19E-05	2.62E-06
**72h**	1.73E-06	1.65E-06	3.68E-06	4.74E-07
	cgd1_3830			
**2h**	5.38E-09	8.55E-10	2.73E-09	8.78E-08
**6h**	2.55E-08	0.00E+00	0.00E+00	2.13E-09
**12h**	2.71E-09	0.00E+00	5.65E-10	3.70E-09
**24h**	6.09E-09	4.60E-09	1.98E-09	4.15E-09
**36h**	2.36E-08	2.27E-08	3.01E-08	1.94E-08
**48h**	1.37E-07	2.65E-07	2.25E-07	2.37E-07
**72h**	6.76E-08	3.35E-08	4.04E-09	3.01E-09
	cgd1_3840			
**2h**	2.08E-06	5.70E-06	2.01E-05	9.40E-06
**6h**	3.91E-06	4.60E-06	1.02E-06	6.74E-06
**12h**	5.30E-06	2.54E-06	8.57E-06	4.32E-06
**24h**	3.30E-06	7.08E-06	1.67E-05	4.63E-06

**36h**	2.16E-06	5.60E-06	6.45E-06	1.92E-06
**48h**	1.22E-06	7.35E-06	6.10E-06	2.39E-06
**72h**	1.76E-06	1.02E-06	4.11E-07	1.54E-07
	cgd5_280			
**2h**	8.81E-09	1.02E-08	5.79E-09	0.00E+00
**6h**	2.08E-08	1.08E-08	6.54E-09	1.09E-08
**12h**	1.70E-07	1.64E-08	8.66E-09	1.03E-07
**24h**	8.47E-08	1.76E-07	3.46E-07	2.41E-07
**36h**	3.69E-07	2.26E-07	2.60E-07	2.16E-07
**48h**	3.21E-07	4.29E-07	2.77E-07	4.91E-07
**72h**	4.43E-07	1.94E-07	1.04E-07	8.38E-08
	cgd5_290			
**2h**	2.68E-04	4.27E-03	1.42E-03	1.73E-04
**6h**	1.68E-04	7.97E-04	1.23E-03	6.80E-04
**12h**	3.20E-05	1.02E-04	1.11E-03	8.77E-05
**24h**	1.66E-04	5.67E-04	2.80E-03	1.94E-04
**36h**	6.23E-05	1.24E-03	7.10E-04	4.40E-05
**48h**	5.29E-05	2.57E-04	5.01E-04	6.91E-05
**72h**	3.62E-05	2.21E-04	7.06E-05	5.92E-06
	cgd5_410			
**2h**	0.00E+00	0.00E+00	0.00E+00	0.00E+00
**6h**	0.00E+00	0.00E+00	0.00E+00	1.52E-08
**12h**	1.04E-06	5.29E-07	1.50E-05	9.77E-07
**24h**	8.00E-07	7.35E-06	1.05E-04	1.33E-06
**36h**	8.70E-06	4.19E-05	6.72E-05	3.03E-06
**48h**	5.46E-06	1.11E-05	4.08E-05	4.44E-06
**72h**	7.84E-06	1.82E-05	3.12E-05	4.35E-06
	cgd5_420			
**2h**	0.00E+00	0.00E+00	0.00E+00	0.00E+00
**6h**	0.00E+00	2.48E-07	0.00E+00	0.00E+00
**12h**	1.22E-07	0.00E+00	9.72E-09	0.00E+00
**24h**	5.55E-08	1.66E-06	3.58E-08	5.06E-08
**36h**	2.06E-06	1.66E-05	9.45E-07	9.65E-07
**48h**	6.44E-04	4.19E-04	8.70E-05	3.81E-05
**72h**	9.95E-05	3.16E-04	5.89E-05	5.72E-05
	cgd5_450			
**2h**	2.47E-06	1.19E-06	9.16E-07	1.63E-06
**6h**	3.50E-06	4.92E-07	6.08E-06	3.29E-06
**12h**	1.59E-06	4.66E-07	1.22E-06	1.63E-06
**24h**	2.32E-06	1.73E-06	1.58E-06	1.60E-06
**36h**	6.02E-07	1.11E-06	7.17E-07	8.57E-07
**48h**	5.77E-07	5.51E-07	5.53E-07	3.58E-07
**72h**	2.75E-07	3.78E-07	2.49E-07	5.46E-07
	cgd5_490			
**2h**	7.97E-08	2.39E-07	0.00E+00	2.72E-07
**6h**	1.18E-06	5.72E-07	4.44E-07	1.12E-06
**12h**	9.69E-08	3.16E-08	3.14E-08	4.17E-08
**24h**	2.69E-07	3.59E-07	6.13E-07	5.10E-07
**36h**	4.28E-07	1.50E-06	2.28E-07	1.65E-07
**48h**	1.28E-07	8.45E-07	1.55E-07	1.39E-07
**72h**	2.37E-07	2.65E-07	8.80E-08	2.01E-07
	cgd5_510			
**2h**	7.19E-05	2.81E-05	1.73E-06	5.59E-06

**6h**	4.22E-05	2.97E-05	4.30E-05	4.03E-05
**12h**	2.25E-06	1.01E-06	2.91E-06	4.37E-06
**24h**	6.01E-06	2.89E-05	1.30E-05	9.61E-06
**36h**	5.87E-06	2.63E-05	5.54E-06	3.54E-06
**48h**	2.99E-06	7.15E-06	2.01E-06	2.67E-06
**72h**	8.72E-07	1.79E-06	2.06E-07	3.49E-07
	cgd5_520			
**2h**	8.91E-06	1.12E-05	3.37E-06	5.25E-06
**6h**	2.57E-06	5.92E-06	1.04E-06	1.43E-06
**12h**	8.17E-07	1.59E-07	2.56E-06	1.12E-06
**24h**	8.11E-07	2.71E-06	7.76E-07	3.95E-07
**36h**	9.19E-07	3.52E-06	1.61E-06	8.55E-07
**48h**	1.75E-07	1.36E-06	2.09E-07	2.18E-07
**72h**	2.68E-07	2.83E-07	2.04E-07	2.87E-07
	cgd5_530			
**2h**	1.07E-06	2.87E-07	2.36E-07	1.11E-06
**6h**	2.81E-07	1.92E-07	7.89E-07	4.63E-07
**12h**	3.76E-07	1.47E-07	1.72E-07	2.78E-07
**24h**	2.93E-07	1.92E-07	2.82E-07	1.90E-07
**36h**	4.65E-07	3.65E-07	3.73E-07	3.95E-07
**48h**	1.58E-07	1.30E-07	1.25E-07	1.54E-07
**72h**	1.49E-07	6.65E-08	5.99E-08	1.21E-07
	cgd5_550			
**2h**	4.55E-05	9.14E-05	0.00E+00	5.67E-06
**6h**	4.46E-04	6.49E-04	6.69E-04	2.24E-04
**12h**	4.72E-05	1.59E-04	4.32E-05	5.65E-05
**24h**	8.83E-05	6.36E-04	1.50E-04	6.13E-05
**36h**	1.56E-04	4.26E-04	1.62E-04	8.43E-05
**48h**	8.82E-05	1.44E-04	0.00E+00	4.60E-05
**72h**	8.70E-05	2.45E-04	9.23E-05	2.89E-05
	cgd5_560			
**2h**	0.00E+00	0.00E+00	0.00E+00	0.00E+00
**6h**	0.00E+00	0.00E+00	0.00E+00	0.00E+00
**12h**	9.98E-05	1.78E-04	9.74E-06	1.44E-05
**24h**	7.42E-06	3.20E-05	5.58E-06	2.74E-06
**36h**	7.13E-06	7.93E-05	2.14E-05	1.50E-05
**48h**	1.91E-05	8.10E-05	2.71E-05	1.45E-05
**72h**	3.66E-05	6.38E-05	2.98E-05	1.23E-05
	cgd5_580			
**2h**	0.00E+00	0.00E+00	0.00E+00	0.00E+00
**6h**	0.00E+00	0.00E+00	0.00E+00	0.00E+00
**12h**	0.00E+00	1.19E-07	5.32E-08	1.30E-07
**24h**	1.67E-07	2.79E-07	9.37E-08	2.75E-08
**36h**	1.35E-07	1.21E-06	2.07E-07	2.23E-07
**48h**	8.03E-07	4.60E-06	8.36E-07	7.88E-07
**72h**	2.35E-07	8.13E-07	3.10E-07	2.18E-07
	cgd5_600			
**2h**	1.64E-07	3.99E-08	0.00E+00	3.71E-08
**6h**	0.00E+00	9.84E-09	0.00E+00	2.19E-09
**12h**	4.86E-08	8.53E-08	3.15E-08	6.19E-08
**24h**	1.01E-07	6.34E-08	1.68E-07	1.05E-07
**36h**	1.02E-07	3.65E-07	9.42E-08	1.13E-07
**48h**	1.88E-07	1.60E-07	1.50E-07	1.52E-07

**72h**	2.10E-08	1.04E-07	1.27E-08	4.38E-08
	cgd5_610			
**2h**	5.35E-06	3.08E-06	8.37E-07	3.89E-06
**6h**	7.12E-06	2.76E-06	1.06E-05	8.44E-06
**12h**	8.73E-07	2.85E-07	1.19E-06	1.44E-06
**24h**	2.33E-06	3.15E-06	5.62E-06	3.83E-06
**36h**	6.71E-07	5.59E-06	1.09E-06	1.16E-06
**48h**	1.12E-06	1.70E-06	1.47E-06	9.18E-07
**72h**	5.54E-08	2.46E-07	5.88E-08	1.15E-07
	cgd5_640			
**2h**	7.58E-05	2.13E-04	7.75E-06	1.63E-05
**6h**	0.00E+00	4.27E-06	0.00E+00	5.14E-06
**12h**	6.00E-05	3.95E-04	3.75E-04	4.38E-05
**24h**	1.05E-04	2.73E-04	4.65E-04	2.34E-05
**36h**	2.95E-04	2.48E-04	9.06E-04	1.25E-04
**48h**	2.90E-04	2.40E-04	3.02E-03	1.16E-04
**72h**	3.10E-04	1.48E-04	1.16E-03	1.79E-04
	cgd5_650			
**2h**	2.94E-07	7.89E-07	3.63E-09	0.00E+00
**6h**	1.53E-07	7.98E-08	2.09E-07	6.47E-07
**12h**	1.88E-06	2.13E-06	3.19E-06	4.91E-07
**24h**	2.45E-06	1.36E-06	2.47E-06	1.15E-06
**36h**	4.08E-07	8.45E-07	2.91E-06	1.78E-06
**48h**	9.30E-07	2.25E-06	5.97E-07	1.54E-06
**72h**	1.00E-07	2.95E-07	1.13E-07	1.25E-07
	cgd5_660			
**2h**	1.46E-07	3.67E-08	0.00E+00	3.26E-08
**6h**	4.53E-07	4.28E-08	4.46E-08	1.47E-07
**12h**	3.81E-08	4.05E-09	6.60E-08	1.27E-08
**24h**	8.62E-08	1.29E-07	1.75E-07	1.41E-07
**36h**	6.56E-08	6.13E-08	1.35E-07	7.69E-08
**48h**	1.39E-07	1.01E-07	5.36E-08	1.40E-07
**72h**	4.90E-08	4.72E-08	3.19E-08	3.04E-08
	cgd5_670			
**2h**	5.57E-06	6.71E-06	8.31E-06	6.30E-06
**6h**	7.31E-06	2.07E-06	4.47E-06	6.14E-06
**12h**	3.19E-07	4.08E-07	4.18E-06	4.59E-07
**24h**	4.19E-06	1.20E-05	1.87E-05	4.94E-06
**36h**	6.86E-07	2.48E-06	3.45E-06	1.39E-06
**48h**	1.19E-06	7.92E-07	8.35E-07	2.04E-06
**72h**	4.39E-07	3.89E-07	1.23E-06	1.17E-05
	cgd5_680			
**2h**	1.53E-05	1.00E-05	1.67E-06	1.28E-05
**6h**	4.42E-05	9.79E-06	2.07E-05	5.77E-05
**12h**	2.52E-06	1.30E-06	2.10E-05	2.76E-06
**24h**	1.08E-05	1.16E-05	4.28E-05	1.96E-05
**36h**	5.82E-06	9.19E-06	9.26E-06	1.38E-05
**48h**	2.82E-06	4.65E-06	3.29E-06	8.75E-06
**72h**	4.46E-06	7.64E-07	1.27E-06	4.85E-06
	cgd5_690			
**2h**	1.44E-07	0.00E+00	3.64E-08	1.19E-07
**6h**	3.16E-07	2.53E-08	7.56E-08	8.56E-08
**12h**	8.64E-07	1.25E-06	5.79E-07	1.13E-07

24h	5.42E-07	4.09E-07	1.38E-06	3.68E-07
36h	1.03E-06	1.11E-06	7.44E-07	9.38E-07
48h	6.65E-07	7.31E-07	6.28E-07	8.34E-07
72h	1.57E-06	2.96E-07	1.33E-07	5.95E-07
	cgd5_700			
2h	3.10E-02	5.16E-03	1.23E-04	3.92E-03
6h	2.51E-02	5.48E-03	4.05E-03	5.06E-03
12h	2.01E-02	7.88E-03	2.34E-03	5.95E-04
24h	1.05E-02	4.33E-03	5.66E-03	7.76E-03
36h	8.92E-03	3.43E-03	9.17E-04	7.87E-03
48h	5.53E-03	1.80E-03	5.71E-04	2.93E-03
72h	2.70E-03	4.84E-04	1.56E-04	8.36E-04
	cgd5_710			
2h	1.56E-07	1.40E-07	2.85E-08	1.48E-07
6h	9.05E-07	6.24E-07	5.11E-07	1.46E-06
12h	2.58E-07	2.51E-07	6.75E-07	1.05E-07
24h	2.89E-07	6.66E-07	4.58E-06	2.42E-06
36h	2.10E-06	1.62E-06	1.84E-06	2.40E-06
48h	6.76E-06	4.05E-06	1.95E-05	1.83E-05
72h	1.70E-05	1.40E-05	1.07E-05	1.65E-05
	cgd5_720			
2h	1.53E-03	3.10E-05	6.53E-06	7.14E-04
6h	7.85E-03	4.64E-05	1.41E-05	5.33E-05
12h	1.64E-03	2.24E-05	4.46E-05	2.94E-05
24h	1.50E-03	7.17E-05	2.84E-05	4.77E-05
36h	1.35E-03	6.38E-05	6.42E-06	1.26E-04
48h	1.07E-03	1.69E-05	8.32E-06	8.23E-05
72h	1.18E-03	6.34E-05	6.89E-06	5.87E-05
	cgd5_730			
2h	4.30E-05	1.42E-05	3.65E-06	2.19E-05
6h	3.66E-06	2.38E-06	3.16E-06	8.74E-06
12h	2.77E-05	4.90E-06	3.33E-05	2.31E-06
24h	2.31E-05	8.63E-06	3.55E-05	3.48E-03
36h	1.02E-05	1.06E-05	8.44E-06	1.43E-05
48h	6.68E-06	3.66E-06	4.09E-06	5.52E-06
72h	2.11E-06	1.81E-06	1.05E-06	1.73E-06
	cgd5_740			
2h	0.00E+00	5.19E-08	0.00E+00	0.00E+00
6h	5.68E-08	3.05E-08	2.65E-08	6.13E-08
12h	1.71E-08	2.73E-09	2.03E-08	6.20E-09
24h	7.96E-08	2.88E-08	3.48E-07	1.28E-07
36h	2.80E-07	1.22E-07	1.04E-07	1.50E-07
48h	2.13E-07	1.53E-07	4.70E-08	3.52E-07
72h	4.88E-08	8.80E-08	4.21E-08	2.61E-08
	cgd5_750			
2h	2.90E-06	1.37E-06	7.64E-07	1.62E-06
6h	2.89E-07	5.98E-08	4.72E-07	2.44E-07
12h	1.12E-05	3.38E-06	9.17E-06	1.20E-06
24h	4.02E-06	7.60E-07	3.69E-06	1.67E-06
36h	1.76E-06	1.14E-06	2.32E-06	2.65E-06
48h	3.99E-06	1.56E-06	4.45E-07	2.57E-06
72h	2.00E-07	4.89E-07	2.83E-07	3.90E-07
	cgd5_760			

**2h**	2.71E-07	5.97E-07	0.00E+00	9.09E-08
**6h**	3.00E-08	3.54E-08	6.03E-08	6.05E-08
**12h**	5.18E-07	1.32E-07	4.70E-07	6.32E-08
**24h**	1.03E-07	5.17E-08	2.83E-07	8.74E-08
**36h**	3.18E-07	1.78E-07	3.10E-07	3.94E-07
**48h**	1.80E-06	9.87E-07	3.19E-07	1.24E-06
**72h**	1.58E-07	1.56E-07	5.17E-07	1.41E-07
	cgd5_770			
**2h**	3.98E-07	2.73E-06	1.77E-07	4.21E-06
**6h**	8.25E-08	3.33E-07	1.82E-06	1.40E-06
**12h**	0.00E+00	0.00E+00	4.59E-07	5.79E-08
**24h**	1.30E-07	1.09E-07	4.33E-07	2.32E-06
**36h**	6.35E-07	6.57E-08	4.66E-07	4.67E-06
**48h**	1.30E-05	5.90E-06	5.95E-06	2.53E-05
**72h**	6.82E-07	6.38E-07	3.21E-07	2.53E-06
	cgd5_780			
**2h**	0.00E+00	0.00E+00	0.00E+00	0.00E+00
**6h**	0.00E+00	0.00E+00	0.00E+00	0.00E+00
**12h**	1.21E-06	8.34E-07	5.57E-06	5.54E-07
**24h**	1.38E-06	4.10E-07	2.21E-06	6.05E-06
**36h**	6.95E-07	6.21E-07	3.12E-06	1.13E-05
**48h**	1.39E-06	6.24E-07	9.35E-07	1.24E-05
**72h**	2.75E-06	2.90E-06	5.41E-06	1.55E-05
	cgd5_790			
**2h**	0.00E+00	0.00E+00	0.00E+00	0.00E+00
**6h**	0.00E+00	0.00E+00	0.00E+00	0.00E+00
**12h**	0.00E+00	0.00E+00	0.00E+00	0.00E+00
**24h**	2.20E-07	1.75E-08	2.44E-08	1.36E-06
**36h**	7.16E-07	8.58E-08	5.76E-07	1.56E-06
**48h**	6.57E-06	9.64E-07	1.77E-06	7.74E-06
**72h**	1.29E-05	3.33E-06	2.99E-06	2.03E-05
	cgd5_800			
**2h**	4.12E-06	3.95E-06	9.48E-07	2.14E-06
**6h**	1.11E-06	7.07E-07	5.17E-07	1.71E-06
**12h**	1.11E-06	1.18E-06	4.47E-06	2.20E-07
**24h**	2.16E-06	6.76E-07	1.30E-06	2.77E-06
**36h**	1.00E-06	5.97E-07	1.42E-06	1.73E-06
**48h**	3.90E-07	1.66E-07	5.04E-07	1.37E-06
**72h**	5.90E-07	7.67E-07	7.26E-07	2.30E-06
	cgd5_810			
**2h**	0.00E+00	0.00E+00	0.00E+00	0.00E+00
**6h**	0.00E+00	0.00E+00	0.00E+00	0.00E+00
**12h**	9.00E-09	3.81E-07	3.60E-07	1.14E-08
**24h**	5.18E-08	6.60E-08	1.24E-07	1.21E-07
**36h**	7.21E-08	1.45E-07	5.05E-07	3.59E-07
**48h**	7.16E-08	9.18E-08	2.62E-07	4.52E-07
**72h**	1.57E-07	1.09E-07	1.12E-07	1.17E-07
	cgd5_820			
**2h**	1.48E-08	1.86E-08	0.00E+00	2.11E-09
**6h**	0.00E+00	0.00E+00	7.47E-09	5.49E-10
**12h**	1.53E-05	2.84E-05	7.20E-06	4.10E-07
**24h**	9.18E-07	1.01E-06	3.54E-07	3.76E-07
**36h**	6.24E-07	2.40E-07	1.63E-06	8.60E-07

**48h**	7.09E-07	4.99E-07	5.91E-07	5.23E-07
**72h**	1.29E-07	1.12E-07	6.29E-08	1.27E-07
	cgd5_830			
**2h**	1.46E-06	7.40E-06	2.25E-07	3.64E-05
**6h**	4.71E-06	1.66E-06	3.80E-06	1.09E-05
**12h**	6.95E-07	9.73E-07	8.79E-07	7.60E-07
**24h**	2.09E-06	1.25E-06	8.38E-06	3.48E-05
**36h**	3.42E-06	7.34E-07	3.68E-06	2.39E-05
**48h**	2.76E-06	9.56E-07	4.29E-06	1.36E-05
**72h**	1.53E-06	6.60E-07	2.91E-07	3.32E-06
	cgd5_840			
**2h**	1.57E-07	2.53E-07	1.06E-07	1.13E-07
**6h**	5.25E-07	6.65E-07	2.34E-06	8.83E-07
**12h**	1.76E-07	2.57E-07	2.64E-07	1.19E-08
**24h**	1.25E-07	3.00E-07	1.34E-06	8.42E-07
**36h**	9.71E-07	1.35E-07	5.64E-07	1.84E-06
**48h**	4.58E-07	7.78E-07	1.31E-06	9.86E-07
**72h**	1.78E-07	8.84E-07	1.74E-07	2.57E-07
	cgd5_870			
**2h**	1.10E-05	4.79E-06	4.23E-07	3.36E-05
**6h**	2.79E-05	2.46E-06	1.64E-05	2.11E-05
**12h**	1.25E-07	1.36E-08	4.13E-06	5.65E-06
**24h**	3.72E-06	8.70E-07	6.89E-06	8.02E-05
**36h**	4.75E-06	1.33E-06	1.29E-05	2.06E-04
**48h**	9.55E-06	2.45E-06	8.34E-06	9.82E-05
**72h**	5.89E-06	1.12E-06	2.24E-06	1.25E-05
	cgd5_880			
**2h**	2.31E-06	2.99E-06	4.91E-07	3.08E-06
**6h**	6.15E-07	1.64E-06	3.48E-06	1.55E-06
**12h**	2.04E-07	6.57E-08	7.44E-07	1.24E-07
**24h**	1.23E-06	1.10E-06	4.46E-06	2.53E-06
**36h**	3.73E-07	7.93E-08	3.04E-07	8.71E-07
**48h**	6.99E-07	1.99E-07	4.83E-07	1.15E-06
**72h**	4.79E-08	5.80E-08	7.97E-08	1.26E-07
	cgd5_890			
**2h**	1.15E-08	0.00E+00	0.00E+00	0.00E+00
**6h**	6.86E-08	1.02E-07	4.55E-07	1.35E-07
**12h**	2.50E-08	0.00E+00	3.22E-08	2.37E-09
**24h**	8.47E-08	7.26E-08	4.40E-07	3.06E-07
**36h**	1.37E-07	4.45E-08	1.05E-07	3.57E-07
**48h**	1.34E-07	1.06E-07	1.33E-07	1.70E-07
**72h**	2.18E-08	1.23E-08	1.66E-08	1.05E-07
	cgd5_900			
**2h**	2.09E-06	3.54E-06	1.04E-05	7.95E-07
**6h**	5.58E-07	6.36E-07	3.53E-06	7.28E-07
**12h**	1.01E-06	5.47E-08	4.97E-07	6.30E-08
**24h**	9.99E-07	6.81E-07	1.59E-06	1.23E-06
**36h**	1.05E-06	1.37E-07	4.76E-07	1.27E-06
**48h**	6.68E-07	1.93E-07	5.50E-07	5.85E-07
**72h**	1.45E-07	1.92E-07	8.04E-07	3.38E-07
	cgd5_1250			
**2h**	2.16E-08	1.30E-08	8.22E-08	0.00E+00
**6h**	6.34E-07	1.19E-06	1.42E-06	4.29E-07

**12h**	4.35E-07	2.96E-07	3.59E-07	1.13E-06
**24h**	2.34E-07	2.99E-07	3.68E-07	1.25E-06
**36h**	2.52E-07	2.35E-06	8.70E-07	1.82E-06
**48h**	3.62E-07	1.83E-07	2.15E-07	8.11E-07
**72h**	6.65E-08	1.28E-07	9.65E-08	1.81E-07
	cgd5_1260			
**2h**	8.44E-06	5.04E-06	2.51E-06	1.71E-06
**6h**	4.93E-05	1.89E-05	1.20E-05	5.04E-05
**12h**	3.96E-07	2.74E-07	1.18E-06	1.13E-06
**24h**	6.55E-06	8.00E-06	1.18E-05	5.41E-05
**36h**	5.13E-06	2.92E-06	7.33E-06	1.57E-05
**48h**	4.25E-06	3.98E-06	2.62E-06	2.12E-05
**72h**	1.22E-06	1.97E-06	1.02E-06	9.04E-06
	cgd5_1270			
**2h**	1.58E-05	2.29E-05	1.46E-05	1.06E-05
**6h**	5.83E-05	3.88E-05	4.17E-05	1.14E-04
**12h**	5.27E-06	3.64E-06	1.11E-05	2.43E-05
**24h**	1.31E-05	1.16E-05	1.99E-05	9.39E-05
**36h**	1.19E-05	6.62E-06	2.60E-05	3.23E-05
**48h**	5.72E-06	5.31E-06	3.75E-06	2.62E-05
**72h**	3.13E-06	1.87E-06	1.42E-06	5.81E-06
	cgd5_1280			
**2h**	3.20E-07	2.56E-07	9.94E-07	5.85E-08
**6h**	9.17E-07	3.93E-07	3.03E-07	5.05E-07
**12h**	2.58E-08	2.32E-08	9.41E-08	8.43E-08
**24h**	6.24E-08	1.28E-07	2.28E-07	3.95E-07
**36h**	2.92E-07	3.28E-07	5.90E-07	3.25E-07
**48h**	1.09E-07	5.70E-08	8.83E-08	2.26E-07
**72h**	8.07E-08	2.28E-07	4.27E-08	6.08E-08
	cgd5_1320			
**2h**	5.45E-06	4.69E-06	1.07E-05	1.39E-05
**6h**	5.78E-06	9.98E-06	6.58E-06	5.00E-05
**12h**	1.67E-06	4.25E-07	1.05E-05	1.28E-05
**24h**	2.55E-06	1.34E-06	2.78E-06	1.65E-05
**36h**	3.44E-06	3.62E-06	7.50E-06	9.89E-06
**48h**	1.57E-06	8.89E-07	7.24E-07	3.48E-06
**72h**	4.07E-07	6.64E-07	3.02E-07	6.97E-07
	cgd5_1340			
**2h**	5.04E-04	2.31E-04	5.91E-04	9.90E-04
**6h**	9.05E-05	4.80E-05	7.13E-05	3.94E-04
**12h**	9.33E-05	4.35E-05	1.79E-04	1.45E-03
**24h**	1.18E-04	1.19E-04	5.18E-05	5.40E-04
**36h**	1.02E-04	6.42E-05	1.01E-04	3.17E-04
**48h**	8.07E-05	3.15E-05	3.66E-05	3.21E-04
**72h**	3.31E-05	4.62E-05	1.87E-05	1.07E-04
	cgd5_1350			
**2h**	1.81E-05	1.47E-05	1.36E-05	1.86E-05
**6h**	6.61E-06	1.49E-06	3.33E-06	9.25E-06
**12h**	3.17E-06	2.20E-06	3.71E-06	2.94E-05
**24h**	1.95E-06	1.67E-06	2.17E-06	3.85E-06
**36h**	3.52E-06	3.13E-06	3.63E-06	5.94E-06
**48h**	1.44E-06	1.05E-06	1.57E-06	3.25E-06
**72h**	4.61E-07	9.71E-07	3.36E-07	3.35E-06

	cgd5_1360			
**2h**	9.03E-05	4.47E-05	5.25E-05	4.62E-05
**6h**	2.82E-03	6.19E-05	1.31E-04	7.65E-04
**12h**	6.81E-06	1.63E-06	1.71E-05	1.75E-04
**24h**	1.71E-05	3.66E-05	8.54E-05	7.62E-04
**36h**	6.50E-06	5.22E-05	3.34E-05	2.29E-04
**48h**	7.67E-04	2.24E-05	2.19E-05	2.80E-04
**72h**	1.45E-05	7.03E-05	5.83E-06	2.17E-04
	cgd5_1370			
**2h**	1.54E-05	1.14E-05	1.05E-05	4.33E-06
**6h**	4.42E-06	1.90E-06	1.68E-06	4.02E-06
**12h**	3.21E-07	1.59E-06	1.01E-06	3.96E-06
**24h**	1.14E-06	1.04E-05	1.35E-06	2.82E-06
**36h**	1.14E-06	1.44E-06	3.54E-06	1.93E-06
**48h**	1.02E-05	4.51E-06	9.96E-06	1.88E-05
**72h**	6.23E-07	1.65E-06	6.28E-07	1.14E-05
	cgd5_1380			
**2h**	3.67E-05	2.13E-05	1.99E-05	1.13E-05
**6h**	5.84E-06	2.39E-06	4.11E-06	2.21E-06
**12h**	5.94E-07	1.84E-07	3.13E-06	1.56E-05
**24h**	7.50E-06	4.23E-06	3.13E-06	1.23E-05
**36h**	1.29E-06	1.40E-06	1.28E-06	4.72E-06
**48h**	3.81E-06	2.45E-06	3.82E-06	1.45E-05
**72h**	3.46E-07	9.41E-07	3.58E-07	3.03E-06
	cgd5_1410			
**2h**	1.36E-07	3.94E-07	2.09E-07	3.83E-08
**6h**	3.07E-06	1.13E-06	7.72E-07	4.94E-07
**12h**	1.23E-07	8.78E-09	3.00E-07	1.86E-06
**24h**	4.19E-07	9.84E-07	4.02E-07	1.24E-06
**36h**	3.24E-07	1.95E-07	3.07E-07	1.21E-06
**48h**	3.37E-07	2.20E-07	2.03E-07	3.98E-07
**72h**	4.95E-09	4.14E-08	1.25E-08	2.25E-07
	cgd5_1430			
**2h**	2.28E-06	0.00E+00	4.51E-07	0.00E+00
**6h**	0.00E+00	0.00E+00	1.95E-07	1.51E-06
**12h**	8.99E-07	0.00E+00	1.26E-08	0.00E+00
**24h**	5.21E-07	6.06E-08	3.75E-07	1.06E-05
**36h**	2.50E-06	2.42E-06	2.38E-06	2.42E-05
**48h**	1.23E-05	1.36E-05	1.13E-05	1.20E-04
**72h**	5.81E-06	7.37E-06	9.93E-07	6.86E-05
	cgd5_1640			
**2h**	2.57E-06	2.52E-06	8.85E-07	1.80E-06
**6h**	3.59E-05	1.97E-05	1.27E-04	4.76E-05
**12h**	6.75E-05	1.15E-04	1.38E-04	5.48E-05
**24h**	1.77E-05	7.33E-05	5.33E-05	1.76E-05
**36h**	4.00E-05	8.03E-05	7.31E-05	6.86E-05
**48h**	1.46E-05	4.54E-05	1.64E-05	1.80E-05
**72h**	5.35E-06	2.58E-06	4.39E-06	3.62E-06
	cgd5_1650			
**2h**	0.00E+00	0.00E+00	0.00E+00	0.00E+00
**6h**	3.18E-04	2.86E-05	6.50E-04	7.16E-05
**12h**	1.79E-02	1.16E-03	2.67E-03	1.04E-02
**24h**	2.00E-03	2.62E-04	1.23E-03	1.32E-03

**36h**	2.36E-03	2.42E-04	5.48E-03	4.28E-03
**48h**	9.43E-04	5.73E-05	1.35E-03	1.88E-03
**72h**	1.23E-03	1.64E-05	2.66E-04	2.26E-04
	cgd5_1660			
**2h**	0.00E+00	0.00E+00	0.00E+00	1.38E-08
**6h**	8.31E-08	1.90E-07	0.00E+00	0.00E+00
**12h**	3.87E-05	8.97E-05	4.75E-05	2.21E-04
**24h**	3.62E-06	3.96E-05	4.67E-06	1.43E-06
**36h**	8.52E-06	1.94E-05	2.56E-05	1.16E-05
**48h**	2.76E-06	1.22E-05	2.50E-06	1.27E-06
**72h**	2.05E-06	5.53E-06	1.61E-06	1.39E-06
	cgd5_1670			
**2h**	0.00E+00	0.00E+00	0.00E+00	0.00E+00
**6h**	9.90E-08	0.00E+00	5.33E-08	7.53E-09
**12h**	1.21E-04	4.03E-04	1.38E-04	4.52E-05
**24h**	9.04E-06	6.42E-05	1.06E-05	2.67E-06
**36h**	2.90E-05	5.51E-05	7.61E-05	7.54E-05
**48h**	7.12E-06	2.37E-05	7.20E-06	4.15E-06
**72h**	2.26E-06	7.05E-06	2.99E-06	1.35E-06
	cgd5_1680			
**2h**	0.00E+00	0.00E+00	0.00E+00	0.00E+00
**6h**	2.65E-09	1.10E-08	7.44E-08	1.34E-08
**12h**	9.13E-07	1.00E-07	9.29E-07	5.41E-07
**24h**	2.82E-07	2.64E-07	2.22E-07	2.04E-07
**36h**	1.49E-06	1.01E-06	1.64E-06	1.57E-06
**48h**	7.92E-07	2.92E-07	3.56E-07	3.81E-07
**72h**	2.66E-07	1.22E-07	1.78E-07	1.17E-07
	cgd5_1690			
**2h**	3.09E-04	1.69E-04	5.44E-05	1.59E-04
**6h**	9.12E-04	4.53E-04	7.94E-04	1.39E-04
**12h**	9.64E-05	1.35E-04	1.16E-03	2.62E-04
**24h**	4.19E-04	1.01E-04	9.11E-04	8.10E-05
**36h**	6.11E-04	6.41E-04	4.48E-04	2.49E-04
**48h**	4.06E-04	7.09E-05	1.67E-04	1.19E-04
**72h**	9.57E-05	4.99E-05	4.44E-05	1.94E-05
	cgd5_1710			
**2h**	1.26E-04	4.09E-04	4.97E-05	1.01E-04
**6h**	1.47E-03	4.95E-04	3.99E-04	1.49E-04
**12h**	8.74E-05	1.57E-04	4.73E-04	1.09E-04
**24h**	1.58E-04	5.59E-04	1.96E-04	6.33E-05
**36h**	7.98E-05	1.92E-04	6.91E-05	5.02E-05
**48h**	5.33E-05	7.52E-05	3.09E-05	2.90E-05
**72h**	6.32E-06	2.03E-05	7.58E-06	2.41E-06
	cgd5_1720			
**2h**	8.50E-07	2.90E-06	2.52E-07	9.31E-07
**6h**	1.53E-05	2.06E-05	1.91E-05	4.66E-06
**12h**	5.66E-07	7.69E-06	4.42E-06	1.07E-06
**24h**	2.53E-06	7.19E-06	4.47E-06	2.23E-06
**36h**	3.08E-06	8.58E-06	1.94E-06	1.33E-06
**48h**	2.77E-06	3.01E-06	2.76E-06	1.29E-06
**72h**	3.12E-07	1.10E-06	5.85E-07	1.03E-07
	cgd5_1730			
**2h**	2.02E-06	2.44E-06	2.36E-07	8.63E-07

**6h**	1.96E-06	2.67E-06	1.82E-06	4.96E-06
**12h**	2.90E-07	3.98E-08	1.80E-06	4.72E-07
**24h**	1.57E-06	3.31E-07	1.51E-06	1.16E-06
**36h**	5.30E-07	1.21E-06	6.73E-07	3.65E-07
**48h**	7.48E-07	1.94E-07	5.22E-07	4.31E-07
**72h**	9.23E-08	1.81E-07	3.89E-08	4.00E-08
	cgd5_1750			
**2h**	0.00E+00	0.00E+00	0.00E+00	0.00E+00
**6h**	0.00E+00	0.00E+00	1.52E-08	0.00E+00
**12h**	9.73E-09	3.95E-08	3.55E-08	3.10E-09
**24h**	1.10E-07	3.05E-08	7.09E-08	4.76E-08
**36h**	3.18E-08	5.04E-08	7.28E-08	5.00E-08
**48h**	1.33E-06	3.30E-07	1.57E-06	1.20E-06
**72h**	2.03E-07	3.64E-07	1.70E-07	1.12E-07
	cgd5_1760			
**2h**	1.55E-05	9.47E-05	5.27E-06	1.41E-05
**6h**	2.59E-05	4.79E-05	3.06E-05	2.35E-05
**12h**	8.74E-06	2.95E-05	5.53E-05	1.77E-05
**24h**	1.69E-05	2.73E-05	2.82E-05	1.31E-05
**36h**	5.20E-06	3.66E-05	8.19E-06	5.73E-06
**48h**	6.99E-06	1.16E-05	9.42E-06	5.57E-06
**72h**	9.23E-07	7.99E-06	1.12E-06	1.34E-06
	cgd5_1770			
**2h**	4.39E-07	1.52E-07	2.72E-07	1.46E-07
**6h**	2.33E-08	2.94E-08	2.56E-07	1.18E-07
**12h**	3.94E-07	5.72E-07	1.01E-06	2.24E-07
**24h**	8.95E-08	9.43E-08	9.10E-07	1.94E-07
**36h**	4.05E-07	5.63E-07	1.22E-06	5.45E-07
**48h**	1.72E-07	1.52E-07	3.79E-07	2.27E-07
**72h**	1.98E-07	1.10E-07	2.51E-07	1.63E-07
	cgd5_1930			
**2h**	0.00E+00	0.00E+00	0.00E+00	0.00E+00
**6h**	3.12E-07	1.48E-07	1.93E-07	1.72E-07
**12h**	8.51E-07	2.27E-06	2.54E-06	2.54E-07
**24h**	3.99E-07	4.02E-07	1.45E-06	2.05E-07
**36h**	4.91E-07	5.61E-07	1.45E-06	7.20E-07
**48h**	4.29E-07	2.70E-07	9.16E-07	1.94E-07
**72h**	1.71E-07	1.76E-07	2.18E-07	1.55E-07
	cgd5_1940			
**2h**	8.10E-09	0.00E+00	0.00E+00	0.00E+00
**6h**	0.00E+00	0.00E+00	0.00E+00	0.00E+00
**12h**	6.33E-07	2.47E-06	1.14E-06	3.59E-07
**24h**	1.65E-07	3.76E-07	2.54E-07	7.22E-08
**36h**	6.50E-08	1.34E-07	2.91E-07	2.31E-07
**48h**	6.35E-08	4.02E-05	1.49E-07	3.08E-08
**72h**	1.32E-07	1.43E-07	2.57E-07	3.36E-07
	cgd5_1950			
**2h**	0.00E+00	0.00E+00	0.00E+00	1.49E-08
**6h**	0.00E+00	0.00E+00	0.00E+00	0.00E+00
**12h**	5.88E-07	1.48E-06	1.09E-06	4.33E-07
**24h**	1.57E-07	2.69E-07	1.63E-07	4.84E-08
**36h**	1.87E-07	1.69E-07	7.55E-07	3.63E-07
**48h**	6.40E-08	4.51E-08	3.31E-07	1.71E-08

**72h**	1.32E-07	1.62E-07	1.11E-07	1.01E-07
	cgd5_1960			
**2h**	1.13E-06	1.07E-06	6.18E-07	1.34E-06
**6h**	1.83E-06	8.44E-07	3.71E-06	1.92E-06
**12h**	5.44E-05	9.94E-05	5.74E-05	4.24E-05
**24h**	1.69E-05	1.60E-05	6.96E-06	2.86E-06
**36h**	1.54E-05	1.61E-05	3.05E-05	1.40E-05
**48h**	4.28E-06	2.71E-06	7.07E-06	1.44E-06
**72h**	2.32E-06	2.52E-06	3.21E-06	2.09E-06
	cgd5_1970			
**2h**	2.05E-07	6.34E-07	1.72E-07	3.20E-07
**6h**	7.52E-07	1.76E-06	4.96E-06	9.06E-07
**12h**	9.06E-07	2.20E-06	2.67E-06	8.27E-07
**24h**	1.95E-07	2.82E-07	1.54E-06	4.95E-07
**36h**	2.10E-06	2.23E-06	3.39E-06	5.16E-06
**48h**	3.45E-07	1.61E-07	6.49E-07	2.77E-07
**72h**	5.17E-07	6.82E-07	2.08E-07	4.55E-07
	cgd5_1980			
**2h**	8.27E-07	1.09E-06	2.85E-07	9.15E-07
**6h**	1.84E-08	6.07E-08	3.57E-08	3.72E-08
**12h**	8.44E-07	2.20E-06	1.04E-06	6.59E-07
**24h**	1.64E-07	1.69E-07	3.67E-07	1.13E-07
**36h**	5.19E-07	1.16E-06	1.47E-06	8.55E-07
**48h**	4.58E-07	2.22E-07	4.88E-07	2.78E-07
**72h**	2.91E-07	2.87E-07	7.90E-07	2.40E-07
	cgd5_1990			
**2h**	7.87E-03	5.83E-03	1.74E-03	6.61E-03
**6h**	3.82E-03	2.44E-03	1.00E-03	1.09E-02
**12h**	4.96E-03	3.35E-03	1.09E-03	1.60E-03
**24h**	2.72E-03	1.87E-04	1.26E-03	5.35E-03
**36h**	1.65E-03	2.57E-03	8.91E-04	3.85E-04
**48h**	1.03E-03	9.97E-04	1.19E-03	1.96E-04
**72h**	6.04E-04	3.15E-04	6.66E-04	1.24E-03
	cgd5_2010			
**2h**	2.21E-06	1.81E-06	1.64E-06	2.31E-06
**6h**	1.56E-07	1.28E-07	3.01E-07	4.28E-07
**12h**	7.55E-07	2.42E-06	3.92E-06	8.35E-07
**24h**	7.95E-07	4.96E-07	2.99E-07	5.62E-07
**36h**	3.93E-07	6.12E-07	6.95E-07	1.87E-07
**48h**	2.39E-07	2.82E-07	2.86E-07	1.74E-07
**72h**	9.00E-08	1.30E-07	2.10E-07	6.75E-08
	cgd5_2020			
**2h**	1.53E-09	0.00E+00	0.00E+00	0.00E+00
**6h**	0.00E+00	2.76E-10	0.00E+00	0.00E+00
**12h**	7.13E-08	0.00E+00	6.70E-08	5.82E-09
**24h**	3.13E-08	3.59E-09	2.52E-08	1.88E-09
**36h**	4.36E-08	8.90E-08	1.33E-07	5.47E-08
**48h**	5.79E-08	1.34E-07	1.10E-07	4.68E-08
**72h**	2.95E-08	3.63E-08	8.82E-08	3.97E-08
	cgd5_2040			
**2h**	1.56E-06	1.24E-08	0.00E+00	0.00E+00
**6h**	2.69E-07	1.51E-07	5.39E-07	0.00E+00
**12h**	1.55E-07	3.48E-07	1.60E-07	2.56E-08

**24h**	5.77E-08	1.97E-07	4.62E-07	4.65E-07
**36h**	2.30E-07	2.53E-07	9.32E-07	5.77E-08
**48h**	4.60E-07	3.44E-07	3.74E-07	8.49E-08
**72h**	2.87E-08	2.04E-08	1.32E-07	6.26E-09
	cgd5_2050			
**2h**	0.00E+00	0.00E+00	0.00E+00	0.00E+00
**6h**	1.09E-08	0.00E+00	0.00E+00	0.00E+00
**12h**	6.01E-07	1.08E-06	7.91E-07	1.74E-07
**24h**	2.82E-07	3.02E-07	8.86E-07	3.34E-07
**36h**	8.36E-07	8.77E-07	3.70E-06	5.25E-07
**48h**	3.01E-06	2.73E-06	4.45E-06	6.11E-07
**72h**	1.77E-07	1.36E-07	7.35E-07	2.32E-07
	cgd5_2060			
**2h**	3.04E-04	1.75E-04	3.31E-04	2.06E-04
**6h**	2.28E-04	1.30E-04	2.68E-04	2.64E-04
**12h**	4.44E-05	2.32E-05	7.75E-04	7.28E-05
**24h**	1.91E-04	1.47E-04	6.21E-04	1.44E-04
**36h**	4.46E-05	4.97E-05	2.53E-04	1.82E-05
**48h**	2.99E-05	1.58E-05	1.40E-04	5.93E-06
**72h**	3.78E-06	6.58E-06	5.13E-06	3.38E-06
	cgd5_2070			
**2h**	1.83E-03	6.46E-04	8.69E-04	9.55E-04
**6h**	1.24E-03	2.22E-03	7.14E-04	7.21E-03
**12h**	2.13E-04	1.42E-03	1.54E-03	1.77E-03
**24h**	4.38E-04	1.88E-03	1.43E-03	6.98E-04
**36h**	1.18E-04	2.69E-03	1.14E-03	1.40E-04
**48h**	1.33E-04	8.62E-04	7.58E-05	2.06E-04
**72h**	5.84E-05	2.54E-04	8.38E-05	9.78E-05
	cgd5_2090			
**2h**	0.00E+00	0.00E+00	0.00E+00	0.00E+00
**6h**	0.00E+00	6.11E-08	6.09E-08	6.37E-07
**12h**	7.49E-08	1.02E-06	2.26E-06	6.48E-07
**24h**	6.59E-07	3.92E-06	2.68E-05	1.74E-06
**36h**	2.87E-06	7.60E-06	1.96E-05	2.22E-06
**48h**	3.31E-06	5.80E-06	3.48E-05	4.87E-06
**72h**	4.65E-06	1.21E-05	8.93E-05	1.33E-05
	cgd5_2100			
**2h**	8.17E-07	1.62E-07	4.82E-08	3.16E-07
**6h**	5.86E-07	3.81E-07	4.47E-07	8.68E-07
**12h**	1.59E-07	1.10E-07	4.58E-07	2.51E-07
**24h**	3.17E-07	4.38E-07	3.85E-07	2.24E-07
**36h**	3.57E-07	4.84E-07	7.95E-07	1.33E-07
**48h**	2.10E-07	1.56E-07	4.05E-07	1.52E-07
**72h**	7.94E-07	2.93E-07	2.91E-07	2.24E-07
	cgd5_2110			
**2h**	1.87E-06	6.94E-07	1.08E-07	4.37E-07
**6h**	3.41E-07	7.41E-07	5.82E-07	6.43E-07
**12h**	1.35E-06	1.03E-07	6.88E-07	4.89E-07
**24h**	2.27E-07	8.19E-07	9.46E-07	3.66E-07
**36h**	7.15E-07	8.05E-07	6.56E-07	2.12E-07
**48h**	3.05E-07	3.30E-07	6.12E-07	3.10E-07
**72h**	3.40E-07	3.82E-07	5.58E-07	4.58E-07
	cgd5_2120			

**2h**	2.50E-06	3.98E-06	1.49E-06	4.34E-06
**6h**	1.16E-06	5.86E-06	9.21E-06	3.01E-06
**12h**	2.97E-06	4.63E-07	1.07E-05	2.17E-06
**24h**	1.24E-06	4.00E-06	1.34E-05	2.62E-06
**36h**	3.09E-06	6.62E-06	7.81E-06	1.25E-06
**48h**	1.17E-06	2.05E-06	2.44E-05	4.44E-07
**72h**	1.84E-06	2.61E-06	7.87E-06	8.69E-07
	cgd5_2130			
**2h**	1.80E-07	8.73E-08	3.20E-08	1.12E-07
**6h**	0.00E+00	7.33E-09	1.74E-08	9.88E-09
**12h**	6.37E-07	3.21E-07	7.85E-07	3.01E-07
**24h**	1.42E-07	2.80E-07	9.62E-07	2.97E-07
**36h**	1.83E-06	1.85E-06	1.86E-06	3.15E-07
**48h**	2.33E-06	9.00E-07	2.34E-06	5.66E-07
**72h**	8.22E-07	7.47E-07	1.36E-06	6.94E-07
	cgd5_2140			
**2h**	1.71E-05	3.08E-04	9.26E-05	1.34E-04
**6h**	1.12E-05	1.40E-04	5.43E-05	6.93E-05
**12h**	2.51E-05	8.00E-05	1.56E-04	6.49E-05
**24h**	8.75E-06	1.16E-04	8.01E-05	3.58E-05
**36h**	8.86E-06	2.84E-04	2.01E-04	8.00E-06
**48h**	1.56E-05	1.76E-04	5.69E-05	1.86E-05
**72h**	2.40E-06	1.14E-04	9.66E-05	4.51E-05
	cgd5_2150			
**2h**	6.24E-06	1.68E-06	8.38E-07	1.31E-06
**6h**	5.73E-06	1.22E-06	1.76E-06	4.32E-06
**12h**	3.31E-06	1.04E-06	2.78E-06	1.49E-06
**24h**	1.81E-06	6.45E-07	1.68E-06	1.18E-06
**36h**	2.02E-06	2.94E-06	4.61E-06	4.45E-07
**48h**	1.76E-06	1.55E-06	2.56E-06	5.43E-07
**72h**	7.36E-07	8.85E-07	1.43E-06	7.14E-07
	cgd5_2190			
**2h**	7.89E-06	3.11E-06	3.98E-07	1.78E-06
**6h**	1.23E-05	4.23E-06	4.14E-06	1.12E-05
**12h**	2.12E-06	3.27E-06	1.93E-06	9.19E-07
**24h**	2.43E-06	1.30E-06	4.39E-06	3.17E-06
**36h**	3.15E-06	6.02E-06	4.76E-06	8.03E-07
**48h**	2.61E-06	2.89E-05	3.14E-06	6.43E-07
**72h**	2.71E-07	3.72E-07	7.09E-07	3.76E-07
	cgd5_2200			
**2h**	1.31E-04	1.71E-04	2.53E-05	4.53E-05
**6h**	8.26E-05	3.77E-05	1.28E-04	1.59E-04
**12h**	9.94E-06	1.75E-05	7.95E-05	3.35E-05
**24h**	4.06E-05	3.17E-05	1.04E-04	2.10E-05
**36h**	6.66E-06	5.65E-05	7.12E-05	5.33E-06
**48h**	1.13E-05	2.82E-05	3.06E-05	3.61E-06
**72h**	4.79E-07	2.73E-06	5.28E-06	1.43E-06
	cgd5_2210			
**2h**	1.46E-06	1.84E-04	4.25E-05	4.32E-05
**6h**	4.13E-05	2.08E-04	1.84E-04	1.12E-04
**12h**	8.60E-07	7.45E-05	1.11E-04	4.46E-05
**24h**	1.03E-05	2.05E-04	2.04E-04	4.05E-05
**36h**	5.02E-06	2.69E-04	1.15E-04	1.62E-05

**48h**	1.08E-05	1.72E-04	5.35E-05	2.88E-05
**72h**	6.74E-07	5.82E-05	3.33E-05	5.21E-05
	cgd5_2230			
**2h**	4.19E-08	0.00E+00	0.00E+00	0.00E+00
**6h**	7.77E-06	3.22E-06	4.47E-06	6.87E-06
**12h**	3.51E-07	2.98E-07	1.30E-06	6.57E-07
**24h**	3.04E-06	5.95E-07	7.55E-06	4.17E-06
**36h**	1.74E-06	6.63E-06	7.34E-06	9.46E-07
**48h**	8.56E-07	1.39E-06	9.29E-06	7.28E-07
**72h**	3.36E-07	3.86E-07	1.43E-06	3.06E-07
	cgd5_2240			
**2h**	0.00E+00	1.99E-07	3.31E-08	5.07E-08
**6h**	5.71E-05	4.64E-05	1.07E-05	4.37E-05
**12h**	2.70E-04	1.88E-04	2.85E-04	1.21E-04
**24h**	7.40E-05	2.57E-05	7.84E-05	4.07E-05
**36h**	4.74E-05	3.80E-05	3.38E-05	5.10E-05
**48h**	3.96E-05	1.41E-05	3.50E-05	1.33E-05
**72h**	1.03E-05	2.69E-06	1.31E-05	6.74E-06
	cgd5_2250			
**2h**	0.00E+00	0.00E+00	0.00E+00	0.00E+00
**6h**	0.00E+00	0.00E+00	0.00E+00	0.00E+00
**12h**	3.18E-04	2.18E-04	5.16E-04	2.40E-04
**24h**	1.48E-04	8.70E-05	3.59E-05	1.71E-05
**36h**	1.19E-05	1.30E-05	2.70E-05	4.61E-05
**48h**	1.76E-05	5.32E-06	2.59E-05	7.87E-06
**72h**	1.50E-05	6.33E-06	6.16E-05	7.57E-06
	cgd5_2280			
**2h**	2.56E-07	1.38E-07	1.12E-08	2.38E-07
**6h**	3.31E-08	1.13E-07	7.69E-08	1.34E-07
**12h**	2.02E-06	1.65E-06	1.10E-06	9.79E-07
**24h**	6.06E-07	2.63E-07	1.58E-06	3.28E-07
**36h**	7.98E-07	7.26E-07	5.44E-07	1.31E-06
**48h**	1.09E-06	3.13E-07	3.84E-06	6.89E-07
**72h**	3.41E-06	1.61E-06	1.69E-06	3.16E-06
	cgd5_2300			
**2h**	2.81E-07	2.31E-07	3.89E-08	9.54E-08
**6h**	3.15E-07	9.20E-08	1.25E-07	2.07E-07
**12h**	7.97E-08	7.65E-09	4.84E-08	8.94E-08
**24h**	8.18E-08	8.42E-08	7.40E-07	1.01E-07
**36h**	4.43E-07	3.23E-07	1.00E-07	2.05E-07
**48h**	8.82E-07	2.86E-07	3.06E-06	6.10E-07
**72h**	3.03E-06	1.07E-06	2.07E-06	2.09E-06
	cgd5_2310			
**2h**	8.72E-08	1.21E-06	1.86E-07	6.48E-07
**6h**	1.64E-06	1.89E-06	2.91E-06	1.69E-06
**12h**	5.54E-07	1.76E-06	1.54E-06	2.55E-06
**24h**	5.21E-07	5.28E-07	4.25E-06	1.52E-06
**36h**	2.91E-06	3.04E-06	1.02E-06	3.27E-06
**48h**	9.60E-07	3.26E-07	1.50E-06	1.16E-06
**72h**	6.64E-07	4.56E-07	7.82E-07	1.19E-06
	cgd5_2320			
**2h**	9.42E-06	4.97E-06	7.45E-06	2.82E-05
**6h**	2.73E-05	1.92E-05	1.29E-05	2.40E-05

**12h**	1.03E-04	4.59E-05	1.09E-04	6.05E-05
**24h**	5.93E-05	2.31E-05	1.31E-04	5.17E-05
**36h**	7.60E-05	5.23E-05	4.32E-05	5.45E-05
**48h**	4.44E-05	9.86E-06	2.10E-04	2.01E-05
**72h**	2.95E-05	1.18E-05	5.13E-05	1.79E-05
	cgd5_2330			
**2h**	5.01E-07	2.68E-06	6.81E-08	9.83E-07
**6h**	4.30E-07	4.63E-07	7.11E-07	1.95E-06
**12h**	7.27E-06	7.90E-06	2.74E-06	4.10E-06
**24h**	1.77E-06	1.01E-06	4.74E-06	3.53E-06
**36h**	7.19E-06	3.88E-06	2.65E-06	6.00E-06
**48h**	2.02E-06	1.04E-06	2.52E-06	2.28E-06
**72h**	1.21E-06	3.05E-07	2.34E-06	8.10E-07
	cgd5_2340			
**2h**	5.01E-06	3.56E-06	1.21E-06	2.31E-06
**6h**	1.65E-05	1.61E-05	1.13E-05	2.00E-05
**12h**	1.25E-05	9.60E-07	1.27E-05	4.78E-06
**24h**	8.05E-06	4.73E-06	2.84E-05	1.34E-05
**36h**	2.28E-05	8.72E-06	7.18E-06	5.92E-06
**48h**	2.01E-05	7.56E-06	2.56E-05	8.43E-06
**72h**	7.26E-06	1.50E-06	2.09E-05	4.56E-06
	cgd5_2350			
**2h**	1.86E-06	2.32E-06	1.43E-06	2.18E-06
**6h**	8.97E-07	1.48E-06	2.63E-06	6.65E-06
**12h**	1.22E-07	2.86E-07	5.12E-06	1.69E-06
**24h**	5.19E-07	6.83E-07	3.30E-06	3.45E-06
**36h**	1.50E-06	9.11E-07	7.56E-07	5.26E-07
**48h**	8.91E-07	9.12E-07	6.09E-07	6.86E-07
**72h**	1.13E-07	6.37E-08	8.02E-07	2.90E-07
	cgd5_2360			
**2h**	5.56E-07	1.63E-07	2.86E-08	0.00E+00
**6h**	2.44E-05	7.41E-06	1.94E-05	6.10E-06
**12h**	6.30E-08	1.39E-07	1.29E-07	3.02E-07
**24h**	1.38E-06	5.89E-07	9.81E-06	2.66E-06
**36h**	2.65E-06	1.74E-06	1.58E-06	5.12E-07
**48h**	4.74E-06	1.14E-06	7.26E-06	2.13E-06
**72h**	5.59E-07	3.11E-07	2.48E-06	1.41E-06
	cgd5_2370			
**2h**	6.16E-06	2.42E-05	7.12E-07	1.10E-05
**6h**	7.28E-05	3.14E-05	1.11E-05	1.23E-04
**12h**	2.89E-06	3.48E-06	1.61E-05	3.12E-05
**24h**	9.87E-06	1.42E-05	1.74E-05	3.20E-05
**36h**	6.71E-06	2.96E-06	4.28E-06	6.07E-06
**48h**	9.23E-06	3.13E-06	8.30E-06	5.13E-06
**72h**	7.88E-07	4.29E-07	5.78E-06	1.35E-06
	cgd5_2380			
**2h**	3.84E-06	2.59E-05	5.19E-06	6.64E-06
**6h**	2.57E-06	2.93E-06	1.48E-05	1.07E-05
**12h**	5.34E-06	4.51E-07	1.32E-05	7.79E-06
**24h**	5.62E-06	2.81E-06	4.28E-05	1.15E-05
**36h**	8.92E-06	3.06E-06	1.32E-05	8.35E-06
**48h**	4.96E-06	1.50E-06	1.73E-05	3.09E-06
**72h**	2.34E-06	2.25E-06	3.48E-05	2.97E-06

	cgd5_2530			
**2h**	0.00E+00	0.00E+00	1.57E-08	1.93E-08
**6h**	9.49E-07	9.49E-07	2.97E-06	8.57E-07
**12h**	2.54E-07	2.54E-07	3.55E-07	4.73E-07
**24h**	3.96E-07	3.96E-07	8.66E-06	1.42E-06
**36h**	8.73E-07	8.73E-07	7.19E-07	1.29E-06
**48h**	2.00E-06	2.00E-06	5.93E-06	1.54E-06
**72h**	4.69E-06	4.69E-06	2.08E-06	3.57E-06
	cgd5_2540			
**2h**	1.15E-06	1.15E-06	5.23E-07	1.63E-06
**6h**	3.07E-07	3.07E-07	6.25E-07	5.78E-07
**12h**	1.49E-07	1.49E-07	4.96E-07	4.82E-07
**24h**	6.69E-07	6.69E-07	8.34E-07	4.53E-07
**36h**	1.26E-07	1.26E-07	1.25E-07	3.34E-07
**48h**	1.58E-07	1.58E-07	1.23E-07	1.48E-07
**72h**	5.02E-08	5.02E-08	1.38E-07	2.99E-07
	cgd5_2560			
**2h**	0.00E+00	0.00E+00	0.00E+00	2.02E-09
**6h**	0.00E+00	0.00E+00	3.86E-09	1.27E-08
**12h**	0.00E+00	0.00E+00	1.93E-08	1.85E-08
**24h**	3.83E-09	3.83E-09	1.11E-07	3.31E-08
**36h**	4.62E-08	4.62E-08	1.01E-07	1.02E-07
**48h**	9.98E-08	9.98E-08	1.17E-07	1.04E-07
**72h**	2.82E-07	2.82E-07	1.26E-07	1.78E-07
	cgd5_2570			
**2h**	3.50E-08	3.50E-08	0.00E+00	1.11E-07
**6h**	0.00E+00	0.00E+00	1.61E-07	1.14E-08
**12h**	1.76E-04	1.76E-04	6.56E-05	6.43E-05
**24h**	7.08E-06	7.08E-06	6.28E-06	2.32E-06
**36h**	8.54E-06	8.54E-06	4.02E-05	2.34E-05
**48h**	1.63E-06	1.63E-06	2.83E-06	2.21E-06
**72h**	1.81E-05	1.81E-05	2.64E-05	1.36E-05
	cgd5_2580			
**2h**	1.68E-08	1.68E-08	0.00E+00	1.88E-08
**6h**	0.00E+00	0.00E+00	6.61E-09	0.00E+00
**12h**	2.04E-07	2.04E-07	8.93E-08	1.88E-07
**24h**	1.71E-08	1.71E-08	4.33E-08	3.62E-08
**36h**	7.96E-08	7.96E-08	1.85E-07	1.64E-07
**48h**	5.36E-08	5.36E-08	4.09E-08	5.68E-08
**72h**	4.08E-08	4.08E-08	1.68E-07	1.20E-07
	cgd5_2590			
**2h**	3.64E-05	3.64E-05	1.28E-06	5.36E-06
**6h**	6.41E-05	6.41E-05	2.14E-05	1.07E-05
**12h**	1.05E-05	1.05E-05	4.55E-06	2.25E-06
**24h**	4.32E-06	4.32E-06	1.34E-05	6.34E-06
**36h**	9.76E-06	9.76E-06	1.76E-05	1.21E-05
**48h**	3.04E-05	3.04E-05	1.37E-05	8.86E-06
**72h**	1.27E-05	1.27E-05	8.26E-06	7.98E-06
	cgd5_2600			
**2h**	6.30E-07	6.30E-07	4.10E-07	3.54E-07
**6h**	1.20E-05	1.20E-05	7.51E-06	4.77E-06
**12h**	1.85E-07	1.85E-07	4.63E-07	5.12E-07
**24h**	1.73E-06	1.73E-06	4.14E-06	2.53E-06

**36h**	7.44E-07	7.44E-07	2.80E-06	2.12E-06
**48h**	1.98E-06	1.98E-06	1.98E-06	1.90E-06
**72h**	4.25E-07	4.25E-07	6.69E-07	2.65E-07
	cgd5_2610			
**2h**	4.75E-03	4.75E-03	2.17E-04	1.10E-04
**6h**	3.03E-03	3.03E-03	8.65E-04	8.31E-04
**12h**	6.19E-05	6.19E-05	7.10E-05	7.71E-06
**24h**	7.04E-04	7.04E-04	9.42E-04	3.00E-04
**36h**	3.48E-04	3.48E-04	3.67E-04	1.93E-04
**48h**	7.54E-04	7.54E-04	3.80E-04	2.82E-04
**72h**	9.26E-05	9.26E-05	1.04E-04	7.63E-05
	cgd5_2640			
**2h**	3.41E-03	3.41E-03	4.99E-05	1.26E-04
**6h**	5.18E-02	5.18E-02	3.35E-03	1.20E-03
**12h**	1.01E-04	1.01E-04	5.01E-04	1.40E-04
**24h**	7.28E-04	7.28E-04	1.41E-03	8.71E-04
**36h**	1.25E-04	1.25E-04	4.07E-04	1.65E-04
**48h**	3.92E-04	3.92E-04	4.54E-04	3.46E-04
**72h**	2.88E-05	2.88E-05	7.00E-05	2.80E-05
	cgd5_2650			
**2h**	2.95E-07	2.95E-07	1.06E-07	7.87E-08
**6h**	2.70E-06	2.70E-06	3.64E-06	1.74E-06
**12h**	5.56E-08	5.56E-08	1.51E-07	5.74E-08
**24h**	2.75E-07	2.75E-07	1.86E-06	1.12E-06
**36h**	1.84E-07	1.84E-07	5.67E-07	2.80E-07
**48h**	2.95E-07	2.95E-07	6.76E-07	6.38E-07
**72h**	7.75E-08	7.75E-08	2.61E-07	8.95E-08
	cgd5_2660			
**2h**	0.00E+00	2.00E-06	0.00E+00	0.00E+00
**6h**	9.44E-07	9.44E-07	6.63E-08	5.99E-08
**12h**	0.00E+00	1.23E-07	0.00E+00	1.56E-08
**24h**	3.34E-07	3.34E-07	4.38E-07	4.11E-08
**36h**	1.60E-06	1.60E-06	2.76E-06	5.19E-07
**48h**	1.21E-05	1.21E-05	5.18E-05	1.39E-05
**72h**	1.31E-04	1.31E-04	1.94E-05	9.68E-06
	cgd5_2670			
**2h**	5.50E-05	0.00E+00	4.20E-04	1.66E-05
**6h**	5.23E-03	4.47E-03	8.74E-03	1.76E-03
**12h**	6.61E-04	2.35E-03	1.89E-03	3.40E-04
**24h**	6.86E-04	4.54E-03	4.04E-03	1.05E-03
**36h**	2.95E-04	3.14E-03	4.46E-03	1.58E-03
**48h**	1.69E-03	4.09E-03	6.68E-03	2.13E-03
**72h**	2.30E-04	2.12E-03	1.48E-03	2.42E-03
	cgd1_3480			
**2h**	2.28E-04	2.02E-04	1.24E-03	1.29E-04
**6h**	5.87E-04	3.64E-04	1.11E-03	1.90E-04
**12h**	2.98E-05	9.21E-05	1.75E-04	1.09E-05
**24h**	1.30E-03	2.05E-04	7.42E-04	3.78E-05
**36h**	5.33E-05	1.34E-04	2.60E-04	2.87E-05
**48h**	2.98E-05	3.95E-05	1.27E-04	1.25E-05
**72h**	2.04E-05	9.14E-06	2.53E-05	8.99E-06
	cgd1_3490			
**2h**	0.00E+00	0.00E+00	0.00E+00	0.00E+00

**6h**	0.00E+00	2.58E-08	0.00E+00	2.43E-08
**12h**	7.22E-06	1.84E-05	1.44E-05	1.02E-05
**24h**	3.26E-06	2.33E-06	4.35E-06	1.74E-06
**36h**	1.50E-06	9.67E-06	2.17E-05	2.37E-06
**48h**	9.63E-07	2.18E-06	4.80E-06	1.02E-06
**72h**	5.39E-07	1.51E-06	3.01E-06	2.20E-07
	cgd1_3650			
**2h**	9.39E-07	2.54E-06	3.40E-07	5.31E-07
**6h**	3.78E-06	7.29E-07	4.24E-07	5.60E-07
**12h**	7.55E-06	8.69E-06	7.12E-06	1.05E-04
**24h**	5.23E-06	1.09E-06	2.38E-06	2.31E-06
**36h**	9.80E-07	1.16E-06	9.91E-07	1.74E-06
**48h**	6.10E-07	4.06E-07	1.00E-06	5.37E-07
**72h**	4.57E-07	1.32E-07	3.26E-07	1.95E-07
	cgd1_3670			
**2h**	1.03E-05	2.02E-05	5.43E-06	1.52E-05
**6h**	1.17E-05	6.82E-06	7.36E-06	1.73E-05
**12h**	2.64E-06	2.49E-06	4.86E-06	4.94E-06
**24h**	2.71E-06	2.80E-06	1.59E-05	1.81E-05
**36h**	6.61E-06	5.62E-06	4.70E-06	8.35E-06
**48h**	3.25E-06	3.11E-06	9.09E-06	5.18E-06
**72h**	3.40E-05	4.12E-06	1.61E-05	1.15E-05
	cgd5_910			
**2h**	9.81E-08	0.00E+00	0.00E+00	2.09E-08
**6h**	0.00E+00	6.34E-09	0.00E+00	2.37E-10
**12h**	7.00E-09	0.00E+00	0.00E+00	1.03E-08
**24h**	1.15E-07	3.02E-09	2.05E-07	4.68E-08
**36h**	4.03E-07	9.92E-08	1.49E-07	1.29E-07
**48h**	1.87E-06	1.06E-06	3.98E-06	7.97E-07
**72h**	3.56E-07	2.27E-07	8.98E-07	2.84E-07
	cgd5_930			
**2h**	2.53E-04	8.10E-05	1.26E-05	8.27E-05
**6h**	5.04E-04	6.86E-05	2.94E-04	2.86E-04
**12h**	2.04E-04	1.36E-05	2.25E-04	1.52E-04
**24h**	2.82E-04	1.02E-04	8.45E-04	3.00E-04
**36h**	5.92E-05	5.47E-05	3.59E-04	9.95E-05
**48h**	2.67E-04	7.64E-05	2.36E-04	1.28E-04
**72h**	1.17E-04	7.83E-05	1.04E-04	8.35E-05
	cgd5_940			
**2h**	2.37E-06	1.05E-06	4.17E-07	2.36E-06
**6h**	5.03E-06	4.70E-06	1.36E-05	1.12E-05
**12h**	7.45E-06	8.18E-06	4.30E-06	5.26E-06
**24h**	3.45E-06	5.22E-06	1.15E-05	8.07E-06
**36h**	4.87E-06	3.56E-06	7.39E-06	8.98E-06
**48h**	3.98E-06	3.93E-06	5.25E-06	4.84E-06
**72h**	3.43E-06	5.46E-06	5.78E-06	4.71E-06
	cgd5_950			
**2h**	1.11E-06	2.14E-07	2.55E-07	2.01E-06
**6h**	1.39E-07	6.81E-08	2.67E-07	3.69E-07
**12h**	5.85E-07	2.44E-07	1.23E-06	8.33E-07
**24h**	4.44E-07	6.34E-07	2.41E-06	5.48E-07
**36h**	2.62E-06	1.37E-06	2.78E-06	2.00E-06
**48h**	1.18E-06	6.45E-07	1.57E-06	6.48E-07

**72h**	4.10E-06	2.89E-06	6.19E-06	2.56E-06
	cgd5_960			
**2h**	4.04E-07	6.49E-08	9.63E-08	2.11E-07
**6h**	9.23E-08	8.20E-08	1.25E-07	1.87E-08
**12h**	2.78E-08	1.12E-07	2.79E-07	1.62E-07
**24h**	1.14E-07	1.50E-07	6.98E-07	1.93E-07
**36h**	1.18E-06	9.24E-07	1.56E-06	1.53E-06
**48h**	5.86E-07	2.70E-07	4.91E-07	2.10E-07
**72h**	1.81E-06	1.74E-06	2.20E-06	8.77E-07
	cgd5_990			
**2h**	5.61E-06	3.15E-06	2.10E-06	7.21E-06
**6h**	8.46E-06	5.55E-06	4.83E-06	4.85E-06
**12h**	1.65E-07	2.85E-07	1.67E-06	2.84E-06
**24h**	9.59E-07	3.81E-07	5.89E-06	2.35E-06
**36h**	2.65E-06	2.46E-06	5.79E-06	2.80E-06
**48h**	1.92E-06	7.96E-07	2.95E-06	1.07E-06
**72h**	8.23E-07	1.62E-06	7.12E-07	1.83E-06
	cgd5_1000			
**2h**	9.02E-09	2.80E-08	0.00E+00	1.19E-08
**6h**	0.00E+00	0.00E+00	0.00E+00	2.64E-08
**12h**	3.96E-05	2.02E-05	4.91E-05	2.53E-05
**24h**	2.93E-06	2.32E-06	5.12E-06	1.43E-06
**36h**	6.15E-06	5.95E-06	4.77E-05	1.76E-05
**48h**	2.03E-06	5.41E-07	6.96E-06	2.14E-06
**72h**	3.46E-06	3.28E-06	5.29E-06	2.37E-06
	cgd5_1010			
**2h**	7.45E-07	1.45E-07	1.66E-07	6.44E-07
**6h**	2.13E-06	1.24E-06	1.67E-06	2.79E-06
**12h**	4.72E-05	2.50E-05	2.40E-05	1.36E-05
**24h**	2.48E-06	1.98E-06	3.51E-06	1.56E-06
**36h**	6.39E-06	5.64E-06	1.13E-05	5.53E-05
**48h**	4.96E-06	1.38E-06	6.38E-06	4.00E-06
**72h**	9.57E-07	2.07E-06	1.52E-06	6.82E-07
	cgd5_1020			
**2h**	3.23E-07	3.04E-07	4.85E-07	3.39E-07
**6h**	1.10E-06	1.39E-06	2.60E-06	1.93E-06
**12h**	1.82E-05	3.11E-05	2.35E-05	1.82E-05
**24h**	3.73E-06	2.12E-06	5.41E-06	3.10E-06
**36h**	2.74E-06	3.27E-06	1.85E-05	1.05E-05
**48h**	3.82E-06	1.45E-06	6.23E-06	3.41E-06
**72h**	5.19E-07	1.28E-06	8.56E-07	4.46E-07
	cgd5_1040			
**2h**	1.96E-05	4.80E-06	4.65E-07	2.32E-06
**6h**	8.66E-05	3.54E-05	1.72E-04	9.89E-05
**12h**	6.26E-05	5.54E-05	1.04E-04	1.04E-04
**24h**	5.08E-05	1.93E-05	2.48E-04	7.75E-05
**36h**	4.68E-05	4.16E-05	1.10E-04	5.08E-05
**48h**	3.07E-05	1.78E-05	6.88E-05	2.37E-05
**72h**	4.66E-06	4.28E-06	1.04E-05	2.96E-06
	cgd5_1070			
**2h**	4.09E-04	3.18E-04	7.07E-05	2.11E-04
**6h**	2.76E-04	3.08E-05	1.14E-03	1.78E-04
**12h**	8.65E-05	4.07E-05	1.68E-03	1.61E-04

**24h**	4.87E-04	5.75E-05	5.93E-04	1.54E-04
**36h**	1.13E-04	6.73E-05	2.79E-04	8.72E-05
**48h**	5.35E-04	2.58E-05	5.12E-04	8.29E-05
**72h**	7.52E-05	1.24E-05	5.98E-05	1.47E-05
	cgd5_1090			
**2h**	2.04E-06	1.61E-06	1.81E-06	9.61E-07
**6h**	6.55E-07	3.39E-07	1.65E-06	8.49E-07
**12h**	1.71E-05	1.58E-05	1.53E-05	2.87E-06
**24h**	5.22E-06	5.63E-07	7.11E-06	2.14E-06
**36h**	1.92E-05	3.56E-06	1.17E-05	2.80E-06
**48h**	1.87E-05	1.05E-06	1.22E-05	1.80E-06
**72h**	1.48E-05	1.69E-06	2.48E-05	2.77E-06
	cgd5_1100			
**2h**	0.00E+00	0.00E+00	5.05E-09	4.75E-08
**6h**	0.00E+00	5.89E-09	0.00E+00	1.32E-08
**12h**	1.28E-06	1.43E-06	1.11E-06	1.74E-07
**24h**	4.45E-07	2.20E-07	5.89E-07	2.05E-07
**36h**	4.91E-08	2.80E-07	1.03E-06	1.27E-06
**48h**	2.53E-07	2.79E-07	5.64E-07	1.53E-07
**72h**	2.86E-08	8.45E-08	5.11E-08	1.98E-07
	cgd5_1120			
**2h**	1.75E-06	5.37E-07	9.45E-08	4.65E-07
**6h**	1.98E-06	3.88E-07	1.14E-06	1.18E-06
**12h**	3.74E-07	4.28E-08	3.93E-07	2.53E-07
**24h**	9.55E-07	8.65E-07	1.26E-06	8.14E-07
**36h**	4.49E-07	5.92E-07	5.21E-07	4.05E-07
**48h**	3.69E-07	5.87E-07	5.76E-07	9.10E-07
**72h**	9.85E-08	1.45E-07	7.16E-08	5.58E-08
	cgd5_1130			
**2h**	2.13E-07	3.46E-07	3.72E-08	2.74E-07
**6h**	4.69E-07	1.13E-06	2.00E-06	8.03E-07
**12h**	1.12E-07	6.92E-08	2.08E-07	1.19E-07
**24h**	4.10E-07	7.00E-07	1.51E-06	9.54E-07
**36h**	2.78E-07	3.71E-07	4.40E-07	3.38E-07
**48h**	1.23E-07	6.22E-07	2.73E-07	4.91E-07
**72h**	7.54E-08	1.30E-07	1.90E-07	9.29E-07
	cgd5_1140			
**2h**	0.00E+00	0.00E+00	0.00E+00	1.14E-05
**6h**	4.49E-06	1.35E-04	2.63E-05	9.22E-05
**12h**	3.03E-07	3.92E-06	4.74E-06	1.51E-06
**24h**	3.06E-06	1.28E-05	3.22E-05	3.41E-05
**36h**	6.00E-06	4.30E-05	2.82E-05	3.37E-05
**48h**	2.46E-06	4.16E-05	1.79E-05	2.45E-05
**72h**	2.20E-06	1.02E-05	1.59E-05	7.28E-06
	cgd5_1160			
**2h**	4.08E-06	7.16E-06	2.17E-06	1.91E-06
**6h**	3.62E-06	1.08E-05	9.15E-06	9.31E-06
**12h**	4.71E-07	2.54E-08	7.91E-06	2.66E-06
**24h**	8.66E-07	5.51E-06	1.55E-05	4.97E-06
**36h**	8.99E-07	3.53E-06	4.19E-06	2.78E-06
**48h**	1.99E-07	2.01E-06	2.42E-06	2.23E-06
**72h**	7.36E-08	5.56E-07	3.06E-07	1.18E-07
	cgd5_1170			

**2h**	5.32E-07	4.18E-07	5.25E-08	1.13E-06
**6h**	4.24E-06	1.37E-05	1.44E-05	7.69E-06
**12h**	2.03E-07	6.67E-08	2.77E-06	1.16E-06
**24h**	1.53E-06	3.52E-06	3.13E-05	9.49E-06
**36h**	4.55E-06	6.44E-06	6.41E-06	1.39E-05
**48h**	3.72E-06	3.57E-06	1.21E-05	6.93E-06
**72h**	1.04E-06	2.76E-06	3.45E-06	2.48E-06
	cgd5_1180			
**2h**	2.79E-06	2.66E-05	0.00E+00	0.00E+00
**6h**	1.28E-04	9.46E-05	1.43E-04	8.07E-04
**12h**	3.23E-06	4.53E-05	2.29E-05	4.22E-05
**24h**	4.53E-05	6.83E-05	1.29E-04	1.81E-04
**36h**	4.04E-05	8.14E-05	3.79E-05	4.06E-04
**48h**	9.13E-05	2.99E-05	2.08E-05	2.63E-04
**72h**	1.40E-05	9.38E-06	1.98E-05	1.85E-04
	cgd5_1190			
**2h**	3.62E-05	1.61E-05	4.79E-06	1.56E-05
**6h**	3.92E-06	4.40E-06	5.12E-06	6.54E-06
**12h**	1.97E-05	1.01E-05	2.40E-05	1.61E-05
**24h**	1.30E-05	1.46E-05	3.04E-05	1.39E-05
**36h**	6.97E-06	8.69E-06	7.16E-06	9.15E-06
**48h**	5.00E-06	4.84E-06	7.54E-06	1.02E-05
**72h**	1.08E-06	1.79E-06	1.61E-06	1.27E-06
	cgd5_1200			
**2h**	2.10E-04	3.69E-04	2.05E-04	8.36E-04
**6h**	3.43E-05	7.87E-05	1.34E-04	2.40E-04
**12h**	1.67E-04	4.20E-04	3.97E-04	4.14E-04
**24h**	1.33E-04	4.89E-04	7.74E-04	3.78E-04
**36h**	3.88E-05	3.42E-04	1.46E-04	3.33E-04
**48h**	7.98E-05	2.85E-04	1.74E-04	2.39E-04
**72h**	9.41E-06	8.54E-05	5.14E-05	3.42E-05
	cgd5_1210			
**2h**	1.84E-06	9.67E-06	1.38E-07	5.43E-06
**6h**	9.06E-08	1.21E-07	1.93E-07	2.14E-07
**12h**	3.79E-08	3.67E-08	2.20E-07	8.40E-07
**24h**	4.39E-07	1.86E-07	8.68E-07	4.56E-07
**36h**	2.59E-07	3.69E-07	5.88E-07	1.12E-06
**48h**	6.04E-05	6.57E-05	3.35E-05	3.18E-05
**72h**	1.15E-06	5.69E-06	2.57E-06	4.35E-06
	cgd5_1230			
**2h**	3.41E-05	1.10E-04	2.02E-04	5.35E-04
**6h**	4.31E-05	5.62E-05	1.45E-04	6.58E-05
**12h**	1.46E-05	1.14E-05	8.10E-05	3.39E-05
**24h**	2.56E-05	4.58E-04	1.55E-04	1.02E-04
**36h**	8.14E-06	6.35E-05	9.65E-05	5.40E-05
**48h**	4.05E-05	7.86E-05	9.51E-05	9.24E-05
**72h**	1.39E-06	1.43E-05	2.34E-05	8.79E-06
	cgd5_1240			
**2h**	6.00E-06	1.81E-06	6.96E-06	2.07E-06
**6h**	1.24E-06	3.81E-07	1.04E-06	1.09E-06
**12h**	1.15E-06	3.31E-07	2.49E-06	9.40E-07
**24h**	2.56E-06	5.14E-07	3.52E-06	1.45E-06
**36h**	5.17E-07	1.05E-06	1.07E-06	8.76E-07

**48h**	1.94E-06	8.10E-07	7.95E-07	6.94E-07
**72h**	5.39E-07	6.85E-07	3.15E-05	2.26E-07
	cgd8_3640			
**2h**	4.29E-06	1.55E-06	7.93E-07	4.00E-06
**6h**	6.96E-06	6.68E-06	8.20E-06	1.45E-05
**12h**	8.43E-07	9.33E-07	3.22E-06	2.17E-06
**24h**	3.22E-06	4.01E-06	5.99E-06	3.29E-06
**36h**	2.86E-06	4.22E-06	3.48E-06	5.68E-06
**48h**	4.45E-06	1.41E-06	2.42E-06	2.89E-06
**72h**	4.01E-07	4.11E-07	4.22E-07	2.06E-07
	cgd8_3650			
**2h**	6.25E-07	3.69E-07	1.05E-07	5.27E-06
**6h**	8.90E-07	1.09E-06	1.10E-06	7.76E-07
**12h**	1.37E-07	4.68E-08	2.02E-07	3.15E-08
**24h**	2.82E-07	6.14E-07	4.94E-07	6.72E-07
**36h**	7.47E-07	3.88E-07	3.29E-07	1.13E-06
**48h**	5.74E-07	1.04E-07	2.74E-07	3.46E-07
**72h**	2.17E-07	2.79E-07	2.10E-07	8.38E-08
	cgd8_3660			
**2h**	1.09E-03	3.40E-04	1.75E-04	1.25E-03
**6h**	6.56E-05	3.68E-05	1.13E-04	1.22E-04
**12h**	1.37E-04	1.21E-04	3.31E-04	5.06E-05
**24h**	3.07E-04	5.53E-04	4.51E-04	2.15E-04
**36h**	1.33E-04	1.03E-04	1.51E-04	1.85E-04
**48h**	1.99E-04	3.68E-05	2.43E-04	2.17E-04
**72h**	8.60E-05	5.31E-05	4.37E-05	5.62E-05
	cgd8_3670			
**2h**	5.60E-07	6.03E-07	3.65E-06	0.00E+00
**6h**	1.77E-07	2.07E-06	0.00E+00	0.00E+00
**12h**	7.41E-06	1.12E-04	1.91E-05	6.36E-06
**24h**	1.00E-05	4.54E-06	1.81E-05	3.16E-05
**36h**	3.36E-05	3.09E-05	4.16E-05	3.39E-05
**48h**	5.01E-04	6.88E-05	1.83E-04	7.06E-05
**72h**	7.26E-05	6.53E-05	6.99E-05	2.27E-05
	cgd8_3680			
**2h**	2.58E-08	1.69E-07	1.39E-07	2.02E-05
**6h**	6.03E-07	2.02E-06	7.37E-07	8.76E-06
**12h**	2.40E-07	1.54E-07	3.24E-07	1.29E-07
**24h**	3.12E-07	3.15E-07	1.53E-06	1.68E-06
**36h**	8.16E-07	1.26E-06	6.93E-07	8.63E-07
**48h**	2.81E-06	2.61E-06	2.69E-06	2.60E-06
**72h**	5.41E-07	3.09E-07	7.62E-07	2.72E-07
	cgd8_3690			
**2h**	9.87E-08	2.96E-07	1.31E-08	1.42E-06
**6h**	1.54E-07	3.68E-07	5.08E-07	2.33E-07
**12h**	1.09E-07	5.01E-07	4.22E-07	3.60E-08
**24h**	6.63E-07	1.56E-07	2.07E-07	4.40E-07
**36h**	2.54E-07	3.53E-07	3.15E-07	5.38E-07
**48h**	8.05E-07	2.90E-07	8.60E-07	6.20E-07
**72h**	1.22E-07	3.76E-07	3.11E-07	1.01E-07
	cgd8_3710			
**2h**	0.00E+00	3.34E-09	0.00E+00	4.47E-08
**6h**	6.20E-09	0.00E+00	0.00E+00	7.75E-10

**12h**	6.69E-07	1.30E-06	4.86E-07	1.16E-07
**24h**	6.89E-08	1.51E-07	1.17E-07	1.00E-07
**36h**	9.99E-08	2.01E-07	3.77E-07	2.62E-07
**48h**	4.80E-08	3.22E-08	1.34E-07	4.74E-08
**72h**	1.47E-08	7.53E-08	1.82E-08	1.61E-07
	cgd8_3720			
**2h**	6.21E-06	2.91E-05	4.05E-06	3.56E-04
**6h**	8.87E-06	2.80E-05	1.13E-04	1.04E-04
**12h**	8.29E-05	2.26E-04	1.74E-04	2.42E-05
**24h**	7.33E-05	1.23E-04	5.81E-05	1.88E-04
**36h**	4.16E-05	1.67E-04	2.29E-04	1.41E-04
**48h**	1.02E-04	1.26E-04	9.25E-05	5.61E-05
**72h**	5.39E-05	8.79E-05	9.90E-05	5.57E-05
	cgd8_3730			
**2h**	1.39E-08	0.00E+00	0.00E+00	1.45E-07
**6h**	0.00E+00	0.00E+00	0.00E+00	8.76E-09
**12h**	1.14E-05	1.74E-05	1.90E-05	2.20E-05
**24h**	2.36E-06	2.59E-06	8.58E-07	1.19E-06
**36h**	1.51E-06	2.40E-06	1.26E-05	1.94E-05
**48h**	6.23E-07	6.78E-07	1.34E-06	6.58E-06
**72h**	6.90E-07	8.63E-07	8.22E-07	1.16E-06
	cgd8_3740			
**2h**	3.30E-05	1.42E-05	7.46E-06	6.32E-05
**6h**	9.33E-06	1.17E-05	2.04E-05	1.52E-05
**12h**	3.23E-05	5.44E-05	2.48E-05	1.42E-05
**24h**	1.85E-05	1.61E-05	2.32E-05	3.15E-05
**36h**	8.87E-06	1.26E-05	1.01E-05	1.33E-05
**48h**	6.37E-06	3.10E-06	5.63E-06	3.16E-06
**72h**	7.98E-07	3.59E-01	2.41E-06	1.07E-06
	cgd8_3750			
**2h**	1.09E-04	5.65E-05	7.28E-05	1.20E-04
**6h**	8.94E-06	1.36E-05	2.48E-05	1.04E-04
**12h**	6.48E-05	6.62E-05	2.68E-05	6.86E-05
**24h**	7.44E-05	3.98E-05	2.04E-05	3.26E-04
**36h**	2.10E-05	2.33E-05	3.83E-05	1.82E-04
**48h**	2.07E-05	7.64E-06	2.43E-05	2.03E-05
**72h**	2.82E-06	3.26E-06	5.04E-06	2.98E-06
	cgd8_3760			
**2h**	5.74E-06	6.33E-06	1.80E-06	1.50E-03
**6h**	6.93E-07	1.39E-06	2.83E-06	4.06E-07
**12h**	2.95E-07	5.12E-07	1.01E-06	8.67E-08
**24h**	1.15E-06	1.99E-06	1.57E-06	1.09E-06
**36h**	1.88E-06	1.17E-06	1.04E-06	4.35E-05
**48h**	1.33E-06	2.68E-07	1.90E-06	3.66E-07
**72h**	5.25E-07	3.91E-07	4.82E-07	1.98E-07
	cgd5_2390			
**2h**	5.45E-06	4.75E-06	1.41E-06	2.13E-06
**6h**	8.46E-07	1.61E-06	4.89E-07	2.83E-07
**12h**	2.81E-07	2.99E-05	6.41E-07	4.26E-07
**24h**	1.65E-06	1.09E-06	1.79E-06	1.77E-06
**36h**	2.48E-07	2.14E-06	3.41E-07	1.22E-06
**48h**	6.32E-07	3.90E-07	3.21E-07	3.30E-07
**72h**	8.79E-08	1.52E-07	8.12E-08	7.27E-08

	cgd5_2400			
**2h**	0.00E+00	0.00E+00	1.31E-07	1.86E-04
**6h**	0.00E+00	0.00E+00	0.00E+00	0.00E+00
**12h**	2.46E-05	1.18E-04	2.05E-06	7.39E-05
**24h**	3.95E-05	2.84E-05	1.10E-05	2.27E-04
**36h**	1.02E-04	1.59E-05	5.14E-06	5.48E-04
**48h**	2.11E-04	1.18E-05	2.50E-05	6.54E-04
**72h**	1.51E-03	9.34E-05	2.84E-04	1.18E-03
	cgd5_2410			
**2h**	0.00E+00	1.31E-06	0.00E+00	0.00E+00
**6h**	0.00E+00	0.00E+00	0.00E+00	0.00E+00
**12h**	0.00E+00	3.97E-06	7.12E-06	1.14E-06
**24h**	1.06E-06	3.13E-06	2.59E-06	2.03E-06
**36h**	4.60E-06	9.12E-06	1.43E-05	5.07E-06
**48h**	5.02E-06	1.02E-04	1.95E-05	2.10E-05
**72h**	2.04E-05	8.79E-05	8.85E-05	5.27E-05
	cgd5_2420			
**2h**	0.00E+00	0.00E+00	1.32E-06	0.00E+00
**6h**	1.93E-04	7.78E-04	7.06E-04	2.32E-04
**12h**	5.63E-04	6.46E-04	4.16E-04	4.69E-04
**24h**	1.14E-04	8.65E-04	3.16E-03	4.87E-04
**36h**	4.43E-04	5.20E-04	1.69E-03	2.61E-03
**48h**	1.71E-04	4.22E-04	8.50E-04	2.21E-03
**72h**	3.59E-04	5.74E-04	8.81E-04	1.59E-03
	cgd5_2430			
**2h**	1.26E-05	9.01E-06	6.15E-06	1.14E-05
**6h**	1.61E-05	6.64E-05	2.88E-05	1.25E-05
**12h**	1.60E-06	1.26E-05	2.48E-05	8.24E-07
**24h**	3.56E-06	4.95E-05	2.75E-05	6.33E-06
**36h**	3.96E-06	8.11E-06	8.07E-06	1.26E-05
**48h**	1.49E-06	9.98E-06	3.16E-06	6.30E-06
**72h**	7.15E-06	8.16E-06	6.18E-06	1.20E-05
	cgd5_2440			
**2h**	2.48E-07	5.07E-08	1.72E-07	6.66E-07
**6h**	2.05E-07	5.35E-08	1.03E-07	7.62E-08
**12h**	1.03E-07	2.16E-07	9.73E-08	6.56E-08
**24h**	1.98E-07	1.02E-07	1.15E-07	2.76E-07
**36h**	1.38E-07	8.30E-08	1.01E-07	1.99E-07
**48h**	7.84E-08	1.58E-07	1.52E-07	2.50E-07
**72h**	6.13E-07	3.86E-07	2.13E-07	2.62E-07
	cgd5_2460			
**2h**	3.76E-06	5.76E-06	1.65E-05	1.61E-05
**6h**	7.86E-06	1.15E-05	7.11E-06	4.06E-06
**12h**	2.89E-06	8.85E-05	9.41E-06	2.55E-06
**24h**	4.62E-06	4.12E-05	1.32E-05	7.71E-06
**36h**	4.04E-06	1.28E-05	7.50E-06	7.01E-06
**48h**	2.40E-06	1.34E-05	7.02E-06	6.50E-06
**72h**	7.21E-06	7.50E-06	5.27E-06	3.47E-06
	cgd5_2470			
**2h**	2.06E-06	6.88E-07	1.49E-06	2.25E-06
**6h**	1.82E-06	3.72E-07	5.13E-07	5.22E-07
**12h**	6.22E-07	1.76E-06	1.55E-06	3.54E-07
**24h**	1.18E-06	1.42E-06	1.40E-06	1.50E-06

**36h**	8.70E-07	8.48E-07	4.76E-07	1.04E-06
**48h**	4.81E-07	2.13E-06	1.00E-06	1.83E-06
**72h**	3.49E-06	1.66E-06	2.27E-06	2.34E-06
	cgd5_2480			
**2h**	3.34E-08	0.00E+00	9.58E-09	7.38E-08
**6h**	2.19E-05	5.92E-08	1.70E-07	2.58E-07
**12h**	4.31E-08	6.97E-08	1.86E-08	5.67E-09
**24h**	8.82E-08	4.17E-07	2.87E-07	1.51E-07
**36h**	2.38E-07	1.11E-07	7.55E-08	8.45E-08
**48h**	9.08E-08	1.91E-07	1.35E-07	3.05E-07
**72h**	6.96E-07	2.23E-07	3.08E-07	3.41E-07
	cgd5_2490			
**2h**	0.00E+00	0.00E+00	2.67E-06	3.87E-07
**6h**	5.53E-06	0.00E+00	4.17E-06	2.73E-06
**12h**	2.71E-06	8.82E-06	2.35E-06	1.60E-06
**24h**	1.50E-06	3.79E-05	1.69E-05	3.11E-06
**36h**	4.16E-06	1.82E-05	2.42E-05	8.18E-06
**48h**	2.66E-06	1.61E-05	1.97E-05	1.07E-05
**72h**	4.08E-06	4.40E-05	1.94E-05	6.37E-06
	cgd5_2500			
**2h**	0.00E+00	7.46E-07	1.36E-07	1.91E-07
**6h**	7.26E-07	1.61E-05	1.75E-06	2.71E-07
**12h**	1.23E-07	2.18E-06	1.69E-07	6.75E-08
**24h**	2.83E-07	9.78E-06	2.01E-06	3.11E-07
**36h**	1.31E-06	5.40E-06	2.17E-06	1.81E-06
**48h**	1.04E-06	8.42E-05	2.77E-06	1.60E-06
**72h**	7.10E-07	1.14E-06	1.96E-06	2.08E-06
	cgd5_2510			
**2h**	0.00E+00	2.71E-05	1.71E-05	2.22E-05
**6h**	9.50E-06	7.99E-05	4.84E-05	1.30E-05
**12h**	1.46E-06	8.73E-05	1.93E-05	1.21E-06
**24h**	3.78E-06	4.53E-04	1.01E-04	1.21E-05
**36h**	1.96E-05	3.44E-04	8.05E-05	2.02E-05
**48h**	8.00E-06	1.38E-04	6.25E-05	2.55E-05
**72h**	2.92E-05	1.80E-04	7.72E-04	6.67E-05
	cgd6_640			
**2h**	4.22E-06	6.21E-04	2.09E-06	4.78E-05
**6h**	9.65E-05	4.55E-04	9.99E-06	1.35E-05
**12h**	4.27E-03	5.19E-04	6.04E-06	5.70E-05
**24h**	2.36E-04	5.72E-04	8.40E-06	3.67E-05
**36h**	3.11E-04	1.14E-04	1.02E-05	5.88E-05
**48h**	3.32E-04	3.84E-05	5.91E-06	6.59E-05
**72h**	4.47E-05	5.21E-04	4.37E-06	2.84E-05
	cgd6_670			
**2h**	0.00E+00	0.00E+00	0.00E+00	0.00E+00
**6h**	0.00E+00	0.00E+00	0.00E+00	0.00E+00
**12h**	0.00E+00	4.05E-09	2.16E-09	5.04E-09
**24h**	1.77E-08	1.38E-08	9.06E-09	5.69E-09
**36h**	2.59E-08	2.93E-08	8.76E-08	2.13E-08
**48h**	2.33E-06	1.83E-07	2.16E-07	2.69E-07
**72h**	1.04E-06	8.87E-07	7.22E-07	6.88E-07
	cgd6_680			
**2h**	4.62E-07	2.36E-05	2.02E-07	2.77E-07

**6h**	8.61E-07	2.69E-07	7.74E-08	5.18E-08
**12h**	2.23E-06	3.34E-07	6.98E-08	9.23E-08
**24h**	9.30E-07	4.57E-07	2.25E-07	2.32E-07
**36h**	1.67E-06	2.97E-07	8.50E-07	4.21E-07
**48h**	3.35E-06	1.66E-07	5.91E-07	4.54E-07
**72h**	6.29E-07	4.88E-07	3.51E-07	5.13E-07
	cgd6_690			
**2h**	5.58E-09	8.31E-10	0.00E+00	1.03E-09
**6h**	1.35E-05	1.43E-06	1.54E-06	1.82E-06
**12h**	6.91E-08	1.97E-07	1.16E-07	7.58E-08
**24h**	5.66E-06	3.33E-06	4.30E-07	3.50E-07
**36h**	4.80E-06	4.20E-07	1.63E-06	8.02E-07
**48h**	1.46E-06	2.17E-07	4.81E-07	4.29E-07
**72h**	4.71E-07	6.19E-07	4.76E-07	4.52E-07
	cgd6_710			
**2h**	3.90E-08	0.00E+00	0.00E+00	0.00E+00
**6h**	0.00E+00	0.00E+00	1.77E-09	0.00E+00
**12h**	0.00E+00	1.98E-08	6.28E-09	1.02E-09
**24h**	9.90E-09	3.43E-07	2.18E-09	3.26E-09
**36h**	5.28E-07	1.15E-07	1.31E-07	3.59E-08
**48h**	5.35E-05	5.88E-06	3.71E-05	3.88E-05
**72h**	7.19E-06	3.02E-06	2.18E-06	6.24E-06
	cgd6_720			
**2h**	0.00E+00	3.68E-05	1.51E-06	4.42E-06
**6h**	5.02E-04	2.41E-04	1.24E-05	3.43E-05
**12h**	7.97E-04	2.02E-05	3.53E-06	4.51E-05
**24h**	1.01E-03	4.00E-04	1.45E-05	4.79E-05
**36h**	7.12E-04	7.41E-05	8.34E-05	1.35E-04
**48h**	5.86E-04	1.10E-04	3.83E-05	1.81E-04
**72h**	8.24E-04	1.09E-04	6.91E-05	1.99E-04
	cgd6_740			
**2h**	0.00E+00	0.00E+00	0.00E+00	0.00E+00
**6h**	0.00E+00	8.91E-07	0.00E+00	0.00E+00
**12h**	0.00E+00	3.86E-06	0.00E+00	3.78E-08
**24h**	1.54E-06	6.79E-06	8.35E-11	1.77E-09
**36h**	4.23E-07	2.64E-06	1.75E-07	1.27E-07
**48h**	2.19E-03	1.05E-04	1.56E-05	1.15E-04
**72h**	5.68E-04	5.48E-04	1.20E-04	4.98E-04
	cgd6_760			
**2h**	0.00E+00	3.81E-09	6.08E-08	1.51E-08
**6h**	2.15E-07	4.93E-07	7.06E-08	1.61E-08
**12h**	5.11E-05	8.52E-06	4.63E-06	4.04E-06
**24h**	2.60E-06	8.16E-06	1.75E-06	1.17E-06
**36h**	3.37E-05	4.34E-06	3.49E-06	2.03E-06
**48h**	3.84E-06	4.59E-06	0.00E+00	1.26E-06
**72h**	8.19E-06	2.22E-06	2.29E-06	1.27E-06
	cgd6_770			
**2h**	3.22E-08	3.01E-07	2.21E-07	1.80E-07
**6h**	7.24E-07	6.50E-08	6.44E-08	5.91E-08
**12h**	1.88E-07	0.00E+00	7.72E-08	6.21E-09
**24h**	4.30E-07	8.36E-08	3.75E-08	4.05E-08
**36h**	6.40E-07	6.16E-06	6.65E-07	3.90E-07
**48h**	3.46E-05	2.97E-04	2.63E-05	1.53E-05

**72h**	6.32E-06	1.13E-05	3.44E-06	6.60E-06
	cgd6_780			
**2h**	7.60E-05	4.06E-04	1.58E-05	3.95E-05
**6h**	4.36E-07	1.33E-07	0.00E+00	3.33E-08
**12h**	4.75E-06	1.86E-06	5.36E-07	4.21E-07
**24h**	3.08E-06	5.50E-06	3.63E-07	1.98E-07
**36h**	1.06E-05	4.48E-06	8.09E-07	7.61E-07
**48h**	1.32E-05	2.79E-06	1.48E-06	1.04E-06
**72h**	1.26E-05	1.36E-05	0.00E+00	1.61E-06
	cgd6_800			
**2h**	0.00E+00	0.00E+00	0.00E+00	1.48E-08
**6h**	1.44E-07	2.27E-09	5.56E-09	0.00E+00
**12h**	2.92E-07	1.70E-07	7.99E-08	4.56E-08
**24h**	5.24E-07	8.77E-07	0.00E+00	6.68E-08
**36h**	4.10E-06	6.27E-07	5.07E-07	1.00E-06
**48h**	8.06E-06	3.54E-06	1.13E-06	3.71E-06
**72h**	9.57E-06	7.76E-06	7.15E-07	4.00E-06
	cgd6_820			
**2h**	0.00E+00	0.00E+00	0.00E+00	0.00E+00
**6h**	0.00E+00	0.00E+00	0.00E+00	0.00E+00
**12h**	1.30E-07	1.48E-09	0.00E+00	3.49E-09
**24h**	1.58E-07	2.18E-07	2.62E-09	8.94E-10
**36h**	2.55E-07	2.31E-07	6.35E-08	3.59E-07
**48h**	5.02E-05	8.41E-05	1.65E-06	3.83E-06
**72h**	8.70E-05	8.36E-04	3.00E-06	1.71E-05
	cgd6_840			
**2h**	3.80E-05	4.78E-08	0.00E+00	0.00E+00
**6h**	1.69E-05	1.60E-05	1.37E-05	3.68E-06
**12h**	5.43E-05	1.57E-06	1.19E-07	3.41E-07
**24h**	6.22E-05	2.85E-05	3.62E-06	1.20E-06
**36h**	1.47E-05	6.52E-06	3.28E-06	1.97E-06
**48h**	2.50E-05	5.37E-06	1.32E-06	6.18E-07
**72h**	6.39E-06	4.65E-07	4.24E-07	6.80E-07
	cgd6_870			
**2h**	0.00E+00	4.26E-08	6.77E-08	5.19E-08
**6h**	0.00E+00	1.24E-06	2.49E-06	5.40E-07
**12h**	0.00E+00	4.57E-07	5.09E-08	4.59E-09
**24h**	1.34E-08	3.55E-06	5.61E-07	4.36E-07
**36h**	1.04E-08	7.97E-07	3.95E-07	3.43E-07
**48h**	2.55E-07	1.41E-06	2.30E-07	4.10E-07
**72h**	8.53E-07	1.25E-07	1.17E-07	2.40E-07
	cgd6_890			
**2h**	2.72E-07	0.00E+00	0.00E+00	0.00E+00
**6h**	2.87E-07	0.00E+00	0.00E+00	0.00E+00
**12h**	2.16E-07	2.62E-04	4.83E-06	2.16E-06
**24h**	3.09E-07	5.90E-05	3.59E-07	3.31E-06
**36h**	5.06E-07	4.64E-05	3.19E-06	9.36E-07
**48h**	2.78E-07	6.28E-05	1.31E-06	4.16E-06
**72h**	2.89E-07	6.27E-05	3.99E-06	5.05E-06
	cgd6_900			
**2h**	0.00E+00	0.00E+00	0.00E+00	0.00E+00
**6h**	1.84E-06	5.54E-05	1.07E-03	2.27E-03
**12h**	2.22E-07	9.03E-06	1.27E-05	3.54E-04

**24h**	1.14E-07	2.46E-05	1.82E-04	2.13E-05
**36h**	1.87E-06	3.01E-05	2.77E-04	1.36E-04
**48h**	2.08E-07	5.53E-05	4.00E-04	7.58E-05
**72h**	4.64E-07	3.96E-05	4.86E-05	5.40E-04
	cgd6_910			
**2h**	0.00E+00	0.00E+00	1.46E-09	2.78E-08
**6h**	5.65E-09	2.35E-07	1.35E-07	7.61E-08
**12h**	0.00E+00	0.00E+00	2.21E-09	1.43E-08
**24h**	1.41E-09	6.84E-08	1.26E-08	2.11E-09
**36h**	2.02E-07	8.82E-08	4.13E-08	3.00E-08
**48h**	1.18E-05	2.59E-08	2.20E-08	2.18E-08
**72h**	1.65E-06	3.29E-08	3.08E-08	1.13E-08
	cgd6_920			
**2h**	6.59E-07	1.86E-05	2.01E-06	0.00E+00
**6h**	1.08E-04	3.57E-04	2.09E-03	2.25E-03
**12h**	5.60E-04	1.02E-04	1.47E-04	4.64E-04
**24h**	6.04E-05	2.96E-04	1.85E-04	1.16E-03
**36h**	9.62E-05	1.77E-04	7.28E-04	1.07E-03
**48h**	9.37E-05	8.11E-05	3.17E-04	3.21E-04
**72h**	5.65E-05	1.43E-05	8.38E-05	2.90E-04
	cgd6_930			
**2h**	0.00E+00	1.22E-04	1.49E-05	3.59E-04
**6h**	0.00E+00	1.25E-04	2.81E-06	2.75E-05
**12h**	4.89E-08	3.05E-05	1.09E-05	5.06E-05
**24h**	8.74E-10	5.66E-05	1.19E-05	1.36E-04
**36h**	1.47E-07	2.40E-05	1.77E-05	5.92E-05
**48h**	3.97E-05	1.71E-05	1.51E-05	1.16E-04
**72h**	1.15E-04	2.67E-06	1.44E-05	1.41E-04
	cgd6_940			
**2h**	6.47E-08	1.56E-04	2.83E-05	9.21E-06
**6h**	1.67E-07	2.31E-05	4.16E-06	5.07E-06
**12h**	7.44E-06	7.07E-04	1.05E-05	3.00E-05
**24h**	1.05E-06	7.68E-05	4.72E-06	5.94E-05
**36h**	2.01E-06	8.21E-05	8.48E-06	2.31E-05
**48h**	7.96E-07	1.55E-04	4.04E-06	1.41E-05
**72h**	5.00E-07	1.39E-05	3.60E-06	3.70E-06
	cgd6_950			
**2h**	1.89E-07	0.00E+00	0.00E+00	0.00E+00
**6h**	2.77E-07	0.00E+00	0.00E+00	0.00E+00
**12h**	4.92E-08	1.17E-04	2.29E-05	7.19E-05
**24h**	3.41E-08	6.33E-06	1.60E-06	1.88E-05
**36h**	4.07E-07	4.09E-05	3.24E-05	1.29E-05
**48h**	1.74E-05	1.30E-05	5.98E-06	1.26E-05
**72h**	7.44E-07	6.25E-07	1.69E-06	9.25E-07
	cgd6_960			
**2h**	2.96E-05	5.32E-05	5.45E-05	3.27E-05
**6h**	2.62E-07	1.56E-04	5.79E-05	2.52E-05
**12h**	5.79E-07	1.19E-03	1.01E-04	1.00E-04
**24h**	7.30E-07	2.88E-04	3.43E-05	8.51E-05
**36h**	3.96E-07	2.68E-04	1.00E-04	3.36E-05
**48h**	9.00E-07	1.85E-04	5.12E-05	2.53E-05
**72h**	3.48E-07	1.80E-04	3.00E-06	9.70E-06
	cgd6_970			

**2h**	0.00E+00	0.00E+00	0.00E+00	0.00E+00
**6h**	3.81E-08	2.31E-07	1.57E-08	1.19E-09
**12h**	4.69E-08	1.14E-08	5.48E-09	4.25E-09
**24h**	1.63E-07	1.00E-07	2.52E-08	8.61E-09
**36h**	3.55E-07	1.60E-07	6.10E-08	7.59E-08
**48h**	1.83E-06	3.63E-07	1.45E-07	6.78E-08
**72h**	4.99E-07	1.72E-07	5.48E-08	1.03E-07
	cgd6_980			
**2h**	0.00E+00	0.00E+00	0.00E+00	0.00E+00
**6h**	0.00E+00	5.50E-06	5.68E-07	1.41E-07
**12h**	1.42E-09	1.37E-05	1.24E-07	1.05E-08
**24h**	1.63E-09	8.90E-06	6.01E-07	5.55E-07
**36h**	4.47E-08	1.38E-05	1.37E-06	1.96E-06
**48h**	8.57E-06	4.43E-06	1.04E-06	9.59E-07
**72h**	3.90E-06	1.28E-05	2.05E-07	1.67E-07
	cgd6_990			
**2h**	9.05E-05	5.26E-03	5.73E-06	1.43E-04
**6h**	1.89E-04	3.72E-03	1.65E-04	2.28E-04
**12h**	2.35E-05	2.34E-06	5.21E-05	4.13E-05
**24h**	1.01E-04	1.45E-05	2.08E-04	2.34E-04
**36h**	1.53E-04	1.79E-05	1.38E-04	1.04E-04
**48h**	1.79E-04	2.33E-05	3.81E-04	2.05E-04
**72h**	2.98E-05	1.49E-06	5.01E-07	3.52E-05
	cgd6_1010			
**2h**	2.14E-08	0.00E+00	0.00E+00	2.85E-07
**6h**	1.68E-06	1.64E-06	4.29E-06	3.84E-07
**12h**	6.23E-07	3.45E-07	6.92E-07	6.65E-07
**24h**	1.12E-06	5.58E-07	5.13E-06	9.30E-06
**36h**	7.49E-07	4.83E-07	1.89E-06	3.60E-06
**48h**	5.93E-06	9.22E-07	1.13E-05	1.61E-05
**72h**	7.12E-06	0.00E+00	1.02E-05	1.86E-05
	cgd6_1020			
**2h**	1.65E-06	6.31E-07	1.04E-06	1.63E-06
**6h**	1.60E-06	3.24E-06	6.99E-06	1.11E-06
**12h**	2.94E-07	2.97E-07	4.61E-06	1.06E-06
**24h**	1.35E-06	5.34E-07	8.02E-06	2.25E-06
**36h**	7.76E-07	4.12E-07	2.44E-06	4.12E-07
**48h**	6.58E-07	1.92E-07	2.34E-06	5.69E-07
**72h**	2.28E-07	0.00E+00	3.87E-07	3.21E-07
	cgd6_1030			
**2h**	0.00E+00	0.00E+00	0.00E+00	0.00E+00
**6h**	0.00E+00	0.00E+00	0.00E+00	0.00E+00
**12h**	4.48E-05	1.43E-05	8.30E-05	4.69E-04
**24h**	1.68E-05	3.36E-06	2.21E-05	2.18E-04
**36h**	4.18E-05	6.06E-06	9.56E-05	3.01E-04
**48h**	2.72E-05	2.13E-06	1.67E-05	6.69E-05
**72h**	7.63E-06	0.00E+00	3.17E-06	7.84E-05
	cgd6_1060			
**2h**	0.00E+00	0.00E+00	0.00E+00	0.00E+00
**6h**	2.79E-07	3.25E-07	1.76E-06	4.84E-07
**12h**	1.41E-07	1.13E-07	3.29E-07	1.36E-07
**24h**	2.02E-07	1.42E-07	2.93E-06	1.92E-06
**36h**	8.57E-07	5.30E-07	3.42E-06	5.98E-07

**48h**	1.45E-06	3.57E-07	2.08E-06	1.44E-06
**72h**	2.37E-06	5.31E-07	2.87E-06	5.12E-06
	cgd6_1070			
**2h**	8.25E-09	0.00E+00	3.69E-08	0.00E+00
**6h**	2.62E-05	2.60E-05	1.24E-04	1.85E-05
**12h**	9.46E-07	5.75E-07	7.09E-06	1.35E-05
**24h**	2.90E-06	1.61E-06	1.08E-04	9.70E-05
**36h**	8.15E-06	5.78E-06	5.68E-05	2.39E-05
**48h**	4.40E-06	1.45E-06	2.18E-05	4.27E-05
**72h**	6.58E-06	1.71E-06	1.90E-05	1.96E-05
	cgd6_1080			
**2h**	4.17E-08	1.53E-09	1.55E-07	3.70E-08
**6h**	1.28E-06	3.00E-06	3.28E-06	2.67E-07
**12h**	3.53E-03	6.92E-03	4.99E-03	1.76E-03
**24h**	9.32E-04	5.53E-04	2.72E-03	9.96E-04
**36h**	1.32E-03	3.45E-04	2.43E-03	5.50E-04
**48h**	7.74E-04	5.56E-04	4.36E-03	7.90E-04
**72h**	1.21E-03	1.65E-04	8.96E-04	2.03E-04
	cgd6_1090			
**2h**	0.00E+00	0.00E+00	0.00E+00	0.00E+00
**6h**	0.00E+00	0.00E+00	0.00E+00	0.00E+00
**12h**	1.14E-05	6.81E-06	2.59E-05	1.43E-05
**24h**	2.22E-06	9.34E-07	8.79E-06	2.48E-06
**36h**	4.30E-06	2.70E-06	3.33E-05	8.98E-06
**48h**	1.25E-06	4.50E-07	5.33E-06	1.90E-06
**72h**	5.27E-07	5.11E-07	6.42E-07	1.51E-06
	cgd6_1100			
**2h**	0.00E+00	0.00E+00	0.00E+00	0.00E+00
**6h**	0.00E+00	0.00E+00	4.81E-08	0.00E+00
**12h**	3.74E-07	2.21E-07	9.72E-07	9.25E-07
**24h**	1.62E-07	8.88E-08	1.44E-05	5.18E-06
**36h**	2.68E-06	1.15E-06	5.47E-06	1.24E-06
**48h**	2.93E-06	1.62E-06	6.83E-06	7.41E-06
**72h**	3.21E-07	1.09E-07	1.15E-07	3.54E-07
	cgd6_1110			
**2h**	7.24E-07	0.00E+00	0.00E+00	6.84E-06
**6h**	3.98E-06	4.32E-06	2.61E-05	2.86E-05
**12h**	5.52E-05	1.56E-04	1.37E-04	3.17E-04
**24h**	1.46E-05	5.23E-06	9.69E-05	3.25E-04
**36h**	3.52E-05	9.96E-06	1.18E-04	5.09E-04
**48h**	2.40E-05	7.40E-06	5.97E-05	1.91E-04
**72h**	3.94E-06	8.68E-07	1.18E-06	7.39E-05
	cgd6_1120			
**2h**	2.11E-05	4.67E-06	6.62E-06	8.54E-05
**6h**	1.44E-05	1.25E-05	6.46E-05	1.95E-05
**12h**	2.48E-06	6.48E-07	1.63E-05	5.23E-05
**24h**	4.59E-06	3.98E-06	3.71E-05	6.44E-05
**36h**	3.74E-06	1.98E-06	7.77E-06	1.37E-05
**48h**	5.61E-06	1.18E-06	1.02E-05	2.24E-05
**72h**	7.81E-07	1.91E-07	5.48E-07	1.35E-05
	cgd6_1130			
**2h**	6.52E-07	0.00E+00	0.00E+00	0.00E+00
**6h**	2.64E-04	7.07E-05	1.22E-03	2.33E-04

**12h**	5.40E-05	0.00E+00	1.65E-04	6.05E-05
**24h**	8.31E-05	2.22E-05	6.63E-03	1.07E-03
**36h**	5.65E-04	2.45E-04	4.07E-03	1.37E-04
**48h**	1.34E-04	2.13E-04	1.20E-03	1.87E-04
**72h**	2.04E-05	2.89E-05	4.96E-05	1.23E-05
	cgd5_2870			
**2h**	0.00E+00	0.00E+00	0.00E+00	1.27E-06
**6h**	0.00E+00	1.70E-05	2.80E-07	2.63E-06
**12h**	1.67E-05	2.07E-05	2.26E-07	4.10E-06
**24h**	6.32E-06	4.43E-05	5.31E-07	5.46E-06
**36h**	5.90E-05	3.64E-05	2.34E-06	1.68E-06
**48h**	3.09E-04	1.92E-04	3.83E-05	1.87E-06
**72h**	7.75E-05	9.83E-06	1.09E-06	2.89E-07
	cgd5_2890			
**2h**	1.71E-04	1.50E-04	5.36E-05	1.51E-06
**6h**	8.40E-05	1.23E-04	8.10E-05	0.00E+00
**12h**	2.80E-05	7.63E-04	4.11E-05	1.64E-06
**24h**	5.06E-04	1.25E-03	3.03E-05	2.96E-06
**36h**	1.94E-04	1.28E-04	4.26E-05	1.29E-05
**48h**	8.36E-05	1.90E-04	1.32E-05	2.29E-05
**72h**	3.08E-04	1.06E-04	8.51E-05	2.22E-05
	cgd5_2920			
**2h**	2.35E-06	0.00E+00	3.20E-07	2.15E-09
**6h**	2.66E-06	0.00E+00	6.86E-07	2.02E-09
**12h**	0.00E+00	0.00E+00	7.43E-08	1.47E-07
**24h**	1.41E-06	4.26E-06	8.96E-07	1.91E-07
**36h**	1.26E-06	1.53E-06	1.27E-06	1.43E-06
**48h**	2.90E-06	9.38E-06	2.77E-06	2.03E-06
**72h**	7.35E-06	7.25E-06	6.43E-06	1.34E-06
	cgd5_2940			
**2h**	0.00E+00	0.00E+00	6.07E-10	4.09E-03
**6h**	0.00E+00	0.00E+00	0.00E+00	4.59E-04
**12h**	5.14E-09	0.00E+00	4.93E-09	5.48E-04
**24h**	2.07E-08	1.39E-07	1.00E-09	1.38E-03
**36h**	1.67E-07	7.21E-08	1.02E-08	4.38E-04
**48h**	5.23E-08	2.86E-07	2.51E-08	2.16E-04
**72h**	7.92E-08	6.53E-07	3.76E-08	9.45E-05
	cgd5_2950			
**2h**	2.99E-05	4.26E-05	5.73E-05	1.92E-06
**6h**	1.21E-05	2.59E-05	1.17E-05	6.39E-07
**12h**	1.10E-05	3.29E-03	3.65E-05	1.03E-06
**24h**	1.42E-04	3.24E-04	8.73E-06	2.29E-06
**36h**	6.56E-05	5.28E-05	1.11E-05	1.61E-06
**48h**	2.36E-05	8.58E-05	9.29E-06	1.21E-06
**72h**	1.44E-05	4.43E-05	2.58E-06	3.00E-07
	cgd5_2960			
**2h**	1.08E-05	4.11E-06	1.32E-05	2.28E-06
**6h**	4.21E-06	3.08E-06	1.03E-05	8.57E-07
**12h**	8.04E-07	1.28E-05	8.59E-06	3.16E-06
**24h**	1.10E-05	2.29E-05	4.25E-06	3.71E-06
**36h**	5.71E-06	4.52E-06	4.00E-06	7.05E-07
**48h**	2.75E-06	5.71E-06	2.30E-06	7.07E-07
**72h**	2.11E-06	8.74E-07	1.86E-05	1.14E-07

	cgd5_2970			
**2h**	2.98E-06	8.30E-07	5.41E-06	1.28E-04
**6h**	2.95E-06	2.43E-06	8.51E-06	6.20E-05
**12h**	2.64E-06	6.14E-06	2.56E-06	1.57E-03
**24h**	4.95E-06	7.09E-06	2.75E-06	6.95E-04
**36h**	3.87E-06	9.61E-07	1.45E-06	2.75E-04
**48h**	2.46E-06	1.95E-06	2.42E-06	5.66E-04
**72h**	8.52E-07	1.11E-06	2.74E-07	2.35E-04
	cgd5_2980			
**2h**	4.98E-05	2.40E-05	3.61E-05	2.00E-03
**6h**	5.36E-05	3.35E-05	6.62E-05	1.07E-03
**12h**	5.42E-06	5.85E-05	1.77E-05	1.15E-02
**24h**	5.63E-05	2.00E-04	3.20E-05	5.99E-03
**36h**	3.86E-05	2.85E-05	3.91E-06	1.00E-03
**48h**	3.10E-05	2.96E-05	5.39E-06	8.40E-04
**72h**	6.28E-06	1.37E-05	7.13E-07	4.16E-04
	cgd5_3010			
**2h**	2.08E-05	0.00E+00	1.05E-04	2.53E-06
**6h**	7.57E-04	7.22E-05	1.05E-04	1.06E-07
**12h**	1.02E-04	1.12E-05	5.04E-06	2.19E-05
**24h**	4.06E-04	5.30E-05	4.16E-05	8.88E-06
**36h**	9.95E-05	3.70E-05	2.47E-05	1.20E-05
**48h**	4.19E-05	8.47E-06	2.11E-05	5.96E-06
**72h**	5.64E-05	5.85E-05	1.58E-05	3.47E-06
	cgd5_3020			
**2h**	5.11E-06	0.00E+00	1.42E-07	0.00E+00
**6h**	8.70E-07	1.57E-05	7.40E-08	0.00E+00
**12h**	0.00E+00	1.56E-05	9.31E-08	4.40E-03
**24h**	7.88E-07	1.63E-05	8.98E-07	7.86E-04
**36h**	9.16E-06	2.46E-05	8.22E-07	6.44E-04
**48h**	1.65E-06	2.51E-05	6.14E-07	5.93E-04
**72h**	5.16E-06	7.61E-06	6.54E-05	3.54E-04
	cgd5_3080			
**2h**	0.00E+00	3.38E-07	4.40E-08	2.57E-08
**6h**	2.52E-08	1.19E-07	7.36E-08	9.43E-07
**12h**	0.00E+00	2.43E-07	5.22E-09	1.10E-06
**24h**	5.94E-08	1.15E-07	9.02E-08	7.48E-07
**36h**	2.37E-07	9.12E-06	2.39E-07	3.07E-06
**48h**	5.03E-06	5.31E-06	1.28E-06	1.22E-06
**72h**	2.48E-06	1.39E-06	2.41E-06	4.00E-06
	cgd6_1140			
**2h**	1.13E-05	0.00E+00	0.00E+00	0.00E+00
**6h**	2.99E-05	5.67E-03	1.16E-03	1.39E-03
**12h**	9.72E-04	1.29E-01	1.92E-02	5.30E-03
**24h**	6.62E-04	1.03E-02	8.63E-03	2.24E-03
**36h**	2.84E-04	6.82E-03	1.18E-02	4.00E-03
**48h**	2.07E-04	1.56E-02	1.01E-02	1.72E-04
**72h**	1.14E-04	4.82E-03	1.60E-03	1.43E-03
	cgd6_1160			
**2h**	1.96E-07	0.00E+00	1.03E-08	4.92E-08
**6h**	3.07E-05	1.29E-04	2.33E-04	3.90E-05
**12h**	9.35E-07	8.66E-06	2.00E-06	1.44E-06
**24h**	1.15E-05	3.67E-05	6.13E-05	1.03E-05

**36h**	3.00E-06	4.66E-06	6.35E-06	7.78E-06
**48h**	5.72E-06	3.75E-06	3.13E-06	3.55E-06
**72h**	1.73E-06	1.56E-06	9.04E-07	8.36E-07
	cgd6_1170			
**2h**	0.00E+00	0.00E+00	9.78E-07	4.05E-06
**6h**	0.00E+00	0.00E+00	3.56E-07	1.20E-06
**12h**	3.79E-04	4.02E-03	2.70E-04	2.05E-03
**24h**	3.41E-04	1.03E-03	2.31E-04	5.10E-04
**36h**	1.78E-04	1.69E-03	2.39E-04	4.13E-04
**48h**	9.74E-05	3.56E-04	1.16E-04	4.46E-04
**72h**	1.01E-04	1.20E-05	2.23E-05	6.83E-05
	cgd6_1180			
**2h**	0.00E+00	0.00E+00	1.16E-04	0.00E+00
**6h**	0.00E+00	0.00E+00	0.00E+00	0.00E+00
**12h**	2.29E-05	9.80E-05	7.89E-06	8.39E-06
**24h**	2.96E-05	8.29E-06	1.61E-06	6.58E-06
**36h**	2.49E-05	6.07E-05	3.93E-05	7.65E-06
**48h**	9.24E-06	8.90E-06	1.91E-05	6.43E-06
**72h**	3.37E-06	2.12E-06	1.98E-06	8.58E-07
	cgd6_1200			
**2h**	2.24E-05	4.51E-06	2.91E-06	1.48E-06
**6h**	2.30E-05	4.15E-06	6.44E-06	4.52E-06
**12h**	1.22E-06	1.05E-05	2.16E-06	1.38E-06
**24h**	2.05E-05	8.37E-06	2.17E-06	3.82E-06
**36h**	4.64E-06	9.70E-06	1.44E-06	2.41E-06
**48h**	1.92E-06	1.83E-06	2.38E-07	6.19E-07
**72h**	1.73E-06	1.67E-06	1.34E-06	4.30E-07
	cgd6_1230			
**2h**	2.10E-06	3.39E-06	2.11E-06	9.95E-07
**6h**	2.09E-07	3.06E-07	8.98E-07	1.94E-07
**12h**	7.66E-07	1.38E-05	1.38E-06	9.53E-07
**24h**	2.40E-06	1.47E-06	3.12E-07	8.34E-07
**36h**	8.66E-07	3.62E-06	2.44E-06	5.79E-07
**48h**	3.71E-07	1.51E-06	2.04E-07	3.93E-07
**72h**	5.02E-07	1.34E-06	1.02E-06	2.43E-07
	cgd6_1270			
**2h**	1.68E-07	0.00E+00	2.89E-08	0.00E+00
**6h**	4.57E-06	9.17E-06	3.86E-06	2.00E-06
**12h**	9.14E-07	9.91E-07	1.42E-06	2.38E-07
**24h**	3.66E-06	9.06E-06	2.25E-06	6.78E-07
**36h**	5.38E-06	1.39E-05	8.18E-06	1.26E-06
**48h**	5.84E-06	6.77E-06	1.13E-06	2.70E-06
**72h**	8.25E-07	8.15E-07	5.37E-07	3.61E-07
	cgd6_1280			
**2h**	3.32E-09	2.81E-08	1.37E-09	0.00E+00
**6h**	1.80E-08	2.52E-07	4.58E-08	2.33E-08
**12h**	3.59E-09	1.15E-07	3.12E-08	1.12E-08
**24h**	4.61E-08	6.32E-07	1.26E-07	6.00E-08
**36h**	1.30E-07	6.53E-07	2.44E-07	1.08E-07
**48h**	1.25E-07	5.72E-07	2.58E-07	8.39E-08
**72h**	5.06E-08	1.04E-07	2.40E-07	6.05E-08
	cgd6_1300			
**2h**	1.97E-06	5.04E-07	1.12E-07	1.18E-07

**6h**	1.49E-06	3.10E-06	1.02E-06	2.17E-06
**12h**	3.65E-09	3.76E-06	1.90E-07	1.03E-07
**24h**	4.22E-07	1.87E-06	3.78E-07	7.14E-07
**36h**	7.10E-07	1.61E-06	7.91E-07	6.15E-07
**48h**	6.70E-07	1.86E-06	1.10E-06	4.67E-07
**72h**	1.62E-06	4.20E-06	1.83E-06	2.24E-06
	cgd6_1310			
**2h**	0.00E+00	0.00E+00	0.00E+00	0.00E+00
**6h**	0.00E+00	0.00E+00	0.00E+00	0.00E+00
**12h**	4.73E-07	1.25E-06	1.17E-06	5.29E-07
**24h**	6.96E-07	5.61E-07	1.70E-07	1.82E-07
**36h**	2.08E-06	2.19E-06	1.41E-06	6.43E-07
**48h**	1.63E-06	2.27E-06	1.51E-06	3.34E-07
**72h**	3.73E-06	1.14E-06	1.11E-05	9.10E-07
	cgd6_1320			
**2h**	2.89E-07	1.55E-08	6.10E-08	1.86E-07
**6h**	8.47E-08	6.31E-07	3.45E-07	7.15E-08
**12h**	1.29E-06	4.70E-07	1.61E-07	4.29E-08
**24h**	7.35E-07	1.25E-06	1.02E-06	2.20E-07
**36h**	8.35E-07	1.79E-06	7.67E-07	5.24E-07
**48h**	1.08E-06	1.33E-06	1.45E-06	4.19E-07
**72h**	5.72E-07	2.27E-07	2.21E-07	2.07E-07
	cgd6_1330			
**2h**	7.16E-07	1.06E-07	4.16E-07	4.28E-08
**6h**	7.11E-07	2.00E-06	4.69E-07	4.08E-07
**12h**	1.63E-07	1.40E-06	4.49E-07	3.18E-07
**24h**	1.43E-06	9.68E-07	6.22E-07	4.97E-07
**36h**	1.39E-06	1.06E-06	7.50E-07	5.86E-07
**48h**	2.17E-06	4.97E-06	9.89E-06	1.97E-06
**72h**	1.00E-06	3.99E-07	6.64E-07	2.23E-07
	cgd6_1340			
**2h**	8.30E-08	0.00E+00	8.64E-09	7.84E-08
**6h**	4.32E-08	1.35E-06	2.83E-07	1.69E-07
**12h**	7.67E-09	6.38E-08	1.15E-08	0.00E+00
**24h**	6.52E-08	1.91E-07	2.28E-07	4.47E-08
**36h**	3.03E-08	7.37E-08	1.39E-07	9.57E-08
**48h**	5.39E-07	1.38E-06	1.07E-06	2.35E-07
**72h**	1.10E-06	1.40E-06	1.74E-06	5.03E-07
	cgd6_1350			
**2h**	3.26E-06	3.22E-06	3.90E-07	1.70E-06
**6h**	2.67E-06	2.42E-05	4.61E-06	2.67E-06
**12h**	8.90E-08	1.00E-05	1.15E-06	1.04E-07
**24h**	1.95E-06	1.17E-05	4.58E-06	3.36E-06
**36h**	8.28E-07	3.58E-06	1.95E-06	1.12E-06
**48h**	1.28E-06	6.54E-06	3.45E-06	1.79E-06
**72h**	3.68E-07	4.53E-07	5.14E-07	7.08E-07
	cgd6_1370			
**2h**	5.04E-04	0.00E+00	0.00E+00	1.64E-04
**6h**	1.51E-03	2.65E-03	1.20E-03	1.45E-03
**12h**	1.54E-03	1.47E-03	6.74E-05	3.04E-04
**24h**	3.73E-04	4.94E-04	2.37E-03	1.17E-04
**36h**	6.65E-04	1.83E-03	3.69E-03	3.24E-04
**48h**	4.02E-04	1.23E-03	2.81E-03	2.92E-04

**72h**	1.05E-03	8.72E-04	8.91E-04	3.35E-04
	cgd6_1380			
**2h**	2.90E-06	1.87E-06	8.65E-07	1.40E-06
**6h**	3.53E-06	3.29E-05	4.77E-06	7.96E-06
**12h**	2.11E-06	1.48E-05	1.89E-06	2.39E-06
**24h**	6.68E-07	1.04E-05	9.55E-06	2.67E-06
**36h**	3.28E-06	1.72E-05	7.76E-06	2.97E-06
**48h**	5.34E-07	2.65E-06	3.39E-06	1.46E-06
**72h**	1.01E-06	2.93E-06	4.48E-07	3.92E-07
	cgd6_1390			
**2h**	1.36E-06	2.78E-06	1.91E-06	3.88E-06
**6h**	3.85E-06	3.48E-05	8.05E-06	1.96E-05
**12h**	1.08E-06	2.19E-05	3.87E-06	3.46E-06
**24h**	8.18E-07	6.41E-06	4.86E-06	4.87E-06
**36h**	1.25E-06	8.50E-06	2.24E-06	5.92E-06
**48h**	2.46E-07	1.94E-06	1.33E-06	1.39E-06
**72h**	3.98E-07	5.30E-06	5.34E-07	1.35E-06
	cgd6_1400			
**2h**	2.91E-06	2.46E-06	2.82E-07	8.42E-07
**6h**	8.60E-07	5.56E-06	1.61E-06	1.44E-06
**12h**	7.18E-08	7.32E-06	3.96E-07	4.15E-08
**24h**	9.90E-08	6.80E-07	7.76E-07	3.18E-07
**36h**	3.58E-07	1.10E-06	6.91E-07	3.79E-07
**48h**	1.37E-07	4.71E-07	2.29E-07	1.64E-07
**72h**	8.01E-08	2.93E-07	1.34E-07	1.13E-07
	cgd6_1410			
**2h**	1.76E-04	1.84E-04	5.55E-05	2.83E-04
**6h**	1.34E-04	1.61E-03	1.63E-04	3.58E-04
**12h**	1.11E-05	6.33E-04	2.31E-05	1.54E-05
**24h**	3.37E-05	2.14E-04	9.27E-05	9.20E-05
**36h**	2.06E-05	1.11E-04	2.99E-05	7.84E-05
**48h**	3.16E-05	5.67E-05	2.65E-05	2.76E-05
**72h**	3.92E-06	5.12E-06	3.98E-06	9.20E-06
	cgd6_1420			
**2h**	8.70E-06	4.69E-08	1.01E-06	3.11E-06
**6h**	1.39E-06	1.70E-05	3.20E-06	5.39E-06
**12h**	3.68E-07	1.27E-05	1.48E-06	5.41E-07
**24h**	1.03E-06	3.40E-06	3.00E-06	2.73E-06
**36h**	1.51E-06	3.13E-06	1.70E-06	2.75E-06
**48h**	2.05E-06	4.05E-06	3.45E-06	1.56E-06
**72h**	4.32E-07	7.85E-07	5.00E-07	6.85E-07
	cgd6_1430			
**2h**	2.87E-04	8.45E-05	3.78E-05	9.18E-05
**6h**	3.87E-05	1.36E-04	5.05E-05	7.32E-05
**12h**	2.74E-04	1.11E-03	1.06E-04	2.39E-04
**24h**	9.46E-05	1.28E-04	1.20E-04	1.30E-04
**36h**	3.80E-05	1.48E-04	8.65E-05	7.05E-05
**48h**	4.10E-05	1.16E-04	5.78E-05	2.35E-05
**72h**	8.30E-06	1.16E-05	6.40E-06	1.17E-05
	cgd6_1440			
**2h**	2.33E-03	8.44E-04	2.47E-04	5.73E-03
**6h**	6.00E-04	7.65E-03	2.90E-04	2.02E-03
**12h**	1.28E-03	1.11E-02	3.61E-04	5.17E-03

**24h**	7.27E-04	1.80E-03	2.09E-04	9.79E-04
**36h**	2.79E-04	1.41E-03	2.38E-04	6.27E-04
**48h**	4.14E-04	2.55E-03	3.43E-04	3.03E-04
**72h**	1.21E-04	1.04E-03	4.05E-05	3.56E-04
	cgd6_1450			
**2h**	7.88E-06	4.21E-06	5.30E-07	1.06E-06
**6h**	2.84E-07	3.29E-07	3.24E-07	6.61E-07
**12h**	1.05E-06	7.83E-06	4.15E-07	8.90E-07
**24h**	1.15E-06	1.56E-06	9.87E-07	2.11E-06
**36h**	3.24E-07	2.91E-06	5.21E-07	7.65E-07
**48h**	2.19E-06	7.26E-06	1.70E-06	6.64E-07
**72h**	1.94E-06	3.99E-06	1.42E-06	2.11E-06
	cgd6_1460			
**2h**	4.95E-06	5.37E-06	8.96E-07	3.15E-06
**6h**	2.60E-06	7.64E-06	1.34E-06	2.07E-06
**12h**	8.58E-07	2.49E-05	6.89E-07	4.40E-07
**24h**	2.26E-06	8.13E-06	1.79E-06	1.11E-06
**36h**	7.81E-07	3.04E-06	9.60E-07	1.13E-06
**48h**	1.76E-06	3.74E-06	9.23E-07	4.25E-07
**72h**	2.83E-07	3.87E-06	5.97E-08	4.61E-07
	cgd6_1470			
**2h**	0.00E+00	9.89E-07	1.75E-06	6.09E-06
**6h**	2.91E-06	7.03E-06	4.40E-06	3.73E-06
**12h**	2.24E-07	1.23E-06	7.68E-07	4.34E-07
**24h**	3.22E-06	1.59E-05	2.10E-06	1.50E-06
**36h**	7.49E-07	2.44E-06	8.88E-07	1.68E-06
**48h**	8.10E-07	1.97E-06	7.93E-07	4.82E-07
**72h**	1.30E-07	1.18E-07	1.50E-07	1.88E-07
	cgd6_1480			
**2h**	8.56E-03	0.00E+00	2.83E-04	2.68E-04
**6h**	6.84E-03	2.49E-02	4.99E-04	2.05E-03
**12h**	2.44E-03	1.29E-02	2.25E-04	0.00E+00
**24h**	1.96E-03	2.37E-02	1.53E-04	3.24E-04
**36h**	8.29E-04	1.08E-02	2.01E-04	4.66E-04
**48h**	8.57E-04	2.82E-03	7.20E-05	2.14E-04
**72h**	1.31E-03	1.08E-03	8.56E-05	9.45E-05
	cgd6_1490			
**2h**	2.00E-06	1.65E-06	2.69E-06	9.85E-07
**6h**	4.87E-06	2.37E-05	9.23E-06	2.43E-06
**12h**	1.06E-06	1.54E-05	2.39E-06	2.07E-06
**24h**	4.83E-06	1.76E-05	4.66E-06	2.69E-06
**36h**	3.98E-06	9.66E-06	3.57E-06	2.24E-06
**48h**	1.07E-06	5.86E-06	1.76E-06	9.24E-07
**72h**	3.65E-07	8.89E-07	5.78E-07	2.97E-07
	cgd6_1500			
**2h**	0.00E+00	0.00E+00	0.00E+00	0.00E+00
**6h**	0.00E+00	0.00E+00	0.00E+00	0.00E+00
**12h**	1.90E-05	2.47E-05	9.66E-06	3.04E-05
**24h**	4.34E-06	7.46E-06	3.34E-06	4.83E-06
**36h**	1.02E-05	2.45E-05	1.40E-05	5.52E-06
**48h**	6.81E-07	2.31E-06	1.28E-06	7.54E-07
**72h**	2.72E-07	2.55E-07	1.68E-07	3.58E-07
	cgd6_1510			

**2h**	1.18E-07	1.07E-07	1.75E-08	1.49E-08
**6h**	0.00E+00	0.00E+00	5.89E-08	6.33E-09
**12h**	1.45E-05	2.41E-05	1.78E-05	3.42E-05
**24h**	1.86E-06	2.79E-06	1.62E-06	2.94E-06
**36h**	2.40E-06	2.25E-05	1.13E-05	1.89E-06
**48h**	1.35E-07	8.43E-07	5.13E-07	4.21E-07
**72h**	1.28E-07	1.91E-08	1.50E-08	1.22E-07
	cgd6_1520			
**2h**	1.23E-04	1.85E-04	4.46E-03	1.32E-03
**6h**	2.53E-04	6.96E-04	2.67E-03	4.19E-03
**12h**	1.22E-04	5.63E-04	6.43E-04	2.25E-03
**24h**	1.15E-04	7.80E-04	4.38E-05	1.78E-03
**36h**	1.69E-04	8.39E-04	1.13E-03	1.44E-03
**48h**	8.51E-05	1.34E-04	3.97E-04	1.02E-04
**72h**	2.26E-05	1.28E-05	1.38E-05	2.62E-04
	cgd6_1530			
**2h**	2.71E-03	4.03E-03	2.85E-04	1.09E-03
**6h**	7.85E-03	2.24E-02	8.45E-04	1.85E-03
**12h**	6.87E-04	1.19E-02	2.34E-04	5.87E-04
**24h**	1.61E-03	4.50E-02	2.74E-04	6.00E-04
**36h**	2.89E-03	1.13E-02	2.08E-04	5.30E-04
**48h**	1.85E-03	9.38E-03	1.29E-04	9.55E-05
**72h**	3.30E-04	3.36E-04	4.73E-05	8.16E-05
	cgd6_1550			
**2h**	1.57E-06	2.18E-07	5.17E-06	7.33E-06
**6h**	5.70E-06	1.41E-05	3.82E-05	2.15E-05
**12h**	3.88E-07	7.37E-06	3.69E-06	9.58E-07
**24h**	1.77E-06	1.83E-05	1.76E-05	1.34E-05
**36h**	3.24E-06	4.28E-06	1.72E-05	1.19E-05
**48h**	1.32E-06	4.24E-06	2.18E-05	1.20E-05
**72h**	3.90E-07	3.56E-07	7.28E-06	1.73E-06
	cgd6_1560			
**2h**	1.21E-06	0.00E+00	1.12E-06	7.22E-07
**6h**	3.10E-06	8.29E-06	9.72E-07	6.02E-07
**12h**	2.15E-07	1.88E-06	2.17E-07	2.42E-07
**24h**	9.09E-07	6.71E-06	5.54E-07	4.02E-07
**36h**	9.84E-07	2.17E-06	5.97E-07	5.68E-07
**48h**	4.28E-07	1.07E-06	4.37E-07	4.45E-07
**72h**	8.74E-08	3.54E-07	1.36E-07	5.94E-08
	cgd6_1570			
**2h**	3.63E-08	0.00E+00	0.00E+00	0.00E+00
**6h**	3.41E-07	7.29E-07	2.53E-08	1.78E-08
**12h**	2.46E-07	2.21E-07	1.25E-07	1.07E-07
**24h**	3.18E-07	4.68E-06	4.33E-07	2.00E-07
**36h**	6.12E-07	1.68E-06	6.48E-07	5.06E-07
**48h**	2.28E-06	4.71E-06	1.08E-06	6.90E-07
**72h**	4.41E-07	6.10E-07	4.33E-07	2.68E-07
	cgd6_1580			
**2h**	7.30E-09	5.45E-08	1.67E-08	7.79E-09
**6h**	1.12E-07	7.81E-07	1.74E-07	7.24E-09
**12h**	2.28E-07	2.03E-06	6.34E-07	1.12E-06
**24h**	4.99E-07	3.69E-06	2.99E-07	6.38E-07
**36h**	1.08E-06	3.58E-06	2.75E-06	9.76E-07

**48h**	1.02E-06	3.97E-06	1.52E-06	9.14E-07
**72h**	4.14E-07	8.08E-07	5.06E-07	3.68E-07
	cgd6_1590			
**2h**	1.12E-04	1.71E-04	1.50E-04	1.09E-04
**6h**	6.08E-05	2.40E-04	7.48E-05	3.41E-05
**12h**	1.19E-05	2.94E-04	4.69E-05	6.07E-06
**24h**	2.60E-05	2.26E-04	7.04E-05	4.84E-05
**36h**	3.85E-05	1.14E-04	3.01E-03	5.07E-05
**48h**	9.36E-06	3.40E-05	3.63E-05	8.59E-06
**72h**	3.31E-06	2.70E-06	7.84E-06	2.16E-06
	cgd6_1750			
**2h**	1.45E-07	2.39E-07	3.13E-07	6.95E-07
**6h**	1.75E-06	7.82E-06	2.28E-06	2.21E-06
**12h**	1.52E-07	1.90E-06	2.84E-07	8.77E-07
**24h**	5.48E-07	8.04E-06	1.59E-06	8.35E-07
**36h**	1.13E-06	2.61E-06	6.43E-07	1.57E-06
**48h**	8.99E-07	3.00E-06	3.86E-07	3.36E-07
**72h**	2.18E-07	2.55E-07	1.26E-07	1.03E-07
	cgd6_1760			
**2h**	2.00E-06	0.00E+00	5.72E-05	1.21E-04
**6h**	1.60E-05	3.94E-05	3.54E-04	7.47E-04
**12h**	6.65E-07	7.90E-06	1.34E-04	8.30E-06
**24h**	5.16E-06	5.75E-05	1.62E-03	3.08E-04
**36h**	1.80E-05	4.74E-05	1.35E-04	1.60E-04
**48h**	1.02E-05	2.91E-03	2.51E-04	7.81E-05
**72h**	3.19E-06	9.78E-06	7.10E-05	1.78E-05
	cgd6_1770			
**2h**	7.51E-07	2.53E-06	1.41E-05	1.46E-05
**6h**	4.84E-06	1.23E-05	2.38E-05	3.50E-05
**12h**	9.89E-08	1.66E-06	2.33E-06	9.13E-07
**24h**	1.70E-06	9.33E-06	2.48E-05	8.85E-06
**36h**	3.76E-06	7.81E-06	6.70E-06	2.14E-06
**48h**	7.61E-07	3.91E-06	3.08E-06	2.57E-06
**72h**	1.86E-07	3.07E-07	1.13E-06	5.89E-07
	cgd6_1780			
**2h**	5.80E-07	7.67E-07	1.47E-06	3.33E-06
**6h**	1.29E-06	1.73E-06	4.52E-06	2.69E-06
**12h**	2.23E-07	1.73E-06	3.71E-07	1.40E-07
**24h**	3.59E-07	2.57E-06	2.64E-06	5.34E-07
**36h**	7.55E-07	1.43E-06	8.72E-07	1.18E-06
**48h**	2.26E-07	8.90E-07	6.33E-07	2.15E-07
**72h**	1.64E-07	1.17E-07	4.53E-08	2.55E-07
	cgd6_1790			
**2h**	1.49E-06	2.39E-06	3.73E-06	3.25E-06
**6h**	6.76E-07	3.35E-06	1.96E-05	4.42E-06
**12h**	1.98E-07	6.20E-06	1.77E-06	4.94E-07
**24h**	8.80E-07	1.33E-05	1.44E-05	2.37E-06
**36h**	1.59E-06	7.03E-06	3.72E-06	2.72E-06
**48h**	2.79E-07	4.66E-07	1.71E-06	4.53E-07
**72h**	4.38E-07	7.76E-07	4.92E-07	4.10E-07
	cgd6_1810			
**2h**	7.08E-08	2.14E-08	1.68E-07	7.00E-08
**6h**	4.56E-07	1.24E-06	4.38E-07	4.92E-07

**12h**	1.30E-07	1.42E-07	9.45E-08	6.36E-08
**24h**	1.48E-07	1.59E-06	6.05E-07	9.85E-08
**36h**	8.14E-07	1.91E-06	4.97E-07	2.94E-07
**48h**	2.96E-07	4.17E-07	1.06E-06	2.03E-07
**72h**	3.84E-07	2.18E-07	7.34E-08	8.80E-08
	cgd6_1820			
**2h**	1.65E-05	2.14E-05	1.38E-04	5.06E-05
**6h**	4.70E-06	1.37E-05	3.39E-05	1.91E-05
**12h**	8.29E-06	4.41E-05	5.12E-05	2.68E-05
**24h**	7.13E-06	2.24E-05	6.05E-05	4.05E-05
**36h**	5.15E-06	1.72E-05	2.17E-05	1.05E-05
**48h**	3.47E-06	9.09E-06	1.72E-05	4.91E-06
**72h**	3.74E-06	4.82E-06	1.35E-05	2.94E-06
	cgd6_1830			
**2h**	3.69E-04	1.71E-03	5.87E-05	1.38E-03
**6h**	2.77E-04	7.25E-04	1.20E-04	3.07E-04
**12h**	2.61E-04	8.88E-03	5.62E-05	1.18E-04
**24h**	5.71E-04	2.52E-03	1.71E-05	2.26E-04
**36h**	2.50E-04	2.53E-03	2.04E-05	2.11E-04
**48h**	3.86E-04	3.44E-03	9.01E-06	4.55E-05
**72h**	2.14E-04	5.30E-04	1.53E-05	2.66E-04
	cgd6_1840			
**2h**	4.64E-05	2.48E-05	1.29E-05	8.82E-04
**6h**	3.34E-04	9.53E-04	5.17E-04	3.21E-04
**12h**	4.62E-06	3.73E-04	1.04E-05	1.74E-04
**24h**	1.86E-04	1.52E-03	1.29E-04	2.60E-05
**36h**	1.62E-04	8.62E-04	2.51E-05	1.21E-04
**48h**	3.24E-04	2.12E-03	2.09E-05	1.40E-04
**72h**	1.02E-04	1.75E-04	9.54E-05	1.31E-04
	cgd6_1860			
**2h**	3.06E-07	1.42E-07	1.86E-06	1.08E-07
**6h**	2.92E-07	2.13E-06	7.99E-07	1.41E-06
**12h**	2.17E-09	0.00E+00	6.81E-07	0.00E+00
**24h**	1.58E-07	1.96E-07	1.30E-06	7.15E-07
**36h**	3.51E-07	8.92E-07	2.83E-06	6.51E-07
**48h**	5.80E-07	2.66E-06	6.03E-06	1.13E-06
**72h**	1.80E-07	1.49E-07	3.58E-06	1.29E-06
	cgd6_1870			
**2h**	5.05E-06	4.98E-05	1.27E-03	2.40E-04
**6h**	1.39E-04	5.17E-04	1.65E-03	6.41E-04
**12h**	2.10E-06	5.63E-05	1.31E-04	9.31E-06
**24h**	1.75E-05	3.79E-04	4.06E-04	1.05E-04
**36h**	3.09E-05	1.02E-04	1.29E-03	1.59E-04
**48h**	1.90E-05	7.88E-05	2.19E-04	4.81E-05
**72h**	4.05E-06	2.33E-06	7.22E-05	1.45E-05
	cgd6_1900			
**2h**	5.51E-07	2.10E-06	9.47E-07	1.19E-08
**6h**	4.69E-07	1.24E-06	3.46E-07	2.92E-07
**12h**	9.24E-07	3.40E-06	2.71E-06	1.90E-07
**24h**	3.03E-07	1.17E-06	7.98E-07	7.61E-07
**36h**	1.83E-06	6.70E-06	2.98E-06	1.93E-06
**48h**	4.61E-07	2.07E-06	1.87E-06	1.32E-06
**72h**	1.15E-06	2.10E-06	1.19E-06	1.95E-06

	cgd6_1910			
**2h**	3.66E-04	3.04E-04	1.27E-04	9.01E-05
**6h**	1.46E-04	1.06E-03	6.90E-03	7.97E-03
**12h**	1.03E-05	1.83E-04	1.44E-03	8.89E-04
**24h**	3.35E-05	2.70E-04	1.33E-03	4.80E-04
**36h**	6.27E-05	6.06E-04	5.13E-04	3.46E-04
**48h**	6.59E-05	3.09E-04	4.45E-04	1.42E-03
**72h**	2.86E-06	7.16E-05	1.68E-04	6.18E-05
	cgd6_1930			
**2h**	0.00E+00	0.00E+00	0.00E+00	0.00E+00
**6h**	0.00E+00	0.00E+00	0.00E+00	0.00E+00
**12h**	8.24E-09	1.82E-08	5.83E-08	4.75E-08
**24h**	2.41E-08	9.76E-09	6.50E-08	1.02E-08
**36h**	6.25E-08	1.65E-07	5.91E-08	4.98E-08
**48h**	4.63E-08	1.07E-07	3.10E-08	1.08E-07
**72h**	1.47E-08	1.52E-07	8.52E-08	7.96E-08
	cgd6_1940			
**2h**	0.00E+00	0.00E+00	0.00E+00	0.00E+00
**6h**	6.98E-07	7.09E-07	2.11E-06	4.07E-07
**12h**	1.44E-07	1.99E-07	2.37E-07	8.44E-08
**24h**	1.77E-07	3.89E-07	3.11E-06	7.06E-07
**36h**	5.93E-07	4.16E-07	7.33E-07	3.92E-07
**48h**	4.25E-07	8.89E-07	2.95E-07	1.00E-06
**72h**	5.99E-08	3.74E-07	5.12E-07	1.93E-07
	cgd6_1950			
**2h**	2.55E-08	6.81E-08	0.00E+00	9.97E-09
**6h**	3.46E-06	4.08E-06	1.19E-05	5.51E-06
**12h**	5.69E-06	5.84E-06	3.35E-05	7.67E-06
**24h**	1.82E-06	7.41E-06	8.73E-06	1.33E-05
**36h**	7.82E-06	1.80E-05	3.89E-05	2.62E-05
**48h**	5.49E-06	1.50E-05	5.81E-06	9.35E-06
**72h**	8.78E-07	4.70E-06	8.51E-06	4.07E-06
	cgd6_1960			
**2h**	0.00E+00	0.00E+00	0.00E+00	0.00E+00
**6h**	4.68E-09	2.79E-08	6.42E-08	4.03E-08
**12h**	1.62E-06	1.44E-06	2.57E-06	4.38E-07
**24h**	2.56E-07	5.19E-07	4.36E-07	1.97E-07
**36h**	7.53E-07	5.41E-07	1.60E-06	1.02E-06
**48h**	2.33E-07	3.94E-07	5.91E-07	2.86E-07
**72h**	1.64E-07	2.53E-07	5.82E-07	3.11E-07
	cgd6_1970			
**2h**	4.69E-07	3.23E-07	1.09E-07	3.95E-07
**6h**	3.22E-07	2.20E-07	4.05E-06	4.70E-07
**12h**	9.95E-08	4.75E-08	5.31E-07	1.99E-07
**24h**	9.49E-08	1.66E-07	9.57E-07	1.63E-07
**36h**	3.23E-07	2.67E-07	3.40E-07	3.71E-07
**48h**	1.62E-07	1.76E-07	3.48E-07	2.12E-07
**72h**	3.52E-08	8.46E-08	9.70E-08	1.73E-08
	cgd6_1990			
**2h**	9.16E-07	0.00E+00	0.00E+00	1.47E-06
**6h**	2.13E-07	6.82E-07	0.00E+00	5.29E-07
**12h**	3.04E-08	4.50E-07	0.00E+00	0.00E+00
**24h**	1.01E-07	3.15E-06	2.56E-07	5.06E-07

**36h**	1.70E-06	1.60E-05	1.01E-05	1.31E-06
**48h**	3.01E-06	9.40E-05	1.53E-05	2.35E-05
**72h**	4.13E-07	2.16E-05	2.13E-06	8.86E-06
	cgd6_2000			
**2h**	2.88E-07	0.00E+00	0.00E+00	8.79E-08
**6h**	1.93E-06	3.72E-05	1.61E-04	7.36E-05
**12h**	1.28E-07	9.16E-07	8.06E-06	2.73E-06
**24h**	7.22E-07	1.90E-05	1.14E-04	3.93E-05
**36h**	1.22E-05	4.28E-05	4.23E-05	3.92E-05
**48h**	2.91E-06	6.24E-05	2.63E-05	3.65E-05
**72h**	3.33E-07	1.22E-05	4.30E-06	4.55E-06
	cgd6_2020			
**2h**	5.35E-06	1.36E-04	9.60E-07	1.43E-05
**6h**	1.07E-05	1.45E-04	5.98E-04	8.10E-05
**12h**	3.04E-06	2.73E-05	4.71E-05	2.08E-05
**24h**	4.40E-06	1.08E-04	3.54E-04	1.52E-04
**36h**	1.57E-05	9.00E-05	6.62E-05	1.05E-04
**48h**	3.71E-05	8.20E-04	5.86E-04	7.24E-04
**72h**	1.15E-05	3.22E-04	6.49E-04	1.87E-04
	cgd6_2030			
**2h**	3.79E-06	5.76E-06	7.51E-07	2.92E-06
**6h**	1.02E-06	8.37E-06	2.71E-05	1.04E-05
**12h**	5.56E-07	1.56E-06	1.59E-05	2.10E-06
**24h**	2.05E-06	7.22E-06	1.75E-05	1.44E-05
**36h**	1.01E-06	3.30E-06	3.07E-06	4.69E-06
**48h**	5.13E-06	1.89E-05	1.64E-05	1.46E-05
**72h**	6.28E-07	8.65E-06	5.53E-06	4.28E-06
	cgd6_2040			
**2h**	4.28E-07	1.26E-06	3.94E-07	5.21E-07
**6h**	2.15E-06	5.68E-07	5.52E-06	1.69E-06
**12h**	4.52E-08	2.28E-07	1.26E-06	4.97E-07
**24h**	9.20E-07	1.52E-06	2.48E-06	2.93E-06
**36h**	8.00E-07	2.00E-06	4.87E-07	1.44E-06
**48h**	9.14E-07	8.62E-07	5.74E-07	1.37E-06
**72h**	8.59E-08	3.79E-07	1.34E-07	2.53E-07
	cgd6_2060			
**2h**	2.60E-05	9.80E-06	3.25E-06	1.04E-05
**6h**	1.55E-05	8.20E-06	4.58E-05	2.10E-05
**12h**	5.27E-06	4.99E-06	1.99E-05	3.84E-05
**24h**	2.37E-06	6.71E-06	1.10E-05	1.28E-05
**36h**	1.56E-05	5.98E-06	6.96E-06	7.03E-06
**48h**	4.78E-06	5.97E-06	1.69E-06	7.14E-06
**72h**	8.27E-06	2.27E-06	8.23E-07	3.53E-06
	cgd6_120			
**2h**	1.48E-07	1.96E-07	0.00E+00	1.89E-08
**6h**	2.38E-04	9.64E-04	2.60E-03	2.04E-03
**12h**	4.88E-05	1.09E-04	5.52E-05	1.27E-03
**24h**	1.22E-04	2.61E-04	1.24E-03	2.58E-04
**36h**	2.36E-04	3.97E-04	2.17E-04	8.92E-04
**48h**	1.20E-04	1.22E-04	4.14E-04	1.33E-04
**72h**	2.80E-06	7.65E-06	3.07E-06	1.63E-05
	cgd6_130			
**2h**	4.01E-06	1.74E-06	1.48E-07	1.27E-05

**6h**	8.95E-07	2.27E-06	3.77E-06	6.77E-06
**12h**	8.09E-07	8.26E-07	2.13E-06	1.02E-05
**24h**	4.84E-06	4.61E-06	5.39E-06	2.07E-06
**36h**	7.09E-07	8.34E-07	7.26E-07	1.40E-06
**48h**	1.07E-06	6.17E-07	5.55E-07	1.19E-06
**72h**	2.99E-07	4.79E-07	6.80E-07	1.16E-06
	cgd6_140			
**2h**	1.18E-05	8.36E-06	1.15E-06	1.04E-05
**6h**	2.64E-06	8.67E-06	1.31E-05	5.44E-06
**12h**	4.68E-06	2.79E-06	8.76E-06	6.65E-06
**24h**	1.21E-05	1.84E-05	2.16E-05	9.32E-06
**36h**	3.47E-06	5.08E-06	4.49E-06	3.57E-06
**48h**	2.91E-06	1.53E-06	2.81E-06	2.16E-06
**72h**	4.54E-07	3.65E-07	1.39E-06	1.06E-06
	cgd6_150			
**2h**	2.02E-05	3.12E-04	4.03E-06	2.07E-04
**6h**	2.18E-05	1.01E-04	5.79E-05	2.62E-04
**12h**	5.34E-06	1.69E-05	1.88E-05	1.10E-04
**24h**	2.05E-05	3.23E-04	3.80E-05	7.63E-05
**36h**	1.44E-05	5.64E-04	1.42E-05	5.67E-05
**48h**	6.33E-06	1.38E-05	7.71E-06	2.71E-05
**72h**	2.45E-06	8.44E-06	4.54E-06	1.60E-05
	cgd6_160			
**2h**	8.04E-09	4.13E-08	2.12E-09	4.11E-08
**6h**	1.97E-09	9.09E-09	2.55E-09	1.08E-08
**12h**	1.78E-07	2.31E-07	2.94E-07	1.17E-07
**24h**	7.13E-08	1.10E-07	4.23E-08	3.09E-08
**36h**	1.35E-07	3.46E-07	3.96E-07	2.90E-07
**48h**	1.09E-07	6.81E-08	6.23E-08	5.42E-08
**72h**	2.39E-08	9.33E-08	1.19E-07	7.79E-08
	cgd6_180			
**2h**	2.03E-07	9.02E-09	1.92E-08	1.16E-06
**6h**	3.78E-07	1.72E-07	1.32E-06	2.39E-06
**12h**	1.71E-07	2.20E-09	7.39E-08	2.55E-07
**24h**	7.37E-08	2.37E-07	9.28E-07	9.51E-07
**36h**	1.54E-07	5.05E-07	3.51E-07	3.65E-07
**48h**	3.05E-07	1.20E-07	1.93E-07	5.11E-07
**72h**	6.50E-08	1.62E-07	2.95E-07	5.03E-07
	cgd6_190			
**2h**	0.00E+00	0.00E+00	0.00E+00	0.00E+00
**6h**	0.00E+00	0.00E+00	0.00E+00	0.00E+00
**12h**	0.00E+00	0.00E+00	4.65E-09	2.23E-09
**24h**	3.22E-09	3.87E-10	2.30E-09	1.93E-09
**36h**	6.22E-08	2.17E-07	5.05E-08	1.30E-07
**48h**	1.72E-06	1.06E-06	8.69E-07	5.73E-07
**72h**	3.86E-07	1.22E-06	2.56E-06	2.92E-06
	cgd6_200			
**2h**	0.00E+00	0.00E+00	0.00E+00	5.38E-06
**6h**	2.32E-06	0.00E+00	0.00E+00	0.00E+00
**12h**	0.00E+00	0.00E+00	2.72E-05	1.63E-06
**24h**	1.69E-05	1.96E-05	1.26E-04	5.00E-07
**36h**	2.15E-04	1.15E-05	1.19E-04	0.00E+00
**48h**	7.24E-03	7.55E-04	8.62E-03	6.28E-04

**72h**	1.45E-02	3.99E-03	2.71E-02	3.20E-03
	cgd6_210			
**2h**	7.36E-08	4.19E-05	0.00E+00	2.62E-08
**6h**	1.37E-08	0.00E+00	2.68E-08	1.25E-07
**12h**	1.57E-08	1.87E-08	6.34E-08	7.08E-08
**24h**	3.09E-08	3.21E-08	3.88E-08	1.10E-08
**36h**	2.33E-08	5.10E-08	3.10E-08	5.99E-08
**48h**	2.92E-07	2.69E-07	2.97E-07	3.54E-07
**72h**	4.37E-08	2.43E-07	1.48E-07	1.42E-07
	cgd6_230			
**2h**	6.88E-07	6.32E-05	0.00E+00	2.79E-05
**6h**	7.81E-06	3.01E-05	1.00E-04	1.22E-04
**12h**	9.95E-07	1.79E-06	4.63E-06	1.98E-05
**24h**	3.56E-06	9.76E-05	1.03E-04	1.44E-04
**36h**	1.17E-06	7.52E-05	1.41E-05	1.12E-04
**48h**	5.79E-06	5.32E-05	1.70E-05	1.31E-04
**72h**	3.57E-07	1.60E-05	4.54E-06	1.13E-04
	cgd6_240			
**2h**	0.00E+00	0.00E+00	0.00E+00	4.29E-05
**6h**	6.76E-07	7.25E-06	6.36E-05	1.99E-04
**12h**	6.98E-07	8.27E-05	2.36E-05	1.02E-04
**24h**	1.09E-05	5.46E-05	9.82E-05	3.38E-04
**36h**	1.46E-05	7.02E-04	6.96E-05	8.50E-05
**48h**	1.41E-05	9.20E-05	4.18E-05	3.70E-05
**72h**	1.74E-06	1.17E-04	9.23E-06	3.75E-04
	cgd6_400			
**2h**	2.05E-05	5.81E-06	1.76E-06	0.00E+00
**6h**	4.38E-06	3.25E-06	1.55E-05	2.30E-05
**12h**	1.07E-06	3.29E-06	4.13E-06	1.84E-05
**24h**	2.01E-06	8.53E-06	1.98E-05	1.52E-05
**36h**	1.19E-06	8.36E-06	5.01E-06	7.02E-06
**48h**	3.63E-07	1.10E-06	3.22E-06	6.41E-06
**72h**	1.85E-07	2.67E-06	8.47E-07	1.14E-06
	cgd6_410			
**2h**	9.02E-06	4.27E-06	0.00E+00	2.37E-06
**6h**	7.07E-06	1.79E-05	6.25E-06	4.68E-06
**12h**	2.64E-06	6.16E-06	1.30E-05	1.52E-05
**24h**	5.63E-06	4.35E-06	1.90E-05	6.24E-06
**36h**	2.17E-06	3.29E-06	2.10E-05	3.47E-06
**48h**	1.98E-06	1.71E-06	1.33E-05	2.56E-06
**72h**	6.44E-07	5.04E-07	9.50E-07	4.21E-07
	cgd6_480			
**2h**	9.43E-10	0.00E+00	0.00E+00	4.62E-09
**6h**	4.05E-08	3.93E-07	3.00E-07	2.78E-07
**12h**	5.33E-08	4.09E-07	2.48E-07	3.90E-08
**24h**	1.46E-07	1.45E-07	2.01E-06	3.03E-07
**36h**	2.18E-07	2.95E-07	8.53E-07	4.66E-07
**48h**	2.49E-07	1.56E-07	1.19E-06	2.11E-07
**72h**	1.06E-06	5.70E-07	8.22E-07	1.78E-07
	cgd6_490			
**2h**	1.26E-06	2.67E-06	3.34E-07	6.79E-06
**6h**	4.28E-07	2.26E-06	4.33E-07	1.96E-06
**12h**	4.53E-07	1.01E-06	4.91E-06	2.10E-06

**24h**	2.53E-06	1.53E-06	1.97E-06	2.56E-06
**36h**	7.36E-07	1.01E-06	2.98E-06	5.09E-07
**48h**	6.68E-07	4.01E-07	7.99E-07	6.23E-07
**72h**	3.74E-07	4.45E-07	3.15E-07	2.15E-07
	cgd6_500			
**2h**	0.00E+00	0.00E+00	0.00E+00	0.00E+00
**6h**	0.00E+00	4.74E-09	0.00E+00	0.00E+00
**12h**	5.97E-08	7.50E-08	2.10E-07	1.43E-07
**24h**	4.91E-08	2.95E-08	6.34E-08	2.40E-08
**36h**	1.23E-07	1.92E-07	5.94E-07	2.13E-07
**48h**	1.33E-07	5.29E-08	1.51E-07	1.60E-07
**72h**	5.98E-08	2.55E-07	2.28E-07	8.73E-08
	cgd6_520			
**2h**	1.01E-06	1.58E-06	4.19E-07	1.87E-06
**6h**	9.13E-07	3.71E-07	4.68E-07	3.28E-07
**12h**	6.72E-06	3.35E-05	4.18E-05	1.08E-05
**24h**	4.11E-06	1.37E-06	2.70E-06	7.33E-07
**36h**	1.40E-06	2.78E-06	8.55E-06	4.14E-06
**48h**	4.52E-07	2.54E-07	1.13E-06	4.47E-07
**72h**	3.42E-07	9.10E-07	4.29E-07	3.60E-07
	cgd6_530			
**2h**	1.55E-06	6.42E-06	2.46E-07	9.31E-06
**6h**	1.48E-06	4.34E-06	8.91E-07	2.40E-06
**12h**	1.66E-07	2.18E-06	2.01E-06	5.18E-07
**24h**	7.43E-07	3.53E-07	1.64E-06	6.73E-07
**36h**	6.47E-07	2.32E-06	1.57E-06	1.50E-06
**48h**	6.80E-07	7.42E-07	1.07E-06	7.16E-07
**72h**	9.19E-07	1.28E-06	9.44E-07	2.11E-06
	cgd6_540			
**2h**	3.73E-04	1.20E-04	6.61E-05	9.19E-05
**6h**	3.84E-04	1.84E-04	6.39E-04	1.18E-04
**12h**	7.32E-06	9.11E-06	9.72E-05	3.17E-05
**24h**	1.83E-04	1.35E-05	5.66E-04	2.77E-05
**36h**	1.57E-04	3.51E-05	1.62E-04	2.56E-05
**48h**	4.40E-04	3.89E-05	2.37E-04	4.89E-05
**72h**	3.62E-04	3.17E-05	6.53E-05	2.06E-05
	cgd6_560			
**2h**	8.88E-08	7.01E-08	2.80E-08	9.10E-08
**6h**	1.07E-07	8.23E-07	2.40E-08	4.55E-07
**12h**	1.33E-08	5.97E-09	3.21E-08	1.72E-08
**24h**	3.73E-08	1.82E-08	9.93E-08	4.03E-08
**36h**	6.76E-08	1.27E-07	2.90E-07	9.26E-08
**48h**	1.06E-07	1.49E-07	1.06E-07	5.11E-08
**72h**	6.78E-08	4.06E-08	1.89E-07	3.07E-08
	cgd6_570			
**2h**	2.54E-04	3.24E-03	6.83E-05	5.28E-04
**6h**	1.05E-03	4.01E-04	5.15E-04	7.03E-04
**12h**	1.01E-04	7.00E-05	2.45E-04	1.83E-04
**24h**	2.17E-04	8.25E-05	4.55E-04	1.15E-04
**36h**	3.57E-04	7.28E-05	2.44E-04	1.48E-04
**48h**	2.68E-04	2.63E-05	1.41E-04	3.06E-05
**72h**	4.39E-05	1.59E-05	4.41E-05	8.82E-06
	cgd6_580			

**2h**	7.98E-05	1.74E-04	3.65E-05	2.67E-04
**6h**	8.20E-05	1.47E-04	2.35E-04	7.07E-04
**12h**	2.67E-06	6.45E-06	3.63E-04	2.39E-05
**24h**	4.33E-05	1.63E-05	5.44E-04	4.75E-05
**36h**	9.02E-05	1.91E-05	3.16E-04	9.51E-05
**48h**	1.51E-04	2.12E-05	2.13E-04	5.69E-05
**72h**	1.23E-04	1.18E-05	1.83E-04	1.73E-05
	cgd6_610			
**2h**	4.77E-04	4.34E-04	4.94E-05	1.30E-02
**6h**	3.58E-04	1.96E-04	1.27E-04	1.74E-03
**12h**	1.37E-04	3.13E-04	2.99E-04	4.21E-05
**24h**	1.99E-04	1.84E-05	7.88E-05	6.27E-05
**36h**	1.41E-04	3.14E-05	1.47E-04	3.02E-05
**48h**	1.24E-03	2.93E-05	1.14E-04	9.24E-04
**72h**	2.62E-04	1.85E-05	2.88E-04	7.73E-06
	cgd6_620			
**2h**	9.43E-06	8.55E-06	6.46E-06	1.99E-05
**6h**	9.28E-07	2.97E-06	1.06E-06	2.37E-06
**12h**	3.47E-06	2.45E-06	1.10E-05	5.20E-06
**24h**	2.42E-06	3.14E-06	1.31E-05	2.61E-06
**36h**	2.35E-06	1.61E-06	1.04E-05	5.26E-06
**48h**	2.62E-06	9.27E-07	4.78E-06	7.46E-06
**72h**	8.15E-07	3.62E-07	1.43E-06	3.61E-07
	cgd5_4490			
**2h**	0.00E+00	0.00E+00	0.00E+00	0.00E+00
**6h**	3.84E-06	3.89E-06	2.42E-06	2.31E-06
**12h**	9.23E-08	1.14E-07	6.88E-08	7.69E-08
**24h**	6.92E-07	4.09E-06	2.51E-06	2.22E-07
**36h**	9.68E-07	5.81E-07	9.19E-07	9.54E-07
**48h**	2.24E-06	3.02E-06	2.04E-06	1.41E-06
**72h**	2.40E-07	1.86E-07	4.25E-07	7.38E-07
	cgd6_10			
**2h**	2.02E-04	1.49E-04	3.27E-04	1.47E-04
**6h**	1.30E-04	1.23E-04	1.09E-04	8.87E-05
**12h**	2.04E-04	4.94E-05	1.01E-04	4.53E-05
**24h**	2.48E-04	1.78E-04	1.54E-04	1.52E-04
**36h**	4.34E-05	5.46E-05	1.06E-04	6.34E-05
**48h**	6.79E-05	1.58E-04	1.10E-04	4.38E-05
**72h**	2.71E-05	1.66E-05	1.17E-05	1.20E-05
	cgd6_20			
**2h**	1.84E-05	1.56E-05	4.01E-05	8.58E-06
**6h**	4.70E-06	4.92E-06	2.98E-06	4.95E-06
**12h**	1.33E-05	4.72E-06	6.90E-06	4.93E-06
**24h**	2.97E-05	1.35E-05	1.86E-05	9.64E-06
**36h**	6.67E-06	5.87E-06	6.68E-06	5.20E-06
**48h**	4.41E-06	5.58E-06	8.79E-06	2.57E-06
**72h**	2.27E-06	1.86E-06	1.96E-06	2.01E-06
	cgd6_30			
**2h**	3.47E-07	1.60E-06	2.53E-06	9.23E-07
**6h**	4.61E-06	5.04E-06	2.25E-06	2.73E-06
**12h**	1.36E-07	2.34E-07	7.63E-07	2.07E-07
**24h**	8.84E-07	3.86E-06	1.01E-06	5.18E-07
**36h**	1.12E-06	1.64E-06	2.73E-06	2.60E-06

**48h**	7.85E-07	7.94E-07	4.40E-07	1.05E-06
**72h**	4.95E-07	8.22E-07	7.91E-07	6.84E-07
	cgd6_40			
**2h**	1.15E-04	2.39E-04	3.00E-04	4.14E-04
**6h**	1.31E-04	7.15E-05	4.81E-05	1.19E-04
**12h**	9.71E-05	7.00E-05	1.82E-04	1.35E-04
**24h**	1.58E-04	1.19E-04	8.49E-05	1.97E-04
**36h**	8.01E-05	9.02E-05	1.87E-04	1.61E-04
**48h**	1.83E-05	2.79E-05	2.80E-05	3.61E-05
**72h**	4.14E-05	2.18E-05	2.61E-05	2.12E-05
	cgd6_50			
**2h**	1.68E-07	2.29E-08	3.02E-07	1.52E-07
**6h**	1.20E-06	1.27E-06	4.55E-07	3.37E-07
**12h**	3.69E-07	6.48E-08	1.50E-07	1.32E-07
**24h**	1.20E-07	5.74E-07	3.57E-07	1.27E-07
**36h**	7.89E-07	1.04E-06	2.06E-06	8.99E-07
**48h**	2.21E-07	5.53E-07	5.56E-07	4.83E-07
**72h**	4.40E-07	2.22E-07	5.07E-07	4.46E-07
	cgd6_60			
**2h**	8.67E-07	4.28E-07	1.50E-05	9.28E-07
**6h**	9.18E-07	1.07E-06	4.08E-07	5.16E-07
**12h**	5.44E-05	1.41E-05	3.79E-05	1.13E-05
**24h**	8.35E-06	1.63E-05	8.36E-06	3.74E-06
**36h**	3.94E-05	1.51E-04	1.68E-04	2.50E-05
**48h**	9.56E-06	3.86E-05	1.90E-05	2.38E-05
**72h**	6.00E-06	3.52E-06	5.28E-06	4.20E-06
	cgd6_70			
**2h**	1.74E-06	3.15E-07	5.35E-07	4.35E-07
**6h**	9.00E-06	1.75E-05	5.90E-06	1.89E-06
**12h**	4.25E-07	2.86E-07	1.25E-06	4.22E-07
**24h**	5.60E-07	1.05E-05	7.47E-06	7.11E-07
**36h**	7.10E-06	8.67E-06	6.89E-06	7.55E-06
**48h**	3.40E-06	8.99E-06	9.17E-06	2.51E-06
**72h**	1.70E-06	5.83E-07	1.30E-06	4.75E-07
	cgd6_80			
**2h**	6.10E-07	5.58E-07	4.55E-07	7.87E-07
**6h**	4.19E-07	5.50E-07	9.60E-07	6.23E-07
**12h**	1.18E-07	3.97E-07	9.10E-07	2.02E-07
**24h**	1.79E-07	1.12E-06	3.49E-07	4.57E-07
**36h**	6.25E-07	8.25E-07	1.38E-06	3.29E-07
**48h**	2.14E-07	4.50E-07	4.53E-07	3.96E-07
**72h**	1.86E-07	4.72E-08	9.90E-08	8.64E-08
	cgd6_90			
**2h**	3.23E-06	1.72E-06	5.34E-06	3.34E-06
**6h**	4.23E-07	3.02E-07	1.79E-07	1.86E-07
**12h**	1.55E-06	1.48E-06	2.05E-06	1.04E-06
**24h**	1.36E-06	1.89E-06	1.00E-06	1.40E-06
**36h**	1.35E-06	1.52E-06	1.66E-06	4.24E-07
**48h**	5.66E-07	1.19E-06	1.30E-06	1.39E-06
**72h**	4.36E-07	1.47E-07	2.85E-07	3.00E-07
	cgd6_100			
**2h**	3.63E-06	3.21E-06	6.83E-06	6.50E-06
**6h**	4.00E-07	9.35E-08	1.64E-07	2.09E-07

**12h**	2.19E-06	1.15E-06	2.00E-06	8.23E-07
**24h**	2.70E-06	2.79E-06	1.50E-06	4.20E-06
**36h**	8.43E-07	1.26E-06	1.54E-06	1.28E-06
**48h**	8.38E-07	3.28E-06	2.04E-06	1.18E-06
**72h**	6.43E-07	1.90E-07	1.67E-07	2.13E-07
	cgd6_110			
**2h**	3.50E-09	0.00E+00	7.08E-09	0.00E+00
**6h**	1.93E-08	5.29E-08	1.65E-08	1.43E-08
**12h**	7.43E-09	6.83E-10	3.88E-08	5.01E-08
**24h**	1.51E-08	1.91E-07	1.04E-07	2.66E-08
**36h**	1.44E-07	1.82E-07	4.73E-07	3.27E-07
**48h**	7.82E-08	2.36E-07	4.75E-07	1.05E-07
**72h**	6.62E-08	8.26E-08	1.44E-07	7.57E-08
	cgd6_1600			
**2h**	8.99E-09	3.53E-07	3.73E-08	1.44E-07
**6h**	1.08E-07	2.74E-07	3.18E-07	1.63E-05
**12h**	1.07E-08	3.20E-08	2.18E-08	1.90E-07
**24h**	3.75E-08	1.57E-07	6.51E-08	3.58E-07
**36h**	1.14E-07	1.36E-07	1.92E-07	1.12E-07
**48h**	1.55E-07	1.20E-07	8.96E-08	1.21E-07
**72h**	5.82E-08	5.69E-08	4.88E-08	2.75E-07
	cgd6_1610			
**2h**	1.22E-07	9.99E-08	4.74E-08	1.68E-08
**6h**	2.33E-08	1.00E-07	4.12E-08	6.08E-08
**12h**	6.50E-08	1.37E-07	1.78E-07	2.52E-07
**24h**	1.58E-07	3.98E-07	3.72E-07	3.04E-07
**36h**	2.35E-07	6.98E-07	3.78E-07	1.96E-07
**48h**	3.25E-07	3.00E-07	2.91E-07	2.43E-07
**72h**	1.51E-07	1.30E-07	1.34E-07	3.29E-07
	cgd6_1620			
**2h**	4.12E-08	9.93E-08	4.50E-08	8.70E-07
**6h**	8.57E-07	7.77E-07	2.74E-06	2.24E-06
**12h**	1.83E-08	3.88E-08	6.06E-08	2.83E-07
**24h**	2.57E-07	5.37E-07	1.99E-07	5.69E-07
**36h**	3.87E-07	6.80E-07	2.61E-07	3.49E-07
**48h**	1.72E-07	1.35E-07	1.99E-07	3.08E-07
**72h**	9.91E-08	1.08E-07	7.09E-08	4.15E-07
	cgd6_1630			
**2h**	0.00E+00	0.00E+00	2.42E-08	0.00E+00
**6h**	1.87E-04	2.26E-04	1.73E-04	4.21E-04
**12h**	1.76E-06	6.18E-06	4.94E-06	5.85E-06
**24h**	2.11E-05	9.98E-05	3.00E-05	6.99E-05
**36h**	2.66E-05	2.54E-05	2.30E-05	1.42E-05
**48h**	8.94E-06	9.82E-06	8.04E-06	1.09E-05
**72h**	1.82E-06	2.08E-06	1.16E-06	2.87E-06
	cgd6_1640			
**2h**	3.08E-06	1.75E-06	1.19E-06	0.00E+00
**6h**	6.01E-04	1.16E-03	6.75E-04	2.34E-05
**12h**	1.20E-05	3.16E-05	2.25E-05	3.24E-06
**24h**	1.16E-04	6.81E-04	1.25E-04	7.75E-06
**36h**	1.61E-04	1.75E-04	7.32E-05	2.30E-06
**48h**	1.96E-04	1.88E-04	6.69E-05	4.24E-06
**72h**	7.06E-05	9.31E-05	4.99E-05	5.52E-06

	cgd6_1670			
**2h**	2.34E-04	2.31E-05	3.98E-05	0.00E+00
**6h**	2.03E-04	6.61E-05	8.37E-05	0.00E+00
**12h**	3.79E-05	1.53E-05	4.88E-05	0.00E+00
**24h**	4.61E-05	1.69E-05	3.77E-05	0.00E+00
**36h**	2.20E-05	2.75E-05	7.38E-05	0.00E+00
**48h**	1.34E-05	2.05E-05	1.32E-05	0.00E+00
**72h**	1.78E-05	3.53E-05	1.65E-05	0.00E+00
	cgd6_1680			
**2h**	5.73E-07	2.54E-07	5.21E-07	0.00E+00
**6h**	1.76E-06	1.43E-06	7.76E-07	8.84E-06
**12h**	2.86E-07	2.93E-07	1.55E-07	8.29E-06
**24h**	4.97E-07	1.04E-06	5.06E-07	7.13E-06
**36h**	7.72E-07	3.63E-07	7.44E-07	2.54E-06
**48h**	6.02E-07	3.31E-07	3.18E-07	4.28E-06
**72h**	1.68E-07	1.02E-07	1.77E-07	2.17E-06
	cgd6_1700			
**2h**	2.64E-04	2.85E-04	6.60E-05	1.82E-05
**6h**	6.33E-04	8.48E-04	4.15E-04	1.96E-05
**12h**	5.22E-04	4.39E-04	3.47E-04	1.08E-05
**24h**	2.26E-04	4.19E-04	1.55E-04	1.61E-05
**36h**	3.35E-04	5.19E-04	2.40E-04	1.69E-06
**48h**	1.46E-04	2.36E-04	2.24E-04	9.31E-07
**72h**	1.33E-04	1.26E-04	1.17E-04	4.70E-06
	cgd6_1710			
**2h**	0.00E+00	0.00E+00	0.00E+00	0.00E+00
**6h**	0.00E+00	0.00E+00	1.17E-07	0.00E+00
**12h**	1.16E-05	9.27E-06	5.62E-05	2.89E-06
**24h**	1.12E-05	4.29E-06	6.48E-06	1.39E-06
**36h**	8.75E-05	2.32E-05	4.43E-05	1.01E-06
**48h**	5.15E-05	1.28E-04	3.81E-05	1.70E-06
**72h**	9.01E-05	2.30E-05	4.86E-05	4.43E-06
	cgd6_1720			
**2h**	1.77E-08	0.00E+00	0.00E+00	1.73E-06
**6h**	6.07E-08	6.91E-08	5.93E-08	1.80E-05
**12h**	2.76E-06	3.00E-06	1.14E-05	1.27E-04
**24h**	1.93E-06	1.23E-06	7.97E-07	2.35E-05
**36h**	3.17E-06	4.83E-06	3.92E-06	1.93E-05
**48h**	6.46E-06	2.07E-06	4.47E-06	1.80E-05
**72h**	7.73E-07	7.28E-07	5.31E-07	2.12E-05
	cgd6_1730			
**2h**	1.06E-06	8.25E-07	2.71E-07	4.02E-06
**6h**	8.22E-07	4.91E-07	3.54E-07	2.17E-05
**12h**	7.18E-07	9.81E-07	1.70E-06	1.03E-05
**24h**	4.49E-07	4.22E-07	6.89E-07	5.20E-06
**36h**	2.83E-06	2.80E-06	1.64E-06	2.64E-05
**48h**	2.04E-06	1.21E-06	1.61E-06	7.74E-06
**72h**	3.26E-07	2.02E-07	3.00E-07	2.80E-06
	cgd6_1740			
**2h**	1.70E-05	9.22E-06	3.15E-06	4.32E-05
**6h**	2.14E-06	1.02E-05	3.74E-06	6.17E-05
**12h**	8.74E-07	3.37E-06	1.17E-06	2.29E-05
**24h**	2.38E-06	4.77E-06	2.24E-06	3.02E-05

**36h**	3.14E-06	5.22E-06	4.85E-06	2.07E-05
**48h**	1.72E-06	2.01E-06	1.36E-06	5.31E-06
**72h**	1.79E-07	2.42E-07	1.07E-07	2.44E-06
	cgd6_2070			
**2h**	1.42E-08	9.09E-09	2.17E-09	3.88E-10
**6h**	1.17E-07	1.92E-07	4.83E-08	3.02E-08
**12h**	6.81E-10	8.82E-09	1.12E-09	3.19E-09
**24h**	7.80E-09	1.01E-07	2.52E-08	8.55E-08
**36h**	7.49E-08	2.62E-08	2.71E-08	8.28E-08
**48h**	3.84E-08	2.65E-08	2.32E-08	5.26E-08
**72h**	1.99E-08	1.66E-10	2.31E-09	4.14E-09
	cgd6_2080			
**2h**	3.02E-05	1.09E-06	2.22E-06	1.43E-06
**6h**	9.53E-06	4.92E-06	2.69E-06	6.34E-06
**12h**	4.92E-07	2.68E-06	3.28E-07	8.91E-07
**24h**	2.42E-06	3.43E-06	3.18E-06	6.67E-06
**36h**	2.45E-06	1.57E-06	1.28E-06	2.52E-06
**48h**	2.25E-06	3.84E-07	7.12E-07	2.74E-06
**72h**	6.65E-07	2.07E-07	1.38E-07	1.11E-06
	cgd6_2090			
**2h**	1.59E-03	3.64E-06	7.23E-06	2.55E-05
**6h**	4.41E-05	6.05E-06	5.33E-06	3.79E-06
**12h**	2.82E-05	3.50E-06	3.39E-07	7.63E-07
**24h**	1.49E-05	1.66E-05	4.98E-06	8.05E-06
**36h**	2.31E-05	5.59E-06	2.21E-06	1.53E-05
**48h**	2.27E-02	1.28E-03	1.35E-03	3.51E-03
**72h**	5.31E-03	6.74E-04	1.05E-04	1.14E-03
	cgd6_2100			
**2h**	1.33E-06	1.85E-07	7.04E-08	1.58E-07
**6h**	5.19E-07	3.15E-07	2.63E-07	1.15E-07
**12h**	1.73E-08	1.37E-07	4.36E-09	3.07E-08
**24h**	3.51E-07	1.86E-07	2.69E-07	3.09E-07
**36h**	5.59E-07	4.19E-07	1.16E-07	7.99E-07
**48h**	2.70E-06	2.58E-07	2.02E-07	6.74E-07
**72h**	1.61E-06	8.60E-08	1.34E-07	3.28E-07
	cgd6_2110			
**2h**	4.35E-07	9.50E-08	1.51E-07	2.50E-07
**6h**	2.07E-07	1.69E-07	5.74E-08	1.27E-07
**12h**	2.04E-08	1.84E-07	4.81E-08	1.31E-08
**24h**	1.21E-07	1.37E-07	1.12E-07	1.79E-07
**36h**	3.37E-07	4.90E-07	4.24E-07	3.33E-07
**48h**	2.55E-06	1.38E-07	1.22E-07	1.60E-07
**72h**	1.18E-07	1.44E-07	9.66E-08	1.39E-07
	cgd6_2120			
**2h**	4.38E-07	3.85E-07	1.06E-07	1.06E-07
**6h**	4.32E-07	4.49E-07	1.14E-07	3.29E-07
**12h**	2.35E-08	4.10E-07	6.44E-10	7.53E-08
**24h**	1.09E-07	2.24E-07	5.92E-08	3.93E-06
**36h**	2.78E-07	2.49E-07	2.40E-07	2.92E-07
**48h**	5.10E-07	1.61E-07	4.98E-08	1.08E-07
**72h**	4.64E-08	1.78E-07	5.06E-08	1.20E-07
	cgd6_2130			
**2h**	8.11E-06	1.62E-06	6.85E-07	1.30E-06

**6h**	6.02E-06	3.57E-06	6.09E-07	2.21E-06
**12h**	3.14E-07	2.17E-06	1.57E-08	6.11E-07
**24h**	4.73E-07	9.78E-07	2.91E-07	7.60E-07
**36h**	6.83E-07	9.20E-07	3.83E-07	4.37E-07
**48h**	4.73E-07	6.17E-07	2.43E-07	4.83E-07
**72h**	5.58E-07	1.50E-07	1.29E-07	2.31E-07
	cgd6_2140			
**2h**	5.70E-06	1.68E-08	4.63E-07	7.75E-07
**6h**	3.23E-06	2.72E-08	8.70E-08	1.53E-06
**12h**	0.00E+00	6.55E-08	8.16E-08	1.10E-07
**24h**	1.62E-06	8.79E-07	9.03E-08	6.27E-07
**36h**	4.64E-06	5.21E-07	2.57E-07	1.28E-06
**48h**	2.48E-05	3.40E-06	1.16E-06	5.69E-06
**72h**	5.31E-06	1.31E-06	6.06E-07	2.51E-06
	cgd6_2150			
**2h**	1.01E-03	4.64E-04	1.50E-04	1.00E-03
**6h**	9.21E-03	1.09E-03	1.61E-04	1.48E-03
**12h**	7.82E-04	7.99E-04	5.80E-05	3.29E-04
**24h**	7.06E-04	8.85E-04	8.71E-05	1.17E-03
**36h**	6.44E-04	3.87E-04	7.47E-05	4.89E-04
**48h**	3.04E-04	3.86E-04	3.56E-05	5.09E-04
**72h**	1.50E-03	1.05E-04	2.31E-05	2.79E-04
	cgd6_2170			
**2h**	4.80E-05	2.19E-05	1.27E-05	2.08E-05
**6h**	1.57E-04	7.67E-05	2.16E-05	1.02E-01
**12h**	1.93E-05	4.81E-05	3.47E-06	1.55E-05
**24h**	2.12E-05	7.42E-05	1.60E-05	2.46E-05
**36h**	8.27E-06	8.85E-06	5.56E-06	1.84E-05
**48h**	1.77E-05	9.53E-06	4.58E-06	7.01E-06
**72h**	1.64E-05	5.30E-07	6.72E-07	3.94E-06
	cgd6_2180			
**2h**	1.03E-03	1.79E-04	1.43E-04	3.87E-04
**6h**	1.01E-03	8.96E-04	1.03E-04	3.57E-04
**12h**	8.24E-05	3.48E-04	3.45E-05	2.52E-04
**24h**	1.98E-04	4.09E-04	9.47E-05	4.30E-04
**36h**	1.09E-04	8.65E-05	4.11E-05	2.51E-04
**48h**	7.73E-04	9.97E-05	2.62E-05	1.88E-04
**72h**	2.82E-05	1.36E-05	3.42E-06	8.26E-05
	cgd6_2190			
**2h**	1.11E-07	0.00E+00	0.00E+00	3.88E-08
**6h**	1.33E-07	9.46E-07	1.92E-07	1.03E-07
**12h**	3.88E-08	2.93E-07	4.66E-08	5.78E-08
**24h**	6.92E-08	1.35E-06	6.62E-08	4.00E-07
**36h**	3.05E-07	3.38E-07	3.66E-07	3.91E-07
**48h**	4.83E-07	1.36E-06	5.79E-08	2.66E-07
**72h**	4.37E-07	3.07E-06	1.58E-07	3.06E-07
	cgd6_2200			
**2h**	1.45E-04	3.37E-07	2.73E-07	2.14E-07
**6h**	1.42E-07	4.71E-07	1.43E-06	8.71E-07
**12h**	7.50E-06	5.68E-06	2.52E-05	4.40E-06
**24h**	5.13E-06	1.29E-06	1.55E-05	4.12E-06
**36h**	7.09E-06	1.82E-06	3.38E-05	2.32E-05
**48h**	5.98E-06	3.17E-07	9.38E-06	4.86E-06

**72h**	5.83E-07	2.30E-07	8.06E-07	5.90E-07
	cgd6_2210			
**2h**	6.39E-07	5.68E-07	1.62E-06	1.40E-06
**6h**	4.96E-06	3.22E-06	1.06E-05	2.99E-06
**12h**	6.85E-05	2.40E-05	1.37E-04	1.16E-05
**24h**	1.27E-05	5.42E-06	6.18E-05	9.51E-06
**36h**	1.17E-05	6.26E-06	9.36E-05	2.99E-05
**48h**	1.31E-05	1.79E-06	1.83E-05	6.14E-06
**72h**	3.56E-06	5.96E-07	1.46E-06	1.26E-06
	cgd6_2220			
**2h**	1.93E-08	3.68E-08	6.72E-08	1.82E-08
**6h**	8.28E-08	6.69E-08	1.90E-07	8.12E-08
**12h**	3.16E-05	4.82E-05	3.87E-05	1.13E-05
**24h**	2.25E-06	5.84E-06	1.92E-05	6.18E-06
**36h**	1.06E-05	4.24E-06	3.23E-05	1.91E-05
**48h**	9.46E-07	6.78E-07	2.14E-06	2.39E-06
**72h**	2.68E-07	1.69E-07	4.16E-07	1.91E-07
	cgd6_2240			
**2h**	1.16E-03	1.36E-05	1.84E-05	1.62E-04
**6h**	2.38E-03	5.38E-05	3.28E-03	9.71E-04
**12h**	8.31E-04	8.96E-06	1.40E-03	2.47E-05
**24h**	4.80E-04	4.65E-05	6.26E-03	7.80E-04
**36h**	3.10E-03	1.41E-04	1.36E-03	1.66E-03
**48h**	4.69E-04	2.17E-05	1.01E-03	5.64E-04
**72h**	1.42E-04	4.72E-05	2.85E-04	2.43E-04
	cgd6_2250			
**2h**	4.25E-06	0.00E+00	0.00E+00	0.00E+00
**6h**	4.44E-07	2.23E-07	2.27E-06	9.09E-07
**12h**	0.00E+00	4.38E-07	1.02E-05	1.66E-05
**24h**	2.30E-05	8.88E-07	1.16E-05	7.06E-06
**36h**	4.86E-05	4.76E-06	9.78E-05	3.37E-05
**48h**	1.87E-03	3.10E-06	1.28E-04	8.53E-05
**72h**	9.22E-05	3.67E-06	1.06E-04	8.13E-05
	cgd6_2260			
**2h**	1.09E-04	9.57E-06	5.57E-05	7.18E-05
**6h**	1.29E-05	2.66E-06	3.68E-05	9.14E-06
**12h**	1.57E-04	3.18E-05	4.74E-04	3.22E-05
**24h**	7.72E-05	1.60E-05	2.82E-04	3.02E-05
**36h**	3.96E-05	1.21E-05	6.72E-05	4.25E-05
**48h**	1.39E-04	2.13E-06	4.98E-05	1.58E-05
**72h**	1.76E-05	5.46E-06	1.24E-05	5.16E-06
	cgd6_2270			
**2h**	3.10E-06	6.30E-07	9.17E-07	4.83E-06
**6h**	7.63E-07	3.86E-07	6.91E-07	3.06E-07
**12h**	2.26E-06	2.30E-07	4.85E-06	8.31E-07
**24h**	6.65E-07	1.32E-06	5.13E-06	8.01E-07
**36h**	5.06E-07	7.46E-07	1.14E-06	1.29E-06
**48h**	4.92E-07	4.03E-07	1.66E-06	5.14E-07
**72h**	8.52E-08	2.20E-07	1.61E-07	1.56E-07
	cgd6_2280			
**2h**	2.52E-04	4.35E-06	4.13E-06	5.30E-05
**6h**	1.69E-04	3.97E-06	1.18E-04	4.63E-05
**12h**	5.80E-04	1.06E-05	3.51E-04	4.53E-05

**24h**	1.56E-04	7.36E-06	1.21E-03	8.94E-05
**36h**	2.30E-04	1.90E-05	3.06E-04	1.92E-04
**48h**	5.21E-04	8.04E-06	3.06E-04	1.12E-04
**72h**	3.95E-05	4.31E-06	1.08E-03	1.35E-05
	cgd6_2290			
**2h**	2.05E-08	7.40E-08	1.00E-07	2.22E-07
**6h**	3.72E-06	2.46E-07	1.43E-05	8.94E-07
**12h**	6.42E-07	9.38E-07	1.02E-06	2.87E-07
**24h**	1.84E-06	2.38E-07	1.48E-05	1.74E-06
**36h**	2.22E-06	1.11E-06	3.18E-06	2.41E-06
**48h**	2.12E-06	4.85E-07	3.35E-06	2.43E-06
**72h**	1.04E-06	4.70E-07	3.02E-06	7.56E-07
	cgd6_2300			
**2h**	4.72E-06	1.33E-07	0.00E+00	2.38E-07
**6h**	5.51E-05	2.91E-05	2.34E-03	1.31E-04
**12h**	2.20E-03	9.38E-06	1.91E-03	1.27E-04
**24h**	2.70E-04	4.11E-05	4.23E-03	1.04E-03
**36h**	7.00E-04	7.14E-05	5.76E-03	9.08E-04
**48h**	1.65E-03	8.20E-05	3.08E-03	1.18E-03
**72h**	2.08E-03	7.65E-06	3.39E-04	6.51E-05
	cgd6_2310			
**2h**	1.65E-07	2.59E-08	4.19E-09	4.95E-08
**6h**	5.27E-08	2.04E-08	3.44E-08	1.90E-08
**12h**	2.91E-07	9.81E-08	1.13E-06	2.00E-07
**24h**	1.83E-07	2.03E-07	2.55E-06	3.55E-07
**36h**	1.07E-06	7.80E-07	3.30E-06	2.05E-06
**48h**	3.90E-07	3.29E-07	1.53E-06	5.44E-07
**72h**	5.37E-08	5.84E-08	8.20E-08	1.06E-07
	cgd6_2320			
**2h**	4.62E-06	5.09E-06	7.32E-07	1.11E-05
**6h**	3.02E-06	3.93E-07	2.03E-05	1.04E-05
**12h**	3.14E-06	1.32E-07	1.65E-05	4.88E-06
**24h**	3.18E-06	1.65E-06	4.23E-05	7.80E-06
**36h**	5.18E-06	4.18E-06	1.84E-05	1.58E-05
**48h**	6.05E-06	1.63E-06	8.44E-06	4.49E-06
**72h**	2.38E-06	7.51E-07	2.20E-04	2.35E-06
	cgd6_3770			
**2h**	8.48E-09	2.52E-08	0.00E+00	6.24E-09
**6h**	3.48E-07	8.17E-08	2.67E-07	4.09E-07
**12h**	1.99E-07	4.52E-07	5.49E-07	8.57E-07
**24h**	4.23E-07	4.84E-07	5.64E-06	7.39E-07
**36h**	1.10E-06	1.31E-06	4.34E-06	1.78E-06
**48h**	7.47E-07	4.89E-07	1.63E-06	7.38E-07
**72h**	2.46E-07	4.62E-07	1.26E-06	3.27E-07
	cgd6_3780			
**2h**	0.00E+00	0.00E+00	0.00E+00	0.00E+00
**6h**	1.01E-05	1.02E-04	5.43E-05	1.60E-04
**12h**	3.22E-05	4.12E-03	1.49E-04	9.97E-04
**24h**	3.36E-05	3.16E-04	1.04E-03	7.93E-04
**36h**	7.77E-05	1.18E-04	1.01E-03	1.07E-03
**48h**	4.78E-05	2.64E-04	5.11E-04	5.52E-04
**72h**	3.17E-05	2.72E-04	3.46E-04	6.29E-04
	cgd6_3790			

**2h**	1.32E-07	4.75E-07	9.70E-07	6.38E-07
**6h**	2.34E-07	1.12E-07	2.91E-07	9.02E-08
**12h**	3.58E-05	5.02E-05	3.65E-05	3.46E-05
**24h**	1.00E-05	1.42E-05	1.01E-05	8.45E-06
**36h**	1.68E-05	6.94E-06	3.85E-05	1.28E-05
**48h**	6.41E-06	4.57E-06	1.43E-05	1.11E-05
**72h**	2.18E-06	2.70E-06	4.80E-06	1.39E-06
	cgd6_3800			
**2h**	1.29E-06	1.97E-06	1.05E-05	3.71E-07
**6h**	2.24E-07	4.27E-06	5.96E-08	6.01E-06
**12h**	6.70E-07	1.28E-06	1.13E-06	3.03E-06
**24h**	2.24E-06	7.09E-06	2.74E-06	2.93E-06
**36h**	1.94E-06	7.50E-06	5.28E-06	5.32E-06
**48h**	2.80E-06	5.51E-06	5.08E-06	3.82E-06
**72h**	7.95E-06	2.17E-05	2.09E-05	1.44E-05
	cgd6_3810			
**2h**	3.14E-06	1.15E-04	1.27E-05	1.73E-05
**6h**	9.27E-06	4.02E-05	1.72E-05	5.60E-05
**12h**	4.99E-07	2.39E-06	1.35E-05	4.06E-05
**24h**	4.47E-06	5.33E-05	4.10E-05	3.50E-05
**36h**	2.96E-05	3.24E-05	6.12E-05	1.67E-05
**48h**	6.68E-06	8.07E-05	2.43E-05	3.26E-05
**72h**	3.24E-05	9.28E-05	5.14E-05	3.94E-05
	cgd6_3820			
**2h**	1.11E-07	1.72E-07	2.41E-07	3.65E-08
**6h**	4.64E-07	1.78E-07	2.64E-07	4.18E-07
**12h**	4.17E-08	5.16E-08	4.49E-07	6.08E-08
**24h**	1.95E-07	1.06E-07	3.91E-07	2.94E-07
**36h**	5.56E-07	6.19E-07	9.72E-07	2.40E-07
**48h**	7.12E-07	5.26E-07	1.95E-06	8.18E-07
**72h**	2.08E-06	1.98E-06	2.81E-06	1.10E-06
	cgd6_3830			
**2h**	1.18E-06	1.59E-05	0.00E+00	1.89E-06
**6h**	0.00E+00	3.84E-07	8.92E-08	3.61E-06
**12h**	1.51E-06	1.90E-04	9.20E-06	6.60E-05
**24h**	2.24E-06	8.12E-05	1.78E-05	4.13E-05
**36h**	2.38E-05	5.71E-04	6.64E-05	1.47E-04
**48h**	2.07E-05	4.19E-04	4.81E-05	1.74E-04
**72h**	4.77E-05	1.05E-03	2.80E-04	2.27E-04
	cgd6_3840			
**2h**	2.28E-06	4.06E-06	4.24E-07	1.63E-06
**6h**	5.34E-07	2.02E-05	4.75E-07	2.90E-06
**12h**	8.84E-07	1.09E-06	3.10E-06	6.08E-06
**24h**	1.97E-06	8.10E-06	3.43E-06	2.56E-06
**36h**	1.60E-06	4.38E-06	2.69E-06	2.88E-06
**48h**	1.08E-06	1.79E-06	3.18E-06	2.36E-06
**72h**	8.90E-07	1.08E-06	8.59E-07	9.97E-07
	cgd6_3860			
**2h**	2.27E-04	7.47E-05	2.67E-04	2.66E-04
**6h**	1.60E-04	2.39E-04	3.10E-03	1.45E-03
**12h**	2.60E-06	1.53E-04	3.13E-05	1.56E-04
**24h**	2.36E-04	8.69E-05	6.22E-03	1.80E-04
**36h**	4.29E-04	1.26E-04	1.42E-03	1.15E-04

**48h**	2.22E-04	2.12E-04	7.62E-04	1.80E-04
**72h**	9.65E-05	1.07E-04	3.41E-04	1.13E-04
	cgd6_3870			
**2h**	1.51E-06	4.60E-06	9.48E-07	2.00E-07
**6h**	2.80E-06	2.44E-06	7.46E-06	1.11E-05
**12h**	1.21E-07	5.31E-07	1.19E-06	1.28E-06
**24h**	1.61E-06	1.95E-06	1.38E-05	4.47E-06
**36h**	1.24E-06	2.18E-06	1.30E-06	1.61E-06
**48h**	1.98E-06	1.93E-06	2.54E-06	9.60E-07
**72h**	6.56E-08	8.67E-07	1.22E-07	1.13E-07
	cgd6_3890			
**2h**	1.72E-05	5.58E-06	2.95E-05	1.25E-04
**6h**	1.97E-05	1.74E-05	1.16E-04	1.07E-03
**12h**	4.25E-05	5.55E-05	1.11E-04	7.25E-04
**24h**	6.29E-05	2.46E-04	1.40E-03	9.93E-04
**36h**	5.72E-04	1.94E-04	1.77E-03	6.37E-04
**48h**	1.19E-03	2.54E-04	5.33E-03	4.73E-03
**72h**	6.44E-04	4.22E-04	2.73E-03	1.33E-03
	cgd6_3900			
**2h**	1.73E-08	0.00E+00	0.00E+00	8.50E-09
**6h**	6.20E-07	2.34E-07	1.04E-06	1.10E-06
**12h**	4.76E-07	2.91E-07	4.30E-07	1.46E-07
**24h**	3.52E-07	3.67E-07	5.90E-06	9.46E-07
**36h**	1.72E-06	9.56E-07	1.06E-06	1.12E-06
**48h**	4.70E-07	8.71E-07	5.36E-07	6.69E-07
**72h**	1.85E-06	4.25E-05	8.22E-07	1.11E-06
	cgd6_2340			
**2h**	2.29E-06	1.70E-06	2.10E-06	5.71E-06
**6h**	8.74E-06	2.91E-06	2.68E-06	8.66E-06
**12h**	2.50E-07	4.39E-06	7.39E-07	3.67E-06
**24h**	3.03E-06	5.25E-06	3.31E-06	4.92E-06
**36h**	1.22E-06	1.85E-06	7.15E-07	2.01E-06
**48h**	5.25E-07	1.30E-06	6.49E-07	2.93E-07
**72h**	3.87E-07	1.41E-07	9.25E-08	9.44E-08
	cgd6_2350			
**2h**	2.19E-06	4.06E-07	1.70E-06	4.48E-06
**6h**	9.05E-07	7.54E-07	2.66E-06	3.43E-06
**12h**	8.51E-07	1.83E-06	8.86E-07	1.37E-05
**24h**	2.18E-06	3.01E-06	1.52E-06	3.86E-06
**36h**	5.37E-07	4.44E-07	4.58E-07	1.22E-06
**48h**	1.54E-07	5.03E-07	3.54E-07	4.10E-07
**72h**	1.88E-07	9.99E-08	7.77E-08	1.04E-07
	cgd6_2360			
**2h**	5.19E-07	1.59E-07	4.52E-07	3.91E-07
**6h**	4.75E-07	2.33E-07	1.91E-07	3.99E-07
**12h**	1.01E-07	2.14E-07	8.59E-08	1.11E-07
**24h**	8.97E-08	3.29E-07	1.53E-07	4.53E-07
**36h**	2.24E-07	1.65E-07	8.33E-08	1.38E-07
**48h**	5.62E-08	1.30E-07	1.07E-07	2.13E-05
**72h**	1.04E-07	2.74E-08	4.10E-08	1.97E-08
	cgd6_2370			
**2h**	1.11E-06	1.57E-07	4.84E-07	3.13E-07
**6h**	2.45E-07	5.74E-07	3.14E-07	2.09E-06

**12h**	0.00E+00	1.17E-07	4.22E-08	5.60E-08
**24h**	4.90E-08	4.86E-07	1.95E-07	7.02E-07
**36h**	3.63E-07	3.73E-07	1.47E-07	4.44E-07
**48h**	8.13E-08	2.47E-07	1.10E-07	2.20E-07
**72h**	2.03E-07	8.72E-08	4.77E-08	5.85E-08
	cgd6_2390			
**2h**	3.52E-05	4.39E-06	2.20E-05	6.50E-05
**6h**	5.10E-05	9.51E-05	1.88E-05	5.91E-04
**12h**	1.58E-06	2.02E-05	1.37E-06	7.62E-06
**24h**	2.41E-05	6.37E-05	9.86E-06	1.83E-04
**36h**	5.25E-05	2.34E-05	2.01E-05	4.04E-05
**48h**	1.05E-05	1.38E-05	5.68E-06	2.18E-05
**72h**	1.26E-05	2.50E-06	2.60E-06	4.13E-06
	cgd6_2410			
**2h**	1.48E-07	8.62E-09	8.24E-07	4.31E-07
**6h**	6.39E-06	2.66E-06	1.82E-06	1.60E-05
**12h**	1.07E-07	3.06E-07	6.90E-08	3.02E-07
**24h**	8.05E-07	4.10E-06	2.37E-07	6.63E-06
**36h**	2.29E-06	2.35E-06	2.49E-06	2.50E-06
**48h**	1.16E-06	6.04E-07	6.05E-07	1.28E-06
**72h**	6.89E-07	2.42E-07	2.71E-07	2.32E-07
	cgd6_2430			
**2h**	2.48E-05	2.93E-06	8.75E-06	1.47E-05
**6h**	6.16E-05	1.06E-04	4.13E-05	1.63E-04
**12h**	6.34E-05	1.88E-05	9.19E-06	1.76E-05
**24h**	1.84E-05	4.21E-05	1.79E-05	7.60E-05
**36h**	2.42E-05	7.68E-06	1.34E-05	1.27E-05
**48h**	7.04E-06	4.21E-06	5.41E-06	3.58E-06
**72h**	1.53E-06	7.87E-07	8.95E-07	9.97E-07
	cgd6_2440			
**2h**	7.76E-06	1.28E-07	1.83E-06	2.94E-05
**6h**	3.95E-07	1.26E-06	1.38E-06	1.36E-06
**12h**	2.28E-03	8.09E-04	7.67E-04	1.27E-03
**24h**	3.67E-04	6.24E-05	1.05E-04	3.74E-04
**36h**	2.41E-04	2.36E-04	1.54E-04	5.47E-04
**48h**	2.07E-04	4.78E-05	1.18E-04	2.12E-04
**72h**	9.64E-05	2.81E-05	4.22E-05	1.14E-04
	cgd6_2450			
**2h**	3.25E-06	1.96E-07	2.67E-06	2.25E-06
**6h**	5.15E-08	7.08E-08	4.48E-08	2.58E-07
**12h**	5.48E-05	4.28E-05	3.29E-05	2.76E-05
**24h**	1.74E-05	4.89E-06	1.29E-05	1.22E-05
**36h**	1.66E-05	3.80E-05	1.28E-05	2.33E-05
**48h**	4.58E-05	3.72E-05	3.88E-05	4.04E-05
**72h**	3.10E-05	1.80E-05	3.09E-05	1.55E-05
	cgd6_2460			
**2h**	3.36E-04	9.97E-05	2.23E-04	5.93E-04
**6h**	5.65E-04	9.53E-04	6.50E-04	2.61E-03
**12h**	3.04E-05	1.74E-04	4.11E-05	3.50E-04
**24h**	3.91E-04	5.46E-04	2.13E-04	9.01E-04
**36h**	9.98E-05	8.85E-05	6.52E-05	1.44E-04
**48h**	9.48E-05	1.01E-04	1.01E-04	1.20E-04
**72h**	5.92E-06	5.01E-06	8.79E-06	1.60E-05

	cgd6_2470			
**2h**	6.59E-06	1.21E-06	1.50E-06	2.19E-06
**6h**	1.70E-06	2.37E-06	1.77E-06	2.23E-06
**12h**	5.35E-07	1.03E-06	1.27E-07	7.34E-07
**24h**	2.66E-06	1.42E-06	1.25E-06	2.55E-06
**36h**	1.50E-06	1.08E-06	9.50E-07	8.83E-07
**48h**	2.72E-06	8.45E-07	1.25E-06	6.23E-07
**72h**	3.07E-07	2.86E-07	1.97E-07	7.89E-08
	cgd6_2480			
**2h**	2.87E-05	1.83E-05	3.75E-05	7.99E-05
**6h**	3.07E-05	1.94E-05	1.25E-05	5.66E-05
**12h**	8.13E-07	1.65E-05	5.23E-06	1.07E-05
**24h**	1.64E-05	1.07E-05	6.19E-06	2.88E-05
**36h**	1.01E-05	6.74E-06	6.92E-06	1.85E-05
**48h**	2.76E-06	3.48E-06	1.75E-06	4.77E-06
**72h**	5.23E-06	2.02E-06	9.37E-07	2.29E-06
	cgd6_2490			
**2h**	4.93E-07	2.30E-07	1.97E-08	5.97E-08
**6h**	4.38E-07	1.87E-07	1.05E-07	4.77E-08
**12h**	7.24E-09	4.35E-09	1.04E-07	2.75E-08
**24h**	2.15E-07	1.54E-07	1.19E-07	1.41E-07
**36h**	8.99E-08	1.60E-07	2.20E-07	1.75E-07
**48h**	2.01E-07	2.06E-07	1.19E-07	1.70E-07
**72h**	4.04E-08	5.32E-08	8.61E-08	6.61E-08
	cgd6_2500			
**2h**	4.56E-06	5.47E-07	3.22E-07	1.63E-06
**6h**	1.96E-07	6.43E-07	3.60E-07	4.19E-07
**12h**	7.57E-07	1.47E-07	2.93E-06	7.18E-07
**24h**	2.36E-06	1.59E-06	5.78E-07	8.71E-07
**36h**	3.57E-07	1.09E-06	9.84E-07	9.39E-07
**48h**	7.22E-07	3.73E-07	1.78E-07	3.19E-07
**72h**	2.14E-07	7.73E-08	1.42E-07	2.34E-07
	cgd6_2510			
**2h**	2.07E-06	6.31E-09	1.29E-07	1.63E-07
**6h**	2.96E-07	2.87E-07	2.51E-07	4.49E-07
**12h**	1.05E-07	2.99E-08	4.76E-07	1.62E-07
**24h**	9.08E-08	2.13E-07	7.71E-08	3.45E-07
**36h**	2.39E-07	1.84E-07	3.08E-07	2.83E-07
**48h**	2.92E-07	1.31E-07	1.44E-07	2.26E-07
**72h**	8.26E-08	1.44E-08	6.79E-08	4.97E-08
	cgd6_2520			
**2h**	6.97E-06	2.04E-08	2.50E-07	9.12E-07
**6h**	2.51E-07	9.06E-08	5.21E-07	3.29E-07
**12h**	1.35E-06	7.21E-08	9.80E-07	1.03E-06
**24h**	2.86E-06	5.26E-07	5.25E-07	1.19E-06
**36h**	1.53E-06	1.02E-06	1.37E-06	3.54E-06
**48h**	1.03E-05	1.92E-07	4.03E-07	1.28E-06
**72h**	1.69E-06	1.59E-07	8.93E-08	4.75E-07
	cgd6_2540			
**2h**	5.67E-07	4.04E-07	5.57E-07	8.23E-07
**6h**	3.77E-07	2.52E-07	1.44E-07	2.04E-07
**12h**	6.82E-07	7.13E-07	2.53E-06	6.13E-07
**24h**	1.86E-06	3.42E-07	1.22E-07	2.12E-07

**36h**	2.16E-07	3.43E-07	3.39E-07	3.72E-07
**48h**	2.93E-07	4.99E-08	1.45E-07	1.14E-07
**72h**	9.59E-08	8.75E-08	5.15E-08	1.93E-07
	cgd6_2550			
**2h**	1.61E-04	7.00E-06	4.58E-05	1.17E-04
**6h**	9.16E-05	5.29E-06	2.72E-05	8.34E-05
**12h**	1.99E-05	3.75E-06	5.10E-05	5.64E-05
**24h**	6.01E-04	3.22E-06	3.89E-05	9.14E-05
**36h**	1.54E-04	7.51E-06	2.90E-05	6.48E-05
**48h**	2.04E-04	1.85E-06	2.74E-05	6.37E-05
**72h**	4.79E-05	1.02E-06	4.95E-06	3.04E-05
	cgd6_2560			
**2h**	1.31E-06	6.91E-07	1.77E-06	3.40E-06
**6h**	3.19E-06	1.69E-06	1.29E-05	8.96E-06
**12h**	2.86E-06	8.11E-08	2.73E-06	1.08E-06
**24h**	2.38E-06	8.03E-07	7.78E-06	5.52E-06
**36h**	2.46E-06	1.43E-06	2.36E-06	0.00E+00
**48h**	1.95E-06	4.21E-07	2.68E-06	3.72E-06
**72h**	5.58E-07	7.23E-08	1.39E-07	1.62E-06
	cgd6_2570			
**2h**	2.05E-06	1.13E-06	6.16E-07	3.21E-06
**6h**	4.18E-05	8.25E-08	8.23E-07	4.34E-07
**12h**	1.08E-06	7.38E-07	6.78E-07	8.30E-07
**24h**	5.68E-07	2.07E-07	6.67E-07	6.02E-07
**36h**	8.28E-07	7.21E-07	7.91E-07	3.20E-06
**48h**	1.37E-06	2.51E-07	1.19E-06	1.97E-06
**72h**	3.01E-06	3.88E-07	1.90E-06	3.21E-06
	cgd6_2580			
**2h**	7.54E-08	4.43E-08	1.18E-07	3.62E-08
**6h**	2.83E-07	4.38E-07	3.91E-06	1.06E-06
**12h**	2.95E-07	2.52E-07	3.83E-07	2.65E-07
**24h**	3.28E-07	2.45E-07	1.91E-06	1.31E-06
**36h**	1.80E-06	9.51E-07	5.30E-07	2.39E-06
**48h**	6.53E-07	3.21E-07	6.91E-07	1.32E-06
**72h**	2.41E-07	1.10E-07	7.39E-08	4.52E-07
	cgd6_2590			
**2h**	1.65E-03	5.54E-05	8.53E-05	7.12E-04
**6h**	5.10E-04	3.35E-05	2.46E-04	2.32E-04
**12h**	6.50E-04	1.09E-05	1.29E-04	1.43E-04
**24h**	8.80E-04	1.62E-05	7.82E-05	2.50E-04
**36h**	2.11E-04	4.15E-05	8.78E-05	2.30E-04
**48h**	1.35E-04	2.30E-05	8.32E-05	2.73E-04
**72h**	7.25E-05	7.52E-06	5.16E-05	1.92E-04
	cgd6_2610			
**2h**	3.21E-07	7.37E-08	3.41E-08	6.41E-07
**6h**	5.54E-07	8.44E-08	4.83E-07	2.44E-07
**12h**	7.07E-08	1.45E-08	1.34E-07	2.92E-07
**24h**	3.26E-07	1.11E-07	4.06E-07	8.37E-07
**36h**	1.25E-06	5.38E-07	3.19E-07	1.80E-06
**48h**	8.43E-06	6.02E-07	1.08E-06	3.89E-06
**72h**	3.60E-06	2.32E-07	4.00E-07	2.37E-06
	cgd6_2620			
**2h**	4.98E-06	4.85E-06	3.53E-06	7.82E-06

**6h**	5.47E-06	7.42E-06	3.21E-05	5.75E-06
**12h**	4.42E-05	7.46E-05	1.79E-05	1.56E-05
**24h**	9.03E-06	1.74E-05	6.72E-06	8.03E-06
**36h**	1.00E-05	3.03E-05	6.67E-06	9.18E-06
**48h**	2.96E-06	5.17E-06	2.01E-06	8.28E-06
**72h**	2.33E-06	2.28E-06	1.17E-06	1.45E-06
	cgd6_2640			
**2h**	1.29E-06	1.99E-06	1.41E-06	1.40E-06
**6h**	2.07E-07	7.18E-06	6.00E-08	1.43E-06
**12h**	1.18E-06	5.11E-06	2.90E-06	6.76E-06
**24h**	1.51E-05	4.58E-06	8.92E-07	3.51E-06
**36h**	4.87E-06	4.39E-06	4.20E-06	9.05E-06
**48h**	6.81E-06	6.58E-06	1.50E-06	4.39E-06
**72h**	6.42E-07	1.09E-05	7.34E-07	2.20E-06
	cgd6_2660			
**2h**	1.36E-07	1.18E-07	2.33E-08	2.81E-07
**6h**	4.42E-07	7.70E-07	1.22E-06	1.22E-06
**12h**	1.35E-07	9.78E-08	1.95E-07	2.81E-07
**24h**	2.75E-07	2.92E-07	4.86E-07	7.02E-07
**36h**	5.31E-07	4.95E-07	5.88E-07	4.39E-07
**48h**	9.14E-07	2.39E-07	1.87E-07	2.62E-07
**72h**	1.76E-07	3.68E-08	4.37E-07	7.37E-08
	cgd6_2670			
**2h**	0.00E+00	0.00E+00	0.00E+00	0.00E+00
**6h**	0.00E+00	0.00E+00	0.00E+00	0.00E+00
**12h**	0.00E+00	0.00E+00	0.00E+00	3.42E-09
**24h**	6.28E-09	1.47E-08	2.03E-08	4.62E-09
**36h**	4.57E-07	5.49E-07	3.47E-07	1.23E-07
**48h**	2.31E-05	4.11E-06	1.20E-06	1.54E-06
**72h**	1.05E-06	3.99E-07	9.65E-07	9.24E-07
	cgd6_2680			
**2h**	0.00E+00	0.00E+00	0.00E+00	0.00E+00
**6h**	1.00E-08	3.93E-08	2.42E-09	0.00E+00
**12h**	9.72E-09	0.00E+00	1.43E-07	1.37E-08
**24h**	8.09E-08	5.45E-08	6.24E-08	6.21E-08
**36h**	1.26E-07	1.18E-07	2.17E-07	1.73E-07
**48h**	5.41E-06	3.81E-06	1.54E-06	1.46E-06
**72h**	1.21E-06	1.31E-07	3.76E-07	5.32E-07
	cgd6_2690			
**2h**	3.17E-06	1.27E-06	1.37E-06	8.34E-07
**6h**	2.31E-06	6.13E-06	3.86E-06	4.03E-06
**12h**	8.17E-07	1.25E-06	1.59E-06	1.45E-06
**24h**	1.68E-06	1.19E-06	2.23E-06	2.35E-06
**36h**	2.97E-06	3.65E-06	3.30E-06	5.35E-06
**48h**	3.28E-06	1.45E-06	1.06E-06	8.51E-07
**72h**	1.03E-06	7.82E-07	4.01E-07	1.00E-06
	cgd6_2700			
**2h**	0.00E+00	0.00E+00	1.69E-04	1.63E-04
**6h**	0.00E+00	7.67E-05	0.00E+00	7.02E-06
**12h**	7.32E-04	1.87E-03	1.57E-03	1.82E-02
**24h**	2.29E-04	1.51E-04	1.44E-03	1.99E-03
**36h**	6.73E-04	7.15E-04	2.92E-03	4.64E-03
**48h**	3.64E-04	6.29E-04	3.09E-03	5.37E-03

**72h**	1.11E-03	2.67E-04	3.80E-03	4.53E-03
	cgd6_2710			
**2h**	3.16E-08	0.00E+00	0.00E+00	0.00E+00
**6h**	0.00E+00	0.00E+00	0.00E+00	5.00E-09
**12h**	4.80E-08	1.05E-07	1.48E-07	5.05E-07
**24h**	8.77E-08	8.14E-08	2.48E-07	7.83E-08
**36h**	5.64E-07	3.11E-07	7.41E-07	5.90E-07
**48h**	1.96E-06	4.89E-07	9.87E-07	7.89E-07
**72h**	1.75E-06	1.19E-07	4.36E-07	5.63E-07
	cgd6_2720			
**2h**	4.64E-05	2.62E-05	2.78E-05	6.46E-05
**6h**	6.34E-06	5.82E-06	1.21E-05	1.21E-05
**12h**	2.40E-05	1.42E-05	2.78E-05	4.84E-05
**24h**	2.93E-05	3.26E-05	2.54E-05	1.70E-05
**36h**	1.03E-05	1.28E-05	9.70E-06	7.57E-06
**48h**	4.91E-05	6.69E-06	6.57E-06	8.52E-06
**72h**	3.32E-06	7.49E-07	7.87E-07	9.86E-07
	cgd6_2740			
**2h**	6.24E-05	3.86E-05	2.68E-06	1.07E-05
**6h**	2.40E-04	3.01E-04	6.38E-04	5.06E-04
**12h**	9.79E-06	2.83E-05	2.43E-05	3.83E-05
**24h**	1.93E-04	5.04E-05	1.53E-04	1.02E-04
**36h**	5.56E-05	6.29E-05	1.84E-05	2.55E-05
**48h**	3.40E-05	1.97E-05	1.04E-05	1.23E-05
**72h**	6.02E-05	1.89E-06	1.57E-06	3.28E-06
	cgd6_2750			
**2h**	1.81E-08	1.38E-08	2.34E-08	4.56E-08
**6h**	2.56E-08	2.30E-09	0.00E+00	3.91E-09
**12h**	2.60E-08	7.42E-09	1.78E-08	1.01E-08
**24h**	2.28E-08	2.27E-08	6.73E-08	4.39E-08
**36h**	3.14E-08	1.03E-07	2.66E-08	1.98E-08
**48h**	2.91E-07	1.60E-07	8.27E-08	8.11E-08
**72h**	1.22E-07	4.31E-08	3.01E-08	5.08E-08
	cgd6_2760			
**2h**	2.27E-04	6.95E-04	6.84E-05	2.46E-04
**6h**	2.35E-05	6.97E-05	2.43E-05	5.11E-05
**12h**	2.75E-04	4.87E-04	7.50E-05	1.35E-04
**24h**	1.17E-04	2.37E-04	4.97E-05	4.38E-05
**36h**	1.16E-04	3.69E-04	3.02E-05	5.19E-05
**48h**	4.84E-04	1.78E-04	2.24E-05	3.85E-05
**72h**	1.50E-04	3.11E-05	1.38E-05	2.81E-05
	cgd6_2770			
**2h**	2.16E-06	9.14E-06	8.34E-07	2.52E-06
**6h**	2.02E-07	8.83E-08	1.29E-06	4.12E-07
**12h**	4.91E-07	9.23E-07	4.40E-07	7.75E-07
**24h**	4.26E-07	4.27E-07	8.69E-07	8.02E-07
**36h**	6.63E-07	4.53E-07	4.91E-07	5.93E-07
**48h**	6.36E-07	2.62E-07	3.80E-07	3.24E-07
**72h**	7.13E-07	7.33E-07	5.08E-07	9.78E-07
	cgd6_2780			
**2h**	9.03E-08	0.00E+00	2.49E-08	1.55E-08
**6h**	1.94E-07	1.13E-06	2.72E-07	3.36E-07
**12h**	3.33E-07	8.72E-07	1.11E-07	5.05E-07

**24h**	1.83E-07	6.06E-07	1.22E-07	9.98E-07
**36h**	1.14E-06	1.23E-06	5.49E-07	4.77E-07
**48h**	2.54E-07	4.13E-07	2.94E-07	8.23E-07
**72h**	3.38E-07	1.17E-07	2.03E-07	3.31E-07
	cgd6_2790			
**2h**	1.14E-07	5.88E-08	2.63E-09	7.11E-08
**6h**	0.00E+00	1.41E-08	9.66E-10	0.00E+00
**12h**	1.96E-05	7.11E-06	4.90E-06	3.20E-06
**24h**	1.92E-06	3.95E-07	2.95E-06	6.50E-07
**36h**	9.03E-07	4.08E-06	4.52E-07	2.93E-06
**48h**	7.42E-06	4.23E-06	3.72E-06	6.43E-06
**72h**	1.16E-06	2.46E-07	2.69E-07	4.04E-07
	cgd6_2800			
**2h**	1.06E-04	0.00E+00	4.33E-06	1.54E-05
**6h**	5.49E-06	2.81E-05	8.01E-07	7.05E-06
**12h**	7.56E-06	2.98E-05	8.95E-06	6.98E-06
**24h**	7.94E-06	1.33E-04	1.34E-05	2.49E-05
**36h**	8.56E-05	1.55E-04	5.02E-05	9.24E-06
**48h**	2.16E-05	5.20E-05	4.99E-05	3.52E-05
**72h**	8.69E-05	1.71E-04	5.18E-05	1.49E-05
	cgd6_2810			
**2h**	1.04E-04	2.86E-03	4.35E-04	3.13E-04
**6h**	3.27E-04	5.05E-04	1.46E-04	1.76E-04
**12h**	2.25E-04	9.28E-04	4.97E-04	7.45E-05
**24h**	7.87E-05	1.15E-04	1.31E-04	1.77E-04
**36h**	1.87E-05	5.94E-04	1.19E-04	8.76E-05
**48h**	9.42E-07	1.76E-04	6.93E-05	4.46E-05
**72h**	6.48E-05	1.41E-04	2.44E-05	2.04E-05
	cgd6_2820			
**2h**	1.22E-06	2.14E-07	3.89E-07	7.93E-07
**6h**	1.06E-06	2.33E-06	4.61E-07	1.21E-06
**12h**	7.37E-07	1.34E-06	1.16E-07	5.32E-07
**24h**	8.06E-07	1.06E-06	3.42E-07	6.87E-07
**36h**	8.17E-08	4.07E-07	1.03E-07	1.94E-07
**48h**	7.77E-08	3.41E-08	8.16E-08	9.69E-08
**72h**	3.90E-08	5.48E-08	3.17E-08	2.07E-07
	cgd6_2850			
**2h**	3.51E-07	6.66E-06	9.75E-07	5.33E-07
**6h**	9.60E-05	6.58E-04	1.12E-04	1.86E-04
**12h**	1.28E-03	2.18E-05	3.23E-05	1.72E-05
**24h**	5.61E-05	2.09E-04	6.76E-05	1.74E-04
**36h**	7.18E-05	2.07E-04	9.85E-05	8.15E-05
**48h**	2.49E-04	1.28E-04	7.34E-05	8.02E-05
**72h**	2.74E-04	5.98E-05	3.95E-05	3.80E-05
	cgd6_2860			
**2h**	0.00E+00	0.00E+00	0.00E+00	0.00E+00
**6h**	8.99E-08	0.00E+00	7.43E-08	0.00E+00
**12h**	3.96E-05	4.11E-06	3.84E-06	1.98E-06
**24h**	2.07E-06	6.06E-06	2.13E-06	1.73E-06
**36h**	4.55E-06	1.16E-05	4.79E-06	3.72E-06
**48h**	2.37E-05	3.01E-05	1.96E-05	3.14E-05
**72h**	1.03E-04	3.79E-05	2.82E-05	2.45E-05
	cgd6_2870			

**2h**	4.62E-07	4.25E-07	1.77E-07	5.73E-07
**6h**	3.44E-07	1.04E-06	1.20E-07	1.05E-06
**12h**	3.23E-06	4.37E-06	1.73E-06	5.76E-07
**24h**	5.60E-07	2.53E-06	7.17E-07	1.78E-06
**36h**	6.67E-07	1.30E-06	1.73E-06	6.86E-07
**48h**	4.42E-07	7.13E-07	9.95E-07	1.18E-06
**72h**	1.47E-06	5.39E-07	9.07E-07	4.65E-07
	cgd6_2880			
**2h**	2.36E-06	9.24E-07	4.55E-06	2.98E-04
**6h**	2.52E-06	4.05E-07	2.74E-06	1.61E-04
**12h**	4.30E-05	1.12E-06	5.80E-06	1.35E-04
**24h**	2.38E-05	9.45E-06	1.04E-05	1.18E-04
**36h**	2.49E-05	2.89E-05	8.06E-06	1.04E-04
**48h**	7.88E-06	5.99E-06	6.08E-06	2.85E-04
**72h**	2.68E-06	3.21E-06	2.53E-06	3.89E-04
	cgd6_2890			
**2h**	4.53E-08	0.00E+00	0.00E+00	0.00E+00
**6h**	0.00E+00	0.00E+00	0.00E+00	0.00E+00
**12h**	1.15E-08	0.00E+00	5.60E-09	3.39E-07
**24h**	1.06E-08	1.38E-08	3.72E-08	7.49E-08
**36h**	4.37E-08	1.23E-07	6.73E-08	8.88E-07
**48h**	9.05E-09	6.83E-08	2.51E-08	1.71E-06
**72h**	1.12E-07	3.15E-08	3.78E-08	3.68E-07
	cgd6_2930			
**2h**	0.00E+00	0.00E+00	0.00E+00	0.00E+00
**6h**	1.04E-06	4.81E-06	2.97E-07	3.73E-06
**12h**	2.23E-06	2.74E-06	5.64E-07	1.24E-06
**24h**	1.95E-06	6.29E-06	1.06E-06	6.90E-06
**36h**	3.00E-06	9.02E-06	8.95E-06	8.01E-06
**48h**	2.62E-05	9.16E-06	1.22E-05	2.68E-05
**72h**	1.39E-05	7.23E-06	6.29E-06	9.11E-06
	cgd6_2940			
**2h**	0.00E+00	0.00E+00	0.00E+00	0.00E+00
**6h**	1.06E-07	3.45E-06	2.09E-06	4.15E-07
**12h**	3.08E-03	1.16E-04	9.14E-07	2.38E-05
**24h**	1.41E-04	1.33E-03	1.73E-05	8.88E-05
**36h**	3.83E-04	9.59E-04	6.26E-04	2.31E-04
**48h**	2.49E-04	5.64E-03	2.13E-04	3.02E-04
**72h**	9.82E-04	1.35E-03	2.20E-04	3.21E-04
	cgd6_3920			
**2h**	5.62E-06	2.24E-06	1.64E-06	1.91E-06
**6h**	1.86E-07	6.64E-08	1.40E-07	7.51E-08
**12h**	9.39E-06	8.05E-06	1.86E-06	1.94E-05
**24h**	1.49E-05	1.22E-06	2.03E-06	5.88E-06
**36h**	8.70E-07	9.09E-07	5.20E-07	1.45E-06
**48h**	2.94E-06	9.00E-07	3.53E-07	5.58E-07
**72h**	3.14E-07	2.51E-07	9.27E-08	3.05E-07
	cgd6_3950			
**2h**	0.00E+00	0.00E+00	1.63E-09	0.00E+00
**6h**	9.93E-08	1.87E-07	2.45E-07	3.85E-07
**12h**	7.71E-08	2.39E-09	1.60E-09	7.61E-09
**24h**	1.36E-07	6.41E-07	7.17E-08	7.85E-08
**36h**	6.63E-08	8.87E-08	4.22E-08	4.39E-07

**48h**	2.67E-07	8.95E-08	3.37E-08	2.07E-07
**72h**	2.89E-08	1.10E-08	5.97E-08	2.82E-08
	cgd6_3960			
**2h**	5.65E-07	1.47E-07	3.51E-07	4.75E-07
**6h**	6.99E-07	1.58E-07	9.58E-07	6.05E-07
**12h**	5.22E-07	1.62E-06	1.92E-07	4.04E-07
**24h**	1.46E-06	4.93E-07	2.51E-07	1.08E-06
**36h**	1.71E-07	3.02E-07	2.85E-07	3.49E-07
**48h**	4.22E-07	1.82E-07	8.47E-08	2.45E-07
**72h**	4.44E-07	2.62E-07	3.36E-07	3.08E-07
	cgd6_3990			
**2h**	1.19E-04	3.36E-05	1.60E-04	2.15E-04
**6h**	2.55E-03	1.07E-03	1.25E-03	2.47E-03
**12h**	1.42E-04	4.05E-04	1.15E-04	1.61E-04
**24h**	4.92E-04	8.05E-04	6.79E-04	5.11E-04
**36h**	2.11E-04	1.58E-04	2.00E-04	4.23E-04
**48h**	8.07E-05	8.27E-05	6.17E-05	8.15E-05
**72h**	1.81E-05	7.21E-06	8.68E-06	1.69E-05
	cgd6_4000			
**2h**	0.00E+00	0.00E+00	0.00E+00	0.00E+00
**6h**	0.00E+00	0.00E+00	0.00E+00	0.00E+00
**12h**	2.63E-06	1.39E-06	7.85E-08	1.63E-06
**24h**	2.48E-07	1.76E-08	4.17E-09	2.38E-07
**36h**	1.07E-07	1.91E-07	1.18E-07	1.93E-07
**48h**	4.75E-08	5.82E-08	1.88E-08	7.57E-08
**72h**	1.09E-07	9.68E-08	6.42E-08	1.23E-07
	cgd6_4020			
**2h**	0.00E+00	0.00E+00	0.00E+00	0.00E+00
**6h**	0.00E+00	0.00E+00	0.00E+00	0.00E+00
**12h**	1.25E-06	6.69E-05	6.95E-07	8.58E-06
**24h**	2.87E-06	1.33E-05	3.41E-07	9.47E-06
**36h**	2.99E-05	4.10E-05	3.14E-06	6.01E-05
**48h**	4.12E-04	4.68E-04	8.07E-06	1.92E-04
**72h**	1.28E-03	6.32E-04	1.18E-04	2.95E-03
	cgd6_4030			
**2h**	0.00E+00	0.00E+00	0.00E+00	0.00E+00
**6h**	3.50E-07	0.00E+00	2.79E-08	1.08E-05
**12h**	5.56E-03	1.19E-02	1.24E-04	7.30E-03
**24h**	6.59E-04	1.63E-03	2.39E-05	1.45E-03
**36h**	2.80E-04	4.32E-03	1.25E-04	6.49E-04
**48h**	6.00E-04	8.12E-04	4.94E-05	5.93E-04
**72h**	6.03E-04	4.04E-03	1.01E-04	5.47E-04
	cgd6_4040			
**2h**	8.61E-09	0.00E+00	1.04E-08	3.72E-09
**6h**	1.06E-06	2.96E-07	2.80E-07	4.90E-07
**12h**	1.77E-07	1.23E-07	3.88E-08	1.11E-07
**24h**	1.73E-07	7.24E-07	3.28E-07	4.21E-07
**36h**	7.36E-07	2.59E-07	3.84E-07	8.61E-07
**48h**	5.33E-07	5.41E-07	2.82E-07	4.58E-07
**72h**	1.95E-06	8.09E-07	8.76E-07	8.85E-07
	cgd6_4050			
**2h**	1.41E-08	0.00E+00	0.00E+00	1.30E-08
**6h**	6.20E-07	4.69E-07	2.06E-07	7.38E-07

**12h**	1.87E-07	2.08E-07	5.20E-08	6.64E-07
**24h**	4.66E-07	5.92E-07	3.29E-07	4.59E-07
**36h**	1.10E-06	5.16E-07	4.93E-07	6.90E-07
**48h**	1.58E-06	1.26E-06	7.51E-07	7.16E-07
**72h**	5.12E-06	2.26E-06	2.79E-06	2.42E-06
	cgd6_4060			
**2h**	1.99E-08	1.96E-08	1.21E-08	4.41E-09
**6h**	2.69E-08	2.47E-08	1.91E-09	4.41E-08
**12h**	5.18E-08	7.88E-09	3.63E-09	3.65E-08
**24h**	3.34E-08	9.25E-08	4.37E-08	6.47E-08
**36h**	1.45E-07	7.57E-08	1.56E-08	8.11E-08
**48h**	6.87E-07	7.35E-07	1.52E-07	4.10E-07
**72h**	1.34E-06	1.32E-06	2.72E-07	5.18E-07
	cgd6_4070			
**2h**	1.10E-03	2.13E-04	2.17E-03	3.86E-04
**6h**	9.12E-04	2.13E-04	6.53E-04	5.19E-04
**12h**	2.47E-04	6.05E-05	1.29E-03	8.75E-05
**24h**	4.59E-04	9.63E-05	4.18E-03	3.55E-04
**36h**	2.93E-04	5.96E-05	1.47E-03	1.69E-04
**48h**	6.90E-04	1.97E-04	4.22E-03	8.67E-04
**72h**	6.37E-04	8.76E-05	4.42E-04	2.60E-04
	cgd6_4080			
**2h**	2.70E-08	0.00E+00	1.46E-06	2.50E-08
**6h**	6.54E-08	1.17E-07	8.39E-08	1.39E-06
**12h**	1.12E-06	5.93E-06	1.60E-07	1.22E-06
**24h**	3.33E-07	4.28E-07	3.50E-07	2.11E-07
**36h**	9.83E-07	5.12E-07	6.73E-07	1.16E-06
**48h**	2.16E-06	1.71E-06	1.96E-07	1.14E-06
**72h**	1.80E-06	2.15E-05	9.68E-07	1.04E-06
	cgd6_2950			
**2h**	2.04E-06	1.63E-06	5.71E-07	6.45E-07
**6h**	4.65E-07	4.27E-06	7.91E-07	1.05E-06
**12h**	1.18E-06	8.69E-06	1.04E-06	8.04E-07
**24h**	1.14E-06	1.35E-05	9.52E-06	2.71E-06
**36h**	5.22E-06	1.08E-05	1.24E-05	5.03E-06
**48h**	3.36E-06	9.83E-06	6.07E-06	4.06E-06
**72h**	1.86E-06	1.04E-06	2.65E-06	2.46E-06
	cgd6_2960			
**2h**	4.71E-06	7.82E-06	2.74E-06	2.25E-06
**6h**	5.61E-06	1.51E-05	9.07E-06	6.39E-06
**12h**	1.92E-07	3.95E-06	1.29E-06	3.21E-07
**24h**	2.27E-06	4.84E-06	4.68E-06	5.08E-06
**36h**	1.84E-06	3.50E-06	2.63E-06	1.78E-06
**48h**	9.85E-07	4.42E-06	2.33E-06	9.13E-07
**72h**	4.30E-07	2.99E-07	5.70E-07	3.81E-07
	cgd6_2970			
**2h**	6.18E-05	1.13E-04	2.07E-05	5.95E-05
**6h**	5.67E-05	1.72E-04	1.15E-05	1.03E-04
**12h**	5.57E-07	2.71E-05	1.37E-06	2.80E-06
**24h**	5.50E-06	4.78E-05	5.35E-06	1.97E-05
**36h**	4.90E-06	1.06E-05	3.14E-06	1.20E-05
**48h**	1.13E-05	2.09E-05	2.57E-06	7.25E-06
**72h**	3.67E-06	8.85E-06	2.06E-06	2.75E-06

	cgd6_2980			
**2h**	2.11E-03	1.87E-03	1.67E-04	2.86E-03
**6h**	1.32E-03	4.63E-03	2.13E-04	7.03E-03
**12h**	4.95E-05	2.89E-04	9.93E-06	7.43E-05
**24h**	2.55E-04	2.24E-03	1.09E-04	1.04E-03
**36h**	5.60E-04	6.45E-04	7.82E-05	8.73E-04
**48h**	4.05E-04	4.51E-04	1.93E-05	7.29E-04
**72h**	1.19E-04	3.20E-04	3.26E-05	2.73E-04
	cgd6_2990			
**2h**	2.64E-06	1.23E-06	5.99E-07	1.30E-06
**6h**	3.23E-07	9.20E-08	5.64E-08	0.00E+00
**12h**	1.36E-07	9.68E-07	9.25E-08	4.73E-08
**24h**	2.81E-07	2.43E-07	4.17E-07	1.47E-07
**36h**	9.02E-07	1.01E-06	1.07E-06	5.47E-07
**48h**	1.38E-06	1.25E-06	6.15E-07	7.32E-07
**72h**	1.17E-06	1.68E-06	8.90E-07	9.86E-07
	cgd6_3000			
**2h**	3.02E-06	1.18E-06	6.16E-07	4.36E-07
**6h**	7.08E-08	3.31E-07	7.71E-08	1.30E-07
**12h**	1.42E-10	1.32E-07	5.16E-08	2.38E-09
**24h**	9.97E-08	1.42E-07	1.43E-07	7.37E-08
**36h**	2.54E-07	4.39E-07	3.42E-07	2.51E-07
**48h**	3.66E-07	3.27E-07	1.88E-07	2.07E-07
**72h**	3.26E-07	4.06E-07	3.80E-07	4.79E-07
	cgd6_3010			
**2h**	1.45E-04	9.83E-05	1.60E-04	6.65E-05
**6h**	9.45E-05	2.13E-04	1.27E-04	1.04E-04
**12h**	1.55E-05	3.80E-04	4.69E-05	9.64E-06
**24h**	9.59E-05	1.35E-04	2.74E-04	1.00E-04
**36h**	5.96E-05	5.86E-05	2.67E-05	4.40E-05
**48h**	1.92E-05	3.65E-05	4.15E-05	3.04E-05
**72h**	1.70E-05	1.13E-05	8.48E-06	7.81E-06
	cgd6_3020			
**2h**	0.00E+00	0.00E+00	6.50E-10	0.00E+00
**6h**	0.00E+00	0.00E+00	0.00E+00	0.00E+00
**12h**	1.08E-07	7.76E-08	1.66E-07	1.28E-07
**24h**	3.37E-08	6.22E-08	6.60E-08	2.51E-07
**36h**	1.30E-07	1.79E-07	3.26E-07	9.01E-08
**48h**	1.44E-07	9.19E-08	1.47E-07	2.56E-07
**72h**	1.24E-07	1.10E-07	9.16E-08	1.21E-07
	cgd6_3030			
**2h**	0.00E+00	0.00E+00	6.87E-09	0.00E+00
**6h**	1.04E-08	0.00E+00	0.00E+00	0.00E+00
**12h**	5.10E-07	4.97E-06	5.00E-07	4.39E-06
**24h**	6.99E-07	1.43E-05	2.02E-06	2.47E-05
**36h**	3.89E-06	6.08E-06	2.65E-06	9.12E-06
**48h**	1.74E-05	1.40E-05	4.16E-06	1.30E-05
**72h**	3.49E-05	2.02E-05	3.70E-06	1.83E-05
	cgd6_3040			
**2h**	3.78E-03	9.29E-04	1.42E-03	3.45E-04
**6h**	1.62E-04	1.80E-03	5.72E-03	2.64E-04
**12h**	4.90E-04	1.38E-03	3.63E-04	7.29E-05
**24h**	7.50E-05	3.56E-04	4.02E-03	8.75E-05

**36h**	2.55E-04	1.08E-03	6.39E-04	1.15E-04
**48h**	1.14E-04	3.58E-04	1.06E-03	5.31E-05
**72h**	2.47E-04	3.19E-04	4.41E-04	1.48E-04
	cgd6_3050			
**2h**	3.02E-05	3.12E-06	2.78E-06	1.20E-04
**6h**	3.27E-06	1.34E-05	5.23E-06	1.94E-05
**12h**	1.12E-03	3.98E-03	1.65E-04	1.08E-03
**24h**	1.58E-04	3.02E-04	2.11E-04	8.38E-04
**36h**	6.25E-04	1.45E-03	4.19E-04	1.08E-03
**48h**	4.22E-04	9.01E-04	4.76E-05	4.95E-04
**72h**	3.67E-05	1.93E-05	6.03E-06	3.26E-05
	cgd6_3060			
**2h**	0.00E+00	0.00E+00	0.00E+00	0.00E+00
**6h**	0.00E+00	0.00E+00	0.00E+00	0.00E+00
**12h**	3.20E-09	1.50E-08	2.13E-09	0.00E+00
**24h**	1.53E-08	2.14E-07	4.41E-08	1.88E-08
**36h**	1.63E-06	7.20E-07	1.56E-07	2.50E-06
**48h**	2.06E-05	7.77E-05	8.59E-07	4.21E-05
**72h**	1.54E-06	8.27E-05	1.40E-07	3.54E-06
	cgd6_4090			
**2h**	0.00E+00	0.00E+00	0.00E+00	0.00E+00
**6h**	0.00E+00	0.00E+00	0.00E+00	0.00E+00
**12h**	1.31E-09	0.00E+00	8.03E-10	3.20E-10
**24h**	1.02E-08	4.65E-09	5.78E-09	2.77E-09
**36h**	1.78E-07	8.11E-08	1.69E-08	2.69E-08
**48h**	4.75E-07	3.53E-07	2.84E-07	3.45E-07
**72h**	8.84E-07	7.36E-07	9.61E-07	3.90E-07
	cgd6_4100			
**2h**	5.77E-06	0.00E+00	4.43E-06	1.52E-07
**6h**	0.00E+00	0.00E+00	0.00E+00	4.52E-08
**12h**	1.01E-05	1.59E-05	5.77E-05	5.95E-07
**24h**	1.07E-04	6.34E-05	3.80E-05	3.77E-06
**36h**	1.28E-04	1.46E-04	8.10E-05	4.81E-06
**48h**	8.11E-04	4.82E-04	7.41E-04	3.57E-05
**72h**	1.51E-03	1.23E-03	9.14E-04	7.91E-05
	cgd6_4110			
**2h**	5.24E-04	3.88E-04	1.12E-04	2.95E-05
**6h**	5.79E-05	4.56E-04	5.86E-04	5.47E-05
**12h**	1.29E-03	2.10E-03	2.12E-03	6.44E-05
**24h**	6.16E-04	1.70E-03	3.58E-04	6.53E-05
**36h**	6.03E-04	4.88E-04	1.10E-03	1.70E-04
**48h**	1.31E-03	3.77E-04	2.36E-04	4.70E-05
**72h**	3.91E-04	1.87E-04	3.85E-04	3.75E-05
	cgd6_4120			
**2h**	2.77E-06	3.51E-06	1.23E-06	5.38E-07
**6h**	1.45E-06	4.16E-06	3.09E-06	3.66E-07
**12h**	1.23E-07	1.23E-07	1.25E-07	3.99E-08
**24h**	2.24E-06	1.53E-06	8.30E-07	4.71E-07
**36h**	2.05E-06	1.22E-06	1.78E-06	5.18E-07
**48h**	2.27E-06	3.74E-07	4.77E-07	2.09E-07
**72h**	5.55E-07	4.21E-07	4.27E-07	2.93E-07
	cgd6_4140			
**2h**	0.00E+00	0.00E+00	0.00E+00	0.00E+00

**6h**	0.00E+00	3.75E-08	1.65E-07	2.16E-09
**12h**	3.48E-06	4.40E-06	6.25E-06	8.19E-07
**24h**	4.86E-06	3.31E-06	4.44E-06	1.10E-06
**36h**	1.44E-05	9.95E-06	1.29E-05	9.28E-06
**48h**	8.38E-06	4.41E-06	8.90E-06	7.71E-07
**72h**	1.17E-05	4.53E-06	1.40E-05	3.11E-06
	cgd6_4150			
**2h**	1.06E-08	3.13E-09	0.00E+00	2.04E-08
**6h**	8.90E-08	1.06E-07	2.03E-07	8.54E-08
**12h**	1.72E-06	3.36E-06	1.70E-06	2.77E-07
**24h**	3.86E-07	3.44E-07	2.28E-07	4.20E-07
**36h**	1.15E-06	9.83E-07	4.16E-07	7.18E-07
**48h**	1.22E-07	2.23E-07	2.46E-07	2.16E-07
**72h**	3.09E-07	1.53E-07	1.19E-07	2.04E-07
	cgd6_4160			
**2h**	6.32E-09	0.00E+00	1.70E-08	0.00E+00
**6h**	0.00E+00	0.00E+00	0.00E+00	0.00E+00
**12h**	1.40E-05	3.67E-05	1.71E-05	1.87E-06
**24h**	1.88E-06	2.66E-06	5.28E-07	3.92E-07
**36h**	4.76E-07	9.03E-07	2.13E-06	1.06E-06
**48h**	2.63E-07	2.16E-07	3.06E-07	1.06E-07
**72h**	2.75E-07	1.06E-07	9.73E-08	4.97E-08
	cgd6_4170			
**2h**	3.19E-04	4.65E-04	2.85E-04	5.48E-05
**6h**	9.08E-04	1.03E-03	6.95E-04	9.53E-05
**12h**	1.63E-03	6.23E-03	4.59E-03	2.79E-04
**24h**	7.02E-04	3.85E-03	3.73E-04	1.01E-04
**36h**	3.27E-04	4.50E-04	9.40E-04	1.75E-04
**48h**	4.58E-04	1.26E-04	5.20E-04	5.80E-05
**72h**	1.53E-04	8.37E-05	1.14E-04	2.26E-05
	cgd6_4180			
**2h**	3.21E-07	1.79E-06	4.16E-07	3.93E-07
**6h**	2.23E-06	2.81E-06	7.29E-07	9.37E-07
**12h**	2.54E-06	6.02E-06	4.83E-06	2.21E-06
**24h**	1.97E-06	2.47E-06	6.25E-07	1.45E-06
**36h**	8.49E-07	1.02E-06	1.43E-06	2.23E-06
**48h**	7.22E-07	5.41E-07	4.51E-07	7.26E-07
**72h**	2.41E-07	1.40E-07	1.77E-07	2.16E-07
	cgd6_4200			
**2h**	5.31E-07	1.12E-07	3.07E-07	2.31E-07
**6h**	5.60E-09	1.54E-08	1.66E-08	3.07E-08
**12h**	4.34E-07	6.69E-07	1.43E-06	1.93E-07
**24h**	3.25E-07	4.57E-07	2.48E-07	3.56E-07
**36h**	8.42E-07	7.97E-07	1.24E-06	8.36E-07
**48h**	5.51E-07	6.42E-07	9.61E-07	7.73E-07
**72h**	3.19E-07	2.46E-07	1.04E-07	1.65E-07
	cgd6_4220			
**2h**	8.66E-07	3.46E-07	1.26E-06	1.04E-06
**6h**	3.87E-07	2.70E-07	1.22E-07	1.43E-07
**12h**	1.96E-06	9.62E-07	1.92E-06	3.57E-07
**24h**	2.13E-06	9.18E-07	5.06E-07	7.78E-07
**36h**	1.08E-06	4.82E-07	1.35E-06	5.51E-07
**48h**	2.53E-06	6.65E-07	4.15E-07	9.71E-07

**72h**	4.04E-07	1.82E-07	1.39E-07	6.17E-08
	cgd6_4230			
**2h**	3.09E-05	2.19E-05	1.01E-04	5.01E-06
**6h**	2.46E-06	1.44E-05	4.23E-06	4.35E-06
**12h**	1.07E-05	1.48E-05	1.80E-05	3.79E-06
**24h**	8.69E-06	1.36E-05	4.35E-06	4.82E-06
**36h**	4.63E-06	1.19E-05	8.78E-06	5.89E-06
**48h**	2.84E-06	2.30E-06	2.84E-06	9.61E-07
**72h**	2.01E-06	2.85E-06	1.10E-05	6.16E-07
	cgd6_4240			
**2h**	0.00E+00	0.00E+00	0.00E+00	0.00E+00
**6h**	0.00E+00	2.50E-09	4.27E-08	0.00E+00
**12h**	7.53E-08	1.19E-07	7.43E-07	1.33E-07
**24h**	2.14E-07	8.09E-08	1.22E-07	2.12E-07
**36h**	3.12E-07	2.51E-07	6.08E-07	1.59E-07
**48h**	4.41E-07	2.93E-07	5.25E-07	5.84E-07
**72h**	7.05E-07	1.45E-07	5.93E-07	3.76E-07
	cgd6_4270			
**2h**	3.30E-08	5.47E-08	2.93E-07	1.82E-07
**6h**	1.98E-08	3.44E-08	1.60E-08	3.32E-08
**12h**	3.02E-06	5.25E-07	2.54E-06	5.36E-07
**24h**	1.24E-06	2.32E-06	5.45E-07	3.07E-07
**36h**	1.14E-06	6.52E-07	1.78E-06	6.97E-07
**48h**	4.76E-07	4.68E-07	6.08E-07	3.76E-07
**72h**	8.73E-07	2.59E-07	1.58E-07	2.89E-07
	cgd6_4280			
**2h**	1.90E-05	1.25E-05	9.52E-06	4.58E-05
**6h**	1.68E-05	4.30E-06	1.18E-05	1.04E-05
**12h**	2.39E-05	8.25E-06	6.35E-05	1.65E-05
**24h**	3.76E-05	2.71E-05	2.29E-05	1.54E-05
**36h**	9.83E-06	5.88E-06	1.63E-05	9.34E-06
**48h**	7.05E-06	3.49E-06	3.51E-06	9.58E-06
**72h**	3.74E-06	7.51E-07	2.30E-06	3.35E-06
	cgd6_4290			
**2h**	2.18E-05	3.25E-05	3.59E-06	1.24E-05
**6h**	3.44E-04	3.97E-04	9.75E-04	3.90E-04
**12h**	3.64E-05	4.02E-05	1.04E-04	2.47E-05
**24h**	3.88E-05	6.70E-05	2.22E-04	1.77E-04
**36h**	7.76E-05	1.37E-04	1.19E-04	5.35E-05
**48h**	2.71E-05	2.47E-05	2.64E-05	3.27E-05
**72h**	7.76E-06	4.60E-06	3.38E-06	6.08E-06
	cgd6_4310			
**2h**	5.89E-05	8.83E-06	0.00E+00	9.69E-05
**6h**	1.56E-03	6.10E-05	3.59E-04	1.13E-03
**12h**	7.42E-04	1.22E-05	1.38E-04	1.12E-04
**24h**	3.26E-04	1.95E-05	1.44E-04	1.32E-04
**36h**	7.80E-04	5.26E-05	1.15E-04	2.33E-04
**48h**	6.91E-04	3.58E-05	1.38E-04	1.28E-04
**72h**	1.00E-04	9.28E-06	4.94E-05	2.37E-04
	cgd6_4320			
**2h**	3.42E-06	3.03E-06	1.85E-06	1.61E-06
**6h**	9.63E-06	5.43E-06	1.30E-05	6.33E-06
**12h**	1.98E-06	4.90E-07	5.06E-06	1.21E-06

**24h**	1.37E-06	1.38E-06	2.65E-06	2.04E-06
**36h**	2.89E-06	1.80E-06	1.72E-06	6.25E-07
**48h**	5.36E-07	6.14E-07	1.33E-06	3.89E-07
**72h**	3.03E-07	2.70E-07	1.72E-07	3.56E-07
	cgd6_4330			
**2h**	0.00E+00	0.00E+00	0.00E+00	0.00E+00
**6h**	0.00E+00	0.00E+00	0.00E+00	0.00E+00
**12h**	1.83E-06	1.33E-06	4.80E-06	3.83E-07
**24h**	3.72E-07	1.35E-06	3.36E-07	2.53E-07
**36h**	2.35E-06	2.04E-06	3.32E-06	1.52E-06
**48h**	1.21E-06	6.19E-07	9.47E-07	1.03E-06
**72h**	7.28E-07	3.09E-07	7.85E-07	1.47E-06
	cgd6_4340			
**2h**	5.83E-06	1.56E-06	7.54E-07	1.48E-05
**6h**	2.33E-06	8.72E-07	9.10E-06	2.17E-06
**12h**	3.62E-06	1.36E-06	1.22E-05	1.66E-06
**24h**	7.61E-07	1.00E-06	4.76E-06	2.32E-06
**36h**	3.81E-06	3.62E-06	5.15E-06	4.21E-06
**48h**	1.65E-06	2.15E-06	2.84E-06	3.14E-06
**72h**	8.18E-07	7.91E-07	1.49E-06	1.63E-06
	cgd6_4350			
**2h**	1.34E-06	3.59E-06	2.91E-07	2.06E-06
**6h**	2.22E-05	9.75E-06	6.06E-05	2.91E-05
**12h**	1.34E-05	3.62E-06	1.86E-05	6.57E-06
**24h**	7.25E-06	5.07E-06	4.94E-05	1.40E-05
**36h**	1.82E-05	2.84E-05	3.26E-05	2.69E-05
**48h**	1.50E-05	1.53E-05	1.02E-05	5.57E-05
**72h**	5.42E-07	9.44E-07	2.63E-06	1.91E-06
	cgd6_4360			
**2h**	6.16E-07	7.98E-08	1.14E-08	1.07E-07
**6h**	3.86E-06	2.00E-06	2.42E-05	1.05E-05
**12h**	4.20E-06	3.80E-06	6.85E-06	7.20E-07
**24h**	1.47E-06	5.42E-07	8.80E-06	3.28E-06
**36h**	3.40E-06	4.21E-06	4.31E-06	2.11E-06
**48h**	6.79E-03	4.04E-06	3.14E-06	4.63E-06
**72h**	3.28E-07	2.21E-07	2.69E-07	4.36E-07
	cgd6_4370			
**2h**	2.03E-06	2.46E-05	7.55E-07	1.54E-06
**6h**	1.49E-06	7.48E-05	1.17E-05	1.36E-05
**12h**	1.68E-06	8.99E-06	1.97E-05	3.57E-06
**24h**	4.44E-06	8.72E-06	1.86E-05	1.08E-05
**36h**	3.07E-06	2.59E-05	1.27E-05	5.41E-06
**48h**	1.03E-05	7.07E-05	2.08E-05	1.98E-05
**72h**	2.45E-06	1.42E-05	1.35E-05	1.95E-05
	cgd6_4380			
**2h**	0.00E+00	0.00E+00	0.00E+00	0.00E+00
**6h**	0.00E+00	0.00E+00	0.00E+00	0.00E+00
**12h**	9.19E-07	7.61E-07	4.13E-06	8.12E-07
**24h**	4.51E-07	2.61E-06	2.96E-06	1.20E-06
**36h**	5.94E-06	2.49E-05	1.62E-05	2.78E-06
**48h**	2.04E-06	1.32E-05	0.00E+00	2.58E-06
**72h**	1.24E-03	1.49E-05	5.15E-06	1.33E-06
	cgd6_4390			

**2h**	0.00E+00	0.00E+00	0.00E+00	0.00E+00
**6h**	1.43E-07	2.83E-07	1.38E-07	6.15E-07
**12h**	1.25E-06	1.17E-06	3.08E-06	1.74E-06
**24h**	5.48E-07	1.38E-06	1.91E-06	1.33E-06
**36h**	2.40E-06	1.19E-06	5.75E-06	3.02E-06
**48h**	1.41E-06	1.30E-06	2.29E-06	2.79E-06
**72h**	9.22E-07	1.85E-07	6.02E-07	5.65E-07
	cgd6_4400			
**2h**	2.57E-05	1.79E-05	1.79E-04	5.41E-05
**6h**	1.86E-04	2.63E-04	2.03E-03	7.75E-04
**12h**	3.44E-06	1.12E-05	1.05E-03	1.30E-04
**24h**	5.76E-05	8.83E-05	4.66E-03	5.13E-04
**36h**	6.67E-05	6.67E-05	7.22E-04	1.78E-04
**48h**	7.20E-05	6.77E-05	9.75E-04	2.34E-04
**72h**	3.36E-05	2.81E-05	5.72E-04	2.03E-04
	cgd6_4410			
**2h**	0.00E+00	0.00E+00	0.00E+00	3.90E-09
**6h**	4.16E-08	8.80E-08	1.12E-07	2.10E-07
**12h**	2.46E-08	1.47E-08	8.82E-08	8.07E-08
**24h**	4.58E-08	5.62E-08	4.58E-07	5.30E-07
**36h**	5.73E-07	3.03E-07	6.82E-07	2.68E-07
**48h**	2.97E-07	1.35E-07	3.25E-07	4.42E-07
**72h**	5.31E-07	1.41E-07	2.91E-06	4.57E-07
	cgd6_4420			
**2h**	6.49E-08	1.06E-07	0.00E+00	9.67E-09
**6h**	4.50E-09	1.82E-08	1.05E-08	1.99E-08
**12h**	7.20E-08	6.75E-07	7.64E-07	3.12E-07
**24h**	1.21E-07	2.37E-07	1.78E-06	8.64E-07
**36h**	1.87E-06	2.38E-06	4.52E-06	2.15E-06
**48h**	3.11E-06	2.76E-06	1.12E-05	6.05E-06
**72h**	8.70E-06	7.58E-06	2.06E-05	1.52E-05
	cgd6_4440			
**2h**	4.61E-08	0.00E+00	0.00E+00	0.00E+00
**6h**	0.00E+00	0.00E+00	0.00E+00	0.00E+00
**12h**	9.31E-07	5.09E-06	5.60E-05	1.66E-06
**24h**	2.76E-06	2.65E-06	2.38E-05	1.37E-05
**36h**	6.25E-06	2.55E-06	4.90E-05	1.25E-05
**48h**	2.11E-04	1.36E-04	1.83E-03	6.53E-04
**72h**	6.73E-04	2.95E-04	2.78E-03	1.27E-03
	cgd6_4460			
**2h**	2.40E-08	4.22E-08	1.97E-08	6.00E-09
**6h**	4.83E-07	3.15E-07	4.26E-07	1.14E-06
**12h**	1.87E-04	3.12E-04	1.58E-04	2.69E-04
**24h**	1.96E-05	1.71E-05	1.13E-05	1.03E-05
**36h**	6.63E-06	7.49E-06	1.46E-05	5.97E-05
**48h**	8.77E-06	5.85E-06	1.42E-05	9.68E-06
**72h**	3.42E-06	7.05E-06	5.01E-06	1.25E-05
	cgd6_4470			
**2h**	2.58E-04	1.65E-04	5.25E-04	3.16E-04
**6h**	1.93E-04	1.50E-04	2.00E-03	6.68E-04
**12h**	5.88E-05	5.71E-05	9.92E-04	3.93E-04
**24h**	1.88E-04	8.35E-05	7.28E-04	3.80E-04
**36h**	9.29E-05	9.18E-05	3.76E-04	1.76E-04

**48h**	7.04E-05	1.36E-05	2.80E-04	1.87E-04
**72h**	3.52E-05	2.83E-05	2.16E-04	1.40E-04
	cgd6_4480			
**2h**	0.00E+00	3.92E-08	0.00E+00	1.99E-08
**6h**	3.23E-08	3.61E-08	8.61E-08	7.73E-08
**12h**	1.72E-08	1.31E-07	3.95E-07	4.78E-08
**24h**	3.50E-08	4.18E-08	1.32E-07	2.01E-07
**36h**	5.95E-07	2.83E-07	5.51E-07	3.52E-07
**48h**	2.46E-07	1.26E-07	6.14E-07	4.28E-07
**72h**	1.59E-07	1.44E-07	2.53E-07	2.74E-07
	cgd6_4490			
**2h**	2.19E-06	5.43E-08	2.72E-07	5.43E-07
**6h**	1.06E-07	9.43E-08	4.25E-07	6.98E-07
**12h**	1.06E-07	1.23E-08	6.36E-07	3.32E-07
**24h**	3.57E-07	2.51E-07	1.07E-06	6.86E-07
**36h**	1.03E-06	5.61E-07	3.36E-06	6.21E-07
**48h**	9.26E-07	3.79E-07	2.92E-06	1.41E-06
**72h**	4.18E-06	1.80E-06	1.86E-05	3.01E-06
	cgd6_4500			
**2h**	1.77E-05	2.49E-07	2.18E-05	4.54E-06
**6h**	2.41E-05	4.29E-05	1.25E-03	6.60E-04
**12h**	4.32E-06	8.45E-06	1.36E-04	4.18E-05
**24h**	2.84E-05	1.54E-05	1.90E-03	2.83E-04
**36h**	4.65E-05	5.34E-05	1.43E-03	1.33E-04
**48h**	9.40E-05	4.57E-05	1.57E-03	3.25E-04
**72h**	2.46E-04	2.00E-04	1.86E-03	2.42E-03
	cgd6_4510			
**2h**	3.40E-07	6.33E-08	4.95E-08	5.57E-08
**6h**	6.69E-07	8.12E-07	9.18E-07	1.94E-06
**12h**	2.98E-08	2.40E-08	9.54E-07	2.60E-07
**24h**	2.23E-07	1.46E-07	9.09E-07	1.37E-06
**36h**	4.27E-07	5.03E-07	1.08E-06	8.24E-07
**48h**	7.56E-07	3.12E-07	1.02E-06	5.32E-07
**72h**	8.89E-07	6.42E-07	1.37E-06	1.69E-06
	cgd6_4530			
**2h**	0.00E+00	5.16E-08	0.00E+00	0.00E+00
**6h**	0.00E+00	5.78E-09	4.69E-06	1.58E-07
**12h**	3.32E-06	1.59E-05	3.79E-05	9.99E-06
**24h**	2.17E-06	2.15E-06	3.23E-05	1.43E-05
**36h**	2.62E-05	2.42E-05	3.74E-04	6.71E-05
**48h**	1.41E-05	9.27E-06	8.24E-05	3.33E-04
**72h**	2.64E-05	9.03E-06	5.62E-04	5.76E-05
	cgd6_5480			
**2h**	0.00E+00	0.00E+00	0.00E+00	0.00E+00
**6h**	7.80E-09	4.14E-09	9.89E-10	0.00E+00
**12h**	0.00E+00	0.00E+00	0.00E+00	0.00E+00
**24h**	9.58E-10	4.46E-10	7.22E-11	2.61E-09
**36h**	1.23E-09	2.84E-10	2.81E-09	1.32E-09
**48h**	1.86E-10	2.12E-09	4.64E-10	2.40E-09
**72h**	1.46E-09	1.17E-09	1.46E-09	0.00E+00
	cgd6_5450			
**2h**	1.98E-08	1.90E-07	2.87E-08	0.00E+00
**6h**	6.84E-07	4.20E-07	1.98E-07	1.25E-06

**12h**	3.07E-06	1.32E-06	5.09E-07	4.11E-07
**24h**	2.75E-06	3.20E-06	8.77E-07	5.49E-06
**36h**	1.09E-05	7.36E-06	3.11E-06	4.68E-06
**48h**	5.40E-06	1.94E-06	1.01E-06	5.97E-06
**72h**	1.12E-06	2.88E-07	2.94E-07	1.40E-06
	cgd6_5440			
**2h**	8.91E-07	3.54E-06	3.35E-06	4.44E-07
**6h**	9.72E-07	6.55E-07	7.13E-07	1.18E-06
**12h**	2.23E-05	6.47E-05	3.86E-05	3.42E-05
**24h**	1.35E-05	2.82E-05	3.59E-06	1.04E-05
**36h**	2.78E-05	3.70E-05	3.03E-05	2.05E-05
**48h**	7.92E-06	9.62E-06	2.33E-06	1.16E-05
**72h**	1.17E-06	9.88E-07	7.22E-07	6.08E-07
	cgd6_3070			
**2h**	5.49E-06	3.68E-06	5.27E-07	3.88E-06
**6h**	3.32E-06	6.27E-06	1.21E-06	3.91E-06
**12h**	2.55E-05	3.51E-06	4.82E-06	6.77E-06
**24h**	7.59E-06	4.57E-06	2.43E-06	2.45E-06
**36h**	7.11E-07	9.65E-07	1.38E-06	1.93E-06
**48h**	5.17E-05	6.52E-07	9.45E-07	4.52E-07
**72h**	5.11E-07	5.44E-07	3.28E-07	8.58E-07
	cgd6_3080			
**2h**	2.07E-04	6.12E-04	4.20E-06	2.11E-04
**6h**	2.66E-04	6.40E-04	1.71E-05	1.31E-04
**12h**	3.43E-02	6.81E-03	8.04E-04	1.76E-03
**24h**	3.20E-03	4.47E-03	1.62E-04	5.92E-04
**36h**	3.50E-03	8.85E-04	5.44E-04	1.23E-03
**48h**	2.29E-03	6.01E-04	7.28E-05	2.83E-04
**72h**	1.72E-03	5.14E-04	1.47E-04	1.37E-04
	cgd6_3090			
**2h**	1.39E-07	1.31E-08	3.11E-09	5.90E-09
**6h**	2.13E-08	7.95E-09	1.63E-08	5.54E-08
**12h**	4.98E-09	0.00E+00	6.10E-08	1.40E-08
**24h**	8.76E-08	6.20E-08	2.37E-07	1.29E-07
**36h**	1.35E-07	1.01E-07	1.45E-07	7.64E-08
**48h**	1.46E-06	4.29E-07	3.20E-07	4.11E-07
**72h**	6.67E-06	9.49E-07	1.22E-06	8.49E-07
	cgd6_3100			
**2h**	2.88E-04	5.40E-04	3.97E-05	2.49E-03
**6h**	6.95E-04	4.97E-04	1.81E-04	1.28E-03
**12h**	3.65E-04	7.91E-04	4.17E-04	7.63E-04
**24h**	2.27E-04	8.55E-04	3.21E-04	9.41E-04
**36h**	2.35E-04	1.61E-04	3.41E-04	4.21E-04
**48h**	6.67E-05	1.53E-04	7.21E-05	2.85E-04
**72h**	3.13E-04	2.23E-04	4.16E-04	2.20E-03
	cgd6_3120			
**2h**	0.00E+00	0.00E+00	0.00E+00	1.61E-08
**6h**	0.00E+00	0.00E+00	0.00E+00	0.00E+00
**12h**	3.70E-04	7.29E-05	5.89E-06	1.21E-05
**24h**	1.15E-05	5.30E-05	5.71E-06	9.63E-06
**36h**	6.47E-05	1.74E-04	5.81E-05	4.86E-05
**48h**	6.66E-04	8.40E-05	2.86E-05	2.23E-05
**72h**	1.92E-04	9.47E-05	2.29E-05	2.17E-05

	cgd6_3130			
**2h**	1.14E-06	2.35E-07	4.53E-07	2.14E-07
**6h**	6.91E-08	4.60E-08	0.00E+00	0.00E+00
**12h**	4.48E-02	3.63E-03	6.82E-04	1.71E-03
**24h**	1.10E-04	3.72E-04	1.72E-05	4.52E-05
**36h**	3.60E-05	4.44E-05	2.33E-04	1.84E-04
**48h**	4.74E-05	2.36E-05	7.88E-06	1.22E-05
**72h**	8.75E-06	1.47E-05	3.54E-06	4.40E-06
	cgd6_3140			
**2h**	9.91E-06	1.29E-06	1.44E-06	2.98E-06
**6h**	4.02E-06	4.60E-06	6.31E-07	3.11E-06
**12h**	3.81E-06	2.00E-06	2.54E-06	3.95E-06
**24h**	4.62E-06	3.29E-06	2.16E-06	7.24E-06
**36h**	8.27E-07	1.70E-06	2.26E-06	2.30E-06
**48h**	3.89E-06	1.67E-06	1.90E-06	2.81E-06
**72h**	8.14E-06	3.24E-06	4.06E-06	2.80E-06
	cgd6_3150			
**2h**	3.43E-06	3.77E-06	0.00E+00	1.40E-06
**6h**	1.61E-03	2.24E-04	3.37E-05	9.94E-05
**12h**	1.14E-03	1.79E-04	4.48E-05	5.69E-05
**24h**	3.42E-04	1.50E-04	2.61E-05	6.74E-05
**36h**	7.93E-05	1.45E-04	5.82E-05	5.67E-05
**48h**	1.06E-03	1.76E-04	4.78E-05	9.91E-05
**72h**	1.60E-03	1.60E-03	1.43E-04	2.73E-04
	cgd6_3160			
**2h**	7.89E-08	1.17E-08	0.00E+00	5.74E-08
**6h**	2.45E-08	7.95E-08	1.95E-07	2.43E-07
**12h**	7.80E-06	4.09E-06	1.79E-06	2.36E-06
**24h**	1.47E-06	1.93E-06	1.38E-06	2.23E-06
**36h**	2.08E-06	2.08E-06	3.62E-06	1.89E-06
**48h**	1.30E-05	3.46E-06	2.87E-06	4.04E-06
**72h**	1.62E-05	2.26E-06	3.27E-06	1.62E-06
	cgd6_3170			
**2h**	2.77E-04	8.13E-04	8.64E-06	2.58E-04
**6h**	8.83E-04	2.05E-03	1.89E-04	2.46E-03
**12h**	1.27E-04	1.13E-04	4.62E-05	1.18E-04
**24h**	9.92E-05	7.31E-04	7.48E-04	6.29E-04
**36h**	3.94E-04	2.94E-04	1.76E-04	3.63E-04
**48h**	4.57E-04	2.51E-04	5.71E-05	5.65E-04
**72h**	5.74E-04	2.32E-04	5.45E-05	6.28E-05
	cgd6_3180			
**2h**	5.76E-04	4.10E-04	3.37E-04	4.41E-04
**6h**	4.27E-03	3.60E-03	4.76E-04	2.49E-03
**12h**	6.36E-04	1.80E-04	5.62E-04	5.30E-04
**24h**	3.70E-04	9.94E-04	9.25E-04	8.65E-04
**36h**	2.73E-04	2.63E-04	2.58E-04	2.14E-04
**48h**	3.87E-04	8.94E-05	1.15E-04	1.77E-04
**72h**	1.28E-04	2.31E-05	1.84E-05	2.03E-05
	cgd6_3190			
**2h**	8.60E-04	3.46E-04	1.65E-04	6.59E-04
**6h**	1.22E-03	1.34E-03	2.84E-04	1.35E-03
**12h**	6.41E-04	2.54E-04	9.27E-04	4.14E-04
**24h**	2.83E-04	5.89E-04	4.65E-04	5.72E-04

**36h**	3.36E-04	4.29E-04	2.88E-04	2.77E-04
**48h**	2.98E-04	9.58E-05	4.53E-05	6.95E-05
**72h**	1.17E-04	5.23E-05	2.80E-05	3.40E-05
	cgd6_4540			
**2h**	0.00E+00	0.00E+00	0.00E+00	0.00E+00
**6h**	0.00E+00	0.00E+00	0.00E+00	0.00E+00
**12h**	6.11E-04	5.96E-05	3.53E-04	9.69E-05
**24h**	8.46E-05	5.69E-05	7.57E-05	2.68E-05
**36h**	1.33E-04	6.69E-05	3.13E-04	5.21E-05
**48h**	1.22E-04	2.57E-05	7.48E-05	3.31E-05
**72h**	5.81E-05	5.91E-06	1.63E-05	7.74E-06
	cgd6_4550			
**2h**	0.00E+00	0.00E+00	0.00E+00	0.00E+00
**6h**	3.89E-08	2.36E-08	4.00E-07	2.75E-07
**12h**	8.67E-07	3.88E-06	1.23E-06	7.64E-07
**24h**	8.92E-07	9.34E-07	1.65E-06	9.06E-07
**36h**	1.25E-06	1.78E-06	1.33E-06	1.01E-06
**48h**	7.16E-07	7.41E-07	1.58E-06	9.06E-07
**72h**	9.33E-07	4.05E-07	1.21E-06	1.02E-06
	cgd6_4570			
**2h**	2.35E-08	1.18E-08	3.87E-08	9.11E-08
**6h**	0.00E+00	3.47E-10	0.00E+00	1.00E-08
**12h**	4.09E-07	8.04E-07	3.05E-07	2.14E-07
**24h**	1.43E-07	2.15E-07	1.73E-07	1.21E-07
**36h**	3.38E-07	3.13E-07	8.20E-07	6.33E-07
**48h**	8.33E-07	7.56E-07	1.51E-06	1.06E-06
**72h**	5.46E-07	3.41E-07	3.99E-07	8.16E-07
	cgd6_4580			
**2h**	8.64E-04	7.33E-05	2.19E-04	3.61E-04
**6h**	1.79E-04	4.41E-05	2.73E-04	1.39E-04
**12h**	3.66E-04	6.81E-04	3.86E-04	1.92E-04
**24h**	1.64E-04	1.03E-04	4.20E-04	1.30E-04
**36h**	2.26E-04	1.01E-04	2.05E-04	1.56E-04
**48h**	2.64E-04	6.11E-05	2.37E-04	9.19E-05
**72h**	9.11E-05	4.99E-04	1.71E-04	8.82E-05
	cgd6_4590			
**2h**	2.89E-05	2.61E-07	1.22E-06	4.71E-06
**6h**	3.30E-06	3.51E-08	3.21E-07	2.41E-07
**12h**	1.24E-08	6.00E-08	7.88E-07	3.66E-07
**24h**	2.21E-06	8.78E-07	3.72E-06	4.76E-07
**36h**	7.51E-06	2.33E-06	2.69E-06	2.38E-06
**48h**	6.12E-05	9.98E-06	1.40E-05	6.71E-06
**72h**	3.37E-05	9.60E-06	4.50E-05	2.47E-05
	cgd6_4600			
**2h**	8.51E-08	3.91E-08	3.83E-08	6.62E-09
**6h**	8.11E-07	2.49E-07	9.02E-07	3.19E-07
**12h**	4.35E-08	1.01E-07	5.85E-08	1.25E-07
**24h**	2.54E-07	1.08E-07	4.69E-07	1.23E-07
**36h**	5.88E-07	2.18E-07	6.62E-07	5.66E-07
**48h**	4.04E-07	7.35E-08	1.68E-07	1.37E-07
**72h**	4.38E-07	1.90E-07	1.19E-06	2.69E-07
	cgd6_4610			
**2h**	0.00E+00	0.00E+00	0.00E+00	0.00E+00

**6h**	2.40E-05	1.96E-07	1.74E-07	5.57E-07
**12h**	6.19E-06	0.00E+00	6.37E-08	1.71E-07
**24h**	5.66E-06	3.92E-08	1.45E-06	4.72E-07
**36h**	2.13E-05	9.29E-07	4.88E-07	5.09E-07
**48h**	2.42E-05	1.30E-06	2.41E-06	6.27E-07
**72h**	5.86E-05	9.84E-07	4.26E-06	3.07E-06
	cgd6_4630			
**2h**	3.28E-06	2.93E-06	1.61E-06	4.89E-06
**6h**	7.02E-06	6.67E-06	1.69E-05	4.60E-05
**12h**	1.57E-06	5.09E-06	5.36E-06	1.07E-05
**24h**	3.66E-06	5.71E-06	1.55E-05	4.10E-06
**36h**	2.13E-06	5.44E-06	2.43E-06	4.40E-06
**48h**	1.65E-06	2.01E-06	2.01E-06	1.83E-06
**72h**	9.34E-07	1.08E-06	2.60E-06	1.96E-06
	cgd6_4650			
**2h**	3.64E-06	1.03E-06	4.16E-07	7.23E-06
**6h**	8.45E-07	1.52E-06	5.56E-06	1.37E-06
**12h**	1.79E-06	7.99E-07	2.61E-06	4.18E-06
**24h**	3.82E-06	1.99E-06	4.35E-06	4.21E-06
**36h**	1.56E-06	9.30E-07	2.18E-06	2.18E-06
**48h**	2.04E-06	1.50E-06	2.52E-06	2.47E-06
**72h**	2.98E-06	1.39E-06	2.73E-06	1.55E-06
	cgd6_4660			
**2h**	3.65E-07	1.22E-07	6.39E-09	5.56E-07
**6h**	1.29E-08	4.34E-09	4.17E-09	1.65E-08
**12h**	1.23E-05	2.51E-06	7.59E-06	5.57E-06
**24h**	3.13E-06	2.57E-06	6.02E-07	4.34E-07
**36h**	6.53E-07	4.70E-07	1.93E-06	1.24E-06
**48h**	2.19E-06	4.89E-07	8.73E-07	9.70E-07
**72h**	8.63E-07	1.33E-07	4.91E-07	3.39E-07
	cgd6_4670			
**2h**	3.86E-07	4.78E-07	1.19E-08	6.93E-07
**6h**	9.42E-08	1.14E-07	2.39E-07	2.69E-07
**12h**	4.18E-08	1.22E-07	2.49E-07	1.81E-07
**24h**	9.94E-08	3.97E-08	5.03E-07	3.12E-07
**36h**	1.98E-07	1.18E-07	2.40E-07	1.59E-07
**48h**	8.33E-08	6.19E-08	1.80E-05	2.07E-07
**72h**	6.89E-06	8.30E-08	2.04E-07	1.43E-07
	cgd6_3210			
**2h**	2.08E-04	7.34E-06	6.33E-06	5.04E-05
**6h**	2.39E-06	5.35E-06	2.04E-06	3.85E-06
**12h**	2.34E-06	1.68E-05	6.19E-06	1.57E-05
**24h**	2.96E-05	4.91E-05	7.10E-06	1.37E-06
**36h**	7.27E-06	1.42E-05	3.79E-06	8.88E-06
**48h**	5.94E-06	1.81E-06	4.23E-06	1.07E-05
**72h**	3.11E-06	5.43E-06	1.39E-06	8.09E-06
	cgd6_3220			
**2h**	4.61E-08	0.00E+00	1.10E-08	3.10E-08
**6h**	3.29E-06	7.73E-06	2.09E-06	3.25E-06
**12h**	7.83E-07	3.57E-06	5.46E-07	1.51E-06
**24h**	1.15E-06	3.27E-06	1.19E-06	1.46E-07
**36h**	5.52E-07	1.10E-06	6.63E-07	1.12E-06
**48h**	4.50E-07	5.31E-07	2.65E-07	8.44E-07

**72h**	6.54E-08	3.02E-07	6.54E-08	4.71E-07
	cgd6_3230			
**2h**	1.23E-05	1.00E-05	6.80E-06	2.24E-05
**6h**	6.03E-06	1.40E-05	2.38E-06	4.86E-06
**12h**	4.92E-06	2.11E-05	1.43E-05	1.83E-05
**24h**	2.50E-05	5.75E-05	1.16E-05	1.01E-06
**36h**	2.34E-06	1.21E-05	5.54E-06	6.93E-06
**48h**	2.01E-06	1.70E-06	1.38E-06	3.22E-06
**72h**	5.43E-07	2.11E-06	9.71E-07	3.16E-06
	cgd6_3240			
**2h**	0.00E+00	5.27E-07	0.00E+00	0.00E+00
**6h**	3.77E-05	7.83E-05	5.53E-06	2.67E-05
**12h**	1.64E-07	2.98E-07	2.24E-07	5.84E-07
**24h**	6.31E-06	2.89E-05	9.54E-06	1.83E-06
**36h**	1.16E-05	2.89E-05	2.56E-06	6.85E-06
**48h**	1.47E-05	9.50E-06	6.04E-07	1.02E-05
**72h**	2.21E-06	7.65E-06	6.44E-07	6.31E-06
	cgd6_3250			
**2h**	2.04E-09	0.00E+00	0.00E+00	0.00E+00
**6h**	3.18E-07	1.09E-06	2.02E-07	4.33E-07
**12h**	1.04E-08	2.78E-07	3.36E-08	2.77E-08
**24h**	3.77E-08	1.36E-07	4.72E-07	3.98E-08
**36h**	1.69E-07	6.95E-07	2.97E-07	5.86E-07
**48h**	1.34E-07	7.29E-08	1.04E-07	1.46E-07
**72h**	4.87E-08	1.51E-07	4.11E-08	1.66E-07
	cgd6_3260			
**2h**	4.71E-09	1.64E-09	0.00E+00	0.00E+00
**6h**	9.77E-09	1.75E-07	3.06E-08	5.21E-09
**12h**	9.73E-09	4.81E-08	4.06E-08	2.32E-08
**24h**	5.27E-08	7.51E-08	2.01E-07	1.11E-08
**36h**	3.91E-07	6.95E-07	2.25E-07	4.79E-07
**48h**	2.38E-07	2.46E-07	0.00E+00	1.50E-07
**72h**	8.75E-08	1.21E-07	6.52E-08	7.31E-07
	cgd6_3270			
**2h**	3.35E-07	0.00E+00	0.00E+00	8.44E-07
**6h**	7.29E-06	5.06E-05	2.75E-06	7.19E-06
**12h**	5.81E-07	1.69E-06	1.89E-06	6.20E-07
**24h**	1.07E-05	2.31E-05	7.13E-06	1.20E-06
**36h**	1.52E-05	3.30E-05	4.40E-06	2.03E-05
**48h**	9.06E-05	2.06E-05	4.28E-06	2.73E-05
**72h**	2.13E-04	1.25E-04	4.99E-05	2.72E-04
	cgd6_3280			
**2h**	5.98E-06	1.13E-06	3.52E-06	5.65E-06
**6h**	4.60E-05	2.63E-04	3.93E-05	1.55E-04
**12h**	8.10E-05	1.09E-04	9.35E-06	4.22E-05
**24h**	8.26E-05	1.51E-04	4.41E-05	1.05E-05
**36h**	4.20E-04	5.56E-04	1.24E-04	3.73E-04
**48h**	1.03E-03	2.56E-03	4.73E-04	3.24E-03
**72h**	8.68E-04	7.38E-03	5.88E-04	3.19E-03
	cgd6_3290			
**2h**	5.43E-04	7.63E-05	2.04E-05	2.47E-04
**6h**	1.23E-04	3.22E-04	7.31E-06	1.48E-04
**12h**	2.83E-05	5.73E-05	2.85E-05	9.43E-05

**24h**	3.73E-04	6.09E-04	4.01E-05	1.35E-05
**36h**	7.66E-05	1.29E-04	2.82E-05	7.49E-05
**48h**	5.42E-05	2.02E-05	1.00E-05	7.05E-05
**72h**	3.04E-05	5.82E-05	8.05E-06	3.17E-05
	cgd6_3300			
**2h**	4.44E-06	3.85E-06	5.65E-07	5.06E-06
**6h**	4.97E-06	2.02E-05	5.70E-07	4.29E-06
**12h**	3.66E-07	4.97E-07	3.39E-07	1.86E-06
**24h**	1.95E-06	3.98E-06	7.97E-07	2.90E-07
**36h**	1.07E-06	4.16E-06	3.46E-07	1.64E-06
**48h**	8.49E-07	7.51E-07	3.53E-07	1.22E-06
**72h**	2.89E-07	8.68E-07	5.63E-08	3.29E-07
	cgd6_3310			
**2h**	5.83E-06	5.18E-06	1.09E-07	6.29E-06
**6h**	3.08E-06	8.44E-06	6.49E-07	1.17E-05
**12h**	8.43E-08	1.47E-06	3.55E-08	2.50E-07
**24h**	3.45E-07	8.99E-07	6.07E-07	8.72E-08
**36h**	1.22E-06	2.42E-06	3.59E-07	1.19E-06
**48h**	3.60E-07	4.90E-07	1.51E-07	9.04E-07
**72h**	9.94E-08	8.51E-07	1.28E-07	4.97E-07
	cgd6_3330			
**2h**	7.19E-05	7.05E-05	0.00E+00	7.61E-04
**6h**	6.87E-05	4.23E-04	3.97E-06	1.26E-04
**12h**	1.80E-05	0.00E+00	4.85E-06	8.81E-05
**24h**	3.86E-05	5.33E-05	5.26E-05	3.10E-05
**36h**	4.91E-05	1.19E-04	8.75E-05	3.01E-05
**48h**	3.23E-05	2.74E-04	1.50E-04	7.96E-05
**72h**	1.24E-05	1.42E-04	4.09E-05	6.44E-04
	cgd6_3350			
**2h**	0.00E+00	0.00E+00	0.00E+00	0.00E+00
**6h**	0.00E+00	1.09E-08	2.73E-08	3.24E-09
**12h**	0.00E+00	0.00E+00	1.44E-08	0.00E+00
**24h**	0.00E+00	4.09E-09	9.38E-09	1.59E-08
**36h**	1.19E-08	2.25E-08	3.18E-09	1.80E-08
**48h**	2.19E-07	8.61E-08	9.14E-08	5.24E-08
**72h**	1.24E-08	5.49E-09	1.88E-08	9.78E-09
	cgd6_3370			
**2h**	7.05E-05	2.56E-05	1.37E-05	1.22E-05
**6h**	1.52E-05	2.01E-05	8.31E-06	1.69E-05
**12h**	8.83E-06	3.38E-06	2.45E-05	8.64E-06
**24h**	5.44E-05	4.61E-05	1.50E-05	8.94E-06
**36h**	1.02E-04	8.11E-06	5.16E-06	2.31E-06
**48h**	1.81E-05	2.18E-06	7.94E-06	2.27E-06
**72h**	5.40E-06	1.97E-06	3.08E-06	1.33E-06
	cgd6_3380			
**2h**	1.44E-03	6.38E-04	1.82E-04	2.48E-04
**6h**	6.38E-04	5.32E-04	4.57E-04	4.47E-04
**12h**	2.19E-04	1.37E-04	5.53E-04	1.07E-04
**24h**	5.79E-04	3.85E-04	5.07E-04	2.55E-04
**36h**	2.68E-04	1.85E-04	1.55E-04	3.48E-05
**48h**	1.45E-04	3.85E-05	1.07E-04	3.68E-05
**72h**	9.05E-05	2.17E-05	3.20E-05	2.35E-05
	cgd6_3390			

**2h**	1.69E-07	2.15E-08	0.00E+00	3.04E-09
**6h**	0.00E+00	0.00E+00	2.25E-08	3.09E-09
**12h**	2.68E-07	1.13E-07	2.10E-06	6.84E-07
**24h**	1.33E-06	2.57E-07	7.70E-07	2.00E-07
**36h**	7.99E-07	1.45E-06	3.13E-06	1.40E-06
**48h**	2.74E-06	1.04E-06	1.75E-06	1.11E-06
**72h**	5.47E-06	1.19E-06	6.25E-06	3.65E-06
	cgd6_3400			
**2h**	7.62E-07	5.29E-07	2.25E-07	4.08E-07
**6h**	4.69E-07	8.27E-07	4.15E-06	7.25E-07
**12h**	1.32E-07	9.97E-07	8.31E-05	6.07E-07
**24h**	4.04E-07	8.52E-07	1.24E-06	6.98E-07
**36h**	3.26E-07	2.74E-06	2.44E-06	1.16E-06
**48h**	1.89E-06	6.14E-07	1.57E-06	1.00E-06
**72h**	1.79E-06	9.46E-07	2.85E-06	2.03E-06
	cgd6_3410			
**2h**	2.36E-08	3.89E-08	0.00E+00	8.64E-09
**6h**	1.13E-08	4.44E-08	2.13E-07	3.03E-07
**12h**	2.73E-08	9.76E-08	1.22E-07	6.32E-08
**24h**	7.66E-08	5.70E-08	1.16E-07	1.54E-07
**36h**	8.14E-08	3.71E-07	3.04E-07	1.74E-07
**48h**	1.18E-07	1.37E-07	9.37E-08	9.49E-08
**72h**	7.31E-08	3.88E-08	1.55E-07	5.88E-08
	cgd6_3420			
**2h**	9.66E-07	1.00E-06	8.30E-08	8.47E-08
**6h**	9.63E-06	1.17E-06	2.71E-06	1.70E-06
**12h**	7.95E-09	6.77E-07	2.53E-07	5.94E-08
**24h**	1.38E-07	4.94E-07	6.23E-07	6.81E-07
**36h**	2.44E-07	7.18E-07	3.34E-07	2.46E-07
**48h**	2.33E-07	2.87E-07	2.39E-07	1.20E-07
**72h**	1.40E-07	1.04E-07	1.07E-07	8.47E-08
	cgd6_3510			
**2h**	1.36E-08	3.13E-08	1.86E-08	3.85E-08
**6h**	3.64E-09	3.44E-08	1.73E-07	6.66E-07
**12h**	2.68E-05	8.01E-05	2.37E-05	7.66E-06
**24h**	1.56E-06	2.46E-06	9.34E-07	5.41E-07
**36h**	7.17E-07	1.22E-06	5.73E-06	1.59E-06
**48h**	8.31E-07	3.45E-07	3.11E-07	1.41E-07
**72h**	2.62E-07	1.07E-07	2.25E-07	1.38E-07
	cgd6_3520			
**2h**	3.04E-05	4.86E-06	2.30E-06	3.95E-06
**6h**	5.83E-06	6.56E-06	1.16E-05	9.59E-06
**12h**	7.17E-06	6.77E-06	3.35E-05	8.64E-06
**24h**	5.24E-06	7.65E-06	1.29E-05	7.07E-06
**36h**	4.14E-06	8.39E-06	1.50E-05	3.68E-06
**48h**	2.94E-06	2.53E-06	4.49E-06	1.91E-06
**72h**	1.09E-06	3.71E-07	2.05E-06	8.95E-07
	cgd6_3550			
**2h**	1.15E-04	7.20E-04	4.39E-03	4.77E-03
**6h**	1.04E-04	1.93E-03	1.04E-03	1.54E-03
**12h**	2.37E-05	9.35E-04	1.40E-03	5.51E-04
**24h**	5.23E-05	3.58E-04	7.50E-04	1.70E-04
**36h**	1.96E-05	6.22E-04	8.55E-04	1.16E-03

**48h**	3.83E-05	9.17E-05	1.07E-03	8.47E-04
**72h**	5.85E-05	3.44E-04	1.53E-03	1.05E-04
	cgd6_3560			
**2h**	1.02E-03	1.24E-03	1.09E-03	4.04E-04
**6h**	1.01E-04	6.34E-04	6.29E-04	2.94E-04
**12h**	8.31E-05	1.92E-04	1.10E-03	2.28E-04
**24h**	3.35E-04	4.82E-04	3.47E-04	1.71E-04
**36h**	8.01E-05	1.09E-03	2.78E-04	7.43E-05
**48h**	6.39E-05	1.79E-04	2.02E-04	7.83E-05
**72h**	1.44E-04	9.49E-05	3.81E-04	9.13E-05
	cgd6_3570			
**2h**	1.02E-05	6.02E-06	5.49E-06	6.25E-06
**6h**	2.74E-07	1.12E-06	3.39E-07	2.80E-07
**12h**	2.89E-05	4.79E-07	2.15E-05	1.09E-06
**24h**	1.01E-06	1.39E-06	1.81E-06	1.15E-06
**36h**	2.62E-06	1.84E-06	1.97E-06	5.35E-07
**48h**	1.91E-06	6.00E-07	2.02E-06	5.66E-07
**72h**	2.18E-04	8.44E-07	6.73E-06	1.86E-05
	cgd6_5490			
**2h**	4.77E-08	0.00E+00	0.00E+00	0.00E+00
**6h**	2.17E-05	1.70E-06	4.74E-07	2.62E-08
**12h**	2.37E-06	4.63E-06	6.10E-08	1.59E-07
**24h**	2.76E-06	6.78E-07	2.16E-07	7.61E-07
**36h**	3.18E-06	1.25E-06	2.57E-07	1.41E-06
**48h**	4.39E-06	2.19E-06	1.36E-07	9.77E-07
**72h**	4.46E-07	1.08E-08	2.48E-08	1.08E-08
	cgd6_5460			
**2h**	0.00E+00	0.00E+00	0.00E+00	0.00E+00
**6h**	0.00E+00	1.49E-08	2.33E-10	1.18E-08
**12h**	1.15E-04	3.50E-05	1.06E-05	1.57E-05
**24h**	2.66E-05	7.10E-06	4.52E-07	3.34E-06
**36h**	1.62E-05	2.47E-06	1.92E-06	7.91E-06
**48h**	7.48E-07	4.35E-07	4.80E-08	1.26E-07
**72h**	4.90E-08	3.92E-08	9.34E-10	0.00E+00
	cgd7_80			
**2h**	7.59E-09	7.96E-07	1.25E-07	1.11E-06
**6h**	1.27E-06	8.64E-08	5.44E-06	3.26E-06
**12h**	5.92E-07	1.44E-06	3.53E-06	1.27E-08
**24h**	2.93E-06	3.42E-06	3.10E-06	1.07E-06
**36h**	5.99E-07	1.81E-06	1.35E-06	1.48E-06
**48h**	4.49E-06	7.95E-06	2.34E-06	3.93E-06
**72h**	1.59E-06	2.51E-06	8.32E-07	1.95E-06
	cgd6_3590			
**2h**	0.00E+00	0.00E+00	0.00E+00	0.00E+00
**6h**	1.99E-06	1.78E-06	6.26E-06	2.34E-04
**12h**	4.23E-08	2.40E-07	5.80E-08	6.15E-07
**24h**	9.54E-07	6.09E-07	2.22E-06	1.97E-05
**36h**	4.36E-06	3.55E-06	1.58E-06	9.95E-06
**48h**	5.24E-06	2.13E-06	2.00E-06	1.76E-05
**72h**	5.52E-06	2.78E-06	4.04E-06	5.03E-06
	cgd6_3610			
**2h**	2.41E-06	4.65E-06	7.38E-07	6.35E-06
**6h**	3.05E-06	1.60E-06	8.86E-07	3.23E-06

**12h**	8.20E-07	9.90E-07	1.98E-06	5.52E-06
**24h**	8.11E-06	3.61E-06	1.65E-06	5.79E-06
**36h**	1.04E-06	1.07E-06	1.15E-06	2.36E-06
**48h**	2.53E-06	8.18E-07	1.21E-06	2.94E-06
**72h**	2.63E-06	2.16E-06	3.91E-06	9.45E-06
	cgd6_3620			
**2h**	9.19E-06	8.67E-06	6.62E-06	2.21E-05
**6h**	3.42E-06	4.96E-06	3.35E-06	7.78E-06
**12h**	6.56E-06	4.41E-06	9.38E-06	2.48E-05
**24h**	1.05E-05	8.09E-06	7.14E-06	1.46E-05
**36h**	5.11E-06	4.16E-06	1.06E-05	1.51E-05
**48h**	6.93E-06	3.83E-06	2.22E-06	7.48E-06
**72h**	4.55E-06	3.65E-06	8.77E-06	1.64E-05
	cgd6_3640			
**2h**	0.00E+00	0.00E+00	0.00E+00	0.00E+00
**6h**	0.00E+00	3.32E-08	4.93E-08	6.51E-07
**12h**	5.69E-07	1.04E-06	6.96E-07	6.54E-06
**24h**	6.95E-07	1.03E-06	1.40E-06	5.52E-05
**36h**	2.17E-06	2.04E-06	3.80E-06	1.45E-05
**48h**	2.73E-06	6.12E-07	5.46E-07	3.07E-06
**72h**	1.24E-06	1.03E-06	2.16E-06	3.17E-06
	cgd6_3650			
**2h**	5.91E-07	0.00E+00	0.00E+00	8.50E-08
**6h**	2.55E-06	2.84E-06	1.95E-06	1.31E-05
**12h**	8.74E-07	5.55E-06	3.80E-06	1.78E-05
**24h**	1.04E-05	1.07E-05	2.24E-05	4.90E-05
**36h**	3.02E-05	2.39E-05	3.18E-05	1.23E-04
**48h**	1.57E-05	9.38E-06	4.87E-06	6.18E-05
**72h**	3.54E-05	1.78E-05	7.28E-05	9.55E-05
	cgd6_3660			
**2h**	1.70E-07	1.83E-07	0.00E+00	0.00E+00
**6h**	1.61E-06	2.56E-07	4.90E-07	8.25E-07
**12h**	4.04E-06	5.54E-06	2.96E-06	2.35E-06
**24h**	1.00E-06	7.23E-07	6.32E-07	1.86E-06
**36h**	2.19E-06	2.81E-06	3.58E-06	3.12E-06
**48h**	1.15E-06	5.47E-07	5.70E-07	1.67E-06
**72h**	1.51E-06	9.70E-07	1.28E-06	1.71E-06
	cgd6_3680			
**2h**	0.00E+00	0.00E+00	0.00E+00	0.00E+00
**6h**	0.00E+00	0.00E+00	1.68E-08	0.00E+00
**12h**	3.88E-05	6.84E-05	3.12E-05	1.05E-04
**24h**	1.26E-05	9.43E-06	6.67E-06	3.95E-05
**36h**	1.35E-05	1.13E-05	1.97E-05	5.02E-05
**48h**	7.30E-06	2.89E-06	4.78E-06	4.57E-05
**72h**	2.41E-06	2.67E-06	6.66E-06	1.14E-05
	cgd6_3690			
**2h**	4.36E-08	3.47E-08	7.69E-08	0.00E+00
**6h**	4.42E-07	1.33E-06	5.47E-07	1.51E-05
**12h**	1.79E-07	1.14E-07	5.59E-07	4.78E-06
**24h**	7.50E-07	8.27E-07	5.89E-07	1.18E-05
**36h**	1.67E-06	2.66E-06	5.22E-06	2.04E-05
**48h**	5.01E-06	4.68E-07	1.03E-06	1.12E-05
**72h**	7.10E-07	9.53E-07	2.61E-06	3.92E-05

	cgd6_3710			
**2h**	5.89E-04	7.71E-04	2.13E-03	1.42E-03
**6h**	2.50E-03	1.10E-03	2.40E-03	4.10E-03
**12h**	6.42E-04	4.06E-04	1.90E-03	2.56E-03
**24h**	1.29E-03	8.90E-04	1.30E-03	5.44E-03
**36h**	6.95E-04	3.22E-04	7.07E-04	1.04E-03
**48h**	4.51E-04	2.31E-04	2.27E-04	9.33E-04
**72h**	4.19E-05	2.20E-05	7.34E-05	5.05E-04
	cgd6_3720			
**2h**	4.23E-05	1.02E-04	0.00E+00	1.28E-04
**6h**	1.13E-04	1.24E-04	8.43E-06	4.72E-06
**12h**	1.18E-05	2.94E-05	5.20E-05	9.56E-04
**24h**	8.42E-05	8.07E-06	1.68E-04	8.84E-04
**36h**	5.81E-05	4.37E-05	1.05E-04	4.46E-04
**48h**	3.33E-04	1.07E-04	9.22E-05	3.34E-04
**72h**	3.61E-05	4.75E-05	2.00E-04	1.21E-03
	cgd6_3730			
**2h**	4.12E-08	4.34E-08	1.10E-07	1.99E-08
**6h**	2.62E-08	1.30E-08	7.52E-09	4.85E-09
**12h**	1.87E-08	6.62E-09	1.06E-08	2.55E-08
**24h**	9.86E-08	2.46E-08	5.07E-08	2.18E-08
**36h**	1.67E-07	1.86E-07	1.18E-07	1.48E-07
**48h**	6.81E-06	2.34E-06	1.44E-06	2.10E-06
**72h**	2.95E-06	2.03E-06	4.34E-06	3.33E-06
	cgd6_3750			
**2h**	1.07E-06	0.00E+00	7.53E-07	1.12E-04
**6h**	0.00E+00	3.50E-08	0.00E+00	4.21E-06
**12h**	2.85E-07	1.66E-07	1.31E-06	8.47E-06
**24h**	7.96E-06	3.72E-06	2.65E-06	1.31E-04
**36h**	2.01E-05	1.02E-05	7.00E-05	2.60E-04
**48h**	7.61E-05	1.48E-05	4.45E-04	1.66E-03
**72h**	2.37E-04	3.57E-05	2.53E-03	6.34E-04
	cgd6_4680			
**2h**	1.18E-05	1.38E-06	7.41E-08	5.61E-07
**6h**	1.40E-06	5.65E-07	6.54E-07	1.21E-06
**12h**	8.09E-07	4.46E-06	2.19E-07	1.61E-07
**24h**	5.23E-07	5.67E-07	1.01E-06	2.08E-06
**36h**	3.14E-07	2.69E-07	6.95E-07	7.99E-07
**48h**	4.79E-07	4.39E-07	5.48E-07	8.32E-07
**72h**	1.20E-06	9.71E-08	6.20E-07	1.01E-06
	cgd6_4700			
**2h**	1.91E-07	3.35E-07	7.52E-09	4.23E-08
**6h**	2.98E-08	0.00E+00	0.00E+00	2.43E-09
**12h**	4.96E-07	4.20E-07	6.35E-07	4.20E-07
**24h**	4.18E-07	5.61E-07	1.51E-07	1.59E-07
**36h**	5.21E-07	5.05E-07	1.17E-06	1.08E-06
**48h**	3.28E-07	3.03E-07	3.51E-07	3.07E-07
**72h**	1.23E-06	5.28E-07	4.48E-07	1.68E-06
	cgd6_4710			
**2h**	3.96E-04	7.44E-05	1.77E-05	1.89E-04
**6h**	1.90E-05	1.59E-05	4.67E-06	1.82E-05
**12h**	1.46E-04	5.43E-06	6.16E-05	9.70E-05
**24h**	1.86E-04	1.08E-04	7.38E-05	5.74E-05

**36h**	2.95E-05	1.64E-05	4.77E-05	5.14E-05
**48h**	2.36E-05	5.43E-06	1.22E-05	1.98E-05
**72h**	1.17E-05	1.73E-06	6.72E-06	1.38E-05
	cgd6_4720			
**2h**	2.19E-06	2.64E-06	3.60E-07	5.52E-07
**6h**	3.95E-07	8.46E-07	3.21E-07	5.93E-07
**12h**	6.00E-07	3.98E-07	1.16E-06	5.84E-07
**24h**	4.10E-07	5.41E-07	3.58E-07	5.60E-07
**36h**	5.90E-07	3.14E-07	3.71E-07	3.90E-07
**48h**	1.75E-07	1.53E-07	2.01E-07	1.20E-07
**72h**	1.34E-07	7.79E-08	1.33E-07	1.38E-07
	cgd6_4730			
**2h**	0.00E+00	0.00E+00	0.00E+00	0.00E+00
**6h**	0.00E+00	0.00E+00	0.00E+00	5.61E-09
**12h**	1.30E-06	1.03E-07	3.86E-07	5.35E-07
**24h**	1.50E-07	3.34E-08	2.02E-07	1.63E-07
**36h**	1.17E-06	6.52E-07	1.49E-06	1.04E-06
**48h**	1.46E-07	8.78E-08	1.88E-07	1.25E-07
**72h**	6.04E-07	8.81E-08	5.59E-07	5.33E-07
	cgd6_4740			
**2h**	4.70E-04	1.80E-05	2.56E-05	1.97E-04
**6h**	1.28E-04	9.59E-06	1.69E-05	9.62E-05
**12h**	3.45E-04	4.05E-05	6.81E-05	3.17E-04
**24h**	2.22E-04	8.16E-05	5.88E-05	1.68E-04
**36h**	2.85E-04	4.24E-05	1.12E-04	1.57E-04
**48h**	1.45E-04	1.42E-05	6.73E-05	5.83E-05
**72h**	5.98E-05	1.95E-05	3.51E-05	4.58E-05
	cgd6_4750			
**2h**	2.46E-07	1.15E-07	1.15E-08	2.52E-08
**6h**	5.46E-07	1.78E-06	7.65E-07	3.22E-06
**12h**	7.56E-08	2.18E-08	9.97E-08	1.24E-07
**24h**	2.82E-07	1.42E-07	2.64E-06	1.34E-06
**36h**	1.54E-06	3.30E-07	3.58E-07	6.40E-07
**48h**	4.27E-07	3.69E-07	3.67E-07	3.50E-07
**72h**	2.84E-07	9.03E-08	1.73E-07	8.94E-07
	cgd6_4760			
**2h**	0.00E+00	0.00E+00	0.00E+00	7.98E-10
**6h**	5.27E-07	8.87E-07	2.18E-06	4.18E-07
**12h**	1.35E-05	3.17E-06	4.69E-06	3.25E-06
**24h**	1.93E-06	1.68E-06	8.42E-06	3.08E-06
**36h**	1.14E-05	4.06E-06	1.34E-05	1.07E-05
**48h**	1.57E-05	1.05E-05	1.72E-05	2.75E-06
**72h**	3.50E-06	1.30E-06	4.89E-06	6.47E-06
	cgd6_4770			
**2h**	9.33E-06	3.94E-06	4.52E-06	1.43E-06
**6h**	3.51E-06	2.86E-06	1.96E-06	6.16E-06
**12h**	1.41E-06	4.48E-07	3.50E-06	2.90E-06
**24h**	3.64E-06	2.34E-06	6.74E-06	5.36E-06
**36h**	2.85E-06	4.77E-06	3.03E-06	1.54E-06
**48h**	2.64E-06	6.63E-07	1.73E-06	1.60E-06
**72h**	6.29E-07	6.94E-07	6.73E-07	9.51E-07
	cgd6_4780			
**2h**	9.84E-04	9.94E-04	6.29E-05	2.85E-04

**6h**	9.80E-04	4.58E-05	1.50E-04	5.77E-04
**12h**	3.25E-04	2.15E-05	1.49E-04	5.01E-04
**24h**	6.11E-04	7.39E-05	3.37E-04	6.51E-04
**36h**	3.50E-04	8.45E-05	1.63E-04	2.87E-04
**48h**	1.70E-04	7.75E-05	2.40E-04	3.51E-04
**72h**	7.32E-05	7.30E-06	2.29E-05	1.44E-04
	cgd6_4790			
**2h**	0.00E+00	2.06E-08	0.00E+00	1.04E-08
**6h**	0.00E+00	0.00E+00	0.00E+00	0.00E+00
**12h**	8.42E-06	6.04E-06	2.61E-06	3.56E-06
**24h**	2.43E-06	2.56E-06	1.55E-06	1.08E-06
**36h**	3.22E-06	6.72E-06	4.58E-06	5.30E-06
**48h**	4.93E-06	1.56E-06	2.58E-06	2.57E-06
**72h**	2.86E-06	6.37E-06	6.27E-06	1.79E-05
	cgd6_4800			
**2h**	0.00E+00	3.19E-08	0.00E+00	0.00E+00
**6h**	0.00E+00	0.00E+00	8.60E-09	0.00E+00
**12h**	0.00E+00	0.00E+00	3.23E-08	1.30E-09
**24h**	8.04E-08	1.17E-08	7.15E-08	1.65E-07
**36h**	2.06E-07	2.39E-07	4.51E-07	2.46E-07
**48h**	2.36E-07	1.19E-07	5.04E-08	1.08E-07
**72h**	1.27E-06	1.38E-06	2.94E-06	3.46E-06
	cgd6_4810			
**2h**	0.00E+00	0.00E+00	0.00E+00	0.00E+00
**6h**	0.00E+00	0.00E+00	0.00E+00	0.00E+00
**12h**	3.57E-07	0.00E+00	4.44E-08	1.35E-06
**24h**	4.48E-07	2.94E-06	1.01E-07	1.19E-06
**36h**	1.87E-06	6.75E-06	2.93E-06	3.11E-06
**48h**	6.83E-06	2.65E-05	7.99E-06	2.82E-06
**72h**	7.31E-06	1.97E-05	6.82E-05	8.94E-06
	cgd6_4840			
**2h**	5.06E-10	0.00E+00	0.00E+00	0.00E+00
**6h**	0.00E+00	1.98E-09	0.00E+00	0.00E+00
**12h**	0.00E+00	0.00E+00	0.00E+00	0.00E+00
**24h**	4.37E-10	6.77E-10	2.92E-10	5.07E-09
**36h**	6.32E-09	2.14E-09	5.62E-10	5.60E-09
**48h**	3.99E-07	3.67E-07	3.14E-07	1.34E-06
**72h**	1.21E-06	4.42E-07	1.68E-06	3.34E-06
	cgd6_4850			
**2h**	4.26E-07	3.38E-07	9.89E-08	0.00E+00
**6h**	2.21E-06	2.48E-06	1.59E-06	4.97E-07
**12h**	1.24E-08	3.31E-08	2.62E-08	1.36E-07
**24h**	1.71E-07	3.62E-07	4.83E-07	4.78E-07
**36h**	7.22E-07	4.74E-07	2.47E-07	1.37E-07
**48h**	1.68E-07	1.81E-07	1.06E-07	1.19E-07
**72h**	6.73E-07	8.61E-08	1.26E-07	6.63E-08
	cgd6_4870			
**2h**	7.33E-09	3.71E-07	3.12E-07	4.36E-06
**6h**	0.00E+00	6.70E-08	3.57E-09	3.68E-08
**12h**	2.11E-06	9.53E-06	1.42E-06	3.94E-05
**24h**	7.73E-07	3.72E-06	6.74E-06	1.03E-05
**36h**	2.10E-06	3.94E-06	7.02E-06	4.10E-05
**48h**	3.53E-06	5.86E-06	7.91E-06	1.13E-04

**72h**	8.52E-06	8.01E-06	8.61E-06	1.21E-04
	cgd6_4880			
**2h**	0.00E+00	1.31E-08	2.44E-08	0.00E+00
**6h**	4.98E-09	0.00E+00	0.00E+00	0.00E+00
**12h**	0.00E+00	0.00E+00	0.00E+00	0.00E+00
**24h**	1.54E-08	4.43E-09	3.52E-09	1.29E-06
**36h**	7.67E-08	3.45E-07	6.73E-08	6.30E-06
**48h**	7.91E-06	1.07E-05	1.62E-05	3.47E-05
**72h**	2.07E-05	1.25E-05	2.17E-05	1.41E-04
	cgd6_4900			
**2h**	2.97E-07	1.41E-06	9.89E-07	7.51E-06
**6h**	8.82E-07	6.73E-06	2.55E-06	7.68E-06
**12h**	7.95E-07	1.98E-06	5.22E-07	9.78E-06
**24h**	5.31E-07	1.40E-06	1.91E-06	1.63E-05
**36h**	9.57E-07	1.16E-06	7.26E-07	3.87E-06
**48h**	3.05E-07	4.97E-07	5.13E-07	1.59E-05
**72h**	6.45E-07	4.17E-07	5.75E-07	6.82E-06
	cgd6_4910			
**2h**	6.22E-05	3.35E-05	3.14E-05	4.86E-05
**6h**	1.08E-05	3.52E-05	1.85E-05	3.26E-03
**12h**	3.29E-05	9.76E-05	1.65E-05	7.60E-05
**24h**	5.19E-05	3.65E-05	1.78E-05	3.03E-05
**36h**	1.12E-05	2.10E-05	1.05E-05	1.72E-05
**48h**	9.57E-06	1.80E-05	7.82E-06	1.35E-05
**72h**	4.89E-06	2.44E-06	3.63E-06	1.31E-05
	cgd6_4920			
**2h**	6.45E-04	4.11E-05	1.46E-02	7.58E-03
**6h**	1.13E-03	5.55E-05	3.23E-03	7.59E-01
**12h**	3.77E-03	2.52E-05	3.87E-03	2.77E+00
**24h**	3.17E-03	3.82E-05	1.54E-03	4.63E+00
**36h**	3.38E-05	3.58E-05	2.02E-03	6.25E+00
**48h**	9.38E-04	8.95E-06	1.24E-03	4.27E+00
**72h**	1.39E-03	2.63E-05	6.56E-03	2.50E+00
	cgd6_4930			
**2h**	0.00E+00	0.00E+00	0.00E+00	0.00E+00
**6h**	1.24E-07	1.19E-06	8.70E-07	1.28E-05
**12h**	1.40E-08	7.30E-08	1.94E-07	2.91E-06
**24h**	1.39E-07	3.03E-07	7.16E-07	2.91E-05
**36h**	1.42E-06	1.07E-06	1.73E-06	1.30E-05
**48h**	5.89E-07	4.94E-07	1.10E-06	3.96E-05
**72h**	1.90E-07	1.81E-07	3.32E-07	2.01E-06
	cgd6_4940			
**2h**	8.67E-09	0.00E+00	0.00E+00	0.00E+00
**6h**	1.41E-09	1.29E-08	2.97E-08	2.49E-07
**12h**	3.92E-08	1.30E-07	1.32E-07	1.31E-06
**24h**	4.19E-08	8.27E-08	3.30E-08	1.62E-06
**36h**	1.94E-07	2.98E-07	5.31E-07	1.31E-06
**48h**	1.03E-06	3.85E-07	7.20E-07	4.65E-06
**72h**	4.91E-07	2.44E-07	7.55E-07	5.21E-06
	cgd6_4950			
**2h**	0.00E+00	0.00E+00	2.19E-08	0.00E+00
**6h**	3.59E-09	0.00E+00	1.10E-09	0.00E+00
**12h**	2.27E-07	1.08E-06	1.94E-07	2.29E-06

**24h**	3.97E-08	7.70E-08	2.49E-09	5.44E-08
**36h**	2.93E-08	5.76E-08	3.30E-08	2.34E-07
**48h**	2.62E-08	1.14E-08	3.44E-08	7.53E-08
**72h**	2.57E-08	9.09E-09	1.50E-08	3.79E-08
	cgd6_4960			
**2h**	8.39E-05	1.16E-04	2.37E-04	1.46E-06
**6h**	2.98E-04	2.01E-03	2.92E-03	2.08E-05
**12h**	1.89E-04	1.09E-03	9.29E-05	4.32E-05
**24h**	1.46E-04	8.63E-04	9.29E-04	2.73E-05
**36h**	2.84E-04	8.27E-04	6.63E-04	2.24E-05
**48h**	2.11E-03	8.39E-04	6.29E-04	6.99E-06
**72h**	1.32E-03	1.76E-03	9.97E-04	2.54E-05
	cgd6_4970			
**2h**	7.00E-07	6.30E-07	4.90E-08	1.99E-07
**6h**	9.51E-07	8.07E-06	1.60E-06	1.15E-06
**12h**	3.23E-06	1.48E-05	8.07E-06	5.10E-07
**24h**	1.16E-06	3.53E-05	2.55E-06	1.21E-06
**36h**	3.92E-07	7.96E-07	1.63E-06	1.63E-06
**48h**	7.48E-07	2.98E-06	1.24E-06	1.02E-06
**72h**	1.54E-06	7.51E-07	4.62E-06	2.09E-06
	cgd6_4990			
**2h**	2.12E-08	0.00E+00	0.00E+00	0.00E+00
**6h**	0.00E+00	2.02E-08	5.45E-08	6.26E-10
**12h**	7.16E-07	4.58E-06	7.33E-07	1.55E-07
**24h**	2.94E-07	6.14E-07	3.91E-07	1.03E-07
**36h**	9.37E-08	1.86E-07	4.47E-07	2.16E-07
**48h**	1.39E-07	4.77E-08	5.34E-07	8.07E-08
**72h**	1.16E-06	3.57E-07	7.90E-07	7.63E-07
	cgd6_5000			
**2h**	1.87E-03	7.07E-03	6.54E-03	1.81E-03
**6h**	4.01E-04	1.62E-03	2.38E-03	2.29E-03
**12h**	7.24E-04	1.62E-02	1.00E-02	5.81E-04
**24h**	6.44E-04	3.45E-03	3.02E-03	1.06E-03
**36h**	1.91E-04	5.03E-04	1.62E-03	6.20E-04
**48h**	1.28E-04	6.92E-04	1.13E-03	2.31E-04
**72h**	1.24E-04	1.35E-04	6.36E-04	2.03E-04
	cgd6_5010			
**2h**	4.55E-06	3.43E-06	2.45E-06	1.29E-05
**6h**	5.43E-07	5.06E-07	1.79E-06	5.03E-07
**12h**	2.21E-06	2.93E-06	7.85E-06	9.50E-07
**24h**	4.58E-07	5.40E-06	1.48E-06	4.33E-07
**36h**	7.69E-07	9.15E-07	1.67E-06	9.61E-07
**48h**	3.50E-07	2.51E-06	3.39E-06	3.82E-07
**72h**	5.26E-06	1.93E-06	6.68E-06	3.22E-06
	cgd6_5020			
**2h**	9.80E-03	1.52E-03	3.33E-04	4.19E-04
**6h**	5.90E-04	1.90E-04	2.61E-04	7.69E-05
**12h**	2.58E-03	1.98E-03	5.51E-05	1.52E-04
**24h**	1.20E-03	2.81E-04	5.08E-04	9.33E-05
**36h**	2.98E-04	9.96E-05	2.19E-04	2.66E-04
**48h**	1.41E-03	2.33E-04	2.50E-04	4.28E-05
**72h**	3.63E-03	7.38E-04	5.20E-04	3.66E-04
	cgd6_5030			

**2h**	2.22E-06	8.84E-07	4.45E-06	1.12E-06
**6h**	1.71E-06	5.83E-07	3.91E-06	1.04E-06
**12h**	4.57E-07	1.21E-06	1.82E-06	1.16E-06
**24h**	3.04E-07	1.81E-06	2.88E-06	1.36E-06
**36h**	8.02E-07	1.13E-06	1.10E-06	1.03E-06
**48h**	4.38E-07	1.31E-06	1.93E-06	7.41E-07
**72h**	1.96E-06	2.45E-06	4.19E-06	2.69E-06
	cgd6_5050			
**2h**	0.00E+00	1.16E-08	0.00E+00	4.17E-09
**6h**	0.00E+00	0.00E+00	0.00E+00	4.44E-09
**12h**	2.28E-08	4.24E-08	1.40E-08	6.70E-05
**24h**	5.05E-09	1.72E-09	2.73E-09	7.94E-09
**36h**	5.06E-08	4.91E-08	1.20E-08	2.71E-08
**48h**	1.36E-07	4.90E-08	9.34E-08	2.48E-08
**72h**	1.30E-06	4.22E-07	1.39E-06	8.31E-07
	cgd6_5060			
**2h**	1.63E-04	7.85E-04	1.16E-04	1.39E-04
**6h**	2.62E-03	1.18E-02	4.35E-03	5.81E-04
**12h**	1.06E-02	3.62E-02	1.43E-02	4.21E-04
**24h**	9.91E-04	6.02E-03	2.88E-03	6.78E-04
**36h**	8.51E-04	2.87E-03	1.90E-03	4.29E-04
**48h**	1.41E-03	1.26E-03	6.45E-04	6.88E-05
**72h**	2.30E-04	3.25E-04	4.33E-04	2.15E-04
	cgd6_5070			
**2h**	9.10E-07	1.81E-07	8.00E-07	6.00E-07
**6h**	7.44E-06	3.30E-05	9.64E-05	3.77E-06
**12h**	3.35E-07	1.02E-06	1.87E-06	2.94E-07
**24h**	2.26E-06	4.80E-06	4.02E-05	8.15E-06
**36h**	4.64E-06	6.40E-06	1.24E-05	4.61E-06
**48h**	5.16E-06	1.94E-06	8.02E-06	3.18E-06
**72h**	9.49E-08	4.52E-07	5.30E-07	1.16E-07
	cgd6_5080			
**2h**	3.23E-04	3.35E-04	1.06E-04	1.09E-04
**6h**	2.94E-04	1.16E-03	2.95E-03	1.96E-04
**12h**	4.84E-06	3.21E-05	7.46E-04	3.21E-05
**24h**	9.73E-05	1.74E-04	1.23E-03	3.04E-04
**36h**	5.60E-05	1.37E-04	2.58E-04	1.03E-04
**48h**	7.00E-05	1.15E-04	2.05E-04	1.09E-04
**72h**	6.88E-06	8.03E-06	1.46E-05	5.96E-06
	cgd6_5090			
**2h**	1.60E-07	5.55E-08	3.74E-08	3.46E-09
**6h**	7.51E-08	1.06E-07	1.11E-07	8.51E-09
**12h**	7.29E-09	8.35E-09	0.00E+00	1.63E-09
**24h**	3.88E-08	2.06E-08	5.88E-08	2.07E-08
**36h**	2.26E-08	3.89E-08	7.44E-08	1.22E-08
**48h**	4.74E-08	2.72E-08	7.41E-08	2.84E-08
**72h**	2.46E-08	1.71E-08	1.63E-07	1.93E-08
	cgd6_5110			
**2h**	6.38E-06	3.35E-05	8.72E-05	2.71E-05
**6h**	1.21E-05	1.31E-05	1.38E-05	5.59E-06
**12h**	2.00E-06	3.14E-06	1.21E-05	2.62E-06
**24h**	1.06E-05	1.07E-05	2.17E-05	1.28E-05
**36h**	2.21E-06	8.26E-06	5.03E-06	1.30E-05

**48h**	3.00E-06	2.10E-06	3.69E-06	2.88E-06
**72h**	5.77E-07	7.98E-07	2.40E-07	9.75E-07
	cgd6_5130			
**2h**	3.40E-05	1.60E-04	1.08E-04	1.13E-04
**6h**	3.00E-05	5.00E-05	2.59E-05	3.32E-04
**12h**	1.50E-05	1.68E-05	4.50E-05	1.74E-04
**24h**	6.46E-05	6.51E-05	1.18E-04	1.91E-04
**36h**	2.11E-05	1.92E-05	2.69E-05	1.02E-04
**48h**	3.07E-05	6.83E-06	1.25E-05	6.54E-05
**72h**	1.01E-05	2.49E-06	8.09E-06	3.53E-05
	cgd6_5140			
**2h**	3.74E-05	3.20E-05	2.19E-05	2.66E-04
**6h**	2.54E-05	1.83E-05	5.28E-05	7.58E-04
**12h**	1.36E-05	6.21E-06	1.27E-05	1.58E-04
**24h**	6.09E-05	5.88E-05	8.54E-05	2.20E-04
**36h**	1.77E-05	2.51E-05	1.14E-05	8.32E-05
**48h**	4.44E-05	1.78E-05	1.92E-05	1.00E-04
**72h**	3.08E-05	1.01E-05	2.86E-05	6.22E-04
	cgd6_5150			
**2h**	5.93E-06	5.22E-06	1.56E-05	1.93E-05
**6h**	4.36E-07	3.77E-06	6.60E-06	5.59E-05
**12h**	1.26E-06	1.64E-06	5.71E-05	1.73E-05
**24h**	1.10E-05	1.39E-05	1.89E-05	2.36E-05
**36h**	9.78E-06	7.17E-06	1.57E-05	2.81E-05
**48h**	1.26E-05	8.53E-06	5.25E-06	7.39E-05
**72h**	1.55E-05	6.33E-06	1.17E-05	5.61E-05
	cgd6_5160			
**2h**	6.53E-08	3.56E-07	6.31E-08	1.08E-06
**6h**	1.52E-08	3.04E-09	0.00E+00	3.92E-08
**12h**	0.00E+00	1.41E-07	1.54E-08	2.77E-08
**24h**	2.62E-08	4.96E-08	6.90E-08	2.24E-08
**36h**	7.23E-08	1.34E-07	8.31E-08	1.13E-07
**48h**	0.00E+00	2.76E-07	1.84E-07	1.91E-07
**72h**	1.83E-06	1.71E-06	3.13E-06	3.39E-06
	cgd6_5170			
**2h**	1.74E-07	8.12E-08	2.09E-07	8.41E-08
**6h**	2.02E-08	3.89E-08	1.63E-08	4.15E-08
**12h**	1.45E-07	2.94E-08	6.08E-08	1.08E-07
**24h**	0.00E+00	1.85E-07	5.27E-07	1.61E-07
**36h**	4.97E-07	4.24E-07	5.20E-07	7.30E-07
**48h**	0.00E+00	2.67E-07	3.57E-07	3.78E-07
**72h**	5.91E-06	3.02E-06	5.48E-06	4.85E-06
	cgd6_5180			
**2h**	2.22E-07	0.00E+00	1.70E-06	5.40E-05
**6h**	0.00E+00	0.00E+00	0.00E+00	5.93E-07
**12h**	2.65E-08	3.04E-07	1.83E-08	1.02E-07
**24h**	0.00E+00	2.75E-07	7.30E-07	2.91E-07
**36h**	4.91E-06	1.74E-06	2.09E-06	1.44E-05
**48h**	0.00E+00	3.80E-06	1.40E-06	2.51E-04
**72h**	1.49E-05	1.00E-05	1.70E-05	1.10E-04
	cgd6_5240			
**2h**	0.00E+00	4.66E-08	0.00E+00	1.30E-08
**6h**	0.00E+00	3.82E-10	1.99E-09	4.65E-09

**12h**	1.60E-07	2.25E-07	1.51E-07	6.77E-07
**24h**	1.51E-07	2.07E-07	5.00E-07	2.33E-07
**36h**	6.63E-07	8.71E-07	5.72E-07	1.30E-06
**48h**	0.00E+00	9.70E-07	8.12E-07	2.55E-06
**72h**	1.57E-06	1.75E-06	3.55E-06	3.67E-06
	cgd6_5250			
**2h**	2.72E-04	4.39E-04	1.27E-04	3.60E-04
**6h**	1.22E-04	1.87E-04	5.12E-04	6.17E-03
**12h**	4.21E-05	5.99E-05	2.02E-04	6.24E-04
**24h**	1.67E-04	1.21E-04	2.69E-04	3.01E-03
**36h**	4.08E-04	2.95E-04	2.03E-04	6.23E-04
**48h**	7.29E-05	7.25E-05	2.50E-05	6.15E-04
**72h**	3.49E-05	2.62E-05	4.03E-05	2.61E-04
	cgd6_5260			
**2h**	8.51E-06	3.01E-06	2.01E-06	9.73E-06
**6h**	4.00E-06	3.43E-06	2.28E-06	4.30E-05
**12h**	0.00E+00	7.88E-07	2.53E-06	6.49E-06
**24h**	0.00E+00	2.37E-06	6.25E-06	1.26E-05
**36h**	8.18E-06	4.05E-06	4.36E-06	1.41E-05
**48h**	2.68E-06	3.83E-06	7.55E-07	1.88E-05
**72h**	0.00E+00	1.47E-06	4.49E-06	7.17E-06
	cgd6_5280			
**2h**	1.76E-06	5.42E-07	1.40E-07	5.95E-07
**6h**	1.44E-06	2.44E-06	1.13E-06	5.55E-06
**12h**	5.67E-06	1.88E-05	1.06E-05	1.45E-05
**24h**	0.00E+00	4.72E-06	2.74E-06	4.30E-06
**36h**	1.71E-06	1.83E-06	1.11E-05	1.55E-05
**48h**	3.37E-06	2.48E-06	1.36E-06	2.11E-06
**72h**	7.73E-07	5.03E-07	1.55E-06	1.09E-06
	cgd6_5290			
**2h**	3.12E-09	0.00E+00	2.74E-09	1.61E-09
**6h**	0.00E+00	0.00E+00	0.00E+00	0.00E+00
**12h**	7.63E-08	4.34E-08	2.06E-08	4.92E-08
**24h**	6.17E-09	1.08E-08	3.00E-09	1.19E-08
**36h**	2.63E-08	2.33E-08	2.27E-08	3.38E-08
**48h**	1.68E-08	9.48E-09	2.28E-09	8.82E-09
**72h**	2.99E-08	1.01E-08	7.88E-09	1.42E-08
	cgd6_5300			
**2h**	0.00E+00	0.00E+00	0.00E+00	1.13E-06
**6h**	0.00E+00	0.00E+00	0.00E+00	0.00E+00
**12h**	2.24E-04	1.29E-04	4.23E-04	7.45E-04
**24h**	2.83E-05	6.99E-05	4.05E-05	8.75E-05
**36h**	4.19E-05	3.81E-05	1.55E-04	1.98E-04
**48h**	1.81E-05	4.82E-06	1.56E-05	9.07E-05
**72h**	4.83E-05	6.48E-06	3.22E-05	9.03E-05
	cgd6_5310			
**2h**	6.55E-08	3.97E-07	2.74E-07	1.41E-08
**6h**	6.43E-09	5.27E-09	3.71E-09	0.00E+00
**12h**	8.43E-07	5.30E-07	8.01E-07	3.31E-07
**24h**	1.35E-06	1.11E-06	1.11E-06	5.83E-07
**36h**	1.75E-06	7.40E-07	1.81E-06	7.90E-07
**48h**	1.05E-06	7.86E-07	6.94E-07	2.74E-07
**72h**	1.93E-07	1.27E-07	9.86E-08	6.95E-08

	cgd6_5320			
**2h**	5.81E-07	1.84E-06	5.28E-08	1.13E-07
**6h**	2.17E-06	2.84E-06	5.51E-06	2.83E-06
**12h**	8.36E-07	6.95E-07	6.09E-07	1.88E-07
**24h**	3.91E-06	2.10E-06	6.23E-06	1.77E-06
**36h**	5.68E-07	2.13E-06	8.66E-07	7.99E-07
**48h**	1.23E-06	1.83E-06	8.21E-07	2.21E-07
**72h**	3.48E-07	1.93E-07	2.46E-07	1.18E-07
	cgd6_5330			
**2h**	0.00E+00	0.00E+00	0.00E+00	0.00E+00
**6h**	2.26E-05	0.00E+00	0.00E+00	0.00E+00
**12h**	5.07E-05	3.59E-04	6.60E-04	2.42E-05
**24h**	2.87E-05	2.20E-04	2.29E-04	1.77E-05
**36h**	5.69E-05	1.56E-04	3.45E-04	2.26E-04
**48h**	2.90E-05	1.78E-04	2.61E-04	2.09E-05
**72h**	7.62E-06	8.59E-05	2.24E-04	2.37E-05
	cgd6_5340			
**2h**	0.00E+00	9.72E-07	4.43E-07	0.00E+00
**6h**	4.35E-06	0.00E+00	0.00E+00	0.00E+00
**12h**	0.00E+00	5.19E-07	0.00E+00	3.95E-08
**24h**	4.04E-07	1.35E-07	0.00E+00	6.91E-07
**36h**	7.88E-06	3.42E-06	1.12E-06	9.95E-08
**48h**	1.33E-04	9.91E-05	7.47E-04	7.88E-07
**72h**	3.37E-05	3.31E-06	3.15E-06	4.41E-07
	cgd6_5350			
**2h**	0.00E+00	0.00E+00	0.00E+00	0.00E+00
**6h**	1.55E-04	2.43E-05	6.34E-05	1.23E-06
**12h**	3.50E-04	3.62E-04	2.62E-04	3.77E-06
**24h**	3.50E-04	1.47E-04	1.65E-04	6.37E-06
**36h**	7.24E-04	5.52E-04	2.01E-04	3.16E-05
**48h**	2.83E-04	2.33E-04	1.17E-04	1.92E-06
**72h**	4.40E-05	8.18E-06	1.11E-05	1.28E-06
	cgd6_5360			
**2h**	0.00E+00	0.00E+00	0.00E+00	0.00E+00
**6h**	0.00E+00	0.00E+00	0.00E+00	0.00E+00
**12h**	4.68E-07	4.48E-07	7.97E-07	2.59E-07
**24h**	1.70E-07	4.07E-07	1.01E-07	4.62E-08
**36h**	7.17E-07	2.89E-07	9.05E-07	5.55E-07
**48h**	1.28E-07	1.68E-07	1.88E-07	1.17E-07
**72h**	4.05E-08	2.00E-08	5.05E-08	3.12E-08
	cgd6_5370			
**2h**	8.57E-05	0.00E+00	0.00E+00	7.21E-08
**6h**	0.00E+00	0.00E+00	0.00E+00	0.00E+00
**12h**	1.41E-03	2.34E-04	7.67E-05	1.78E-06
**24h**	1.30E-04	5.04E-05	2.33E-05	1.67E-06
**36h**	1.04E-04	3.50E-05	6.66E-05	4.10E-06
**48h**	4.43E-05	4.59E-05	2.76E-05	3.40E-07
**72h**	2.00E-05	1.29E-06	1.04E-05	1.50E-07
	cgd6_5380			
**2h**	1.50E-04	1.33E-03	3.14E-03	3.40E-05
**6h**	9.21E-06	1.47E-04	4.06E-05	4.14E-06
**12h**	5.07E-04	5.38E-03	8.51E-04	2.22E-04
**24h**	3.06E-04	3.02E-03	2.39E-03	6.34E-05

**36h**	2.56E-05	1.98E-04	3.28E-04	1.48E-04
**48h**	2.34E-05	1.91E-04	1.49E-04	1.39E-05
**72h**	1.70E-05	2.35E-04	5.41E-05	1.15E-05
	cgd6_5390			
**2h**	1.43E-04	3.45E-05	8.37E-05	3.04E-06
**6h**	2.24E-03	4.20E-04	2.42E-04	1.04E-05
**12h**	4.15E-05	1.43E-05	1.44E-04	2.87E-06
**24h**	2.23E-04	1.61E-04	5.78E-04	8.40E-06
**36h**	1.12E-04	6.88E-05	5.14E-04	1.20E-05
**48h**	6.42E-05	8.16E-05	6.76E-05	1.63E-06
**72h**	5.17E-05	1.41E-05	4.96E-05	1.69E-06
	cgd6_5400			
**2h**	3.52E-04	2.04E-04	7.96E-05	5.05E-05
**6h**	3.12E-05	3.56E-05	4.76E-05	5.29E-05
**12h**	1.16E-04	3.49E-05	2.85E-04	2.53E-05
**24h**	7.94E-04	2.35E-04	9.39E-05	6.26E-05
**36h**	5.65E-05	7.13E-05	9.51E-05	2.53E-05
**48h**	9.27E-05	4.96E-05	7.11E-05	1.50E-05
**72h**	1.46E-05	4.07E-06	7.00E-06	1.29E-06
	cgd6_5410			
**2h**	3.62E-02	3.45E-01	7.56E-02	3.40E-03
**6h**	1.90E-02	3.06E-02	4.91E-02	2.26E-03
**12h**	2.72E-02	1.60E-02	8.13E-02	6.95E-04
**24h**	6.03E-02	4.12E-02	2.58E-02	3.20E-03
**36h**	6.82E-03	1.47E-02	3.31E-02	8.04E-04
**48h**	6.90E-03	1.27E-02	1.61E-02	3.05E-04
**72h**	2.91E-03	3.66E-03	3.05E-03	1.22E-04
	cgd6_5420			
**2h**	7.32E-08	7.30E-07	0.00E+00	8.18E-08
**6h**	8.70E-08	3.04E-07	5.77E-08	1.16E-07
**12h**	5.98E-07	1.10E-06	1.27E-06	8.33E-07
**24h**	7.79E-07	3.43E-07	5.37E-07	2.33E-07
**36h**	3.72E-07	8.31E-07	1.04E-06	1.11E-06
**48h**	3.49E-07	3.13E-07	7.64E-07	7.66E-08
**72h**	1.00E-07	3.84E-07	1.62E-07	3.51E-07
	cgd6_5510			
**2h**	1.03E-05	1.06E-05	1.27E-06	3.29E-06
**6h**	1.57E-06	1.08E-06	1.56E-07	4.39E-07
**12h**	2.86E-06	2.19E-07	3.48E-07	2.06E-06
**24h**	2.51E-05	8.07E-06	2.71E-07	8.72E-07
**36h**	7.85E-06	5.42E-06	2.88E-07	5.48E-07
**48h**	1.65E-05	1.61E-06	2.18E-07	2.51E-06
**72h**	1.77E-06	1.94E-07	1.06E-07	7.65E-07
	cgd6_5520			
**2h**	1.01E-06	2.00E-06	2.56E-06	8.24E-07
**6h**	9.52E-08	6.31E-07	6.51E-07	2.57E-07
**12h**	7.29E-08	7.44E-08	6.77E-07	2.78E-07
**24h**	3.22E-07	6.46E-07	3.03E-07	6.20E-07
**36h**	6.54E-07	5.38E-07	2.92E-07	1.83E-07
**48h**	4.14E-07	3.79E-07	4.36E-07	2.21E-07
**72h**	2.32E-07	7.55E-08	4.03E-08	1.26E-07
	cgd7_10			
**2h**	1.42E-06	2.02E-06	5.32E-06	1.32E-06

**6h**	3.46E-08	1.19E-07	2.81E-07	9.37E-08
**12h**	5.71E-06	5.60E-06	2.55E-06	3.48E-06
**24h**	2.38E-06	5.24E-06	6.64E-07	1.08E-06
**36h**	1.26E-06	8.18E-07	5.51E-07	7.60E-07
**48h**	7.11E-07	5.54E-07	6.45E-07	4.00E-07
**72h**	2.27E-07	1.03E-07	3.57E-08	1.41E-07
	cgd7_30			
**2h**	8.60E-06	1.73E-06	2.34E-06	8.48E-07
**6h**	2.48E-06	4.38E-06	5.82E-06	5.48E-06
**12h**	1.37E-06	7.06E-07	1.19E-06	1.72E-06
**24h**	2.44E-06	6.31E-06	3.18E-06	4.55E-06
**36h**	3.51E-06	4.18E-06	9.43E-07	1.96E-06
**48h**	3.43E-06	1.20E-06	7.94E-07	7.81E-07
**72h**	2.42E-07	2.52E-07	3.35E-08	1.64E-07
	cgd7_40			
**2h**	0.00E+00	0.00E+00	0.00E+00	0.00E+00
**6h**	2.37E-08	3.93E-08	9.35E-08	7.22E-07
**12h**	5.52E-06	1.03E-06	6.50E-07	2.74E-06
**24h**	6.69E-06	2.89E-06	9.62E-07	7.58E-06
**36h**	2.45E-05	6.90E-06	1.19E-06	6.11E-06
**48h**	3.42E-05	3.44E-06	2.05E-06	3.29E-06
**72h**	1.49E-05	2.14E-06	2.73E-07	4.94E-06
	cgd7_60			
**2h**	0.00E+00	0.00E+00	0.00E+00	0.00E+00
**6h**	4.62E-05	2.41E-05	1.76E-06	5.51E-05
**12h**	1.49E-05	0.00E+00	5.60E-07	4.69E-05
**24h**	3.76E-05	1.07E-05	3.55E-06	5.18E-05
**36h**	5.12E-05	1.70E-04	1.09E-05	3.87E-04
**48h**	1.78E-04	8.26E-05	1.70E-05	6.80E-04
**72h**	1.11E-04	3.47E-05	8.53E-06	3.48E-04
	cgd7_70			
**2h**	2.27E-08	0.00E+00	2.11E-08	0.00E+00
**6h**	4.64E-09	1.66E-09	2.58E-09	0.00E+00
**12h**	1.72E-09	3.08E-09	8.30E-09	0.00E+00
**24h**	1.11E-08	1.84E-08	3.86E-09	1.08E-08
**36h**	2.02E-07	1.77E-07	2.70E-08	4.10E-08
**48h**	2.61E-06	9.35E-07	5.33E-07	6.42E-07
**72h**	2.23E-06	5.08E-07	4.02E-07	9.14E-07
	cgd7_90			
**2h**	1.59E-07	4.47E-08	1.63E-07	6.46E-08
**6h**	5.30E-07	1.39E-06	1.06E-06	1.03E-06
**12h**	1.23E-07	5.10E-07	1.95E-07	6.88E-08
**24h**	6.72E-07	3.21E-06	1.30E-06	4.10E-07
**36h**	6.87E-07	1.96E-06	5.91E-07	5.24E-07
**48h**	1.48E-06	1.39E-06	1.08E-06	5.08E-07
**72h**	2.75E-07	4.43E-07	3.63E-07	7.93E-07
	cgd7_100			
**2h**	1.05E-06	8.60E-07	2.58E-06	1.04E-06
**6h**	3.12E-06	3.12E-06	3.23E-06	3.91E-06
**12h**	4.20E-06	1.05E-05	2.23E-06	5.19E-06
**24h**	2.70E-06	3.28E-06	1.95E-06	1.59E-06
**36h**	1.40E-06	2.87E-06	2.03E-06	1.64E-06
**48h**	1.01E-06	4.40E-07	8.48E-07	7.94E-07

**72h**	4.06E-07	2.74E-04	4.73E-07	7.92E-07
	cgd7_110			
**2h**	6.44E-04	1.10E-03	4.95E-03	1.84E-03
**6h**	4.92E-04	4.79E-03	4.62E-03	2.77E-03
**12h**	2.58E-04	6.83E-03	4.32E-03	1.00E-03
**24h**	5.25E-04	9.01E-03	3.99E-03	1.99E-03
**36h**	2.81E-04	1.85E-03	1.71E-03	1.06E-03
**48h**	1.61E-04	1.07E-03	1.60E-03	7.20E-04
**72h**	3.00E-05	2.11E-03	5.21E-04	3.92E-04
	cgd7_120			
**2h**	2.44E-05	2.29E-06	5.13E-05	5.25E-05
**6h**	8.97E-07	7.33E-05	2.87E-05	9.37E-06
**12h**	3.58E-03	1.14E-02	1.19E-02	1.60E-02
**24h**	5.88E-04	1.98E-03	1.30E-03	3.30E-03
**36h**	2.23E-04	3.62E-03	2.34E-03	9.38E-04
**48h**	6.92E-05	2.29E-03	6.64E-04	5.75E-04
**72h**	2.03E-05	3.59E-03	3.14E-04	3.17E-04
	cgd7_130			
**2h**	5.14E-04	2.81E-04	6.56E-04	6.80E-04
**6h**	1.41E-03	4.99E-03	5.52E-03	1.68E-03
**12h**	3.02E-04	1.98E-03	9.13E-04	1.53E-04
**24h**	4.49E-04	2.93E-03	1.56E-03	5.74E-04
**36h**	4.13E-04	6.94E-04	8.68E-04	4.97E-04
**48h**	2.21E-04	6.20E-04	4.81E-04	3.12E-04
**72h**	2.60E-05	1.07E-04	1.08E-04	1.13E-04
	cgd7_140			
**2h**	0.00E+00	3.29E-05	0.00E+00	3.28E-05
**6h**	4.72E-06	4.46E-05	7.50E-06	1.62E-04
**12h**	3.31E-06	7.47E-05	2.40E-05	0.00E+00
**24h**	2.15E-06	3.51E-04	1.77E-04	1.03E-04
**36h**	8.26E-06	6.31E-05	5.52E-05	7.27E-05
**48h**	3.03E-05	2.05E-05	2.27E-05	3.56E-05
**72h**	2.19E-05	2.66E-04	5.20E-04	8.46E-05
	cgd7_160			
**2h**	1.18E-08	2.13E-08	4.41E-08	9.18E-08
**6h**	3.79E-08	1.19E-06	9.54E-07	6.78E-08
**12h**	9.13E-08	1.77E-07	1.09E-07	1.36E-07
**24h**	1.78E-07	2.84E-07	1.18E-06	2.22E-07
**36h**	1.48E-07	4.05E-07	2.64E-07	5.37E-07
**48h**	5.26E-07	1.08E-06	7.00E-07	8.53E-07
**72h**	1.24E-07	1.26E-06	8.92E-07	1.14E-06
	cgd7_180			
**2h**	0.00E+00	0.00E+00	7.09E-07	0.00E+00
**6h**	1.46E-08	3.20E-05	1.33E-04	1.33E-05
**12h**	1.10E-06	1.50E-05	2.67E-05	7.80E-06
**24h**	7.96E-06	3.32E-04	7.91E-05	2.54E-05
**36h**	9.69E-06	1.08E-04	5.93E-05	9.37E-05
**48h**	2.44E-04	6.48E-04	1.18E-03	1.01E-04
**72h**	3.44E-04	9.67E-04	2.00E-03	3.54E-03
	cgd7_200			
**2h**	1.12E-06	1.23E-06	7.81E-07	9.39E-07
**6h**	3.06E-06	5.47E-06	1.88E-06	3.12E-06
**12h**	8.19E-07	6.50E-06	3.25E-06	7.80E-07

**24h**	2.61E-06	4.33E-06	2.04E-06	1.25E-06
**36h**	4.11E-07	1.52E-06	1.49E-06	9.30E-07
**48h**	7.73E-07	2.68E-06	9.22E-07	2.70E-07
**72h**	1.52E-07	1.62E-07	2.23E-07	1.70E-07
	cgd7_210			
**2h**	6.98E-06	6.48E-06	1.06E-05	9.99E-06
**6h**	5.11E-06	3.18E-06	3.20E-06	6.66E-06
**12h**	6.86E-06	4.82E-05	1.94E-05	7.13E-06
**24h**	1.01E-05	1.91E-05	8.51E-06	1.04E-05
**36h**	4.02E-06	6.51E-06	4.44E-06	3.05E-06
**48h**	2.20E-06	3.70E-06	3.08E-06	1.06E-06
**72h**	3.91E-07	1.94E-06	1.17E-06	9.81E-07
	cgd7_230			
**2h**	7.76E-06	1.19E-06	1.98E-06	8.61E-07
**6h**	1.72E-06	3.28E-06	3.22E-06	1.96E-06
**12h**	7.53E-07	2.78E-06	1.90E-06	1.42E-07
**24h**	1.28E-06	3.42E-06	1.65E-06	8.26E-07
**36h**	1.20E-06	2.24E-06	1.22E-06	6.46E-07
**48h**	4.88E-07	5.83E-07	8.39E-07	2.64E-07
**72h**	3.29E-07	8.76E-07	3.20E-07	2.29E-07
	cgd7_240			
**2h**	1.59E-04	4.10E-04	3.43E-04	3.59E-07
**6h**	9.03E-06	5.21E-04	8.91E-06	4.93E-07
**12h**	4.76E-04	5.00E-06	2.84E-04	4.81E-06
**24h**	3.24E-04	2.03E-04	4.77E-04	2.05E-06
**36h**	1.20E-03	1.07E-03	1.07E-03	3.40E-06
**48h**	1.78E-03	9.65E-04	1.12E-03	5.67E-06
**72h**	1.05E-03	3.42E-04	2.56E-03	6.06E-06
	cgd7_250			
**2h**	2.05E-05	8.24E-06	5.10E-06	5.52E-06
**6h**	1.01E-07	2.48E-06	7.79E-06	1.11E-06
**12h**	2.90E-06	4.88E-07	3.83E-06	4.11E-06
**24h**	4.54E-06	4.48E-06	5.46E-06	3.75E-06
**36h**	1.53E-06	1.71E-06	1.65E-06	6.89E-07
**48h**	1.10E-06	1.08E-06	1.37E-06	1.03E-06
**72h**	7.33E-07	2.18E-07	6.42E-07	1.18E-06
	cgd7_260			
**2h**	2.33E-02	1.18E-04	1.38E-02	6.69E-06
**6h**	3.25E-04	2.78E-04	1.81E-03	1.86E-05
**12h**	7.68E-04	1.50E-03	1.23E-03	1.56E-05
**24h**	1.25E-03	2.11E-04	1.99E-03	5.06E-05
**36h**	4.13E-04	2.01E-04	5.62E-04	1.33E-04
**48h**	4.57E-04	4.74E-05	3.76E-04	3.05E-05
**72h**	3.04E-04	1.24E-04	1.68E-04	1.76E-05
	cgd7_270			
**2h**	1.57E-07	0.00E+00	4.90E-07	1.04E-05
**6h**	0.00E+00	0.00E+00	0.00E+00	0.00E+00
**12h**	4.61E-07	2.21E-07	2.04E-07	1.02E-05
**24h**	5.25E-07	3.66E-08	4.93E-07	6.56E-06
**36h**	2.26E-06	2.97E-06	7.29E-06	2.21E-05
**48h**	3.74E-06	6.47E-07	7.78E-06	8.40E-05
**72h**	1.08E-05	4.18E-06	1.01E-05	4.61E-05
	cgd7_280			

**2h**	0.00E+00	0.00E+00	0.00E+00	0.00E+00
**6h**	0.00E+00	0.00E+00	0.00E+00	0.00E+00
**12h**	1.81E-05	4.54E-06	2.45E-05	1.21E-05
**24h**	8.64E-06	4.09E-06	1.89E-05	8.36E-06
**36h**	3.70E-05	2.51E-05	1.26E-04	5.25E-05
**48h**	2.28E-05	2.82E-06	3.41E-05	3.89E-05
**72h**	5.05E-05	1.14E-05	1.19E-04	2.50E-05
	cgd7_290			
**2h**	0.00E+00	0.00E+00	0.00E+00	0.00E+00
**6h**	0.00E+00	0.00E+00	0.00E+00	0.00E+00
**12h**	1.38E-04	7.96E-05	1.92E-04	2.57E-06
**24h**	1.67E-04	2.44E-05	4.55E-04	6.44E-06
**36h**	5.93E-04	4.99E-04	6.68E-04	1.29E-05
**48h**	4.07E-04	1.16E-04	8.76E-04	5.16E-06
**72h**	3.61E-04	2.78E-04	6.04E-04	2.28E-05
	cgd7_300			
**2h**	1.52E-06	0.00E+00	0.00E+00	0.00E+00
**6h**	5.64E-08	0.00E+00	0.00E+00	0.00E+00
**12h**	0.00E+00	0.00E+00	1.46E-07	2.08E-07
**24h**	7.70E-07	6.91E-08	4.47E-06	5.88E-08
**36h**	1.01E-05	7.95E-06	8.44E-05	1.44E-06
**48h**	2.13E-02	5.41E-03	3.95E-02	1.54E-04
**72h**	1.19E-02	6.96E-03	2.38E-02	1.74E-04
	cgd7_320			
**2h**	2.76E-04	1.11E-04	1.39E-03	4.54E-04
**6h**	1.61E-04	4.33E-04	4.54E-03	1.71E-03
**12h**	3.32E-05	1.52E-04	2.66E-03	2.44E-04
**24h**	2.01E-04	2.74E-04	1.32E-03	1.01E-03
**36h**	5.72E-05	1.01E-04	4.11E-04	1.74E-04
**48h**	5.49E-05	5.22E-05	3.12E-04	2.09E-04
**72h**	2.39E-06	6.89E-06	2.04E-04	4.90E-05
	cgd7_330			
**2h**	1.40E-08	0.00E+00	0.00E+00	0.00E+00
**6h**	6.76E-07	7.88E-07	6.59E-06	1.24E-05
**12h**	2.46E-07	1.62E-07	1.97E-07	3.12E-06
**24h**	4.58E-07	3.38E-07	3.54E-06	7.29E-06
**36h**	8.92E-07	6.78E-06	1.61E-06	6.63E-06
**48h**	1.06E-06	7.11E-07	9.64E-07	1.66E-06
**72h**	1.81E-07	7.51E-07	4.19E-07	1.01E-06
	cgd7_340			
**2h**	8.57E-07	2.22E-07	4.08E-06	9.78E-07
**6h**	8.27E-05	9.41E-05	5.58E-04	6.94E-05
**12h**	7.56E-05	1.99E-05	1.66E-04	1.16E-05
**24h**	1.34E-04	2.76E-05	3.02E-04	2.77E-05
**36h**	7.86E-05	1.61E-04	1.99E-04	3.48E-05
**48h**	1.33E-04	8.71E-05	2.28E-04	2.62E-05
**72h**	6.76E-05	1.12E-05	8.45E-05	6.76E-06
	cgd7_350			
**2h**	0.00E+00	0.00E+00	0.00E+00	5.33E-08
**6h**	2.30E-08	2.27E-07	2.03E-07	2.21E-06
**12h**	5.42E-07	6.26E-07	5.04E-07	5.11E-06
**24h**	3.26E-07	3.45E-07	4.89E-07	3.92E-06
**36h**	3.13E-07	3.15E-06	1.29E-06	3.99E-06

**48h**	8.41E-07	1.02E-06	7.81E-07	5.16E-06
**72h**	1.70E-07	8.64E-07	2.60E-07	1.62E-06
	cgd7_370			
**2h**	4.14E-04	0.00E+00	2.38E-04	2.12E-06
**6h**	1.35E-04	3.34E-04	7.51E-04	5.75E-06
**12h**	1.16E-02	2.95E-02	1.19E-02	5.42E-05
**24h**	8.10E-03	5.94E-03	7.79E-03	9.14E-06
**36h**	7.75E-03	1.32E-02	2.55E-02	3.44E-05
**48h**	4.15E-03	1.69E-03	1.42E-02	1.92E-05
**72h**	8.13E-03	1.60E-03	1.28E-02	2.02E-05
	cgd7_380			
**2h**	0.00E+00	7.81E-07	0.00E+00	0.00E+00
**6h**	1.11E-05	4.84E-05	5.83E-05	6.05E-05
**12h**	2.07E-06	1.03E-05	1.87E-06	1.37E-05
**24h**	3.47E-06	1.61E-05	7.83E-06	3.55E-05
**36h**	2.33E-06	3.58E-05	3.59E-06	2.69E-05
**48h**	2.30E-05	3.71E-05	1.81E-05	3.19E-05
**72h**	4.31E-06	1.02E-05	5.71E-06	1.46E-05
	cgd7_400			
**2h**	0.00E+00	0.00E+00	0.00E+00	4.91E-09
**6h**	4.04E-08	0.00E+00	0.00E+00	0.00E+00
**12h**	2.88E-09	1.53E-08	1.38E-09	2.38E-09
**24h**	8.52E-09	3.05E-09	3.46E-08	1.15E-08
**36h**	5.44E-08	1.73E-07	9.76E-08	1.39E-07
**48h**	4.01E-05	6.59E-05	1.31E-04	5.94E-05
**72h**	1.32E-04	1.44E-04	2.93E-04	1.27E-04
	cgd7_410			
**2h**	0.00E+00	0.00E+00	0.00E+00	1.68E-06
**6h**	1.42E-06	4.93E-06	3.19E-06	3.43E-06
**12h**	1.07E-05	8.09E-05	6.00E-06	6.05E-05
**24h**	1.57E-05	2.48E-04	1.82E-05	8.18E-05
**36h**	7.72E-05	4.01E-04	7.34E-05	1.01E-03
**48h**	1.19E-04	2.93E-04	3.64E-05	3.36E-04
**72h**	1.39E-05	1.50E-04	5.62E-06	6.18E-05
	cgd7_420			
**2h**	9.97E-04	2.01E-03	7.08E-05	1.57E-03
**6h**	1.65E-04	8.11E-04	3.71E-04	7.29E-04
**12h**	3.28E-04	1.93E-03	6.02E-04	1.49E-03
**24h**	3.07E-04	2.31E-03	1.27E-04	6.56E-04
**36h**	4.64E-04	1.67E-03	6.63E-05	5.36E-04
**48h**	2.25E-04	4.42E-04	4.88E-04	4.94E-04
**72h**	1.59E-04	5.07E-04	6.44E-05	1.07E-04
	cgd7_450			
**2h**	4.89E-02	1.31E-03	4.70E-03	5.54E-03
**6h**	1.45E-02	2.58E-03	2.92E-02	5.72E-04
**12h**	7.31E-03	3.65E-04	2.16E-02	1.23E-02
**24h**	2.39E-03	2.22E-03	6.96E-03	2.13E-03
**36h**	1.94E-03	1.55E-03	2.53E-04	8.69E-04
**48h**	4.56E-03	1.47E-03	5.67E-03	8.64E-03
**72h**	1.87E-03	1.07E-03	7.24E-03	4.70E-02
	cgd7_460			
**2h**	5.78E-05	8.77E-04	8.63E-06	7.44E-04
**6h**	5.28E-05	1.26E-03	1.01E-04	5.28E-04

**12h**	3.92E-05	4.24E-04	7.12E-05	3.67E-04
**24h**	4.26E-05	1.59E-03	3.08E-05	5.58E-04
**36h**	2.21E-04	1.34E-03	1.18E-04	9.09E-04
**48h**	1.68E-04	1.20E-03	1.52E-04	1.03E-03
**72h**	6.11E-04	1.57E-03	3.56E-04	1.60E-03
	cgd7_470			
**2h**	1.32E-07	2.73E-08	1.17E-08	8.25E-08
**6h**	1.73E-07	5.07E-07	3.28E-07	8.68E-07
**12h**	1.43E-07	6.13E-07	2.61E-07	2.32E-07
**24h**	1.25E-07	7.59E-07	2.89E-07	7.40E-07
**36h**	9.61E-07	1.58E-06	9.58E-07	1.98E-06
**48h**	3.28E-07	1.50E-06	4.88E-07	1.31E-06
**72h**	1.21E-06	8.82E-07	1.24E-06	1.24E-06
	cgd7_480			
**2h**	3.64E-05	2.30E-05	6.52E-06	2.85E-05
**6h**	4.04E-07	1.07E-06	8.02E-07	2.56E-06
**12h**	1.22E-04	1.23E-04	1.73E-04	6.72E-05
**24h**	3.83E-05	1.32E-04	9.80E-06	2.83E-05
**36h**	4.21E-05	4.34E-05	3.99E-05	4.63E-05
**48h**	1.09E-05	1.41E-05	1.69E-05	1.16E-05
**72h**	1.77E-05	1.40E-05	1.25E-05	9.62E-06
	cgd7_490			
**2h**	3.43E-07	1.98E-07	4.14E-07	6.83E-08
**6h**	3.67E-07	2.11E-06	2.72E-06	1.89E-06
**12h**	3.25E-06	1.78E-05	3.71E-06	6.05E-06
**24h**	6.25E-07	1.49E-05	7.37E-07	5.36E-06
**36h**	1.11E-05	1.61E-05	5.31E-06	1.04E-05
**48h**	3.81E-06	1.15E-05	1.89E-06	7.88E-06
**72h**	3.40E-06	4.53E-06	1.59E-06	3.10E-06
	cgd7_500			
**2h**	2.00E-06	3.63E-06	6.23E-07	1.30E-06
**6h**	2.88E-07	6.58E-07	3.56E-07	4.46E-07
**12h**	3.80E-07	4.42E-07	6.88E-07	9.41E-07
**24h**	1.98E-07	1.26E-06	4.65E-07	9.11E-07
**36h**	2.02E-07	9.15E-07	3.00E-07	4.30E-07
**48h**	2.60E-07	4.08E-07	3.18E-07	3.32E-07
**72h**	2.15E-07	3.08E-07	2.38E-07	2.60E-07
	cgd7_580			
**2h**	0.00E+00	0.00E+00	0.00E+00	0.00E+00
**6h**	0.00E+00	0.00E+00	0.00E+00	0.00E+00
**12h**	1.51E-05	1.63E-04	3.24E-05	6.03E-05
**24h**	2.48E-06	6.29E-05	5.87E-06	3.86E-05
**36h**	6.60E-05	1.53E-04	3.18E-05	2.10E-04
**48h**	1.07E-04	1.36E-04	8.64E-05	2.41E-04
**72h**	1.19E-04	3.04E-04	1.84E-04	3.10E-04
	cgd7_630			
**2h**	1.87E-06	1.17E-06	3.01E-07	5.44E-07
**6h**	6.72E-08	1.13E-06	3.46E-07	5.84E-07
**12h**	3.94E-06	3.29E-06	4.05E-06	4.78E-06
**24h**	3.88E-07	2.70E-06	4.71E-07	2.43E-06
**36h**	1.79E-06	2.41E-06	1.00E-06	1.97E-06
**48h**	1.01E-06	9.73E-07	7.46E-07	1.05E-06
**72h**	1.30E-06	9.00E-07	2.02E-06	1.99E-06

	cgd7_1470			
**2h**	2.75E-05	6.94E-05	6.02E-05	1.15E-04
**6h**	1.23E-05	4.50E-05	1.04E-04	8.84E-05
**12h**	7.76E-07	1.51E-07	6.10E-05	6.61E-06
**24h**	9.38E-06	1.14E-05	4.30E-05	5.23E-06
**36h**	7.51E-06	1.99E-06	1.35E-05	2.22E-05
**48h**	1.72E-05	4.79E-06	2.12E-05	3.93E-05
**72h**	6.69E-06	1.20E-06	1.74E-05	2.25E-05
	cgd7_1480			
**2h**	8.34E-03	4.35E-03	2.48E-03	2.27E-02
**6h**	7.04E-03	3.90E-03	7.28E-03	3.04E-02
**12h**	1.32E-04	4.21E-05	9.79E-04	1.47E-03
**24h**	2.78E-03	1.63E-03	1.41E-03	4.93E-03
**36h**	9.91E-04	5.53E-04	7.66E-04	4.48E-03
**48h**	1.50E-03	1.06E-03	8.29E-04	9.78E-04
**72h**	2.48E-03	8.64E-04	2.04E-03	7.23E-04
	cgd7_1490			
**2h**	1.59E-05	1.37E-05	9.75E-06	1.55E-05
**6h**	3.24E-05	3.91E-05	4.93E-05	2.39E-05
**12h**	3.61E-07	6.42E-07	3.18E-06	1.60E-06
**24h**	5.93E-06	9.78E-06	1.47E-05	2.29E-06
**36h**	2.64E-06	1.85E-06	3.21E-06	5.69E-06
**48h**	4.10E-06	1.22E-06	2.07E-06	1.69E-06
**72h**	1.18E-06	6.54E-07	2.32E-06	2.69E-06
	cgd7_1500			
**2h**	1.19E-07	1.59E-08	0.00E+00	1.45E-06
**6h**	3.67E-08	3.41E-08	1.23E-07	2.26E-08
**12h**	7.07E-09	1.92E-09	2.40E-08	9.86E-09
**24h**	4.07E-08	2.35E-08	2.40E-08	1.74E-08
**36h**	5.71E-08	4.80E-08	1.04E-07	7.06E-08
**48h**	5.59E-07	1.07E-07	2.41E-07	5.86E-07
**72h**	1.63E-06	6.48E-07	2.69E-06	2.39E-06
	cgd7_1510			
**2h**	5.43E-07	4.55E-08	1.45E-08	1.47E-07
**6h**	2.83E-08	6.12E-08	4.35E-08	2.03E-07
**12h**	6.27E-07	7.80E-07	1.71E-06	7.87E-07
**24h**	1.22E-07	4.54E-07	1.42E-07	2.93E-08
**36h**	3.11E-07	3.52E-07	9.48E-07	1.38E-06
**48h**	4.38E-07	1.66E-07	1.87E-07	7.23E-07
**72h**	1.13E-06	7.58E-07	1.96E-06	2.24E-06
	cgd7_1540			
**2h**	6.83E-06	2.87E-06	3.55E-06	2.05E-05
**6h**	1.86E-06	1.35E-06	3.88E-06	3.21E-05
**12h**	1.12E-06	1.77E-06	5.48E-06	4.39E-06
**24h**	7.57E-07	1.49E-06	6.82E-06	1.29E-06
**36h**	1.88E-06	1.39E-06	5.72E-06	1.94E-05
**48h**	4.79E-06	1.45E-06	4.88E-06	9.11E-06
**72h**	8.35E-07	1.17E-06	4.73E-06	1.04E-05
	cgd7_1550			
**2h**	6.74E-05	3.64E-05	2.66E-05	1.58E-04
**6h**	2.82E-05	3.42E-05	1.08E-04	7.89E-05
**12h**	5.42E-06	3.39E-06	2.12E-04	4.54E-05
**24h**	1.65E-05	5.25E-05	9.95E-05	1.43E-05

**36h**	1.57E-05	2.32E-05	6.16E-05	5.07E-05
**48h**	2.08E-05	7.32E-06	3.07E-05	6.30E-05
**72h**	8.34E-06	6.09E-06	1.59E-05	1.85E-05
	cgd7_1580			
**2h**	2.40E-06	3.92E-07	3.47E-07	1.97E-06
**6h**	8.06E-07	1.03E-06	1.65E-06	1.89E-06
**12h**	2.33E-07	4.90E-08	1.24E-06	4.89E-07
**24h**	5.45E-07	7.08E-07	1.06E-06	3.49E-07
**36h**	5.10E-07	1.21E-06	1.21E-06	1.53E-06
**48h**	1.80E-06	2.98E-07	1.21E-06	2.32E-06
**72h**	4.92E-07	9.94E-07	7.27E-07	1.20E-06
	cgd7_1610			
**2h**	3.62E-06	1.10E-06	4.13E-07	5.94E-06
**6h**	1.47E-06	1.64E-06	5.53E-06	7.93E-06
**12h**	5.72E-08	6.15E-08	2.07E-06	1.16E-06
**24h**	7.28E-07	9.44E-07	9.51E-06	9.60E-07
**36h**	1.38E-06	2.26E-06	2.89E-06	4.50E-06
**48h**	1.34E-06	2.96E-07	1.36E-06	3.25E-06
**72h**	1.32E-07	1.58E-07	1.59E-07	3.83E-07
	cgd7_1630			
**2h**	4.44E-06	8.29E-07	1.36E-06	6.85E-06
**6h**	1.34E-06	3.14E-06	1.30E-05	1.12E-05
**12h**	5.07E-09	2.20E-08	5.29E-06	4.13E-07
**24h**	5.63E-07	3.94E-07	7.10E-06	1.20E-06
**36h**	3.19E-07	6.41E-07	7.58E-07	2.72E-06
**48h**	8.70E-07	2.34E-07	1.26E-06	1.80E-06
**72h**	6.08E-08	3.89E-07	2.39E-06	7.16E-07
	cgd7_1640			
**2h**	1.83E-06	5.88E-07	7.89E-07	3.95E-06
**6h**	1.92E-06	2.19E-06	6.14E-06	5.69E-06
**12h**	2.91E-07	2.44E-07	4.27E-06	1.59E-06
**24h**	7.71E-07	1.16E-06	6.70E-06	1.20E-06
**36h**	9.25E-07	8.78E-07	1.36E-06	2.11E-06
**48h**	3.53E-06	1.93E-07	1.26E-06	1.28E-06
**72h**	1.13E-07	1.71E-07	3.03E-07	3.02E-07
	cgd7_1650			
**2h**	2.44E-06	4.01E-07	3.19E-07	3.59E-06
**6h**	1.43E-06	1.62E-06	6.38E-06	6.61E-06
**12h**	3.50E-05	1.79E-05	1.94E-04	7.92E-05
**24h**	6.51E-06	1.18E-05	3.27E-05	1.15E-05
**36h**	6.91E-06	1.23E-05	7.28E-05	9.60E-05
**48h**	8.76E-06	9.74E-07	1.31E-05	2.35E-05
**72h**	1.62E-06	5.65E-06	4.90E-06	7.66E-06
	cgd7_640			
**2h**	8.68E-06	6.18E-07	7.66E-06	1.66E-06
**6h**	9.67E-07	3.29E-06	1.54E-06	2.77E-04
**12h**	2.18E-06	4.15E-07	9.36E-07	4.08E-06
**24h**	4.95E-06	1.06E-06	1.57E-06	1.07E-05
**36h**	1.37E-06	1.21E-06	1.91E-06	3.03E-06
**48h**	1.07E-06	6.62E-07	1.82E-06	1.34E-06
**72h**	9.61E-07	8.55E-07	6.71E-07	1.11E-06
	cgd7_650			
**2h**	1.93E-06	3.68E-08	1.80E-07	1.12E-06

**6h**	5.18E-06	3.77E-05	7.63E-06	2.91E-05
**12h**	9.37E-06	1.10E-06	4.76E-06	2.46E-05
**24h**	1.62E-05	1.32E-05	1.32E-05	8.71E-05
**36h**	1.29E-05	5.20E-06	1.63E-05	3.87E-05
**48h**	4.24E-05	5.53E-06	1.95E-05	3.02E-05
**72h**	4.60E-06	2.11E-06	2.55E-06	9.95E-06
	cgd7_660			
**2h**	0.00E+00	0.00E+00	0.00E+00	0.00E+00
**6h**	0.00E+00	0.00E+00	4.66E-09	2.37E-09
**12h**	8.51E-08	6.93E-09	7.76E-08	3.56E-06
**24h**	6.21E-08	1.41E-08	2.29E-08	7.33E-08
**36h**	9.59E-08	8.49E-08	2.00E-07	9.51E-08
**48h**	2.42E-07	1.36E-07	1.88E-07	2.02E-07
**72h**	8.60E-08	1.60E-07	4.47E-07	2.15E-07
	cgd7_670			
**2h**	1.61E-07	6.31E-09	6.12E-08	3.46E-08
**6h**	7.48E-09	5.28E-08	9.52E-08	9.69E-08
**12h**	1.47E-07	9.13E-08	1.40E-07	4.71E-07
**24h**	3.35E-08	3.55E-08	5.55E-08	9.21E-08
**36h**	9.39E-08	1.27E-07	1.95E-07	1.05E-07
**48h**	2.76E-08	7.51E-08	2.87E-08	4.73E-08
**72h**	5.96E-08	2.32E-08	1.35E-07	4.54E-08
	cgd7_680			
**2h**	1.51E-08	2.32E-09	5.86E-08	2.64E-08
**6h**	0.00E+00	0.00E+00	1.62E-09	2.38E-08
**12h**	6.67E-08	4.09E-08	1.73E-08	3.61E-08
**24h**	3.17E-08	2.18E-08	2.11E-08	8.78E-08
**36h**	6.81E-08	5.09E-08	5.47E-08	5.05E-08
**48h**	1.91E-07	3.67E-07	2.19E-07	4.67E-07
**72h**	7.11E-08	1.75E-07	6.01E-08	6.20E-08
	cgd7_690			
**2h**	2.22E-07	7.19E-08	8.62E-08	4.03E-09
**6h**	2.63E-07	2.39E-07	6.82E-08	1.58E-06
**12h**	3.39E-09	2.83E-08	5.13E-08	7.40E-08
**24h**	1.62E-07	1.65E-07	1.65E-07	1.20E-06
**36h**	2.41E-07	8.69E-08	1.49E-07	3.61E-07
**48h**	1.52E-07	3.49E-08	2.04E-07	6.27E-07
**72h**	1.29E-07	3.39E-07	2.05E-07	6.50E-07
	cgd7_700			
**2h**	3.29E-08	0.00E+00	2.24E-09	3.87E-09
**6h**	2.31E-07	1.30E-07	1.76E-07	3.17E-07
**12h**	3.00E-08	6.93E-09	4.67E-09	1.26E-08
**24h**	6.97E-08	7.33E-08	1.72E-07	7.60E-08
**36h**	5.79E-08	5.84E-08	2.43E-08	1.44E-07
**48h**	6.64E-08	2.08E-08	5.75E-08	1.03E-07
**72h**	4.35E-08	6.67E-08	9.69E-08	1.57E-07
	cgd7_720			
**2h**	0.00E+00	0.00E+00	0.00E+00	0.00E+00
**6h**	0.00E+00	0.00E+00	1.38E-09	0.00E+00
**12h**	1.15E-06	3.53E-07	8.52E-07	3.01E-06
**24h**	8.43E-08	4.53E-08	9.79E-08	1.81E-07
**36h**	1.47E-07	7.31E-07	4.87E-07	1.16E-07
**48h**	1.17E-07	9.51E-08	3.23E-07	2.10E-07

**72h**	3.75E-08	2.17E-07	1.68E-07	3.82E-08
	cgd7_730			
**2h**	0.00E+00	0.00E+00	0.00E+00	0.00E+00
**6h**	5.04E-06	1.33E-06	7.21E-07	9.27E-06
**12h**	1.16E-07	3.61E-09	1.39E-09	2.29E-05
**24h**	2.53E-07	3.58E-07	1.34E-06	9.83E-06
**36h**	1.43E-06	5.27E-07	5.85E-07	1.19E-05
**48h**	9.10E-07	2.32E-07	3.66E-07	1.09E-05
**72h**	5.03E-07	2.60E-07	1.75E-07	6.94E-06
	cgd7_740			
**2h**	2.12E-07	4.12E-09	1.66E-08	6.31E-07
**6h**	1.83E-08	2.49E-08	4.78E-08	8.29E-08
**12h**	2.90E-09	2.65E-08	1.39E-08	5.37E-09
**24h**	5.39E-08	4.24E-08	1.98E-07	1.38E-07
**36h**	2.01E-07	4.80E-08	3.31E-07	1.41E-07
**48h**	3.55E-07	8.58E-08	2.05E-07	2.00E-07
**72h**	5.27E-08	6.08E-08	5.57E-08	1.08E-07
	cgd7_750			
**2h**	2.93E-06	1.48E-07	1.19E-07	6.62E-07
**6h**	4.40E-08	9.28E-08	2.02E-07	3.08E-07
**12h**	1.81E-07	7.16E-08	3.93E-07	1.63E-06
**24h**	3.12E-07	4.59E-07	1.67E-06	8.25E-07
**36h**	3.84E-06	2.38E-06	1.63E-06	1.03E-05
**48h**	4.97E-06	1.51E-06	2.12E-06	8.54E-06
**72h**	1.37E-06	9.07E-07	3.34E-07	1.86E-06
	cgd7_760			
**2h**	1.28E-02	1.14E-02	3.47E-03	4.29E-01
**6h**	1.32E-02	1.00E-02	4.85E-03	1.23E-01
**12h**	1.24E-02	8.54E-03	4.33E-03	2.59E-01
**24h**	9.71E-03	1.14E-02	6.79E-03	7.06E-01
**36h**	5.92E-04	1.25E-02	1.87E-03	0.00E+00
**48h**	1.99E-03	3.31E-03	6.13E-04	0.00E+00
**72h**	1.26E-03	1.89E-03	5.02E-04	1.01E-02
	cgd7_770			
**2h**	0.00E+00	0.00E+00	0.00E+00	0.00E+00
**6h**	6.25E-05	4.03E-04	5.37E-05	0.00E+00
**12h**	6.18E-05	1.29E-04	3.24E-04	1.07E-05
**24h**	7.28E-05	4.39E-04	1.12E-03	9.25E-06
**36h**	3.01E-04	2.44E-04	6.97E-04	3.78E-05
**48h**	3.09E-04	2.33E-04	7.84E-04	4.24E-05
**72h**	5.80E-04	2.97E-03	1.58E-03	4.67E-05
	cgd7_780			
**2h**	0.00E+00	0.00E+00	0.00E+00	0.00E+00
**6h**	4.03E-06	5.13E-05	1.95E-05	2.52E-05
**12h**	1.21E-04	7.16E-05	3.80E-05	9.14E-05
**24h**	2.36E-05	1.23E-04	5.44E-05	8.76E-05
**36h**	4.09E-05	6.09E-05	4.72E-05	7.56E-05
**48h**	2.15E-05	4.84E-05	2.23E-05	2.48E-04
**72h**	8.67E-06	7.84E-06	4.57E-06	4.62E-05
	cgd7_790			
**2h**	6.30E-02	2.80E-02	3.03E-02	1.16E-02
**6h**	3.80E-02	4.68E-02	2.18E-02	6.71E-03
**12h**	2.48E-02	4.35E-02	3.18E-02	2.88E-03

**24h**	2.13E-02	3.66E-02	1.69E-02	3.08E-03
**36h**	6.16E-03	5.83E-03	3.97E-03	1.78E-03
**48h**	4.30E-03	7.81E-03	2.89E-03	2.66E-03
**72h**	1.54E-03	1.70E-03	1.13E-03	1.21E-03
	cgd7_820			
**2h**	4.55E-07	8.11E-09	6.12E-07	8.52E-07
**6h**	1.23E-07	5.95E-07	2.91E-07	3.05E-07
**12h**	9.19E-09	2.09E-07	1.86E-07	2.90E-08
**24h**	1.39E-07	4.35E-07	2.14E-07	2.83E-08
**36h**	1.33E-07	1.33E-07	2.01E-07	8.87E-08
**48h**	3.79E-08	4.18E-08	3.58E-08	8.18E-08
**72h**	2.45E-07	1.10E-07	1.60E-07	2.75E-07
	cgd7_830			
**2h**	2.47E-03	3.41E-03	1.64E-03	1.64E-04
**6h**	1.05E-03	9.30E-04	4.47E-04	1.33E-04
**12h**	2.98E-04	2.15E-03	1.26E-03	7.25E-05
**24h**	7.65E-04	1.45E-03	5.97E-04	2.10E-05
**36h**	4.54E-04	1.16E-04	3.35E-04	3.01E-05
**48h**	1.37E-04	3.32E-04	6.87E-05	2.63E-05
**72h**	3.23E-04	4.91E-04	4.24E-04	4.12E-05
	cgd7_840			
**2h**	1.09E-08	0.00E+00	7.92E-09	0.00E+00
**6h**	0.00E+00	0.00E+00	7.08E-10	3.56E-09
**12h**	0.00E+00	6.71E-09	4.41E-09	7.83E-10
**24h**	3.14E-08	1.58E-08	1.56E-08	8.46E-09
**36h**	1.39E-07	9.05E-07	7.21E-08	5.92E-08
**48h**	3.11E-06	2.00E-05	6.47E-06	2.29E-05
**72h**	3.16E-05	3.57E-05	2.60E-05	3.25E-05
	cgd7_860			
**2h**	5.29E-03	1.55E-03	8.30E-03	8.03E-04
**6h**	2.15E-03	4.75E-04	1.15E-03	2.39E-04
**12h**	1.50E-03	5.38E-03	1.81E-03	2.80E-04
**24h**	3.23E-03	1.01E-03	1.41E-03	5.68E-05
**36h**	8.11E-04	6.89E-04	4.91E-04	1.30E-04
**48h**	1.58E-03	1.03E-03	1.14E-03	3.26E-04
**72h**	2.31E-03	2.70E-03	2.01E-03	5.14E-04
	cgd7_880			
**2h**	5.69E-07	2.46E-08	4.77E-07	1.63E-06
**6h**	2.80E-06	7.01E-06	4.04E-06	2.55E-06
**12h**	6.75E-07	1.81E-06	2.13E-06	6.86E-07
**24h**	1.68E-06	3.93E-06	3.13E-06	4.17E-07
**36h**	1.74E-06	1.95E-06	1.61E-06	9.98E-07
**48h**	1.57E-06	1.52E-06	1.30E-06	1.55E-06
**72h**	2.23E-06	1.63E-06	1.58E-06	2.28E-06
	cgd7_900			
**2h**	1.02E-09	0.00E+00	0.00E+00	0.00E+00
**6h**	4.93E-08	1.77E-07	2.46E-08	2.32E-08
**12h**	1.94E-07	1.52E-07	1.53E-07	1.55E-07
**24h**	1.54E-07	8.94E-07	6.63E-07	6.21E-08
**36h**	5.48E-07	4.54E-07	5.22E-07	1.27E-07
**48h**	3.20E-07	2.68E-07	2.86E-07	2.70E-07
**72h**	1.91E-07	2.39E-07	1.20E-07	1.16E-07
	cgd7_910			

**2h**	0.00E+00	0.00E+00	0.00E+00	0.00E+00
**6h**	0.00E+00	0.00E+00	0.00E+00	3.15E-07
**12h**	5.84E-05	2.52E-04	2.57E-05	7.82E-05
**24h**	5.24E-06	6.36E-06	5.97E-06	3.55E-06
**36h**	8.98E-06	5.28E-05	2.76E-05	2.51E-05
**48h**	2.27E-05	3.90E-05	1.07E-05	4.46E-05
**72h**	3.18E-05	9.24E-05	1.72E-05	5.46E-05
	cgd7_920			
**2h**	3.76E-05	3.12E-04	9.29E-05	5.86E-04
**6h**	6.99E-05	3.16E-04	4.33E-05	1.70E-04
**12h**	9.40E-06	1.81E-04	4.03E-05	1.79E-05
**24h**	2.14E-05	7.44E-05	3.97E-05	2.32E-05
**36h**	2.21E-05	3.33E-05	2.05E-05	3.20E-05
**48h**	1.69E-05	4.07E-05	1.17E-05	7.44E-05
**72h**	2.63E-05	4.39E-05	8.60E-06	9.62E-05
	cgd7_930			
**2h**	9.29E-04	4.84E-05	5.06E-03	7.28E-04
**6h**	6.86E-05	6.09E-04	3.34E-04	2.98E-04
**12h**	7.35E-05	1.72E-03	1.47E-03	1.93E-04
**24h**	9.47E-04	2.82E-03	4.96E-03	8.91E-05
**36h**	2.42E-03	1.90E-03	4.44E-03	1.78E-04
**48h**	6.56E-04	1.85E-03	4.49E-03	9.64E-05
**72h**	1.08E-03	1.71E-03	2.37E-03	3.16E-04
	cgd7_1670			
**2h**	0.00E+00	1.89E-08	0.00E+00	8.61E-07
**6h**	2.44E-05	2.30E-05	5.53E-05	5.86E-05
**12h**	1.17E-04	2.64E-05	2.47E-05	3.51E-05
**24h**	3.95E-05	1.07E-05	3.52E-05	3.94E-05
**36h**	9.41E-05	1.80E-05	2.76E-05	3.02E-05
**48h**	4.86E-05	1.36E-05	2.16E-05	2.02E-05
**72h**	5.06E-05	6.56E-06	2.55E-05	1.46E-05
	cgd7_1680			
**2h**	5.07E-04	2.08E-05	3.37E-06	4.73E-05
**6h**	3.50E-04	7.61E-05	3.97E-05	1.77E-04
**12h**	2.72E-03	4.61E-04	1.42E-04	3.33E-04
**24h**	1.41E-03	3.20E-04	8.67E-05	2.64E-04
**36h**	4.43E-04	7.04E-05	7.77E-05	1.58E-04
**48h**	1.80E-03	3.84E-04	3.87E-04	2.03E-04
**72h**	3.94E-03	8.07E-04	8.25E-04	5.67E-04
	cgd7_1690			
**2h**	2.37E-08	3.05E-09	1.09E-08	3.19E-08
**6h**	0.00E+00	3.54E-09	2.26E-09	2.25E-09
**12h**	1.99E-07	4.97E-08	7.18E-08	9.79E-08
**24h**	1.76E-07	1.24E-07	3.69E-08	4.07E-08
**36h**	1.87E-07	3.42E-07	1.08E-07	1.96E-07
**48h**	1.76E-05	2.76E-06	8.68E-06	3.39E-06
**72h**	5.05E-05	1.07E-05	4.30E-05	2.40E-05
	cgd7_1700			
**2h**	1.67E-05	3.24E-07	3.80E-07	4.81E-06
**6h**	1.01E-05	1.52E-05	1.72E-06	5.58E-06
**12h**	1.90E-04	4.23E-05	1.73E-05	3.97E-05
**24h**	4.38E-05	1.87E-05	5.83E-06	7.03E-06
**36h**	6.39E-05	3.23E-05	2.13E-05	3.89E-05

**48h**	5.47E-05	1.99E-05	1.00E-05	8.80E-06
**72h**	2.89E-05	4.84E-06	7.43E-06	1.32E-05
	cgd7_1710			
**2h**	1.08E-06	2.11E-07	5.15E-08	1.06E-07
**6h**	4.87E-06	4.72E-06	8.98E-07	3.22E-06
**12h**	1.83E-06	5.98E-07	4.96E-07	6.42E-07
**24h**	1.44E-06	7.15E-07	2.01E-06	2.70E-06
**36h**	1.56E-06	1.28E-06	9.03E-07	1.03E-06
**48h**	1.00E-06	3.95E-07	7.01E-07	4.84E-07
**72h**	4.45E-07	4.65E-07	8.63E-07	8.03E-07
	cgd7_1720			
**2h**	2.79E-05	4.20E-07	3.76E-08	1.19E-06
**6h**	1.59E-07	7.88E-09	0.00E+00	5.42E-07
**12h**	2.63E-05	4.18E-06	1.79E-06	2.80E-06
**24h**	9.41E-06	3.23E-06	7.77E-07	2.71E-06
**36h**	1.20E-05	5.38E-06	1.89E-06	4.10E-06
**48h**	1.92E-05	9.15E-06	9.98E-06	4.21E-06
**72h**	1.84E-05	1.24E-05	1.02E-05	1.74E-05
	cgd7_1730			
**2h**	1.77E-08	2.79E-08	1.01E-08	4.67E-08
**6h**	0.00E+00	0.00E+00	0.00E+00	2.64E-09
**12h**	0.00E+00	0.00E+00	0.00E+00	4.13E-09
**24h**	1.50E-08	2.18E-08	1.69E-09	1.70E-08
**36h**	1.45E-08	4.72E-08	1.69E-07	2.01E-07
**48h**	6.16E-05	1.91E-05	3.99E-05	3.80E-05
**72h**	2.25E-05	2.36E-05	4.38E-05	2.42E-05
	cgd7_1750			
**2h**	6.06E-04	1.11E-05	3.50E-06	3.69E-05
**6h**	3.91E-04	1.27E-05	8.31E-05	1.11E-04
**12h**	2.28E-04	6.09E-05	2.60E-05	1.74E-05
**24h**	6.23E-04	1.97E-05	1.14E-05	4.27E-05
**36h**	4.92E-04	2.40E-05	7.99E-06	1.48E-05
**48h**	4.79E-05	3.91E-05	2.50E-05	1.37E-05
**72h**	1.42E-04	1.34E-05	8.72E-06	1.14E-05
	cgd7_1760			
**2h**	6.07E-07	5.92E-07	1.64E-08	9.88E-07
**6h**	3.55E-06	7.49E-07	6.22E-07	1.01E-06
**12h**	5.97E-07	2.12E-07	1.38E-07	1.13E-07
**24h**	1.59E-06	2.43E-07	5.53E-07	7.50E-07
**36h**	1.56E-06	9.91E-07	2.34E-07	3.20E-07
**48h**	4.63E-06	8.13E-07	1.04E-06	7.66E-07
**72h**	8.63E-07	4.33E-07	7.06E-07	6.08E-04
	cgd7_1770			
**2h**	9.20E-08	3.29E-08	0.00E+00	2.59E-07
**6h**	3.59E-07	2.27E-07	1.52E-07	2.83E-07
**12h**	1.18E-06	8.66E-07	5.32E-07	9.10E-07
**24h**	4.14E-07	2.46E-07	4.24E-07	1.02E-06
**36h**	8.35E-07	1.07E-06	4.00E-07	5.40E-07
**48h**	2.41E-05	3.61E-07	7.41E-07	5.15E-07
**72h**	2.78E-07	3.12E-07	2.06E-07	2.18E-07
	cgd7_1780			
**2h**	1.23E-05	1.94E-06	4.81E-07	1.39E-05
**6h**	5.54E-06	1.47E-06	9.60E-07	4.75E-06

**12h**	1.11E-06	7.12E-07	1.15E-06	2.73E-06
**24h**	3.28E-06	1.87E-06	2.39E-06	3.09E-06
**36h**	3.73E-06	3.12E-06	6.55E-07	6.09E-07
**48h**	3.65E-06	9.04E-07	5.29E-07	1.35E-06
**72h**	1.86E-06	5.59E-07	2.86E-07	3.86E-07
	cgd7_1790			
**2h**	8.59E-09	1.80E-06	2.50E-07	1.89E-05
**6h**	0.00E+00	2.28E-06	8.49E-07	5.13E-06
**12h**	1.38E-08	2.07E-07	6.15E-07	8.30E-06
**24h**	1.29E-07	7.13E-06	1.95E-06	1.24E-05
**36h**	6.49E-08	2.82E-06	1.70E-06	6.55E-06
**48h**	3.77E-06	1.31E-06	6.13E-07	2.29E-06
**72h**	3.11E-07	3.16E-07	5.65E-07	9.89E-07
	cgd7_940			
**2h**	7.19E-04	1.93E-04	2.26E-04	1.45E-03
**6h**	1.17E-03	6.46E-04	2.37E-03	1.09E-03
**12h**	6.47E-04	8.46E-04	1.12E-03	1.52E-03
**24h**	8.68E-04	7.24E-04	1.38E-03	7.80E-04
**36h**	5.58E-04	2.89E-04	3.16E-04	4.24E-04
**48h**	2.28E-04	9.67E-05	1.58E-04	4.67E-04
**72h**	2.47E-04	3.11E-05	4.39E-05	1.57E-04
	cgd7_950			
**2h**	5.13E-09	3.57E-09	0.00E+00	3.58E-09
**6h**	9.33E-05	2.20E-05	4.66E-05	2.01E-05
**12h**	2.05E-06	2.06E-06	1.53E-06	1.13E-06
**24h**	5.18E-06	8.24E-06	3.15E-05	7.43E-06
**36h**	5.82E-06	7.47E-06	5.71E-06	4.63E-06
**48h**	3.85E-06	1.46E-06	5.96E-06	3.63E-06
**72h**	2.67E-07	3.87E-07	4.03E-07	8.55E-07
	cgd7_960			
**2h**	0.00E+00	0.00E+00	0.00E+00	3.82E-07
**6h**	0.00E+00	1.09E-06	0.00E+00	4.40E-07
**12h**	8.90E-05	2.22E-04	2.28E-05	1.17E-05
**24h**	8.95E-06	1.11E-05	1.35E-05	7.92E-06
**36h**	4.30E-05	8.14E-06	5.10E-05	2.82E-05
**48h**	3.83E-05	1.22E-05	6.89E-05	1.36E-05
**72h**	1.04E-04	8.18E-06	1.47E-05	1.23E-05
	cgd7_970			
**2h**	4.79E-09	6.25E-09	1.06E-09	6.66E-08
**6h**	5.45E-07	5.72E-08	1.16E-07	1.14E-07
**12h**	4.31E-09	3.61E-08	1.18E-08	2.24E-08
**24h**	4.79E-08	4.74E-08	4.36E-08	4.93E-08
**36h**	4.82E-08	9.03E-08	5.34E-08	1.03E-07
**48h**	5.76E-08	2.27E-08	9.14E-08	4.18E-08
**72h**	7.30E-08	1.03E-07	9.76E-06	1.91E-07
	cgd7_980			
**2h**	0.00E+00	0.00E+00	0.00E+00	0.00E+00
**6h**	0.00E+00	0.00E+00	0.00E+00	1.12E-08
**12h**	9.25E-06	1.66E-05	1.15E-05	5.01E-06
**24h**	4.30E-06	4.89E-06	1.88E-06	6.77E-06
**36h**	1.59E-04	6.32E-06	8.68E-05	1.19E-05
**48h**	2.85E-05	4.40E-06	1.26E-04	1.78E-05
**72h**	4.88E-05	1.99E-05	3.09E-04	6.66E-05

	cgd7_1000			
**2h**	9.50E-08	2.76E-08	0.00E+00	2.86E-07
**6h**	8.70E-08	8.35E-09	4.31E-08	5.73E-08
**12h**	2.39E-07	1.71E-07	3.22E-07	2.77E-07
**24h**	3.12E-07	5.71E-07	3.87E-07	1.10E-07
**36h**	4.94E-07	2.60E-07	2.50E-07	3.36E-07
**48h**	2.08E-06	6.75E-07	1.44E-06	8.70E-07
**72h**	5.77E-06	4.61E-06	6.53E-06	8.86E-06
	cgd7_1020			
**2h**	1.52E-05	3.82E-05	8.30E-05	5.28E-04
**6h**	6.30E-05	1.20E-05	4.05E-05	3.83E-05
**12h**	4.09E-05	9.03E-06	1.61E-04	3.65E-05
**24h**	4.31E-05	2.80E-05	2.40E-05	9.72E-06
**36h**	3.95E-05	3.73E-05	1.39E-05	2.51E-05
**48h**	2.73E-05	1.18E-05	9.49E-05	1.15E-05
**72h**	1.20E-05	1.20E-05	1.35E-06	1.66E-05
	cgd7_1090			
**2h**	9.14E-07	0.00E+00	7.80E-07	0.00E+00
**6h**	3.77E-04	8.83E-05	8.59E-04	7.02E-04
**12h**	2.65E-04	1.55E-04	2.58E-04	1.57E-04
**24h**	2.20E-04	1.74E-04	7.75E-04	4.26E-04
**36h**	4.62E-04	5.45E-04	4.30E-04	7.24E-04
**48h**	2.88E-04	2.43E-04	3.37E-04	3.05E-04
**72h**	2.02E-04	8.72E-05	2.86E-04	1.68E-04
	cgd7_1110			
**2h**	6.33E-07	6.35E-07	1.35E-07	5.80E-07
**6h**	2.20E-06	6.18E-07	2.67E-06	1.65E-06
**12h**	1.54E-07	8.67E-07	8.00E-07	2.09E-07
**24h**	5.47E-07	8.21E-07	8.69E-07	4.47E-07
**36h**	5.63E-07	7.26E-07	6.09E-07	3.28E-07
**48h**	3.65E-07	4.13E-07	2.71E-07	6.04E-07
**72h**	1.41E-07	6.69E-08	1.32E-07	1.32E-07
	cgd7_1150			
**2h**	3.90E-07	2.90E-07	6.58E-08	5.01E-07
**6h**	5.17E-07	1.19E-07	4.64E-07	7.31E-07
**12h**	3.74E-08	2.19E-07	1.64E-07	8.75E-08
**24h**	2.01E-07	2.52E-07	1.97E-07	1.54E-07
**36h**	1.45E-07	2.04E-07	1.14E-07	1.23E-07
**48h**	2.13E-07	5.87E-07	2.65E-07	4.45E-07
**72h**	3.04E-07	2.21E-07	9.88E-07	2.86E-07
	cgd7_1160			
**2h**	2.66E-06	2.82E-06	2.21E-07	1.83E-06
**6h**	6.76E-07	2.07E-07	4.83E-07	1.61E-06
**12h**	5.32E-07	7.94E-08	8.03E-07	6.39E-07
**24h**	6.30E-07	9.75E-07	4.98E-07	8.85E-07
**36h**	8.94E-07	5.67E-07	9.09E-07	1.18E-06
**48h**	5.45E-06	4.72E-07	8.75E-07	1.74E-06
**72h**	2.08E-06	8.17E-07	4.00E-06	1.17E-05
	cgd7_1180			
**2h**	2.00E-05	1.12E-06	5.83E-06	6.00E-06
**6h**	1.87E-06	1.03E-06	6.09E-06	1.31E-06
**12h**	1.55E-06	4.92E-06	2.32E-05	1.41E-06
**24h**	4.57E-06	4.30E-06	3.59E-06	1.27E-06

**36h**	1.81E-06	1.33E-06	1.45E-06	2.12E-06
**48h**	1.20E-06	6.90E-07	1.29E-06	9.33E-07
**72h**	9.62E-07	7.90E-07	1.01E-06	7.20E-07
	cgd7_1190			
**2h**	2.45E-07	2.15E-08	1.19E-07	7.02E-08
**6h**	3.17E-09	0.00E+00	0.00E+00	1.99E-09
**12h**	1.12E-05	3.35E-05	2.82E-05	1.22E-05
**24h**	2.65E-06	6.89E-06	1.06E-06	8.08E-07
**36h**	3.94E-07	3.90E-07	3.16E-06	2.06E-06
**48h**	3.17E-07	1.54E-07	1.87E-07	3.43E-07
**72h**	1.05E-07	1.33E-07	1.51E-07	7.93E-08
	cgd7_1200			
**2h**	2.27E-07	3.28E-09	1.51E-08	1.52E-08
**6h**	0.00E+00	3.42E-09	0.00E+00	1.01E-08
**12h**	8.02E-06	2.72E-05	9.20E-06	5.29E-06
**24h**	3.23E-06	5.62E-06	1.84E-06	7.77E-07
**36h**	1.85E-06	1.66E-06	3.99E-06	2.81E-06
**48h**	1.20E-06	4.62E-07	1.45E-06	1.20E-06
**72h**	1.02E-06	7.23E-07	1.52E-06	9.78E-07
	cgd7_1220			
**2h**	8.66E-06	9.80E-07	2.49E-06	3.53E-06
**6h**	9.16E-07	6.49E-07	7.88E-07	5.13E-07
**12h**	3.58E-06	6.86E-06	7.45E-06	3.75E-06
**24h**	5.82E-06	5.88E-06	3.90E-06	1.43E-06
**36h**	2.09E-06	9.20E-07	1.32E-06	2.12E-06
**48h**	4.50E-06	1.86E-06	1.28E-06	2.10E-05
**72h**	1.21E-06	3.34E-07	3.59E-07	1.03E-06
	cgd7_1230			
**2h**	5.10E-06	1.43E-06	1.91E-06	3.33E-06
**6h**	1.71E-06	4.99E-07	1.20E-07	6.12E-07
**12h**	3.61E-07	1.47E-06	1.81E-06	1.14E-06
**24h**	4.48E-06	1.56E-06	6.59E-07	6.89E-07
**36h**	7.57E-07	6.52E-07	4.11E-07	7.88E-07
**48h**	2.07E-07	2.00E-07	1.73E-07	3.20E-07
**72h**	3.86E-07	1.96E-07	4.50E-07	4.62E-07
	cgd7_1240			
**2h**	4.69E-07	1.82E-09	0.00E+00	1.03E-07
**6h**	0.00E+00	0.00E+00	0.00E+00	6.73E-10
**12h**	4.21E-08	9.55E-08	2.46E-07	3.05E-08
**24h**	1.48E-07	2.53E-07	3.03E-08	5.42E-08
**36h**	1.53E-07	1.37E-07	1.04E-07	1.05E-07
**48h**	1.60E-06	6.15E-07	6.50E-07	5.97E-07
**72h**	4.72E-06	1.72E-06	4.17E-06	2.56E-06
	cgd7_1250			
**2h**	7.49E-08	2.07E-08	0.00E+00	3.49E-07
**6h**	0.00E+00	5.83E-09	2.27E-08	2.92E-09
**12h**	1.71E-07	5.22E-07	2.94E-07	1.10E-07
**24h**	1.75E-07	9.16E-07	8.26E-08	5.10E-08
**36h**	2.19E-07	1.49E-07	2.35E-07	4.42E-07
**48h**	2.03E-06	1.53E-06	5.35E-07	1.99E-06
**72h**	2.47E-06	7.35E-07	3.65E-06	2.36E-06
	cgd7_1260			
**2h**	2.01E-06	7.96E-08	4.86E-08	5.14E-07

**6h**	0.00E+00	2.43E-07	1.45E-07	1.13E-07
**12h**	1.77E-05	3.07E-05	8.88E-06	9.20E-06
**24h**	2.87E-06	8.88E-06	2.20E-06	6.46E-07
**36h**	8.93E-06	3.35E-06	1.38E-05	1.50E-05
**48h**	2.38E-05	4.68E-06	5.67E-06	4.44E-06
**72h**	1.32E-05	3.35E-06	7.33E-06	7.37E-06
	cgd7_1270			
**2h**	0.00E+00	0.00E+00	0.00E+00	1.56E-09
**6h**	5.50E-09	0.00E+00	7.92E-09	0.00E+00
**12h**	6.22E-07	2.53E-06	7.12E-07	4.38E-07
**24h**	2.89E-07	4.61E-07	7.92E-07	2.59E-07
**36h**	1.08E-06	6.82E-07	1.21E-06	1.13E-06
**48h**	5.35E-07	2.27E-07	3.48E-07	3.25E-07
**72h**	3.91E-07	4.40E-07	1.23E-06	4.01E-07
	cgd7_1280			
**2h**	8.98E-06	1.45E-06	7.73E-07	1.97E-06
**6h**	3.00E-05	1.23E-04	2.61E-04	6.52E-05
**12h**	5.56E-04	9.51E-04	2.89E-04	1.13E-04
**24h**	1.43E-04	1.57E-04	1.80E-04	7.91E-05
**36h**	1.31E-04	9.04E-05	1.73E-04	1.34E-04
**48h**	1.79E-03	5.91E-05	7.50E-05	6.54E-05
**72h**	7.10E-06	5.20E-06	4.36E-06	4.53E-06
	cgd7_1290			
**2h**	0.00E+00	0.00E+00	0.00E+00	0.00E+00
**6h**	0.00E+00	3.64E-07	0.00E+00	0.00E+00
**12h**	0.00E+00	0.00E+00	3.06E-06	1.26E-06
**24h**	1.65E-05	1.04E-05	2.96E-05	5.10E-06
**36h**	7.47E-05	1.45E-04	8.05E-05	6.43E-05
**48h**	1.48E-03	2.22E-03	3.68E-03	5.20E-03
**72h**	6.75E-05	2.74E-04	2.78E-04	9.00E-05
	cgd7_1300			
**2h**	2.07E-08	2.63E-08	5.40E-09	0.00E+00
**6h**	8.24E-08	2.00E-07	1.81E-07	2.51E-07
**12h**	4.12E-07	4.69E-07	5.61E-07	7.35E-07
**24h**	2.74E-07	2.91E-04	4.13E-07	2.84E-07
**36h**	5.55E-07	5.83E-07	4.65E-07	9.19E-07
**48h**	2.98E-07	1.60E-07	1.11E-07	4.24E-07
**72h**	1.83E-07	1.83E-07	2.58E-07	1.35E-07
	cgd7_1320			
**2h**	0.00E+00	8.89E-06	5.92E-07	8.34E-06
**6h**	2.57E-05	1.50E-06	5.63E-06	3.68E-06
**12h**	7.70E-06	2.18E-06	3.04E-05	2.26E-06
**24h**	3.04E-05	1.06E-06	5.21E-06	6.69E-06
**36h**	1.38E-05	7.37E-07	1.38E-05	5.86E-06
**48h**	5.33E-05	2.04E-06	9.20E-05	3.92E-06
**72h**	4.25E-05	2.39E-06	1.16E-05	7.32E-06
	cgd7_1350			
**2h**	8.21E-09	2.38E-09	0.00E+00	2.45E-08
**6h**	1.02E-08	0.00E+00	0.00E+00	1.53E-08
**12h**	4.51E-07	4.65E-07	1.65E-07	1.89E-07
**24h**	2.72E-07	1.05E-07	1.77E-07	7.36E-08
**36h**	7.17E-07	1.49E-07	9.30E-07	4.06E-07
**48h**	1.73E-06	5.32E-07	1.64E-06	4.51E-07

**72h**	4.91E-07	2.02E-07	4.80E-07	2.75E-07
	cgd7_1370			
**2h**	2.17E-04	4.81E-06	5.36E-06	7.30E-05
**6h**	1.86E-04	2.16E-05	1.83E-04	1.15E-04
**12h**	1.17E-03	7.58E-05	5.17E-04	2.06E-04
**24h**	4.20E-04	4.93E-05	3.06E-04	1.85E-04
**36h**	1.40E-04	1.19E-05	3.37E-04	6.36E-05
**48h**	3.97E-04	7.76E-06	1.68E-04	6.66E-05
**72h**	1.39E-04	4.75E-06	1.22E-04	3.80E-05
	cgd7_1380			
**2h**	0.00E+00	1.31E-06	2.20E-07	4.33E-06
**6h**	8.77E-06	3.92E-06	7.47E-06	1.28E-05
**12h**	1.89E-05	2.96E-06	9.42E-06	7.32E-06
**24h**	7.45E-06	1.29E-06	8.04E-06	2.91E-06
**36h**	4.33E-06	1.77E-06	3.09E-06	3.24E-06
**48h**	2.84E-06	1.50E-07	2.02E-06	1.09E-06
**72h**	1.53E-06	6.25E-07	1.90E-06	7.69E-07
	cgd7_1390			
**2h**	2.66E-07	3.63E-07	4.50E-07	1.63E-06
**6h**	1.94E-06	1.01E-06	1.16E-06	5.16E-06
**12h**	6.87E-06	4.12E-06	7.82E-06	1.06E-05
**24h**	4.56E-06	9.17E-07	2.84E-06	1.58E-06
**36h**	1.63E-06	7.57E-07	2.75E-06	2.89E-06
**48h**	1.26E-06	1.55E-07	8.83E-07	8.70E-07
**72h**	1.04E-06	3.07E-07	5.65E-07	8.41E-07
	cgd7_1400			
**2h**	2.04E-06	2.97E-07	7.47E-08	3.36E-06
**6h**	1.73E-06	4.84E-07	1.46E-06	2.39E-06
**12h**	7.36E-06	2.92E-06	7.88E-06	6.78E-06
**24h**	3.68E-06	1.18E-06	3.34E-06	2.82E-06
**36h**	4.63E-06	2.29E-06	7.30E-06	5.25E-06
**48h**	2.11E-06	6.40E-07	3.07E-06	2.32E-06
**72h**	7.43E-07	6.31E-07	7.03E-07	6.43E-07
	cgd7_1410			
**2h**	1.01E-06	3.78E-07	3.79E-07	3.94E-06
**6h**	7.42E-07	2.30E-07	3.83E-07	1.22E-06
**12h**	7.20E-07	4.34E-07	1.48E-06	9.93E-07
**24h**	7.63E-07	3.38E-07	6.00E-07	6.37E-07
**36h**	4.39E-07	2.41E-07	2.65E-07	2.87E-07
**48h**	3.38E-07	4.53E-08	2.33E-07	1.47E-07
**72h**	3.05E-06	3.83E-06	7.97E-06	1.00E-05
	cgd7_1420			
**2h**	7.09E-03	1.54E-03	7.84E-03	2.36E-02
**6h**	1.33E-02	4.84E-04	9.32E-03	9.53E-03
**12h**	1.83E-02	4.86E-03	2.15E-02	5.95E-03
**24h**	2.20E-03	7.57E-04	5.97E-03	4.95E-03
**36h**	1.51E-04	9.39E-04	6.18E-03	2.25E-03
**48h**	1.31E-03	2.06E-04	1.14E-03	2.51E-03
**72h**	8.95E-04	2.53E-04	3.79E-03	5.47E-04
	cgd7_1430			
**2h**	1.00E-04	8.80E-06	2.33E-07	2.40E-05
**6h**	1.17E-03	1.05E-04	7.24E-04	8.26E-04
**12h**	3.34E-04	1.65E-05	7.06E-05	7.14E-05

**24h**	4.51E-04	4.59E-05	7.34E-04	3.44E-04
**36h**	8.97E-04	1.16E-04	5.63E-04	2.78E-04
**48h**	8.88E-04	8.08E-05	5.90E-04	2.84E-04
**72h**	6.54E-04	3.08E-05	1.86E-04	8.93E-05
	cgd7_1440			
**2h**	1.88E-07	6.04E-08	2.45E-08	1.09E-07
**6h**	7.65E-08	3.52E-08	7.25E-08	1.68E-07
**12h**	9.27E-08	3.06E-09	1.16E-07	1.08E-07
**24h**	1.46E-07	7.11E-08	8.16E-07	7.34E-07
**36h**	8.14E-07	2.07E-07	5.58E-07	1.76E-07
**48h**	1.59E-06	4.79E-07	2.01E-06	1.25E-06
**72h**	7.66E-07	3.37E-07	7.51E-07	1.93E-07
	cgd7_1450			
**2h**	0.00E+00	2.19E-06	2.12E-07	2.52E-06
**6h**	7.44E-07	5.30E-07	8.29E-07	2.40E-06
**12h**	4.12E-07	1.79E-07	1.79E-06	1.12E-06
**24h**	9.77E-07	6.12E-07	2.19E-06	1.40E-06
**36h**	1.07E-06	1.43E-06	9.88E-07	6.13E-07
**48h**	1.18E-06	5.09E-07	1.09E-06	7.60E-07
**72h**	1.65E-06	4.15E-07	1.09E-06	6.63E-07
	cgd7_1460			
**2h**	0.00E+00	0.00E+00	0.00E+00	4.08E-04
**6h**	6.40E-04	1.16E-04	4.72E-04	1.62E-03
**12h**	8.84E-05	3.86E-05	2.69E-04	1.19E-04
**24h**	4.39E-04	3.21E-02	4.54E-04	2.33E-04
**36h**	8.67E-04	7.40E-05	1.27E-04	1.12E-04
**48h**	1.06E-04	7.15E-06	1.58E-04	4.81E-05
**72h**	1.40E-04	1.05E-05	4.34E-05	3.42E-05
	cgd7_4070			
**2h**	3.16E-08	2.81E-08	5.22E-09	2.71E-09
**6h**	2.59E-06	2.60E-06	2.77E-06	1.92E-06
**12h**	1.64E-07	3.78E-07	6.30E-08	1.26E-07
**24h**	2.65E-07	6.12E-07	2.28E-06	6.16E-07
**36h**	5.76E-07	9.11E-07	5.58E-07	1.07E-06
**48h**	2.63E-07	5.04E-07	6.15E-07	5.35E-07
**72h**	1.19E-07	2.22E-07	8.08E-08	3.39E-07
	cgd7_4080			
**2h**	1.44E-07	1.43E-07	1.54E-09	4.81E-08
**6h**	1.33E-05	2.82E-05	2.66E-05	1.25E-05
**12h**	7.43E-07	1.28E-05	1.50E-06	1.16E-06
**24h**	1.65E-06	1.08E-05	3.19E-05	1.19E-05
**36h**	6.11E-06	1.74E-05	9.12E-06	1.34E-05
**48h**	5.28E-06	9.75E-06	6.92E-06	8.98E-06
**72h**	1.02E-06	1.74E-06	2.54E-06	3.78E-06
	cgd7_4100			
**2h**	5.06E-08	4.36E-06	0.00E+00	4.94E-07
**6h**	5.46E-05	9.62E-04	3.38E-04	1.66E-04
**12h**	5.96E-05	1.32E-03	1.17E-04	8.78E-05
**24h**	2.80E-05	3.59E-04	4.08E-04	2.37E-04
**36h**	1.18E-04	6.58E-04	3.99E-04	6.48E-04
**48h**	7.19E-05	3.88E-04	2.49E-04	3.22E-04
**72h**	2.13E-05	1.24E-04	4.87E-05	5.59E-05
	cgd7_4110			

**2h**	5.08E-06	1.40E-06	3.60E-06	1.81E-06
**6h**	5.76E-07	2.30E-06	1.29E-07	9.84E-07
**12h**	1.51E-06	2.15E-06	2.50E-06	1.42E-06
**24h**	1.50E-06	5.92E-06	3.44E-06	1.20E-06
**36h**	3.30E-06	1.84E-06	1.67E-06	1.13E-06
**48h**	7.83E-07	4.29E-07	8.04E-07	8.68E-07
**72h**	2.19E-07	6.22E-07	1.12E-06	7.49E-07
	cgd7_4120			
**2h**	2.24E-07	2.08E-08	9.27E-09	1.96E-07
**6h**	0.00E+00	7.93E-08	8.23E-09	1.38E-09
**12h**	3.89E-07	1.28E-06	7.57E-07	7.10E-07
**24h**	5.84E-08	8.36E-07	2.47E-07	9.22E-08
**36h**	3.28E-07	3.94E-07	1.50E-06	1.08E-06
**48h**	2.07E-07	1.75E-07	1.31E-07	2.72E-07
**72h**	1.26E-07	1.57E-07	1.24E-07	1.35E-07
	cgd7_4130			
**2h**	4.88E-07	2.68E-07	2.87E-07	2.46E-07
**6h**	1.11E-07	2.12E-07	1.64E-07	1.57E-07
**12h**	4.82E-07	3.38E-07	4.45E-07	2.89E-07
**24h**	1.73E-07	2.07E-06	5.43E-07	3.05E-07
**36h**	5.24E-07	9.04E-07	6.00E-07	6.10E-07
**48h**	1.04E-07	3.93E-07	3.84E-07	2.74E-07
**72h**	9.18E-08	3.22E-07	3.40E-07	6.30E-07
	cgd7_4140			
**2h**	1.10E-05	3.31E-05	1.83E-05	7.48E-05
**6h**	5.85E-06	1.01E-04	3.94E-05	4.40E-05
**12h**	1.24E-06	1.98E-06	8.61E-06	8.23E-06
**24h**	1.05E-06	4.00E-05	6.89E-05	4.26E-05
**36h**	1.78E-05	7.62E-05	4.28E-05	3.40E-05
**48h**	1.04E-05	6.82E-05	4.07E-05	6.80E-05
**72h**	4.43E-06	1.38E-05	1.63E-05	1.57E-04
	cgd7_4150			
**2h**	9.34E-07	0.00E+00	4.13E-06	1.79E-06
**6h**	1.20E-05	5.69E-04	4.35E-04	1.90E-04
**12h**	7.85E-06	9.07E-05	1.42E-05	2.63E-05
**24h**	6.61E-06	1.77E-04	3.22E-04	1.16E-04
**36h**	1.63E-04	2.94E-04	2.12E-04	2.52E-04
**48h**	7.85E-05	1.89E-04	3.69E-04	3.62E-04
**72h**	1.83E-05	7.67E-05	3.35E-05	1.03E-04
	cgd7_4160			
**2h**	1.35E-08	6.41E-09	0.00E+00	6.76E-09
**6h**	4.16E-07	2.12E-06	2.65E-06	8.68E-07
**12h**	4.00E-07	7.93E-07	3.47E-07	2.20E-07
**24h**	1.46E-07	7.50E-07	3.77E-06	1.31E-06
**36h**	2.23E-06	1.75E-06	3.24E-06	2.27E-06
**48h**	1.12E-06	9.60E-07	2.69E-06	1.47E-06
**72h**	8.46E-08	4.49E-07	2.03E-07	3.12E-07
	cgd7_4170			
**2h**	0.00E+00	0.00E+00	0.00E+00	2.88E-08
**6h**	0.00E+00	3.64E-08	7.04E-07	8.04E-08
**12h**	2.33E-06	1.97E-05	3.32E-06	2.58E-06
**24h**	8.40E-07	7.33E-06	8.37E-06	2.94E-06
**36h**	4.68E-06	1.57E-05	1.93E-05	2.07E-05

**48h**	8.16E-06	8.08E-06	1.29E-05	1.61E-05
**72h**	1.43E-06	5.77E-06	1.44E-06	5.16E-06
	cgd7_4180			
**2h**	2.51E-08	2.72E-08	3.11E-09	8.92E-08
**6h**	2.46E-07	1.05E-06	2.00E-06	1.39E-06
**12h**	2.12E-07	2.66E-08	1.49E-07	1.07E-07
**24h**	2.05E-07	4.54E-07	1.58E-06	5.96E-07
**36h**	1.31E-06	1.73E-06	1.69E-06	1.28E-06
**48h**	2.04E-06	1.05E-06	1.58E-06	1.84E-06
**72h**	2.14E-07	4.22E-07	5.42E-07	5.25E-07
	cgd7_4190			
**2h**	0.00E+00	8.75E-07	2.90E-06	4.23E-06
**6h**	2.36E-06	4.98E-06	8.92E-06	4.47E-06
**12h**	8.43E-07	5.60E-07	2.54E-06	2.63E-06
**24h**	1.48E-06	1.87E-06	8.66E-06	3.56E-06
**36h**	1.02E-05	5.68E-06	3.61E-06	2.57E-06
**48h**	4.88E-06	1.23E-06	2.00E-06	3.31E-06
**72h**	3.61E-07	9.06E-07	1.99E-07	3.52E-07
	cgd7_4200			
**2h**	1.25E-05	2.91E-05	1.70E-05	3.44E-05
**6h**	9.01E-05	1.07E-05	4.58E-05	7.11E-06
**12h**	1.20E-06	2.55E-07	1.00E-05	6.49E-06
**24h**	3.64E-05	3.57E-06	2.16E-05	7.21E-06
**36h**	1.69E-05	3.91E-06	6.48E-06	8.22E-06
**48h**	7.98E-06	3.32E-06	9.01E-06	7.07E-06
**72h**	6.78E-06	6.68E-07	3.14E-06	6.38E-06
	cgd7_4210			
**2h**	0.00E+00	2.38E-08	0.00E+00	0.00E+00
**6h**	2.47E-05	8.00E-06	2.49E-05	2.49E-06
**12h**	8.66E-07	2.48E-06	1.11E-06	3.20E-07
**24h**	2.19E-06	1.46E-06	9.15E-06	3.73E-06
**36h**	5.55E-06	2.92E-06	3.17E-06	3.19E-06
**48h**	2.72E-06	1.55E-06	2.26E-06	1.79E-06
**72h**	9.06E-07	4.28E-07	6.87E-07	1.18E-06
	cgd7_4220			
**2h**	1.80E-06	6.21E-07	6.73E-07	1.18E-06
**6h**	3.22E-05	1.14E-05	2.92E-05	1.75E-05
**12h**	4.80E-07	1.97E-07	5.29E-06	4.44E-07
**24h**	2.96E-06	4.83E-06	1.27E-05	5.96E-06
**36h**	4.04E-06	4.09E-06	3.43E-06	4.22E-06
**48h**	2.81E-06	1.18E-06	2.94E-06	7.11E-07
**72h**	6.96E-07	3.28E-07	7.14E-07	5.96E-07
	cgd7_4230			
**2h**	1.70E-07	4.36E-07	7.88E-08	1.85E-07
**6h**	2.92E-06	2.14E-06	6.09E-06	1.27E-06
**12h**	1.86E-07	7.97E-07	3.51E-07	2.12E-07
**24h**	6.59E-07	6.62E-07	3.44E-06	8.63E-07
**36h**	1.17E-06	2.87E-06	1.42E-06	2.49E-06
**48h**	1.90E-06	8.67E-07	1.23E-05	9.40E-07
**72h**	1.10E-06	6.44E-07	7.02E-07	7.26E-07
	cgd7_4240			
**2h**	2.56E-07	1.12E-07	0.00E+00	1.90E-07
**6h**	1.06E-06	8.97E-07	3.59E-06	5.89E-07

**12h**	6.53E-08	1.62E-07	3.22E-07	6.02E-08
**24h**	1.75E-07	2.27E-07	1.53E-06	6.64E-07
**36h**	4.19E-07	7.62E-07	6.77E-07	1.10E-06
**48h**	4.08E-07	2.01E-07	2.51E-07	3.58E-07
**72h**	2.72E-07	1.35E-07	3.45E-07	8.05E-07
	cgd7_4250			
**2h**	2.80E-06	6.90E-07	1.80E-06	4.72E-06
**6h**	2.95E-06	8.88E-07	3.44E-06	6.47E-07
**12h**	1.61E-07	1.39E-07	1.45E-06	5.00E-07
**24h**	1.21E-06	8.49E-07	1.77E-06	2.09E-06
**36h**	1.73E-06	1.49E-06	1.56E-06	1.96E-06
**48h**	1.59E-06	2.76E-07	1.62E-06	9.42E-07
**72h**	4.76E-07	3.70E-07	5.89E-07	7.03E-07
	cgd7_4260			
**2h**	1.44E-02	1.82E-03	1.02E-02	5.46E-03
**6h**	1.17E-02	2.77E-03	3.76E-03	6.20E-03
**12h**	1.15E-03	1.29E-05	1.95E-03	8.99E-04
**24h**	2.00E-03	6.61E-04	3.65E-03	1.19E-04
**36h**	1.41E-03	8.69E-04	2.04E-03	6.22E-04
**48h**	5.16E-04	2.14E-04	7.33E-04	1.53E-04
**72h**	2.41E-04	1.45E-04	1.66E-03	3.74E-04
	cgd7_4280			
**2h**	1.02E-07	5.91E-07	2.90E-07	1.23E-06
**6h**	4.09E-08	1.58E-08	1.07E-07	2.38E-08
**12h**	2.81E-04	3.89E-04	4.45E-04	5.40E-04
**24h**	5.58E-05	4.34E-05	6.61E-05	1.45E-05
**36h**	7.22E-05	7.38E-05	1.62E-04	3.28E-04
**48h**	4.33E-05	2.75E-05	3.50E-05	2.97E-05
**72h**	3.88E-06	7.82E-07	2.36E-06	2.44E-06
	cgd7_4290			
**2h**	1.25E-06	3.89E-06	6.00E-07	1.41E-06
**6h**	6.35E-07	3.95E-07	6.57E-07	2.65E-07
**12h**	2.59E-06	1.66E-06	3.57E-06	1.71E-06
**24h**	1.50E-06	2.20E-06	1.87E-06	5.49E-07
**36h**	8.49E-07	8.54E-07	1.04E-06	1.38E-06
**48h**	3.18E-07	2.12E-07	5.07E-07	6.44E-07
**72h**	1.60E-07	8.50E-08	1.28E-07	1.46E-07
	cgd7_4300			
**2h**	1.24E-04	4.36E-06	2.50E-05	3.67E-05
**6h**	4.87E-06	0.00E+00	7.19E-06	2.16E-07
**12h**	2.41E-02	2.21E-03	3.33E-02	4.23E-03
**24h**	1.85E-03	7.69E-04	9.03E-04	1.00E-03
**36h**	3.97E-04	6.76E-05	1.98E-03	1.88E-03
**48h**	4.63E-04	2.11E-05	5.60E-04	2.21E-04
**72h**	2.45E-04	3.98E-06	2.14E-04	8.31E-05
	cgd7_4310			
**2h**	1.82E-04	4.24E-03	1.26E-03	1.22E-01
**6h**	4.23E-04	2.21E-04	9.78E-05	1.56E-06
**12h**	1.66E-03	8.15E-04	1.39E-03	3.00E-04
**24h**	8.35E-04	5.88E-04	3.81E-04	1.68E-04
**36h**	2.66E-04	2.15E-03	3.52E-04	2.34E-04
**48h**	2.04E-03	9.48E-03	5.52E-03	2.33E-03
**72h**	7.64E-04	1.72E-04	7.95E-04	5.20E-04

	cgd7_4320			
**2h**	3.89E-05	1.93E-05	3.98E-05	2.74E-05
**6h**	2.21E-05	6.52E-06	3.28E-05	1.03E-05
**12h**	2.33E-05	9.25E-06	1.35E-04	2.90E-05
**24h**	7.72E+01	2.26E-05	9.82E-05	1.97E-05
**36h**	2.07E-05	1.16E-05	9.07E-05	2.23E-05
**48h**	6.00E-06	2.22E-06	4.83E-05	1.22E-05
**72h**	1.39E-05	4.97E-07	2.48E-05	2.51E-06
	cgd7_4330			
**2h**	9.74E-07	8.72E-07	1.03E-06	1.13E-06
**6h**	1.67E-09	1.89E-07	9.77E-08	3.48E-08
**12h**	2.43E-05	5.38E-05	2.83E-05	8.90E-06
**24h**	1.32E-05	2.05E-05	7.45E-06	2.41E-06
**36h**	4.51E-06	2.86E-06	1.34E-05	7.84E-06
**48h**	3.31E-06	1.61E-06	5.44E-06	4.61E-07
**72h**	7.18E-07	5.61E-07	6.06E-07	4.63E-07
	cgd7_4340			
**2h**	0.00E+00	0.00E+00	0.00E+00	0.00E+00
**6h**	0.00E+00	0.00E+00	5.77E-09	0.00E+00
**12h**	1.79E-06	1.75E-06	3.76E-06	3.32E-07
**24h**	6.69E-07	1.39E-06	6.14E-07	1.14E-07
**36h**	1.93E-06	9.34E-07	1.93E-06	7.52E-07
**48h**	1.15E-06	4.05E-07	1.29E-06	1.30E-07
**72h**	6.35E-08	7.45E-08	8.42E-08	2.86E-08
	cgd7_4350			
**2h**	0.00E+00	0.00E+00	0.00E+00	9.82E-07
**6h**	0.00E+00	0.00E+00	0.00E+00	0.00E+00
**12h**	3.69E-03	6.12E-03	6.44E-03	6.50E-04
**24h**	9.88E-04	1.69E-03	1.34E-03	2.69E-04
**36h**	6.63E-05	1.08E-03	1.85E-03	9.60E-04
**48h**	2.05E-04	1.70E-04	1.42E-03	9.59E-05
**72h**	1.35E-04	1.96E-04	1.04E-04	1.90E-04
	cgd7_4370			
**2h**	1.58E-05	7.36E-06	9.43E-06	4.30E-06
**6h**	2.51E-05	2.03E-05	3.53E-05	2.87E-06
**12h**	3.84E-06	0.00E+00	1.70E-05	1.73E-06
**24h**	1.09E-05	3.00E-05	3.27E-05	1.22E-05
**36h**	8.78E-05	4.82E-05	5.94E-05	6.60E-06
**48h**	3.14E-05	8.47E-06	4.30E-05	3.21E-06
**72h**	2.46E-05	7.84E-06	8.22E-06	4.96E-06
	cgd7_4380			
**2h**	1.57E-04	1.70E-04	2.77E-05	2.69E-04
**6h**	1.20E-03	1.26E-03	1.19E-03	2.72E-04
**12h**	1.28E-04	8.78E-04	8.88E-04	3.19E-05
**24h**	2.92E-04	4.76E-04	1.57E-03	1.72E-03
**36h**	9.29E-04	5.88E-04	5.48E-04	2.57E-04
**48h**	5.48E-04	1.96E-04	3.74E-04	5.25E-05
**72h**	9.82E-05	1.04E-04	6.46E-05	1.25E-04
	cgd7_4390			
**2h**	7.49E-06	3.73E-06	1.42E-06	1.21E-06
**6h**	1.43E-07	5.83E-07	4.45E-05	7.96E-07
**12h**	2.59E-07	7.58E-07	3.96E-06	5.64E-07
**24h**	3.44E-07	2.08E-06	6.54E-06	1.04E-06

**36h**	1.68E-05	1.53E-05	7.45E-06	2.17E-06
**48h**	2.56E-05	8.81E-06	1.84E-05	2.28E-06
**72h**	1.09E-05	1.23E-05	1.87E-05	3.20E-06
	cgd7_4400			
**2h**	4.30E-05	1.80E-05	2.40E-06	1.59E-05
**6h**	2.75E-06	1.00E-05	0.00E+00	1.73E-06
**12h**	3.15E-04	1.23E-03	4.74E-04	1.23E-04
**24h**	2.56E-04	5.94E-04	1.99E-04	6.63E-05
**36h**	6.87E-04	2.89E-04	5.66E-04	3.88E-04
**48h**	1.66E-03	3.94E-04	5.56E-04	1.12E-04
**72h**	6.54E-04	1.95E-04	1.70E-04	2.35E-04
	cgd7_4420			
**2h**	3.79E-05	2.76E-05	1.91E-05	7.46E-05
**6h**	2.08E-04	5.34E-04	3.17E-04	6.03E-05
**12h**	8.42E-05	2.27E-04	1.39E-04	7.68E-05
**24h**	8.60E-05	1.70E-04	1.78E-04	4.18E-05
**36h**	3.20E-04	1.57E-04	1.39E-04	1.38E-04
**48h**	3.16E-04	6.87E-05	1.37E-04	3.03E-05
**72h**	3.70E-05	2.08E-05	3.66E-05	3.74E-05
	cgd7_4440			
**2h**	1.74E-06	9.83E-07	4.22E-07	1.03E-06
**6h**	3.37E-05	7.36E-05	6.68E-05	9.86E-06
**12h**	6.96E-07	1.66E-06	2.41E-06	9.42E-07
**24h**	1.04E-05	1.08E-05	2.49E-05	4.86E-06
**36h**	8.54E-06	6.34E-06	3.61E-06	3.53E-06
**48h**	1.11E-05	5.22E-06	4.84E-06	1.12E-06
**72h**	8.15E-07	1.08E-06	4.09E-07	2.18E-05
	cgd7_4470			
**2h**	7.08E-05	2.59E-05	0.00E+00	7.77E-06
**6h**	6.50E-06	0.00E+00	0.00E+00	5.78E-07
**12h**	8.55E-03	2.07E-02	6.99E-03	1.00E-03
**24h**	1.51E-03	7.56E-04	1.18E-03	2.27E-04
**36h**	1.14E-03	4.52E-04	9.29E-04	5.23E-04
**48h**	1.03E-04	5.37E-04	1.07E-03	3.21E-05
**72h**	2.59E-04	3.56E-04	5.39E-04	1.05E-04
	cgd7_4480			
**2h**	3.33E-04	1.52E-04	2.15E-04	1.35E-03
**6h**	1.31E-04	2.56E-04	1.89E-04	1.59E-04
**12h**	6.42E-05	2.40E-04	2.90E-04	8.28E-05
**24h**	2.16E-04	3.81E-04	2.13E-04	1.19E-04
**36h**	1.68E-04	1.18E-04	1.07E-04	4.55E-05
**48h**	1.97E-04	5.69E-05	1.39E-04	2.97E-05
**72h**	4.84E-05	1.75E-05	2.64E-05	2.80E-05
	cgd7_4490			
**2h**	0.00E+00	0.00E+00	0.00E+00	0.00E+00
**6h**	0.00E+00	0.00E+00	4.88E-08	0.00E+00
**12h**	3.19E-06	1.26E-05	7.99E-06	3.29E-06
**24h**	4.22E-06	4.27E-06	4.23E-06	1.37E-06
**36h**	2.75E-05	2.00E-05	2.65E-05	2.46E-05
**48h**	7.33E-06	6.92E-06	1.08E-05	7.46E-06
**72h**	5.45E-06	4.44E-06	4.49E-06	2.90E-06
	cgd7_1800			
**2h**	1.06E-07	6.37E-08	3.03E-08	2.54E-08

**6h**	9.76E-09	9.90E-08	0.00E+00	1.86E-08
**12h**	1.05E-08	1.70E-08	5.76E-08	2.74E-08
**24h**	6.89E-09	2.00E-08	8.10E-09	1.24E-08
**36h**	1.44E-07	5.70E-08	6.98E-08	5.38E-08
**48h**	1.87E-06	1.70E-06	6.57E-07	7.00E-07
**72h**	1.77E-07	1.98E-07	4.94E-07	1.31E-07
	cgd7_1810			
**2h**	0.00E+00	0.00E+00	0.00E+00	0.00E+00
**6h**	3.68E-07	1.83E-07	2.74E-07	3.84E-07
**12h**	3.37E-08	6.74E-08	1.48E-08	2.50E-08
**24h**	5.30E-08	2.54E-07	9.87E-08	5.23E-07
**36h**	3.02E-07	5.25E-07	1.45E-07	2.94E-07
**48h**	6.74E-07	6.45E-07	2.00E-07	1.89E-07
**72h**	1.16E-07	9.96E-08	1.10E-07	9.24E-08
	cgd7_1820			
**2h**	0.00E+00	0.00E+00	0.00E+00	0.00E+00
**6h**	1.91E-05	1.02E-05	6.93E-06	1.10E-05
**12h**	2.67E-07	4.64E-07	1.17E-07	1.28E-09
**24h**	1.53E-06	7.56E-06	5.90E-06	1.73E-05
**36h**	3.11E-05	1.50E-05	4.64E-06	6.21E-06
**48h**	4.71E-05	1.62E-05	7.06E-06	1.05E-05
**72h**	4.03E-05	7.55E-06	3.21E-06	4.28E-06
	cgd7_1830			
**2h**	0.00E+00	0.00E+00	0.00E+00	0.00E+00
**6h**	2.56E-07	3.99E-07	1.11E-06	4.41E-07
**12h**	1.05E-06	1.88E-06	6.42E-07	6.22E-07
**24h**	9.69E-07	1.13E-06	1.72E-06	4.54E-06
**36h**	4.69E-06	3.63E-06	4.08E-06	2.77E-06
**48h**	3.28E-06	2.70E-06	2.13E-06	2.86E-06
**72h**	4.87E-07	6.70E-07	1.44E-06	7.03E-07
	cgd7_1840			
**2h**	1.69E-06	4.48E-07	7.41E-08	7.68E-07
**6h**	3.10E-07	1.45E-07	1.12E-07	1.20E-07
**12h**	4.90E-06	8.16E-06	1.79E-06	3.31E-06
**24h**	1.33E-06	1.55E-06	6.44E-07	6.77E-07
**36h**	1.99E-06	2.17E-06	2.00E-06	2.73E-06
**48h**	1.60E-06	1.00E-06	2.28E-06	1.43E-06
**72h**	1.30E-06	1.19E-06	1.51E-06	1.32E-06
	cgd7_1850			
**2h**	0.00E+00	0.00E+00	0.00E+00	0.00E+00
**6h**	3.67E-07	3.99E-06	3.42E-06	4.00E-06
**12h**	4.33E-05	2.62E-05	1.96E-05	1.98E-05
**24h**	7.40E-05	5.86E-05	5.04E-05	7.29E-05
**36h**	4.51E-04	2.07E-04	7.13E-05	1.05E-04
**48h**	2.41E-04	7.71E-05	5.06E-05	3.98E-05
**72h**	1.02E-04	2.02E-05	2.80E-05	2.07E-05
	cgd7_1860			
**2h**	0.00E+00	0.00E+00	0.00E+00	0.00E+00
**6h**	8.80E-09	1.29E-07	7.27E-08	1.16E-07
**12h**	6.38E-09	0.00E+00	2.48E-09	9.76E-09
**24h**	6.62E-08	1.10E-07	1.04E-07	1.35E-07
**36h**	3.11E-07	1.43E-07	1.45E-07	2.18E-07
**48h**	3.50E-07	2.37E-07	2.38E-07	9.94E-08

**72h**	1.06E-07	1.11E-07	3.54E-07	6.73E-08
	cgd7_1880			
**2h**	6.60E-07	1.04E-06	1.03E-06	8.96E-07
**6h**	9.59E-06	5.78E-06	3.15E-06	6.13E-06
**12h**	5.62E-07	1.22E-06	1.12E-06	1.97E-06
**24h**	2.03E-06	2.05E-06	1.78E-06	3.33E-06
**36h**	3.15E-06	1.35E-06	1.26E-06	1.18E-06
**48h**	1.62E-06	7.27E-07	7.11E-07	7.54E-07
**72h**	7.75E-07	5.17E-07	1.15E-06	5.80E-07
	cgd7_1890			
**2h**	5.35E-07	4.53E-07	1.48E-07	3.47E-07
**6h**	2.01E-05	3.17E-05	7.06E-05	6.15E-05
**12h**	3.40E-06	7.86E-06	2.66E-06	6.64E-06
**24h**	4.49E-06	8.34E-06	3.32E-05	4.08E-05
**36h**	1.98E-05	2.36E-05	8.34E-06	9.51E-06
**48h**	1.11E-05	8.62E-06	7.03E-06	8.26E-06
**72h**	1.89E-06	1.69E-06	1.05E-06	1.00E-06
	cgd7_1900			
**2h**	4.45E-06	4.32E-06	1.08E-06	7.36E-06
**6h**	9.64E-06	1.94E-05	1.46E-05	1.45E-05
**12h**	8.81E-07	1.89E-06	1.50E-06	2.84E-06
**24h**	3.83E-06	4.96E-06	6.51E-06	9.69E-06
**36h**	9.13E-06	7.69E-06	2.57E-06	2.55E-06
**48h**	3.38E-06	3.07E-06	3.53E-06	3.56E-06
**72h**	1.82E-06	8.77E-07	1.40E-06	5.87E-07
	cgd7_1920			
**2h**	0.00E+00	0.00E+00	0.00E+00	2.63E-04
**6h**	0.00E+00	1.91E-05	0.00E+00	0.00E+00
**12h**	2.76E-05	3.16E-05	7.22E-05	2.16E-05
**24h**	1.38E-04	7.35E-05	2.35E-05	5.00E-04
**36h**	1.98E-04	7.98E-05	2.17E-05	4.83E-04
**48h**	6.19E-05	1.05E-04	2.24E-05	5.13E-04
**72h**	2.44E-04	8.00E-05	1.20E-04	5.61E-04
	cgd7_1930			
**2h**	0.00E+00	0.00E+00	0.00E+00	0.00E+00
**6h**	3.25E-03	2.34E-03	3.56E-03	8.00E-03
**12h**	8.30E-04	2.27E-03	1.60E-03	1.23E-03
**24h**	5.30E-04	1.24E-03	1.86E-03	8.76E-03
**36h**	9.24E-04	1.37E-03	8.29E-04	4.74E-03
**48h**	3.08E-04	9.18E-04	6.06E-04	3.21E-03
**72h**	6.99E-04	1.34E-03	6.33E-04	2.78E-03
	cgd7_4500			
**2h**	0.00E+00	0.00E+00	1.89E-09	0.00E+00
**6h**	0.00E+00	2.49E-08	3.77E-09	0.00E+00
**12h**	3.87E-06	1.28E-05	1.69E-05	9.34E-06
**24h**	2.20E-06	7.10E-06	2.67E-06	5.89E-06
**36h**	6.60E-06	5.46E-06	8.75E-06	1.17E-05
**48h**	3.98E-06	2.83E-06	4.80E-06	5.49E-06
**72h**	3.52E-07	4.43E-07	1.82E-07	1.44E-07
	cgd7_4520			
**2h**	1.51E-05	4.62E-06	2.77E-06	4.39E-06
**6h**	1.79E-06	6.77E-06	2.65E-06	4.07E-06
**12h**	1.28E-06	1.67E-06	5.60E-06	4.84E-06

**24h**	5.90E-06	7.33E-06	5.84E-06	6.63E-06
**36h**	2.48E-06	2.72E-06	9.04E-07	1.72E-06
**48h**	3.56E-06	3.85E-06	2.47E-06	1.88E-06
**72h**	1.08E-06	6.97E-07	4.49E-07	1.44E-06
	cgd7_4530			
**2h**	0.00E+00	2.29E-07	3.89E-07	0.00E+00
**6h**	0.00E+00	9.21E-07	3.68E-07	1.31E-07
**12h**	1.28E-04	5.22E-05	2.31E-05	2.49E-05
**24h**	3.44E-05	4.48E-05	1.06E-05	7.45E-06
**36h**	4.73E-05	3.99E-05	2.92E-05	3.03E-05
**48h**	2.39E-04	6.54E-05	9.62E-05	7.17E-05
**72h**	1.29E-04	4.23E-05	2.69E-05	3.03E-05
	cgd7_4540			
**2h**	0.00E+00	5.66E-09	4.19E-09	8.38E-09
**6h**	6.98E-09	1.49E-07	1.46E-08	4.39E-08
**12h**	8.34E-06	2.25E-05	1.41E-05	8.56E-06
**24h**	1.73E-06	3.04E-06	1.04E-06	1.09E-06
**36h**	5.63E-06	9.55E-06	1.44E-05	1.03E-05
**48h**	2.69E-06	5.17E-07	1.51E-06	2.63E-06
**72h**	1.48E-07	1.82E-07	5.75E-08	1.15E-07
	cgd7_4560			
**2h**	2.09E-07	3.74E-08	2.15E-08	0.00E+00
**6h**	0.00E+00	1.50E-08	3.40E-09	0.00E+00
**12h**	4.20E-10	0.00E+00	3.22E-09	1.76E-08
**24h**	7.32E-09	1.11E-08	7.39E-09	1.19E-08
**36h**	3.46E-08	1.72E-07	9.26E-09	2.90E-08
**48h**	5.65E-07	1.72E-07	3.05E-07	1.15E-07
**72h**	3.46E-07	5.84E-07	6.00E-07	6.00E-07
	cgd7_4570			
**2h**	1.41E-06	2.53E-07	1.08E-07	7.52E-08
**6h**	0.00E+00	5.99E-08	2.89E-08	7.67E-08
**12h**	1.71E-09	3.94E-08	4.20E-08	5.83E-08
**24h**	4.29E-08	4.19E-08	1.03E-07	6.13E-08
**36h**	8.82E-08	5.21E-08	1.91E-08	6.76E-08
**48h**	5.53E-07	2.32E-07	1.50E-07	4.29E-08
**72h**	5.38E-07	1.43E-07	1.38E-07	2.27E-07
	cgd7_4580			
**2h**	9.69E-04	6.07E-05	3.10E-05	2.93E-05
**6h**	1.52E-04	3.43E-04	6.32E-05	9.18E-05
**12h**	1.02E-05	3.60E-05	3.80E-05	1.93E-05
**24h**	7.15E-05	7.87E-05	8.12E-05	6.78E-05
**36h**	1.08E-04	5.13E-05	1.53E-05	1.97E-05
**48h**	7.32E-05	4.46E-05	4.54E-05	1.99E-05
**72h**	5.27E-05	1.06E-05	9.41E-06	5.18E-06
	cgd7_4590			
**2h**	1.40E-06	9.07E-07	7.72E-07	1.71E-06
**6h**	4.14E-07	1.61E-06	7.03E-07	1.05E-06
**12h**	1.57E-06	3.07E-06	3.85E-06	6.03E-06
**24h**	1.11E-06	3.19E-06	1.54E-06	1.03E-06
**36h**	7.94E-07	8.55E-07	3.82E-07	1.87E-06
**48h**	1.24E-06	5.78E-07	8.20E-07	6.80E-07
**72h**	5.52E-07	1.74E-07	1.50E-07	5.71E-07
	cgd7_4600			

**2h**	1.39E-06	1.43E-07	5.75E-08	1.13E-08
**6h**	3.12E-07	1.42E-06	3.72E-07	9.93E-07
**12h**	3.98E-08	1.06E-07	1.15E-07	1.76E-08
**24h**	9.81E-08	8.81E-08	4.57E-07	7.16E-07
**36h**	2.57E-07	3.34E-07	8.96E-08	2.20E-07
**48h**	2.66E-07	7.84E-08	1.00E-07	1.86E-07
**72h**	1.31E-07	1.40E-07	6.39E-08	6.86E-08
	cgd7_4610			
**2h**	3.93E-07	4.38E-08	0.00E+00	0.00E+00
**6h**	1.12E-07	9.38E-08	2.15E-09	0.00E+00
**12h**	2.92E-07	2.66E-07	8.94E-08	6.20E-08
**24h**	2.74E-07	4.22E-07	3.20E-07	1.40E-07
**36h**	4.72E-07	7.08E-07	2.69E-07	8.59E-08
**48h**	2.27E-06	9.52E-07	6.31E-07	7.18E-07
**72h**	5.23E-06	1.16E-06	1.14E-06	6.02E-07
	cgd7_4620			
**2h**	2.18E-04	1.00E-05	1.20E-05	0.00E+00
**6h**	1.28E-04	2.38E-04	5.44E-05	4.40E-04
**12h**	6.73E-05	2.64E-05	4.01E-05	2.42E-05
**24h**	3.47E-05	7.34E-05	1.58E-04	3.27E-04
**36h**	1.35E-04	9.01E-05	6.81E-05	1.16E-04
**48h**	2.83E-04	2.37E-04	2.25E-04	2.19E-04
**72h**	3.34E-04	5.55E-05	8.18E-05	9.87E-05
	cgd7_4630			
**2h**	8.17E-05	3.62E-06	1.28E-06	4.15E-06
**6h**	2.68E-06	1.08E-05	5.67E-06	8.89E-06
**12h**	5.79E-06	6.38E-06	1.13E-05	3.04E-06
**24h**	1.80E-06	5.47E-06	2.75E-05	7.33E-06
**36h**	1.60E-05	4.86E-06	3.13E-06	2.75E-06
**48h**	5.80E-06	1.34E-06	2.85E-06	1.92E-06
**72h**	6.03E-06	1.65E-06	2.96E-06	1.26E-06
	cgd7_4640			
**2h**	2.96E-05	1.08E-05	3.85E-06	3.68E-05
**6h**	1.70E-07	3.37E-07	0.00E+00	2.37E-05
**12h**	3.00E-05	2.47E-04	1.68E-04	4.12E-03
**24h**	3.24E-05	1.37E-05	3.77E-04	1.61E-03
**36h**	1.06E-05	2.34E-05	8.74E-05	4.22E-04
**48h**	1.14E-05	1.22E-05	3.11E-05	4.15E-05
**72h**	4.68E-06	7.43E-06	6.67E-05	2.83E-04
	cgd7_4650			
**2h**	6.59E-03	4.11E-04	5.68E-04	0.00E+00
**6h**	2.15E-04	5.92E-04	1.61E-04	2.79E-05
**12h**	6.69E-06	1.26E-04	0.00E+00	1.60E-02
**24h**	6.10E-04	8.06E-04	7.72E-04	5.70E-03
**36h**	2.31E-04	2.08E-04	1.06E-04	2.27E-04
**48h**	2.88E-04	8.81E-05	3.84E-04	4.26E-05
**72h**	8.21E-05	2.38E-05	1.27E-04	0.00E+00
	cgd7_4660			
**2h**	1.76E-02	1.33E-02	7.94E-03	0.00E+00
**6h**	5.30E-03	4.88E-02	0.00E+00	4.87E-02
**12h**	2.06E-03	7.91E-05	0.00E+00	5.67E-02
**24h**	1.83E-03	2.79E-02	6.09E-03	7.70E-02
**36h**	1.29E-03	3.88E-03	1.90E-03	4.13E-02

**48h**	1.27E-03	1.24E-03	0.00E+00	2.03E-03
**72h**	3.02E-04	2.14E-03	0.00E+00	1.27E-03
	cgd7_4670			
**2h**	2.44E-05	2.78E-05	1.32E-06	0.00E+00
**6h**	2.17E-05	2.87E-05	5.85E-07	1.51E-03
**12h**	2.47E-06	7.33E-06	4.74E-07	0.00E+00
**24h**	6.15E-06	1.76E-05	6.53E-06	2.14E-05
**36h**	5.70E-06	1.18E-05	7.21E-04	4.06E-05
**48h**	1.07E-05	4.46E-06	2.07E-06	4.67E-07
**72h**	1.06E-06	1.56E-06	2.06E-06	9.26E-06
	cgd7_4680			
**2h**	0.00E+00	0.00E+00	0.00E+00	0.00E+00
**6h**	6.11E-07	3.57E-06	4.58E-09	6.88E-06
**12h**	3.58E-05	7.58E-05	1.23E-04	1.56E-03
**24h**	2.93E-05	2.40E-05	5.95E-05	2.69E-04
**36h**	3.39E-05	1.49E-04	0.00E+00	4.42E-04
**48h**	2.79E-05	1.18E-05	4.25E-03	6.93E-03
**72h**	4.23E-06	2.11E-05	1.49E-06	3.54E-05
	cgd7_4690			
**2h**	0.00E+00	2.65E-06	0.00E+00	0.00E+00
**6h**	7.61E-05	1.67E-04	4.24E-04	7.15E-03
**12h**	7.84E-05	1.06E-04	7.44E-05	5.35E-03
**24h**	2.08E-04	3.90E-04	1.13E-03	2.27E-02
**36h**	5.77E-04	3.95E-04	5.55E-03	6.18E-03
**48h**	8.55E-04	4.85E-04	7.72E-04	1.00E-02
**72h**	2.79E-04	1.14E-04	1.18E-03	4.46E-02
	cgd7_4700			
**2h**	5.59E-07	0.00E+00	0.00E+00	0.00E+00
**6h**	0.00E+00	0.00E+00	0.00E+00	0.00E+00
**12h**	0.00E+00	5.10E-07	0.00E+00	0.00E+00
**24h**	5.97E-07	3.33E-06	2.66E-04	0.00E+00
**36h**	6.05E-06	7.19E-06	7.01E-05	0.00E+00
**48h**	7.75E-06	1.86E-05	0.00E+00	1.81E-05
**72h**	8.71E-05	1.89E-05	2.08E-04	6.30E-04
	cgd7_4710			
**2h**	0.00E+00	6.48E-08	0.00E+00	0.00E+00
**6h**	0.00E+00	1.91E-07	0.00E+00	0.00E+00
**12h**	2.80E-07	9.92E-08	0.00E+00	0.00E+00
**24h**	6.19E-08	7.76E-08	8.70E-09	4.79E-05
**36h**	3.12E-06	7.32E-07	1.36E-06	4.56E-06
**48h**	1.99E-05	1.03E-05	1.06E-04	5.38E-05
**72h**	2.61E-05	3.75E-06	3.37E-06	2.05E-04
	cgd7_4720			
**2h**	0.00E+00	3.96E-04	0.00E+00	0.00E+00
**6h**	1.58E-10	1.09E-03	3.44E-10	0.00E+00
**12h**	1.64E-05	4.05E-04	1.71E-03	8.17E-04
**24h**	3.15E-06	7.16E-04	0.00E+00	1.28E-04
**36h**	2.27E-06	1.55E-04	0.00E+00	6.82E-05
**48h**	1.28E-06	1.02E-04	2.75E-06	1.82E-06
**72h**	5.20E-07	8.66E-05	5.23E-07	5.69E-06
	cgd7_4730			
**2h**	2.07E-04	0.00E+00	2.73E-03	7.87E-03
**6h**	5.63E-04	1.28E-03	6.29E-01	3.82E-02

**12h**	5.51E-05	5.62E-05	5.45E-03	4.52E-03
**24h**	1.17E-04	5.27E-04	7.96E-02	1.31E-02
**36h**	2.05E-04	5.27E-04	3.93E-03	1.09E-02
**48h**	2.16E-04	3.72E-04	5.39E-03	8.56E-03
**72h**	2.97E-04	6.30E-04	6.82E-03	1.32E-02
	cgd7_4740			
**2h**	1.28E-07	0.00E+00	0.00E+00	0.00E+00
**6h**	8.48E-07	4.48E-06	7.37E-07	9.67E-05
**12h**	6.04E-08	2.49E-07	1.59E-04	8.10E-06
**24h**	1.86E-07	1.77E-06	5.85E-05	1.11E-05
**36h**	1.07E-06	1.03E-06	1.53E-04	3.03E-04
**48h**	1.34E-06	8.95E-07	5.00E-08	1.76E-06
**72h**	8.50E-07	4.01E-07	1.10E-06	2.61E-05
	cgd7_4750			
**2h**	4.46E-04	1.50E-04	4.17E-04	5.84E-03
**6h**	3.18E-04	3.87E-04	6.13E-05	6.77E-02
**12h**	7.17E-05	9.01E-05	1.02E-03	2.87E-01
**24h**	1.00E-04	3.34E-04	1.55E-03	7.51E-02
**36h**	2.05E-04	2.67E-04	7.35E-04	2.59E-02
**48h**	1.91E-04	1.86E-04	3.34E-03	4.05E-03
**72h**	1.42E-04	1.29E-04	9.52E-04	1.70E-01
	cgd7_4760			
**2h**	6.24E-07	0.00E+00	3.32E-07	1.06E-06
**6h**	2.64E-07	9.06E-07	3.76E-07	3.31E-07
**12h**	4.30E-08	7.17E-08	5.44E-07	5.61E-06
**24h**	1.74E-07	3.76E-07	4.91E-07	4.90E-07
**36h**	2.41E-07	2.12E-07	4.57E-07	1.85E-07
**48h**	1.54E-07	1.47E-07	1.79E-07	2.59E-07
**72h**	1.26E-07	2.02E-08	4.66E-07	1.89E-07
	cgd7_4790			
**2h**	1.49E-05	2.05E-04	8.47E-05	4.64E-05
**6h**	5.48E-07	1.20E-06	2.61E-05	6.15E-06
**12h**	3.76E-06	1.76E-04	1.69E-04	1.79E-05
**24h**	3.46E-06	1.05E-04	2.43E-05	4.23E-06
**36h**	2.51E-06	1.57E-05	3.70E-05	5.34E-06
**48h**	1.29E-05	2.82E-05	9.51E-05	2.61E-05
**72h**	1.35E-05	3.66E-05	2.32E-04	2.70E-05
	cgd7_4810			
**2h**	6.91E-09	2.83E-08	0.00E+00	6.47E-08
**6h**	0.00E+00	3.45E-09	0.00E+00	5.61E-09
**12h**	0.00E+00	0.00E+00	0.00E+00	1.47E-09
**24h**	3.55E-09	1.71E-08	2.83E-08	3.73E-08
**36h**	4.86E-08	1.12E-07	1.67E-07	3.61E-07
**48h**	2.74E-05	2.16E-05	1.27E-04	3.93E-05
**72h**	1.51E-05	2.77E-05	7.09E-05	3.56E-05
	cgd7_4820			
**2h**	0.00E+00	0.00E+00	0.00E+00	2.93E-09
**6h**	0.00E+00	0.00E+00	0.00E+00	0.00E+00
**12h**	5.76E-10	4.23E-08	5.03E-08	2.91E-08
**24h**	9.37E-10	5.67E-08	4.94E-08	1.65E-08
**36h**	3.69E-08	2.30E-08	6.41E-08	6.00E-08
**48h**	1.31E-08	9.16E-09	1.90E-08	2.39E-08
**72h**	4.84E-08	3.77E-08	1.10E-07	2.73E-08

	cgd7_4830			
**2h**	0.00E+00	0.00E+00	0.00E+00	0.00E+00
**6h**	0.00E+00	0.00E+00	0.00E+00	0.00E+00
**12h**	6.14E-07	6.45E-07	2.40E-06	7.54E-07
**24h**	7.61E-08	1.00E-06	4.01E-07	4.80E-07
**36h**	1.41E-06	6.09E-07	2.00E-06	1.84E-06
**48h**	1.93E-07	2.22E-07	6.63E-07	3.58E-07
**72h**	4.97E-07	2.56E-07	3.40E-07	2.65E-07
	cgd7_4840			
**2h**	1.20E-08	2.84E-08	6.63E-09	8.34E-09
**6h**	1.44E-09	1.79E-08	4.96E-08	5.65E-09
**12h**	8.63E-07	1.48E-06	1.40E-06	7.53E-07
**24h**	1.57E-07	4.65E-07	2.19E-07	2.24E-07
**36h**	1.24E-06	8.05E-07	8.11E-07	2.31E-06
**48h**	4.35E-07	1.40E-07	6.08E-07	3.47E-07
**72h**	2.77E-07	2.62E-07	4.11E-07	2.56E-07
	cgd7_4860			
**2h**	3.05E-06	1.90E-06	9.24E-07	4.00E-06
**6h**	3.58E-07	9.70E-07	1.80E-06	9.19E-07
**12h**	8.55E-07	1.77E-06	4.35E-06	2.47E-06
**24h**	1.61E-06	2.60E-06	2.68E-06	1.96E-06
**36h**	5.42E-07	1.23E-06	7.06E-07	7.31E-07
**48h**	4.91E-07	2.95E-07	5.75E-07	8.69E-07
**72h**	1.50E-07	2.42E-07	3.00E-07	3.03E-07
	cgd7_4880			
**2h**	2.11E-05	3.93E-04	1.23E-03	4.47E-04
**6h**	8.65E-06	2.45E-04	2.57E-04	9.55E-05
**12h**	3.77E-05	3.59E-04	5.07E-04	1.73E-04
**24h**	3.07E-05	4.38E-05	2.40E-04	1.68E-04
**36h**	2.77E-05	1.70E-04	2.98E-05	3.55E-04
**48h**	1.94E-03	6.63E-04	1.74E-03	6.90E-03
**72h**	5.47E-04	1.03E-03	1.51E-03	1.61E-03
	cgd7_4900			
**2h**	5.89E-07	2.00E-07	0.00E+00	5.17E-07
**6h**	3.45E-07	1.72E-07	1.08E-06	3.50E-07
**12h**	0.00E+00	4.24E-08	7.77E-09	8.23E-08
**24h**	1.05E-07	1.22E-07	3.20E-07	8.87E-07
**36h**	6.28E-07	3.82E-07	8.20E-08	3.47E-07
**48h**	6.43E-07	1.46E-07	2.91E-07	3.38E-07
**72h**	9.95E-07	5.32E-07	2.62E-06	5.97E-07
	cgd7_4910			
**2h**	1.50E-07	9.88E-06	0.00E+00	7.51E-09
**6h**	0.00E+00	1.76E-09	0.00E+00	0.00E+00
**12h**	2.20E-05	1.41E-04	2.83E-04	3.95E-05
**24h**	7.35E-06	3.19E-05	4.53E-06	8.97E-06
**36h**	1.82E-06	7.64E-06	9.34E-06	1.34E-05
**48h**	4.15E-06	2.86E-06	3.26E-06	3.51E-06
**72h**	1.99E-07	2.48E-07	8.00E-07	3.70E-07
	cgd7_4920			
**2h**	3.08E-08	1.25E-06	0.00E+00	1.37E-09
**6h**	2.44E-08	3.88E-08	0.00E+00	6.53E-07
**12h**	1.16E-07	2.44E-06	2.97E-06	2.27E-07
**24h**	6.26E-07	1.38E-06	1.43E-06	1.63E-06

**36h**	9.75E-07	4.11E-06	2.41E-06	1.61E-06
**48h**	7.76E-06	1.65E-05	7.60E-06	5.74E-06
**72h**	7.58E-07	3.43E-06	8.16E-06	2.03E-06
	cgd7_4930			
**2h**	7.89E-07	2.54E-04	7.67E-05	1.10E-05
**6h**	1.07E-05	2.07E-04	2.00E-03	2.22E-04
**12h**	1.03E-05	1.68E-04	2.46E-04	4.54E-05
**24h**	2.34E-05	2.96E-04	1.10E-03	1.05E-03
**36h**	1.98E-04	1.02E-04	4.16E-04	8.21E-04
**48h**	3.74E-04	2.11E-04	5.35E-04	3.66E-04
**72h**	1.60E-04	1.32E-03	6.03E-04	2.52E-04
	cgd7_4940			
**2h**	2.58E-08	1.65E-08	0.00E+00	4.55E-09
**6h**	1.45E-07	4.94E-08	4.59E-08	6.23E-08
**12h**	1.07E-08	2.68E-09	1.59E-07	6.01E-08
**24h**	1.22E-07	2.46E-07	9.92E-07	3.81E-07
**36h**	1.93E-07	5.32E-07	6.25E-07	4.30E-07
**48h**	2.22E-06	1.83E-06	2.96E-06	3.14E-06
**72h**	2.49E-06	1.68E-06	1.82E-06	8.65E-06
	cgd7_4950			
**2h**	2.23E-07	1.09E-06	1.59E-07	6.97E-07
**6h**	3.55E-06	1.19E-06	2.10E-06	1.44E-06
**12h**	2.50E-07	1.24E-06	6.65E-07	3.17E-07
**24h**	7.95E-07	1.02E-05	5.55E-05	3.63E-06
**36h**	1.81E-06	4.28E-06	5.66E-06	3.53E-06
**48h**	1.55E-05	3.13E-05	2.70E-05	4.08E-05
**72h**	3.45E-05	5.50E-05	5.72E-05	5.82E-05
	cgd7_4960			
**2h**	9.15E-06	4.78E-06	2.02E-06	1.72E-05
**6h**	1.99E-05	1.94E-05	8.50E-06	6.37E-06
**12h**	8.95E-07	4.10E-06	9.34E-06	3.97E-06
**24h**	5.03E-06	2.05E-05	1.15E-05	6.70E-06
**36h**	2.49E-06	9.67E-06	3.87E-06	6.38E-06
**48h**	2.31E-06	4.91E-06	5.21E-06	4.18E-06
**72h**	1.64E-06	1.12E-06	1.94E-06	2.17E-06
	cgd7_4970			
**2h**	1.12E-03	2.59E-04	2.46E-04	1.88E-04
**6h**	3.08E-05	1.93E-04	1.27E-04	3.37E-06
**12h**	2.21E-03	6.98E-04	3.53E-04	1.11E-04
**24h**	1.37E-03	1.55E-04	6.91E-04	1.51E-04
**36h**	9.47E-04	5.81E-04	6.38E-04	4.59E-04
**48h**	1.70E-04	1.61E-04	8.84E-04	7.34E-04
**72h**	2.18E-03	4.75E-04	6.37E-04	1.63E-03
	cgd7_4980			
**2h**	3.65E-07	6.73E-08	0.00E+00	5.46E-08
**6h**	2.70E-09	1.75E-08	1.70E-08	4.74E-08
**12h**	2.10E-08	6.88E-08	2.44E-07	6.67E-08
**24h**	3.57E-08	1.36E-07	1.10E-07	1.34E-07
**36h**	4.03E-07	4.21E-07	3.05E-07	3.22E-07
**48h**	2.55E-07	2.09E-07	1.11E-07	2.11E-07
**72h**	2.23E-07	3.44E-08	1.15E-07	2.64E-07
	cgd7_5000			
**2h**	2.19E-05	1.51E-05	4.74E-06	1.09E-05

**6h**	1.76E-05	4.57E-05	1.37E-05	1.18E-05
**12h**	3.56E-06	5.13E-06	3.40E-05	1.79E-05
**24h**	5.60E-06	9.88E-06	3.01E-05	1.19E-05
**36h**	9.08E-06	8.45E-06	1.19E-05	8.58E-06
**48h**	3.67E-06	2.28E-06	3.24E-06	4.31E-06
**72h**	1.35E-06	7.01E-07	1.04E-06	1.00E-06
	cgd7_5020			
**2h**	0.00E+00	8.35E-08	0.00E+00	1.59E-08
**6h**	2.16E-07	2.83E-07	1.52E-06	1.03E-06
**12h**	1.89E-07	1.19E-06	6.76E-07	2.20E-07
**24h**	1.63E-07	5.78E-07	1.69E-06	4.72E-07
**36h**	1.33E-06	3.72E-06	1.82E-06	1.26E-06
**48h**	2.14E-06	4.09E-06	7.81E-06	4.75E-06
**72h**	7.30E-06	4.03E-06	3.60E-06	3.07E-06
	cgd7_5030			
**2h**	0.00E+00	0.00E+00	0.00E+00	0.00E+00
**6h**	0.00E+00	0.00E+00	0.00E+00	0.00E+00
**12h**	4.49E-03	4.75E-05	2.58E-04	4.38E-04
**24h**	6.28E-04	3.53E-05	1.34E-05	7.25E-05
**36h**	3.37E-04	3.03E-05	4.51E-05	2.32E-05
**48h**	3.88E-03	2.93E-05	2.06E-04	1.34E-04
**72h**	2.41E-03	5.49E-05	3.06E-04	5.04E-04
	cgd7_5040			
**2h**	2.73E-08	1.57E-09	1.87E-08	2.97E-08
**6h**	5.30E-08	2.78E-08	1.14E-07	4.34E-08
**12h**	1.95E-08	3.65E-09	2.36E-08	8.18E-09
**24h**	7.82E-08	6.54E-08	1.05E-07	1.70E-07
**36h**	2.24E-08	1.95E-07	1.63E-07	9.65E-08
**48h**	1.85E-06	8.13E-07	1.83E-06	1.41E-06
**72h**	1.03E-06	4.95E-07	1.43E-06	9.28E-07
	cgd7_5050			
**2h**	0.00E+00	0.00E+00	0.00E+00	0.00E+00
**6h**	2.38E-09	6.60E-07	2.36E-06	0.00E+00
**12h**	0.00E+00	4.73E-06	7.15E-07	1.13E-06
**24h**	2.70E-07	1.44E-06	1.80E-06	3.47E-06
**36h**	7.64E-06	7.24E-05	9.62E-06	1.78E-05
**48h**	5.57E-04	8.52E-04	8.79E-04	6.23E-04
**72h**	8.49E-04	8.38E-04	4.21E-04	7.01E-04
	cgd7_5060			
**2h**	1.06E-04	1.07E-05	1.16E-05	4.56E-06
**6h**	2.63E-05	4.59E-05	2.33E-05	1.42E-05
**12h**	3.58E-06	2.59E-06	2.37E-05	4.31E-06
**24h**	7.26E-06	1.09E-05	2.23E-05	2.27E-05
**36h**	1.31E-05	1.08E-05	5.70E-06	2.44E-06
**48h**	5.26E-06	1.80E-06	3.12E-06	2.28E-06
**72h**	3.37E-06	8.94E-07	1.31E-06	1.77E-06
	cgd7_5080			
**2h**	6.31E-03	5.14E-04	3.72E-04	9.18E-04
**6h**	7.09E-03	3.90E-03	1.45E-03	5.03E-03
**12h**	2.59E-04	4.73E-05	1.48E-04	6.82E-04
**24h**	1.50E-03	5.22E-04	9.01E-04	2.11E-03
**36h**	5.30E-04	4.76E-04	3.32E-04	3.58E-04
**48h**	4.66E-04	2.57E-04	3.02E-04	3.07E-04

**72h**	1.73E-04	3.37E-05	6.74E-05	3.75E-05
	cgd7_5090			
**2h**	1.58E-04	2.27E-05	1.61E-05	5.59E-05
**6h**	6.95E-06	1.06E-05	7.16E-06	1.72E-05
**12h**	2.22E-06	1.42E-06	6.00E-06	1.39E-05
**24h**	1.03E-04	5.38E-05	1.57E-05	4.04E-05
**36h**	1.69E-05	1.32E-05	8.77E-06	9.48E-06
**48h**	7.20E-05	2.24E-05	1.11E-05	2.07E-05
**72h**	1.86E-05	9.08E-06	1.48E-05	1.19E-05
	cgd7_5100			
**2h**	2.50E-07	3.65E-07	4.00E-08	2.63E-07
**6h**	4.67E-07	9.41E-07	4.30E-07	2.56E-07
**12h**	4.44E-08	8.85E-08	3.05E-08	4.11E-08
**24h**	2.78E-07	2.97E-07	3.76E-07	1.09E-06
**36h**	4.72E-07	6.39E-07	6.18E-07	4.69E-07
**48h**	1.05E-06	3.59E-07	5.01E-07	4.59E-07
**72h**	1.18E-07	2.46E-07	1.49E-07	2.73E-07
	cgd7_5110			
**2h**	0.00E+00	1.23E-08	0.00E+00	1.57E-08
**6h**	1.77E-09	0.00E+00	1.16E-09	1.40E-09
**12h**	1.10E-08	3.80E-09	1.32E-08	9.55E-09
**24h**	5.61E-09	1.71E-08	1.85E-08	7.57E-09
**36h**	2.98E-08	6.69E-08	3.30E-08	3.26E-08
**48h**	2.27E-06	3.35E-07	1.02E-06	1.69E-06
**72h**	1.17E-06	3.45E-06	1.67E-06	2.44E-06
	cgd7_5120			
**2h**	1.00E-04	7.49E-06	0.00E+00	1.31E-05
**6h**	1.20E-03	2.43E-03	8.13E-04	1.49E-03
**12h**	2.66E-05	3.64E-05	1.21E-06	5.97E-05
**24h**	4.38E-04	4.12E-04	9.01E-04	8.29E-04
**36h**	3.00E-04	2.08E-04	1.54E-04	1.48E-04
**48h**	7.29E-04	6.61E-04	1.48E-03	7.68E-04
**72h**	2.26E-04	1.46E-03	1.36E-03	1.13E-03
	cgd7_5130			
**2h**	0.00E+00	2.35E-07	0.00E+00	0.00E+00
**6h**	0.00E+00	0.00E+00	0.00E+00	0.00E+00
**12h**	3.60E-07	4.75E-07	0.00E+00	0.00E+00
**24h**	2.55E-07	0.00E+00	4.10E-08	6.00E-08
**36h**	8.61E-06	3.09E-06	3.03E-06	2.49E-06
**48h**	6.26E-04	4.07E-04	4.59E-04	7.05E-04
**72h**	1.05E-03	1.17E-03	1.51E-03	7.26E-04
	cgd7_5140			
**2h**	0.00E+00	0.00E+00	0.00E+00	0.00E+00
**6h**	0.00E+00	1.47E-09	0.00E+00	0.00E+00
**12h**	0.00E+00	0.00E+00	0.00E+00	0.00E+00
**24h**	0.00E+00	0.00E+00	0.00E+00	2.23E-10
**36h**	4.29E-09	1.55E-09	1.74E-09	7.12E-09
**48h**	1.56E-06	2.27E-07	8.94E-07	6.20E-07
**72h**	1.75E-07	1.63E-07	9.29E-07	4.23E-07
	cgd7_5150			
**2h**	7.83E-08	0.00E+00	0.00E+00	1.27E-08
**6h**	9.74E-10	4.18E-09	3.00E-09	0.00E+00
**12h**	3.58E-09	0.00E+00	0.00E+00	1.07E-08

**24h**	2.25E-08	3.03E-08	2.28E-08	1.95E-08
**36h**	7.14E-06	4.11E-08	5.96E-08	3.13E-08
**48h**	2.37E-06	5.49E-07	1.03E-06	8.67E-07
**72h**	1.62E-06	6.20E-07	3.73E-06	8.93E-07
	cgd7_5170			
**2h**	5.83E-06	8.70E-07	4.32E-06	4.43E-06
**6h**	6.58E-07	1.82E-06	1.20E-06	9.53E-07
**12h**	6.73E-07	2.29E-06	7.19E-07	1.52E-06
**24h**	8.55E-07	2.39E-06	1.67E-06	3.60E-06
**36h**	1.57E-06	1.72E-06	2.93E-06	1.06E-06
**48h**	1.36E-06	8.67E-07	1.28E-06	1.30E-06
**72h**	2.79E-07	4.55E-07	6.48E-07	3.63E-07
	cgd7_5180			
**2h**	7.50E-04	4.29E-04	1.62E-04	5.74E-04
**6h**	4.98E-04	1.82E-03	1.53E-03	1.02E-03
**12h**	8.15E-04	4.74E-04	7.26E-04	4.22E-04
**24h**	1.33E-03	1.19E-03	9.48E-04	9.51E-04
**36h**	4.12E-04	2.56E-04	3.13E-04	1.35E-04
**48h**	5.72E-04	9.04E-05	1.52E-04	1.61E-04
**72h**	1.32E-04	4.84E-05	8.36E-05	5.33E-05
	cgd7_5190			
**2h**	3.75E-07	1.38E-07	1.51E-07	1.14E-06
**6h**	4.25E-08	1.30E-07	8.10E-08	1.14E-07
**12h**	1.19E-07	1.18E-07	4.49E-08	1.04E-07
**24h**	4.43E-08	1.12E-07	1.58E-07	4.37E-07
**36h**	1.74E-07	1.38E-07	2.23E-07	1.34E-07
**48h**	5.65E-07	2.28E-07	2.82E-07	4.58E-07
**72h**	7.35E-08	9.66E-08	2.58E-07	1.57E-07
	cgd7_5200			
**2h**	1.29E-04	1.06E-03	1.10E-04	8.21E-03
**6h**	4.30E-04	4.59E-04	4.28E-04	5.20E-04
**12h**	7.11E-04	2.96E-04	5.92E-04	1.28E-03
**24h**	1.58E-04	1.23E-03	1.48E-03	6.15E-03
**36h**	3.35E-04	1.63E-03	3.80E-04	7.13E-04
**48h**	2.41E-04	2.31E-04	1.06E-04	1.74E-03
**72h**	1.63E-04	5.78E-04	6.81E-04	8.88E-04
	cgd7_5210			
**2h**	7.90E-07	2.09E-06	6.91E-07	1.87E-06
**6h**	7.18E-07	7.16E-07	3.58E-07	7.83E-07
**12h**	1.37E-06	1.49E-06	1.97E-06	1.78E-06
**24h**	1.75E-06	3.16E-06	3.80E-06	1.55E-06
**36h**	7.85E-07	9.79E-07	1.32E-06	1.14E-06
**48h**	4.46E-07	3.19E-07	1.16E-06	5.89E-09
**72h**	1.65E-07	1.33E-07	7.37E-07	2.04E-07
	cgd7_5220			
**2h**	9.10E-04	5.90E-04	5.05E-05	6.04E-04
**6h**	1.71E-03	4.73E-04	3.80E-04	6.24E-04
**12h**	1.09E-04	6.39E-04	1.03E-04	1.50E-04
**24h**	3.71E-04	1.78E-03	4.06E-04	4.26E-04
**36h**	2.77E-04	2.86E-04	1.69E-04	1.03E-04
**48h**	3.24E-04	2.33E-04	4.14E-04	4.62E-06
**72h**	1.28E-04	9.35E-05	2.97E-04	2.71E-05
	cgd7_5230			

**2h**	3.82E-03	9.97E-04	1.46E-03	5.64E-03
**6h**	3.18E-03	6.33E-04	7.96E-04	1.07E-03
**12h**	9.50E-04	2.41E-04	1.12E-03	1.49E-03
**24h**	1.78E-04	1.77E-04	3.36E-05	5.82E-04
**36h**	6.67E-04	1.83E-04	2.36E-04	4.06E-04
**48h**	8.80E-05	1.31E-04	5.14E-04	7.40E-06
**72h**	1.31E-04	1.31E-04	5.69E-04	1.29E-04
	cgd7_5240			
**2h**	1.12E-06	5.17E-07	5.44E-07	8.45E-08
**6h**	3.56E-07	3.84E-06	1.68E-06	2.16E-06
**12h**	8.00E-05	7.87E-05	4.58E-05	2.34E-05
**24h**	1.57E-05	3.81E-05	6.09E-06	7.92E-06
**36h**	8.53E-06	1.60E-05	3.53E-05	1.04E-05
**48h**	1.24E-05	2.29E-06	2.13E-05	2.86E-07
**72h**	6.66E-06	4.36E-06	1.07E-05	2.64E-06
	cgd7_5250			
**2h**	4.85E-06	2.57E-05	1.14E-05	1.60E-05
**6h**	6.84E-06	6.60E-06	1.72E-06	7.66E-06
**12h**	1.56E-05	1.41E-05	1.46E-05	7.89E-06
**24h**	6.92E-06	2.08E-05	4.99E-06	7.29E-06
**36h**	4.90E-06	7.71E-06	1.14E-05	3.79E-06
**48h**	6.08E-06	2.26E-06	8.16E-06	7.90E-08
**72h**	4.90E-06	5.95E-06	1.21E-05	2.26E-06
	cgd7_5270			
**2h**	1.82E-06	1.34E-06	7.52E-07	1.92E-06
**6h**	2.69E-06	3.51E-06	3.09E-06	1.87E-06
**12h**	3.21E-07	3.92E-07	1.15E-06	8.69E-07
**24h**	1.07E-06	1.47E-06	2.63E-06	1.48E-06
**36h**	6.98E-07	1.28E-06	9.97E-07	5.02E-07
**48h**	5.78E-07	3.06E-07	1.22E-06	1.52E-08
**72h**	2.05E-07	1.45E-07	1.21E-06	2.40E-07
	cgd7_5280			
**2h**	0.00E+00	1.63E-06	0.00E+00	0.00E+00
**6h**	1.90E-04	2.01E-04	9.89E-05	1.27E-04
**12h**	6.31E-06	4.25E-05	7.46E-06	4.45E-06
**24h**	2.51E-05	7.24E-05	6.55E-05	5.67E-05
**36h**	5.60E-05	5.93E-05	5.00E-05	2.31E-05
**48h**	5.29E-05	6.30E-05	3.60E-05	3.74E-07
**72h**	6.32E-06	1.03E-05	9.94E-06	3.90E-06
	cgd7_5300			
**2h**	7.61E-05	1.89E-04	2.04E-07	1.58E-05
**6h**	3.43E-04	4.39E-04	2.21E-04	2.76E-04
**12h**	1.97E-07	0.00E+00	1.07E-06	5.99E-06
**24h**	5.47E-05	7.60E-05	9.39E-05	5.16E-05
**36h**	8.57E-05	1.22E-04	7.06E-05	2.04E-05
**48h**	1.25E-04	1.48E-04	8.11E-05	1.49E-06
**72h**	8.09E-06	8.04E-05	2.23E-05	4.10E-06
	cgd7_5310			
**2h**	1.54E-05	4.10E-05	5.44E-06	2.95E-05
**6h**	4.09E-06	5.55E-07	5.26E-06	5.27E-06
**12h**	4.42E-06	1.59E-05	8.61E-06	9.47E-06
**24h**	1.18E-05	1.22E-05	6.68E-06	7.24E-06
**36h**	3.08E-06	9.46E-06	5.14E-06	1.85E-06

**48h**	3.20E-06	5.84E-06	8.16E-06	1.17E-07
**72h**	3.38E-06	2.08E-06	3.52E-06	1.68E-06
	cgd7_5330			
**2h**	3.95E-09	0.00E+00	0.00E+00	2.57E-08
**6h**	0.00E+00	0.00E+00	8.46E-09	1.96E-09
**12h**	1.00E-05	4.84E-05	3.86E-06	4.04E-06
**24h**	6.19E-07	1.57E-06	1.21E-06	5.97E-07
**36h**	3.65E-07	9.52E-07	1.57E-06	3.83E-06
**48h**	2.05E-07	2.45E-07	6.58E-07	6.28E-09
**72h**	1.53E-07	1.15E-07	1.64E-07	4.21E-08
	cgd7_5340			
**2h**	0.00E+00	2.66E-08	0.00E+00	0.00E+00
**6h**	0.00E+00	2.59E-09	0.00E+00	0.00E+00
**12h**	3.75E-06	7.85E-06	2.46E-06	9.88E-07
**24h**	4.09E-07	8.10E-07	4.66E-07	2.24E-07
**36h**	3.59E-07	3.57E-07	1.73E-06	7.36E-07
**48h**	2.89E-07	3.68E-07	3.07E-07	8.21E-09
**72h**	5.78E-08	1.18E-07	3.47E-07	4.18E-08
	cgd7_5350			
**2h**	1.15E-06	3.52E-06	6.02E-08	0.00E+00
**6h**	3.22E-06	3.09E-06	8.25E-06	2.17E-06
**12h**	7.45E-07	1.94E-06	1.08E-06	1.99E-06
**24h**	2.43E-06	2.35E-06	1.02E-05	6.01E-06
**36h**	9.15E-06	1.89E-05	9.60E-06	8.07E-06
**48h**	5.50E-06	1.23E-05	1.38E-05	6.30E-07
**72h**	2.38E-05	3.64E-06	1.25E-05	1.03E-05
	cgd7_5500			
**2h**	1.30E-06	2.32E-07	3.14E-06	1.44E-06
**6h**	0.00E+00	0.00E+00	7.27E-07	1.06E-08
**12h**	8.43E-08	3.17E-06	5.32E-05	4.19E-07
**24h**	3.89E-06	1.35E-05	7.86E-06	1.15E-05
**36h**	1.36E-05	9.10E-05	4.80E-05	2.83E-05
**48h**	1.05E-04	8.43E-05	1.35E-04	5.00E-04
**72h**	6.77E-06	5.95E-05	6.31E-05	4.79E-05
	cgd7_5510			
**2h**	0.00E+00	0.00E+00	0.00E+00	1.22E-08
**6h**	0.00E+00	0.00E+00	0.00E+00	0.00E+00
**12h**	1.06E-09	9.61E-09	2.15E-09	5.53E-09
**24h**	5.51E-09	4.52E-09	1.89E-08	3.71E-09
**36h**	1.94E-08	9.44E-08	3.28E-08	1.22E-08
**48h**	2.61E-08	1.64E-06	2.22E-08	3.30E-08
**72h**	7.28E-09	1.05E-06	9.23E-08	6.51E-08
	cgd7_5520			
**2h**	3.00E-08	0.00E+00	0.00E+00	0.00E+00
**6h**	7.74E-08	7.06E-08	6.61E-08	1.67E-08
**12h**	1.74E-05	1.33E-04	1.67E-04	2.98E-05
**24h**	1.24E-05	1.68E-05	2.25E-05	1.39E-05
**36h**	2.04E-06	1.14E-04	8.02E-05	6.34E-05
**48h**	6.35E-07	7.86E-05	2.06E-06	9.99E-06
**72h**	1.63E-07	4.21E-05	8.54E-07	7.98E-07
	cgd8_10			
**2h**	3.68E-08	2.49E-09	2.75E-08	1.25E-09
**6h**	7.27E-07	1.10E-07	3.69E-07	7.48E-08

**12h**	6.90E-11	0.00E+00	2.42E-08	2.95E-09
**24h**	5.81E-08	6.78E-09	1.08E-07	1.28E-07
**36h**	3.24E-08	4.31E-08	4.18E-08	9.18E-08
**48h**	1.41E-08	5.00E-08	4.69E-08	8.63E-08
**72h**	2.53E-09	8.69E-08	1.04E-08	1.10E-08
	cgd8_20			
**2h**	0.00E+00	0.00E+00	0.00E+00	0.00E+00
**6h**	0.00E+00	0.00E+00	0.00E+00	0.00E+00
**12h**	1.08E-06	1.20E-06	1.94E-06	5.70E-07
**24h**	7.07E-08	1.02E-08	1.83E-08	4.23E-08
**36h**	6.04E-08	1.94E-08	2.68E-07	2.58E-07
**48h**	2.33E-09	9.08E-08	4.95E-09	1.67E-08
**72h**	2.07E-08	4.15E-08	4.10E-08	6.63E-08
	cgd8_30			
**2h**	2.31E-07	1.15E-08	1.19E-08	2.93E-07
**6h**	8.87E-09	2.78E-09	0.00E+00	2.53E-09
**12h**	1.23E-07	1.41E-07	1.05E-07	5.12E-08
**24h**	3.54E-08	1.96E-08	6.66E-08	3.51E-08
**36h**	3.74E-08	2.44E-08	1.85E-08	1.14E-07
**48h**	7.00E-09	1.19E-08	1.73E-08	8.21E-08
**72h**	1.90E-08	2.67E-07	2.50E-08	9.14E-08
	cgd8_40			
**2h**	5.25E-10	8.29E-10	0.00E+00	0.00E+00
**6h**	2.24E-09	0.00E+00	0.00E+00	0.00E+00
**12h**	1.77E-06	2.71E-06	2.36E-06	1.31E-06
**24h**	1.47E-07	9.90E-08	2.93E-07	1.23E-07
**36h**	2.15E-07	1.49E-07	1.26E-06	1.25E-06
**48h**	9.28E-08	6.41E-08	2.26E-07	1.63E-07
**72h**	4.66E-08	7.50E-08	1.39E-07	1.47E-07
	cgd8_50			
**2h**	7.49E-07	0.00E+00	8.32E-08	5.66E-06
**6h**	3.01E-08	1.66E-08	0.00E+00	1.11E-07
**12h**	3.77E-06	5.79E-06	1.02E-05	6.63E-06
**24h**	5.27E-07	1.19E-06	2.71E-06	2.31E-06
**36h**	2.45E-06	3.58E-06	1.63E-05	2.51E-05
**48h**	1.31E-06	3.87E-06	3.70E-06	8.60E-06
**72h**	3.31E-07	4.58E-06	9.47E-07	1.00E-05
	cgd8_60			
**2h**	5.56E-05	6.65E-06	1.08E-04	4.58E-04
**6h**	4.07E-06	3.14E-06	1.03E-05	7.92E-06
**12h**	1.08E-05	3.92E-05	7.28E-05	3.68E-05
**24h**	2.24E-05	2.05E-05	6.98E-05	5.60E-05
**36h**	3.39E-05	2.96E-05	5.02E-05	6.86E-05
**48h**	1.59E-05	1.81E-05	6.28E-05	9.09E-05
**72h**	9.66E-06	1.48E-05	1.34E-05	2.65E-05
	cgd8_70			
**2h**	2.00E-06	2.28E-08	5.62E-07	1.99E-06
**6h**	1.19E-06	6.07E-07	6.61E-06	1.72E-06
**12h**	1.74E-07	2.32E-07	1.53E-07	2.27E-07
**24h**	3.16E-07	1.78E-07	4.37E-06	2.39E-06
**36h**	8.31E-07	9.52E-07	1.61E-06	2.98E-06
**48h**	2.32E-06	9.37E-07	2.37E-06	4.54E-06
**72h**	3.55E-07	4.59E-07	5.31E-07	6.81E-07

	cgd8_80			
**2h**	3.01E-06	4.77E-07	2.14E-06	6.13E-06
**6h**	1.01E-06	3.27E-07	2.32E-06	1.97E-06
**12h**	6.55E-07	4.91E-07	3.61E-06	1.83E-06
**24h**	9.33E-07	4.87E-07	4.48E-06	2.31E-06
**36h**	1.03E-06	5.96E-07	1.40E-06	2.03E-06
**48h**	7.01E-07	4.00E-07	2.03E-06	3.78E-06
**72h**	1.73E-07	1.34E-07	1.87E-07	7.67E-07
	cgd8_90			
**2h**	8.65E-08	8.13E-09	2.49E-08	2.89E-08
**6h**	4.24E-07	2.63E-07	6.00E-07	1.72E-07
**12h**	1.38E-08	3.32E-09	4.38E-08	7.11E-08
**24h**	7.30E-08	1.32E-08	4.90E-07	2.81E-07
**36h**	2.70E-07	1.97E-07	3.16E-07	3.58E-07
**48h**	3.33E-07	1.56E-07	2.83E-07	4.98E-07
**72h**	3.39E-07	4.11E-07	2.91E-07	1.52E-07
	cgd7_5360			
**2h**	5.06E-07	7.23E-08	8.10E-07	6.50E-07
**6h**	6.12E-07	5.73E-07	1.20E-06	4.63E-07
**12h**	4.57E-08	9.54E-08	4.09E-07	9.06E-08
**24h**	3.50E-07	3.14E-07	8.25E-07	2.74E-07
**36h**	2.42E-07	2.76E-07	3.00E-07	6.98E-08
**48h**	2.19E-07	8.82E-08	3.60E-07	2.53E-07
**72h**	1.45E-07	8.49E-08	1.64E-07	6.16E-08
	cgd7_5370			
**2h**	9.72E-07	1.49E-07	7.47E-08	5.68E-07
**6h**	8.20E-07	2.50E-06	1.19E-06	1.75E-06
**12h**	1.47E-08	3.93E-08	3.60E-07	7.12E-08
**24h**	6.23E-07	1.04E-06	2.06E-06	2.05E-07
**36h**	3.05E-07	4.47E-07	4.56E-07	1.20E-07
**48h**	4.81E-07	4.48E-07	6.84E-07	3.35E-07
**72h**	3.40E-07	2.12E-07	2.34E-07	9.59E-08
	cgd7_5380			
**2h**	0.00E+00	0.00E+00	0.00E+00	0.00E+00
**6h**	2.09E-08	6.42E-09	4.46E-08	1.53E-08
**12h**	1.73E-09	0.00E+00	2.67E-09	1.30E-10
**24h**	4.37E-09	4.83E-09	2.41E-08	1.32E-08
**36h**	2.61E-08	8.75E-08	2.13E-08	1.38E-08
**48h**	5.52E-08	4.86E-08	4.41E-08	2.64E-08
**72h**	4.78E-08	4.73E-08	1.81E-07	2.77E-08
	cgd7_5390			
**2h**	0.00E+00	0.00E+00	0.00E+00	0.00E+00
**6h**	0.00E+00	0.00E+00	0.00E+00	0.00E+00
**12h**	3.06E-06	0.00E+00	5.29E-06	1.21E-06
**24h**	3.87E-06	2.41E-06	3.63E-06	2.55E-06
**36h**	6.00E-06	8.30E-06	8.83E-06	3.31E-06
**48h**	8.09E-06	3.85E-06	4.52E-05	9.82E-06
**72h**	7.39E-06	3.90E-06	6.13E-05	1.15E-05
	cgd7_5410			
**2h**	1.20E-07	5.11E-08	1.25E-07	2.95E-07
**6h**	2.83E-07	4.65E-07	8.92E-07	3.92E-07
**12h**	1.21E-08	8.55E-08	5.42E-07	5.15E-08
**24h**	1.52E-07	1.25E-07	8.11E-07	1.63E-07

**36h**	2.44E-07	3.49E-07	4.38E-07	1.68E-07
**48h**	1.92E-07	1.43E-07	4.65E-07	2.04E-07
**72h**	1.00E-07	1.29E-07	2.59E-07	8.15E-08
	cgd7_5420			
**2h**	6.43E-05	1.25E-05	0.00E+00	3.68E-04
**6h**	1.15E-04	2.47E-05	2.31E-04	6.11E-05
**12h**	8.91E-06	1.64E-05	3.10E-05	5.04E-05
**24h**	2.57E-05	1.34E-05	1.20E-04	5.39E-05
**36h**	2.42E-05	5.39E-05	6.29E-05	7.17E-05
**48h**	1.55E-05	1.76E-05	5.25E-05	3.07E-05
**72h**	8.16E-06	1.91E-05	1.17E-05	6.54E-06
	cgd7_5440			
**2h**	8.97E-04	1.10E-04	2.72E-05	1.71E-04
**6h**	5.05E-04	3.85E-04	1.01E-03	1.43E-04
**12h**	5.58E-05	7.22E-05	4.53E-04	3.30E-05
**24h**	1.13E-04	1.54E-04	4.54E-04	1.61E-04
**36h**	1.94E-04	3.09E-04	1.59E-04	8.44E-05
**48h**	4.90E-04	1.50E-04	9.58E-05	4.65E-05
**72h**	3.98E-05	4.00E-05	1.77E-05	1.40E-05
	cgd7_5450			
**2h**	0.00E+00	0.00E+00	0.00E+00	0.00E+00
**6h**	0.00E+00	0.00E+00	0.00E+00	0.00E+00
**12h**	0.00E+00	6.73E-07	0.00E+00	2.49E-08
**24h**	1.13E-06	3.47E-07	1.41E-06	0.00E+00
**36h**	3.53E-05	2.45E-05	4.77E-06	2.07E-06
**48h**	1.19E-03	1.90E-03	8.84E-04	2.79E-04
**72h**	5.18E-04	1.08E-03	6.76E-04	5.46E-04
	cgd7_5460			
**2h**	1.05E-04	1.26E-05	1.71E-05	2.61E-05
**6h**	2.42E-05	1.60E-05	2.07E-04	5.79E-05
**12h**	1.41E-07	1.22E-05	2.09E-05	2.05E-06
**24h**	7.73E-06	1.95E-05	1.39E-04	5.12E-05
**36h**	6.63E-05	1.00E-04	2.69E-05	1.70E-05
**48h**	1.17E-04	4.04E-05	3.56E-05	3.59E-05
**72h**	3.90E-05	1.32E-05	1.26E-05	3.47E-06
	cgd7_5470			
**2h**	9.85E-07	5.50E-07	1.11E-06	9.45E-07
**6h**	2.85E-07	3.94E-07	3.69E-07	1.86E-07
**12h**	7.58E-07	1.65E-06	2.09E-06	1.22E-07
**24h**	3.34E-07	6.38E-07	6.11E-07	1.95E-07
**36h**	1.61E-07	2.22E-07	2.09E-07	7.28E-08
**48h**	5.38E-07	3.61E-07	3.88E-07	1.68E-07
**72h**	4.70E-08	6.16E-08	1.03E-07	4.55E-08
	cgd7_5480			
**2h**	6.64E-04	1.28E-04	3.91E-05	1.10E-04
**6h**	9.24E-05	1.28E-04	9.85E-05	5.53E-05
**12h**	4.27E-06	1.18E-04	4.92E-05	1.30E-05
**24h**	4.61E-05	2.28E-04	7.49E-05	3.13E-05
**36h**	1.72E-04	2.72E-04	8.55E-05	4.64E-05
**48h**	1.20E-04	7.31E-05	5.45E-05	7.25E-05
**72h**	4.67E-05	1.42E-05	5.40E-05	6.16E-06
	cgd7_5490			
**2h**	3.34E-04	2.57E-06	4.82E-06	2.93E-06

**6h**	2.55E-07	1.09E-07	1.55E-07	9.86E-08
**12h**	4.65E-07	8.12E-07	4.77E-07	6.16E-07
**24h**	1.39E-06	8.22E-07	1.34E-06	1.98E-07
**36h**	7.21E-07	9.21E-06	4.45E-07	4.87E-07
**48h**	9.84E-07	3.51E-07	6.13E-07	4.45E-07
**72h**	3.99E-07	1.44E-07	2.66E-07	3.15E-06
	cgd8_470			
**2h**	0.00E+00	0.00E+00	0.00E+00	0.00E+00
**6h**	1.63E-08	4.68E-05	7.35E-08	8.18E-04
**12h**	2.23E-05	1.83E-05	2.98E-05	1.58E-03
**24h**	9.79E-05	5.51E-05	6.98E-05	1.29E-03
**36h**	1.88E-04	1.84E-04	2.13E-04	6.41E-03
**48h**	2.56E-04	6.77E-05	1.38E-04	9.91E-03
**72h**	5.34E-04	2.67E-04	6.43E-04	4.14E-03
	cgd8_480			
**2h**	1.93E-07	1.47E-07	3.87E-07	2.40E-04
**6h**	2.46E-06	6.24E-06	4.60E-06	2.36E-03
**12h**	1.00E-06	1.17E-06	1.06E-06	1.10E-03
**24h**	3.44E-06	2.13E-06	3.18E-06	3.03E-03
**36h**	1.82E-06	2.69E-06	4.10E-06	9.30E-04
**48h**	2.12E-06	1.34E-06	2.16E-06	1.63E-03
**72h**	8.71E-07	1.15E-06	3.04E-06	3.59E-04
	cgd8_490			
**2h**	0.00E+00	0.00E+00	0.00E+00	0.00E+00
**6h**	0.00E+00	1.08E-07	8.63E-09	0.00E+00
**12h**	3.02E-05	1.51E-05	8.25E-06	1.03E-03
**24h**	2.05E-05	2.31E-05	1.67E-05	1.27E-03
**36h**	3.89E-05	3.46E-05	5.05E-05	1.02E-03
**48h**	6.88E-05	3.25E-05	7.18E-05	2.02E-03
**72h**	9.22E-05	4.31E-05	1.19E-04	4.42E-04
	cgd8_510			
**2h**	9.85E-06	1.16E-05	1.91E-05	1.01E-03
**6h**	5.25E-06	9.49E-06	1.68E-05	1.82E-03
**12h**	1.37E-05	5.95E-06	1.25E-05	9.33E-03
**24h**	1.18E-05	1.37E-05	9.97E-06	3.10E-03
**36h**	8.67E-06	9.44E-06	7.90E-06	2.22E-03
**48h**	5.48E-06	2.36E-06	6.00E-06	8.60E-03
**72h**	1.44E-06	3.00E-06	5.06E-06	1.49E-03
	cgd8_590			
**2h**	1.81E-07	1.47E-08	1.21E-08	0.00E+00
**6h**	7.63E-07	4.18E-06	1.56E-06	8.73E-03
**12h**	3.07E-07	1.09E-07	6.35E-08	4.61E-04
**24h**	2.63E-07	5.97E-07	1.70E-06	7.07E-03
**36h**	8.84E-07	1.52E-06	2.22E-06	2.62E-03
**48h**	9.98E-07	3.46E-07	1.09E-06	2.50E-03
**72h**	7.70E-07	8.81E-07	1.37E-06	0.00E+00
	cgd8_600			
**2h**	4.05E-03	1.56E-03	3.27E-03	1.75E-02
**6h**	8.94E-04	2.21E-03	1.72E-03	1.42E-02
**12h**	1.78E-03	8.35E-04	1.16E-03	3.95E-02
**24h**	3.41E-03	4.29E-03	2.51E-03	7.12E-02
**36h**	1.42E-03	1.13E-03	1.10E-03	2.00E-02
**48h**	1.01E-03	9.76E-04	1.53E-03	2.46E-02

**72h**	2.52E-04	4.02E-04	7.29E-04	4.17E-03
	cgd8_610			
**2h**	0.00E+00	3.17E-06	8.05E-06	0.00E+00
**6h**	3.60E-05	1.43E-04	5.60E-05	2.73E-04
**12h**	5.74E-05	5.80E-06	7.61E-06	1.03E-03
**24h**	5.70E-05	3.17E-05	1.35E-04	2.41E-03
**36h**	1.58E-04	7.32E-05	7.36E-05	4.27E-03
**48h**	2.10E-04	5.51E-05	1.62E-04	2.89E-03
**72h**	5.51E-04	3.56E-05	8.89E-05	6.30E-04
	cgd8_620			
**2h**	0.00E+00	0.00E+00	0.00E+00	0.00E+00
**6h**	0.00E+00	0.00E+00	0.00E+00	0.00E+00
**12h**	0.00E+00	0.00E+00	6.11E-09	0.00E+00
**24h**	1.11E-07	5.52E-08	3.44E-08	1.97E-04
**36h**	4.98E-07	8.56E-07	3.49E-07	3.65E-04
**48h**	1.14E-04	1.67E-05	2.65E-05	9.67E-03
**72h**	4.18E-05	1.07E-05	2.34E-05	3.56E-03
	cgd8_630			
**2h**	2.36E-07	1.34E-08	1.13E-07	0.00E+00
**6h**	3.07E-07	2.10E-06	8.97E-07	2.72E-04
**12h**	8.82E-08	0.00E+00	1.13E-07	1.62E-03
**24h**	2.21E-07	1.10E-07	1.37E-06	5.69E-03
**36h**	1.97E-06	9.00E-07	1.01E-06	1.05E-03
**48h**	2.47E-06	5.81E-07	1.35E-06	3.82E-03
**72h**	5.31E-07	1.19E-06	1.60E-06	3.92E-04
	cgd8_640			
**2h**	3.16E-08	2.38E-07	1.86E-07	8.44E-05
**6h**	5.70E-08	2.30E-06	7.24E-07	9.14E-03
**12h**	3.80E-06	1.87E-06	1.51E-06	1.04E-02
**24h**	1.89E-06	2.38E-06	4.52E-06	3.74E-02
**36h**	3.23E-06	8.25E-06	6.19E-06	2.53E-02
**48h**	8.46E-06	4.14E-06	1.30E-05	1.89E-02
**72h**	6.17E-06	3.04E-06	5.41E-06	8.67E-03
	cgd8_660			
**2h**	0.00E+00	0.00E+00	0.00E+00	-1.17E+10
**6h**	0.00E+00	0.00E+00	0.00E+00	-1.29E+09
**12h**	2.32E-04	1.04E-04	4.62E-05	5.52E-03
**24h**	4.20E-05	4.24E-05	2.34E-05	2.07E-03
**36h**	9.27E-05	6.49E-05	9.93E-05	8.17E-03
**48h**	4.75E-05	2.67E-05	4.16E-05	9.00E-03
**72h**	1.15E-05	5.24E-06	1.02E-05	2.05E-03
	cgd8_710			
**2h**	0.00E+00	0.00E+00	0.00E+00	0.00E+00
**6h**	0.00E+00	0.00E+00	0.00E+00	0.00E+00
**12h**	8.92E-06	5.02E-06	0.00E+00	1.33E-02
**24h**	4.58E-06	2.48E-06	2.96E-02	3.69E-03
**36h**	7.16E-05	3.39E-05	4.96E-03	1.90E-02
**48h**	2.18E-05	1.10E-05	0.00E+00	3.97E-02
**72h**	4.14E-05	1.60E-05	7.00E-03	4.24E-02
	cgd8_730			
**2h**	0.00E+00	0.00E+00	1.14E-08	9.88E-09
**6h**	0.00E+00	0.00E+00	0.00E+00	0.00E+00
**12h**	0.00E+00	0.00E+00	0.00E+00	6.90E-10

**24h**	0.00E+00	0.00E+00	8.18E-10	0.00E+00
**36h**	3.93E-10	3.38E-09	6.24E-09	1.00E-08
**48h**	9.24E-07	1.29E-06	4.49E-06	4.40E-06
**72h**	8.88E-07	1.66E-06	2.23E-06	9.60E-07
	cgd8_740			
**2h**	2.02E-06	7.40E-06	9.47E-07	1.79E-05
**6h**	4.56E-05	2.74E-05	2.01E-05	3.67E-05
**12h**	1.36E-07	1.09E-06	5.20E-06	4.19E-06
**24h**	2.23E-05	4.01E-05	1.95E-05	6.08E-05
**36h**	1.65E-05	1.77E-05	5.20E-06	2.62E-05
**48h**	2.56E-05	8.19E-06	1.18E-05	1.78E-05
**72h**	4.52E-06	3.20E-06	1.49E-06	3.35E-06
	cgd8_770			
**2h**	0.00E+00	0.00E+00	0.00E+00	0.00E+00
**6h**	1.87E-07	1.57E-06	2.72E-07	3.76E-07
**12h**	5.59E-06	1.48E-05	9.22E-07	3.33E-06
**24h**	6.02E-06	2.51E-05	1.18E-05	2.28E-05
**36h**	3.47E-05	4.56E-05	1.37E-05	3.29E-05
**48h**	1.23E-04	4.30E-05	3.57E-05	5.72E-05
**72h**	1.26E-04	9.19E-05	7.03E-05	4.83E-05
	cgd8_780			
**2h**	0.00E+00	0.00E+00	0.00E+00	0.00E+00
**6h**	0.00E+00	0.00E+00	5.77E-09	0.00E+00
**12h**	0.00E+00	0.00E+00	0.00E+00	2.00E-09
**24h**	4.44E-09	9.03E-11	2.88E-09	2.46E-08
**36h**	8.73E-09	2.24E-08	7.62E-09	1.85E-08
**48h**	2.77E-08	8.91E-09	4.55E-08	4.84E-08
**72h**	1.37E-07	2.56E-06	5.68E-07	4.40E-07
	cgd8_800			
**2h**	2.56E-05	4.71E-05	3.51E-07	5.51E-06
**6h**	1.09E-04	8.54E-05	1.14E-04	9.77E-05
**12h**	8.30E-07	9.68E-06	3.24E-06	1.40E-05
**24h**	1.64E-05	3.80E-05	9.76E-05	2.28E-04
**36h**	3.35E-05	7.14E-05	2.61E-05	4.15E-05
**48h**	4.73E-05	2.67E-05	4.95E-05	2.91E-05
**72h**	2.27E-04	2.04E-04	3.81E-04	1.33E-04
	cgd8_810			
**2h**	1.64E-08	2.98E-08	1.64E-08	1.81E-08
**6h**	1.29E-07	1.64E-07	7.86E-07	1.03E-06
**12h**	2.31E-07	3.58E-07	2.89E-07	5.40E-07
**24h**	1.69E-07	2.47E-07	7.39E-07	1.19E-06
**36h**	1.17E-06	2.15E-06	9.23E-07	3.16E-06
**48h**	1.12E-06	9.76E-07	1.31E-06	1.24E-06
**72h**	1.64E-06	3.37E-06	5.94E-06	2.35E-06
	cgd8_820			
**2h**	0.00E+00	0.00E+00	0.00E+00	0.00E+00
**6h**	0.00E+00	0.00E+00	0.00E+00	3.89E-09
**12h**	1.22E-06	6.94E-06	7.63E-07	1.48E-06
**24h**	2.30E-07	7.53E-07	1.38E-07	3.51E-07
**36h**	2.51E-07	7.21E-07	9.66E-07	3.00E-06
**48h**	1.68E-06	2.29E-06	1.50E-06	2.04E-06
**72h**	2.97E-07	2.78E-07	1.35E-06	3.82E-07
	cgd8_840			

**2h**	0.00E+00	0.00E+00	0.00E+00	0.00E+00
**6h**	1.05E-07	5.86E-07	7.60E-07	1.14E-06
**12h**	6.47E-09	2.35E-07	2.31E-08	4.04E-08
**24h**	1.39E-07	2.72E-07	3.51E-07	1.60E-06
**36h**	3.20E-07	6.40E-07	3.11E-07	7.61E-07
**48h**	3.04E-07	3.14E-07	8.77E-07	6.01E-07
**72h**	2.10E-07	2.28E-07	1.56E-07	1.12E-07
	cgd8_860			
**2h**	1.23E-04	8.07E-05	1.03E-05	1.87E-04
**6h**	3.37E-05	4.47E-05	1.02E-04	1.02E-04
**12h**	1.29E-05	5.53E-05	5.47E-05	5.73E-05
**24h**	1.08E-04	3.17E-04	1.59E-04	1.47E-04
**36h**	1.74E-04	2.23E-04	5.27E-05	2.12E-04
**48h**	4.46E-04	6.61E-04	5.11E-04	5.38E-04
**72h**	5.41E-04	3.27E-04	2.89E-04	2.33E-04
	cgd8_880			
**2h**	1.45E-03	1.31E-03	1.37E-04	1.31E-03
**6h**	8.05E-05	4.15E-04	4.60E-04	6.83E-04
**12h**	3.08E-04	1.49E-03	5.19E-04	5.92E-04
**24h**	3.96E-04	9.43E-04	3.41E-04	8.20E-04
**36h**	1.21E-04	4.59E-04	1.05E-04	3.34E-04
**48h**	5.03E-04	2.52E-04	3.77E-04	4.93E-04
**72h**	6.00E-04	3.45E-04	4.37E-04	3.15E-04
	cgd8_900			
**2h**	8.39E-03	1.06E-02	3.12E-03	4.12E-02
**6h**	6.75E-03	2.05E-03	1.18E-03	1.75E-02
**12h**	7.59E-03	1.77E-03	6.18E-03	1.39E-02
**24h**	5.64E-04	4.41E-04	4.05E-03	2.07E-02
**36h**	4.61E-04	1.18E-03	2.52E-03	1.15E-02
**48h**	1.12E-03	2.92E-04	1.64E-03	1.19E-02
**72h**	3.50E-04	1.35E-03	2.71E-03	3.63E-03
	cgd8_910			
**2h**	4.76E-07	4.30E-07	5.96E-08	8.11E-07
**6h**	3.75E-07	1.78E-07	1.51E-07	3.98E-07
**12h**	6.25E-08	4.16E-09	7.49E-08	4.22E-07
**24h**	1.19E-07	2.93E-07	1.40E-07	1.35E-06
**36h**	2.02E-07	3.89E-07	1.93E-07	6.82E-07
**48h**	1.42E-06	1.02E-06	2.94E-06	2.50E-06
**72h**	2.32E-06	2.64E-06	5.77E-06	3.25E-06
	cgd8_920			
**2h**	0.00E+00	0.00E+00	0.00E+00	0.00E+00
**6h**	0.00E+00	0.00E+00	0.00E+00	0.00E+00
**12h**	0.00E+00	1.66E-09	0.00E+00	1.18E-09
**24h**	1.05E-09	5.65E-09	2.62E-09	2.90E-09
**36h**	5.88E-08	7.40E-08	6.05E-08	3.77E-08
**48h**	1.76E-06	1.10E-06	9.79E-07	1.55E-06
**72h**	2.63E-06	1.02E-06	6.35E-06	4.73E-06
	cgd8_930			
**2h**	0.00E+00	0.00E+00	0.00E+00	0.00E+00
**6h**	4.68E-08	3.92E-07	9.53E-08	8.76E-08
**12h**	4.69E-09	0.00E+00	2.05E-09	1.72E-09
**24h**	3.23E-08	2.05E-08	6.45E-08	2.13E-08
**36h**	4.44E-08	7.62E-08	3.56E-08	2.06E-08

**48h**	6.38E-08	4.87E-08	7.93E-08	6.04E-08
**72h**	9.49E-08	5.69E-08	4.77E-08	1.19E-07
	cgd8_950			
**2h**	2.56E-05	4.39E-04	0.00E+00	0.00E+00
**6h**	6.17E-04	4.37E-04	3.06E-03	2.53E-05
**12h**	1.05E-04	1.26E-04	2.86E-04	6.64E-04
**24h**	9.82E-05	2.68E-04	1.24E-03	1.78E-04
**36h**	2.70E-04	5.69E-04	1.73E-04	6.66E-04
**48h**	5.12E-05	3.74E-04	1.21E-03	1.61E-04
**72h**	8.41E-05	1.43E-04	1.92E-04	9.08E-05
	cgd8_1000			
**2h**	1.95E-06	0.00E+00	7.04E-07	1.31E-06
**6h**	5.74E-07	6.17E-06	2.52E-07	2.92E-08
**12h**	3.60E-07	0.00E+00	2.34E-07	1.13E-07
**24h**	2.03E-06	1.73E-07	1.46E-06	3.94E-07
**36h**	7.69E-06	3.06E-07	2.59E-07	1.26E-06
**48h**	3.19E-04	1.20E-05	6.28E-05	8.62E-05
**72h**	5.99E-04	1.48E-04	1.38E-04	6.91E-04
	cgd8_1010			
**2h**	1.86E-08	2.99E-09	0.00E+00	4.54E-08
**6h**	5.45E-09	1.24E-09	0.00E+00	1.77E-08
**12h**	0.00E+00	1.21E-09	0.00E+00	5.08E-09
**24h**	5.11E-09	9.52E-09	8.81E-09	2.73E-09
**36h**	1.33E-08	2.45E-08	3.30E-08	3.23E-08
**48h**	1.21E-06	4.82E-07	1.05E-06	1.02E-06
**72h**	2.01E-06	1.03E-06	3.57E-06	5.65E-06
	cgd8_1020			
**2h**	5.35E-06	1.46E-07	0.00E+00	7.67E-07
**6h**	0.00E+00	1.06E-06	0.00E+00	1.27E-06
**12h**	0.00E+00	0.00E+00	3.74E-07	2.61E-07
**24h**	1.68E-05	2.37E-06	1.51E-06	1.54E-06
**36h**	2.44E-05	2.11E-05	7.67E-06	2.70E-05
**48h**	6.86E-04	3.91E-04	1.00E-03	2.01E-03
**72h**	1.00E-03	1.36E-03	2.62E-03	3.53E-03
	cgd8_1030			
**2h**	2.01E-06	1.46E-06	5.73E-08	6.34E-07
**6h**	2.70E-05	4.15E-05	3.22E-05	2.21E-05
**12h**	4.79E-08	1.07E-07	4.27E-06	2.21E-06
**24h**	1.33E-05	8.70E-06	3.20E-05	1.12E-05
**36h**	2.69E-05	1.93E-05	9.34E-06	8.33E-06
**48h**	4.37E-05	8.82E-06	1.80E-05	2.53E-05
**72h**	3.79E-05	1.55E-05	1.57E-05	3.73E-05
	cgd8_1040			
**2h**	2.35E-08	4.72E-09	0.00E+00	0.00E+00
**6h**	0.00E+00	5.53E-08	5.35E-08	3.88E-09
**12h**	0.00E+00	0.00E+00	1.97E-08	1.00E-09
**24h**	1.47E-08	1.43E-08	8.01E-08	1.84E-08
**36h**	1.14E-07	4.72E-08	4.11E-08	4.33E-08
**48h**	1.28E-07	1.36E-07	7.76E-08	2.24E-07
**72h**	1.08E-07	1.94E-07	5.09E-07	4.70E-07
	cgd8_1050			
**2h**	1.29E-03	9.61E-04	3.30E-03	9.93E-03
**6h**	9.42E-04	3.25E-04	1.32E-03	2.31E-04

**12h**	2.96E-03	4.39E-03	5.40E-03	1.61E-02
**24h**	1.77E-03	1.21E-03	1.02E-03	3.07E-04
**36h**	6.33E-04	6.90E-04	8.81E-04	2.13E-04
**48h**	9.95E-05	6.61E-04	2.04E-03	3.32E-04
**72h**	5.04E-04	2.74E-04	8.53E-04	1.59E-03
	cgd8_1060			
**2h**	5.17E-03	1.66E-03	1.64E-03	3.49E-03
**6h**	2.13E-04	1.18E-04	1.61E-04	2.29E-04
**12h**	4.79E-03	2.88E-03	4.82E-03	1.80E-03
**24h**	3.54E-03	1.17E-03	1.06E-03	4.79E-04
**36h**	1.03E-03	5.38E-04	4.40E-04	7.59E-04
**48h**	3.91E-04	2.58E-04	3.49E-04	4.88E-04
**72h**	4.14E-04	2.14E-04	1.65E-04	3.06E-04
	cgd8_1080			
**2h**	1.74E-07	6.72E-07	0.00E+00	0.00E+00
**6h**	2.96E-06	1.02E-06	5.26E-07	1.32E-06
**12h**	2.12E-06	3.82E-06	2.71E-06	3.29E-06
**24h**	1.85E-06	3.40E-06	1.43E-05	4.50E-06
**36h**	1.25E-05	1.78E-05	6.78E-06	1.42E-05
**48h**	2.86E-05	9.17E-06	1.64E-05	2.28E-05
**72h**	1.89E-05	1.26E-05	2.03E-05	2.78E-05
	cgd8_1120			
**2h**	2.55E-05	1.27E-06	2.50E-06	4.31E-06
**6h**	7.50E-07	5.38E-07	6.12E-07	9.23E-07
**12h**	1.18E-06	2.39E-06	1.97E-06	3.03E-06
**24h**	2.17E-06	2.13E-06	1.70E-06	6.35E-07
**36h**	9.21E-07	1.79E-06	1.11E-06	1.56E-06
**48h**	1.99E-06	4.57E-07	9.37E-07	2.32E-06
**72h**	9.53E-07	5.07E-07	1.75E-06	2.18E-06
	cgd8_1160			
**2h**	1.12E-07	5.71E-07	7.06E-07	6.88E-07
**6h**	3.73E-09	2.05E-09	8.20E-08	5.97E-08
**12h**	4.92E-10	3.44E-08	7.96E-07	9.26E-09
**24h**	7.78E-08	1.15E-07	2.98E-07	5.38E-08
**36h**	1.51E-06	1.58E-06	6.87E-07	8.94E-07
**48h**	1.11E-04	3.27E-05	1.15E-04	3.97E-05
**72h**	1.49E-06	1.07E-06	4.76E-06	4.07E-06
	cgd8_1170			
**2h**	1.20E-07	1.56E-07	1.48E-07	1.58E-07
**6h**	1.50E-06	4.89E-07	9.57E-07	9.39E-07
**12h**	1.94E-07	2.22E-07	1.16E-07	8.46E-08
**24h**	3.68E-07	3.77E-07	7.16E-07	7.63E-07
**36h**	8.69E-07	5.93E-07	5.94E-07	3.01E-07
**48h**	1.46E-06	2.76E-07	1.27E-06	7.06E-07
**72h**	3.51E-07	3.41E-07	8.56E-07	5.20E-07
	cgd8_1180			
**2h**	0.00E+00	0.00E+00	8.95E-09	0.00E+00
**6h**	2.38E-09	9.20E-09	0.00E+00	7.22E-09
**12h**	1.15E-06	1.45E-06	7.98E-07	1.05E-06
**24h**	2.31E-07	1.83E-07	1.00E-07	1.50E-07
**36h**	8.29E-08	9.04E-08	1.52E-07	3.08E-07
**48h**	6.13E-08	1.92E-08	3.99E-08	2.67E-08
**72h**	1.90E-08	1.78E-08	1.84E-08	1.18E-08

	cgd8_1200			
**2h**	1.30E-06	6.79E-06	2.97E-06	6.28E-06
**6h**	3.35E-06	2.42E-05	2.51E-05	1.43E-05
**12h**	4.79E-07	1.87E-06	1.42E-05	4.32E-06
**24h**	5.89E-06	1.00E-05	9.81E-06	9.17E-06
**36h**	3.20E-06	1.07E-05	9.09E-06	3.39E-06
**48h**	8.31E-06	5.83E-06	1.93E-05	5.85E-06
**72h**	2.58E-06	5.73E-06	2.96E-05	9.73E-05
	cgd8_1220			
**2h**	0.00E+00	0.00E+00	0.00E+00	0.00E+00
**6h**	0.00E+00	0.00E+00	0.00E+00	0.00E+00
**12h**	2.65E-09	2.79E-09	0.00E+00	0.00E+00
**24h**	1.27E-09	1.32E-08	5.04E-09	2.83E-09
**36h**	3.44E-08	2.38E-08	2.76E-08	2.49E-08
**48h**	2.67E-07	1.70E-07	2.95E-07	3.86E-07
**72h**	1.13E-06	6.45E-07	5.82E-06	3.65E-06
	cgd8_1230			
**2h**	0.00E+00	0.00E+00	0.00E+00	0.00E+00
**6h**	0.00E+00	3.34E-09	1.53E-08	0.00E+00
**12h**	9.67E-09	1.04E-08	6.85E-09	1.09E-08
**24h**	1.78E-08	5.17E-08	1.33E-07	6.73E-08
**36h**	2.23E-07	1.32E-07	1.63E-07	1.12E-07
**48h**	1.44E-07	7.48E-08	3.54E-07	2.38E-07
**72h**	8.57E-08	6.81E-08	3.61E-07	2.02E-07
	cgd8_1240			
**2h**	0.00E+00	0.00E+00	0.00E+00	0.00E+00
**6h**	5.53E-09	2.38E-08	3.57E-08	1.88E-09
**12h**	1.16E-08	4.61E-08	1.81E-07	4.01E-08
**24h**	6.99E-08	1.27E-07	7.62E-07	2.39E-07
**36h**	3.02E-07	8.31E-07	4.92E-07	2.27E-07
**48h**	4.14E-07	1.42E-07	7.11E-07	3.11E-07
**72h**	5.03E-08	6.49E-08	1.27E-07	1.59E-07
	cgd8_1260			
**2h**	1.12E-08	1.09E-07	0.00E+00	0.00E+00
**6h**	7.96E-08	2.67E-06	7.56E-06	8.34E-07
**12h**	2.61E-06	2.84E-05	1.03E-05	5.41E-06
**24h**	2.01E-06	8.27E-06	3.41E-05	8.33E-06
**36h**	8.53E-06	6.89E-05	1.77E-05	1.62E-05
**48h**	4.95E-05	3.67E-05	1.13E-04	2.76E-05
**72h**	7.12E-06	3.22E-05	9.04E-05	3.62E-05
	cgd8_1270			
**2h**	1.81E-06	1.80E-06	2.67E-07	2.37E-06
**6h**	2.17E-06	6.18E-06	6.44E-06	8.29E-06
**12h**	1.23E-07	1.38E-06	2.57E-06	7.56E-07
**24h**	1.63E-06	1.20E-06	8.28E-06	5.06E-05
**36h**	4.29E-06	5.33E-06	9.96E-06	4.49E-06
**48h**	4.92E-06	1.24E-06	4.41E-06	2.28E-06
**72h**	2.08E-07	4.31E-07	1.31E-06	5.60E-07
	cgd8_1290			
**2h**	2.06E-07	2.25E-07	0.00E+00	7.67E-08
**6h**	1.10E-07	1.55E-07	3.03E-07	6.69E-08
**12h**	4.20E-09	1.73E-09	5.91E-08	9.61E-09
**24h**	5.53E-08	1.27E-07	3.06E-07	1.91E-07

**36h**	8.00E-08	1.72E-07	8.67E-08	4.53E-08
**48h**	1.17E-07	4.19E-08	1.79E-07	5.28E-08
**72h**	1.15E-08	2.84E-08	5.52E-08	6.72E-08
	cgd8_1330			
**2h**	1.28E-07	2.45E-08	3.37E-09	4.65E-08
**6h**	2.03E-07	2.33E-07	1.69E-07	1.52E-07
**12h**	2.08E-08	1.02E-07	6.87E-08	1.15E-08
**24h**	3.22E-08	4.60E-08	1.39E-07	7.38E-08
**36h**	2.00E-08	1.14E-07	3.55E-08	3.99E-08
**48h**	9.62E-08	2.84E-08	7.45E-08	1.74E-08
**72h**	1.35E-08	2.32E-08	4.91E-08	3.53E-08
	cgd8_1350			
**2h**	0.00E+00	0.00E+00	0.00E+00	0.00E+00
**6h**	0.00E+00	6.58E-10	0.00E+00	0.00E+00
**12h**	5.66E-08	1.29E-07	5.84E-08	6.35E-08
**24h**	1.77E-08	9.70E-09	9.72E-09	8.82E-09
**36h**	3.21E-08	2.66E-08	4.53E-08	2.26E-08
**48h**	1.24E-07	7.03E-08	1.75E-07	5.05E-08
**72h**	4.25E-08	4.21E-08	1.29E-07	1.01E-07
	cgd8_1360			
**2h**	7.74E-06	1.83E-06	8.17E-06	7.26E-06
**6h**	1.63E-06	1.59E-06	5.83E-04	7.60E-06
**12h**	2.17E-06	5.33E-05	9.29E-06	4.40E-06
**24h**	4.18E-06	2.26E-06	3.26E-06	7.45E-06
**36h**	1.14E-06	3.65E-06	6.96E-06	5.95E-06
**48h**	1.78E-06	3.15E-06	3.25E-06	4.96E-06
**72h**	2.53E-07	1.28E-06	4.35E-07	2.16E-06
	cgd8_1370			
**2h**	0.00E+00	0.00E+00	3.77E-07	0.00E+00
**6h**	0.00E+00	0.00E+00	1.06E-05	1.49E-05
**12h**	2.40E-05	2.68E-05	2.38E-05	4.51E-08
**24h**	2.13E-06	1.56E-05	1.33E-05	1.73E-05
**36h**	7.66E-07	1.60E-04	2.87E-05	7.07E-06
**48h**	1.47E-06	2.24E-05	1.34E-05	2.02E-06
**72h**	4.28E-06	1.21E-04	1.47E-05	8.22E-06
	cgd8_1380			
**2h**	6.40E-07	6.45E-08	4.52E-07	1.36E-06
**6h**	2.98E-07	7.82E-07	1.90E-06	1.58E-06
**12h**	1.85E-07	1.04E-06	6.10E-08	5.87E-07
**24h**	3.35E-07	4.92E-07	9.73E-08	8.80E-07
**36h**	2.00E-07	5.78E-07	7.03E-08	2.98E-06
**48h**	1.10E-07	1.30E-07	8.42E-08	4.13E-07
**72h**	5.37E-08	2.38E-07	8.01E-08	6.98E-07
	cgd8_1400			
**2h**	6.22E-07	1.24E-07	6.86E-07	1.33E-06
**6h**	6.08E-07	1.14E-06	9.07E-07	8.47E-07
**12h**	2.75E-08	1.46E-07	2.42E-08	4.53E-08
**24h**	2.10E-07	6.05E-07	2.04E-07	4.82E-07
**36h**	1.62E-07	4.43E-07	3.56E-07	2.37E-06
**48h**	1.38E-07	5.42E-07	1.54E-07	1.02E-06
**72h**	4.69E-07	1.34E-06	2.33E-07	2.90E-06
	cgd8_1430			
**2h**	4.16E-07	0.00E+00	0.00E+00	0.00E+00

**6h**	6.21E-08	2.29E-05	2.64E-05	2.31E-05
**12h**	6.52E-05	1.94E-03	1.36E-04	1.04E-04
**24h**	1.46E-05	2.01E-04	3.88E-05	2.24E-05
**36h**	1.11E-05	3.84E-04	6.65E-05	1.49E-05
**48h**	6.82E-06	1.81E-04	4.91E-05	1.75E-05
**72h**	2.08E-05	3.39E-04	2.31E-05	6.07E-05
	cgd8_1440			
**2h**	2.56E-06	2.11E-05	4.89E-05	1.16E-03
**6h**	7.66E-07	1.62E-05	5.09E-05	5.51E-04
**12h**	4.35E-07	1.69E-04	4.36E-06	8.75E-05
**24h**	3.56E-06	6.29E-05	7.71E-06	1.42E-04
**36h**	2.00E-06	2.65E-05	7.62E-06	1.07E-04
**48h**	1.32E-06	3.82E-05	1.31E-05	3.88E-05
**72h**	5.43E-06	9.60E-05	1.61E-05	8.76E-05
	cgd8_1450			
**2h**	3.15E-06	3.78E-06	1.95E-05	1.08E-04
**6h**	1.13E-06	2.45E-06	3.78E-06	4.22E-05
**12h**	2.20E-07	1.44E-05	4.69E-07	1.74E-05
**24h**	1.90E-06	5.25E-06	1.27E-06	2.22E-05
**36h**	5.39E-07	1.39E-06	1.37E-06	1.73E-05
**48h**	2.65E-07	1.77E-06	1.09E-06	9.85E-06
**72h**	1.94E-07	1.46E-06	4.83E-07	4.18E-06
	cgd8_1500			
**2h**	1.03E-05	1.41E-05	8.47E-05	3.69E-03
**6h**	3.48E-06	2.53E-05	1.63E-04	1.56E-04
**12h**	1.69E-06	2.33E-05	1.18E-05	1.30E-04
**24h**	6.38E-06	7.05E-05	1.49E-05	3.20E-04
**36h**	8.36E-06	3.51E-05	5.28E-05	1.96E-04
**48h**	3.33E-06	1.81E-05	2.41E-05	6.60E-05
**72h**	6.08E-06	2.90E-05	8.10E-06	1.38E-04
	cgd8_1510			
**2h**	0.00E+00	0.00E+00	0.00E+00	0.00E+00
**6h**	5.43E-06	1.11E-04	4.10E-04	1.65E-04
**12h**	2.55E-06	7.33E-05	3.01E-05	2.31E-05
**24h**	1.64E-05	4.55E-04	8.36E-05	6.54E-05
**36h**	2.39E-05	2.40E-04	3.08E-04	2.95E-04
**48h**	3.81E-05	8.22E-04	7.19E-04	2.70E-04
**72h**	8.70E-05	1.78E-03	2.31E-04	1.18E-04
	cgd8_1520			
**2h**	0.00E+00	0.00E+00	0.00E+00	0.00E+00
**6h**	5.27E-07	2.47E-06	1.73E-06	3.33E-07
**12h**	9.26E-08	8.29E-07	4.66E-08	6.37E-08
**24h**	2.28E-07	7.02E-06	1.21E-06	3.01E-07
**36h**	5.33E-07	1.13E-06	2.17E-06	7.20E-07
**48h**	2.29E-07	1.70E-06	1.18E-06	5.88E-07
**72h**	9.21E-08	2.04E-07	4.22E-07	2.49E-07
	cgd8_1530			
**2h**	1.23E-09	0.00E+00	0.00E+00	0.00E+00
**6h**	2.24E-06	8.48E-06	3.38E-05	3.00E-06
**12h**	2.03E-07	2.37E-06	1.78E-07	2.48E-07
**24h**	5.46E-07	1.15E-05	2.33E-06	7.47E-07
**36h**	2.38E-06	2.41E-06	2.43E-06	4.61E-06
**48h**	1.61E-06	2.32E-06	3.80E-06	1.76E-06

**72h**	2.82E-07	1.85E-07	9.59E-08	3.13E-07
	cgd8_1540			
**2h**	0.00E+00	0.00E+00	0.00E+00	0.00E+00
**6h**	2.89E-08	4.29E-06	6.39E-07	5.56E-08
**12h**	1.04E-06	6.81E-06	1.19E-06	1.41E-06
**24h**	2.28E-07	1.42E-05	4.77E-06	1.29E-06
**36h**	7.04E-06	1.94E-05	1.71E-05	2.16E-05
**48h**	7.74E-07	1.07E-05	7.10E-04	1.94E-05
**72h**	2.47E-06	5.69E-06	1.33E-04	1.41E-05
	cgd8_1560			
**2h**	1.48E-06	1.66E-06	2.86E-06	1.51E-06
**6h**	1.84E-06	4.90E-07	6.20E-06	6.14E-06
**12h**	3.87E-07	3.75E-05	2.97E-06	1.27E-07
**24h**	1.95E-06	1.89E-06	1.79E-06	2.70E-05
**36h**	1.42E-05	7.91E-07	2.39E-06	0.00E+00
**48h**	1.62E-06	1.85E-06	2.27E-06	6.64E-06
**72h**	5.02E-07	1.78E-06	2.23E-06	3.43E-06
	cgd8_1600			
**2h**	1.09E-06	2.15E-07	3.03E-07	9.97E-07
**6h**	4.88E-07	9.10E-07	3.77E-06	9.28E-06
**12h**	1.06E-06	1.98E-06	1.08E-06	2.88E-04
**24h**	6.89E-07	1.27E-06	1.44E-06	2.61E-05
**36h**	8.98E-07	1.67E-06	1.38E-06	3.36E-05
**48h**	1.27E-06	2.41E-06	8.26E-07	4.75E-05
**72h**	2.43E-06	8.30E-06	2.16E-06	7.20E-05
	cgd8_1610			
**2h**	7.13E-04	3.26E-06	1.96E-04	2.07E-05
**6h**	2.52E-03	2.42E-03	1.09E-03	3.06E-03
**12h**	4.69E-04	7.93E-04	8.26E-05	5.47E-05
**24h**	2.29E-04	1.30E-03	3.03E-04	8.04E-03
**36h**	3.65E-04	7.40E-04	2.25E-04	7.00E-03
**48h**	9.59E-04	9.33E-04	4.46E-04	3.81E-03
**72h**	7.84E-04	1.69E-02	4.10E-04	2.84E-02
	cgd8_1620			
**2h**	7.57E-09	0.00E+00	0.00E+00	0.00E+00
**6h**	4.10E-07	3.39E-07	3.08E-07	2.02E-07
**12h**	6.60E-08	7.79E-08	7.28E-08	0.00E+00
**24h**	6.21E-08	1.07E-06	3.25E-07	2.44E-07
**36h**	5.79E-07	2.18E-07	8.07E-07	7.25E-06
**48h**	4.58E-07	6.51E-07	5.64E-07	4.97E-07
**72h**	2.01E-07	6.26E-07	3.66E-07	1.73E-06
	cgd8_1630			
**2h**	0.00E+00	0.00E+00	0.00E+00	0.00E+00
**6h**	1.84E-08	1.63E-07	8.90E-08	2.23E-06
**12h**	2.87E-08	1.95E-07	3.65E-07	0.00E+00
**24h**	2.13E-08	1.05E-06	4.70E-07	1.81E-06
**36h**	6.51E-07	9.84E-07	5.92E-07	1.12E-05
**48h**	4.33E-07	8.93E-07	6.24E-07	4.48E-06
**72h**	2.98E-07	1.56E-06	3.12E-07	2.90E-06
	cgd8_1650			
**2h**	7.26E-05	5.18E-05	1.79E-03	1.58E-05
**6h**	2.76E-05	1.99E-05	5.73E-04	6.40E-06
**12h**	6.91E-05	7.43E-05	6.81E-04	0.00E+00

**24h**	7.40E-05	7.59E-05	2.44E-04	4.75E-04
**36h**	6.50E-05	9.14E-05	2.33E-04	1.57E-04
**48h**	3.27E-05	7.06E-05	2.40E-04	2.47E-03
**72h**	2.49E-05	8.48E-05	1.02E-04	1.04E-02
	cgd8_1680			
**2h**	2.21E-03	1.50E-03	1.27E-02	3.13E-03
**6h**	2.70E-03	2.81E-03	8.10E-03	0.00E+00
**12h**	9.64E-04	2.97E-03	1.39E-02	8.76E-03
**24h**	1.86E-03	3.47E-03	9.26E-04	1.35E-02
**36h**	1.41E-03	1.23E-03	7.37E-04	2.65E-02
**48h**	3.42E-03	4.31E-03	9.77E-04	1.84E-02
**72h**	1.48E-03	3.16E-03	1.07E-03	3.67E-02
	cgd8_1690			
**2h**	3.16E-09	0.00E+00	0.00E+00	0.00E+00
**6h**	0.00E+00	0.00E+00	1.75E-08	0.00E+00
**12h**	2.49E-07	3.40E-07	2.98E-07	1.74E-06
**24h**	1.15E-07	1.32E-07	2.65E-08	1.05E-06
**36h**	1.59E-07	2.75E-07	9.73E-07	2.04E-06
**48h**	8.73E-06	1.69E-05	6.20E-06	9.35E-03
**72h**	7.68E-06	2.32E-05	1.27E-05	1.46E-02
	cgd8_1700			
**2h**	0.00E+00	0.00E+00	0.00E+00	0.00E+00
**6h**	0.00E+00	0.00E+00	0.00E+00	0.00E+00
**12h**	4.71E-09	1.12E-08	1.74E-08	0.00E+00
**24h**	7.56E-09	1.60E-07	3.31E-08	1.61E-08
**36h**	1.49E-07	2.97E-07	1.09E-07	1.14E-07
**48h**	9.17E-06	9.24E-06	6.11E-06	4.06E-05
**72h**	1.18E-05	4.59E-05	1.63E-05	4.15E-05
	cgd8_1710			
**2h**	1.95E-07	1.34E-07	1.92E-07	1.28E-08
**6h**	1.28E-07	1.41E-07	9.11E-08	3.51E-06
**12h**	1.17E-07	3.50E-07	8.63E-08	0.00E+00
**24h**	2.71E-07	3.13E-07	2.28E-07	1.25E-06
**36h**	3.06E-07	4.98E-07	1.45E-07	6.75E-07
**48h**	2.25E-07	3.43E-07	6.89E-08	2.41E-07
**72h**	9.90E-08	6.32E-07	3.45E-07	4.25E-07
	cgd8_1720			
**2h**	0.00E+00	0.00E+00	0.00E+00	0.00E+00
**6h**	0.00E+00	0.00E+00	0.00E+00	0.00E+00
**12h**	8.34E-09	8.16E-09	2.68E-09	0.00E+00
**24h**	1.51E-08	2.10E-08	1.72E-09	0.00E+00
**36h**	4.35E-08	1.12E-07	6.41E-08	2.04E-07
**48h**	8.26E-06	1.61E-05	1.85E-05	1.54E-05
**72h**	3.76E-06	1.62E-05	7.89E-06	9.36E-06
	cgd8_1740			
**2h**	1.09E-08	0.00E+00	2.51E-08	0.00E+00
**6h**	6.66E-08	1.72E-08	1.08E-08	0.00E+00
**12h**	0.00E+00	1.12E-08	3.71E-09	0.00E+00
**24h**	3.06E-08	1.42E-08	5.81E-08	8.36E-08
**36h**	2.97E-07	1.82E-07	1.71E-07	1.21E-06
**48h**	1.23E-06	1.14E-06	8.32E-07	3.74E-06
**72h**	8.25E-07	1.42E-06	1.36E-06	1.57E-06
	cgd7_1940			

**2h**	0.00E+00	0.00E+00	0.00E+00	0.00E+00
**6h**	0.00E+00	0.00E+00	5.23E-09	0.00E+00
**12h**	3.60E-07	4.50E-06	2.27E-06	1.34E-06
**24h**	3.09E-07	5.15E-07	7.01E-07	4.36E-07
**36h**	4.35E-07	2.13E-06	2.29E-06	4.35E-07
**48h**	3.70E-07	8.18E-07	7.47E-07	2.04E-07
**72h**	9.73E-08	2.64E-07	1.45E-07	1.27E-07
	cgd7_1950			
**2h**	0.00E+00	0.00E+00	1.01E-08	0.00E+00
**6h**	0.00E+00	0.00E+00	6.82E-08	0.00E+00
**12h**	1.85E-07	7.74E-07	5.34E-06	2.55E-05
**24h**	1.08E-06	2.14E-06	3.68E-06	3.97E-05
**36h**	9.45E-07	1.31E-05	1.48E-05	1.65E-05
**48h**	2.52E-06	2.10E-05	1.88E-05	4.70E-05
**72h**	4.99E-06	9.09E-06	1.27E-05	6.77E-05
	cgd7_1960			
**2h**	3.82E-08	0.00E+00	0.00E+00	0.00E+00
**6h**	0.00E+00	0.00E+00	9.94E-09	0.00E+00
**12h**	3.35E-04	1.11E-03	9.41E-04	2.60E-03
**24h**	2.66E-05	1.06E-04	8.31E-05	2.53E-04
**36h**	1.30E-05	2.66E-04	1.46E-04	8.45E-05
**48h**	6.84E-06	4.83E-05	2.44E-05	2.73E-05
**72h**	3.72E-06	7.65E-06	4.19E-06	2.31E-05
	cgd7_1970			
**2h**	0.00E+00	0.00E+00	0.00E+00	0.00E+00
**6h**	1.16E-05	1.61E-04	1.28E-04	9.81E-05
**12h**	1.84E-05	7.05E-05	2.71E-05	1.83E-04
**24h**	6.83E-06	2.58E-04	1.23E-04	2.67E-05
**36h**	2.09E-05	6.06E-05	6.90E-05	7.84E-05
**48h**	1.56E-05	9.11E-05	3.42E-05	6.95E-05
**72h**	7.31E-06	3.54E-05	8.49E-06	1.62E-05
	cgd7_1980			
**2h**	3.84E-07	0.00E+00	8.00E-09	2.02E-08
**6h**	1.05E-07	6.70E-06	1.26E-06	1.16E-06
**12h**	4.87E-07	8.66E-07	1.35E-06	1.63E-06
**24h**	1.14E-07	5.17E-06	3.84E-06	7.60E-07
**36h**	2.92E-06	2.87E-06	2.63E-06	3.49E-06
**48h**	4.97E-07	1.30E-06	1.33E-06	1.04E-06
**72h**	3.33E-07	5.16E-07	2.13E-07	3.30E-07
	cgd7_1990			
**2h**	1.15E-04	1.09E-04	3.25E-04	1.05E-03
**6h**	9.13E-06	3.40E-04	1.67E-04	3.47E-04
**12h**	5.44E-05	9.09E-04	3.25E-04	9.12E-04
**24h**	1.73E-05	4.61E-04	2.34E-04	4.72E-04
**36h**	8.80E-05	2.39E-04	1.27E-04	4.12E-04
**48h**	5.32E-05	2.17E-04	1.24E-04	2.95E-04
**72h**	4.69E-05	8.42E-05	6.71E-05	1.68E-04
	cgd7_2000			
**2h**	0.00E+00	0.00E+00	1.52E-05	6.77E-05
**6h**	1.71E-04	1.01E-03	2.55E-04	4.31E-05
**12h**	1.08E-04	4.29E-04	1.68E-03	8.81E-05
**24h**	3.02E-04	1.91E-03	4.51E-03	7.07E-05
**36h**	2.48E-03	7.77E-04	5.29E-03	7.11E-05

**48h**	1.48E-03	9.07E-04	7.36E-03	8.87E-05
**72h**	9.66E-04	5.23E-04	2.37E-03	1.15E-04
	cgd7_2010			
**2h**	0.00E+00	0.00E+00	0.00E+00	0.00E+00
**6h**	0.00E+00	1.59E-08	3.05E-09	4.29E-09
**12h**	1.80E-07	2.40E-07	4.74E-07	2.60E-07
**24h**	3.87E-08	3.32E-07	5.91E-07	5.69E-08
**36h**	9.11E-07	6.85E-07	9.55E-07	7.51E-07
**48h**	3.97E-07	9.87E-07	6.82E-07	4.58E-07
**72h**	7.30E-07	3.42E-07	2.66E-07	4.00E-07
	cgd7_2020			
**2h**	0.00E+00	0.00E+00	0.00E+00	0.00E+00
**6h**	5.11E-06	1.69E-05	1.36E-05	1.13E-05
**12h**	6.04E-07	3.60E-07	1.12E-06	2.13E-07
**24h**	4.05E-07	1.53E-05	1.96E-05	8.84E-07
**36h**	5.95E-06	3.30E-06	4.58E-06	2.31E-06
**48h**	2.65E-06	4.02E-06	2.88E-06	2.12E-06
**72h**	6.75E-07	7.97E-07	7.86E-07	7.97E-07
	cgd7_2030			
**2h**	4.43E-08	3.94E-09	4.19E-08	3.15E-08
**6h**	6.64E-07	1.06E-06	1.16E-06	2.05E-06
**12h**	1.30E-07	1.75E-07	2.25E-07	2.58E-08
**24h**	1.43E-07	1.48E-06	1.92E-06	1.54E-07
**36h**	2.72E-07	9.51E-07	5.98E-07	3.55E-07
**48h**	7.32E-07	9.38E-07	1.00E-06	4.96E-07
**72h**	9.48E-08	1.71E-07	9.81E-08	2.15E-07
	cgd7_2040			
**2h**	1.40E-06	2.91E-07	7.32E-07	1.32E-07
**6h**	1.58E-07	5.66E-07	3.51E-07	5.79E-07
**12h**	7.44E-08	3.53E-08	2.01E-07	2.94E-08
**24h**	1.60E-07	9.18E-07	9.29E-07	8.59E-08
**36h**	3.28E-07	4.82E-07	1.84E-07	1.45E-07
**48h**	2.29E-07	7.00E-08	2.14E-07	1.43E-07
**72h**	4.82E-08	9.76E-08	7.14E-08	6.20E-08
	cgd7_2050			
**2h**	8.16E-05	3.89E-05	1.60E-04	2.88E-04
**6h**	6.72E-06	2.01E-04	3.73E-04	4.99E-04
**12h**	1.95E-06	1.49E-05	6.06E-05	2.13E-06
**24h**	3.98E-06	2.14E-04	1.44E-04	5.89E-05
**36h**	2.25E-05	4.71E-05	6.46E-05	1.25E-04
**48h**	1.23E-05	2.39E-05	3.36E-05	7.44E-05
**72h**	2.55E-06	8.13E-06	7.59E-06	2.41E-05
	cgd8_100			
**2h**	2.93E-06	1.18E-05	1.87E-07	3.50E-06
**6h**	6.90E-05	6.98E-05	7.21E-05	2.47E-05
**12h**	3.01E-06	3.50E-07	1.68E-04	2.12E-06
**24h**	1.47E-05	1.36E-05	6.39E-05	4.37E-05
**36h**	2.31E-05	1.29E-05	2.32E-05	1.18E-05
**48h**	1.32E-05	3.73E-06	5.54E-06	1.25E-05
**72h**	2.46E-06	1.33E-06	1.99E-06	2.56E-06
	cgd8_110			
**2h**	2.42E-04	3.22E-04	5.76E-05	1.16E-04
**6h**	1.62E-03	2.53E-04	1.10E-03	4.85E-04

**12h**	2.53E-04	7.23E-04	6.86E-04	1.28E-04
**24h**	2.72E-04	2.50E-04	6.57E-04	1.35E-03
**36h**	3.89E-04	2.94E-04	4.31E-04	6.90E-04
**48h**	2.69E-04	3.99E-04	5.84E-04	2.25E-04
**72h**	1.33E-04	1.90E-04	3.88E-04	1.74E-04
	cgd8_150			
**2h**	0.00E+00	0.00E+00	8.05E-09	0.00E+00
**6h**	4.86E-08	1.56E-07	2.25E-07	7.25E-08
**12h**	9.93E-09	2.65E-09	2.54E-08	6.83E-09
**24h**	2.91E-08	1.35E-08	3.19E-07	1.49E-07
**36h**	5.10E-08	4.87E-08	4.36E-08	6.75E-08
**48h**	8.56E-08	2.48E-08	6.74E-08	3.36E-08
**72h**	3.30E-08	2.85E-08	2.82E-08	2.96E-08
	cgd8_160			
**2h**	2.33E-07	1.17E-07	2.91E-09	1.68E-07
**6h**	9.94E-07	1.49E-06	5.19E-07	4.42E-07
**12h**	9.97E-10	1.72E-08	5.26E-08	3.83E-08
**24h**	5.85E-08	6.75E-08	3.22E-07	1.41E-07
**36h**	2.27E-07	1.29E-07	2.13E-07	1.50E-07
**48h**	4.76E-08	2.46E-08	4.89E-08	3.65E-08
**72h**	1.57E-08	9.22E-09	3.36E-08	1.29E-08
	cgd8_170			
**2h**	1.89E-05	2.42E-05	4.83E-06	2.21E-05
**6h**	4.08E-06	8.86E-06	1.02E-05	6.21E-06
**12h**	5.68E-06	2.19E-06	1.65E-05	1.18E-05
**24h**	7.64E-06	8.22E-06	1.17E-05	5.73E-06
**36h**	2.47E-06	2.98E-06	2.42E-06	2.76E-06
**48h**	3.20E-06	5.12E-07	1.16E-06	2.11E-06
**72h**	4.24E-07	8.34E-07	3.68E-07	2.97E-07
	cgd8_180			
**2h**	9.04E-06	1.35E-05	7.54E-07	7.80E-06
**6h**	9.86E-06	7.23E-06	2.00E-05	7.13E-06
**12h**	2.78E-06	2.10E-06	4.28E-06	2.56E-06
**24h**	1.04E-05	3.77E-06	2.67E-05	1.35E-05
**36h**	4.64E-06	5.31E-06	2.95E-06	2.89E-06
**48h**	5.99E-06	1.30E-06	4.74E-06	3.99E-06
**72h**	1.19E-06	2.41E-06	9.44E-07	5.50E-07
	cgd8_190			
**2h**	1.20E-05	3.98E-06	2.03E-06	8.45E-06
**6h**	2.16E-06	4.04E-07	2.39E-06	1.10E-06
**12h**	4.45E-06	3.91E-06	1.15E-05	5.69E-06
**24h**	7.54E-06	7.79E-06	4.55E-06	5.17E-06
**36h**	1.84E-06	2.77E-06	2.07E-06	1.80E-06
**48h**	1.87E-06	5.78E-07	1.79E-06	1.40E-06
**72h**	6.37E-07	9.38E-07	1.26E-06	8.04E-07
	cgd8_200			
**2h**	4.78E-09	9.84E-09	4.68E-09	0.00E+00
**6h**	1.28E-08	5.61E-09	0.00E+00	1.38E-08
**12h**	1.46E-07	5.49E-07	4.15E-07	2.52E-07
**24h**	3.75E-07	1.62E-07	4.28E-07	2.95E-07
**36h**	6.10E-07	9.64E-07	8.15E-07	8.12E-07
**48h**	5.20E-07	2.19E-07	4.63E-07	6.05E-07
**72h**	4.85E-07	3.58E-07	1.03E-06	5.89E-07

	cgd8_210			
**2h**	1.12E-05	3.17E-05	4.73E-06	3.30E-05
**6h**	2.75E-05	8.62E-06	3.34E-05	1.09E-05
**12h**	1.13E-05	5.92E-06	2.79E-05	1.43E-05
**24h**	3.82E-05	2.67E-05	5.10E-05	4.05E-05
**36h**	2.01E-05	2.06E-05	1.67E-05	9.20E-06
**48h**	2.16E-05	9.91E-06	1.65E-05	1.34E-05
**72h**	1.20E-05	6.77E-06	1.46E-05	4.62E-06
	cgd8_220			
**2h**	2.13E-07	4.69E-07	1.71E-07	1.80E-07
**6h**	3.06E-07	1.14E-06	1.26E-05	2.98E-06
**12h**	6.36E-08	2.11E-07	1.61E-06	8.13E-07
**24h**	2.26E-06	4.11E-06	9.12E-06	5.00E-06
**36h**	4.59E-06	6.51E-06	6.51E-06	4.35E-06
**48h**	1.27E-05	8.91E-06	1.25E-05	7.32E-06
**72h**	8.21E-06	7.52E-06	9.81E-06	4.58E-06
	cgd8_230			
**2h**	5.12E-06	4.65E-06	2.16E-06	8.22E-06
**6h**	1.59E-06	8.27E-07	4.70E-06	1.35E-06
**12h**	1.29E-06	5.75E-07	5.06E-06	3.26E-06
**24h**	2.91E-06	3.07E-06	5.55E-06	6.78E-06
**36h**	1.15E-06	1.78E-06	1.29E-06	1.01E-06
**48h**	1.72E-06	5.48E-07	1.42E-06	1.38E-06
**72h**	3.98E-07	2.55E-07	2.26E-07	2.00E-07
	cgd8_240			
**2h**	3.38E-06	4.52E-06	5.22E-08	3.23E-05
**6h**	1.52E-06	5.58E-07	7.27E-06	4.22E-06
**12h**	1.11E-06	2.53E-06	3.39E-06	1.31E-05
**24h**	5.74E-06	4.25E-06	1.28E-05	7.10E-06
**36h**	3.18E-05	5.49E-05	3.90E-05	4.30E-05
**48h**	7.57E-05	2.21E-05	6.91E-05	6.69E-05
**72h**	2.68E-04	7.57E-05	2.11E-04	1.51E-04
	cgd7_2520			
**2h**	2.44E-05	1.67E-05	2.56E-06	1.95E-05
**6h**	3.62E-05	1.84E-05	2.02E-05	2.21E-05
**12h**	3.60E-06	1.89E-06	5.22E-06	9.17E-06
**24h**	8.34E-06	1.20E-05	1.01E-05	1.09E-05
**36h**	1.92E-05	5.25E-06	1.02E-05	4.52E-06
**48h**	9.40E-06	6.36E-06	6.63E-06	5.51E-06
**72h**	5.05E-06	2.07E-06	4.21E-06	1.88E-06
	cgd7_2530			
**2h**	9.45E-06	4.26E-06	4.63E-07	4.80E-06
**6h**	1.46E-05	6.68E-06	7.10E-06	3.55E-05
**12h**	8.19E-07	2.46E-07	4.01E-06	5.92E-06
**24h**	5.26E-06	3.34E-06	8.92E-06	1.22E-05
**36h**	1.02E-05	2.38E-06	4.24E-06	5.17E-06
**48h**	5.79E-06	3.04E-06	2.54E-06	2.20E-06
**72h**	1.63E-06	6.40E-07	2.92E-07	4.20E-07
	cgd7_2550			
**2h**	1.61E-05	6.36E-06	1.72E-06	1.21E-05
**6h**	8.79E-07	1.12E-06	9.04E-07	3.21E-06
**12h**	7.15E-06	1.27E-06	3.75E-06	8.41E-06
**24h**	3.60E-06	3.34E-06	2.30E-06	2.85E-06

**36h**	4.39E-06	2.04E-06	5.17E-06	3.61E-06
**48h**	3.52E-06	1.63E-06	2.51E-06	1.15E-06
**72h**	1.68E-06	5.32E-07	8.96E-07	1.09E-06
	cgd7_2560			
**2h**	7.96E-05	3.43E-05	1.62E-05	4.30E-05
**6h**	5.26E-06	5.02E-06	4.76E-06	1.42E-05
**12h**	1.03E-05	3.30E-06	9.06E-06	1.23E-05
**24h**	1.68E-05	1.39E-05	6.60E-06	6.51E-06
**36h**	3.65E-05	5.25E-06	8.08E-06	4.46E-06
**48h**	1.24E-05	5.34E-06	4.58E-06	2.90E-06
**72h**	1.39E-05	1.21E-06	1.02E-06	1.58E-06
	cgd7_2570			
**2h**	6.83E-04	1.60E-03	2.76E-04	4.64E-04
**6h**	5.36E-04	5.24E-04	1.13E-04	2.83E-04
**12h**	2.43E-04	1.12E-04	2.43E-04	1.66E-04
**24h**	3.04E-04	4.40E-04	2.78E-04	1.71E-04
**36h**	6.18E-04	1.28E-04	2.10E-04	1.12E-04
**48h**	7.57E-04	1.77E-04	1.73E-04	3.67E-05
**72h**	1.72E-04	4.64E-05	1.12E-04	2.56E-05
	cgd7_2580			
**2h**	1.16E-05	8.80E-06	4.09E-07	3.44E-06
**6h**	1.08E-05	1.89E-06	2.06E-06	6.45E-06
**12h**	6.31E-07	2.24E-07	2.37E-07	8.78E-07
**24h**	2.01E-06	1.52E-06	1.57E-06	3.33E-06
**36h**	1.32E-05	2.22E-06	3.35E-06	2.20E-06
**48h**	3.02E-05	7.89E-06	9.90E-06	4.92E-06
**72h**	1.80E-05	1.35E-05	1.34E-05	4.67E-06
	cgd7_2590			
**2h**	1.62E-04	3.93E-04	5.04E-04	1.37E-02
**6h**	1.08E-04	2.42E-04	3.79E-04	3.57E-02
**12h**	1.12E-05	6.26E-05	1.04E-04	1.63E-02
**24h**	3.90E-05	1.59E-04	2.98E-04	1.94E-03
**36h**	1.32E-04	5.14E-05	9.93E-05	4.93E-03
**48h**	2.91E-04	2.50E-04	5.83E-04	6.46E-03
**72h**	1.55E-04	1.20E-04	2.33E-04	5.48E-03
	cgd7_2600			
**2h**	1.15E-03	2.41E-04	7.84E-05	1.26E-04
**6h**	3.55E-03	4.19E-04	1.18E-03	8.94E-04
**12h**	1.06E-04	9.79E-06	4.44E-05	5.17E-05
**24h**	3.39E-04	1.36E-04	5.16E-04	2.44E-04
**36h**	6.58E-04	3.68E-04	3.65E-04	1.23E-04
**48h**	2.96E-04	6.09E-04	2.90E-04	8.95E-05
**72h**	9.06E-05	4.98E-05	2.66E-05	1.70E-05
	cgd7_2610			
**2h**	2.20E-04	2.66E-04	1.48E-06	6.65E-05
**6h**	5.50E-04	2.75E-03	6.41E-03	2.44E-03
**12h**	1.65E-05	2.71E-05	2.47E-04	9.29E-04
**24h**	7.94E-05	6.47E-04	3.65E-03	1.18E-03
**36h**	6.11E-04	6.79E-04	1.76E-03	4.32E-04
**48h**	1.90E-04	3.05E-04	1.08E-03	3.88E-04
**72h**	1.71E-04	1.79E-04	2.00E-04	1.47E-04
	cgd7_2620			
**2h**	5.27E-07	8.27E-07	3.27E-07	1.29E-06

**6h**	2.45E-06	1.50E-07	2.22E-07	2.48E-06
**12h**	1.13E-05	8.92E-06	1.14E-05	1.53E-05
**24h**	6.53E-05	6.55E-06	1.74E-05	1.53E-05
**36h**	1.31E-04	3.77E-05	4.41E-05	2.97E-05
**48h**	2.60E-04	1.97E-05	2.57E-05	1.03E-05
**72h**	9.40E-06	1.07E-05	2.56E-06	1.45E-06
	cgd7_2640			
**2h**	7.94E-06	1.26E-05	8.11E-06	1.95E-05
**6h**	2.05E-06	4.65E-06	8.13E-06	1.24E-05
**12h**	9.13E-07	2.73E-06	5.12E-06	1.01E-05
**24h**	2.06E-06	3.70E-06	3.92E-06	9.17E-06
**36h**	3.60E-06	3.62E-06	5.14E-06	3.20E-06
**48h**	2.21E-06	4.43E-06	4.32E-06	3.65E-06
**72h**	1.18E-06	5.22E-07	1.17E-06	6.02E-07
	cgd7_2670			
**2h**	1.42E-05	5.37E-05	6.33E-05	8.40E-05
**6h**	4.46E-06	5.48E-06	7.80E-06	2.15E-05
**12h**	2.72E-05	6.38E-05	4.42E-05	7.66E-05
**24h**	1.63E-05	2.57E-05	2.01E-05	2.66E-05
**36h**	2.25E-05	1.48E-05	2.11E-05	2.17E-05
**48h**	4.34E-06	9.81E-06	1.18E-05	6.74E-06
**72h**	3.79E-06	1.59E-06	3.01E-06	4.60E-06
	cgd8_1750			
**2h**	0.00E+00	0.00E+00	0.00E+00	0.00E+00
**6h**	0.00E+00	0.00E+00	0.00E+00	0.00E+00
**12h**	0.00E+00	0.00E+00	7.21E-08	0.00E+00
**24h**	7.88E-08	9.70E-07	9.99E-08	1.08E-07
**36h**	3.65E-07	1.14E-06	3.64E-07	1.41E-07
**48h**	8.28E-06	2.63E-05	1.27E-05	2.31E-05
**72h**	2.63E-06	8.64E-06	1.91E-06	5.55E-06
	cgd8_1760			
**2h**	0.00E+00	3.99E-06	0.00E+00	0.00E+00
**6h**	0.00E+00	0.00E+00	0.00E+00	0.00E+00
**12h**	3.16E-04	7.77E-04	7.10E-04	6.61E-03
**24h**	6.78E-04	4.38E-05	5.71E-04	1.84E-03
**36h**	2.32E-04	8.05E-05	1.20E-04	2.56E-03
**48h**	3.37E-04	1.44E-05	7.60E-05	3.17E-03
**72h**	4.73E-04	1.76E-05	1.51E-04	2.48E-03
	cgd8_1790			
**2h**	0.00E+00	0.00E+00	0.00E+00	0.00E+00
**6h**	0.00E+00	0.00E+00	0.00E+00	0.00E+00
**12h**	2.74E-06	2.52E-06	4.47E-06	4.11E-06
**24h**	5.25E-07	2.13E-07	1.20E-06	8.58E-07
**36h**	5.54E-07	7.80E-07	4.87E-07	1.75E-06
**48h**	9.84E-08	2.17E-07	1.16E-07	6.00E-07
**72h**	2.06E-08	1.22E-07	3.92E-08	1.14E-07
	cgd8_1830			
**2h**	4.51E-06	7.11E-05	1.70E-06	3.10E-06
**6h**	5.03E-06	4.29E-04	1.31E-05	3.35E-05
**12h**	5.71E-06	5.24E-04	1.00E-05	3.38E-05
**24h**	4.33E-06	4.39E-05	1.05E-05	2.23E-05
**36h**	5.11E-06	9.75E-05	5.39E-06	2.01E-05
**48h**	3.50E-06	1.25E-03	1.01E-06	1.96E-05

**72h**	8.40E-06	5.03E-05	4.99E-06	1.06E-05
	cgd8_1840			
**2h**	2.21E-07	8.01E-08	2.47E-08	7.31E-08
**6h**	2.64E-07	8.24E-07	2.56E-07	7.40E-08
**12h**	1.41E-07	1.03E-07	3.51E-08	3.21E-07
**24h**	7.86E-08	3.02E-07	1.43E-07	2.00E-07
**36h**	1.37E-07	2.13E-07	1.47E-07	1.14E-07
**48h**	7.62E-08	1.49E-07	3.21E-08	2.13E-07
**72h**	5.53E-08	1.54E-07	7.05E-08	1.27E-07
	cgd8_1860			
**2h**	2.32E-06	7.42E-05	3.51E-07	2.28E-06
**6h**	4.57E-07	9.68E-05	1.79E-06	2.47E-06
**12h**	5.34E-07	1.17E-04	5.35E-07	3.34E-06
**24h**	7.77E-07	4.70E-05	1.22E-06	4.71E-06
**36h**	2.60E-06	1.10E-04	2.14E-06	2.31E-06
**48h**	3.41E-06	2.23E-04	1.05E-06	3.76E-06
**72h**	1.96E-06	1.80E-04	1.79E-06	3.34E-06
	cgd8_1870			
**2h**	1.46E-07	1.02E-07	1.14E-08	2.20E-08
**6h**	1.32E-08	4.30E-07	3.01E-08	3.89E-08
**12h**	5.85E-08	2.37E-07	6.08E-08	7.28E-08
**24h**	2.41E-08	2.12E-07	3.16E-08	1.24E-07
**36h**	2.12E-07	3.89E-07	1.53E-07	1.08E-07
**48h**	4.72E-07	1.34E-06	1.28E-06	2.56E-07
**72h**	2.93E-07	5.85E-07	4.68E-07	2.43E-07
	cgd8_1880			
**2h**	6.78E-09	0.00E+00	0.00E+00	0.00E+00
**6h**	3.21E-06	3.40E-05	5.47E-06	8.06E-06
**12h**	2.58E-07	2.43E-05	2.06E-07	5.35E-06
**24h**	2.49E-06	2.23E-05	2.60E-06	1.50E-05
**36h**	9.15E-06	2.00E-05	1.29E-05	8.02E-06
**48h**	1.18E-05	3.13E-05	2.91E-06	3.73E-05
**72h**	1.02E-05	4.65E-05	1.15E-05	3.44E-05
	cgd8_1900			
**2h**	0.00E+00	4.83E-08	2.84E-09	0.00E+00
**6h**	0.00E+00	0.00E+00	3.87E-09	0.00E+00
**12h**	5.93E-07	6.31E-06	5.50E-07	1.02E-06
**24h**	1.87E-07	1.40E-06	1.81E-07	3.31E-07
**36h**	2.09E-07	2.14E-06	3.57E-07	7.36E-07
**48h**	3.29E-07	1.58E-06	1.21E-07	1.66E-07
**72h**	8.78E-08	1.75E-06	1.35E-07	1.62E-07
	cgd8_1910			
**2h**	1.28E-06	4.03E-06	2.67E-07	1.23E-07
**6h**	2.95E-07	4.87E-06	3.66E-07	6.17E-07
**12h**	3.54E-07	7.33E-06	1.91E-06	2.21E-06
**24h**	1.46E-06	1.56E-06	1.31E-06	2.17E-06
**36h**	1.12E-06	3.22E-06	1.23E-02	1.36E-06
**48h**	2.25E-06	5.55E-06	1.18E-06	8.66E-07
**72h**	7.34E-07	2.15E-06	5.32E-07	1.60E-06
	cgd8_1930			
**2h**	2.69E-04	8.97E-06	1.82E-05	2.15E-05
**6h**	2.46E-05	1.03E-04	7.06E-05	6.25E-04
**12h**	3.90E-06	4.61E-05	3.08E-06	4.53E-04

**24h**	2.47E-04	5.08E-05	2.26E-05	5.53E-04
**36h**	4.78E-04	1.89E-05	1.79E-04	2.23E-04
**48h**	9.88E-05	2.02E-05	1.38E-05	4.66E-04
**72h**	2.29E-05	1.67E-05	6.39E-05	2.39E-04
	cgd7_3910			
**2h**	1.96E-05	4.48E-05	6.64E-05	3.60E-05
**6h**	1.85E-04	1.56E-03	2.23E-04	3.98E-04
**12h**	1.92E-05	1.30E-06	1.95E-05	3.99E-05
**24h**	1.27E-04	1.52E-05	4.47E-05	2.33E-04
**36h**	9.65E-05	4.10E-05	5.38E-05	5.91E-05
**48h**	1.41E-04	2.46E-05	1.44E-05	4.24E-05
**72h**	5.32E-05	4.46E-06	1.04E-05	1.81E-05
	cgd7_3920			
**2h**	2.90E-05	5.29E-08	4.09E-08	1.15E-08
**6h**	2.86E-07	1.17E-07	4.97E-07	9.45E-09
**12h**	1.95E-06	3.05E-08	1.53E-08	1.27E-07
**24h**	3.96E-06	2.76E-07	4.07E-07	5.69E-07
**36h**	1.52E-06	2.96E-07	4.13E-07	2.21E-07
**48h**	6.42E-06	4.13E-07	3.07E-07	3.29E-07
**72h**	3.86E-06	3.47E-07	3.88E-07	3.33E-07
	cgd7_3930			
**2h**	1.28E-04	5.44E-06	1.04E-05	1.76E-05
**6h**	8.87E-05	4.00E-05	1.03E-04	1.11E-04
**12h**	1.61E-05	3.21E-06	2.54E-05	1.65E-04
**24h**	7.82E-05	2.23E-05	1.62E-04	1.09E-04
**36h**	1.91E-05	1.90E-05	4.32E-05	4.41E-05
**48h**	1.76E-05	2.86E-05	5.34E-05	5.88E-05
**72h**	1.75E-05	3.05E-06	2.09E-05	8.81E-05
	cgd7_3940			
**2h**	1.25E-05	2.41E-06	6.64E-06	2.34E-06
**6h**	1.93E-04	2.05E-06	6.67E-06	7.52E-06
**12h**	2.30E-04	6.28E-07	3.58E-06	2.01E-05
**24h**	8.45E-05	4.05E-06	1.43E-05	1.02E-05
**36h**	7.64E-05	2.30E-06	6.85E-06	4.84E-06
**48h**	4.84E-05	2.62E-06	4.28E-06	3.21E-06
**72h**	7.19E-05	4.34E-07	3.22E-06	2.68E-06
	cgd7_3950			
**2h**	3.84E-07	5.56E-07	1.50E-07	1.70E-08
**6h**	2.40E-07	6.61E-08	2.14E-07	3.26E-07
**12h**	6.93E-08	0.00E+00	8.46E-09	6.58E-08
**24h**	1.30E-07	5.68E-08	7.69E-08	1.68E-07
**36h**	6.26E-08	1.15E-07	1.19E-07	5.40E-08
**48h**	6.48E-08	6.25E-08	2.58E-08	2.19E-08
**72h**	7.39E-08	1.01E-08	4.41E-08	4.71E-08
	cgd7_3960			
**2h**	1.13E-06	6.83E-07	1.90E-06	2.03E-07
**6h**	1.88E-06	3.39E-07	4.57E-07	1.45E-06
**12h**	9.64E-07	6.91E-08	5.49E-08	2.05E-07
**24h**	1.60E-06	2.59E-07	3.06E-07	8.37E-07
**36h**	1.03E-06	3.42E-07	3.48E-07	3.49E-07
**48h**	1.63E-06	3.97E-07	1.33E-07	2.24E-07
**72h**	5.99E-07	7.24E-08	7.72E-08	1.61E-07
	cgd7_3980			

**2h**	2.05E-05	2.13E-06	1.21E-05	1.77E-05
**6h**	3.73E-04	1.27E-04	8.41E-04	5.17E-04
**12h**	5.76E-05	8.84E-06	4.04E-05	2.96E-04
**24h**	1.14E-04	5.28E-05	2.31E-04	1.26E-03
**36h**	3.49E-05	2.87E-04	7.30E-04	7.95E-04
**48h**	5.15E-05	1.53E-04	4.29E-04	7.89E-04
**72h**	3.40E-05	1.96E-05	1.93E-04	1.93E-04
	cgd7_3990			
**2h**	1.11E-08	9.26E-10	1.57E-09	6.14E-09
**6h**	1.61E-07	2.39E-08	1.75E-08	3.72E-08
**12h**	3.30E-07	1.62E-07	1.93E-07	3.55E-08
**24h**	1.44E-07	8.43E-08	4.52E-08	1.70E-07
**36h**	5.82E-07	2.64E-07	1.18E-07	1.74E-07
**48h**	3.43E-06	2.83E-07	1.51E-07	2.15E-07
**72h**	9.78E-07	7.23E-08	1.54E-07	3.01E-07
	cgd7_4010			
**2h**	1.20E-08	0.00E+00	0.00E+00	0.00E+00
**6h**	0.00E+00	0.00E+00	0.00E+00	0.00E+00
**12h**	2.31E-06	2.61E-07	4.10E-07	3.10E-07
**24h**	2.05E-07	1.78E-07	6.00E-07	1.92E-07
**36h**	2.40E-06	2.28E-07	2.35E-07	3.07E-07
**48h**	8.22E-07	1.49E-07	5.51E-08	6.31E-08
**72h**	5.89E-07	1.17E-08	4.65E-08	4.77E-08
	cgd7_4020			
**2h**	4.21E-07	2.48E-09	1.60E-08	2.54E-08
**6h**	9.62E-08	0.00E+00	5.03E-09	0.00E+00
**12h**	3.35E-03	1.33E-03	8.95E-03	4.59E-03
**24h**	4.26E-04	3.44E-04	7.85E-04	2.85E-04
**36h**	5.13E-04	5.08E-05	1.67E-04	3.34E-04
**48h**	1.23E-04	2.73E-05	1.69E-05	3.75E-05
**72h**	1.47E-05	4.04E-07	1.04E-06	9.20E-07
	cgd7_4030			
**2h**	0.00E+00	0.00E+00	0.00E+00	0.00E+00
**6h**	7.41E-08	0.00E+00	4.09E-09	3.73E-09
**12h**	1.67E-05	9.60E-07	5.70E-06	1.65E-06
**24h**	1.01E-06	5.18E-07	7.94E-07	2.91E-07
**36h**	8.59E-06	2.85E-07	5.14E-07	1.15E-06
**48h**	3.79E-06	5.59E-07	1.67E-07	2.51E-07
**72h**	2.01E-06	1.71E-08	9.41E-08	9.37E-08
	cgd7_4040			
**2h**	1.99E-07	1.44E-09	0.00E+00	1.71E-09
**6h**	1.33E-07	0.00E+00	7.01E-09	7.46E-09
**12h**	3.73E-07	5.32E-08	1.86E-07	2.00E-07
**24h**	8.12E-08	3.02E-08	7.61E-08	8.04E-08
**36h**	1.49E-06	8.64E-08	8.77E-08	1.79E-07
**48h**	1.61E-06	1.27E-07	1.04E-07	2.34E-07
**72h**	9.10E-07	2.05E-08	8.75E-08	7.76E-08
	cgd8_260			
**2h**	7.18E-07	2.42E-07	2.63E-06	1.62E-06
**6h**	1.21E-06	1.65E-06	2.66E-06	6.99E-07
**12h**	5.33E-08	1.16E-06	3.40E-08	4.07E-07
**24h**	5.50E-07	1.57E-06	3.71E-07	2.98E-06
**36h**	1.06E-06	8.88E-07	2.67E-07	9.12E-07

**48h**	2.63E-07	1.08E-06	6.26E-07	1.14E-06
**72h**	1.55E-07	5.68E-07	7.57E-08	2.94E-07
	cgd8_270			
**2h**	1.52E-08	0.00E+00	1.53E-07	5.60E-08
**6h**	2.33E-07	3.01E-07	7.78E-07	2.55E-07
**12h**	1.07E-08	1.32E-09	6.85E-09	2.45E-08
**24h**	6.82E-08	2.67E-07	5.29E-08	2.72E-07
**36h**	9.32E-08	1.12E-07	4.09E-08	2.30E-07
**48h**	4.81E-08	8.00E-06	9.28E-08	1.26E-07
**72h**	4.02E-08	9.32E-08	2.49E-08	1.14E-07
	cgd8_280			
**2h**	9.69E-08	5.37E-07	3.80E-07	2.83E-06
**6h**	4.73E-08	3.69E-08	1.16E-07	2.81E-07
**12h**	1.28E-06	1.07E-07	2.44E-07	5.53E-07
**24h**	3.38E-07	1.48E-07	2.81E-07	6.69E-07
**36h**	1.16E-07	1.04E-07	5.01E-08	2.19E-07
**48h**	2.20E-08	1.39E-07	6.36E-08	7.01E-08
**72h**	5.47E-08	6.03E-08	1.70E-08	5.20E-08
	cgd8_290			
**2h**	0.00E+00	0.00E+00	0.00E+00	0.00E+00
**6h**	1.14E-08	7.37E-08	3.14E-08	1.05E-08
**12h**	2.66E-09	3.69E-09	1.77E-08	1.23E-08
**24h**	5.05E-08	9.29E-08	3.39E-08	2.06E-07
**36h**	1.91E-07	9.59E-08	5.45E-08	1.07E-07
**48h**	2.64E-08	6.81E-08	2.63E-08	5.21E-08
**72h**	2.59E-08	2.99E-08	7.50E-09	4.28E-08
	cgd8_340			
**2h**	5.79E-07	8.51E-07	8.92E-07	2.52E-06
**6h**	7.50E-07	3.75E-06	1.32E-06	4.06E-06
**12h**	1.32E-06	6.29E-07	1.45E-07	4.74E-06
**24h**	1.53E-06	4.37E-06	6.88E-07	4.65E-06
**36h**	1.56E-06	2.64E-06	8.88E-07	3.15E-06
**48h**	5.38E-07	1.93E-06	4.81E-07	1.63E-06
**72h**	8.00E-07	2.05E-06	3.79E-07	1.54E-06
	cgd8_350			
**2h**	3.63E-07	1.63E-07	1.17E-06	1.22E-07
**6h**	3.76E-06	3.12E-06	3.03E-06	2.02E-06
**12h**	1.18E-07	7.02E-08	8.17E-08	1.61E-07
**24h**	5.49E-07	9.80E-07	1.66E-07	3.14E-06
**36h**	4.79E-07	4.22E-07	3.01E-07	3.40E-07
**48h**	1.58E-07	2.40E-07	8.61E-08	1.53E-07
**72h**	6.01E-08	7.29E-08	2.08E-08	6.57E-08
	cgd8_360			
**2h**	3.76E-05	2.63E-04	5.89E-05	2.69E-04
**6h**	3.35E-05	2.48E-04	3.18E-05	2.12E-04
**12h**	4.68E-05	1.47E-04	1.00E-05	1.36E-04
**24h**	5.15E-05	3.91E-04	9.85E-06	4.74E-04
**36h**	3.06E-05	2.11E-04	2.59E-05	1.75E-04
**48h**	2.48E-05	2.36E-04	1.30E-05	1.55E-04
**72h**	3.69E-06	1.84E-04	5.95E-06	4.43E-05
	cgd8_380			
**2h**	3.47E-09	0.00E+00	1.54E-08	7.86E-08
**6h**	1.62E-09	0.00E+00	0.00E+00	4.41E-09

**12h**	7.18E-08	4.55E-08	5.82E-09	1.02E-07
**24h**	1.19E-07	2.74E-08	6.80E-08	2.95E-08
**36h**	8.61E-08	9.38E-08	7.55E-08	1.42E-07
**48h**	2.00E-07	1.64E-07	9.36E-08	2.37E-07
**72h**	8.64E-08	1.37E-07	1.37E-07	1.81E-07
	cgd8_410			
**2h**	2.43E-04	8.66E-04	1.92E-04	2.87E-03
**6h**	2.47E-05	5.92E-05	1.22E-05	1.48E-04
**12h**	1.77E-04	1.36E-03	1.54E-04	2.45E-03
**24h**	8.58E-04	2.01E-03	2.72E-04	3.00E-03
**36h**	2.61E-04	1.35E-03	2.18E-04	1.48E-03
**48h**	1.18E-04	1.91E-03	1.44E-04	8.56E-04
**72h**	4.62E-05	1.10E-03	5.53E-05	6.05E-04
	cgd8_420			
**2h**	0.00E+00	0.00E+00	0.00E+00	0.00E+00
**6h**	1.16E-07	0.00E+00	0.00E+00	8.34E-08
**12h**	1.05E-05	4.29E-05	3.40E-06	1.12E-04
**24h**	1.70E-05	2.11E-04	2.92E-06	1.09E-04
**36h**	1.61E-05	2.00E-04	1.37E-05	2.27E-04
**48h**	9.07E-06	2.67E-04	3.20E-05	4.21E-04
**72h**	8.59E-06	2.14E-04	2.93E-05	1.97E-04
	cgd8_430			
**2h**	4.98E-05	8.25E-05	7.98E-05	1.41E-04
**6h**	3.95E-04	6.99E-04	2.84E-04	3.49E-04
**12h**	2.75E-05	8.68E-05	1.52E-05	2.74E-04
**24h**	2.61E-04	3.82E-04	4.89E-05	6.17E-04
**36h**	6.52E-05	7.37E-05	4.52E-05	1.20E-04
**48h**	4.46E-06	7.35E-05	1.84E-05	6.13E-05
**72h**	3.21E-06	8.31E-06	4.66E-06	6.53E-06
	cgd8_450			
**2h**	2.64E-05	1.60E-05	4.32E-05	4.15E-04
**6h**	1.06E-05	3.78E-05	5.80E-06	2.79E-05
**12h**	1.06E-05	1.58E-05	2.41E-06	1.55E-04
**24h**	3.10E-05	8.07E-05	2.53E-06	5.36E-05
**36h**	2.11E-05	9.63E-05	1.90E-05	1.32E-04
**48h**	5.17E-06	6.58E-05	1.65E-05	5.11E-05
**72h**	6.07E-06	6.36E-05	1.45E-05	5.29E-05
	cgd7_2070			
**2h**	0.00E+00	0.00E+00	0.00E+00	3.21E-05
**6h**	1.20E-08	2.82E-06	2.29E-07	0.00E+00
**12h**	1.89E-05	7.83E-04	5.61E-05	7.29E-04
**24h**	2.14E-05	2.26E-04	3.00E-05	1.90E-04
**36h**	1.78E-05	1.04E-03	1.39E-04	7.70E-04
**48h**	1.17E-05	1.36E-04	6.10E-05	4.07E-04
**72h**	1.48E-06	1.62E-05	9.97E-06	1.21E-05
	cgd7_2080			
**2h**	1.40E-07	4.57E-06	1.56E-06	4.68E-06
**6h**	5.25E-07	2.47E-05	1.87E-06	8.07E-06
**12h**	6.06E-07	1.80E-06	1.79E-07	8.70E-07
**24h**	2.30E-06	6.98E-06	4.98E-06	1.44E-06
**36h**	5.79E-06	1.12E-05	3.76E-06	8.80E-06
**48h**	2.88E-06	4.41E-05	9.88E-06	6.00E-05
**72h**	3.44E-06	1.52E-05	1.54E-05	8.73E-05

	cgd7_2090			
**2h**	6.78E-06	9.37E-05	3.16E-05	9.64E-05
**6h**	1.81E-05	1.02E-04	5.24E-05	8.97E-05
**12h**	2.03E-06	2.56E-05	4.73E-06	1.58E-05
**24h**	7.37E-06	6.50E-05	3.36E-05	1.14E-04
**36h**	1.17E-05	1.33E-05	5.60E-06	4.60E-05
**48h**	1.62E-06	1.97E-05	1.59E-05	7.60E-05
**72h**	2.00E-06	9.60E-06	4.27E-06	2.40E-05
	cgd7_2100			
**2h**	3.17E-09	1.52E-08	2.92E-08	1.51E-08
**6h**	7.42E-07	1.00E-06	9.43E-07	1.33E-06
**12h**	3.33E-07	6.59E-07	3.34E-07	2.88E-06
**24h**	1.90E-07	1.29E-06	4.20E-07	4.92E-07
**36h**	1.19E-06	1.25E-06	2.19E-06	1.77E-06
**48h**	2.85E-07	6.37E-07	4.03E-07	1.30E-06
**72h**	2.17E-07	9.74E-08	1.60E-07	3.78E-07
	cgd7_2110			
**2h**	1.12E-02	1.64E-02	2.14E-03	1.66E-01
**6h**	6.54E-01	6.02E-02	2.14E-01	2.96E-01
**12h**	2.56E-02	3.52E-02	3.77E-01	3.92E-03
**24h**	1.73E-03	3.16E-02	2.68E-02	1.24E-02
**36h**	7.67E-02	6.48E-03	2.75E-03	5.87E-03
**48h**	4.11E-02	4.87E-03	4.02E-04	7.61E-03
**72h**	4.51E-03	1.40E-03	1.19E-03	1.41E-02
	cgd7_2120			
**2h**	0.00E+00	0.00E+00	2.94E-07	2.04E-06
**6h**	1.59E-06	3.78E-06	2.56E-05	9.39E-05
**12h**	3.37E-06	1.75E-05	4.60E-06	2.73E-05
**24h**	2.67E-06	3.48E-04	4.86E-05	1.82E-04
**36h**	4.46E-05	2.99E-04	3.76E-05	4.24E-04
**48h**	9.80E-06	1.22E-04	7.97E-05	7.12E-04
**72h**	5.69E-06	2.85E-04	7.51E-05	2.05E-04
	cgd7_2130			
**2h**	2.53E-08	1.65E-08	2.26E-08	3.67E-08
**6h**	9.23E-08	1.03E-06	5.56E-07	1.25E-06
**12h**	4.36E-09	3.46E-08	1.48E-08	7.76E-08
**24h**	2.80E-08	6.59E-07	1.99E-07	4.91E-07
**36h**	3.72E-07	2.16E-07	1.78E-07	1.32E-06
**48h**	1.06E-07	2.20E-07	1.43E-07	2.04E-06
**72h**	1.13E-07	1.68E-07	1.84E-07	1.08E-06
	cgd7_2140			
**2h**	0.00E+00	0.00E+00	0.00E+00	0.00E+00
**6h**	3.17E-08	2.76E-07	1.12E-07	1.14E-07
**12h**	3.07E-07	1.17E-07	4.03E-08	1.14E-06
**24h**	6.57E-08	1.00E-06	3.37E-07	4.36E-07
**36h**	8.64E-07	3.97E-07	3.14E-07	1.83E-06
**48h**	2.40E-07	1.52E-07	2.64E-07	1.30E-06
**72h**	7.83E-08	7.23E-08	1.78E-07	6.66E-07
	cgd7_2160			
**2h**	1.91E-06	1.19E-06	5.09E-06	3.08E-05
**6h**	3.80E-06	3.83E-05	3.47E-05	9.88E-05
**12h**	1.33E-07	4.66E-06	5.68E-07	9.56E-07
**24h**	2.14E-06	2.55E-05	9.17E-06	2.07E-05

**36h**	5.22E-06	3.88E-06	3.45E-06	2.24E-05
**48h**	2.49E-06	3.88E-06	3.30E-06	2.40E-05
**72h**	3.60E-07	6.33E-07	3.06E-06	7.13E-06
	cgd7_2170			
**2h**	9.26E-06	3.04E-05	5.22E-05	3.71E-04
**6h**	1.11E-05	2.36E-04	2.06E-04	3.26E-04
**12h**	3.06E-06	1.20E-04	3.94E-05	9.16E-05
**24h**	1.86E-05	1.59E-04	1.03E-04	5.27E-04
**36h**	1.61E-05	4.17E-05	2.51E-05	2.97E-04
**48h**	1.44E-05	3.76E-05	4.09E-05	2.42E-04
**72h**	1.37E-06	4.71E-06	2.70E-06	0.00E+00
	cgd7_2190			
**2h**	1.35E-07	3.34E-07	4.34E-07	1.41E-06
**6h**	6.37E-07	4.23E-07	7.02E-07	3.94E-06
**12h**	1.25E-08	3.07E-07	1.90E-07	3.24E-08
**24h**	3.61E-07	4.91E-07	3.99E-07	1.47E-06
**36h**	2.25E-07	1.34E-07	1.61E-07	6.31E-07
**48h**	1.25E-07	1.36E-07	1.83E-07	9.87E-07
**72h**	4.26E-08	1.06E-07	1.44E-07	6.59E-07
	cgd7_2200			
**2h**	2.17E-06	1.46E-05	1.17E-05	1.30E-05
**6h**	1.58E-06	2.61E-06	1.30E-05	5.32E-05
**12h**	1.14E-06	3.56E-06	5.54E-06	1.37E-05
**24h**	1.87E-06	8.49E-06	5.92E-06	4.86E-05
**36h**	5.16E-06	3.11E-06	3.91E-06	6.81E-05
**48h**	1.37E-06	2.35E-06	3.12E-06	1.07E-04
**72h**	9.20E-07	3.14E-06	2.10E-06	1.90E-05
	cgd7_2210			
**2h**	2.16E-07	1.25E-07	6.82E-08	1.58E-07
**6h**	0.00E+00	3.17E-09	2.79E-08	3.68E-08
**12h**	1.20E-06	4.13E-07	2.08E-06	1.40E-06
**24h**	6.16E-07	4.31E-07	5.25E-07	6.18E-07
**36h**	9.10E-07	3.88E-07	2.43E-06	1.58E-06
**48h**	4.49E-07	1.96E-07	8.65E-07	7.01E-07
**72h**	1.47E-07	6.86E-08	4.55E-07	2.56E-07
	cgd7_2260			
**2h**	7.26E-07	2.52E-07	1.53E-06	5.24E-07
**6h**	1.83E-06	1.37E-06	2.55E-06	2.58E-06
**12h**	8.09E-08	1.09E-08	8.92E-07	5.43E-07
**24h**	8.46E-07	5.56E-06	2.09E-06	2.12E-06
**36h**	9.74E-07	3.77E-07	9.65E-07	8.64E-07
**48h**	5.12E-07	4.26E-07	7.21E-07	5.49E-07
**72h**	2.28E-07	1.74E-07	8.37E-07	2.02E-07
	cgd7_2270			
**2h**	2.15E-06	5.37E-07	3.72E-06	9.74E-07
**6h**	1.02E-06	1.41E-06	2.70E-07	8.65E-07
**12h**	4.47E-06	1.93E-06	4.33E-06	1.66E-06
**24h**	1.26E-06	1.78E-06	1.65E-06	1.19E-06
**36h**	1.69E-06	1.43E-06	2.96E-06	3.27E-06
**48h**	8.40E-07	9.52E-07	2.76E-06	1.46E-06
**72h**	4.98E-07	7.50E-07	2.03E-06	5.66E-07
	cgd7_2280			
**2h**	1.06E-05	2.45E-06	1.03E-05	4.22E-06

**6h**	3.98E-05	6.36E-05	5.24E-05	1.83E-05
**12h**	4.33E-06	9.97E-07	1.40E-05	1.18E-05
**24h**	9.08E-06	8.95E-06	2.64E-05	1.28E-05
**36h**	9.93E-06	6.54E-06	6.91E-06	8.43E-06
**48h**	2.98E-06	2.78E-06	5.60E-06	2.72E-06
**72h**	1.59E-06	3.13E-07	3.26E-06	2.33E-06
	cgd7_2290			
**2h**	6.51E-05	5.59E-05	1.49E-04	1.00E-04
**6h**	1.18E-05	1.52E-04	9.36E-05	2.19E-05
**12h**	4.18E-05	7.54E-05	4.00E-04	7.52E-05
**24h**	4.02E-05	2.09E-04	1.42E-04	1.09E-04
**36h**	3.25E-05	6.30E-05	8.33E-05	4.56E-05
**48h**	3.08E-05	5.83E-05	3.88E-04	2.80E-05
**72h**	1.69E-05	3.53E-05	5.43E-05	1.15E-05
	cgd7_2300			
**2h**	1.51E-05	2.10E-06	1.22E-05	7.35E-06
**6h**	2.56E-05	1.58E-05	2.54E-05	1.52E-05
**12h**	5.34E-06	8.27E-07	1.42E-05	1.15E-05
**24h**	1.27E-05	9.06E-06	1.41E-05	1.81E-05
**36h**	1.26E-05	4.59E-06	1.14E-05	1.31E-05
**48h**	4.49E-06	1.71E-06	5.83E-06	3.65E-06
**72h**	2.05E-06	8.29E-07	2.86E-06	1.48E-06
	cgd7_2310			
**2h**	0.00E+00	0.00E+00	0.00E+00	0.00E+00
**6h**	0.00E+00	2.27E-07	0.00E+00	3.87E-09
**12h**	8.43E-08	7.15E-07	9.55E-07	2.37E-07
**24h**	7.17E-07	4.23E-06	6.07E-06	3.05E-06
**36h**	2.76E-06	7.60E-06	1.06E-05	3.25E-06
**48h**	7.15E-06	1.20E-05	1.82E-05	9.23E-06
**72h**	4.94E-06	1.66E-05	2.18E-05	1.24E-05
	cgd7_2320			
**2h**	0.00E+00	0.00E+00	0.00E+00	0.00E+00
**6h**	1.49E-08	4.05E-09	9.46E-09	8.60E-09
**12h**	1.01E-08	9.89E-09	4.73E-09	3.94E-09
**24h**	1.93E-08	1.49E-08	1.02E-07	6.59E-08
**36h**	9.84E-08	1.05E-07	1.33E-07	1.79E-07
**48h**	3.95E-07	2.48E-07	4.67E-07	4.31E-07
**72h**	1.29E-07	2.34E-07	3.10E-07	2.06E-07
	cgd7_2330			
**2h**	0.00E+00	2.01E-06	0.00E+00	6.54E-07
**6h**	9.86E-04	5.54E-03	1.45E-02	5.09E-03
**12h**	1.37E-04	4.44E-04	2.63E-03	3.56E-04
**24h**	3.28E-04	1.01E-03	7.68E-03	2.37E-03
**36h**	5.82E-04	1.01E-03	2.91E-03	8.62E-04
**48h**	6.12E-04	1.12E-03	1.81E-03	7.32E-04
**72h**	3.25E-05	3.07E-04	6.97E-04	2.63E-05
	cgd7_2340			
**2h**	0.00E+00	2.00E-08	0.00E+00	1.47E-08
**6h**	1.55E-08	0.00E+00	0.00E+00	0.00E+00
**12h**	9.29E-07	1.13E-06	4.71E-06	2.24E-06
**24h**	6.05E-07	4.97E-06	2.12E-06	1.66E-06
**36h**	7.41E-07	5.34E-06	1.00E-05	4.52E-06
**48h**	3.36E-06	6.54E-06	7.85E-06	3.06E-06

**72h**	9.26E-08	1.97E-06	1.78E-06	2.35E-07
	cgd7_2360			
**2h**	5.56E-08	2.47E-08	7.51E-09	2.95E-09
**6h**	6.07E-07	1.68E-07	8.88E-07	5.69E-07
**12h**	1.22E-07	7.08E-08	1.69E-07	1.05E-07
**24h**	2.29E-07	1.27E-07	6.19E-07	7.55E-07
**36h**	2.59E-07	3.43E-07	5.28E-07	4.13E-07
**48h**	9.70E-07	5.04E-07	8.10E-07	3.69E-07
**72h**	1.16E-07	9.18E-08	7.25E-07	2.13E-07
	cgd7_2370			
**2h**	0.00E+00	0.00E+00	1.07E-05	0.00E+00
**6h**	8.21E-07	1.87E-06	1.13E-06	0.00E+00
**12h**	7.00E-06	2.48E-05	1.74E-06	1.86E-05
**24h**	3.08E-06	3.72E-05	4.44E-04	6.10E-05
**36h**	4.76E-05	3.06E-04	4.98E-04	3.18E-04
**48h**	3.62E-05	2.27E-04	3.50E-04	1.56E-04
**72h**	3.66E-05	5.88E-04	1.38E-04	1.19E-04
	cgd8_1940			
**2h**	1.26E-06	8.01E-07	2.49E-07	2.22E-05
**6h**	4.65E-07	2.40E-06	4.92E-07	1.86E-05
**12h**	1.30E-08	2.39E-07	6.87E-06	2.09E-07
**24h**	3.62E-07	3.94E-07	3.17E-07	4.23E-07
**36h**	5.22E-07	2.41E-07	1.14E-07	5.35E-07
**48h**	2.39E-07	1.02E-07	2.33E-07	7.11E-06
**72h**	8.37E-07	2.93E-08	4.73E-08	1.30E-07
	cgd8_1960			
**2h**	0.00E+00	0.00E+00	0.00E+00	0.00E+00
**6h**	3.81E-06	0.00E+00	0.00E+00	6.18E-06
**12h**	1.87E-08	0.00E+00	0.00E+00	6.35E-06
**24h**	5.56E-06	2.16E-06	2.55E-06	3.63E-07
**36h**	2.24E-05	2.23E-05	1.03E-05	1.04E-06
**48h**	1.10E-03	4.74E-04	1.99E-03	2.51E-04
**72h**	2.36E-03	1.23E-03	4.29E-03	6.31E-05
	cgd8_1970			
**2h**	1.88E-06	1.67E-06	1.94E-07	6.73E-05
**6h**	1.53E-07	5.69E-07	1.14E-07	6.48E-06
**12h**	2.86E-08	5.01E-08	6.15E-08	4.31E-07
**24h**	2.01E-07	2.05E-07	7.88E-08	4.79E-08
**36h**	2.12E-07	1.49E-07	7.64E-08	1.58E-07
**48h**	1.40E-06	2.30E-07	8.40E-07	3.28E-06
**72h**	1.50E-06	4.53E-07	1.20E-06	2.17E-06
	cgd8_1980			
**2h**	0.00E+00	0.00E+00	0.00E+00	2.30E-07
**6h**	5.70E-05	6.07E-05	5.39E-05	2.52E-05
**12h**	3.06E-06	3.06E-05	6.60E-06	1.50E-05
**24h**	7.07E-05	2.45E-05	3.29E-05	3.90E-06
**36h**	4.96E-05	1.98E-04	4.17E-05	4.38E-06
**48h**	1.33E-04	1.14E-04	1.51E-04	1.40E-04
**72h**	3.69E-04	2.37E-04	4.25E-04	6.89E-05
	cgd8_1990			
**2h**	0.00E+00	0.00E+00	3.78E-09	0.00E+00
**6h**	0.00E+00	0.00E+00	0.00E+00	1.90E-07
**12h**	0.00E+00	5.24E-10	9.32E-08	7.63E-08

**24h**	1.73E-06	2.36E-07	5.66E-08	5.65E-08
**36h**	8.32E-07	1.17E-06	4.77E-07	9.16E-07
**48h**	2.92E-05	7.21E-06	3.03E-05	1.14E-04
**72h**	4.95E-05	2.06E-05	1.40E-04	1.68E-04
	cgd8_2000			
**2h**	1.28E-05	7.37E-06	3.76E-06	1.10E-05
**6h**	1.72E-06	1.15E-06	4.84E-07	1.35E-06
**12h**	1.79E-06	5.13E-06	3.74E-06	4.21E-06
**24h**	4.68E-06	1.15E-05	2.27E-06	2.63E-06
**36h**	2.44E-06	2.23E-06	1.60E-06	1.08E-06
**48h**	2.37E-06	1.17E-06	2.23E-06	8.15E-06
**72h**	2.99E-06	2.23E-06	5.23E-06	1.08E-06
	cgd8_2010			
**2h**	2.28E-07	6.47E-07	0.00E+00	1.48E-06
**6h**	3.49E-07	1.80E-07	1.96E-07	8.05E-07
**12h**	4.75E-08	2.28E-08	6.18E-08	3.50E-07
**24h**	2.43E-07	2.77E-04	2.24E-07	4.80E-08
**36h**	3.79E-07	5.45E-07	1.60E-07	2.59E-07
**48h**	8.46E-07	3.77E-07	4.25E-07	5.57E-06
**72h**	1.27E-06	4.62E-07	2.77E-06	3.79E-07
	cgd8_2020			
**2h**	1.94E-05	1.32E-05	4.92E-06	1.82E-04
**6h**	4.42E-06	5.37E-06	3.23E-06	1.56E-04
**12h**	0.00E+00	5.33E-06	3.53E-08	2.27E-05
**24h**	1.32E-05	4.50E-05	4.76E-06	1.02E-05
**36h**	2.76E-05	1.13E-05	1.10E-05	1.88E-05
**48h**	5.74E-05	3.07E-05	2.44E-05	8.55E-05
**72h**	3.46E-05	2.97E-05	9.96E-05	5.38E-05
	cgd8_2030			
**2h**	9.15E-06	4.85E-06	2.16E-06	1.29E-05
**6h**	1.45E-06	1.38E-06	9.26E-08	8.84E-07
**12h**	2.05E-06	5.83E-06	2.26E-06	1.09E-05
**24h**	6.19E-06	3.41E-06	1.49E-06	5.79E-07
**36h**	3.93E-06	1.32E-06	2.12E-06	7.51E-07
**48h**	2.26E-06	1.00E-06	1.02E-06	3.27E-06
**72h**	2.20E-05	7.19E-07	2.21E-06	6.01E-06
	cgd8_2040			
**2h**	3.59E-06	2.80E-06	0.00E+00	0.00E+00
**6h**	2.06E-06	3.76E-07	0.00E+00	0.00E+00
**12h**	1.17E-04	2.78E-04	5.26E-05	2.44E-05
**24h**	8.16E-05	1.26E-04	1.77E-05	1.30E-05
**36h**	1.74E-04	7.11E-05	7.05E-05	3.78E-05
**48h**	2.69E-04	6.11E-05	9.45E-05	2.19E-04
**72h**	1.88E-04	9.02E-05	3.13E-04	9.00E-05
	cgd8_2050			
**2h**	1.85E-05	8.69E-06	3.44E-06	6.85E-05
**6h**	1.07E-06	4.71E-06	6.10E-07	9.85E-06
**12h**	2.22E-06	1.71E-06	8.17E-06	1.26E-05
**24h**	2.02E-05	1.49E-05	9.25E-06	8.02E-07
**36h**	5.43E-06	2.74E-06	1.27E-06	1.22E-06
**48h**	3.56E-06	1.44E-06	1.38E-06	3.06E-06
**72h**	7.01E-07	3.07E-07	4.34E-07	2.76E-07
	cgd8_2060			

**2h**	9.55E-07	1.05E-06	2.48E-08	1.57E-06
**6h**	4.84E-08	2.79E-08	2.87E-08	2.72E-07
**12h**	0.00E+00	0.00E+00	0.00E+00	2.56E-06
**24h**	4.56E-08	2.66E-08	9.44E-09	1.15E-08
**36h**	1.27E-07	1.12E-07	8.38E-08	4.80E-08
**48h**	9.23E-07	3.40E-07	1.20E-06	2.12E-04
**72h**	1.39E-06	9.41E-07	3.00E-06	3.96E-06
	cgd8_2070			
**2h**	1.41E-06	8.02E-06	1.57E-07	1.31E-08
**6h**	9.99E-08	2.00E-07	4.37E-09	0.00E+00
**12h**	0.00E+00	0.00E+00	9.55E-07	0.00E+00
**24h**	9.08E-08	7.17E-08	2.73E-08	8.15E-05
**36h**	2.75E-07	2.87E-07	8.79E-08	2.54E-07
**48h**	4.80E-06	3.54E-06	1.09E-05	1.31E-05
**72h**	3.10E-06	1.20E-05	6.90E-06	1.92E-05
	cgd8_2080			
**2h**	1.77E-04	2.84E-04	1.63E-07	3.56E-05
**6h**	3.50E-07	1.81E-05	3.73E-08	4.33E-06
**12h**	4.37E-03	1.87E-02	2.28E-03	2.61E-03
**24h**	1.94E-03	1.17E-03	3.48E-04	2.05E-04
**36h**	4.03E-04	9.53E-04	7.97E-04	2.80E-03
**48h**	7.20E-04	5.22E-04	4.57E-04	4.41E-04
**72h**	3.19E-04	6.73E-04	5.90E-04	7.63E-04
	cgd8_2090			
**2h**	4.71E-03	3.62E-03	1.85E-04	8.19E-03
**6h**	5.41E-04	1.10E-03	2.83E-04	4.34E-04
**12h**	7.10E-04	4.71E-04	7.43E-04	1.58E-03
**24h**	1.32E-03	1.69E-02	5.97E-04	7.04E-04
**36h**	4.62E-04	9.00E-04	2.14E-04	7.04E-04
**48h**	5.54E-04	5.09E-04	4.06E-04	5.60E-04
**72h**	1.73E-04	2.94E-04	2.27E-04	4.97E-04
	cgd8_2100			
**2h**	3.62E-04	1.35E-03	9.44E-05	3.62E-03
**6h**	1.29E-04	2.08E-04	5.14E-05	6.09E-04
**12h**	3.19E-04	1.08E-03	2.01E-04	4.75E-04
**24h**	3.44E-04	9.83E-04	1.27E-04	1.34E-04
**36h**	1.68E-04	4.20E-04	6.21E-05	2.40E-04
**48h**	1.62E-04	2.00E-04	1.26E-04	1.62E-04
**72h**	8.63E-05	1.77E-04	2.62E-04	3.34E-04
	cgd8_2110			
**2h**	1.51E-08	0.00E+00	1.33E-08	1.55E-09
**6h**	1.77E-06	2.69E-06	8.10E-07	1.53E-06
**12h**	6.70E-08	2.36E-07	5.09E-08	9.87E-08
**24h**	1.87E-07	5.22E-07	6.59E-07	1.45E-06
**36h**	8.75E-07	2.01E-06	2.49E-07	1.61E-06
**48h**	3.67E-07	4.38E-07	4.72E-07	2.58E-07
**72h**	9.99E-08	3.63E-07	7.04E-07	5.71E-07
	cgd8_2120			
**2h**	2.17E-07	1.38E-06	1.78E-07	1.83E-06
**6h**	1.23E-06	3.01E-06	1.30E-06	1.94E-06
**12h**	5.09E-08	8.97E-08	2.47E-07	3.47E-07
**24h**	3.36E-07	6.25E-07	6.81E-07	2.31E-06
**36h**	3.04E-07	1.42E-06	2.50E-07	5.36E-07

**48h**	2.35E-07	2.24E-07	2.14E-07	3.18E-07
**72h**	2.04E-08	1.39E-07	1.24E-07	8.36E-08
	cgd8_2130			
**2h**	1.77E-03	3.08E-03	6.20E-04	5.31E-03
**6h**	1.06E-03	4.30E-03	1.41E-03	1.19E-04
**12h**	1.10E-03	1.61E-03	8.25E-04	2.77E-05
**24h**	8.43E-04	7.94E-04	1.72E-03	7.67E-05
**36h**	1.37E-04	3.44E-03	4.28E-04	3.84E-04
**48h**	1.09E-04	2.52E-04	1.12E-03	1.05E-03
**72h**	1.80E-04	6.17E-04	9.72E-04	1.66E-03
	cgd8_2140			
**2h**	1.30E-07	5.17E-08	9.16E-09	1.24E-07
**6h**	6.01E-08	1.93E-07	2.24E-07	4.90E-08
**12h**	1.98E-07	1.61E-07	2.66E-07	3.05E-07
**24h**	2.66E-07	9.45E-07	5.50E-07	1.40E-07
**36h**	2.06E-07	3.12E-07	1.10E-07	1.70E-07
**48h**	2.13E-07	1.79E-07	2.61E-07	9.09E-08
**72h**	1.24E-07	2.69E-07	4.82E-07	3.31E-07
	cgd8_2150			
**2h**	0.00E+00	0.00E+00	0.00E+00	4.20E-09
**6h**	2.57E-08	2.72E-07	2.86E-07	1.27E-07
**12h**	2.59E-06	2.75E-06	1.42E-06	1.63E-06
**24h**	7.91E-07	1.90E-06	3.42E-06	2.07E-06
**36h**	4.84E-06	1.12E-05	4.70E-06	7.50E-06
**48h**	5.49E-06	3.29E-06	4.29E-06	3.30E-06
**72h**	6.06E-07	2.38E-06	1.69E-06	1.32E-06
	cgd8_2160			
**2h**	0.00E+00	0.00E+00	0.00E+00	0.00E+00
**6h**	6.87E-08	6.17E-07	5.98E-08	4.00E-07
**12h**	8.89E-07	2.01E-06	3.66E-07	1.49E-06
**24h**	3.37E-07	6.53E-07	1.61E-07	3.62E-07
**36h**	3.60E-07	1.18E-06	6.97E-07	9.89E-07
**48h**	6.30E-07	6.33E-07	3.45E-07	4.62E-07
**72h**	2.13E-07	8.28E-07	4.99E-07	4.04E-07
	cgd8_2180			
**2h**	0.00E+00	0.00E+00	0.00E+00	0.00E+00
**6h**	0.00E+00	0.00E+00	0.00E+00	0.00E+00
**12h**	0.00E+00	6.92E-09	0.00E+00	0.00E+00
**24h**	5.35E-09	3.29E-08	4.05E-10	4.25E-07
**36h**	5.37E-07	6.39E-07	6.48E-08	2.63E-07
**48h**	1.61E-05	9.32E-06	4.58E-06	4.53E-06
**72h**	1.20E-06	2.42E-06	2.50E-06	1.12E-06
	cgd8_2190			
**2h**	1.29E-06	5.23E-07	2.61E-08	3.30E-08
**6h**	7.01E-08	6.17E-08	4.96E-09	0.00E+00
**12h**	3.76E-10	2.63E-08	7.85E-09	0.00E+00
**24h**	1.13E-08	8.94E-09	2.15E-08	1.94E-06
**36h**	6.44E-08	2.14E-07	1.47E-08	3.32E-08
**48h**	1.08E-07	1.97E-07	1.08E-07	7.66E-08
**72h**	4.11E-08	1.43E-07	3.01E-07	1.52E-07
	cgd8_2200			
**2h**	1.45E-07	3.37E-07	6.06E-08	6.97E-08
**6h**	1.76E-06	7.54E-07	1.48E-06	8.40E-06

**12h**	5.11E-08	1.90E-07	2.75E-07	2.30E-06
**24h**	6.49E-07	3.51E-07	5.63E-07	3.73E-06
**36h**	1.23E-07	6.36E-07	1.52E-07	7.61E-08
**48h**	3.89E-07	7.59E-08	2.88E-07	1.30E-06
**72h**	1.97E-08	1.63E-07	6.79E-08	4.26E-08
	cgd8_2210			
**2h**	0.00E+00	0.00E+00	2.32E-08	0.00E+00
**6h**	1.81E-06	8.89E-07	9.65E-07	1.89E-04
**12h**	5.57E-07	5.56E-06	1.46E-05	4.25E-04
**24h**	2.07E-06	2.87E-06	4.08E-05	5.55E-05
**36h**	1.57E-06	1.38E-05	1.94E-05	1.05E-05
**48h**	2.05E-05	9.58E-06	1.29E-04	7.15E-05
**72h**	7.71E-07	3.23E-06	1.17E-05	7.09E-06
	cgd8_2220			
**2h**	0.00E+00	0.00E+00	0.00E+00	0.00E+00
**6h**	0.00E+00	0.00E+00	0.00E+00	0.00E+00
**12h**	0.00E+00	1.83E-08	2.14E-07	5.65E-07
**24h**	3.40E-07	1.33E-07	4.45E-07	3.18E-07
**36h**	6.33E-07	3.77E-06	2.98E-06	1.96E-07
**48h**	1.01E-05	1.15E-05	3.27E-05	5.80E-05
**72h**	6.59E-06	1.38E-05	1.88E-05	1.31E-05
	cgd8_2260			
**2h**	3.49E-09	9.10E-08	1.02E-08	4.48E-08
**6h**	5.11E-07	9.77E-07	6.33E-07	1.10E-06
**12h**	1.60E-07	7.70E-07	5.26E-07	6.75E-07
**24h**	3.32E-07	6.03E-07	6.85E-07	4.23E-07
**36h**	7.90E-07	1.60E-06	2.41E-06	1.95E-07
**48h**	4.41E-07	3.82E-07	7.62E-07	2.89E-06
**72h**	1.74E-07	4.50E-07	6.50E-07	2.87E-07
	cgd8_2270			
**2h**	0.00E+00	0.00E+00	0.00E+00	0.00E+00
**6h**	6.55E-09	1.43E-09	0.00E+00	0.00E+00
**12h**	4.24E-07	4.63E-06	2.14E-06	3.82E-06
**24h**	5.38E-07	1.20E-06	4.11E-07	3.82E-07
**36h**	4.31E-07	2.43E-06	1.48E-06	2.51E-07
**48h**	9.06E-07	5.62E-07	1.86E-06	4.17E-06
**72h**	3.10E-06	3.14E-06	4.00E-06	1.01E-06
	cgd8_2300			
**2h**	1.95E-03	4.84E-03	2.83E-03	1.30E-04
**6h**	5.74E-03	3.26E-03	2.07E-03	1.00E-03
**12h**	9.15E-04	4.62E-03	6.53E-04	1.16E-03
**24h**	1.60E-03	2.97E-03	2.95E-03	5.01E-04
**36h**	5.55E-03	5.57E-03	5.14E-04	8.58E-05
**48h**	4.61E-03	1.95E-03	6.06E-04	3.45E-04
**72h**	1.64E-04	1.97E-03	2.99E-04	5.23E-05
	cgd8_2320			
**2h**	9.02E-07	2.22E-05	0.00E+00	0.00E+00
**6h**	1.02E-03	1.13E-03	5.43E-04	1.04E-03
**12h**	2.14E-04	8.37E-04	1.30E-04	2.40E-04
**24h**	4.83E-04	3.58E-04	3.22E-04	6.94E-05
**36h**	1.04E-03	1.40E-03	1.24E-04	1.23E-05
**48h**	2.07E-03	2.87E-04	1.71E-04	7.46E-05
**72h**	7.24E-04	4.70E-04	1.26E-04	6.60E-05

	cgd8_2360			
**2h**	8.40E-07	2.25E-06	1.19E-06	2.90E-06
**6h**	2.09E-06	3.63E-06	2.41E-05	2.69E-05
**12h**	9.01E-08	2.45E-07	1.75E-06	1.21E-06
**24h**	1.56E-06	2.30E-06	9.88E-06	5.11E-06
**36h**	2.93E-06	4.16E-06	6.37E-06	1.44E-06
**48h**	3.18E-06	6.60E-06	1.31E-05	7.47E-05
**72h**	2.46E-05	1.57E-05	7.29E-05	1.12E-05
	cgd8_2370			
**2h**	3.31E-07	8.57E-07	9.09E-08	9.11E-07
**6h**	4.73E-07	6.28E-07	6.35E-06	8.82E-06
**12h**	9.41E-08	3.38E-07	2.40E-06	3.31E-06
**24h**	3.13E-07	9.83E-07	6.49E-06	1.93E-06
**36h**	8.23E-07	4.49E-06	2.71E-06	2.62E-07
**48h**	7.18E-06	7.66E-06	1.09E-05	1.42E-05
**72h**	6.55E-06	3.10E-06	3.13E-05	6.24E-06
	cgd8_2380			
**2h**	0.00E+00	1.56E-08	0.00E+00	1.27E-08
**6h**	1.32E-07	5.45E-08	1.11E-07	1.64E-07
**12h**	1.43E-08	1.53E-08	2.02E-08	9.87E-09
**24h**	3.67E-08	8.00E-08	9.66E-08	5.67E-08
**36h**	2.27E-07	2.56E-07	1.96E-07	1.10E-08
**48h**	1.51E-07	2.91E-07	1.58E-07	1.17E-07
**72h**	9.27E-08	1.74E-07	1.42E-07	5.52E-08
	cgd8_2390			
**2h**	0.00E+00	0.00E+00	0.00E+00	0.00E+00
**6h**	0.00E+00	0.00E+00	0.00E+00	0.00E+00
**12h**	6.17E-08	6.86E-07	4.80E-08	1.75E-07
**24h**	4.40E-08	9.62E-08	3.36E-08	4.23E-08
**36h**	4.74E-07	6.79E-07	4.04E-07	3.63E-08
**48h**	8.19E-06	1.88E-07	7.79E-07	1.22E-05
**72h**	2.76E-06	3.11E-07	1.17E-06	6.87E-06
	cgd8_2410			
**2h**	6.76E-09	1.58E-08	0.00E+00	0.00E+00
**6h**	2.60E-07	4.69E-07	3.22E-07	3.38E-05
**12h**	1.19E-07	9.66E-08	7.04E-07	4.22E-06
**24h**	3.13E-07	1.94E-07	9.76E-07	8.62E-06
**36h**	6.58E-07	4.29E-07	5.10E-07	3.10E-06
**48h**	3.50E-07	2.03E-07	3.11E-07	1.44E-05
**72h**	4.70E-08	1.98E-07	2.57E-07	2.03E-08
	cgd8_2420			
**2h**	5.89E-07	1.85E-07	1.83E-07	8.25E-06
**6h**	2.34E-07	2.74E-07	4.59E-07	4.00E-05
**12h**	3.68E-08	5.43E-08	4.71E-07	7.51E-06
**24h**	2.11E-07	5.21E-07	6.37E-07	2.24E-06
**36h**	3.34E-07	4.21E-07	3.48E-07	2.57E-06
**48h**	1.75E-07	7.82E-08	6.24E-07	2.15E-07
**72h**	8.67E-08	1.93E-07	2.71E-07	1.12E-06
	cgd8_2430			
**2h**	6.43E-06	5.23E-07	2.44E-07	5.85E-04
**6h**	1.92E-06	6.13E-07	3.57E-06	1.11E-04
**12h**	3.02E-07	1.10E-07	5.45E-06	3.42E-07
**24h**	2.14E-06	5.20E-07	4.97E-06	1.17E-06

**36h**	2.26E-06	1.11E-06	2.46E-06	8.90E-06
**48h**	3.46E-06	8.15E-07	9.12E-06	2.99E-06
**72h**	3.04E-06	9.79E-07	2.21E-06	3.36E-06
	cgd8_2450			
**2h**	8.15E-06	2.13E-06	8.44E-07	5.01E-05
**6h**	4.13E-07	2.70E-07	1.12E-06	4.74E-07
**12h**	8.90E-08	2.19E-07	1.67E-06	5.98E-08
**24h**	4.33E-07	9.42E-07	1.23E-06	1.23E-07
**36h**	7.31E-07	1.03E-06	7.49E-07	3.35E-07
**48h**	7.58E-07	2.56E-07	7.23E-07	3.06E-07
**72h**	4.35E-07	8.44E-07	8.08E-07	7.45E-07
	cgd8_2460			
**2h**	2.41E-06	3.11E-07	1.20E-06	1.42E-05
**6h**	4.63E-07	2.24E-07	4.97E-06	7.87E-06
**12h**	2.58E-07	1.02E-07	1.35E-06	1.03E-06
**24h**	3.51E-07	4.94E-07	6.18E-06	1.41E-06
**36h**	1.36E-06	1.06E-06	8.09E-07	2.32E-06
**48h**	9.70E-07	9.75E-08	3.17E-07	1.04E-06
**72h**	1.29E-07	9.89E-08	1.36E-07	2.18E-07
	cgd8_2470			
**2h**	5.10E-07	3.06E-07	2.96E-07	1.14E-06
**6h**	2.36E-07	2.75E-08	3.84E-07	1.37E-07
**12h**	2.90E-07	8.70E-08	8.73E-07	4.31E-08
**24h**	1.62E-07	3.54E-07	1.75E-07	3.69E-08
**36h**	4.81E-07	2.77E-07	6.31E-07	2.29E-07
**48h**	3.08E-07	1.24E-07	2.46E-07	6.35E-08
**72h**	2.03E-07	7.18E-08	2.26E-07	1.73E-07
	cgd8_2490			
**2h**	2.82E-08	2.47E-08	3.55E-07	1.39E-06
**6h**	0.00E+00	0.00E+00	0.00E+00	0.00E+00
**12h**	7.60E-05	2.28E-05	1.86E-04	6.45E-06
**24h**	1.90E-05	5.41E-06	6.59E-06	4.50E-06
**36h**	3.77E-06	2.33E-06	2.37E-05	1.61E-05
**48h**	2.91E-06	3.52E-07	3.38E-06	4.18E-06
**72h**	1.36E-07	2.63E-07	7.18E-07	3.35E-08
	cgd8_2500			
**2h**	3.32E-06	2.65E-06	3.30E-06	8.60E-06
**6h**	9.97E-06	1.11E-06	1.99E-05	3.86E-06
**12h**	3.52E-07	8.57E-07	4.02E-06	5.38E-07
**24h**	3.63E-06	2.39E-06	7.16E-06	1.03E-06
**36h**	1.23E-06	5.62E-06	3.19E-06	2.22E-06
**48h**	1.84E-06	1.42E-06	2.12E-06	8.98E-07
**72h**	4.12E-07	7.96E-07	1.10E-06	9.73E-08
	cgd8_2510			
**2h**	3.01E-06	1.85E-06	2.81E-06	2.48E-05
**6h**	4.27E-06	3.92E-07	9.55E-06	1.21E-06
**12h**	5.91E-08	4.55E-07	8.77E-07	1.20E-07
**24h**	1.65E-06	3.06E-07	5.25E-06	6.64E-07
**36h**	9.65E-07	1.44E-06	1.28E-06	3.40E-06
**48h**	1.86E-06	7.16E-07	2.01E-06	9.70E-06
**72h**	4.82E-07	2.10E-07	1.79E-06	1.49E-07
	cgd8_2520			
**2h**	4.34E-05	2.41E-05	1.30E-05	1.97E-04

**6h**	6.10E-06	1.07E-06	7.39E-06	1.05E-05
**12h**	5.03E-06	1.19E-06	2.40E-05	5.26E-06
**24h**	2.74E-05	1.11E-05	1.15E-05	1.28E-05
**36h**	5.92E-06	2.12E-06	8.18E-06	9.04E-06
**48h**	6.25E-06	1.68E-06	4.73E-06	2.96E-05
**72h**	3.90E-06	8.39E-07	6.07E-06	2.74E-06
	cgd8_2540			
**2h**	0.00E+00	0.00E+00	0.00E+00	4.27E-06
**6h**	0.00E+00	3.97E-08	0.00E+00	0.00E+00
**12h**	5.71E-04	1.53E-04	1.09E-03	8.97E-06
**24h**	1.70E-04	4.18E-05	1.63E-04	1.14E-05
**36h**	2.78E-04	3.66E-05	4.95E-04	8.83E-05
**48h**	2.15E-04	8.86E-06	2.16E-04	1.30E-05
**72h**	6.05E-05	1.19E-05	5.42E-05	9.13E-06
	cgd8_2550			
**2h**	2.09E-07	3.50E-08	0.00E+00	0.00E+00
**6h**	1.71E-06	5.51E-07	1.07E-05	1.81E-05
**12h**	7.93E-07	5.56E-06	1.56E-05	1.93E-06
**24h**	2.69E-06	1.34E-06	1.19E-05	4.89E-06
**36h**	1.36E-05	4.15E-06	2.85E-05	1.53E-05
**48h**	1.10E-05	1.98E-06	1.49E-05	3.33E-05
**72h**	3.28E-06	5.22E-07	4.99E-06	1.90E-06
	cgd8_2560			
**2h**	4.94E-06	2.78E-06	1.54E-06	5.42E-06
**6h**	3.40E-06	3.92E-06	1.63E-04	9.91E-06
**12h**	2.94E-07	1.03E-05	4.65E-05	1.11E-06
**24h**	1.86E-06	2.51E-06	1.80E-05	3.03E-06
**36h**	1.80E-06	1.19E-06	5.41E-06	6.73E-06
**48h**	1.20E-06	6.40E-07	1.74E-06	4.75E-06
**72h**	8.43E-08	3.26E-07	3.98E-07	1.64E-06
	cgd8_2580			
**2h**	0.00E+00	7.02E-08	0.00E+00	0.00E+00
**6h**	0.00E+00	7.19E-08	9.79E-07	0.00E+00
**12h**	4.58E-07	4.10E-06	2.60E-06	0.00E+00
**24h**	6.11E-07	1.60E-06	3.83E-06	0.00E+00
**36h**	1.25E-06	4.47E-06	3.16E-06	0.00E+00
**48h**	1.50E-06	4.39E-06	2.72E-06	0.00E+00
**72h**	4.85E-07	1.06E-06	1.26E-06	0.00E+00
	cgd8_2620			
**2h**	0.00E+00	0.00E+00	0.00E+00	0.00E+00
**6h**	0.00E+00	3.36E-07	8.94E-08	3.26E-08
**12h**	2.57E-09	2.67E-08	1.17E-08	1.08E-08
**24h**	1.72E-08	5.33E-08	1.01E-07	8.27E-08
**36h**	5.78E-08	2.54E-07	7.31E-08	1.45E-07
**48h**	7.04E-08	9.04E-08	4.47E-08	2.16E-07
**72h**	1.07E-08	1.41E-07	3.72E-08	3.82E-08
	cgd8_2630			
**2h**	7.18E-06	1.44E-06	0.00E+00	1.18E-07
**6h**	3.62E-05	6.65E-05	7.64E-05	1.36E-05
**12h**	5.96E-06	2.79E-06	1.70E-06	8.99E-07
**24h**	1.81E-06	3.39E-04	3.14E-05	8.51E-06
**36h**	3.33E-05	3.04E-05	1.21E-05	8.87E-06
**48h**	6.19E-06	9.16E-06	7.12E-06	7.71E-06

**72h**	7.81E-07	1.91E-06	6.96E-07	7.40E-07
	cgd8_2640			
**2h**	0.00E+00	0.00E+00	0.00E+00	0.00E+00
**6h**	0.00E+00	0.00E+00	0.00E+00	0.00E+00
**12h**	1.16E-07	1.70E-06	2.93E-07	4.52E-07
**24h**	1.40E-07	2.00E-06	2.45E-06	2.34E-06
**36h**	5.73E-06	7.01E-06	1.21E-06	2.33E-06
**48h**	1.93E-06	1.71E-06	6.77E-07	5.10E-06
**72h**	4.98E-07	1.22E-06	7.06E-07	7.88E-07
	cgd8_2650			
**2h**	1.01E-06	5.81E-06	0.00E+00	0.00E+00
**6h**	2.20E-04	1.46E-03	9.66E-04	4.79E-04
**12h**	1.87E-04	3.68E-04	3.10E-04	8.41E-05
**24h**	9.10E-05	6.45E-04	6.26E-04	2.61E-04
**36h**	4.05E-04	7.67E-04	3.03E-04	3.57E-04
**48h**	1.18E-03	3.74E-04	1.29E-04	5.00E-04
**72h**	9.07E-05	2.87E-04	5.36E-05	5.48E-05
	cgd8_2660			
**2h**	4.47E-06	4.93E-06	1.27E-06	2.25E-06
**6h**	1.17E-06	2.97E-06	1.29E-06	9.59E-07
**12h**	3.89E-06	8.07E-06	9.43E-06	2.46E-06
**24h**	4.11E-06	5.87E-06	2.74E-06	1.26E-06
**36h**	1.00E-06	3.20E-06	1.19E-06	1.53E-06
**48h**	8.74E-07	6.79E-07	4.87E-07	1.02E-06
**72h**	1.27E-07	7.78E-07	2.40E-07	4.36E-07
	cgd8_2670			
**2h**	7.68E-07	5.08E-07	5.03E-08	1.32E-07
**6h**	1.37E-07	7.17E-08	4.90E-08	2.12E-07
**12h**	4.70E-07	1.57E-06	9.54E-07	5.99E-07
**24h**	6.40E-07	1.31E-06	4.84E-07	1.80E-07
**36h**	4.92E-07	8.99E-07	2.22E-07	6.05E-07
**48h**	1.18E-05	1.27E-05	2.43E-06	4.20E-06
**72h**	1.24E-07	6.55E-07	1.02E-07	2.20E-07
	cgd8_2710			
**2h**	1.37E-03	0.00E+00	0.00E+00	1.81E-04
**6h**	2.99E-07	2.65E-05	1.71E-05	0.00E+00
**12h**	8.71E-06	7.51E-06	7.39E-05	2.98E-05
**24h**	8.77E-06	1.42E-04	5.75E-05	1.35E-05
**36h**	1.67E-05	7.20E-04	1.38E-04	7.99E-04
**48h**	1.44E-05	5.82E-04	2.19E-04	1.02E-03
**72h**	5.21E-05	6.26E-04	8.67E-05	2.61E-04
	cgd8_2720			
**2h**	3.18E-05	3.74E-05	1.93E-06	5.45E-06
**6h**	1.26E-05	1.32E-04	6.95E-05	6.69E-05
**12h**	5.68E-06	1.76E-06	1.23E-05	1.21E-05
**24h**	8.95E-06	3.96E-05	7.05E-05	3.61E-05
**36h**	1.23E-05	3.11E-05	1.02E-05	1.54E-05
**48h**	2.42E-05	1.59E-05	7.24E-06	2.17E-05
**72h**	9.13E-06	1.40E-05	2.38E-06	1.07E-05
	cgd8_2750			
**2h**	0.00E+00	0.00E+00	0.00E+00	0.00E+00
**6h**	0.00E+00	0.00E+00	0.00E+00	0.00E+00
**12h**	1.76E-04	4.29E-04	1.17E-04	1.48E-04

**24h**	1.50E-05	7.46E-05	1.91E-05	1.52E-05
**36h**	1.50E-04	1.42E-04	1.68E-04	4.65E-04
**48h**	2.65E-04	2.07E-04	1.68E-04	6.62E-04
**72h**	7.79E-05	1.83E-04	8.02E-05	5.51E-05
	cgd8_2760			
**2h**	0.00E+00	0.00E+00	0.00E+00	0.00E+00
**6h**	1.62E-04	7.47E-05	1.66E-04	4.65E-05
**12h**	1.39E-04	5.42E-04	6.53E-05	4.17E-04
**24h**	1.32E-04	5.91E-04	6.67E-04	3.81E-04
**36h**	8.74E-05	1.81E-03	4.71E-04	1.27E-03
**48h**	2.81E-04	9.91E-04	5.27E-04	1.82E-04
**72h**	9.04E-05	1.33E-03	6.27E-04	3.03E-03
	cgd8_2780			
**2h**	0.00E+00	0.00E+00	0.00E+00	0.00E+00
**6h**	1.01E-06	1.55E-06	6.70E-06	1.10E-01
**12h**	2.07E-07	3.75E-07	4.91E-06	1.28E-03
**24h**	3.08E-07	1.17E-06	2.23E-06	2.38E-04
**36h**	4.50E-07	8.13E-07	1.17E-06	7.55E-05
**48h**	3.78E-07	2.06E-07	3.39E-07	2.48E-04
**72h**	4.35E-08	8.29E-08	1.85E-07	8.47E-07
	cgd8_2790			
**2h**	8.00E-09	0.00E+00	0.00E+00	7.25E-08
**6h**	0.00E+00	2.59E-08	5.69E-09	1.48E-07
**12h**	3.46E-05	6.96E-05	3.02E-05	7.55E-05
**24h**	5.67E-06	7.75E-06	4.74E-06	7.32E-06
**36h**	1.02E-06	2.94E-06	7.32E-06	1.10E-05
**48h**	2.10E-06	1.40E-06	2.80E-06	7.25E-05
**72h**	2.20E-07	1.08E-06	6.68E-07	2.33E-06
	cgd8_2800			
**2h**	7.27E-09	1.15E-09	0.00E+00	0.00E+00
**6h**	2.63E-08	9.79E-09	1.79E-08	3.62E-08
**12h**	1.80E-08	1.22E-09	3.74E-09	1.37E-08
**24h**	9.01E-09	8.74E-09	1.09E-08	1.89E-08
**36h**	7.18E-09	4.74E-08	2.47E-08	2.14E-08
**48h**	7.56E-07	1.97E-07	1.72E-07	2.12E-06
**72h**	1.64E-07	2.07E-07	5.48E-07	2.71E-07
	cgd8_2810			
**2h**	4.98E-06	1.42E-05	3.58E-06	1.77E-03
**6h**	3.55E-05	1.08E-04	1.32E-04	1.10E-03
**12h**	4.38E-07	2.59E-06	1.28E-05	1.39E-04
**24h**	5.53E-06	5.55E-05	8.57E-05	4.89E-04
**36h**	1.38E-06	3.13E-05	2.25E-05	2.53E-04
**48h**	9.73E-06	2.57E-05	7.38E-05	6.66E-02
**72h**	1.03E-06	1.35E-05	2.98E-05	7.98E-04
	cgd8_2820			
**2h**	1.09E-07	1.07E-07	0.00E+00	2.11E-07
**6h**	3.48E-07	7.00E-07	5.65E-07	7.25E-07
**12h**	7.45E-08	6.16E-08	5.82E-07	1.18E-07
**24h**	9.19E-08	2.31E-07	5.15E-07	4.23E-07
**36h**	3.08E-07	2.75E-07	7.06E-07	4.44E-07
**48h**	1.09E-07	1.17E-07	1.13E-07	2.28E-06
**72h**	3.43E-08	9.26E-08	1.14E-07	3.10E-07
	cgd8_2830			

**2h**	2.03E-07	7.48E-07	3.50E-07	1.41E-04
**6h**	9.26E-06	5.97E-05	1.53E-04	2.41E-03
**12h**	5.30E-07	1.84E-06	1.42E-05	9.08E-04
**24h**	1.30E-06	3.25E-05	3.99E-04	7.60E-04
**36h**	5.26E-06	5.45E-05	2.50E-05	8.34E-03
**48h**	6.32E-06	2.49E-05	4.33E-05	2.76E-01
**72h**	1.38E-06	1.94E-05	1.58E-05	3.01E-05
	cgd8_2840			
**2h**	0.00E+00	0.00E+00	0.00E+00	0.00E+00
**6h**	4.61E-08	1.83E-07	1.17E-07	7.64E-06
**12h**	2.18E-06	1.87E-05	3.77E-06	9.58E-05
**24h**	2.95E-07	3.62E-06	2.50E-06	2.45E-06
**36h**	7.73E-07	1.05E-05	5.77E-06	4.12E-05
**48h**	9.89E-07	2.45E-06	3.90E-06	7.34E-03
**72h**	4.10E-07	2.14E-06	1.02E-06	1.46E-06
	cgd8_2850			
**2h**	5.85E-09	1.46E-08	3.29E-08	1.21E-08
**6h**	1.40E-08	5.90E-09	2.47E-08	0.00E+00
**12h**	1.89E-09	1.85E-08	2.72E-08	3.06E-08
**24h**	1.95E-08	3.01E-08	1.10E-07	2.94E-08
**36h**	3.69E-08	1.66E-07	1.22E-07	2.60E-07
**48h**	3.80E-08	4.86E-08	1.01E-07	2.82E-06
**72h**	2.65E-08	3.88E-08	3.05E-08	8.36E-09
	cgd8_2860			
**2h**	3.92E-06	3.19E-06	1.75E-06	5.01E-06
**6h**	9.19E-06	5.47E-06	2.84E-05	6.34E-06
**12h**	5.25E-07	2.13E-06	6.10E-06	4.39E-06
**24h**	2.22E-06	4.20E-06	1.33E-05	4.40E-06
**36h**	1.24E-06	4.32E-06	2.76E-06	3.49E-06
**48h**	1.35E-06	1.49E-06	1.88E-06	7.90E-06
**72h**	5.94E-07	4.45E-07	5.26E-07	4.65E-06
	cgd8_2870			
**2h**	1.07E-04	6.82E-05	1.40E-04	7.44E-05
**6h**	4.66E-04	1.23E-04	4.88E-04	1.16E-03
**12h**	3.51E-05	6.45E-05	2.56E-04	5.71E-05
**24h**	1.28E-04	9.99E-05	1.82E-04	3.98E-05
**36h**	3.97E-05	6.97E-05	3.62E-05	4.79E-05
**48h**	2.24E-05	2.29E-05	3.85E-05	3.24E-04
**72h**	1.67E-05	1.01E-05	9.04E-06	1.52E-05
	cgd8_2890			
**2h**	5.70E-04	3.20E-04	4.72E-03	1.11E-01
**6h**	1.01E-03	1.12E-03	2.38E-02	6.77E-01
**12h**	2.24E-04	1.26E-03	4.04E-03	1.26E-01
**24h**	1.19E-04	2.22E-03	3.48E-03	1.12E-02
**36h**	4.46E-04	5.26E-04	4.06E-04	1.45E-02
**48h**	2.35E-04	1.69E-04	3.65E-03	6.99E-01
**72h**	5.46E-05	2.62E-04	3.16E-04	3.36E-04
	cgd8_2910			
**2h**	2.91E-06	4.13E-08	0.00E+00	9.01E-08
**6h**	1.55E-07	1.24E-07	4.39E-07	3.16E-06
**12h**	1.16E-07	3.45E-07	3.70E-07	5.70E-07
**24h**	1.08E-07	2.21E-07	1.32E-06	1.12E-06
**36h**	8.48E-07	1.56E-06	1.02E-06	2.16E-06

**48h**	4.00E-07	4.46E-07	5.02E-07	6.20E-05
**72h**	2.98E-07	2.90E-07	4.23E-07	7.56E-07
	cgd8_2940			
**2h**	8.90E-07	1.63E-05	4.03E-07	8.26E-05
**6h**	2.05E-04	5.31E-05	5.94E-05	2.35E-04
**12h**	1.69E-05	2.49E-06	2.78E-05	5.22E-05
**24h**	1.70E-05	6.44E-06	4.27E-05	2.50E-04
**36h**	1.39E-05	1.87E-05	2.07E-08	5.29E-05
**48h**	4.47E-05	7.56E-06	1.49E-05	1.35E-04
**72h**	9.15E-07	2.07E-06	1.94E-06	4.45E-05
	cgd8_2950			
**2h**	9.38E-04	7.68E-04	5.06E-04	1.92E-05
**6h**	9.67E-04	2.34E-03	1.05E-03	9.36E-05
**12h**	4.38E-04	0.00E+00	1.02E-03	1.60E-04
**24h**	6.87E-04	1.19E-03	5.17E-03	3.62E-05
**36h**	3.29E-04	2.45E-03	1.16E-06	1.77E-04
**48h**	3.88E-04	4.36E-03	5.88E-03	4.39E-05
**72h**	6.37E-04	2.91E-03	2.92E-03	8.01E-06
	cgd8_2960			
**2h**	1.95E-03	4.44E-04	2.64E-05	4.72E-05
**6h**	4.42E-04	5.27E-04	2.64E-04	9.99E-05
**12h**	4.95E-05	1.45E-05	1.96E-04	6.65E-05
**24h**	1.97E-04	8.67E-04	5.21E-04	4.59E-04
**36h**	1.40E-04	2.13E-04	3.49E-07	1.63E-04
**48h**	3.02E-04	1.61E-04	1.95E-04	5.39E-05
**72h**	1.24E-05	2.79E-05	2.83E-05	8.00E-05
	cgd8_2970			
**2h**	0.00E+00	6.40E-07	4.76E-07	1.41E-06
**6h**	5.89E-06	1.90E-06	2.58E-06	3.35E-06
**12h**	4.50E-07	1.75E-07	7.78E-07	1.66E-06
**24h**	4.37E-07	1.96E-06	1.65E-06	2.14E-06
**36h**	1.55E-06	1.07E-06	1.69E-09	6.67E-07
**48h**	1.22E-06	5.65E-07	3.72E-07	6.74E-07
**72h**	1.58E-07	1.73E-07	1.04E-07	1.85E-07
	cgd8_2980			
**2h**	3.14E-05	7.33E-05	1.03E-05	1.72E-04
**6h**	2.09E-04	3.69E-04	1.84E-04	7.04E-04
**12h**	2.41E-05	3.94E-05	5.97E-05	2.16E-04
**24h**	1.52E-04	1.86E-04	1.65E-04	3.65E-04
**36h**	6.09E-05	8.72E-05	1.04E-07	1.85E-03
**48h**	2.88E-04	5.15E-05	7.03E-05	5.96E-05
**72h**	1.18E-04	8.10E-06	1.42E-05	2.05E-04
	cgd8_2990			
**2h**	7.08E-07	2.03E-07	7.60E-08	1.50E-06
**6h**	4.38E-07	4.25E-07	4.41E-07	2.24E-05
**12h**	2.12E-07	2.74E-07	2.19E-07	3.97E-06
**24h**	4.06E-07	5.31E-07	7.77E-07	1.31E-05
**36h**	1.04E-06	1.54E-06	1.11E-09	7.02E-06
**48h**	3.82E-06	9.44E-07	3.99E-07	1.75E-05
**72h**	1.57E-07	2.92E-07	4.24E-07	5.48E-06
	cgd8_3000			
**2h**	3.93E-05	4.18E-05	2.09E-05	9.70E-04
**6h**	3.38E-05	7.65E-05	2.92E-05	3.23E-03

**12h**	1.37E-05	1.34E-05	3.66E-05	1.52E-03
**24h**	9.70E-05	8.87E-05	7.31E-05	4.85E-04
**36h**	2.93E-05	7.66E-05	3.26E-08	5.40E-04
**48h**	4.70E-05	2.59E-05	2.97E-05	6.91E-04
**72h**	3.16E-06	8.95E-06	6.11E-06	3.45E-04
	cgd8_3020			
**2h**	4.78E-09	1.37E-08	0.00E+00	6.26E-08
**6h**	3.64E-08	3.72E-09	2.70E-09	4.59E-07
**12h**	2.48E-06	3.59E-06	1.21E-06	9.81E-06
**24h**	5.01E-07	6.93E-07	1.05E-06	2.92E-06
**36h**	1.97E-06	1.66E-06	3.66E-09	1.19E-05
**48h**	1.12E-06	6.30E-07	5.36E-07	6.76E-06
**72h**	2.88E-08	5.79E-08	8.67E-08	7.33E-07
	cgd8_3030			
**2h**	8.84E-07	0.00E+00	0.00E+00	0.00E+00
**6h**	0.00E+00	0.00E+00	0.00E+00	3.17E-06
**12h**	1.59E-04	1.11E-04	7.57E-05	8.88E-05
**24h**	4.20E-05	5.20E-05	4.01E-05	1.48E-05
**36h**	4.17E-05	9.70E-05	2.08E-07	5.00E-05
**48h**	8.68E-05	2.17E-05	4.14E-05	5.98E-06
**72h**	3.59E-06	6.98E-06	1.05E-05	2.41E-05
	cgd8_3040			
**2h**	3.33E-07	4.34E-08	0.00E+00	8.47E-06
**6h**	3.24E-07	6.70E-08	9.12E-08	2.15E-05
**12h**	4.56E-06	5.57E-06	2.33E-06	1.72E-04
**24h**	4.08E-06	1.94E-06	3.42E-06	6.74E-05
**36h**	8.37E-07	1.53E-06	4.50E-09	5.07E-05
**48h**	1.14E-06	4.44E-07	7.57E-07	3.20E-05
**72h**	7.80E-08	2.04E-07	2.42E-07	1.45E-05
	cgd8_3050			
**2h**	1.04E-03	7.18E-04	1.45E-03	9.18E-05
**6h**	1.38E-03	7.90E-04	4.71E-03	2.52E-04
**12h**	7.40E-04	4.46E-04	9.64E-04	2.77E-03
**24h**	1.01E-03	1.63E-03	2.16E-03	5.63E-04
**36h**	5.09E-04	1.30E-03	1.15E-06	2.92E-04
**48h**	9.38E-04	4.03E-04	4.56E-04	2.30E-04
**72h**	2.44E-05	1.36E-04	2.29E-04	6.28E-05
	cgd8_3060			
**2h**	1.02E-05	1.40E-05	2.77E-06	4.26E-05
**6h**	5.78E-05	4.34E-05	1.54E-04	1.54E-04
**12h**	3.89E-05	3.56E-06	1.83E-05	7.92E-05
**24h**	2.99E-05	1.91E-05	2.29E-04	1.83E-04
**36h**	6.06E-05	9.39E-05	2.24E-07	7.03E-05
**48h**	8.33E-05	2.87E-05	3.72E-05	3.76E-05
**72h**	5.31E-06	1.05E-05	2.72E-05	1.05E-04
	cgd7_2380			
**2h**	6.70E-06	9.29E-07	5.04E-06	3.72E-06
**6h**	6.12E-06	6.51E-06	3.58E-06	1.72E-05
**12h**	1.31E-06	8.76E-07	1.68E-06	1.35E-06
**24h**	6.05E-06	3.83E-06	4.09E-06	5.20E-06
**36h**	2.62E-06	3.25E-06	2.93E-06	2.48E-06
**48h**	1.33E-06	9.22E-07	2.26E-06	4.20E-06
**72h**	6.86E-07	8.27E-08	9.01E-07	1.26E-06

	cgd7_2400			
**2h**	1.77E-04	1.77E-04	2.58E-04	8.33E-05
**6h**	9.11E-05	1.20E-04	1.24E-04	2.23E-05
**12h**	3.18E-03	6.96E-03	9.46E-04	4.22E-04
**24h**	7.90E-05	7.26E-04	4.85E-04	1.92E-04
**36h**	3.76E-04	2.93E-04	5.60E-04	1.84E-04
**48h**	2.62E-04	1.65E-04	4.89E-04	1.54E-04
**72h**	2.59E-04	6.01E-05	2.48E-04	2.25E-04
	cgd7_2410			
**2h**	1.26E-04	1.39E-05	6.92E-06	2.84E-05
**6h**	1.50E-05	2.48E-05	1.42E-06	2.16E-06
**12h**	1.12E-03	6.46E-04	6.02E-04	2.31E-04
**24h**	3.83E-04	1.91E-04	1.22E-04	1.24E-04
**36h**	2.11E-04	1.06E-04	1.38E-04	1.02E-04
**48h**	1.08E-04	4.15E-05	6.02E-05	4.67E-05
**72h**	7.26E-05	1.32E-05	2.81E-05	1.96E-05
	cgd7_2430			
**2h**	9.87E-06	4.23E-06	1.16E-05	1.77E-05
**6h**	1.37E-05	2.09E-05	8.75E-06	1.70E-05
**12h**	7.23E-06	9.96E-06	1.41E-05	6.90E-06
**24h**	9.50E-06	1.23E-05	9.64E-06	1.61E-05
**36h**	1.42E-05	1.44E-05	1.13E-05	5.35E-06
**48h**	1.88E-06	2.10E-06	2.65E-06	5.82E-06
**72h**	1.98E-06	8.10E-07	3.40E-06	2.29E-06
	cgd7_2440			
**2h**	1.58E-06	5.85E-07	7.99E-07	9.14E-07
**6h**	2.80E-06	4.32E-06	3.48E-06	3.94E-06
**12h**	3.56E-07	2.41E-07	1.81E-06	9.70E-07
**24h**	9.89E-07	1.41E-06	2.39E-06	2.61E-06
**36h**	4.88E-07	1.22E-06	1.34E-06	8.58E-07
**48h**	6.25E-07	4.21E-07	9.17E-07	6.97E-07
**72h**	2.10E-07	1.62E-07	3.82E-07	2.89E-07
	cgd7_2450			
**2h**	1.22E-07	1.18E-08	2.34E-08	6.60E-08
**6h**	1.08E-07	5.74E-08	2.65E-07	1.30E-07
**12h**	4.27E-08	7.82E-09	1.02E-07	2.24E-08
**24h**	7.67E-08	9.22E-08	1.56E-07	2.86E-07
**36h**	1.98E-07	1.30E-07	1.18E-07	1.64E-07
**48h**	1.29E-07	8.93E-08	2.34E-07	3.38E-07
**72h**	1.13E-07	5.52E-08	1.27E-07	1.32E-07
	cgd7_2460			
**2h**	6.79E-09	2.40E-08	1.13E-08	4.93E-08
**6h**	1.64E-07	1.77E-07	1.78E-07	1.33E-07
**12h**	5.18E-09	6.14E-08	2.04E-08	3.37E-08
**24h**	9.80E-08	1.02E-07	1.80E-07	1.12E-07
**36h**	1.45E-07	3.63E-07	2.40E-07	1.45E-07
**48h**	3.32E-07	1.25E-07	2.95E-07	1.60E-07
**72h**	2.16E-07	1.34E-07	3.67E-07	1.60E-07
	cgd7_2470			
**2h**	1.35E-07	1.45E-08	4.43E-08	7.20E-08
**6h**	6.46E-08	1.24E-07	1.57E-07	1.88E-07
**12h**	9.33E-10	2.69E-08	9.60E-08	3.81E-08
**24h**	5.55E-08	5.62E-08	1.39E-07	2.38E-07

**36h**	3.80E-07	8.16E-07	5.47E-07	6.43E-07
**48h**	4.30E-06	3.11E-06	4.32E-06	2.99E-06
**72h**	3.08E-07	2.53E-07	5.79E-07	4.28E-07
	cgd7_2480			
**2h**	2.54E-07	5.20E-08	1.71E-08	5.59E-08
**6h**	2.03E-07	1.22E-07	4.47E-07	3.64E-07
**12h**	6.07E-08	1.33E-07	2.08E-07	1.20E-07
**24h**	2.51E-07	1.57E-07	5.35E-07	4.13E-07
**36h**	6.63E-07	8.43E-07	1.44E-06	4.01E-07
**48h**	4.57E-07	6.38E-07	5.58E-07	8.06E-07
**72h**	1.75E-07	1.44E-07	2.91E-07	1.21E-07
	cgd7_2490			
**2h**	3.43E-06	4.38E-08	0.00E+00	8.25E-08
**6h**	1.50E-07	9.29E-08	2.61E-06	3.01E-06
**12h**	1.54E-07	0.00E+00	2.31E-07	9.86E-08
**24h**	7.12E-07	9.24E-07	2.85E-06	1.26E-06
**36h**	7.57E-06	7.44E-06	4.22E-06	1.49E-06
**48h**	6.95E-05	6.71E-05	4.39E-05	2.04E-05
**72h**	1.51E-05	6.72E-06	1.25E-05	5.91E-06
	cgd7_2500			
**2h**	2.87E-06	1.30E-07	2.93E-07	4.44E-07
**6h**	2.01E-07	1.12E-06	6.15E-07	1.97E-06
**12h**	9.33E-08	1.24E-07	3.45E-07	1.85E-07
**24h**	1.08E-06	8.19E-07	1.05E-06	9.82E-07
**36h**	5.02E-06	3.33E-06	1.68E-06	1.29E-06
**48h**	6.77E-06	3.77E-06	5.81E-06	1.81E-06
**72h**	3.79E-06	1.57E-06	3.49E-06	1.24E-06
	cgd7_2510			
**2h**	3.13E-08	1.98E-07	0.00E+00	3.54E-08
**6h**	2.07E-07	3.90E-07	6.64E-07	1.08E-06
**12h**	4.54E-08	4.89E-08	1.66E-07	4.84E-08
**24h**	1.79E-07	1.40E-07	8.20E-07	1.71E-06
**36h**	3.01E-06	9.19E-07	7.75E-07	4.32E-07
**48h**	7.47E-07	6.09E-07	1.09E-06	3.84E-07
**72h**	3.17E-07	2.54E-07	4.39E-07	4.83E-07
	cgd8_3070			
**2h**	4.55E-06	3.27E-06	6.94E-07	4.38E-05
**6h**	1.25E-05	1.60E-05	4.63E-05	0.00E+00
**12h**	1.09E-05	4.73E-05	2.89E-07	2.80E-04
**24h**	9.76E-07	1.87E-05	3.69E-05	8.82E-05
**36h**	1.26E-04	1.59E-05	5.73E-05	4.85E-04
**48h**	2.76E-05	7.36E-06	1.97E-05	1.44E-04
**72h**	3.94E-06	3.92E-06	5.52E-06	5.69E-05
	cgd8_3080			
**2h**	2.42E-04	7.65E-05	1.79E-03	5.25E-04
**6h**	6.27E-05	6.63E-05	2.75E-04	1.09E-03
**12h**	4.19E-05	7.11E-05	6.76E-08	7.74E-04
**24h**	9.84E-06	2.20E-04	1.51E-04	5.02E-04
**36h**	8.15E-05	4.10E-05	4.39E-05	5.17E-04
**48h**	6.99E-05	1.80E-05	5.05E-05	2.01E-04
**72h**	1.33E-05	9.57E-06	1.23E-05	1.57E-04
	cgd8_3090			
**2h**	8.88E-07	4.29E-07	1.85E-06	2.44E-06

**6h**	2.31E-07	5.45E-07	6.81E-07	1.40E-06
**12h**	2.32E-07	7.18E-07	5.83E-10	7.94E-07
**24h**	3.19E-08	6.52E-07	1.86E-06	4.72E-07
**36h**	7.90E-07	5.40E-07	4.13E-07	2.83E-07
**48h**	4.63E-07	2.32E-07	2.95E-07	6.39E-07
**72h**	6.99E-08	1.60E-07	2.73E-07	2.38E-07
	cgd8_3100			
**2h**	2.46E-06	7.29E-07	2.24E-06	0.00E+00
**6h**	4.13E-06	1.16E-05	1.08E-05	1.72E-04
**12h**	4.99E-07	7.10E-08	3.39E-09	8.14E-05
**24h**	2.25E-07	4.93E-06	1.57E-05	1.71E-05
**36h**	3.39E-06	4.85E-06	1.45E-06	2.15E-06
**48h**	2.57E-06	4.21E-07	1.24E-06	4.85E-06
**72h**	1.37E-07	1.81E-07	3.87E-07	0.00E+00
	cgd8_3110			
**2h**	1.91E-05	8.99E-06	2.39E-05	2.23E-04
**6h**	2.12E-06	4.35E-06	4.14E-06	2.00E-05
**12h**	9.46E-06	5.76E-06	2.74E-08	1.39E-05
**24h**	8.84E-07	1.80E-05	1.16E-05	1.60E-05
**36h**	1.47E-05	7.34E-06	8.49E-06	4.33E-06
**48h**	3.15E-06	7.52E-07	1.16E-06	8.97E-06
**72h**	1.14E-06	1.51E-06	3.73E-06	2.45E-06
	cgd8_3120			
**2h**	3.65E-08	5.35E-09	1.95E-08	1.57E-06
**6h**	9.10E-09	0.00E+00	0.00E+00	6.41E-07
**12h**	3.32E-05	8.95E-06	4.59E-09	1.64E-05
**24h**	3.53E-08	8.46E-07	1.91E-07	3.14E-06
**36h**	9.32E-07	3.48E-07	1.90E-06	4.56E-06
**48h**	1.53E-07	1.16E-07	9.93E-08	8.25E-07
**72h**	9.18E-08	8.54E-08	1.36E-07	9.68E-07
	cgd8_3130			
**2h**	0.00E+00	0.00E+00	0.00E+00	0.00E+00
**6h**	0.00E+00	1.18E-09	0.00E+00	0.00E+00
**12h**	9.74E-06	6.11E-06	3.14E-09	6.34E-06
**24h**	2.15E-08	8.62E-07	1.82E-07	4.35E-07
**36h**	2.41E-06	8.55E-07	3.81E-06	6.48E-06
**48h**	6.44E-07	1.00E-07	3.05E-07	1.96E-06
**72h**	2.93E-08	5.63E-08	7.04E-08	8.69E-07
	cgd8_3140			
**2h**	2.28E-06	1.34E-06	1.77E-06	3.57E-05
**6h**	3.42E-06	1.46E-06	3.38E-06	2.02E-04
**12h**	4.45E-07	2.99E-06	3.01E-09	4.26E-05
**24h**	1.28E-07	2.44E-06	5.48E-06	1.59E-05
**36h**	2.51E-06	2.44E-06	1.79E-06	2.41E-05
**48h**	1.97E-05	1.04E-05	8.67E-06	9.01E-05
**72h**	1.27E-06	1.80E-06	3.62E-06	3.04E-05
	cgd8_3150			
**2h**	3.31E-03	1.81E-03	1.58E-03	9.25E-05
**6h**	3.28E-03	2.48E-03	2.73E-03	2.03E-04
**12h**	2.54E-03	3.18E-03	4.95E-06	2.72E-04
**24h**	1.19E-04	2.77E-03	5.04E-03	8.21E-05
**36h**	1.21E-03	1.06E-03	8.64E-04	5.30E-05
**48h**	1.21E-03	6.43E-04	1.38E-03	4.66E-05

**72h**	1.25E-04	6.97E-04	6.09E-04	2.76E-05
	cgd8_3160			
**2h**	4.46E-07	5.30E-07	4.03E-06	3.43E-06
**6h**	9.21E-06	2.97E-06	2.69E-06	3.20E-05
**12h**	5.00E-07	9.00E-07	1.32E-08	5.70E-05
**24h**	1.44E-06	2.52E-05	2.44E-05	1.58E-05
**36h**	9.83E-06	9.32E-06	5.14E-06	8.79E-06
**48h**	3.22E-05	9.17E-06	1.03E-05	1.29E-05
**72h**	1.08E-05	1.85E-05	9.07E-06	3.86E-05
	cgd8_3170			
**2h**	4.19E-05	3.14E-05	3.92E-05	3.62E-05
**6h**	9.92E-05	5.75E-05	7.80E-05	1.03E-04
**12h**	5.18E-06	5.88E-06	9.66E-09	2.00E-04
**24h**	9.93E-07	3.24E-05	7.41E-05	4.81E-05
**36h**	4.96E-05	4.60E-05	2.13E-05	4.93E-05
**48h**	8.73E-05	8.98E-06	1.78E-05	6.47E-05
**72h**	1.38E-05	1.08E-05	7.28E-06	4.67E-05
	cgd8_3180			
**2h**	6.49E-04	9.16E-05	2.06E-04	8.80E-07
**6h**	4.22E-03	1.99E-03	5.59E-03	9.94E-05
**12h**	3.82E-04	5.29E-04	4.22E-07	7.67E-06
**24h**	3.19E-05	7.62E-04	3.41E-03	4.99E-05
**36h**	1.99E-03	1.43E-03	2.04E-03	1.08E-05
**48h**	4.69E-04	2.20E-04	8.90E-04	2.21E-05
**72h**	2.36E-05	3.91E-04	5.06E-04	8.79E-06
	cgd8_3190			
**2h**	6.46E-07	7.61E-07	3.09E-07	5.54E-07
**6h**	6.36E-06	5.54E-06	9.97E-06	9.73E-06
**12h**	1.38E-07	3.46E-07	2.37E-06	5.72E-07
**24h**	1.09E-06	1.72E-06	7.27E-06	2.22E-06
**36h**	1.12E-05	1.21E-06	2.54E-06	5.42E-06
**48h**	1.61E-06	1.92E-06	2.22E-06	1.88E-05
**72h**	5.13E-07	3.43E-07	1.88E-07	1.49E-08
	cgd8_3200			
**2h**	1.72E-06	0.00E+00	0.00E+00	5.09E-06
**6h**	1.05E-03	1.01E-03	1.01E-03	9.03E-05
**12h**	3.56E-05	0.00E+00	5.29E-05	8.15E-07
**24h**	2.57E-04	1.26E-04	5.22E-04	1.17E-04
**36h**	2.23E-04	7.67E-05	8.95E-05	2.77E-05
**48h**	1.31E-04	7.08E-05	2.93E-04	2.04E-05
**72h**	2.69E-05	6.15E-05	2.01E-05	2.50E-06
	cgd8_3210			
**2h**	2.73E-08	7.34E-08	0.00E+00	1.09E-05
**6h**	8.69E-07	4.80E-07	7.59E-07	1.63E-06
**12h**	4.01E-09	1.51E-08	6.93E-09	3.13E-08
**24h**	1.04E-07	1.03E-07	2.57E-07	2.50E-07
**36h**	1.17E-07	1.77E-07	1.12E-07	1.15E-07
**48h**	1.39E-07	6.49E-08	9.77E-08	9.48E-08
**72h**	3.39E-09	6.83E-08	4.37E-08	1.95E-09
	cgd8_3220			
**2h**	6.22E-05	8.39E-05	0.00E+00	0.00E+00
**6h**	4.80E-05	1.43E-03	0.00E+00	0.00E+00
**12h**	3.37E-05	0.00E+00	0.00E+00	0.00E+00

**24h**	3.27E-05	1.19E-04	0.00E+00	0.00E+00
**36h**	2.48E-05	8.22E-04	0.00E+00	0.00E+00
**48h**	8.24E-06	5.08E-04	0.00E+00	0.00E+00
**72h**	4.52E-06	4.18E-05	0.00E+00	0.00E+00
	cgd8_3230			
**2h**	7.10E-05	6.30E-05	2.90E-05	1.38E-04
**6h**	8.98E-06	1.12E-05	1.35E-05	1.56E-05
**12h**	6.02E-05	7.44E-05	6.34E-05	2.18E-05
**24h**	6.29E-05	7.54E-05	5.02E-05	1.44E-05
**36h**	2.55E-05	2.83E-05	2.30E-05	2.18E-05
**48h**	4.96E-06	2.74E-06	1.03E-05	5.79E-06
**72h**	3.66E-06	6.85E-06	4.10E-06	4.63E-07
	cgd8_3240			
**2h**	8.54E-04	4.76E-04	2.59E-04	4.51E-04
**6h**	8.37E-06	2.23E-05	1.41E-05	7.89E-06
**12h**	4.12E-03	6.38E-03	1.48E-03	9.14E-04
**24h**	1.76E-03	9.64E-04	6.35E-04	4.61E-04
**36h**	6.16E-04	6.47E-04	2.02E-03	1.53E-03
**48h**	6.63E-04	8.14E-04	5.93E-04	1.49E-04
**72h**	1.54E-04	8.19E-04	3.14E-04	1.37E-05
	cgd8_3250			
**2h**	8.62E-07	2.22E-07	1.40E-07	4.59E-07
**6h**	9.95E-07	1.42E-06	6.29E-07	7.63E-07
**12h**	1.99E-07	5.06E-07	4.38E-07	2.19E-07
**24h**	3.87E-07	4.59E-07	1.71E-06	7.48E-07
**36h**	1.92E-06	1.93E-06	1.86E-06	1.49E-06
**48h**	2.15E-06	9.28E-07	2.22E-06	5.44E-07
**72h**	5.27E-07	1.44E-06	6.59E-07	3.37E-07
	cgd8_3270			
**2h**	6.55E-07	1.22E-06	2.12E-06	1.39E-05
**6h**	1.85E-04	4.94E-05	1.75E-04	2.87E-05
**12h**	1.63E-04	4.25E-04	1.22E-04	1.87E-03
**24h**	2.14E-05	2.06E-05	9.63E-05	2.66E-05
**36h**	1.94E-05	3.01E-05	4.94E-05	4.47E-05
**48h**	4.63E-05	2.14E-05	1.42E-04	2.73E-05
**72h**	4.83E-05	4.93E-05	4.75E-05	2.59E-05
	cgd8_3280			
**2h**	9.98E-07	1.39E-06	3.63E-07	1.18E-06
**6h**	1.04E-07	1.37E-07	4.51E-07	1.52E-07
**12h**	1.65E-06	3.83E-06	3.03E-06	7.69E-07
**24h**	1.18E-06	1.21E-06	8.27E-07	3.23E-07
**36h**	1.34E-06	1.55E-06	1.83E-06	1.15E-06
**48h**	5.78E-07	4.05E-07	7.01E-07	3.71E-07
**72h**	2.58E-07	3.11E-07	1.27E-07	4.60E-08
	cgd8_3290			
**2h**	7.53E-05	5.66E-05	2.08E-04	7.66E-05
**6h**	3.24E-04	2.27E-04	3.86E-04	1.28E-04
**12h**	3.94E-04	4.91E-04	3.19E-04	1.32E-04
**24h**	1.66E-04	2.48E-04	2.69E-04	1.54E-04
**36h**	7.11E-05	1.07E-04	1.36E-04	6.59E-05
**48h**	2.87E-04	1.32E-04	2.40E-04	3.39E-04
**72h**	5.97E-05	8.74E-05	1.95E-05	1.60E-06
	cgd8_3300			

**2h**	2.02E-07	3.49E-07	5.40E-08	6.46E-07
**6h**	9.21E-07	6.45E-07	1.02E-06	1.50E-06
**12h**	9.60E-06	1.65E-04	4.77E-06	3.10E-06
**24h**	2.66E-06	3.77E-06	2.21E-06	1.93E-06
**36h**	2.91E-06	4.47E-06	4.15E-06	8.72E-06
**48h**	2.05E-06	2.04E-06	2.52E-06	1.61E-05
**72h**	2.21E-07	4.98E-07	2.54E-07	4.47E-08
	cgd8_3310			
**2h**	8.97E-08	2.36E-08	6.05E-08	2.14E-07
**6h**	1.51E-06	3.58E-07	1.98E-06	3.22E-06
**12h**	3.48E-07	3.08E-07	1.68E-07	4.86E-07
**24h**	2.69E-07	2.74E-07	3.67E-07	1.84E-06
**36h**	4.93E-07	7.17E-07	3.43E-07	2.77E-06
**48h**	1.59E-07	1.74E-07	3.32E-07	5.46E-06
**72h**	3.24E-07	2.43E-07	7.63E-08	8.93E-09
	cgd8_3320			
**2h**	1.22E-03	1.93E-04	1.71E-04	2.20E-04
**6h**	1.44E-03	2.06E-04	9.44E-04	2.06E-03
**12h**	1.94E-05	1.33E-06	2.97E-04	1.23E-04
**24h**	3.50E-04	2.44E-05	3.25E-04	1.82E-03
**36h**	2.41E-04	1.21E-05	1.32E-04	5.96E-05
**48h**	1.16E-04	7.41E-06	3.10E-04	3.76E-04
**72h**	3.29E-05	6.35E-06	3.82E-05	2.32E-05
	cgd8_3330			
**2h**	6.49E-06	6.80E-06	6.06E-06	5.40E-05
**6h**	1.32E-05	3.13E-05	2.16E-05	3.50E-05
**12h**	3.04E-06	8.49E-07	7.16E-06	1.27E-04
**24h**	2.97E-05	4.66E-06	1.39E-05	3.16E-05
**36h**	4.66E-06	1.34E-06	3.25E-06	2.77E-05
**48h**	2.81E-06	1.12E-06	4.02E-06	2.05E-05
**72h**	1.40E-06	8.58E-07	1.61E-06	6.40E-06
	cgd8_3360			
**2h**	1.85E-07	3.02E-07	1.13E-07	0.00E+00
**6h**	1.04E-06	2.10E-06	1.46E-06	0.00E+00
**12h**	6.04E-08	1.78E-08	1.50E-07	4.22E-07
**24h**	3.75E-07	1.71E-07	5.03E-07	7.00E-07
**36h**	2.70E-07	1.85E-07	3.85E-07	3.02E-07
**48h**	3.18E-07	2.95E-07	6.13E-07	1.00E-06
**72h**	7.78E-08	1.13E-07	1.10E-07	1.98E-07
	cgd8_3370			
**2h**	9.94E-03	3.57E-04	1.20E-03	4.04E-03
**6h**	3.85E-02	2.69E-03	2.26E-02	3.27E-02
**12h**	3.21E-03	1.84E-03	9.08E-03	4.24E-03
**24h**	7.51E-03	4.49E-04	7.88E-03	5.45E-03
**36h**	6.33E-03	5.09E-04	3.43E-03	1.70E-03
**48h**	2.45E-03	9.55E-05	2.96E-03	1.93E-03
**72h**	7.05E-04	1.32E-04	1.10E-03	3.65E-04
	cgd8_3380			
**2h**	2.16E-03	1.62E-04	2.24E-04	3.67E-04
**6h**	2.83E-04	6.33E-05	9.36E-04	4.59E-04
**12h**	8.96E-04	9.14E-06	1.03E-03	2.97E-04
**24h**	1.08E-03	1.15E-04	5.75E-04	5.42E-04
**36h**	4.87E-04	6.41E-05	2.98E-04	2.08E-04

**48h**	3.81E-04	1.35E-05	3.39E-04	1.96E-04
**72h**	6.44E-05	1.41E-05	1.10E-04	4.99E-05
	cgd8_3390			
**2h**	8.19E-03	4.82E-04	4.05E-03	2.12E-03
**6h**	6.94E-04	7.20E-05	5.13E-04	4.73E-03
**12h**	6.25E-03	6.24E-05	4.81E-03	9.99E-04
**24h**	6.25E-03	1.75E-04	1.70E-03	9.77E-04
**36h**	3.20E-03	1.21E-04	2.42E-03	7.86E-04
**48h**	8.15E-05	2.63E-05	2.27E-03	1.11E-03
**72h**	7.79E-04	1.26E-04	1.58E-03	9.38E-04
	cgd8_3410			
**2h**	1.80E-05	8.74E-06	5.41E-06	4.73E-05
**6h**	1.32E-06	1.12E-06	1.84E-06	8.97E-06
**12h**	6.80E-06	1.15E-06	9.03E-06	5.47E-06
**24h**	1.65E-05	3.23E-06	2.64E-06	2.53E-06
**36h**	1.23E-05	3.20E-06	5.37E-06	3.12E-06
**48h**	1.08E-05	7.91E-07	5.98E-06	7.38E-06
**72h**	2.12E-06	1.42E-06	4.55E-06	2.66E-06
	cgd8_3420			
**2h**	2.43E-03	2.68E-04	1.95E-04	5.12E-04
**6h**	9.61E-03	3.24E-04	2.06E-02	5.42E-03
**12h**	1.87E-03	2.19E-05	1.89E-03	6.77E-04
**24h**	4.17E-03	5.99E-05	2.16E-03	1.04E-03
**36h**	2.41E-03	1.57E-04	2.61E-03	9.46E-04
**48h**	2.05E-04	2.59E-05	3.16E-03	1.15E-03
**72h**	1.34E-03	5.69E-05	5.28E-04	3.25E-04
	cgd8_3430			
**2h**	1.17E-05	1.55E-06	3.28E-06	2.23E-06
**6h**	1.10E-04	3.51E-05	4.44E-04	1.88E-04
**12h**	6.10E-05	6.01E-06	2.11E-05	8.51E-06
**24h**	1.56E-04	2.34E-06	6.45E-05	4.11E-05
**36h**	1.77E-04	1.98E-05	1.41E-04	6.02E-05
**48h**	1.12E-04	5.83E-06	8.63E-05	3.67E-05
**72h**	2.53E-05	3.36E-06	2.71E-05	5.42E-06
	cgd8_3440			
**2h**	1.25E-06	8.00E-06	1.36E-06	1.27E-06
**6h**	2.17E-06	3.73E-06	2.93E-06	7.07E-06
**12h**	2.18E-07	9.81E-08	1.42E-06	3.72E-07
**24h**	2.35E-06	8.91E-07	2.08E-06	1.34E-06
**36h**	8.26E-07	6.06E-07	6.25E-07	8.48E-07
**48h**	7.32E-07	7.59E-07	7.62E-07	5.78E-07
**72h**	9.03E-08	2.83E-07	3.67E-07	1.19E-07
	cgd8_3450			
**2h**	1.40E-04	7.89E-05	5.35E-05	5.13E-05
**6h**	4.05E-04	2.14E-04	4.38E-04	4.35E-04
**12h**	7.36E-05	9.44E-06	1.27E-04	6.49E-05
**24h**	1.64E-04	3.49E-05	1.87E-04	9.30E-05
**36h**	1.06E-04	2.05E-05	9.14E-05	4.61E-05
**48h**	5.39E-05	1.81E-05	8.15E-05	2.27E-05
**72h**	1.12E-05	3.24E-06	1.52E-05	1.00E-05
	cgd8_3460			
**2h**	1.29E-05	1.06E-05	1.78E-06	1.94E-06
**6h**	2.03E-06	3.77E-06	7.74E-06	7.02E-06

**12h**	1.35E-06	2.77E-07	6.37E-06	9.66E-07
**24h**	4.61E-06	8.08E-07	5.42E-06	3.24E-06
**36h**	2.61E-06	2.80E-06	4.44E-06	1.54E-06
**48h**	1.66E-06	4.57E-07	1.56E-06	2.95E-06
**72h**	1.87E-07	5.07E-07	1.16E-06	6.05E-07
	cgd8_3470			
**2h**	9.91E-07	5.96E-07	2.23E-05	8.21E-06
**6h**	1.40E-06	4.57E-06	9.85E-05	2.67E-05
**12h**	1.23E-05	5.17E-06	9.76E-05	3.46E-05
**24h**	3.58E-06	3.67E-06	1.79E-05	1.06E-05
**36h**	1.07E-06	1.75E-06	2.43E-04	7.03E-06
**48h**	9.29E-07	1.76E-06	1.19E-04	2.17E-05
**72h**	4.00E-07	6.99E-07	4.15E-05	2.52E-06
	cgd8_3480			
**2h**	5.01E-04	3.44E-04	6.06E-03	1.28E-02
**6h**	7.07E-04	1.15E-03	0.00E+00	2.70E-02
**12h**	3.60E-04	4.89E-04	1.64E-03	5.47E-03
**24h**	1.04E-03	4.38E-04	1.90E-03	1.21E-02
**36h**	3.58E-04	1.19E-04	7.58E-04	6.38E-03
**48h**	1.15E-04	1.81E-04	1.09E-04	5.03E-03
**72h**	3.79E-05	3.50E-05	1.19E-04	4.29E-04
	cgd8_3500			
**2h**	4.04E-07	0.00E+00	5.25E-05	3.32E-05
**6h**	0.00E+00	7.93E-09	4.27E-07	8.57E-06
**12h**	3.66E-06	2.16E-06	1.39E-05	2.86E-04
**24h**	6.28E-06	4.44E-06	8.20E-06	2.13E-04
**36h**	1.29E-05	5.21E-06	3.81E-05	2.44E-04
**48h**	9.41E-06	7.04E-06	1.34E-05	1.42E-03
**72h**	1.04E-05	1.09E-05	2.27E-05	3.21E-04
	cgd8_3510			
**2h**	1.18E-07	7.68E-08	9.94E-05	3.21E-06
**6h**	1.52E-08	4.64E-09	1.45E-07	7.38E-06
**12h**	3.06E-07	2.88E-08	5.34E-07	1.27E-06
**24h**	6.15E-07	5.28E-07	4.81E-07	1.42E-04
**36h**	1.49E-06	3.58E-07	4.20E-06	3.94E-05
**48h**	9.14E-07	4.17E-07	7.97E-07	8.21E-05
**72h**	5.41E-07	7.77E-07	4.24E-06	1.40E-05
	cgd8_3520			
**2h**	1.07E-03	8.88E-04	4.63E-03	1.18E-02
**6h**	5.83E-04	4.00E-04	6.81E-04	5.22E-03
**12h**	3.84E-04	1.51E-03	4.96E-03	5.99E-03
**24h**	1.90E-03	8.51E-04	7.64E-04	9.42E-03
**36h**	8.65E-04	3.62E-04	7.93E-04	2.71E-03
**48h**	1.66E-04	2.68E-04	2.02E-04	2.45E-03
**72h**	4.35E-05	8.48E-05	3.94E-05	9.53E-04
	cgd8_3530			
**2h**	3.31E-06	1.19E-06	7.37E-06	1.61E-05
**6h**	5.80E-07	3.55E-07	2.13E-06	1.86E-05
**12h**	6.70E-07	4.45E-06	1.12E-06	8.59E-06
**24h**	1.60E-06	8.44E-07	1.67E-06	1.64E-05
**36h**	8.75E-07	9.27E-07	1.81E-06	1.10E-05
**48h**	3.34E-07	4.47E-07	3.97E-07	6.19E-06
**72h**	1.99E-07	2.79E-07	6.45E-07	2.54E-06

	cgd8_3560			
**2h**	9.76E-07	3.38E-07	3.33E-06	1.38E-05
**6h**	2.21E-07	2.42E-07	5.07E-07	7.26E-06
**12h**	8.76E-06	2.31E-06	4.57E-06	4.05E-05
**24h**	2.24E-06	1.03E-06	4.31E-07	1.55E-05
**36h**	8.32E-07	6.40E-07	2.49E-06	6.67E-06
**48h**	4.06E-07	2.05E-07	1.38E-07	6.31E-06
**72h**	1.11E-07	1.09E-07	5.12E-08	1.31E-06
	cgd8_3580			
**2h**	5.63E-05	8.49E-05	3.31E-04	1.02E-03
**6h**	2.56E-04	2.54E-07	3.03E-05	3.99E-04
**12h**	5.34E-03	1.30E-03	1.24E-04	6.23E-04
**24h**	1.09E-03	6.63E-05	4.53E-05	5.21E-04
**36h**	4.48E-04	2.58E-04	1.98E-04	1.16E-04
**48h**	2.51E-04	1.79E-04	3.95E-05	6.48E-04
**72h**	1.85E-04	1.51E-05	4.88E-05	1.74E-04
	cgd8_3590			
**2h**	2.26E-03	2.45E-04	6.79E-04	2.23E-03
**6h**	1.01E-03	2.14E-03	4.68E-04	8.15E-04
**12h**	1.57E-02	7.92E-03	3.71E-03	5.12E-03
**24h**	2.64E-03	1.73E-03	3.44E-04	1.31E-03
**36h**	1.72E-03	1.47E-03	1.39E-03	1.31E-03
**48h**	4.25E-04	4.43E-04	1.49E-04	3.89E-04
**72h**	2.22E-04	9.29E-05	1.02E-04	3.35E-04
	cgd8_3600			
**2h**	1.54E-06	4.08E-08	3.48E-05	1.35E-05
**6h**	1.23E-07	2.92E-07	5.31E-06	3.48E-06
**12h**	4.45E-05	1.56E-05	3.27E-05	1.40E-03
**24h**	5.43E-06	4.28E-06	1.80E-05	5.87E-05
**36h**	1.20E-05	1.23E-05	3.87E-05	3.64E-04
**48h**	7.90E-06	7.83E-06	7.66E-06	2.06E-04
**72h**	5.83E-06	9.67E-07	2.23E-06	1.13E-04
	cgd8_3610			
**2h**	0.00E+00	0.00E+00	0.00E+00	0.00E+00
**6h**	0.00E+00	1.78E-05	0.00E+00	0.00E+00
**12h**	4.02E-03	1.03E-03	3.76E-01	4.83E-04
**24h**	3.37E-04	1.46E-04	7.79E-03	1.83E-04
**36h**	1.14E-03	4.90E-04	8.13E-02	4.33E-04
**48h**	2.16E-04	8.13E-04	6.84E-03	1.69E-04
**72h**	2.82E-04	2.75E-03	5.86E-03	1.15E-04
	cgd8_3620			
**2h**	0.00E+00	0.00E+00	2.29E-08	2.24E-07
**6h**	8.57E-07	1.05E-06	1.27E-05	3.01E-06
**12h**	8.20E-07	6.20E-07	3.81E+00	2.10E-06
**24h**	5.99E-07	6.57E-07	5.54E-02	1.85E-06
**36h**	1.54E-06	1.23E-06	2.53E-06	9.17E-06
**48h**	8.19E-07	1.00E-06	1.80E-06	7.45E-06
**72h**	6.22E-07	9.92E-07	2.64E-07	4.79E-06
	cgd8_3780			
**2h**	1.49E-08	6.09E-07	0.00E+00	0.00E+00
**6h**	2.58E-07	3.01E-06	8.66E-07	2.31E-07
**12h**	3.33E-07	1.47E-06	2.21E-06	0.00E+00
**24h**	5.23E-07	7.56E-07	1.96E-06	1.38E-05

**36h**	1.49E-06	1.10E-06	1.78E-06	1.35E-04
**48h**	3.33E-06	9.64E-07	1.92E-06	6.59E-06
**72h**	4.00E-07	8.11E-07	1.31E-06	3.82E-06
	cgd8_3790			
**2h**	0.00E+00	4.29E-06	0.00E+00	0.00E+00
**6h**	4.26E-06	4.10E-05	1.58E-04	3.84E-05
**12h**	4.49E-06	1.07E-05	8.12E-05	8.60E-05
**24h**	2.76E-05	4.09E-04	3.24E-04	4.68E-04
**36h**	6.96E-05	1.39E-04	1.49E-04	2.86E-05
**48h**	2.40E-04	5.78E-05	1.77E-04	4.65E-05
**72h**	8.54E-05	5.10E-05	6.82E-05	3.57E-04
	cgd8_3800			
**2h**	2.33E-05	2.56E-05	2.07E-05	1.27E-05
**6h**	2.05E-06	3.95E-06	9.59E-07	1.66E-06
**12h**	3.46E-06	8.24E-06	9.38E-06	2.63E-06
**24h**	1.00E-05	7.20E-06	1.01E-05	6.81E-06
**36h**	2.11E-06	2.60E-06	3.19E-06	2.15E-06
**48h**	5.08E-06	1.07E-06	4.68E-06	1.08E-05
**72h**	1.46E-06	1.18E-06	4.76E-06	2.01E-05
	cgd8_3820			
**2h**	1.72E-08	1.25E-08	4.11E-08	0.00E+00
**6h**	9.88E-08	3.74E-07	3.27E-07	9.74E-08
**12h**	1.52E-07	4.88E-07	1.54E-07	5.45E-08
**24h**	8.43E-08	1.98E-07	4.46E-07	1.29E-07
**36h**	3.39E-07	1.91E-07	2.12E-07	1.16E-07
**48h**	5.12E-07	2.53E-07	3.79E-07	3.02E-07
**72h**	1.81E-07	1.10E-07	2.45E-07	4.01E-07
	cgd8_3830			
**2h**	0.00E+00	0.00E+00	0.00E+00	5.79E-09
**6h**	1.42E-09	1.82E-08	1.59E-08	8.28E-09
**12h**	4.34E-06	1.35E-05	2.35E-06	5.27E-07
**24h**	3.29E-07	2.21E-07	2.06E-07	1.07E-07
**36h**	3.22E-07	1.33E-07	9.33E-07	1.73E-07
**48h**	8.85E-08	5.56E-08	5.52E-08	1.34E-07
**72h**	6.77E-08	6.99E-08	2.17E-07	1.03E-07
	cgd8_3840			
**2h**	2.57E-07	9.86E-07	6.41E-07	2.33E-07
**6h**	3.24E-07	4.27E-07	5.58E-07	1.85E-07
**12h**	1.41E-07	7.22E-08	7.36E-07	3.27E-07
**24h**	1.40E-07	2.78E-07	3.04E-07	3.20E-07
**36h**	2.33E-07	3.07E-07	6.24E-07	1.66E-07
**48h**	1.62E-07	1.35E-07	3.16E-07	2.96E-07
**72h**	1.88E-07	1.88E-07	3.25E-07	2.31E-07
	cgd8_3850			
**2h**	9.78E-05	6.12E-05	1.41E-07	3.82E-05
**6h**	5.01E-05	3.40E-04	1.61E-04	6.25E-05
**12h**	3.03E-05	1.02E-04	2.48E-05	9.72E-06
**24h**	2.81E-05	5.51E-05	1.34E-04	2.76E-05
**36h**	8.74E-05	8.52E-05	5.60E-05	2.09E-05
**48h**	1.14E-04	5.20E-05	1.07E-04	2.88E-05
**72h**	1.27E-05	1.21E-05	2.90E-05	1.56E-05
	cgd8_3860			
**2h**	4.29E-09	0.00E+00	0.00E+00	3.21E-09

**6h**	2.68E-08	1.51E-08	1.93E-08	9.10E-09
**12h**	1.83E-08	3.24E-08	3.94E-08	8.52E-09
**24h**	2.81E-08	8.04E-08	3.24E-07	5.89E-08
**36h**	2.56E-07	1.70E-07	2.02E-07	6.57E-08
**48h**	2.43E-07	1.11E-07	3.08E-07	1.54E-07
**72h**	6.67E-08	9.98E-08	1.23E-07	7.74E-08
	cgd8_3870			
**2h**	9.37E-05	9.72E-05	4.44E-06	2.20E-05
**6h**	1.90E-04	3.64E-04	3.53E-04	3.53E-04
**12h**	2.20E-05	1.26E-05	2.03E-04	1.78E-05
**24h**	6.25E-05	1.58E-04	3.57E-04	1.12E-04
**36h**	3.81E-05	6.80E-05	7.64E-05	1.55E-05
**48h**	9.40E-05	2.12E-05	6.79E-05	2.46E-05
**72h**	3.76E-05	1.57E-05	3.12E-05	5.80E-06
	cgd8_3880			
**2h**	6.28E-04	5.37E-04	1.01E-04	5.14E-05
**6h**	3.60E-04	2.40E-03	2.21E-03	5.68E-04
**12h**	1.75E-05	1.13E-05	4.60E-04	3.07E-05
**24h**	1.52E-04	3.38E-04	8.93E-04	2.52E-04
**36h**	6.67E-05	1.19E-04	1.30E-04	2.06E-05
**48h**	3.91E-04	1.16E-04	2.68E-04	2.51E-04
**72h**	2.63E-05	8.66E-05	4.39E-05	4.72E-06
	cgd8_3890			
**2h**	2.73E-04	2.95E-04	6.75E-05	8.78E-05
**6h**	3.83E-05	1.32E-04	1.33E-04	2.45E-04
**12h**	8.70E-05	2.23E-04	3.59E-05	2.81E-05
**24h**	2.32E-05	8.84E-05	9.52E-05	4.05E-05
**36h**	5.53E-05	3.57E-05	7.74E-05	1.58E-05
**48h**	5.01E-05	2.28E-05	7.84E-05	3.46E-05
**72h**	7.78E-06	1.14E-05	1.03E-05	4.45E-06
	cgd8_3900			
**2h**	1.29E-05	2.83E-05	6.45E-06	9.74E-06
**6h**	1.05E-04	2.56E-04	3.69E-04	1.63E-04
**12h**	5.07E-06	1.02E-05	1.80E-05	3.82E-06
**24h**	2.13E-05	3.50E-05	9.40E-05	5.58E-05
**36h**	3.44E-05	6.70E-05	3.90E-05	1.03E-05
**48h**	1.29E-05	8.30E-06	1.00E-05	8.74E-06
**72h**	7.37E-06	5.93E-06	1.45E-05	5.71E-06
	cgd8_3910			
**2h**	4.24E-06	5.86E-06	1.36E-07	7.28E-06
**6h**	3.04E-06	8.43E-04	4.37E-06	2.35E-05
**12h**	9.78E-08	0.00E+00	1.73E-06	9.48E-06
**24h**	1.94E-06	4.08E-06	1.59E-05	2.45E-05
**36h**	1.39E-06	2.83E-06	1.30E-05	2.50E-05
**48h**	1.26E-05	2.56E-05	8.20E-05	6.51E-05
**72h**	1.14E-05	1.86E-05	1.72E-04	5.03E-05
	cgd8_3920			
**2h**	3.51E-06	8.81E-05	9.44E-07	4.38E-06
**6h**	2.79E-06	8.10E-05	3.01E-05	2.15E-05
**12h**	1.57E-07	1.51E-06	5.18E-06	2.47E-05
**24h**	1.83E-06	2.56E-06	3.21E-05	2.11E-05
**36h**	2.46E-06	8.15E-06	1.84E-05	7.14E-06
**48h**	1.14E-05	2.77E-05	5.58E-05	2.80E-04

**72h**	1.35E-05	3.11E-05	2.82E-04	8.64E-05
	cgd8_3930			
**2h**	0.00E+00	0.00E+00	0.00E+00	0.00E+00
**6h**	0.00E+00	1.33E-08	3.93E-09	6.26E-09
**12h**	2.99E-08	1.57E-06	4.55E-08	2.59E-07
**24h**	8.19E-08	5.61E-08	2.19E-07	7.34E-08
**36h**	1.33E-07	5.36E-07	2.76E-07	8.30E-07
**48h**	1.50E-07	1.39E-07	2.66E-07	1.13E-06
**72h**	2.35E-07	3.05E-07	6.16E-07	1.19E-06
	cgd8_3950			
**2h**	1.66E-06	3.69E-07	0.00E+00	9.03E-07
**6h**	0.00E+00	3.28E-07	3.28E-06	5.15E-06
**12h**	9.98E-08	1.36E-05	6.49E-06	1.25E-05
**24h**	4.47E-07	1.35E-06	1.56E-04	4.18E-05
**36h**	4.03E-06	7.49E-05	2.29E-05	5.00E-05
**48h**	1.86E-06	1.25E-05	1.80E-05	6.11E-05
**72h**	2.94E-06	3.99E-06	3.13E-06	7.89E-06
	cgd8_3960			
**2h**	4.57E-07	8.95E-07	1.27E-06	4.35E-06
**6h**	1.89E-07	3.94E-07	5.99E-07	9.96E-07
**12h**	3.76E-09	1.10E-08	2.97E-07	5.22E-07
**24h**	1.31E-07	9.87E-08	8.37E-07	2.47E-07
**36h**	3.49E-07	6.68E-07	3.46E-07	3.90E-07
**48h**	9.94E-08	6.10E-07	1.79E-07	5.41E-07
**72h**	3.90E-08	1.51E-06	9.87E-08	2.87E-08
	cgd8_3980			
**2h**	0.00E+00	0.00E+00	0.00E+00	3.89E-09
**6h**	0.00E+00	0.00E+00	0.00E+00	0.00E+00
**12h**	5.40E-07	2.14E-06	1.49E-06	1.37E-06
**24h**	7.58E-08	2.44E-07	2.08E-07	9.60E-08
**36h**	3.06E-07	1.16E-06	2.16E-06	2.82E-06
**48h**	1.16E-07	1.13E-06	5.64E-07	4.27E-07
**72h**	3.02E-07	3.51E-07	1.06E-06	5.78E-07
	cgd8_4010			
**2h**	3.39E-06	1.58E-06	6.30E-07	6.58E-06
**6h**	1.52E-06	3.43E-06	1.55E-06	5.67E-06
**12h**	3.79E-08	7.97E-08	1.65E-06	1.94E-05
**24h**	9.47E-07	6.11E-07	2.38E-06	1.05E-06
**36h**	3.87E-07	1.96E-06	2.16E-06	1.53E-06
**48h**	3.41E-07	1.66E-06	4.86E-07	4.58E-07
**72h**	7.31E-08	1.10E-07	6.01E-08	4.53E-08
	cgd8_4020			
**2h**	6.69E-09	0.00E+00	0.00E+00	0.00E+00
**6h**	4.24E-09	6.08E-08	0.00E+00	9.51E-09
**12h**	7.87E-06	4.86E-05	2.66E-05	2.83E-05
**24h**	3.03E-06	1.14E-01	2.42E-06	1.23E-06
**36h**	1.82E-06	1.01E-05	1.28E-05	1.31E-05
**48h**	4.70E-07	1.67E-06	2.12E-06	3.24E-06
**72h**	1.47E-07	2.37E-07	2.85E-07	3.87E-07
	cgd8_4030			
**2h**	0.00E+00	0.00E+00	0.00E+00	0.00E+00
**6h**	0.00E+00	0.00E+00	0.00E+00	0.00E+00
**12h**	1.10E-04	2.14E-03	7.88E-04	1.42E-04

**24h**	1.10E-04	2.02E-04	1.31E-04	3.35E-06
**36h**	4.25E-04	7.77E-04	3.86E-04	2.34E-05
**48h**	5.72E-05	2.94E-05	7.60E-05	7.99E-06
**72h**	1.46E-04	3.04E-06	3.38E-04	1.37E-05
	cgd8_4040			
**2h**	0.00E+00	0.00E+00	0.00E+00	0.00E+00
**6h**	9.90E-05	5.93E-04	2.46E-03	1.48E-04
**12h**	1.34E-06	1.28E-04	3.54E-04	1.68E-05
**24h**	4.20E-05	1.95E-04	1.16E-03	8.30E-05
**36h**	1.45E-04	2.50E-04	6.95E-04	2.89E-04
**48h**	1.93E-04	6.51E-04	1.42E-04	9.56E-05
**72h**	5.75E-05	6.60E-05	5.66E-04	3.01E-05
	cgd8_4050			
**2h**	1.23E-05	3.71E-05	2.69E-04	3.20E-05
**6h**	4.80E-05	4.97E-04	2.49E-04	1.90E-04
**12h**	2.12E-06	1.75E-05	3.38E-05	3.58E-05
**24h**	1.04E-05	7.11E-05	7.03E-05	3.68E-05
**36h**	1.10E-05	2.48E-05	1.89E-05	1.22E-05
**48h**	3.53E-06	7.73E-06	1.37E-05	1.32E-05
**72h**	4.83E-07	9.83E-07	1.19E-06	7.48E-07
	cgd8_4070			
**2h**	0.00E+00	0.00E+00	0.00E+00	0.00E+00
**6h**	0.00E+00	0.00E+00	4.45E-08	0.00E+00
**12h**	3.39E-08	3.58E-07	6.41E-08	1.81E-07
**24h**	2.03E-08	5.79E-07	2.82E-07	3.10E-07
**36h**	7.37E-07	1.01E-06	3.19E-07	2.19E-07
**48h**	1.77E-07	1.64E-07	2.46E-07	6.72E-07
**72h**	4.62E-07	2.84E-07	4.06E-07	2.58E-07
	cgd8_4090			
**2h**	6.09E-05	8.23E-06	4.13E-05	6.45E-05
**6h**	0.00E+00	2.16E-07	1.34E-06	4.01E-06
**12h**	2.61E-04	2.40E-04	0.00E+00	8.28E-05
**24h**	2.68E-04	2.67E-05	3.74E-05	3.54E-06
**36h**	8.01E-05	3.88E-05	1.87E-05	4.15E-05
**48h**	6.73E-05	2.37E-05	1.66E-04	2.12E-05
**72h**	8.65E-06	1.92E-05	4.55E-05	3.49E-05
	cgd8_4100			
**2h**	3.30E-07	2.61E-07	7.16E-05	5.69E-08
**6h**	1.74E-07	1.04E-07	5.09E-08	8.99E-08
**12h**	7.65E-08	2.81E-07	1.85E-06	5.24E-07
**24h**	1.29E-06	2.23E-07	3.17E-06	2.91E-07
**36h**	6.54E-08	1.35E-07	4.63E-07	1.93E-07
**48h**	6.76E-08	9.98E-08	2.64E-06	7.99E-08
**72h**	4.02E-08	5.05E-08	2.81E-06	1.62E-07
	cgd8_4130			
**2h**	5.06E-07	8.03E-08	5.29E-04	2.22E-07
**6h**	3.33E-06	1.26E-06	6.60E-05	1.68E-06
**12h**	0.00E+00	3.06E-07	1.12E-07	4.90E-06
**24h**	5.55E-07	1.12E-06	2.38E-06	5.35E-06
**36h**	2.45E-06	7.32E-07	1.07E-05	1.29E-06
**48h**	1.42E-06	7.23E-07	3.42E-05	1.09E-06
**72h**	3.02E-07	1.37E-06	3.99E-06	1.32E-06
	cgd8_4140			

**2h**	1.26E-04	0.00E+00	2.46E-05	2.09E-05
**6h**	1.58E-04	2.92E-05	6.85E-06	7.23E-05
**12h**	2.71E-06	1.73E-06	1.36E-05	3.39E-05
**24h**	2.28E-05	6.69E-05	1.86E-04	2.04E-05
**36h**	6.94E-05	2.63E-05	1.16E-04	3.33E-05
**48h**	1.53E-04	4.86E-05	1.19E-04	5.21E-05
**72h**	2.85E-05	3.90E-05	5.23E-05	3.11E-05
	cgd8_4150			
**2h**	7.44E-07	7.66E-07	6.79E-04	1.86E-05
**6h**	3.23E-06	1.57E-06	4.38E-07	3.06E-05
**12h**	9.99E-08	7.99E-07	3.75E-07	4.68E-06
**24h**	5.32E-07	8.02E-07	1.18E-06	1.07E-05
**36h**	3.01E-07	3.88E-07	2.51E-06	4.60E-06
**48h**	2.54E-07	2.39E-07	9.48E-07	3.03E-06
**72h**	1.89E-07	9.99E-07	3.03E-07	1.33E-06
	cgd8_4160			
**2h**	7.30E-06	8.99E-06	3.67E-04	4.51E-05
**6h**	7.07E-05	4.74E-06	1.75E-05	1.58E-05
**12h**	6.33E-06	6.84E-06	5.72E-05	1.60E-05
**24h**	8.08E-06	7.48E-06	3.38E-05	4.92E-05
**36h**	1.84E-05	8.61E-06	8.06E-05	3.58E-05
**48h**	0.00E+00	0.00E+00	0.00E+00	7.71E-05
**72h**	6.90E-05	1.25E-05	4.66E-05	1.55E-04
	cgd8_4200			
**2h**	1.42E-06	2.04E-07	6.73E-06	3.14E-06
**6h**	0.00E+00	4.41E-07	9.19E-08	5.66E-07
**12h**	4.79E-07	2.06E-07	1.33E-07	4.25E-07
**24h**	2.25E-07	3.92E-07	1.10E-06	1.37E-06
**36h**	1.61E-07	1.13E-07	3.29E-06	1.41E-06
**48h**	5.19E-07	3.52E-07	2.38E-06	6.62E-07
**72h**	1.55E-07	6.90E-07	1.76E-07	0.00E+00
	cgd8_4210			
**2h**	3.64E-04	1.73E-04	1.24E-03	1.26E-04
**6h**	0.00E+00	2.45E-06	2.71E-06	4.76E-05
**12h**	4.14E-05	8.59E-05	2.92E-05	6.75E-05
**24h**	1.56E-04	7.96E-05	4.60E-05	3.82E-05
**36h**	9.92E-05	4.88E-05	1.44E-05	7.81E-05
**48h**	3.47E-04	1.13E-04	1.36E-04	2.60E-05
**72h**	2.18E-04	2.29E-04	0.00E+00	3.98E-05
	cgd8_4220			
**2h**	2.06E-05	4.91E-05	1.65E-03	4.98E-05
**6h**	2.55E-06	3.37E-06	1.63E-06	1.02E-05
**12h**	1.04E-05	2.66E-05	1.12E-05	3.44E-05
**24h**	1.85E-05	7.33E-06	3.03E-05	1.35E-05
**36h**	5.05E-06	4.64E-06	1.12E-05	2.21E-05
**48h**	7.33E-06	4.70E-06	1.24E-05	1.29E-05
**72h**	1.56E-06	9.65E-06	1.84E-06	1.34E-05
	cgd8_4230			
**2h**	0.00E+00	0.00E+00	1.13E-06	0.00E+00
**6h**	2.94E-07	5.79E-07	5.13E-06	7.10E-06
**12h**	1.67E-09	1.34E-08	0.00E+00	0.00E+00
**24h**	2.37E-07	6.70E-07	3.65E-07	2.12E-07
**36h**	3.36E-06	7.41E-08	2.57E-07	2.39E-07

**48h**	7.60E-06	6.80E-06	1.60E-05	1.29E-05
**72h**	5.56E-06	1.35E-05	0.00E+00	3.83E-06
	cgd8_4240			
**2h**	1.04E-04	1.79E-07	4.01E-05	2.32E-06
**6h**	2.13E-06	4.40E-06	3.81E-06	1.36E-05
**12h**	6.90E-08	5.10E-07	1.96E-07	7.96E-07
**24h**	4.58E-07	1.51E-06	1.89E-06	3.78E-06
**36h**	3.45E-07	3.86E-07	6.23E-07	2.78E-06
**48h**	5.07E-07	3.61E-07	6.56E-07	3.07E-06
**72h**	3.75E-07	3.50E-07	8.65E-08	7.59E-07
	cgd8_4260			
**2h**	8.04E-06	5.07E-06	7.16E-03	6.64E-05
**6h**	5.68E-06	6.09E-06	2.54E-04	8.31E-06
**12h**	3.81E-06	1.36E-05	3.64E-06	8.12E-06
**24h**	9.49E-06	5.31E-06	3.09E-06	1.20E-05
**36h**	6.67E-06	7.15E-06	4.02E-06	1.38E-05
**48h**	1.50E-05	1.79E-06	7.80E-06	4.22E-05
**72h**	4.54E-07	1.97E-06	1.99E-06	1.19E-06
	cgd8_4280			
**2h**	2.65E-06	1.14E-06	7.46E-06	0.00E+00
**6h**	1.71E-06	3.91E-06	0.00E+00	0.00E+00
**12h**	2.55E-07	1.23E-05	0.00E+00	0.00E+00
**24h**	1.78E-06	1.87E-06	0.00E+00	0.00E+00
**36h**	1.27E-06	2.18E-06	0.00E+00	0.00E+00
**48h**	1.99E-06	6.77E-06	0.00E+00	0.00E+00
**72h**	2.26E-06	1.49E-05	0.00E+00	0.00E+00
	cgd8_4290			
**2h**	1.99E-08	2.02E-08	2.69E-08	2.58E-05
**6h**	0.00E+00	0.00E+00	0.00E+00	2.48E-08
**12h**	5.86E-10	0.00E+00	9.72E-08	2.00E-08
**24h**	3.47E-08	7.94E-08	2.36E-08	9.43E-08
**36h**	8.79E-08	5.57E-08	7.88E-08	1.82E-07
**48h**	4.85E-06	4.66E-06	6.32E-06	4.95E-04
**72h**	3.28E-06	9.29E-06	1.09E-05	2.64E-04
	cgd8_4300			
**2h**	4.66E-09	0.00E+00	0.00E+00	0.00E+00
**6h**	5.83E-09	0.00E+00	6.31E-09	1.57E-09
**12h**	0.00E+00	0.00E+00	6.14E-09	1.06E-09
**24h**	2.60E-08	5.07E-08	1.67E-08	6.93E-09
**36h**	1.02E-07	1.32E-07	5.66E-08	8.62E-08
**48h**	2.26E-06	1.29E-06	7.43E-06	4.17E-06
**72h**	2.92E-06	2.58E-06	4.21E-06	2.99E-06
	cgd8_4320			
**2h**	3.16E-07	6.39E-06	3.86E-06	4.23E-06
**6h**	1.70E-07	1.76E-06	1.53E-07	3.93E-07
**12h**	7.39E-07	3.05E-05	3.22E-06	1.87E-07
**24h**	3.16E-07	5.78E-06	1.18E-06	3.48E-07
**36h**	2.29E-06	1.49E-05	1.40E-06	1.97E-06
**48h**	4.15E-07	6.26E-06	5.00E-06	5.35E-06
**72h**	4.68E-06	2.99E-05	1.14E-05	1.19E-05
	cgd8_4340			
**2h**	1.07E-03	5.16E-04	2.23E-03	4.44E-03
**6h**	1.15E-04	9.32E-04	2.23E-03	2.78E-03

**12h**	1.52E-04	5.10E-04	5.28E-03	1.84E-05
**24h**	3.84E-04	5.76E-04	2.83E-03	2.52E-04
**36h**	2.60E-04	9.42E-05	6.31E-04	1.78E-03
**48h**	5.13E-05	8.08E-05	9.30E-04	4.67E-04
**72h**	3.16E-05	9.62E-05	6.33E-04	1.42E-04
	cgd8_4360			
**2h**	8.56E-06	6.78E-05	2.23E-05	1.35E-05
**6h**	3.95E-05	2.28E-04	8.29E-05	1.56E-04
**12h**	2.00E-06	2.34E-05	7.54E-05	2.89E-06
**24h**	4.63E-06	3.72E-05	3.73E-05	6.18E-06
**36h**	1.02E-05	4.10E-05	1.46E-05	9.56E-06
**48h**	1.32E-06	8.72E-06	5.99E-06	5.58E-06
**72h**	1.60E-06	4.65E-06	3.41E-06	2.33E-06
	cgd8_4380			
**2h**	1.65E-07	6.08E-07	1.36E-07	2.17E-07
**6h**	1.76E-07	6.45E-07	3.63E-07	7.38E-07
**12h**	5.09E-08	1.24E-07	3.91E-07	3.72E-08
**24h**	1.32E-07	3.80E-07	5.84E-07	6.17E-08
**36h**	2.83E-07	3.54E-07	2.11E-07	2.09E-07
**48h**	1.44E-07	1.75E-07	2.42E-07	2.39E-07
**72h**	2.01E-07	1.95E-07	2.61E-07	1.12E-07
	cgd8_4390			
**2h**	0.00E+00	0.00E+00	0.00E+00	4.94E-10
**6h**	0.00E+00	0.00E+00	0.00E+00	0.00E+00
**12h**	0.00E+00	0.00E+00	0.00E+00	0.00E+00
**24h**	3.26E-10	8.69E-11	2.84E-09	2.71E-10
**36h**	3.53E-08	1.33E-09	1.56E-08	5.52E-08
**48h**	1.10E-05	4.88E-06	2.43E-05	8.25E-06
**72h**	6.68E-06	6.52E-06	7.88E-06	6.87E-06
	cgd8_4400			
**2h**	1.34E-06	5.37E-07	3.84E-07	1.14E-06
**6h**	3.83E-07	3.92E-07	2.15E-06	2.22E-06
**12h**	3.14E-08	5.82E-08	3.94E-06	4.01E-08
**24h**	1.17E-07	3.25E-07	1.72E-06	1.07E-07
**36h**	6.29E-07	4.68E-07	3.70E-07	4.30E-07
**48h**	1.90E-07	3.27E-07	5.24E-07	3.71E-07
**72h**	6.92E-07	5.40E-07	8.92E-07	4.40E-07
	cgd8_4410			
**2h**	2.16E-06	2.96E-06	1.71E-06	3.55E-06
**6h**	2.13E-06	2.70E-06	3.58E-06	1.11E-05
**12h**	1.29E-07	5.16E-07	2.05E-06	7.68E-08
**24h**	4.85E-07	1.32E-06	2.98E-06	3.03E-07
**36h**	3.94E-07	1.45E-06	4.92E-07	5.11E-07
**48h**	6.86E-07	4.39E-07	1.58E-06	8.80E-07
**72h**	1.37E-07	5.92E-07	1.92E-07	1.85E-07
	cgd8_4420			
**2h**	5.14E-07	4.47E-07	3.85E-07	1.30E-05
**6h**	2.46E-07	9.53E-07	2.62E-06	3.01E-06
**12h**	1.17E-08	5.43E-08	1.68E-07	2.52E-09
**24h**	1.05E-07	1.18E-07	3.59E-07	5.08E-08
**36h**	1.37E-07	1.48E-07	1.02E-07	1.08E-07
**48h**	5.51E-07	2.45E-07	4.10E-07	1.33E-06
**72h**	1.28E-07	4.12E-07	2.02E-07	3.10E-07

	cgd8_4430			
**2h**	3.06E-06	7.73E-07	1.12E-06	4.13E-06
**6h**	8.30E-07	2.70E-06	2.58E-06	1.14E-04
**12h**	1.58E-07	1.78E-07	1.28E-06	5.46E-08
**24h**	3.54E-07	2.00E-07	1.09E-06	1.68E-07
**36h**	1.27E-06	4.19E-07	5.94E-07	3.07E-07
**48h**	1.28E-07	1.25E-07	1.18E-07	1.72E-07
**72h**	2.19E-07	1.14E-07	1.93E-07	7.32E-08
	cgd8_4450			
**2h**	0.00E+00	0.00E+00	1.65E-08	0.00E+00
**6h**	0.00E+00	0.00E+00	0.00E+00	0.00E+00
**12h**	0.00E+00	6.00E-09	0.00E+00	8.15E-09
**24h**	9.18E-09	4.50E-09	8.32E-08	1.47E-07
**36h**	4.09E-08	3.23E-08	1.90E-08	3.85E-07
**48h**	2.90E-07	1.38E-07	5.92E-07	1.04E-06
**72h**	2.28E-07	2.83E-07	8.68E-07	7.50E-07
	cgd8_4460			
**2h**	1.17E-06	4.48E-07	8.08E-06	3.87E-05
**6h**	2.78E-06	2.42E-06	1.53E-05	1.23E-05
**12h**	9.03E-07	5.28E-07	2.55E-06	3.14E-04
**24h**	2.11E-06	1.04E-06	6.22E-06	5.70E-04
**36h**	7.57E-07	7.77E-07	1.24E-06	1.09E-05
**48h**	7.96E-07	5.68E-07	2.24E-06	8.71E-06
**72h**	9.00E-07	1.32E-06	1.71E-06	1.51E-06
	cgd8_4470			
**2h**	6.61E-06	1.62E-06	0.00E+00	9.30E-05
**6h**	3.25E-04	9.09E-04	4.43E-04	6.71E-04
**12h**	8.22E-05	3.90E-04	2.46E-04	4.18E-04
**24h**	1.75E-04	2.52E-04	2.64E-04	3.71E-04
**36h**	2.00E-04	4.87E-04	1.82E-04	5.21E-04
**48h**	2.13E-04	3.35E-04	1.29E-04	8.17E-04
**72h**	4.12E-05	2.33E-04	1.48E-04	2.02E-04
	cgd8_4480			
**2h**	0.00E+00	0.00E+00	0.00E+00	0.00E+00
**6h**	0.00E+00	0.00E+00	0.00E+00	1.85E-08
**12h**	7.28E-06	9.35E-06	2.73E-05	1.02E-05
**24h**	1.09E-06	2.91E-06	3.63E-06	3.66E-07
**36h**	1.79E-06	1.28E-06	2.05E-05	1.48E-05
**48h**	1.53E-06	1.79E-06	8.76E-06	5.48E-06
**72h**	1.09E-06	1.59E-06	8.96E-06	4.52E-06
	cgd8_4490			
**2h**	0.00E+00	0.00E+00	0.00E+00	0.00E+00
**6h**	0.00E+00	0.00E+00	0.00E+00	0.00E+00
**12h**	1.98E-03	9.28E-04	4.14E-05	1.07E-04
**24h**	2.14E-04	7.32E-05	1.86E-05	1.72E-05
**36h**	1.01E-04	4.08E-05	3.52E-05	2.66E-05
**48h**	1.16E-04	1.18E-04	6.04E-05	2.85E-05
**72h**	1.17E-04	9.72E-05	6.36E-05	1.86E-05
	cgd8_4500			
**2h**	1.05E-03	1.37E-04	7.11E-05	6.79E-04
**6h**	3.04E-04	1.03E-04	3.57E-05	9.89E-05
**12h**	1.21E-03	2.07E-04	8.65E-05	1.95E-04
**24h**	3.28E-03	3.75E-04	8.04E-05	1.26E-04

**36h**	3.39E-04	4.14E-04	8.54E-05	1.01E-04
**48h**	5.86E-05	1.32E-04	7.21E-05	1.53E-04
**72h**	4.32E-04	1.49E-04	1.52E-04	8.21E-05
	cgd8_4540			
**2h**	8.68E-09	1.06E-09	0.00E+00	2.32E-07
**6h**	4.04E-10	0.00E+00	1.04E-08	9.77E-10
**12h**	6.87E-08	3.01E-08	1.41E-07	1.36E-07
**24h**	3.34E-08	3.14E-08	1.59E-08	3.38E-08
**36h**	1.04E-07	4.53E-08	1.14E-07	8.61E-08
**48h**	4.81E-08	4.14E-08	5.03E-08	1.36E-07
**72h**	8.00E-08	2.64E-08	6.73E-08	2.85E-08
	cgd8_4550			
**2h**	0.00E+00	0.00E+00	0.00E+00	0.00E+00
**6h**	0.00E+00	0.00E+00	0.00E+00	0.00E+00
**12h**	4.57E-08	2.68E-08	9.62E-09	5.60E-08
**24h**	1.12E-09	1.73E-09	6.76E-10	7.11E-09
**36h**	6.97E-09	1.74E-08	2.47E-08	7.76E-09
**48h**	1.57E-08	1.51E-08	3.31E-08	2.33E-08
**72h**	7.68E-09	1.29E-08	2.47E-08	3.46E-08
	cgd8_4570			
**2h**	8.51E-08	1.15E-08	1.51E-09	5.52E-07
**6h**	4.61E-08	1.02E-08	1.03E-07	1.57E-07
**12h**	2.63E-06	1.47E-06	5.37E-06	1.36E-06
**24h**	4.95E-07	1.79E-07	5.75E-07	3.07E-07
**36h**	8.31E-07	4.91E-07	1.14E-06	2.11E-06
**48h**	2.78E-07	2.44E-07	4.13E-07	5.94E-07
**72h**	1.19E-07	7.38E-08	3.18E-07	1.02E-07
	cgd8_4620			
**2h**	1.41E-05	1.69E-05	5.82E-06	2.91E-05
**6h**	4.01E-06	4.67E-06	1.09E-05	9.19E-06
**12h**	5.96E-06	5.27E-06	8.14E-06	5.07E-06
**24h**	5.84E-06	6.12E-06	8.50E-06	2.02E-06
**36h**	4.68E-06	2.22E-06	4.67E-06	4.74E-06
**48h**	8.10E-06	3.93E-06	5.12E-06	1.17E-05
**72h**	2.51E-06	1.64E-06	4.45E-06	2.26E-06
	cgd8_4640			
**2h**	4.31E-06	2.99E-05	1.66E-06	8.06E-05
**6h**	4.66E-07	3.58E-06	6.47E-07	1.71E-06
**12h**	1.38E-06	5.24E-06	1.07E-06	1.78E-07
**24h**	7.38E-07	8.64E-07	4.14E-06	3.05E-07
**36h**	2.34E-06	6.54E-06	2.85E-06	1.80E-06
**48h**	2.22E-06	2.71E-06	2.91E-06	2.76E-05
**72h**	1.84E-06	5.96E-06	7.40E-06	1.19E-06
	cgd8_4660			
**2h**	0.00E+00	0.00E+00	0.00E+00	0.00E+00
**6h**	0.00E+00	3.67E-09	1.78E-09	0.00E+00
**12h**	3.02E-08	3.61E-08	1.51E-08	1.45E-07
**24h**	1.13E-08	7.25E-09	1.05E-09	1.09E-09
**36h**	1.18E-08	7.47E-09	1.08E-08	3.05E-08
**48h**	1.42E-07	8.83E-08	5.48E-08	1.43E-07
**72h**	8.02E-08	2.15E-08	9.43E-08	4.51E-08
	cgd8_4710			
**2h**	1.33E-04	2.06E-04	6.25E-04	6.43E-05

**6h**	5.91E-05	3.77E-06	1.03E-05	9.79E-04
**12h**	1.24E-03	2.22E-04	1.41E-03	3.40E-03
**24h**	1.98E-03	3.25E-04	9.60E-04	1.40E-04
**36h**	4.47E-04	1.14E-04	3.98E-04	6.52E-04
**48h**	3.81E-04	5.38E-05	3.60E-04	1.62E-03
**72h**	2.42E-04	9.54E-05	3.32E-04	1.09E-03
	cgd8_4730			
**2h**	1.46E-06	3.61E-08	0.00E+00	1.17E-05
**6h**	1.94E-05	2.46E-06	2.31E-05	1.06E-04
**12h**	1.60E-04	1.62E-05	1.45E-04	1.86E-04
**24h**	3.71E-05	1.58E-05	1.18E-04	8.26E-05
**36h**	6.14E-05	1.38E-05	9.95E-05	1.68E-04
**48h**	4.09E-05	1.49E-05	8.43E-05	4.80E-05
**72h**	1.87E-05	4.50E-06	2.84E-05	6.49E-05
	cgd8_4740			
**2h**	4.01E-08	7.38E-08	1.61E-07	2.14E-06
**6h**	2.45E-07	6.59E-08	9.43E-08	1.41E-06
**12h**	2.37E-09	3.19E-10	2.17E-08	4.76E-09
**24h**	9.28E-08	7.01E-08	1.02E-07	1.04E-07
**36h**	1.76E-07	5.60E-08	6.10E-08	9.56E-08
**48h**	7.78E-07	1.33E-07	4.54E-07	1.31E-06
**72h**	5.36E-07	5.71E-08	3.68E-08	1.20E-07
	cgd8_4760			
**2h**	3.88E-08	1.45E-07	8.61E-08	1.34E-06
**6h**	2.41E-07	1.12E-07	1.09E-07	2.15E-06
**12h**	1.73E-07	8.90E-09	6.19E-07	4.48E-07
**24h**	3.51E-07	1.46E-07	2.63E-07	5.69E-07
**36h**	2.21E-07	1.81E-07	2.39E-07	1.61E-07
**48h**	2.43E-07	2.49E-08	1.03E-07	2.21E-07
**72h**	1.17E-07	5.29E-08	6.51E-08	1.87E-07
	cgd8_4770			
**2h**	8.04E-05	8.18E-05	3.92E-05	8.02E-05
**6h**	1.65E-03	9.18E-05	2.52E-03	4.51E-04
**12h**	3.68E-05	4.00E-06	3.06E-04	1.16E-04
**24h**	7.39E-05	2.41E-05	4.97E-04	2.21E-04
**36h**	3.51E-04	1.00E-04	3.15E-04	4.98E-05
**48h**	2.22E-04	4.48E-05	3.25E-04	2.06E-05
**72h**	7.71E-05	1.25E-05	7.04E-05	4.61E-05
	cgd8_4790			
**2h**	1.83E-05	6.26E-04	7.48E-04	5.20E-05
**6h**	9.08E-04	8.49E-04	1.96E-04	6.69E-05
**12h**	2.53E-04	2.39E-04	5.11E-04	1.43E-04
**24h**	2.94E-04	7.66E-04	4.42E-04	7.42E-05
**36h**	4.44E-04	8.26E-04	2.85E-04	1.38E-05
**48h**	1.82E-04	2.67E-04	3.45E-04	1.01E-05
**72h**	4.59E-04	1.21E-04	1.30E-04	1.27E-05
	cgd7_2680			
**2h**	1.06E-07	1.12E-06	6.03E-07	1.44E-05
**6h**	2.31E-06	1.09E-06	5.15E-06	6.36E-05
**12h**	5.24E-06	1.63E-06	4.61E-06	1.44E-05
**24h**	1.26E-06	8.59E-07	6.74E-06	1.14E-05
**36h**	1.87E-06	1.58E-06	1.81E-06	1.55E-05
**48h**	3.16E-06	5.90E-07	4.44E-06	3.59E-05

**72h**	9.42E-07	6.27E-07	1.30E-06	1.19E-05
	cgd7_2700			
**2h**	3.70E-07	4.61E-07	8.91E-08	2.42E-05
**6h**	7.47E-08	2.26E-08	8.61E-07	2.80E-05
**12h**	1.57E-07	2.67E-07	1.11E-06	1.16E-05
**24h**	4.11E-07	5.00E-07	2.69E-06	1.31E-05
**36h**	1.43E-06	1.14E-06	1.91E-06	8.92E-06
**48h**	7.33E-06	2.94E-06	4.02E-06	3.23E-05
**72h**	1.03E-05	2.40E-06	6.89E-06	3.58E-05
	cgd7_2710			
**2h**	4.02E-07	3.68E-06	4.32E-07	2.99E-05
**6h**	8.58E-07	1.29E-06	7.09E-06	1.40E-04
**12h**	1.59E-07	3.77E-07	7.37E-07	8.98E-06
**24h**	4.33E-07	1.02E-06	1.43E-06	1.28E-05
**36h**	1.77E-06	3.36E-06	9.74E-07	1.44E-05
**48h**	1.44E-06	1.13E-06	8.17E-07	1.62E-05
**72h**	6.84E-07	4.83E-07	1.24E-07	4.14E-06
	cgd7_2720			
**2h**	1.22E-06	1.69E-05	2.77E-06	4.38E-04
**6h**	3.30E-06	6.00E-06	2.76E-06	2.36E-04
**12h**	1.84E-07	2.76E-07	9.93E-07	2.33E-05
**24h**	8.48E-07	2.42E-06	4.04E-06	7.72E-05
**36h**	2.35E-06	7.23E-06	2.12E-06	7.88E-05
**48h**	1.14E-06	2.21E-06	8.97E-07	3.33E-05
**72h**	1.35E-06	3.58E-07	3.09E-07	1.25E-05
	cgd7_2740			
**2h**	1.26E-06	2.60E-08	0.00E+00	1.44E-07
**6h**	3.12E-06	2.42E-06	3.67E-07	7.31E-06
**12h**	6.36E-08	1.12E-08	4.08E-08	5.38E-08
**24h**	1.67E-06	2.94E-07	4.80E-07	1.05E-06
**36h**	3.93E-07	3.03E-07	2.12E-07	6.34E-07
**48h**	9.89E-07	3.83E-07	3.44E-07	5.75E-07
**72h**	1.90E-07	1.59E-07	3.80E-08	1.89E-07
	cgd7_2760			
**2h**	1.24E-05	5.52E-07	0.00E+00	1.21E-05
**6h**	1.98E-06	1.24E-06	2.21E-08	1.51E-07
**12h**	2.44E-06	3.58E-08	1.26E-07	4.72E-08
**24h**	7.41E-06	1.02E-06	3.86E-07	4.76E-07
**36h**	5.87E-06	2.45E-06	3.11E-07	5.19E-07
**48h**	2.34E-05	4.46E-06	1.67E-06	3.32E-06
**72h**	1.16E-05	5.44E-06	1.19E-06	2.27E-06
	cgd7_2770			
**2h**	6.02E-07	4.87E-09	0.00E+00	9.28E-09
**6h**	3.35E-08	1.84E-08	2.66E-09	0.00E+00
**12h**	2.76E-06	5.87E-07	2.68E-07	2.64E-07
**24h**	2.98E-06	7.00E-07	6.49E-07	8.28E-07
**36h**	4.39E-06	3.16E-06	1.29E-06	2.03E-06
**48h**	1.43E-05	1.90E-06	2.05E-06	5.21E-06
**72h**	9.72E-06	3.63E-06	2.30E-06	3.41E-06
	cgd7_2780			
**2h**	0.00E+00	0.00E+00	0.00E+00	0.00E+00
**6h**	0.00E+00	1.31E-07	0.00E+00	0.00E+00
**12h**	2.04E-04	3.25E-04	9.24E-06	8.82E-06

**24h**	3.77E-05	1.56E-05	1.07E-06	2.19E-06
**36h**	9.16E-06	1.94E-05	4.18E-06	6.31E-06
**48h**	8.69E-06	2.94E-06	7.73E-07	1.15E-06
**72h**	2.28E-05	1.05E-05	8.83E-07	1.43E-06
	cgd7_2790			
**2h**	7.71E-05	1.24E-04	8.59E-07	3.83E-06
**6h**	1.51E-04	2.43E-04	9.83E-06	8.55E-06
**12h**	1.31E-05	1.56E-04	6.07E-06	1.05E-06
**24h**	1.68E-05	1.03E-04	6.24E-06	6.17E-06
**36h**	1.41E-05	4.99E-05	5.95E-06	3.33E-06
**48h**	9.30E-06	1.95E-05	5.15E-06	4.03E-06
**72h**	2.09E-05	4.24E-05	1.12E-06	2.51E-06
	cgd7_2820			
**2h**	7.57E-05	3.21E-05	0.00E+00	4.84E-06
**6h**	2.67E-05	1.02E-05	2.38E-06	1.60E-06
**12h**	1.86E-05	3.70E-05	2.82E-06	1.22E-06
**24h**	1.31E-05	1.89E-05	6.00E-06	8.70E-06
**36h**	1.03E-04	4.99E-05	3.84E-06	5.84E-06
**48h**	6.37E-05	2.31E-04	4.29E-05	7.92E-05
**72h**	1.66E-05	5.72E-05	8.90E-06	2.11E-06
	cgd7_2830			
**2h**	3.77E-06	1.16E-06	2.28E-07	2.17E-06
**6h**	9.68E-07	1.48E-06	4.26E-07	9.51E-07
**12h**	3.22E-07	2.30E-07	2.63E-07	3.21E-07
**24h**	1.69E-06	2.74E-07	4.33E-07	6.55E-07
**36h**	4.95E-07	9.45E-07	3.67E-07	7.07E-07
**48h**	9.43E-07	4.29E-07	1.83E-07	3.95E-07
**72h**	4.39E-07	1.65E-07	8.40E-08	8.72E-08
	cgd7_2850			
**2h**	1.47E-04	1.47E-05	8.13E-06	2.09E-05
**6h**	8.52E-04	1.62E-05	7.80E-06	2.08E-05
**12h**	1.82E-04	7.29E-06	8.24E-06	5.75E-06
**24h**	3.52E-04	1.06E-05	9.54E-06	1.39E-05
**36h**	7.69E-05	6.34E-06	6.62E-06	8.05E-06
**48h**	1.79E-04	7.28E-06	2.62E-06	5.92E-06
**72h**	1.14E-05	1.53E-06	4.82E-07	6.83E-07
	cgd7_2860			
**2h**	0.00E+00	3.78E-10	1.24E-08	4.26E-10
**6h**	1.38E-09	4.15E-09	8.49E-08	9.30E-08
**12h**	9.94E-08	1.49E-08	5.70E-08	3.65E-08
**24h**	3.14E-08	5.35E-08	7.71E-08	1.30E-07
**36h**	1.18E-07	9.27E-08	1.18E-07	2.80E-07
**48h**	2.52E-07	5.43E-08	1.17E-07	3.00E-07
**72h**	3.79E-08	5.98E-08	2.98E-08	1.36E-08
	cgd7_2870			
**2h**	0.00E+00	0.00E+00	0.00E+00	0.00E+00
**6h**	2.36E-06	5.68E-07	6.91E-08	1.98E-07
**12h**	1.12E-04	2.97E-04	9.36E-05	4.14E-05
**24h**	1.82E-05	3.18E-05	3.81E-06	7.49E-06
**36h**	1.27E-05	3.12E-05	2.16E-05	3.56E-05
**48h**	2.00E-05	2.02E-05	1.59E-06	3.71E-06
**72h**	5.72E-06	9.20E-06	6.49E-07	1.88E-06
	cgd7_2900			

2h	3.14E-06	2.25E-06	3.23E-07	3.25E-06
6h	1.03E-06	1.07E-06	1.31E-06	1.87E-06
12h	6.60E-07	2.05E-07	1.25E-06	4.52E-07
24h	1.22E-06	4.89E-07	1.11E-06	7.92E-07
36h	2.51E-06	9.83E-07	1.97E-06	1.09E-06
48h	2.00E-06	4.88E-07	8.80E-07	1.19E-06
72h	6.03E-07	2.65E-07	4.21E-07	2.56E-07
	cgd7_2910			
2h	4.49E-04	6.43E-04	2.69E-05	2.34E-04
6h	7.78E-05	7.67E-04	9.60E-05	3.60E-04
12h	8.60E-05	1.38E-04	1.79E-04	3.92E-04
24h	9.71E-05	2.54E-04	7.93E-05	9.15E-05
36h	1.53E-04	2.19E-04	7.89E-05	2.64E-04
48h	8.88E-05	9.55E-05	1.46E-04	1.19E-04
72h	2.10E-05	6.03E-05	6.79E-05	2.92E-05
	cgd7_2920			
2h	3.49E-05	5.78E-05	6.42E-05	1.53E-05
6h	1.09E-05	8.28E-05	5.50E-05	2.44E-05
12h	7.17E-06	1.17E-05	7.78E-05	1.70E-05
24h	1.26E-05	5.40E-05	1.31E-04	3.61E-05
36h	4.01E-05	4.79E-05	5.07E-05	4.62E-05
48h	1.77E-05	1.76E-05	7.67E-05	2.19E-05
72h	5.28E-06	1.31E-05	1.61E-05	5.22E-06
	cgd7_2930			
2h	1.68E-07	2.10E-07	2.36E-07	4.43E-07
6h	1.92E-06	4.26E-06	4.18E-06	3.10E-06
12h	1.49E-07	9.16E-08	3.87E-07	0.00E+00
24h	4.96E-07	9.30E-07	2.64E-06	3.50E-06
36h	2.80E-05	1.07E-06	4.71E-07	1.12E-06
48h	8.77E-07	8.41E-07	1.98E-06	2.98E-06
72h	3.42E-07	5.42E-07	1.54E-07	5.31E-07
	cgd7_2950			
2h	4.89E-04	4.99E-05	2.84E-07	4.74E-03
6h	5.93E-04	1.58E-04	1.06E-05	1.78E-02
12h	4.32E-04	1.71E-04	6.49E-06	3.62E-03
24h	6.04E-04	7.46E-05	1.93E-05	3.49E-04
36h	1.48E-03	2.77E-04	1.36E-05	1.81E-02
48h	9.53E-04	1.13E-04	1.12E-05	7.26E-03
72h	1.12E-04	3.79E-05	6.68E-06	1.44E-03
	cgd7_2960			
2h	1.11E-07	1.18E-06	6.59E-06	3.72E-07
6h	1.95E-06	1.41E-05	1.15E-04	4.54E-06
12h	3.56E-06	4.86E-06	1.38E-05	1.03E-06
24h	2.82E-06	2.65E-06	2.31E-05	2.74E-06
36h	9.20E-06	8.39E-06	1.09E-05	4.93E-06
48h	3.95E-06	2.87E-06	2.25E-05	1.81E-06
72h	5.56E-07	1.80E-06	8.96E-07	6.06E-07
	cgd7_2980			
2h	9.81E-06	0.00E+00	0.00E+00	0.00E+00
6h	8.70E-04	2.03E-04	1.97E-05	9.10E-04
12h	2.40E-04	2.36E-04	4.72E-06	4.29E-04
24h	2.40E-05	1.37E-04	7.67E-06	6.87E-04
36h	7.02E-04	6.43E-05	1.83E-05	1.58E-03

**48h**	1.96E-04	3.66E-05	6.53E-06	8.82E-04
**72h**	2.20E-04	3.83E-05	2.71E-06	1.94E-04
	cgd7_2990			
**2h**	8.87E-08	5.76E-08	0.00E+00	5.49E-08
**6h**	2.75E-08	2.09E-08	2.11E-07	1.42E-08
**12h**	0.00E+00	0.00E+00	5.67E-08	0.00E+00
**24h**	1.59E-09	4.39E-09	2.01E-08	4.30E-09
**36h**	2.97E-08	3.33E-08	1.55E-08	2.09E-08
**48h**	2.08E-07	7.06E-08	6.03E-07	1.39E-07
**72h**	5.51E-08	1.19E-07	1.95E-07	5.86E-08
	cgd7_3030			
**2h**	5.15E-06	9.97E-06	2.32E-05	2.87E-06
**6h**	1.22E-05	2.00E-05	3.02E-04	2.18E-05
**12h**	1.70E-06	2.42E-06	1.51E-04	2.37E-06
**24h**	5.70E-06	8.95E-06	2.26E-04	1.38E-05
**36h**	9.17E-06	1.64E-05	1.04E-04	1.88E-05
**48h**	7.74E-06	6.31E-06	1.36E-04	1.15E-05
**72h**	1.18E-06	2.52E-06	1.49E-05	3.15E-07
	cgd7_3040			
**2h**	0.00E+00	3.23E-08	3.94E-07	0.00E+00
**6h**	7.66E-08	8.39E-08	1.25E-06	2.69E-08
**12h**	5.16E-07	4.50E-07	1.00E-05	4.40E-07
**24h**	4.81E-07	4.97E-07	1.24E-06	2.54E-07
**36h**	9.74E-07	4.70E-07	2.43E-06	1.58E-06
**48h**	1.02E-06	1.41E-07	9.14E-06	1.44E-06
**72h**	1.49E-07	5.18E-07	1.10E-06	1.85E-07
	cgd7_3050			
**2h**	1.81E-04	4.86E-04	8.33E-06	3.93E-05
**6h**	3.18E-05	1.10E-04	5.15E-05	2.10E-05
**12h**	1.77E-05	4.00E-05	7.12E-05	2.40E-05
**24h**	3.56E-05	3.73E-05	2.82E-05	2.64E-05
**36h**	4.94E-05	6.19E-05	2.23E-05	3.00E-05
**48h**	5.69E-05	6.53E-05	3.50E-05	5.12E-05
**72h**	8.56E-06	2.04E-05	3.01E-05	3.47E-06
	cgd7_3060			
**2h**	9.76E-07	2.81E-07	2.28E-05	7.70E-07
**6h**	7.55E-07	4.48E-07	6.05E-06	1.26E-06
**12h**	1.90E-07	1.02E-07	2.19E-06	2.46E-07
**24h**	4.25E-07	1.61E-07	2.51E-06	9.10E-07
**36h**	1.27E-06	6.45E-07	7.51E-06	1.23E-06
**48h**	9.68E-07	4.83E-07	5.23E-06	7.99E-07
**72h**	1.41E-07	1.81E-07	1.82E-06	2.42E-07
	cgd7_3010			
**2h**	1.33E-02	7.37E-04	3.79E-03	3.11E-02
**6h**	2.27E-03	3.32E-04	1.71E-03	1.38E-02
**12h**	7.02E-03	3.65E-03	2.14E-03	9.90E-03
**24h**	1.86E-03	5.09E-04	8.95E-04	2.40E-02
**36h**	1.63E-03	1.01E-03	7.63E-04	9.56E-03
**48h**	8.30E-04	1.56E-03	7.79E-04	1.55E-02
**72h**	5.96E-04	4.99E-04	2.79E-04	3.86E-03
	cgd7_3070			
**2h**	1.09E-06	1.15E-06	3.57E-06	3.47E-06
**6h**	1.57E-05	1.11E-05	1.28E-04	2.53E-04

**12h**	1.56E-06	2.28E-06	1.61E-05	2.60E-05
**24h**	4.69E-06	2.66E-06	2.15E-05	5.66E-05
**36h**	8.25E-06	5.90E-06	1.66E-05	1.16E-05
**48h**	3.82E-06	3.24E-06	5.64E-06	8.02E-06
**72h**	2.52E-07	4.85E-07	6.78E-07	4.58E-07
	cgd7_3080			
**2h**	0.00E+00	1.46E-08	0.00E+00	7.96E-08
**6h**	2.33E-08	1.20E-08	1.67E-08	6.72E-09
**12h**	7.98E-08	7.43E-08	3.78E-07	2.63E-07
**24h**	5.21E-08	1.77E-07	2.59E-06	5.80E-07
**36h**	2.75E-07	2.32E-07	2.60E-06	1.03E-06
**48h**	6.66E-07	3.99E-07	1.74E-06	3.66E-06
**72h**	2.82E-07	4.11E-07	3.24E-06	5.80E-07
	cgd7_3090			
**2h**	5.37E-08	1.19E-09	0.00E+00	1.57E-08
**6h**	0.00E+00	0.00E+00	0.00E+00	0.00E+00
**12h**	1.26E-08	0.00E+00	1.07E-06	3.65E-07
**24h**	1.02E-07	6.52E-08	5.45E-07	3.25E-07
**36h**	2.40E-07	4.71E-07	1.52E-06	2.28E-06
**48h**	7.64E-07	3.78E-07	4.99E-06	4.34E-06
**72h**	2.42E-07	7.53E-07	6.22E-06	7.09E-07
	cgd7_3100			
**2h**	4.49E-06	2.20E-06	2.94E-06	1.54E-06
**6h**	2.77E-06	2.11E-06	1.21E-05	6.16E-06
**12h**	2.16E-07	8.03E-07	3.43E-06	1.56E-06
**24h**	2.17E-06	1.02E-06	3.54E-06	3.44E-06
**36h**	1.62E-06	1.12E-06	1.46E-06	1.69E-06
**48h**	1.35E-06	8.12E-07	4.16E-06	1.43E-06
**72h**	2.26E-06	8.60E-07	7.96E-07	8.55E-07
	cgd7_3110			
**2h**	2.73E-07	7.15E-08	1.34E-06	6.60E-07
**6h**	1.57E-06	1.14E-06	1.97E-05	7.19E-06
**12h**	4.03E-07	1.45E-07	4.34E-06	1.45E-06
**24h**	8.51E-07	4.27E-07	1.09E-05	5.22E-06
**36h**	2.06E-06	2.13E-06	7.30E-06	4.36E-06
**48h**	2.49E-06	9.78E-07	5.18E-06	3.48E-06
**72h**	2.02E-06	2.11E-06	7.28E-06	2.21E-06
	cgd7_3120			
**2h**	7.05E-05	3.80E-05	1.98E-06	1.49E-05
**6h**	2.18E-07	1.99E-07	2.08E-06	0.00E+00
**12h**	4.99E-05	8.21E-05	1.09E-05	9.12E-04
**24h**	5.87E-05	4.65E-05	4.61E-06	1.56E-04
**36h**	5.60E-05	1.25E-04	2.03E-05	2.57E-04
**48h**	7.04E-04	2.13E-03	4.64E-05	7.70E-04
**72h**	2.97E-03	5.16E-03	1.17E-04	5.25E-03
	cgd7_3160			
**2h**	2.10E-06	8.89E-07	3.48E-06	1.02E-06
**6h**	3.04E-07	5.56E-07	4.79E-06	6.98E-07
**12h**	1.12E-07	3.79E-07	7.74E-06	2.36E-06
**24h**	2.70E-07	3.02E-07	5.09E-06	1.03E-06
**36h**	5.50E-07	6.52E-07	4.76E-06	8.12E-07
**48h**	4.83E-07	3.57E-07	6.82E-06	7.49E-07
**72h**	5.48E-07	6.54E-07	3.23E-06	5.08E-07

	cgd7_3180			
**2h**	1.38E-04	7.28E-05	1.38E-04	6.08E-05
**6h**	5.53E-05	8.15E-05	4.35E-04	3.36E-04
**12h**	1.62E-05	1.91E-05	4.82E-04	2.07E-04
**24h**	2.64E-05	3.59E-05	3.91E-04	2.64E-04
**36h**	3.69E-05	4.61E-05	2.07E-04	1.13E-04
**48h**	1.70E-05	1.46E-05	1.55E-04	5.46E-05
**72h**	6.48E-06	5.34E-06	8.79E-05	1.58E-05
	cgd7_3190			
**2h**	4.72E-05	2.34E-05	2.00E-04	8.05E-05
**6h**	4.16E-05	5.01E-05	1.17E-03	3.13E-04
**12h**	1.43E-04	1.23E-04	9.66E-04	5.84E-04
**24h**	4.49E-05	5.91E-05	6.29E-04	2.23E-04
**36h**	1.63E-05	2.45E-05	8.67E-04	1.98E-04
**48h**	3.08E-05	1.73E-05	3.18E-04	9.87E-05
**72h**	4.96E-06	8.12E-06	1.21E-04	1.56E-05
	cgd7_3200			
**2h**	7.00E-04	3.65E-04	1.00E-06	3.17E-04
**6h**	2.79E-04	1.31E-04	6.67E-07	1.20E-04
**12h**	2.48E-04	2.03E-04	3.97E-06	4.66E-04
**24h**	1.19E-04	1.43E-04	4.30E-06	5.51E-05
**36h**	4.54E-04	2.12E-04	4.60E-06	8.90E-05
**48h**	1.58E-04	3.01E-04	7.63E-06	1.21E-04
**72h**	6.19E-04	2.09E-04	4.28E-06	4.77E-05
	cgd7_3220			
**2h**	1.03E-06	1.30E-06	1.34E-05	7.35E-07
**6h**	8.58E-07	4.74E-07	6.92E-06	3.18E-07
**12h**	2.03E-07	4.92E-07	2.02E-06	4.33E-07
**24h**	1.70E-07	1.37E-07	2.91E-06	6.50E-07
**36h**	9.95E-07	5.87E-07	6.59E-06	5.54E-07
**48h**	4.26E-07	1.34E-07	1.92E-06	2.64E-07
**72h**	2.83E-07	2.60E-07	1.37E-06	2.10E-07
	cgd7_3230			
**2h**	1.49E-07	1.83E-07	7.21E-08	7.51E-08
**6h**	2.29E-07	4.80E-07	1.08E-07	1.70E-06
**12h**	1.57E-08	9.00E-09	7.89E-08	6.11E-07
**24h**	1.12E-07	1.09E-07	1.19E-07	3.33E-07
**36h**	8.72E-08	8.13E-08	8.75E-08	1.57E-07
**48h**	1.84E-07	1.80E-07	1.30E-07	1.54E-07
**72h**	1.65E-08	2.81E-08	8.86E-08	4.51E-08
	cgd7_3250			
**2h**	3.89E-06	1.55E-06	1.11E-06	3.36E-06
**6h**	4.70E-05	1.72E-05	2.58E-06	1.65E-05
**12h**	4.19E-06	9.45E-06	4.51E-06	1.88E-05
**24h**	2.10E-05	1.01E-05	4.12E-06	4.18E-05
**36h**	4.19E-06	3.42E-06	2.16E-06	4.48E-06
**48h**	1.06E-05	3.23E-06	3.43E-06	2.27E-05
**72h**	2.88E-06	3.72E-06	2.77E-06	4.96E-05
	cgd7_3270			
**2h**	4.70E-05	1.24E-05	6.13E-06	1.66E-05
**6h**	2.62E-05	2.02E-05	1.10E-05	1.87E-05
**12h**	8.15E-06	5.61E-06	1.83E-05	1.58E-05
**24h**	1.99E-05	1.44E-05	1.11E-05	8.42E-06

**36h**	3.85E-06	5.01E-06	4.47E-06	6.63E-06
**48h**	5.73E-06	1.53E-06	2.34E-06	4.43E-06
**72h**	1.05E-06	7.12E-07	1.07E-06	1.27E-06
	cgd7_3280			
**2h**	1.24E-06	2.78E-07	1.21E-07	5.98E-07
**6h**	4.21E-06	2.71E-06	1.17E-06	2.16E-06
**12h**	1.45E-07	2.15E-07	3.83E-07	1.64E-07
**24h**	4.26E-07	4.51E-07	6.33E-07	5.82E-07
**36h**	2.73E-07	2.70E-07	3.04E-07	3.15E-07
**48h**	2.91E-07	1.31E-07	1.23E-07	5.50E-07
**72h**	4.10E-08	1.15E-07	9.39E-08	6.19E-08
	cgd7_3290			
**2h**	3.00E-04	9.28E-05	7.66E-04	1.22E-04
**6h**	2.25E-04	3.76E-04	2.44E-03	1.33E-04
**12h**	2.57E-04	1.15E-04	2.84E-03	1.90E-04
**24h**	1.58E-04	2.57E-04	7.75E-04	1.13E-04
**36h**	4.11E-05	8.94E-05	7.49E-04	1.03E-04
**48h**	7.61E-05	5.35E-05	2.17E-04	6.02E-05
**72h**	5.39E-05	6.59E-05	2.59E-04	9.69E-06
	cgd7_3320			
**2h**	9.32E-06	8.40E-07	1.83E-06	5.05E-06
**6h**	6.16E-07	1.32E-06	5.87E-08	4.09E-06
**12h**	3.95E-05	5.23E-05	6.00E-06	8.02E-05
**24h**	5.85E-06	1.83E-05	3.38E-06	1.92E-05
**36h**	3.70E-06	3.36E-06	1.11E-05	2.11E-05
**48h**	3.07E-06	4.86E-06	3.53E-06	1.96E-05
**72h**	3.50E-06	3.24E-06	2.30E-06	7.39E-06
	cgd7_3340			
**2h**	0.00E+00	5.11E-09	0.00E+00	0.00E+00
**6h**	1.45E-08	6.96E-09	0.00E+00	0.00E+00
**12h**	1.14E-05	7.07E-06	8.26E-06	1.52E-05
**24h**	9.96E-07	2.12E-06	4.33E-07	1.41E-06
**36h**	5.57E-07	6.15E-07	5.01E-06	8.77E-06
**48h**	3.98E-07	3.62E-07	3.38E-07	1.72E-06
**72h**	1.34E-07	5.40E-07	4.57E-07	9.05E-07
	cgd7_3350			
**2h**	5.72E-04	4.37E-05	0.00E+00	6.89E-05
**6h**	3.37E-04	2.65E-04	5.46E-04	1.57E-05
**12h**	2.27E-04	2.62E-04	8.75E-04	3.11E-05
**24h**	2.09E-04	2.67E-04	9.30E-04	1.19E-04
**36h**	2.03E-04	2.22E-04	1.09E-03	1.08E-04
**48h**	1.04E-04	1.48E-04	2.34E-04	1.61E-05
**72h**	1.27E-04	1.88E-04	5.05E-04	4.61E-05
	cgd7_3360			
**2h**	2.87E-07	2.59E-06	1.29E-07	2.63E-06
**6h**	2.61E-07	3.40E-06	1.07E-06	9.24E-06
**12h**	1.39E-05	0.00E+00	6.39E-07	3.42E-05
**24h**	2.02E-05	3.90E-05	2.06E-05	2.83E-06
**36h**	2.27E-05	1.26E-04	1.33E-05	9.74E-06
**48h**	7.86E-05	5.35E-05	2.61E-05	2.49E-05
**72h**	7.60E-05	1.14E-04	2.13E-06	2.37E-05
	cgd7_3370			
**2h**	9.71E-08	5.67E-09	0.00E+00	6.45E-08

**6h**	0.00E+00	2.79E-09	1.39E-09	4.13E-08
**12h**	1.82E-08	4.83E-09	1.14E-08	5.95E-07
**24h**	1.07E-08	2.48E-08	4.23E-08	4.82E-08
**36h**	1.76E-08	2.51E-08	4.65E-08	8.44E-08
**48h**	5.90E-08	5.48E-08	9.20E-08	1.93E-07
**72h**	7.30E-08	7.91E-08	1.29E-07	7.30E-07
	cgd7_3380			
**2h**	3.86E-08	7.32E-09	0.00E+00	0.00E+00
**6h**	0.00E+00	0.00E+00	0.00E+00	0.00E+00
**12h**	4.43E-07	1.49E-07	2.45E-07	3.79E-07
**24h**	4.38E-08	3.87E-08	3.82E-08	1.81E-07
**36h**	5.65E-08	1.43E-07	2.26E-07	1.27E-06
**48h**	1.39E-06	1.55E-06	8.91E-07	2.64E-06
**72h**	4.52E-07	1.61E-07	5.16E-07	1.37E-06
	cgd7_3390			
**2h**	1.78E-06	1.19E-06	1.85E-07	6.09E-06
**6h**	1.44E-06	7.83E-07	7.53E-07	2.38E-06
**12h**	2.30E-06	4.07E-06	7.36E-06	6.36E-06
**24h**	9.37E-07	1.06E-06	8.64E-07	1.39E-06
**36h**	3.68E-07	6.35E-07	1.50E-06	4.95E-06
**48h**	2.61E-07	2.90E-07	4.90E-07	8.92E-07
**72h**	1.76E-07	2.71E-07	4.29E-07	1.04E-06
	cgd7_3400			
**2h**	4.31E-07	1.23E-07	0.00E+00	4.98E-08
**6h**	2.67E-08	1.50E-08	7.56E-08	4.24E-07
**12h**	1.81E-06	5.71E-07	5.06E-07	1.53E-06
**24h**	1.47E-06	3.60E-07	5.16E-07	6.87E-07
**36h**	2.81E-06	2.36E-07	9.37E-07	1.86E-06
**48h**	2.37E-06	1.14E-06	7.13E-07	1.27E-06
**72h**	2.49E-06	5.00E-07	8.81E-07	7.71E-07
	cgd7_3420			
**2h**	1.74E-04	1.93E-05	9.33E-05	5.32E-04
**6h**	1.47E-05	8.33E-05	9.90E-05	8.41E-05
**12h**	5.29E-06	3.41E-05	7.44E-05	1.98E-04
**24h**	2.81E-04	6.11E-05	2.08E-05	8.00E-05
**36h**	7.73E-06	1.94E-05	2.80E-05	1.22E-04
**48h**	3.57E-05	2.61E-05	6.53E-05	2.67E-04
**72h**	2.84E-05	2.50E-05	3.42E-05	1.48E-04
	cgd7_3450			
**2h**	5.35E-05	1.04E-03	3.91E-04	1.58E-03
**6h**	2.29E-04	1.67E-03	8.17E-04	2.71E-03
**12h**	6.63E-06	7.17E-05	4.66E-04	1.92E-04
**24h**	2.57E-05	4.89E-04	3.92E-04	6.75E-04
**36h**	3.81E-05	1.04E-04	1.06E-04	1.02E-03
**48h**	6.49E-05	1.48E-04	2.21E-04	6.03E-05
**72h**	2.19E-05	1.70E-04	1.05E-04	1.39E-04
	cgd7_3460			
**2h**	1.49E-06	9.30E-08	3.73E-08	3.68E-07
**6h**	9.50E-07	6.35E-07	4.21E-07	5.70E-07
**12h**	5.26E-08	5.54E-08	1.76E-07	2.45E-07
**24h**	3.52E-07	1.33E-07	3.49E-07	2.68E-07
**36h**	4.21E-07	9.66E-08	2.91E-07	7.57E-07
**48h**	9.56E-07	1.41E-07	1.60E-07	2.64E-07

**72h**	2.72E-07	9.32E-08	8.89E-08	2.13E-07
	cgd7_3470			
**2h**	1.03E-05	8.21E-05	0.00E+00	1.12E-05
**6h**	4.21E-06	1.10E-04	3.63E-05	6.15E-05
**12h**	8.37E-06	2.73E-05	1.69E-05	1.33E-04
**24h**	5.54E-06	6.85E-05	2.78E-05	1.71E-05
**36h**	1.01E-05	4.92E-05	2.30E-05	5.61E-05
**48h**	1.38E-05	2.74E-05	2.81E-05	9.81E-04
**72h**	5.09E-06	2.11E-05	9.79E-06	6.50E-04
	cgd7_3480			
**2h**	2.80E-06	7.21E-07	2.57E-06	6.26E-06
**6h**	1.82E-06	4.49E-06	3.62E-06	5.27E-06
**12h**	7.76E-07	2.51E-07	2.67E-06	1.39E-06
**24h**	4.17E-06	3.37E-06	2.94E-06	2.12E-06
**36h**	1.34E-06	6.31E-07	7.50E-07	2.16E-06
**48h**	2.61E-06	6.93E-07	8.48E-07	1.49E-06
**72h**	2.44E-06	2.44E-07	2.78E-07	2.80E-07
	cgd7_3490			
**2h**	1.44E-04	3.54E-05	3.93E-05	1.29E-04
**6h**	1.87E-05	3.53E-05	1.94E-05	4.94E-05
**12h**	2.37E-05	1.30E-05	4.55E-05	4.16E-05
**24h**	8.47E-05	4.26E-05	1.21E-05	1.34E-05
**36h**	3.97E-05	8.41E-06	6.85E-06	2.26E-05
**48h**	6.32E-05	1.52E-05	1.17E-05	2.30E-05
**72h**	5.90E-05	6.37E-06	2.01E-04	3.25E-06
	cgd7_3510			
**2h**	7.58E-05	8.95E-06	9.89E-06	5.43E-05
**6h**	1.81E-05	4.03E-05	1.10E-05	2.28E-05
**12h**	5.58E-05	1.04E-05	1.57E-05	1.83E-05
**24h**	4.57E-05	1.62E-05	1.18E-05	1.30E-05
**36h**	7.76E-05	1.13E-05	1.43E-05	2.86E-05
**48h**	1.58E-04	1.86E-05	2.38E-05	4.94E-05
**72h**	1.02E-04	1.08E-05	9.70E-06	1.17E-05
	cgd7_3540			
**2h**	6.53E-04	3.37E-04	6.59E-05	2.67E-04
**6h**	2.70E-04	1.03E-04	5.98E-05	9.43E-05
**12h**	7.33E-05	3.54E-05	9.87E-05	7.73E-05
**24h**	1.62E-04	4.81E-05	6.98E-05	6.40E-05
**36h**	1.24E-04	2.13E-05	2.20E-05	7.29E-05
**48h**	1.20E-04	3.09E-05	2.44E-05	6.18E-05
**72h**	2.18E-04	2.95E-05	7.79E-06	1.64E-05
	cgd7_3560			
**2h**	9.94E-08	4.40E-08	3.26E-08	2.25E-07
**6h**	6.56E-08	1.33E-07	2.15E-08	3.85E-08
**12h**	1.08E-07	6.02E-09	4.94E-08	2.88E-08
**24h**	1.21E-07	2.13E-07	6.32E-08	6.31E-08
**36h**	1.02E-07	3.99E-08	7.60E-08	2.60E-07
**48h**	5.51E-07	5.28E-08	9.02E-08	1.41E-07
**72h**	8.65E-08	2.47E-08	1.96E-08	3.07E-08
	cgd7_3580			
**2h**	2.11E-05	2.51E-05	1.19E-05	2.44E-05
**6h**	3.91E-06	2.93E-06	9.68E-07	1.77E-06
**12h**	1.76E-06	2.55E-06	3.87E-06	3.55E-06

**24h**	1.04E-05	4.24E-06	1.70E-06	1.73E-06
**36h**	7.34E-06	3.20E-06	1.91E-06	3.03E-06
**48h**	4.29E-06	1.02E-06	7.61E-07	2.30E-06
**72h**	3.17E-06	9.82E-07	8.01E-07	1.44E-06
	cgd7_3780			
**2h**	4.27E-05	1.28E-04	4.14E-05	9.06E-05
**6h**	5.47E-05	4.01E-05	5.47E-05	5.27E-05
**12h**	4.83E-05	6.29E-05	1.20E-04	6.73E-05
**24h**	3.53E-05	5.90E-05	4.96E-05	3.98E-05
**36h**	1.87E-05	2.21E-05	3.55E-05	2.93E-05
**48h**	1.06E-05	6.67E-06	1.05E-05	1.81E-05
**72h**	6.35E-06	7.35E-06	7.36E-06	1.37E-05
	cgd7_3790			
**2h**	9.95E-08	7.38E-08	8.82E-08	2.74E-07
**6h**	1.87E-08	2.99E-09	1.78E-08	5.86E-09
**12h**	3.24E-04	3.41E-04	3.19E-04	1.58E-04
**24h**	3.94E-05	4.13E-05	2.79E-05	1.09E-05
**36h**	7.66E-06	1.02E-05	7.22E-05	4.25E-05
**48h**	2.95E-06	2.02E-06	5.70E-06	2.66E-06
**72h**	1.03E-06	9.03E-07	9.32E-07	1.33E-06
	cgd7_3800			
**2h**	2.41E-07	1.44E-07	2.97E-07	1.45E-06
**6h**	3.80E-06	2.31E-06	1.32E-06	1.35E-06
**12h**	2.63E-06	4.26E-06	5.20E-06	1.42E-06
**24h**	1.07E-06	1.56E-06	9.29E-07	8.25E-07
**36h**	2.70E-07	3.41E-07	9.60E-07	5.96E-07
**48h**	4.30E-07	2.45E-07	5.10E-07	3.25E-07
**72h**	1.00E-07	8.79E-08	1.02E-07	1.02E-07
	cgd7_3810			
**2h**	2.25E-05	1.73E-04	1.97E-05	1.05E-04
**6h**	3.14E-05	1.70E-04	1.89E-04	6.55E-05
**12h**	3.31E-04	4.43E-04	5.34E-04	3.22E-04
**24h**	1.23E-04	2.58E-04	3.38E-04	1.17E-04
**36h**	2.70E-04	2.67E-04	4.79E-04	2.50E-04
**48h**	2.33E-04	1.23E-04	4.75E-04	8.27E-05
**72h**	1.67E-04	1.62E-04	7.21E-05	3.66E-05
	cgd7_3820			
**2h**	2.16E-09	0.00E+00	0.00E+00	0.00E+00
**6h**	0.00E+00	3.11E-09	0.00E+00	0.00E+00
**12h**	1.40E-07	2.90E-07	1.41E-07	1.05E-07
**24h**	4.22E-08	3.04E-08	4.30E-08	3.82E-08
**36h**	9.76E-08	8.47E-08	3.37E-07	2.01E-07
**48h**	1.21E-07	7.37E-08	1.23E-07	8.08E-08
**72h**	7.55E-08	1.21E-07	7.95E-08	5.17E-08
	cgd7_3830			
**2h**	1.75E-09	1.55E-08	4.85E-09	4.10E-08
**6h**	2.04E-07	1.03E-07	3.34E-08	1.60E-07
**12h**	9.92E-06	1.48E-05	9.06E-06	4.59E-06
**24h**	6.78E-07	1.02E-06	2.58E-07	2.98E-07
**36h**	2.24E-07	3.51E-07	1.47E-06	9.39E-07
**48h**	1.15E-07	1.29E-07	2.39E-07	2.00E-07
**72h**	5.98E-07	1.64E-07	1.71E-07	1.10E-07
	cgd7_3840			

**2h**	4.80E-07	4.06E-07	3.64E-07	9.54E-07
**6h**	1.06E-06	4.18E-07	5.34E-07	1.56E-06
**12h**	8.46E-06	6.21E-06	1.07E-05	1.71E-05
**24h**	1.09E-06	9.12E-07	7.82E-07	2.05E-06
**36h**	2.51E-07	3.70E-07	2.41E-06	1.19E-05
**48h**	3.78E-07	2.88E-07	1.45E-06	5.52E-06
**72h**	1.51E-07	3.36E-07	1.19E-06	6.83E-05
	cgd7_3850			
**2h**	1.94E-09	3.51E-09	1.74E-08	0.00E+00
**6h**	0.00E+00	0.00E+00	0.00E+00	0.00E+00
**12h**	1.71E-06	1.42E-06	1.42E-06	1.00E-06
**24h**	2.94E-07	2.77E-07	1.92E-07	1.76E-07
**36h**	1.44E-07	2.01E-07	7.59E-07	3.30E-07
**48h**	3.89E-08	3.19E-08	1.12E-07	9.38E-08
**72h**	3.70E-08	3.84E-08	1.99E-08	1.19E-08
	cgd7_3860			
**2h**	0.00E+00	0.00E+00	0.00E+00	0.00E+00
**6h**	0.00E+00	0.00E+00	0.00E+00	0.00E+00
**12h**	1.12E-06	9.75E-07	2.06E-06	8.62E-07
**24h**	3.02E-07	1.19E-06	5.32E-07	3.73E-07
**36h**	4.63E-07	6.58E-07	1.94E-06	1.28E-06
**48h**	4.62E-07	2.56E-07	6.31E-07	5.34E-07
**72h**	3.09E-07	1.96E-07	7.73E-07	6.03E-07
	cgd7_3870			
**2h**	0.00E+00	0.00E+00	0.00E+00	0.00E+00
**6h**	0.00E+00	0.00E+00	0.00E+00	0.00E+00
**12h**	1.41E-03	9.98E-04	2.00E-03	8.52E-05
**24h**	3.17E-04	1.30E-04	3.61E-04	2.62E-04
**36h**	2.81E-04	2.22E-04	8.64E-04	3.29E-04
**48h**	2.40E-04	2.95E-04	3.73E-04	3.72E-04
**72h**	2.02E-05	5.85E-05	9.62E-05	5.37E-04
	cgd7_3880			
**2h**	2.57E-04	2.48E-04	0.00E+00	6.43E-04
**6h**	3.91E-03	7.32E-03	8.70E-03	3.95E-03
**12h**	3.83E-04	9.46E-04	1.44E-03	1.87E-03
**24h**	5.83E-04	3.33E-04	6.96E-04	6.27E-04
**36h**	5.78E-04	5.74E-04	9.42E-04	2.57E-04
**48h**	1.98E-04	2.77E-04	4.06E-04	5.01E-04
**72h**	1.35E-04	3.98E-04	1.76E-04	6.64E-05
	cgd7_3900			
**2h**	7.39E-07	3.61E-07	3.43E-07	7.93E-07
**6h**	1.09E-06	1.49E-06	5.65E-07	8.69E-07
**12h**	1.03E-07	1.93E-07	3.14E-07	1.05E-07
**24h**	7.91E-08	1.08E-07	2.62E-07	7.79E-07
**36h**	3.38E-07	5.99E-07	1.06E-06	3.01E-07
**48h**	1.56E-07	1.40E-07	2.65E-07	2.39E-07
**72h**	1.33E-07	2.45E-07	2.49E-07	2.52E-07
	cgd7_3590			
**2h**	1.09E-05	6.03E-06	1.64E-05	4.21E-05
**6h**	1.91E-06	1.26E-06	5.88E-06	5.73E-06
**12h**	3.25E-06	2.28E-06	2.10E-05	5.15E-06
**24h**	6.57E-06	4.44E-06	5.71E-06	3.72E-06
**36h**	2.17E-06	1.68E-06	2.61E-06	3.15E-06

**48h**	1.21E-06	2.13E-06	2.09E-06	2.65E-06
**72h**	9.39E-07	1.07E-06	1.76E-06	1.02E-06
	cgd7_3600			
**2h**	6.64E-08	2.91E-08	0.00E+00	4.70E-09
**6h**	2.02E-06	5.78E-07	7.19E-07	1.02E-06
**12h**	1.42E-08	0.00E+00	1.35E-08	7.01E-08
**24h**	4.57E-07	8.88E-08	9.28E-07	3.99E-07
**36h**	1.32E-07	1.96E-07	2.58E-07	3.41E-07
**48h**	2.47E-07	1.19E-07	1.79E-07	2.24E-07
**72h**	5.27E-08	3.51E-08	1.70E-07	8.70E-08
	cgd7_3620			
**2h**	3.47E-06	6.00E-07	1.40E-05	2.89E-05
**6h**	1.43E-05	1.04E-05	6.22E-06	2.39E-05
**12h**	1.60E-06	1.77E-06	1.64E-05	4.49E-06
**24h**	7.74E-06	1.06E-06	1.66E-05	6.61E-06
**36h**	6.05E-06	2.63E-06	6.04E-06	1.65E-05
**48h**	1.40E-05	2.49E-06	2.13E-05	1.35E-05
**72h**	4.83E-06	6.61E-07	1.11E-05	8.23E-06
	cgd7_3630			
**2h**	1.74E-06	3.23E-07	2.26E-06	1.32E-05
**6h**	1.44E-06	9.45E-08	1.25E-06	6.92E-07
**12h**	1.73E-06	1.46E-06	8.44E-06	2.98E-06
**24h**	1.64E-06	4.85E-07	5.24E-06	1.39E-06
**36h**	2.00E-06	7.44E-07	6.38E-06	3.39E-06
**48h**	1.70E-06	6.41E-07	1.91E-05	4.35E-06
**72h**	1.11E-06	1.25E-06	3.41E-06	2.25E-06
	cgd7_3640			
**2h**	1.21E-07	8.09E-08	2.56E-07	1.21E-07
**6h**	9.56E-08	9.21E-08	2.21E-07	1.34E-07
**12h**	1.69E-07	1.22E-07	4.25E-07	2.05E-07
**24h**	8.08E-08	1.20E-07	2.02E-07	2.10E-07
**36h**	1.38E-07	1.36E-07	1.44E-07	2.99E-07
**48h**	8.86E-08	6.60E-08	1.75E-07	1.83E-07
**72h**	1.20E-07	5.82E-08	6.03E-08	1.35E-07
	cgd7_3650			
**2h**	5.48E-06	2.25E-06	1.17E-05	1.75E-05
**6h**	1.36E-06	2.94E-07	1.32E-06	1.61E-06
**12h**	1.55E-05	2.15E-06	1.57E-05	4.59E-06
**24h**	4.83E-06	1.53E-06	8.16E-06	5.46E-06
**36h**	3.86E-06	2.38E-06	2.03E-06	4.09E-06
**48h**	1.92E-06	1.21E-06	5.75E-06	2.49E-06
**72h**	1.09E-06	8.03E-07	2.61E-06	1.55E-06
	cgd7_3660			
**2h**	1.65E-07	5.25E-08	4.90E-06	3.51E-06
**6h**	4.45E-04	3.88E-05	3.50E-05	3.58E-04
**12h**	1.50E-05	1.94E-06	3.23E-05	4.89E-06
**24h**	2.00E-05	6.34E-06	4.06E-05	8.06E-05
**36h**	8.65E-05	1.90E-05	2.78E-05	6.44E-05
**48h**	5.53E-05	9.78E-06	1.01E-04	9.14E-05
**72h**	2.50E-05	9.59E-06	1.70E-05	2.24E-05
	cgd7_3670			
**2h**	0.00E+00	8.37E-08	0.00E+00	6.99E-05
**6h**	1.39E-02	3.94E-03	6.31E-04	3.43E-02

**12h**	1.36E-03	8.36E-04	1.58E-04	5.69E-03
**24h**	1.33E-03	6.41E-04	5.20E-04	1.22E-02
**36h**	3.86E-03	1.50E-03	2.06E-04	8.67E-03
**48h**	2.14E-03	7.79E-04	1.52E-04	6.09E-03
**72h**	3.62E-04	8.84E-05	5.45E-05	2.10E-04
	cgd7_3680			
**2h**	1.53E-08	0.00E+00	0.00E+00	0.00E+00
**6h**	1.52E-07	4.10E-07	1.78E-06	6.33E-07
**12h**	1.37E-07	9.83E-08	2.49E-06	2.60E-07
**24h**	5.64E-07	3.01E-07	3.77E-06	1.53E-06
**36h**	8.59E-07	1.67E-06	9.37E-06	3.11E-06
**48h**	2.71E-06	1.32E-06	4.91E-06	3.97E-06
**72h**	1.76E-06	1.12E-06	3.30E-06	1.85E-06
	cgd7_3710			
**2h**	0.00E+00	2.87E-07	0.00E+00	7.80E-05
**6h**	1.24E-04	2.43E-05	1.10E-05	4.48E-04
**12h**	0.00E+00	2.24E-07	6.43E-06	9.37E-06
**24h**	3.65E-05	3.53E-06	2.70E-06	6.75E-04
**36h**	1.82E-05	3.58E-05	1.61E-06	2.23E-04
**48h**	2.82E-05	1.29E-05	1.80E-06	3.58E-04
**72h**	2.68E-05	5.06E-05	4.30E-06	2.82E-04
	cgd7_3730			
**2h**	5.54E-06	4.94E-07	8.73E-06	1.04E-06
**6h**	5.75E-07	3.54E-07	1.46E-06	5.98E-07
**12h**	2.17E-08	4.80E-08	9.11E-07	3.01E-08
**24h**	7.87E-08	2.91E-08	9.11E-07	3.82E-07
**36h**	9.87E-08	2.83E-07	1.61E-06	4.46E-07
**48h**	3.45E-07	2.70E-07	2.43E-05	1.08E-06
**72h**	2.29E-07	1.40E-07	7.05E-07	1.45E-07
	cgd7_3750			
**2h**	1.46E-04	5.59E-06	4.33E-06	8.90E-05
**6h**	7.14E-05	3.18E-05	8.02E-05	9.17E-05
**12h**	1.26E-05	1.37E-06	1.72E-06	4.67E-06
**24h**	6.71E-05	3.81E-06	2.87E-05	3.91E-05
**36h**	4.13E-05	2.13E-05	7.28E-06	4.49E-05
**48h**	4.74E-05	5.84E-06	1.45E-05	4.84E-05
**72h**	1.20E-05	4.43E-06	3.89E-06	2.98E-05
	cgd7_3760			
**2h**	1.95E-07	1.04E-07	1.04E-07	2.27E-07
**6h**	4.29E-09	8.81E-08	7.13E-09	2.48E-08
**12h**	2.56E-09	1.79E-08	4.23E-08	2.16E-08
**24h**	2.86E-08	1.46E-08	7.07E-08	2.10E-08
**36h**	5.26E-08	6.59E-08	1.05E-07	1.40E-07
**48h**	4.27E-07	1.50E-07	4.24E-07	3.57E-07
**72h**	2.46E-07	1.46E-07	7.06E-07	3.74E-07
	cgd7_3770			
**2h**	1.69E-06	7.62E-06	5.02E-07	9.17E-06
**6h**	5.93E-06	4.79E-05	6.79E-06	1.02E-05
**12h**	1.36E-06	3.70E-06	1.02E-06	8.96E-07
**24h**	1.96E-06	6.63E-06	8.89E-06	5.84E-06
**36h**	2.52E-06	7.78E-06	2.15E-06	3.11E-06
**48h**	1.39E-05	2.65E-05	2.26E-05	1.63E-05
**72h**	5.80E-06	1.53E-05	7.50E-06	9.74E-06

	cgd8_4800			
**2h**	3.52E-03	3.77E-06	0.00E+00	4.32E-04
**6h**	1.01E-03	1.00E-05	8.99E-04	9.16E-05
**12h**	2.23E-04	5.40E-05	6.29E-05	2.36E-03
**24h**	2.19E-04	3.99E-06	3.68E-04	8.32E-04
**36h**	1.14E-04	5.26E-05	3.43E-04	1.88E-05
**48h**	1.69E-04	5.05E-06	5.56E-04	1.90E-04
**72h**	2.85E-05	1.02E-05	6.86E-04	1.05E-03
	cgd8_4810			
**2h**	2.36E-08	4.81E-07	9.19E-09	3.17E-07
**6h**	1.58E-07	3.32E-06	1.04E-06	5.21E-07
**12h**	1.38E-07	7.47E-07	6.77E-07	6.56E-07
**24h**	1.57E-07	1.50E-06	1.04E-06	6.98E-07
**36h**	3.77E-07	5.33E-06	1.15E-06	8.75E-07
**48h**	4.59E-07	4.65E-06	3.93E-07	6.89E-07
**72h**	2.84E-07	1.84E-06	3.64E-07	1.39E-06
	cgd8_4830			
**2h**	0.00E+00	0.00E+00	0.00E+00	0.00E+00
**6h**	0.00E+00	0.00E+00	3.07E-09	2.36E-11
**12h**	4.35E-09	2.24E-08	1.82E-08	3.03E-08
**24h**	3.20E-08	2.68E-08	3.86E-08	4.19E-08
**36h**	1.70E-06	3.25E-06	1.15E-06	1.75E-06
**48h**	8.15E-05	1.89E-04	1.42E-04	1.03E-04
**72h**	1.19E-05	2.94E-05	7.21E-06	6.61E-06
	cgd8_4870			
**2h**	5.29E-07	4.73E-07	4.12E-07	4.58E-07
**6h**	7.88E-08	1.57E-07	1.60E-08	1.16E-07
**12h**	1.48E-07	1.18E-06	1.62E-07	3.32E-07
**24h**	5.35E-07	7.58E-07	7.56E-08	1.46E-07
**36h**	2.55E-07	3.72E-07	1.53E-07	2.69E-07
**48h**	9.38E-08	2.13E-07	1.24E-07	1.27E-07
**72h**	1.51E-07	5.91E-08	8.55E-08	4.00E-08
	cgd8_4880			
**2h**	2.53E-04	1.17E-03	1.14E-04	1.89E-04
**6h**	1.65E-05	3.52E-04	3.66E-05	4.08E-05
**12h**	1.18E-04	2.36E-03	1.36E-04	1.26E-04
**24h**	1.07E-04	4.44E-04	6.95E-05	5.83E-05
**36h**	8.27E-05	4.38E-04	7.41E-05	1.03E-04
**48h**	4.55E-05	2.49E-04	7.08E-05	1.06E-04
**72h**	2.52E-05	1.11E-04	2.71E-05	3.25E-05
	cgd8_4910			
**2h**	1.47E-07	1.93E-08	1.28E-08	2.41E-08
**6h**	1.52E-06	4.50E-06	2.00E-06	4.05E-06
**12h**	1.14E-07	6.63E-07	2.00E-06	7.53E-07
**24h**	5.86E-07	1.02E-06	2.15E-06	1.35E-06
**36h**	8.04E-07	9.40E-07	1.37E-06	2.72E-06
**48h**	1.83E-06	6.42E-07	1.25E-06	1.16E-06
**72h**	5.29E-07	6.56E-07	4.75E-07	4.86E-07
	cgd8_4890			
**2h**	0.00E+00	0.00E+00	0.00E+00	0.00E+00
**6h**	6.28E-08	5.23E-08	3.44E-08	9.74E-07
**12h**	5.30E-10	3.13E-09	8.56E-09	1.47E-08
**24h**	1.34E-08	2.32E-08	3.46E-08	1.45E-07

**36h**	2.02E-08	4.93E-08	8.24E-08	3.23E-07
**48h**	3.38E-08	5.80E-08	5.78E-08	1.55E-07
**72h**	1.79E-08	2.03E-08	1.68E-08	4.26E-07
	cgd8_4950			
**2h**	1.36E-08	0.00E+00	1.58E-08	0.00E+00
**6h**	5.04E-10	0.00E+00	9.45E-10	0.00E+00
**12h**	2.89E-09	1.43E-08	4.73E-08	1.02E-07
**24h**	6.55E-09	6.18E-08	1.57E-08	3.08E-07
**36h**	9.94E-08	5.17E-08	1.31E-07	3.35E-07
**48h**	6.21E-08	2.11E-07	2.42E-07	1.93E-06
**72h**	3.75E-07	4.54E-07	4.23E-07	4.28E-06
	cgd8_4980			
**2h**	1.39E-04	4.06E-08	8.42E-07	6.70E-08
**6h**	4.48E-04	1.99E-06	7.97E-06	4.40E-07
**12h**	1.69E-11	0.00E+00	1.08E-06	3.64E-07
**24h**	5.55E-05	3.44E-06	3.82E-06	1.92E-09
**36h**	4.81E-06	2.79E-06	1.32E-06	8.89E-07
**48h**	9.05E-07	3.31E-07	1.45E-06	1.86E-06
**72h**	4.14E-04	1.82E-06	1.81E-06	8.07E-07
	cgd8_4990			
**2h**	1.11E-05	1.10E-05	0.00E+00	4.84E-06
**6h**	8.93E-05	8.84E-05	5.90E-05	1.30E-05
**12h**	5.07E-05	4.60E-05	7.08E-06	1.22E-06
**24h**	1.55E-04	7.54E-06	2.29E-05	5.56E-06
**36h**	3.14E-04	6.75E-05	2.80E-05	2.09E-06
**48h**	6.74E-05	1.10E-05	1.07E-05	1.25E-05
**72h**	3.41E-05	4.57E-06	9.84E-06	6.64E-07
	cgd8_5000			
**2h**	8.09E-06	3.17E-06	5.09E-07	5.88E-06
**6h**	1.87E-07	3.57E-07	4.73E-07	9.89E-07
**12h**	3.63E-07	4.96E-07	2.25E-06	9.06E-07
**24h**	1.63E-06	8.99E-07	8.82E-07	5.64E-07
**36h**	4.24E-07	2.83E-07	3.83E-07	5.10E-07
**48h**	2.67E-07	1.29E-07	3.29E-07	5.72E-07
**72h**	2.57E-07	1.81E-07	2.15E-07	5.41E-07
	cgd8_5020			
**2h**	6.18E-08	1.05E-07	0.00E+00	6.66E-07
**6h**	9.91E-08	2.51E-06	4.10E-06	6.06E-06
**12h**	1.15E-07	1.10E-06	7.62E-07	4.07E-06
**24h**	8.11E-08	2.62E-07	8.26E-07	2.53E-06
**36h**	2.62E-07	1.35E-06	1.93E-06	7.45E-06
**48h**	2.12E-07	9.96E-07	1.28E-06	1.13E-05
**72h**	2.92E-07	3.66E-07	4.66E-07	1.06E-06
	cgd8_5030			
**2h**	0.00E+00	0.00E+00	0.00E+00	4.77E-06
**6h**	0.00E+00	0.00E+00	2.40E-05	2.26E-06
**12h**	1.08E-02	1.62E-02	7.09E-04	3.25E-04
**24h**	1.44E-03	9.28E-04	2.43E-04	1.09E-05
**36h**	2.79E-03	7.71E-04	6.58E-04	4.41E-04
**48h**	1.06E-03	1.72E-04	2.72E-04	6.78E-06
**72h**	3.99E-04	6.96E-05	1.45E-04	3.44E-05
	cgd8_5050			
**2h**	0.00E+00	0.00E+00	0.00E+00	0.00E+00

**6h**	0.00E+00	0.00E+00	0.00E+00	0.00E+00
**12h**	2.89E-10	3.12E-09	0.00E+00	2.61E-09
**24h**	4.61E-09	8.37E-09	5.02E-09	2.78E-09
**36h**	1.41E-08	1.00E-08	1.41E-08	3.50E-08
**48h**	2.49E-07	1.67E-07	5.79E-07	1.07E-06
**72h**	5.12E-07	2.29E-07	3.04E-07	1.77E-06
	cgd8_5080			
**2h**	2.25E-04	3.90E-06	5.74E-07	4.61E-08
**6h**	0.00E+00	0.00E+00	0.00E+00	3.18E-07
**12h**	1.09E-05	3.80E-07	2.26E-07	3.42E-07
**24h**	3.24E-06	3.42E-08	7.78E-08	1.07E-08
**36h**	1.18E-05	4.51E-08	7.51E-08	5.34E-07
**48h**	9.51E-06	9.83E-07	1.17E-06	7.35E-06
**72h**	1.61E-04	5.30E-06	1.96E-05	6.52E-05
	cgd8_5090			
**2h**	0.00E+00	0.00E+00	1.30E-07	0.00E+00
**6h**	0.00E+00	0.00E+00	0.00E+00	1.75E-06
**12h**	0.00E+00	0.00E+00	0.00E+00	1.45E-05
**24h**	4.12E-08	2.99E-07	4.23E-08	1.73E-06
**36h**	6.54E-08	1.18E-06	6.96E-07	7.91E-06
**48h**	1.04E-05	3.07E-05	9.94E-05	1.24E-04
**72h**	8.40E-04	1.21E-04	3.45E-04	4.35E-05
	cgd8_5120			
**2h**	1.92E-06	5.52E-05	2.95E-05	2.02E-07
**6h**	2.38E-05	8.41E-04	1.13E-03	5.16E-06
**12h**	1.82E-06	1.21E-04	1.43E-04	7.70E-06
**24h**	4.08E-06	2.18E-04	7.04E-05	2.87E-05
**36h**	2.28E-05	2.47E-04	1.49E-04	5.26E-06
**48h**	1.99E-05	1.64E-04	1.91E-04	1.66E-05
**72h**	1.48E-05	7.57E-05	7.83E-04	2.59E-06
	cgd8_5130			
**2h**	7.42E-07	7.83E-07	1.76E-06	1.02E-04
**6h**	3.44E-07	1.54E-06	6.86E-07	7.63E-05
**12h**	3.04E-07	7.01E-07	4.63E-07	3.78E-05
**24h**	2.77E-07	4.19E-07	2.49E-07	5.81E-06
**36h**	2.01E-07	5.28E-07	2.05E-07	2.30E-05
**48h**	2.89E-07	4.82E-07	5.48E-07	1.69E-05
**72h**	2.73E-07	2.25E-07	4.71E-07	5.01E-06
	cgd8_5140			
**2h**	6.16E-09	2.34E-07	0.00E+00	1.59E-06
**6h**	1.49E-07	2.62E-07	7.38E-07	4.43E-06
**12h**	1.97E-08	8.01E-08	4.76E-08	1.29E-06
**24h**	3.79E-08	2.46E-08	2.19E-07	3.11E-06
**36h**	3.62E-07	3.74E-07	1.63E-07	1.74E-05
**48h**	1.77E-07	1.01E-07	8.36E-08	3.53E-06
**72h**	1.05E-07	9.13E-08	6.89E-08	3.24E-06
	cgd8_5150			
**2h**	1.00E-06	5.08E-07	4.06E-07	6.11E-07
**6h**	6.89E-07	4.02E-07	4.30E-07	7.72E-06
**12h**	4.24E-08	2.29E-08	2.47E-07	1.76E-07
**24h**	3.17E-07	6.27E-08	4.76E-07	2.50E-07
**36h**	1.85E-07	1.08E-07	2.95E-07	3.67E-07
**48h**	3.97E-07	1.70E-07	5.40E-07	2.38E-07

**72h**	9.11E-08	3.77E-08	7.69E-08	1.38E-07
	cgd8_5160			
**2h**	5.36E-07	3.58E-07	1.55E-06	6.81E-07
**6h**	6.28E-07	4.24E-07	4.56E-07	2.71E-07
**12h**	1.73E-05	7.46E-06	9.41E-06	7.26E-06
**24h**	5.50E-06	2.02E-06	2.04E-06	1.42E-06
**36h**	2.31E-06	9.10E-07	3.65E-06	4.86E-06
**48h**	1.32E-06	8.57E-07	3.45E-06	2.89E-06
**72h**	1.57E-06	3.81E-07	1.30E-06	1.52E-06
	cgd8_5190			
**2h**	2.20E-06	2.71E-07	1.28E-06	7.07E-07
**6h**	0.00E+00	5.67E-10	2.60E-09	7.46E-10
**12h**	4.24E-08	5.60E-08	3.05E-07	9.16E-08
**24h**	3.01E-07	4.88E-08	1.28E-07	9.63E-08
**36h**	4.15E-07	1.29E-07	6.50E-07	2.93E-07
**48h**	5.98E-07	2.63E-07	7.70E-07	4.22E-07
**72h**	5.79E-07	4.25E-07	6.24E-07	1.84E-07
	cgd8_5200			
**2h**	3.12E-04	9.96E-06	4.04E-04	2.79E-05
**6h**	1.79E-04	2.29E-05	2.03E-04	2.47E-05
**12h**	1.36E-04	1.25E-05	3.20E-04	2.31E-05
**24h**	1.48E-04	2.64E-05	1.78E-04	4.52E-05
**36h**	9.04E-05	1.52E-05	1.98E-04	2.76E-05
**48h**	5.11E-05	2.52E-05	1.20E-04	3.79E-05
**72h**	4.07E-05	6.00E-06	7.03E-05	5.30E-06
	cgd8_5210			
**2h**	0.00E+00	0.00E+00	0.00E+00	1.33E-04
**6h**	5.70E-06	6.35E-05	1.51E-05	4.11E-05
**12h**	1.44E-05	4.03E-04	1.67E-06	5.01E-03
**24h**	3.93E-05	4.46E-05	2.14E-07	9.67E-04
**36h**	1.45E-05	6.26E-05	6.87E-05	4.25E-06
**48h**	4.35E-05	1.50E-04	1.08E-05	3.02E-06
**72h**	6.40E-05	1.49E-04	1.42E-05	7.75E-03
	cgd8_5220			
**2h**	0.00E+00	2.92E-06	0.00E+00	5.21E-06
**6h**	5.61E-05	1.57E-03	2.69E-04	3.90E-03
**12h**	1.69E-05	9.46E-06	1.75E-05	6.44E-05
**24h**	6.87E-05	2.56E-04	1.47E-04	1.27E-03
**36h**	1.12E-04	9.08E-04	1.51E-04	1.81E-03
**48h**	9.20E-05	9.85E-04	3.06E-04	1.72E-03
**72h**	3.87E-05	4.28E-04	1.75E-04	1.62E-03
	cgd8_5230			
**2h**	3.01E-04	9.03E-06	1.94E-05	2.77E-05
**6h**	0.00E+00	1.29E-03	8.14E-03	1.53E-03
**12h**	1.75E-02	7.53E-03	1.16E-02	2.92E-03
**24h**	3.93E-03	8.27E-04	7.16E-03	1.39E-03
**36h**	9.24E-03	2.22E-03	1.85E-02	6.94E-03
**48h**	1.97E-02	6.62E-03	4.35E-02	1.36E-02
**72h**	1.04E-02	1.77E-03	2.39E-02	4.11E-03
	cgd8_5240			
**2h**	2.14E-03	3.02E-04	7.61E-04	9.07E-04
**6h**	6.47E-04	5.79E-04	2.42E-03	1.47E-03
**12h**	3.83E-05	3.03E-05	9.58E-05	1.54E-04

**24h**	3.89E-04	1.82E-04	2.68E-04	4.10E-04
**36h**	8.24E-04	1.15E-04	9.89E-04	4.25E-04
**48h**	1.59E-04	7.11E-05	2.43E-04	2.35E-04
**72h**	4.21E-05	9.00E-05	1.65E-03	1.96E-04
	cgd8_5250			
**2h**	1.76E-07	7.53E-09	0.00E+00	0.00E+00
**6h**	1.24E-06	2.16E-07	5.00E-07	6.10E-07
**12h**	1.37E-08	1.41E-08	2.45E-07	4.41E-08
**24h**	1.98E-07	1.42E-07	7.37E-07	3.15E-07
**36h**	4.77E-07	2.70E-07	6.85E-07	3.14E-07
**48h**	6.73E-07	4.80E-07	1.33E-06	3.55E-07
**72h**	2.69E-07	2.42E-07	9.63E-07	2.12E-07
	cgd8_5260			
**2h**	4.05E-08	0.00E+00	1.74E-08	5.44E-07
**6h**	3.18E-07	7.21E-07	1.01E-06	1.94E-06
**12h**	1.42E-08	4.83E-08	4.86E-08	5.08E-07
**24h**	7.91E-08	2.97E-07	1.02E-06	1.51E-06
**36h**	2.66E-07	3.54E-07	5.23E-07	3.63E-06
**48h**	1.43E-07	2.49E-07	4.94E-07	1.97E-06
**72h**	5.02E-08	4.63E-08	5.00E-08	5.97E-07
	cgd8_5270			
**2h**	1.35E-04	2.32E-05	2.19E-05	6.71E-05
**6h**	5.48E-05	1.12E-06	6.27E-05	2.33E-05
**12h**	9.72E-04	1.88E-04	2.64E-04	3.28E-04
**24h**	2.62E-04	6.92E-05	2.54E-04	1.98E-04
**36h**	3.80E-04	2.54E-04	1.45E-03	2.13E-03
**48h**	4.47E-04	7.38E-05	9.49E-04	2.08E-04
**72h**	5.42E-04	1.16E-04	9.72E-04	1.03E-04
	cgd8_5280			
**2h**	1.11E-06	6.53E-07	3.52E-07	1.12E-06
**6h**	9.76E-07	6.95E-07	1.50E-07	9.43E-06
**12h**	3.65E-07	2.67E-07	3.66E-07	5.55E-07
**24h**	9.60E-07	3.60E-07	2.62E-07	3.82E-07
**36h**	2.71E-07	1.08E-07	1.26E-07	2.73E-07
**48h**	5.19E-07	1.08E-07	2.25E-07	1.81E-07
**72h**	1.13E-07	3.44E-08	1.57E-07	5.13E-08
	cgd8_5290			
**2h**	3.54E-05	5.37E-06	1.32E-07	2.34E-04
**6h**	5.84E-05	5.03E-07	1.06E-07	2.28E-06
**12h**	8.37E-05	1.04E-04	2.39E-05	2.58E-04
**24h**	1.43E-05	2.79E-05	9.68E-06	4.35E-05
**36h**	2.95E-05	5.87E-06	3.42E-06	6.40E-05
**48h**	3.60E-05	4.18E-06	2.85E-06	4.94E-05
**72h**	2.03E-05	2.73E-06	2.02E-06	1.52E-05
	cgd8_5300			
**2h**	1.07E-04	2.97E-06	5.19E-07	8.53E-05
**6h**	4.03E-05	1.62E-06	3.02E-07	4.01E-05
**12h**	5.67E-04	6.68E-04	1.34E-04	1.04E-03
**24h**	4.73E-04	4.02E-05	2.82E-05	2.10E-04
**36h**	1.99E-04	5.37E-05	8.68E-05	9.30E-04
**48h**	5.15E-04	3.64E-05	5.09E-05	8.19E-04
**72h**	8.66E-05	7.64E-06	1.39E-06	8.83E-05
	cgd8_5310			

**2h**	0.00E+00	0.00E+00	0.00E+00	0.00E+00
**6h**	1.88E-09	3.63E-10	0.00E+00	8.14E-10
**12h**	4.20E-07	4.90E-07	6.84E-07	2.68E-07
**24h**	4.15E-07	4.22E-08	4.45E-08	7.21E-08
**36h**	1.95E-07	1.39E-07	6.05E-07	3.47E-07
**48h**	2.93E-07	4.65E-08	5.44E-08	1.67E-07
**72h**	3.30E-08	1.06E-08	2.11E-08	1.12E-08
	cgd8_5320			
**2h**	1.20E-07	5.94E-09	1.57E-09	0.00E+00
**6h**	6.60E-09	6.12E-08	0.00E+00	1.79E-08
**12h**	0.00E+00	7.24E-10	3.96E-10	1.36E-08
**24h**	5.15E-08	1.55E-09	8.20E-09	3.49E-08
**36h**	1.30E-07	2.79E-08	7.37E-09	5.16E-08
**48h**	4.34E-06	3.92E-07	3.41E-07	2.06E-06
**72h**	5.06E-07	3.87E-08	5.02E-08	1.46E-07
	cgd8_5330			
**2h**	9.48E-07	2.21E-07	5.84E-08	2.15E-07
**6h**	2.68E-07	3.65E-07	5.41E-08	1.90E-07
**12h**	4.42E-08	3.36E-07	7.21E-08	1.31E-07
**24h**	2.29E-07	3.74E-08	7.72E-08	3.38E-07
**36h**	3.23E-07	2.89E-07	2.81E-07	1.90E-07
**48h**	1.66E-05	6.86E-06	8.04E-06	1.74E-05
**72h**	5.24E-07	2.51E-07	7.95E-07	4.90E-07
	cgd8_5340			
**2h**	2.91E-05	7.13E-06	2.35E-06	2.21E-05
**6h**	2.54E-06	4.30E-06	2.70E-06	6.92E-06
**12h**	4.24E-06	1.01E-05	1.85E-05	1.52E-05
**24h**	2.15E-05	6.58E-06	1.22E-05	2.76E-05
**36h**	4.84E-06	1.28E-05	1.67E-05	1.87E-05
**48h**	1.32E-05	4.58E-06	9.77E-06	2.74E-05
**72h**	1.90E-06	6.70E-07	3.68E-06	2.31E-06
	cgd8_5350			
**2h**	3.62E-07	3.72E-08	1.48E-08	1.60E-06
**6h**	2.05E-08	2.87E-08	1.62E-08	0.00E+00
**12h**	2.95E-06	1.03E-06	5.49E-07	1.27E-06
**24h**	4.49E-06	3.81E-07	4.80E-07	9.05E-07
**36h**	7.76E-07	7.45E-07	5.00E-07	1.76E-06
**48h**	2.93E-06	4.15E-07	7.05E-07	9.68E-07
**72h**	3.97E-07	1.25E-07	1.03E-07	1.74E-08
	cgd8_5370			
**2h**	1.01E-06	1.57E-05	0.00E+00	8.62E-07
**6h**	3.55E-07	2.75E-05	2.57E-05	1.61E-06
**12h**	3.56E-07	0.00E+00	5.17E-05	9.25E-07
**24h**	8.95E-07	9.29E-06	2.93E-04	3.66E-06
**36h**	1.96E-06	1.37E-05	4.72E-05	2.21E-06
**48h**	3.42E-06	5.02E-06	3.69E-04	4.24E-06
**72h**	2.88E-06	6.27E-06	5.94E-04	3.50E-06
	cgd8_5380			
**2h**	5.75E-04	1.15E-03	9.56E-06	1.62E-04
**6h**	5.95E-05	3.51E-05	9.25E-06	2.51E-04
**12h**	2.93E-04	1.66E-04	4.77E-05	1.38E-03
**24h**	4.94E-04	4.17E-05	4.25E-05	5.55E-04
**36h**	1.74E-04	2.28E-05	2.89E-05	6.24E-04

**48h**	4.96E-04	3.97E-05	3.58E-05	7.31E-04
**72h**	1.36E-04	9.69E-06	9.43E-06	3.03E-04
	cgd8_5390			
**2h**	3.27E-06	4.91E-07	8.87E-08	5.15E-06
**6h**	5.03E-07	8.81E-07	4.01E-07	3.36E-06
**12h**	6.05E-07	7.57E-07	2.92E-07	3.36E-06
**24h**	1.82E-06	5.43E-07	9.55E-07	1.41E-06
**36h**	7.42E-07	6.12E-07	2.71E-07	4.09E-06
**48h**	9.44E-07	1.09E-06	4.93E-07	2.82E-06
**72h**	1.05E-06	2.77E-07	4.21E-07	7.35E-07
	cgd8_5400			
**2h**	2.11E-09	2.70E-06	1.43E-10	3.17E-08
**6h**	5.66E-10	1.06E-08	3.80E-09	3.13E-08
**12h**	1.04E-08	2.15E-08	6.63E-09	1.17E-07
**24h**	2.56E-08	5.98E-09	4.73E-08	6.54E-08
**36h**	1.62E-07	2.77E-08	3.82E-08	7.81E-07
**48h**	7.14E-08	6.31E-08	1.12E-07	6.05E-07
**72h**	1.02E-07	2.33E-08	3.59E-08	1.03E-07
	cgd8_5410			
**2h**	6.38E-07	1.22E-04	0.00E+00	1.57E-06
**6h**	2.16E-05	6.16E-04	9.24E-06	1.05E-05
**12h**	1.10E-04	1.39E-03	3.61E-06	1.58E-03
**24h**	2.62E-05	1.52E-04	4.07E-05	3.61E-04
**36h**	8.43E-06	2.00E-07	3.65E-05	1.97E-06
**48h**	8.62E-06	4.79E-07	2.91E-05	2.76E-04
**72h**	1.35E-05	3.77E-04	1.76E-05	3.30E-04
	cgd8_5420			
**2h**	6.22E-05	1.01E-04	6.19E-05	1.84E-03
**6h**	1.35E-05	2.74E-05	1.34E-05	3.19E-04
**12h**	5.64E-04	2.31E-03	4.47E-04	1.77E-03
**24h**	2.66E-04	5.69E-04	1.07E-04	4.68E-04
**36h**	5.41E-05	1.53E-04	6.34E-05	5.66E-04
**48h**	1.12E-03	7.00E-05	6.67E-05	3.49E-04
**72h**	3.89E-05	1.30E-04	2.31E-05	2.01E-04
	cgd1_1040			
**2h**	0.00E+00	0.00E+00	0.00E+00	0.00E+00
**6h**	0.00E+00	0.00E+00	0.00E+00	0.00E+00
**12h**	2.89E-03	1.48E-04	1.25E-03	6.62E-05
**24h**	5.99E-04	2.24E-05	3.77E-04	3.31E-04
**36h**	1.80E-04	3.19E-05	9.58E-04	9.19E-05
**48h**	2.79E-04	7.06E-05	5.59E-04	1.30E-04
**72h**	3.29E-04	1.71E-06	3.27E-03	9.49E-04
	cgd1_1070			
**2h**	2.59E-07	9.66E-08	5.80E-08	6.87E-07
**6h**	3.37E-08	3.31E-07	1.27E-07	2.67E-07
**12h**	2.65E-07	7.29E-07	1.90E-07	3.84E-07
**24h**	1.44E-07	3.77E-07	1.99E-07	4.58E-07
**36h**	1.93E-07	2.67E-07	2.26E-07	3.96E-07
**48h**	5.89E-08	1.16E-07	9.51E-08	1.71E-07
**72h**	3.57E-08	4.42E-08	4.74E-08	4.43E-08
	cgd1_1200			
**2h**	6.53E-06	1.98E-05	1.44E-05	1.36E-04
**6h**	1.06E-07	1.02E-06	2.74E-07	6.30E-06

**12h**	1.89E-04	2.80E-03	1.30E-04	7.57E-04
**24h**	3.98E-05	4.05E-04	1.88E-05	2.04E-04
**36h**	1.07E-05	1.03E-04	3.15E-05	1.40E-04
**48h**	8.04E-06	4.82E-05	1.52E-05	7.76E-05
**72h**	1.30E-05	1.12E-04	1.37E-05	3.79E-05
	cgd1_1320			
**2h**	2.74E-09	1.50E-08	2.94E-08	3.42E-08
**6h**	3.34E-08	3.68E-08	6.88E-08	1.24E-07
**12h**	3.05E-08	9.69E-09	1.05E-08	7.81E-08
**24h**	6.86E-09	1.37E-08	1.16E-07	6.84E-08
**36h**	2.24E-08	4.23E-08	3.89E-08	7.67E-08
**48h**	1.91E-08	2.19E-08	3.15E-08	3.78E-08
**72h**	1.39E-08	1.34E-08	3.45E-09	3.24E-08
	cgd1_1540			
**2h**	1.84E-07	1.56E-07	5.67E-08	1.19E-07
**6h**	2.15E-07	1.63E-07	2.48E-07	2.46E-07
**12h**	3.31E-07	2.92E-07	3.96E-07	4.82E-07
**24h**	3.46E-07	2.34E-07	7.61E-07	8.66E-07
**36h**	1.50E-06	3.21E-06	1.42E-06	1.32E-06
**48h**	8.65E-07	4.93E-07	9.14E-07	8.73E-07
**72h**	2.52E-07	3.79E-07	1.57E-07	3.18E-07
	cgd1_1590			
**2h**	2.01E-04	3.27E-03	1.63E-04	3.55E-03
**6h**	2.44E-04	1.27E-03	2.77E-04	1.11E-03
**12h**	4.54E-05	4.39E-04	2.10E-04	1.14E-03
**24h**	2.85E-04	1.39E-03	3.69E-04	1.38E-03
**36h**	6.92E-05	3.52E-04	8.01E-05	4.56E-04
**48h**	4.13E-05	2.57E-04	1.19E-04	4.48E-04
**72h**	3.48E-05	1.31E-04	4.58E-05	1.29E-04
	cgd1_160			
**2h**	9.38E-07	1.90E-06	1.28E-07	5.71E-06
**6h**	4.81E-06	8.94E-06	1.92E-06	7.02E-06
**12h**	2.21E-08	2.00E-08	1.78E-06	1.42E-06
**24h**	6.94E-07	5.72E-07	1.20E-06	1.04E-06
**36h**	2.16E-07	5.94E-07	1.48E-07	7.64E-07
**48h**	2.69E-07	5.44E-07	2.18E-07	7.04E-07
**72h**	6.32E-08	1.17E-07	5.23E-08	3.38E-07
	cgd1_1600			
**2h**	1.04E-06	1.50E-06	4.74E-07	1.24E-06
**6h**	8.90E-07	6.86E-07	4.31E-07	5.24E-07
**12h**	3.24E-07	1.80E-07	4.87E-07	4.19E-07
**24h**	7.63E-07	1.24E-06	7.15E-07	3.70E-07
**36h**	3.80E-07	3.80E-07	3.33E-07	4.77E-07
**48h**	3.76E-07	2.50E-07	4.02E-07	2.14E-07
**72h**	2.15E-07	1.75E-07	2.77E-07	2.98E-07
	cgd1_1620			
**2h**	1.31E-06	1.46E-07	2.47E-06	1.20E-06
**6h**	1.02E-07	7.84E-08	5.32E-07	1.64E-06
**12h**	2.88E-07	3.91E-08	1.54E-06	9.78E-07
**24h**	1.68E-06	5.19E-07	6.25E-07	4.48E-07
**36h**	3.27E-07	8.97E-08	6.75E-07	8.06E-07
**48h**	1.11E-06	1.13E-07	3.11E-07	8.32E-07
**72h**	1.96E-07	6.30E-08	4.34E-07	1.84E-07

	cgd1_1720			
**2h**	1.56E-06	2.23E-07	2.03E-07	1.62E-07
**6h**	1.72E-06	1.15E-06	8.10E-07	5.79E-07
**12h**	2.50E-08	9.11E-09	4.27E-08	1.71E-08
**24h**	4.18E-07	2.57E-07	6.04E-07	3.76E-07
**36h**	4.36E-07	1.45E-07	9.57E-08	8.56E-08
**48h**	4.71E-07	1.21E-07	9.23E-08	9.91E-08
**72h**	1.10E-07	7.44E-08	5.42E-08	9.21E-08
	cgd1_1730			
**2h**	1.53E-07	4.01E-08	2.43E-08	2.77E-08
**6h**	1.29E-04	1.70E-04	1.20E-04	1.31E-04
**12h**	1.87E-06	1.67E-06	2.87E-06	2.23E-06
**24h**	3.22E-05	9.40E-06	4.77E-05	3.45E-05
**36h**	1.53E-05	1.45E-05	1.15E-05	1.31E-05
**48h**	1.89E-05	7.69E-06	5.84E-06	7.65E-06
**72h**	7.38E-07	3.57E-07	6.64E-07	4.25E-07
	cgd1_1900			
**2h**	2.26E-07	2.77E-08	3.20E-07	1.20E-07
**6h**	2.21E-07	5.59E-08	8.12E-07	3.72E-07
**12h**	1.41E-08	8.16E-08	7.92E-08	4.00E-08
**24h**	1.52E-07	3.22E-08	4.87E-07	2.70E-07
**36h**	1.13E-07	7.31E-08	1.07E-07	2.01E-07
**48h**	5.11E-07	1.93E-07	7.72E-07	8.08E-07
**72h**	2.48E-07	2.38E-07	3.62E-07	3.04E-07
	cgd1_2040			
**2h**	7.55E-05	6.96E-06	1.87E-04	2.57E-04
**6h**	3.07E-05	1.37E-05	3.11E-04	3.51E-04
**12h**	1.08E-04	6.41E-05	8.45E-04	3.35E-04
**24h**	5.30E-05	1.07E-05	2.27E-04	2.53E-04
**36h**	7.32E-05	2.52E-05	3.82E-04	6.84E-04
**48h**	1.25E-04	1.46E-05	4.66E-04	6.43E-04
**72h**	2.76E-05	1.41E-05	3.52E-04	1.73E-04
	cgd1_2050			
**2h**	1.85E-05	6.20E-06	9.41E-05	2.31E-04
**6h**	8.58E-06	4.52E-05	9.12E-05	4.60E-04
**12h**	2.06E-06	5.84E-07	1.83E-04	9.76E-05
**24h**	1.47E-05	4.56E-06	8.94E-05	8.86E-05
**36h**	5.81E-06	2.94E-06	3.39E-05	6.74E-05
**48h**	8.19E-06	1.03E-06	9.87E-05	1.70E-04
**72h**	9.98E-07	1.11E-06	2.25E-05	1.36E-05
	cgd1_2340			
**2h**	0.00E+00	8.03E-09	0.00E+00	3.50E-09
**6h**	7.97E-08	7.86E-08	2.71E-07	2.64E-07
**12h**	9.78E-09	2.17E-08	4.80E-08	5.18E-09
**24h**	1.74E-08	2.60E-08	2.01E-07	1.72E-07
**36h**	7.53E-08	4.77E-08	3.37E-08	7.39E-08
**48h**	1.36E-07	9.82E-08	5.55E-08	5.40E-08
**72h**	1.99E-08	2.30E-08	1.10E-08	2.62E-08
	cgd1_290			
**2h**	1.31E-07	2.44E-08	0.00E+00	9.16E-08
**6h**	3.03E-07	1.47E-06	5.48E-06	6.66E-06
**12h**	9.50E-08	1.00E-07	4.74E-07	2.16E-07
**24h**	1.64E-07	3.59E-07	2.74E-06	1.15E-06

**36h**	5.12E-07	7.28E-07	9.84E-07	1.31E-06
**48h**	7.96E-07	8.77E-07	1.23E-06	8.19E-07
**72h**	3.18E-07	3.10E-07	7.51E-07	5.09E-07
	cgd1_2950			
**2h**	1.49E-04	3.82E-05	1.86E-06	8.71E-06
**6h**	2.64E-05	7.98E-05	7.44E-05	5.59E-05
**12h**	1.55E-06	3.44E-05	2.34E-05	2.26E-05
**24h**	8.23E-05	2.03E-05	1.19E-05	2.83E-05
**36h**	5.71E-05	5.08E-05	7.36E-06	6.07E-06
**48h**	9.49E-05	9.93E-05	3.76E-06	2.59E-05
**72h**	1.04E-04	8.07E-05	1.51E-05	6.26E-06
	cgd1_300			
**2h**	2.32E-04	5.79E-04	5.01E-05	4.32E-05
**6h**	5.11E-04	1.12E-03	1.81E-03	3.56E-05
**12h**	2.36E-04	1.10E-04	9.46E-04	2.62E-05
**24h**	4.37E-05	4.96E-04	1.91E-04	1.31E-04
**36h**	6.02E-05	1.47E-04	2.20E-04	1.94E-05
**48h**	1.07E-04	2.12E-04	8.37E-05	6.71E-05
**72h**	1.93E-04	1.30E-04	4.96E-05	4.30E-05
	cgd1_310			
**2h**	4.41E-08	1.50E-09	5.78E-09	9.31E-09
**6h**	1.55E-07	2.48E-07	2.04E-07	4.81E-08
**12h**	4.89E-06	2.10E-06	4.84E-07	4.69E-07
**24h**	9.62E-07	1.15E-07	1.21E-07	2.83E-07
**36h**	3.46E-07	1.57E-07	8.57E-08	2.62E-07
**48h**	1.08E-06	8.33E-08	1.88E-07	2.36E-07
**72h**	2.02E-07	1.71E-07	7.51E-08	1.89E-07
	cgd1_320			
**2h**	3.18E-07	7.35E-08	1.02E-07	4.87E-08
**6h**	7.13E-08	2.76E-08	3.18E-08	5.87E-08
**12h**	9.75E-06	7.23E-06	4.53E-06	3.50E-06
**24h**	5.63E-06	9.04E-07	3.58E-07	4.22E-07
**36h**	7.35E-07	1.61E-07	6.78E-07	1.28E-06
**48h**	9.66E-07	1.40E-07	5.52E-07	1.82E-07
**72h**	2.83E-07	8.36E-08	4.81E-08	2.66E-07
	cgd1_3280			
**2h**	1.95E-07	3.46E-07	7.12E-08	1.26E-07
**6h**	1.20E-07	4.25E-07	1.07E-07	6.39E-08
**12h**	5.58E-09	2.47E-08	1.80E-08	4.44E-08
**24h**	1.17E-07	7.04E-08	1.21E-07	1.10E-07
**36h**	6.09E-08	5.96E-08	3.22E-08	3.41E-07
**48h**	1.44E-07	7.77E-08	1.80E-07	7.37E-08
**72h**	4.97E-08	4.49E-08	2.27E-08	4.54E-08
	cgd1_330			
**2h**	0.00E+00	0.00E+00	0.00E+00	0.00E+00
**6h**	4.92E-04	5.53E-04	1.51E-03	3.05E-05
**12h**	1.52E-04	1.00E-04	5.19E-04	5.62E-06
**24h**	4.22E-04	3.72E-05	6.62E-04	4.99E-05
**36h**	3.86E-04	1.13E-04	4.36E-04	4.57E-05
**48h**	9.28E-04	3.40E-04	1.78E-04	4.48E-05
**72h**	2.30E-04	4.43E-05	2.56E-05	1.71E-05
	cgd1_3370			
**2h**	3.00E-07	2.49E-07	0.00E+00	3.49E-08

**6h**	1.34E-04	5.02E-05	8.01E-05	6.44E-07
**12h**	7.53E-05	4.99E-07	2.00E-05	1.62E-07
**24h**	1.02E-04	5.64E-06	1.17E-06	4.82E-06
**36h**	2.63E-04	3.14E-05	2.91E-05	3.94E-06
**48h**	9.05E-04	8.42E-05	5.94E-05	1.95E-05
**72h**	1.08E-03	9.22E-05	7.00E-05	2.42E-05
	cgd1_3380			
**2h**	9.05E-07	3.74E-07	7.62E-06	1.45E-07
**6h**	1.68E-05	6.70E-06	1.13E-04	1.94E-06
**12h**	8.36E-07	6.57E-07	2.36E-05	5.06E-07
**24h**	5.20E-06	2.63E-06	5.79E-05	3.57E-06
**36h**	6.82E-06	3.69E-06	1.64E-04	1.85E-06
**48h**	5.70E-06	4.41E-06	2.85E-05	1.54E-06
**72h**	4.81E-06	1.70E-06	2.63E-05	7.82E-07
	cgd1_340			
**2h**	0.00E+00	3.00E-08	0.00E+00	0.00E+00
**6h**	1.46E-05	1.05E-05	6.49E-05	3.21E-06
**12h**	2.88E-04	5.17E-05	1.69E-04	2.87E-06
**24h**	1.02E-04	1.81E-05	4.15E-05	9.38E-06
**36h**	3.99E-04	4.01E-05	2.32E-04	1.06E-05
**48h**	4.67E-04	2.29E-05	1.15E-04	1.81E-05
**72h**	3.74E-04	5.05E-05	5.54E-05	1.08E-05
	cgd1_360			
**2h**	4.35E-04	2.29E-03	2.54E-06	4.76E-02
**6h**	2.46E-05	6.01E-04	5.00E-07	4.97E-03
**12h**	5.45E-05	1.43E-03	8.04E-06	1.62E-02
**24h**	3.52E-04	1.37E-03	3.90E-06	7.87E-03
**36h**	2.37E-05	5.17E-04	2.14E-06	6.21E-03
**48h**	3.92E-05	1.18E-03	1.26E-06	5.10E-03
**72h**	2.34E-05	4.56E-04	3.05E-06	1.81E-03
	cgd1_3810			
**2h**	3.29E-04	2.91E-04	3.60E-03	3.66E-04
**6h**	1.65E-03	1.58E-03	8.45E-03	7.98E-04
**12h**	1.96E-04	2.54E-04	1.61E-03	4.59E-04
**24h**	7.71E-04	2.06E-04	4.09E-03	1.09E-03
**36h**	2.64E-04	1.85E-04	1.20E-03	2.78E-04
**48h**	1.92E-04	4.87E-05	5.25E-04	1.21E-04
**72h**	6.72E-05	2.15E-05	2.01E-04	3.79E-05
	cgd1_3850			
**2h**	8.38E-06	5.62E-02	1.90E-05	7.11E-05
**6h**	1.89E-08	1.64E-04	3.88E-06	5.81E-05
**12h**	2.57E-06	0.00E+00	1.19E-04	2.08E-04
**24h**	1.96E-05	1.33E-03	1.61E-04	1.27E-03
**36h**	1.01E-06	2.41E-03	1.47E-04	3.16E-04
**48h**	8.54E-07	6.68E-04	1.04E-04	6.86E-06
**72h**	1.35E-05	8.92E-04	3.51E-05	1.51E-04
	cgd1_3860			
**2h**	5.09E-06	3.88E-07	1.93E-07	9.49E-08
**6h**	1.28E-07	1.57E-07	7.67E-08	2.47E-07
**12h**	3.33E-08	1.59E-08	9.87E-08	2.35E-08
**24h**	1.91E-06	1.05E-07	9.23E-08	1.33E-07
**36h**	1.27E-05	7.10E-08	1.50E-07	2.57E-07
**48h**	2.37E-05	8.05E-08	2.22E-07	2.51E-07

**72h**	2.34E-07	8.02E-08	9.35E-08	7.41E-08
	cgd1_40			
**2h**	0.00E+00	0.00E+00	0.00E+00	0.00E+00
**6h**	1.55E-04	3.00E-06	4.24E-05	4.29E-05
**12h**	8.47E-05	1.29E-07	1.06E-05	5.74E-06
**24h**	2.41E-04	1.18E-06	6.84E-05	5.01E-05
**36h**	1.67E-04	4.55E-06	3.51E-05	2.14E-05
**48h**	1.38E-04	4.80E-06	2.35E-05	2.87E-05
**72h**	2.19E-04	9.89E-07	2.12E-06	2.20E-06
	cgd1_470			
**2h**	2.15E-05	2.72E-06	4.23E-06	5.13E-06
**6h**	8.48E-06	3.88E-06	6.95E-06	1.60E-05
**12h**	6.85E-06	6.24E-07	4.25E-06	4.06E-06
**24h**	1.68E-05	1.51E-06	8.70E-06	9.77E-06
**36h**	1.15E-05	6.42E-06	7.83E-06	8.25E-06
**48h**	5.42E-05	3.07E-06	6.05E-06	5.30E-06
**72h**	3.06E-06	5.59E-07	1.00E-06	1.01E-06
	cgd1_50			
**2h**	2.12E-05	1.95E-07	3.16E-08	2.36E-07
**6h**	5.77E-05	2.97E-06	1.65E-05	4.98E-05
**12h**	1.97E-06	1.86E-07	6.05E-06	1.72E-06
**24h**	5.38E-05	1.02E-06	1.59E-05	1.33E-05
**36h**	2.55E-05	2.85E-06	8.86E-06	7.55E-06
**48h**	3.97E-05	1.58E-06	2.47E-06	4.05E-06
**72h**	6.82E-06	3.20E-07	1.54E-06	5.62E-07
	cgd1_500			
**2h**	2.67E-10	0.00E+00	1.78E-09	0.00E+00
**6h**	0.00E+00	5.26E-08	1.39E-07	1.96E-07
**12h**	4.43E-09	2.52E-09	1.27E-08	1.93E-08
**24h**	3.26E-08	2.46E-08	2.54E-07	1.66E-07
**36h**	1.08E-07	2.01E-07	1.42E-07	1.39E-07
**48h**	9.09E-07	2.29E-07	5.40E-07	6.95E-07
**72h**	2.00E-06	1.34E-06	1.67E-06	1.37E-06
	cgd1_520			
**2h**	8.03E-04	2.87E-02	1.97E-03	4.16E-03
**6h**	5.76E-04	2.16E-02	2.24E-03	1.10E-02
**12h**	8.26E-05	1.10E-02	2.70E-03	6.65E-03
**24h**	4.00E-04	1.33E-02	9.14E-04	1.76E-02
**36h**	1.21E-04	1.62E-03	1.45E-03	1.22E-03
**48h**	2.23E-04	1.10E-02	9.36E-04	3.08E-03
**72h**	1.42E-04	2.02E-03	4.36E-04	2.67E-03
	cgd1_550			
**2h**	3.06E-05	6.70E-04	7.49E-04	9.46E-04
**6h**	4.69E-05	9.56E-04	1.58E-04	2.24E-04
**12h**	4.71E-07	1.30E-05	3.18E-05	3.64E-04
**24h**	6.27E-05	8.68E-05	7.39E-04	2.48E-03
**36h**	2.00E-05	2.11E-04	2.16E-04	1.60E-03
**48h**	8.63E-06	2.71E-04	1.78E-04	4.73E-04
**72h**	7.19E-05	4.10E-04	1.33E-03	7.12E-04
	cgd1_600			
**2h**	0.00E+00	1.97E-05	8.05E-04	5.00E-04
**6h**	2.60E-04	2.81E-05	6.70E-05	2.03E-04
**12h**	1.87E-05	2.76E-06	1.55E-04	2.71E-04

**24h**	2.50E-04	1.40E-05	1.33E-04	1.63E-04
**36h**	7.05E-05	1.04E-05	1.29E-04	1.11E-04
**48h**	1.76E-04	7.94E-06	7.52E-05	2.29E-04
**72h**	5.78E-05	2.16E-04	3.42E-05	5.37E-05
	cgd1_640			
**2h**	1.11E-02	1.23E-03	1.39E-02	6.26E-03
**6h**	7.31E-05	2.34E-05	3.80E-05	1.39E-04
**12h**	8.77E-03	4.58E-04	6.01E-03	2.96E-03
**24h**	8.39E-03	1.30E-03	2.40E-03	6.20E-04
**36h**	0.00E+00	2.80E-04	1.42E-03	6.75E-04
**48h**	3.39E-03	3.44E-04	5.18E-04	4.24E-04
**72h**	7.28E-04	7.49E-05	2.74E-04	1.18E-04
	cgd1_710			
**2h**	0.00E+00	0.00E+00	0.00E+00	0.00E+00
**6h**	0.00E+00	0.00E+00	0.00E+00	0.00E+00
**12h**	7.87E-08	2.26E-08	6.02E-08	1.48E-07
**24h**	2.20E-07	4.63E-08	3.89E-08	2.39E-08
**36h**	1.73E-07	3.45E-07	4.63E-07	4.89E-07
**48h**	8.36E-07	4.30E-07	4.75E-07	3.20E-07
**72h**	1.14E-06	6.29E-07	5.91E-07	5.70E-07
	cgd3_3430			
**2h**	0.00E+00	0.00E+00	5.56E-09	2.87E-08
**6h**	0.00E+00	0.00E+00	0.00E+00	5.20E-09
**12h**	0.00E+00	2.46E-09	1.38E-09	6.51E-09
**24h**	2.95E-10	9.26E-09	1.16E-08	2.77E-07
**36h**	1.45E-07	1.57E-08	2.62E-08	6.06E-07
**48h**	2.36E-06	5.43E-07	5.45E-07	2.78E-05
**72h**	5.62E-08	1.10E-07	2.38E-07	7.38E-07
	cgd3_3820			
**2h**	0.00E+00	0.00E+00	0.00E+00	0.00E+00
**6h**	0.00E+00	1.51E-06	0.00E+00	2.90E-07
**12h**	1.42E-05	3.51E-05	1.05E-04	2.14E-05
**24h**	6.65E-05	4.52E-05	5.33E-04	7.84E-05
**36h**	3.04E-04	1.13E-04	4.50E-06	2.00E-04
**48h**	6.37E-04	1.71E-05	1.44E-04	1.77E-04
**72h**	5.85E-04	3.34E-05	3.97E-05	1.92E-04
	cgd3_3850			
**2h**	4.02E-02	6.89E-05	1.06E-03	1.42E-02
**6h**	3.79E-03	3.58E-01	3.53E-04	2.45E-03
**12h**	1.81E-03	4.03E-04	6.51E-05	8.12E-03
**24h**	1.18E-03	2.88E-04	3.59E-04	7.42E-03
**36h**	6.00E-04	1.68E-04	2.00E-04	5.12E-03
**48h**	6.04E-04	2.19E-04	7.16E-04	7.15E-04
**72h**	1.48E-03	7.36E-05	1.10E-04	1.86E-03
	cgd3_4100			
**2h**	6.31E-06	1.34E-04	3.38E-05	9.98E-05
**6h**	2.00E-06	6.09E-06	4.36E-06	1.77E-05
**12h**	1.34E-06	9.65E-06	7.76E-06	1.52E-05
**24h**	3.02E-06	3.83E-06	3.80E-06	7.01E-05
**36h**	3.73E-07	4.92E-06	3.81E-06	1.39E-05
**48h**	9.82E-07	2.81E-06	4.52E-06	1.54E-05
**72h**	7.68E-07	1.31E-06	9.64E-07	3.78E-06
	cgd3_4110			

**2h**	3.69E-09	0.00E+00	1.36E-07	4.93E-08
**6h**	3.64E-08	4.97E-07	1.35E-07	9.00E-07
**12h**	1.12E-08	8.91E-08	6.98E-09	5.62E-08
**24h**	2.41E-08	2.34E-07	1.46E-07	0.00E+00
**36h**	4.42E-08	4.70E-07	3.33E-07	1.25E-06
**48h**	1.26E-07	2.15E-07	1.02E-07	1.51E-06
**72h**	5.72E-08	5.81E-08	2.83E-07	3.80E-07
	cgd3_4170			
**2h**	0.00E+00	0.00E+00	2.29E-05	4.38E-07
**6h**	0.00E+00	7.21E-07	3.66E-07	1.14E-07
**12h**	6.00E-08	3.23E-08	1.60E-05	5.78E-07
**24h**	3.06E-08	1.03E-05	7.44E-05	6.28E-06
**36h**	3.13E-07	3.62E-05	1.99E-05	5.40E-06
**48h**	3.51E-07	4.13E-05	1.36E-05	3.11E-06
**72h**	8.67E-08	3.33E-05	5.52E-07	1.24E-06
	cgd3_4220			
**2h**	3.42E-06	3.73E-05	4.64E-05	3.89E-05
**6h**	1.35E-06	9.75E-06	2.27E-05	3.02E-05
**12h**	1.83E-06	4.81E-05	3.10E-05	2.36E-05
**24h**	1.04E-06	1.47E-05	6.68E-06	1.15E-04
**36h**	6.10E-07	2.54E-05	1.90E-05	1.88E-05
**48h**	7.99E-07	7.10E-06	8.40E-06	4.10E-05
**72h**	3.19E-07	1.65E-06	1.51E-06	2.58E-06
	cgd1_780			
**2h**	2.23E-07	1.90E-06	0.00E+00	4.33E-06
**6h**	1.70E-04	1.31E-06	3.56E-04	1.87E-04
**12h**	3.91E-07	0.00E+00	4.36E-07	9.41E-08
**24h**	5.96E-06	6.97E-07	4.54E-06	3.47E-05
**36h**	6.23E-05	2.19E-06	8.09E-06	1.20E-05
**48h**	1.01E-05	5.43E-07	5.73E-06	1.35E-05
**72h**	2.74E-05	2.38E-06	2.51E-06	2.64E-05
	cgd1_850			
**2h**	8.56E-03	5.47E-05	1.18E-02	4.57E-03
**6h**	2.55E-02	1.03E-04	2.51E-02	1.41E-02
**12h**	1.20E-03	1.06E-05	9.69E-03	4.17E-03
**24h**	6.85E-03	9.28E-06	4.43E-03	2.56E-02
**36h**	4.59E-03	9.25E-06	2.55E-03	3.85E-03
**48h**	2.84E-03	2.23E-06	2.11E-03	1.14E-04
**72h**	8.71E-04	6.74E-06	1.60E-03	1.29E-03
	cgd1_870			
**2h**	5.21E-07	1.46E-07	1.71E-08	2.04E-07
**6h**	2.56E-06	9.35E-07	1.61E-06	2.02E-06
**12h**	5.08E-08	2.36E-07	9.98E-08	6.65E-08
**24h**	3.05E-07	2.06E-07	2.72E-07	7.26E-07
**36h**	6.97E-07	1.18E-06	4.07E-07	7.84E-07
**48h**	4.45E-06	6.91E-06	2.72E-06	1.03E-06
**72h**	1.06E-06	3.96E-06	1.20E-06	7.38E-07
	cgd1_880			
**2h**	1.21E-04	1.74E-03	8.84E-05	1.40E-04
**6h**	9.83E-05	5.93E-04	1.34E-04	2.39E-04
**12h**	1.29E-05	4.93E-04	4.78E-05	2.76E-05
**24h**	7.46E-05	2.25E-04	3.42E-05	9.00E-05
**36h**	4.43E-05	2.44E-04	2.16E-05	4.16E-05

**48h**	4.64E-05	1.98E-04	2.38E-05	2.00E-05
**72h**	1.08E-05	1.13E-04	3.18E-05	1.32E-05
	cgd1_910			
**2h**	1.59E-06	1.87E-06	1.34E-06	2.39E-06
**6h**	1.07E-06	7.18E-07	4.97E-07	1.16E-06
**12h**	1.19E-06	3.92E-06	1.11E-06	1.99E-06
**24h**	1.87E-06	7.16E-06	6.32E-07	2.35E-06
**36h**	1.67E-06	4.62E-06	8.97E-07	1.74E-06
**48h**	1.16E-06	1.38E-05	6.90E-07	1.50E-06
**72h**	3.82E-07	1.10E-05	2.38E-07	2.46E-07
	cgd1_930			
**2h**	0.00E+00	9.18E-07	0.00E+00	0.00E+00
**6h**	9.33E-04	8.03E-06	1.15E-03	3.30E-05
**12h**	6.53E-04	1.12E-05	1.61E-03	4.51E-04
**24h**	3.62E-04	3.23E-06	2.87E-04	6.72E-04
**36h**	8.48E-04	7.15E-06	7.94E-04	6.62E-05
**48h**	7.65E-04	2.92E-06	5.53E-04	5.56E-04
**72h**	7.28E-05	1.50E-06	1.39E-04	1.25E-04
	cgd2_10			
**2h**	0.00E+00	5.60E-09	0.00E+00	0.00E+00
**6h**	4.29E-05	1.10E-07	1.72E-04	2.76E-05
**12h**	1.01E-04	2.70E-07	7.11E-05	8.01E-05
**24h**	1.38E-04	1.31E-07	1.52E-04	5.41E-06
**36h**	2.44E-04	1.14E-06	9.27E-05	7.03E-05
**48h**	5.11E-04	7.13E-07	7.48E-04	2.39E-04
**72h**	1.32E-03	0.00E+00	2.60E-03	7.19E-05
	cgd2_1000			
**2h**	1.80E-06	2.36E-06	2.39E-07	9.62E-07
**6h**	1.03E-05	3.40E-05	3.30E-05	5.29E-05
**12h**	1.18E-06	4.16E-06	3.46E-06	2.58E-06
**24h**	4.91E-06	2.25E-05	5.08E-06	1.39E-05
**36h**	1.01E-05	1.91E-05	8.48E-06	4.73E-06
**48h**	7.35E-06	6.37E-05	1.43E-06	2.74E-06
**72h**	2.37E-06	4.29E-05	2.49E-06	1.48E-06
	cgd2_1010			
**2h**	7.30E-06	2.73E-05	8.31E-07	7.84E-06
**6h**	5.27E-06	6.10E-05	7.29E-06	1.47E-05
**12h**	4.67E-07	4.28E-06	2.98E-06	1.73E-06
**24h**	7.39E-06	1.45E-05	4.12E-06	9.35E-06
**36h**	7.10E-06	1.08E-04	6.77E-06	6.57E-06
**48h**	5.70E-06	2.23E-04	3.04E-06	3.62E-06
**72h**	1.53E-06	4.99E-05	2.70E-06	1.74E-06
	cgd2_1030			
**2h**	2.98E-05	1.19E-02	1.88E-05	1.07E-04
**6h**	8.27E-06	8.18E-04	1.45E-05	4.18E-05
**12h**	5.16E-07	9.91E-05	4.68E-06	7.85E-06
**24h**	7.28E-06	5.49E-04	5.59E-06	1.63E-05
**36h**	1.09E-05	5.59E-03	1.06E-05	1.16E-05
**48h**	3.11E-06	2.17E-03	2.00E-06	3.46E-06
**72h**	8.84E-07	2.36E-03	1.07E-06	1.95E-06
	cgd2_110			
**2h**	3.02E-07	1.69E-07	1.90E-07	3.58E-07
**6h**	7.67E-08	6.30E-08	3.35E-08	1.99E-07

**12h**	1.38E-07	1.44E-07	3.16E-07	6.31E-08
**24h**	5.93E-07	6.61E-07	2.11E-07	1.11E-07
**36h**	7.78E-08	1.95E-07	7.98E-08	5.00E-08
**48h**	6.08E-07	5.06E-08	7.00E-08	1.69E-07
**72h**	3.53E-08	5.97E-08	3.07E-08	1.22E-06
	cgd2_1140			
**2h**	0.00E+00	0.00E+00	0.00E+00	0.00E+00
**6h**	5.45E-08	2.97E-07	9.43E-07	8.72E-07
**12h**	2.07E-08	3.73E-08	3.30E-07	2.87E-08
**24h**	4.08E-08	4.41E-07	1.23E-06	9.30E-07
**36h**	1.61E-07	4.79E-07	3.72E-07	2.84E-07
**48h**	2.75E-07	1.52E-06	5.87E-07	8.81E-07
**72h**	1.42E-08	3.83E-07	2.15E-07	2.83E-07
	cgd2_120			
**2h**	7.36E-07	3.11E-06	5.50E-07	1.54E-06
**6h**	6.94E-06	6.49E-06	2.04E-05	7.87E-06
**12h**	2.36E-07	5.04E-07	1.75E-06	5.49E-07
**24h**	1.71E-06	1.98E-06	2.38E-06	1.44E-06
**36h**	1.11E-06	1.76E-06	3.75E-07	3.74E-07
**48h**	3.86E-07	5.77E-07	9.29E-07	7.12E-07
**72h**	1.55E-07	1.55E-07	1.19E-07	1.48E-07
	cgd2_1210			
**2h**	5.26E-07	2.58E-06	1.13E-05	5.55E-05
**6h**	2.83E-07	1.95E-06	5.70E-06	1.10E-05
**12h**	2.00E-07	2.42E-07	1.31E-05	7.07E-06
**24h**	6.06E-07	9.36E-06	7.07E-06	4.92E-06
**36h**	1.20E-06	6.33E-06	4.44E-06	5.96E-06
**48h**	8.88E-07	5.52E-06	2.43E-05	2.48E-05
**72h**	7.36E-07	3.81E-06	2.59E-06	7.35E-06
	cgd2_130			
**2h**	6.48E-05	3.14E-05	1.36E-04	4.48E-05
**6h**	5.13E-04	1.41E-03	3.47E-04	1.69E-03
**12h**	2.56E-05	1.34E-03	5.51E-04	2.04E-05
**24h**	1.62E-04	1.30E-03	1.55E-04	3.04E-04
**36h**	9.44E-05	8.33E-04	1.15E-04	1.93E-04
**48h**	3.21E-05	4.83E-04	4.99E-05	1.05E-04
**72h**	7.03E-06	2.91E-04	3.84E-05	6.20E-06
	cgd2_1360			
**2h**	5.64E-06	1.34E-05	1.09E-05	8.16E-06
**6h**	1.77E-06	4.74E-06	3.49E-06	7.46E-06
**12h**	5.07E-06	5.12E-06	1.47E-05	7.52E-06
**24h**	3.26E-06	4.48E-06	6.17E-06	6.25E-06
**36h**	5.47E-06	5.14E-06	4.46E-06	3.54E-06
**48h**	2.38E-06	1.45E-06	9.29E-06	1.01E-05
**72h**	1.32E-06	5.82E-07	9.34E-07	1.16E-06
	cgd2_1390			
**2h**	7.93E-08	1.90E-08	1.12E-07	2.89E-07
**6h**	2.98E-08	8.79E-08	2.23E-07	7.43E-07
**12h**	2.90E-08	3.48E-08	2.70E-08	5.99E-08
**24h**	1.59E-08	4.74E-08	1.36E-07	7.39E-08
**36h**	1.73E-07	1.59E-07	2.42E-07	2.66E-07
**48h**	2.18E-07	1.03E-07	2.92E-07	4.30E-07
**72h**	2.62E-07	1.85E-07	5.64E-07	5.41E-07

	cgd2_1640			
**2h**	0.00E+00	0.00E+00	0.00E+00	0.00E+00
**6h**	1.65E-05	3.43E-06	1.10E-07	1.32E-07
**12h**	3.24E-04	1.67E-06	1.49E-07	2.70E-07
**24h**	7.64E-05	1.30E-04	1.72E-07	9.67E-08
**36h**	1.17E-03	8.52E-05	8.14E-07	4.90E-07
**48h**	7.55E-04	2.06E-05	4.98E-08	1.36E-07
**72h**	3.94E-05	5.14E-05	2.03E-06	8.61E-07
	cgd2_170			
**2h**	4.06E-06	4.55E-06	1.23E-05	9.40E-06
**6h**	1.69E-05	1.89E-05	3.85E-05	2.98E-05
**12h**	1.11E-06	7.03E-07	1.56E-05	7.56E-06
**24h**	5.24E-06	5.24E-06	1.02E-05	8.99E-06
**36h**	7.42E-06	1.92E-06	5.72E-06	8.21E-06
**48h**	1.76E-06	1.14E-06	1.98E-06	2.34E-06
**72h**	7.14E-07	3.32E-07	5.05E-07	5.28E-07
	cgd2_1790			
**2h**	1.23E-05	5.70E-05	1.43E-04	1.03E-04
**6h**	1.11E-06	5.78E-05	2.02E-04	3.20E-04
**12h**	2.22E-06	3.40E-05	4.00E-04	8.07E-06
**24h**	1.61E-05	7.83E-05	1.19E-04	1.61E-04
**36h**	1.84E-06	7.50E-05	3.88E-04	6.02E-04
**48h**	5.48E-06	3.47E-05	7.82E-05	2.26E-05
**72h**	8.64E-07	1.49E-05	3.60E-05	8.40E-06
	cgd2_1860			
**2h**	2.14E-03	2.98E-04	8.00E-07	2.10E-06
**6h**	2.69E-04	8.68E-04	1.75E-06	7.16E-06
**12h**	4.73E-05	8.30E-05	1.89E-06	1.63E-06
**24h**	3.95E-04	8.82E-04	1.61E-06	2.31E-06
**36h**	2.00E-04	8.11E-05	2.24E-06	1.34E-06
**48h**	5.21E-04	3.05E-04	2.94E-07	4.41E-06
**72h**	2.74E-05	1.92E-04	6.25E-07	5.34E-06
	cgd2_1890			
**2h**	8.66E-09	3.64E-08	9.02E-06	1.03E-06
**6h**	7.84E-08	1.09E-07	8.73E-07	1.96E-06
**12h**	1.36E-07	3.21E-08	3.19E-07	8.93E-08
**24h**	5.24E-08	4.45E-08	3.70E-07	2.71E-07
**36h**	2.91E-07	1.62E-07	2.12E-06	3.15E-06
**48h**	3.31E-07	1.31E-07	2.59E-06	1.41E-06
**72h**	6.51E-07	3.93E-07	2.18E-06	1.48E-06
	cgd2_210			
**2h**	1.29E-06	4.10E-07	1.43E-07	3.92E-07
**6h**	5.12E-05	5.75E-07	3.40E-06	1.47E-06
**12h**	1.49E-07	1.05E-08	1.94E-07	2.19E-07
**24h**	1.42E-06	2.44E-07	1.37E-06	7.04E-07
**36h**	6.04E-07	4.55E-07	1.12E-06	2.57E-07
**48h**	3.89E-06	1.45E-06	1.23E-06	3.64E-07
**72h**	1.07E-06	2.49E-07	2.07E-07	2.33E-07
	cgd2_2120			
**2h**	0.00E+00	3.32E-09	0.00E+00	3.05E-08
**6h**	0.00E+00	7.47E-09	1.34E-08	2.83E-08
**12h**	3.62E-04	3.20E-04	1.06E-04	6.99E-05
**24h**	5.63E-05	6.83E-05	9.46E-05	3.42E-05

**36h**	3.73E-04	1.31E-04	2.12E-04	2.09E-04
**48h**	7.04E-04	1.41E-04	1.99E-04	1.74E-04
**72h**	9.67E-04	9.74E-05	2.18E-04	1.13E-04
	cgd2_2190			
**2h**	3.42E-04	1.17E-06	1.14E-06	1.94E-06
**6h**	7.88E-05	1.65E-06	9.22E-07	1.51E-06
**12h**	7.06E-05	5.54E-07	1.00E-06	8.16E-07
**24h**	1.01E-04	1.67E-06	2.68E-06	1.12E-06
**36h**	2.72E-05	8.37E-07	1.18E-06	1.40E-06
**48h**	9.86E-05	1.30E-06	2.51E-06	1.11E-06
**72h**	1.04E-04	6.63E-07	2.87E-07	3.84E-07
	cgd2_220			
**2h**	2.89E-04	6.05E-05	1.58E-05	3.05E-05
**6h**	7.20E-04	1.13E-04	2.12E-04	2.29E-04
**12h**	2.44E-04	1.77E-06	2.96E-05	1.64E-05
**24h**	9.31E-05	1.50E-05	1.09E-04	4.26E-05
**36h**	1.93E-04	2.60E-05	4.34E-05	4.33E-05
**48h**	4.21E-04	1.32E-05	3.36E-05	3.12E-05
**72h**	5.91E-04	1.99E-05	4.21E-05	1.79E-05
	cgd2_230			
**2h**	1.72E-06	8.10E-04	3.31E-04	6.35E-02
**6h**	3.47E-07	1.51E-04	9.79E-05	1.17E-05
**12h**	5.82E-07	1.02E-03	3.78E-04	1.03E-05
**24h**	2.95E-06	2.14E-04	5.67E-04	1.82E-06
**36h**	2.40E-07	6.34E-04	3.98E-04	1.18E-03
**48h**	6.58E-07	2.94E-04	1.78E-04	2.35E-04
**72h**	6.14E-07	3.07E-04	3.71E-04	6.52E-05
	cgd2_2300			
**2h**	3.14E-05	1.41E-05	7.58E-05	1.32E-04
**6h**	7.09E-05	2.53E-05	3.65E-05	7.75E-05
**12h**	1.72E-05	4.37E-05	8.44E-05	1.14E-04
**24h**	1.57E-05	3.85E-05	1.84E-04	4.22E-05
**36h**	8.69E-06	5.76E-05	1.73E-04	9.84E-05
**48h**	4.70E-06	5.33E-05	4.49E-04	1.38E-04
**72h**	2.08E-05	3.13E-05	1.34E-04	4.71E-05
	cgd2_2310			
**2h**	1.34E-08	2.19E-09	2.29E-09	7.78E-08
**6h**	2.22E-08	9.66E-09	9.53E-09	3.04E-08
**12h**	7.33E-08	5.84E-08	8.14E-08	9.27E-08
**24h**	8.06E-08	3.93E-08	6.12E-08	2.15E-08
**36h**	2.32E-08	2.35E-08	1.31E-07	8.78E-08
**48h**	7.04E-08	2.74E-08	5.19E-08	4.30E-08
**72h**	2.28E-08	1.68E-08	1.04E-08	3.37E-08
	cgd2_2400			
**2h**	4.32E-04	1.83E-04	1.65E-04	9.36E-04
**6h**	2.59E-05	1.48E-04	3.27E-04	5.01E-04
**12h**	3.40E-04	1.69E-04	3.11E-04	2.44E-04
**24h**	1.02E-04	1.20E-04	2.94E-04	1.58E-04
**36h**	8.28E-05	8.55E-05	2.97E-04	1.76E-04
**48h**	1.02E-04	4.16E-05	1.65E-04	1.93E-04
**72h**	4.26E-05	2.61E-05	4.00E-05	3.00E-05
	cgd2_2420			
**2h**	0.00E+00	7.92E-04	8.91E-04	4.06E-03

**6h**	6.74E-05	7.34E-04	2.63E-03	3.79E-03
**12h**	1.44E-05	1.37E-03	4.22E-03	1.52E-03
**24h**	4.36E-06	8.28E-04	2.64E-03	8.76E-04
**36h**	2.44E-06	3.59E-04	2.14E-03	1.07E-03
**48h**	6.69E-06	4.70E-04	8.42E-04	9.91E-04
**72h**	2.98E-06	6.33E-04	6.87E-04	4.35E-04
	cgd2_2440			
**2h**	3.18E-06	8.04E-07	1.04E-06	1.51E-06
**6h**	1.28E-06	8.75E-07	1.65E-06	1.61E-06
**12h**	4.58E-06	1.05E-06	6.00E-06	6.17E-06
**24h**	3.25E-06	1.69E-06	1.53E-06	2.09E-06
**36h**	1.27E-06	7.53E-07	3.11E-06	2.52E-06
**48h**	2.64E-06	7.74E-07	1.06E-06	1.26E-06
**72h**	1.30E-06	4.32E-07	4.85E-07	4.19E-07
	cgd2_2560			
**2h**	2.82E-03	2.38E-04	3.09E-04	1.03E-03
**6h**	2.01E-03	1.50E-04	2.32E-04	4.65E-04
**12h**	8.56E-04	1.18E-05	2.36E-04	1.09E-04
**24h**	2.64E-04	4.62E-05	2.14E-04	9.80E-05
**36h**	1.36E-04	1.03E-04	3.47E-04	2.04E-04
**48h**	1.20E-03	3.66E-05	1.06E-04	1.41E-04
**72h**	4.68E-04	2.37E-05	2.25E-05	2.06E-05
	cgd2_270			
**2h**	1.63E-05	1.47E-05	1.11E-05	5.72E-06
**6h**	1.63E-05	1.85E-05	1.11E-05	4.22E-05
**12h**	1.99E-06	1.55E-06	6.81E-06	5.08E-06
**24h**	6.53E-06	4.67E-06	1.27E-05	1.75E-05
**36h**	8.04E-06	3.42E-06	3.73E-06	4.26E-06
**48h**	4.57E-06	1.81E-06	5.78E-06	1.28E-06
**72h**	1.26E-06	4.30E-07	7.35E-07	4.26E-06
	cgd2_2850			
**2h**	1.49E-07	3.34E-08	0.00E+00	1.15E-08
**6h**	3.88E-07	8.87E-08	5.45E-08	1.81E-08
**12h**	3.98E-08	2.63E-08	7.64E-08	4.16E-08
**24h**	5.21E-08	1.84E-08	5.23E-08	1.52E-07
**36h**	8.54E-08	4.32E-08	7.65E-08	1.28E-07
**48h**	5.68E-07	1.37E-07	3.37E-07	1.29E-07
**72h**	8.44E-08	6.02E-08	1.40E-07	3.59E-07
	cgd2_2900			
**2h**	6.13E-09	2.57E-09	0.00E+00	1.73E-08
**6h**	2.09E-08	5.24E-09	1.96E-08	5.94E-08
**12h**	2.29E-06	4.39E-07	6.81E-07	1.16E-06
**24h**	5.15E-07	7.79E-08	3.07E-07	5.52E-07
**36h**	6.50E-07	2.94E-07	1.64E-06	7.00E-06
**48h**	4.21E-07	1.65E-07	6.11E-07	4.87E-07
**72h**	1.16E-06	4.57E-07	1.72E-06	1.35E-06
	cgd2_2950			
**2h**	1.07E-03	6.82E-05	4.86E-04	1.06E-03
**6h**	1.56E-03	5.71E-05	8.42E-04	1.60E-04
**12h**	1.64E-04	3.47E-06	1.85E-04	1.87E-04
**24h**	1.44E-04	6.84E-06	2.78E-04	7.69E-04
**36h**	1.91E-04	8.54E-05	3.31E-04	7.36E-03
**48h**	2.28E-04	2.78E-05	2.75E-04	5.46E-04

**72h**	8.61E-05	3.81E-05	7.04E-05	3.84E-06
	cgd2_300			
**2h**	5.12E-06	1.03E-06	1.78E-06	1.35E-05
**6h**	1.48E-05	4.48E-06	7.32E-06	3.90E-05
**12h**	2.74E-07	2.49E-08	2.20E-06	1.01E-05
**24h**	2.20E-06	6.52E-07	6.50E-06	7.79E-06
**36h**	2.19E-06	2.14E-06	2.16E-06	9.85E-06
**48h**	3.03E-06	1.17E-06	1.99E-06	1.92E-06
**72h**	4.67E-07	4.83E-07	8.56E-07	1.56E-06
	cgd2_310			
**2h**	4.60E-05	6.84E-06	1.12E-05	1.45E-04
**6h**	1.05E-05	1.54E-06	2.54E-06	1.28E-04
**12h**	2.28E-05	8.17E-07	2.51E-05	1.94E-05
**24h**	1.34E-05	2.41E-06	1.24E-05	1.71E-05
**36h**	6.00E-06	3.70E-06	1.27E-05	1.08E-05
**48h**	1.92E-05	4.95E-06	2.45E-05	1.52E-05
**72h**	2.58E-05	2.26E-06	7.30E-06	3.11E-05
	cgd2_3120			
**2h**	1.63E-06	1.00E-06	1.18E-06	1.25E-06
**6h**	3.64E-07	1.60E-07	4.33E-07	7.43E-07
**12h**	7.67E-07	2.92E-07	4.60E-07	1.40E-06
**24h**	7.78E-07	3.13E-07	1.74E-07	8.68E-07
**36h**	1.88E-07	1.57E-07	2.69E-07	6.42E-07
**48h**	4.47E-07	3.75E-07	2.46E-07	4.01E-07
**72h**	3.24E-07	3.57E-07	7.69E-07	5.01E-07
	cgd2_3410			
**2h**	2.16E-06	6.07E-07	5.44E-07	1.12E-06
**6h**	1.16E-05	7.84E-06	2.45E-05	5.15E-05
**12h**	4.13E-07	3.03E-07	1.21E-06	1.90E-06
**24h**	4.95E-07	7.17E-07	6.54E-06	2.11E-05
**36h**	1.31E-06	1.92E-06	2.74E-06	4.06E-06
**48h**	2.73E-06	3.77E-06	5.50E-06	1.27E-05
**72h**	1.22E-06	1.61E-06	5.06E-06	2.89E-06
	cgd2_3520			
**2h**	1.18E-07	3.62E-07	2.71E-07	1.68E-07
**6h**	5.46E-08	2.39E-08	4.90E-09	7.00E-09
**12h**	9.45E-09	3.39E-08	2.03E-08	1.10E-08
**24h**	1.19E-07	8.45E-09	1.33E-08	3.78E-08
**36h**	2.72E-08	9.70E-08	4.31E-08	7.17E-08
**48h**	1.99E-08	1.83E-08	3.15E-08	9.60E-08
**72h**	2.44E-08	9.30E-08	3.87E-08	4.47E-07
	cgd2_360			
**2h**	6.92E-06	4.72E-06	3.45E-06	3.69E-06
**6h**	1.90E-04	1.74E-05	2.31E-05	3.65E-05
**12h**	1.66E-06	2.84E-07	5.13E-06	3.59E-06
**24h**	3.96E-06	3.50E-06	9.42E-06	7.06E-06
**36h**	2.00E-06	2.60E-06	2.41E-06	3.66E-06
**48h**	1.10E-06	1.04E-06	3.41E-06	2.12E-06
**72h**	6.10E-07	6.68E-07	4.82E-07	8.03E-07
	cgd2_3740			
**2h**	7.39E-07	1.80E-07	1.19E-07	4.52E-07
**6h**	3.27E-08	3.83E-08	4.42E-08	1.44E-07
**12h**	3.47E-07	1.91E-07	7.99E-07	7.36E-07

**24h**	3.02E-07	6.94E-07	1.65E-07	1.98E-07
**36h**	4.94E-07	4.94E-07	1.25E-06	1.51E-06
**48h**	1.86E-07	3.11E-07	4.04E-07	1.08E-06
**72h**	2.74E-07	6.01E-07	6.49E-07	7.02E-07
	cgd2_3940			
**2h**	0.00E+00	0.00E+00	0.00E+00	0.00E+00
**6h**	3.47E-07	0.00E+00	0.00E+00	0.00E+00
**12h**	1.41E-06	1.43E-05	6.42E-06	2.05E-05
**24h**	4.94E-06	6.12E-06	7.83E-06	8.14E-06
**36h**	7.86E-06	3.21E-05	3.33E-05	1.90E-04
**48h**	6.59E-05	1.68E-04	1.55E-04	3.76E-04
**72h**	2.15E-05	8.54E-05	1.02E-04	1.36E-04
	cgd2_40			
**2h**	1.30E-06	9.68E-07	0.00E+00	4.46E-07
**6h**	2.19E-06	3.52E-06	6.39E-06	1.93E-05
**12h**	3.13E-06	5.17E-07	4.44E-08	1.44E-07
**24h**	6.11E-07	6.51E-07	3.67E-06	7.51E-06
**36h**	3.19E-06	4.13E-06	6.49E-06	6.92E-06
**48h**	1.89E-06	3.53E-06	5.69E-06	5.68E-06
**72h**	8.40E-06	9.09E-06	1.75E-05	5.79E-06
	cgd2_410			
**2h**	6.33E-05	6.14E-05	3.86E-05	3.96E-05
**6h**	4.69E-05	6.12E-05	2.45E-05	4.78E-05
**12h**	2.11E-06	2.50E-06	4.52E-06	6.47E-06
**24h**	4.59E-06	3.67E-06	8.95E-06	6.86E-06
**36h**	2.71E-06	3.52E-06	3.37E-06	3.79E-06
**48h**	1.04E-06	1.57E-06	3.05E-06	2.60E-06
**72h**	1.22E-07	3.36E-07	1.56E-07	2.46E-07
	cgd2_4120			
**2h**	0.00E+00	3.30E-06	2.64E-05	1.56E-05
**6h**	1.38E-03	1.79E-03	2.47E-03	3.08E-03
**12h**	1.54E-04	6.84E-04	5.50E-04	6.82E-04
**24h**	6.23E-05	1.08E-04	7.51E-04	4.04E-04
**36h**	2.00E-04	4.16E-04	8.32E-04	7.05E-04
**48h**	1.15E-04	2.68E-04	5.03E-04	4.14E-04
**72h**	6.78E-05	2.12E-04	2.96E-04	1.37E-04
	cgd2_4140			
**2h**	7.53E-02	1.45E-04	4.31E-05	2.94E-06
**6h**	3.89E-04	2.23E-04	7.33E-04	1.59E-06
**12h**	3.10E-04	3.65E-04	2.30E-04	6.10E-04
**24h**	9.72E-05	3.59E-05	2.22E-04	5.64E-06
**36h**	1.46E-04	1.15E-04	1.82E-04	4.14E-06
**48h**	1.98E-04	9.24E-05	1.42E-04	6.83E-06
**72h**	1.06E-04	2.75E-04	1.28E-04	9.76E-07
	cgd2_4170			
**2h**	1.58E-06	8.72E-07	1.15E-06	2.53E-06
**6h**	5.26E-07	2.46E-07	4.06E-07	8.09E-07
**12h**	2.59E-06	2.49E-06	7.50E-06	3.96E-06
**24h**	3.61E-07	1.23E-06	7.40E-07	8.69E-07
**36h**	4.09E-07	9.51E-07	1.58E-06	1.56E-06
**48h**	2.99E-07	3.13E-07	9.48E-07	1.11E-06
**72h**	1.17E-07	1.40E-06	7.43E-07	6.12E-07
	cgd2_420			

**2h**	7.32E-06	6.09E-06	1.34E-05	9.23E-06
**6h**	8.28E-06	3.95E-06	1.70E-06	3.77E-06
**12h**	7.55E-06	9.94E-06	1.07E-05	7.31E-06
**24h**	1.08E-05	1.38E-05	5.79E-06	8.47E-06
**36h**	2.78E-06	4.10E-06	5.37E-06	6.90E-06
**48h**	2.20E-06	4.98E-06	5.40E-06	5.70E-06
**72h**	1.70E-06	2.15E-06	1.62E-06	1.58E-06
	cgd2_430			
**2h**	4.86E-06	2.73E-06	7.60E-06	1.03E-05
**6h**	3.16E-06	1.54E-06	5.24E-07	2.48E-06
**12h**	1.56E-06	2.68E-06	1.30E-06	2.05E-06
**24h**	1.14E-06	1.14E-06	5.08E-07	1.07E-06
**36h**	8.06E-07	7.38E-07	7.22E-07	1.05E-06
**48h**	6.75E-07	6.38E-07	5.29E-07	8.52E-07
**72h**	7.86E-07	1.20E-06	8.14E-07	6.49E-07
	cgd2_450			
**2h**	2.99E-06	4.43E-06	3.74E-06	2.06E-06
**6h**	2.15E-05	1.68E-05	6.36E-06	7.45E-06
**12h**	4.41E-07	2.50E-07	1.30E-06	1.18E-06
**24h**	6.15E-07	6.66E-07	1.40E-06	1.38E-06
**36h**	7.91E-07	8.23E-07	1.44E-06	1.34E-06
**48h**	4.02E-07	1.06E-06	8.48E-07	5.44E-07
**72h**	1.48E-07	1.23E-07	3.06E-07	9.64E-08
	cgd2_480			
**2h**	0.00E+00	0.00E+00	0.00E+00	4.93E-09
**6h**	2.82E-07	1.60E-07	2.38E-07	2.17E-07
**12h**	1.31E-07	5.20E-08	8.15E-08	7.65E-08
**24h**	1.77E-08	5.68E-08	1.62E-07	9.88E-08
**36h**	3.57E-07	3.24E-07	4.54E-07	5.38E-07
**48h**	9.12E-08	1.09E-07	1.96E-07	1.88E-07
**72h**	4.43E-07	5.41E-07	8.28E-07	4.88E-07
	cgd2_490			
**2h**	0.00E+00	2.41E-09	0.00E+00	2.02E-08
**6h**	0.00E+00	0.00E+00	1.69E-08	1.52E-08
**12h**	0.00E+00	0.00E+00	0.00E+00	0.00E+00
**24h**	3.32E-09	8.05E-09	3.05E-08	2.57E-08
**36h**	1.70E-08	5.19E-08	4.63E-08	1.13E-07
**48h**	1.98E-06	2.52E-06	2.83E-05	5.14E-06
**72h**	6.11E-06	1.09E-05	4.15E-05	1.58E-05
	cgd2_50			
**2h**	5.09E-03	8.94E-04	2.10E-04	7.46E-05
**6h**	1.20E-03	2.01E-03	3.20E-04	5.77E-04
**12h**	1.28E-03	1.47E-03	1.04E-04	1.66E-03
**24h**	4.21E-03	1.11E-03	3.10E-05	3.89E-05
**36h**	8.19E-04	1.02E-03	4.23E-05	1.98E-04
**48h**	1.41E-03	1.89E-03	1.20E-04	7.61E-05
**72h**	8.29E-04	2.25E-04	6.91E-06	4.94E-05
	cgd2_610			
**2h**	0.00E+00	0.00E+00	0.00E+00	0.00E+00
**6h**	0.00E+00	2.58E-08	6.78E-08	3.61E-06
**12h**	8.00E-07	7.25E-06	2.45E-05	4.09E-05
**24h**	5.14E-07	2.08E-06	6.96E-06	3.87E-06
**36h**	1.77E-06	1.70E-05	2.38E-05	2.85E-05

**48h**	2.28E-06	3.83E-05	2.77E-05	1.89E-05
**72h**	5.87E-07	1.09E-05	3.81E-05	1.16E-05
	cgd2_620			
**2h**	8.04E-07	1.86E-06	4.55E-07	3.96E-06
**6h**	3.53E-05	9.33E-05	4.97E-04	5.20E-04
**12h**	1.91E-06	7.42E-06	4.61E-05	2.33E-05
**24h**	3.68E-06	1.05E-05	1.01E-04	9.58E-05
**36h**	1.20E-05	3.41E-05	4.71E-05	3.03E-05
**48h**	5.54E-06	2.10E-05	8.20E-05	5.37E-05
**72h**	1.00E-06	5.40E-06	7.00E-06	3.59E-06
	cgd2_640			
**2h**	0.00E+00	1.18E-08	0.00E+00	1.58E-09
**6h**	0.00E+00	0.00E+00	0.00E+00	0.00E+00
**12h**	1.26E-05	3.36E-05	1.71E-05	3.23E-05
**24h**	1.79E-06	2.79E-06	6.17E-06	4.30E-06
**36h**	2.65E-06	7.06E-06	1.06E-05	1.10E-05
**48h**	1.24E-06	1.29E-06	6.79E-06	6.25E-06
**72h**	4.24E-07	6.93E-07	8.66E-07	4.32E-07
	cgd2_70			
**2h**	1.23E-06	1.15E-06	9.35E-07	1.62E-06
**6h**	2.89E-07	3.56E-07	4.55E-07	4.82E-07
**12h**	2.41E-06	3.23E-04	2.03E-06	2.96E-06
**24h**	1.37E-06	1.68E-06	1.40E-06	2.82E-06
**36h**	9.86E-07	5.66E-07	3.56E-07	5.87E-07
**48h**	1.12E-06	5.99E-07	2.37E-06	1.77E-06
**72h**	8.28E-06	4.97E-06	6.74E-06	6.00E-06
	cgd2_720			
**2h**	2.57E-04	2.21E-03	1.38E-05	8.74E-04
**6h**	8.22E-05	5.42E-04	1.08E-03	1.84E-05
**12h**	1.68E-05	2.26E-04	4.63E-04	1.43E-04
**24h**	7.11E-05	5.38E-04	5.54E-04	5.61E-04
**36h**	3.31E-05	4.73E-04	5.94E-04	3.50E-04
**48h**	5.70E-05	5.40E-04	2.23E-04	3.80E-04
**72h**	1.78E-05	6.53E-05	3.07E-04	2.62E-05
	cgd2_730			
**2h**	1.34E-03	5.41E-06	0.00E+00	3.82E-06
**6h**	9.42E-04	5.52E-05	4.71E-06	5.54E-06
**12h**	7.19E-05	4.87E-06	1.26E-06	2.07E-06
**24h**	6.95E-04	1.85E-05	3.72E-06	4.58E-06
**36h**	1.26E-04	4.84E-05	8.85E-07	1.25E-06
**48h**	7.69E-04	1.17E-04	1.25E-06	3.20E-06
**72h**	1.24E-03	3.23E-05	1.60E-06	2.30E-06
	cgd2_740			
**2h**	2.85E-08	5.59E-08	3.58E-07	1.76E-06
**6h**	0.00E+00	3.41E-09	1.75E-09	5.28E-07
**12h**	3.67E-06	1.48E-05	9.11E-05	9.62E-05
**24h**	2.65E-06	3.00E-06	8.45E-06	9.60E-06
**36h**	2.60E-06	4.23E-06	8.04E-05	1.04E-04
**48h**	3.61E-06	2.39E-06	1.30E-05	1.47E-05
**72h**	1.28E-06	1.37E-06	1.54E-05	1.12E-05
	cgd2_750			
**2h**	4.82E-08	1.20E-07	7.04E-07	5.32E-07
**6h**	2.98E-06	2.74E-06	4.49E-05	1.98E-04

**12h**	6.67E-08	2.13E-08	6.48E-06	2.23E-05
**24h**	1.74E-07	4.42E-07	3.10E-05	6.01E-05
**36h**	6.47E-07	2.28E-06	8.42E-06	1.72E-05
**48h**	1.05E-06	2.35E-06	2.70E-05	7.10E-05
**72h**	1.30E-06	2.59E-06	2.88E-05	2.77E-05
	cgd2_760			
**2h**	1.03E-08	2.70E-09	3.14E-08	2.32E-08
**6h**	3.51E-07	1.18E-07	5.99E-07	1.35E-06
**12h**	2.63E-08	1.37E-08	3.40E-08	1.90E-07
**24h**	1.69E-07	1.77E-08	2.25E-07	2.43E-07
**36h**	1.32E-07	2.07E-07	4.26E-07	2.75E-07
**48h**	3.64E-07	1.14E-07	4.04E-07	7.00E-07
**72h**	1.89E-07	2.00E-07	1.74E-07	1.47E-07
	cgd2_770			
**2h**	1.55E-07	8.57E-08	2.03E-05	1.64E-06
**6h**	2.98E-07	7.46E-08	5.24E-07	1.46E-06
**12h**	1.25E-08	4.81E-08	1.42E-07	1.82E-07
**24h**	1.35E-07	3.16E-08	3.31E-07	4.73E-07
**36h**	2.86E-07	2.56E-07	9.74E-07	1.28E-06
**48h**	1.68E-07	1.49E-07	8.74E-07	2.11E-06
**72h**	1.93E-07	1.96E-07	6.46E-07	6.68E-07
	cgd2_810			
**2h**	1.54E-07	3.06E-08	1.15E-08	4.53E-09
**6h**	9.75E-06	8.25E-08	4.95E-07	7.51E-08
**12h**	2.24E-05	1.53E-05	8.23E-06	3.03E-06
**24h**	1.46E-06	1.28E-06	1.94E-06	6.91E-07
**36h**	2.54E-06	1.92E-06	5.60E-06	3.60E-06
**48h**	1.85E-06	1.14E-06	1.33E-06	1.61E-06
**72h**	3.45E-07	3.41E-07	2.68E-07	3.76E-07
	cgd2_820			
**2h**	8.37E-03	6.43E-05	1.47E-05	1.15E-05
**6h**	5.88E-05	3.28E-05	4.06E-05	3.03E-05
**12h**	3.49E-05	1.23E-05	3.05E-05	8.26E-06
**24h**	1.19E-04	2.21E-05	5.89E-05	1.80E-05
**36h**	1.95E-05	1.21E-05	2.33E-05	1.72E-05
**48h**	3.59E-05	1.30E-05	1.43E-05	1.33E-05
**72h**	8.26E-06	3.50E-06	5.64E-06	2.04E-06
	cgd2_830			
**2h**	0.00E+00	1.39E-07	1.31E-08	5.69E-08
**6h**	8.47E-06	3.57E-05	6.15E-07	4.66E-07
**12h**	1.40E-06	1.76E-08	5.82E-08	9.00E-08
**24h**	7.15E-07	2.70E-07	5.95E-07	3.51E-07
**36h**	2.06E-06	8.05E-07	6.02E-07	6.11E-07
**48h**	7.09E-06	6.83E-07	5.53E-07	1.20E-06
**72h**	4.54E-06	9.06E-07	4.76E-07	4.08E-07
	cgd2_900			
**2h**	1.50E-06	2.21E-07	9.70E-09	1.28E-07
**6h**	5.65E-06	4.48E-06	3.35E-06	1.99E-06
**12h**	2.70E-08	6.73E-08	2.47E-07	1.14E-07
**24h**	7.41E-07	4.02E-07	5.98E-07	6.54E-07
**36h**	1.20E-06	5.86E-07	2.96E-07	5.09E-07
**48h**	2.12E-06	3.97E-07	1.52E-07	3.68E-07
**72h**	2.05E-06	2.95E-07	4.06E-07	7.35E-07

	cgd2_910			
**2h**	0.00E+00	1.07E-05	1.01E-05	6.90E-06
**6h**	1.80E-07	6.42E-06	2.83E-03	1.53E-04
**12h**	8.72E-08	3.12E-05	6.77E-04	9.77E-05
**24h**	4.37E-08	3.87E-05	2.12E-04	5.47E-04
**36h**	3.88E-07	1.47E-04	1.10E-04	1.41E-03
**48h**	6.17E-07	6.53E-05	1.19E-04	7.88E-05
**72h**	8.66E-07	1.54E-04	4.13E-04	6.70E-03
	cgd2_980			
**2h**	3.02E-06	2.40E-06	6.76E-07	1.67E-06
**6h**	8.10E-07	2.44E-06	7.01E-07	4.88E-07
**12h**	5.73E-07	3.45E-07	9.97E-07	1.34E-06
**24h**	1.09E-06	1.31E-06	1.52E-06	3.89E-07
**36h**	5.02E-07	4.78E-07	7.79E-07	8.89E-07
**48h**	1.86E-06	3.74E-07	2.90E-07	8.82E-07
**72h**	8.95E-07	2.28E-07	4.90E-07	4.17E-07
	cgd2_990			
**2h**	4.39E-05	1.35E-05	1.13E-06	9.57E-06
**6h**	1.23E-05	3.47E-05	1.44E-05	2.14E-05
**12h**	4.47E-06	4.01E-06	1.01E-05	1.56E-05
**24h**	4.02E-06	1.11E-05	1.13E-05	6.75E-06
**36h**	4.39E-06	6.52E-06	8.88E-06	2.51E-05
**48h**	6.74E-06	3.40E-06	1.60E-06	6.53E-06
**72h**	1.39E-06	1.76E-06	1.09E-06	4.38E-06
	cgd3_1020			
**2h**	1.62E-04	9.49E-07	4.92E-07	5.65E-07
**6h**	1.91E-07	2.20E-07	1.87E-07	1.95E-07
**12h**	2.44E-07	9.91E-08	2.85E-07	2.24E-07
**24h**	3.40E-07	2.18E-07	2.21E-07	2.87E-07
**36h**	1.89E-07	1.36E-07	1.44E-07	1.90E-07
**48h**	3.86E-07	8.28E-08	6.92E-08	2.39E-07
**72h**	4.89E-08	4.74E-08	6.86E-08	6.63E-08
	cgd3_1410			
**2h**	4.25E-04	2.73E-04	4.63E-04	2.45E-04
**6h**	2.78E-03	2.10E-03	7.33E-04	4.84E-04
**12h**	1.96E-03	4.01E-04	3.26E-04	3.98E-04
**24h**	1.01E-03	4.29E-04	6.16E-04	4.18E-04
**36h**	1.19E-03	2.60E-04	5.84E-04	5.44E-04
**48h**	9.14E-04	7.03E-04	9.24E-04	6.48E-04
**72h**	4.98E-04	2.34E-04	3.93E-04	3.16E-04
	cgd3_150			
**2h**	0.00E+00	1.95E-09	0.00E+00	0.00E+00
**6h**	1.50E-07	2.67E-07	6.40E-07	4.69E-07
**12h**	3.28E-07	5.21E-07	1.07E-06	3.21E-06
**24h**	8.47E-07	1.65E-06	4.19E-06	1.70E-06
**36h**	1.39E-06	1.89E-06	2.08E-06	2.43E-06
**48h**	5.38E-06	1.56E-06	1.81E-06	1.36E-06
**72h**	2.59E-06	2.35E-06	2.48E-06	2.33E-06
	cgd3_2260			
**2h**	1.55E-08	5.77E-09	0.00E+00	4.22E-08
**6h**	5.48E-08	1.68E-08	7.96E-10	1.17E-08
**12h**	2.04E-05	9.35E-06	9.81E-06	2.12E-05
**24h**	3.28E-06	1.82E-06	1.46E-06	1.07E-06

**36h**	4.04E-06	2.15E-06	5.30E-06	1.14E-05
**48h**	1.01E-06	4.34E-07	8.98E-07	6.08E-07
**72h**	2.35E-07	3.87E-07	2.44E-07	3.24E-07
	cgd3_2420			
**2h**	3.26E-06	4.76E-05	3.41E-06	3.14E-06
**6h**	1.09E-07	0.00E+00	0.00E+00	1.39E-07
**12h**	0.00E+00	0.00E+00	0.00E+00	5.90E-08
**24h**	1.76E-05	9.61E-05	1.06E-04	3.39E-06
**36h**	2.10E-05	1.36E-04	1.11E-04	5.53E-05
**48h**	2.67E-04	7.51E-04	2.05E-04	1.48E-04
**72h**	1.11E-04	6.66E-05	2.75E-04	3.80E-05
	cgd3_2830			
**2h**	1.33E-03	1.09E-03	1.96E-03	7.73E-04
**6h**	3.48E-03	2.18E-03	9.70E-03	1.14E-03
**12h**	1.44E-03	4.88E-04	2.21E-03	1.50E-03
**24h**	1.67E-03	2.03E-03	3.53E-03	1.66E-03
**36h**	9.91E-04	1.23E-03	1.88E-03	1.05E-03
**48h**	6.60E-04	7.56E-04	8.05E-04	4.69E-04
**72h**	1.18E-04	2.39E-04	2.12E-04	5.30E-05
	cgd3_3220			
**2h**	4.64E-07	4.27E-07	2.41E-07	3.81E-07
**6h**	1.60E-06	9.22E-07	1.85E-06	1.57E-05
**12h**	5.29E-07	9.76E-08	4.39E-06	1.27E-06
**24h**	2.82E-06	2.87E-06	1.32E-05	1.87E-05
**36h**	1.10E-05	1.70E-05	2.85E-05	1.37E-05
**48h**	7.46E-06	3.67E-06	1.72E-05	6.70E-06
**72h**	1.62E-06	2.82E-06	5.15E-06	2.72E-06
	cgd3_4300			
**2h**	5.60E-09	1.04E-09	0.00E+00	1.31E-09
**6h**	2.17E-08	6.23E-09	2.76E-08	2.82E-08
**12h**	8.73E-08	1.40E-08	8.45E-09	8.26E-09
**24h**	2.19E-08	1.67E-09	3.82E-09	9.42E-09
**36h**	7.05E-09	8.25E-09	9.53E-09	7.38E-09
**48h**	3.78E-09	5.69E-09	1.86E-08	1.10E-08
**72h**	4.47E-09	1.30E-08	8.46E-09	3.00E-09
	cgd3_550			
**2h**	9.46E-05	2.79E-05	1.18E-05	1.13E-05
**6h**	2.39E-05	7.62E-06	1.07E-05	5.40E-05
**12h**	1.64E-05	8.94E-06	1.99E-05	4.10E-05
**24h**	3.30E-05	8.31E-06	1.91E-05	1.80E-05
**36h**	8.93E-06	5.27E-06	7.29E-06	3.00E-04
**48h**	8.44E-06	1.26E-06	8.56E-06	3.90E-06
**72h**	1.72E-06	1.17E-06	1.87E-06	1.24E-06
	cgd3_650			
**2h**	1.04E-03	4.07E-06	8.45E-07	1.26E-06
**6h**	2.49E-06	8.46E-07	6.64E-07	4.00E-06
**12h**	1.48E-06	4.41E-07	7.46E-07	8.69E-07
**24h**	1.26E-06	3.41E-07	1.34E-06	1.25E-06
**36h**	1.14E-06	5.65E-07	1.14E-06	1.27E-06
**48h**	2.26E-06	2.73E-07	9.47E-07	4.14E-07
**72h**	7.80E-07	1.09E-07	1.55E-07	2.81E-08
	cgd3_690			
**2h**	1.27E-04	2.98E-07	4.04E-08	2.91E-07

**6h**	2.23E-06	1.58E-06	3.25E-06	4.51E-06
**12h**	7.15E-08	1.46E-07	3.54E-07	8.56E-07
**24h**	7.32E-07	3.91E-07	3.67E-06	2.61E-06
**36h**	1.52E-06	9.53E-07	1.06E-06	1.71E-06
**48h**	1.08E-06	4.96E-07	1.30E-06	6.62E-07
**72h**	2.35E-07	2.82E-07	2.71E-07	1.00E-07
	cgd4_1010			
**2h**	5.82E-06	6.47E-07	1.51E-06	1.24E-06
**6h**	2.89E-07	3.01E-07	3.38E-07	2.90E-06
**12h**	2.58E-06	4.07E-06	2.22E-06	3.62E-06
**24h**	1.79E-06	1.35E-06	1.17E-06	1.57E-06
**36h**	4.01E-07	4.99E-07	5.26E-07	3.93E-07
**48h**	7.90E-07	1.46E-07	3.27E-07	2.59E-07
**72h**	1.66E-07	1.19E-07	4.67E-08	3.60E-08
	cgd4_1020			
**2h**	7.01E-06	9.17E-07	4.24E-06	3.67E-01
**6h**	8.76E-07	4.20E-07	2.71E-06	1.99E-04
**12h**	3.52E-04	4.31E-06	1.70E-05	3.34E-03
**24h**	2.85E-06	2.10E-06	3.06E-06	3.99E-03
**36h**	4.21E-07	4.82E-07	8.98E-07	4.37E-04
**48h**	1.30E-06	2.77E-07	3.72E-07	1.62E-04
**72h**	2.64E-07	1.12E-07	1.96E-07	1.02E-04
	cgd4_1030			
**2h**	4.41E-06	8.80E-07	3.56E-06	1.76E-06
**6h**	1.33E-05	2.70E-06	5.93E-06	1.47E-05
**12h**	1.01E-06	4.38E-07	3.17E-06	5.59E-06
**24h**	4.27E-06	1.67E-06	2.68E-06	7.79E-06
**36h**	2.36E-06	1.86E-06	1.58E-06	1.14E-06
**48h**	4.54E-06	5.29E-07	1.62E-06	1.24E-06
**72h**	7.61E-07	2.79E-07	4.88E-07	2.70E-07
	cgd4_1080			
**2h**	0.00E+00	1.18E-05	3.81E-06	4.17E-05
**6h**	5.13E-05	6.90E-05	2.89E-05	2.11E-04
**12h**	1.54E-06	4.59E-06	1.72E-05	7.72E-05
**24h**	6.02E-06	3.85E-05	5.56E-05	4.76E-05
**36h**	1.54E-05	2.68E-05	3.96E-05	5.33E-05
**48h**	3.21E-06	5.83E-05	7.98E-05	4.38E-05
**72h**	2.12E-05	6.81E-05	2.04E-05	1.76E-05
	cgd4_1090			
**2h**	1.01E-06	2.83E-06	1.89E-06	1.46E-04
**6h**	1.22E-07	0.00E+00	4.36E-05	2.67E-05
**12h**	1.68E-07	1.99E-05	2.34E-05	1.36E-04
**24h**	2.52E-07	1.94E-04	5.87E-05	1.16E-04
**36h**	5.01E-07	3.40E-05	1.73E-05	4.16E-05
**48h**	4.77E-07	8.33E-06	2.89E-05	1.04E-05
**72h**	8.66E-07	2.68E-05	1.38E-04	4.51E-05
	cgd5_3070			
**2h**	2.14E-09	0.00E+00	0.00E+00	2.42E-09
**6h**	9.56E-10	6.58E-09	0.00E+00	6.81E-08
**12h**	0.00E+00	0.00E+00	0.00E+00	3.16E-08
**24h**	3.07E-09	2.40E-09	6.42E-09	3.27E-09
**36h**	5.35E-08	5.06E-09	2.53E-08	1.13E-08
**48h**	7.31E-06	2.16E-06	4.41E-06	3.02E-06

**72h**	2.11E-06	1.67E-06	1.15E-06	5.71E-06
	cgd5_3160			
**2h**	6.41E-06	2.37E-07	1.08E-08	1.09E-07
**6h**	2.51E-08	1.41E-08	7.03E-09	2.20E-09
**12h**	2.28E-03	2.80E-03	1.02E-03	6.51E-04
**24h**	4.92E-04	5.72E-04	6.02E-05	4.77E-05
**36h**	7.46E-05	1.02E-04	1.51E-04	2.42E-04
**48h**	1.30E-04	9.10E-05	2.93E-05	6.60E-05
**72h**	1.13E-05	1.91E-05	6.58E-06	7.17E-06
	cgd5_3170			
**2h**	2.08E-05	5.16E-06	0.00E+00	3.23E-07
**6h**	1.67E-05	2.29E-04	6.47E-05	6.44E-07
**12h**	1.20E-02	9.69E-03	6.03E-03	1.22E-03
**24h**	8.33E-03	1.25E-03	2.59E-03	4.40E-04
**36h**	1.33E-02	2.66E-03	4.81E-03	1.57E-03
**48h**	1.68E-02	6.10E-03	8.68E-03	4.93E-03
**72h**	1.14E-02	6.48E-03	6.30E-03	2.79E-03
	cgd5_3210			
**2h**	2.16E-04	0.00E+00	0.00E+00	0.00E+00
**6h**	0.00E+00	0.00E+00	0.00E+00	0.00E+00
**12h**	4.66E-04	2.43E-04	1.49E-04	3.37E-05
**24h**	1.19E-04	1.08E-04	9.94E-05	1.99E-05
**36h**	9.46E-05	1.59E-04	4.24E-04	7.98E-05
**48h**	1.42E-04	7.88E-05	3.66E-04	4.70E-05
**72h**	2.85E-04	3.74E-05	1.34E-04	9.56E-05
	cgd5_3220			
**2h**	0.00E+00	0.00E+00	0.00E+00	0.00E+00
**6h**	2.50E-06	2.86E-06	2.95E-06	1.26E-06
**12h**	1.52E-07	5.79E-07	8.45E-07	1.59E-07
**24h**	5.27E-07	7.83E-07	1.15E-06	5.28E-07
**36h**	1.00E-06	1.53E-06	1.65E-06	1.56E-06
**48h**	2.21E-06	1.52E-06	1.50E-06	1.19E-06
**72h**	2.90E-07	3.35E-07	3.92E-07	5.33E-07
	cgd5_3270			
**2h**	6.08E-09	0.00E+00	0.00E+00	5.75E-10
**6h**	0.00E+00	4.95E-09	7.37E-10	0.00E+00
**12h**	1.74E-07	5.41E-07	1.17E-07	2.71E-08
**24h**	1.60E-07	7.27E-08	1.83E-08	9.53E-09
**36h**	2.48E-07	3.71E-07	1.81E-07	1.35E-07
**48h**	6.21E-07	2.80E-07	2.38E-07	1.90E-07
**72h**	6.12E-07	3.75E-07	3.65E-07	3.34E-07
	cgd5_3280			
**2h**	4.33E-05	1.84E-05	7.78E-05	5.13E-04
**6h**	2.31E-05	2.79E-05	2.24E-04	5.37E-03
**12h**	2.11E-06	5.20E-06	1.83E-05	1.86E-03
**24h**	1.10E-05	6.41E-06	3.98E-04	1.89E-03
**36h**	2.86E-05	4.68E-06	7.08E-05	3.03E-03
**48h**	1.14E-05	5.66E-06	1.06E-04	2.90E-03
**72h**	1.86E-05	3.50E-06	2.83E-04	1.91E-03
	cgd5_3290			
**2h**	6.44E-08	4.72E-08	2.43E-08	3.45E-08
**6h**	2.32E-09	2.79E-09	3.84E-09	7.61E-09
**12h**	4.65E-09	1.72E-08	8.52E-09	6.90E-10

**24h**	8.97E-09	6.84E-09	2.12E-08	3.99E-09
**36h**	2.20E-08	8.85E-09	9.54E-09	6.08E-09
**48h**	2.82E-08	1.76E-08	2.65E-08	2.14E-08
**72h**	3.13E-08	3.72E-08	2.03E-08	2.04E-08
	cgd5_3340			
**2h**	2.52E-05	4.82E-05	1.15E-05	1.47E-05
**6h**	1.13E-05	3.11E-05	1.83E-05	7.74E-06
**12h**	5.62E-06	3.24E-05	1.97E-05	5.71E-06
**24h**	1.85E-05	2.52E-05	2.03E-05	8.95E-06
**36h**	1.69E-05	1.68E-05	8.47E-06	6.05E-06
**48h**	1.00E-05	6.71E-06	1.42E-05	6.72E-06
**72h**	6.38E-06	4.29E-06	3.07E-06	3.53E-06
	cgd5_3350			
**2h**	1.51E-06	1.30E-06	2.18E-06	1.79E-06
**6h**	3.45E-07	4.96E-07	1.26E-06	4.85E-07
**12h**	6.44E-07	7.70E-07	1.58E-06	7.34E-07
**24h**	5.91E-07	8.40E-07	1.13E-06	1.03E-06
**36h**	7.25E-07	5.00E-07	1.36E-06	8.69E-07
**48h**	7.30E-07	3.14E-06	1.06E-06	8.47E-07
**72h**	6.91E-07	2.82E-07	3.79E-07	7.36E-07
	cgd5_3380			
**2h**	0.00E+00	0.00E+00	0.00E+00	0.00E+00
**6h**	3.17E-07	1.15E-06	1.98E-06	1.25E-06
**12h**	7.74E-08	1.83E-07	2.59E-07	2.19E-07
**24h**	1.12E-07	3.60E-07	1.44E-06	1.58E-06
**36h**	1.83E-06	3.54E-06	1.50E-06	1.82E-06
**48h**	1.18E-06	9.47E-07	9.68E-07	1.47E-06
**72h**	3.93E-07	5.14E-07	2.28E-07	8.81E-07
	cgd5_3420			
**2h**	2.85E-09	1.89E-09	0.00E+00	0.00E+00
**6h**	6.18E-10	5.96E-10	1.07E-09	0.00E+00
**12h**	8.26E-11	8.39E-10	6.39E-09	2.01E-10
**24h**	1.52E-09	9.53E-10	1.02E-09	7.23E-09
**36h**	6.24E-09	1.66E-08	7.59E-09	1.55E-08
**48h**	3.52E-07	1.23E-07	7.95E-08	2.80E-07
**72h**	1.45E-07	2.47E-07	8.82E-08	4.34E-08
	cgd5_3430			
**2h**	9.16E-06	1.48E-06	1.09E-06	2.33E-05
**6h**	2.98E-05	2.98E-06	2.24E-05	1.53E-04
**12h**	3.65E-07	8.25E-08	1.93E-06	2.81E-06
**24h**	4.40E-06	3.06E-06	2.42E-05	8.12E-05
**36h**	4.44E-06	2.97E-06	4.94E-06	1.92E-05
**48h**	7.97E-06	1.58E-06	3.95E-06	1.99E-05
**72h**	2.61E-06	1.66E-06	8.71E-07	1.13E-05
	cgd5_3450			
**2h**	1.48E-06	3.53E-07	1.10E-07	3.06E-05
**6h**	5.09E-06	2.73E-06	3.07E-05	6.24E-06
**12h**	1.64E-07	2.17E-07	8.08E-07	7.95E-07
**24h**	8.02E-07	9.74E-07	2.71E-06	4.37E-06
**36h**	1.20E-06	7.41E-07	3.08E-06	7.87E-07
**48h**	6.01E-07	2.58E-07	2.68E-07	4.24E-07
**72h**	4.89E-08	1.90E-07	2.15E-08	1.08E-07
	cgd5_3460			

**2h**	1.66E-07	6.86E-08	1.29E-08	3.18E-07
**6h**	1.66E-07	1.60E-07	5.59E-07	6.27E-07
**12h**	6.66E-07	1.78E-06	1.31E-06	4.63E-07
**24h**	3.37E-07	4.28E-07	7.67E-07	9.00E-07
**36h**	1.22E-06	2.26E-06	3.62E-06	3.23E-06
**48h**	1.33E-06	2.28E-07	7.77E-07	1.16E-06
**72h**	2.26E-07	3.67E-07	1.13E-07	2.30E-07
	cgd5_3490			
**2h**	1.13E-04	2.41E-03	1.09E-05	7.19E-06
**6h**	7.19E-05	9.86E-03	3.66E-03	4.30E-05
**12h**	2.25E-05	1.48E-03	5.63E-04	1.04E-05
**24h**	9.62E-05	7.76E-03	1.40E-04	8.14E-05
**36h**	2.19E-05	8.74E-04	7.59E-04	4.51E-05
**48h**	9.55E-05	2.44E-03	1.85E-03	1.85E-05
**72h**	1.71E-04	5.22E-04	2.19E-04	3.33E-06
	cgd5_350			
**2h**	2.61E-03	3.39E-05	2.50E-04	2.04E-04
**6h**	1.84E-03	3.52E-05	3.07E-03	1.83E-04
**12h**	1.19E-04	1.34E-06	1.53E-04	5.87E-05
**24h**	2.65E-04	1.63E-05	5.88E-04	6.88E-05
**36h**	2.59E-04	2.24E-05	5.43E-04	8.13E-05
**48h**	8.86E-04	1.70E-05	3.22E-04	6.50E-05
**72h**	3.00E-04	6.99E-06	1.12E-04	3.66E-04
	cgd5_3530			
**2h**	3.52E-06	1.39E-07	8.00E-09	3.67E-07
**6h**	9.63E-06	3.68E-06	1.47E-05	6.30E-05
**12h**	7.46E-06	3.92E-07	3.67E-06	7.83E-06
**24h**	2.44E-06	1.32E-06	1.86E-05	1.85E-05
**36h**	6.30E-06	1.54E-06	6.00E-06	2.21E-05
**48h**	1.38E-05	9.84E-07	1.21E-05	1.07E-05
**72h**	9.82E-07	2.33E-07	2.84E-07	1.78E-06
	cgd5_3600			
**2h**	0.00E+00	0.00E+00	0.00E+00	0.00E+00
**6h**	0.00E+00	0.00E+00	0.00E+00	0.00E+00
**12h**	2.37E-07	1.27E-07	0.00E+00	5.77E-08
**24h**	2.40E-08	2.62E-08	5.70E-09	1.19E-08
**36h**	2.24E-08	1.09E-08	1.61E-07	1.05E-07
**48h**	9.17E-09	4.91E-09	2.28E-08	1.03E-08
**72h**	7.25E-09	3.42E-08	0.00E+00	9.85E-09
	cgd5_3680			
**2h**	8.86E-07	2.07E-07	0.00E+00	5.63E-05
**6h**	2.39E-07	1.91E-07	3.87E-07	3.60E-06
**12h**	1.46E-09	9.17E-10	1.62E-07	6.60E-08
**24h**	8.83E-08	1.37E-07	1.30E-06	4.26E-07
**36h**	2.03E-07	1.13E-07	7.11E-08	3.20E-07
**48h**	1.98E-07	5.92E-08	1.02E-07	5.05E-07
**72h**	6.90E-08	8.94E-08	4.08E-08	4.88E-08
	cgd5_3690			
**2h**	1.42E-04	9.80E-06	2.13E-05	1.60E-04
**6h**	3.67E-05	7.86E-06	2.48E-05	1.86E-04
**12h**	6.79E-05	1.78E-05	1.14E-04	4.65E-04
**24h**	4.61E-05	2.78E-05	3.41E-05	1.02E-04
**36h**	4.10E-05	7.50E-06	4.38E-05	2.05E-04

**48h**	6.15E-05	7.81E-06	1.61E-05	1.16E-04
**72h**	3.68E-06	2.38E-06	8.44E-07	4.68E-05
	cgd5_3770			
**2h**	1.24E-06	6.29E-07	7.81E-07	5.88E-07
**6h**	6.40E-08	1.02E-07	2.57E-08	4.25E-08
**12h**	1.61E-07	3.36E-07	3.46E-07	2.50E-07
**24h**	1.89E-07	2.33E-07	2.16E-07	1.31E-07
**36h**	7.54E-08	1.50E-07	2.64E-07	2.25E-07
**48h**	2.16E-07	4.31E-08	1.74E-07	1.16E-07
**72h**	5.23E-08	1.48E-07	7.15E-08	7.03E-08
	cgd5_390			
**2h**	7.18E-05	2.44E-03	2.87E-03	4.56E-03
**6h**	1.45E-04	1.04E-03	5.96E-03	1.63E-02
**12h**	3.26E-05	6.08E-04	3.27E-03	2.33E-03
**24h**	1.88E-04	1.13E-03	1.33E-02	4.02E-03
**36h**	2.21E-04	8.78E-04	8.24E-03	3.52E-03
**48h**	1.57E-04	5.21E-04	3.09E-03	2.57E-03
**72h**	6.93E-05	1.98E-04	6.16E-04	6.20E-04
	cgd5_400			
**2h**	5.55E-06	0.00E+00	0.00E+00	0.00E+00
**6h**	1.41E-04	2.61E-04	1.92E-06	9.04E-06
**12h**	7.80E-06	3.34E-06	2.72E-08	4.72E-08
**24h**	5.91E-06	4.32E-05	2.23E-05	1.40E-05
**36h**	1.41E-05	1.64E-04	2.39E-06	1.22E-05
**48h**	5.74E-05	7.56E-05	3.27E-05	1.33E-05
**72h**	7.43E-05	1.83E-04	7.76E-06	1.10E-05
	cgd5_4090			
**2h**	2.09E-05	1.30E-05	1.27E-06	5.56E-06
**6h**	1.88E-06	1.19E-06	9.31E-08	3.74E-07
**12h**	1.00E-05	4.88E-06	4.18E-06	4.83E-06
**24h**	1.04E-05	1.13E-05	2.39E-06	1.14E-06
**36h**	4.95E-06	3.53E-06	2.45E-06	4.41E-06
**48h**	7.68E-06	2.40E-06	6.54E-07	1.57E-06
**72h**	1.99E-06	1.44E-06	9.39E-07	1.18E-06
	cgd5_4520			
**2h**	1.48E-05	5.77E-06	1.32E-06	1.78E-06
**6h**	6.12E-06	5.44E-06	1.36E-06	4.12E-06
**12h**	1.53E-06	8.47E-07	9.43E-07	9.11E-07
**24h**	1.58E-06	1.33E-06	3.20E-06	1.22E-06
**36h**	4.44E-06	2.50E-06	2.76E-06	1.50E-06
**48h**	6.56E-06	1.77E-06	1.43E-06	1.46E-06
**72h**	4.14E-06	1.11E-06	1.29E-06	9.77E-07
	cgd5_630			
**2h**	1.54E-04	9.23E-04	7.78E-06	1.92E-05
**6h**	1.18E-04	2.48E-04	8.53E-05	1.89E-04
**12h**	2.24E-04	2.54E-04	2.36E-05	2.94E-05
**24h**	4.38E-05	3.45E-04	7.07E-05	3.44E-05
**36h**	4.52E-05	3.28E-04	8.28E-05	4.20E-05
**48h**	3.46E-03	2.27E-04	8.84E-05	6.94E-05
**72h**	1.64E-04	4.25E-04	3.13E-05	1.06E-05
	cgd5_90			
**2h**	1.52E-04	2.24E-04	3.34E-03	2.71E-03
**6h**	2.11E-04	2.49E-04	4.70E-04	5.12E-03

**12h**	8.43E-05	7.71E-05	2.45E-03	1.34E-03
**24h**	4.88E-05	2.69E-04	8.17E-04	1.46E-03
**36h**	1.72E-05	2.06E-04	2.88E-03	1.48E-03
**48h**	4.41E-05	7.94E-05	3.76E-04	9.82E-04
**72h**	1.57E-05	4.33E-05	2.95E-05	3.00E-04
	cgd5_920			
**2h**	2.08E-05	4.09E-06	3.93E-07	5.73E-07
**6h**	1.95E-05	2.72E-06	1.30E-06	3.86E-06
**12h**	2.53E-06	8.18E-07	3.16E-07	5.59E-07
**24h**	1.18E-06	1.06E-06	3.05E-06	1.18E-05
**36h**	2.00E-05	1.53E-06	1.86E-06	1.33E-06
**48h**	3.60E-06	1.72E-06	2.58E-06	2.16E-06
**72h**	4.27E-06	1.06E-06	2.10E-07	5.79E-07
	cgd6_1000			
**2h**	0.00E+00	0.00E+00	0.00E+00	2.54E-08
**6h**	0.00E+00	0.00E+00	0.00E+00	3.76E-08
**12h**	2.63E-05	4.37E-06	1.66E-06	2.47E-06
**24h**	2.13E-05	2.08E-06	2.18E-07	1.76E-07
**36h**	5.17E-06	3.10E-06	5.24E-06	4.94E-06
**48h**	1.13E-05	1.83E-06	2.90E-06	9.45E-07
**72h**	3.20E-06	1.19E-06	9.74E-07	1.19E-07
	cgd6_1040			
**2h**	3.80E-06	3.35E-07	6.63E-07	3.57E-07
**6h**	3.97E-07	5.25E-06	1.36E-05	1.23E-06
**12h**	2.09E-05	1.75E-06	1.73E-05	2.33E-06
**24h**	2.79E-05	4.26E-06	4.19E-05	2.65E-06
**36h**	2.66E-05	6.43E-06	4.79E-05	4.20E-06
**48h**	6.86E-05	1.17E-05	4.13E-05	1.36E-05
**72h**	2.13E-05	8.41E-06	5.93E-05	4.83E-06
	cgd6_1050			
**2h**	1.09E-03	5.90E-05	4.48E-04	1.42E-05
**6h**	5.12E-04	3.65E-05	1.13E-04	1.93E-05
**12h**	3.29E-04	6.32E-05	1.79E-04	1.17E-05
**24h**	5.83E-04	2.10E-05	1.49E-04	2.51E-05
**36h**	1.28E-04	9.17E-06	9.99E-05	6.34E-06
**48h**	8.97E-05	1.73E-05	8.96E-05	1.96E-05
**72h**	1.17E-04	1.27E-05	1.73E-04	9.13E-06
	cgd6_1150			
**2h**	2.43E-08	0.00E+00	0.00E+00	1.07E-08
**6h**	6.51E-07	2.93E-07	2.74E-07	1.97E-07
**12h**	1.44E-07	4.55E-07	3.69E-07	5.39E-08
**24h**	1.58E-07	1.32E-07	2.87E-07	1.96E-07
**36h**	3.53E-07	3.07E-07	1.17E-06	2.44E-07
**48h**	4.41E-07	2.00E-07	4.23E-07	3.20E-07
**72h**	2.51E-07	2.05E-07	7.61E-07	3.04E-07
	cgd6_1190			
**2h**	0.00E+00	2.04E-09	0.00E+00	0.00E+00
**6h**	2.06E-06	4.78E-07	1.46E-06	5.78E-07
**12h**	6.52E-08	4.18E-08	2.99E-07	2.34E-08
**24h**	1.65E-07	1.84E-07	3.27E-06	7.25E-07
**36h**	1.64E-06	7.47E-07	2.01E-06	1.02E-06
**48h**	9.85E-07	3.33E-07	8.72E-07	4.56E-07
**72h**	5.90E-07	2.36E-07	3.31E-07	2.47E-07

	cgd6_1210			
**2h**	1.11E-04	9.09E-06	8.12E-05	7.79E-06
**6h**	1.41E-05	2.32E-06	1.12E-05	1.02E-06
**12h**	2.33E-05	6.05E-07	4.34E-05	8.37E-07
**24h**	1.83E-05	4.98E-06	2.44E-05	2.26E-06
**36h**	2.77E-05	3.41E-06	1.87E-05	8.90E-07
**48h**	2.94E-05	2.16E-06	4.15E-05	6.42E-06
**72h**	3.50E-05	5.50E-06	6.64E-05	4.46E-06
	cgd6_1220			
**2h**	1.73E-04	3.95E-06	9.84E-06	4.51E-06
**6h**	0.00E+00	1.01E-08	0.00E+00	0.00E+00
**12h**	4.86E-06	0.00E+00	1.06E-05	1.43E-07
**24h**	8.45E-06	1.23E-06	3.01E-06	6.89E-09
**36h**	3.49E-06	2.86E-07	2.30E-05	1.48E-06
**48h**	5.39E-04	1.24E-04	6.30E-04	1.73E-04
**72h**	1.64E-04	1.97E-04	8.35E-04	9.11E-05
	cgd6_1240			
**2h**	0.00E+00	0.00E+00	0.00E+00	0.00E+00
**6h**	0.00E+00	0.00E+00	0.00E+00	0.00E+00
**12h**	2.90E-08	1.93E-08	3.23E-08	6.81E-09
**24h**	5.38E-09	2.53E-09	2.88E-08	2.06E-09
**36h**	2.85E-08	4.06E-08	5.07E-08	7.07E-08
**48h**	4.88E-08	2.34E-08	7.86E-08	4.53E-08
**72h**	3.21E-08	8.01E-08	3.21E-08	3.67E-08
	cgd6_1250			
**2h**	2.63E-07	1.89E-07	1.29E-08	9.50E-08
**6h**	3.44E-07	4.53E-07	1.42E-06	2.22E-07
**12h**	3.85E-07	1.33E-08	1.23E-07	1.79E-02
**24h**	6.22E-08	5.74E-08	8.21E-07	1.14E-07
**36h**	2.45E-07	2.26E-07	6.56E-07	9.41E-08
**48h**	4.05E-07	1.08E-07	3.69E-07	2.42E-07
**72h**	4.59E-08	1.64E-07	1.15E-07	5.66E-08
	cgd6_1260			
**2h**	2.30E-07	1.05E-07	1.87E-06	3.08E-08
**6h**	4.03E-08	2.58E-07	3.68E-07	2.45E-07
**12h**	9.91E-08	6.78E-10	5.16E-07	5.66E-09
**24h**	1.57E-07	7.05E-08	3.09E-06	9.36E-08
**36h**	5.94E-07	1.72E-07	5.43E-07	1.63E-07
**48h**	2.51E-06	2.10E-07	9.42E-07	1.99E-07
**72h**	5.75E-07	2.35E-07	2.54E-08	3.77E-08
	cgd6_1360			
**2h**	0.00E+00	2.24E-06	0.00E+00	0.00E+00
**6h**	0.00E+00	1.74E-07	3.04E-05	1.34E-07
**12h**	2.63E-03	4.08E-03	2.43E-03	1.03E-03
**24h**	3.70E-04	5.72E-04	4.44E-04	3.34E-04
**36h**	9.08E-04	5.12E-04	1.52E-03	6.14E-04
**48h**	1.36E-03	2.55E-03	4.24E-03	2.59E-03
**72h**	4.73E-04	3.00E-04	8.19E-04	1.67E-04
	cgd6_1540			
**2h**	8.57E-06	5.20E-07	0.00E+00	1.31E-08
**6h**	9.44E-06	2.42E-06	2.63E-06	1.75E-07
**12h**	3.72E-05	9.33E-06	3.25E-05	5.79E-06
**24h**	2.45E-05	7.04E-06	6.59E-05	8.24E-06

**36h**	1.18E-04	2.95E-05	1.08E-04	2.69E-05
**48h**	1.01E-04	1.77E-05	1.01E-04	4.70E-05
**72h**	2.93E-05	6.22E-06	3.14E-05	4.03E-06
	cgd6_1650			
**2h**	7.05E-06	2.92E-04	4.81E-05	3.31E-04
**6h**	1.05E-04	2.95E-04	3.61E-04	3.56E-04
**12h**	3.11E-05	2.09E-04	5.07E-04	1.15E-04
**24h**	1.38E-05	2.99E-04	9.07E-05	7.36E-04
**36h**	5.85E-05	2.96E-04	4.46E-05	1.70E-03
**48h**	4.68E-05	9.44E-05	5.15E-05	1.35E-04
**72h**	8.53E-06	2.76E-04	8.15E-05	5.88E-05
	cgd6_1660			
**2h**	0.00E+00	8.60E-08	0.00E+00	0.00E+00
**6h**	0.00E+00	0.00E+00	4.42E-06	3.65E-09
**12h**	2.90E-05	4.10E-05	5.80E-04	1.06E-04
**24h**	1.04E-05	6.95E-06	8.12E-05	8.01E-06
**36h**	7.49E-06	2.29E-05	2.77E-04	1.60E-05
**48h**	3.51E-06	4.84E-06	3.97E-05	2.40E-06
**72h**	1.14E-06	2.89E-06	3.92E-05	1.07E-06
	cgd6_1690			
**2h**	1.08E-07	2.08E-08	0.00E+00	4.29E-10
**6h**	0.00E+00	7.12E-09	1.94E-06	4.25E-08
**12h**	3.05E-06	3.74E-06	7.10E-05	5.08E-06
**24h**	2.19E-06	2.38E-06	3.41E-05	1.68E-06
**36h**	1.40E-06	1.89E-06	4.12E-05	2.37E-06
**48h**	6.28E-07	9.75E-07	7.41E-06	8.57E-07
**72h**	2.63E-07	7.50E-07	1.69E-05	4.40E-08
	cgd6_170			
**2h**	8.32E-09	0.00E+00	0.00E+00	0.00E+00
**6h**	0.00E+00	0.00E+00	0.00E+00	4.73E-09
**12h**	0.00E+00	0.00E+00	0.00E+00	0.00E+00
**24h**	5.40E-09	9.32E-10	2.36E-09	7.79E-09
**36h**	5.80E-09	1.04E-08	1.34E-08	9.34E-09
**48h**	3.85E-08	4.13E-08	6.82E-08	1.78E-08
**72h**	1.63E-08	3.20E-08	6.42E-08	2.66E-08
	cgd6_1800			
**2h**	1.73E-06	4.78E-07	3.06E-06	1.37E-07
**6h**	1.96E-06	2.13E-06	5.50E-06	1.10E-06
**12h**	2.32E-07	2.93E-08	1.33E-05	6.00E-07
**24h**	1.84E-06	1.17E-06	3.73E-05	3.20E-06
**36h**	2.62E-06	2.37E-06	1.05E-05	1.46E-06
**48h**	5.81E-07	9.16E-07	4.51E-06	4.64E-07
**72h**	5.54E-08	9.47E-07	8.75E-06	2.02E-07
	cgd6_1880			
**2h**	1.68E-06	9.64E-07	1.25E-06	3.49E-07
**6h**	3.59E-08	3.32E-07	8.96E-07	4.86E-08
**12h**	4.43E-08	1.05E-07	1.05E-06	4.05E-07
**24h**	4.91E-07	5.60E-07	5.65E-07	2.22E-07
**36h**	2.76E-07	5.29E-07	2.98E-07	5.50E-07
**48h**	8.31E-08	2.40E-07	3.83E-07	7.29E-08
**72h**	2.24E-07	1.59E-07	3.21E-07	2.65E-07
	cgd6_1890			
**2h**	7.53E-05	5.05E-05	3.25E-04	2.29E-05

**6h**	5.81E-06	3.81E-05	5.85E-04	1.20E-05
**12h**	1.18E-05	1.69E-05	7.25E-04	2.07E-05
**24h**	2.85E-05	6.63E-05	2.64E-04	1.37E-05
**36h**	1.76E-05	4.31E-05	1.71E-04	2.53E-05
**48h**	1.05E-05	9.65E-06	6.74E-05	4.39E-06
**72h**	2.03E-06	6.80E-06	5.82E-05	1.16E-06
	cgd6_1920			
**2h**	0.00E+00	0.00E+00	0.00E+00	0.00E+00
**6h**	0.00E+00	9.51E-10	1.01E-08	1.49E-09
**12h**	7.34E-09	1.74E-08	1.73E-07	8.80E-08
**24h**	2.33E-08	8.58E-08	1.67E-07	4.64E-08
**36h**	4.48E-07	1.31E-07	3.65E-07	5.26E-07
**48h**	1.45E-07	1.72E-07	1.52E-07	7.30E-08
**72h**	1.37E-07	8.42E-08	9.10E-08	6.92E-08
	cgd6_1980			
**2h**	6.94E-07	1.72E-06	9.04E-07	3.23E-07
**6h**	1.45E-06	3.70E-06	5.34E-06	1.63E-06
**12h**	7.49E-08	9.32E-08	2.90E-06	4.86E-07
**24h**	4.17E-07	6.07E-07	3.39E-06	8.04E-07
**36h**	9.83E-07	1.81E-06	4.55E-06	1.11E-06
**48h**	4.84E-07	7.75E-07	2.83E-06	5.81E-07
**72h**	1.67E-07	3.49E-07	7.39E-07	1.28E-08
	cgd6_2010			
**2h**	3.85E-09	0.00E+00	0.00E+00	1.83E-09
**6h**	2.16E-08	4.64E-07	2.75E-07	3.89E-07
**12h**	1.61E-08	1.88E-08	4.85E-08	2.88E-08
**24h**	5.49E-08	1.31E-07	4.34E-07	5.19E-07
**36h**	2.11E-07	3.99E-07	1.84E-07	4.54E-07
**48h**	1.19E-07	1.45E-07	1.24E-07	8.10E-08
**72h**	3.44E-08	5.92E-08	2.48E-08	1.20E-08
	cgd6_2050			
**2h**	2.11E-04	9.76E-05	1.36E-04	5.19E-05
**6h**	3.64E-05	6.32E-05	1.39E-03	8.48E-05
**12h**	2.51E-05	1.92E-05	4.44E-04	1.73E-05
**24h**	5.95E-05	9.92E-05	7.46E-04	1.30E-04
**36h**	9.58E-05	8.81E-05	4.73E-04	3.63E-05
**48h**	7.83E-05	4.54E-05	3.20E-04	2.02E-05
**72h**	1.62E-05	3.28E-05	2.18E-04	2.20E-06
	cgd6_2160			
**2h**	2.52E-05	4.10E-05	5.60E-06	1.58E-05
**6h**	2.96E-06	1.39E-05	6.52E-06	6.24E-06
**12h**	9.51E-07	9.05E-07	5.16E-06	3.32E-06
**24h**	6.45E-06	5.04E-06	5.85E-06	4.10E-06
**36h**	3.45E-06	3.32E-06	6.00E-06	5.73E-06
**48h**	6.25E-06	2.30E-06	4.73E-06	1.73E-05
**72h**	1.33E-06	9.97E-07	2.96E-06	1.17E-06
	cgd6_220			
**2h**	3.23E-08	9.05E-08	1.01E-07	1.55E-07
**6h**	0.00E+00	1.29E-08	2.20E-09	7.36E-09
**12h**	1.84E-09	3.88E-08	3.64E-08	3.22E-08
**24h**	1.43E-08	4.74E-08	1.43E-08	5.81E-09
**36h**	1.82E-08	6.07E-08	1.51E-08	4.21E-08
**48h**	2.24E-08	5.72E-08	3.51E-08	1.21E-08

**72h**	2.27E-08	3.04E-08	1.73E-08	1.41E-08
	cgd6_2230			
**2h**	6.52E-07	5.39E-07	4.53E-07	3.12E-07
**6h**	9.20E-07	7.79E-07	2.01E-07	2.60E-07
**12h**	1.09E-07	5.34E-08	5.13E-08	1.80E-07
**24h**	5.78E-07	4.54E-07	3.89E-07	8.05E-07
**36h**	5.00E-07	8.44E-07	2.85E-07	3.51E-07
**48h**	7.39E-07	5.18E-07	2.15E-07	5.46E-07
**72h**	3.65E-07	6.58E-07	1.85E-07	1.82E-07
	cgd6_2330			
**2h**	3.40E-07	1.77E-06	1.82E-07	1.38E-08
**6h**	5.78E-07	3.88E-06	5.49E-07	7.42E-07
**12h**	6.03E-04	8.15E-04	3.94E-04	3.98E-04
**24h**	1.37E-04	2.14E-04	3.62E-05	7.51E-05
**36h**	3.37E-05	6.25E-05	1.01E-04	1.07E-04
**48h**	1.58E-05	1.39E-05	2.11E-05	2.33E-05
**72h**	5.27E-06	6.52E-06	3.22E-06	2.25E-06
	cgd6_2400			
**2h**	9.81E-08	3.76E-07	9.70E-08	2.30E-08
**6h**	8.33E-08	3.94E-07	2.41E-07	2.16E-07
**12h**	9.17E-09	1.40E-08	1.42E-08	2.75E-08
**24h**	2.17E-08	6.21E-08	7.85E-08	1.58E-07
**36h**	8.69E-08	1.18E-07	9.29E-08	7.45E-08
**48h**	1.32E-07	1.06E-07	1.77E-07	6.37E-08
**72h**	1.65E-07	8.64E-08	2.80E-08	5.14E-08
	cgd6_2420			
**2h**	2.09E-04	1.12E-04	1.76E-04	1.15E-04
**6h**	6.11E-04	9.71E-05	9.07E-04	5.47E-04
**12h**	1.64E-05	3.00E-05	1.55E-04	1.33E-04
**24h**	1.38E-04	2.74E-05	4.94E-04	5.70E-04
**36h**	3.04E-04	3.34E-05	2.60E-04	2.70E-04
**48h**	2.51E-04	2.30E-05	2.37E-04	2.33E-04
**72h**	7.26E-05	7.24E-05	1.05E-04	1.88E-04
	cgd6_250			
**2h**	0.00E+00	1.28E-06	0.00E+00	1.59E-07
**6h**	1.46E-06	1.47E-05	3.95E-06	2.28E-06
**12h**	1.29E-08	1.65E-06	9.22E-08	1.07E-08
**24h**	8.95E-07	1.19E-05	2.06E-06	6.35E-06
**36h**	4.17E-06	1.64E-05	6.55E-06	7.33E-07
**48h**	7.56E-06	1.39E-05	2.94E-06	5.59E-06
**72h**	1.18E-05	2.12E-05	5.31E-07	2.67E-06
	cgd6_2530			
**2h**	7.35E-05	6.92E-04	1.45E-04	6.46E-05
**6h**	2.25E-05	7.60E-05	1.84E-05	3.40E-05
**12h**	1.72E-05	1.72E-04	2.92E-05	6.23E-05
**24h**	4.96E-05	2.15E-04	4.94E-05	4.50E-05
**36h**	8.15E-05	5.28E-05	7.15E-05	5.79E-05
**48h**	1.09E-04	7.59E-05	7.12E-05	5.03E-05
**72h**	7.17E-05	2.51E-04	4.41E-05	4.66E-05
	cgd6_260			
**2h**	0.00E+00	0.00E+00	0.00E+00	0.00E+00
**6h**	9.77E-07	3.27E-05	7.79E-07	1.36E-07
**12h**	2.68E-08	3.66E-07	6.77E-09	1.56E-07

**24h**	2.09E-07	3.28E-06	1.63E-06	4.37E-06
**36h**	3.99E-04	6.20E-05	5.93E-06	9.59E-06
**48h**	1.43E-05	5.74E-05	5.29E-06	7.56E-06
**72h**	3.74E-05	1.84E-05	3.45E-06	5.73E-06
	cgd6_2600			
**2h**	4.77E-04	1.46E-03	9.74E-04	3.49E-04
**6h**	8.42E-06	5.03E-05	6.42E-05	1.46E-05
**12h**	2.67E-04	1.20E-03	4.78E-04	3.13E-04
**24h**	1.77E-04	3.95E-04	1.07E-04	1.43E-04
**36h**	1.03E-04	1.79E-04	2.76E-04	1.11E-04
**48h**	1.01E-04	1.13E-04	1.17E-04	7.28E-05
**72h**	5.87E-05	5.41E-05	2.75E-05	4.95E-05
	cgd6_2630			
**2h**	3.35E-08	6.22E-09	9.30E-09	1.91E-08
**6h**	5.74E-08	3.57E-08	3.18E-08	4.19E-08
**12h**	8.73E-10	4.83E-09	3.72E-09	3.67E-09
**24h**	5.76E-09	1.24E-08	1.41E-08	5.98E-08
**36h**	3.45E-08	2.06E-08	1.79E-08	1.81E-08
**48h**	1.68E-08	2.29E-08	6.11E-09	3.61E-08
**72h**	2.35E-08	2.06E-08	1.27E-08	4.58E-09
	cgd6_2650			
**2h**	3.03E-05	3.45E-04	4.86E-05	5.16E-05
**6h**	9.67E-06	1.77E-04	3.77E-05	4.58E-05
**12h**	2.54E-06	1.44E-05	2.83E-05	2.28E-05
**24h**	1.42E-05	1.27E-04	2.00E-05	6.20E-05
**36h**	1.28E-05	6.60E-05	1.87E-05	1.56E-05
**48h**	1.38E-05	3.80E-05	1.50E-05	7.50E-05
**72h**	8.05E-06	1.16E-05	9.44E-06	4.62E-06
	cgd6_2830			
**2h**	5.22E-06	5.86E-05	1.07E-05	1.94E-05
**6h**	1.16E-05	3.08E-05	1.17E-05	3.76E-06
**12h**	4.84E-08	0.00E+00	5.52E-07	2.32E-07
**24h**	3.98E-06	3.98E-06	4.25E-06	6.04E-06
**36h**	1.03E-05	3.05E-05	9.24E-06	3.00E-06
**48h**	5.03E-06	5.00E-05	6.52E-05	8.70E-05
**72h**	1.43E-05	3.24E-05	1.00E-05	1.70E-05
	cgd6_2840			
**2h**	3.18E-06	2.31E-06	1.70E-06	1.03E-06
**6h**	1.35E-07	1.04E-07	2.89E-07	9.08E-08
**12h**	2.89E-07	3.84E-07	3.71E-07	2.55E-07
**24h**	6.39E-07	3.99E-07	2.26E-07	2.17E-07
**36h**	9.42E-07	1.10E-06	1.05E-06	9.78E-07
**48h**	3.28E-06	2.27E-06	2.74E-06	2.05E-06
**72h**	2.11E-06	4.02E-06	9.99E-07	3.22E-06
	cgd6_2900			
**2h**	0.00E+00	0.00E+00	0.00E+00	0.00E+00
**6h**	0.00E+00	0.00E+00	0.00E+00	0.00E+00
**12h**	0.00E+00	1.33E-05	1.80E-05	1.06E-07
**24h**	4.23E-06	7.96E-07	1.58E-06	1.74E-07
**36h**	2.66E-05	3.19E-06	6.76E-05	1.67E-06
**48h**	8.34E-05	6.66E-05	4.48E-04	4.00E-06
**72h**	6.24E-05	2.35E-05	2.87E-04	3.55E-06
	cgd6_2910			

**2h**	0.00E+00	0.00E+00	0.00E+00	0.00E+00
**6h**	0.00E+00	0.00E+00	0.00E+00	0.00E+00
**12h**	1.48E-06	4.30E-06	3.34E-06	2.02E-06
**24h**	1.10E-05	1.09E-06	1.52E-05	5.69E-07
**36h**	1.30E-05	5.57E-06	9.78E-06	1.62E-06
**48h**	1.22E-04	1.94E-05	4.10E-04	2.06E-05
**72h**	2.17E-05	2.28E-05	1.09E-04	1.33E-05
	cgd6_2920			
**2h**	0.00E+00	0.00E+00	0.00E+00	0.00E+00
**6h**	0.00E+00	0.00E+00	0.00E+00	0.00E+00
**12h**	0.00E+00	0.00E+00	0.00E+00	0.00E+00
**24h**	6.22E-08	7.45E-07	1.08E-07	3.14E-06
**36h**	3.35E-07	4.47E-06	6.03E-07	1.58E-05
**48h**	1.86E-05	3.26E-05	1.70E-05	2.42E-04
**72h**	1.08E-05	1.59E-04	1.60E-05	3.48E-04
	cgd6_300			
**2h**	5.27E-09	0.00E+00	0.00E+00	0.00E+00
**6h**	0.00E+00	2.00E-09	0.00E+00	0.00E+00
**12h**	7.11E-10	1.34E-08	1.96E-08	2.18E-08
**24h**	7.70E-09	1.43E-08	4.47E-08	5.22E-08
**36h**	1.84E-08	2.15E-07	7.33E-08	3.25E-07
**48h**	2.55E-06	2.43E-06	2.33E-06	5.36E-06
**72h**	3.88E-06	3.25E-06	3.43E-06	3.63E-06
	cgd6_310			
**2h**	1.53E-04	8.37E-05	3.87E-06	3.33E-05
**6h**	4.57E-04	2.36E-04	1.44E-04	2.44E-05
**12h**	1.53E-06	3.96E-06	1.30E-05	7.22E-06
**24h**	1.71E-04	1.81E-05	2.72E-04	9.10E-06
**36h**	4.48E-04	4.69E-05	9.83E-05	3.03E-05
**48h**	6.41E-04	2.04E-04	3.73E-04	6.99E-05
**72h**	1.89E-03	2.81E-04	2.05E-03	1.98E-04
	cgd6_3110			
**2h**	1.84E-05	4.32E-05	9.19E-06	5.85E-05
**6h**	2.27E-07	8.62E-07	1.51E-07	4.04E-06
**12h**	9.47E-06	4.43E-05	1.17E-05	4.27E-05
**24h**	1.32E-05	1.31E-05	6.55E-06	1.25E-05
**36h**	4.97E-06	1.16E-05	1.41E-05	1.65E-05
**48h**	4.31E-06	4.36E-06	4.88E-06	7.40E-06
**72h**	1.28E-06	2.28E-06	3.17E-06	2.28E-06
	cgd6_320			
**2h**	3.72E-07	7.83E-07	9.88E-08	3.27E-07
**6h**	2.18E-08	4.17E-08	3.59E-08	1.63E-07
**12h**	1.84E-07	2.35E-07	3.12E-07	3.40E-07
**24h**	2.26E-07	3.64E-07	2.19E-07	2.22E-07
**36h**	2.84E-07	3.20E-07	5.23E-07	4.50E-07
**48h**	2.08E-07	4.64E-07	2.83E-07	3.20E-07
**72h**	5.65E-07	5.74E-07	5.56E-07	8.28E-07
	cgd6_3200			
**2h**	3.59E-07	8.38E-05	2.68E-06	1.31E-05
**6h**	0.00E+00	2.82E-05	0.00E+00	4.23E-06
**12h**	4.31E-06	0.00E+00	7.12E-06	4.33E-06
**24h**	4.42E-06	2.11E-05	1.65E-07	3.62E-05
**36h**	4.01E-05	1.62E-05	2.34E-05	2.36E-05

**48h**	2.10E-04	4.39E-05	9.66E-05	1.84E-05
**72h**	5.15E-04	3.31E-04	1.28E-03	4.99E-04
	cgd6_3320			
**2h**	7.13E-06	3.40E-05	3.38E-05	1.87E-06
**6h**	2.02E-05	3.64E-05	1.75E-05	3.37E-06
**12h**	0.00E+00	0.00E+00	2.58E-07	0.00E+00
**24h**	1.06E-06	1.24E-06	2.41E-05	2.72E-06
**36h**	7.02E-06	7.28E-06	1.24E-05	1.05E-06
**48h**	4.09E-05	2.70E-06	3.08E-05	1.47E-06
**72h**	6.32E-05	1.14E-05	1.12E-05	1.40E-06
	cgd6_3340			
**2h**	7.11E-05	1.64E-04	1.63E-05	1.62E-04
**6h**	9.66E-05	2.94E-04	1.04E-04	1.18E-03
**12h**	1.12E-05	7.37E-05	4.84E-05	1.99E-04
**24h**	4.41E-05	1.41E-04	1.74E-04	3.09E-04
**36h**	6.61E-05	7.43E-05	3.38E-05	1.44E-04
**48h**	2.81E-05	5.58E-05	2.42E-05	1.12E-04
**72h**	1.05E-05	9.62E-06	6.14E-06	3.83E-05
	cgd6_340			
**2h**	3.46E-07	4.90E-07	1.45E-07	1.99E-07
**6h**	2.78E-07	7.54E-07	8.16E-07	1.24E-06
**12h**	2.23E-07	7.59E-07	7.48E-07	4.63E-07
**24h**	1.67E-07	2.54E-07	1.73E-06	6.72E-07
**36h**	7.42E-07	5.91E-07	6.58E-07	1.28E-06
**48h**	8.58E-07	6.40E-07	2.91E-06	1.23E-06
**72h**	4.62E-07	3.65E-07	2.22E-07	4.74E-07
	cgd6_3440			
**2h**	5.12E-04	1.32E-03	1.37E-04	3.04E-04
**6h**	1.93E-04	9.20E-04	9.01E-05	1.46E-04
**12h**	1.07E-04	1.68E-04	2.63E-04	7.72E-04
**24h**	3.41E-04	4.68E-04	3.91E-04	1.22E-04
**36h**	2.58E-04	1.77E-04	2.39E-04	4.23E-04
**48h**	1.25E-04	1.23E-04	1.66E-04	1.18E-04
**72h**	6.16E-05	6.83E-05	4.61E-05	9.46E-05
	cgd6_3450			
**2h**	9.60E-05	5.59E-05	4.31E-05	2.68E-05
**6h**	7.66E-05	1.07E-05	1.92E-05	8.05E-06
**12h**	3.49E-05	2.96E-05	4.82E-05	7.76E-06
**24h**	6.78E-05	5.26E-05	3.19E-05	1.56E-05
**36h**	9.89E-06	1.39E-05	1.07E-05	6.42E-06
**48h**	1.18E-05	1.97E-05	1.24E-05	9.44E-06
**72h**	1.35E-05	1.40E-05	7.56E-06	2.26E-06
	cgd6_3460			
**2h**	0.00E+00	5.42E-08	0.00E+00	0.00E+00
**6h**	0.00E+00	9.73E-09	1.05E-08	7.10E-10
**12h**	3.41E-08	1.06E-08	6.72E-08	6.35E-09
**24h**	6.25E-08	9.73E-09	1.61E-07	1.07E-05
**36h**	2.78E-07	3.03E-07	4.62E-07	3.20E-07
**48h**	8.70E-05	8.87E-05	6.25E-05	4.89E-05
**72h**	9.38E-05	3.13E-04	8.28E-05	2.97E-05
	cgd6_3470			
**2h**	1.06E-04	2.16E-04	5.53E-04	1.75E-05
**6h**	5.55E-05	7.38E-05	2.02E-04	4.12E-06

**12h**	4.25E-03	4.66E-03	4.87E-03	8.15E-04
**24h**	4.78E-04	3.66E-04	5.87E-04	3.38E-04
**36h**	5.41E-04	1.64E-04	1.43E-03	5.12E-04
**48h**	9.09E-05	1.47E-04	4.12E-04	1.27E-04
**72h**	1.20E-04	3.48E-04	2.47E-04	6.21E-06
	cgd6_3480			
**2h**	4.77E-05	1.26E-04	3.39E-05	2.65E-06
**6h**	5.57E-06	3.26E-06	5.92E-06	2.45E-06
**12h**	1.83E-05	7.28E-06	2.38E-05	2.21E-06
**24h**	2.58E-05	1.95E-05	6.85E-06	1.58E-06
**36h**	1.55E-05	1.77E-05	1.12E-05	1.11E-06
**48h**	4.95E-06	5.03E-06	2.30E-06	1.28E-06
**72h**	2.11E-06	6.62E-06	5.59E-07	1.88E-07
	cgd6_3490			
**2h**	7.78E-04	2.63E-04	1.71E-05	7.20E-07
**6h**	6.39E-04	8.44E-05	4.03E-05	1.89E-05
**12h**	9.66E-05	5.13E-04	3.05E-04	3.12E-06
**24h**	6.91E-05	8.65E-05	8.25E-05	2.51E-05
**36h**	1.38E-04	7.72E-05	2.60E-04	2.98E-05
**48h**	2.08E-04	2.55E-04	1.48E-04	1.09E-05
**72h**	3.48E-05	1.91E-04	2.25E-05	8.25E-07
	cgd6_3530			
**2h**	4.62E-06	1.14E-05	3.07E-06	1.91E-06
**6h**	3.93E-06	2.00E-06	3.75E-06	1.55E-06
**12h**	1.40E-07	3.89E-07	2.51E-06	2.16E-07
**24h**	1.11E-06	9.34E-07	2.77E-06	9.33E-07
**36h**	1.58E-06	9.23E-07	1.40E-06	6.75E-07
**48h**	9.38E-07	1.38E-06	8.75E-07	1.08E-06
**72h**	3.91E-06	2.43E-06	1.99E-06	8.40E-07
	cgd6_3580			
**2h**	6.19E-04	4.85E-04	3.30E-04	7.95E-05
**6h**	2.15E-04	4.82E-04	1.01E-03	2.00E-04
**12h**	9.71E-05	5.50E-05	1.18E-03	1.75E-05
**24h**	1.59E-04	1.54E-04	6.46E-04	8.92E-05
**36h**	9.05E-05	6.02E-04	4.83E-04	6.28E-05
**48h**	1.21E-04	2.01E-04	2.53E-04	2.70E-05
**72h**	2.68E-04	3.16E-04	1.48E-04	1.51E-05
	cgd6_3600			
**2h**	0.00E+00	0.00E+00	0.00E+00	0.00E+00
**6h**	0.00E+00	0.00E+00	0.00E+00	0.00E+00
**12h**	1.22E-08	9.24E-08	2.86E-07	3.94E-09
**24h**	2.07E-08	1.48E-08	5.70E-08	3.24E-08
**36h**	1.54E-07	4.27E-08	1.65E-07	5.19E-08
**48h**	3.24E-07	4.51E-07	5.33E-07	3.40E-07
**72h**	7.41E-07	1.41E-06	1.03E-06	7.23E-07
	cgd6_3630			
**2h**	0.00E+00	0.00E+00	0.00E+00	0.00E+00
**6h**	0.00E+00	0.00E+00	1.64E-08	6.82E-10
**12h**	1.75E-05	1.49E-05	1.87E-05	2.31E-06
**24h**	3.09E-06	3.48E-06	2.16E-06	7.41E-07
**36h**	1.61E-06	1.48E-06	4.58E-06	3.42E-06
**48h**	1.23E-06	7.24E-07	1.32E-06	1.71E-06
**72h**	1.05E-06	1.13E-06	7.99E-07	3.74E-07

	cgd6_3670			
**2h**	7.87E-08	0.00E+00	0.00E+00	4.14E-08
**6h**	1.41E-07	5.37E-08	2.52E-07	5.82E-08
**12h**	4.66E-07	3.25E-08	7.50E-07	1.24E-07
**24h**	1.49E-07	3.31E-07	3.94E-07	1.78E-07
**36h**	5.74E-07	1.72E-07	7.90E-07	1.97E-07
**48h**	6.44E-07	6.96E-07	1.05E-06	8.72E-07
**72h**	1.11E-06	1.69E-06	1.80E-06	7.87E-07
	cgd6_3760			
**2h**	5.41E-06	3.14E-05	1.27E-06	2.01E-06
**6h**	1.01E-06	3.28E-07	1.19E-06	9.61E-07
**12h**	7.31E-07	1.35E-06	3.40E-06	9.62E-07
**24h**	1.50E-06	1.93E-06	1.60E-06	1.39E-06
**36h**	1.26E-06	2.05E-06	1.04E-06	1.22E-06
**48h**	3.85E-07	9.60E-07	4.53E-07	1.14E-06
**72h**	6.65E-07	1.58E-06	8.83E-07	4.88E-07
	cgd6_3850			
**2h**	1.10E-02	4.90E-03	1.21E-01	5.09E-03
**6h**	1.27E-02	2.81E-03	5.03E-02	1.08E-02
**12h**	6.67E-03	4.33E-03	6.70E-02	8.32E-04
**24h**	1.05E-02	4.61E-03	5.92E-02	2.57E-03
**36h**	1.74E-03	9.26E-04	1.28E-02	2.40E-03
**48h**	6.32E-04	8.56E-04	1.52E-02	1.38E-03
**72h**	6.35E-04	2.35E-04	8.42E-03	5.65E-03
	cgd6_390			
**2h**	4.80E-07	0.00E+00	0.00E+00	2.32E+11
**6h**	0.00E+00	0.00E+00	0.00E+00	2.67E+03
**12h**	5.91E-05	2.07E-05	4.79E-04	2.67E-06
**24h**	7.07E-05	2.99E-05	1.76E-04	4.11E-07
**36h**	1.32E-05	2.70E-06	1.88E-04	1.84E-06
**48h**	2.53E-04	2.64E-05	8.25E-04	1.23E-05
**72h**	4.41E-04	6.27E-05	1.49E-03	3.45E-05
	cgd6_3910			
**2h**	1.32E-03	1.30E-03	7.60E-03	4.63E-03
**6h**	3.09E-04	1.24E-04	3.07E-03	1.86E-05
**12h**	3.67E-03	4.36E-03	6.51E-02	1.79E-03
**24h**	1.10E-03	1.03E-03	8.48E-03	1.85E-03
**36h**	8.23E-04	9.74E-05	9.04E-03	3.48E-03
**48h**	8.42E-04	1.65E-04	5.65E-03	1.43E-03
**72h**	5.58E-04	9.33E-05	7.70E-03	2.53E-04
	cgd6_3930			
**2h**	2.67E-07	2.62E-07	0.00E+00	3.58E+06
**6h**	1.60E-07	1.60E-08	0.00E+00	2.71E-06
**12h**	1.19E-03	6.26E-04	9.85E-03	1.53E-04
**24h**	1.88E-04	5.35E-05	6.62E-04	6.94E-05
**36h**	1.15E-04	2.05E-05	2.71E-03	3.27E-04
**48h**	1.29E-04	2.08E-05	3.90E-04	2.92E-05
**72h**	3.90E-04	3.44E-05	1.45E-03	2.05E-04
	cgd6_3970			
**2h**	8.15E-07	2.92E-07	4.63E-07	7.76E-08
**6h**	2.85E-07	2.94E-07	1.14E-06	1.70E-07
**12h**	2.53E-07	5.88E-08	6.85E-07	2.64E-08
**24h**	3.10E-07	1.69E-07	5.12E-07	3.20E-07

**36h**	0.00E+00	5.25E-08	2.97E-07	2.53E-07
**48h**	1.13E-07	4.47E-08	2.00E-07	0.00E+00
**72h**	9.11E-08	5.04E-08	1.19E-07	3.09E-07
	cgd6_3980			
**2h**	9.23E-04	1.21E-04	1.17E-02	1.40E-04
**6h**	3.94E-04	2.18E-05	9.24E-03	3.56E-05
**12h**	5.29E-04	1.35E-04	1.26E-02	1.14E-04
**24h**	8.53E-04	2.48E-04	6.26E-03	3.09E-04
**36h**	4.01E-04	8.16E-05	5.54E-03	3.16E-04
**48h**	1.90E-04	5.15E-05	5.17E-03	4.73E-04
**72h**	3.78E-04	2.70E-05	1.84E-03	6.65E-05
	cgd6_4010			
**2h**	5.46E-08	0.00E+00	0.00E+00	4.26E+05
**6h**	0.00E+00	0.00E+00	0.00E+00	2.26E+05
**12h**	6.75E-08	9.87E-08	7.07E-06	1.90E-02
**24h**	9.94E-08	1.78E-07	6.24E-07	1.13E-07
**36h**	4.06E-07	1.95E-07	2.65E-06	2.65E-06
**48h**	1.92E-06	7.44E-07	3.88E-06	4.47E-06
**72h**	1.25E-06	2.62E-07	6.38E-06	1.71E-06
	cgd6_4190			
**2h**	9.41E-06	3.33E-06	2.46E-05	9.83E-06
**6h**	7.97E-06	7.34E-06	1.78E-05	4.74E-05
**12h**	1.30E-06	7.23E-07	7.16E-06	2.92E-06
**24h**	1.54E-06	4.67E-06	5.59E-06	1.36E-05
**36h**	2.08E-06	1.40E-06	2.10E-06	1.07E-05
**48h**	1.71E-06	7.69E-07	2.06E-06	7.24E-06
**72h**	1.05E-06	5.25E-07	1.26E-06	2.01E-06
	cgd6_4210			
**2h**	2.51E-07	1.25E-07	8.10E-07	6.07E-08
**6h**	8.09E-09	2.44E-09	7.70E-08	1.63E+03
**12h**	5.80E-07	7.99E-07	2.13E-06	8.26E-04
**24h**	7.37E-07	5.49E-07	1.02E-06	4.51E-07
**36h**	1.19E-06	6.25E-07	2.09E-06	1.62E-06
**48h**	1.94E-06	6.36E-07	2.03E-06	3.00E-07
**72h**	1.89E-06	7.31E-07	3.74E-06	1.42E-06
	cgd6_4250			
**2h**	1.77E-05	0.00E+00	0.00E+00	2.20E+05
**6h**	2.71E-04	4.02E-04	2.89E-02	5.69E-03
**12h**	6.14E-05	6.29E-04	1.96E-02	1.16E-04
**24h**	1.11E-04	8.20E-05	6.57E-02	3.47E-03
**36h**	1.60E-04	2.62E-04	5.60E-02	6.17E-04
**48h**	1.19E-04	2.16E-04	9.64E-02	2.35E-03
**72h**	3.11E-04	2.09E-04	2.10E-02	2.01E-06
	cgd6_4260			
**2h**	2.30E-06	2.54E-07	2.55E-06	1.42E-06
**6h**	4.46E-07	1.46E-07	1.68E-07	2.95E-07
**12h**	1.13E-06	2.45E-08	5.38E-07	3.78E-07
**24h**	4.80E-07	4.46E-07	2.95E-07	4.79E-08
**36h**	1.62E-07	7.01E-08	1.22E-07	2.07E-07
**48h**	2.29E-07	8.12E-08	1.34E-07	2.12E-07
**72h**	6.76E-08	4.50E-08	8.63E-08	3.99E-09
	cgd6_430			
**2h**	0.00E+00	0.00E+00	0.00E+00	2.35E+06

**6h**	0.00E+00	1.92E-09	0.00E+00	2.36E+04
**12h**	9.00E-08	7.97E-08	5.53E-08	4.44E-09
**24h**	1.53E-07	2.24E-08	1.69E-08	8.82E-08
**36h**	9.20E-08	2.22E-08	1.37E-07	2.11E-07
**48h**	8.83E-08	3.07E-08	1.39E-07	1.38E-07
**72h**	1.89E-07	1.15E-07	3.72E-08	4.92E-07
	cgd6_4300			
**2h**	0.00E+00	0.00E+00	0.00E+00	0.00E+00
**6h**	0.00E+00	0.00E+00	4.77E-09	0.00E+00
**12h**	5.29E-09	8.52E-08	1.12E-08	2.10E-09
**24h**	1.78E-08	7.55E-08	3.04E-07	8.84E-08
**36h**	4.32E-07	6.47E-07	1.20E-07	1.79E-07
**48h**	1.61E-07	3.90E-07	1.83E-07	5.59E-08
**72h**	1.83E-07	1.49E-07	3.60E-07	1.68E-08
	cgd6_4450			
**2h**	6.20E-08	8.03E-08	3.64E-09	6.43E-09
**6h**	2.25E-07	6.92E-07	6.57E-07	5.54E-07
**12h**	2.31E-07	5.24E-07	1.68E-07	8.76E-08
**24h**	1.69E-07	4.21E-07	5.89E-07	3.61E-07
**36h**	1.37E-07	2.59E-07	2.13E-07	3.33E-07
**48h**	4.00E-06	3.01E-06	4.31E-06	6.78E-06
**72h**	4.75E-06	3.27E-06	6.81E-06	7.24E-06
	cgd6_450			
**2h**	2.80E-05	7.04E-06	1.95E-07	4.35E-07
**6h**	8.35E-06	9.99E-06	8.92E-07	7.16E-04
**12h**	4.97E-07	2.81E-07	7.85E-07	7.29E-07
**24h**	4.13E-06	1.15E-05	3.55E-06	3.79E-06
**36h**	1.05E-05	6.85E-06	2.54E-06	1.48E-06
**48h**	7.11E-06	8.95E-06	2.94E-06	1.69E-06
**72h**	7.58E-06	3.20E-06	7.62E-07	8.44E-07
	cgd6_4520			
**2h**	1.22E-07	4.35E-07	2.13E-08	5.87E-08
**6h**	2.54E-07	1.82E-06	1.32E-06	3.38E-07
**12h**	1.40E-06	1.49E-06	4.51E-07	2.04E-07
**24h**	9.84E-07	3.65E-06	9.09E-07	5.08E-07
**36h**	9.25E-07	1.75E-06	8.37E-07	1.31E-06
**48h**	5.09E-06	2.88E-06	2.37E-06	1.85E-06
**72h**	4.40E-06	3.62E-06	1.10E-05	5.97E-06
	cgd6_4560			
**2h**	2.32E-04	1.73E-04	1.66E-06	3.56E-06
**6h**	2.90E-05	1.83E-04	1.82E-06	8.87E-06
**12h**	1.87E-04	1.36E-04	4.95E-05	2.95E-05
**24h**	1.04E-04	3.17E-04	4.59E-05	3.05E-05
**36h**	2.29E-04	2.40E-04	1.24E-04	1.18E-04
**48h**	2.78E-04	1.24E-04	4.93E-05	7.43E-05
**72h**	3.14E-04	1.62E-04	6.12E-05	2.75E-05
	cgd6_4620			
**2h**	1.83E-04	1.01E-04	1.94E-05	1.86E-05
**6h**	7.31E-04	2.83E-04	6.14E-05	4.42E-05
**12h**	2.71E-05	2.49E-05	1.03E-04	1.80E-05
**24h**	4.26E-05	5.51E-05	4.39E-05	4.04E-05
**36h**	4.78E-05	3.84E-05	2.19E-05	1.78E-05
**48h**	2.23E-05	2.67E-05	1.73E-05	1.17E-05

**72h**	3.40E-06	2.58E-06	2.31E-06	2.05E-06
	cgd6_4690			
**2h**	7.70E-06	4.59E-06	4.05E-07	1.54E-06
**6h**	1.12E-05	8.89E-06	1.04E-06	1.17E-06
**12h**	6.27E-07	1.08E-06	1.82E-06	7.47E-07
**24h**	1.45E-06	3.67E-06	1.90E-06	2.56E-06
**36h**	1.60E-06	2.25E-06	2.98E-06	3.10E-06
**48h**	2.02E-06	1.49E-06	5.47E-07	6.60E-07
**72h**	5.72E-07	5.90E-07	9.92E-07	9.27E-07
	cgd6_4820			
**2h**	2.86E-06	0.00E+00	0.00E+00	0.00E+00
**6h**	0.00E+00	0.00E+00	4.65E-08	1.43E-08
**12h**	6.40E-07	2.29E-06	2.48E-08	2.14E-08
**24h**	1.95E-07	4.34E-07	9.18E-08	1.53E-08
**36h**	4.86E-07	5.45E-07	4.06E-07	1.74E-07
**48h**	1.17E-05	2.56E-06	7.37E-07	9.28E-07
**72h**	1.08E-05	4.41E-06	8.06E-06	5.64E-06
	cgd6_4830			
**2h**	7.51E-04	2.25E-03	3.93E-05	1.54E-04
**6h**	2.87E-04	3.29E-04	5.31E-05	7.51E-05
**12h**	1.71E-04	2.38E-04	1.73E-04	1.12E-04
**24h**	2.39E-04	8.77E-04	1.18E-04	1.10E-04
**36h**	2.71E-04	3.10E-04	6.48E-05	6.18E-05
**48h**	2.32E-04	2.66E-04	9.05E-05	4.14E-05
**72h**	3.99E-04	1.64E-04	5.68E-05	4.76E-05
	cgd6_4860			
**2h**	7.06E-04	7.66E-04	3.51E-05	1.86E-04
**6h**	1.44E-05	7.67E-05	1.62E-05	1.97E-05
**12h**	1.04E-04	1.28E-04	5.45E-05	3.25E-05
**24h**	5.25E-03	1.62E-04	8.55E-05	5.57E-05
**36h**	4.66E-05	6.99E-05	2.72E-05	1.64E-05
**48h**	7.01E-05	3.55E-05	2.56E-05	2.08E-05
**72h**	1.89E-05	1.65E-05	7.97E-06	9.59E-06
	cgd6_4980			
**2h**	0.00E+00	0.00E+00	0.00E+00	0.00E+00
**6h**	0.00E+00	0.00E+00	0.00E+00	0.00E+00
**12h**	1.16E-07	2.40E-07	1.52E-07	1.37E-07
**24h**	4.30E-08	9.32E-08	1.06E-07	4.17E-08
**36h**	3.54E-08	6.77E-08	1.71E-07	1.78E-07
**48h**	3.80E-08	1.25E-06	1.25E-07	7.30E-08
**72h**	7.70E-09	1.89E-08	7.75E-09	1.43E-08
	cgd6_5100			
**2h**	0.00E+00	0.00E+00	0.00E+00	0.00E+00
**6h**	1.97E-06	6.07E-04	1.34E-05	5.48E-06
**12h**	0.00E+00	7.89E-04	8.03E-05	4.09E-06
**24h**	4.16E-06	8.33E-02	1.43E-04	5.00E-05
**36h**	8.59E-05	1.59E-01	8.18E-05	1.46E-04
**48h**	2.09E-05	1.33E-01	4.40E-05	1.56E-05
**72h**	1.27E-05	6.59E-02	4.61E-05	1.64E-05
	cgd6_5120			
**2h**	3.56E-06	1.25E-06	4.60E-07	1.94E-06
**6h**	3.34E-06	1.06E-06	1.45E-06	1.49E-06
**12h**	7.23E-07	1.59E-07	6.05E-07	4.19E-07

**24h**	2.39E-06	1.44E-06	1.23E-06	7.61E-07
**36h**	7.75E-07	6.14E-07	4.26E-07	4.79E-07
**48h**	3.85E-07	2.83E-07	4.66E-07	4.31E-07
**72h**	1.53E-07	8.90E-08	2.44E-07	2.11E-07
	cgd6_5220			
**2h**	2.07E-07	0.00E+00	0.00E+00	0.00E+00
**6h**	0.00E+00	0.00E+00	0.00E+00	0.00E+00
**12h**	4.35E-07	5.13E-09	9.83E-06	5.77E-08
**24h**	4.09E-07	2.85E-08	2.23E-08	1.61E-08
**36h**	1.13E-07	2.05E-08	2.18E-07	3.09E-07
**48h**	3.27E-06	6.35E-07	2.39E-06	2.16E-06
**72h**	7.30E-06	4.17E-06	7.34E-06	1.46E-05
	cgd6_5230			
**2h**	0.00E+00	1.65E-08	0.00E+00	7.98E-08
**6h**	7.46E-07	0.00E+00	0.00E+00	2.58E-08
**12h**	1.35E-07	9.01E-08	3.32E-08	1.64E-08
**24h**	9.66E-08	3.76E-08	1.85E-08	1.57E-08
**36h**	7.75E-08	5.49E-08	9.84E-08	1.05E-07
**48h**	1.46E-06	7.52E-07	1.00E-06	9.62E-07
**72h**	2.62E-06	3.48E-06	7.07E-06	1.18E-04
	cgd6_5270			
**2h**	3.60E-06	1.37E-06	3.92E-07	1.53E-06
**6h**	4.44E-06	1.14E-06	2.83E-06	1.37E-06
**12h**	3.87E-06	1.43E-05	4.59E-06	2.94E-06
**24h**	1.29E-06	9.93E-07	1.11E-06	7.46E-07
**36h**	7.51E-07	1.76E-06	2.42E-06	5.26E-06
**48h**	7.78E-07	5.65E-07	9.14E-07	8.19E-07
**72h**	2.17E-07	4.31E-07	7.41E-07	8.83E-07
	cgd6_5430			
**2h**	3.59E-05	4.32E-05	3.69E-05	7.51E-05
**6h**	9.15E-05	2.32E-05	3.85E-05	2.86E-05
**12h**	1.13E-05	1.09E-05	2.91E-05	1.59E-05
**24h**	1.86E-05	1.20E-05	2.57E-05	1.23E-05
**36h**	8.59E-06	9.50E-06	1.43E-05	1.56E-05
**48h**	5.52E-06	2.69E-06	8.01E-06	6.15E-06
**72h**	9.61E-07	1.65E-06	2.30E-06	2.45E-06
	cgd6_5470			
**2h**	2.03E-06	0.00E+00	0.00E+00	0.00E+00
**6h**	0.00E+00	0.00E+00	0.00E+00	0.00E+00
**12h**	1.20E-06	0.00E+00	1.97E-06	8.69E-07
**24h**	2.29E-07	2.74E-05	8.51E-07	3.88E-06
**36h**	5.03E-07	1.36E-05	4.49E-06	5.28E-06
**48h**	5.87E-07	7.57E-06	2.41E-06	1.90E-06
**72h**	7.75E-07	1.17E-05	4.27E-06	2.54E-06
	cgd6_550			
**2h**	0.00E+00	0.00E+00	3.71E-06	0.00E+00
**6h**	0.00E+00	4.91E-09	0.00E+00	0.00E+00
**12h**	0.00E+00	0.00E+00	0.00E+00	1.84E-07
**24h**	2.49E-06	1.30E-07	8.34E-07	5.48E-07
**36h**	1.87E-06	6.61E-07	1.00E-05	8.57E-06
**48h**	7.48E-05	4.01E-05	1.05E-03	5.38E-04
**72h**	7.79E-05	1.76E-04	1.05E-03	1.33E-03
	cgd6_5500			

**2h**	1.27E-05	4.62E-07	0.00E+00	7.20E-05
**6h**	7.78E-06	2.77E-06	4.59E-05	1.84E-05
**12h**	4.09E-06	1.66E-06	3.60E-05	7.14E-06
**24h**	1.94E-05	2.74E-06	1.32E-05	4.47E-05
**36h**	5.24E-06	8.76E-07	2.54E-05	5.16E-05
**48h**	4.27E-06	5.89E-07	1.59E-05	4.89E-05
**72h**	3.76E-06	9.82E-08	4.61E-06	0.00E+00
	cgd6_630			
**2h**	3.59E-05	1.14E-06	3.26E-06	9.19E-06
**6h**	1.54E-05	9.32E-07	1.74E-06	3.27E-06
**12h**	1.57E-05	1.54E-06	9.62E-06	1.06E-05
**24h**	1.10E-05	4.28E-06	2.23E-06	2.86E-06
**36h**	9.29E-06	1.32E-06	5.07E-06	4.25E-06
**48h**	2.83E-06	6.12E-07	4.36E-06	3.39E-06
**72h**	3.51E-06	6.13E-07	2.79E-06	2.09E-06
	cgd6_660			
**2h**	0.00E+00	0.00E+00	0.00E+00	1.03E-05
**6h**	1.27E-06	1.53E-06	2.32E-05	1.98E-05
**12h**	2.37E-07	1.18E-06	8.18E-06	6.14E-07
**24h**	3.75E-07	7.06E-05	6.00E-06	1.32E-05
**36h**	2.12E-06	1.68E-04	1.40E-05	8.88E-05
**48h**	8.12E-06	3.57E-05	5.19E-05	5.47E-05
**72h**	1.12E-05	2.50E-04	2.21E-04	4.59E-05
	cgd6_700			
**2h**	0.00E+00	0.00E+00	0.00E+00	0.00E+00
**6h**	1.86E-04	1.66E-06	9.10E-06	3.80E-06
**12h**	9.84E-06	1.98E-07	5.07E-07	1.99E-07
**24h**	3.79E-06	9.60E-07	3.35E-06	4.09E-06
**36h**	3.94E-05	1.59E-06	1.11E-06	3.57E-06
**48h**	2.13E-05	4.38E-07	2.10E-06	4.20E-06
**72h**	1.19E-06	2.76E-07	1.59E-07	1.57E-07
	cgd6_750			
**2h**	2.43E-06	3.39E-06	2.60E-05	4.11E-05
**6h**	5.26E-06	2.24E-04	1.31E-04	1.49E-04
**12h**	3.27E-06	1.72E-05	6.48E-05	1.12E-04
**24h**	6.64E-06	1.61E-04	2.90E-04	7.83E-05
**36h**	1.08E-05	1.04E-04	1.44E-04	5.38E-04
**48h**	2.79E-05	4.10E-04	2.16E-04	5.21E-04
**72h**	4.77E-05	2.45E-03	7.95E-04	5.01E-04
	cgd6_810			
**2h**	5.35E-05	0.00E+00	0.00E+00	1.48E-05
**6h**	4.12E-05	2.20E-05	5.71E-05	3.84E-05
**12h**	3.62E-05	1.88E-05	5.53E-05	2.59E-05
**24h**	2.34E-05	6.23E-06	8.34E-05	2.05E-05
**36h**	3.20E-04	2.04E-05	5.91E-05	1.32E-05
**48h**	1.67E-04	2.42E-05	6.38E-05	9.85E-05
**72h**	6.29E-04	3.10E-05	3.12E-04	7.23E-04
	cgd6_830			
**2h**	2.93E-06	3.21E-06	1.13E-06	2.31E-06
**6h**	2.98E-06	1.04E-05	2.36E-06	4.10E-06
**12h**	2.65E-07	5.32E-07	1.19E-06	1.38E-06
**24h**	2.83E-06	4.48E-06	4.16E-06	8.58E-06
**36h**	2.29E-06	3.50E-06	2.08E-06	3.33E-06

**48h**	4.16E-06	2.46E-06	2.89E-06	3.54E-06
**72h**	6.63E-07	1.24E-06	1.69E-06	1.36E-06
	cgd6_860			
**2h**	3.68E-05	1.40E-04	3.12E-05	2.79E-05
**6h**	3.71E-05	2.08E-04	1.12E-04	3.29E-05
**12h**	1.98E-06	3.31E-04	1.05E-05	8.78E-06
**24h**	2.73E-05	6.60E-05	6.46E-05	5.11E-05
**36h**	1.93E-05	2.96E-04	4.07E-05	2.72E-05
**48h**	2.27E-05	7.56E-05	7.87E-05	2.36E-05
**72h**	3.99E-06	5.50E-05	2.80E-05	1.81E-05
	cgd6_880			
**2h**	0.00E+00	0.00E+00	0.00E+00	4.54E-08
**6h**	0.00E+00	0.00E+00	0.00E+00	5.68E-08
**12h**	2.08E-05	2.91E-04	2.66E-05	1.46E-05
**24h**	1.32E-05	1.48E-04	1.85E-05	2.12E-05
**36h**	3.02E-05	2.60E-04	1.75E-04	4.81E-05
**48h**	3.85E-04	3.28E-04	9.57E-04	3.97E-04
**72h**	5.09E-05	1.44E-04	4.94E-04	7.69E-05
	cgd7_1030			
**2h**	3.77E-04	2.50E-04	3.93E-04	2.66E-04
**6h**	1.32E-04	1.86E-04	1.37E-04	3.74E-05
**12h**	4.23E-05	2.66E-04	2.08E-04	4.18E-05
**24h**	2.34E-04	2.30E-04	1.99E-04	1.03E-04
**36h**	6.03E-05	1.72E-04	8.77E-05	5.01E-05
**48h**	4.25E-05	9.59E-05	1.35E-04	3.53E-05
**72h**	1.93E-05	8.34E-05	2.47E-04	8.01E-05
	cgd7_1040			
**2h**	2.28E-05	1.74E-04	7.64E-05	2.10E-05
**6h**	7.19E-07	7.59E-05	6.53E-07	2.26E-07
**12h**	2.64E-05	2.36E-04	1.74E-05	1.26E-05
**24h**	1.78E-05	2.66E-05	1.10E-05	1.63E-05
**36h**	1.31E-05	3.24E-05	1.94E-05	4.90E-05
**48h**	1.63E-05	1.93E-05	2.74E-05	1.30E-05
**72h**	6.47E-06	5.91E-05	9.22E-05	9.48E-07
	cgd7_1050			
**2h**	4.46E-06	1.95E-05	1.32E-06	4.91E-07
**6h**	0.00E+00	1.61E-05	4.12E-09	1.85E-07
**12h**	2.06E-06	2.11E-05	4.88E-06	1.79E-06
**24h**	3.92E-06	1.05E-05	4.54E-07	1.11E-06
**36h**	7.55E-07	9.46E-06	9.94E-07	1.61E-06
**48h**	6.41E-07	4.99E-06	2.69E-06	8.19E-07
**72h**	4.54E-07	1.51E-06	2.72E-06	3.63E-07
	cgd7_1070			
**2h**	6.03E-08	4.02E-07	6.68E-09	1.81E-08
**6h**	6.70E-08	1.29E-06	8.11E-08	1.52E-07
**12h**	1.32E-09	4.65E-08	7.69E-08	5.81E-09
**24h**	2.71E-08	5.11E-08	1.21E-07	9.05E-08
**36h**	6.24E-08	6.04E-07	1.30E-07	8.26E-08
**48h**	1.19E-07	3.21E-07	2.16E-07	4.28E-08
**72h**	3.13E-08	1.66E-07	1.16E-07	2.69E-08
	cgd7_1080			
**2h**	1.31E-03	1.81E-04	2.42E-04	6.16E-04
**6h**	2.88E-04	4.40E-04	2.84E-04	3.54E-04

**12h**	2.29E-04	4.79E-05	5.20E-05	4.98E-05
**24h**	2.75E-04	1.78E-04	1.23E-04	1.23E-04
**36h**	1.71E-04	1.58E-04	4.86E-05	6.96E-04
**48h**	1.40E-04	7.25E-05	7.87E-05	1.84E-04
**72h**	1.48E-04	5.03E-05	1.45E-04	8.61E-05
	cgd7_1100			
**2h**	1.19E-04	3.65E-04	0.00E+00	2.44E-04
**6h**	1.04E-04	6.20E-04	9.34E-04	3.52E-04
**12h**	9.18E-05	6.07E-04	7.52E-04	1.40E-04
**24h**	3.41E-04	2.99E-04	1.71E-04	3.99E-04
**36h**	2.52E-04	3.03E-04	3.12E-04	4.37E-04
**48h**	2.68E-04	1.16E-04	1.53E-04	3.73E-04
**72h**	3.50E-04	1.61E-04	9.11E-04	3.73E-04
	cgd7_1120			
**2h**	2.54E-06	2.92E-06	1.69E-06	2.90E-07
**6h**	2.48E-06	9.78E-07	1.49E-06	3.35E-07
**12h**	5.30E-07	1.03E-06	7.48E-07	2.80E-07
**24h**	8.29E-07	7.64E-07	9.15E-07	7.75E-07
**36h**	6.40E-07	7.17E-07	6.00E-07	7.97E-07
**48h**	3.99E-07	6.52E-07	1.14E-06	8.48E-07
**72h**	3.24E-07	2.67E-07	6.40E-07	5.01E-07
	cgd7_1170			
**2h**	1.93E-04	9.33E-04	6.55E-05	3.86E-05
**6h**	4.16E-05	8.34E-04	1.53E-04	3.39E-05
**12h**	4.69E-06	1.36E-05	1.19E-04	2.06E-05
**24h**	4.43E-05	1.96E-04	1.81E-04	6.78E-05
**36h**	7.37E-05	2.93E-04	1.60E-04	6.62E-05
**48h**	2.97E-05	1.65E-04	1.23E-04	2.05E-05
**72h**	2.64E-05	7.33E-05	1.82E-04	1.79E-04
	cgd7_1210			
**2h**	9.44E-08	8.02E-08	0.00E+00	7.03E-06
**6h**	9.15E-09	0.00E+00	0.00E+00	0.00E+00
**12h**	8.10E-06	1.17E-05	6.44E-05	5.76E-05
**24h**	1.75E-06	8.21E-06	1.24E-05	2.72E-05
**36h**	5.71E-06	8.95E-06	1.14E-04	1.63E-04
**48h**	4.32E-06	3.25E-06	5.07E-05	3.84E-05
**72h**	1.99E-06	1.79E-06	3.71E-05	6.04E-05
	cgd7_1310			
**2h**	0.00E+00	0.00E+00	0.00E+00	3.32E-08
**6h**	0.00E+00	5.81E-06	1.28E-05	2.03E-05
**12h**	1.38E-06	7.23E-07	5.57E-06	4.72E-06
**24h**	2.41E-06	3.07E-06	2.77E-05	1.98E-05
**36h**	4.27E-06	5.48E-06	1.50E-05	3.27E-05
**48h**	6.75E-06	3.23E-06	1.71E-05	1.23E-05
**72h**	4.63E-06	5.07E-07	3.18E-06	2.01E-06
	cgd7_1330			
**2h**	4.57E-08	0.00E+00	0.00E+00	0.00E+00
**6h**	2.97E-05	0.00E+00	0.00E+00	0.00E+00
**12h**	1.22E-04	2.55E-04	1.07E-03	4.37E-04
**24h**	3.21E-05	3.08E-05	8.65E-05	1.14E-04
**36h**	1.32E-05	4.93E-05	8.10E-04	1.84E-04
**48h**	4.38E-05	4.14E-05	4.20E-04	1.42E-04
**72h**	5.63E-05	6.25E-05	2.07E-03	5.67E-04

	cgd7_1340			
**2h**	0.00E+00	0.00E+00	0.00E+00	9.48E-09
**6h**	3.09E-09	2.59E-09	0.00E+00	0.00E+00
**12h**	2.14E-06	3.86E-06	3.88E-06	1.16E-06
**24h**	2.32E-06	1.88E-06	3.64E-07	3.78E-07
**36h**	1.21E-06	2.41E-06	3.43E-06	1.41E-06
**48h**	8.78E-07	3.48E-07	8.86E-07	2.76E-07
**72h**	1.01E-07	9.91E-08	1.74E-07	9.16E-08
	cgd7_1360			
**2h**	1.40E-06	5.91E-07	2.90E-06	1.12E-06
**6h**	2.15E-07	4.46E-07	1.04E-06	8.15E-07
**12h**	1.93E-06	1.12E-06	9.90E-07	1.02E-06
**24h**	2.01E-07	4.94E-07	1.47E-06	6.14E-07
**36h**	6.44E-07	7.01E-07	7.05E-07	1.04E-06
**48h**	3.02E-07	9.73E-08	9.82E-07	1.47E-06
**72h**	2.36E-07	2.43E-07	6.98E-07	6.07E-07
	cgd7_150			
**2h**	4.98E-08	0.00E+00	0.00E+00	0.00E+00
**6h**	0.00E+00	0.00E+00	0.00E+00	0.00E+00
**12h**	9.39E-09	8.51E-08	0.00E+00	0.00E+00
**24h**	2.77E-09	3.11E-09	3.40E-08	3.22E-06
**36h**	1.12E-08	2.97E-08	3.65E-08	8.15E-08
**48h**	7.79E-08	2.02E-08	1.19E-06	5.18E-07
**72h**	5.43E-08	4.39E-08	4.05E-07	3.06E-06
	cgd7_1520			
**2h**	8.65E-08	2.29E-07	8.46E-08	3.81E-07
**6h**	2.46E-07	3.39E-07	1.16E-06	2.90E-06
**12h**	1.72E-07	2.78E-07	5.21E-07	1.53E-06
**24h**	3.34E-07	5.21E-07	6.23E-07	7.49E-07
**36h**	1.65E-07	5.29E-07	1.02E-06	1.43E-06
**48h**	1.95E-07	1.69E-07	7.89E-07	9.12E-07
**72h**	2.21E-07	1.11E-07	5.14E-07	6.17E-07
	cgd7_1530			
**2h**	1.47E-04	2.03E-04	4.16E-04	1.86E-03
**6h**	1.67E-05	4.51E-05	6.79E-05	1.64E-04
**12h**	4.43E-05	7.15E-05	2.98E-04	4.38E-04
**24h**	9.79E-05	3.17E-04	1.55E-04	2.49E-04
**36h**	1.40E-05	4.55E-05	1.16E-04	1.43E-04
**48h**	1.52E-05	2.90E-05	8.40E-05	1.47E-04
**72h**	6.93E-06	5.82E-06	1.00E-04	6.24E-05
	cgd7_1560			
**2h**	1.45E-06	7.95E-07	2.45E-07	1.50E-06
**6h**	1.87E-08	3.68E-08	2.53E-08	4.09E-07
**12h**	3.61E-08	1.14E-08	1.06E-07	2.05E-07
**24h**	4.24E-07	1.86E-06	7.08E-07	6.05E-07
**36h**	4.80E-07	9.43E-07	9.69E-07	6.37E-07
**48h**	7.59E-07	1.03E-06	1.56E-06	4.69E-06
**72h**	8.42E-07	3.95E-07	2.18E-06	9.88E-07
	cgd7_1570			
**2h**	3.24E-06	5.01E-07	2.63E-06	2.84E-05
**6h**	7.70E-07	5.75E-07	2.04E-06	7.74E-06
**12h**	7.64E-08	2.93E-09	9.20E-07	8.59E-07
**24h**	9.35E-07	1.89E-06	2.22E-06	2.87E-06

**36h**	1.25E-06	3.04E-06	4.78E-06	4.02E-06
**48h**	2.41E-06	3.01E-06	1.08E-05	1.60E-05
**72h**	2.35E-06	1.61E-06	1.62E-05	8.43E-06
	cgd7_1600			
**2h**	1.01E-06	1.31E-06	1.92E-06	2.23E-06
**6h**	1.76E-07	2.68E-07	3.64E-08	4.70E-07
**12h**	3.64E-07	7.16E-07	3.50E-07	6.32E-07
**24h**	5.57E-07	1.03E-06	6.97E-07	4.64E-07
**36h**	4.73E-07	6.40E-07	5.63E-07	4.13E-07
**48h**	2.75E-07	2.89E-07	7.84E-07	6.07E-07
**72h**	1.21E-07	1.62E-07	5.72E-07	3.95E-07
	cgd7_1620			
**2h**	3.11E-05	7.15E-04	3.24E-04	2.25E-04
**6h**	1.45E-05	1.95E-04	9.66E-05	1.08E-04
**12h**	9.50E-05	2.13E-03	3.48E-04	4.68E-04
**24h**	4.10E-05	1.33E-03	1.74E-04	2.00E-04
**36h**	1.68E-05	2.86E-04	1.77E-04	3.15E-04
**48h**	1.74E-05	2.25E-04	3.32E-04	1.14E-04
**72h**	2.14E-05	2.76E-04	2.77E-04	4.95E-05
	cgd7_1660			
**2h**	2.40E-08	0.00E+00	0.00E+00	3.58E-09
**6h**	8.29E-09	1.38E-08	7.13E-09	8.36E-09
**12h**	2.86E-06	4.27E-06	3.57E-06	1.25E-06
**24h**	9.20E-07	6.28E-07	4.59E-07	4.24E-07
**36h**	1.50E-07	3.89E-07	1.24E-06	7.50E-07
**48h**	3.52E-07	3.99E-07	5.07E-07	3.69E-07
**72h**	1.60E-07	2.81E-07	5.73E-07	3.04E-07
	cgd7_170			
**2h**	1.96E-05	1.82E-04	2.51E-05	4.06E-05
**6h**	2.38E-05	5.21E-05	1.58E-05	1.66E-05
**12h**	1.81E-05	1.51E-04	1.68E-04	1.17E-05
**24h**	9.29E-06	1.32E-04	1.03E-04	3.41E-05
**36h**	3.09E-06	7.50E-05	1.02E-04	2.22E-05
**48h**	5.28E-06	4.53E-05	8.81E-05	1.38E-05
**72h**	1.17E-05	1.05E-04	4.73E-04	1.90E-05
	cgd7_1740			
**2h**	1.26E-04	2.40E-05	1.96E-07	8.87E-05
**6h**	1.85E-05	2.00E-06	3.88E-07	4.97E-06
**12h**	8.18E-06	6.46E-07	7.82E-07	7.40E-06
**24h**	1.07E-05	6.80E-06	1.05E-06	1.36E-05
**36h**	1.16E-05	4.04E-06	8.96E-07	7.80E-06
**48h**	6.42E-06	6.55E-07	2.00E-07	6.76E-06
**72h**	1.26E-06	5.46E-07	1.67E-07	2.08E-06
	cgd7_1870			
**2h**	0.00E+00	0.00E+00	0.00E+00	0.00E+00
**6h**	0.00E+00	1.31E-08	0.00E+00	0.00E+00
**12h**	0.00E+00	0.00E+00	0.00E+00	0.00E+00
**24h**	0.00E+00	4.65E-10	1.64E-10	3.74E-09
**36h**	1.58E-08	1.68E-08	4.05E-09	3.35E-08
**48h**	4.20E-08	9.95E-09	1.07E-08	3.79E-08
**72h**	4.88E-09	1.60E-08	7.23E-08	9.74E-08
	cgd7_190			
**2h**	0.00E+00	0.00E+00	0.00E+00	0.00E+00

**6h**	4.13E-08	1.00E-08	0.00E+00	1.22E-08
**12h**	5.24E-08	9.54E-09	1.19E-08	1.29E-08
**24h**	1.17E-08	2.50E-08	1.17E-08	8.64E-08
**36h**	8.42E-08	3.73E-08	2.95E-08	2.77E-07
**48h**	1.18E-07	4.92E-08	2.36E-08	1.36E-07
**72h**	2.07E-07	8.30E-08	2.27E-07	9.69E-08
	cgd7_20			
**2h**	1.49E-05	1.18E-06	2.42E-06	1.92E-06
**6h**	5.95E-07	2.17E-07	2.67E-07	8.26E-07
**12h**	1.55E-06	4.61E-07	1.08E-06	1.69E-06
**24h**	1.57E-06	1.81E-06	3.69E-07	9.35E-07
**36h**	6.47E-07	4.36E-07	6.93E-07	2.52E-06
**48h**	9.24E-07	4.92E-07	2.94E-07	6.47E-07
**72h**	2.47E-07	1.69E-07	3.50E-08	2.19E-08
	cgd7_2060			
**2h**	4.44E-06	0.00E+00	0.00E+00	0.00E+00
**6h**	2.26E-05	3.67E-04	2.72E-03	1.17E-04
**12h**	3.17E-06	2.53E-04	3.85E-04	2.36E-06
**24h**	4.61E-06	2.06E-04	1.35E-03	5.28E-05
**36h**	1.67E-05	2.81E-04	1.82E-03	1.56E-04
**48h**	1.64E-05	7.96E-05	3.53E-04	5.07E-05
**72h**	3.02E-06	5.27E-05	6.88E-04	1.62E-05
	cgd7_2150			
**2h**	0.00E+00	0.00E+00	0.00E+00	0.00E+00
**6h**	1.20E-06	3.65E-05	1.48E-04	2.50E-06
**12h**	1.78E-06	1.78E-05	5.62E-05	6.13E-07
**24h**	1.79E-07	2.47E-05	1.17E-04	2.72E-06
**36h**	1.37E-06	4.05E-05	4.56E-05	5.96E-06
**48h**	2.51E-06	1.77E-05	6.77E-05	2.44E-06
**72h**	6.22E-07	1.88E-05	8.35E-04	3.04E-06
	cgd7_2180			
**2h**	4.17E-05	3.92E-06	1.41E-07	1.52E-05
**6h**	3.32E-05	4.51E-07	5.30E-08	4.70E-06
**12h**	8.90E-06	0.00E+00	3.73E-07	1.89E-06
**24h**	1.54E-05	1.02E-06	1.35E-07	3.00E-06
**36h**	5.87E-06	9.95E-07	1.75E-07	2.71E-06
**48h**	2.04E-05	6.65E-07	4.33E-07	4.91E-06
**72h**	1.53E-05	8.28E-07	4.85E-07	1.81E-06
	cgd7_220			
**2h**	1.36E-03	8.04E-05	1.58E-05	2.60E-04
**6h**	7.89E-04	1.37E-04	7.61E-05	1.33E-04
**12h**	6.55E-04	3.31E-05	1.63E-05	8.95E-05
**24h**	1.63E-04	3.31E-05	4.27E-05	9.51E-05
**36h**	6.58E-04	3.05E-05	2.12E-05	6.16E-05
**48h**	1.44E-04	1.45E-05	1.47E-05	5.28E-05
**72h**	7.63E-05	1.10E-05	7.56E-06	6.71E-06
	cgd7_2220			
**2h**	1.82E-06	2.24E-05	0.00E+00	4.59E-05
**6h**	0.00E+00	9.14E-06	0.00E+00	0.00E+00
**12h**	1.78E-05	3.03E-04	5.24E-04	4.54E-05
**24h**	4.46E-06	1.98E-04	2.22E-04	5.59E-05
**36h**	7.57E-06	1.69E-04	3.59E-03	1.84E-04
**48h**	3.88E-06	9.82E-05	7.40E-05	1.65E-05

**72h**	2.90E-06	5.62E-05	6.25E-04	1.64E-05
	cgd7_2230			
**2h**	3.34E-05	0.00E+00	0.00E+00	0.00E+00
**6h**	0.00E+00	3.57E-07	0.00E+00	6.20E-07
**12h**	7.81E-05	6.29E-06	4.77E-05	1.91E-05
**24h**	1.16E-05	1.51E-05	4.93E-05	7.09E-06
**36h**	4.60E-05	7.82E-05	1.72E-04	5.75E-05
**48h**	8.82E-05	3.77E-05	1.11E-04	2.52E-05
**72h**	4.37E-05	2.01E-05	1.33E-04	5.28E-05
	cgd7_2240			
**2h**	1.06E-04	9.23E-06	2.24E-05	3.53E-06
**6h**	1.77E-05	4.89E-06	7.48E-06	1.33E-06
**12h**	4.56E-05	7.24E-06	3.85E-05	3.03E-06
**24h**	4.78E-05	1.64E-05	1.23E-05	4.15E-06
**36h**	1.07E-05	4.01E-06	8.09E-06	3.23E-06
**48h**	1.25E-05	2.31E-06	7.23E-06	5.34E-06
**72h**	3.39E-06	8.54E-07	6.16E-06	4.86E-07
	cgd7_2250			
**2h**	2.28E-03	2.54E-02	2.50E-03	2.04E-02
**6h**	7.84E-03	2.34E-02	1.87E-02	8.20E-02
**12h**	9.11E-04	1.15E-02	2.58E-03	1.03E-02
**24h**	1.11E-03	1.44E-02	2.79E-03	5.34E-03
**36h**	1.55E-03	5.92E-03	1.32E-03	1.20E-02
**48h**	6.09E-04	3.53E-03	9.10E-04	1.12E-02
**72h**	1.78E-04	9.70E-04	4.70E-04	1.48E-03
	cgd7_2350			
**2h**	4.90E-08	1.46E-08	0.00E+00	0.00E+00
**6h**	1.35E-08	4.79E-09	8.71E-09	2.11E-08
**12h**	3.73E-07	7.37E-07	2.44E-07	6.51E-09
**24h**	1.01E-07	3.38E-08	1.55E-07	2.81E-08
**36h**	6.38E-07	1.51E-07	9.33E-07	2.85E-07
**48h**	1.25E-06	1.06E-07	5.08E-07	1.28E-07
**72h**	2.74E-07	2.56E-07	1.23E-06	3.71E-08
	cgd7_2390			
**2h**	6.30E-07	3.88E-07	2.31E-07	7.85E-08
**6h**	3.00E-06	3.89E-07	8.38E-07	4.78E-07
**12h**	4.96E-07	6.37E-08	5.69E-07	1.35E-07
**24h**	7.33E-07	4.00E-07	6.94E-07	4.34E-07
**36h**	6.34E-07	4.56E-07	5.31E-07	4.79E-07
**48h**	7.12E-07	2.42E-07	4.23E-07	2.79E-07
**72h**	1.89E-07	1.54E-07	7.00E-07	1.26E-07
	cgd7_2420			
**2h**	2.00E-03	7.52E-03	2.28E-03	1.94E-03
**6h**	3.04E-03	9.94E-03	1.91E-03	4.97E-03
**12h**	7.59E-04	1.07E-03	1.18E-03	1.45E-03
**24h**	1.83E-03	6.90E-03	1.78E-03	2.83E-03
**36h**	2.38E-04	6.18E-03	5.74E-04	1.52E-03
**48h**	2.72E-04	1.94E-03	6.28E-04	1.79E-03
**72h**	1.82E-04	4.81E-04	4.04E-04	3.36E-04
	cgd7_2630			
**2h**	7.24E-07	6.02E-07	2.09E-07	1.28E-07
**6h**	1.84E-07	2.25E-07	5.49E-07	1.83E-07
**12h**	7.61E-08	6.21E-08	3.34E-07	4.92E-08

**24h**	2.59E-07	3.64E-07	8.09E-07	3.92E-07
**36h**	4.28E-07	4.63E-07	3.67E-07	2.27E-07
**48h**	2.61E-07	1.90E-07	5.67E-07	2.44E-07
**72h**	1.06E-07	8.32E-08	9.83E-08	5.32E-08
	cgd7_2730			
**2h**	1.38E-06	1.33E-06	2.04E-07	5.58E-07
**6h**	4.29E-07	4.01E-07	1.13E-06	8.95E-07
**12h**	7.21E-07	1.83E-07	2.69E-06	3.92E-07
**24h**	1.36E-06	9.21E-07	1.54E-06	8.21E-07
**36h**	5.24E-07	1.03E-06	1.01E-06	6.43E-07
**48h**	5.83E-07	3.91E-07	9.05E-07	5.39E-07
**72h**	5.24E-07	1.56E-07	4.01E-07	2.08E-07
	cgd7_2800			
**2h**	5.03E-08	1.20E-07	9.21E-08	2.44E-08
**6h**	3.01E-07	1.15E-07	7.16E-07	1.04E-07
**12h**	2.21E-07	6.28E-09	4.70E-08	4.59E-09
**24h**	9.53E-08	1.59E-07	4.33E-07	1.61E-07
**36h**	3.14E-07	1.27E-07	1.42E-07	1.29E-07
**48h**	7.55E-07	5.49E-07	1.15E-06	4.34E-07
**72h**	5.62E-07	1.32E-07	1.85E-07	1.71E-07
	cgd7_2810			
**2h**	1.62E-05	1.59E-06	1.17E-05	9.25E-07
**6h**	2.62E-05	4.76E-06	2.68E-05	8.01E-06
**12h**	3.95E-06	1.07E-06	1.54E-06	2.19E-07
**24h**	6.64E-06	6.65E-06	3.32E-05	3.31E-06
**36h**	3.50E-05	5.61E-06	2.66E-05	2.89E-06
**48h**	3.74E-05	3.38E-06	4.53E-05	1.54E-05
**72h**	3.88E-06	4.97E-07	1.59E-06	9.40E-07
	cgd7_2890			
**2h**	6.30E-04	3.60E-03	6.95E-04	1.53E-03
**6h**	1.24E-03	5.90E-03	1.49E-03	8.79E-03
**12h**	2.66E-04	1.80E-03	3.76E-04	1.30E-03
**24h**	3.25E-04	2.36E-03	1.08E-03	2.79E-03
**36h**	1.88E-04	9.88E-04	7.36E-04	2.55E-03
**48h**	3.86E-04	4.54E-04	5.02E-04	2.69E-04
**72h**	7.73E-05	2.54E-04	2.25E-04	2.09E-04
	cgd7_2940			
**2h**	3.82E-06	3.78E-06	1.65E-06	2.40E-06
**6h**	4.03E-05	6.71E-05	9.51E-05	2.14E-05
**12h**	7.63E-06	3.38E-05	4.15E-06	5.90E-06
**24h**	1.62E-05	2.21E-05	4.64E-05	1.20E-05
**36h**	5.83E-05	4.63E-05	2.87E-05	2.16E-05
**48h**	3.21E-05	1.17E-05	4.73E-05	5.70E-06
**72h**	7.47E-06	3.70E-06	7.96E-06	1.38E-06
	cgd7_3020			
**2h**	1.21E-05	4.68E-06	3.78E-06	1.34E-05
**6h**	4.42E-06	9.31E-06	2.46E-06	5.14E-06
**12h**	3.84E-06	2.22E-06	3.90E-06	1.57E-06
**24h**	4.09E-06	5.51E-06	2.91E-06	3.41E-06
**36h**	4.98E-06	3.41E-06	5.88E-06	5.45E-06
**48h**	1.28E-05	4.30E-06	5.06E-06	2.51E-06
**72h**	1.65E-06	2.06E-06	3.17E-06	1.98E-06
	cgd7_310			

**2h**	5.01E-05	1.17E-05	7.18E-05	6.20E-05
**6h**	2.20E-04	1.53E-05	4.89E-05	1.88E-05
**12h**	4.71E-05	4.14E-06	1.88E-05	1.20E-06
**24h**	4.87E-05	8.61E-06	2.33E-05	7.91E-06
**36h**	8.73E-05	1.26E-05	2.42E-05	1.40E-05
**48h**	6.60E-04	3.79E-05	1.45E-04	6.48E-05
**72h**	6.85E-04	1.33E-04	2.68E-04	5.00E-05
	cgd7_3150			
**2h**	0.00E+00	3.71E-05	0.00E+00	0.00E+00
**6h**	0.00E+00	0.00E+00	0.00E+00	1.11E-05
**12h**	7.13E-07	6.96E-06	3.77E-05	7.30E-07
**24h**	5.08E-07	3.22E-05	6.95E-05	1.44E-05
**36h**	6.10E-06	1.01E-04	9.56E-05	1.05E-04
**48h**	2.07E-05	4.41E-05	1.45E-04	5.24E-05
**72h**	9.51E-06	1.19E-04	1.48E-04	7.41E-05
	cgd7_3210			
**2h**	6.97E-06	3.12E-05	0.00E+00	8.52E-05
**6h**	0.00E+00	3.58E-05	1.96E-05	1.21E-05
**12h**	1.37E-02	9.11E-03	1.62E-03	1.05E-03
**24h**	9.26E-04	4.92E-04	6.28E-04	2.73E-04
**36h**	9.23E-04	6.21E-04	1.94E-03	1.32E-03
**48h**	5.11E-04	2.09E-04	8.31E-04	3.48E-04
**72h**	7.94E-05	1.15E-04	3.36E-04	9.15E-05
	cgd7_3240			
**2h**	5.80E-06	1.06E-04	2.45E-05	2.67E-04
**6h**	1.05E-05	1.42E-04	5.00E-05	1.68E-04
**12h**	1.75E-06	8.81E-06	1.57E-05	3.43E-05
**24h**	5.26E-06	2.07E-05	2.60E-05	5.54E-05
**36h**	3.37E-06	1.85E-05	1.61E-05	2.40E-05
**48h**	9.52E-06	7.74E-06	1.51E-05	2.19E-05
**72h**	1.47E-06	1.24E-05	1.78E-05	1.59E-05
	cgd7_3260			
**2h**	9.86E-07	1.11E-04	2.09E-05	6.75E-06
**6h**	2.73E-06	2.98E-04	1.44E-04	2.18E-04
**12h**	3.78E-07	7.10E-06	5.87E-06	5.06E-06
**24h**	6.93E-07	1.32E-05	3.93E-05	6.94E-05
**36h**	4.56E-06	2.04E-05	2.10E-05	5.21E-05
**48h**	2.27E-06	3.03E-05	2.86E-05	7.31E-05
**72h**	6.53E-07	4.45E-06	5.93E-06	4.27E-06
	cgd7_3300			
**2h**	5.81E-03	2.15E-03	4.01E-03	5.78E-04
**6h**	2.88E-03	1.48E-03	1.05E-03	1.52E-04
**12h**	3.37E-03	1.87E-03	1.27E-03	3.74E-04
**24h**	1.87E-03	6.61E-04	5.08E-04	6.09E-04
**36h**	1.78E-03	6.64E-04	4.63E-04	3.31E-04
**48h**	1.99E-03	4.91E-04	1.05E-03	1.31E-04
**72h**	3.34E-04	4.15E-04	5.43E-04	6.59E-05
	cgd7_3310			
**2h**	3.84E-06	1.99E-06	5.65E-06	1.17E-05
**6h**	2.73E-06	4.20E-06	1.50E-06	3.89E-06
**12h**	2.54E-06	2.91E-06	3.82E-06	2.64E-06
**24h**	3.15E-06	1.56E-06	1.69E-06	9.75E-07
**36h**	9.24E-07	9.74E-07	1.77E-06	1.41E-06

**48h**	1.32E-06	5.91E-07	8.84E-07	6.68E-07
**72h**	4.94E-07	4.49E-07	3.99E-07	5.05E-07
	cgd7_3330			
**2h**	1.71E-07	0.00E+00	0.00E+00	0.00E+00
**6h**	0.00E+00	4.12E-08	0.00E+00	0.00E+00
**12h**	1.07E-05	1.17E-05	5.57E-06	5.16E-06
**24h**	1.34E-06	3.60E-06	6.94E-07	9.39E-07
**36h**	1.22E-06	1.73E-06	6.53E-06	3.33E-06
**48h**	1.11E-06	6.98E-07	9.79E-07	1.81E-06
**72h**	6.20E-07	7.84E-07	1.14E-06	1.97E-07
	cgd7_3430			
**2h**	3.53E-05	7.86E-05	7.92E-05	2.35E-04
**6h**	2.65E-06	2.87E-05	1.57E-06	4.77E-05
**12h**	1.96E-05	1.09E-04	4.17E-05	8.49E-05
**24h**	1.78E-05	4.41E-05	3.43E-05	2.99E-05
**36h**	9.61E-06	6.05E-05	4.28E-05	5.64E-05
**48h**	3.69E-05	5.05E-05	4.89E-05	7.94E-05
**72h**	1.05E-05	1.51E-05	1.87E-05	3.08E-05
	cgd7_3500			
**2h**	5.25E-05	3.53E-04	2.37E-04	2.58E-04
**6h**	1.76E-05	1.80E-04	1.02E-04	6.31E-04
**12h**	2.62E-05	2.00E-05	7.60E-05	1.39E-04
**24h**	2.28E-05	1.01E-04	1.12E-04	1.79E-04
**36h**	2.84E-05	1.72E-04	5.18E-05	1.05E-04
**48h**	3.49E-05	1.21E-04	9.87E-05	1.69E-04
**72h**	1.48E-05	3.85E-05	3.04E-05	3.63E-05
	cgd7_3550			
**2h**	6.60E-04	1.80E-03	9.19E-04	4.65E-03
**6h**	1.22E-04	2.86E-04	1.31E-04	3.78E-04
**12h**	1.83E-04	1.84E-04	2.54E-04	7.44E-04
**24h**	2.89E-04	6.94E-04	2.26E-04	6.07E-04
**36h**	7.82E-05	2.02E-04	1.39E-04	2.90E-04
**48h**	2.45E-04	2.51E-04	3.48E-04	2.66E-04
**72h**	2.67E-05	9.81E-05	1.36E-04	7.79E-05
	cgd7_3570			
**2h**	6.55E-05	7.90E-06	5.30E-06	2.55E-04
**6h**	8.00E-05	1.16E-05	8.78E-06	7.94E-05
**12h**	1.55E-05	9.97E-06	3.58E-06	1.84E-05
**24h**	4.42E-05	7.76E-06	1.26E-05	2.25E-04
**36h**	6.79E-05	1.96E-05	7.68E-06	2.03E-04
**48h**	8.70E-05	1.28E-05	2.87E-05	1.53E-04
**72h**	1.54E-04	5.71E-06	3.94E-06	2.50E-05
	cgd7_3610			
**2h**	9.41E-07	5.75E-07	6.58E-07	1.89E-06
**6h**	1.68E-06	1.64E-06	1.84E-06	8.33E-06
**12h**	1.63E-07	9.72E-08	5.61E-07	6.05E-07
**24h**	1.30E-06	6.59E-07	1.42E-06	4.20E-06
**36h**	1.97E-06	9.48E-07	4.10E-07	1.44E-06
**48h**	7.08E-07	2.03E-07	3.23E-07	1.12E-06
**72h**	2.03E-07	1.11E-07	5.38E-08	3.06E-07
	cgd7_3690			
**2h**	1.34E-06	0.00E+00	0.00E+00	0.00E+00
**6h**	2.40E-07	3.09E-08	2.64E-08	3.12E-07

**12h**	0.00E+00	2.27E-08	2.29E-08	3.64E-07
**24h**	1.68E-08	6.12E-09	1.25E-08	1.36E-07
**36h**	2.60E-08	2.75E-08	2.19E-08	1.30E-07
**48h**	7.70E-08	1.98E-08	3.71E-08	1.19E-07
**72h**	8.35E-08	2.31E-08	5.35E-08	5.48E-07
	cgd7_3720			
**2h**	6.93E-04	4.91E-05	4.77E-06	2.11E-04
**6h**	9.19E-04	7.90E-05	1.29E-05	5.25E-04
**12h**	1.93E-05	5.37E-07	2.27E-05	5.64E-05
**24h**	1.50E-04	3.88E-05	3.85E-05	3.57E-04
**36h**	2.68E-04	4.51E-05	1.07E-05	1.10E-04
**48h**	2.66E-04	2.35E-05	2.84E-05	1.06E-04
**72h**	8.98E-05	2.38E-05	7.02E-06	6.66E-05
	cgd7_3740			
**2h**	8.78E-08	1.52E-08	4.84E-08	1.03E-07
**6h**	4.68E-07	2.75E-07	8.88E-08	1.02E-06
**12h**	1.16E-08	1.03E-08	1.21E-08	3.18E-08
**24h**	1.08E-07	3.77E-08	1.58E-07	5.40E-07
**36h**	1.67E-07	1.78E-07	1.05E-07	5.51E-07
**48h**	1.70E-07	3.51E-08	1.26E-07	7.81E-08
**72h**	3.98E-08	7.74E-08	1.68E-08	5.86E-08
	cgd7_390			
**2h**	2.48E-06	6.08E-07	1.64E-08	9.53E-06
**6h**	1.69E-05	3.24E-06	2.30E-06	1.88E-04
**12h**	1.46E-05	4.65E-06	4.47E-07	6.88E-05
**24h**	1.25E-05	4.63E-06	2.25E-06	1.03E-04
**36h**	2.64E-05	5.59E-06	1.90E-06	8.97E-05
**48h**	4.65E-05	9.99E-06	1.98E-06	1.15E-04
**72h**	4.46E-05	6.08E-06	4.57E-06	6.06E-05
	cgd7_4000			
**2h**	0.00E+00	0.00E+00	0.00E+00	0.00E+00
**6h**	0.00E+00	0.00E+00	0.00E+00	0.00E+00
**12h**	3.76E-06	1.38E-06	7.89E-07	6.78E-06
**24h**	1.19E-06	2.41E-07	7.42E-08	4.02E-06
**36h**	1.12E-06	4.07E-07	8.84E-07	9.53E-06
**48h**	2.06E-06	1.30E-07	5.87E-07	1.44E-06
**72h**	1.55E-06	7.52E-08	3.72E-07	2.10E-06
	cgd7_4060			
**2h**	3.00E-06	2.27E-06	8.66E-07	2.09E-06
**6h**	3.30E-06	5.78E-06	5.53E-06	1.27E-05
**12h**	3.79E-07	5.03E-07	1.40E-06	2.53E-06
**24h**	1.05E-06	1.77E-06	2.43E-06	5.00E-06
**36h**	1.35E-06	1.50E-06	1.84E-06	1.22E-06
**48h**	2.00E-06	6.93E-07	3.98E-06	1.63E-06
**72h**	4.12E-07	4.31E-07	4.30E-07	5.85E-07
	cgd7_4270			
**2h**	0.00E+00	2.22E-07	0.00E+00	3.88E-07
**6h**	0.00E+00	2.84E-08	1.59E-07	1.66E-07
**12h**	9.48E-07	1.15E-06	7.91E-07	6.05E-07
**24h**	5.75E-07	3.38E-07	3.54E-07	7.47E-07
**36h**	1.45E-06	1.60E-06	1.97E-06	2.49E-06
**48h**	3.01E-06	1.04E-06	1.93E-06	1.21E-06
**72h**	5.75E-07	3.94E-07	2.23E-07	7.17E-07

	cgd7_440			
**2h**	2.73E-03	6.46E-04	3.79E-05	3.48E-03
**6h**	1.30E-04	2.72E-04	5.14E-05	6.44E-04
**12h**	3.54E-04	3.59E-04	3.29E-04	1.02E-03
**24h**	1.56E-03	7.62E-04	2.58E-04	1.88E-03
**36h**	3.19E-04	2.69E-04	7.09E-05	2.92E-03
**48h**	1.66E-03	1.59E-04	2.31E-04	3.57E-04
**72h**	2.09E-04	1.51E-04	6.11E-05	1.99E-04
	cgd7_4430			
**2h**	2.54E-04	3.81E-04	0.00E+00	7.01E-05
**6h**	7.92E-04	1.83E-03	9.09E-04	2.94E-04
**12h**	3.49E-04	5.24E-04	1.40E-03	1.46E-04
**24h**	2.10E-04	1.35E-04	3.42E-04	1.95E-04
**36h**	3.36E-04	2.57E-04	2.95E-04	2.40E-04
**48h**	1.48E-03	1.35E-03	8.99E-04	4.05E-04
**72h**	4.49E-04	2.89E-04	9.85E-04	1.25E-04
	cgd7_4510			
**2h**	1.89E-03	2.24E-03	3.00E-04	2.15E-03
**6h**	7.43E-05	2.42E-04	4.79E-05	3.25E-04
**12h**	4.46E-04	1.12E-03	7.20E-04	1.21E-03
**24h**	1.87E-03	7.64E-04	2.13E-04	2.12E-03
**36h**	5.49E-04	1.08E-03	6.61E-04	2.62E-03
**48h**	1.13E-03	3.34E-04	3.75E-04	7.75E-04
**72h**	1.48E-04	2.27E-04	3.60E-05	2.38E-04
	cgd7_4550			
**2h**	2.04E-06	6.85E-05	0.00E+00	1.69E-05
**6h**	1.02E-04	2.15E-03	1.16E-03	1.35E-04
**12h**	1.96E-05	6.16E-04	5.33E-05	1.80E-05
**24h**	9.22E-06	2.12E-04	3.88E-04	7.60E-05
**36h**	2.28E-05	6.11E-04	9.44E-05	1.07E-04
**48h**	3.20E-05	6.22E-04	1.44E-04	5.36E-05
**72h**	1.18E-05	2.23E-04	1.08E-04	4.76E-05
	cgd7_4770			
**2h**	2.34E-06	4.29E-05	2.30E-04	3.37E-06
**6h**	6.33E-06	1.03E-06	2.98E-05	1.10E-05
**12h**	8.23E-06	0.00E+00	1.83E-05	1.13E-05
**24h**	3.20E-06	7.87E-05	1.78E-05	4.94E-06
**36h**	1.93E-06	9.08E-06	1.58E-05	3.14E-05
**48h**	5.49E-06	5.34E-05	3.42E-05	1.56E-05
**72h**	6.85E-06	1.52E-04	2.13E-04	1.57E-05
	cgd7_4800			
**2h**	8.68E-07	4.00E-07	7.84E-07	1.77E-06
**6h**	0.00E+00	0.00E+00	0.00E+00	2.05E-07
**12h**	2.76E-07	2.24E-08	7.31E-08	2.07E-08
**24h**	1.38E-07	3.77E-09	1.85E-08	3.79E-08
**36h**	1.58E-07	5.60E-08	4.83E-08	3.91E-07
**48h**	5.96E-06	4.42E-06	4.41E-06	7.29E-06
**72h**	2.02E-05	9.59E-06	2.85E-05	2.16E-05
	cgd7_4850			
**2h**	3.43E-07	2.41E-08	6.23E-08	5.92E-07
**6h**	0.00E+00	2.02E-08	3.50E-08	1.53E-07
**12h**	3.78E-07	1.80E-07	3.12E-07	1.52E-07
**24h**	8.54E-08	8.92E-08	1.84E-07	5.07E-08

**36h**	3.88E-07	7.20E-08	2.26E-07	5.79E-07
**48h**	2.23E-07	8.56E-08	8.27E-08	4.40E-07
**72h**	2.04E-07	2.59E-07	9.61E-07	8.75E-07
	cgd7_4890			
**2h**	0.00E+00	0.00E+00	0.00E+00	0.00E+00
**6h**	1.11E-06	1.49E-07	1.07E-06	1.55E-07
**12h**	3.83E-07	4.47E-08	1.43E-07	8.25E-08
**24h**	4.27E-07	7.81E-08	1.63E-06	8.87E-07
**36h**	2.17E-06	2.75E-07	8.01E-07	2.00E-06
**48h**	1.56E-06	1.78E-07	5.81E-07	1.06E-06
**72h**	4.93E-07	1.48E-07	1.01E-06	8.47E-07
	cgd7_4990			
**2h**	3.72E-06	1.58E-05	8.09E-05	2.69E-06
**6h**	0.00E+00	4.86E-07	0.00E+00	4.01E-06
**12h**	7.19E-05	4.73E-03	2.10E-04	3.78E-05
**24h**	1.29E-05	2.02E-04	6.71E-05	1.63E-05
**36h**	1.34E-05	4.12E-04	2.00E-04	1.03E-04
**48h**	2.55E-05	4.14E-04	1.19E-04	7.51E-05
**72h**	7.81E-05	1.80E-03	5.73E-04	1.12E-04
	cgd7_5070			
**2h**	7.12E-07	2.63E-07	7.53E-07	1.30E-06
**6h**	6.78E-07	4.55E-07	9.77E-07	1.69E-06
**12h**	9.58E-08	4.12E-09	1.92E-07	7.08E-08
**24h**	1.65E-07	2.54E-07	6.73E-07	2.75E-07
**36h**	5.74E-07	1.69E-07	3.01E-07	5.48E-07
**48h**	2.02E-06	4.28E-04	5.51E-07	1.58E-06
**72h**	5.53E-07	2.89E-07	1.41E-07	2.71E-07
	cgd7_510			
**2h**	4.08E-05	1.83E-05	2.10E-04	1.09E-04
**6h**	6.34E-05	1.61E-05	6.76E-05	1.33E-04
**12h**	2.63E-05	3.24E-06	1.39E-04	5.30E-05
**24h**	6.35E-05	2.07E-05	4.29E-05	1.03E-04
**36h**	1.65E-05	1.09E-05	2.79E-05	1.70E-04
**48h**	1.44E-05	9.78E-06	6.81E-05	6.70E-05
**72h**	2.07E-05	1.36E-05	7.47E-05	7.92E-05
	cgd7_5160			
**2h**	2.12E-04	2.52E-05	2.05E-04	2.67E-04
**6h**	2.71E-04	9.85E-05	3.38E-04	8.83E-04
**12h**	8.99E-06	2.44E-07	1.77E-04	4.00E-05
**24h**	8.15E-05	2.20E-05	2.38E-04	1.58E-04
**36h**	7.28E-05	2.34E-05	5.68E-05	1.55E-04
**48h**	1.11E-04	4.51E-05	2.76E-04	1.29E-04
**72h**	2.92E-04	5.54E-05	1.04E-03	5.46E-04
	cgd7_5320			
**2h**	2.07E-06	1.96E-07	9.32E-07	1.27E-06
**6h**	4.50E-06	4.68E-08	2.69E-07	3.51E-07
**12h**	2.53E-06	1.26E-07	1.26E-06	2.39E-07
**24h**	8.67E-07	2.59E-07	4.70E-07	3.26E-07
**36h**	4.73E-07	5.52E-08	2.72E-07	1.59E-07
**48h**	7.23E-07	8.21E-08	2.12E-07	5.16E-07
**72h**	1.11E-06	3.45E-08	1.63E-07	1.99E-07
	cgd7_540			
**2h**	0.00E+00	0.00E+00	0.00E+00	0.00E+00

**6h**	0.00E+00	0.00E+00	0.00E+00	0.00E+00
**12h**	0.00E+00	0.00E+00	0.00E+00	1.83E-07
**24h**	1.15E-06	8.10E-08	3.29E-07	9.90E-07
**36h**	1.13E-06	3.70E-07	9.43E-07	8.97E-06
**48h**	4.36E-06	5.60E-07	4.60E-06	9.56E-06
**72h**	1.36E-05	3.96E-07	1.33E-06	1.65E-06
	cgd7_5400			
**2h**	0.00E+00	0.00E+00	0.00E+00	0.00E+00
**6h**	0.00E+00	0.00E+00	0.00E+00	0.00E+00
**12h**	0.00E+00	4.32E-06	2.23E-06	3.28E-06
**24h**	3.90E-06	1.46E-07	1.60E-06	9.30E-07
**36h**	2.09E-06	4.92E-07	5.27E-07	4.46E-06
**48h**	2.98E-05	5.66E-07	6.91E-06	1.54E-05
**72h**	7.06E-06	2.41E-06	1.21E-05	3.13E-05
	cgd7_550			
**2h**	0.00E+00	0.00E+00	0.00E+00	0.00E+00
**6h**	0.00E+00	0.00E+00	0.00E+00	0.00E+00
**12h**	3.19E-04	1.19E-03	3.26E-04	2.76E-03
**24h**	1.57E-04	1.26E-03	4.13E-05	6.39E-04
**36h**	1.70E-04	5.12E-04	3.71E-04	2.52E-03
**48h**	3.40E-05	1.62E-04	1.14E-04	1.87E-04
**72h**	2.35E-06	2.05E-05	1.23E-05	9.38E-05
	cgd7_570			
**2h**	3.27E-06	4.82E-06	5.34E-07	1.53E-05
**6h**	4.25E-07	4.14E-06	4.10E-07	7.01E-06
**12h**	1.16E-06	1.08E-06	9.88E-07	3.56E-06
**24h**	3.62E-06	4.28E-06	1.45E-06	3.99E-06
**36h**	7.47E-07	7.24E-07	3.31E-07	2.25E-06
**48h**	8.63E-07	7.77E-07	1.43E-06	2.60E-06
**72h**	3.57E-07	4.47E-07	1.15E-06	2.37E-06
	cgd7_590			
**2h**	0.00E+00	0.00E+00	0.00E+00	0.00E+00
**6h**	0.00E+00	0.00E+00	0.00E+00	0.00E+00
**12h**	1.72E-07	3.54E-07	7.49E-08	1.01E-07
**24h**	2.76E-08	1.37E-07	3.06E-08	2.72E-07
**36h**	9.69E-08	3.03E-07	2.65E-07	5.94E-07
**48h**	1.57E-07	1.95E-07	2.23E-07	2.71E-07
**72h**	3.69E-08	6.40E-08	1.10E-07	1.34E-07
	cgd7_600			
**2h**	0.00E+00	0.00E+00	0.00E+00	0.00E+00
**6h**	0.00E+00	0.00E+00	0.00E+00	0.00E+00
**12h**	4.50E-08	2.76E-07	1.21E-07	9.45E-07
**24h**	6.89E-08	2.34E-07	7.66E-08	2.68E-07
**36h**	7.02E-08	2.87E-07	3.10E-07	1.48E-06
**48h**	6.78E-08	1.11E-07	1.01E-07	5.95E-07
**72h**	1.75E-08	1.19E-07	9.25E-08	1.94E-07
	cgd7_610			
**2h**	1.05E-07	5.08E-07	4.16E-08	1.11E-06
**6h**	1.97E-08	2.08E-07	2.77E-08	4.11E-07
**12h**	7.24E-08	1.03E-07	1.20E-07	2.42E-07
**24h**	1.05E-07	1.42E-07	6.74E-08	2.64E-07
**36h**	7.67E-08	1.28E-07	1.62E-07	2.01E-07
**48h**	1.04E-07	9.33E-08	6.82E-08	9.98E-08

**72h**	2.18E-08	2.22E-08	5.39E-08	1.19E-07
	cgd7_800			
**2h**	1.12E-08	0.00E+00	0.00E+00	1.06E-07
**6h**	7.19E-09	2.45E-08	0.00E+00	7.39E-08
**12h**	1.79E-07	1.14E-06	5.14E-08	1.97E-07
**24h**	4.94E-08	6.59E-08	1.01E-08	8.04E-08
**36h**	1.47E-08	1.15E-07	6.21E-08	1.96E-07
**48h**	5.53E-08	2.93E-08	7.36E-08	1.79E-07
**72h**	2.04E-08	3.22E-08	1.95E-07	7.85E-08
	cgd7_810			
**2h**	0.00E+00	0.00E+00	0.00E+00	0.00E+00
**6h**	1.07E-07	7.00E-07	1.33E-07	1.46E-06
**12h**	8.57E-08	0.00E+00	1.99E-08	0.00E+00
**24h**	3.62E-08	9.86E-08	1.24E-07	5.91E-07
**36h**	1.93E-07	7.43E-07	1.13E-07	8.11E-07
**48h**	2.03E-07	3.59E-07	3.08E-07	1.26E-06
**72h**	2.79E-07	2.47E-07	1.75E-06	9.72E-07
	cgd7_850			
**2h**	9.14E-09	2.09E-08	9.33E-09	0.00E+00
**6h**	0.00E+00	1.51E-08	0.00E+00	0.00E+00
**12h**	0.00E+00	0.00E+00	3.73E-09	3.02E-08
**24h**	2.15E-08	6.16E-09	4.20E-09	6.89E-08
**36h**	1.15E-08	4.77E-08	1.37E-08	4.52E-08
**48h**	3.30E-06	3.34E-06	9.74E-06	6.96E-06
**72h**	9.68E-06	1.64E-05	1.71E-05	1.14E-05
	cgd7_870			
**2h**	2.95E-08	6.51E-08	0.00E+00	3.62E-07
**6h**	1.44E-07	4.58E-07	1.67E-07	1.25E-06
**12h**	2.85E-08	5.07E-07	2.74E-07	6.41E-07
**24h**	2.99E-07	8.62E-07	5.25E-07	2.99E-06
**36h**	4.35E-07	2.34E-06	6.69E-07	3.35E-06
**48h**	9.45E-07	1.99E-06	8.94E-07	2.95E-06
**72h**	1.45E-07	4.04E-07	3.43E-07	5.57E-07
	cgd7_890			
**2h**	8.77E-07	4.10E-06	0.00E+00	1.52E-06
**6h**	1.07E-06	4.10E-06	2.94E-06	8.88E-06
**12h**	3.51E-08	2.18E-07	2.95E-07	1.44E-06
**24h**	4.36E-07	1.23E-06	8.45E-07	5.77E-06
**36h**	5.05E-07	1.16E-06	7.94E-07	1.79E-06
**48h**	4.80E-07	7.73E-07	1.05E-06	1.96E-06
**72h**	3.16E-07	2.62E-07	4.85E-07	2.95E-07
	cgd7_990			
**2h**	0.00E+00	4.62E-05	1.02E-05	3.38E-06
**6h**	2.46E-05	3.14E-05	9.89E-05	1.83E-05
**12h**	5.73E-06	7.57E-05	1.61E-04	2.33E-05
**24h**	2.16E-05	5.09E-05	1.46E-04	6.17E-05
**36h**	1.24E-04	3.74E-05	1.28E-04	3.06E-05
**48h**	3.27E-04	7.86E-05	5.52E-04	7.17E-05
**72h**	6.38E-04	2.14E-04	6.30E-04	1.76E-04
	cgd8_1070			
**2h**	0.00E+00	3.93E-07	0.00E+00	2.82E-07
**6h**	1.51E-08	4.59E-07	3.28E-07	4.07E-07
**12h**	3.26E-08	9.88E-08	4.19E-08	8.54E-08

**24h**	6.32E-08	2.02E-07	6.78E-07	7.84E-07
**36h**	7.94E-07	9.79E-07	5.98E-07	4.78E-07
**48h**	4.02E-07	5.00E-07	9.37E-07	8.85E-07
**72h**	6.31E-07	6.37E-07	2.88E-06	1.10E-06
	cgd8_1100			
**2h**	5.09E-04	2.79E-05	4.53E-06	9.82E-06
**6h**	7.17E-05	6.32E-05	2.93E-06	6.45E-06
**12h**	5.88E-05	3.64E-05	1.99E-05	4.77E-05
**24h**	7.52E-05	1.00E-04	1.01E-05	9.35E-06
**36h**	2.76E-05	1.36E-05	4.20E-06	4.68E-06
**48h**	1.72E-05	5.27E-06	4.53E-06	4.83E-06
**72h**	1.15E-05	4.91E-06	2.73E-06	1.53E-06
	cgd8_1110			
**2h**	0.00E+00	0.00E+00	0.00E+00	0.00E+00
**6h**	1.43E-06	1.32E-06	5.60E-07	4.17E-08
**12h**	8.63E-06	3.38E-07	3.78E-07	5.11E-07
**24h**	9.15E-06	1.66E-05	2.46E-06	4.22E-06
**36h**	1.03E-04	2.65E-05	3.46E-06	6.12E-06
**48h**	9.91E-05	1.69E-05	5.43E-06	4.85E-06
**72h**	2.71E-05	1.55E-05	3.26E-06	1.60E-06
	cgd8_1130			
**2h**	5.67E-06	0.00E+00	0.00E+00	0.00E+00
**6h**	0.00E+00	3.30E-06	0.00E+00	0.00E+00
**12h**	9.86E-05	2.19E-04	5.29E-06	5.44E-06
**24h**	1.37E-05	1.44E-05	2.83E-08	2.66E-07
**36h**	5.44E-06	1.14E-06	2.87E-07	4.01E-06
**48h**	5.16E-06	1.04E-06	2.06E-07	2.29E-07
**72h**	5.14E-06	2.15E-06	1.14E-06	7.13E-07
	cgd8_1140			
**2h**	0.00E+00	0.00E+00	0.00E+00	0.00E+00
**6h**	0.00E+00	3.34E-06	0.00E+00	1.93E-06
**12h**	3.05E-05	1.10E-04	1.37E-04	4.36E-04
**24h**	2.35E-06	3.37E-05	1.98E-05	5.59E-05
**36h**	4.88E-06	1.69E-05	4.20E-05	8.59E-05
**48h**	6.54E-06	5.18E-06	1.43E-05	4.31E-05
**72h**	1.06E-05	2.42E-05	2.53E-05	1.82E-04
	cgd8_1190			
**2h**	0.00E+00	1.26E-06	0.00E+00	0.00E+00
**6h**	0.00E+00	0.00E+00	2.69E-09	0.00E+00
**12h**	1.44E-08	3.73E-08	2.34E-09	1.07E-09
**24h**	7.80E-09	8.19E-09	3.29E-09	1.91E-10
**36h**	3.56E-09	1.16E-09	4.43E-09	5.25E-10
**48h**	5.70E-08	3.19E-09	1.41E-09	9.89E-10
**72h**	2.56E-08	6.70E-09	8.72E-08	4.78E-09
	cgd8_1210			
**2h**	6.48E-05	2.96E-05	2.54E-07	8.94E-07
**6h**	2.32E-05	2.82E-05	9.09E-07	3.23E-06
**12h**	5.65E-06	1.11E-06	2.67E-06	2.23E-06
**24h**	3.42E-05	1.35E-05	2.42E-06	3.04E-06
**36h**	1.23E-05	1.26E-05	1.25E-06	9.94E-07
**48h**	6.73E-05	1.12E-05	3.39E-06	7.02E-06
**72h**	4.20E-05	9.47E-06	5.39E-06	2.76E-06
	cgd8_1250			

**2h**	0.00E+00	0.00E+00	0.00E+00	0.00E+00
**6h**	9.56E-06	1.64E-05	4.90E-06	3.02E-06
**12h**	4.73E-06	9.69E-04	1.65E-06	2.96E-07
**24h**	3.94E-06	4.36E-06	1.09E-05	3.17E-06
**36h**	1.49E-05	1.01E-05	3.96E-06	4.35E-06
**48h**	1.32E-05	8.76E-06	4.19E-06	3.63E-06
**72h**	1.03E-05	2.76E-06	3.43E-06	1.06E-06
	cgd8_130			
**2h**	3.65E-05	1.11E-03	2.74E-05	3.42E-04
**6h**	6.74E-05	4.52E-04	5.91E-04	2.68E-03
**12h**	1.25E-05	4.78E-04	5.74E-05	2.20E-04
**24h**	1.93E-05	5.27E-04	3.08E-04	3.06E-04
**36h**	2.44E-05	2.22E-04	6.73E-05	2.17E-04
**48h**	2.57E-05	2.13E-04	1.03E-04	2.88E-04
**72h**	6.60E-05	2.70E-04	7.73E-04	2.97E-05
	cgd8_1300			
**2h**	3.41E-07	1.61E-07	8.03E-09	1.28E-07
**6h**	0.00E+00	8.69E-09	1.31E-08	2.13E-07
**12h**	3.43E-08	9.43E-08	3.39E-08	1.10E-08
**24h**	5.40E-07	2.10E-07	1.85E-07	1.34E-08
**36h**	5.63E-06	1.16E-06	1.84E-07	1.43E-07
**48h**	2.28E-06	1.38E-06	7.06E-07	2.51E-07
**72h**	5.77E-06	1.10E-06	4.57E-07	1.80E-07
	cgd8_1310			
**2h**	2.00E-06	2.57E-07	0.00E+00	8.10E-09
**6h**	7.06E-06	2.98E-07	1.04E-08	1.90E-08
**12h**	1.50E-06	1.24E-07	2.89E-08	2.42E-08
**24h**	1.67E-07	1.20E-07	4.23E-08	6.86E-08
**36h**	1.02E-06	3.66E-07	6.86E-08	2.97E-08
**48h**	2.60E-06	2.57E-07	3.34E-08	6.14E-08
**72h**	2.35E-06	1.44E-07	6.69E-08	6.71E-08
	cgd8_1340			
**2h**	1.30E-05	2.08E-04	1.72E-05	6.03E-05
**6h**	1.17E-05	3.00E-04	2.73E-03	8.43E-04
**12h**	2.25E-06	1.09E-04	7.63E-04	4.24E-05
**24h**	4.06E-06	2.32E-04	2.60E-03	3.04E-04
**36h**	4.26E-06	1.47E-04	5.09E-04	1.61E-04
**48h**	3.40E-06	4.38E-05	1.76E-04	3.18E-04
**72h**	1.52E-06	1.10E-04	5.91E-05	2.61E-04
	cgd8_1390			
**2h**	1.02E-05	4.94E-07	2.80E-08	2.12E-07
**6h**	0.00E+00	1.62E-08	5.48E-08	6.34E-08
**12h**	9.04E-07	0.00E+00	9.13E-09	4.65E-08
**24h**	1.88E-06	8.93E-08	6.92E-08	2.09E-08
**36h**	6.38E-06	8.03E-07	9.47E-08	2.10E-07
**48h**	1.23E-04	2.90E-06	1.31E-06	1.70E-06
**72h**	9.15E-05	2.35E-06	4.45E-06	9.92E-07
	cgd8_140			
**2h**	8.46E-09	6.11E-09	1.08E-08	4.89E-08
**6h**	6.61E-07	1.94E-06	8.07E-07	6.50E-07
**12h**	6.44E-08	3.35E-08	1.64E-07	1.42E-07
**24h**	5.04E-07	7.97E-07	2.32E-06	1.73E-06
**36h**	2.27E-06	1.19E-06	1.14E-06	1.95E-06

**48h**	2.06E-06	1.51E-06	3.68E-06	2.93E-06
**72h**	7.29E-07	3.14E-07	6.85E-07	7.02E-07
	cgd8_1410			
**2h**	3.07E-06	0.00E+00	3.33E-05	0.00E+00
**6h**	2.47E-05	2.16E-05	1.96E-05	1.11E-05
**12h**	7.85E-06	4.75E-05	1.99E-05	2.57E-06
**24h**	9.64E-06	1.05E-04	1.96E-05	6.11E-05
**36h**	1.31E-05	4.53E-05	3.54E-05	4.49E-05
**48h**	4.06E-05	2.54E-05	5.06E-05	1.23E-04
**72h**	8.21E-05	6.29E-05	7.16E-05	3.85E-04
	cgd8_1420			
**2h**	1.25E-06	0.00E+00	0.00E+00	1.50E-08
**6h**	2.48E-05	3.34E-06	1.34E-05	1.92E-06
**12h**	6.21E-07	1.84E-08	9.49E-07	4.32E-08
**24h**	9.15E-06	1.89E-06	2.43E-05	2.78E-06
**36h**	6.62E-06	2.66E-06	5.22E-06	4.18E-07
**48h**	1.13E-05	2.26E-06	2.08E-05	1.19E-06
**72h**	5.42E-05	3.29E-06	2.76E-05	1.47E-06
	cgd8_1460			
**2h**	3.82E-06	1.15E-06	9.58E-07	1.20E-06
**6h**	1.71E-07	4.35E-07	4.06E-07	1.58E-07
**12h**	4.58E-07	5.70E-08	1.29E-06	7.06E-07
**24h**	1.95E-06	9.54E-07	2.37E-06	4.54E-07
**36h**	5.28E-07	7.22E-07	7.02E-07	8.00E-07
**48h**	1.20E-06	2.47E-07	1.18E-06	3.59E-07
**72h**	9.33E-07	9.78E-07	8.72E-07	6.11E-07
	cgd8_1470			
**2h**	8.38E-04	4.73E-04	3.57E-04	4.13E-05
**6h**	3.21E-04	4.14E-04	3.40E-04	2.53E-05
**12h**	4.90E-05	1.95E-05	7.84E-05	5.33E-06
**24h**	1.72E-04	1.61E-04	1.59E-04	4.37E-05
**36h**	1.22E-04	1.18E-04	2.03E-04	2.45E-05
**48h**	1.22E-04	1.19E-04	3.53E-04	1.56E-05
**72h**	3.67E-04	1.99E-04	4.58E-04	2.89E-05
	cgd8_1480			
**2h**	5.89E-05	5.84E-06	2.02E-05	6.02E-06
**6h**	1.04E-03	9.78E-05	1.73E-04	1.24E-05
**12h**	1.18E-03	5.29E-05	4.12E-04	1.00E-05
**24h**	4.66E-04	4.60E-05	3.03E-04	1.69E-05
**36h**	3.57E-04	7.01E-05	4.08E-04	1.79E-05
**48h**	2.05E-04	5.39E-05	3.15E-04	1.58E-05
**72h**	6.38E-05	1.72E-05	5.74E-05	2.35E-06
	cgd8_1490			
**2h**	8.26E-08	5.13E-08	7.11E-08	7.46E-08
**6h**	7.18E-08	6.28E-08	1.12E-07	2.10E-08
**12h**	1.74E-07	2.59E-07	1.79E-07	2.51E-08
**24h**	8.69E-08	8.68E-08	1.95E-07	2.03E-08
**36h**	1.20E-07	6.87E-08	1.57E-07	4.45E-08
**48h**	1.84E-07	1.16E-07	1.86E-07	4.49E-08
**72h**	3.24E-07	2.17E-07	3.40E-07	8.53E-08
	cgd8_1550			
**2h**	2.89E-06	2.86E-06	1.41E-07	1.08E-06
**6h**	3.65E-07	1.16E-07	1.17E-07	3.82E-07

**12h**	1.10E-06	2.26E-07	8.53E-07	3.62E-07
**24h**	1.21E-06	1.49E-06	2.49E-07	6.99E-07
**36h**	5.43E-07	5.83E-07	3.73E-07	5.61E-07
**48h**	1.14E-06	1.27E-06	1.27E-06	3.56E-07
**72h**	3.09E-07	2.35E-07	6.55E-07	2.52E-07
	cgd8_1640			
**2h**	2.52E-08	1.96E-08	0.00E+00	7.92E-09
**6h**	1.05E-08	8.92E-08	2.45E-07	1.59E-08
**12h**	1.57E-06	3.55E-07	8.35E-07	1.38E-07
**24h**	1.35E-06	9.23E-07	2.47E-06	7.08E-07
**36h**	4.08E-06	1.85E-06	4.85E-06	1.16E-06
**48h**	4.82E-06	1.98E-06	8.70E-06	5.21E-07
**72h**	6.35E-06	1.41E-06	8.79E-06	4.02E-07
	cgd8_1810			
**2h**	5.06E-09	9.25E-08	5.45E-09	2.26E-08
**6h**	0.00E+00	5.20E-09	2.45E-09	3.70E-09
**12h**	1.93E-08	3.43E-08	4.41E-08	5.65E-08
**24h**	4.63E-08	2.02E-07	2.06E-08	3.17E-08
**36h**	7.41E-08	3.96E-08	5.38E-08	1.69E-08
**48h**	5.56E-08	4.69E-08	9.00E-08	2.89E-08
**72h**	3.43E-08	2.02E-08	4.50E-08	1.40E-08
	cgd8_1820			
**2h**	3.33E-05	1.14E-05	9.98E-06	8.65E-06
**6h**	1.97E-05	8.80E-05	3.60E-05	2.20E-05
**12h**	2.32E-06	1.93E-06	1.15E-05	5.30E-06
**24h**	8.62E-06	2.97E-05	1.81E-05	1.76E-05
**36h**	1.16E-05	1.13E-05	1.73E-05	6.31E-06
**48h**	9.11E-06	6.48E-06	8.45E-06	1.60E-05
**72h**	2.60E-06	8.33E-07	3.54E-06	5.03E-07
	cgd8_1920			
**2h**	9.98E-05	1.23E-03	1.47E-04	2.29E-05
**6h**	3.71E-04	6.77E-04	6.05E-04	7.34E-04
**12h**	8.51E-05	1.54E-04	1.58E-04	7.95E-05
**24h**	2.70E-04	8.46E-04	2.28E-04	5.82E-04
**36h**	1.02E-04	1.13E-04	3.55E-04	3.40E-04
**48h**	1.04E-04	1.35E-04	1.60E-04	2.41E-04
**72h**	9.22E-05	2.50E-04	1.08E-04	9.89E-05
	cgd8_1950			
**2h**	1.47E-06	1.59E-06	3.17E-08	2.16E-06
**6h**	3.15E-07	1.78E-06	2.24E-07	1.69E-06
**12h**	1.26E-07	7.74E-08	4.19E-07	3.06E-07
**24h**	5.31E-07	6.08E-07	4.96E-07	5.80E-07
**36h**	6.87E-07	5.57E-07	5.87E-07	2.74E-07
**48h**	7.86E-07	3.05E-07	1.15E-07	1.10E-07
**72h**	7.06E-07	2.12E-07	5.69E-07	1.21E-06
	cgd8_2230			
**2h**	0.00E+00	0.00E+00	0.00E+00	0.00E+00
**6h**	0.00E+00	0.00E+00	0.00E+00	0.00E+00
**12h**	2.72E-09	1.26E-07	1.05E-07	1.19E-07
**24h**	7.72E-08	6.37E-08	1.56E-07	6.87E-08
**36h**	6.36E-07	3.02E-07	1.64E-07	3.57E-07
**48h**	2.11E-06	6.80E-07	1.02E-06	1.21E-06
**72h**	1.99E-06	1.98E-06	1.52E-06	2.44E-06

	cgd8_2240			
**2h**	0.00E+00	0.00E+00	0.00E+00	0.00E+00
**6h**	0.00E+00	0.00E+00	0.00E+00	0.00E+00
**12h**	4.76E-08	2.82E-08	4.11E-08	3.61E-08
**24h**	1.93E-08	3.36E-08	4.77E-08	4.42E-08
**36h**	1.87E-07	6.06E-08	5.36E-08	8.05E-08
**48h**	4.93E-07	1.71E-07	5.74E-07	3.73E-07
**72h**	5.73E-07	3.92E-07	3.99E-07	5.13E-07
	cgd8_2280			
**2h**	7.75E-04	4.38E-04	5.68E-04	1.61E-03
**6h**	5.44E-05	2.70E-04	1.04E-05	4.71E-04
**12h**	9.39E-04	9.51E-04	1.29E-03	2.67E-04
**24h**	9.55E-04	4.87E-04	5.75E-04	2.39E-04
**36h**	4.36E-04	9.60E-04	6.57E-04	2.04E-04
**48h**	8.09E-04	2.16E-04	3.55E-04	3.15E-04
**72h**	1.04E-03	8.36E-04	9.47E-04	1.69E-04
	cgd8_2290			
**2h**	0.00E+00	4.76E-08	0.00E+00	0.00E+00
**6h**	0.00E+00	0.00E+00	0.00E+00	0.00E+00
**12h**	5.43E-07	1.21E-06	7.09E-07	1.94E-07
**24h**	3.67E-07	1.47E-07	4.65E-07	3.59E-07
**36h**	5.23E-07	3.46E-07	5.60E-07	1.56E-06
**48h**	5.80E-07	2.53E-07	3.53E-07	1.52E-06
**72h**	2.09E-06	1.44E-06	2.24E-06	6.03E-06
	cgd8_2310			
**2h**	6.90E-05	2.44E-04	0.00E+00	1.39E-05
**6h**	5.75E-05	7.21E-04	4.54E-05	1.48E-05
**12h**	2.17E-05	3.50E-05	1.79E-05	7.22E-06
**24h**	2.05E-05	8.23E-05	1.42E-05	2.39E-05
**36h**	4.61E-05	2.18E-03	2.88E-05	3.27E-05
**48h**	2.85E-04	7.50E-03	1.19E-04	1.93E-05
**72h**	8.09E-06	1.75E-03	9.50E-05	1.65E-04
	cgd8_2350			
**2h**	6.30E-04	9.52E-03	1.16E-03	9.90E-04
**6h**	1.25E-03	2.39E-02	1.93E-03	1.50E-04
**12h**	1.06E-04	1.66E-03	7.78E-04	5.76E-05
**24h**	3.42E-04	1.34E-03	7.40E-04	6.14E-04
**36h**	3.36E-04	2.59E-03	5.72E-04	4.03E-05
**48h**	4.39E-04	1.17E-03	3.45E-04	1.93E-04
**72h**	1.25E-03	7.51E-04	5.05E-04	4.68E-04
	cgd8_2400			
**2h**	0.00E+00	0.00E+00	0.00E+00	0.00E+00
**6h**	0.00E+00	2.91E-09	0.00E+00	0.00E+00
**12h**	1.77E-06	5.25E-06	1.65E-06	1.87E-06
**24h**	5.25E-07	7.02E-07	1.68E-07	1.39E-07
**36h**	5.23E-07	2.80E-07	1.18E-06	1.65E-06
**48h**	4.23E-07	2.19E-07	1.56E-07	8.46E-07
**72h**	5.77E-07	2.41E-07	1.94E-07	5.76E-07
	cgd8_250			
**2h**	5.69E-06	2.60E-06	9.07E-07	7.74E-06
**6h**	2.87E-06	2.09E-05	1.80E-05	2.89E-05
**12h**	5.21E-07	8.36E-07	2.60E-06	1.67E-06
**24h**	4.70E-06	5.64E-06	4.08E-05	1.26E-05

**36h**	9.99E-06	9.17E-06	4.26E-06	5.60E-06
**48h**	5.90E-06	3.99E-06	4.19E-06	6.20E-06
**72h**	2.49E-06	2.62E-06	1.37E-06	1.48E-06
	cgd8_2570			
**2h**	0.00E+00	4.09E-03	6.79E-05	0.00E+00
**6h**	2.69E-05	9.96E-03	2.17E-04	1.04E-05
**12h**	8.93E-06	0.00E+00	3.62E-05	1.07E-05
**24h**	1.86E-05	8.35E-04	1.99E-04	4.62E-05
**36h**	5.63E-05	8.58E-04	1.68E-04	5.47E-05
**48h**	1.77E-04	1.87E-03	3.57E-05	1.85E-05
**72h**	1.71E-04	2.39E-03	5.30E-05	3.25E-05
	cgd8_2590			
**2h**	0.00E+00	0.00E+00	0.00E+00	0.00E+00
**6h**	2.08E-06	0.00E+00	0.00E+00	0.00E+00
**12h**	1.81E-06	7.04E-07	3.43E-07	5.54E-07
**24h**	2.39E-06	5.03E-04	5.76E-07	7.02E-07
**36h**	3.67E-06	2.06E-03	2.62E-06	2.80E-06
**48h**	5.50E-06	4.42E-03	2.76E-06	6.72E-07
**72h**	2.12E-06	1.91E-03	2.33E-06	3.62E-06
	cgd8_2600			
**2h**	0.00E+00	0.00E+00	1.26E-07	2.56E-07
**6h**	6.30E-08	6.35E-06	6.51E-07	2.92E-06
**12h**	3.20E-07	8.73E-07	3.60E-07	5.35E-05
**24h**	1.99E-07	1.84E-07	2.36E-06	1.27E-06
**36h**	1.89E-06	2.04E-06	5.94E-06	3.52E-06
**48h**	2.45E-06	2.09E-06	3.30E-06	7.03E-06
**72h**	2.38E-07	2.35E-07	7.01E-07	1.28E-06
	cgd8_2610			
**2h**	0.00E+00	0.00E+00	0.00E+00	0.00E+00
**6h**	0.00E+00	0.00E+00	0.00E+00	0.00E+00
**12h**	3.06E-05	3.15E-06	2.72E-06	0.00E+00
**24h**	9.56E-06	1.15E-06	1.89E-06	0.00E+00
**36h**	5.69E-05	3.75E-06	7.17E-06	0.00E+00
**48h**	2.77E-05	6.97E-06	6.12E-06	0.00E+00
**72h**	2.82E-05	3.63E-06	1.58E-06	0.00E+00
	cgd8_2680			
**2h**	8.20E-07	5.89E-09	0.00E+00	2.21E-08
**6h**	1.98E-08	2.42E-08	0.00E+00	0.00E+00
**12h**	6.22E-08	5.05E-08	1.12E-07	6.46E-08
**24h**	9.05E-07	1.67E-07	4.51E-08	3.39E-08
**36h**	2.55E-07	2.33E-07	1.02E-07	5.22E-08
**48h**	7.82E-06	2.23E-06	1.49E-06	1.15E-06
**72h**	4.25E-07	1.77E-07	4.15E-08	6.92E-08
	cgd8_2690			
**2h**	1.30E-05	6.88E-06	1.97E-07	9.56E-05
**6h**	4.50E-06	2.47E-07	6.08E-06	1.15E-07
**12h**	2.51E-06	3.27E-05	1.18E-04	1.86E-05
**24h**	7.04E-06	1.05E-04	2.86E-04	7.24E-05
**36h**	1.75E-06	1.43E-04	8.97E-05	6.42E-05
**48h**	3.11E-06	4.68E-05	7.03E-06	1.27E-04
**72h**	9.72E-06	1.02E-04	8.59E-05	1.55E-05
	cgd8_2740			
**2h**	0.00E+00	0.00E+00	0.00E+00	0.00E+00

**6h**	0.00E+00	0.00E+00	4.29E-09	0.00E+00
**12h**	4.47E-06	4.65E-06	4.61E-06	1.02E-06
**24h**	2.41E-06	5.24E-07	7.60E-07	3.13E-07
**36h**	3.77E-06	1.48E-06	4.14E-06	3.12E-06
**48h**	2.62E-06	2.69E-07	4.16E-07	3.05E-07
**72h**	7.69E-06	3.79E-06	3.35E-06	1.91E-06
	cgd8_2900			
**2h**	1.45E-05	0.00E+00	2.71E-08	0.00E+00
**6h**	0.00E+00	1.25E-07	1.12E-07	8.14E-08
**12h**	3.65E-06	3.05E-07	1.18E-07	1.17E-07
**24h**	3.27E-06	2.04E-07	5.57E-07	1.27E-07
**36h**	4.83E-06	7.51E-07	1.14E-06	6.14E-07
**48h**	1.81E-05	7.24E-07	7.96E-07	5.84E-07
**72h**	6.78E-06	6.21E-07	3.38E-07	1.29E-06
	cgd8_2920			
**2h**	5.61E-05	2.46E-07	3.33E-07	0.00E+00
**6h**	1.38E-04	1.32E-04	9.15E-05	6.74E-05
**12h**	2.45E-05	6.36E-05	6.16E-05	2.21E-05
**24h**	3.68E-05	5.19E-05	3.29E-04	1.31E-04
**36h**	5.24E-05	1.08E-04	2.26E-04	1.06E-04
**48h**	4.99E-05	1.44E-04	2.04E-04	1.31E-04
**72h**	3.84E-05	4.09E-05	5.24E-05	1.15E-05
	cgd8_300			
**2h**	3.21E-05	4.82E-06	3.98E-06	2.94E-03
**6h**	2.73E-05	2.10E-06	3.01E-06	5.34E-06
**12h**	2.35E-05	1.56E-05	1.48E-05	9.58E-06
**24h**	3.11E-05	1.36E-05	9.42E-06	6.97E-06
**36h**	9.10E-06	4.01E-06	5.66E-06	5.30E-06
**48h**	8.46E-06	2.12E-06	2.30E-06	2.74E-06
**72h**	5.26E-06	1.08E-06	1.52E-06	5.55E-07
	cgd8_3010			
**2h**	0.00E+00	2.41E-09	0.00E+00	0.00E+00
**6h**	1.33E-06	1.23E-06	4.35E-07	5.25E-07
**12h**	2.04E-07	1.51E-07	5.01E-07	1.15E-07
**24h**	8.80E-07	7.08E-07	9.73E-07	6.07E-07
**36h**	9.85E-07	7.05E-07	1.05E-06	7.55E-07
**48h**	2.16E-06	9.85E-07	6.21E-07	6.21E-07
**72h**	2.21E-07	9.97E-08	9.99E-08	5.38E-08
	cgd8_320			
**2h**	3.54E-06	1.06E-07	0.00E+00	1.76E-08
**6h**	2.45E-07	9.02E-09	4.33E-08	1.62E-08
**12h**	1.30E-04	9.59E-05	4.77E-05	9.91E-06
**24h**	1.43E-05	1.59E-05	5.02E-06	3.70E-06
**36h**	1.05E-05	2.54E-06	1.17E-05	6.19E-06
**48h**	8.90E-06	1.02E-06	1.16E-06	1.19E-06
**72h**	3.64E-06	5.08E-07	5.27E-07	3.95E-07
	cgd8_330			
**2h**	2.11E-05	8.68E-08	2.82E-08	6.78E-08
**6h**	2.12E-05	7.81E-08	0.00E+00	5.03E-08
**12h**	2.41E-04	2.73E-05	1.96E-05	3.25E-06
**24h**	6.43E-05	7.71E-06	3.96E-06	1.89E-06
**36h**	8.50E-05	7.09E-06	1.61E-05	5.67E-06
**48h**	1.55E-04	2.93E-06	5.33E-06	2.30E-06

**72h**	1.43E-04	3.84E-06	3.17E-06	1.74E-06
	cgd8_3340			
**2h**	6.33E-06	2.34E-04	7.83E-04	4.26E-05
**6h**	8.46E-06	4.57E-05	2.35E-04	4.46E-04
**12h**	7.71E-06	6.41E-05	1.18E-04	1.94E-04
**24h**	6.41E-06	1.23E-04	1.76E-04	4.61E-04
**36h**	3.75E-06	3.30E-05	1.28E-04	4.28E-05
**48h**	2.70E-06	4.10E-05	2.93E-05	3.84E-05
**72h**	2.05E-06	4.55E-05	7.36E-05	8.71E-05
	cgd8_3400			
**2h**	3.17E-04	8.76E-06	1.04E-05	4.61E-06
**6h**	2.88E-04	8.65E-06	4.98E-06	2.33E-06
**12h**	2.10E-04	9.39E-06	5.14E-05	9.97E-06
**24h**	5.11E-04	9.01E-05	2.42E-05	9.47E-06
**36h**	6.61E-05	2.62E-05	2.42E-05	1.00E-04
**48h**	1.67E-04	1.35E-05	2.06E-05	4.76E-05
**72h**	1.09E-04	8.67E-06	4.87E-06	3.06E-05
	cgd8_3550			
**2h**	4.80E-04	6.12E-05	1.02E-04	7.75E-05
**6h**	2.25E-04	1.92E-05	8.98E-06	4.12E-05
**12h**	6.18E-04	4.78E-05	2.41E-04	1.46E-04
**24h**	7.22E-04	6.09E-05	4.14E-05	2.33E-05
**36h**	1.35E-04	2.81E-05	9.13E-05	4.23E-05
**48h**	2.85E-04	8.87E-06	4.31E-05	1.96E-05
**72h**	7.24E-05	8.24E-06	1.47E-05	5.12E-06
	cgd8_3570			
**2h**	1.44E-07	1.62E-06	2.75E-08	1.70E-06
**6h**	1.75E-07	2.58E-07	8.26E-08	1.20E-06
**12h**	5.38E-06	1.84E-05	2.09E-05	2.93E-05
**24h**	1.73E-06	2.02E-05	3.03E-06	4.22E-06
**36h**	3.98E-07	1.82E-06	6.60E-06	1.22E-05
**48h**	9.03E-07	9.38E-07	9.71E-07	1.86E-06
**72h**	3.43E-07	7.63E-07	5.44E-07	1.51E-06
	cgd8_3630			
**2h**	1.41E-06	2.59E-06	2.25E-06	5.96E-06
**6h**	2.76E-07	5.20E-07	1.17E-07	9.60E-07
**12h**	1.38E-07	4.04E-07	3.52E-07	1.59E-06
**24h**	4.63E-07	2.18E-06	9.95E-07	1.13E-06
**36h**	4.46E-07	1.42E-06	1.49E-06	2.99E-06
**48h**	1.32E-06	9.84E-07	1.70E-06	1.57E-06
**72h**	3.92E-07	4.38E-07	1.06E-06	1.20E-06
	cgd8_370			
**2h**	0.00E+00	0.00E+00	0.00E+00	2.25E-06
**6h**	1.12E-05	1.27E-05	2.22E-05	6.18E-06
**12h**	1.04E-05	2.06E-05	1.62E-04	2.61E-05
**24h**	4.12E-05	6.28E-05	1.12E-04	6.29E-05
**36h**	1.58E-04	9.86E-05	1.04E-04	2.61E-05
**48h**	1.62E-04	6.73E-05	1.38E-04	3.60E-05
**72h**	1.90E-04	6.53E-05	1.70E-04	1.13E-05
	cgd8_3700			
**2h**	6.67E-07	2.20E-06	6.45E-07	3.16E-06
**6h**	1.54E-08	2.36E-06	1.73E-06	3.06E-06
**12h**	1.64E-07	1.01E-06	2.00E-06	2.53E-06

**24h**	9.16E-07	2.44E-06	5.46E-06	1.47E-06
**36h**	1.34E-07	1.29E-06	1.04E-06	2.04E-06
**48h**	5.05E-07	4.31E-07	2.90E-07	8.01E-07
**72h**	1.84E-07	2.04E-07	2.17E-07	5.56E-07
	cgd8_3810			
**2h**	4.14E-05	2.33E-04	1.03E-04	8.80E-04
**6h**	6.83E-06	8.00E-05	2.67E-05	2.19E-04
**12h**	9.13E-06	3.02E-05	7.24E-05	1.72E-04
**24h**	2.53E-05	1.19E-04	4.22E-05	1.23E-04
**36h**	8.07E-06	5.34E-05	3.56E-05	9.90E-05
**48h**	1.15E-05	2.28E-05	3.52E-05	1.45E-04
**72h**	6.80E-06	1.18E-05	3.29E-05	2.85E-05
	cgd8_390			
**2h**	8.31E-07	3.76E-06	2.43E-06	1.30E-05
**6h**	6.70E-07	2.08E-06	1.51E-06	5.96E-06
**12h**	6.93E-07	3.67E-07	5.57E-06	6.33E-06
**24h**	9.92E-07	5.68E-06	1.11E-06	3.52E-06
**36h**	1.12E-06	3.82E-06	1.72E-06	6.32E-06
**48h**	3.78E-06	3.86E-06	6.58E-06	1.29E-05
**72h**	4.30E-06	3.91E-06	5.02E-06	4.07E-06
	cgd8_3990			
**2h**	0.00E+00	1.49E-06	0.00E+00	0.00E+00
**6h**	0.00E+00	0.00E+00	0.00E+00	0.00E+00
**12h**	6.97E-06	2.59E-04	7.15E-05	6.25E-05
**24h**	1.11E-06	1.62E-05	3.94E-06	1.91E-05
**36h**	3.72E-06	3.56E-05	5.03E-05	1.28E-04
**48h**	2.04E-06	1.58E-05	1.79E-05	1.86E-05
**72h**	4.08E-06	2.95E-05	3.39E-05	1.43E-04
	cgd8_400			
**2h**	2.48E-06	5.49E-06	1.68E-06	8.79E-06
**6h**	7.60E-06	1.25E-05	1.15E-05	1.79E-05
**12h**	4.42E-07	2.13E-06	3.96E-06	4.83E-06
**24h**	2.13E-06	8.38E-05	9.90E-06	1.56E-05
**36h**	2.02E-06	3.01E-06	3.00E-06	2.65E-06
**48h**	1.85E-06	2.73E-06	2.23E-06	6.18E-06
**72h**	6.01E-07	1.18E-06	2.64E-06	1.54E-06
	cgd8_4060			
**2h**	1.91E-09	2.29E-09	0.00E+00	9.24E-09
**6h**	3.51E-07	8.36E-07	1.11E-06	1.52E-06
**12h**	8.54E-08	1.86E-07	1.01E-07	6.40E-08
**24h**	5.84E-08	2.21E-07	3.16E-07	4.00E-07
**36h**	2.82E-07	4.07E-07	2.21E-07	1.89E-07
**48h**	2.27E-07	2.81E-07	2.60E-07	3.25E-07
**72h**	2.08E-07	1.54E-07	4.74E-07	1.82E-07
	cgd8_4250			
**2h**	0.00E+00	0.00E+00	0.00E+00	4.60E-08
**6h**	0.00E+00	1.92E-08	1.23E-08	4.41E-09
**12h**	1.29E-08	0.00E+00	2.08E-09	3.17E-09
**24h**	2.50E-08	6.08E-08	1.59E-07	3.24E-07
**36h**	1.94E-07	3.23E-07	3.42E-07	3.60E-07
**48h**	5.11E-07	6.75E-07	8.58E-07	3.42E-06
**72h**	3.08E-07	3.85E-07	7.26E-07	7.13E-07
	cgd8_4310			

**2h**	0.00E+00	0.00E+00	0.00E+00	0.00E+00
**6h**	0.00E+00	1.07E-06	2.59E-08	0.00E+00
**12h**	0.00E+00	0.00E+00	2.65E-08	3.65E-08
**24h**	1.04E-06	2.09E-05	9.04E-07	1.73E-06
**36h**	4.92E-06	5.10E-05	8.37E-06	1.35E-05
**48h**	1.39E-04	2.01E-04	2.67E-04	3.58E-04
**72h**	1.23E-04	4.31E-04	2.45E-04	4.15E-04
	cgd8_4350			
**2h**	6.36E-05	9.58E-05	4.61E-05	3.89E-05
**6h**	1.31E-04	1.58E-04	1.87E-04	1.66E-04
**12h**	9.32E-06	1.47E-05	4.63E-05	1.93E-05
**24h**	8.10E-05	3.22E-05	8.88E-05	5.37E-05
**36h**	5.20E-05	3.40E-05	3.20E-05	1.88E-05
**48h**	3.28E-05	1.90E-05	1.16E-05	9.14E-06
**72h**	6.68E-06	4.44E-06	6.48E-06	8.46E-06
	cgd8_440			
**2h**	4.50E-05	7.86E-05	1.61E-05	6.39E-05
**6h**	7.86E-05	1.38E-04	1.28E-04	1.30E-04
**12h**	8.65E-06	1.98E-05	3.18E-05	2.35E-05
**24h**	5.34E-05	5.25E-05	5.27E-05	4.72E-05
**36h**	4.17E-05	4.80E-05	1.19E-05	2.20E-05
**48h**	1.88E-05	1.12E-05	1.29E-05	7.36E-06
**72h**	7.69E-06	1.05E-05	8.00E-06	1.19E-05
	cgd8_4510			
**2h**	6.42E-03	6.14E-03	1.38E-04	1.47E-03
**6h**	1.76E-03	3.66E-03	3.48E-04	4.30E-03
**12h**	2.71E-04	4.73E-04	1.93E-04	2.62E-04
**24h**	7.81E-04	1.43E-03	2.89E-04	7.20E-04
**36h**	7.47E-04	5.73E-04	1.10E-04	1.09E-03
**48h**	2.88E-04	3.31E-04	4.08E-05	6.94E-04
**72h**	1.91E-04	1.28E-04	1.96E-05	3.47E-04
	cgd8_4560			
**2h**	2.01E-04	2.82E-06	0.00E+00	9.11E-05
**6h**	1.10E-03	4.62E-04	7.57E-05	3.99E-04
**12h**	2.08E-04	3.35E-04	7.45E-05	9.77E-05
**24h**	1.05E-04	1.23E-04	8.14E-05	2.03E-04
**36h**	1.28E-04	3.72E-04	7.46E-05	2.44E-04
**48h**	3.51E-04	5.72E-05	2.23E-05	4.89E-05
**72h**	9.59E-05	7.32E-05	1.15E-05	4.34E-05
	cgd8_4580			
**2h**	0.00E+00	0.00E+00	0.00E+00	0.00E+00
**6h**	0.00E+00	0.00E+00	0.00E+00	0.00E+00
**12h**	4.94E-05	5.30E-05	1.92E-04	4.86E-06
**24h**	2.84E-05	8.91E-06	7.23E-05	1.95E-06
**36h**	3.79E-05	3.05E-05	7.34E-05	1.38E-05
**48h**	2.27E-05	7.89E-06	7.61E-05	1.52E-06
**72h**	2.59E-05	2.14E-06	7.08E-05	7.38E-06
	cgd8_4600			
**2h**	5.76E-09	7.63E-09	7.40E-09	1.92E-08
**6h**	2.16E-09	4.77E-08	3.01E-08	1.23E-07
**12h**	3.02E-08	7.22E-08	4.38E-08	3.71E-08
**24h**	7.10E-08	5.75E-08	1.39E-07	7.93E-08
**36h**	2.85E-07	1.33E-07	3.95E-07	2.76E-07

**48h**	1.37E-07	7.58E-08	1.09E-07	3.54E-07
**72h**	2.01E-07	1.27E-07	1.26E-07	1.87E-07
	cgd8_4610			
**2h**	2.07E-08	0.00E+00	3.42E-08	1.15E-07
**6h**	1.66E-08	0.00E+00	0.00E+00	0.00E+00
**12h**	7.63E-08	0.00E+00	0.00E+00	1.61E-07
**24h**	1.16E-08	2.84E-09	3.87E-08	1.56E-08
**36h**	1.79E-07	1.78E-07	1.35E-07	1.54E-07
**48h**	4.83E-07	2.26E-07	8.44E-08	8.25E-07
**72h**	1.25E-06	5.39E-07	7.60E-07	1.12E-06
	cgd8_4670			
**2h**	1.51E-04	1.11E-04	1.40E-03	4.24E-05
**6h**	5.34E-05	2.40E-05	5.66E-04	3.75E-05
**12h**	7.74E-06	6.24E-06	4.79E-05	5.83E-06
**24h**	4.39E-05	2.40E-05	1.21E-04	8.09E-06
**36h**	1.01E-05	4.05E-06	7.77E-05	1.30E-05
**48h**	1.37E-05	1.58E-05	5.44E-05	5.27E-06
**72h**	8.68E-06	5.80E-06	6.96E-05	3.81E-06
	cgd8_4680			
**2h**	2.43E-06	2.93E-06	1.93E-06	7.42E-06
**6h**	2.88E-07	6.24E-07	1.16E-07	5.17E-07
**12h**	8.66E-07	1.20E-06	1.60E-06	1.68E-06
**24h**	2.45E-06	1.55E-06	1.46E-06	1.89E-06
**36h**	1.24E-06	6.48E-07	7.76E-07	1.32E-06
**48h**	6.50E-07	4.31E-07	7.26E-07	1.57E-06
**72h**	7.78E-07	3.77E-07	3.58E-07	5.42E-07
	cgd8_4690			
**2h**	0.00E+00	0.00E+00	0.00E+00	0.00E+00
**6h**	0.00E+00	0.00E+00	0.00E+00	0.00E+00
**12h**	1.06E-07	3.22E-07	2.90E-07	2.17E-07
**24h**	1.40E-07	1.46E-07	9.51E-08	7.22E-08
**36h**	1.74E-07	1.15E-07	3.84E-07	4.29E-07
**48h**	6.32E-07	3.68E-07	9.23E-07	1.33E-06
**72h**	1.46E-06	6.33E-07	9.60E-07	1.40E-06
	cgd8_4700			
**2h**	1.46E-05	4.70E-05	6.73E-07	0.00E+00
**6h**	4.22E-06	1.95E-05	0.00E+00	3.18E-05
**12h**	0.00E+00	0.00E+00	3.79E-07	1.07E-06
**24h**	7.20E-07	1.65E-06	1.29E-08	2.19E-06
**36h**	1.63E-05	3.72E-05	5.04E-06	3.64E-05
**48h**	8.14E-05	3.72E-05	1.04E-06	5.09E-06
**72h**	3.87E-06	2.11E-05	1.26E-05	6.85E-06
	cgd8_4720			
**2h**	1.57E-05	1.26E-05	5.66E-06	5.16E-06
**6h**	9.54E-05	1.49E-04	7.38E-05	5.56E-05
**12h**	3.07E-06	7.50E-06	6.29E-06	2.45E-06
**24h**	2.25E-05	4.04E-05	5.84E-05	4.37E-05
**36h**	3.76E-05	2.16E-05	9.80E-06	1.17E-05
**48h**	1.99E-05	1.43E-05	1.90E-05	9.99E-06
**72h**	7.70E-06	5.86E-06	8.83E-06	7.30E-07
	cgd8_4750			
**2h**	1.55E-03	1.06E-04	3.97E-05	1.02E-04
**6h**	1.75E-04	2.47E-05	3.51E-05	3.76E-05

**12h**	5.24E-05	2.24E-05	6.77E-05	1.56E-05
**24h**	3.15E-04	1.89E-04	2.65E-04	7.46E-05
**36h**	1.10E-04	3.99E-05	3.29E-05	1.63E-05
**48h**	9.41E-05	3.91E-05	9.50E-05	3.78E-05
**72h**	5.37E-05	1.41E-05	1.27E-05	5.98E-06
	cgd8_500			
**2h**	0.00E+00	0.00E+00	8.72E-09	0.00E+00
**6h**	0.00E+00	0.00E+00	1.71E-09	0.00E+00
**12h**	5.52E-06	2.92E-05	3.30E-06	1.37E-06
**24h**	7.93E-07	1.54E-06	6.65E-07	3.28E-07
**36h**	1.43E-06	1.05E-06	1.48E-06	1.11E-06
**48h**	7.73E-07	6.32E-07	5.06E-07	3.91E-07
**72h**	3.40E-07	4.41E-07	4.23E-07	2.57E-07
	cgd8_520			
**2h**	0.00E+00	0.00E+00	0.00E+00	0.00E+00
**6h**	0.00E+00	5.48E-08	0.00E+00	0.00E+00
**12h**	4.10E-04	6.88E-04	9.50E-05	5.22E-05
**24h**	1.02E-04	4.76E-05	1.36E-05	5.46E-06
**36h**	3.38E-05	2.60E-05	9.34E-05	3.07E-05
**48h**	3.75E-05	1.09E-05	1.86E-05	1.12E-05
**72h**	2.04E-05	9.01E-06	2.31E-05	2.48E-06
	cgd8_540			
**2h**	0.00E+00	0.00E+00	0.00E+00	0.00E+00
**6h**	0.00E+00	0.00E+00	0.00E+00	0.00E+00
**12h**	2.66E-03	1.25E-03	7.13E-04	1.34E-04
**24h**	2.41E-04	5.02E-04	2.86E-04	1.04E-04
**36h**	1.84E-04	3.83E-04	7.74E-04	3.78E-04
**48h**	1.73E-04	2.68E-04	3.65E-04	2.57E-04
**72h**	1.87E-03	3.88E-04	2.13E-03	2.10E-04
	cgd8_550			
**2h**	2.49E-06	2.28E-06	2.77E-08	6.54E-07
**6h**	2.20E-05	1.02E-05	2.60E-05	1.12E-05
**12h**	1.07E-06	1.57E-06	5.02E-06	1.74E-06
**24h**	3.89E-06	2.56E-06	1.54E-05	4.63E-06
**36h**	6.06E-06	1.03E-05	4.58E-06	3.50E-06
**48h**	4.24E-06	1.90E-06	3.29E-06	2.91E-06
**72h**	3.53E-06	1.60E-06	2.83E-06	3.28E-07
	cgd8_560			
**2h**	2.74E-07	1.39E-07	2.03E-07	1.53E-07
**6h**	5.14E-07	5.94E-07	2.85E-07	1.24E-07
**12h**	8.39E-08	1.19E-07	8.27E-08	4.46E-08
**24h**	2.39E-07	3.42E-07	1.76E-07	1.64E-07
**36h**	4.11E-07	2.88E-07	2.96E-07	2.82E-07
**48h**	4.51E-07	4.48E-07	4.25E-07	3.77E-07
**72h**	4.39E-07	4.14E-07	8.96E-07	4.40E-07
	cgd8_580			
**2h**	7.81E-07	7.62E-07	1.04E-06	4.98E-07
**6h**	3.08E-06	3.10E-06	3.82E-06	1.58E-06
**12h**	4.85E-07	7.62E-07	1.04E-06	5.04E-07
**24h**	1.03E-06	1.67E-06	2.25E-06	1.66E-06
**36h**	3.80E-06	1.39E-06	2.88E-06	1.56E-06
**48h**	3.04E-06	2.93E-06	2.61E-06	1.90E-06
**72h**	1.02E-06	5.11E-07	1.75E-06	8.56E-07

	cgd8_650			
**2h**	9.27E-06	1.20E-05	3.82E-06	5.22E-06
**6h**	3.83E-05	2.72E-05	2.84E-05	2.55E-05
**12h**	2.83E-06	1.64E-06	1.61E-05	2.55E-06
**24h**	1.09E-05	1.05E-05	1.54E-05	8.15E-06
**36h**	1.05E-05	5.84E-06	7.43E-06	1.86E-06
**48h**	2.06E-06	3.01E-06	2.75E-06	1.11E-06
**72h**	7.81E-07	7.13E-07	7.81E-07	2.16E-07
	cgd8_670			
**2h**	0.00E+00	0.00E+00	0.00E+00	0.00E+00
**6h**	0.00E+00	0.00E+00	0.00E+00	0.00E+00
**12h**	9.82E-05	3.15E-05	5.43E-05	1.24E-05
**24h**	6.36E-05	2.07E-05	1.48E-05	4.33E-06
**36h**	7.87E-05	2.02E-05	4.59E-05	2.14E-05
**48h**	3.38E-05	9.36E-06	4.11E-05	2.42E-05
**72h**	1.14E-05	2.59E-06	6.93E-06	2.57E-06
	cgd8_680			
**2h**	0.00E+00	0.00E+00	0.00E+00	0.00E+00
**6h**	0.00E+00	7.76E-10	0.00E+00	0.00E+00
**12h**	2.12E-04	1.05E-04	8.61E-05	4.41E-05
**24h**	2.56E-05	4.77E-05	1.31E-05	1.94E-05
**36h**	1.13E-04	3.95E-05	9.91E-05	7.84E-05
**48h**	7.39E-05	2.35E-05	2.39E-05	1.03E-04
**72h**	1.77E-05	5.39E-06	1.08E-05	5.34E-06
	cgd8_700			
**2h**	2.63E-09	0.00E+00	0.00E+00	0.00E+00
**6h**	0.00E+00	0.00E+00	0.00E+00	0.00E+00
**12h**	5.34E-07	7.95E-07	5.09E-07	6.02E-08
**24h**	1.73E-07	7.20E-07	1.11E-07	1.60E-08
**36h**	5.42E-07	5.66E-07	3.84E-07	5.97E-07
**48h**	1.26E-06	4.25E-07	4.79E-07	2.39E-07
**72h**	2.27E-06	8.91E-07	6.69E-06	2.07E-06
	cgd4_1230			
**2h**	7.29E-03	1.81E-05	5.41E-06	1.08E-05
**6h**	2.08E-04	3.52E-04	1.09E-04	8.67E-05
**12h**	2.51E-06	8.51E-06	4.56E-05	1.52E-05
**24h**	2.30E-05	2.40E-05	1.58E-04	4.51E-05
**36h**	2.08E-05	1.13E-05	1.08E-05	1.97E-05
**48h**	8.90E-06	1.35E-05	1.24E-05	8.53E-06
**72h**	3.16E-06	9.32E-07	2.52E-06	1.82E-06
	cgd4_140			
**2h**	3.35E-03	4.74E-07	8.67E-08	1.12E-06
**6h**	1.10E-07	2.38E-07	1.02E-07	2.56E-07
**12h**	9.70E-09	3.21E-08	3.13E-08	8.21E-08
**24h**	1.16E-07	1.18E-07	3.19E-07	3.22E-07
**36h**	1.08E-07	2.15E-07	4.15E-08	2.17E-07
**48h**	1.36E-07	9.39E-08	5.75E-08	1.50E-07
**72h**	6.72E-08	4.69E-08	2.92E-08	5.16E-08
	cgd4_1590			
**2h**	4.81E-03	5.38E-04	1.51E-05	1.50E-04
**6h**	2.08E-04	3.92E-04	7.69E-05	8.78E-05
**12h**	1.68E-05	1.36E-04	4.95E-05	2.83E-05
**24h**	2.17E-04	2.17E-04	1.10E-04	8.51E-05

**36h**	7.58E-05	3.60E-04	7.11E-05	5.08E-05
**48h**	1.27E-04	1.21E-04	3.19E-05	4.40E-05
**72h**	7.34E-05	1.33E-04	1.01E-05	5.45E-06
	cgd4_1650			
**2h**	0.00E+00	0.00E+00	0.00E+00	0.00E+00
**6h**	1.71E-06	2.05E-06	1.34E-06	7.86E-07
**12h**	1.85E-07	1.67E-06	2.71E-07	2.67E-07
**24h**	7.11E-07	2.41E-07	1.34E-06	5.66E-07
**36h**	1.43E-06	1.07E-06	1.05E-06	1.03E-06
**48h**	7.63E-07	9.06E-07	8.84E-07	3.43E-07
**72h**	1.50E-07	2.21E-07	2.82E-07	1.25E-07
	cgd4_1700			
**2h**	7.72E-04	8.14E-04	2.71E-05	1.18E-04
**6h**	5.30E-05	9.67E-05	1.25E-05	1.74E-05
**12h**	2.91E-05	2.58E-04	5.01E-05	5.69E-05
**24h**	2.24E-04	1.72E-04	6.86E-05	3.11E-05
**36h**	7.18E-05	8.19E-05	3.57E-05	2.79E-05
**48h**	5.09E-05	4.30E-05	5.84E-05	2.73E-05
**72h**	3.37E-05	2.31E-05	5.29E-05	9.33E-06
	cgd4_1830			
**2h**	1.97E-07	4.20E-07	1.00E-07	2.03E-07
**6h**	9.00E-08	1.23E-07	1.06E-07	1.15E-07
**12h**	1.70E-07	1.27E-06	3.91E-07	3.59E-07
**24h**	8.36E-07	4.62E-07	3.21E-07	2.03E-07
**36h**	8.17E-07	8.75E-07	7.95E-07	9.07E-07
**48h**	6.66E-07	6.40E-07	3.81E-07	3.87E-07
**72h**	3.03E-07	3.28E-07	3.02E-07	1.07E-07
	cgd4_1940			
**2h**	6.67E-05	9.98E-02	7.10E-04	2.72E-03
**6h**	1.28E-04	7.47E-01	1.47E-01	3.73E-02
**12h**	4.81E-06	1.76E-01	2.08E-02	1.23E-03
**24h**	6.91E-05	2.42E-02	2.63E-02	6.78E-03
**36h**	3.13E-05	8.09E-02	2.03E-02	1.55E-03
**48h**	1.06E-04	6.59E-02	2.21E-02	2.13E-05
**72h**	3.54E-05	3.25E-02	2.10E-02	3.65E-03
	cgd4_2020			
**2h**	6.47E-06	2.82E-06	6.80E-07	3.09E-06
**6h**	3.37E-07	8.23E-07	2.25E-07	6.41E-07
**12h**	1.46E-07	3.16E-07	5.81E-07	5.08E-07
**24h**	2.19E-06	1.15E-06	5.42E-07	9.85E-07
**36h**	3.98E-07	9.11E-07	5.32E-07	1.11E-06
**48h**	5.63E-07	5.71E-07	7.71E-07	1.23E-06
**72h**	3.58E-07	6.13E-07	4.44E-07	2.21E-07
	cgd4_2040			
**2h**	1.80E-06	4.83E-07	8.29E-07	6.31E-07
**6h**	1.34E-06	1.56E-06	5.91E-07	1.09E-06
**12h**	3.05E-08	1.17E-07	5.04E-07	2.87E-07
**24h**	5.74E-07	4.78E-07	7.02E-07	1.37E-06
**36h**	3.13E-07	7.87E-07	4.24E-07	6.42E-07
**48h**	3.70E-07	2.97E-07	4.92E-07	5.04E-07
**72h**	1.62E-07	1.45E-07	1.25E-07	9.08E-08
	cgd4_2110			
**2h**	0.00E+00	0.00E+00	0.00E+00	0.00E+00

**6h**	1.85E-09	2.47E-09	3.77E-09	1.19E-08
**12h**	1.69E-05	1.16E-04	9.14E-05	2.47E-05
**24h**	6.56E-06	3.79E-06	5.49E-06	3.37E-06
**36h**	1.56E-06	4.14E-06	1.92E-05	1.63E-05
**48h**	6.46E-06	4.95E-06	9.58E-06	5.40E-06
**72h**	5.33E-06	8.96E-06	4.43E-06	2.13E-06
	cgd4_2260			
**2h**	2.42E-04	1.41E-03	1.66E-04	7.76E-05
**6h**	3.01E-04	6.42E-04	6.06E-04	3.78E-04
**12h**	4.23E-05	3.07E-05	2.08E-04	7.46E-05
**24h**	1.11E-04	7.51E-05	3.02E-04	1.66E-04
**36h**	9.80E-05	1.61E-04	1.93E-04	1.28E-04
**48h**	4.18E-05	5.62E-05	8.78E-05	3.01E-05
**72h**	1.82E-05	2.24E-05	2.98E-05	1.74E-05
	cgd4_2270			
**2h**	3.21E-05	9.30E-06	1.00E-06	1.48E-06
**6h**	5.00E-05	4.37E-06	7.65E-06	1.95E-04
**12h**	2.48E-07	2.70E-07	2.15E-06	6.66E-06
**24h**	4.69E-06	1.10E-06	3.61E-06	5.91E-06
**36h**	1.15E-06	1.57E-06	2.07E-06	5.69E-06
**48h**	1.22E-06	1.71E-06	1.54E-06	2.82E-06
**72h**	2.04E-07	2.11E-07	2.27E-07	3.58E-07
	cgd4_2300			
**2h**	0.00E+00	5.91E-09	0.00E+00	1.04E-08
**6h**	2.89E-06	6.23E-06	1.35E-05	4.08E-06
**12h**	3.07E-06	1.24E-06	1.85E-06	1.46E-06
**24h**	1.19E-06	1.74E-06	6.45E-06	4.48E-06
**36h**	2.90E-06	4.14E-06	6.10E-06	3.06E-06
**48h**	2.82E-06	4.89E-06	2.53E-06	2.13E-06
**72h**	7.72E-07	1.26E-06	6.94E-07	1.32E-06
	cgd4_2350			
**2h**	0.00E+00	0.00E+00	0.00E+00	0.00E+00
**6h**	2.25E-08	2.41E-08	3.83E-07	3.92E-07
**12h**	6.10E-05	4.61E-04	8.90E-04	4.44E-04
**24h**	1.77E-05	4.75E-05	5.88E-05	6.20E-05
**36h**	2.06E-06	2.54E-05	9.86E-05	1.57E-04
**48h**	1.50E-06	1.08E-05	1.20E-05	5.01E-05
**72h**	6.41E-07	5.03E-06	7.30E-06	2.32E-05
	cgd4_2370			
**2h**	1.82E-06	3.70E-06	3.08E-07	4.94E-07
**6h**	1.80E-05	2.24E-05	3.10E-05	5.08E-05
**12h**	4.51E-07	9.40E-07	2.23E-06	1.49E-06
**24h**	2.69E-06	2.23E-06	2.05E-05	1.67E-05
**36h**	2.86E-06	3.49E-06	2.07E-06	2.92E-06
**48h**	1.00E-06	1.27E-06	1.79E-06	1.51E-06
**72h**	5.21E-07	1.35E-07	3.42E-07	3.85E-07
	cgd4_2400			
**2h**	8.87E-04	1.88E-03	8.68E-04	1.05E-04
**6h**	3.41E-03	1.90E-03	2.11E-03	2.27E-03
**12h**	6.37E-04	5.53E-04	1.63E-03	8.53E-05
**24h**	9.61E-04	2.41E-04	9.90E-04	2.49E-04
**36h**	1.46E-03	3.34E-04	3.82E-04	1.96E-05
**48h**	9.51E-04	6.42E-04	1.43E-04	9.81E-05

**72h**	6.09E-04	2.13E-04	2.16E-04	9.36E-06
	cgd4_2460			
**2h**	1.49E-06	5.52E-06	9.56E-06	7.08E-05
**6h**	7.32E-07	4.94E-06	5.05E-06	5.94E-05
**12h**	9.74E-06	3.36E-05	3.00E-05	1.63E-04
**24h**	3.08E-06	2.95E-06	5.84E-06	5.10E-05
**36h**	1.13E-06	3.74E-06	4.72E-06	8.20E-05
**48h**	1.29E-06	4.39E-06	5.06E-06	7.87E-05
**72h**	1.74E-06	1.42E-06	9.36E-07	3.79E-05
	cgd4_2530			
**2h**	4.43E-07	2.78E-07	5.91E-07	5.93E-07
**6h**	6.05E-07	7.92E-07	7.23E-07	3.02E-06
**12h**	7.01E-07	6.02E-07	4.54E-07	6.20E-07
**24h**	2.65E-07	1.54E-07	5.28E-07	1.17E-06
**36h**	2.01E-07	1.28E-07	1.92E-07	6.46E-07
**48h**	1.40E-07	1.98E-07	3.01E-07	1.38E-06
**72h**	6.25E-08	8.62E-08	8.49E-08	3.89E-07
	cgd4_2540			
**2h**	2.97E-07	4.87E-08	2.64E-07	1.96E-07
**6h**	1.40E-06	1.05E-06	2.04E-06	7.72E-06
**12h**	3.41E-07	4.86E-07	4.01E-07	7.24E-07
**24h**	8.35E-07	4.37E-07	3.27E-06	3.25E-06
**36h**	1.48E-06	9.97E-07	1.57E-06	8.47E-06
**48h**	2.75E-06	1.43E-06	1.12E-06	4.79E-06
**72h**	2.98E-07	5.49E-07	5.31E-07	9.05E-07
	cgd4_2680			
**2h**	5.71E-05	3.56E-03	4.73E-04	2.25E-05
**6h**	1.21E-04	3.31E-03	1.17E-03	1.82E-04
**12h**	1.50E-05	1.01E-05	5.33E-04	4.13E-06
**24h**	1.46E-04	2.77E-04	3.80E-04	2.73E-05
**36h**	7.24E-05	1.03E-03	3.31E-04	2.77E-06
**48h**	8.06E-05	5.03E-04	1.82E-04	2.69E-05
**72h**	3.84E-05	3.57E-04	5.45E-04	1.58E-06
	cgd4_2910			
**2h**	9.59E-08	1.77E-07	0.00E+00	1.96E-06
**6h**	8.20E-09	5.23E-08	9.33E-08	1.66E-06
**12h**	7.55E-07	6.45E-07	4.33E-07	3.09E-06
**24h**	2.43E-07	6.32E-08	1.44E-07	2.35E-06
**36h**	5.91E-07	7.86E-07	9.24E-07	3.88E-05
**48h**	2.75E-07	8.90E-08	1.27E-07	4.45E-06
**72h**	1.13E-07	2.15E-07	1.36E-07	2.47E-06
	cgd4_2950			
**2h**	1.55E-07	3.00E-07	1.22E-07	1.47E-06
**6h**	5.50E-07	1.69E-07	1.42E-07	2.53E-05
**12h**	7.46E-06	4.06E-06	1.84E-06	7.49E-06
**24h**	1.78E-06	8.80E-07	4.25E-07	3.83E-07
**36h**	3.57E-07	2.15E-07	8.69E-07	5.73E-07
**48h**	4.63E-07	2.09E-07	2.17E-07	6.14E-07
**72h**	8.17E-08	4.92E-08	4.54E-08	7.05E-06
	cgd4_2990			
**2h**	1.55E-06	2.76E-07	1.09E-07	1.60E-07
**6h**	6.35E-07	1.90E-07	1.73E-07	1.00E-06
**12h**	2.98E-06	1.04E-06	7.96E-07	1.25E-06

**24h**	7.06E-06	8.92E-07	4.98E-07	7.41E-07
**36h**	1.47E-06	2.96E-07	4.28E-07	4.19E-07
**48h**	3.28E-06	2.71E-07	5.30E-07	7.81E-07
**72h**	6.80E-07	2.53E-07	2.02E-07	9.62E-08
	cgd4_3000			
**2h**	5.68E-05	6.07E-06	3.91E-06	1.75E-05
**6h**	6.84E-06	1.31E-05	9.66E-06	2.03E-05
**12h**	2.66E-06	8.92E-07	1.08E-05	5.14E-06
**24h**	2.08E-05	3.92E-06	1.06E-05	5.56E-06
**36h**	3.67E-06	1.76E-06	1.58E-06	1.80E-06
**48h**	1.43E-05	1.14E-06	1.39E-06	2.22E-06
**72h**	2.90E-06	4.21E-07	3.07E-07	3.04E-07
	cgd4_3020			
**2h**	2.73E-06	3.05E-06	3.83E-06	7.00E-06
**6h**	1.88E-06	2.03E-06	2.39E-06	6.38E-06
**12h**	2.71E-06	8.20E-07	6.16E-06	3.81E-06
**24h**	2.25E-05	2.11E-06	4.56E-06	2.39E-06
**36h**	1.68E-06	1.36E-06	1.30E-06	1.90E-06
**48h**	2.59E-06	4.54E-07	8.93E-07	1.03E-06
**72h**	1.20E-06	1.83E-07	2.41E-07	4.35E-07
	cgd4_3080			
**2h**	8.26E-04	8.64E-04	2.32E-03	2.56E-03
**6h**	2.81E-03	4.35E-03	9.51E-03	1.69E-02
**12h**	3.64E-04	2.39E-04	3.62E-03	2.01E-03
**24h**	3.35E-03	6.93E-04	4.10E-03	5.29E-03
**36h**	7.26E-04	8.54E-04	6.92E-04	9.34E-04
**48h**	8.73E-04	5.01E-04	1.21E-03	1.38E-04
**72h**	1.88E-04	2.12E-04	1.94E-04	1.74E-05
	cgd4_3130			
**2h**	6.59E-06	7.65E-06	2.22E-05	4.99E-05
**6h**	6.96E-06	1.75E-05	2.61E-05	5.48E-05
**12h**	6.75E-06	5.27E-06	2.11E-05	1.82E-05
**24h**	3.86E-05	7.41E-06	2.37E-05	1.86E-05
**36h**	4.66E-06	7.45E-06	9.69E-06	1.40E-05
**48h**	6.20E-06	5.48E-06	9.10E-06	1.24E-05
**72h**	1.39E-06	2.80E-06	1.75E-06	3.11E-06
	cgd4_320			
**2h**	0.00E+00	0.00E+00	0.00E+00	1.08E-08
**6h**	4.72E-08	5.23E-08	1.51E-07	3.11E-08
**12h**	0.00E+00	3.68E-09	3.17E-09	1.22E-08
**24h**	5.35E-09	6.46E-09	2.32E-08	1.96E-08
**36h**	3.44E-08	1.45E-08	6.06E-08	1.32E-08
**48h**	7.57E-08	6.63E-08	9.84E-08	1.24E-07
**72h**	2.59E-08	1.64E-08	2.47E-08	1.63E-08
	cgd4_3260			
**2h**	1.27E-08	8.64E-08	8.27E-08	4.92E-07
**6h**	8.60E-09	1.04E-08	2.79E-09	6.05E-09
**12h**	3.73E-09	1.07E-09	2.87E-08	1.41E-08
**24h**	5.97E-09	9.16E-09	5.85E-08	1.73E-08
**36h**	6.25E-09	4.61E-08	5.23E-08	1.54E-07
**48h**	8.59E-08	2.75E-08	4.07E-08	6.18E-08
**72h**	1.94E-08	3.84E-08	7.78E-08	1.41E-07
	cgd4_3270			

**2h**	1.34E-04	3.30E-04	1.30E-04	1.33E-04
**6h**	3.95E-03	3.10E-05	1.18E-03	1.80E-03
**12h**	5.25E-04	2.06E-04	2.60E-04	2.76E-05
**24h**	2.83E-05	4.36E-04	2.30E-04	5.84E-04
**36h**	9.95E-04	5.65E-04	1.80E-04	4.50E-05
**48h**	6.13E-04	1.15E-04	2.01E-04	2.67E-05
**72h**	3.54E-04	6.72E-05	1.61E-05	1.53E-04
	cgd4_3340			
**2h**	4.89E-03	4.03E-03	0.00E+00	7.69E-04
**6h**	1.63E-03	1.69E-04	9.01E-04	3.59E-04
**12h**	2.56E-03	1.01E-03	2.50E-04	8.64E-04
**24h**	5.58E-03	5.93E-05	1.33E-03	1.22E-04
**36h**	4.49E-04	5.51E-04	9.74E-04	1.58E-04
**48h**	4.86E-04	2.07E-04	2.00E-04	7.10E-04
**72h**	2.31E-04	3.17E-04	1.78E-04	2.90E-05
	cgd4_3500			
**2h**	0.00E+00	0.00E+00	0.00E+00	0.00E+00
**6h**	1.18E-09	0.00E+00	0.00E+00	0.00E+00
**12h**	2.86E-08	0.00E+00	0.00E+00	0.00E+00
**24h**	0.00E+00	4.12E-09	9.97E-08	9.81E-10
**36h**	2.39E-08	6.07E-08	1.52E-05	1.66E-08
**48h**	4.76E-07	5.24E-07	2.11E-05	2.01E-07
**72h**	3.99E-08	9.74E-07	6.97E-07	7.58E-09
	cgd4_3530			
**2h**	0.00E+00	3.12E-08	4.74E-05	0.00E+00
**6h**	0.00E+00	2.40E-08	0.00E+00	5.20E-09
**12h**	7.26E-04	1.46E-03	1.06E-02	1.82E-03
**24h**	2.66E-04	1.05E-03	4.26E-03	2.72E-04
**36h**	4.33E-04	4.75E-04	6.55E-03	1.56E-03
**48h**	3.66E-04	2.33E-04	3.10E-03	8.74E-04
**72h**	4.89E-05	2.85E-05	4.27E-04	1.40E-05
	cgd4_3550			
**2h**	8.33E-05	3.76E-05	7.88E-05	1.56E-04
**6h**	1.49E-05	1.43E-05	3.48E-05	1.47E-05
**12h**	1.57E-05	6.18E-06	4.73E-05	1.72E-05
**24h**	3.16E-05	3.59E-05	3.67E-05	3.22E-05
**36h**	1.49E-05	1.06E-05	1.07E-05	2.62E-05
**48h**	1.51E-05	8.69E-06	2.07E-05	1.38E-05
**72h**	1.23E-06	8.82E-07	1.42E-06	2.31E-06
	cgd4_3570			
**2h**	7.88E-07	1.55E-06	2.20E-06	7.98E-06
**6h**	4.44E-08	3.74E+00	6.41E-08	7.22E-08
**12h**	2.30E-06	3.42E-06	2.19E-05	5.82E-06
**24h**	1.15E-06	5.72E-06	3.60E-06	1.48E-06
**36h**	2.85E-06	3.55E-06	1.15E-05	9.22E-06
**48h**	1.53E-06	8.06E-07	3.87E-06	8.05E-07
**72h**	1.20E-06	1.06E-06	2.55E-06	4.57E-07
	cgd4_3620			
**2h**	2.97E-05	1.65E-07	3.73E-07	4.75E-07
**6h**	0.00E+00	3.06E-08	8.23E-09	7.16E-09
**12h**	2.17E-04	5.10E-04	2.85E-04	2.94E-04
**24h**	1.17E-04	1.29E-04	4.54E-05	5.30E-05
**36h**	2.32E-05	3.01E-05	6.87E-05	1.64E-04

**48h**	2.93E-05	1.96E-05	3.07E-05	1.65E-05
**72h**	8.46E-06	5.77E-06	6.52E-06	1.26E-05
	cgd4_3630			
**2h**	2.86E-02	2.58E-02	8.15E-05	1.34E-01
**6h**	5.58E-03	3.15E-03	1.36E-04	6.04E-02
**12h**	1.99E-02	9.70E-03	1.19E-03	1.19E-01
**24h**	6.73E-03	2.98E-03	8.47E-05	8.04E-02
**36h**	3.20E-03	4.12E-04	5.85E-05	1.20E-05
**48h**	4.81E-03	1.88E-03	3.99E-04	2.26E-03
**72h**	2.97E-03	2.69E-04	2.74E-05	2.50E-02
	cgd4_3640			
**2h**	1.88E-04	2.25E-04	2.33E-03	4.84E-05
**6h**	2.08E-05	3.46E-05	1.19E-03	2.84E-05
**12h**	6.23E-05	9.55E-05	1.65E-03	1.86E-05
**24h**	4.51E-05	4.04E-05	1.81E-04	1.16E-05
**36h**	2.05E-05	3.33E-05	1.81E-04	1.54E-05
**48h**	9.70E-06	7.27E-06	2.43E-04	1.08E-05
**72h**	8.59E-06	1.62E-05	5.15E-05	4.93E-06
	cgd4_3670			
**2h**	2.85E-03	2.38E-03	1.48E-02	1.40E-03
**6h**	7.76E-04	8.70E-04	9.40E-03	1.35E-03
**12h**	4.22E-04	9.04E-04	1.56E-02	6.48E-04
**24h**	0.00E+00	6.46E-04	2.48E-03	5.57E-04
**36h**	5.16E-04	4.95E-04	1.19E-03	3.52E-04
**48h**	3.98E-04	1.42E-04	9.16E-04	3.94E-04
**72h**	1.09E-04	1.12E-04	2.98E-04	3.47E-05
	cgd4_3720			
**2h**	0.00E+00	0.00E+00	0.00E+00	0.00E+00
**6h**	0.00E+00	0.00E+00	0.00E+00	0.00E+00
**12h**	0.00E+00	0.00E+00	0.00E+00	0.00E+00
**24h**	0.00E+00	1.86E-09	2.09E-08	8.53E-10
**36h**	2.13E-08	2.41E-08	1.50E-08	5.10E-09
**48h**	2.66E-06	5.69E-07	8.93E-06	1.53E-06
**72h**	1.17E-06	1.03E-06	7.95E-06	1.63E-06
	cgd4_3880			
**2h**	1.53E-07	2.59E-07	9.86E-08	1.55E-07
**6h**	2.59E-07	1.51E-07	2.97E-07	3.11E-07
**12h**	3.54E-08	1.58E-08	4.54E-08	1.18E-08
**24h**	1.11E-07	1.39E-07	2.11E-07	1.47E-07
**36h**	4.96E-08	8.12E-08	3.60E-08	1.52E-07
**48h**	5.50E-07	5.91E-08	1.00E-07	9.93E-08
**72h**	1.47E-08	4.53E-08	7.53E-08	4.67E-08
	cgd4_3910			
**2h**	8.92E-06	3.58E-06	3.05E-06	4.52E-06
**6h**	1.59E-06	1.84E-06	8.78E-07	1.89E-06
**12h**	3.80E-06	1.66E-06	3.60E-06	3.77E-06
**24h**	2.63E-06	2.74E-06	1.06E-06	3.75E-06
**36h**	4.47E-06	2.21E-06	2.17E-06	2.90E-06
**48h**	1.71E-06	9.12E-07	1.01E-06	1.54E-06
**72h**	7.94E-07	8.54E-07	3.45E-07	4.03E-07
	cgd4_4020			
**2h**	1.32E-03	1.82E-04	7.99E-06	2.92E-03
**6h**	4.43E-03	6.85E-05	1.45E-03	9.36E-04

**12h**	6.58E-04	9.62E-06	1.42E-04	4.01E-04
**24h**	2.13E-03	2.30E-05	2.57E-04	1.81E-04
**36h**	8.73E-04	4.89E-05	5.33E-04	1.69E-03
**48h**	3.73E-04	2.30E-05	1.07E-04	3.22E-04
**72h**	2.61E-04	6.26E-06	3.19E-05	5.24E-03
	cgd4_410			
**2h**	3.68E-03	4.71E-04	1.86E-05	2.19E-03
**6h**	6.82E-03	2.29E-05	2.82E-05	2.36E-05
**12h**	6.97E-04	1.29E-04	9.21E-05	5.22E-05
**24h**	8.98E-04	2.33E-05	2.54E-04	1.17E-04
**36h**	6.05E-04	1.81E-05	2.28E-05	5.33E-03
**48h**	4.60E-04	7.30E-06	4.39E-05	1.02E-04
**72h**	1.94E-04	2.40E-06	1.07E-04	2.54E-03
	cgd4_4210			
**2h**	1.25E-04	5.34E-04	1.32E-06	1.51E-04
**6h**	1.30E-05	1.13E-04	5.30E-05	3.75E-05
**12h**	6.65E-06	3.83E-05	3.34E-04	3.43E-05
**24h**	1.70E-05	6.70E-05	6.68E-05	2.20E-05
**36h**	8.98E-06	2.37E-07	2.82E-05	1.28E-05
**48h**	7.71E-06	1.33E-05	2.42E-07	5.77E-06
**72h**	7.54E-06	3.00E-05	1.47E-05	7.95E-06
	cgd4_4490			
**2h**	5.76E-08	0.00E+00	0.00E+00	1.48E-09
**6h**	1.20E-07	1.41E-07	3.05E-07	1.59E-07
**12h**	1.24E-08	1.12E-09	2.94E-08	2.83E-08
**24h**	6.52E-08	8.95E-08	3.89E-07	1.48E-07
**36h**	8.66E-08	1.31E-07	1.01E-07	9.64E-08
**48h**	1.62E-07	1.04E-06	5.48E-07	1.31E-07
**72h**	5.91E-08	7.44E-07	8.52E-08	5.05E-08
	cgd4_520			
**2h**	0.00E+00	0.00E+00	0.00E+00	0.00E+00
**6h**	1.56E-09	3.00E-09	1.77E-09	1.51E-09
**12h**	2.56E-07	5.31E-07	1.08E-06	3.09E-07
**24h**	2.17E-07	3.63E-07	5.71E-07	3.40E-07
**36h**	3.96E-07	8.06E-07	1.52E-06	7.93E-07
**48h**	4.90E-07	1.97E-06	3.45E-07	2.13E-07
**72h**	8.05E-08	5.01E-07	2.59E-07	4.16E-08
	cgd4_580			
**2h**	1.10E-05	2.91E-05	6.24E-05	2.20E-06
**6h**	9.35E-05	7.80E-04	1.58E-03	4.66E-04
**12h**	1.05E-05	1.29E-04	2.56E-04	7.21E-05
**24h**	2.41E-05	1.41E-04	1.92E-04	1.55E-04
**36h**	2.27E-05	1.93E-04	1.87E-04	8.74E-05
**48h**	7.15E-05	7.00E-05	6.69E-05	5.41E-05
**72h**	1.55E-05	9.66E-05	1.85E-04	3.76E-05
	cgd4_630			
**2h**	0.00E+00	0.00E+00	0.00E+00	0.00E+00
**6h**	3.15E-09	2.04E-08	8.10E-08	3.16E-09
**12h**	1.76E-08	3.95E-09	1.57E-08	4.66E-09
**24h**	1.40E-09	6.18E-09	1.60E-08	2.75E-09
**36h**	5.88E-09	6.32E-09	4.82E-08	7.38E-09
**48h**	1.69E-08	3.80E-08	3.84E-08	1.34E-08
**72h**	1.14E-08	1.60E-08	1.76E-08	1.05E-08

	cgd4_880			
**2h**	1.82E-06	2.50E-06	5.52E-06	1.26E-06
**6h**	8.30E-07	5.35E-07	3.21E-06	9.32E-07
**12h**	3.71E-07	8.04E-07	8.77E-06	8.60E-07
**24h**	1.17E-06	1.03E-06	4.79E-06	1.02E-06
**36h**	5.86E-07	7.57E-07	1.09E-06	6.54E-07
**48h**	6.13E-07	2.30E-06	1.47E-06	3.61E-07
**72h**	3.38E-07	1.02E-06	1.04E-06	2.35E-07
	cgd5_10			
**2h**	1.29E-04	1.13E-03	1.09E-03	2.16E-04
**6h**	4.88E-05	1.35E-04	7.59E-04	1.43E-04
**12h**	4.17E-05	2.80E-04	4.97E-04	6.04E-05
**24h**	9.76E-05	1.22E-04	1.82E-04	6.46E-05
**36h**	3.65E-05	1.44E-04	1.02E-04	5.30E-05
**48h**	4.29E-05	8.35E-05	9.22E-05	2.93E-05
**72h**	2.32E-05	3.06E-05	2.83E-05	1.45E-05
	cgd5_1030			
**2h**	1.83E-06	1.17E-04	2.35E-04	9.44E-05
**6h**	2.01E-06	1.47E-05	8.20E-05	9.51E-06
**12h**	2.83E-07	1.06E-05	2.11E-05	2.36E-06
**24h**	1.19E-06	1.28E-04	3.88E-05	3.93E-06
**36h**	2.94E-06	4.60E-05	1.17E-04	8.10E-06
**48h**	1.50E-06	1.59E-05	2.07E-05	1.17E-06
**72h**	9.56E-06	1.05E-05	1.70E-04	5.95E-07
	cgd5_1050			
**2h**	9.08E-08	8.51E-08	2.61E-08	2.80E-08
**6h**	2.24E-06	1.73E-07	1.69E-06	2.60E-06
**12h**	3.85E-07	2.39E-07	5.70E-07	4.80E-07
**24h**	3.24E-07	2.90E-07	3.98E-07	1.98E-07
**36h**	3.48E-07	3.37E-07	6.68E-07	5.46E-07
**48h**	2.34E-07	2.66E-07	3.72E-07	3.04E-07
**72h**	8.26E-08	6.22E-08	1.60E-07	2.82E-07
	cgd5_1080			
**2h**	3.40E-04	1.60E-04	1.08E-04	1.80E-05
**6h**	4.51E-04	3.82E-04	9.18E-05	2.43E-04
**12h**	5.60E-04	3.36E-04	2.83E-04	8.44E-06
**24h**	4.90E-04	2.71E-04	2.73E-04	6.64E-06
**36h**	1.64E-04	3.37E-04	9.04E-05	8.54E-06
**48h**	8.64E-04	7.31E-05	8.14E-05	5.85E-05
**72h**	2.29E-04	5.74E-05	4.92E-05	7.24E-06
	cgd5_1150			
**2h**	3.68E-07	8.33E-07	2.88E-07	7.05E-07
**6h**	9.07E-07	6.20E-07	8.04E-07	4.29E-06
**12h**	1.09E-07	2.32E-07	2.17E-07	4.73E-07
**24h**	4.67E-07	6.12E-07	6.28E-07	7.62E-07
**36h**	4.64E-07	5.85E-07	2.47E-07	4.20E-07
**48h**	1.56E-07	2.35E-07	1.72E-07	5.37E-07
**72h**	9.97E-08	1.07E-07	6.57E-08	9.01E-08
	cgd5_1290			
**2h**	5.61E-04	1.33E-04	3.54E-05	4.07E-06
**6h**	5.02E-05	2.62E-04	1.06E-04	1.30E-05
**12h**	1.31E-05	1.57E-04	3.07E-05	3.12E-05
**24h**	1.29E-04	2.82E-04	4.75E-04	5.04E-06

**36h**	1.19E-04	5.47E-04	9.37E-05	7.11E-05
**48h**	9.54E-05	1.98E-04	1.74E-04	5.61E-05
**72h**	5.62E-05	1.07E-04	1.05E-04	1.64E-05
	cgd5_1310			
**2h**	0.00E+00	2.33E-06	0.00E+00	2.08E-08
**6h**	0.00E+00	3.08E-06	5.04E-04	4.21E-07
**12h**	0.00E+00	0.00E+00	2.98E-04	9.91E-07
**24h**	0.00E+00	0.00E+00	3.80E-03	6.99E-07
**36h**	0.00E+00	0.00E+00	1.29E-03	2.47E-07
**48h**	0.00E+00	0.00E+00	1.41E-03	7.67E-07
**72h**	0.00E+00	0.00E+00	4.56E-04	3.48E-07
	cgd5_1420			
**2h**	9.29E-08	0.00E+00	6.58E-09	0.00E+00
**6h**	6.35E-08	1.17E-06	8.95E-08	1.22E-06
**12h**	0.00E+00	1.32E-05	1.27E-06	2.89E-06
**24h**	4.81E-07	1.44E-05	1.26E-06	2.52E-08
**36h**	3.48E-06	8.40E-05	3.84E-06	1.15E-06
**48h**	4.51E-05	5.33E-05	3.57E-05	2.90E-06
**72h**	6.95E-06	8.21E-05	9.94E-06	6.65E-06
	cgd5_1470			
**2h**	1.49E-04	1.40E-04	5.55E-05	4.40E-04
**6h**	2.53E-04	6.11E-05	1.94E-04	4.19E-04
**12h**	9.85E-05	1.39E-04	1.46E-04	7.13E-04
**24h**	1.81E-04	1.83E-04	1.98E-04	2.74E-04
**36h**	1.31E-04	7.03E-05	1.33E-04	5.30E-04
**48h**	6.43E-05	6.57E-05	6.22E-05	1.48E-04
**72h**	2.84E-05	2.18E-05	2.03E-05	7.50E-05
	cgd5_1520			
**2h**	1.39E-08	0.00E+00	0.00E+00	9.37E-08
**6h**	0.00E+00	2.37E-08	1.27E-08	5.14E-07
**12h**	2.09E-05	6.57E-05	1.15E-05	7.68E-04
**24h**	4.95E-06	4.17E-06	9.78E-07	2.94E-05
**36h**	4.59E-07	1.40E-06	2.88E-06	8.87E-05
**48h**	7.44E-07	4.34E-07	3.41E-07	2.52E-05
**72h**	1.44E-06	3.46E-07	2.25E-07	2.10E-05
	cgd5_1530			
**2h**	2.14E-09	0.00E+00	0.00E+00	3.76E-09
**6h**	8.68E-07	1.55E-07	5.02E-07	2.76E-06
**12h**	1.60E-07	1.11E-07	9.34E-08	8.31E-07
**24h**	1.29E-07	9.07E-08	5.53E-07	8.10E-07
**36h**	3.49E-07	1.91E-07	3.35E-07	3.64E-06
**48h**	4.52E-07	2.31E-07	4.23E-07	9.83E-07
**72h**	6.42E-08	8.82E-08	1.61E-08	3.11E-07
	cgd5_1540			
**2h**	0.00E+00	1.78E-09	0.00E+00	0.00E+00
**6h**	1.27E-07	2.71E-08	1.84E-07	4.67E-05
**12h**	1.78E-07	7.14E-08	3.80E-08	2.22E-05
**24h**	1.02E-07	1.58E-06	4.45E-07	1.61E-05
**36h**	6.63E-07	1.45E-06	2.46E-07	7.19E-06
**48h**	1.97E-07	3.20E-07	2.58E-07	1.10E-05
**72h**	6.47E-08	1.04E-06	2.50E-08	3.49E-05
	cgd5_1740			
**2h**	3.01E-06	7.08E-07	6.15E-07	1.32E-06

**6h**	4.77E-06	1.16E-06	1.96E-06	5.50E-06
**12h**	3.33E-07	3.77E-08	3.16E-07	2.91E-07
**24h**	6.37E-06	3.50E-07	3.10E-06	1.66E-06
**36h**	1.42E-06	5.06E-07	3.88E-07	1.78E-06
**48h**	4.67E-06	5.69E-07	1.29E-06	2.14E-06
**72h**	4.70E-07	3.82E-07	4.02E-07	6.06E-07
	cgd5_1790			
**2h**	1.60E-05	2.12E-07	4.94E-07	1.91E-06
**6h**	3.56E-06	2.00E-07	5.86E-07	1.29E-06
**12h**	2.02E-06	6.14E-08	6.81E-07	6.31E-07
**24h**	5.44E-06	4.23E-07	1.42E-06	1.14E-06
**36h**	1.46E-06	2.34E-07	5.36E-07	8.61E-07
**48h**	3.52E-06	3.28E-07	8.00E-07	1.01E-06
**72h**	4.78E-06	2.84E-07	8.06E-07	8.03E-07
	cgd5_1840			
**2h**	3.65E-06	1.06E-06	1.15E-06	5.24E-06
**6h**	3.15E-07	3.86E-07	9.18E-07	1.28E-06
**12h**	1.62E-06	7.03E-07	1.52E-06	1.38E-06
**24h**	8.39E-06	1.26E-06	2.41E-06	2.05E-06
**36h**	1.07E-06	5.83E-07	8.71E-07	7.21E-07
**48h**	1.46E-06	2.47E-07	1.00E-06	9.01E-07
**72h**	5.19E-07	1.64E-07	2.47E-07	1.71E-07
	cgd5_2000			
**2h**	1.13E-03	1.98E-05	4.41E-04	7.76E-04
**6h**	4.29E-05	3.67E-06	3.26E-05	1.25E-04
**12h**	2.90E-04	5.29E-06	2.10E-04	1.96E-04
**24h**	6.03E-04	1.25E-05	9.68E-05	9.55E-05
**36h**	2.04E-04	1.48E-05	1.11E-04	1.27E-04
**48h**	6.47E-04	8.30E-06	9.88E-05	9.21E-05
**72h**	2.84E-04	2.95E-06	2.15E-05	1.98E-05
	cgd5_2030			
**2h**	0.00E+00	0.00E+00	0.00E+00	0.00E+00
**6h**	4.34E-09	6.35E-09	1.46E-08	9.79E-09
**12h**	6.31E-08	2.63E-08	1.15E-08	2.25E-08
**24h**	3.90E-08	1.36E-08	7.85E-08	6.50E-08
**36h**	4.30E-08	4.67E-08	1.06E-07	1.02E-07
**48h**	1.76E-07	3.25E-08	1.22E-07	5.98E-08
**72h**	3.38E-08	1.97E-08	2.14E-08	2.19E-08
	cgd5_2080			
**2h**	0.00E+00	0.00E+00	0.00E+00	0.00E+00
**6h**	3.29E-10	6.82E-10	1.66E-08	2.75E-10
**12h**	4.55E-09	1.42E-09	2.05E-08	1.63E-08
**24h**	1.01E-07	2.08E-08	9.51E-08	7.61E-08
**36h**	8.49E-08	9.67E-08	1.39E-07	1.30E-07
**48h**	1.10E-06	2.44E-07	6.86E-07	9.70E-07
**72h**	1.44E-06	3.76E-07	1.70E-06	9.92E-07
	cgd5_2450			
**2h**	0.00E+00	1.34E-08	0.00E+00	2.36E-07
**6h**	3.28E-06	1.07E-06	3.84E-06	1.29E-05
**12h**	2.25E-07	8.41E-08	4.28E-07	1.70E-07
**24h**	2.90E-06	3.15E-07	4.47E-06	6.80E-06
**36h**	4.83E-06	2.32E-07	9.20E-07	2.35E-06
**48h**	2.06E-05	3.80E-07	1.68E-06	3.48E-06

**72h**	1.28E-05	1.41E-06	4.21E-06	1.70E-06
	cgd5_2630			
**2h**	1.19E-04	2.97E-04	2.80E-03	1.14E-02
**6h**	6.10E-05	7.14E-05	2.09E-04	1.24E-03
**12h**	1.11E-04	4.69E-05	6.30E-04	1.02E-03
**24h**	8.34E-05	1.99E-04	7.36E-04	9.61E-04
**36h**	3.16E-05	1.57E-04	4.40E-04	4.58E-04
**48h**	8.18E-05	4.59E-05	6.90E-04	6.02E-04
**72h**	4.97E-05	3.40E-05	4.36E-04	3.21E-04
	cgd5_270			
**2h**	0.00E+00	3.14E-05	0.00E+00	2.40E-06
**6h**	5.89E-05	1.14E-05	1.11E-04	1.33E-05
**12h**	7.49E-05	8.47E-06	4.61E-05	1.55E-04
**24h**	4.84E-06	2.22E-07	3.19E-06	6.86E-06
**36h**	5.56E-05	4.01E-05	2.05E-06	6.33E-06
**48h**	5.38E-05	6.86E-06	3.55E-06	5.93E-06
**72h**	2.31E-05	2.43E-05	1.14E-06	5.49E-06
	cgd5_2700			
**2h**	3.27E-06	0.00E+00	1.58E-04	1.57E+02
**6h**	1.21E-06	1.28E-06	3.96E-06	5.48E+01
**12h**	1.29E-05	5.71E-06	3.95E-06	2.96E+01
**24h**	0.00E+00	0.00E+00	1.98E-05	2.34E+01
**36h**	8.84E-07	4.41E-06	1.13E-05	6.75E+00
**48h**	1.85E-05	0.00E+00	8.74E-05	7.47E+00
**72h**	1.06E-07	5.06E-07	4.57E-07	3.00E+00
	cgd5_2710			
**2h**	3.66E-05	8.50E-08	3.11E-08	1.89E-07
**6h**	6.95E-07	1.01E-07	2.80E-08	1.50E-07
**12h**	8.54E-07	1.13E-07	1.70E-08	8.29E-08
**24h**	2.32E-07	9.83E-08	6.17E-08	8.00E-08
**36h**	8.18E-08	1.50E-07	4.05E-08	9.74E-08
**48h**	1.28E-06	1.18E-07	3.54E-08	1.13E-07
**72h**	1.66E-07	3.56E-08	5.57E-09	4.56E-09
	cgd5_2720			
**2h**	3.65E-04	1.89E-05	7.45E-06	3.08E-05
**6h**	5.92E-06	8.52E-05	8.63E-07	2.57E-06
**12h**	8.45E-05	1.17E-05	2.05E-05	2.55E-05
**24h**	7.34E-05	1.89E-05	4.01E-06	7.65E-06
**36h**	4.97E-06	1.16E-05	3.16E-06	4.92E-06
**48h**	1.34E-05	8.29E-06	9.76E-07	2.15E-06
**72h**	1.66E-06	4.43E-06	4.41E-07	1.15E-06
	cgd5_2750			
**2h**	1.08E-05	1.17E-04	6.78E-08	9.08E-06
**6h**	6.55E-05	9.58E-05	9.33E-07	2.65E-05
**12h**	6.21E-04	5.68E-05	1.55E-05	8.62E-05
**24h**	1.57E-05	1.62E-05	4.77E-06	1.40E-05
**36h**	1.51E-05	1.68E-05	8.52E-06	4.08E-05
**48h**	1.93E-05	1.39E-05	7.82E-06	6.53E-05
**72h**	1.86E-04	5.27E-05	2.07E-05	6.89E-05
	cgd5_2780			
**2h**	4.77E-04	9.25E-04	8.39E-05	3.69E-04
**6h**	2.03E-03	2.05E-03	3.33E-04	1.19E-03
**12h**	1.17E-03	1.32E-04	1.43E-04	2.45E-04

**24h**	9.04E-04	9.59E-04	1.39E-04	4.73E-04
**36h**	3.53E-04	6.78E-04	6.52E-05	1.68E-04
**48h**	5.84E-04	1.04E-04	5.83E-05	7.19E-05
**72h**	1.01E-04	3.52E-05	1.02E-05	1.11E-05
	cgd5_2800			
**2h**	2.42E-06	1.20E-06	3.41E-04	2.86E-04
**6h**	5.67E-06	2.59E-06	1.60E-03	1.51E-04
**12h**	1.59E-04	4.05E-05	1.18E-02	1.34E-03
**24h**	4.37E-05	7.00E-05	1.50E-03	7.10E-04
**36h**	1.34E-05	3.34E-05	4.04E-03	7.37E-04
**48h**	3.75E-05	1.41E-05	1.43E-03	1.24E-03
**72h**	3.87E-06	8.48E-06	1.10E-03	8.56E-04
	cgd5_2900			
**2h**	0.00E+00	0.00E+00	0.00E+00	0.00E+00
**6h**	0.00E+00	0.00E+00	0.00E+00	0.00E+00
**12h**	7.24E-05	1.05E-05	2.19E-05	1.04E-04
**24h**	3.01E-05	9.90E-06	2.00E-06	5.32E-06
**36h**	4.65E-05	2.76E-05	5.94E-05	1.01E-04
**48h**	5.18E-05	1.38E-06	4.85E-05	2.01E-04
**72h**	9.49E-05	3.72E-06	7.33E-05	1.50E-04
	cgd5_3040			
**2h**	0.00E+00	2.42E-05	3.92E-06	6.14E-06
**6h**	1.26E-03	1.56E-05	3.64E-05	4.59E-05
**12h**	6.38E-06	1.63E-06	5.34E-06	5.27E-06
**24h**	1.37E-05	1.05E-05	1.79E-05	1.03E-05
**36h**	2.22E-06	6.08E-06	3.93E-06	5.68E-06
**48h**	7.34E-06	1.35E-05	2.98E-06	2.06E-06
**72h**	6.75E-07	2.83E-06	5.37E-07	7.22E-07
	cgd5_3050			
**2h**	0.00E+00	0.00E+00	0.00E+00	0.00E+00
**6h**	0.00E+00	7.49E-07	0.00E+00	0.00E+00
**12h**	2.15E-04	4.22E-05	2.39E-03	2.52E-03
**24h**	6.50E-05	4.18E-05	6.62E-05	1.23E-04
**36h**	8.80E-06	7.68E-06	1.17E-03	1.01E-03
**48h**	2.58E-06	3.44E-06	8.85E-05	4.03E-04
**72h**	2.88E-05	2.90E-06	2.24E-05	5.11E-05
	cgd5_3060			
**2h**	0.00E+00	0.00E+00	0.00E+00	0.00E+00
**6h**	0.00E+00	0.00E+00	0.00E+00	1.79E-10
**12h**	1.54E-08	2.74E-09	5.48E-09	9.85E-09
**24h**	5.04E-09	2.56E-08	2.46E-08	6.98E-09
**36h**	3.63E-08	7.70E-07	8.08E-08	9.42E-08
**48h**	1.80E-07	8.60E-07	1.41E-07	5.93E-08
**72h**	1.67E-07	4.00E-07	5.08E-08	9.38E-08
	cgd7_4450			
**2h**	6.10E-03	1.65E-02	3.05E-01	1.54E-03
**6h**	2.01E-01	1.54E-01	1.27E+01	4.83E-01
**12h**	1.19E-03	1.62E-02	1.64E+00	1.52E-02
**24h**	2.79E-02	4.92E-02	3.87E+00	8.10E-02
**36h**	3.60E-02	1.49E-01	2.30E+00	5.94E-02
**48h**	3.23E-02	1.01E-01	1.08E+00	4.80E-02
**72h**	2.38E-03	9.75E-03	3.56E-01	4.35E-03
	cgd5_3720			

**2h**	4.52E-06	1.17E-05	3.68E-05	6.04E-06
**6h**	1.20E-05	1.82E-05	6.79E-05	4.29E-05
**12h**	1.34E-06	0.00E+00	3.36E-05	1.35E-05
**24h**	1.10E-05	1.40E-05	8.04E-05	2.03E-05
**36h**	5.58E-06	5.10E-06	3.23E-05	5.44E-06
**48h**	3.02E-06	2.74E-06	2.89E-05	1.11E-05
**72h**	4.60E-07	8.43E-07	9.33E-06	2.00E-06
	cgd5_4450			
**2h**	2.34E-07	2.62E-07	1.01E-06	8.49E-08
**6h**	8.69E-08	1.85E-07	5.97E-06	1.02E-06
**12h**	7.19E-08	2.08E-07	1.10E-05	1.93E-07
**24h**	9.04E-07	7.93E-07	1.19E-05	1.28E-06
**36h**	5.87E-07	1.64E-06	1.80E-05	1.76E-06
**48h**	2.04E-06	2.34E-06	4.23E-05	1.27E-05
**72h**	4.46E-06	4.67E-06	7.01E-05	6.40E-06
	cgd5_4540			
**2h**	3.26E-04	2.39E-04	2.96E-02	1.98E-04
**6h**	5.85E-04	3.68E-04	7.44E-02	1.49E-03
**12h**	4.19E-05	8.99E-05	5.32E-03	2.86E-04
**24h**	1.87E-04	2.51E-04	2.69E-02	5.12E-04
**36h**	1.44E-04	1.74E-04	3.18E-02	5.35E-04
**48h**	1.83E-04	4.11E-04	1.94E-02	7.44E-04
**72h**	1.07E-04	1.10E-04	8.29E-03	1.65E-04
	cgd5_4560			
**2h**	4.26E-04	3.94E-04	2.73E-04	1.28E-04
**6h**	2.68E-04	1.93E-04	2.85E-03	4.71E-04
**12h**	8.93E-06	8.65E-05	1.30E-04	1.84E-05
**24h**	1.02E-04	2.07E-04	1.03E-03	1.49E-04
**36h**	6.97E-05	1.83E-04	2.86E-04	1.60E-04
**48h**	5.09E-05	2.55E-05	3.74E-04	6.79E-05
**72h**	7.90E-06	9.93E-06	3.96E-05	1.41E-05
	cgd5_4580			
**2h**	3.70E-05	4.19E-05	7.67E-05	2.44E-05
**6h**	4.18E-05	3.54E-05	1.06E-03	3.68E-05
**12h**	1.05E-05	3.53E-05	6.26E-04	3.18E-05
**24h**	2.66E-05	3.27E-05	1.06E-03	3.81E-05
**36h**	2.79E-05	5.85E-05	1.13E-03	4.69E-05
**48h**	3.41E-05	2.78E-05	1.83E-03	8.34E-05
**72h**	1.36E-05	1.13E-05	1.29E-04	1.22E-05
	cgd5_4600			
**2h**	4.07E-07	2.37E-07	0.00E+00	6.19E-07
**6h**	2.32E-06	5.54E-07	3.48E-05	1.10E-06
**12h**	1.12E-06	8.26E-06	6.11E-06	3.40E-06
**24h**	2.59E-06	3.24E-06	1.60E-05	2.73E-06
**36h**	6.86E-06	6.42E-06	2.21E-05	6.31E-06
**48h**	1.89E-06	1.71E-06	3.16E-05	5.00E-06
**72h**	3.52E-07	6.45E-07	1.03E-05	9.12E-07
	cgd6_1850			
**2h**	0.00E+00	0.00E+00	0.00E+00	0.00E+00
**6h**	0.00E+00	0.00E+00	0.00E+00	4.79E+01
**12h**	0.00E+00	0.00E+00	0.00E+00	-2.77E+10
**24h**	0.00E+00	0.00E+00	0.00E+00	1.30E+03
**36h**	0.00E+00	0.00E+00	0.00E+00	-2.76E+10

**48h**	0.00E+00	0.00E+00	0.00E+00	4.47E+03
**72h**	0.00E+00	0.00E+00	0.00E+00	2.42E-07
	cgd6_3430			
**2h**	3.15E-07	2.92E-07	1.08E-06	1.05E-06
**6h**	3.48E-07	3.52E-07	2.17E-06	2.23E-06
**12h**	1.42E-09	6.49E-08	7.14E-07	2.11E-07
**24h**	5.16E-07	7.10E-07	3.75E-06	4.76E-07
**36h**	1.94E-07	5.11E-07	1.06E-06	1.94E-07
**48h**	1.83E-07	9.64E-08	8.59E-07	6.12E-07
**72h**	8.88E-08	1.73E-07	6.10E-07	1.15E-07
	cgd6_330			
**2h**	1.37E-06	2.29E-07	1.48E-05	3.34E-06
**6h**	8.14E-08	1.93E-08	1.18E-05	1.88E-07
**12h**	2.36E-05	5.14E-05	9.19E-05	6.45E-06
**24h**	2.31E-06	7.34E-06	6.57E-05	1.66E-06
**36h**	5.84E-06	1.58E-05	8.94E-05	6.25E-06
**48h**	9.44E-06	2.81E-06	4.82E-04	8.42E-06
**72h**	2.44E-06	4.55E-06	2.55E-04	5.75E-06
	cgd7_1140			
**2h**	0.00E+00	0.00E+00	0.00E+00	0.00E+00
**6h**	0.00E+00	0.00E+00	0.00E+00	0.00E+00
**12h**	1.06E-08	9.54E-07	1.06E-08	1.18E-07
**24h**	2.60E-08	2.89E-07	2.20E-07	1.75E-07
**36h**	1.12E-07	7.35E-07	2.75E-07	6.47E-08
**48h**	4.57E-08	2.12E-07	1.09E-06	2.08E-07
**72h**	3.40E-08	8.35E-07	4.85E-07	1.30E-07
	cgd6_380			
**2h**	0.00E+00	0.00E+00	0.00E+00	0.00E+00
**6h**	0.00E+00	0.00E+00	0.00E+00	0.00E+00
**12h**	6.73E-06	8.84E-04	3.31E-06	3.26E-04
**24h**	6.42E-06	1.11E-04	5.74E-06	3.80E-06
**36h**	3.56E-06	7.91E-04	2.95E-06	1.75E-05
**48h**	9.66E-05	3.05E-04	1.04E-04	1.98E-04
**72h**	2.02E-05	9.98E-04	9.50E-06	1.69E-04
	cgd7_1910			
**2h**	3.71E-07	1.80E-07	0.00E+00	3.85E-07
**6h**	1.51E-05	5.61E-05	1.17E-05	2.91E-05
**12h**	3.22E-06	3.19E-05	5.17E-06	5.84E-06
**24h**	1.05E-05	1.08E-05	2.77E-05	3.18E-05
**36h**	1.50E-05	8.52E-05	2.28E-05	2.19E-05
**48h**	3.72E-05	2.19E-05	3.76E-05	2.46E-05
**72h**	1.09E-05	1.15E-05	6.15E-06	7.76E-06
	cgd7_3000			
**2h**	1.73E-06	1.20E-06	3.16E-07	8.51E-07
**6h**	1.76E-06	6.82E-06	5.71E-07	2.75E-06
**12h**	2.17E-07	2.34E-06	4.41E-07	4.62E-07
**24h**	2.70E-06	2.21E-06	8.97E-07	2.36E-06
**36h**	9.30E-07	2.80E-06	1.68E-06	5.96E-07
**48h**	2.84E-06	1.78E-06	1.26E-06	1.23E-06
**72h**	4.21E-07	9.01E-07	1.58E-06	1.50E-06
	cgd7_3140			
**2h**	0.00E+00	0.00E+00	0.00E+00	0.00E+00
**6h**	2.86E-06	5.77E-06	4.01E-07	2.52E-06

**12h**	1.34E-06	2.71E-05	3.19E-07	8.26E-07
**24h**	1.05E-06	1.33E-06	1.13E-06	9.61E-07
**36h**	4.51E-06	5.37E-06	5.32E-06	2.32E-06
**48h**	4.73E-06	1.43E-06	2.23E-06	1.32E-06
**72h**	5.10E-07	2.36E-06	8.87E-07	4.30E-06
	cgd7_4090			
**2h**	0.00E+00	0.00E+00	0.00E+00	6.89E-06
**6h**	0.00E+00	0.00E+00	0.00E+00	1.98E-05
**12h**	2.64E-03	1.29E-02	1.77E-03	3.82E-03
**24h**	7.24E-04	7.46E-03	3.65E-04	1.15E-03
**36h**	8.07E-03	1.87E-02	1.69E-03	8.79E-03
**48h**	1.31E-02	7.35E-03	2.27E-03	4.06E-03
**72h**	3.00E-02	5.55E-02	7.51E-04	7.07E-03
	cgd7_430			
**2h**	1.91E-06	1.42E-05	3.49E-07	5.37E-07
**6h**	1.69E-06	6.34E-06	1.05E-07	3.31E-07
**12h**	6.30E-06	6.75E-06	3.65E-07	0.00E+00
**24h**	1.01E-06	2.93E-05	6.17E-06	4.93E-06
**36h**	3.17E-06	3.33E-05	2.84E-06	1.21E-06
**48h**	5.36E-06	3.12E-05	1.19E-05	1.48E-05
**72h**	5.92E-07	2.35E-05	4.66E-06	1.74E-05
	cgd7_50			
**2h**	0.00E+00	0.00E+00	0.00E+00	4.78E-09
**6h**	8.07E-08	1.70E-08	7.64E-07	5.61E-07
**12h**	6.36E-08	7.60E-07	1.72E-07	2.18E-07
**24h**	2.49E-07	4.91E-07	1.85E-06	3.77E-07
**36h**	2.63E-06	9.85E-07	1.04E-06	7.35E-07
**48h**	1.96E-06	9.61E-07	2.10E-06	3.22E-06
**72h**	2.01E-06	6.60E-07	2.12E-06	1.43E-06
	cgd7_520			
**2h**	8.85E-08	1.22E-06	2.03E-07	1.50E-06
**6h**	1.17E-06	1.56E-06	6.43E-08	7.35E-07
**12h**	1.79E-08	3.61E-08	3.49E-08	2.08E-07
**24h**	5.61E-07	4.41E-07	5.21E-07	2.25E-07
**36h**	3.61E-07	5.06E-07	3.78E-07	2.01E-07
**48h**	9.61E-07	3.41E-07	1.31E-06	4.34E-07
**72h**	2.52E-06	8.37E-07	1.66E-06	2.37E-06
	cgd7_5260			
**2h**	0.00E+00	1.77E-05	0.00E+00	6.42E-03
**6h**	0.00E+00	5.33E-05	2.32E-02	3.76E-02
**12h**	1.88E-01	1.15E-05	0.00E+00	1.08E-02
**24h**	1.18E-01	4.41E-04	4.33E-02	4.25E-02
**36h**	4.27E-01	5.28E-03	3.50E-02	6.57E-02
**48h**	4.18E-01	2.70E-02	1.08E-01	7.82E-02
**72h**	6.10E-01	9.85E-03	7.07E-02	2.06E-01
	cgd7_710			
**2h**	0.00E+00	0.00E+00	0.00E+00	0.00E+00
**6h**	0.00E+00	0.00E+00	0.00E+00	0.00E+00
**12h**	3.08E-05	1.16E-06	1.37E-04	3.67E-06
**24h**	1.38E-05	6.27E-07	2.85E-06	3.49E-06
**36h**	8.82E-06	1.35E-06	1.59E-06	1.21E-05
**48h**	2.31E-05	2.10E-06	6.35E-06	8.95E-06
**72h**	7.42E-05	6.00E-06	2.80E-05	4.95E-05

	cgd8_310			
**2h**	0.00E+00	3.01E-08	0.00E+00	1.67E-05
**6h**	6.45E-05	1.56E-06	7.90E-06	5.82E-05
**12h**	4.19E-02	8.43E-04	3.73E-03	7.13E-03
**24h**	8.19E-03	3.09E-04	3.45E-04	1.18E-03
**36h**	1.98E-03	9.47E-04	2.95E-04	1.99E-03
**48h**	1.64E+00	3.81E-05	9.49E-05	7.29E-04
**72h**	1.07E-03	1.10E-05	1.74E-04	1.15E-03
	cgd8_1090			
**2h**	2.80E-04	1.48E-06	8.26E-07	1.02E-04
**6h**	1.54E-05	3.88E-08	0.00E+00	0.00E+00
**12h**	2.91E-04	5.34E-06	1.09E-05	3.73E-05
**24h**	3.15E-04	2.54E-06	2.39E-05	3.09E-05
**36h**	3.06E-04	8.87E-06	6.85E-05	1.11E-04
**48h**	2.66E-04	2.01E-06	1.28E-04	8.02E-05
**72h**	4.14E-04	6.05E-06	1.49E-04	1.33E-04
	cgd8_1150			
**2h**	3.14E-06	7.56E-07	4.56E-07	6.68E-06
**6h**	1.44E-06	5.59E-07	1.51E-06	4.40E-06
**12h**	8.63E-08	2.14E-07	0.00E+00	1.18E-06
**24h**	2.19E-06	5.75E-07	2.73E-06	2.06E-06
**36h**	8.72E-06	3.16E-06	7.80E-07	4.31E-05
**48h**	1.69E-05	3.33E-06	4.27E-06	6.82E-06
**72h**	1.42E-05	2.76E-06	1.06E-05	8.40E-06
	cgd8_1890			
**2h**	0.00E+00	2.25E-07	4.35E-07	2.59E-06
**6h**	1.00E-05	5.56E-07	1.48E-06	2.34E-06
**12h**	1.21E-06	6.98E-07	9.17E-07	7.71E-07
**24h**	3.55E-06	9.04E-07	2.89E-06	5.13E-06
**36h**	4.87E-06	2.81E-06	2.37E-06	2.69E-06
**48h**	7.27E-06	8.34E-07	1.87E-06	3.62E-06
**72h**	1.64E-06	1.28E-06	4.05E-06	4.35E-06
