# Supplementary material for: The Cryptosporidium Parvum Transcriptome during In Vitro Development
Source: PLoS One. 2012 Mar 15;7(3):e31715. doi: 10.1371/journal.pone.0031715 (PMC3305300; doi:10.1371/journal.pone.0031715)
Supplement: Table S2 — Primers used for C. parvum RT-PCR. (PDF) [file pone.0031715.s005.pdf]

Plate	Gene	Left Primer Sequence	Right Primer Sequence
1	cgd2_1060	TAATGGGTTCGAGAAGAAATGG	TGAGCTATGACAGTAAGGGCAA
1	cgd2_1100	CACATCAGGTAAAGGAGCATCA	TCCATTGCTTCGTGAATAGAAA
1	cgd2_1110	CGCTCCTTACCTAAATCTTCCC	CTGATTCTTTCCAATGACGGTT
1	cgd2_1150	ATCTCTTTCCATCTGCCACTTC	ATCAGCTCTGCAAACATACCAA
1	cgd2_1160	TCTGACTCTCAAGCCAATGAAA	TAATTGAACCAGCTCACCAGAA
1	cgd2_1170	AGGTGACTCTGCAACATCTGAA	GCCAATAAAGCTATGGTCTGCT
1	cgd2_1190	ATAATGGATGCAACTCCCGAA	CGCGTTTACAATGCTGAGAAA
1	cgd2_1200	TGTGCTACTTGTTGGCTTTACAC	GGTACAATGGCCTTCCTATGTT
1	cgd2_1220	AATACCCACTAAGCCCAACAAA	AAATGTTCCCTCCTTCCCTATC
1	cgd2_1270	GAGATTCCTTCCTGGTTTGTTG	TAAGCTCTTCTGGGCATGAACT
1	cgd2_1290	CATGTTCTCATTTGGATTTACAGG	TTCAGGAGATGGACTTGATGG
2	cgd2_1310	CCAACTGGAGCTAAATGCAAA	TCCACCTCCTAATACCGAAAGA
2	cgd2_1350	AAATGAGGGTGAAGGTATGGAA	AATCCAATGACAGGGAGAAAGA
2	cgd2_1370	CAAGCAATACCAACCACAAAGA	ACAGACTTCTCGATTCCTGACAA
2	cgd2_1450	ATTGGAGGCATTACAGGTGACT	TGGCAGTGTGCTAGTAAGGAAA
2	cgd2_1470	GCAGCAGGAAGAACTCAAGTAAA	TGCTTTCCAATTCGCTATCC
2	cgd2_1490	ATCGTACAAGGGTGTGTTTCATT	ACAAGAGATGGCAGTGTTCTCA
2	cgd2_1510	GCAAAGTTCACAATCCAAGATG	ACCATTTATTCCCATGCAGTTC
2	cgd2_1520	CAGTTTATTCCTCCTGGGATTG	TATTGGCCTGACTTGTCGATT
2	cgd2_1530	GGAAATAGATGGAAAGCAATAGGA	CGAGCAATATGCCTTGAATAGA
3	cgd2_1610	CTCCTTGGCTGCTATGCTAACT	GCAATATGCTTTGCCCAATAA
3	cgd2_1620	TGAGATGCAACGAACACAATAA	ATTCCCTTCAAATCGCTCAA
3	cgd2_1740	GTTGTAATGCTAACGCCCATTT	AACCTCATTTCTCTCCCATTCA
3	cgd2_1760	AATATGATTCCGCCTTCATTACA	TGCTTGATGATTCTTTGCTTTC
3	cgd2_1800	AATGAAGGGATGCCAATAAAGA	CTTCTTCTGCTCTGGTGTGAGA
3	cgd2_1830	CTGAAGCATTTGGACAGAAAGA	TTGCGACTTTAATTGGGTTATG
3	cgd2_1850	GAGGCCATTAAACGTAATCCAA	AACTTTGGGTCTAAATCGAGGG
3	cgd2_1910	TGGTGGTTCAGAGAATGTTGTT	CATCCCACGATCTTTGTTACTG
3	cgd2_1920	CAGCCCATTATTCATCAGAAAGT	TGTGGTAAATTCGGCTATCAAG
4	cgd2_1330	AAGAATCCCAAGAGGAACGAA	GTACAACAATTTCTCCGCCAA
4	cgd2_1340	TCCAAGTTCAGCTACTTCACCA	TAAAGGGTATCTTTGAATGCGG
4	cgd2_1380	GATTCTGAGCTGCCTCCATT	CTAAATGCAACCCTCTTATTACACTTT
4	cgd2_1410	TATTCAACCATTCAACACCGAA	TTCTTTGTGAGATAATTCCGCC
4	cgd2_1420	AAGAGGAAGAATATCGTAGGAAACAAG	CGGGTGGTTATCAATACATTCC
4	cgd2_1430	ATCCACTATCTCAGCCACCATT	TTGCTCATTCTGGTCTCTTTCA
4	cgd2_1440	AGAATATGCAATGGAGGCAGTT	GCAAATGCAAGGCAAGTATTATC
4	cgd2_1500	AGATCCTATTGAACCAGCAGCA	ACCAATGCACTAGCCAGCTTT
4	cgd2_1590	ACGATAGGGAGCTATGAATGTGA	CAACGTGGAACTAGGACAAACA
4	cgd2_1680	TGGCACATCAACTAGCCAAA	CTACAAAGAAAGGGATTGCTTGA
4	cgd2_1690	GTGTCAGTCGCCACCTCTTT	CCCACACTTTGAGCTGTTTGT
4	cgd2_1700	GGTATGCAACAAGATCCAGCA	ATATAGCCATCTCCGTTAATATCCAGT
5	cgd2_1710	TATTAACAGCGAGGAAACTGGG	TTCTTCTTGCGGACTAGCAAA
5	cgd2_1730	ATATTCAAGCTCGCTGCCAA	TGAGTCTAACCAAGAAATCGGG
5	cgd2_1750	AAAGGGTGCTCAGTTCCAAAG	TCAATGTAGCCAATTTCTTCGTC
5	cgd2_1810	AAAGTGGTCATTTCTGCTAGGG	CTGCTGTTGTGACTGGAGAGTT
5	cgd2_1870	CTTTGCTTCTCGCCTTGTAACT	ATTCTTTATTGGAGCTGGTGGA
5	cgd2_1880	TAGGAAGCTGCGATTACAATGA	CAAGTGCCAATAGTTCAGCAAG
5	cgd2_1900	AACCAGAAGCTGTTATGATGAGG	TAGGCAATTCTTGATTGAGCAG
5	cgd2_1940	AGAACTATTCGTCTTCAACTATGGG	GTCGATCTATTCGTGATGTCGT
5	cgd2_1950	GGACAGAAATGGTTGGAGTGA	TTGGAACTTAGAGGAGTGTCAGG
5	cgd2_1980	ATCCATCTGAAACTTCCTCCAA	CCTCATCTCCTAGCACAATTCC
5	cgd2_2000	TTGATGATTCTCGATGTTCCTG	TCCTTTGAGGCCAGAGTGTAGT
5	cgd2_2020	AATCCTACATTAGTCGATGGGTATG	TGGAATCTCTTCGTCTCTCCTC
6	cgd2_1280	CCTTCGTCACTGGTTGTAGAGA	GTTTCTTCTTGTTTGCGATTCA
6	cgd2_1550	GAATCCCTCAGCATAATTCAACA	TCTTCTGCATGGTCGAGTCTAA
6	cgd2_1780	AATTCACAGTCCTCCCAAGAAA	TTCTGCACCCTTTCGATAAACT
6	cgd2_1820	CCGGAGGCTAGACAACGTAA	GGAATGAAGTAACGATGGGTGT
6	cgd2_1840	TGGAAGAAGACGAAAGGCATAG	TTGATCTTGTGGGATCTAAGGG

6	cgd2_2040	GACTTTCCTTCGAGAGTTGGG	TCTTGTTGTAGAAGCTCGTCCTC
6	cgd2_2060	TTCAATTAAGCGGGAGTCAAA	GTTGTATTTCCCAATATCGCAAG
6	cgd2_2100	AAGTTCCCTAACACTGCTCCTG	AGCATCTGGGCGTATAACAACT
6	cgd2_2130	CTTTGGTCTGGTAGAGGGAGAA	ATTTGAATTTGAGCCATCACCT
6	cgd2_2140	CAACTACTTCCAAACCAATCCC	TTAATAGTGGCATTTCGGATGA
6	cgd2_2160	CTAAATTGGGACTTGGAATTGG	GTTTGTTCATTTCATCAGTGTGG
7	cgd2_2210	TGCGCTACAACCAGATAACAAG	TGCCTTAGACCTGACTATTCTCAAA
7	cgd2_2220	TATTTGATGGGCCTTTGTCTTT	TGTTTGATGTGTGTAGCATTGATT
7	cgd2_2230	ATTGGTTCAAGAAGAGACGAGG	TGAAATGCTACTTCCTCCCAG
7	cgd2_2260	CCAGTCCCTGAAATAGGAAACA	TTGTCGTTCCATCCACCTTT
7	cgd2_2270	ACTATTTGAGTGGTGGCTTGGT	CTTGAATGCGTTTCACTTTGAG
7	cgd2_2280	TTTATCCATAGAAGAGCAGGGAGA	CAAAGGAAAGTCCGAGATTTGA
7	cgd2_2290	GTGAAGCCAATTTATATCCCAATC	CAACTATTTCGCTCAACCTTATCC
7	cgd2_2320	GGGACTGCACAAGTAATGTCAA	ATCTCTTCCTGCCCAGATAATG
7	cgd2_2340	GCAGGAGCAGGAGTATTTCAGT	TGGCAATCTTCAATTATACAGCC
7	cgd2_2350	CTTCTAGCGAAGCCAATTCAGT	AGGCGATAATAAATTCAACCCA
7	cgd2_2360	TATACAAGCTATGGAAACGCCA	ATTAAGGCAACTAATCGCTCCA
7	cgd2_2380	TTAGTAGTGCTTTATTCAGCGATGTT	TTCAGTACAATGCGTAGATGGTG
8	cgd2_2390	AAGTAAAGCAAGCAAACTAACACCT	AGCAATCATCAAAGTATGGCTG
8	cgd2_2410	CTCTCCATTGATAACTCCTTGTGA	AATGCGTGTTTCTTGGTATTGA
8	cgd2_2430	TCGAACCCAAAGATTGCTAAA	GATGTTGAAGTCTGCCTCCTG
8	cgd2_2450	GTTCATTTCTTGCTCCACACAG	AGAAGTATCAGCATTTGACCCAC
8	cgd2_2460	GCATGATCTCCTTTCCAATTCA	CGTCATCACCTAAATTCTTATTCTTCA
8	cgd2_2480	CAAATGATGACACAAGAATGGAG	TTTCGTTTAGGAAGGGATTGTC
8	cgd2_2490	TCGTCTAAACAAAGACTAAGCGTC	CATAATCCGCAAATACAAGTGG
8	cgd2_2500	AATCGGCATAGATTTGGATATTG	TCTCCGTTACTTATTAGCATCCCT
8	cgd2_2510	TTCGTAAACCCAATCATGTCAA	AATGTGCCTTCCACAGTTCTTC
8	cgd2_2530	CGCCCTATATCCCAGTAGTTGT	TTTCATTGCCAGTTTCTGATGT
8	cgd2_2540	GTGTTATATCGAAGAGTTATTGCCC	CCACTTCCAGTTAGCTCCCTAC
8	cgd2_2580	TTCACATACCACCTGTCTTTGG	TCAACCATTATCCCAACCCTAC
9	cgd2_2590	AAAGAGTGGGAATTGTATGGAAGA	GCAAGTAGCAACGAAGAACCA
9	cgd2_2610	TATCAGCCCATTCAACTAACCC	ACATTAGCCAAGATCGCAGTTT
9	cgd2_2620	ATGAAGGTGAAGTGGATGTGG	CAAATTCGGTATGCCTGTAAATAA
9	cgd2_2630	TTTGCTGTCTTTGAAGGGTCTC	ATGAGCCGCACTTATTCATGTT
9	cgd2_2650	TGGATACTCAATCGTTCCTATTACTG	ATTTGTGATGTGTGCTTTGCTC
9	cgd2_2660	GGTTTCTCTCCGTTCGTATTGT	AACGCGAATCTAGGAGTCATTT
9	cgd2_2670	GCGAAGCATTCAAAGAGAGAA	CTTGCTGGATAAGTAACGCTCA
9	cgd2_2680	GTTTAATTGGCATGAAGCTGTG	GATCCTGGGAAGCTGATAGAGA
9	cgd2_2690	TTTCGAGTCTTCTACACTGATTATGG	CCTAGCTTCTTCCAATTTACTGGTT
9	cgd2_2700	CCAAAGAATCAGAGGAACGAAG	AAAGGTTGAAATTGTGGCAAAG
9	cgd2_2710	GGTACAGCAGAAAGCATATTACCA	TTAACAACGCTTGAATGTAGCC
9	cgd2_2720	AGATTATTGCTTTGTGCCTTCTG	ATTCTTAATTGCGATCTTTGCC
10	cgd2_2730	CTGAATCTAATATGGCGTCGGT	CTTTCCCGTTCGAGTGTGTATT
10	cgd2_2750	ATGGCTGCGTTAGCACTACTTT	TTCAGGAATTTGGCTATGGACT
10	cgd2_2760	TGTGAAAGAGCGTATTGGACAC	ATGCTGAATGACAACTTCCCTT
10	cgd2_2770	TGCAATCATCAAAGGAACAGAG	ATTATGCAGGCAACTGAATGTG
10	cgd2_2780	TGTTTGCTGGGATTGATAAGTG	TTTAATGGTGGAAATCTCGTCTG
10	cgd2_2790	GAGGAAAGAATTGAAAGGCAAA	ATGGGTCGTAAACAAACAGGTC
10	cgd2_2800	GAAAGCATTGAACTAGAGACAAAGAAG	CCAAAGCAGATGGCTTATCATT
10	cgd2_2810	TTATTCCGCAGTGAAGATGAGTT	AATTCCTTCCTCCTTTCGTTTC
10	cgd2_2840	CCTGAGCCTTTAATCACCAATC	AAATTCATTCACGCAAGTTTCA
10	cgd2_2860	AAACCAGTCTTTCGAGGCTTAGT	ACAATTAACCAATCGCCTTCC
10	cgd2_2880	TTCCGTAGTTTGGAGAGATCAAG	GCATTGGAAGGAGAGATAGGAG
10	cgd2_2890	TGCGACATATTTAACACTTAGACGA	AAACTGACAAAGCAATCCCAAG
11	cgd2_2930	CATTGGAAACAGAGTGGTTGG	TACAAGAGCATAGGCAGGAACA
11	cgd2_2940	TCTAGTAACCAAAGGAGGGCAG	CCACCTATTCCCAGAGAGTTTG
11	cgd2_2960	CTCAGAATGACAGAGATGCTCAA	GATCCGCCAAAGGTTTATAGTG
11	cgd2_2970	CGTCCAATTAAGGGAGAAGTTG	TTTCGGGTAAAGCTGACTGG
11	cgd2_2980	CCCACAAGCAGTCTTACAACAA	TATGAAGGAAATGGAAAGGGAA

11	cgd2_2990	GGCTTTAGACCTGTTGGAATGT	CATTGATTTGGAATGTCTCTTCA
11	cgd2_3000	TGCAGAGAAGGAAAGGGATTAG	ATGTGTAACCACCACCTGACAC
11	cgd2_3010	GGAGATAAAGGAACGGGAAAGT	CTCAGCTCTTGACTTCTTGCTG
11	cgd2_3020	TTAACGCAGTTTCATCGTGTG	GACTCCAAATCCTCATCACTGTC
11	cgd2_3030	TAACTCTGATGACGAACCACCA	GCCTCTACTGCTCTTTCTTGTCC
11	cgd2_3040	CTTCTGCATCAACTCTTGGAAA	CATGACACTGGAATACTCGAAACT
11	cgd2_3050	TGCATTCAAGTGGAGGTCAAA	AAGGTTCTACACCCACGCAAG
12	cgd2_3060	GTGTATCTGTGCTTTCCTTGCATT	GCATGATCTCGGGAATACCTCT
12	cgd2_3070	GAAAGGCACAAGAATTTAAGCAA	TGTTGAGCAGATGGAGAAGGT
12	cgd2_3080	TAAGCGCATCTCCTCTAATTCC	CCTGAACCTGCTCCAAATCTAC
12	cgd2_3090	GCTCAGGAACAGCCTTTGTAGT	GAAACACTATCGCCAACAACCT
12	cgd2_3140	AATACTTCCACAACAACAACAACAA	TCACTTATTCCACCACTACTTGGA
12	cgd2_3170	GGAGACGAATACGAAAGAACCA	TCTGCATTCTCACACATCTTCC
12	cgd2_3180	ATGGGAGCCAACTCATTCTTT	TAGTCCTTCTTAGCCCTGCTTG
12	cgd2_3190	AAGCAAGAGTACGGACCAGAAG	TGAATGAATGAATCCAACCAAA
12	cgd2_3200	TCCTATTACTCTTTCCTCCGGTC	AATGCCTCATAGACAAACTCAGG
12	cgd2_3210	CTTTCTGACTTCCCATCGGTT	TCATTATCAAAGTTACGGATTGTGTT
12	cgd2_3220	CCAGGAATAGACAAAGAGGTGG	GGCTTTAGTTCTCCAACAATGAA
12	cgd2_3230	ATAACTGCCTCTGGAGAATGGA	GAGCAGAACCCTTTGGAAGTAA
13	cgd2_3130	ATGGGAATTGATTGCTTCTGTT	AGGACTTGTTGAGCATCTTGGT
13	cgd2_3240	CCATGTGAATGAAAGAGGGATT	ATTGACCCATTGAGCAGAGTTT
13	cgd2_3250	GCACAACAAGCATTTAGCAGAG	AGTGAAGAATCCCACATCATCC
13	cgd2_3260	GTGGTATTGATGAGGACTTTGGA	ACTTCGACGGTAAATTGATTGG
13	cgd2_3330	CAGTCGATTTCCAGAGAGGAGT	TTCTTCCCAAATGATCCAAAGT
13	cgd2_3340	GAACAGGAACAGGAACAGGAAC	GTCTAATTGCCGATGGGATAAA
13	cgd2_3360	TCAAGGAGATTTGGAAGAGGTAGA	TCATGTCTCACGACTAGAATCCC
13	cgd2_3370	GAGCTGATTGACACTTGTAGAGAAA	TGTTCTTTGTTGAAATTGGGAG
13	cgd2_3400	TCCTATCAAACTGGCCTAATGAA	TGCGCCTTCAGATACAAATAAA
13	cgd2_3430	AGCTAAGTGTTGTGTGGGTTCA	CTTCTGCTGCCAATTCTCTCTT
13	cgd2_3440	AACTTTCCTTGGATTGACCACA	ACGACGAAGAAGATGAAGAGGA
13	cgd2_3450	ACGTGTTATATGAATGCTGGGTT	TGATATGGAGAAATTGCATCTGTT
14	cgd2_3460	AGCCATAAACCAGATATGCCC	CAGACTTGCTTCCTTTGGAGTT
14	cgd2_3480	TTTATGGTGCAGAAGGCTCTAA	CAGGAAACCACATCAAAGAATG
14	cgd2_3510	GAATGCAACCTAATTGGCCTC	GGTCTAAATCCAAATTCACAACCA
14	cgd2_3530	AGGACTAGAAGCAGGACTGGG	CGACTCTGATTGCACTTGTTCT
14	cgd2_3540	ATCAACCAGCACATCATTCTTG	TTCAAACAAACATCACAAAGCC
14	cgd2_3550	ACTTCTGCTTCCACTTCTGCTT	AACTTGTTGTTGTTGTTGCTGG
14	cgd2_3580	TTTATTGGGAGTAAGGGATGCT	TCATCTTCTTCACCATCTGACC
14	cgd2_3590	ATTTGGGTCTTTCTTCGATTCA	CACCTTCTTCATCCCATTCTGT
14	cgd2_3600	TGATCCACTTAACCTTTCTCCAA	GGTCTGATCTAATAATGCTCTGAATG
14	cgd2_3610	GAAACGAGTGTAGCTGAAGGAAA	GATGCCCTCTAACCGAAAGTAA
14	cgd2_3620	TTTATTTCTTACGCCAGCAACC	AGCTCCAAATCTAATCTCGCCT
14	cgd2_3630	TAACTGGATGAAGGAACGCC	TCTCCTCTATATTTCGCTCGCT
15	cgd2_3640	GGGTAGCTTTGGATTTGAGTTG	CTTGAATAAGATGGACGCGAA
15	cgd2_3650	GGCGATTCTACAGACGAGGTAA	TATGTGCTGAAGGAACCCAAA
15	cgd2_3660	ACTCCATCAGGCCAACAATAAC	CATTTCCCATTCCTTGATCCT
15	cgd2_3670	TCTCCACAAAGAACGTCAAGAA	AAATCAACATCTCCAACAGTGAAA
15	cgd2_3680	CTTGAGAAAGAGGGTGAACTGG	GGACACAAATAAAGATCCAAGCA
15	cgd2_3690	TATGAATTTGGATGGCCTCTTT	ATGAAATCTTCTCCGATGCTGT
15	cgd2_3750	ATCAAGGCCATCAACTCTTTGT	TCACTGCTTTCATTTCCATCC
15	cgd2_3770	GGTTGTGAAGAGTTAGTTATGATGGTT	ATTTGTTCCACCTCGGCTACT
15	cgd2_3780	ACAACCACCAGCATCTCATTC	ATGTATCATCCCTTCCACCAAA
15	cgd2_3810	ATTGAGATTGATGGAGATTATTACGA	TAAGGGCATTTATTCATTGGGT
15	cgd2_3850	TCCTTCCTCTTCTTCAGCACTT	TCATGCTCTTCAACTTTGCATT
15	cgd2_3860	TGGTGATAGATTTCCGATACCC	CCTGCTGATAATGCTAATGCTG
16	cgd2_3870	AGGATCAAGTTCAAGCTCAAGG	TTTCCCGCATAATCTTCTTGTT
16	cgd2_3900	AACAACTACGACAACAACTTCAGG	TGGATTATTCTTGGTCATTTGAGA
16	cgd2_3930	TTTAGGGAGTACATTTCTTGGTGA	AGGTGTCCAGTTTATGGAATCTTT
16	cgd2_3950	TCAACTGATGCAGGAAGAAAGA	TGTTGGAAATCACAGAATCCAC

16	cgd2_3960	TGAATTTGCCAGACTTTATCCA	TATTGGCTCGGTTCCATTTATT
16	cgd2_3980	CTAGCCCTGCCTCCATTAGTC	GCTATCATTCTGCTCCATTTCC
16	cgd2_3990	TTGTTGTCTTTGGAGTTGATGTG	ATGCTTCTGTGATTGCATCTTG
16	cgd2_4000	ATATCCAGCGTATGCCGTTC	TGCAAACATGAAAGTAGAATAAACCT
16	cgd2_4030	ATTTCTCATGCTTCAATGGGTT	AAATTACCAATCAATGTTCCCAAG
16	cgd2_4050	TTCAGCTTCTTGTGCATTACCA	GATCCATCCATCCATTTCATTC
16	cgd2_4060	TGAGAAGGCATTACCACATGAA	GGGACAACAATACATAAAGGCAAG
16	cgd2_4070	GAGATCCAGAGGAGCAGAAGAA	GCACACTCAGAAATCACTCACC
17	cgd2_4090	CCAAGCCTCCAAACAAAGATAC	CTGTCTACTTCTTCATCGCTGCT
17	cgd2_4110	GAAAGCTCTTGTTGCCACTCTT	GATTTCCAGTGATTGCTTGTTG
17	cgd2_4130	AGAGTCGTTTAAGGCGTCCA	AGCTTCTGCTGCTTTCACAAC
17	cgd2_4150	TTCTTATGCAGCTTTCCCACTT	AGGTTCTGTGGAGGTTGTTGAG
17	cgd2_4160	TGAAATAGGATGGAATGCAAGTAA	TTAAAGTATCAACAGCGACAGGAC
17	cgd2_4180	CACCACCATCTGTAGTATTGCC	CTGTTGATGCAAATCTGACCTT
17	cgd2_4190	AAGTTACAAGGCGAGGGAAGA	GGTAAACTACTGCATCCAACCAA
17	cgd2_4200	TGGCAATCTATCATCCTCTCTGT	TAATCGTCTTGGCTGTACCCTT
17	cgd2_4220	AGTTTGTTGAGGAGGTAATGGG	CCAAAGTTGATTGGCTTCATC
17	cgd2_4240	GTCAGGAATTAGGCCAGTCATC	CCAGCAATAGCATCATACCAAA
17	cgd2_4250	CAAATCTTGGTATTGCGATTGA	TCAGCCAGTTCATTTATGCTTG
17	cgd2_4260	CATTGCTAATCCAACAACAGGA	TCACCCAAGAAGCTACAGTCAA
18	cgd2_4270	GCTCACTTCCTAACTCCACCAG	ACCATCTTGCCACTCTGTTCTT
18	cgd2_4280	GAATCTTGGAATTTGCGTAGGA	TTGTTGAATACTCCATTGCCTCT
18	cgd2_4300	AAGTTTGTTGATGGGATTCAGG	CAATTTACTACGCCCAGAGCA
18	cgd2_4320	GAGTTGAAGTTTCGGATTCTGG	CAACAGGAGTTAGTTCGGGTCT
18	cgd2_4330	GGACTTCTCTGACATTCCTTTGAC	ATTGAGCTGATCCCATTTGAAG
18	cgd2_4340	TCATCTCATTCAGGCTCTTCAC	TACAATCGACCAAATCCTCAAA
18	cgd2_4360	CACGATTGTAGCAACCAGTCTAA	TCCAGAGTTCCCAATTAAGTCC
18	cgd2_4380	ATTCCAGGTGAGCATCGAGTAG	TGTTGTTGTTGTTCGTCTTGGT
18	cgd3_30	GCCATTATATTTGGAGGCGA	TTTAACCAACCCTGCACAAAG
18	cgd3_40	TAACGCCCATTTAGGGATAGAA	AAAGTCTCAACGGAAGTACCCA
18	cgd3_50	AAAGGCCATCATTGGTATTGAC	TTGCGAGCTACAGCATTTCTT
18	cgd3_70	AAGTCAGGCAAAGAACACAAATC	TTTGAAGAAATTAAGGGAGGAGAA
19	cgd3_80	TTTACTCAACTCAAGATCACGCA	CTTTATCATTCGGGTTTCTCCA
19	cgd3_90	ACGAAATTAAGGAAATACCGCA	CTTCATCAGTTGGTTCAGCTTCT
19	cgd3_100	TGTTTCCGAGTTTAGTCAAGCA	AAAGATCCCAACCAGGAGAAA
19	cgd3_110	ATGTGGTAACTGAGCTGATGGA	CCAAACCTATCCCTTCCAAATAC
19	cgd3_140	ATCAGAAGATGACGAGTTGGCT	TTTGGATGACTGAATATGGCAC
19	cgd3_160	CCAAATACTCAACCTCAGATCCA	AGGTGTTGCTTTAGAGTGGGAA
19	cgd3_190	AAATGTTTACCAAATGGGATGG	ACTCTGTTGTTGTTGTGGTTGG
19	cgd3_210	AATTAGATATGCCCGACTCCAA	TTTCCAAGATTTCAGGTGTCAGT
19	cgd3_220	AACTCAAACCTCACCAAACACA	CCTGAACTAACCTGCTCATATCC
19	cgd3_250	AGAGAGTGCCATTTCCAAGAGT	CTATCCAGTCCCATCTTCCATC
19	cgd3_260	TAAGGATGGAGAGGAATCGAAA	TTTGAATTGGAAGGTAGCTGGT
19	cgd3_270	GGTGGAGGATATGCAGAAGAAG	TTATTGAACTGGTTGGACGAAGT
20	cgd3_280	GGTCAGTAAGCTCAGTTCGTCA	TGGTCTTCTTGGATTGGTTCTT
20	cgd3_300	ATACTTCATCGGCATCATCTCC	TTCCTGCATAGCTCGTGACTT
20	cgd3_310	GGCATCAGGAGAGGTTGTATCT	ATTCTTTGCGTCATTCCACTTT
20	cgd3_330	GTGGAGGAGCTGTTAAAGGAAA	AATGCACCACTGCGTCTATCT
20	cgd3_340	GCCATTCTTATTCTTCAGCAACTC	CAGATGAGCCTGGTAAACACCT
20	cgd3_350	TTTCCAGCCTTAGTCACCTTTC	CTTGGGAGTTGAGATGGAGTTT
20	cgd3_380	TATGGCTACATCTCCACTCCCT	AGCATCTGGGCTACTTCTTCTG
20	cgd3_390	TGGGAATTATAGATGGGAGTTCA	TCTTCACTTTCCTCCGTCTTCT
20	cgd3_400	ACTCTATGAATTGTTCCCGTCC	TTTCTGATTTGGATCTCACCTTC
20	cgd3_410	TTCGCAAGAGGAAATAAACGA	TCCCACATCAATATAGCCAGAA
20	cgd3_420	GAGAATATAGGTCTGGAAGCGG	AGAAATCAAGCAACCCTCAACT
20	cgd3_430	ATCCAGATGAGGAGAGGTTTCA	TGTACGTTGCAGATTGAGCTTT
21	cgd3_440	CTACGAGAACAACCACAACCAC	ATAGGCGCTAGTCCTCACATTC
21	cgd3_450	AGCCTTCCCACTTTATTCCTCT	GAAGATTCGATTTGTTCATTTGG
21	cgd3_460	TTGAAAGGGAAAGTCGTAATGG	ATCTCAAGTCCAAGAGGAGCAA

21	cgd3_470	CGTCGATTTATCCCTTGTTTGT	TTAGGTTTGGACGCCAGTAATC
21	cgd3_480	CCCAGAGAGGATGAGAAACTTG	TTATTGGATTCTTCCATTTGCC
21	cgd3_500	CATTGGGTTCACTAAAGCAGGT	GCGGAGCATCTAGTCAGTCTTC
21	cgd3_510	TATGTGTCTCAAGGATGCCAAG	CACCACCAATAATAACGAAGCA
21	cgd3_520	ACCCAAACGGAAATAGAGAACA	GAATTGCCAGAAGGATTGAGAG
21	cgd3_530	GCTTGATTGTAGCTCTTTGGCT	TGCTGTTTGAGAGTTGGAGAAA
21	cgd3_560	TATCAATAGTCAAAGCTGCCGA	TCAACCGAGTTTATGCCTTCTT
21	cgd3_570	TTGTACTTGTCTCAGCCTCTTCTG	TCTTGGAAGGAATGAATGGTTT
21	cgd3_580	TTGGACATTAACTTTGGTTCTCC	ACCTGTTCTCTACCCATCATCAG
22	cgd3_590	TATACCGTCAGGAAATGAGCAA	CACAGAAGAGACAAATCACGCT
22	cgd3_600	GGCTCCATATCAAGTTTCTGCT	CTACGTCCAAACCACTCCTCTC
22	cgd3_610	CCATACCACAATGGAAGGAACT	AATGTTAATGCAAAGAAGAGCCA
22	cgd3_620	TTCTCTGTCTTCTTTCCTTCCTCTAC	TGAGATATACAACCCAATACAAGGG
22	cgd3_630	AATAGGAGAATCATGCAGTGGAA	TTTGCCTTAGAAATTGAGGAAGAC
22	cgd3_640	AGTAATGGTTGGTTAGATACTGGTGA	ATTCCCTCAATTCTTTCTGGTG
22	cgd3_660	TATCTGGAGGGAGCTTTGAGAC	ATCGAATGCAGCTAGAACAACA
22	cgd3_670	GGATGGGTTTAGAGGAAGAGGT	TGGAGTTGAGCTTGGGTCTATT
22	cgd3_680	GCAAATAGGAAGAATGGAGGAA	ACCCTTTACTTTGAAGCCAACA
22	cgd3_710	CGCCTTATTCGGGTTTAGAAG	CTGACCTTTCCACCTCTATTTCA
22	cgd3_720	TGTGTAATCCAATTCAAGCAGG	ACCATATCCTCCAACACCATTC
22	cgd3_730	TAGGACTGATGTGGTGGACAAG	TTGGATAAACTCTGAAAGCAACAG
23	cgd3_740	AATCCAGGCGCAGAATTTAGT	GTGAGAAACCATCCAATATCCAA
23	cgd3_750	CTCCTACAGCTTCATTGGGAAC	TGTCCGTAGCTCTGTTGCTCT
23	cgd3_760	GGCTTGTTCTAATTGCCTGTG	ACTGAACTCTCGCTGTTGAATG
23	cgd3_770	GGGTTAAGAGGGTTTCTACTCCA	GATGGCAAATAACAATCAACCA
23	cgd3_790	AAAGTGAAGAGGACGCCAAA	TTCGTTCCAAATGTCAAAGAGA
23	cgd3_800	TGGGACAAATGGAGCTATAAGAA	GATTCCTTGGACATTCACACAA
23	cgd3_810	ACATGCTCTGTTCCATTCTCAA	AAATTCGGCTCCTTTCTTACCT
23	cgd3_850	AAGTGAGACAGGATCAACACCTC	GCTCGACATAATCAAGGAAACC
23	cgd3_860	CCAGAATGCTTGGAGAAGATG	TGATTCCGCTAAGTTGGAGTTT
23	cgd3_880	GAGTTCCAAGTTTCTTCCAGCA	AAGGACGTGATGGTTGAGTTTC
23	cgd3_890	TGTTCCCTTTATTTCTTGTGGAG	AGGTGCATTTATTGGAGGTTGT
23	cgd3_930	GGCTTGCATTCTTGAGGTTAGT	TCCTTGTTCTCCATTATTCGCT
24	cgd3_950	TGAAGCTGATTTGGGCTACTTT	CATGGTCTTGATCTTCTTTCTGG
24	cgd3_960	CCTGAAGAAGAAATTAAACGCC	AATTGAGGATGGTGACAGTGATT
24	cgd3_970	GCAACAAACAGAATGATGGAAA	CCAACCAAACATGAAAGGAGA
24	cgd3_980	CTGCGTTGTAGCAGTTGGTG	CAATAGCTGATGATGGGTTTCC
24	cgd3_1010	AAGATTGCTTGAGAGGAGCAGT	TTCGTATTCACTTATTGGCTTTGA
24	cgd3_1030	GTCATCTTCGTCATCTTCGTCA	TACGCTGTTGTTTGCCATATTC
24	cgd3_1060	AATGGAGTTTACGGACAAGGAA	CTTGATGGGAAGTTGTATTGGAG
24	cgd3_1070	GCATTAGATGGACAAGCTGAAGT	AAACCAAGCAACCAAGGATAAG
24	cgd3_1160	CAAGGTCAAGATCGGAATCAA	GATGATAAAGGTCCACTAGAACCAA
24	cgd3_1200	CATCAACTAGCAATGAGCATGAA	ATAACTGCGTCACTCTCCAACA
24	cgd3_1210	CATTCTTGATAAGGCTGTTGGTAA	GTCCAAGCTCATAAACTGAAATGA
24	cgd3_1220	GAAACCAAAGAGAAGGAGAGCC	ATTTGAACCATCATCACGAACA
25	cgd3_1230	TCAGTCGAAGATTACGAGCAAA	AAGCCTCATCAGAAAGAAACTCC
25	cgd3_1250	TGAATCGTATCTTGATTGACGG	TCTAGCACCACGAGGAATCTTT
25	cgd3_1270	CTCGTCAAGTTCATCTCTGGG	TTGCTGTTCTGTTAGTTCTGTTCTTT
25	cgd3_1280	TACATCAATTTCCAGACCCTCA	AATGCCTCAACTTTGTTCTCATC
25	cgd3_1290	CCAGCAAGATGTGTGGAAAGT	CTTGGAGTCACCTGTGGAGAA
25	cgd3_1300	ACCAAGTTATTTCTGTTGCTCGT	CTCAATCTCACCTGCATCAATC
25	cgd3_1310	ATCAACGAACTGGGATTTAGAGA	TTGTTTCCTGTTGCTCATTCA
25	cgd3_1320	GGTATTTGGATTTGAATGAGGG	ATAAAGAGCTTGGTAGCAGAAGAAA
25	cgd3_1330	AAGGATGACAAGAAAGATGACGA	CACTACCTGACGATTCAGAGGTT
25	cgd3_1340	GAAGCTCGTTTATCCCATTGTT	TCTCTGCAAACTTCAACCTCTG
25	cgd3_1350	ATCAGCAAATCAGCTACGAACA	TGGAGGTAGAGAGGACGAAGAG
25	cgd3_1360	GGTGGGAGAATATGAGGGAAA	TCTTGAGGCAAATCTTTCGTG
26	cgd3_1370	TCGTAGACCAACAAGAAACGAA	AAACCATTGTATTCCCAACTGTCT
26	cgd3_1380	CAACATTGAGAGGGAAATCCTG	AAATCTCCTGAACGCTCCAAC

26	cgd3_1390	GATTCGCGTTTGTGGAGTTT	GCTCAGAAGTATCGCTTTGGTT
26	cgd3_1400	AGAATGGAGGATAGATGCGAGA	CTTGGAAGCTGTTGAAAGAAGG
26	cgd3_1420	GATTGTGGTGGTCTTGCTGTC	TGGGCTAAGTCTATCTCCAATAACC
26	cgd3_1440	GGGAAATGGGAAGAAGTTAAAGA	TCCTTCAATAGGCTTAAATGGG
26	cgd3_1490	TGGAAGATGGAGATTGTTGTTG	TCATTCAGCTTGTCCTTGATTG
26	cgd3_1510	GAGGTCCACTCAAGCACAGTAG	ACCATAAGTGCCTTCTCCAATC
26	cgd3_1520	CCCTGAGAGTTGTGTAATAGAATGG	TCCCAAATAAAGTAATCATAGTCGTTG
26	cgd3_1540	TCCCAGTAGCAGGAGGTTATGT	TGGCTTAACTCACTCCCAAAGT
26	cgd3_1560	GGAATACTTACGCATGGATGGA	TGGGAGAAGGAATTACACCTGA
26	cgd3_1570	CTGAATGGGATGATATGGTCAA	GATCAGCAGAAGCAGCATAGAA
27	cgd3_1590	CACAAGGATGTTGCAGTTGAAG	GGTGTTAATATGAGCGAACCCA
27	cgd3_1650	TGTTAGGGCAGTTTCTGGAGTT	ACGATAGCATTTAGGAGGTGGA
27	cgd3_1660	AAGTTGTCCAAGGTCCCTCA	GGGCATTTCATTGTTACGTTC
27	cgd3_1690	CAGCTATTATTGTGGATGCTGG	TTCGTCTGATAACCCAATTTCA
27	cgd3_1710	TTTCTTCTGTTCTTTGGCATTTC	CATTTATTCTGGCATCCTTTCTG
27	cgd3_1730	CTGTAGCTCTGCCTCAACAAGA	CGGATAAATCTAAACCAACTCCA
27	cgd3_1740	TCCAAACATAAAGGGAGCAAGT	TCTTCATCCTCATCCTCATCCT
27	cgd3_1750	CAGACGATGAAGAACTTTCACTACC	TGACTCTTTGCTGGTCAAATCA
27	cgd3_1780	AGGAATCTCCAGACCAAGCTAA	TACAGGTTCAGGTTCAGGTTCA
27	cgd3_1790	CATTTGGAGCATGGAATAGACA	CAGGGAAGAAGCGTTATTGAAG
27	cgd3_1810	ACTGCTACGGGTGCTATTCTTC	GATAAACATTCTGCCCACCTTC
27	cgd3_1830	AAGCAGTGGATCGAAGGATAAA	ACTCTCTCATTTGGGCTTGTGT
28	cgd3_1840	AACTTTGATTTGGCAGGTGATT	ATTGTGAGAGCCAGGAAGAGAG
28	cgd3_1850	TCCTGTTGGGATTACGAATGA	AAAGATGTGCCAAAGAAAGCAC
28	cgd3_1860	GAGTAAGGGCTAGTGGAGCAGA	AATTGGGCATTAAACAGCAAAG
28	cgd3_1870	CTTTAGTTATGCCGATCCAACC	GTGACCACATCCTCTACAATTCC
28	cgd3_1880	GATGTTGGCTTAACTGACTCCC	CTGTTTCATGTTGATTTCCCAA
28	cgd3_1890	GAGCTGTTTGCTTTATGATCCC	CTCCGGGTTTGAGAAATCTATG
28	cgd3_1900	CAAGGTTGTTAGCTCAGATGGA	TTTCTCCAAAGCAATAAGTCACC
28	cgd3_1910	AGGAGCATGTTAATTCAAGGAAA	TTGTTCCAAACGATCAAATCA
28	cgd3_1920	GATCGTGTGTTCTCAAGCATTT	ATGGAGTTGCCTTTCTATCACC
28	cgd3_1940	AAAGGCTGAGTTTGACGACATT	AGCTTCTCCCTCCTCGGTATT
28	cgd3_1950	AAACAGGGATAGGCCCACAG	TTATTCCAGGCTTCATATTGTATTTCC
28	cgd3_1960	ACGATAGGCAAGCTGATGAAA	AATGTGCGAATAACATCGACAG
29	cgd3_1980	TCGGAGTCAAGAAGGGTAATGT	TGTGCATCAAGAAGAGCAGTTT
29	cgd3_2000	TTATCCTGCTCCACAAAGAGGT	TCTCCAAAGCTCCCTTACAATC
29	cgd3_2010	TATTTGCTACCATGCAATCCAG	AAGATCAATTCCCGTTGAAGAG
29	cgd3_2030	TCCATGTGAAGAGCTACGAAAG	AAATTCCACAGAGTTTGGCTG
29	cgd3_2040	GGCCAAGATAAGCAGAGAAAGA	TGGGAATTTGAACACAGAATCA
29	cgd3_2050	ACTCGCACTCTTTGGACAGTTT	TCTACCTGCTCTTGGCTGTACTC
29	cgd3_2060	CTCAAGTAACAACCCAACGTCA	AATTCACCACTAAGCCCTACCA
29	cgd3_2070	AAGAGGGTTCAGAGAAGATTTGG	GGTGATTAGGTCCAACAGGGT
29	cgd3_2090	ATAACTTGGCGGATGTAGTCGT	CTGTAACGATCAAGAGACGTGG
29	cgd3_2100	CCTGGGTAGCACGATTGTTT	AACACCTTTCCTATTATTTGCCC
29	cgd3_2120	CAAACACCTGGAGAAGGTAACA	AAACATCCATCTGAAGCAACAA
29	cgd3_2130	AGGGCTCGAAACATGAACTAAA	TTAATCTGGATGTCGAATGGG
30	cgd3_1800	CCTGGGCTAACTCTTGCATTAC	CAAGTCTGAGGTCATTCCCAA
30	cgd3_2140	GCATTAAAGTTGTTGGGATTGG	GCTAGGAGTATTTCGGGCTCTT
30	cgd3_2160	GCTAGTGGCTTCCCTGACATT	GTAGTGGCTCTGAAAGGCTGAC
30	cgd3_2170	AAAGAAAGTCCCAAATCAGCAA	GATCTACAATCAGGAGCCAACC
30	cgd3_2200	CCAAGTCGAATATGCAAACAAA	ACAAATCCAGCAACACAACAAC
30	cgd3_2210	ATACTCGGGAAGGACTCTCAAA	CCGCCTTTATCTCCAGAAACT
30	cgd3_2250	GAACTGCCTGTGGAATTTGG	CTCTGCCTCTTCTCTCTGCTTC
30	cgd3_2280	TGACATCTTTCCCATCTTTCAG	CAGAGGAGCATAATTCCACAGTT
30	cgd3_2300	AATCCTTTAATCCCGTGACTTTC	AAACAAACCAATATGGCAGGAG
30	cgd3_2310	TGGGAGACATGGAACAATTAGAC	AATGCTGTGCCCTTTGTATCTT
30	cgd3_2320	AGATGTACCAAAGTCGCAGGTT	CCTCAGCTATGTCTTCCCAGTT
30	cgd3_2330	GCTGGTTTCCCTGATCTTACAC	AAATACGCAGATTCCATTGACC
31	cgd3_2340	TTGATTGCTAATTCTAACCTTTCCA	TTCCCGAATAAATATCCAACCA

31	cgd3_2360	GTGTGTCGGTATATGGTGATGG	ATAAATCTTGCACCCATTTGCT
31	cgd3_2370	ATGTTGGCATCTCATATCCGT	GCTGTAAACAATGCGAGTCATC
31	cgd3_2400	TGAGAAAGAACTGCACTCACAAA	ATGCTGGAAACTTAATGCCCT
31	cgd3_2430	CATCCAAAGAATTAACAGAAGTCAAA	AATTACTGAGGTGATGCTACACGA
31	cgd3_2440	GCAGGGCAGTTATCTCAAGAAG	TTAAGACGCTCCAAATCCTCAC
31	cgd3_2450	GAAGCAACAAATATAAGTAAGGACGA	CTTCTAACACTAATTCAGGCCCA
31	cgd3_2460	TTGTTTGCACTTGGGTCAGTAG	CGTTAGCAAGCGGTCTATTCTT
31	cgd3_2470	TTGTTGATAATTGGGAGGATTTG	AGCTCTTGGCATCTTCTCTGTT
31	cgd3_2520	GACGTTTCGAGTCATTTGGATT	AAGATTGAAAGCGGTGAAGAAG
31	cgd3_2540	AGGTAAAGCACCTCGTAAGCAA	CGTACTAGCCTCTGGAATGGAA
31	cgd3_2560	CTTTGGGCAATTAGAAATCCTG	TCAACCTCAATCTCAAACATCCT
32	cgd3_2570	CTTGGTAGCGGAATTGGTATTT	GAATTAGATTTGGCATGTTCTTCA
32	cgd3_2650	CGAGTATTTGGAACCACCTCTG	ATTTCCGTTTCATTGGGAGAA
32	cgd3_2670	AGCCCTGTTCTTGATGTATGCT	TGAGATTCCTTTACGCATTTCTG
32	cgd3_2690	AACCGTGCTTGGAGAAGTTTAG	GAATTATCTCAATCTCACCGCC
32	cgd3_2770	CATGGGATTTATTTGATGACGA	TAACGAACATACAACCGAAGGA
32	cgd3_2780	AGCATATTACTCAATGGCACCC	TACTGCTCCGTATAATCCACCC
32	cgd3_2790	ATTCCCTGTTGATGAGGTGTTT	TTAATTGTTGTCGTTGCGTTTC
32	cgd3_2820	GGAAACTTGTGGGCATTGTT	CAGGAGTATATCTAAGGCAGCGA
32	cgd3_2850	TAAATGCAAATGAAATGCCAAA	CGAATGGTGAATAGCTTCCTGT
32	cgd3_2860	CGAAAGGTTTGGACTATTGGG	CAGAAACAGATTGATCCAGTACCAC
32	cgd3_2890	GGAATACGTGCTTGATTGGTCT	ACTACAGGCGGAAATCTTGTTG
32	cgd3_2900	CATGAATCTGTTACGTCATCCAA	AAGAGTGCCTCCAGAGCAAA
33	cgd3_2910	GCTTGTTAAGTGGCGTGAGAAC	TACGAGCTTCTGCTTCAGTGTG
33	cgd3_2930	AAATACAAACAAATCAACCGGAA	ATATCGTCTATGGAACCGGAAA
33	cgd3_2940	AAATAGCCGGGATTCATACTTG	AACTTCGCATCCTCTTTGTGA
33	cgd3_2980	TAGCTCAAACATAATCGGCCTT	TCCCAGTTGGTAATAGTGGCTT
33	cgd3_2990	GGTCGGTAGAACCAAAGACACT	CTTGACTGCTCCATCTCCATAA
33	cgd3_3010	TTTACCGAGGATTAGTTCTTTGTTG	CGCTGAGTCTTCATCTGACTTG
33	cgd3_3020	TTGAGATTGGGAATGAGAATGA	TGTTATGTTGTCTCTGCGATGTT
33	cgd3_3030	CAAACTCTCAACCTGCACAATC	ATGCTGGCGTTGTAAGACATAA
33	cgd3_3040	ATTACGAATGGATGGAATTTGG	GAAGTAGCAGGATAACGGGTGT
33	cgd3_3080	CAGAGGTTAAGCAGGGAACTTG	CAATTCTGGATCTCTTTGAGCTTT
33	cgd3_3100	GAACAACTACTTCCAGCATCCC	CATATTGGACAACTCAAGGCAA
33	cgd3_3110	TGCAATACTCTTCATCCCAGAA	TTCAGGAAATCCCATTGTAACC
34	cgd3_2700	TTTGGGAATTAGATTAGTTGAAAGGT	TGAGCCTATCTGCTTGTTTGAG
34	cgd3_2710	AGATCAAGTCAAACCACCACCT	GTACCATTTAAGCGGAGCTGTC
34	cgd3_2730	TGGCGGAAATGACTGTACTATG	TGAAGATGACAGAATTGGGAAA
34	cgd3_3130	ATGGTAGAGGCAGATAGCCAAG	AACCTTATTGGTACGCATTCATC
34	cgd3_3140	TTCTTCCTCAAATCCATCACCT	TCCGATGTTGTGTACTTCATTCTT
34	cgd3_3200	GCGATATTTACAGAAGCGCAA	ATGGATGCAAAGTTGAAGAACC
34	cgd3_3210	TCGTAAGCTCGGTTAGCTCAGT	AGTGCTATGAAGTGTTCCGTCC
34	cgd3_3240	AATCCAGGGAATCATCAACAGA	GGTCGCTTTGACCGAATACA
34	cgd3_3270	CATAGGCTCTACAGTGAATGGGA	TGGAAATCGAGTATTATGCCAAC
34	cgd3_3320	AATTAAACAAGACGAGGCAATGA	ATGTTGGAATCAGCAGGAATCT
34	cgd3_3330	CCTGCTTTCTGGTTGATACACA	TGTTGGTATATGGATGATGGGA
34	cgd3_3340	GAATAACCAGACGGAAGGTTTG	TACTGAGAGCTGGATGAGACGA
35	cgd3_3350	TTTAAGGTTTATTCGTGTCGGG	GGTTGAAAGGCGAAATGTTAGA
35	cgd3_3360	TGTTTACTTTGTCCCATCCTCC	CAATGTCGTTTGAGAGTTGTTCTT
35	cgd3_3370	TTTATGCAAAGATCCCTCCAAG	GAGTAAGAGACCGCAAATGGAA
35	cgd3_3380	GAAGATCACATCCGAAGAGGTT	GCTTGCTGAAATAGCCAGACTT
35	cgd3_3390	GAAGCAGCAATGTGTCTCAAAG	TCCAAACAAATATCCAAGCAAGT
35	cgd3_3400	AGAACTATAAGCCCACCACCAA	TCTTAACTTGGGATGAAACGAAA
35	cgd3_3410	AATTCATCCGGGTCAGTCATAG	AGAGAGAAGTCCGAAGAAAGCA
35	cgd3_3420	ATTCTGCTCTGCACCATTTCTC	GATCTTCTAAATCCCTGCTCCC
35	cgd3_3440	GAAGCAATCAAGAAGGAACAGG	AGCTGGCACAGTAATAACAGCA
35	cgd3_3470	TGTCAGGTATTGGAGATGAAGTTAGT	TCAGGGCTATCATTAAGAGGAAA
35	cgd3_3480	ACTTTATGGTTGCGAGGACATT	AAAGGTGGAGTAGAGCATTTGG
35	cgd3_3540	AAGCAGATTACAATGGATGAAGG	ACATTCTCGACATTTGTTTCAGTT

36	cgd3_3560	TCATGCAGATTCACTTGGCTAC	GCAATAGCTGAAACAAATCCAAC
36	cgd3_3570	TTTGGTGCAGAATGGTTGTAAG	TATATCCTTGCCCTTGTGGTTT
36	cgd3_3580	GGACTTATTGGCTTTCCTTCTG	CTACCTTTACCATCCTTGGCTC
36	cgd3_3590	ATCCAGCCTACTCTTTCTTAAACCA	GGATGTGTCCAAAGTATTTCATTCA
36	cgd3_3600	TTCTGCTGTAATGTTTGGAGGA	AGTTGTTAGGGAGTTTCTTTGCC
36	cgd3_3610	AGGGAGTGGGTTTGTAAAGTTG	AAGGACTATCTGTGTGTGAACCTG
36	cgd3_3680	ATTCGCTTGGATATGGATGACT	CCTGCTCCTGTTACTGTTCCTC
36	cgd3_3700	TTACAGAGGATGATTGGGTTAATG	TAGCAAGACATACCGGAGGAA
36	cgd3_3710	ACTCCTCACACAACGACTTCAC	CTCTTCCAATCAAACTCATCCA
36	cgd3_3740	TTGCCCATGTGTTATTGGTATT	GATTTGACAGTTGGTCTTCCATT
36	cgd3_3770	CAAACATCAAAGAGTGGTGAGG	GCTTCTCAAGGAATGGTGAGTT
36	cgd3_3800	CAAATAATAGTAACAACGCAACATCA	TCCATCAATAGAAGACGAAGCA
37	cgd3_3840	TCTTAGCATGGACAACAACACC	TCTGCTCCAATACAATCAGAAATG
37	cgd3_3860	GGAGAGCTGATGAATGAGTTGG	CTCTTCCGGTCGAATATCCTCT
37	cgd3_3880	CGGTATCGGAAGAAGATGATAAA	AGCACTTTGAATAGGATTTGGG
37	cgd3_3890	GGCCAAGAAGGGTTCATCTAA	ACCAAACGAGCAGTACCATTTC
37	cgd3_3900	CCTATGATGACTTCCAATGCTG	TATAGCTTACATCCGCCACTGA
37	cgd3_3910	TCAGAAACCAACTTTACAGCCA	GCTGAATTAGTATTGGAAGCGG
37	cgd3_3920	AAGAGGAGCTGGTGGTACTGG	AGCTGAACAGTGGAGATGATAGTTC
37	cgd3_3930	TAACAAGACTGACAAGGCTCCA	CCACTTAATACGCAGATACCACC
37	cgd3_3950	CCTACTTAACTCTTTAATGGAAATGGG	CTGGAATGCTATCAGAACCAGAA
37	cgd3_3960	GCTGGTCTGGAAGGAAGATACA	TCATTTGGTAGTTGATCTGACGAG
37	cgd3_3970	TGGTTTCCAACTTTACGTCTTCT	ACTTGTTCCATTCTTCCTCCTG
37	cgd3_3980	ATCTGAATGGCAAGAATGTGG	TCAAGTTCAAGCTCGGGTTC
38	cgd3_4010	GGGTAACTTTGGTGATATGGGT	TCTTACTCTGTGCTTCAGTCCAA
38	cgd3_4020	TGCACTTATCAAATTCCATTGTC	CTTCTCAACCTTGCTTCTGAGTT
38	cgd3_4050	TCAGACTAAACAATAAACACGGATTG	TGGGTTCATATCTTCGTACTTGG
38	cgd3_4060	TCTCTTGATTCTTTGGATTTGGA	GCGCATAATAACTGTAACCTTGACT
38	cgd3_4070	ACATTTCCAGCGTTGTTATTCC	ATTGACGATAGACCCATTCCTG
38	cgd3_4090	TTGTGAGCTTGATTCCACATTC	AATACACGACGAGGTTGAGGTT
38	cgd3_4180	GGAGGACAGTAAGCGAGGTAGA	TCCATAACAGATCCAACTCCAA
38	cgd3_4200	ATTTAGCATTTGAGCATTTGGG	GCGTCTCATCAATATCTTCCGT
38	cgd3_4250	TAACTCCATTAGACGCGGAGA	ACTCAACGCAATCTTTCTGGTT
38	cgd3_4270	ATGCCTCGAACCAAAGAACTAA	GAGTTGCCTTTCTATTTGCATTTC
38	cgd3_4280	AAATGATGAGGAACAGCAAACC	TATATCGAACGTGCAGAGAGGA
38	cgd3_4290	GACATTTAGTGGCATGGATGAA	GAAATAGGATCTGGGTGGTTAGG
39	cgd3_4330	GAAGAAAGCCGTATCTCAAAGAA	GTCAAAGAAACAGTGGTGATGG
39	cgd3_4340	GTATATGTGGGAATGACAGCGA	CCAGCATCAAACTCCAATGA
39	cgd3_4350	TATTTATGGGTTTGCGCTCC	GGTTTAAGGTGATTGCAGAAAGA
39	cgd4_10	TTTCTTTCTCACCACCACCTTC	CAACACTGATCTTCTGGGCTTT
39	cgd4_30	ATCGTCAACACTGGTTCCTCTT	ACCGCTCTTGCATTTATTTCC
39	cgd4_40	CTTAAACAATCCAATGAGGCAGT	TGGGCAAACATATCGTAGTCTC
39	cgd4_60	CATCTTCGATTGTGAGGCAA	CTTCTCCCAGTAGCTCAGTTTCA
39	cgd4_70	ATAAGAACATCCAGAGGCGAAA	TTGTGGAATAAGTTGACGAGGA
39	cgd4_80	CACCTCTTTGTTGCGTTCTTACT	TCCTTGGATGTTAGCTGGTTCT
39	cgd4_100	TCAATAGTTGCAGGTACGCTTAGT	TCCGTGTGAGTGGTTTATCTGT
39	cgd4_110	TTTATTGAGGAAATGGTGAGAAGTAGA	TAAAGCCTGGTTAAGCTCCAAA
39	cgd4_120	GCACCTCAAACTCATCTCCAA	TCCAGTATGTCCAGAATGTACCC
40	cgd4_130	ACAGAAATCAGTGCATCAGGAG	TTGTAACCAAGCTGTCCAAAGA
40	cgd4_150	CCGGTGAAGGTGGTAGAAGA	AGATAGAAGTGCTGCTTCCCAA
40	cgd4_160	TCAAGCCACTCTAATCAAAGCA	AACCTCTCCCAAATCCTCAACT
40	cgd4_190	ATTGAGAGACCAATTCAACGGA	TTCTTATCATTCGGGAGACCAA
40	cgd4_210	CTGGAGCCCTGCTTTAACTCT	TTCTTCCTTTGATGTTGGCTTT
40	cgd4_230	AGGTGAGGAAATCTCAAACGTAA	GTAACTGACGTAGAGCTGAAGGAA
40	cgd4_240	TCATTTGGAATTGTTTGTGAGG	TGGCTCTTCATCTCCTGTTGT
40	cgd4_250	CGAGGAACAGCCAGTAGAACA	TCCATCCCGAGAAATTACCA
40	cgd4_260	TGCTATCTGTGTGCTCGAAACT	TTAAATCTTGTTGCTGCCTGAA
40	cgd4_280	CAACTTCTCCCAATCCCAAG	TTGCTTGAACTCAGAAAGGAAA
40	cgd4_290	GAGTTATTATTGACGGCGAACC	TGTAAGGGTATTAGTGCAGTTTCAAG

40	cgd4_300	TTTGATTGGAGATCACAACCTTT	TTTCATACATTAACCTTCCCTCATC
41	cgd4_310	TCAGAATTTGTTGGCTCACATC	TGATGAAATAACGGAATTAGAACTTG
41	cgd4_340	GTTGTTACGGATAATTCAGGAGAAA	TTCGCTAACTCACTCTGGTCAA
41	cgd4_360	AAGCATGGATGAATTGAGTGTG	TGAATGTCGTTTCCTGTGTCTT
41	cgd4_370	TGTAATCGCCGGATCTCTTATT	TCCAAATTCTAAACATTCTTCGG
41	cgd4_390	TTGGTGCTTCCTTAATTCCAAA	ATCTTCGCATGGGCTATCAA
41	cgd4_420	AATCTTTGCTGGGAGTATGTTTG	TTCATTGTTTGCATTTCCACTC
41	cgd4_440	GGACCAGTTAATGCTGTTGAAGT	CTGGAGGAAATACCCAAGAGTG
41	cgd4_450	TTGGAGAAGTTGAATTGGAAAGA	TACGGACTGCAAATTAGGACAA
41	cgd4_460	AACTCAACCCGAATCCAAGTAA	CATCCTTAACAGATTTCATTTCACA
41	cgd4_470	CCAAAGGGTTCAAATGTTGTCT	AGGCTCTTCTACCTCTTGCCTT
41	cgd4_480	TCAGTTATGGGAGGTAGCGTTC	CATTGCTTCATTCCTTTCTTCC
41	cgd4_490	TGGCTTATGTTTGTGGTCTTTG	CATTTCGTCGTCTCCTCTATCC
42	cgd4_500	TTGAATGTCCACCTGAATTTGT	GAGGAGTAGCAGATGACCCTGT
42	cgd4_510	AATTCCTCATGGTACGGGTATG	CCATTCAAAGCTCCTCCTACTG
42	cgd4_540	AGTTTGGAGCATTTGACGATTT	CTCAATTCCATTCCAGGGTCT
42	cgd4_550	GGACGATGATGAAGATAGAGCA	AGCTGAAGATGGAGACCAAAGT
42	cgd4_560	TGTGTGCAGAAATAAACCAAGAA	TTTGGGAACTCAACAACCATC
42	cgd4_590	AATCACTCGTGTAGACTGCCAA	CGTTCCCAGAAGGCATAAGA
42	cgd4_600	ATTTGAGTATGGGTCTGGCTTG	GCTGCATTCCATTGTTCACTT
42	cgd4_610	GTGGAGAAGGAGGTGAATACGA	TCTCAAAGCATAAACAGGAGCA
42	cgd4_640	CGAGACTGGGAGATTAGATTGG	AGCCAAGGAACTGCTGAAATAC
42	cgd4_660	TGACTCCAAATAAGGTATGGGTAAA	TGCTAAGACACCAAATCCCAA
42	cgd4_670	TGCTCTTGAATTTGCTCCATT	TCCCTTGAACAGACTTACCACA
42	cgd4_680	AGTCCATTGCATAGAAGAAGCC	TGAGACCAAACCTCATCATCAC
43	cgd4_690	TCCGGTTTCTATGTTTGATCCT	TGCATTCTTAAATTCTGCCTCA
43	cgd4_700	TCAAAGATGGTTCATTGTGGAG	GATCGAGAAAGAGCAAGCAAGT
43	cgd4_720	CTAGGGTCAGCCATTGAAGAGT	CAAAGAAGGGATTTGGAGCA
43	cgd4_730	GGAGCAAAGCAAAGAATACTCAA	TGGACTTTCAAGAGGCTTCAC
43	cgd4_740	TATGGCAGATGGTTCATTCAAG	AGGAGCTGTACTCCCAATTTCTC
43	cgd4_750	GGAACCTCAGACCAAGATGAGT	CCATTGATCCAAGTAATCCACA
43	cgd4_760	TGATGAAGATCAAATAAACTCACAAA	GATTGGAGGATTGAGATGTGG
43	cgd4_770	AATGGGATTTCGATGATGATTT	TTGTATTTCGGCATTGTTATTGTT
43	cgd4_780	GGAAATAAAGGAAAGCGTACTCAA	TTCCACCAAATGAATTACCTCC
43	cgd4_790	TGTCAAGAGGAAATCAAAGAGATATTG	GCACCTCCAGGGTTTGTCTATT
43	cgd4_820	CAGGAGGAGCTGATGGTATTG	TTTCTAATTCGGTACTCTAGGGATGT
43	cgd4_850	GTGCTGGATGTATTTGGGATTT	AAGGCAACTGAAGCAAAGTGA
44	cgd4_910	TTTGATATGAGAAACAGAACACAAACT	TCCTTGTAAGATTAGTTGACCCATT
44	cgd4_920	TCCTGCCGAATTTAGTCTTTGT	TCTTGCTCCATGCCTAGAAACT
44	cgd4_930	AGATTAGGAGTTGTAGCAGCAGG	TCCATTCCATGATAAACCATCTT
44	cgd4_940	GAGAAGATGAGGATGGGAAGAA	AACCTCTTGGCTTGTTTGTAGTG
44	cgd4_950	ACAAGTGGTTGGGATGGAAA	GATTTGTTTGATTGATGACCGA
44	cgd4_960	ATTCCCACAGGTAAGTCGAGAA	GATCATTTCCACCAGGGTATGT
44	cgd4_980	CTGTTAATACTGCTTCATCGTCAAA	GTAAGAACTTGAATCTTGAACCTGAGT
44	cgd4_990	TCAGGAGGATTTGGTGTTGTT	TCTGAAAGTTGATTACTCCCACC
44	cgd4_1050	AGACTTTCATCCTAAAGCATTCATC	AACAAACATTTACACAATCAGCAGT
44	cgd4_1100	TTATGTTTGGGAGACTGATCCAC	ATCTCTGCCCTGAACTCCAA
44	cgd4_1110	TCAGGGTGAGACTGGAGTAGGT	GCCTTTCATATTCTGCTGTTCC
44	cgd4_1130	AAGTAATGCTCATGCAAACATCTC	GTGTTGATACATTTCCCATCATTC
45	cgd4_180	TCATCCAAACGCTCTAGTTAGGT	TTTCTCAAACCTTTATTGTTGTCATT
45	cgd4_1140	CTGAAAGTGAACGCCATGATAC	GATTCATCCGAACCCATACAA
45	cgd4_1150	TTTGAAGCTGGATTAAAGAAACATAG	CATATAATGAATCTCCGTCATCGT
45	cgd4_1160	AGTGCCAATTCATTATTTACCATTC	AAAGACCAGACCCAACCAGTAA
45	cgd4_1170	TCTGATGATGTTGACTTGGAGG	CTTCAGGTATTCCACCCTTCTTT
45	cgd4_1210	CGCCTCCAATTTACGATAAGAA	TGGTACAACATAGCCTTCCACA
45	cgd4_1220	AATGAAACTGAAATAAATAAATCTGGG	TTCCTGGTTACTTGGGAGACTT
45	cgd4_1240	AACAATGCTTTAACAGACGATCAA	TCTAAGTAATTCGGCACCACCT
45	cgd4_1270	CAAATGGATTGGCTTGGACT	TGGTTTGTTCTGTTGACCATCT
45	cgd4_1280	AGTTTGTCATTCCATTGCACTTT	ATGTGAAGGTTTGTATCATTTGGA

45	cgd4_1300	GGAGTAGTAGCTGGAGCGGTAG	CTTGAACGCCATCAGTAACATC
45	cgd4_1310	TGGTTCTACTGCTGGGCTAGTT	TGGTTGTTCTTGCTTCACATTC
46	cgd2_1040	AACTGGTCGAAGATTGTGCTTT	ACCTTCCTGATCCTTCATTAACC
46	cgd2_1080	CCAGAGCTTGACATTTCTGATTT	CATATTTGCCACTCACAGCATT
46	cgd2_1090	CAAGCCATTAAGAAAGAAGGGA	AGATGAGAGTGGGACTTGGAAA
46	cgd2_1120	GTAGTTTCTGGAGCCGCACT	CAGGAGTACCATCAACCTTTCC
46	cgd2_1130	CCAGCAAGGCAGTTTACTATCA	TATTTCGCTTGGAATTTGACC
46	cgd2_1230	AAACAATGCAGAGGAGCAAGA	TCACAACATAATTCAAATGCACC
46	cgd2_1240	TTTGGGAAGAGGCTGAGAAAG	AGGCAATCAGAAATATGCGAAG
46	cgd2_1260	AATTCTTTCATCCAAGCACCAG	TGTCAACCATGTATCAACGTCC
46	cgd2_1460	TATAATGGGCAGGTTCATCCA	GGCTTGTCTGGGACTGATCTAA
47	cgd2_1570	GAAGTTGGTGTAGAAGGGTGCT	CAGATCCGCAAGTTTCAAGTC
47	cgd2_1580	TGTCCAAATCGTTCTTGTGATT	CTTATGGATCTGAAAGGATGGG
47	cgd2_1720	CTACATGAATTGGTTTGTTTGCTT	AATTGAGCGATTACCTAGTGCC
47	cgd2_1770	GGAGTAAGGTTAAAGGATTGCGT	ACATCATCGCCAAATACAGACA
47	cgd2_1930	TTCCTCCTAACCATTTCTTCTCC	CAGGTTCACTACCAACTTCCCT
47	cgd2_2010	GTCATGCCTATGCTCAACCAC	AGACCCAATACTGCAACGGA
47	cgd2_2050	TTGGTCTCCACAAGGAAGAGTT	AAACTGCTACGCCAATATGGTT
47	cgd2_2070	AGAACAAAGGCCGCTAATAACA	CAAAGAGGATTGGTAAGGGACA
48	cgd2_2110	CTTTATGGGATGCGGTTACATT	AGTTGGAGCTGAACCACTTGA
48	cgd2_2240	ATTTCTAATGCAAACAGCTCGG	AGGCCACTAAATGATGATGGAA
48	cgd2_2550	ATCATCAACCATTTCAGCAAGA	TCTCTCATTTACAATCCAGGCA
48	cgd2_2910	ATTGGGATGTGAAGCAAGAAGT	AGCAAATGAATCCAACTGAACC
48	cgd2_2920	AGACAACAGCTATGTTACCGCA	AAAGGAAGCCATTACAGTCCAA
48	cgd2_3100	ACATTCAGTTTGGCTGGAGTTT	GAGGCTAATGGACTTTCCGTC
48	cgd2_3110	TATCGCCAAAGTCCTGCTATTT	TCACTCAAACGGTGCTTATCTG
48	cgd2_3270	GTGGTATTGATGAGGACTTTGGA	ACTTCGACGGTAAATTGATTGG
48	cgd2_3290	CTCAAAGTACCCGTTTCCTGAC	TCACATTTATCGCATTCCAAAC
48	cgd2_3300	GAATCTTTGGTGGAATCTTGGA	TGTGCATCTTTAACCTGTTCATTT
48	cgd2_3310	CAATTACAAGGGTTGGTAGGATG	CACTATCTCCTGTCCCACTGAA
49	cgd2_3320	AGAACGCAGGATTGTGTAGATG	TCCCTCTTGTTGCTTAATTTCC
49	cgd2_3350	ATGAAGTTGTTGCCGATGTTT	GATTTGAGTAGCAGTTGGAGCA
49	cgd2_3380	TATGCCACGTTCAGTTCCATAC	TTACACCTTCCATAGTCCCACC
49	cgd2_3420	TCGATTCTGGAGCTGGTAAAGT	CGGTTATGGGTGTCATTTCTTC
49	cgd2_3490	TCAATGGACAAATGGTATGGAA	TCCGCCTACAACAACTAATCCT
49	cgd2_3700	AATTCACCTCCTCCTGACTTGA	ATCCTCCCATTTCTGACTACGA
49	cgd2_3710	CCACCAGCTTTAGAATCAGCA	ATCTATTCCACATCGCATATAACTTTC
49	cgd2_3730	TCAGCGATACTGCAAAGAATGT	TAGCAATGGAAGAAAGCTGGA
49	cgd2_3920	GAGGCGGATACGATCTGAATAG	GAATTGCTGCTGAGATTTGTTG
49	cgd2_4020	TTATGGACTTTCTACATGCTCCC	TTCATATAATTCTTCTGAGGCTTTCTT
49	cgd2_4040	TGGCACACCTTTACCAATTTC	TTTGGGATGATTCTTGTATTGATTT
49	cgd2_4080	GGGTAGTTTCAGCTATTGCACC	ACCTTCATTTGATGCTTGGTCT
50	cgd2_4210	AAACTTAGGGTTGTGGAAGGATT	TCAAGTAAAGTCAATTCGATGGTT
50	cgd2_4370	GTCATCCTCTTCTTCACGCC	TTTCTAGTTCAGCTTCAACAGCC
50	cgd3_120	GACAACGATTTGAACAAGAGAAACT	ATTATTGCCATTCACCTTTCCA
50	cgd3_130	ATTGCAGATATAGAGGCCGAAA	CATCAAACCCTTCACCTTTACC
50	cgd3_170	AAAGAACTTGGAACATGGAATAAAGT	GTTGCGATATGGTTTGCTGTT
50	cgd3_200	AGTGGAGGAAGCAATACGAAAG	GTTCAAGCTCTCCCGTAGGTAA
50	cgd3_230	ACCAATCTTGCTTGAGACTTCC	TTCTCCTTTAACATCCGAAACC
50	cgd3_320	AAATGCAATCAAACCACACAAA	TTACAATCAAACCAAGAGAATCCA
50	cgd3_490	GAAGCTGGAAAGGAATCTGAAA	GCACATGAAACAAAGACAGGAA
50	cgd3_540	AGCTATTCTATCAGCAGCATTTCA	CAACAGAACCTCCCACCATT
50	cgd3_820	CAATCAAGGTTCAAGCAATTCA	GATAATACCAGCCTTCGCTCC
50	cgd3_830	AAAGGGAATTGCTCGTATCTTG	CGGTCATCTTCTTAGCTTGTTGT
51	cgd3_840	ACTGATGCTGAGTTGGAATCTTT	GTTGGACTTGTCTTCTTATCCCA
51	cgd3_870	CATTATAGTTTGTCCCGCAATGA	TTTCAATTTCTTGCATTCCACC
51	cgd3_910	TCTGGATGTCAATGGCTAGGT	CCAATCTCCATATCCTATAATTTGTCT
51	cgd3_920	AGCCATCTCAACCACAAATACC	CCGCTACCACTCTGAACAAAC
51	cgd3_990	CAGAAAGATTTACCTGAATGGTTAGA	AATGAGATGATTGCGACTCTTG

51	cgd3_1040	TGATAAACTCGCCTCGTTATTATTT	AGAAATCTTTGCTCATTTGGTTG
51	cgd3_1050	ATGCAGGGACTAACTTTGGAGA	TAATCCGAACGCATCTTTCTTT
51	cgd3_1110	ATGTAGCAATGAGTGAGCCAGA	TCCTAAACTACCAATCCCTCCA
51	cgd3_1450	CGTGGAATTGACGTTGTTAGAG	CTGAGAGCTTGTTGTGCTGAGT
51	cgd3_1460	ATTTCCTCCAGTATCGTTGCC	ATTGCCTGGGTGTTGTTACTCT
51	cgd3_1470	CTGAGAGGATGGCAGTTGTTG	ATAGGCTCTGGCGTCTTTCTTT
51	cgd3_1480	AAGGAACATCAGCAAAGATTTCA	ACCCTCCATGTCCATCAATTAC
52	cgd3_1500	AATTGGCTCAAAGGTAGGGTTT	GCTACTGCTCGATTCTTGTTGTT
52	cgd3_1550	GATAATAAATTGGCAGAGGAATATGAA	ATTTGAAGATCCCGCATGAA
52	cgd3_1580	TTTGGATTGACATGAATGAACC	GTTGTGGACTTGTCTGTGCTCT
52	cgd3_1600	ACCGAAATGCAAATTCACAAG	GTGATGATGTTGTTGTTGGAGG
52	cgd3_1610	GATTACGAATGTATGCTCGACTTG	CCAGATTTGTGTTCCTTCTTCC
52	cgd3_1620	CTCTTAGCCTTTCCTGCCATAA	TGATGAGAATATAGCCTTGCTCC
52	cgd3_1630	AAGCGCAGGCTCACTAACTAAA	AAGTAATTGTTGATTGTCTCTTATCGG
52	cgd3_1640	TTTATCACCTTCCACAACCACTT	CAACGCTCATTTCGTCTATTTG
52	cgd3_1670	ACCATATAAACTTGCACTCTTCCA	CGCCATGTATGCTTTACCAG
52	cgd3_1700	AGCACTGAAAGCACCTTAAACA	CATCGTAGAGTTTGCCTGAAGA
52	cgd3_1760	AGCGAGCATCCTTCTCCTTT	GGAGGGCTTTAACTTGCTGTTT
52	cgd3_1770	CCAGAGAATGAGGGAAGTTAGTG	TAGTGGAATTGGAATGAGCAAG
53	cgd3_1820	TGTTGTCTGGTACGGAGAAATG	CAAGAAGGAACCTAGCCTGAGA
53	cgd3_1990	AAGTCAGGTGGGTCTGATGTTT	ATGATCCTTGGCTATTCTGCTC
53	cgd3_2020	TCCCAGATGTCAGAATAATCCC	GACGGACAATATCAACGCATAA
53	cgd3_2080	TATTAGGCTTTGTTTGCAGGGT	TCTCTGTGAATTGGACTTGGTG
53	cgd3_2110	GCGGAGATTTCTATGGGTACAG	TGTATGTAAGGTTTGGAGCGATT
53	cgd3_2150	TGGATCGTATTCTATTTGTCTCCA	CGCTATATCTGAGCCACTTTAGG
53	cgd3_2180	AGAGGCTATTGGTGTTGCTGTT	GGGAGAGAAGCCAAATGTAATG
53	cgd3_2190	AAAGCGTTTGTTGATAGTGGTG	TGTTATTGAGAATGGAAAGAAGGA
53	cgd3_2220	AATCTCGCAAATGATCCGTC	GGGTTCCATAGGGTAATGCTC
53	cgd3_2230	GTGACATTGGCTGTGATAATGAA	ATAGCTTCACCTTCGCCATAAA
53	cgd3_2240	GGGTTCATTCGGACATTAGAAA	GCTTATTTCGATCTTGTCATCCA
53	cgd3_2410	TCTTTCTCAGCCAATTCAAACA	CCAAACCTGATGATACCTTCGT
54	cgd3_2390	TTTAACTGCAATATGGTTCAGGG	ACGCCTTCTCCACTGCTTACT
54	cgd3_2490	TTTCAAGTGGCTACACACCATC	ATGCGAATAAGAGACCACCCT
54	cgd3_2500	TTAGCGGCAAGACTAAGAAACAC	TGTACTCATCCAATCGTTCAGG
54	cgd3_2510	CACATTTCAGACCGCATTTCT	CGCATAATCTTCGTCATCTTCA
54	cgd3_2530	ACTGCTCCCTGCCTATCTGAC	ATCTGCACCAAGAACAACACAA
54	cgd3_2550	AACAATAAGTGGTGGGCAAAGT	TGCATAGCATCAAGAACCCTAA
54	cgd3_2580	TTATATCAATCATCGGCTCCTTT	TTCTTGTAGAGTCATCGCTTGG
54	cgd3_2590	CAAGACGCAGACGAGTCAAA	CCTTCATTCTTTCTCCTGCAAC
54	cgd3_2600	GCTTTCTTTGCTATTCCGTTTG	TGCCATGATTATTGGTGGAAC
54	cgd3_2610	TCAGGAAATGAAATTGAGGAAGA	TGCGTTTCTGTATGAAATGTGAA
54	cgd3_2620	GGAGCGAAGATGATTTGACTTT	TTTGATTGGCCTGTTACTCCTT
54	cgd3_2630	GCGAATTGCATTAAAGTTCCA	AGAAACCTCCTTTCCATCACAA
55	cgd3_2640	GATTATTGAAACATTTGATCTCCCA	TTAATATCTGACTCGTAACCACCAAA
55	cgd3_2740	CATATTCTGGAGAGAGCGATGA	ATCATTCGTTGCCTTACCTTCT
55	cgd3_2750	ATCAAAGAGGCGGAGATTATTG	AGGTTTCTGATTTCCAAGGTCTAA
55	cgd3_2870	GCGACAATCATCCTTGGTATCT	TGGATCTTTATTGATGTGGGCT
55	cgd3_2920	ATATAAACTCTGATGTCGGGTGG	TTAAATTGCCCAGAAACCTCTT
55	cgd3_2950	AGAAGCGTACCATTTCACCAAG	CTCCTTATTCCTATGCCCGATT
55	cgd3_2960	ATCGCGTTAATAGATGCACAAA	ATGCAGGATCAATCGTCTAGGT
55	cgd3_2970	AAGTATGGAAGCCCTTTGACTG	TCACAAATAACACTTGGCTGCT
55	cgd3_3050	AGCAACATAGGAGTAAGCCCAA	GAGCCCGAACAAATAGGTGTAA
55	cgd4_800	CAATGTCAACTCCATTTCCTCA	GAATTATCTTGCCCTGATGTCC
55	cgd4_870	GATCGTGGTTCTTTAGATTATGGAG	TAAGGCAAGTTCTTTGGAAACC
55	cgd4_970	TCCAAGCATTCAGATCAGGTTT	AACTTCATAGGCTCCACCTCCT
56	cgd3_3060	TTGGCTTCATTTCACTCAACAG	TCAATAGAAACAAGGGCGATG
56	cgd3_3070	AGACAATGAGGAAGCATGTGAG	GTTCTCCAGAGGGAGTGAATGT
56	cgd3_3090	TGGTGCAAGATTTCGTTTATTG	TTCTGGTTAAGAATGGACTGCC
56	cgd3_3120	GCCTCCGTCTACTATGGCTTAC	TCTTATATCTGCGAGGTTGTGATT

56	cgd3_3150	ATCTGAAATTGGTCTTGTTGGG	CCGATTGTTGGTTCCTGATACT
56	cgd3_3160	ACTCCTGAAGAAGCACAGAAGG	TGGTTAGTTGAGATGCAGATGG
56	cgd3_3170	TCACTCAGGTGGAACAAATTCA	TGCTTGTCATCATATCTGCCTC
56	cgd3_3180	ACTCGTCTTCTGCCAATTCAGT	AATGCCTTTCGTTATTTGTGCT
56	cgd3_3190	CTGGATTGGGCTTTCTTCATAG	ATGTTCTGGCTCTCTATCATTGG
56	cgd3_3230	AATTTGTAAAGTGGGCTGCATAA	TTATCGAACCTTCCTCCTTTGA
56	cgd3_3290	GGAAGAAGAGCAAATGGAGAAC	TTTGGGAACTGAATAACGACAA
56	cgd3_3310	CATGACGAGAGGTCCAATCTTT	AATAAGTCAGCAAATCCCATTCC
57	cgd3_3450	TCAAGCGTTCAGGTTATTGAAG	CCAACAAATTCCTGCTTATTCC
57	cgd3_3460	GCTGCTCCTTCATACTCTGGTT	GATAGGGATGTCCTTGCATGTT
57	cgd3_3490	TCGCATTAACACAAGTTCTCAAA	GGGTCCATAGAATAACGATCCA
57	cgd3_3500	AGTTGGTGCAGCTTTGGAGT	AAGTGCTGAATTGATTGCCTTT
57	cgd3_3520	TATGGCGCATGTGTATTTCTTC	ACGACAACCAGTGCTGATATTG
57	cgd3_3550	GAGCTGTATTATCGCAAGGGAC	GAGGGAAGAATGGAGGGTAGTT
57	cgd3_3620	CCGTTTAGATAGATTGATTCCTGTT	TTAATTCGTTTCCTGCATCATC
57	cgd3_3690	CAACTCCAGGTAGAATGCAGG	CTTTGAATCTCTGGCGGTATCT
57	cgd3_3830	ACACTGGCGATTACCATTCACT	AAACCTCCTCAAACAAATCAAGAA
57	cgd3_3870	TTGGAGGACTTCAGAGTGTTTG	ACCTCATCTGGTCTCTTCATCC
57	cgd3_3940	GTAAACATGGATGCAAAGCTCA	CACTTCTTGATTTGTAGATGGTCCT
57	cgd3_4030	TGTGCAAACAACACAAAGCA	CGAAGACTGCTAGAATAAACCTCAA
58	cgd3_4040	CCCGAGAAAGAATTGAAAGACA	GCTGACGGTTAATTTGAGCATT
58	cgd3_4080	CAAATGAGTTCTCCTCCATGATT	ACTTTGAGTTGTCTGCCTGTTCT
58	cgd3_4120	ATGAATAGGCAAGGTGCAAAGT	ACAAATCTACCATCTTCTGTTGTCTCT
58	cgd3_4130	TTAAACTGCTTTGGTCTTTCCAG	GTAACAGAACCATTTGTATCGCC
58	cgd3_4140	CGCTGGGAAACTTAGGATTATG	TCCGTACTCATTTCTGTTGGTG
58	cgd3_4150	ATTGTGGCAACCTTCCATATTC	TGACTTGGCTCCTTCTTCTTGT
58	cgd3_4190	CGCTTCCTCTTTATCAGTTGTTG	AAGAACCTTGGAAATGGATTGA
58	cgd3_4210	TGGAAGAGTGCAAGAAAGTCAA	TAGCCAACGTGGAATTAACAGA
58	cgd3_4230	CTAATGTGATTGAGGTCTGGCTT	CCTTTAATAGATATAGGCTCATTGCTG
58	cgd3_4240	ATTGTTGATTCCTCCGTTGTTT	ATGGGCTACGAAATGTAGGGT
58	cgd3_4260	ACATTAAACACGAATGCAAGCC	TCTTCAATATCAGGCCGTTCC
58	cgd4_400	GGCATTATTAGCACTTAAACTTGGA	CTTCTTCAAATCGTCCCAACA
59	cgd4_350	GGAAAGTTCATGGTGTTAGTTTCA	GATCAATTACAACAGGCCAACA
59	cgd4_380	GTCCACTTTATTGCCTCATGGT	ATCCAAAGAAACATCGTCCAAA
59	cgd4_430	TGTAAGGCCAGTCGAACAAA	TAATTCGCTCTCAAACAAACCA
59	cgd4_1320	CGGATAAATCTGGTCTTCTTCC	AACTTTCCATCCATTCAACTCC
59	cgd4_1330	ATATCCAACATCAATCGACCAA	TCAGGTACTTCTCCTTGTCCTGT
59	cgd4_1340	AATGGTTCCAATACAAATCAATATCA	ATGATGAGGAGGAGTGAGGAGA
59	cgd4_1350	GCTTTGATATGGAACGGATCTT	CTTGCTCTTCCTCTTGCTCTTC
59	cgd4_1370	TGGAGTTAATGGTAGGGATGGA	ACGAATCCAAGGGAACATAACA
59	cgd4_1380	TTGGATGCTTGGTTTAAGATGA	GACAATAAAGGAGCTAATCCCGT
59	cgd4_1390	GGAAAGCAATACAAAGTGGAGG	GCAGAGAATATCATCCAAGCAAC
59	cgd4_1400	AACCTATCCAGGTCCAATTCCT	GAGGTCCAACTCCAATTTCAAC
59	cgd4_1420	ATACGACCCAATGATACAAGCC	GTAATCTTCCTAGTCCTGCTCCTG
60	cgd4_1430	GCCAAGCCATACTCTTCCTACA	CTGATCCCTCTCTTCTTTATTCTTTCT
60	cgd4_1440	TGAATCTGAACCTGAACCTGAA	TGAAGCCTGACAACTACTCCAA
60	cgd4_1450	AATAGGAATGGGAATGGGAGTT	GTTTGAACTTGGATTTGAGCTTG
60	cgd4_1470	CCGGATTTGGAGATATGATGTT	CAGCTATTGATTGGAAATGGGT
60	cgd4_1480	TTATGGATTTGTTAAGGGTGGAA	GTAAAGGACATGGATCATTAAACTCA
60	cgd4_1500	TGAAAGCTCGGAAGTTGGTAA	TTGCATCCACAATATCCTCATC
60	cgd4_1580	AAATGAATCCCATCAGGAAGAA	TGCAACTTCGGTTGTATATTTGA
60	cgd4_1600	TGAATACAATCAATAGCACGCC	GCACCCAGTTGAGGAGTTAAAG
60	cgd4_1610	ATCAGGTGGTTATTTGATGGCT	CTGTAAGGGAATCTTTGCTGGT
60	cgd4_1620	TCATTCTTTCCAAGTGAGGATACTTT	TTCATTCTTGTTTCCCAATCGT
60	cgd4_1680	AGTGCCAGCAATGGAATCTAA	AAATAGTGCGGATTTGAAGGAA
60	cgd4_1690	ATTTCAGGAGAGAGTGGAGCAG	TCCCAAATACTTCTAACAACGGA
61	cgd4_1720	GGTTGTATTTCATCTCAAGCAGG	TCAGTGTATCTGTATAATCGCCAAA
61	cgd4_1730	AAAGGAAATATGGGCTGGATAAA	CTGCAATAGAACGAGCAATCAA
61	cgd4_1770	AACTTAGAACAGGAAATTGTAGACAGG	ATTGACCTTGGAATCACGCT

61	cgd4_1780	ATGCCCGATATGCTATGAAAGT	ATCCACCAATCTATGCTCACAA
61	cgd4_1790	TTAGCGTTGCTTGGTTAAAGGT	CTGATGGACAAAGAATCCCAA
61	cgd4_1800	TCTGATTGGCTATTGAAGTATTGTG	CCCAGGCTCATTCCTAAGTG
61	cgd4_1840	GGCCATAAGAAAGATGATGGAG	AACAATGAAATAAGAGCCGGAA
61	cgd4_1850	CTGAACCTGAAGAAACATATTGGA	TTTCATTATTTGCCACCACACT
61	cgd4_1860	GCACTTGAAAGCAGCAGAATAA	AATTGGAGTGGCTGAAAGAAGT
61	cgd4_1870	GTTCACGAGGGTATTCCTGTTC	TACTGAATCTTTCGCTTGCATC
61	cgd4_1880	TTTGGGTTCCTAGCAGAAGAGT	TTTGCATTGTTCAGTTTGTGATT
61	cgd4_1890	AGAAATATAATCCATTCGGTTCCA	GACACTGATTCTCCATTCCACA
62	cgd4_1910	TCAGCATCTCTTCTGCCTTTCT	TGCCTTAATGATATTTGGAACGA
62	cgd4_1920	GCAAGGATTTGCTCAGTTAAGG	TCTCTCAAGGAGTTGTCTGGGT
62	cgd4_1930	GTTGTAAACACTATTGCCACCTTG	CCATTCTTGTTCTCGCTATCATT
62	cgd4_1950	TGCAGATTGTATCAGAAGACGG	TTGAGACGGCTAATGGGAAA
62	cgd4_1960	ATGACTGCGTGTTATTGCTGTG	GCTCGTTGTAGTAGATAGTGATGCTG
62	cgd4_1970	TATGGATTGGTATTGGTGTTGG	TAGTTTGCATTGGGATCTTGAC
62	cgd4_1980	GGAATGGGATTTATTGTTGGTG	TAATGGCAACCCTGGAAGATAC
62	cgd4_2000	TAAGGAAATTGTCAGGGAGTGG	CTTCTCTTGTTTGGCTACCTTCA
62	cgd4_2010	CAGTGTTCCTTACTGGAGCAGA	AATTCTTACGACATCAACATTCCA
62	cgd4_2030	CGGTTGCATTAAGAGAAATTAGAAA	CTCTGCATTCCATCTCCACA
62	cgd4_2070	AAATAGAGTTGGAAACGAAGATCAA	CGCAATGAATAGTGCAGAAACT
62	cgd4_2080	CCAGGAATTTGTTGTTGCTGT	TGGACTCTCCTTTGCTCGTATT
63	cgd4_2090	CAGGTACAAAGTTGAGGCCAGT	ATCCGAGTCAGCATCTTGTTCT
63	cgd4_2100	ATGACTTATAGGAAGGGCGTTG	CTTGTGTGTGAGGAACAGAACC
63	cgd4_2120	CCGTCCTCTCTTCGATATTGTT	TCAAGTGCTCGCTTAGTTGTTC
63	cgd4_2130	TAAAGAAGAGGAGAGGGCAGG	TCATTTGTGATTCGATTTGCTT
63	cgd4_2140	AAACAACTCTCCGGTTGAAGAA	GTCTTGCTCTGAAGGGATGAAC
63	cgd4_2150	CGATTTCGCCCACACTTACT	CATGGCGCTTATCTCCTTTATC
63	cgd4_2160	CATCCGTTGAGAGCTGTGATT	GTTGACCAAGAGAATCGGACA
63	cgd4_2170	AATAATAAGAATGAATGGGTGGAAA	GCATGAAAGAGTTAATGGTGTGA
63	cgd4_2180	TCAGACAGGACTTATCCCAGAAA	TGCTAATCTTAATGTTGCGCTG
63	cgd4_2200	GTAGGAACCTCCAGTGAGTTGG	CAGTCTCCCTCCTGCTATCATC
63	cgd4_2210	TGCACAGATAATTGCAGAAGGT	CGCCTATAACCCAAATTCCTAA
63	cgd4_2220	TTGGCCCGATTGAAACTAAC	TCAGGCTTAACTAATGCGATTCT
64	cgd4_2230	AATGTTCCAAATCTACTACCGCA	GAAATGATCGCATATTCTCTTGG
64	cgd4_2240	GTAACATGACCATTTGTGGGAA	TCAGTTTGTGCTGCTTGAATG
64	cgd4_2250	CAAATTACCTCTTTGCTTTCTTGTT	TTACGTGATCTCTTCGATCCTG
64	cgd4_2280	TCTTCAATGCCAAGAAATCCTC	TTTGTCTCCTCTGTTCCTTCATCT
64	cgd4_2290	AATGGGTCAAGCTACATTTGATTT	GTATGGAGGTGAAACAGTAATCCC
64	cgd4_2310	CTAATTGGCTTTGGAACAAGGA	GTGCCCTAGAGAAATGGATGAG
64	cgd4_2320	TAGATTCACAGAAAGAAACTTCCAAC	ATTCCGCAAATTCATCACATC
64	cgd4_2330	TCAATAGGTTTCTATGCTGCGA	CGCATCGTTTCTTTCAGGTT
64	cgd4_2340	GGCATTACTTATCTTGCTCTCCTT	TCCTCATTGCTGAACTTTACTCC
64	cgd4_2360	GATGGTATGAAGGTTGAAAGTGG	TATTGTTGTTGGTTGTCTGGCT
64	cgd4_2380	ATGGAAGAGATACTGGGTGGAA	AACCGTACTTATGGCTGGAAGA
64	cgd4_2390	AAGGAGTTCAGCATCCAATGAG	CCCACGTATCCATTACTGTTCC
65	cgd4_2410	CACTGCAACTAAACAAAGAGGTTTC	TGCTTGGCTTGTAATCTGAGG
65	cgd4_2420	GCCTCAATACTTCTAGGTGCAGG	TCGCAGCAATATGAAGAGGAGT
65	cgd4_2430	GTTAAATGATGGAATTGCCGAA	AGTATTGTTGTGAGGGCCAAGA
65	cgd4_2470	AACAACCTTGGTATGCTCCATT	GGCCGCAATTACTCTTACAACT
65	cgd4_2480	TTACTCTTTCTGCTAATCACTCGG	ATGCTGTGTGAGGAATTGTGTT
65	cgd4_2490	ATGCAATCACAGGTTGTTCAGT	GTGGGTCTTTCATATCTGCCTT
65	cgd4_2500	TAACCCACTGTTGAGGGTCTTT	CTCTTGATTCGATGCACTCTGT
65	cgd4_2510	GCAAGTTCTGTGTCAAATGCTC	TATTCACTCCTCCAAGCTCGTT
65	cgd4_2520	AGAAGCGATCAGGCAAAGAA	GATCCGAGTGCCAAATTATACC
65	cgd4_2550	TTTATTTGTTAGGGTGGGTGGT	TCCGAATACACTAGAAAGCTCAAA
65	cgd4_2560	GCAGCTTCAATTCCTGTTTCA	ATGAGCTTCCATTGTTTCCTGT
65	cgd4_2570	AAATATCTTGCTGGCTTCCAAA	ACCACCTGCATAAACAATACCAC
66	cgd4_2580	CTATCCCTTCAACTCGCAAGAC	CTCTCTGATCTGGCCTGTACCT
66	cgd4_2600	AGAGCCGAATAACCTACTTCCA	TCTCTTACTCCTGTTCCATCCC

66	cgd4_2610	AAGCCGACCCAACACTATCA	TCCAATTTCTTCCACATCGTCT
66	cgd4_2620	TTATGCTGTTGATGAGGCTTCTG	GGATCTAACTTGCGAATAGACTTCTTG
66	cgd4_2630	TGATGCAAGTATGTCTAAGCGG	GGCTCTGATGGATTGATTGATT
66	cgd4_2640	AAATAGGAAGGAACGCCCATAA	CTTCTGCCATAGAAAGCATCGT
66	cgd4_2650	ACGCTAATTGATCCTTCTGTTATTG	GAGAAATTGTTGGGAAGTTTGG
66	cgd4_2660	GACCCATCTAATGCTGAAGAAAGT	AAGAGGAGTACCACCAAAGCAA
66	cgd4_2670	ATTGTAATGGGTGAATGGAAGG	GCATTGGCGGATATTTAGGTAG
66	cgd4_2690	TTAGCTCAAGTCCCAGTCCAA	TAGTACCTCACGCAAAGACGAA
66	cgd4_2700	TGACGAGAAATATCAGTGTCCC	TTCGTTGGAAATAAGCTCTCCT
66	cgd4_2710	TATCTGTAATCAGGGCAGCTCA	TTCATAAATCCATCTACGCCTGT
67	cgd4_2720	AGCGAAGGAGCGATAGTTGTAG	CTTCAGTAGCAGGATTGTTCCC
67	cgd4_2740	AACATAGAGGAGAGCCTGATGG	GACATGGTTGATGGTTTGATTG
67	cgd4_2750	TGGATTTCTCTAGGGTCTCACAA	CTGCCAAACTCATTACTGCATC
67	cgd4_2760	GCAATGTGCAGTCTTTCTTTCA	TGCATTTCCACTTAATACCCTCA
67	cgd4_2770	GCATCAAACTATGGCAATGAAG	ACAGGCTCAGTTACAGTTGTGG
67	cgd4_2780	AATTACCCTTGCTGATGCTTTG	TTGGCATTCCCTTATTCTTGAC
67	cgd4_2790	CCCAGAATGTATCTCTCCTAACTTG	AATTGCGTTATCTAGCATTGGAC
67	cgd4_2810	TACTTGGTGTTAGGTGGTTGGA	TTTGGAGGAGTGTAATGAGAACTG
67	cgd4_2820	GATGCAATAAACACGGAATTTG	CTTCTGTTCGGTAGGCTCTTCT
67	cgd4_2830	ATTCGCAACGGAGCAGATAA	CAGCAAATGTACGAAGGCATACT
67	cgd4_2840	AATACGGGCTTTCACTTAATGGT	CCGACCACAAATGTTACTGAACT
67	cgd4_2850	GGAACTATATTGCAGAACTTGGG	GGCTTTGCGTGGTGTATTATC
68	cgd4_2860	ACAGAGGTGATGTTGTTCCAAA	TTAATTCCACATTTGAAGCCTG
68	cgd4_2870	CCGTGGCTATCAAATTACCAA	CTTTCCACAAACACAGAAGCAA
68	cgd4_2880	AAATAAGAATAATAATGGCTGGGTTG	GTCCTGACCTTAGCTGCCTTC
68	cgd4_2890	TAAGCTGCAATGAAAGTGAAATG	TTGGTTTAATGTTTGTGCGTTC
68	cgd4_2920	CAAACCGACCTAATAAGCGAAA	TGCATCTTTGTTCAAGCTCATC
68	cgd4_2930	AGGATATTCAAATTCGGCCAC	GGCAAAGCCCTTCTATTTCTTT
68	cgd4_2940	ATGCCAGACAAAGACAAGGAA	CGCATTGTTTATATTCGCTCAA
68	cgd4_2960	ATCCTCAGACTCAACCAAATCC	CCAAATGAGATCCCTAAATGGT
68	cgd4_2980	ACGATGTTTCAATCCATTCCAC	TTCCTCTTCTTCAGCCTCTTCA
68	cgd4_3010	AAATCAAACGGAAATACAGTCAAA	TAATGTGCCCAGTGCTAACTTC
68	cgd4_3030	ACTTGTCTCGCTCAGAAACTCC	GTGATCTAGGTGGCGTACCATT
68	cgd4_3040	TTTATCTCTTTCCGCACATAAGG	TACATGCTTCACCAAATCCAAC
69	cgd4_3050	AACGCAATTAGAAGAGGCGTAA	GGCATAAATAACGCAGAACCA
69	cgd4_3070	TGACAAGGAGAACAACAAGGTC	TAACTGGTTTGCTTTGGCTTTAC
69	cgd4_3090	GCTGATTATAGATGCCCACCA	GAACCATCCTCCCATGTTTATC
69	cgd4_3100	CGGTAAGCATGTAAGTGTGGAA	ACATCTATAAACTGGAGGCGGA
69	cgd4_3110	GCGGATTACTTTCAATGCTTCT	AAACCATACCATACCGATCCTG
69	cgd4_3120	ATGTTTGGAGACTTTGAGGCAC	TGTGATATGCAGTTAATGGTGGA
69	cgd4_3150	GTTTGGAAATGAGCGAGAAGAG	GGGTGCAAGACTCCCATAGTAA
69	cgd4_3160	CATCGTTTGATGTGTCTCGTCT	AAATTGGTTGCTTGGTTTGATT
69	cgd4_3180	CAACAAGAGAACTTTGCGTTCA	ATTGCATAACCATATCAAGCCC
69	cgd4_3200	TCCACATCCATCTATTTCCACA	AGCTCCTACCTTGCTACTTTGCT
69	cgd4_3210	CCAATATCAACCACTGTCCAAA	GTTGTGTGTTCCCTTGAATGAG
69	cgd4_3220	AAATCAACTGGTGGTAAGGCAC	GCAATTTCACGAACCAATCTTT
70	cgd4_3230	ATATGGTCCTGAGTGGAGCAGT	TGATGAAGTTGTTCTCTGGCTTT
70	cgd4_3240	TAACTCAATCGACCCAACTCAA	TCAACAGGAATTGCATTGTCTAA
70	cgd4_3250	AGTTCTCTTCTTGGAGGTGGG	CATGCAAGTCCATCTTCTTCAC
70	cgd4_3280	ATCGCTATCTCAAATTCCGTGT	TACTACTGCTGCTCACTCCACC
70	cgd4_3290	CCAATTCCAGTAAGAAAGCGAG	TTCCAAATTCAACATCAACAGG
70	cgd4_3300	GATCGAGGTTGAGAGAGTGGAG	CTTCTACTGGCTTTGGGACTTG
70	cgd4_3310	TCGATACCAGACCATCATCAAA	GCGGAGTAGTCGTAAATCCAAA
70	cgd4_3320	TCTTGAAACAGGAGTTGAAGCA	TTCTATGCCAAGGAGCGTATTT
70	cgd4_3330	TGCAAGAGAAATCACACCACTC	CTGCACTCCAAACATCACATTC
70	cgd4_3350	GCAGCACATAAAGGTGAGTTTG	TGAACAGATCCATAACTGGGAA
70	cgd4_3360	GAACATTATTGGCTTCCAGGTC	GAAACTGGGTTCTGTTGTCTCC
70	cgd4_3370	TTAATTCTTGACTTCCAACTTCCA	TGAAACCAAATCCATCATCAAC
71	cgd4_3380	CAAGGATGTTCCGAGAAATGA	ATAGGTCCCAGCCATTCACA

71	cgd4_3390	TGACAAACTCAGAACATGGCTT	AATACCTCCTTGTGAAACTGCC
71	cgd4_3400	CAGAAATGGACTATGATGCCAA	CCAGTATGTAACCACCCATCCT
71	cgd4_3410	TGGAGCGGGTTACTTTACTGTT	ATCACGGACACATTCACAACTC
71	cgd4_3420	ATGACTAAAGGAAGCCGAATCA	CATGCTCAGTGTTAATGGTCGT
71	cgd4_3430	TGTCTGGTCAAGGAGAAAGTGA	TCAGAAAGTTCGTCCAATCCTAA
71	cgd4_3440	CTTTACAGGACCAAGGATCTTCA	TGCGACATCAATTCTTTAGGAG
71	cgd4_3450	CATGGAGACTGGAGTGATGAAG	TGGGCACAAAGCTGTAGTAAGA
71	cgd4_3460	TGAACTGAATGCTTATTCGACC	AAATGGAACTGGAACTTGAACC
71	cgd4_3470	TTGAACCAGAAAGTGGTATTGATG	GGAGCCAAATCTTATTGTTGGA
71	cgd4_3520	ACTTCAGTTCCAGCTTCTGCTC	TGGAGGTGCCAGATAAGTCAA
71	cgd4_3540	TGCTCATTGTGGAGTTAAGCA	GATCAGGGATTGGTTTGTGAG
72	cgd4_3560	AAGTTGTGGAAGGAGTCTATGTTTATC	CACTCAATACTTGTTGTTCCTGCT
72	cgd4_3580	CTTCAGTTGTGCGATCAATTTC	ACACCAGTCTTATCCTCCTCCA
72	cgd4_3590	GGGAGATGAACGAAGATAACTCA	TCACCAAAGGTCTCGCATACT
72	cgd4_3600	CCGACTACCCATTACAACATCA	AACAGGAATAAGTTGGGCAAGA
72	cgd4_3610	TTCCTTGCTATTATGGGATCGT	TATTTGGCTCAAGTGTAGCGG
72	cgd4_3650	GGTACTGCTGGTTCAAGAGAAGA	AACCTTCGATACGATTCCTCAA
72	cgd4_3680	AAGTTTAGTATTAGCAGTTCAAGCAGG	AGTTGATCCCTTTCCAACACTC
72	cgd4_3690	AGTTGGGATAATTGGAAGCTCA	CTCTTGTGCTCATTCCTCCTCT
72	cgd4_3700	CAAATTCCTGTTCTGATTGGAGT	TGTTGAATGGCTACCTGAAATG
72	cgd4_3740	CACAAAGAAGAGTGATTGGGAAA	TTTCAATAAACATGCCGTCAAA
72	cgd4_3750	CACACCCTATTTCTCCTCCTTG	CATCCTGTAACTTCATGGTTTCC
72	cgd4_3760	GCATATAAGATTCAAGAACGACTGG	AGGAAGGAACACAAACCTGAAA
73	cgd4_3770	ACAATATGAGCGCCAAGCTATT	TCTGCATTCTCTCCATTTACCA
73	cgd4_3790	AAATGACAAAGATAGCAATACAGTTGA	AGTTCAATCACTGCTCCCAGA
73	cgd4_3810	CACCCATCATCTGGTATAAGGAA	TAGAGCTTGTTGACTTGGCAGA
73	cgd4_3820	CAAAGGTAGTTCAAGAGACGGAA	GAAGTCAGAGTCAATGGATGAGAA
73	cgd4_3830	AAGCCGAGTTCATAAGACCAAA	TTTCCGGGTATCGAAGAAAGT
73	cgd4_3840	TAACCCATCTTCTTCCTCCTCA	ATCGGGTCTGAGCTTAACAATC
73	cgd4_3850	TTGAGAGAGGTTGATGTTGCTC	CCCTTTGCTATAATTGTTGGATG
73	cgd4_3860	GCAAAGACAAAGTCATCCACAA	CATTTCAATAGCTCCTCCTCCA
73	cgd4_3870	TTAGTGGCTGTAGTGAATCCCA	ACCTTCTCCACGTATCAATCAAA
73	cgd4_3950	GTCACAGCAATTCTCAAATCCA	CTTATCAGTTGCCGCCTTAATC
73	cgd4_3970	CAGTACCTGTAAGAAAGACAAGACCAA	GCTGCATTAGTTTGATGTGAAGAAG
73	cgd4_3980	TGATCCAATAATACCATCTTCCATT	AAGTTCCTTAATTGACTGGTTGAGA
74	cgd4_3990	AATGGCCCTCCAATAACTCA	CCTTGATTTCTCGTATATTTCTCCA
74	cgd4_4000	AATACATACCATCCTTACTGCTTGG	TGGCATCCTCAATAATCTGCT
74	cgd4_4010	GATACAATGGGAGGTGCAAGA	TCCATGATTAGTTGGAAGAGCA
74	cgd4_4040	GCAAACTTAACAAAGGCGGA	GTGAGAATGATCTTGAGGAGGG
74	cgd4_4050	ATTGTTGATCGTGTTGGTGAAA	GCAGTACCTAATAAAGTTGGTGGAA
74	cgd4_4060	CCAATCTGTGAAATGTGTGATGT	TTCTTTCTGTTGAATGGTGTGG
74	cgd4_4080	CTTGGAGGTTTACCAGAAGGTTT	ACACCAGATACTTTGCCCTGTT
74	cgd4_4100	ACAGAATGTGCTCCACCAAGT	ACTGTAACCAAGCTAATGCGGT
74	cgd4_4110	CTGTGTTGTTGGATGGGATTT	TTGCTTAATGGATGGTCTTTCTC
74	cgd4_4120	AGGCGAGAACAAGAAAGATGAA	ATTTGAGGCTAAGTTTGGAGCA
74	cgd4_4140	TGTAAGAAATGGAGAACCACCAG	TTGAGCATTACCATCACCAAGA
74	cgd4_4220	ATGAATACTTTGGGAGGATGGA	TTGTCGCTCTGTGAGTAAATTCTT
75	cgd4_4230	GGAGAAACAGGGAGTGGTAAATC	GCAACATACTCTGCCAATGAAA
75	cgd4_4240	AGTTTCGGTTTAGACTTGGGTTC	GAGTCCATTACTTTCGTGAATTGTT
75	cgd4_4260	AGAGTTATTGGACCCACTTGATG	ACTATTGGAACCATTCTTTCGG
75	cgd4_4270	AGTAATGCGAAGGATGGGATAA	AGAAGTGGGTTCATCTAAATTGCT
75	cgd4_4280	TTCTATTTGGGAGTGTCTTCTGG	AATGAATTAGTAGGAGCTGAAATGG
75	cgd4_4290	TTGAACATCCAGTGAACCATTT	AAATCTTGCTCTTCCTTGACCTT
75	cgd4_4300	CAGCAATGACAGTAGGGAAAGA	CAACTCTGGCTGTACCTTAGCA
75	cgd4_4310	AACAGGCAGTATATTTGGAGGATT	AACCGACTTTCTGGACCTCTT
75	cgd4_4320	TCAAATGGAGATGAAAGACCAG	ATCAAGCAAGTCGTGTTCAGAG
75	cgd4_4330	GAAATGTTGCTTCGAGTGGTTT	TTAAGAAATGTAGCCCTTTCGCT
75	cgd4_4340	TGGGATGGATATGTTCAGGATT	TATTTGCGGTCTCCTTCACTTT
75	cgd4_4350	TGATGTTAGCAGGCAAGAAGAA	TTGTTTGAGATGGACAGTTTGG

76	cgd4_4360	GACAGATGAATCCGAAGCAAA	CCAAGCTCTCAATCAAGTCCA
76	cgd4_4370	GGAAAGAACGGACTCAATAAGAAA	AATTCCTGTTTCAAGGCCAA
76	cgd4_4390	AGACTTGGGATACACTTTGGGA	TCTTCTCTGCATCTTCTTGATTTCT
76	cgd4_4400	GCAATTCATCATCCAAGCAA	TGGCTAATTCTATTCCAAACCC
76	cgd4_4410	AATACCGAGCCAAACCTAACAA	ACCTGCCTCTTCTCCGATTAC
76	cgd4_4420	CAGGAAAGTAAGCCAAGCAGA	GTTGTAGTGCCTCCATCTTTGA
76	cgd4_4440	ACGAGCCTACAATAGAAGCACAG	ATGCGAAACGATTACAACACAG
76	cgd4_4450	TATTGCACTTCTCATCGGTTTG	CTACAACAGGACCAGATACCCAC
76	cgd4_4460	GGAGTTGTGACTTGGGATTAGG	GAGTGCGGCTTAATTGTTCTTT
76	cgd4_4470	GTGTAGATCGGGACTTGGTGAG	GGGCCACCTCTCTCTAGCTTT
76	cgd4_4480	AGAAGAGCAAGATGAACCAACC	CCAGAACTCACTACCAAGCAAA
76	cgd4_4500	CCATAAATGTCGGTAGCAGGA	TAGAATTTGTTGCGTCCAAAGA
77	cgd5_20	AAATTCCATCAATACCACCACC	ACAATTCCTGAGTTCTTGGTTCA
77	cgd5_30	TGCAAATGAAAGAAGATTGAGTAGA	CTGAGGAGCTTAGAATTGGAGTAAA
77	cgd5_40	AACTGGAGGATATTGCGAAGAA	CGAAGCAGAATTAACAATCGAAG
77	cgd5_50	GAGCAGGGAATTAAGGGAATCT	AATGGCACTCTTTGTATTGGGT
77	cgd5_60	AAATCAGGAAGTGGACAAGTGG	TCATCAGAAATGCTCTCTCCAAG
77	cgd5_70	GACAGCAGTAAAGATGGAGGGA	TGTGATTGTATTTGGAGGCTGA
77	cgd5_80	CGTGGATTATGAGAGTAGTGAGTTG	TACAAGGTTGCTTATCATTGGG
77	cgd5_100	CCAGCAATAATGTCAGCAGTGT	ATAGCGGGAGTATCATGTGGTT
77	cgd5_110	TGGTGAAATAGATCCAAATCCTG	CTCTCTCTTGCCTTCCTCTTTG
77	cgd5_120	AGCCAAATCGTAGTTCTTTCTCC	GCATACGCACTTCTTCTCCAA
77	cgd5_130	AGAATACCAAATCCAACTCCCA	ACCCAATAAGTCCATGATCCC
77	cgd5_150	ATGGTAGCAGAAATACGGAGGA	GCTTCTTTGTTTCGGAGGAAG
78	cgd2_30	GAAGATTTGCTTCTCCTCCAACT	TCTTGACATGCACCAGGATTAC
78	cgd2_60	GAAGCTATTGGAGGTGTGAAGG	TGATGGAACAATTACTGAGGCA
78	cgd2_80	ATGTAGGATCAGTGGCAACAAG	GTCCAAGCAACCATATTTGAGTAA
78	cgd2_100	CATCAGCACTCCTCAAGAACA	GGGATCTAATGCCTCATACTCAA
78	cgd5_170	TCTTTCAACATCCTTCTCCAGTT	ACTTCTGACGATTCCCATTCA
78	cgd5_210	GCTGAGATACCTTGGGATAGGA	AGGATGATGATGAGGAGGAAGA
78	cgd5_220	GATGATGATGCTTATGGTGGTG	TTTGTTGTTTGCGGAGTTCTT
78	cgd5_230	AAGACAAAGAGAAGAAGCTGAAAGA	TGAATTGTTTCCATTATCACCAA
78	cgd5_240	CTTCTCAAGATTGGTCAGATTGG	TGATTGACTTTCATCCTCACTATCA
78	cgd5_300	TCAGAAATTGGTTCAGGTGATG	GGATTCTAAGCTGATTTGGAGGT
78	cgd5_310	AGATGGTATGGATCAAATTCAGG	AAAGCGTTCCTTTGCACTTT
78	cgd5_320	GGATATAGTCAAGGTGGTGCATT	AATTTCTTCATTGCTCTTTCAGGT
79	cgd2_140	ACATTGATAAGAGAGGAAGTGTTTCA	GATGATTGCTGAACCTATAACTACAAA
79	cgd2_160	TGCACAAGATATGAAAGGCTCA	TGCAATAACTCTTCTACCAGCAAA
79	cgd2_180	TGTGGAGGAGATGGTTATTCTTG	ATTTCCCTTTCCAACCTCATTC
79	cgd2_240	TACGCTCTGGTTTATATGCAGG	GATCTTTGCTTGCCACTCATT
79	cgd2_250	AAGTTAATGGATGCTGAGGTGG	AATTGTGCCTCTTCCTTCTCTCT
79	cgd2_260	AAGCTCCCAGAAGATAGTTCCC	TCGTCCTCATAGTTATCGCTTTC
79	cgd2_290	AAATAGGGTAGGCATTCAACCTAA	TGTTCGTATCAAGAGTTGGAGAAG
79	cgd2_320	AATTGGAGAGGATGATTGTACTGAA	TCTTTCCCACCCTAGCATACAC
79	cgd2_330	GTAGTTTCATTAGCATGGCAACC	AGTTGTTCGACCCTGATTCTGT
79	cgd2_340	GCAAGTTATTCAGAGGCATTCAC	CTCCATGTTGACTCCCATTCTT
79	cgd2_370	ACAGCCAGCCAGAACTCAAA	TGCACCATCAATTCTATCTTCTTCT
79	cgd2_390	CAGTCTCAAGAAGGTAATCAGGG	ATCAGGTCTTTCCAATCCTAACA
80	cgd2_440	TTCCTTTGATGGCACTCGTC	GGGAAAGTAGGGCAGCAGAA
80	cgd2_470	TCAGATGGTGACTTGGAGATTG	AATGAAGATGGTGGTTGTTGTG
80	cgd2_500	GAAGTAGGTCGTCCAAGCAGG	ACCAGAAATCTTGCTCTTCCATC
80	cgd2_510	GAGCAGAATCGTCCATATTCAGT	AGCTTCCATTTCATCCTTGTTT
80	cgd2_520	TATGCGCTATTGTATCACACCC	CCATTGCATCCCTGTTAATGT
80	cgd2_550	ACTCAAGAAGCACACAGGGAA	GCACTATCAGCCCAAAGTAACC
80	cgd2_560	TCTTGAAATGTTGAGTGAATTGGT	TTGGTTCTTCATCTGCTTCCTT
80	cgd2_570	CCTCAAAGAAAGGAGCCAAGT	AACGTACCCAACTCAGAAGGAA
80	cgd2_580	CTGACAATAAATGCAATCAGCC	ATTTCCAGAACCAACATTCACC
80	cgd2_590	TGTGCTTAAATATGCCGATAACA	CAAGAACTGAACCAGCAAAGAA
80	cgd2_600	CAAGACTCATAGAGCAGCCTCA	AAATACTCAAAGCAGCCTCCAC

80	cgd2_630	AAGAATTTGGAGGTCAGGAAGAC	AGTACCGGAGATAACAGGCTCA
81	cgd2_660	TTGGAATTACTATCAGCTTCTTTGG	AAGATCATTTGCGTTCGAGATT
81	cgd2_670	TTCAATATGATGCAAAGTATGAGAAGA	CGTGATAAAGAAGCAGAGATTGG
81	cgd2_680	TCAGCAAAGGAGTTGTTGGTAG	CTCTTCCTGTCCCTCATCTTGT
81	cgd2_690	ACGGTAATGGTAATGGTAACGG	GCCTTGTCCATATTGGTTATGAG
81	cgd2_700	CATCGTTCAGATGAGGTATCTTTG	CCTTGTTCTTGTCTTTGGTCTTG
81	cgd2_710	TTCTGGTAGCAACATGGTAATTCTT	ATGTATTGGATGCCTTCTTGGA
81	cgd2_780	AGTTACAACAGCATCAAAGCCA	TTGCATCTTGGGTCGTTTACT
81	cgd2_790	CGTTAGAAGATGGATTGGAAGG	AACCACAATTTACTGGGCATTT
81	cgd2_800	TGAAAGCGATACAGATGATGGT	GTTTGTAGGGATTAGCTGGTCAA
81	cgd2_850	CGATCTCCCTCCAGATAACAAC	GGAAGTCCTGCAATATCAACATC
81	cgd2_860	GAGCAGCAAGGAAGATAACTCAA	TCTAGCAACAGAAGCGACAGAC
81	cgd2_870	GTCTTCTTCAGGTATTTCTCGCA	TATCATCAACTCCAAGAGCCAA
82	cgd1_10	TTTATTCCTGTTGTTGCACTGG	ATAAACGCAAAGATTGAGGCAT
82	cgd1_20	GAACGCTTATGTACTCGCCTCT	TGCTATCTGTCTTGCTCTCTCG
82	cgd1_30	TCCAGATGGGAAAGTTCTGTTT	TCTGTTCTTCCTAGCCTTGAGC
82	cgd2_880	GAGAGGTAGTAAAGCGAACGGA	TTCCTGTGATTCTAATGGCAAA
82	cgd2_890	TAAGAAGGGTGAAAGGGAACAG	AGGCAGGCTTGATAGGAATACA
82	cgd2_920	TTCCCACTTCCTCCTTTAGACA	CCCTCATCAGTTAGAACAACCC
82	cgd2_930	ATACTTGCCCATTTGACTTCGT	AGTTCTCGTGCTTGTTCCTTATTT
82	cgd2_940	CAAATATAGCCGAACCTTCCAG	TTCGTCATCAAAGTCATCATCC
82	cgd2_950	ACCTAGATAGCATGAATCCACAAA	TCCAAATAACTCCAGATTTCCC
82	cgd2_960	GAGTTTCTAGGGATTGACTGGG	TAAGCATTTCCTCGTGTGTCTC
82	cgd2_970	ATATGAACCCAGAACTGCAACA	GCTTTAATAGTCCAAGGAGGCA
82	cgd2_1020	TGTAGGATTGGAGCAAATGAAA	GAAACAATATCCGTGGTGGAA
83	cgd1_60	ATCTGGGGAACTTTTTGCAGTA	CACAGGAGGAACTTTATCAGGG
83	cgd1_70	AAGGCAAATTCAAAACCAAAAA	ATAACTCTCGCTTTCCAACTCG
83	cgd1_80	GCAGAATGGCAAAATCACATTA	TTTTCAGGTTTGGGTGGTATTC
83	cgd1_90	GGGGTCTGTGAAATGGAAAATA	AATAAGAGAGGCTGCATTGCTC
83	cgd1_100	TTGAAAATAAGTTCGGGGCTAA	TCATAATTTGGTGGGGAAAATC
83	cgd1_110	TTAGAAAATTCGGCTCAGATTCA	AAAAGCCCATTCAAAACGACTA
83	cgd1_120	AGTTGCTCTCCTGATTTGAAGG	GGAATTAGAGACGAGAAGCCAA
83	cgd1_130	CTGAAGCTCGAAGAGGAATTGT	TTTCCATTTGAGTTGTGATTGC
83	cgd1_140	ATTTTGAAGCCGTAAATGAGGA	AACTTTGCTTCTTCCCACAAAA
83	cgd1_150	AGCTATGGAAGGAACTGCAAAG	TCAGAGGAGGCTCAGATAAAGC
83	cgd1_180	CTGCACTAAGAAAGGCAGGAGT	AAAAATTCCCATTACCCCATTC
83	cgd1_190	TTTAACTCAAACTCAGGCGGAT	AAATCCGTAAGCAAGGCAATAA
84	cgd1_200	CACAGATGGGATAATTTGGGTT	TGACAAACCAAGAGCCTAACAA
84	cgd1_210	TTTTGCATTCCAGCATACCAT	AAAGATAGGGTCCAAAATGAATGA
84	cgd1_220	CAAACTATGGAATGTGCAGACG	CCTCCATGCCCTGTTAGAAATA
84	cgd1_230	GAACAGAGCACCAAATGTACCA	GACACAATTTCTCTCCTGGCTT
84	cgd1_240	TATGTTTGCACGAGGATGATTT	TTGTTTGATCTTTGTGGCTTGT
84	cgd1_250	TCCATAGAATTGGTAGGGTTGG	CGAGCCTCTTGATTCTTCTCAT
84	cgd1_260	TCAATGTAGTAGGCTTGGGGAT	GGAGCCAGATTCTTCATTTGAT
84	cgd1_270	AACAGCACCAAAAAGTGAGACA	GGAATGTCACGTCCAGGAAT
84	cgd1_280	GTTTTATTCCGCAAAAGTCAGG	GCCTGGAAAAATGAAAGACAAG
84	cgd1_370	GGGAGATCAGATGAAGATGGAG	TTTCGGGTTCAGAAAAAGGATA
84	cgd1_380	CTTTTGGAGCAATAGGAGCATT	TTGATGAATTTGTATCGGCTTG
84	cgd1_390	TGATGAAAATGTCCAAATCCAA	CTTCAATTTCCTGTACGGCTTC
85	cgd1_400	ATCAAGATGAACTGGTTGCTGA	CCCTATGACATCCTGTTGTGAA
85	cgd1_410	GGCTTTTCTGTGGGTTTAGATG	GATGCCACATTTCTTGTTCAAA
85	cgd1_420	ATTGGAATAAAAGGCCCAGATT	AGTTCCTCCAACACCTTGAAAA
85	cgd1_430	TCGCAAATAAAGCAGATTTTGA	GCATTGCTTGTAGCATGTTGA
85	cgd1_440	ATTTGCCATTAGACCAAACGAC	GATTCCCATCATCCCTACAAGA
85	cgd1_450	TTGACCAATTTTTGAACGATGA	CTTGAAAAGGAAGATTCGGATG
85	cgd1_460	TCGAATGAAGGATTTTTCCAAG	GGTGGCTCCTCATATTGGTTTA
85	cgd1_480	TTTCATACGATCCCAAGTCAAGT	GGTTTCGTGAGAACTGGATGA
85	cgd1_510	GAGGAGTTGGAGGAGGGTTACT	GAAGTTTTGAAGGCCACATCTC
85	cgd1_540	TGCATCATGTGGTGTAATGAGA	ACTAGCAGCACAACTGAAACCA

85	cgd1_560	AGTGACCTTCATAGCCAAATTCA	TTAGGCTTCCTTACATCCCAAA
85	cgd1_570	TTCCCAAGATGAAGCTCTTTGT	AACTGCCATTGCTGCTCTTAAT
86	cgd1_580	CCTGGAGCAGGAAATATGAGAC	AATAAAGCAGCCTTTGCTGAAC
86	cgd1_590	ATTTTCGAGTCTTTGTACGGGA	TGGAGCCATTATATCCCATTTC
86	cgd1_610	CAGTGAACAGACTTCGATTTGG	CTGGATTTCTTGTTCTTGCCTT
86	cgd1_620	TCTTCAAAACCAGGGCAATACT	TCCTTTAATGATTGGATTTCGG
86	cgd1_650	TCATTCCGCTAATAATCCCATC	AAAAGGTCATTAAGCGCAGAAA
86	cgd1_660	GCTCAGCAGATCAACAAATCAC	TTCAATGGGCTCTGAAGTTGTA
86	cgd1_670	CAATGGAAGCATGGAGTGATAA	ATTGGCTTTGGCTGACTATGTT
86	cgd1_680	GGAGGTTGAAAAGAATGCTGAA	ACGTCCTCCTTAGTTTTGGCTT
86	cgd1_690	GGAGCAAAGTCAATCCGAAATA	TGATCCCTCTGGTTTGATGAAT
86	cgd1_700	TCAAATTCAAAATCATTCGTGG	TCTGTGGCAATATCCATTGAAG
86	cgd1_720	TAGAAGGTTTGTTAGGGGGTGA	AATCTGCCACTATTCTCCCAAA
86	cgd1_730	AATTTATGATCCAAGCAAAAGCA	CCAGTAACAAAAAGAACTGGGC
87	cgd1_740	ACACAGGAAAGGACAACAAGGT	TTGACCAACCTTTATACCCCAC
87	cgd1_750	GAAACTGTCGATACCCTTCGTC	GACATATCCATCCTCTTGGAGC
87	cgd1_760	TTCGTTAATTCATTGGAAAGGG	ACCTCAAAACCGCTAGAATTGA
87	cgd1_770	CTTTTCATCCTTGTTGAGGCTT	TGTATTATGCTTTGGGTGTTGG
87	cgd1_790	CATGAACTTGGAGAACATGGAA	TCCAGGGATTTCAGTACAAGGT
87	cgd1_800	TGGGCCAGACTTACTGAAAAAT	TTCTACAATGACCACACCAAGG
87	cgd1_810	AATTGGTGGAAGACTCAAAGGA	ATTGCAGTGGATTTGATGATTG
87	cgd1_820	TGCAAAGGAAAGGCAATCTAAT	CCATAGCCAACACAAGAATCAA
87	cgd1_830	CTGTTGATGGTTTTCAAGGTCA	TAAACCCTGCATTTGTCTTCCT
87	cgd1_860	AGGTGAGTTTGACTGGTTATTGC	AGACCCATTCCATCCTTGATT
87	cgd1_890	TTGGAGCAAACGATGACATTAC	GGCTGAATAGAAGAGTGGGATG
87	cgd1_940	AAATGCCTCCTCTGGATGTAAA	TTCAACAAAACAGACATCAGGG
88	cgd1_950	GGAGTGGTATCGGTAAAATCCA	AATTGTTTGGGTATTTGGTTGC
88	cgd1_960	AGGCCAGATTTAAGGTATGCAA	ATGGACACTCAGCCCTACATTT
88	cgd1_970	CATGAGAAACAGTTGCATTGGT	ACCCATTGATGTTGTTATTCCC
88	cgd1_980	TCTGGATGGTTTCGTTCCTACT	ATCCCATTGTTTTTGTGAATCC
88	cgd1_990	GGGCCAACATCTGATAGGTTTA	ACATTTCTTGGTTTTCGTGCTT
88	cgd1_1000	TTGGAAACAGCAATGTGAGAAT	TTCCAGAACCAAGATTACACCA
88	cgd1_1010	TTTGACCATGACGAATTTATGC	GATCTTGCCGAAGGAATTGTAG
88	cgd1_1020	CTAGTGGGATAAAGCCATCTGC	TCTGGCAAAAACTAAAGCTGAAA
88	cgd1_1030	CTCCACATACATCACTTTCCCA	GTGTTTCCTGAGTCATTTGTTGTC
88	cgd1_1050	TGGATTATTTGTGGGGTAATCTTT	AGATCCAGAGAACACAGGAAGG
88	cgd1_1060	ACGGAAAAGAATAGCCAAATCA	TCTTTCCTCATACCGTTTCGAT
88	cgd1_1080	TTATGAGAGTCCCCAAACTGCT	CCATGAAAATCAAAACGAACAA
89	cgd1_1090	CATGAAATGCACAAGAAGGAAG	GCACTATTTGTTCCCATGTTGA
89	cgd1_1100	GGATTCATGCTTTTTAGGTTGC	ACTCTTCCTGCTACCCCACATA
89	cgd1_1110	AACTTTTATGAATGCTGGTGGG	CCAAGTCCATTACGGCAAGTAT
89	cgd1_1120	TTGTCAAAGCTATAAGGGCACA	CTCGTCACTCCTATTGTTGCTG
89	cgd1_1130	CGCATATTCCAATTCAAGAACA	CGATTTTAAGTCCGTTTTGGAG
89	cgd1_1140	AAGTTCTTCTCAAGCATGGAGC	AGGACCTTCAAGCCATAAGTGA
89	cgd1_1150	ACATGCTTTGAATGTTCTGCAT	AGTTGGATTCTTCTGAATTGGC
89	cgd1_1160	TGACGATAAAAATTGCATGGAG	TTCTTGGTATCCCCTCCCTATT
89	cgd1_1170	CTTCTAAGTTTGGGAGGGGACT	AAAATCATGCCTTCTTCTCTCG
89	cgd1_1180	CAAGATGGCTGTTGATATGGAA	CCCATTAGACTCTTTGGTTTGC
89	cgd1_1190	ATTTCGGCCTGTGTATCTTCTC	TTGTAAAAGCCCTCCTATACCG
89	cgd1_1210	TTGGAGAATTAGCAAGGGTCAT	CTTCTTGCATCTCCTGGATCTT
90	cgd1_1220	CAAAACGCTCGTATCTTCCTCT	GTGAAGGTTTCCTTGGAGTTTG
90	cgd1_1230	TATGACAGCCAGAATTGGTCAG	GGTTCTCAATGGACCCAAAATA
90	cgd1_1240	ACCCCTTCAGATTTTACCCAAT	CATGGGAGTTTTGTGAGATCAA
90	cgd1_1250	AGTGGTAAGGCGTCCAGAATTA	TGGCCTCATTTCAACATAACTG
90	cgd1_1260	GAAAGCCAGAAGGAGAAAAGTG	TGTTACCAGGATCTGAACCAAA
90	cgd1_1270	TTAACTTGGTCAGGCGTACCTT	TATTGTCCTCAAATCCCATTCC
90	cgd1_1280	GACCAGAGGAAGAGTGTCCAAC	TAGATCCTCAATGTGGTCATGC
90	cgd1_1290	TGACTTTGATCGCTTGGTAGAA	AACCTGTGCGGCAATAGTTAGT
90	cgd1_1300	TTTCCGAACCTCCTATGAAAAA	ACAAATGGCTTTCAGGAACACT

90	cgd1_1310	ATCTATTAGGGTTGACTGGGCA	TGAGATGGCGCTGTTAAAGATA
90	cgd1_1330	ATCAAAAATTCGGCTTCTCAAA	TCTGGCATCAAGAAGATAAGCA
90	cgd1_1340	TTTTGGGTAATAAGCTGGATGG	GCATTTTCGACCTCTATGGAAC
91	cgd1_1350	CATCGACTTTTGGAAATGAACA	ACTGCCAGAAGATCCAACAAAT
91	cgd1_1360	TCACCGGAAAACAATGATGATA	TTTCGGAACCTTAATCTGTGCT
91	cgd1_1370	GATGTGAATAGTGATGGCTGGA	CCATCTCCGTCTTTATCTGCTT
91	cgd1_1380	TGACCAAATCAACAAATATGGC	CACCACTACAAAGATGACAGGG
91	cgd1_1390	CCAGGTTCTCTCTGCTCATCTT	CCTGGTCAACCTCTTCAAAAAC
91	cgd1_1400	TATAATTCCGTTTCAGCCCTGT	AGCTTGTGTTCCCTGATTCATT
91	cgd1_1410	TCAGTCCAATCATTCGTTTCTG	AAGGCTTCGTTCTTGTTCTTTG
91	cgd1_1420	AACGTATCCATTCCATTCTTCG	TTGCCAACCTTGTACTTCCTTT
91	cgd1_1430	GAACTCAAATCAATGCAGCAAC	AATATGGCTCTTTTGAAGGATCAG
91	cgd1_1440	TAGAAAGGAGCTTGGAATCGAG	ATACATTTGCTCTTCCAGCCAT
91	cgd1_1450	TCAAGCGAAGAAGCAATTAACA	ACGCTGGATTGTTTGAGGTTAT
91	cgd1_1460	ATTAAACGACATTGGGAATTGG	CCTGTTCAACTTCATTGCTCTG
92	cgd1_1480	AAAATACAGCAAGTGGGGCTAA	CGCAGAATTAGACGATGATGAA
92	cgd1_1490	GTCCATTAACGATGCAGAAACA	TTCCAGACAAATAGCCTTCCAT
92	cgd1_1500	AATTAAGAAAAACACGAGCCGA	TACCAGAAGAAACCTGTGCTGA
92	cgd1_1510	CCCAGAAGTAACAACTCCAAGG	AATTCCAGCCCAATTATCTTCA
92	cgd1_1520	ATTGGAAAGAAGATGAATGCGT	AAATGCCCAAATAGTCCAAATG
92	cgd1_1530	TTTATGGCACTACGAACAAAGC	GGTTGAGTAAGGACCAAGAATGA
92	cgd1_1550	ACGATGAGGGACTCTTATGGAA	GCTATTGGAAGTAATGGATCGC
92	cgd1_1560	GTGAAAGCAGTTGTGTTTCTGG	GAGAGCTAGACGGGCTATTTGA
92	cgd1_1570	ATCCTTGACAATAGCGACGAAT	CCCTAGTAAACCTTTGCCTCCT
92	cgd1_1580	ATGATAGCAATAGCGGAGGTGT	TACAATCTGCCTCTAACCAGCA
92	cgd1_1610	TTAAGAAATCCAAACATTGGGG	CTTGACTAATCTGCCTCCATCC
92	cgd1_1630	AGAGATTAGGTGGGCGGATATT	AGCGTTGAGCTTTTGTTGTCTT
93	cgd1_1640	AACTGAAGCGTTAATTTCCCAA	AATGGATTGAGTGGCTTCCTTA
93	cgd1_1650	TTTAATGCAGATTGGTGTAGTTTGA	TTTGCCTGAAACAAAAGACTGA
93	cgd1_1670	TTGGCATAAAACTCTGCTGGTA	TTAGCGAGTGAGAAAGTTCACG
93	cgd1_1680	AAAGCAGGTTTTACTGTTGGGA	ATTCATCGTAGCTTTCAGGAGC
93	cgd1_1690	AAACAAAGGAGATTACCGCAAA	CGCTAGGTATTGGGTGAAAAAC
93	cgd1_1700	TATACCTCCTCTCCATGCCAGT	ACAAGAACTCAACCCTACCGAA
93	cgd1_1710	TGCTGCAAAAGATGAAGAGAAG	GCTGTTTCCTTAGCCCTTGTTA
93	cgd1_1740	AGCTAAACAAAGGCTAAAGGCA	ATCTTGAGCTTCCATTTCTCGT
93	cgd1_1750	TTTTGGGGTGTAGTATGTCGAA	CATCCCACTCAATTTCATTGTC
93	cgd1_1760	ACTGGTGAAGAAGTACCCCTGA	CCACATCATTTTGTTGTATGGG
93	cgd1_1780	AGACCCACACTTATCCATCAGG	AGTTTTTATCGAATCAGGGCAA
93	cgd1_1790	ACATCTTTAATTGGCAAGGCAT	TACGGTCTTGGCCTGAATATCT
94	cgd1_1800	ACCGTGGAGAAAAGATCAAAAA	GTCTGTCAACGAATCATCGGTA
94	cgd1_1810	GCTATTGGACAACATCAACCAA	CAAGTCACGCTTTGAGAACAAG
94	cgd1_1820	GCTTGGTCCATTTCTTTAATGC	CATTGACATAATTTTTGATTCCCA
94	cgd1_1830	TTTGGGGCGGTATTCATATTAG	ATCCAATTTCCCTGGAGTTTCT
94	cgd1_1840	GAATATGGCAACGGATATGGAT	CACTGATAACGTAAATCGCCAA
94	cgd1_1850	CTGAAACATCGTCAAGTTCTGG	TTCCCACATCGAATATCTTGC
94	cgd1_1860	TTTGTTTGTTGTTATGCCAAGC	ATCCCCCTTTTAGTCAGTCCAT
94	cgd1_1870	TGTTGGAAAATGATGATGGAAA	AACCCTGCTCCAGATGAAAATA
94	cgd1_1880	GGGAAAACCCCACACTTTTATT	ACCTGCATATTATTGCGATTCC
94	cgd1_1890	GCGATGGCCTAATTCTTACTGA	AAAAGCCAACTTCTTCGGGTAT
94	cgd1_1910	CAAATGGGCAGAAATTACCACT	ATTTTACATAGAACGGCATCGC
94	cgd1_1980	TGGATGGAGAAGTTGATGTCAG	CTAAAATGCAAAGCCCAGAAAC
95	cgd1_2010	CAATCGCCCTCTACCTACAAAC	TGGATCGCTCTTTTGGTTATTT
95	cgd1_2080	TTTAATGGGAGCTTCGGTCTTA	TATCTTTTGAATGGTCGTGCTG
95	cgd1_2090	CTGGGCTCTGAGTCTATCCAAT	TTTCTTAGGGGACGAATGAAAA
95	cgd1_2100	TTGTGCTTGCTCCTATATGGTTT	AAATGGGAAATAGCCTCGAAAT
95	cgd1_2110	TTATTGGGCCGTTTAGTACCAC	TCTTTATGCCCTTTGTTTTCGT
95	cgd1_2120	TCAGATTTTTGTGTCCATCCTG	TGCAATTAGCCTCACACATTCT
95	cgd1_2130	GCCAAAGTGATTCAATAGAGGG	AGAGGGTAGGCATTCTAGGGTC
95	cgd1_2140	TATTTAAGCACGCAAATCCAGA	CCTCCAGTTTGAAGGCAATAAC

95	cgd1_2150	TATCTCACTCGCAAGAAGGACA	GATGACCCCCTACAGCATACTC
95	cgd1_2160	ATTTGAAAGCCGCATAGAAAAA	CAAGTCTTTCCACAAGAAACCC
95	cgd1_2170	TTAAAGGTGCGTGTATATGCGT	TTCAGCCAATGGAAGAGTGTTA
95	cgd1_2180	GCGCAAAGTAATTCAAACAACA	TTTCCCCATACCATTTTGAGAC
96	cgd1_2200	AGCAATCAGCTAACCCCAGTAG	TACGTTGCGCGAATCTAAATAA
96	cgd1_2220	TTAGCACAGGAAGTGTTGGTTG	GATATGTTTTGGGACCACGTTT
96	cgd1_2250	TTTTGGCTCAATTCACTTTTCAT	CCATCTCTCATAGTTGCGTTGA
96	cgd1_2260	ATGGGATAACTCTGGACCTCAA	GCCTACCAAGTTTTTGTCTGCT
96	cgd1_2280	AATAGGGGGAAACACGAGAAAT	ACAGTCCATCTGTTTTGGAGGT
96	cgd1_2290	AAAGCCAGGAGTTGACAAAAAC	AGAAGCTACCACTAACGCTTGG
96	cgd1_2300	CAACTTGCATTATACCGTTGGA	AAATGAATTGCACCCAAGATTT
96	cgd1_2320	GGAATGGAAAAGAAGTGGAGTG	ACTCACAAGCGTCATCTCTGAA
96	cgd1_2330	ACCGATTACAAACGCAGAGAAT	CACCGCATTCAAAGAGGATTA
96	cgd1_2350	ATAATAAGGCACCCATTTGTCG	TGGGATTGATAAAATGCTGTTG
96	cgd1_2360	GAGATTCTTGTAGAGGAGGCGA	AGGAAGTTCACCACCGACTTTA
96	cgd1_2370	TACAAAGAAAAAGGGCTGGAAG	GAATCCAAAGATGAAGAAGCGT
97	cgd1_2400	AGCGAAGTTCTTTCGATTCAAG	TCAACTACAATAAATCGGCACG
97	cgd1_2420	TAAGCTGTCATCCGTCCCTATT	ACATTCACCTCCATCCAAAATC
97	cgd1_2430	TGACAGCATTAAATCGTATGGC	GGTCGAAAGCAGAGAGTTGATT
97	cgd1_2460	GTTATATTAGACCGCAAAGCCG	AATTGACACTTGTTGCATTTGG
97	cgd1_2470	ACTTTATTTGCGGGAAAACTCA	ATCGGCTGTTTTATTGGAGGTA
97	cgd1_2490	TTCAGGGTTAGATGAACAGGGT	CTCTCACTGGCACTCGTAATTG
97	cgd1_2510	ACGGAACATTTATTGCGACTCT	AATGGGAATATGAAGCCATTTG
97	cgd1_2530	CCTTGCTGTGACCAACTATCAA	CATTTTTACCTGCTCCTGGTTC
97	cgd1_2540	GGCAATTTATCAAAACTCCTGC	AATGTCAATCTCATTCCCCATC
97	cgd1_2580	CTCTTGCTAGGAGGCTGTTGAT	GACTCCAATAATGCCGTAGTCC
97	cgd1_2590	GGGACGACAATGGAATAGAAAA	GGGTTTCTTTAATGCTCAGTGG
97	cgd1_2600	GTTGTTTGAAGGAAGTTGGGAA	TGAAGCCCCCAGTTGATAATAG
98	cgd1_2630	GAAATAAGCCACCAACAAAAGC	AATAGCTTTGCATCTTTGGGAA
98	cgd1_2640	AATTAACATGCCCTCAATGCTT	TTGGTGCTTTGTAGAATGTTGG
98	cgd1_2660	GCTCTAATTTATCGCCCTTTGA	CGCATACAAAACAAAGTTCAGG
98	cgd1_2690	CACCCTTGTTTCCTTTGATGA	TCTGCCATTTCCTCATCTTTTT
98	cgd1_2700	AAACTTACTGTGGGCATGGAGT	ACTTCCATCTAATGTTGCCGAT
98	cgd1_2740	TTTGACGACAGCAACATTAACC	CACAAGTCTTCCCCTTCTATGG
98	cgd1_2750	TCAATTTTTATTGGGAAAGGGA	CTGTCTTGATGTGAAACGGAAG
98	cgd1_2760	CGAGGATGAATGTACCAGATGA	TGGATATTGGAAAGAAAAGCGT
98	cgd1_2770	TAACCTGCTTCGAGATACAGCA	TATCTGCAACAACAGTCCATCC
98	cgd1_2780	ACTGCGGATACAACCACTTTCT	GCAATGATTTCCAAACGAAGAT
98	cgd1_2790	GCCAGACTATCAGTACCAGAGGA	GGATTTATTCCAACACCTTCCA
98	cgd1_2800	AGTTTCATCTTTTCCACCTCCA	TTTAACATCATCAACTCCGCTG
99	cgd1_2810	GAATTTGGTAAAAACCCCCATT	ATTGCATTTCCAGCTCCATACT
99	cgd1_2830	TATTGGCATCAGAAATTAGCCA	GTTGAGCATGTTGCTGTAGGAC
99	cgd1_2840	TTGGCAAAGTTATTGTGACAGG	AATTTTTCCCTTTTGGTTGGTT
99	cgd1_2850	TCTGCATTTAGAGCAATAGCCA	ACTTACATTCTGCCTCCCGATA
99	cgd1_2860	TGAGCAAGGACAGCTAAAATCA	ACATAGAAATCTTCCCCCAGGT
99	cgd1_2870	AATTTCATGGTTAATGGAACGG	TTTTGTGACGAACTGAAACACC
99	cgd1_2880	TGCAACTAAACCTCACCTAATGAA	TCTAATGGCAGTGTTTTCAACATAA
99	cgd1_2890	TCAATTCCCCTTTAGCAGGTTA	GTTATGCCCACTCCAAACAAAT
99	cgd1_2900	TGCTAGAAATTGCATCCAGGTA	GCAGATGTGATTAGGAGTGCTG
99	cgd1_2910	TTATGGTTGGGAATCTTATGGC	TAGTTGATTGATTCTTTGGGGG
99	cgd1_2920	TCTAAAAATGAGGCAGAAAGGC	CGATCCCAAATCTTTTCTTGAG
99	cgd1_2930	AAAAGCAATGTTGGGCTTTCTA	AGTTCTTGGAAATGATTGGGG
100	cgd1_2940	AGAATCCATTGACCTATTGGGA	CCAAATCTTGTTTCCCATTCTC
100	cgd1_2960	ATGCTAGTGGAGAGGAGTGGAG	TTTGGACCTGAAACCTCAAAGT
100	cgd1_2970	ATCATCATCTAACGCCACTCCT	GGAATCCACCTCCTTCTTTCTT
100	cgd1_2980	TAATCCAGAGGGAGCAATGAAA	GTTAGCAGACCACTGCATCCTT
100	cgd1_2990	AATGAACGCAAGAATGGTAGGT	AGGGAAATTAAAATGTGTTCCAAA
100	cgd1_3000	AAGAAGGCCGTTGTCGTAAATA	CCTGGAGAAGTGGCTTAATGTC
100	cgd1_3010	TATCCCTGCATTCTTAATGGCT	GGAAGTGACTGGTGAAAGGAAC

100	cgd1_3130	GAGGATTTTGGGATGGATGATA	AATGCTGCTAAAACATCCGACT
100	cgd1_3150	CATGCAGAATTTTTGACACTTTTC	ATTTAACCACTTTGCAGCATTACA
100	cgd1_3170	AGTTTGATTTGAACGATGGCTT	CTTCCTCTTCCTCTTCCTCCTC
100	cgd1_3180	CCATGCAAATCCCGTATTATTT	TGTTGAGTGGGGTAGAACCAG
100	cgd1_3190	CCATAAGAACTCCCTTTGATCG	CTACTCCAAAAGAGTCGTGCCT
101	cgd1_3200	GCGCTCAGAAGAACGTAAATATC	TGGAGCATAGAATCCACCAATA
101	cgd1_3210	CTCCGATTTTAATTCCTCCTCC	ACAGCATCAACCACATTCTTTG
101	cgd1_3220	CCAAAGTCAATTCCCTCAAATC	TGAGTGTAGATGGTGAAGGTGG
101	cgd1_3230	TAGGAATGGGAGCAAATTCTGT	ACTACTCGCTGTTCCAACCATT
101	cgd1_3240	TTTGGCAGAATTTATGGGAGAT	TTATGGCTTGTTGAAATTGGTG
101	cgd1_3250	TTTGGTTATTAGTATTGGTGCCG	CCCATTGAAGGAAGTGATGAGT
101	cgd1_3260	CTTTTGAGAGAAAATCTGAAATGGT	TAGATCCTGGTGGTCTTCCTCT
101	cgd1_3270	AACTTGGAAAACAAGCAAGAGG	CAGAAAGAAGTGATTGAGCACG
101	cgd1_3290	GAAAGGAAGAGGGAAAGGAAGA	AGGTGATGCCAATGTTGATGTA
101	cgd1_3350	ACATCGAGAATTTGGTTGGACT	ACCAAGTATTGTTTTAATTCATGCTG
101	cgd1_3390	ATTTCTTGTTCAAATGGATGGC	ATACACGGGATTCTTCATCTGG
101	cgd1_3440	TGTTGACAGGACCAGCTAATTG	CACTCATTACTGCATCAGCTCC
102	cgd1_3480	TTCAATCCTTTTGTGTGACTGG	CCTCAGCATCCTTCAAAATCTC
102	cgd1_3490	GAAGGACCTCCACAACAAAGTC	TCCAATAAAAGATCCAAGTCCAA
102	cgd1_3510	TTGTACGCATTTCAATTTCAGG	GTTTGCTATATGCCTTTCCGTC
102	cgd1_3530	CGTTCTAGGGGTTGTGGAGTAG	TTTAGTGGAGGTCTTGGAGCAT
102	cgd1_3540	CTTGGTTTACTTCTGCTTGGCT	TTGCAGATTCGTGATTGTTACC
102	cgd1_3550	GAATGAAAAGGATGGTGATGGT	TATTTCCATCTCAGTGGTGGTG
102	cgd1_3570	GGAGAATTTGATGGAGAAAACG	TTCAGTTGGTGAAAGCCCTAAT
102	cgd1_3580	TGCGGATTTTCATTGTATATGATT	ATCATCTTCCGAATCTTTAGCG
102	cgd1_3590	GGAAAGTTAAGTGGGGAAGGAT	CTTATCATTGTCATCAGCCCAA
102	cgd1_3600	ACAGGATCAAGCTCTAAATGGG	ATGATCGGTAAAACAGCATTCA
102	cgd1_3610	TGGTGCTCCTTCTGAAACATTA	ATAAGCCCAGCAGAAAATGAAC
102	cgd1_3620	AACACCGTTTGAAGACTTTCGT	TTTTGGTAAACAATTTGCATCG
103	cgd1_3630	TGTAGAGATGGCTTGACTTCCA	ATACAGAACTTTGCTCCGCTTC
103	cgd1_3640	TTGGTATTTGGTGGTGTCAAAA	TCCGCTTACTGTAGATTGCTGA
103	cgd1_3650	GGCCGGATATGATTTTACTGTG	AACCAGCTTCAAGGTTTTGAGA
103	cgd1_3670	ATTCAGCGGATTTATTGGAAGA	CATTTAGTGGAACCAAAGCTCC
104	cgd1_3750	GGAGGATTTTCACAAGGATCAG	TGATAAAGCACCACAGGATCAG
104	cgd1_3790	ATCCACAATTTTCTTCAGCGAT	TTTAGCTCATGCTTGGCATCTA
104	cgd1_3820	AAAAAGGGCTTCCACTAAGGAC	GTATTGTTTAGAATCGCCTCCG
104	cgd1_3830	AGATCGGCACAAGAACTAGAGG	AGCTGCACTTGAAACGTGATTA
104	cgd1_3840	CCATGTCAGAATTTGGAAGTGA	CCTTACCCCACCAAGATCATTA
104	cgd5_380	GCATCTTCAACGTCTTCCTCTT	CTGTTCATCTGCTTGTGTTTCC
104	cgd5_390	TGTTGACCTTTCACAAACCAGA	AAGAATCCCATCCATGAACTCTAA
104	cgd5_410	TGTCAACAATAAACCCAAACGA	CTTGGAAGAAGATGCTCCAGTT
105	cgd5_420	TTCAACTCCAACTCCATCTTCA	CTCTTTCTTCCAATGCTCCATC
105	cgd5_450	CAACGATAGAAGAAGGTCACACA	GAAGAAGAGGATGAGGATGAGG
105	cgd5_490	CTCAACCGTAATGAATGGAAATG	TCCACGGAAATTAGTGTATCAGG
105	cgd5_510	TGCACAAGAATTACGTGGTAGG	TCCTTCTATTACACTTTGCGGTG
105	cgd5_520	CGGTAGATGAAGGAATTAAGGG	TGCACGAATTTGATTTGCTT
105	cgd5_530	CAAGAGAAGAAGAAGGAATGGG	CTTGATTGTATCGTATGAGGAGGA
105	cgd5_550	TGAAGATGGAAATGAAGATGGA	TAAACAAAGAAGCCCAAAGCA
105	cgd5_560	GGGCTCAGAAAGTTAAAGGGAA	TGTGTTTGTTTGTTTCCAGATTGT
105	cgd5_580	TGCCAAATACACTGGTAATGGA	GCATGATTCTTTAATGGCTTCAC
105	cgd5_600	ATGAGCGATTGGTTACTCTGTG	GAGCCATTCATTAACTTTCTCTTTG
105	cgd5_610	ATCAGTACCTTCCGATTCTCCA	ATATACAGCCTCTCCAGCCAAA
105	cgd5_640	TTCCAAACATTCAGCCAAGA	ACAGGAACAGGCACATATTTCA
106	cgd5_650	AGATGGAGAACGGACACTACAAA	AATATGATGGTGATGAGAATGCC
106	cgd5_660	GAAATCGGTATAGAATCGCACC	GCATAGTAATCTACCAGAATGGGAA
106	cgd5_670	GGAGTTAAAGTTGGCAGAATGG	GGCATCGTAATCTTTGGACCT
106	cgd5_680	TAGGATGGTTCAGTTGCATGTT	CTAATGATGGGTCACAATTCCA
106	cgd5_690	AAGGCGTTGGGTACAGTTCA	TGCAAGCATTTCAGTTATTTGG
106	cgd5_700	TCTCCTCAGATTGTTGATGGAA	ATTATTCGCGTTAGTATTGCCC

106	cgd5_710	ATTTGGAGGAGTGAATAACGGA	AAATGGGACCTTAGTGAAGCAA
106	cgd5_720	TTTCTCATTCCGACTTACTCCC	TAAATCCTGCCCATTCACATAA
106	cgd5_730	AGCCACCAACAATTATCAAACC	GCCTCTTCCTTCTCTCCATCTT
106	cgd5_740	AAAGGTCACTCAAAGGAAGGGT	ATCCCAAAGATTGATAATGCGA
106	cgd5_750	AATTGATGGCACCGAGTTTATT	GCAAATATCGGGAATGTGAGA
106	cgd5_760	GAATCTCAAGGATGTTTGCCTC	CCTGTTAAAGTCCGTCCATCTC
107	cgd5_770	GTGAAGATGGTGTAATTGTGGG	TCCTCGGTAATAACTGGAACCT
107	cgd5_780	TCAAGAAGGTGACAGAGCTGAA	GGGAATGGTGGAATAAAGAACA
107	cgd5_790	CAATGATCCCAAACAAAGAGAA	TGTGTTCCAAATATCTCCCAAA
107	cgd5_800	ACTCAAGGGCGACTAGGAAAG	ATTGGTCATTTCATCACAACCA
107	cgd5_810	ATCTATGTCGATTCTTCCGCAC	CATGGGAACTCCATAACCTGAC
107	cgd5_820	CGAATGGAAGAATGTCTCTGAA	AGGCTTGGTAGCTCAATACCTG
107	cgd5_830	CTATTTGGTTTGGGAAAGACGA	TGAGTGTTTGGAATGAGACCTG
107	cgd5_840	AGAGCAAGAGGTGAAACTCCAA	AAGCAAATCCCAAATTCCATC
107	cgd5_870	TGTGCGTGCTAAAGGATAATACA	TGGGCTAGACTTTCAATTCGTT
107	cgd5_880	AATTGGAATGTAATGCTGATGAGTT	ATAAATATCCGAGTCCTCTCCTTGT
107	cgd5_890	GCCGAATCTGAAACAAGTGAA	GAAGCATTATCCAGCTCCTCAA
107	cgd5_900	AATTTGAGAGAGGGCTTTATTCC	GATCTTCCCAGACAGTTCCAGT
108	cgd5_910	CATGGAACAACTCATAAGGCAA	TGGTGCAGAATACAATCCAAAG
108	cgd5_930	TTGGAAGAAGCGATTGAAGTGT	TCGCGGTGTTTACATCCATAA
108	cgd5_940	AGTAAGAAGCAACCGACTTCCA	CTTTGAAGCATTACCAGCCAA
108	cgd5_950	TAAGGGCGATCCAAATAAACTC	GATGTTCCCAAGAAATGTAGCC
108	cgd5_960	CTTGCACGAGAAGAAAGCAAC	ATGAATATGATCCACCACCTCC
108	cgd5_990	TGGCAATAAGCCCTTTAACAAC	GATCACGAATTGGATGGAAACT
108	cgd5_1000	CCCGGAACTATTGGATTAAGAA	TGGGATACAGTCACAAGAAGGA
108	cgd5_1010	GCTGCAAACCAATCAATCTCTT	CTGTTCTTGACCCATTCCATCT
108	cgd5_1020	ATATGCTTGACACTCAGCTCCC	ACGTTGCTTGGTAAAGTCCAAA
108	cgd5_1040	TCATTTGCTGTATTCATTTGCC	CATTTCCACCAATATCCATCCT
108	cgd5_1070	TTCAAACATTCAGCTATTCCCA	TTGCTCGGTCTTTATTAGTTCGT
108	cgd5_1090	GTATTAGAAGTGGGAACGCGAA	TATCCAAGTGGTCCTTTCAACC
109	cgd5_1100	AGATGAAATTAGCCCACAGGAA	TAAATGCAGGGTGTATGGTGTC
109	cgd5_1120	GGAAACAACGTACACTGGAACA	AAGAATTGATGGGTCAGGAGAA
109	cgd5_1130	CTAGCAGTGACGAAACATGGAG	ATATCCCAAATCCTGAATGCC
109	cgd5_1140	TTGATGTAAGAGATGTCGCAGAA	TGGCTATGAAATTAGGAAGGGA
109	cgd5_1160	TAAACTGCGATGGTGATATGCT	AGAAAGCGAGACTGTTGTACCC
109	cgd5_1170	GCTTCATACTGGGCTTTCTCTT	TTGAGCCAATAGTGCCAGATAC
109	cgd5_1180	GTAAGAGGCCATTTGAAGCAAC	TCTGCAATTCCCATACAGTCAG
109	cgd5_1190	CTGGAATGAAAGAAGCTGAAGG	TGACCTCGTAGTTAGGGTTGCT
109	cgd5_1200	ATCACAACAGATCGCATTGAAC	TTCATCAACACCACTTCCTCC
109	cgd5_1210	CACTACAACAACAACCGCCA	ATTCGTTTCCTTAGTCGCTTCA
109	cgd5_1230	TAGAAGGCTGGTCTGTGTTTCA	TGAGGGAGTGTCGTGTAGTAATGT
109	cgd5_1240	GCGGCATCAGAGGAATAATAAG	GAACCATGTAATTGGGAACCAC
110	cgd5_1250	ACTTTGAGCAAGCCTATCGTTC	ATAAACGGGTTCCAATAAAGCA
110	cgd5_1260	TCAAATGCTCGTTAAATCAATCC	CCAAGTTAGTATTCCGCTCCAA
110	cgd5_1270	GTTTCAGTTGTCGCGTTATTCA	TCAAAGCCAAAGAACCATAGAAA
110	cgd5_1280	AGCAACAAGCAAAGAATCCAAG	CGTGCGAGATTATAGAGGCATT
110	cgd5_1320	CAATCCACAGCTAATTCCCAA	TATCCGCCAATATCCAATCTTC
110	cgd5_1340	ATTCAGATTCCGCTTATTTCCC	TTACAGCAACAGTCGAGACGAG
110	cgd5_1350	ATCGGGTCTTCCTTACGACATT	AATCTTTCTGCGAGCTTCCATT
110	cgd5_1360	AATTGTGTTTGCGATTTCTGG	TTGGTGGACAAAGGTGTTGA
110	cgd5_1370	ATTAGTTCGCTTCCATCCATAAA	AATATAGTTCGGCACAGTTTGAGA
110	cgd5_1380	GGAAATAATCACATCACATGACAGAA	TATTGAAACCCTTCCCAAGCA
110	cgd5_1410	ACCAGTATATCTTTCTTGGGTGAATG	TGAAGGCAAAGTTGGGTTAATG
110	cgd5_1430	AAACCTCAATGGCAACGTACTC	TTACCCATGTATTCCTCATGTCC
111	cgd5_1440	ATAACAAGGAGCAACAGGCATT	TCTAATTCTCCGATTCTCAACCA
111	cgd5_1450	CCAAACATTCTTGATAGTCGCA	ACGTTCATTCGTTTCTTGGTTT
111	cgd5_1460	ATTCCAAATAGCGACCAAGAAA	TCTGGATGAGTATCAACTGGGA
111	cgd5_1480	CATTCACTCCAAGTCTCACACC	GTAATTCAAGTGCAAGTGCGTC
111	cgd5_1490	CCCAGTGAAATATGGGTAAATGA	TTTGCCATTCTGGTAGCTCTCT

111	cgd5_1500	TGCTTATGACGCTATGGATTGA	GCTAATTGAACCAGATACAGCGA
111	cgd5_1580	TCGGCTATATTGAAACACCAAA	AGTAAATGCCTTACGCTTGCTC
111	cgd5_1590	CCCTACAAATCACGAAAGCAG	GGCCTTAGTACCAGCTAATGGA
111	cgd5_1600	TAAACAGCCTTCGAGTTGCC	GCCATATTCGTGTAGGAGTTCATT
111	cgd5_1610	CCCGGAAATTAAGTGTCTTGTT	AATGTTGTCATTGTTCGTCACC
111	cgd5_1620	AAGCATTACCCGAGACATAGGA	AATTAGGCTCGTTTACAGGCATT
111	cgd5_1630	TGTGTTTATTATTCCTGCGTGG	TCCTACAATTCCCGAAAGAGAG
112	cgd5_1640	ATGAAGGTGAGTATTCCAACGG	ATGACCACACTTAACGCCTCTT
112	cgd5_1650	TGAACGAACTTAATAGGCCAGAC	TTTCCACTCCACACAACATTTC
112	cgd5_1660	TAGGCTGCAAAGATGGTAAAGG	TAAGTTCATTTGGGATTGTCGG
112	cgd5_1670	TATCTCCAACGGACCAAACAG	CCATAAGGATATGCAACAAATGG
112	cgd5_1680	ATCGCATCAATAGTTCGTCCTT	ACCGTACTGGCACAAATCATC
112	cgd5_1690	AGGACTATCCGCCTCTATCTCC	TCTTGCCACTCAGGAATTAACA
112	cgd5_1710	ATCAGGCCGTATTAAGTCACCA	TCCTTTACCTAGTCCGCTCAAA
112	cgd5_1720	TGATCCTGCTACTTCGGCTACT	TCCCTTCGTGTATTTGTCCATT
112	cgd5_1730	GCACACCTGATAGAAATAAACGG	ATGATACTTGCTGCGTCCATAA
112	cgd5_1750	GAATACATGGTGAGTGCGATGA	CTTGCGAATTTGATGTCTCCTT
112	cgd5_1760	TAACTGAATTGGGAGAGGTGCT	ATAGGTGTTGATAGGGCCAAGA
112	cgd5_1770	GTGACCCTATTTCATTGTGCTTAGA	GCAAGAGGAACTTTGGGAGG
113	cgd5_1780	CAGATATTCATGGCGCAACTAC	GCATATACTCATTTGTCCTGCCT
113	cgd5_1800	TCAAAGTCAGTCAACTCATCACAA	AGCGTTATAGCTCAGGTCACAAG
113	cgd5_1810	ACAAGATTGAGGATGAAACTGAAA	GTCGAAATAATCCCTTAAACCAAA
113	cgd5_1820	TGATAATTGATAGGCACGTTGG	CGGAGAAATCCAATGCTAAATC
113	cgd5_1850	GTGCGACCTTACAAGCCAAC	TCCCAAATAAGTGCTACCGATACT
113	cgd5_1860	GCCAGATCAATAAGTTCCATATCA	CCAAAGAAGGTAATTCAGAGCC
113	cgd5_1870	CAATCTCAAGAGTTATGGGCGT	GTCCATTTGATCTGTTCCATCC
113	cgd5_1880	CACGAGGTGCAAGATATGAGAA	TGGTGAAGTATCAAGCCATCAA
113	cgd5_1890	TCCTGGGAACTCGTTATTTCA	AGCTTGTGGATTTATTTGCGTT
113	cgd5_1900	ATTTGAAGTTTGTCGGCGTATC	GCCTCTCTTCTGTCATCAATCC
113	cgd5_1910	GTGCCATTATTCTCATCAAGCC	CCTCTTCCCATTCTATTTCTCTTACA
113	cgd5_1920	GGTCTGTTATTGAGCCTGGAAC	AATCTGAAAGCCCAGAAATTGA
114	cgd5_1930	AACTAATGCTAGAAGGGCGATG	GTTATGAATGTCGCAAACAACC
114	cgd5_1940	GGCTTCAAACAAACTACCTCCA	GAACCAACCAGTAAACCAGGAA
114	cgd5_1950	GCTGCTTCCTTGCCTAAATG	GCATCGCTTAAATTGCTCGT
114	cgd5_1960	CTCGACTTGATTACAGAGGCAA	AAATGTTCTTCCACATCTCCGT
114	cgd5_1970	GAAGCCCACAGATTTCCATTAG	AATACGAACCATCTTCTACGCC
114	cgd5_1980	GGGTGAAGACCTAAGTACCAAAGA	ACGTTTGCGAGATGTGATAGTG
114	cgd5_1990	TTGGATGGAACACATTAAAGACA	TTGAATCCGACAACTGAAGAAA
114	cgd5_2010	CAAGAAGTAGCGAAGGAAATGG	CGCATCATAGCAGGATCAATAA
114	cgd5_2020	AAAGTTGGTGGGATGAAAGAGA	AAGGAATATAGCCGAGGTTTCC
114	cgd5_2040	ACTGCGACCTATCCCATCAC	AAATTCTATCCCACGTCCACC
114	cgd5_2050	GAGTCCATTTGCATTACCAACA	GTTCATCTGCTTTACCCTCGTC
114	cgd5_2060	GCCAATATAGAGAAGGAGGCAA	CATATAACCGTACTCGGCATCA
115	cgd5_2070	TCTGGAAGAGCAGGAAGAAGAG	CAAACAGCATAAACGTCCTCAA
115	cgd5_2090	GGTGGCGGATAAAGAAGTTACA	TTCTCGTGCAAATAAGAGTCCA
115	cgd5_2100	AGGTCGTGGTGGTCCTCTTT	GCAGTTTGGAATCTTTGATGTTCT
115	cgd5_2110	AGTAAACAGCAGCCAAAGTCATC	ATCGCTAAACAAGCCATCATCT
115	cgd5_2120	TGATGAAGTTGTTTATTCTGGCTT	AGTGAGTTGGCTGGAATCTTG
115	cgd5_2130	AGGAGGAGTACATTGGATTTGG	AACAGGAATCTGCTGAGGAAGT
115	cgd5_2140	CCTATATGCAGAAACCCAAACC	TTAGAGCCGCAGTTGAATACTTT
115	cgd5_2150	CTGGCACTTGTTCATCTCATTC	AAATGGCAAACACAATTAACCTC
115	cgd5_2190	AGATGTTCCGTTGATTTGGAGT	AGATTGGGCCGTTTCTGTATT
115	cgd5_2200	GCAATATGGAGAGCTTGAGGAG	TAAGGTGGTGTGGTTGTTGAAG
115	cgd5_2210	GAGAGCTGTTTCTATCCGCAA	TTATGCAACCAAAGTAGAGGCA
115	cgd5_2230	ACAATCTGCAACTGGATCAACA	AGGAACAGCAACAATTCCAAAG
116	cgd5_2240	TTCTTGGATTTGTGGATGATGT	CACCTGTCTGAAATAAAGGCAA
116	cgd5_2250	ATGGTTCTTTGGTAAGCACTCC	TCCTCCTTTCCTCTAATTCATCC
116	cgd5_2280	TAGAAGCAGGAAGGCAATCAA	AGATACTGGGCTCGATAAAGGAC
116	cgd5_2300	AGTCCAAAGGTTCCAGCATCA	AACACTCCGCCAATCAGAGAG

116	cgd5_2310	CTTCCATTCCAAAGATGGTCTC	ACCCTGCAACACCTTGTCTATT
116	cgd5_2320	CGAGTTGTTGTATCTCTAACGGG	TCGTGATCCAGCCCTATATTTC
116	cgd5_2330	GTAGGACGCCCAAAGTACAAGA	GGTCCAGAGAGCTTCCATATCA
116	cgd5_2340	GAAGAAATCAATCAGCCAGACC	TCCGAGGTGTTATCTCCACTTT
116	cgd5_2350	ATGGAGAGAGATGCCCGTACTT	TGCCATAGAACCCTTTCCTTTC
116	cgd5_2360	CAGAATCCGAGTTACATTCACAA	TCAGGTTGTTTGCTATTGCTTC
116	cgd5_2370	GCTCAGGCTTTGTCCACTACTAA	TCACCTTCTTCTTCCTCCTCTTC
116	cgd5_2380	AGATTGGCCTCCACCAGATAC	TCTTTGGGTCTTAAATCATGGG
117	cgd5_2390	GCAAGAATATCTCAAGCCAGGA	GGGTGAGTCCTTTGGTCAATAA
117	cgd5_2400	TTGGAACAATTAACATGGGCTT	TCCCTGAATCCTCAAACATCTC
117	cgd5_2410	AATTTGTCATTATTGGGATGGTTT	TTGATTTATTGGAACCTGTGGA
117	cgd5_2420	CTCCAGGTTCAAAGACAAATCC	TTTAGAATCCGTTGTTTCCGTT
117	cgd5_2430	TGATGTTGAGAATGTATTGGTTGA	GTTGTTGGGCCTTGATTTGT
117	cgd5_2440	CAACTGAAGATATGCTGATTGGA	AACGGTAGTAAGTGGAGCAAGG
117	cgd5_2460	TACTTCCCTGATGGTACTCCGT	AGGATGTTTGGTTGAAGCTGTT
117	cgd5_2470	CTCAATTTCCCTCAATCATCGT	AAGAGATCCACTTCCAGTTCCA
117	cgd5_2480	GTGTTTGGCTGCTTATACTCCC	GGTTTCCTTCCTGATGCTCTT
117	cgd5_2490	TTCTTCCACATCTCAAATCCAA	ACAGAGCAAGTAATGGCAAGG
117	cgd5_2500	GGAATATGGGTGGAAGTCAAAG	CACATCAAACGAAGAATGAGGA
117	cgd5_2510	TATTAAAGGAGCTTCACCACCC	AATCCTGTCTTGTTCTCATCCAG
118	cgd5_2530	ATTTGGTTACGCATGTCCTCTT	CCTGCCTTTGAGAAATAGTTGG
118	cgd5_2540	GGGATTAGCTCGATTTCTGATG	AATCTTGCCATCTTGACCTGTT
118	cgd5_2560	GAGCTGTATATTTCGGGATTGG	GCTGTCTCTATTGCCTGTTTGA
118	cgd5_2570	AGGAGAGCAACGATGACTTGA	TGATGATGATGCGGTAGAAGAC
118	cgd5_2580	CATTTACTGCCTGAATGTGCTT	CTTTCCCATGTGTTGGTCATAG
118	cgd5_2590	TGGGTTTAAGCTCTTCTCAAGG	TGGACTTCTTTCCAATCAACACT
118	cgd5_2600	AGCCAAAGAAACCAACAAAGAA	GAAACAGATTGAGCATCCAGAAC
118	cgd5_2610	ATTGCTTGAGAGTGAGAGAGGG	TTTCCAAGTGTTTGATGATAAGGA
118	cgd5_2640	CGTCCCAAATACTAAGCTCCAC	TCCAGTTTCCATTTCCAGAGTT
118	cgd5_2650	ATCTCTGGTATTGCGATCCTTT	AGCTTATGCCCATTATATCTGTGA
118	cgd5_2660	TTCTATTGCCAGACCCACCTAC	AAAGATGATGCGGAGAAATCAG
118	cgd5_2670	TGTCAAGTTTGGTGTCAGCTTC	TTTCAAGTGCTTCTGGATCGT
119	cgd5_2680	ATGGATCGCTGGATTTATTGTC	TGACGTGAATATCGCTGAATGT
119	cgd5_2690	AAGCAGAAGCAACTGAAGGAAA	CATCCAAAGGCTCATATCCACT
119	cgd5_2730	AGCAATGATGAATGGAAGAAGAA	CTAAGTCGTGATTCTGGAAGGG
119	cgd5_2740	GTAACAAGGCGAAATAAGGAGGT	CAACGTCTCCAAATCCAATACA
119	cgd5_2760	AGGCAATAAAGGAGGCAGAGTT	CATCAATTTCTTCTGGTTGGCT
119	cgd5_2770	CCTACTCGGGCTATGCTACTTG	ATTCTACCGCCATTCTCCATC
119	cgd5_2790	TACTTGCTTCTTTGGGAGGAAA	AGATCAGGTCGTTGTATTGGGT
119	cgd5_2810	ATAGATTCATTAGGCGGGCAA	TGAGCCATTATTCTGTTGTGGA
119	cgd5_2820	CGCCACAATTACTCCAACAAG	ATCCCAACCTGTACTGAAAGCA
119	cgd5_2840	AAGTATCACGGGCCAAACAC	TTCCATCTTCTCCTTCACTTCC
119	cgd5_2850	TTGGACTGGATATGGAAATGAA	TCCCTCAATAACTTGGACTCTTG
119	cgd5_2860	ATGTGATGAACCTTCGAGAATAGA	TATCTTCTGCTTCACCGCTTT
120	cgd5_2870	TGCCCTTTCTATCTCTCAAACC	TTGAAATTGTTCGCATATCTTTGT
120	cgd5_2880	TTGGACTAGGGTTAGGACAAGG	TCTCAATTCACTTCCTGCTTCA
120	cgd5_2890	TGGGAGAAATCCAACAAACC	TGTAACCATCAGGAACCTCAAA
120	cgd5_2920	GATGGTGTTAGATGGATGTGGA	TGTGATATTAAGCTCGTAGGGAGA
120	cgd5_2940	ATTGTAATTCTGCAAACGGGAC	TCGTTATCATCATCTTTCTCTCCA
120	cgd5_2950	TGATCCAAATTATGACCCATCAG	AATCTGTTCGCATTCTTCCAAA
120	cgd5_2960	ATACTTGGTCAGGCACCTTCA	CATAACACCCGTAGTCTCCTCA
120	cgd5_2970	GGGCTATTGCTGATAAATGGTT	CATCCTCCTCAAATACACCTCA
120	cgd5_2980	AATGAAGATGATAATGAAGGAGATGA	TGTAATTGAGGAACCATCGAAA
120	cgd5_3010	TTTCATTAACACCATCTGACCAA	AGGAGGAACACAAACAGGAGAG
120	cgd5_3020	AGACCCTGATGAGAAGAAGCC	TATTGTTTCCTGCCTCTGTGAA
120	cgd5_3080	GGAAGATATGATTTGGTTATTCCAG	CAAGAGATTCAGGGAAAGATGAC
121	cgd5_3090	GATTCAAATGACCCAAACAAGTC	TCTTTGCTAACCCAAATCCTCT
121	cgd5_3130	AAGGGAAGAAATGACCAACTCA	CCTCTCACCTCTGAATACCCAC
121	cgd5_3140	CCACGAGGTTCATCTATTCACA	TACCTTTGTTAGCCCATCTTCC

121	cgd5_3190	CCAACTGAATCTGAAATCACCA	TTGAAAGAACTTACCTCCCTTACTACA
121	cgd5_3200	CTTCTCGCTGCTTGTATGTCTG	GCCTTGGTTTCTTCTTCATTTCT
121	cgd5_3230	AGGTTGGATTTGGGTAGTTATTGA	AGCATGTTCCCATACATCACAG
121	cgd5_3300	GACAAAGATCCACTTCTTCGGT	GATGGGTTGAGGTCCAGTAGAG
121	cgd5_3310	GAGTTGGGCCTTCACAAATAAC	TCTGTTTCCAAGATGTGGATTCT
121	cgd5_3330	CCATCTGCTAGAATGTCGGTTT	AGTTATCCCAAGGTCTCCATCC
121	cgd5_3360	GAATCAGGGAACTTTGTTGGAG	GAATTGTTCTTGGAAATCCGTC
121	cgd5_3370	CAGAGATTCCCAACTTCACCTT	TATTGCCAGAAACGCTATTTGA
121	cgd5_3390	TAAATGAACAAGATGCGAGGAA	TGTAGCCATAAATCTCCGTCAA
122	cgd5_3410	TTGCAGGTAATGTGAAGCAACT	AGAGAATACCATAAGGCACCCA
122	cgd5_3470	AGAAGCTCAAGCCTATCCAGAA	TTCTCCAACCTTTCACAACTACC
122	cgd5_3500	TTGTGGTTCAATTCCTCTTGG	CAAATGGTGAGACAGTTGTGAAA
122	cgd5_3510	CTTGAGAAGGATGCAAGTGGTT	TCTACGGACAATAAGGCTCTGG
122	cgd5_3520	CCAATGAGTATTAGGGAGGCTTACA	TCCATTATCTGGGTGGTTCCTT
122	cgd5_3540	GAAATAGAGAAAGCAGGGAGCA	TTCTTTGAACTGCCATCAGAAA
122	cgd5_3550	ATAACCCACCAGTTCCGCTT	CATTGCTTGGGAAGAGTTGTTT
122	cgd5_3560	AAGTTCCATTCCAATCAAGGG	TTCTTGGTGAGGAGAAATACGG
122	cgd5_3570	CTCTAAATGCCGAATGTGCTAA	GCAACAGCTTCAACACCTCTAA
122	cgd5_3590	ATGAAATTGCTTTCTTCGTTGG	TGAATCCTCTCCATCTTTCCTG
122	cgd5_3620	CAGGAAAGTCAACATCAGCAGT	TTTGAAGGAACATAAGCAACCA
122	cgd5_3630	ATGGCACCTCAAGTTTCTCATC	GTATCTATCGCACACAACGTCAA
123	cgd5_3640	AGACTTAACAATAGGCGATACGAAGT	GCACCTCTAGCAATACTCCACC
123	cgd5_3650	TGCAACAATGCCAGATAAGAAC	TTTGCTCTAGCTTACCGACCA
123	cgd5_3700	GACGACCCAAGTAGTGGAAAGA	TGCTAAGTTTGTTGCCATTTGA
123	cgd5_3730	TTGTGTGATGAAGAATTTGGAGA	CAGCAAGATGAAGAGGAGTTTG
123	cgd5_3740	GCTGAGTCTTGTGAGGAGGATT	TCCAACAACCTTACGTGGATTAC
123	cgd5_3750	TTTCAATAGGAGATTGCGTTCA	ATGGCGGAATTAAAGTATTTGG
123	cgd5_3780	ATCGGAGAATATAAAGGGAGGG	ATGGGATTGGACTATGTCTTGG
123	cgd5_3790	TGGTCACTCAGGAAAGAGAGAA	AGCACTTGCAGATAATGCAGAC
123	cgd5_3810	AAATGGTATTGCTAATGAAGGTATTGT	TTTGGTGCTTTAGGAGGACTTT
123	cgd5_3850	TCAAGCATCATCTCCATCAGTT	GGGTTAAGAATTTGACTTCGGTT
123	cgd5_3870	GGGACGAGAAACTAACTACTGCC	TGGCTTTAATTGCATCACTCTG
123	cgd5_3920	GAATGGAGATGGAACATTGGA	AGCCTCTTCTGATGTAAGTGGG
124	cgd5_3940	TTCATCAGACTCAGCTTCTTGG	CTTATACCATCATAAACCCTTTCATTT
124	cgd5_3950	TAGGAACTCCAAATCCTGCAAC	CATCCTCTTCTCCTGTCATTACCT
124	cgd5_3960	GGACATGCTAACACTGAATGGA	TTATCACTATCAACCCACCCAAC
124	cgd5_3970	TCCTTGGGTTAATTCTTGCTTT	TGCAATGGATCTTCTCTTCATC
124	cgd5_3980	AACATCAGGTGGAAATCCTTTG	AATGCCCTGTAATATCATCCCTT
124	cgd5_3990	TCATCATCAGACTCGACAGTTTCT	CTAAACATACACCTCCATGCCC
124	cgd5_4010	TTTCAAGATTACTTTCTTCCTTCTCC	TGTATTCACCATTTCTGGAGTTTC
124	cgd5_4040	AATGGTTGGTGGTGATACTCCT	CCTGTAGTTCTTTGCTTTGCTTC
124	cgd5_4060	AACACACGAATAAACATGAAACAAA	TTAGCATTACCGCTTCCTCCT
124	cgd5_4070	ATTACCTACCTCTTGGCGCTTT	TAAATCACACATTGGACCCTCA
124	cgd5_4080	GGAAATACATCATTTGGAAGAACAA	CCTGGAGATGAAACCGAATCT
124	cgd5_4100	TTCCACAAGAGTGTCATCCGT	TTCAAATCCTCCTCCAATACCTAA
125	cgd5_4110	TTCACAAGGCAATATAAGTCTCCA	TCAAGCTCTTCACCAAGTTCATT
125	cgd5_4130	ATATAGCAAATGGTCAAACACCC	GCAGAGCCTGTTCTCTTTCTTT
125	cgd5_4150	CTGCAAGAACAAGGCTCAGTAA	CATCTGGACACTGGAAAGACAA
125	cgd5_4170	ATACAAATATGGAGGGAGGCAA	CCGCTTGGTGTCATAAACTCT
125	cgd5_4210	AACAGAGATGGTGGTTCAGGAG	TGTTGGATCAGTGCCTTGTTT
125	cgd5_4220	GGTTTAGTATCTGGATTAGGGCAA	CCTCCTCTTTGAGTTTGATTATTAGG
125	cgd5_4230	GATGATGTTGCTCAGAATCCAA	TGGTGCCCATAATAAATCCAA
125	cgd5_4240	TGATGTTGCATGGTTTATCATTGT	CCCAACTTTCTGCTCTTTCCA
125	cgd5_4250	AACCACAGCTTCTACTGCTCCT	TATTGCTACAAACACCAGCCAC
125	cgd5_4260	TGTTATCAGTTAGTGGCTGGGA	TCATTTCTTCAGTGGAACTTGTG
125	cgd5_4280	GCGAGCTACTTCCTGAAACAA	GTGTCTTTGCACAATTTCCAGT
125	cgd5_4290	TGAAGAAGAGGAAGGAGGATATGA	TCGCATCTACCAAAGAAACTCC
126	cgd5_4300	AAGTCCACCATCCACTAAGCAG	AGCATCATCATCCGAAGCTATT
126	cgd5_4320	AAGTTTGTTCCTCCATTAGCACA	GAAGGTCAATATCGGAAAGCAA

126	cgd5_4330	AGGATGTGAGGTACAATCTGGG	TTGTCGGAAAGTTCTTCTTCTTG
126	cgd5_4350	TCCTTCTTCAGAGCCTTCTCC	GCTGGTAATGTTTGGAGGTATCA
126	cgd5_4360	CTTGCCTAACTTCAGCCTTGTT	TCCTGGCTTGGGATAGTCTTAC
126	cgd5_4370	CGTGTATATGCTTGGAGCTTTG	GCCATTTCGTTTGGAAGTAGTC
126	cgd5_4400	CATTGGATTTGGAGTTATTCAAGG	GCTCTAGCAACACCTTCGTCA
126	cgd5_4420	CATTGTTTGCGATCCTCCTT	TTTCCTGTAGATGGATGAGTATTTGA
126	cgd5_4430	TTTGGAATACTCAATACCCTGATAAA	ACCATAAGCATCACCAGTAGCA
126	cgd5_4440	TCAACTACCAAGAAAGAGGAATGA	ATCAGGAGTGAATGTGTGTGCT
126	cgd5_4470	TTATGCCAAGTATGAATGGTGG	CTTGTTCTGGTTGTTGATCCTG
126	cgd5_4480	CGAGTATGAAGAGCGAGAAGGT	TGGGAGGAATTGTATTTGAAGG
127	cgd5_4490	AAACCAGACCCAATACCGAAC	AATAGCATCCTCAAAGGCATTATC
127	cgd6_10	GCCTCGAAGTTGTTATCGTTCT	TGTAGAGAGACATCCCACAGGTT
127	cgd6_20	TAATTGAGAATGGTGCAGATGG	AATTGCAGTGATTTGAGGAACA
127	cgd6_30	TGCGGTGATATTCTTTATTTAGTTGA	AGCTTTACCAGCTCTTCCCAC
127	cgd6_40	GGTGAAGAAGAAGAGCAACCAG	ACCAGAAGTACCGAGAGATGGA
127	cgd6_50	AGACATTCCTCCTCCACCTTCT	GCTTTGCTTAATTGTCGGATGT
127	cgd6_60	GTAATGGAAGGCTGGACAAGAG	TGATGAAGATGACCCTGAAGAA
127	cgd6_70	TATAGGGCATGAAATAGGCCAC	TGGATCTAAAGAGAAACCAAATGAC
127	cgd6_80	ACAAATCGCCACAGAGTGAGTT	ACAGGGCAATCAGTTGTCTCTC
127	cgd6_90	CATGGTTCTTTACGGGAATTGT	AGACCAACAGAAAGGGATGAAA
127	cgd6_100	AATTTGTGTAGGAGCCCAGGA	GGATTCTCAATTTCAATAGCCTCA
127	cgd6_110	GCAGTAAAGACGAAATCCCAAA	ATATCAGGCTTCTGACCCTCCT
128	cgd6_120	TCCAAAGTGTCAATGTTCCATC	CTTAATGCTGAGTTCGTGCTTG
128	cgd6_130	GCTTCCGATATGCCTTTATGAG	CAGTAGCCGAAAGAATTGAACTG
128	cgd6_140	GGGAGTACGAATTGTTGAGAGG	GCTAGAAGTAGAGCTGACGGGA
128	cgd6_150	TCAATGGATTTGGAAAGAAACA	AACCCGTTAATACTGATGAGCC
128	cgd6_160	CAATCCAGAACTCATCCAATTTC	GGCCTGAAAGGATATGTCAAAG
128	cgd6_180	TGACTTAGACGATGAAGAACCTGA	CGCCAATTAAAGTATCAAAGGG
128	cgd6_190	AATGCAAACTAATAAAGATTTGGCTAC	AAGAGCAAGATGGACATGGATT
128	cgd6_200	TCATCAACAACAAGCAGTTTCA	TTATCTCCTCTCTAGCAACGCA
128	cgd6_210	GCAACCTATTGTAGCAGACCAAC	CTCCTCCACCTCTATGTGAACC
128	cgd6_230	TTGAACCAACCAGCAAAGAA	ACTTGAAACCTGCATTTCCC
128	cgd6_240	GAGGAGATTATGAGCCAAAGGA	TTCCTGATGATGGTTGAGGTAA
128	cgd6_400	ACCAATTCCAGGTCATCCAA	ATATTGTTTGGCGGTAATCCAG
129	cgd6_410	TAACCAGATTGGATTGGATGAA	CTCCTGAATTTGTTGGTCCTG
129	cgd6_480	AAGACGGTAAGTGTAAACCAACAAA	CATCTTCAACAACCAAATAACCTG
129	cgd6_490	GTTATCTTTGTTGGTGGTATTGGAG	GCTTCTGGTATAAGGTGGCAAA
129	cgd6_500	ATGTTGGGAGAAATTGATGAGAA	GATGACCATTTAGCACATATTGGA
129	cgd6_520	CTCTTCAATGGCTTCATGCTAA	GGTGCAACATATCCTGGTGA
129	cgd6_530	CAATTTCAGGTCCAGGTTTAGG	AAATCCGCTTATTGGCTCTATTC
129	cgd6_540	TTCCTAAATCTCCATCAAGACCA	GCCTTTAATTGTTCCATTTCCA
129	cgd6_560	GAAATACCCTGAAGCTCCCATT	GATCTGTTCCTCCACATTTACCA
129	cgd6_570	TGTTAATCCAAAGGGAGAAATGA	AATAGAAGGAGCAGCAGCAGAG
129	cgd6_580	TTCAAGTTCGTCTTCAACATCATC	GCATCCATAATCGTCCTTTGTT
129	cgd6_610	TCTTGGTAGAGTCGTTGATGCT	CCAACAATAGCTTCTCTTTGTCC
129	cgd6_620	AAAGATCCAAGCAGTGGTACAATAAG	CGGTGCATAATACCTTGTGAATG
130	cgd6_640	CAAACTGGATCAAATATACTCACACC	TTGCTAGAATCAACATGAACACAA
130	cgd6_670	AAGGTTTGGATTCATTTCCCTC	ACATTCCATTTCCATCAGCATT
130	cgd6_680	ACGAACTTGACGCTCATTCTTT	AAATGTTGTGCCATTGATGATTT
130	cgd6_690	AGAAAGCAGAATGGGCAACTAA	GTGAGTCCAGGCATAAGTCCA
130	cgd6_710	GCAACTGCTGAACATCAAGATAA	GCTCAGATTTACCAAAGAATGGA
130	cgd6_720	AGTATCGGGATCAAGGCAAGT	TTCCATAGGATCTCCAACCAAG
130	cgd6_740	GAACCAAACACAAGAAATTACAACC	TTCTGCATTGAGTGAGGCATAG
130	cgd6_760	CGGCTCCAAATTCTATTCAAAC	CTAATCCAGAAATGGCTCCTTG
130	cgd6_770	TGGAAACGTAGTTGGTAATGGA	ATTTCAGGGTTGATTGAGGTGT
130	cgd6_780	TAATTCGGATGTGGAGTGTTTG	CTCTTGTCCTGATTGTAAGCCC
130	cgd6_800	ACTCGCTTATGCACCAATCC	ATACTACCACCGGCAGAGAAGA
130	cgd6_820	GGTGGTAAGAATTGGATAACTGCT	CTTGACCTCATTTGCTCTAAGTTTATT
131	cgd6_840	CCAAGCAATCTCTCTCATTTCC	AACGTCTGTTCCAAATACCCAA

131	cgd6_870	CTTGCCATGTATGTAGCAGTATGA	AGAAGAGTATCATTAAGCAATTTCCC
131	cgd6_890	ATGCTTTCAACTCCTCCACAG	TCCACTAAATCTGTCTGAGTCCC
131	cgd6_900	AAAGGGTAAATAATCCAACTCGTCT	TTGGGAAAGAAATATGGGAAACT
131	cgd6_910	AGTGAAGATCAAGAATCAACCGA	GCGAGTTTATCACCATACTGCTT
131	cgd6_920	TGCATTATTGAGCGATTCATTT	TCTGTTCATCAAGTCCACCAAC
131	cgd6_930	CCAGAGTTTGTAAAGTTGCTCCA	TCTCATAAGTCCATCCCATTCTCT
131	cgd6_940	TGGTGGTAAAGTTGGATGGG	GGCCATAAGGACGTGTAGGTAA
131	cgd6_950	CACCTGAACATCCTATTCCAGTC	ATTGCTGTTGGCTTAATGGTTT
131	cgd6_960	TCCTTCATTCTGGTCATCTTCA	AAGCATCCTAATGTGTCTGGGT
131	cgd6_970	AGAAGGCAGAGAGGGATGAGT	GTTTCAGATTCGATAATGATATTGCT
131	cgd6_980	TCGTTTCTATGAAGCTAACCCAG	AAAGTTCAGGCATGGTGCTAA
132	cgd6_990	ATGTCGGCAGTTGGTATGTCT	TCTGGTGGCGTATTAACCTTATC
132	cgd6_1010	AAAGCGGAGTAGGGAAAGTTGT	AAATTCGGTAATCTGGTGTTGG
132	cgd6_1020	ATGCAACTGGTGGTCATATTCA	CAAACTTCCTCCTATCACAGCC
132	cgd6_1030	AGTTAGATCACGAGGAAAGCGA	TTGAGGCTAAGTTTGATGCTGA
132	cgd6_1060	GGCAAGTCAACTGGAAGATGTAA	CTAAAGCCCTTCTTTCCCAATC
132	cgd6_1070	CGCATGGGTAATAAATCCAAA	TGCTACTACTTGAGTTGCGGTC
132	cgd6_1080	AGAACTGGAGAAGACGCAGAAG	CTTTGAGCTGGAGTAGTGGGTT
132	cgd6_1090	AATTCGATGGGTCATTATTTGG	CCTTGGGAACTTTGTTTCTCTTT
132	cgd6_1100	TGAAAGCACTCCCTCATCTAACT	CATCTTCTTCCTCAAACTCGAAA
132	cgd6_1110	TATTGCCACAAGAGAACCTGAA	CCATCTAATCCAATTCCTCCAA
132	cgd6_1120	GTTTACTAGCCAGACATTTGGAAGA	TGAGACGTGTTCAATTAGCCTTT
132	cgd6_1130	TCTCACTAACAGAACGTGTGTCC	CCCAATAAATCACCTACCAATTCT
133	cgd6_1140	ATGACTGACGCATTCCCTACTT	TTTGGTTTGGAGAGGTTTCTGT
133	cgd6_1160	AATTGCCATGTAGGTGATTGG	GACCTTGTTCCCGTCATAGTTT
133	cgd6_1170	TTAATGGAAATAGCGAAGGGAG	CTCTGGGCTTATTATCTTTGCC
133	cgd6_1180	AAGCAAGTTTCAATGGGAGAAG	TCAACGGCTAGATAACCACCTAA
133	cgd6_1200	TGAAGAGGCTAAAGACAAATCCA	GTAATCCAGTACACCCGATAGCA
133	cgd6_1230	ACCGGCTAGATTAACCAATTCA	ACTATTCCAAACCCAATGCAAG
133	cgd6_1270	TGCGACATATACAGATGGCTTT	GCCTGGTTTAAGTGGAAGAAGA
133	cgd6_1280	GTGATTTCTGGAGCTTTGCATT	CACAAACAGGTTCTTTCATCCC
133	cgd6_1300	CTAAGTCATCTCGCTGTTCGG	ATCTTGACCATCCTGACCATTC
133	cgd6_1310	TACAAATGCCGGACTGAGAAC	ACTGAAAGGAGATGGAGGAGG
133	cgd6_1320	AATACCGTCCAAAGTCACCAA	TGCAGGAACAATATCGACTCTACT
133	cgd6_1330	AAGCGTGGATTTAACACGAACT	CATATCTGCACGAATCACCAAC
134	cgd6_1340	AAAGTTCCAAGGTTATTGGGTTC	GGATTGAGTTTATCGACTGCTCTT
134	cgd6_1350	TGAATATAATGGGATTGGAGGAA	ATCGTTCTCTAAAGGATCGTAATCTC
134	cgd6_1370	TTGCGGATGTGTCTTGTAGATT	TTGCTCAGTCTCAATGTGGTTT
134	cgd6_1380	AATTATCAATGGGAGCCAAGAA	GGGTCATTCTAACTCTATGCGG
134	cgd6_1390	TGGTATAGAAGCAAAGCAACCA	CGACAAGCACTTCATCATTCTC
134	cgd6_1400	CAGGATGCTTTGTATTTACTGCC	ACCCGTTGAGATGTAGTTTGCT
134	cgd6_1410	TGAGAAAGCACCATTAAGCAAA	TGGTATCCTTCATTCCAAATCC
134	cgd6_1420	AGTCTCTCGCAGCACCTATCTC	ATTCATTCTTGCTTCTGCCTTC
134	cgd6_1430	TGCTCCTCAGTAAATCCTCCTC	GATCATGTGAGTTTGACGCATT
134	cgd6_1440	TCAAATTCCCACGATACAAAGA	TCATTTCTCCGCTATCAGAATG
134	cgd6_1450	GGTTCCACTTTCATAACCCTGA	TGAGCCATTCAACAAATAATCAAA
134	cgd6_1460	GAGAAATCAACCGAAATGCTAAA	ATTGGTCTTCCCAACCTATCAA
135	cgd6_1470	AAATGAAAGCATCCAATCAAATC	GCCTGATATGCCCTACTAAACC
135	cgd6_1480	TTACTTGGGACAACGAAGACATT	TTTCTACATACAACCCATCCTTCA
135	cgd6_1490	ACTCAAGCTGGGCTAAAGAATG	CGAATGACGATGAGGTATCAAA
135	cgd6_1500	GCATGTTTAATACTCTGCTCCGT	GAAGAGAACCCATAATTCCCAA
135	cgd6_1510	TGACATTTATCTTTGCGTTCGT	GCATTTATCCCGACTTCTGTCT
135	cgd6_1520	TTTGCCTCCATTATCACTACCA	AAATTGCTTTACACCCTCGTTT
135	cgd6_1530	TTAATGAGAGCATTGGATGGTG	TGTTGAGCTTGAAACACTTGAGA
135	cgd6_1550	TCAATTCCCACTAATTTCTGCC	ATTATACGTTGCATAGCCGTCC
135	cgd6_1560	AACAGTAGAGGTGGAGGGTACAGA	CGTCCCGACATAGTGATAAACC
135	cgd6_1570	CTGGGTCGTTTGACCAGATAA	GTCTCCAGGCACTTCTAAATCTTC
135	cgd6_1580	GACTTATTCCGGTTTGTGTGTTC	AATTTCTCTCTTTGCATCGCTT
135	cgd6_1590	ATTGGACAAGGGATGAAAGAAA	TGCCCTATACTGACGAAGTTGA

136	cgd6_1600	GAACTTTCGGATGTGAGCTGT	CCTCTTATATTCTCTCTCTTTCCACC
136	cgd6_1610	AAACCACTCTTCGTCTTGCC	AAGTCCATGTTTACACCCTTCC
136	cgd6_1620	GATCTTCATGCGATTCCTCTTC	CCTTAATGCCGTTCTTTGTTTC
136	cgd6_1630	AGAACTGGCTTAAATGTGACTGG	CATCAGGAACGTCATCTTCATC
136	cgd6_1640	GGACATTCCAGCACATTAGACA	TTACCAATCTTACGGATGCAGA
136	cgd6_1670	TGGTTGTGTTGGTAAACCTTCTT	TTTGGCTCAATGGTAGTGAATG
136	cgd6_1680	GAGATCATGCCACAAATTACCA	ATTCCCAACGTAGACTTCCCTT
136	cgd6_1700	CGCTTTAACTGCCGTAACATTC	TTTCTTAGCTCATCACGCCATT
136	cgd6_1710	CTTAAATCCATATTGCCCGAGA	TCATCCATTCTCTCTCCATTCC
136	cgd6_1720	TCGAAGCTGAAGGGTGAACT	GACCTCGTATCCTGTAGCCATC
136	cgd6_1730	GCCGACATTCTGTTGTTTGTAG	GTGTGTCATCTGCCTTGTGTTT
136	cgd6_1740	AAATGCTATGGTAACAGGGTGG	CGATGAGGTATTTGAAGGAAGG
137	cgd6_1750	ATAAGGATGGGTCATTTCGTTG	GCGCCTATTATGTCAATTTCGT
137	cgd6_1760	TTATACTTGGTGTTGGAGATCGG	GTTTAATCCGCCCATAGCTTC
137	cgd6_1770	TATACCCAATGAATGCTGACGA	GGTTGGAACGTAAGTGCTTGTT
137	cgd6_1780	GCAGAAGGGCATTGTAATTTGT	CTTTGATTGAGTGGGAACGG
137	cgd6_1790	ACAATGGAACAAATAGCGCAG	TGAATCGTTTACAGAGAGCCAA
137	cgd6_1810	GCTGAACTCGAATCAACACAAA	GCTCTTTCAACTGCAATACGG
137	cgd6_1820	TGTTTATCCCGAGCATCAAATAC	ACGCCTCTCTTCTTTCCGTT
137	cgd6_1830	GTCAGCTCCTACAGACGCAAC	TTTCGCTCTATCAATGTCCTCA
137	cgd6_1840	AGAGGCCAACTCAAAGCACTAA	TTTCAACTCGTCCGTCCATAA
137	cgd6_1860	CTTCCAGCAAAGCAATAACCAG	TCCCATATTCATAAGCATCCCA
137	cgd6_1870	ACGAACACAAGCGGATCAAG	TTTCTGCAATAGTTGGATCTTCTACAC
137	cgd6_1900	GCGGATTCAAATACACCAGAAC	TGATCCCTTATCCTTTGCACTC
138	cgd6_1910	TGAATATAAAGGGCAATCCCAC	TGTGGATCTGGAACGTAACTTG
138	cgd6_1930	GAAATGAGCGAGCTATACATTGATT	AAGATTATTCCTTCCCTCGTCTG
138	cgd6_1940	ATGAGCGATGGGATGGTAAA	GCTGGCCTGAAGAATTGAGTAG
138	cgd6_1950	CGAGTCTGAGGGAGCTAAGAAG	AAGCAAATCCCAATCACAAAGT
138	cgd6_1960	GTGCGACTGGAATCTTGTAATG	ATTTGATCTATGCTCATCGCC
138	cgd6_1970	ACATTTCTGAGTCTGGGCACTT	CTTGAGAATAGTTTGCCATCCAC
138	cgd6_1990	TCCAAATCCAATGAAAGTGAAA	TCAAATTACCGCAAGTACCAAA
138	cgd6_2000	TTCTCCAGTTGCTCTCCAAAC	GATCCAAAGGACCATCAGAAAC
138	cgd6_2020	TTCGTCGCTACTTGGGTCTATT	ACCGATACTTCAGGAGGAGAGAG
138	cgd6_2030	GTTTGCATTGTCTGGTGGATTT	TCGTATTGACATCATAACCGCA
138	cgd6_2040	GCCGTAGTAAGATATGAGGCGA	CAAAGCAGCAGTAAGCATGAGA
138	cgd6_2060	CGACTTTCCTGAGTTCCTTTGT	CTGGGTATTAGCTTCATTTCGG
139	cgd6_2070	CGACATAAAGAAACTCGCAGAA	GATCAGGGCAACATTGAAATAAC
139	cgd6_2080	AATCGAGCTTAATGGGTTAGCA	CTTGACGCCTTCCATATCAACT
139	cgd6_2090	TATTTCTGCTCATCCACACCAC	TGCATTTCATTTGCTGAATAGG
139	cgd6_2100	TCCAAACTTGCCACTACCAAA	CCGCGAATAACCCATCTAACT
139	cgd6_2110	GACGTGAAATACGAGCAAACAG	CAATGGTACGGCTTCATTCA
139	cgd6_2120	AGCACTTTATTTCTTCCTGAAACAC	CCAAATGTTCTTCATAGTTCTCACTT
139	cgd6_2130	GTACAGCTTGGGCTTGAACAG	CCTAAGACATCAGCAACAATTCC
139	cgd6_2140	TATTTGGGCAACAACAAAGATG	AATCATAGGAAAGCATCCACAAA
139	cgd6_2150	TGCTGTGAATAATGAAATTGGG	TTTAACCATTGAGCCAGGTATTG
139	cgd6_2170	AGCAGAGGTGAACCCTATGAAA	CCTACGGATAGAGAATGAACGG
139	cgd6_2180	CAAAGTCACATGCTCTAGCCAC	TGCTTCTTCTTCTTCCAACCTC
139	cgd6_2190	GGAGAAAGGATGGATGGAGTAA	AGGAACCACTCTAATGCAAACC
140	cgd6_2200	TGAAGGTGAAAGGCAGAATACC	AACCGGAGTAACAAGCATTGAG
140	cgd6_2210	TAAGCCCATTTAACGAAGCAAC	CTAATACTGCCATCATCGTCCA
140	cgd6_2220	AGCTCCTTTGGGATTTAGAGCA	GTACAAACACCAGTCGCCCTT
140	cgd6_2240	GGATTTCTGACCATTCTCATCC	TTTCCTCATCTCCGACGTATTT
140	cgd6_2250	AAGAGCCTTGGAATGCAGAA	TCTGTCCTCAGAAAGTGACCAA
140	cgd6_2260	TCAGCAGGATGTATTGTTGGAG	AAATTGTTTGCGTCCCAGAG
140	cgd6_2270	GACCAGGAGATCGAAGGTATGT	GCTGAAGACATTAGTGGAACGA
140	cgd6_2280	TTCACTGGAATCTTGAGACGG	TGACTCTTCACTAGGTCTTTCAATGT
140	cgd6_2290	TGGATGACCTTTACGACGATTT	TCGTTACTAGAGGGAGTTGCGT
140	cgd6_2300	CTCAACTTTGCTTGCTATCCCT	TTTGGAATGCTACTTTCTTCCC
140	cgd6_2310	AAATTGCTCTCCCGAAGTTACA	GGAATGGTCACATACTCCATCA

140	cgd6_2320	TTCTCAAGGAACTCCACCTCTC	AGAATAGATGCGCCATAATCGT
141	cgd6_2340	GCAGATGTGACCCAAGTACAAA	ATTAGCGGAGCACTTAAACCAA
141	cgd6_2350	ATCTCTGGAGGTATAAGCGGTG	GGTAAGCCATAAAGCACGTAGAA
141	cgd6_2360	CGGAAGATAAATTGCTCGCTAC	ACATAACCCAATATGGTCGCTC
141	cgd6_2370	AATTGACAGAAGATGCGATTTG	GTGGGTCTCCAAAGAGAGGTAG
141	cgd6_2390	CTTATTTGGAGTCGTTCAAGGG	TCTTCTGGAGTTCTTGCTTGTG
141	cgd6_2410	ATTTATATTTCGGCACCAAACC	TGCTTCTCACACTTGGAATAATG
141	cgd6_2430	TGCAAGAAGAAAGAAGAGAGCA	ACTTCACCAATAGCAGGGAAAG
141	cgd6_2440	TCATGGATAGTCGTAACTCTTGCT	CCCATCTTTATGCCTCAATTTC
141	cgd6_2450	AGTTATTGGATGCAGGAAAGTCA	GCAGTTTACCCATCTTCTTGGA
141	cgd6_2460	CCCATTAAGGTTGGAGATTATGTT	TGAGTCTTACGTGCTCGATTCT
141	cgd6_2470	ACTTTATGAGGCTGAGGGTCTG	CAACGTAAGAAATGGATGACGA
141	cgd6_2480	TGCGATCAACAAACTTCAACTC	AGCCCTAGCGTAGTCAAATCAG
142	cgd6_2490	AGGAATTGTCTTGGCATTAGCA	GTAGCGAGTTAGCGACTTCAGG
142	cgd6_2500	GGAATAAGCTGGTGCATGAAGT	ACGATTGCTGGCTAAAGAAAGA
142	cgd6_2510	AGTCATAGATTTGAACCCACAACA	GCCACCATGATTGATAACACTTT
142	cgd6_2520	TGTTTAGGGCTCAATCAGGAA	CAGGTCAATGCAACAACAAAGT
142	cgd6_2540	AAAGCGTTGATTTGGAAGTCTATT	ACTCCTCGTTTGTGAAGTTTGAG
142	cgd6_2550	TTGATGATTTAGGTGCAAATGG	AACCTTTGGTCATTGTCAGTTTCT
142	cgd6_2560	ATGCCTGATGAAGATGATGAAA	ATTTGGAATAATGAAGACCCGA
142	cgd6_2570	CAGGTACGAAGGATATTGGGAA	CTGTAGGCTGTGGAATTGTGAA
142	cgd6_2580	CAGCACCTACATCTTTCCCTTC	ACGTACAACTGATGGTGTTTGG
142	cgd6_2590	ATGGTTAATATGGCTTTGCGTT	TTCGCTTGAGACTGCTTCATT
142	cgd6_2610	TGACTCTGTTTCTTGCTTGGAG	GTCCGTCTTGTTAATCCTTGGT
142	cgd6_2620	ATTTAGTACGTCCGAGCAGTGG	GGATCAAGAACCTGTGAGAAGG
143	cgd6_2640	TCATTTATGGTTTGTGGCTTGA	CTTGGCTGTTTCAGGGCTATT
143	cgd6_2660	ACTCTTTGTTTGCTTCCTCTGG	GCGCCTATTCTTGCATTTATCT
143	cgd6_2670	GGGTTGCTTTGAGTAGGCATT	TCATCGTACACAGTAAATCTGGCT
143	cgd6_2680	TCCAGAGGTTTCACTGCTTACA	GGAGCTTGAACTACGAACACAA
143	cgd6_2690	CCTAAGCCAGAAGATGAGGTTG	TAACTGCGAGATTTGGAGCTTT
143	cgd6_2700	GGCTCTTGTTTCATTCTTCACC	TTTCATCCCATATTCCACCATT
143	cgd6_2710	AGCTGCATATCTACTGGCTTGAC	GCCCAAATGTGATAGAGTGGA
143	cgd6_2720	AGAAATGCTAGGAGAAGCGAGA	GCAATACTTGGCTGTGCAATAA
143	cgd6_2740	TTGATACGCCTACCTCTCTGGT	TACTCGACAAACCCAAATCCTC
143	cgd6_2750	AGTTCACCCGCCTTATTTCTTT	AAGCAAGCATTCCTCTCTCATC
143	cgd6_2760	GTTGACGGTGTATCACATTACGA	TATCACGAGCCGAAGATGAA
143	cgd6_2770	CAGCAGCAATCTTGAGTAATGG	GCTTTAACTTTCATTGGTTGGG
144	cgd6_2780	CAGGGAAGGAAATGTATGGAAA	CAAGAATGGAAACAAAGGGAAA
144	cgd6_2790	CATAGAGCTGTGGCATCTACAAA	CCAAAGTTAATCCTTCTGCTCC
144	cgd6_2800	TTAGCGGAATCTTGTAATGCTGT	AAACGTGATCTGTGATGCTTTG
144	cgd6_2810	CCCTTTGGGAGTATGTATGGG	TTTGGGTTCTTTGTCCTCATCT
144	cgd6_2820	GGTATGGAGACTTTGAGGTTCTG	ACAATTTAGCTGGAAGAGTGGAA
144	cgd6_2850	TCCGCCAATACAAACTAGAACC	CATAATAGGTCCAAGACGCACC
144	cgd6_2860	TGCTGGTTGCCACATAAATAAA	CCTCAATATCCATCGAAAGCA
144	cgd6_2870	CCAGGAGCATCATCTACCATCT	TATAACTGGGCTGAAACTGGCT
144	cgd6_2880	AGACTCGTTCTCTGGTATTGGG	TGTTGAAAGCTCCTTATCTTGTTG
144	cgd6_2890	GAAGCGAGCAAAGCGATAAG	CTGTGAGGTTCGGGTAATGTTT
144	cgd6_2930	TATTCAAAGCCATAGCGACTCA	GACGCAAAGAAAGTGTCCAAG
144	cgd6_2940	TGAATTACTTTCCAAGCAGCAA	TATCCATCCTGTCATTTGGGA
145	cgd6_2950	GCGCTAACAACCCTCTAACATC	CATTTGCTGTTGCTGTTCACTT
145	cgd6_2960	GCAACTAATTCAAGAAAGTCCCA	AAATGAACCAGCAATAATAGAGGAG
145	cgd6_2970	AATTCGGTCATTTCCATGTTTC	TAGCATAAGGTTCAAAGCGTCA
145	cgd6_2980	AAAGAGCATGAAAGACGAACTTG	TTTGAACATCTGGTAGAAAGGGA
145	cgd6_2990	CTCCAATTCAGCCATTATTTCC	CTGCAAGCTCATTCAACAAATC
145	cgd6_3000	CTTTGCTATAACTCAGGCTCCAA	GACCGATAGTTTGTTCCGTTTC
145	cgd6_3010	GAGAGCCACCAAAGATACCAAG	AAACCCAAATCCTCTTGAGACA
145	cgd6_3020	ATGATTTATGAGTGGGTGGCTC	AGATTGAAAGACCGACAACCTG
145	cgd6_3030	AATGGGTGAAATGAAACAGGTC	TTCTTACAAGCCACAAACGATG
145	cgd6_3040	AGATGAGTTGGAACGGATAGTGA	TCTCCTTCATTCGATTTGCC

145	cgd6_3050	CTCCAGGTTCACCATATTCTCC	TATTCAGACCCATCAACTCCAA
145	cgd6_3060	TGATGGAAAGCGAAGATGATG	CCATTCTCTAATTCATTCCCAAGA
146	cgd6_3070	CCAGCTTCATTCCATAACTCTTG	AGGCATTTGTATTGGCTCTTTG
146	cgd6_3080	ACCCACGATACAATTTCTGGAC	TTGACCAGCTAAATCAACAGGA
146	cgd6_3090	AAAGATTTCCACAATAGCCCAA	CAAGGAACTTTGAATAAACAACCA
146	cgd6_3100	TGATTGTTGGGACAAGAGTTACA	TTATCAAGCCCTATTTGGTTAAGAG
146	cgd6_3120	TGGAATGAACAAGAATAATGATGAA	TCACAAGCAATTACACTAGACCAAA
146	cgd6_3130	TTCAAATTCTAACCAACCTCCTTT	GGCGCTTTACCTTGAGTTCTT
146	cgd6_3140	CTCACAAGATTGGTCTGGTTCA	GTAACTCCGCCAGTTTCAGATT
146	cgd6_3150	TCCTATCCACTTCTATTGACCCA	TGGTAGTCTTGATCCCTGTCCT
146	cgd6_3160	CTTCAGTAGTTATCGGTTCGGG	CCTGCATCTTCTCACTTGTTTG
146	cgd6_3170	GGCGAAGCTATTGGGTTACTT	TCTCTCAAATCCTTCACATCCA
146	cgd6_3180	AAGCTCCTTACCATGAAATTGG	TGGACATTCAGCCTTAGATTTG
146	cgd6_3190	CAGGAGGTACATTTGGTCGTCT	TATCAGCGTTAGTCATTGCTGG
147	cgd6_3210	GCAATCCAGTCTCAGGTAATCC	TTGCTGTGCTAACTCTCTTGTTG
147	cgd6_3220	ACATCAGACGAGGGAAGAGAAC	CTCCTGCTAACTTGGGACCTT
147	cgd6_3230	GGCCCATTTGGATTAAGAGTT	CATCAATTCACTTCTGGATGCT
147	cgd6_3240	TTCAGTTGTTGCAGAGTCCAAG	CCTCATGTATTGGGTGTTTATATGG
147	cgd6_3250	GCAAATAGGTCTCTTCAGCTTCATC	CCTCGTCATGTTGCTTCTTTGT
147	cgd6_3260	AAAGAATGTCAAATACCACACGAA	TAGGCTCACCAATATCTCCCA
147	cgd6_3270	GGATTTGTGAAACTAGCAAGCA	TCAACATCAGCATTCAACCTTT
147	cgd6_3280	TTAGCTCAAGGCGATACAATCA	GTGAGAAGCATGAACAGTGGAG
147	cgd6_3290	CAAATGTTTCCAGAATTGCATC	TCCGAGACTTTCTTCTTCCTGT
147	cgd6_3300	TGACCCAAATCCTCCTAAGAAA	CATGCTGGTAATCAATCATCAAA
147	cgd6_3310	GCAATCAAAGACCCTTCTAAACA	TCCTTTCTTCCTGATCCATTTC
147	cgd6_3330	TTAAACGCGAACTCACACAATC	TGATCCTTCTCTACACAGGCAA
148	cgd6_3350	GGAGTAGATCGGGTTTGAATGA	CAAATATAAACTGGGAACAACCACTAC
148	cgd6_3370	TGAGCGGTTCCATTATAGGTTT	ATTACCATTTGCATCTCCTGCT
148	cgd6_3380	TTCTTTGATGAAGTTGCACAGG	CTTCGCCAGTAGATGGGTTCT
148	cgd6_3390	ATTGGTCATTCACTTCCATTCC	CGACCTAACACAACTCCAAACA
148	cgd6_3400	AAGGCAAGGATATACACAAGCAA	CCAAAGACCATAAATCAGCCTC
148	cgd6_3410	CTTCCTCCTCCTCTTCCTCTTC	GCAGATTGTTTAGGATGGTATGATT
148	cgd6_3420	ATAGCGTGATTGGAGGATCAG	CTTCGTCATCTTCTTCATCTTCTTT
148	cgd6_3510	CCTCTTGTTAGTCCAGCAGTCTC	CTCTTAATTGCCACCTTTGGTT
148	cgd6_3520	AGAGGCCAATGCTTATTCAGTC	ATTCTCATTTCCTGTTCCTCCA
148	cgd6_3550	TTCATTTACACAAGGACCAGGA	TTCCCATTTCTATGTGAGCAAC
148	cgd6_3560	CTTATTGGGCCGTTACATCAA	TTCCAAGCATTCTCACATCAAC
148	cgd6_3570	AAATTCGACGGTTTCTTTATGG	ATGCGTCCTGACTTGTGTATGT
149	cgd6_3590	TCAAAGATCATGTCCCAACAAA	GACCATATCACTGCTTAACTCGG
149	cgd6_3610	AATGCCACTCTTTGATGTTCCT	AGTCTCTCAGTTCCCTGCTCAC
149	cgd6_3620	AGGATTTAACAGCCAAACAGGA	TGAGAGACCCATTCTTTCTACCA
149	cgd6_3640	TCAGAGAGCTAATCCAAGACCC	ACTCTACATCGAAAGCGGAGAC
149	cgd6_3650	TTCTTCACCAAATACTGCAAGGT	AAATTCTCATCATCCGCAAAGT
149	cgd6_3660	TGAAGTCTCATGTTGTGGTGACT	CTAATCTTGTATCGCATGTGTGTG
149	cgd6_3680	GCAAGTCAAAGAACTCACCAAG	TCCCACAGAACTGGATGATAAA
149	cgd6_3690	CAATTTAGACCCAACCACACAA	TTGAGCATTAGGAACAAATCCA
149	cgd6_3710	AATATGGCTAAAGCACACGGTT	TCTCTTCTTGGCTCTTCCAGTC
149	cgd6_3720	TCATAGAAACGATGTATGGCACTT	TTCCTGCTTGAGTAGAACCTCC
149	cgd6_3730	CGATTACATTCAAGCCTCAACA	ATTGTTTCCAGATCCCATACCA
149	cgd6_3750	TTGGGTGAGTATGGAGTTGATG	CTGCAATTAGAAACCTACCAGGA
150	cgd6_3770	CTTTGAATGAAGCAACCACAAC	GATAGTCCAGAAACCATGAGTGAA
150	cgd6_3780	TTGGAAGATCAAATCAAAGGAAA	AATGAATGAAACAAACACCAACC
150	cgd6_3790	AATGGAAAGCTCACAGGTATGG	GCTTCCTTAACGGCTTGATAGA
150	cgd6_3800	TCCCATTAGAAGTTGGCTTCAC	AACGCATTTACATTGAGCTGG
150	cgd6_3810	ATACTTGGGAACTTGGTGGAGA	TCTTTAACACATGGTGCTTTGG
150	cgd6_3820	CCAATATGGTACGGGTTCTTGT	CATCGAATGGTAATTCAGCAAA
150	cgd6_3830	CACAGAAGATGGTTGACTTTGCT	TTGGATGAAGTTTCGCCATT
150	cgd6_3840	TGACAGCGACTCCATTCTACTT	ATTCGACTCCCAAAGTCATTTC
150	cgd6_3860	TGAATGACCCTTGGAGAGACTT	TCTGATCCCAACTTCCATAACC

150	cgd6_3870	AGCATTCCATATTACACACCACC	AAGAAAGAAACCGATGATTCCA
150	cgd6_3890	TCTTGTGGTATGGCTTAGGAAGA	TGCCAATCTTGTTGTGTAGTTTG
150	cgd6_3900	GTTACAGCCAATCAAGAAGAAGG	AGATCCGTAGCAGAAGCTGAGT
151	cgd6_3920	AAGGAGTTCCCAAGTAAGAATCAC	AGCAAGTTTATCGCTCTCATTTC
151	cgd6_3950	AATTGTGGCTTTCCTTGGTG	AGAATTGTAGGTTTGATTGAACAGG
151	cgd6_3960	CTGAAGGAATCGGAGATCAAAC	CTCCAGAATGAGCCTATCCATC
151	cgd6_3990	AAGAGACCAACTGACAAGCCAC	GCTCATGGTGCATTTCTACTGA
151	cgd6_4000	AGCATGGAATGGAGTTTCTTCT	TGGTCGTAATAAGTTTCCCACA
151	cgd6_4020	TTATGCCTCCAACTACTTCCAAA	GCTTCAACTTCGATACTCCTTGA
151	cgd6_4030	GGGATGTAATGAAGGAAGATGG	TCTTAGCTGTTGGGTCCTGTTT
151	cgd6_4040	CAAGATCAGAAGATCCCATTGA	GTTCAGCTCCACGTTAAAGACA
151	cgd6_4050	GAGGCCCATATTCTCCCTATTC	ACAGCTCGCACCAAACTCTACT
151	cgd6_4060	GAAGCTGCCAAATACACATCAA	AACTCCAACCTCTAAACGGGA
151	cgd6_4070	TTTCATCTCCTCAGTCATCAACA	TTCATCAAATCTGCTTCCCTCT
151	cgd6_4080	GGATGGGTTTCAAGATAAATCAA	ATGCGATTGGTAGTAAGGGAAG
152	cgd6_4090	AAACCCAAAGTTATGAGGAAAGG	AACTCTTCCAATATCCCAAGTTCA
152	cgd6_4100	TTTACATGGCGTTTCTCATTCA	CCATTCTAGGGAAGGGATGG
152	cgd6_4110	GGTGGAGGATGTTTATTGGAAA	TATCCCATTCAAGGCGTAATCT
152	cgd6_4120	GATCAATGCACGATGGAGTAAG	TGCAAGATGCTGAGAAATAGGA
152	cgd6_4140	ATCACACGCTTTCTCAGGATTT	TCCCTTTGTATTCCTTGATTGG
152	cgd6_4150	AGGAGCATCTTCTACTGCAACC	GACGTTTAATAGGGCCAACATC
152	cgd6_4160	GAAGCCACAGATTGTTGTGAAA	ATCCAAGCCTCAGCACTAACC
152	cgd6_4170	TTCATTCTCAAACCCAAATCCT	AGGAGGCTGCATGTTGTTATTT
152	cgd6_4180	CCAGACGGGAGGATATGTTTAG	GTGTTTGCAGGAGAATTAGGATTT
152	cgd6_4200	ACTGCAAACGAATCAAACCTCT	GGATCACCACCAACGTCTAGTAA
152	cgd6_4220	ACCCTTGAGTCCTCTTTGATTG	GGAAATATGGATGTTCTTTGCC
152	cgd6_4230	ATAAGAAGGTGGGTCCAAAGG	ATTCCCAGGCGATAATGAACT
153	cgd6_4240	CACAAAGGCATGGTTTAAGATTG	CCTGGTGATTTATTGGGATTTG
153	cgd6_4270	AATGTTGCTCTAGCCAATCTTCTT	AAGTTCATGGTTGGATCTTGCT
153	cgd6_4280	AGTTTGTTCTTTGGGAAATCTGAC	CGGTCGCCATGTAGTGTATAAG
153	cgd6_4290	TTCATGCAGGGAATAATGACAG	AAATTGGTGATGGTGAAACTCC
153	cgd6_4310	TCTATGGCCTCCAAATGAAACT	TTAAAGACAAGAAGGCTGGACAA
153	cgd6_4320	AAACATCAAGACCATTGCTGAA	GCAACACGTTCTACCTCATCC
153	cgd6_4330	AGCAAACAATGCAACCAATAAC	TGACTTCTGGTCTTCCCTTTCT
153	cgd6_4340	TGAGATCAGAAGTGAATGGGAA	CGGTGATGCAAGAAGTTAGAGA
153	cgd6_4350	TACAATTAGGTATTGACCCGCC	TAACCATTCTTGCTCCTTCTCC
153	cgd6_4360	CTTGCACTTGGTTTGACTTGCT	CGAAACTGAAGGCTGTGGATAA
153	cgd6_4370	AAACTCGTTATCATGGATTGGG	TATACCGTTGTCATTCCTGGTG
153	cgd6_4380	TGTAGAGATCATTGCCCTGAAA	AACTTTGTTGGAGAAATAAACCCA
154	cgd6_4390	ATGACAGGAGAAGCAAACATACC	CATGCAAATTCTGATATGAAACAA
154	cgd6_4400	TATTTAAGGCCAAACAACGACA	TCACACCAAGGAGCTATAACCA
154	cgd6_4410	GATTTAAGTACCGGATCATGGG	TGAAATGGGCTTATCCTTACAAA
154	cgd6_4420	GGTGGTTATCATGCCCTTAGTT	TGTTTCAGGATCTCCCTTCTTC
154	cgd6_4440	CAAGCAACTCCACAAACTTCAC	TTTCTTCGTAATATGGGAACGG
154	cgd6_4460	ACAGGTGTAAGCACAGCCATAA	ATCTCTTGACTTCCCATCCAAA
154	cgd6_4470	AGATCCATTCCACAACATCTCC	TCCATCAATAGCGCAAACATAG
154	cgd6_4480	AATGCAAAGGATAAATACGGGA	CCGGAAATTGACTCCAAATAAG
154	cgd6_4490	ATCAAAGCAGTCAAAGCCCTAA	TGAGATTTGTTGGTTGCAGAAG
154	cgd6_4500	TTGACCCTTCCCATAGTTGATT	CTCCATTAAGGCATCTTTCCAG
154	cgd6_4510	ATGAGAGTGAGGCAATCAGACA	AAATTCAACCTCTTTGGCCTTT
154	cgd6_4530	GTTGAAAGTTCTGGGAAGGTTG	TGTCTCGTTGCTTCTGTTGTG
155	cgd6_4540	GATTGCTTTGCTGTATGCTTGT	TCTTCCTGGCTCTGTATTTGTG
155	cgd6_4550	AGGCAAAGAGGGAGGATACTTT	ATCCGTCAATTCCATTACTTCC
155	cgd6_4570	GGAAATGATAATGGGAGTGGAA	ATGAAGTAAAGAGCCTGGTTGG
155	cgd6_4580	TGTTACTCCAGAGGCAGAAGATG	TACATAATTGAGGGCAGGGAAG
155	cgd6_4590	TCAATGCAAATGCTACAAATGA	AAGTGCAAAGGAGTACAGAGCA
155	cgd6_4600	GAGACGACCAAGATTGAGACAAA	CAGCCAAGTCGAAAGGCTAA
155	cgd6_4610	TGTCAACACTCGGTTTGGTAAG	TAAGCTCATTATCGCATCCTCC
155	cgd6_4630	GCAACTAGAAAGGCCAGATTGA	ACATTGACCTGGACGAGAAGAA

155	cgd6_4640	AGATTGGATGCCAGAATGGAC	TTGCTTTGTTTCAGTAGTTCCCA
155	cgd6_4650	GTTTATGGTATGGGCTTGCATT	CGAAGAAGAACTGTGTGTGGTC
155	cgd6_4660	CAAGATGGCTTTAACACAAGCA	ATCCATTTCCAGGGTTAGGAGT
155	cgd6_4670	AGCAGCACTAGAGAGGAGTTGG	CATCTTGTTTGAATCTCGGTCA
156	cgd6_4680	CCTAGTCAAGAGCCCAGAAAGA	GCAATCAGAGAGCTAACCAACA
156	cgd6_4700	AAATGGATGAGGACTGGCATAG	AGAAACCCACAATAAAGCCAAA
156	cgd6_4710	TCCACAACAGAAGAGGATTTGA	AAATGAGCTTGGGATAAAGCAG
156	cgd6_4720	ACAACAGCAAACAGCAGGTAGA	GGGTCTTTGAGATGATAACGGA
156	cgd6_4730	ACGCCACACAAATCAGTACATT	TCAATGCAAGTAGTAGAAACCCTCT
156	cgd6_4740	CCCTCGATACCTTTCCATTTC	TCTGTGGAGTAGACCAACTAGGATT
156	cgd6_4750	AAGGGTACGAGATTGTGAAACC	GGAACATCCTTACTTTCTCCCA
156	cgd6_4760	AGCCCTACAATGCAACCTTATC	ACAAGTTACGCCAGACATAGCA
156	cgd6_4770	TGATATGACTGGGACAGGTTCTT	ATGACCTTGTTTACTCCCTCCA
156	cgd6_4780	TTTGATGATGGTTCTTCCGTG	GCTGCTGTGGTATTCTCTCTCC
156	cgd6_4790	CTAACACCGCCACTAATGACAA	TGAATAGAGTCCAGCCAAATGA
156	cgd6_4800	GCTTCCCTAATAACGTCACAGAA	GTTTCACCTGCAAGAAATCCTC
157	cgd6_4810	TTGAAGGAAATGGCAGAAATG	CTCCAAGTATTGTAACCGGAGG
157	cgd6_4840	TGCAAATAATAGAGGAGCACCAG	ACTCCCTTCCAGTTAATCCCAG
157	cgd6_4850	GACTCCCTTAGAACAGAATGGG	GCAATCACTCTACAAGCAGCA
157	cgd6_4870	CCAGGAAGAAGGAATCAAGAAA	TGACAAGATGAAAGGAGAATTTGA
157	cgd6_4880	GGCACTAATCAACAAAGGGAAC	TTGCTGGATCAACATAATCACC
157	cgd6_4900	TGGTAAAGTCCAGGGTGTTTCT	GAATCTTCGTCTCCCTCCAAC
157	cgd6_4910	GCCTTTGAGCCAGAATATGAGT	GATTTCTACGATGCCAGGAAAG
157	cgd6_4920	TCTAATGTGAAAGGCAAGACCA	TTTGAAGGTAGTTGTATGGAGGG
157	cgd6_4930	TGCTAGTGCTACACATGACCAA	CCTAATAACAGGATCTTCATCTTCACT
157	cgd6_4940	AATACGACCAAGAGGAAAGCAA	ATCCTGACATTCCAAGTGAACC
157	cgd6_4950	CTTCAACAACAGCAAGGGAAGT	CCAAACGAGGAGTTGGATAAGA
157	cgd6_4960	TTGGTCAATAGGATGCGTTATG	TCCAATGATATTCCTTCTCGGT
158	cgd6_4970	ATGTGGCAATATCAACAAACGA	CTGCATCAACTTTCTCACAACC
158	cgd6_4990	CCGACAGAGAGAGGGATTTATG	ATCAGAAGTTGTTCCTGCACCT
158	cgd6_5000	TTGATAATGTGCCAGGTTCAAG	ATATTCGTGGGAGATGAGTTGG
158	cgd6_5010	AATGTATATCAATGCAGAATCAGGAG	TGAACAGAGACAAGTAATGTGGGT
158	cgd6_5020	AGATTACCTTGGAATGGCACAA	TCTGAATCGTTTGGGACAGAA
158	cgd6_5030	CAAGCCTCGAAGTGTTCAGATT	TCCCTGCTATTTCAAGAGGTGA
158	cgd6_5050	AAGGAAGCCAATTTCACTCATC	AGGTGACTGTTCATTCTCATCAAG
158	cgd6_5060	ATTATAGTGGCGGTCAGAGTCC	TTTATGTTGTTGTCCGTCGTTT
158	cgd6_5070	TGGGAAATCCTTACTCTGCTTT	CTTGTCGCTTCATATTTGTTGG
158	cgd6_5080	TCAGGAAATTGAACAAGCAGAA	TGCTCCTAATACAGCTCCAAGTC
158	cgd6_5090	AAACGTCAGTTCCCACTATAAATGA	GTAGCAGGTCCAACAATTCCA
158	cgd6_5110	AAATGAGTGGTTGCTTGGATTT	TGATGAGACCAGTTTGAGGAGA
159	cgd6_5130	TCAAGTTCCCAAGAGTATCTCCA	CGACCTGTCCTCTAATTTCATTCT
159	cgd6_5140	GCATCCCAAGTACATAAAGCAA	TCTTCCATACAACCAGTCCAAA
159	cgd6_5150	ACAGCTTTGTTATGCCCACTTT	TTGGACTTGGATGTCTCTGATTT
159	cgd6_5160	CAGATTATGAATATAGAAGAGGCCAAA	GTTCTCCTGAAAGACTGATCCAA
159	cgd6_5170	GGTATTCGCCTTCTTAAACCAA	CCTTATCTTGGCTCATTCCAAC
159	cgd6_5180	GGTTACTTAATGATGGAGGGCA	TTCCCTTTCTGGAGGAGGTATT
159	cgd6_5240	AGAGAGGGAGAATGTGATGGAA	AATTGGAGAAGCTCTTTCGTTG
159	cgd6_5250	TTATATGGAAGCCGTTGGAAGT	CATTACACCTCCGTTTGAGACA
159	cgd6_5260	TCTCTACGTTCTTTCTGGAGGG	AAGGTGATTTGTTGGCTCATCT
159	cgd6_5280	AGGCAAATCCAATAGACCAGAA	ACTGAGACGCTCTGCTATCTCC
159	cgd6_5290	ACCAGAGAGTTTCCAGGTATCAA	GAATCCATCACAAGGAGAGACA
159	cgd6_5300	ACTCAGAATATGTGCAGGAGGG	TTTGACCTTGTGAATCTCTTTGG
160	cgd6_5310	GGAGTGTAAGAAATCAGCTTGGA	GATGGAGAGTTATGCCTTCCTG
160	cgd6_5320	AGGCAGATCAGAATCAAGGAAA	CCAACATAGCCAGTTACCCATC
160	cgd6_5330	TTACAAACCAGCAAGCTCTTCA	TCCTTCAAAGCTCCAATATGCT
160	cgd6_5340	GCTCAGTATTCCTTGGCTCATT	TCCTAGAAGACCAGCTCCAAAC
160	cgd6_5350	GGTTCCAATCCTAAAGAGCGT	TCTCTCCCTTTGAGTCTTCCAG
160	cgd6_5360	ATCAGGGAAGAAGAGGAGGAA	CAGGATCACTCTGAACGAATGT
160	cgd6_5370	TTCCCAGAAGATAACAAAGAAGAGA	CAGTTTAGGACAAAGCGATGAA

160	cgd6_5380	TTGTTCAGAGACTGCAATGAGAG	TCCAAGTTCAAGACAAAGAGCA
160	cgd6_5390	ACCCTTTCTTAACTCCTCCTTCA	TTCCAACCTCCAAATATCAGTTC
160	cgd6_5400	AGGAGATTGGCTCTGGATGTAA	GTAGGCTGAGAGGTTATGGGAA
160	cgd6_5410	TTCCCACAGATTGACATTGAAG	GGAGTTTCCAGAGAAGCAAGAA
160	cgd6_5420	CCGTTGGACAGGAGTTTGTATT	AAGGTAAGACAGCAAGTGGAGG
161	cgd6_5440	CTGCATGTGGAAAGGTATTTGA	GACATCGAAATCTTGTGGTTGA
161	cgd6_5450	TGGAAAGAAGGTTACAAGCAAAG	AATTCTGCGGAGTAAAGCAAAG
161	cgd6_5460	TGTAGCTCCTATTCAACATCCATT	CAAATGCCAACATGATTTCTTC
161	cgd6_5480	ACAACCATGCGATGATGAAA	AACCTCTTGGCCTTGAATTGT
161	cgd6_5490	TGAAGGTTTGAGTAGAGGTGGAA	TGATGACGATGATGACAATGAA
161	cgd6_5510	ATACCTGGGCTCCACTCTCA	TCATTAAAGCTCTCTTCATCCGT
161	cgd6_5520	CAATTTCTCAAACACAATTATACGGAA	CCGACTTGAATATGGAGAGTACCA
161	cgd7_10	ATGGAGGACAATCATCACACAG	AGCCAGAGCCATCATAAGTCA
161	cgd7_30	AAGGCCAGCAAATACTCAACTC	ATCAACCTTGTTCTCTTTCCCA
161	cgd7_40	TGCTTCGAGAATCCAAACTACA	CAATTCCAGCGATAGGACTCA
161	cgd7_60	TCAACGAAGAGCCTAACTCACA	TAGCAACGCAATTTCCTCATT
161	cgd7_70	ACCACGTATATGAGCAGAACTCAC	CCTTGCGAAATAACAGCAGAC
162	cgd7_80	TATTGATTGGCTGATTGATGGA	GTTGGAGATGATAGAAGTGTGCTG
162	cgd7_90	AGCCTCCAAGCACTAACTGTGT	AACTGAGAAGGGAATGGGAAGT
162	cgd7_100	ATAGCCACGCTTCTAAATGCTC	ACAAAGGTAGGACCAGTCGTGT
162	cgd7_110	GGCGTCAAATGGCTATATCTTC	TTTCTCTGTCAATGTATGCGGT
162	cgd7_120	ATCTTCGGGATCAGAAAGTGAA	TTGGGTAGCTTGGAGGAGTAAA
162	cgd7_130	GAGTCAAGTCAAAGACAATGGGT	TCTTCTGGTGCTATCAGTTGGA
162	cgd7_140	CACAGAACCCTCCAAATACACA	TCTGCCATCCCTAATATCCTTC
162	cgd7_160	TATGTTTGGGCAGTTAAGGTGA	CACGAGTATGCTGGAGAATTTG
162	cgd7_180	CCAAATGAGGAAACTAACCTAAGAAG	TTGCATAGCCAACTGGAACA
162	cgd7_200	GAATCGTATTGCTTCCGTTTCT	CAAACCCAGTAGCCAAATCTTC
162	cgd7_210	CTGTTGTGAAGATGGATGCTGT	CACCTTTCTGTGGCTCTTTCTC
162	cgd7_230	AACAAGGAGTAAGGTGTGACGG	GGCTACAGCAGGCAATATAGGA
163	cgd7_240	TTAGGGAAACTCTTGATGGTGATT	CTTCAGATGGGAGGATGGATT
163	cgd7_250	GGAAATGTTGGAACCGAGATAA	GAATGGACTGAAACCAGGAAGA
163	cgd7_260	TGCCCAGAGGAAGAAATAAGTG	TTTAGTGGTGAGGGTTGAGAGG
163	cgd7_270	CAATCTGCAATCCTTGTCTTCA	TATGAAATGTCCAATGCCACTC
163	cgd7_280	TTTAGGAAGTGGAACATACGGG	ACTCACGAATAGAAGTGGCAGG
163	cgd7_290	TTATCAAGCAGGTTCAATGCC	TGCCTCCAAATCAATCTCTACTC
163	cgd7_300	ACACAGTTAGGCAGTGGTGTTG	TCTGCTTTCACAAGACAAGGAA
163	cgd7_320	AAGCCAACAGAGCTTAATCCAC	TGGCCTTCTTGACCTTATTCA
163	cgd7_330	ACATGGATACAAACATGGAGACA	GTGGGACTTTGCTGTAGAATGA
163	cgd7_340	AGGCATTATTGAACAGGCTTTG	TTCCGTAAATTCGTGATCGTTT
163	cgd7_350	CTTAGGGATGGGTATTGGGACT	CCATGAACTGTGTAGGGAGGTT
163	cgd7_370	GACCCAAGTTTGTTAAGGCATC	TTTCATCGCAATACAGTCCATC
164	cgd7_380	TCTTTGGGACTGGAATAGTCGT	TAATTTCGCCATAAGTTGAGCC
164	cgd7_400	AACCATCTACAATCCCAGAATCA	GGTTGCATCTACCAATCCATCT
164	cgd7_410	AACAGGAACAGGAACAGGGAC	TTGCACCTAAATCTACACGTTCTTT
164	cgd7_420	CGTGTTCAAGCCAAAGTATGAG	TCTGGTTCACATCCAAGTTCC
164	cgd7_450	TTTGGTTCATACTTCCTTCATGTC	AAGCTCCAATAAATCCTTCAACC
164	cgd7_460	CGATGGAGGGTATTATGGAGATT	TTCGCATCCTTTACTGAGCTTT
164	cgd7_470	AAATGTCGGTAAATACTGCCCTAA	ATCTGAACCTAGCGGAATCAAG
164	cgd7_480	GGTCTTATCACCCAGGAACAAA	GAACATGGAACAACTGCCTTCT
164	cgd7_490	GTAGCTCTGGCAATACTGGTGA	TCCTCATCTAGCTTCTGGCTCT
164	cgd7_500	CATGGAAGGTTGGATGATGAC	GAAATCCCTACTTGGCACTGAA
164	cgd7_580	TTTGAAGGCTCAGTTTCCTCTC	GCTTCTCCAATTTATGAACCCA
164	cgd7_630	CAGTACCAGAGATGCTGGATGA	CTGGCTCAAATGATGAAACAAA
165	cgd7_640	AATAGTGTTCCCGAGATCCAAA	ATTATCATCCAAAGCTCCCAAA
165	cgd7_650	GGACTAAGCACAACGGTAGGAC	TATAAGGCAAGTTCGTGAGCAA
165	cgd7_660	GCATGGGAACTTACAGGGTTT	AATGGATGACGTGGCAATAAG
165	cgd7_670	AAGCCGTTGGAGAACTTAGAAA	AAGGTGAGAATAAGGGCAAGAA
165	cgd7_680	AAATTCTGGCATCAGTGGGTAA	CATGATCTTTCTGGTCTTGCTTG
165	cgd7_690	TGAGGAAACCTACTATGAAGAGGAA	GCTTTACTGCTGGCTTGACC

165	cgd7_700	CAATAATAAAGCAAGTGTGTGGAGA	AATTCCTTCTCTCTAAGTTTACCCAA
165	cgd7_720	CCCAATTAGAGGAGATGAAGAATG	ACGTCCCTTTGAAGTTGTCTTG
165	cgd7_730	TCCCTCAATCCAACATTACAAA	CAGTTCCCACACACAAATTCTC
165	cgd7_740	GGAGAAGAAAGACGAAGCTGAA	CAGATATTGCTGATCCCATGAA
165	cgd7_750	GAGATGATATTGGTGGATGGAAA	TGAAGAAAGCACAAACTCCAAG
165	cgd7_760	GCTGATGGAGAGATTGAGAGGT	TTTGCTTTGCTTGTTGTTGTCT
166	cgd7_770	TTTATTATTGGAGAGCCTTGGG	TCCCATCGAGAGATTCCATAGT
166	cgd7_780	TTCTTCCATTACCATTCATCCC	AGTTTGGAGTGCGGTATTCTGT
166	cgd7_790	ACTAGAGATGGCAGGCGATAAG	TCTTCTACATCAACCTCGACAGAA
166	cgd7_820	CACTCGTACTGTGGCTGAAATG	ACTGACGCAAGAGAGGAATTGT
166	cgd7_830	TTTATGAGGTGGAACAGGAACA	GATGCCTAAATTCGACTCCTTC
166	cgd7_840	AAGCCAGAGAGAATGATAAAGGG	TGCCTGAATACAAATCTCGAATAA
166	cgd7_860	TTATAGATGACGTGGCATTGGA	GCAATCTGTTGATGATTTGGTG
166	cgd7_880	GTTGTTCCACCAGAAGAAGGTC	AACTTTAGGAGAGCCATCAATCC
166	cgd7_900	CACTCAACCAAATCCCAAGAA	GATGATGATGAGGAAGAGGAGG
166	cgd7_910	GCACATAGTTCAATGGTTGGTG	TTGCTAAGAATGGTCTAGTGGGA
166	cgd7_920	CCTGAGAGTAAGGACGAGAAACA	TAATGTTCCAAGACAATGCCAA
166	cgd7_930	TTTAACCAAACGCACTTTCTTG	TAATTCCCTCGTCAATCCCTT
167	cgd7_940	ACTTGTTCGTAATCTGCGCTTT	TATTCCACAAATCCGAATCCTC
167	cgd7_950	CAACTCTTCTTTGCTGGTGTTG	GTTGTTGGACTAAATGCCTTCC
167	cgd7_960	CCTTCGGATTTATCTTCTGGTTT	TTCTCCCGTCTCTTCTGGATT
167	cgd7_970	AGCTTTGGTTTCTGGTCACTCT	AGAGGATTCTGTTTGCTCCATT
167	cgd7_980	TGCATTAGCACCAGAAATACCA	TAACCATACATTCCCAACGACA
167	cgd7_1000	GTTCCTGACCAGATTACAATAAATGA	ATACTTTCTCGATCCACTGCCT
167	cgd7_1010	TGGATGAAGAAGGGAAAGAAGA	TTTGTCTTATGAAATGCCTCCC
167	cgd7_1020	TACTGTTTGGATCACGGGTATG	TCAATATCTTCACCATCGTCTTGT
167	cgd7_1090	TTCGTAGCTTACAGACCTTCTCAA	GATTTCCCGTAGTTTCATGTCC
167	cgd7_1110	CGCAAATTAGGTCTCCAATCTC	GGAATTAGAATGTGAACGGGAA
167	cgd7_1150	CTCCAAATATCTCAAGAGGAGCA	CCTTTCTAATATCCATCCCAACTCT
167	cgd7_1160	AATGGCGTTTCTTCTACTCAGG	ACTGAACCTTCTTGATTCCCAA
168	cgd7_1180	TGAACCACAAACACTTTCATCC	AGTTCCTCAATCTCCCATTCCT
168	cgd7_1190	CTCTTCCTCTTCCTCTTCCTCC	ACCTCCGTTTAAGAATGACCAA
168	cgd7_1200	AGGAGGAAGAGGAGAAGGAGAA	ATTGTGGAGAAGATAAGCCGAA
168	cgd7_1220	ATGTACCCAGTCATGGCAAATC	TGACCAATTCAGACCCTCTCTC
168	cgd7_1230	AACTGAGGGATTTCCAAGTGTC	TGCATCAATTTCACACCTTCTT
168	cgd7_1240	TGGGTAGAACAAACCTCAACAA	GAAACCGCACATATAAACCACA
168	cgd7_1250	TGCGTACAGAAACTGGCAAA	GTTACACCACCATAAGTAGTCCACC
168	cgd7_1260	TAATCAAGCTCAACCTCAAGCA	ACTTCGTCCACACTAGCAAACA
168	cgd7_1270	GGTAAGATGGTAGTGCCTGGTC	ATTTCCTTGGTTGTTGATTTGG
168	cgd7_1280	GTATGTTCGGTCTCGGAAAGAG	GAACCTTCGTTTGAGGAATCAC
168	cgd7_1290	TTCCAGAAGGTGGAGAAAGATG	GAGCTTGGTCTGCTACAATTCC
168	cgd7_1300	GGTGTGTCTATGAACTCGGAAAG	GAAAGTCGTTGGTTCTTCCATC
169	cgd7_1320	TCTGTGGATTCTTGGTCAGTAGG	CATTATTGTCCCAACCTGGAAA
169	cgd7_1350	CTACAAGACAAAGCCAACCCAG	CCGGAATAGCATGATTAAGGG
169	cgd7_1370	AAGGGTTTCAAGTGGTTCTTCA	TTGTCTTCTGCTTGTTTGTGGT
169	cgd7_1380	TGGGTGAAGAAGGTATTCCAAG	GGTAGAAATCCAACAAGACTCCC
169	cgd7_1390	AGGAAGAAGCTGCATTTAGAAAGA	TGGATGAGCAAACAACCAATAA
169	cgd7_1400	ACCGGGATCAGTATCAAGTAAATC	TAATATCAGTGCCGCTTGTCC
169	cgd7_1410	AGTCTTTGATTGGGCTCGTAAA	ATTCTGTTCCAGTTTGGTGGAG
169	cgd7_1420	ACAGCTCAATCTTGCTTTACGA	TTTCAGCCAATTCTATCCCTCT
169	cgd7_1430	TCTCCAAGAAGAAATAGGAAGGG	GGAGTATCCAAATGAGCGAAAG
169	cgd7_1440	GGTAGAGTTGAATTTGGATGGG	CATTGCGATACCCGATTTATTT
169	cgd7_1450	AAGCAAGACTGCAAAGAGGATT	ACTGATATGTATGCGGATGCTG
169	cgd7_1460	TATTGAACCCAGATCCCAGAGT	AAGTTTGCATCTTCCACCTGTT
170	cgd7_1470	GGTTCCAAAGAATCCTCCATT	TGTGCAAACTCTCCAACCTTAC
170	cgd7_1480	TTTGGGAACATGGGTTATTGA	GGTCAGAAAGAATAGTTAGAGGTGAAA
170	cgd7_1490	AAGATGAGTGCAAGTGTCCAAA	ATTGCCATGAGACATCAACATC
170	cgd7_1500	GGAAAGAAGAAACAAGGCTCAA	ATCACCCAGAAATGCCTGTAA
170	cgd7_1510	ACGCAACCAATCACAAGTATCA	AGGGAAACATGGAACAAATCCT

170	cgd7_1540	TCACCCATAGCCTGTAAACTCA	CCCAAGCTGTCTATATTGCTCC
170	cgd7_1550	CTCCAATACAATCAGGAGACCC	TGGCTGGTACAAACATCACTTT
170	cgd7_1580	GCTAAAGAACTTGGTATTCCTCCA	CGGTTGGATCTAGGGTATGAAA
170	cgd7_1610	TACAAGAGCAAGGAGTTGGAGG	GGAGCCAATAGAGAACCCATTT
170	cgd7_1630	TATTTGTTCTGGTGATGGGACA	GGATTTAGGCTGCATGTTGAGT
170	cgd7_1640	ATAGACGCCGACAGATTCAAAG	GAACCCTGAAGACCAACAAAGA
170	cgd7_1650	TAGCAGAAGACCAAGGAACCA	CATGGAACCAACTACAAGCATC
171	cgd7_1670	ATTGTGCTTCAATCTGTCTCCA	TGTTCAAGGCCCATCTTAGTTT
171	cgd7_1680	TGTTGTTCTGGGAAATAAGTTGG	GCATCTACGTTCGTTGCATTT
171	cgd7_1690	AATCACAACAGGAAGTGAGCAG	ACAGACTTTCCCATTACCTCCA
171	cgd7_1700	GAACAGAATCCTTTGCTCTTTACATC	TGAAGTCTGAACCTCTCTGCTTG
171	cgd7_1710	GACTTTGGGATGCAACTAGACC	ATGTGATGAAGAGTGCCAGAAA
171	cgd7_1720	TCTTCCAGAGGATACATGGGTAA	GAAAGAAAGGCTGCTACAAGGA
171	cgd7_1730	AATATGGGAAATAGCGAGTGGA	CCTGGATTTGAGTTTCTCTTGG
171	cgd7_1750	GCAAGAATTGACTTGGATAGTGG	TTCTGGAATTATCTCGTCTGGG
171	cgd7_1760	TAACTCGGCTTGGCATATCTTT	GGCTGCTCCTACTATTGACTGG
171	cgd7_1770	CAAGGATCTGGATGGAATTGTT	GAAACACCTGGATGGTCACAC
171	cgd7_1780	GATCAGGGAGGCATTATCAGAC	ATGCGCTGTAACCAAAGAAACT
171	cgd7_1790	TGAAACAAGATATTCCTGGGCT	TAAAGCTGTTCACAAGGCTCAA
172	cgd7_1800	CTTCGACTTGTGTTGGGTATGA	GATTTGGACAGGTTCTTCTTGG
172	cgd7_1810	TACAGGTAAAGGCCCAAACAAG	CCAACCCGCTAGAAGAGTAATG
172	cgd7_1820	TTCCTCCTTGCTATTCATCTTCA	CTTCCTCGTCATCATCATCAAA
172	cgd7_1830	GACTTTGGAAACTTCGTGTGTG	CAGGATAACACCCTTCTCTTGG
172	cgd7_1840	AACACCTCCGCTAATGTTGAGT	GGTTCCTTTCTGGACAGCTATTT
172	cgd7_1850	TTATGGAGGCAGCAAGGTTATT	ATTGTTTGGGTAATGCTTGGAG
172	cgd7_1860	GAAACAGAGATATGCCCTTACAAGAA	AATGGCTACCAATCCGAAGG
172	cgd7_1880	AGATCGTCTTGAGATTGGAGTGT	ACTGGACCTCCCTTTCTCTTCT
172	cgd7_1890	AGCTTGGCAGACATTTCAGATT	GATGAATACTTACCAGGCGGAC
172	cgd7_1900	AAAGGCTATTGTGGTTGATGAGA	CGAAGGACGACTTTAGAGCATT
172	cgd7_1920	TTTCCTGGGTTTACTACAAGATGA	ATTAGGCAGATGCAAATAGATTGA
172	cgd7_1930	TTCAACTCTTTCCAACAGCATC	TCGACCCTCTCAAATGATACAA
173	cgd7_1940	AGATCAAGCCAAATACCTCCAA	GCGTAAGCTGTGTTCCTAAACC
173	cgd7_1950	TTGTAGAAGGAAGGAAGCCAAA	CACGAAACCTTAGTCAAGCCA
173	cgd7_1960	GCCTGTTGTTCTGATAAGTTGCT	TGTTCCAATGTCTGATGACTCC
173	cgd7_1970	AAACAAAGAGTCGGTTGGATGT	TTCAGCTTTCTCATCTTGTGGA
173	cgd7_1980	TCAAACACCTTTACAACCCAGA	CCACTTCTCGATCAATCATATCC
173	cgd7_1990	TGTCGTGCGTAGGATTAAGAGA	TGCCATCCGCTTCTAAGTATG
173	cgd7_2000	TCTCCAATAGATTCGCAAGACA	TGAACACCAAATAATGAGACCAA
173	cgd7_2010	ACCCTCCTCTGCATCTTACTGA	CATCACATTGCCTTGAATGACT
173	cgd7_2020	CTTAACACAAATTCCTCCAGCC	GGTCGTCTCTGTGGAAATCAG
173	cgd7_2030	CCTCTTGCAGTTCTCCATCATT	GTAGTGCAGACAACAATCCGTG
173	cgd7_2040	CATTGGAAGTCCGGTTATTGTT	CTAATGAAGGTATCTCCGTGGTTT
173	cgd7_2050	ACTGGCACTGGGAAGAGAAAG	TTACCGAAATAGACGCTTCACC
174	cgd7_2070	AGGAGAATGTTGGAAGAGATGG	TTAGATTTGAATGGCGGAAAGT
174	cgd7_2080	TGATACAGGATTATTTGGGATTCA	TAGCTCTTTCAAGCTCCCTTTC
174	cgd7_2090	AATGGTGGCATCTGAAGTGTATT	CTAATTCAACAACCTCGCCTTC
174	cgd7_2100	ATCTCTCCCAAGCGAAGTATTG	CCAGTAAATGCAAAGACTCATCC
174	cgd7_2110	TTTCAAGGCTAAGGTTTCCAAG	TTCTACAGCAATCATCAGCTCC
174	cgd7_2120	TTAATGAGGGTATCCCAGACAAC	AGTATCCTTCCCATCAAAGCC
174	cgd7_2130	TGGTGGGATAGAAGAGTATGGG	GAACGCTTAGAACTTTGTCCGT
174	cgd7_2140	GCTATTGCGAAAGAATCAAGAAA	ATAAAGGTGGCCTAGAGGGAGT
174	cgd7_2160	CTACAAGAAGCGAATTGAGGATTT	TAGGCTCTTCAGGCTCCATAAC
174	cgd7_2170	TTGAGGAGCTGATGAAGTCAAA	GGCCTTTATTGTGTGAATAGCC
174	cgd7_2190	CCTCTAAGCAAGCAATAGAAGACA	GTCCATTTGAGACTTTGGAAGAA
174	cgd7_2200	ACACTCTTTGTTACCGATGCTG	TGGGCTCTACTTCTTGAGATACATT
175	cgd7_2210	AATGGGAAATGGAACAAAGAGAG	CAATCTTACCAATCTTCTTATCTGGG
175	cgd7_2260	CCAAGGCAAGATTTACTTACAGG	GTTCACCTTTACTACATACCAATAGCA
175	cgd7_2270	CCTGCAACTTCACCACAATCT	ATCACCAATTCCTCCATTTGAC
175	cgd7_2280	AGACTTAGAGGCGGTGTAATTGAG	AGCTTCTTCTTTGGACGGATTT

175	cgd7_2290	AGTAGATGCCCGCAATGATAGT	TATCGAGTAGGATGTGGAAGGG
175	cgd7_2300	CATTCGTGAAGAGAAGCGAGT	CAGCATCAGTTGGTAGTGCTAAA
175	cgd7_2310	TGCAAGCATTTATGGAGATTGT	TCACTTTCATCACTGGCTGTTT
175	cgd7_2320	ATGTCTTCATTTCCTCCAAATATCC	CCCACTCTCTGCACTCTTGTTT
175	cgd7_2330	AGCAACTCACGTACCCACTCTAC	TTCCAAATTCTCCTTGCAGTTT
175	cgd7_2340	CAGGCAGGAGTATATGAAAGCA	TCGTCAGGTTTCCAAGTATCAA
175	cgd7_2360	GCCAAAGTATTTCTCCAAACTTCA	CATACGAGCCGGGATACAAA
175	cgd7_2370	CAATGGATGAAGGGATTATTTGA	TTAATGAGGAATCAGCCGAAGT
176	cgd7_2380	CTTTCAATGTTCATCACTTTCCC	CCTCCGAATTTACTGATTCCAC
176	cgd7_2400	TTTCTTTATGGCAAAGGTGTTGT	CAATCACTGTATCTCCGGGTTT
176	cgd7_2410	TTAATATGCGAAAGAGTAGACACCC	ATCCATTTGCTCCATGAAACTC
176	cgd7_2430	CAAATGTATGTCCTATGCCCAA	ATGATTGTTATCCAATGCTCCA
176	cgd7_2440	GATAAGGTCATCGACTCCTCGT	ACTGGGATATTGTTGGGAGAAA
176	cgd7_2450	CACAAATTACGAGCAGGAATGA	CCAATAGCCAAACAAGAAGGAA
176	cgd7_2460	CTGGAAAGGGATCTAAACACGA	AATTCGCCAACATAAATGGAAA
176	cgd7_2470	CAAGTATTGCAGGAACAAACGA	CTCAACCAAGCGATCTACACAA
176	cgd7_2480	GTTCTTGGGCTAAACTTCTTGG	ATTCGATATGCTGGTCTCCTTT
176	cgd7_2490	TACTCCATTTCATTCACTCGCA	TGTAACGCTCAATCTACCTTTGG
176	cgd7_2500	TGTGAGTGGGATGAAGAGTTTG	GGTAATTCGCTGCCTAAGAAATAA
176	cgd7_2510	ACTACTGTCAACGCAACTCTGG	GCGCAGCAAATGTTAATAGAGA
177	cgd7_2520	TGCTTTATCATTTGAGAGTCGG	AAACATCTTCATCTTCAGCATCAG
177	cgd7_2530	CTAAGGATTGTTTGGGAGTTGG	GCCCTTCTGATGTATGGGATAA
177	cgd7_2550	AATTCATACGAGCGATGGAGTAA	CCCTTATCTGCCAAACTCAAAG
177	cgd7_2560	AGAGAAGGCATAATTGAGAGACAGTT	TTGAATTGGAGCTTGTTTCCTT
177	cgd7_2570	TGGAAGGCAAACTCACTAAACC	TGTCACCACTATCATCTGCTCC
177	cgd7_2580	GACAATTTCTTCGTCCAGGGT	TCCTTTATTCTCGTGGTCTGCT
177	cgd7_2590	TTCTCGCAATTCTAGGTATTCACTT	TTTCAACTTTGCCATTACAATCA
177	cgd7_2600	TTATTGCTGAGACCAACGGAG	GTATAGATGTTTGGGTGGCGAG
177	cgd7_2610	TTAACTGAAGGAAAGGCAAAGG	GGGTTGTTGTAGGAGATGGAAG
177	cgd7_2620	AAACTCTGGCAAGGAAACACTT	TCACCAATTACAGGTTGAGATGA
177	cgd7_2640	AGTCTTGTATTGAGAGGCTTGACTT	GACGCCTTTGCTAAATTCTCTT
177	cgd7_2670	AATATCAGGAACTGCGAATGGT	ATTCCGCGTATCTCTTTCTCTG
178	cgd7_3780	GTACCGCTGTTTCAGGTAGGAC	CGATGGTCGAAAGTAATCACAA
178	cgd7_3790	GCGAAGTGTATTGTTCAAGCAG	GGAGGTGTATGGGACTAAACCA
178	cgd7_3800	GATTCCTGATGGAGACTTGGTT	AAAGCGAGATGTTCTTTCCACT
178	cgd7_3810	TCATAAATATCCTTGGTCGCCT	TGTCTTCCGTACATGCTGACTT
178	cgd7_3820	GTTGACGAGATGGCTGATACAA	GAATATGCTCCCTAAATGCCCT
178	cgd7_3830	GGCACTCAAAGCCTCTACTTTC	GCCAACAACAGAAACAATACCTC
178	cgd7_3840	GAATTGGTGGGCTGTTAGTTTC	AGAGCAGAGTTGTGGAAAGATTG
178	cgd7_3850	ACGACTCTTCACTTGGCTGTTG	CGGATGCTCTTTCTAATTGGGT
178	cgd7_3860	TAGGGAACAAGAATGGGAGAGA	GGCTCTGTATTTGTCGGTTTGT
178	cgd7_3870	AAGAGCTTTCAAGTTTGCATGAG	TTTGCCCTTATCAACTCCTTTG
178	cgd7_3880	TGAACAGGATATTGTCGTTGGA	TTTGTTGTTGGGAAATAAGGAGA
178	cgd7_3900	ATTGATGTAGATAAGGCGGAGG	GTTGAGTCTTCTTCTGCTCCAGT
179	cgd7_3910	CGCAGTCCTACTCAAATTCTCC	TTTCTGCATTCTTCTTCGTCAA
179	cgd7_3920	GTGAACGTATAATGAATAAAGAGCGA	TACTTCCTCTCCCAATGCAACT
179	cgd7_3930	AGGAAGTAAATATGCTAAATGCAAAGA	TTAAACTCCAAAGCCTCATCAAA
179	cgd7_3940	TCAAAGACGAGCAATAGTTCCA	AATCTCCAAAGCACCCACAG
179	cgd7_3950	CACACGAAGCACTCAAGCTATT	GTTTGTCTTTCCCTCTCCCTTT
179	cgd7_3960	ATCAAGTTAGCAAATCCATGCC	ATAACGGTGCTTCAGTATTTCCA
179	cgd7_3980	ATTATATCCGGTGGGAACTTGA	TTTGTTATTAGGTATGGCGGCT
179	cgd7_3990	AAGGGCAAAGTATCCAAACAGA	AACCAACATATTCATTGGGACTAAA
179	cgd7_4010	AACGATGACCAACCATCCTATC	CCTTAATCTTTCCTTCGCCTTT
179	cgd7_4020	ATCTATTCCTCCAAGCGTACCA	CATTATTGGGTTCAAGTCACCA
179	cgd7_4030	ACATTGCCACAAGGAATAGGAC	CTTTGCTCCATTACAGACTCCC
179	cgd7_4040	AGTTGGGTCAATCTGGGATG	GTAAACTACGGAAAGGAGCGAC
180	cgd7_4050	GCCAATCCAAATTACAGACTTGA	ACTCCAACTTCTTTGCCTTCTCT
180	cgd7_4070	AGATTGTGATCTTGAACCCTCGT	CCAACCTAGACGCTATGGAACA
180	cgd7_4080	TGGAAGTATGGCAGATGGAAG	TGCAGGGAACATTAACAAAGTG

180	cgd7_4100	AATTGGTCAGATTGTGGTTTCC	TTGAGCATGAGCCCTACTAACA
180	cgd7_4110	AGGCTTTCTTGAGACTGAGGGT	CAAGCATTGAAATATGCCCTTT
180	cgd7_4120	CTAAGATCATGGAATTGTGGTGG	TGCTTGGTTGGCTCATCTATC
180	cgd7_4130	TGAGTCTAACACCAACTGCTGAA	CCATGTTGAGGTAATCCGCT
180	cgd7_4140	TTGGTTGTAGGTACTGTCGGG	CGTCTCCACATGCAATAAAGTC
180	cgd7_4150	CCTGAATATCTCTGCTGCTCCT	TTGGGTACTGTGCTTGATTCTG
180	cgd7_4160	CAAAGCATTCAACGAGAAACTG	GTGCATCTTCCAAGGGTATGA
180	cgd7_4170	TGTTTGGCTATCTCGTTTCTTG	CACGTTAATTCTGGTGGATTTG
180	cgd7_4180	ACAGATGGGAACAGAATAGCGT	AAAGCATTTCACCAACAAACAA
180	cgd7_4190	AAAGAAGGCTCAAGGACATCAA	AACACTCATGGACTTTCGGTCT
181	cgd7_4200	CTTGGGTTCCATTAAGCTCATC	TTGAAGAATACGAGAGATGGTGTC
181	cgd7_4210	CAGAGTGCTCAAGAGTTTGCC	AATGAATCCCATCAGGAAGAAA
181	cgd7_4220	AAATCCAACATCCAACAGCTTC	CCAAATCTATCCCTTCGCATAA
181	cgd7_4230	GAGATTTCTTGGAGCGATAGGTT	ATTCCTCTTTGCAGGATTTGAG
181	cgd7_4240	GTCTCTCCCAAAGCAATTTCAG	CTTTAGTCCCAGTCGTGGTGTT
181	cgd7_4250	ATGATCGCCATTTGGACTATTT	TGCAGTTCTCAAGTATCTCGCT
181	cgd7_4260	TGAGTTGATGAGAGGCAAAGAA	TCTTCCATAGTTCTGGTGCTGA
181	cgd7_4280	TAACATCAAGCCCTCCTTCATT	GTACCTTCTCATCCCATGCTTC
181	cgd7_4290	CAAAGACTATCAGCAAAGCCAA	CCATTCCCAGGATAAATAACGA
181	cgd7_4300	TTTATTTAGAGCAAAGAAGAAGTCCC	ACTGCTGTTCAAGAAGCGAAC
181	cgd7_4310	TTACAAACACCACAGGCAGTTC	TTGCGATACATTTCAAGGACAA
181	cgd7_4320	ATGAAGTAAATCCCGAAGCAAA	AAATTATCCAAGATTGAAACAGATGAA
182	cgd7_4330	ATTGGTCTGAGCGGAGATAGAA	CTGTACCCAGAATGAATAGCCC
182	cgd7_4340	TGGAGACAATTTCAGAGCAAGA	GCAATTTATGGGACAGCCTAAC
182	cgd7_4350	CAAACCATATCTTCGGGATCAG	TTTCTACCGCTATCCAACCATC
182	cgd7_4370	TTGAAGATCATACACTTGGAAACC	GCTCTAATGGGTGAGGTACTTTG
182	cgd7_4380	TTTGCTACAAGAACAGTGCCTC	ATACACCAATAATGCACCCACA
182	cgd7_4390	TTAGATCCAGAAACCAGAGCTACA	CGGATGAGCTGCCTTAAACT
182	cgd7_4400	AGCCTGTTTCATTTATTGTGCC	TCTTGAACATATTGCTCTGCGT
182	cgd7_4420	CAGATATGGTTGCTGGAATTGA	TTCAACAGGAATAAAGGTGGCT
182	cgd7_4440	GGCTAAGGCAATGGAAGAAGT	GTCTGTTTGATCCTGCTCTGTG
182	cgd7_4470	TTATAGATGGTTCCTGTCCTCCA	AAGTGTTCCTGTTCGTCCAAGT
182	cgd7_4480	TGGGAGATCAAGAGGTTATGGA	TATAGTGGATGTGAGGCGTTGA
182	cgd7_4490	CTGAAGGAATCTCCAAATCCAA	TATCATCACATCCAAATCCCAA
183	cgd7_4500	CCAAGACCACAACCACTACAAA	CCTAAGACGAAAGATGGGAATG
183	cgd7_4520	ACCCTGAATGGTATGCTCAAGT	CTAATTCTTTGTCGTTGGCCTC
183	cgd7_4530	AAGAAATGGGACACTCCAGAAA	TCTCTAATGGAAACTGGTGCCT
183	cgd7_4540	CGATGAACACTCCCTAACCTCT	CCATCTCCAGAAAGCGAAATAC
183	cgd7_4560	AACTTTGGGTGTTCTCCACACT	CTGAATCCACATCTTTGTTCCA
183	cgd7_4570	TGAGGAGGAAGAGGCTTTAGTG	GATATTTGGACCTTTGGCGTT
183	cgd7_4580	AGGAAGTAATCAGAAGGGAGGG	TCCTGAACATCATCCAATACCA
183	cgd7_4590	CGAAGAAGGGTACGATTTGAAG	AGCATTGAGGATCGCTAAGAAC
183	cgd7_4600	TAGAATTGGGAGAACAGGGAGA	CCTGAAGGGAAGTATTTGTGCT
183	cgd7_4610	TTCAACCTACCACCCTCAGAA	CTGGCTGTGATGCTGACTTTAC
183	cgd7_4620	TCTCTCGGACCAAACTACCACT	AAAGCTACGGCTCTGATTGTTC
183	cgd7_4630	AGTAATTGGCGGAGTTATGGTG	TGAAGCAGACGAAGAAATACCC
184	cgd7_4640	TATATTTGTGTTGCGGATGGAG	AACTCGCATTTCTGTTCCCTT
184	cgd7_4650	GGTTAAGAAGGCAGTGATACCAAA	TTATTCCGTCTACATCCAAGCC
184	cgd7_4660	CAGATTCATTAACCGAGAAGCC	TTTCATCTTCCAAATCCAATCC
184	cgd7_4670	GAGGAAATCGAGGAAGAGGTTT	TTGAGACGAAATTGAGCTTGG
184	cgd7_4680	TCAAATGGTGAAACAAATGAGG	GACTCTCCTCGTCCCAAGTAAA
184	cgd7_4690	TTAGGCATCTAGGCTCCTCAAC	GAAGGCATTTGTCATGGTTTCT
184	cgd7_4700	ATTCAGATGCCAAGAGTTTGATT	TGTTCGTTTGGTCTCACAGATT
184	cgd7_4710	AGAGGAATGATGGGAGTTCAAG	TATCAGACACAGAGGAAGCGAC
184	cgd7_4720	CGCTTTCGTCATATCGTTCA	GGACAACTACACAAGCCATTCA
184	cgd7_4730	TTTATCTAAGGGTGCTTCCCAG	CACTTGCTGCTGGATCTGTATT
184	cgd7_4740	CTCCCAAGATGGAGACTACTGG	TGAATGTTCTGGGTTGTTTGAG
184	cgd7_4750	TTAATTTGCCCAACTCCCAG	AGTACAAGTTTCCTCGTTCTCCC
185	cgd7_4760	ATTTCTGATTTGCCTTGAAACC	ACTGAGCACCCATCTTTCTCTC

185	cgd7_4790	CAGGTAATTTCTCTCCATCCCA	TTCGACTTCTATTTCTGCCTCC
185	cgd7_4810	CAACTCAACAAGCACCAACTTC	TGGCTCAACATAACACCACTCT
185	cgd7_4820	CTTTGAGGAATTTGAACATGGG	CTCTGCTAACCTCTCTTGGCAC
185	cgd7_4830	CTAAACTGCGCCCTCATATTGT	TGGAATCTCCCTTATTCATTGG
185	cgd7_4840	GCTCCAAGGGAACAAGAGAAG	CGTCTTTATCAACCTCAACATCAA
185	cgd7_4860	CCCTCTCTCTTGAACCTGAAAC	AAATTCCAGCTCTTCCTCTGG
185	cgd7_4880	CTTCTGATGATTTGGAGATTGAAG	TTGTGGAAGTTTCTGTTGGTGT
185	cgd7_4900	GATACGGTGGATGGAGTTATTAGG	CGCCTTTCTCTTTGTCTTCATT
185	cgd7_4910	CATGTTGCCATCCAATACAACT	CTGTCCAAGAAAGGAAGAATGG
185	cgd7_4920	TCAGGAGAAAGAAATGGAGACC	GCGACATTATTACGAGCATCAA
185	cgd7_4930	TATGGAAGCATCGGAATATCAA	TTCTGCTGTATGACCTCCTGAA
186	cgd7_4940	TGCAATTCACTGGAGACTTTGT	CAAACATCATTCTCACCTTCCA
186	cgd7_4950	GTTTCTGGGACTAGGTTTGGTG	TGTCCGTTCTTCCGTTCATAG
186	cgd7_4960	ATTCAGTTCAATCAGGCACAAA	CCTTCACAAGTCTCCTCAATCA
186	cgd7_4970	TTGATTGTGACGGATGGATAAG	TTCCTGGCATAAGTAAATCGTTC
186	cgd7_4980	CTGGTGGAATGGGTTTCTTTAC	GCTCCTCCTGGTTTAATTCTCA
186	cgd7_5000	GGAGGAACTTGTGCTATTCCAG	GACTCCTTCTTTCCATCAACCA
186	cgd7_5010	GAGGAGGAGGAGGAAGAAGAAC	TATATTTGGAAGGAGGCTTTGG
186	cgd7_5020	TGCAAATCTTGGTAACTTGCTC	CATGCCTCAGTATCAGTAAACCC
186	cgd7_5030	TCTTCCTCCATTTATTCCTCCA	TTGGCCTATCTGGTTTCATCTT
186	cgd7_5040	GCTCAAGTTCAAGCTCTTACCC	AACTCAGACCCTTTCCATCTTG
186	cgd7_5050	AAAGAAGGGAACGAGGAAGAAA	TTCGTGGTAGAAATGGGATTGT
186	cgd7_5060	GCTGTAATGAAGCCAACAGACA	AAATCAGCACATACACGCTCAA
187	cgd7_5080	TTCCTCAAATTCCAATCAAGAGA	GGGAGAAGAGGTCCAGGTAGTT
187	cgd7_5090	TCATAGCAAAGGAGGAAATACAGA	AGAGCCTTCCGATCTCAAATAA
187	cgd7_5100	GCCAACCTTGCATAGATACAGAA	TGGATGGAATAGAACTGGAGTAGAA
187	cgd7_5110	ACCAAGAACTTAACAACGGGAA	CCATTCAACTCATAAATCTGGCT
187	cgd7_5120	TTGGATGTTGTTGGAGATATGAA	CGTAAGTAATGGAGATTGGTGAGA
187	cgd7_5130	TGATGAAATAATGAGAAAGCCAAA	TCAAAGTTGGTATCAGGGCTAAA
187	cgd7_5140	ACTCGCTGGAAGTAATGTTGGT	CTCCTCACCCTTTGGTGTAGTC
187	cgd7_5150	CCTAAATGCTTACCTGGCTTTG	ATCCCTCTCTACATCGTGGCT
187	cgd7_5170	AAGCAGGAGAAACTGCATACATC	GCCTCTATACCCAGCATCAATC
187	cgd7_5180	TTACAACATCCTTCCAGCTTCC	ACAAGACGCTCCTCTAAATCCA
187	cgd7_5190	GCTATCACGTCCCGTACTGTC	CAGGATTGCTTCCCTAATATAACAA
187	cgd7_5200	GAGCTTCACTCTGGTGGTCTTT	TTTCAGGGTTTACTCGGGTTT
188	cgd7_5210	ATTCTATCTCCAACTGCTCCCA	CAGGAATACCAAGAAATGAGCC
188	cgd7_5220	TTAAGGCTCTCAAACTTAGCGG	AAACCAACCAATCCATCAACTC
188	cgd7_5230	TGTAAAGAATGGATCGAAACCC	TCTTCTCCAAAGCAGCCAGT
188	cgd7_5240	TCCTGTCTGATAATGAAGGCAA	GGACCATAAAGTGAATTGGGAG
188	cgd7_5250	TTGAATTAGGTTGTGGTTGTGG	GACTGCCAAGAAGTGAGGAGTT
188	cgd7_5270	ATTGAAGTTGTTGGAGGCGA	ACACGACCCATTGTTTCATCTT
188	cgd7_5280	TTGAGCAAGACCTACCAAGTGA	CTCCTCATCTCCATCGGAAC
188	cgd7_5300	TTCTGAGCAAAGCAATACACAAC	ACAATAAACTCAAGTAACCAGGCTC
188	cgd7_5310	TCCTTTAGCACAAACAACAACAA	CAATTTCCTCCTGATCGTCTTC
188	cgd7_5330	AAGAACAGGAGGAGGAGGAGTT	TCCACCAGCCTTATTATTTGCT
188	cgd7_5340	ACCTCACCTGCATCCTTTACTG	TGCTACCTCTGATTTCTTGGCT
188	cgd7_5350	TATGTTGGGAATTTGGTAGGGT	CCAATACAATCGCCACGTAA
189	cgd7_5360	CGATGAATGCTATCAACAAGAAA	GAGCGTCTATAATGCGAATCTG
189	cgd7_5370	TAGTTCGAGCAGAAACACCTCA	CCCTAATCCATGTAACTCCACAG
189	cgd7_5380	CGACCATGAACCATTAGAGGA	AGATGCTTACACATTGATTGGCT
189	cgd7_5390	TTTCCTCCTAAAGCACTCGAAA	TCTGACACCACATCATTCTCATC
189	cgd7_5410	GGTGATGATTCTGGCCTATCTC	CCTTGCCTTCCTTGTCTTATTG
189	cgd7_5420	TTTGCTTTCAGTGGACCTATCA	TTAGCCTTGGAAACATCCTCA
189	cgd7_5440	GCTCATCGGTTTGCTTATCTTC	TTGATATTGGACCTCTGGCTTT
189	cgd7_5450	GAATGCTCCTTCGCTCACTTT	TTGTACTTTCCTCAATCGCCTC
189	cgd7_5460	GTTTGAAGCGGAACATAGAACC	TTGCCATTTGGAGTTGTTACTG
189	cgd7_5470	CTCCATGCTCTGCTCCTTACTC	GTTTGCCACAGTAGTTTCCACA
189	cgd7_5480	TTCCAAGCAACTAGAAGCAGAAC	ATCTTGACTTTCGCCAGTGATT
189	cgd7_5490	GCATGGCTTTGATCCTAATTTC	CTGTTTCAGAGTCCGCTTTCTT

190	cgd7_5500	CAGAGTTACATGGCAATCCTCA	TTTACATCTACCATTTCCGTTGG
190	cgd7_5510	AACCAAGATGACGCAGAGAAGT	ATGCTTGAATCCGAAAGAAATG
190	cgd7_5520	TATGGCGGTCTAACTAAGGGAA	CCATTTATCAAGCCAAGCTCA
190	cgd8_10	CGGAACCTATGCAGTATTTAGGG	CACACATTTGCCATTCTCACTC
190	cgd8_20	GCAGGATGAAAGAAGGACAAAG	GCTTCCCTAAATAATCCCTCCA
190	cgd8_30	GATCGAGGAGAGTATCAAGGTCA	AATCGGTGATGTATCTGTTGGA
190	cgd8_40	AAGGATGGCTAATGCTGTGTTT	ACAAACGTGTCTCTTTCCCACT
190	cgd8_50	CGAATGAACAAGAAGTAGTTGCC	TTCGCTAGTTGAAGACCCAGA
190	cgd8_60	TCAAAGTACAGCTTCGGGATTT	AATTCTTTACAAGCCGGTCTCA
190	cgd8_70	TAGTTGCGAAATTGATTGCTTG	CTTCTTGAAAGGATCTTGCGTT
190	cgd8_80	GATTAGAGGCAATGGATGTCGT	TGGAAGTGTTTCTGGTTCATCTT
190	cgd8_90	CTTCATCTACTGGGAATCAGGG	ACCTCCTTCGTCATTATCCAAA
191	cgd8_100	TAGAGAGGCTTTGGGATTGTGT	TGATTATTGGATGCAGGAGATG
191	cgd8_110	CCAATATCAAGAAGGTTTGCGT	TTTAGGAAATGGTGGGACAAAG
191	cgd8_150	AGAGGAGGTGGAACAATGAGAA	GATCCCTTGGAATGACCAATAA
191	cgd8_160	CCTTAGCCTTGATGGTGAGAAA	CGCTGTACTCTAAAGGATGGGA
191	cgd8_170	TCCAAACAAGAGATGCCAGTAA	TGACCAGTTTCCAGTGCTAAGA
191	cgd8_180	TCAAGCCAGAGAAATACAACCA	AGACATCTAATCAGCCCAGGAA
191	cgd8_190	AATGGAGAGCAGGTCCAAACT	CATTTCAAGTTCATCGCTTTCA
191	cgd8_200	AGAAACGAGGTTGAGAGGAATG	AACCATATCCAACAAATGGGTC
191	cgd8_210	TTCAAGCTGTTATTGATGCTGG	CGTTTCCATATTGGTGTGCTAA
191	cgd8_220	AGCTTGGGCACGTTTGTATT	TTGAATGGGTCAGAGTTAGAAGG
191	cgd8_230	GTTGGAAACAATGAAGGAGGAA	GTCACATTTGGATCTGCATCAC
191	cgd8_240	TTCCACTTTGGGAGGAGTAATG	CCAACCCTACACACAATGCTAA
192	cgd8_260	CAGTAAACCCTAACGCCATACC	GGCAAAGAGAATATCCATCCAG
192	cgd8_270	ATTGCCTAGATGACCAAGAACC	CCCAGAAGGTGTATTCTCCAA
192	cgd8_280	CGAAATTGAAGAGAAGGAGGAA	CTTAGAACTGTCGGAATTGGCT
192	cgd8_290	CTTGGAGATGTTGTTGATAGAGGA	ATGGTTTCCTAAGAGTCCCACA
192	cgd8_340	TACTGCTAATGGATCTTCCCGT	CCCTGTTTGTGGTTCATTCTTT
192	cgd8_350	GAGAACGCAATAAAGAGCAGAAA	CAAATCCTAAACCAAACCCTGA
192	cgd8_360	TTACAGAAGGAATGCGGTGTC	TGCAAACAATCTTACCATCCAA
192	cgd8_380	GAATTGGGATATTGCTCTGACTG	GCCATAAACTGATGTAGACCCTG
192	cgd8_410	TTGTGCTATTACTTGGATGAGGG	CTTGTTGGAACTGCTCTGTTGA
192	cgd8_420	CAAAGGATCAGAAGGAACCTCA	TTCCGTTAATTTCCCTGTTTCA
192	cgd8_430	AAACAACTGAGAGCAAGAAGCC	TGCATAAAGCTGGTAGGAAACA
192	cgd8_450	TTGATCCACCCTGATAAGACAA	ACTAATTCCTCAGCACGTTTCC
193	cgd8_470	TTGGCATTGAGCACTTACTTAGAC	GTCTGGTTGATTATTCTCTTCATCAG
193	cgd8_480	AAGATTTGGAGGGATGAACTTG	AAATACATTGATCTGGCCCTGT
193	cgd8_490	GAGGAAGCTGGAAAGAACAGAA	TCAATTTCGTGGTAATCGCTC
193	cgd8_510	TGAAGATTCCACTTCTTGGAGG	ATTCCATTGCTTGGAGCTGTT
193	cgd8_590	TGAGAAGGCAGCATATAAAGGTC	AGTTCCAAGAAGAAAGTCCATGTC
193	cgd8_600	TTTATGGGCATGGTTTGTCTC	CATTGTAAAGACCCAATTTCTCAA
193	cgd8_610	AAGGAGGTGAAGATCGAGATGA	TTGGACGCTTTAGATTTGCTTT
193	cgd8_620	TCAAGTCAATGGAAAGTTATGGAA	ATATTTGATCCGCCATCCTCT
193	cgd8_630	TCACCTAATGCTCAAACTTGTCTC	GTTGGGTTTCAATACTTTGTTTCA
193	cgd8_640	GGTGAAATGGAAGATGATGGTT	TCAATATGCCTCCCAAATTCTC
193	cgd8_660	GGAATACCAGGAGGTGTACCAG	TGTTGGTTTGTTTGGAACTGTC
193	cgd8_710	TGTTTGGATAAGAGATACCGCC	TAGCAATAGATTTCGCAGTCCC
194	cgd8_730	GAAGGAGATTATATTGATGCTTTGCT	CCCTTAAATTCCAGCTTTCCA
194	cgd8_740	ATAGGCTCCCAAATAAGGTTCA	TTGTTTCAAGGCAATCCTCA
194	cgd8_770	TGAGGCCAGAGATATGGAGATT	TTCGTCCACTTTAGGTTTCCAG
194	cgd8_780	AGGAGAATGAGATAAATCAAACGG	GCAAATGAGAACTGCATACCAC
194	cgd8_800	AAGAGTGCATCATCAATTTCCA	TTGTATCAGGAATAAGCCCAAGA
194	cgd8_810	AAGGAGAAGGAAGAGCTGGATT	GAGATTGGCTTGAAACTATGGG
194	cgd8_820	AATCACTTCCAACTCCTCCAAA	ACCTCCGTTCTTCTCCTTCTCT
194	cgd8_840	TACATTGGAGAAAGTGCGAGAG	ATCAGCAGAAGTACCTTGGGAG
194	cgd8_860	TTAACTTCGACCCACACAGAGA	AACCCTAAGACCAATTTCACCA
194	cgd8_880	CAGCTAGGAAACTCAACAGATGG	TTTCTGGATATGGATTGCTCCT
194	cgd8_900	TCCAGTGTTATGCCTCTAATTCC	TTAGCTCGTCCATACCTTCAATC

194	cgd8_910	GCCATTGAAATATCGTCCAGTT	ACATCCCAACATAAAGCCATTC
195	cgd8_920	CAATTAAAGGTTCCCATGCTCT	AAAGCTGATAATGTTGGAGGAAA
195	cgd8_930	GCACTTTGGTCATGGGATATTG	GTTACTGTGCATTCCTCGCTTC
195	cgd8_950	TTCCTTCTTCTAGTGGCAAACC	TTATTTCTGTCAACACATGGACCT
195	cgd8_1000	TTGTAAAGGAGTTTGTTTGTGATGTT	AGCCACCAAGGAGTAGTCTTCA
195	cgd8_1010	CTCAAATTATCGCCATTCAAACT	GGGAAGGAAGAAATGAGACAGA
195	cgd8_1020	GTTGATTTGGGATTTGACCATT	TTTGTAATCTTGCTCCACCTGA
195	cgd8_1030	AACTTTGGGAATTAAGGCAGGT	TGCGGTTTCAGGTCTCATAAA
195	cgd8_1040	AAGTTTATAGTAATTGGAGATGCTGGA	TCGATTAGAAGAAGAATAGAGGGAAA
195	cgd8_1050	ATATTTCGGGAGCATCGAATAG	TTTGCTTGTTCTCCATTCTTCA
195	cgd8_1060	TGAGATGGCAGAATCAGAAGAA	TCCAACAAACTCACAAGGAAGA
195	cgd8_1080	AATGTAAACCAGGCAATTACGG	TGTCCAGTATCGGGAAAGAGTT
195	cgd8_1120	GGTCATTTATTTGTGAGTTTGGG	CCTCTTTCCCTTTCATCATCAC
196	cgd8_1160	GCAGTTTCAACAGATCAATCCA	ATCTCCTCATTTGTAAGCCCAA
196	cgd8_1170	GGTATATGTTTCCAGAATAAGCCAA	CTCTATGATGAATTAAAGCAGGGAA
196	cgd8_1180	CTGCTTATAGACAACAATCCGCT	GCTGCAACTTTCTTCATACCATT
196	cgd8_1200	CAACGGAAGAACAAGGAGAGTT	TGTTGAAGCACTTGGTAATCTTG
196	cgd8_1220	CGACAACGACGAAGATGACTAC	CCTGCTCAAGTTCTAATTCGCT
196	cgd8_1230	AATCTCAAGTAGGTCTTGCTCCTG	ACTCGAATGTTGGGCTCTTTC
196	cgd8_1240	GTTTGAGTTAGGTGGCTCTTCC	GAGTTCCAATTTCCATTCCATC
196	cgd8_1260	TATCTGTCCTTGCGATAAAGCC	GTGGAAGAGATGTTGACGATGA
196	cgd8_1270	CCCACAATCTTACGAACCAGA	AACCAAAGGAGTTTGACTTGGA
196	cgd8_1290	GGAGGAGAGATTCAGGACTAAAGG	CCACTATAATTTGGAGGATTCTTGAC
196	cgd8_1330	ATCTGCATCTATACTAGGAGGACCA	CAGCAGGTGAGGATATTAAGGTTT
196	cgd8_1350	GGTGCAAGAAGAGAGGAATTTGA	GCTACATACTGAACTTTCCAAACGAC
197	cgd8_1360	GCTCCAAGAGACCAAGAGAGAG	GAGGTGAAGAAGGAGAACAAGG
197	cgd8_1370	CCAAGTCCTGAAAGAGGAGGT	TTCTGTGTTATTATTCTCGTGGCT
197	cgd8_1380	CTCAGCTTGACGATTCTAACGA	GATTCTTTCAATTTCCCATCCA
197	cgd8_1400	ACTGCTTACGTTGCTTATGGTG	CTGCTCAAGTTCTGGATTGTGT
197	cgd8_1430	TTTGCTAATATGGGAGATAGTGGA	CCATTCATGTTCAGGAGGTAGA
197	cgd8_1440	ATTGGAGGATCTGCTATTTATTCTTT	TTCTTGTTGTTGTAGCTGGTGTC
197	cgd8_1450	TGGACTGGAGCCATTAACTAGAA	ATTAAGATAACCACCTGCGGAA
197	cgd8_1500	TTTAATAGGTGGGATTGACGCT	CTCTGAACTCCTCAAAGGGAGA
197	cgd8_1510	TTCCCAACAAATGGTGAACTAA	GGTATTTCCTGTTTCAGAGGTTG
197	cgd8_1520	GCGAACATTTAGAGCTGAGTCC	AGCTGCATTTGGTAAAGATGGT
197	cgd8_1530	AGATGATGAACAGTACCGAGGG	ATGACCTTCTAACGAACCTCCA
197	cgd8_1540	TCCCTAACATTTATTACCACCACC	GGTCCGATAAGTTGAATCCTTG
198	cgd8_1560	CTATGGCAAATAATGGTAGCCC	TGGAGGTAGAACTGTGGCATT
198	cgd8_1600	ACTTCGACAGGGTTTCCTTCTT	CACACCATTACTTCCATTTCCC
198	cgd8_1610	TCTTGATTCTTTCCAGCTCACA	ACATCCGCTTCTTGAACACTTT
198	cgd8_1620	AGATGTTAAGAGCCTGAAACGG	CCTGTTCTACTGAAAGATGGCAC
198	cgd8_1630	GAATTATGGAAGTTGATGGAGGA	GAATTAGCAATAGAGAAATCAGGGA
198	cgd8_1650	TGCACTGATATTGGATCTCTTCA	TCCCTACCAGAAATACGACCA
198	cgd8_1680	TTGGAGAGATGATGCAGGTAGA	ATTGAAGTTGATTGAGCTGGGT
198	cgd8_1690	ACTGATGAAATGCCTGGAAGAA	CCTAATTCACGCTGGATCTTTG
198	cgd8_1700	CGAATACGACTTTGCGTTCA	CATTTGTCTGCCATCATCATTC
198	cgd8_1710	GACCGAGATTGAGGTAGCTTTG	ACGTTGATTAGACTTGGGCTTG
198	cgd8_1720	ACCCAAATTCTGATGAAGGAAA	ACATCCCAAAGTCAAAGAAGGA
198	cgd8_1740	AATTTGGAGCTGTTGACTTTCC	CCTTCATCTTGCTCTGTGTTTG
199	cgd8_1750	CCACAAACAACTGAATCCACAG	TACCTCCTCCATTACCTCCTCC
199	cgd8_1760	AATTCCATCCAATGACTTGCTT	TTTCCACTTTCAGCCACTTTCT
199	cgd8_1790	GCTTGGATGGTTTAATTGTGTTG	CGCATTTGATTTCTTCTTTGGT
199	cgd8_1830	AAAGAATACTGAATACGGAGGCAA	TTGCTAACCAGTCCCAACTTCT
199	cgd8_1840	GCCGCATATCTCCTCTACCTAA	GGTTTGGAGTCTTCTCTCAAGG
199	cgd8_1850	CACTTAGCTGGTACTGGTGCTG	GGCGACCTTAATTGCACTAACT
199	cgd8_1860	TGGTGACTTGTGTATTGGAAGG	CTGAGGCGGAGATTGAGTATTT
199	cgd8_1870	CAAGCGTAACTACATTGGGAGAA	TGCAAACAGTCCTTAACAACCA
199	cgd8_1880	AGGTCGGTAATGAAGAAGAAGAAA	TCAAACGAAACTGCATCAAATAA
199	cgd8_1900	ACAAGGAGCATTGAGAAACCC	ATTCGCATGTTTGCATAGTTCTT

199	cgd8_1910	GGATATGGTTAAGAATGCTGGG	AACCCTTTGTAAGATGGTGGAA
199	cgd8_1930	TTAGCACTTGGTGATTTGGTTG	CGCTTTAACGAGTCCAATGTC
200	cgd8_1940	CTTTGTAGTGAAGTATGGTGTCCAA	CTTATCTCCTCTTTGTTCATGCC
200	cgd8_1960	TTCCGTAGTTGAGGAGATTGTG	TCTTCGCCTGTATTTATTATTGGA
200	cgd8_1970	CTCTTCACTCCTATTTGCCCAC	CTCTTCTTCCATCCAAAGCAAC
200	cgd8_1980	TTTCAATAGACTGGTCCTTACTTCCT	TCTGCCTCTACTTTGAGAAGCC
200	cgd8_1990	TGAGGAATTACCAAACCACCA	ATCCACAAGTTTCCACCAAAGT
200	cgd8_2000	GATCCCATTGAGTCTTGGCTAC	AAGGTGCATAAAGATGGAGGTG
200	cgd8_2010	ATCCAGCTAAATGCAGTAGAGTCC	CCAGCCGTGAGTTATTCCAG
200	cgd8_2020	TTCCAACATCCTTACAAATCTTCA	CAACAAATACCCTATCGCTTTCA
200	cgd8_2030	CCTGACAAGAGCGTAGTGAATG	GGCAATCATCCTTAAATCCAAC
200	cgd8_2040	AGTCAAAGTCAGAACCAAAGCC	TTATCATTGGCGCATTAAACTCT
200	cgd8_2050	GTCGAAAGAGGAACCTGAAGAA	CTGACATGGTCGATAACTCCAA
200	cgd8_2060	ACAACATCCTTTACATCGCTCA	CAATCAGGAGTCTGCCAATAGA
201	cgd8_2070	GAGTAATCTTTCAAGGGAGGAGAA	TGAGAACGTGGAGGATCAACT
201	cgd8_2080	TATGTTCAAATGGCTCCTCAAA	TGATGTGGCTGATAAGGAAATG
201	cgd8_2090	ATGTTCGAGTTTAGTGGATGGG	TTGCTATTCAGACATTCAGCGT
201	cgd8_2100	AATTCTGAGCCGATTCCAGTAG	TTCATTCGCTTCTTCCTTATCC
201	cgd8_2110	GGTTCTGGTGAATCTAGTGGCT	TGGACATCTTGGACAATTTCTG
201	cgd8_2120	GGCTCAGATGATGAAGAGGAAG	CTTCTTGGAAATAATGGTTGCTG
201	cgd8_2130	TGGCATCTTGGCTTTCTTTC	TTCCTCGATTCATCATCATTTGT
201	cgd8_2140	GGATGCGTCTGTAATCTTATCCA	AATCACAAGAGCCTATAATCCCAG
201	cgd8_2150	GAAGCTGTTGCACTTGATGTTT	CTAAGATGACCGGAGCCACT
201	cgd8_2160	ACAAGGGATTTGGTACGGATAA	GGATAAAGCGTCAACTACAGCA
201	cgd8_2180	TGGTGAAATTGAAGCAAGAAGA	AACCATTCCGTTGGAGAATAAA
201	cgd8_2190	CAGCTCTTGTTGAGATTTCTGCT	AGATGACGTTGATACCTGCCTT
202	cgd8_2200	AAAGTACGAAGATGGTGGTTTGA	GGATGCTCCCTTAAATCTGTTG
202	cgd8_2210	GATTTAAGCCGTCCAGTGAGTAA	TTGTCCAAATAATCCTGTTCCA
202	cgd8_2220	TTGGATTCATTAGGAGAAATTGG	ATGTTGCTACCCAAGACACAGA
202	cgd8_2260	AGCAGATTTCCCAACCTTAACA	CCATGTATCATCTCCAGCGAC
202	cgd8_2300	TTGGATGGTCTAAATTCCGTCT	TTCCTCATCTTCCTCTTCTTCCT
202	cgd8_2320	CCATCTGGGTCTACTAATGTTGTG	TTTGGTCTTCCTCTCCTACCTG
202	cgd8_2330	CTATTGGAGGAGCTGATATGCC	TATGAGAAAGGATTGCACCAGA
202	cgd8_2360	GATCTATCTTTGGCGGAATCAA	TCTAACTATCATCCCACACCCAA
202	cgd8_2370	TTCAGGAGGAATTAGCGATGAC	GTTTCCAGATTCGCCTTGTTAG
202	cgd8_2380	AGGGTTTGAGAGTGGATCAGAA	CAACAGAGTATGAAGGATTTGGG
202	cgd8_2390	GAGTCTAGGGTTGGAATCACCTT	GTCTCCTTATTTGGATGTTCGG
203	cgd8_2410	GCTTGGATGTGGAACAGAGAG	CAGTCAGAAACTGCGAGAGAAA
203	cgd8_2420	AAACTAAGAAGTCATCCACCATTGA	GAGCTATTTCTGGGCTCGTCT
203	cgd8_2430	TGAAGGATTTACCAAGTATGCTGA	GTTCCTGCTCCATGTCTTCTTT
203	cgd8_2450	CAGAGCATCCATAATTCAACCA	GAATCAACAAGCAAAGGAGAGAA
203	cgd8_2460	AAGATTGGGTGTCTTCTCAACC	TACGGGTCTTCTTTCTTGCTTT
203	cgd8_2470	ATGACAAACGATAGAGCAAGCA	AGCTAGATCCCAACGTAACAGG
203	cgd8_2490	CCTCGGTTACTGTTTCCACAA	AGTTCTTGTTTCTCCATCCCAA
203	cgd8_2500	CATGCTATCTCAAACCAGGAGG	CCATCCCATTTATCAGTCCGT
203	cgd8_2510	GGCTCAAGCAATAATCCAATCA	TACCAAGTAATGTTGCCATCCC
203	cgd8_2520	TCAAATGAGTGGTAGAGCAGGA	AAAGGTTGTCCAGTAAGCATTGA
203	cgd8_2540	CATGGTTGTTCTCTGTCCAGTC	TCTTCTCTTCTCCAAGTATCAGCA
203	cgd8_2550	AGGTACACCATCAGCAACCTCT	TGCGTGTTGTTCTTCTCACTCT
204	cgd8_2560	CAGCAAGCTATCATCATTCAGG	AATACATGACCGCAAGGAATCT
204	cgd8_2580	CCTAAAGGTGGCTTCTTGATGT	GTGCTGCTGCTACAAACTCACT
204	cgd8_2620	GGCCAATGAAATCTGCACTAA	ATGAACACTTTCTCGCTCACCT
204	cgd8_2630	TCAGAAGTGTGGTGGGAAACT	GGGAATTGAAGAATCAAATAGTCC
204	cgd8_2640	TCAGAGAAATATAAGCTGGGTGAA	AACTCTAATCGTGCCATCAACA
204	cgd8_2650	TTGGGCAGGACTAACTCATCTT	CTCACACAGCAAGCAATACACA
204	cgd8_2660	GTACAAAGTGATTGATGGGCAA	AAGTGTGTTCAAAGTTGTTGGTGT
204	cgd8_2670	GGCTGTTCAATGGCTAATGTTT	CCGTTTGATGTCGTAATAGCTTC
204	cgd8_2710	TATTATTGGACGGAGAGGTTGG	TTATTTCTGGTCCGCTTCTTTC
204	cgd8_2720	TGTCCATACACAAATGCTTTCAC	TGATCTAGCCAAACTTCCAACA

204	cgd8_2750	TTACTCCAGATGGCTTTGATTG	TGAGCTGAGGTTATCAGGTTTG
204	cgd8_2760	TTTCGGAGTACAACCTGATGATT	GCCCACCTAGAAATATCGCA
205	cgd8_2780	CAACAATTTAGACCACTCTGCACT	GAGGCGCTTATATTTGGATTTG
205	cgd8_2790	CCATGACTTGATGATTGAAGGA	ATTGCTGCCTTACACTTGGATT
205	cgd8_2800	AAGACTGCAACTATGACGGAGG	ACAATACCCATCACCTACCCAA
205	cgd8_2810	CTATTCTTGGGAATGGATTTGG	TGTGTATTGCATTGTGAGCAAC
205	cgd8_2820	GTCGGATGGCTTACTTCAACA	ACGCACTACTCGATATACCCAAA
205	cgd8_2830	GAGCATTGTTACTTGTGCCTGT	TTCTTTGAGCCTATTCCCATTT
205	cgd8_2840	TAAATGGTGGAGGTGGAGACTT	GATTGATCTTCGGTTTCTGCTC
205	cgd8_2850	ACTTCATCAAACCAGTAACAAGCA	GCAAACCACTCGACATTAACAA
205	cgd8_2860	AGAGTGACAACTTTGGCTACGA	CCCATAATACCTGCTGAAGTGA
205	cgd8_2870	ATTGAAGAAGGTGTTGTGCCTT	ACTCTTGATGTTGTGCCAATTCT
205	cgd8_2890	GGAAATTAAGACAAGAGAAGTCATCAA	TTTCGTACATTGACCATACTCACAA
205	cgd8_2910	GGGATGGTATTATGGTTGATGG	CCTGTGCCAATTAAGGAAACTC
206	cgd8_2940	CCTCTCAAGGGAACTATTTGGA	TGCCTCGTCCATGACTTTATT
206	cgd8_2950	ATCACAACTCCTGAGAAAGGGA	TTTCCTCTGATGCTATTGGGTT
206	cgd8_2960	GTTCAATGGAGCGAACATATCA	TTCTAAGCGATGGACAGTTTCA
206	cgd8_2970	AGCTTTATTTCCCAGCCTATTACA	GAAGGGCTCACTCTCTGGATAA
206	cgd8_2980	TTGGGTGGTAGATACAATGCTG	GACCCTGGAAGACTAATTGCTG
206	cgd8_2990	TGGAGCAATCGTACTTAATTCATC	AAAGCCCTTCAACTACCCATAA
206	cgd8_3000	TTGAAGCACTGATGGAATATGG	AGATTAACAAGCAGGACAACACAA
206	cgd8_3020	ACAAACAATGGGAAACACGAC	CCATAAATGCACGTAAATCCTCT
206	cgd8_3030	TCAGATGTAAACTATGCGAAAGAAA	TGATGTTCTGCTGGGTGAAG
206	cgd8_3040	TACAAGTATTTCCGGGTTGTCC	AGAAGTATCCATTCGCACCATT
206	cgd8_3050	TTCTACTCAACAAGTCGCTCCA	ATTTACGCCAGTTGGATTCTGT
206	cgd8_3060	TGTCTCAAGCGTCTACAGAAATAAA	GTGGACTCACGAATTTGAATGA
207	cgd8_3070	GTAGCAAGCCGTCCACTTACA	TATACAACCAACTGCCCAAACA
207	cgd8_3080	GGGTACTCCATGAAAGCTCAAC	TCATTCTCTGTGGCAATTTCTCT
207	cgd8_3090	AGATATTGGGTCTCATTGCGTT	ATAAACCATACTCGCACCTTCC
207	cgd8_3100	GTATTTCTAGGCAACTTTGGTGG	TGAATTGCTTCTTTAATCGCTG
207	cgd8_3110	CTGAGAAATCTGGAAAGTGGCT	CGCTCAAGAGTCAACCAAATAA
207	cgd8_3120	ACTTGCGCTAAATGATAATGCC	ATTGCTTCTTGATTTGCCTCC
207	cgd8_3130	CTGCACCAACACTTTCACCTAC	CTTGAATCTCACCATTCCTCCT
207	cgd8_3140	CTGTAATTTGGAGAGGCCCA	TAGAGAGATGCTCGTCGGAAGT
207	cgd8_3150	TTTACCAACAATCTCGAAACAGG	CGTAATTGCAGAGCCAACAAC
207	cgd8_3160	TTCGTGGAAGAAAGTTAAATGGA	AGAAATTGAGGAATATCATCCGAC
207	cgd8_3170	ATCTGGGCAGGCTAAAGTTGT	TTGCTATCGCTGTGTACTCTCCT
207	cgd8_3180	AGTAAGGGTAGCAGCCATTCAG	TTAATTCCATTTACCACTGCGA
208	cgd8_3190	GGAAAGTGTCAGGTAATGGGAA	TACTGTGTGTTTGATGGTGGGT
208	cgd8_3200	AAATGAATATCTCCCAGAGCGT	TTTAGCGATGCTTTCTTCTTCA
208	cgd8_3210	CCAAACGATGAACAACCTACTG	ACTTTCCATTCAAGTCCCTTTG
208	cgd8_3220	TCACGCTGGTGGCTGTATTT	CTTTCCGGCTGCAATTCTTT
208	cgd8_3230	AGGATACCAGTGCCGAATCTAA	CCATGAAACACCTTTACACCCT
208	cgd8_3240	TTACTGAAATCGCCTCCATTC	TTGAGTGCTCTTGTTCGCTACT
208	cgd8_3250	AATGGTGTCTGAGGTGTTCAAGT	GAGTTTATCGCTGCTCCAAGA
208	cgd8_3270	TATGCCAAGTTTCAAGCAAAGA	CCAAATTAGATTCATAGGTGCGT
208	cgd8_3280	GCATAAGTCGAGCAGTACCCTT	GAAGATACATTTGCCCTGGAAC
208	cgd8_3290	GTGTTGCTGAACCGATGTCTT	TTCAAGTTCCATACTGCTTCCA
208	cgd8_3300	CAAGACAGACACAACAGGATGG	ACAGAGTGTGCATTTCCAACAA
208	cgd8_3310	CAATGTTGCAGTTGTTCGCT	ATCCCATTCTACTCCACTTCTCC
209	cgd8_3320	GAGGTTTGCTTCAACTTTGGTC	TCAGGTCTATTTGCAGGTATTTCA
209	cgd8_3330	TGACAGTGAGGGAGACAAGAGA	CATCAAAGCAATACGCATCAA
209	cgd8_3360	GTTTGAATGCCAAATATGCTGA	CCGCGATATAATCTTGTGTTTG
209	cgd8_3370	TTAGATGATAACGCTTCGTGGA	TACAACTCCAACTTTCTGCCCT
209	cgd8_3380	TGTAGGGTCGATTACAAGAGCA	TGAGTAGAGGGAGTAACCAGGAA
209	cgd8_3390	GCCTAGAAGTTGCAGTTCGG	TTGCAGGAAACATCCCATTT
209	cgd8_3410	AACTTTGGGTAGAGTTGCCAGA	TGACAGGATACTTCTATAACTTGGCTT
209	cgd8_3420	TTGCGCGTATATTAAGTTTGGA	GTCATAAGGCGATGTGGAAAG
209	cgd8_3430	CCCAACAATCTTCTCCTCTTTG	TCCCTCATCAAGTTTCAAGACC

209	cgd8_3440	GAATCTCTTCGCCATTTGTCTC	CCCAAACAATACTCCAGCTCA
209	cgd8_3450	TGCTGTAAGACCCGATACTCCT	ATACATTCCCTTGATGGCTGTT
209	cgd8_3460	AGAAGGGAGATTTGGGAATGA	ATTGAGATAAAGAAGGCGCAAC
210	cgd8_3470	ACAATTAACAAGCTCGAATCCCT	ACATGGTTACAAGGAAGGCAAC
210	cgd8_3480	TAAAGACTCCAGGCGGTAGAAA	CTAACACACGTTGGACATTTGG
210	cgd8_3500	CAGATCCAAACACGAGAACAGA	TGCGAAGTTTCTTGGGAGTT
210	cgd8_3510	TGAATTTGACCGGAGTTTCATT	ACTGTAAGAAGTGGCGTCGG
210	cgd8_3520	CCTGATACAGAGGCAAAGAAGG	CAATAACAAATGCACGGGTAGA
210	cgd8_3530	AACTTTCCAACGCACATTCC	GCTGGTAGGGCTCAAATACAAG
210	cgd8_3560	ATGTTCCGCGAGAGAGTAAGAG	GCCTTGGAAAGTGTGTTTGAG
210	cgd8_3580	TATCATTGGCCGTTTGTAGTTG	TTACTTTGTTGAAAGCCTCCGT
210	cgd8_3590	TTCCAATTAGGCTTCATGTTCC	GAATAACCTTTCGCTTGCCTC
210	cgd8_3600	TGCCATATTGGAAGAGGCTAA	CCTTCATTGGATTCTACACCCT
210	cgd8_3610	TGCATGAGCTTCACTATCCAG	TCTCCTGATGGGATTTCGTT
210	cgd8_3620	GCGGCTCTGAATCTACGAAA	AATGATCCGAATATAGTAACCAGCA
211	cgd8_3640	GGCAAACACATTTCTCTTGTTG	CGTTGTGAATCGACTTTGTAGG
211	cgd8_3650	ATATGGAGATGGATGGCTTGAC	CTGATGGTAATTCAGCTTTGAGAA
211	cgd8_3660	CTTTGAATCCTGGCCTAAATGA	TTCGTGAATTAGCGGTTTATCTG
211	cgd8_3670	TTAGCGAAGAGTTTGTTGCTCC	CCACCTTTGCATCTGTCTTTG
211	cgd8_3680	AAATCAGCAAGAGCAAATGGTT	ATTCCACAGGTGTTATTCCCAG
211	cgd8_3690	ATGGTGGATCTTGGTACTGGAA	AAACTACGAGCCCTTTGCCT
211	cgd8_3710	AGGTAAGGAAGACAAAGCACCA	ATCAAAGGATCGACAGGAACAG
211	cgd8_3720	CCATGCTGTGGTGAAACATACT	TTACGAATTGGATTATTGGGCT
211	cgd8_3730	CAAGTTCAGAGCAACCCACTC	TAAAGCCCAAGCAAATATCCC
211	cgd8_3740	AAAGTGGAGAAGATGCAGAAGG	AGCCGGAAGGAAGTAACATACA
211	cgd8_3750	CCCTCTGCAATTACCTATCACC	ACTAGGCTTCTGCTGCTGTTCT
211	cgd8_3760	GGGTAATGTAGTTTGGGATGGA	CACACTGAATGGATTGTCGAGT
212	cgd8_3780	AAGAATCGTTTAGTGCCTCTGG	TGTCTGTATTTGCTGGTCTCGT
212	cgd8_3790	TCCATCTCCAAAGCATCAAAC	ATCTCATCCCATTCTCTCCAAA
212	cgd8_3800	CACGCCCTAATGCTAATTCTCT	TGCTGAGTAGTGACGAGGAAAC
212	cgd8_3820	ATCAGGTTCGATATAATTGCGG	GAGAGCCTATTACGTGGGTCAA
212	cgd8_3830	GGTTCTGCCACTTAAATCAACTTC	GCTAACACTGCAATACCAACCA
212	cgd8_3840	GCAGAGGCTATTCAGGACCA	ATATCGACGGAAATCCCAGAG
212	cgd8_3850	TACTGGTGTGCGTTTCCAATAA	TTCTCCCATGATACATTCCTCC
212	cgd8_3860	AATGAGCAGGTTGAGGAAACAG	AAGCAGGTTTGTAGTTTGCAGG
212	cgd8_3870	CTTTCTGGAGGAGCAAACAGAG	TTCACCAGGCTCATGGAATAA
212	cgd8_3880	TCTGGCTATGGGAATAGAGGAA	ACCACCTGGAACAAAGTGAGAC
212	cgd8_3890	TTGCTGAAGAGATGGTTGAAGA	ATTTAGAGGCATGTGATTTGGG
212	cgd8_3900	TATTCATGCAGCTCTTAGCCAG	CCTCTTCCAAATAAGTCGGTTG
213	cgd8_3910	TTGGATTGATTGGAGTTATTTGG	CAGTGTTTGCTTTGTACTTGGG
213	cgd8_3920	TTGCCAGTGCTCTAAACCTTG	CGACAGGAATCTCCAAATCTTC
213	cgd8_3930	CAAAGGACGGAACCTATTTGAC	GGAGGCCATTACCTACTTGAGA
213	cgd8_3950	TTAATTGAAGCCCAAGGAACAG	GGCCAGTAAGGAACAAATAGCA
213	cgd8_3960	AATATGGATTGGAGTGAAGATGG	CATTGCTTGTTACTATGAGCGAA
213	cgd8_3980	ACTCTGGAGGAAGGTTATGCC	CTGGACAGCAATTTCATCTCTTT
213	cgd8_4010	ATCAAAGAGAGATGCAAGAGCC	AAACCACCACTAGAAATGGATGA
213	cgd8_4020	CGATGGAGAAATTGAGGAAGAA	CCAGCAGGAGAGAATGACCTAC
213	cgd8_4030	TTGATGTGCCAATAGAATGGTG	TTGCTTCTGGAATTGATGGAGT
213	cgd8_4040	TCATCTTATCAACAATCAGAACGTAA	TTTAACAACAAGTGTGTGCCCT
213	cgd8_4050	TAAGAATGGGCTACAGCAAGGT	CTCTTTCACGGAAGCATTGTC
213	cgd8_4070	CTCGAAGAAGAAGAAGGGACAA	CTTCCATCACAATAATCACCCA
214	cgd8_4090	TTGTTGCTGGAATATGGTGGA	TACAATCGGAAGATGCCCAA
214	cgd8_4100	GCAGATCAAACACATCCTGAAA	CCTTCATCATCCATTGCCTC
214	cgd8_4130	AAGTGGAGGTTCAAACAGAGGA	AATTCCAAAGCTCATTAACCCA
214	cgd8_4140	TTAGAGTTGGCCCTGAATACCA	GGAAGAGGATGAGGAGGAAGAA
214	cgd8_4150	AGGAGGGAATAGAGATGGATCAG	ACCATTATTGCTACCGCTTCC
214	cgd8_4160	CCGCTCCTGTTAAGGCTATTT	TGTGTTGATGCTTTCAAGGTTT
214	cgd8_4200	ATCATGGTGGATTAGGAGGAAA	GCTGGATAAGATTGTGTTGCTG
214	cgd8_4210	TTGGACTTTGGACTAGATTCAGATAA	AACTTCCTCGAATGAGCACC

214	cgd8_4220	CTATCTCAAAGAACTGGCACCC	GGCGTATATGTTCACACCAAAGT
214	cgd8_4230	TGGAGCTAAATGCAAGAAACAA	GAACTCGAATAGTTACACCACGAA
214	cgd8_4240	GTCGTCAAGTTTCTTTGGCATT	CTAATCCTCCCATTCCCGTATT
214	cgd8_4260	GAAGCCAGTAATAGATCAAGGAACA	GCTCACCTTCAAATAAGAAACGA
215	cgd8_4280	CTATGTATGGTGGCAATGTGATG	ATTGGCTCTGGTACACGACTCT
215	cgd8_4290	AAGGTAAATCCCAGATAAGCCAG	AACAGACAGGATCTCTTGGTGAA
215	cgd8_4300	AATGACTGAAACTGAACTCACACAG	AGCAAGAAGTACCTTAGCACGTTT
215	cgd8_4320	ACTGAGCACTATGATGAGCCAA	TACCGAATCTAACAGGTGCAAA
215	cgd8_4340	TCAATTTCGTTTCTGGAGGATT	ATTCTGGCTTTGCTAATGGAAC
215	cgd8_4360	GCAGAGACGTGGTTATATTGGAGA	TGTCTGGATGGAAGTAAATCGG
215	cgd8_4380	AATTTAGGCAGGAAAGAGCAGG	GGGACTCAGAAGAAGACCCAA
215	cgd8_4390	GCTTCAGGTTTCTTGTTTCCAC	GACCAATAACTCCAACTCCAAATC
215	cgd8_4400	GGAGAGAGAGAAACACAAACAGG	ATGTTGTCATGGGTTCGTCA
215	cgd8_4410	AACGCTTTGGGTAAGATCAGAG	CAGGTTGAGATAATTGAGGGAAA
215	cgd8_4420	AGGAATGGGAGCAAATGAACT	GAATATAAAGTCAAATCCAAGGGAAA
215	cgd8_4430	AAGTGAATTGGGAACTGAAGGA	GGAGATTTACACATAGAACATGCAAC
216	cgd8_4450	ATTCATCCCATTAGCTCTTCCA	AGAGAATGTAAGTCAAAGCCAAAGA
216	cgd8_4460	ACGTCCATTTGAGTATCTCCGT	GCTTCCAGTTTCCATCACTCTC
216	cgd8_4470	AATGGGACAACAAGTTCAAGGT	TTGAGACGGATGGTCTTACAAA
216	cgd8_4480	AGAGGAGTTCCAATAGCTTGGTT	TACGAGGCGGTTCTTTCTTACT
216	cgd8_4490	TCCTGCAAATAATGGAATACCTAAA	TTGGAGATGAAGATCGTGAATG
216	cgd8_4500	GATTTGAGAAGAGTAGCCTTTGGA	TTTGCTATTTACCATTGCCATTT
216	cgd8_4540	GGCTCTATGTACTAAACTTGAAAGGAA	GTACCAAACATCATTCCACCCT
216	cgd8_4550	GAGGGTTAATGACAACTCAAGCA	CAAATTCAGCACATTCAGAGGA
216	cgd8_4570	GCAAAGAAACAGACAGACCACA	AGAAACCATCAACTCCAACAGG
216	cgd8_4620	CTGAAACATATCGCAAATCCAA	AGCGGCATCGTCTTCTAGTATC
216	cgd8_4630	TCTGTTCTCCTCCTATTGCTCC	CAGTAAGGTGATGGAACCAGTGT
216	cgd8_4640	CAACACTTGGAGGAATAGATGGA	TACCAGGAACATCGAAACGAC
217	cgd7_2680	ATTCAAATCCCAGTTCCGAGT	ATTGCGGTCATTGTAAATTCCT
217	cgd7_2690	CCGTAGTTAAGGAGCTTGATGG	TTTCGCTTTGTTTGTTTATTTCTTC
217	cgd7_2700	CAGAGGCGTATGTTGAATTGTC	CCTGGATATACCGCAACTTTCT
217	cgd7_2710	ATTTACGTGGCTGGATTGAAGT	AAGGAAGGCTAGATAAGGGTGG
217	cgd7_2720	CTACGGATGCTACGAATGAGAA	ATAGGGCTGATGAGAGAGTTGG
217	cgd8_4660	GGACTTGTTGTTCCTCCAAAGT	AGAACTCGCTACAATGCTTTCC
217	cgd8_4710	GCCTATTGTGCTCTTTGTGGA	TCCTCATTATCATTGGCCTCTT
217	cgd8_4730	ATTGGAACAACTAACTCATCCAGTATC	TTCTTGGTAGGAGATCAATGCC
217	cgd8_4740	ACCGAGTTATCACCTTCCAGAG	CAACATAAAGCCCTAGCCCA
217	cgd8_4760	CCAATAATAGATACCGTTGGAGAGA	CCATACAATGAAAGCATGAACAA
217	cgd8_4770	TCCTGGGATAGAAATTGGAGAA	AATGACTGCATGTGGAAGATTG
217	cgd8_4790	TGAAGCTACTGCAATTTATGGTG	TTGTTAGTCCAGAACAGAGCAAA
218	cgd7_2740	ATGAAAGCAGAATGGGAGTTAGA	ATGGTATCCAATCCAAACAACA
218	cgd7_2760	TGGTGGAAGCCCTTTAATTCT	AATAGGGTAGCCAAAGCAAACA
218	cgd7_2770	ATAAATCTAGTTGCTGGCGGAA	CAGGTTTGGAAATTGGACAGA
218	cgd7_2780	CTTGTGAATGGTAGTGCTTTGG	TCCATGTTTAATTCCGCTCTTT
218	cgd7_2790	TTCAACAAGGGACATTTCAACA	CTGCAACTTCGGCATCTTCT
218	cgd7_2820	GAAGTTGGTCAGAAAGGTGGTC	TTGTCGTGTCAATCTCTTCCC
218	cgd7_2830	CAGAGGAAGGAGAGGTGGTAGA	ACTCCAGTGAGAAGGCACAAA
218	cgd7_2840	GATATGCAAGATTCCGGGTAAA	GTTAGGGTGAAACAGTGGTGGT
218	cgd7_2850	CGATGTAAATGGAGGTTCCTGT	ATCGCATCCTAAATTCCCAAA
218	cgd7_2860	ACGAGTTAGAGAAGGATGACGG	GCAGCCTAAATTGGAGTTGATT
218	cgd7_2870	TTCGATGACACAAGAGGGTATG	AACTTTATCGCGTTTGGATCAG
218	cgd7_2900	GGATACCTCCAAATCACAGCA	AGCACAGAACGTAAGCAACTCA
219	cgd7_2910	TGGTTCTACAATTTCATGCGAC	TCCATTTGAATCGTTAGCATCTT
219	cgd7_2920	TCCTACTGGAGACATTCCTCGT	GCCTTCGATGATTCCTTATCTG
219	cgd7_2930	GGAGACTGCTAATACTGAATGCC	CCAAGTGAATATAGAGGAAGATTTGA
219	cgd7_2950	TTGTTGACGGTGTATTTGACCT	TCATAAGCTCTCCACGTTCATC
219	cgd7_2960	AGCAGGTAGTTGCGATTGAAGA	TACTAAAGGTGTTTCCCAGCCA
219	cgd7_2980	TCAGTCAATTCTCAGCCATGTT	TTGATGCGTTCCTCTTCTATCTC
219	cgd7_2990	AAGAATGAAAGATTTATGGGATCAAC	GGAGATGTTGTTTGTTAATGGAACT

219	cgd7_3010	GGATGTTCATGTTGTTGCTGTT	TATGTATGGCTTAATGGCGTTG
219	cgd7_3030	GAGCACAATAATACATCGCCAA	TCACCAAGAAGTTGAGAGCAAA
219	cgd7_3040	CGTATGTATTGGCACAGTTGCT	CGTTCCCTCTTCCTAAATCTCA
219	cgd7_3050	TTTAAGCATTTACCAAGCGTCA	CTGCCATTACACATCCAACACT
219	cgd7_3060	ATTCGTATCAAGACCGTTTACTCA	GCAAATAGGTAACAAAGCGACA
220	cgd7_3070	CTAAGGGAATGCGAAGTTGAAG	CTCCATGATATTGTGTGGTGAGA
220	cgd7_3080	GAGGAGTGCATCAAGGCTATTT	TGGTCTATTTACTGGGTTTGGG
220	cgd7_3090	TAGCAGGAGATACCGATTGCC	GGTTCGACCATTCATAATAACCAAG
220	cgd7_3100	AACCCATCGTGATCTTAGCATT	GGGCGGTGAGTATTAAGTGAGT
220	cgd7_3110	ATTGTTGATATTTCTGGCTGGG	TCGTTTCCAAGTTCTCCATTCT
220	cgd7_3120	TTGGAGATGGGAGCCTACAAG	CGTTCTATGGTGTATCCGTCGT
220	cgd7_3130	TTTCCGTTGATAGTGTGGATTATGT	TTACGCACTTCTTGTCCATTCA
220	cgd7_3160	TGCGCTGGGTAATAGAGTGTT	GCATTGAGCCATTATTTAGTTGATT
220	cgd7_3180	AAGTTTCGCAAGATGACCCTAA	TAATGCAAGCTGATGCTGTTTC
220	cgd7_3190	GTCAATGGCGCAAGATAGATAA	TGGGTCGAAATATCAGGAGAA
220	cgd7_3200	TCATCAGTGCTTTCCGTTTCT	TCACCTCCATTAAGGCTCTTTC
220	cgd7_3220	GTTGAGCATAGGGAAATTGGTG	GTGCTGCTTTATCTGCGATTCT
221	cgd7_3230	GAACTCCGAAATGTGATACAAGG	ACTCTAGTTGAAGCGCCAGAAG
221	cgd7_3250	AGTGGTGGCGAGTATTTGTCTT	AGCAATCTATCCTTCTCCGAACT
221	cgd7_3270	AGCTTTACAGAGAATGTGCCGT	AGATGAATCGTTGGAATTTAGCA
221	cgd7_3280	CCAAAGCTCCCACTTATGGTT	CCCGAATAGAACCCAATAATCTC
221	cgd7_3290	TTCTGGTGTATTGAACGCAACT	TCCATCATCTGTCTCTGAATGG
221	cgd7_3320	AGTTCCTTTGGAGGAGTTGGA	AGGATGAACAATTACGGACCAG
221	cgd7_3340	GGAGAGCAAATACGGAAACAAA	GCTAAAGTCAGAAATGCAAACGA
221	cgd7_3350	TTAGCTTTGAGAGTCGGAGGAG	ATGCTGAGGGATCAATAACGAC
221	cgd7_3360	GTGCGTATTGGCATCATCAG	TTGTAACGAAATAGGAGTGGACATAA
221	cgd7_3370	CAATATGAGCTTCGAGAGACAGAA	GAGAATGCAATGAAATACCCATC
221	cgd7_3380	GGATAATGGTAAAGGAAGCGTG	CCCTGCTGTATTTGATGCTATTT
221	cgd7_3390	ATCTCCCACTCAAAGCCAGTT	GATCCTGCAAAGTTATCGGAAG
222	cgd7_3400	CAATATAGAGAGCAAACCATTCACA	ATTGAGTCGGTGCCATTTACTT
222	cgd7_3420	TGTTGTGAAGCCTGACTATTTGA	TTTCATGGACTGTACTGGGAAAC
222	cgd7_3450	TTTCGAGGTCGCTATCACTTTC	GGCACTTCCATTTGCTGTATG
222	cgd7_3460	GGCTATGGGCAGATTGTGTT	AAGCGTCAAATAGATAGAGAATAGGG
222	cgd7_3470	ATTGGCTGGAAATTAACGGAA	TTAAAGCAAGCCTGTTGGAAGT
222	cgd7_3480	CCACCTAGTTTCACATGGGTTC	CAAATGTTCCCGCAAATAGTGT
222	cgd7_3490	TAAGGACAGGCGAGAACTTTG	GCTATCGGCATCTTATTTGCTT
222	cgd7_3510	TCAGATGGCAAACAGGCTACT	GTCAAAGGTTCCAATGTTCCTC
222	cgd7_3530	TTCGTCTACAATATGCGTTGCT	ATCAAATGTTCCACCTTCGTTT
222	cgd7_3540	ACAAGCCGTCAAAGAGAAGAAG	TCGACATTTAGCGCAATACAAC
222	cgd7_3560	GTCTGTGCTCATTGGCTGTC	GATCGCATGTAAGGGTGGTT
222	cgd7_3580	AAAGGGTATCCAAGAAAGGGAA	GAAAGCTCAATTTCGTACCACC
223	cgd7_3590	GATTTGTTCGATGATGTGAAACC	AGTGCTCGTCGTTTATATTGCC
223	cgd7_3600	AGTATTCTCGTTCCATCATCCC	ACTTCGTTGTAGAGATCGCACTT
223	cgd7_3620	TATGAATGGTACGCCTTTGCTT	CCATACCGAATTATCGCCTACA
223	cgd7_3630	GGACCTAGTCGAATCAAGAGGA	TGCGCTTATTGAAAGTTGTGTT
223	cgd7_3640	ATTCTTGTGCATTACAAACAGGG	AAACCGCAACTACATCACCATT
223	cgd7_3650	CAGACTCCGTACATTCCAATCA	GATGTCAGTTTGCGTAGAAATCC
223	cgd7_3660	ACCCTGACGTAGAAATTGAGGA	TCCTGTGGCGATAGAATAGTGA
223	cgd7_3670	TGTCCCTTCTGGAGCTACTTTC	ATTCGTCAGCAAATGAACCAC
223	cgd7_3680	CCCTCAGCTTTGGATTGTAGTT	GGATCGCTTCATCGTATTTCTC
223	cgd7_3710	TCAGTGTGAGTCTTCAAATAACGAG	TCTTCACGTTTGCATGTTGTATC
223	cgd7_3730	AACGATACTGCTAGGCGATTATTT	CGACCAAACCTTTCTTGTCTTT
223	cgd7_3750	ACTTAGATCACGGAAGGCAGG	TCCCATAGGCACAAATAACTTCA
224	cgd7_3760	AATTGGAACGCCTTATTATTTGTC	GATCGGTTTCTCAACATACCCT
224	cgd7_3770	TCACAACAAGCAAACATTTCTCA	GATTCGGCTACCCATTTCATC
224	cgd8_4800	AGA GCT TCC AAC ACA AGG AGA C	TTT CAC TAC ACG CTC CCA AAC
224	cgd8_4810	GAA TCT CCA GAC TAC TTT GAC TTT GA	TCG CCA TCA TCA TCT TCT TCT
224	cgd8_4830	TCA CCA ACA ACA TCA TCA TCA G	ATT CTT TCC TGG CGT ATA TGG A
224	cgd8_4870	TGG AAC AAC CAT CTA CTG CAT C	AAC TTA CTT TGA TTC TTG GCT CTC A

224	cgd8_4880	TCA GGA GTT GTT GGA AGT CGT	CCA TAC TGA CCT TGT TGA TGG A
224	cgd8_4890	GAA TGA GGA GGA CTC AGA CAG G	CAA TTC TGC AAT CAA GAC AAT CA
224	cgd8_4910	AAT TGG AGA AGG TCT GGT TTC A	GAT GAA CAA GCT CAC AAG CAA G
224	cgd8_4930	TTG ATT TAT TTC CTT GCC TCG T	AAT TTG TAA ACT TGA TGG TCT GGA A
224	cgd8_4940	CTG TAG TTG GGT CTG TAG TTG GG	AGT TCC TAT GGA CGA AGA CGA A
224	cgd8_4950	GGG ACA TTC ATT CAG ACC AAG T	ACA TAC CAG AAG GAG CAG TAG CA
225	cgd8_4980	TGC TAT TTG GGA GCT TAA CCA G	TTT GGG ATC AGA AGG ATC AGA G
225	cgd8_4990	TTT ATA TCC AAC CCA ATC AGG AAG	GAT CTG ACC AGT CCC AGG TAA A
225	cgd8_5000	ATT TCT GAT GGG AAT TGT CTG G	CGA CCT GTC TTG CTA TAC CTT CA
225	cgd8_5020	ATA TGA GGA AGC ATT GGA GGA G	TGT CAA CAT TCC AGT TAC CCA G
225	cgd8_5030	TTT GAT GTT GAG GTT GTA TGC C	CCT GAG CCA TAT TGG TTT CTC T
225	cgd8_5050	GCC ACT TAT ATC TTG TTT CGC TG	AAT TCG ATT GTT GTT TCA TTT CTT G
225	cgd8_5060	GTG CAA CCA GAA CAA CAA ACA C	CAA ATG CTC CAC TTC CCA TAC T
225	cgd8_5080	TTA TTG GGT ATG ACT CTC CAT CTT T	TTC GCT AAA CTC CTC TTT CTG TC
225	cgd8_5090	TCC AGG TTT ATT TCC AAG GTG T	CTA ATA CTG ATT GAT AAT GCC CAG AA
225	cgd8_5120	TAT CTG GAG CCT TGG AAT TTG T	TGT GTA AGT GTT CTG GCA AAG G
225	cgd8_5130	CTT GGA GTA ACG AAC AAC ATT CA	TCA GGG TAC ATT TCA GCT TCT TT
225	cgd8_5140	CAG ATA CTC ACT GGG ATG ACG A	CAA AGT GGT TTG ACG GTA TGA A
226	cgd8_5150	ATT TAT GCT CTC TTC TTC CAA ACC	ATG AAC AAA TTC TGG CTC TCC T
226	cgd8_5160	GCA ACT TCC CTA CAG GAA CAA G	AGC AAA CTC TGG CTT GGA ATA A
226	cgd8_5180	CAC TTT ACA ACA GAT GCC TCC C	TCC AGA TGT CAA TAG GCT GTC C
226	cgd8_5190	TAC GAG CAT TAA ACC AGG GAC T	TGG AGA TGG AAC TAA TTT GGA GA
226	cgd8_5200	TTG TTT GGC TCC TTA TTG GTG	AAG TTA CGC AGT GGC TAG AAG G
226	cgd8_5210	TTG GAA TTG AGA GAA AGG GAG A	TTG GGA TGA TTA TGT TGA CAG G
226	cgd8_5220	GGC TGC GCT ACT ATT TCC TTT	TTG GGC TCT CTT GTT TGA GTT T
226	cgd8_5230	TGG TCG TGG TAA AGG AGG TAA G	TCG TAG ATC AAG GCG GAA ATA C
226	cgd8_5240	AGC TTT CAC CTG TTA GCG ATT T	TCT TAT CCT TTA CGC ATC TTG GT
226	cgd8_5250	GCG CAT TGA AAG AAT AGT AGC A	TCA TTT CCA GAC CCT TGT TTA TC
226	cgd8_5260	GAA GAG AGC CAA TCC AAA CTC A	GCT TCT CAA CCA TTT CTG CTG T
226	cgd8_5270	TTG GAT GTT TAG TGT TGT GGT TG	AGG TAA ATC TGG AAT TGC TCT CC
227	cgd8_5280	ACT TGA GGA CAA GAG CGA AGA A	GGT CTA ACC TCG CAG TAA CCA A
227	cgd8_5290	TTC AGG ATC TGG AGG TTT AGG A	GCC CAC TGA GGA AGA GTA AGA A
227	cgd8_5300	ATG ATG GTG AGT ATT CCG GTT C	TTG AGA AGG GTA CTT TGG CAT T
227	cgd8_5310	ACC CAT TTA TCC AGA GGG AAG T	CCA GAC CAT CTT CTT CTT GTC C
227	cgd8_5320	GAA TAA ATG GCT TTC ACC ACC T	TAC TCC CAA GTT CCA AAC ATC A
227	cgd8_5330	GTT CAC CAA CTC ATC ACC TTC A	GTC AAA TGC CCA GTC TTT CCT
227	cgd8_5340	GAC GTA TTG CGC TCT TGA GTA A	AAC CTC AGC AGA TTG AGA AAC C
227	cgd8_5350	GAC GGG TTC CTA GAT GAG TTT G	TGC CAG CCA ATA TGT TTA TCA G
227	cgd8_5370	GGA CAT CGC CTT CGT AAT AGA C	TTT CAC AAT CAG CAT CAT TTC C
227	cgd8_5380	TGT CTT GGG TCT GGT GAT TAT G	GTG CAG TGG TGG ATT GAG TTT
227	cgd8_5390	AGA ACC GTG TCG TTA ATT CCA G	AAT TGC TCC GCT ACT CTT TCT TC
227	cgd8_5400	GAA CTC GAA TAC AAG CAG AGC A	GAA ATA GAG TAT CGC CGC ATT T
228	cgd8_5410	TGG AAC AAA GCT AAG ACA ACC C	TTC GCA AAC TTC TTC ATC ATC TT
228	cgd8_5420	TAA AGA TGG TGG AAA GGA TGG A	TCC AAT ATG AGG GAA GGA TTG A
228	cgd1_10	TAT TCC TGT TGT TGC ACT GGT T	ATA AAC GCA AAG ATT GAG GCA
228	cgd1_1040	TTG AAA GAT GAT GAG ATG GAA CA	TTG ATT CTC TAT TGA TGT TGG TTG A
228	cgd1_1070	AGG CTA TGG ATT TGT TTC ATT CA	CAT TTC GTC TTG TCA TTT CGT C
228	cgd1_120	GCT CAT CTA ATG TTG GGA TGT TG	AAT TTC TTT CTT CCG CCA CTT T
228	cgd1_1200	TTG AGT GTA TCG AGT GGT CTG G	GAG TTC TGG GAG CAT CTT GGT
228	cgd1_1320	CTC ACT AAG AAT GCA GGA AGA ATG	CTT GCC CAT ATT TGA GTA TCC C
228	cgd1_1540	CTG AGG ATG GCT CTC TCT ATC AA	AAG GAG GGT GAA TGA ACT AGC A
228	cgd1_1590	TAA GTG CTC TGT TGC GTT CTG T	TGT GTA TGC TCT TGA TTG TCT TCA
228	cgd1_160	CAA TTC AAG AAG AGC AAC GAA A	TAA TGT CGG AGG AAC CAT AAC A
228	cgd1_1600	CTG TAG CAC CAG TAC ACG CAT T	CCG GAA CTT TGG TCA TTA GGT
229	cgd1_1620	AAA GGC ACC GTT CAG CTA TTA C	AAA GAA CCA TTA GGC TCG CA
229	cgd1_1720	GAA CCT CCA CAC TAC GAT AGC C	GGT CGT TCA TAA CTT TCT TGC TG
229	cgd1_1730	CCT CTA GTC TTG CCG TTT GAA G	CCA GCT AAT GAA CCA TCC AAG T
229	cgd1_190	TTA TTG CCT TGC TTA CGG ATT T	TTT GTG AAA GTA TCT GGC CTC C

229	cgd1_1900	TGC AGA GGA TGT AGA ATA GGG AA	CAC TGG GCT CAT TAC GAT TAC A
229	cgd1_20	ACT GGT CTT GGT CAG CTC CTT	TCA TAT TCA CAG CTC TCG TAG CA
229	cgd1_2040	TAC ATT CCT CAG TTC CAA AGC C	TGC AGT ATT CCC TGT TTC TGT G
229	cgd1_2050	GGA TGG TAC AGC CAC TGC TT	TAA TCA CTT CGT TTG TAT CTG GTT TG
229	cgd1_2190	CAA GCA TAT TGA TAA CTC AGG CAA	TGT ATC CCA GTA ACA GTG GCA G
229	cgd1_2340	ATC TAT ACG AGG CAA GCC AAA C	GCC ATC ATC ATC TGT AAT GCT C
229	cgd1_290	GGA CGG AAG CAT TGA AAG AGT	ATC TTG TTG TTG GTA CAT CGC A
229	cgd1_2950	TTG AAG AAG CGA AAG CAA GAG	CCA CTT CCA TCC CAA TAT GAA
230	cgd1_30	TCA TTA GCA TCC GAG TGT CTT G	CAG GTA ATC CGC AAT AGT AGC C
230	cgd1_300	TCA GCT ACC AAG AGA TTG ATT CC	TCC AGA AAT TGC GAA ACT AAC A
230	cgd1_310	CGC AGC AGG ATA TGA TGT TAG A	ACA TTC CAT ACC GAT GTT ACC C
230	cgd1_3160	TTT GTT TGG AAC TCT CAT CCA G	AAG CTC ATA AAC CTC AGC CAG T
230	cgd1_320	GTC CAT TCT TCC ACA AGG AAC T	ACA TCT CCT AAC CAT TCC AAC C
230	cgd1_3280	GAC CTG CGA TAT TAA GCA TTT GT	GTC ATC ATT ATT GCC GTC CTC
230	cgd1_330	GAT CAC TTC AAC TCT GCA TTG G	TTC TCA GGA TGC TCA ATA GGG T
230	cgd1_3370	GAT GGC TAT AAA TGG ACC CAA A	TTC GTA AAC ACA CAT ATT CCT TTC A
230	cgd1_3380	ACG CAC ATT ACA AGT ATT CAA CAT AA	TGA AAG ATC AAG TAA AGC CAG AAA
230	cgd1_340	TCC TAC GAT GGC TTC TTC TCA	ACA GCA AGA CGA TGG ATA CAA A
230	cgd1_360	AGA GCC ACA AGA AGG AAA GAT G	TTT GGT AGG GAT TCT GGG ATA A
230	cgd1_3810	CAT TAG AGG AAA CTC CAT TCG G	AAT GTT GAG AGG GAA ATT GTG G
231	cgd1_3850	TTT CTC AAA TCA AAG CAG CAG A	ATA ATA GTT CCC AGC CAA CGA A
231	cgd1_3860	GTC CAC TTA GAT TGG CTG TTC C	GCT CAC CTC TAT CGG TTT CAT C
231	cgd1_40	TATTATGCGATGGACTCACTGG	GGAGTTGAAGGAAATCGAGAAA
231	cgd1_470	AGAAGTGGAGTGGCTGTGAAA	TAAACTGGAGCTTGTGGAATCA
231	cgd1_50	GAAATGATACACCCATCGACCT	TCCACGAGCTATAACAACCAATAA
231	cgd1_500	ACTGTTGCTCTGGTTGCAGTT	CTCAGAATGTGTTTCGGTTTGA
231	cgd1_520	TTATGGTTTGATTGGTTCTTTGG	CCGGCAGCTAAACTACTGAGAC
231	cgd1_550	TTCCCATATTTCACAACAAGGA	TCATTGCGTTTATTCTTTGACC
231	cgd1_60	ATGTTCCCTGATAAAGTTCCTCC	GGGTTTCAGATCACGATGAATAA
231	cgd1_600	ACTTATTCGACTGCGAGATTGG	TCAGGGCTTTAACTCCAAGAGA
231	cgd1_640	TCTTTAGCTCCACTTCCTCCTG	GGTTTCAACGCCAAATAGAGAC
231	cgd1_710	TGGAAAGAATGAGAATGGATGA	GGATTCACTAACAAGATCACAAACA
232	cgd1_780	TTCTTTCCATGTTTGGTACATTTC	AATCCACCTTTCTCTTGGAGTTT
232	cgd1_850	CATCAAAGGAGTTACCACCACA	TTAGAAAGAGCACCAACACCAA
232	cgd1_870	GCCAAAGACTGCCAATAACTTC	GAGCCATGAAACCTGGAATAAC
232	cgd1_880	CATCTATGCACGGAGATATGGA	TAAGTTTCTGGTGAGACAGGCA
232	cgd1_90	GAATAATGGTTTCATGCCCAGT	CAGTTCCGATTGGTAGGTGATT
232	cgd1_910	CTTCTTCGGCTTCATTATCCTC	CGGCAGTTAAATCCATAAATCC
232	cgd1_930	TGATGATGATGAGGATGAATTTG	TTCTTTGCTTCTTGGAGCTACTG
232	cgd2_10	GCAATCGCACATCATACACA	TTTCATAGAGAGTTCAAACCATTCTC
232	cgd2_100	CATCAGCACTCCTCAAGAACA	GGGATCTAATGCCTCATACTCAA
232	cgd2_1000	GACGGCTAACAATAATGGGAAA	GATCCCTCGACAATAGGTCTCA
232	cgd2_1010	CAGGTTTAGCTTTGGCTTCTGT	ACACTTTCGTGCTCGTGATAAA
232	cgd2_1030	ACAATCTGGAAAGGTTGATGGT	TGCCAAATACTACAGCAGGAAA
233	cgd2_110	GAAGAACTGCAAGAGCTGGAAA	CAAACATGGTCTGCTTGTGTGT
233	cgd2_1140	TCGATTGGAGTTTCTCTTGGTT	GATATTTCCTCGTCCTTGCTTG
233	cgd2_120	CGTAAAGCTCACAGAAACGGA	GCTGCTTCTTTCATAGCAACCT
233	cgd2_1210	CGGAAGATATGTGCATTGACC	TCCTTGAGAATTGCCTCTTGTT
233	cgd2_130	TATCCAAGCTCCAATCTGTTCC	TCACCTTCTTCTTCCTCCTCTTC
233	cgd2_1360	GCTATTACCAACTGAAATCGGG	GTAGAATCAAGGTGGGTGAAGG
233	cgd2_1390	ACCTCCTAATGGCGATGCTAC	CACTTTCTCTAAGGTGCTCCAGTC
233	cgd2_1640	TATAACCTCATGGACCGAAACC	TTAAGGAGGCCATTCCTATCTG
233	cgd2_170	CCAGAAAGCAGTTTGTTGTTGA	CAGTTTGCATCTTGGACCTTG
233	cgd2_1790	AAACACGAAGCAGGAATGAAA	TCAAGCAACATCGAGAGTGAGT
233	cgd2_1860	TTGGATCTCACTGGCTAAATCA	TGATACGCTATTCCCATTCTCA

233	cgd2_1890	AATTGGCCTCCCAGACTATTTC	GCACAACTTTCCTCGTTCATTT
234	cgd2_210	TGGCTATATGATGAACGACTTGA	AGAGCGTATAAATTGGCTTGGA
234	cgd2_2120	CCAAGTTTGAGGTCGCAAGT	TTCTTGTCTGCATCTGCCTG
234	cgd2_2190	CCTTTACCAAATCCAAGCGA	TGATGGTGCAGTCATAGTAGGG
234	cgd2_220	CAAGACCTGATTGTACCGTTGA	TTGATGCTGTTGTTGTTGTTGA
234	cgd2_230	TTTGGATTGGTTCTTGGAATCT	TCAAACGTGGCTTCTTTATTCTT
234	cgd2_2300	TTTCTATGAGAGGCGCTAAGGT	CGTTCCAATGTATCAAATCCAA
234	cgd2_2310	AGGTGCAACATGGCTTTATTCT	GCGACCAAATATCCCACTTCT
234	cgd2_240	TACGCTCTGGTTTATATGCAGG	GATCTTTGCTTGCCACTCATT
234	cgd2_2400	ATGGCACCTGTATTCTCATTGG	TTGACATAACTCGACCAGCCTT
234	cgd2_2420	TTCCCTCTAATTTCATGTTTGGA	TGAGCCCTTATTTGATGTGTTCT
234	cgd2_2440	CGTTGAGTTATTCCGAGCAAG	CGATGGTTACATATTGGGCAG
234	cgd2_2560	TGTTGAAGTTAGATTTGGGAAGG	AAGATTGGAGTTCTGTTGAATGG
235	cgd2_260	AAGCTCCCAGAAGATAGTTCCC	TCGTCCTCATAGTTATCGCTTTC
235	cgd2_270	GACTAGGAGCTGTTTGCGACTT	GGTATGCCTGAGCAGTTTCTTT
235	cgd2_2850	AATAAACAATGGGAACGAGGAGT	AATCCAAATCTGAGTAACGAGCA
235	cgd2_290	AAATAGGGTAGGCATTCAACCTAA	TGTTCGTATCAAGAGTTGGAGAAG
235	cgd2_2900	TACACGATACATTGCTTCCACA	CACTCTTGTCCAAAGATCAAAGAC
235	cgd2_2950	TCCGAAAGTGAATATCCCAAGT	TCCACGCCATCCAAGTATAAG
235	cgd2_30	GAAGATTTGCTTCTCCTCCAACT	TCTTGACATGCACCAGGATTAC
235	cgd2_300	CTGCACCAAACTCAACTCAGAC	TAGTCTCGCCACCATAAATCAA
235	cgd2_310	ACTTTCTGCATACCTTGCCTTC	TTCCTGCTGTCATACCTGCTAC
235	cgd2_3120	AAACAGACCGCAAGGATATACAA	CAGATTCCCAAATTCATCAACA
235	cgd2_3410	TGGCACTATCGAGAATATGGAA	CAACATCCCAAACAGTGAAAGA
235	cgd2_3520	CTCAATTCTCGGCATTAGATGTT	AAGCATTTCCTCAAGCTCCTC
236	cgd2_360	GGAGGTGATGATTGGGATAAGA	CCTTGAATCTGGAGAAGAACCA
236	cgd2_3740	GGCAAAGCCACTATGAAAGATAA	TGAAACTTAATGGGTCACTTGCT
236	cgd2_3940	TCTGAACTGGGATTTGTCAGG	CCAGGTTGTGTCCATCTTGTT
236	cgd2_40	TCTAAATGTGAAGAGCCCAGACA	GCCGCCTCAATTACCATAGTTT
236	cgd2_410	CAGGTTCTAATCAAGGGCAGTC	CGATGACGAATATGAGGATGAA
236	cgd2_4120	TTACTCGTGGTGATGGTACTGG	CAAATACAACATGCTTTCCGTC
236	cgd2_4140	TGCCAAATTAAGAGGCAAGG	TTGGTGTTTGAATTGTCTTTCTGT
236	cgd2_4170	TGAGAAAGCACGTAAAGAAGCA	AAAGGAACAAAGCCAACCCT
236	cgd2_420	ATACGGAAATAAAGGAAAGCCC	AGAGTTTGAGGAACCACCTTGA
236	cgd2_430	GTAAAGCACCTGTTCCAACTCC	GCCTCTCACTCCTCCTCTTCTT
236	cgd2_450	GAAACCCGACTCAACCTAAACA	CCTCTGATTGCTCATTACCTCC
236	cgd2_480	GGTAAGATTTCCTTTGGATCTATGACT	CTTGACAGAATCAGGATCATTTAGTTT
237	cgd2_490	GGAGGAGTATTTACACGACCAGTT	TCATTACATCCATCTTTCTTTCATTT
237	cgd2_50	TTGGGAAGAAATTGGTCAAGTAA	AAAGGGAAAGAACGCAAAGAA
237	cgd2_610	GAAAGTTGATGCAAAGAATGGAG	TCCTCCTGAGAGCTGAAGAAAC
237	cgd2_620	CTGTAACTAGCAGCAGAGAGAACCT	TGTATGCCAAAGTCATTACGAGA
237	cgd2_640	AGGTTGATGGTAGCAAGATGGT	TGGAATGATAAAGATGGAGATGG
237	cgd2_70	GGAATAGTGGGTAGATCAGGGA	GGAACTTGAGGAAGAACTCCAA
237	cgd2_720	TGACCAGCCACAAGTATCAATC	TGATGTATTTCCAGTTTGACGG
237	cgd2_730	TTGAAGTATGCAAATGGGAGG	TCTCTAAATCCTGGTGGAGGAA
237	cgd2_740	ATATGTCCGATGAAATGCAACA	AAATTACGACCAACAACGCAG
237	cgd2_750	TACAATGAAGAATGGCTCTTGG	TGCACAGATATTGCGTAGTTGA
237	cgd2_760	CCCAGACTACGATGAAGCAATAC	GAAAGCTCTGCTGCCCTACTAA
237	cgd2_770	ACACGCATTACAAGAGCACAAG	CCACTTAGGTCCAGAGGATGAG
238	cgd2_810	ATGGCAATGGCTTTATTTCTG	CCATCAACGTCAGCTTCTCTAA
238	cgd2_820	CAACAACGTAATGGCAAGAAGA	CTCCCTGAATTTGAAGAATTGG
238	cgd2_830	ATATGGATGGGCTTTACTTTGG	CTGGACGAACGAATACACTTTG
238	cgd2_840	AATTCTTAATGTCAGGCCAACC	CTCGTGGTCACTTCTACCTCCT
238	cgd2_900	GGGCTACATTTGTTGAGCTTTC	CCAGAAGATCGGTGGAGTTTAG
238	cgd2_910	TTTGGACTCTCGGCTGTTTATT	TGCATTTAGCACACTTGAGTAGGT

238	cgd2_980	CGCTTTGTGAGATGCTAGAGG	AGTTTGGTCCTGTCTATGGGAA
238	cgd2_990	AAGGTGCTGGCAAATATACAGG	AAATCACTTCATCCAGTTCGCT
238	cgd3_100	ATGTTTCCGAGTTTAGTCAAGCA	AAAGATCCCAACCAGGAGAAA
238	cgd3_1020	ACCAAGGAATCAACTAGGCAGA	AGTCATTCGGCTTTGGAAATAA
238	cgd3_110	AAATGTGGTAACTGAGCTGATGG	TCCAAACCTATCCCTTCCAAA
238	cgd3_1110	AGGACCCAAATAATCGTCTCAA	CTCATGCTTTCACCCATACTCA
239	cgd3_1410	GCGGTACGGGTTTCACTATC	ACCAAGTGGAGTCGTTAAAGGT
239	cgd3_150	GAACACAAACAAAGAGCAACCA	TCCGAGTTATTCACATGACCTCT
239	cgd3_190	ATGTTTACCAAATGGGATGGAA	ACTCTGTTGTTGTTGTGGTTGG
239	cgd3_2260	GAGCGTGGAGTTGTTCAAGAG	ACGTTAGCACCAATGAAGGAGT
239	cgd3_2420	TTACTCTCATTCATTTGCTGCTGT	TCACTAAAGGTAAATCTCCAAGGG
239	cgd3_270	AAGAAGAAGAGGAGGTGGAAGG	TTATTGAACTGGTTGGACGAAGT
239	cgd3_2700	TTTGGGAATTAGATTAGTTGAAAGGT	TTGTCTTGAGCCTATCTGCTTG
239	cgd3_280	GGTCAGTAAGCTCAGTTCGTCA	TTGGTCTTCTTGGATTGGTTCT
239	cgd3_2830	TCTTGAATTGGACGAAGGTTG	GAAATGTCTCGGAAGGCTAATG
239	cgd3_30	TTGATTTCTTGTATGAACTTGGAAA	CTTGAATAGTCTGCGCTGGTAA
239	cgd3_300	TTCCGCTCCTAATACAACATCC	TTCCTGCATAGCTCGTGACTT
239	cgd3_3220	GATGAAACAAACAAATCGGAAATAA	TCAGCACAATAGCATCTCGG
240	cgd3_330	TGTGGAGGAGCTGTTAAAGGA	AATGCACCACTGCGTCTATCT
240	cgd3_3430	ATCCTGAGAAAGAAGGACACCA	ATCATAAACCCACCAACATTCC
240	cgd3_3820	TTCTGTGTTGACTGATGCCTTT	CCAACTCCTGATTCTTTCCCT
240	cgd3_3850	GAGTGACGGCAAGATTGAGAC	TTCTATTCAAAGGCGAATTACCA
240	cgd3_40	TTAACGCCCATTTAGGGATAGA	AAAGTCTCAACGGAAGTACCCA
240	cgd3_400	CTATGAATTGTTCCCGTCCACT	ATTTCTGATTTGGATCTCACCTTC
240	cgd3_410	CGCAAGAGGAAATAAACGAGA	TCCCACATCAATATAGCCAGAA
240	cgd3_4100	AGGTGCAGTTCCATCATACTTG	ACATTTCATCTTCATTCAAAGCC
240	cgd3_4110	GTCGAGGTTGATGGAGGACTT	CTTGTTGGTGTGTGTCTTGCTT
240	cgd3_4170	CAATTTCATCATTGGCCTTATTT	TAAGATTCGACTTGGACGACTG
240	cgd3_4220	AAAGCATGTGGGAAATGTAGTTG	CAGATGATAATGAAGTAATCCACCC
240	cgd3_430	ATCCAGATGAGGAGAGGTTTCA	AGACCCTTGTACGTTGCAGATT
241	cgd3_4300	GCTAGAAACACTGCAACCGAC	CCTAATGCCGAGTACGACTAAGA
241	cgd3_550	CAGCAACTCTTACAGCAGGTCA	GACTCAAGCATTTCAGGAGCA
241	cgd3_650	AAGAAAGACAACAACATGATCCAA	AATCCCAACAAGAATAACAAAGAGA
241	cgd3_690	AACTCCGTCAAAGAACTCCAAA	GGAACTTGTGAAATCCTCTCCA
241	cgd3_70	AGGCAAAGAACACAAATCTGAAC	TTTGAAGAAATTAAGGGAGGAGAA
241	cgd4_100	CATGAGCGAATCAACAAATGA	GCTTTCGGGTCTTCAAAGTG
241	cgd4_1010	AGAGACAAGAGGAGGAACAGGA	AAAGCCAACAAGAAACAACGA
241	cgd4_1020	ATGATTGTATTTATGATTTGGGATTTG	GCATCAGCTCCTCTTTCATCTT
241	cgd4_1030	CAAACAAAGGTGGAATGAGTGA	TGGAAGTGGTTCTAAGGAATGG
241	cgd4_1080	AGATGAAGTAAGATCAGGAATGGTG	TTGAATGTGTTACCAACCAGAATAA
241	cgd4_1090	TTCAATATGAGGAAGCCAAGAAA	TTTAATCGGGTCATCCCAAA
241	cgd4_120	GCGTATTCATCTTCAGTTCCATC	CATCTGGTAAATCATCGCACA
242	cgd4_1230	GCCAAGATAACCAAGAAGAACACT	GGACGGGATGAGATTTGATTT
242	cgd4_140	CAGTGCTACAATGGAAACAGGA	CATCATCAATGTCAACTCCACC
242	cgd4_1590	GCTGGTCTCGGAAGTTTGATAG	TCACCATCTGCACGTAAATCTC
242	cgd4_1650	AAGGTTGATGTCGATTCTCTCC	CCACACTCTTTACTTCCTTACCATT
242	cgd4_1700	GAAGGCTCCATATTGCAGAAAC	TCCATTTGATACACACCAGCA
242	cgd4_1830	GTTTAGAGAATTTACGCAAGCTGA	CCATATACGACCACGACACAA
242	cgd4_190	ATTGAGAGACCAATTCAACGGA	TTTCTTATCATTCGGGAGACCA
242	cgd4_1940	AGGCCCAGTTCTTTGTATGGT	TCTGCCCTAATTGTACCTGGA
242	cgd4_2020	AATGCAGCGATAATGAAAGTGA	GCCTATTTGCCCTACCAACA
242	cgd4_2040	ATTATTTCCCAGCCTCAATCAA	GTGCTTAATGTCGATGCAGAAG
242	cgd4_2110	TGGTGATCCATTTACAGACGAG	AGAACTGATTGTGGTGAAAGCA
242	cgd4_2260	CCATGCAATGTTGTAGTTCCAG	TGTGAAGCCGTAACTTTCTTCC
243	cgd4_2270	GTTTGGGAAGTGTTTATCGGAG	ACAATATCACGGCAACAAAGG

243	cgd4_2300	GTGAAACCTATCTCCCGCATAA	ATTCGCATGGGCTATTAAGAAA
243	cgd4_2350	TGATACAGGTTTGGGAATAGGG	ATATTGGTACGTGATCGCTGG
243	cgd4_2370	GATGGAGTGTATAAAGGACGCAG	ATGTGACTCTTCCAGACAACGA
243	cgd4_240	GAGAGGATGCAACGAAATGATAG	CAAATGATCCAGTTCCCAAAGT
243	cgd4_2400	TTAAAGGCATCCGTGAATTTG	GAATCTTCTGACTCGCTACGCT
243	cgd4_2460	GACAATGCACGATAATAAACAAGG	TGGGTCTGTCTAAGTCCAAAGTC
243	cgd4_2530	CATAAACAATACGTGCTTAATTCCA	GCATAAATCTTTGAATCGCCTT
243	cgd4_2540	AGAAGTACGAGAAGCCGTTGAG	AACGTGGCTGTTGTGTGATTAG
243	cgd4_2680	TTAGAAATTGAAAGTAACCCGACAG	AGAATGAAACAATGCCAAATGTAA
243	cgd4_290	GAGTTATTATTGACGGCGAACC	ATGTAAGGGTATTAGTGCAGTTTCAAG
243	cgd4_2910	AATTTCTCAAGTTCTCGCCTCTT	ATCATCATCTACCAACGGCTCT
244	cgd4_2950	CAATCTTGGAAAGTCGTTGGA	AAATGATCTGGTCGCTCTTGTT
244	cgd4_2990	AATCCACACAATGGAAGATGAA	CATCGAATCTCGTTAGGATGTCT
244	cgd4_300	TTTGATTGGAGATCACAACCTTT	ATTTCATACATTAACCTTCCCTCATC
244	cgd4_3000	ACGCCTCTAACTCGAAGTACCA	CTGAAACGCCAATACGATTTCT
244	cgd4_3020	CAGATATTCCCTTACCGAACCA	GTCCATGTTTGGCTTTGACTTT
244	cgd4_3080	ATCCAACGATGAGTGTGAAGG	TCAGAAATACCTGCTTGTTCCA
244	cgd4_3130	AAGTGCTTTGGGCAACTGTATT	AACTTTGCGTAACTCCCTACCA
244	cgd4_320	CTTCTGCCCTAAATTCGTCAA	CTGATTGGGTCAACACGTACTC
244	cgd4_3260	AATGTACCTGAAGGGTAAGTTGAATAA	CAATCAAGTCTGCATGTGTAAGAA
244	cgd4_3270	CAAAGGCTAAGTTCAAGGATGG	TGTGCCAAGAACCCTAAGAAGT
244	cgd4_3340	TCACATCCAGCTCCAAGTACAG	ATTCGCATTCATAATTCCATCC
244	cgd4_350	GGAAAGTTCATGGTGTTAGTTTCA	GAGATCAATTACAACAGGCCAA
245	cgd4_3500	TTTAAGAATGAAACCATTTGGAGA	ACCTAAACTTGAAACCACAAGACA
245	cgd4_3530	CATGGTTTGGAAGCAGAAGAGT	TTGCTGTGGTTGTGATGGTAGT
245	cgd4_3550	CCACACAAGTTAAGCCAGAAGA	CAAGATCCAAAGTGACAAATGG
245	cgd4_3570	ATTTGAAGTAAATGAGCCAGGAA	TACAAGAATTTGCCATTATTTGAGA
245	cgd4_3620	AGAAGAACCAAAGAAGTCCGAG	AGCTGGCTTATCCTGTGGAG
245	cgd4_3630	TCTGTAAGCGCAAGTTCTGTGT	TCTTCCTCCTCTTTCTCCTCCT
245	cgd4_3640	TGTCAAGAAAGAAGTTTAAGAACCC	TGAAGTAACAGAAGAATGAGTGGAA
245	cgd4_3670	ATATCTAAAGGCAGTTCCGGTG	TGTTGATCCTGTTGGTCTTGTT
245	cgd4_3720	ATAGCCCTTCAATTCTTCCTCC	TGCATGTTCACCTTGGTAATG
245	cgd4_3880	ACTGGCGAACATGGAAATAATAG	CATAAACTCCAACAAGTCCTGATAAA
245	cgd4_390	CATCACGCTTATTGGTGCTTC	ATCTTCGCATGGGCTATCAA
245	cgd4_3910	GGTAGAAGTAGAAGTGATCTTGGGAA	CCTTATCTGAAGGAGTTGTTGTTGA
246	cgd4_40	CTTAAACAATCCAATGAGGCAGT	CTGGGCAAACATATCGTAGTCTC
246	cgd4_4020	TCCAGAGGATGTACTAAGACCAGA	TTGCTTGTATGGAGAAGGAGTG
246	cgd4_410	AATCGGCAGAGATGTTATCAAAG	TCTCCAGCTTCGTGTCTAACC
246	cgd4_420	AATCTTTGCTGGGAGTATGTTTG	TCATTGTTTGCATTTCCACTCT
246	cgd4_4210	CCTGAATGACGTGTTTATGTTGA	TCCTAAATGTGACTGAATATCTGGAA
246	cgd4_4490	AAACTCTGCCGTTGTTGAATCT	CATTGCTTGCTTCATTCCCT
246	cgd4_520	GTGACGATGAAGATGATTATGAAGA	GCACCTCCTATTTCCCTTGAC
246	cgd4_580	TCCAGTAGAGATTTGCCCACTT	TATAATGCACCACAGTCCCATC
246	cgd4_630	TGCAATAGTCAAGAAACCAACTTC	GCTTGTTCTACGCTTCCGTATT
246	cgd4_880	ACAAGAATGGTGCAGTTTAGGAA	AGCAATAAGATTTCCCGATTGA
246	cgd5_10	CTGTTGACCCTTCTATTGAGCC	GGCTTTCTTCTCCTTCTTCTCC
246	cgd5_1030	GACTCGCGTATCGTTGTATCTG	AATTTGGGTTGTGCTCTTCCT
247	cgd5_1050	CCAGGTAACGCTCTTGAACC	GCCAACAATTCTGCAATAACAC
247	cgd5_1080	GATATGTTTCATGCGGATTTGTT	TTAAATGCTTTGCTTTCGACCT
247	cgd5_110	CTTTATGATCTTCCTCCTGCAAA	TTGAATTTGGATTTCTCGTTGA
247	cgd5_1150	GGTGCTCGATGTTCTAAGTCAA	ATCTTCTTCTCCCAAAGGAATGT
247	cgd5_1290	TGAAGTACGGAATTATGGGTGA	TTCCCTTGCACAACAACAAC
247	cgd5_130	GATGCAAAGCTACCAAATCCA	TGAAACTCCAATGAATCCAACA
247	cgd5_1310	TCAAACTGAAGCAGATGCAATAC	TTGAGAGTTCCACAGAACGAAC
247	cgd5_1420	TTTATGCTTCGTGTAGCCCTCT	GCAGATTTGTCAGGAAATAATGG

247	cgd5_1470	CGTCATTATCAAGTGAAGATTTGG	CGTGGATTGTGGGCTAGTTT
247	cgd5_1520	CTATTGAACAAGGGCTTAAACAGTC	TAAATCTGAGCTATTTCTGTGCTCTAA
247	cgd5_1530	AACTTTCTTTCCAATCACCATCA	GCTTGCGCTTGCTACTACATT
247	cgd5_1540	GATTGATTGGTTCACGTTAGGAC	TGAGCTATGCCACTTGTTATTCA
248	cgd5_170	TTCTTTCAACATCCTTCTCCAGT	ACTTCTGACGATTCCCATTCA
248	cgd5_1740	AGCATCCTGTTCCTTTAGAACCT	ATTCGATCTTGCCTTTCCATT
248	cgd5_1790	AATGGGAGTGTAGGATTGTAGAGTTAG	CTTTAGAGATGAATTTGGCATAGAAA
248	cgd5_1840	ACTTTATGACGAATTGGATGGG	AAGTCAGTGCGGGTTTAGAATG
248	cgd5_20	GCTGTGAGAATTGCAGACTTGA	CTGCCTGAGAAACAAACCCTAA
248	cgd5_2000	AAAGAACTACCATCCGACCAAA	TTGCTTTACAATATGCTTCCATTCT
248	cgd5_2030	AACAATGAGGCAAGTAACACAAGAG	GACATTTGATTCCAGACTTATTTCAAC
248	cgd5_2080	AGCGAAGGTAATCCAAACAGAG	ATACAAATCGCAAAGCAAGTCA
248	cgd5_2180	ACCGCTCCACTAAAGAAAGATG	AGGAGTAGCAGGAGTAGCAGGA
248	cgd5_2450	GTTCCAAGATGTCAAGGTTGTATG	TCCAAATACCTCTCATCCATTTC
248	cgd5_2550	GGATGGTAGAACTCTCTCTGACTACA	TTCCTTATCTTGAATCTTTGCCTT
248	cgd5_2630	TGTGGATCACTAGAATCAAACAGAA	CATAGGGCTTTACTTCCCTCAA
249	cgd5_270	AGATCCAGAGAATATAAACAAGCCA	TATTTGTAATTGCCAGAGCTGATT
249	cgd5_2700	TTTGGCTTTAATCTTGGTAATTCAG	TCCTTCCTCATCATTCTTCTCC
249	cgd5_2710	GAAACAGAAGCAGAAACAGAAACA	CCACATCAAATTCACATAGACGA
249	cgd5_2720	CTTTATTGTTTCTGGTCGTGGTT	CAATGTTGACGGAATTTGGA
249	cgd5_2750	GGATATTCGTGCCCTTATTTGT	TCTTTGCGTTATTTGGATGAGA
249	cgd5_2780	CACAAGAATCCAAATGATCCAA	TGCTGGTAAATCCAAATAACGA
249	cgd5_2800	TTTCCTTAAATCAATCCCAGAAAC	AAGACCATACGATCCTTTACCTTG
249	cgd5_2900	TGATGGTAATCAACTTTGGGTG	TGGATCTCCTGTAATGGGTTTC
249	cgd5_30	TGGTACACACTACTGAAGGAGAGAA	GCTCTTAGCTTGCCTAGATTACTTG
249	cgd5_3040	GAAGAACGATTCACCACTCACC	GGAACAAATACTGCAACTGCCT
249	cgd5_3050	GCACGAAGTTGTTAGCTCCTC	TTGATTTCATTAAGTAGTTGGGTCA
249	cgd5_3060	AAGACCTTCTAAGCGATCCACA	ACAAGTGCCTTTACTGATCCAAA
250	cgd5_3070	AAATATGGGTGAAATGGCTGAA	GACCAAGAATGAAGAGCTTTGG
250	cgd5_310	GATGGTATGGATCAAATTCAGGT	AAAGCGTTCCTTTGCACTTT
250	cgd5_3160	ATGAACCCAAAGGTAAATCGTG	TACGACCAGAGGCATACAATGA
250	cgd5_3170	TACTATCGCATCCAGAGAAATCC	TGTCCTCCAGTGAACTTGGTAA
250	cgd5_3210	CAACGAAAGGATTCAAGAAACAA	TTTCATCATAGACATCATCGTAATCTG
250	cgd5_3220	ATTGCTAGTGGAATGGCTGAGT	GAGGATACTTTGGGTAAATTCTGGT
250	cgd5_3270	CGTCCTAGAGGTTTCGGATTT	TACACTCAACCCACTTCCCTTT
250	cgd5_3280	TTGAGAGAATCCGTAATGTTGG	TTTAGCCGTTCTTCTTCTATTTGTT
250	cgd5_3290	GGCCAAGGATTTAACAGAAGAA	AATGTGAGAAAGATCAGGAGGG
250	cgd5_3340	ATGTGAGGCGGACTTTAGACTT	TAATTTGTTCTGTGCCTTCTGG
250	cgd5_3350	AGGATTGAAAGCACAAGGTGAG	ATACCACCCTTCCTGTTGGAG
250	cgd5_3380	ACTCAAGCCCAATGAACCTTT	GCTGCTCCCTCTTAATGACCT
251	cgd5_3420	CAGGAGCAATTACACATTTCCA	GTTCAGATTCCCAAGTTCCATC
251	cgd5_3430	AAAGAAGAGGCCGATAGATGTG	TGGTGATGATTATGTTGTGCTTC
251	cgd5_3450	ATCGTAACACAGCATCCCAAG	AACATCTTCTTCATCAGCAGCA
251	cgd5_3460	CAGGGAAGGATGGATTTGATAG	GCCAGTCATAGTTTGTTGTGGA
251	cgd5_3490	TCTGTTATTGCAGCTTTGCTCT	TCTATCGCCAACTTTGTTCTTG
251	cgd5_350	TCTACAGAACTATCAAACCAGGAATCT	CATTATCATCCTCATCTCCATCAA
251	cgd5_3530	TGTAGGGCGTATATGAGGCTTG	CAATTCGTCATTTGAGAGTATTTGGT
251	cgd5_3570	CAAACTTGACTCTAAATGCCGA	GCAACAGCTTCAACACCTCTAA
251	cgd5_3600	GTTACCACAAGAAGAAGTAGCTTTGA	CACTAATAACCTCCCTGAATAGATTTG
251	cgd5_3680	CCCTTAAACCATTCAAATCCTG	CATCCAATGTACTCCACCCAC
251	cgd5_3690	ATTTCCCTAATTCATGCTTTGCT	TGAGTCTTTATTCTGATTCCATTCTCT
251	cgd5_3770	ACTTCTCCATTTCACGAACCAA	ATAACCCGATACACCTCCAACA
252	cgd5_390	TTGACCTTTCACAAACCAGAAA	AATCCCATCCATGAACTCTAAATC
252	cgd5_40	GAAGATGAAAGTGACGAGGGAA	CGAAGCAGAATTAACAATCGAAG
252	cgd5_400	TTCATTACCATTCCAACTTCCTG	AAATTCTTCATCTTGGGAGTCGT

252	cgd5_4090	AGGCTTCCTCCTGGTTCTATTT	AATTCCTCCAACTCCTCCTCTT
252	cgd5_420	TTCAACTCCAACTCCATCTTCA	ATCTCTTTCTTCCAATGCTCCA
252	cgd5_450	CAACGATAGAAGAAGGTCACACA	AAGAGGATGAGGATGAGGAGG
252	cgd5_4520	GATGTTATGACTGCTCGCTTTG	AAATGTTGCTGGTGGTTTATTTG
252	cgd5_50	AATTTCCTCCTTCAGCATCATC	GCTGCCCTGGTACTTGTTATTC
252	cgd5_630	TTGAAGCCAAATTCTGATCCTT	CACCATTTCTAAGTTTCTTGCGT
252	cgd5_90	CGGCTAATCTTTCATTGTTTGA	TTTGCATTCTTAATCATCGGAG
252	cgd5_920	AATGTGCCCAAAGAATGAAGA	ATTAGCTGCAACCATCCAAACT
252	cgd6_1000	AATGGAGATGGAGAACAACAGG	TAGCATTTTCTGCCTCCAATTT
253	cgd6_1040	GTGGCACCTGCTTATCATAGAA	TTCAAGGATGAATTCGCAAGTA
253	cgd6_1050	AAAACCTTTGGATGTGGTTCTG	TGATCTTCAAAGCTTCTTCCGT
253	cgd6_1150	ACCAGGAACAGTTTAACTCGGA	CTTATTGCATTGTTGGCTTCAA
253	cgd6_1190	AGAAATGAAGAATCTCGCAAGC	AGACCCACGGTATCGTTCTTTA
253	cgd6_1210	TTCAACTAACGAAGGCATGCTA	ATCGGTTCATTCAAATCCATCT
253	cgd6_1220	TCCAACTGCGAGAAAGAAAAA	TGTAACACTTTTCAGCATTAGCCTAT
253	cgd6_1240	CATCTTGGTGTAGTCAAAACCG	CCACAAATCGATACTTAGCCCT
253	cgd6_1250	AAACAAATTTTGGTGGAAGTGC	ATATTCAGAGTTTTCAGCCCCA
253	cgd6_1260	TGGGTACCTTGGGTATCTTTCTT	ATTTGAGTATTTGTCGTGCCCT
253	cgd6_1360	TACTCTTTTCATGCCAGAACCA	TCGGACGTAAAGAGTTGTTTGA
253	cgd6_1540	AAACTTCGAAGCAATCGAAAAG	TCAGTTAAGCCACCTGTGAATG
253	cgd6_1650	TCGGGAAAGACCACACTATTCT	TTCTTCTGTTCCCAGGGATG
254	cgd6_1660	GTATGCCCTCCTGGATACACAT	TTGGAACTACACATGGCTTTTG
254	cgd6_1690	AGTAAACCAGGTTACGGACGAA	TGTAGAAATTTCCAATTGGGCT
254	cgd6_170	CAAACCAAATTTCCACAATTCA	GCCATTCAGCACTTTGTTGTAA
254	cgd6_1800	ATTGCAATCATTGGTTAAAGGG	TGATGTATGGCTCATTTTCTGG
254	cgd6_1880	AGCAACTGAAAGCCCATCTTAC	ATTGAATGCCCTTAATTCGAGA
254	cgd6_1890	TAAACTGGCAAAACGAACAAAA	CAGTTGCAATCAAATGGACAGT
254	cgd6_1920	GTAACTGCTCTCCTGACAGGGT	CGGATTTTAATTCAGCCTTTTG
254	cgd6_1980	GCTTTTCCATCAGAACCTCATT	TCCAGAAATTTGTTTGCCTGTA
254	cgd6_2010	CGGAAAAAGTAAACGATGAAGG	GAAAATATGCAGTTTGCACCAA
254	cgd6_2050	AACTTTTGGAGAAAGCAGGATG	TATCCTTCGCTGTCTGATTCAA
254	cgd6_2160	ATACGGCGGAAGAATATAAGCA	AGCCAACGCAGATATTTCAACT
254	cgd6_220	CAAGTACAGGAACATCAGCAGG	CATCACCAGATTGTTGTTCGAT
255	cgd6_2230	GGGGTCGACTATTCAATACAGC	ACCATTTGATGCCCAATAATTC
255	cgd6_2330	ATCCGTGTTTCTGTCGTTCTTT	TGCTCTTAATGAAGCTATGCCA
255	cgd6_2400	CGAGTGCAACTATGGATCTCAG	ATAATATGCATCCGAAACACCC
255	cgd6_2420	TTATTCTTTATGCCACATTGCG	CAATCATTCGAGCTGAATCTGA
255	cgd6_250	AGATTCACCTTGGTGCGATATT	TTCTTCCCATTTTGAGGGTTTA
255	cgd6_2530	TTAATAAGACTCAAAAGGCGGG	GCTTTGCTCGCTCTTTATATCC
255	cgd6_260	TAGTCATGTTTCCTGGAGGGTT	TAAAGGATTCCACCAAGTTTCG
255	cgd6_2600	TGAATTTCTTGGCATCATTGAC	GGGAAAGGAATTGCAGTACAAG
255	cgd6_2630	GGATGGATTGACATTTGGTTCT	GTAAAAGATGCTGTTTCCCCTG
255	cgd6_2650	TGATCCGGAAAAGAGGAATAGA	GTGACGATCAAATCCATGAAAA
255	cgd6_2830	AAGGAAGGAAACACCAAACAAA	TTCCCAACAAGACATGATTCTG
255	cgd6_2840	CAGAGAGGGAGTCAGGAAAAGA	AGGAGGAAATCTTTGAGGTTCC
256	cgd6_2900	TTTATGTCCTAGAGGGTTCGGA	ATCCAATTTACATCGTCCCAAA
256	cgd6_2910	CTGCAATTTGAATTTTGATGGA	TTTGGTTAGGTTGAAAATTCGG
256	cgd6_2920	TGTTTGATACCATTGGTGCTTC	ACTGAATCCAAAACAAAGCCAT
256	cgd6_300	AATGGTTAAGAGCTTATTGGTTTGA	AATGCACCAAAGAAACCAAGTT
256	cgd6_310	TCAAATTCTGAAAAACAATCGGT	TCCCTTCCTTTCCATCATTAAA
256	cgd6_3110	GTTCCTCCCATTCCTCCTATTC	CGATGGATTCAACACAATGATTA
256	cgd6_320	AGTGAAAATTCTCAAACATCGGA	GATAGTCCAAGAAGGCTTTCCA
256	cgd6_3200	AGCTCAAAGAGAAGGTGGGAA	TCTCCATTGACATCATTCCAAG
256	cgd6_3320	ATCTTGGAATAAATGTGAGCCG	TTCCATCCAAGTTACCCAAATC
256	cgd6_3340	CCAAACTGGAGAAAACCAAAAG	TACGTGTCTTGGCATCACTACC

256	cgd6_340	GTAGTTGTTGCTGCTAACGGTG	ACCAAGAAATTCCCAAGATTGA
256	cgd6_3440	TTGGCCAGAAAAAGCTGATTA	CAAATTGATTCGTCAATCCAAG
257	cgd6_3450	TGAGCTGGAAACTCACGTTTTA	ATCCCATAGTCCAAAGCTGAGA
257	cgd6_3460	AAGTTGCTACTGGCCAAGCTAC	TCCATGCAGGCATTAACATTAG
257	cgd6_3470	ATGGGTGGTAAACAATCAGTCC	TCTTAAAGCTTCAGCCTCTGCT
257	cgd6_3480	TGAACCAAATGGAATTGTTGAA	GGCCAATTACCACCAGAATTT
257	cgd6_3490	TCATATTTTCTCAATGGGGTGA	CACTTCCTTCAGCACCTGTAAA
257	cgd6_3530	ACCCAATTAATGGAGGAGGATT	AACGATGATCCATCTTTTGACC
257	cgd6_3580	TCAATGATTCATCTCCACAAGG	ATCCATTGGCTCATAATCCATC
257	cgd6_3600	GAGGCGCTTATTCACCATTTAG	CATGACGCAGCTTGTTGATATT
257	cgd6_3630	GCTCTTAAAGCTGGCACAGAAT	CATCATCATCATCAGCGTAGGT
257	cgd6_3670	AATTGGGAAAGGTTGGAATACA	GCAGCTCAAGAGTTGCAAGTAA
257	cgd6_3740	GAAACCAGGAATTAGGCTTGAA	TTTAAATCCAACAGAAATCCCC
257	cgd6_3760	CACAAGTTTTTGAAGAGGGAGG	GCGCTAAATCCTATTGCAGTCT
258	cgd6_3850	TTTTAACCCTAAGGCATTTCCA	CAGCATCTGCAGCAAGTAAAAC
258	cgd6_390	TTTGAGAAGCCGACAGTAACAA	CTGGAAGGTCTTCATCAACCAT
258	cgd6_3910	TGCAGAATCAAAAAGCTGACAT	TCTTCACCCCCATTTTCACTAC
258	cgd6_3930	TCAGCAGGAGTTCAAGAATCAA	TGGCTTCAAAGGTGGTTTTACT
258	cgd6_3970	AGTATCCCAAGCCAAAATGAGA	GCAAATCTGCAATATGGTTCCT
258	cgd6_3980	AGAGGGTGAAAAGAAAACAGGAT	TTGTGAACTTCCGAAGCATTTA
258	cgd6_4010	AGCCTAAACTCAAGCTCACGTC	TGTTTCTCCAGGCTTAATTGGT
258	cgd6_4190	GTGGGGTTTCACTAAGTTCACC	AGAGATCCACGGCAACTAATGT
258	cgd6_4210	ACAATGGAGGGTGATATGAAGG	CTCCAAATACGAAACTCGAACC
258	cgd6_4250	TTGACGTTAAAGAAATTCGAACA	TCATACTTTGGAAAATCCATGC
258	cgd6_4260	CAGGGATTGACTATTCAAAATGG	CGTGATACGTGTCCAGGTTCTA
258	cgd6_430	AGACGATATGGGAACTATTCATAAAG	AATCCATTAAACTTCCTTTCCAA
259	cgd6_4300	AAATTTGAAAATGAAATTGGCG	AATATGCGATCTTCCAATTGCT
259	cgd6_4450	AAGAAGTGTGTTGTGGGAGGAT	CCGAGTCCTTTTGATTGGATAA
259	cgd6_450	TGAAAGATTTAATTGTCATGGAGC	CTATTTGCATTGCTTGGATGTT
259	cgd6_4520	GAAATCAAGGGGAGAAGGAGTT	CTCCCAAGACTTTGCTAGAGGA
259	cgd6_4560	CAATTCCTCAAGGTTTGGAAAG	TATGCTTAGCAGCAGAGCAAAG
259	cgd6_4620	ACCATGACTACGAGGGAGTTGT	TGACAACATTTGATGGGTGAAT
259	cgd6_4690	CTCCAGACCCCAATTTAGTCAG	CCTTGAACAGTTAAATCCTGGC
259	cgd6_4820	TGCGAAACCAAAGTAACAGTTG	ATTTAATTCCCCATCCCAAAAC
259	cgd6_4830	TTCATCTAGAACTCGCCAGACA	ATCCCTGATTTGAGACCAACTG
259	cgd6_4860	AAGTAGGATTAGGCATTTGGCA	TACATTTTCCATTAATTCCCGC
259	cgd6_4980	GCTCCACTAAATATTCGCACAA	CATTGCCAGTAAAAATGACTGC
259	cgd6_5100	TCTTTTCCCAAGCTCAAACATT	TTATGAGAATTGGACTCGGCTT
260	cgd6_5120	GCAATGATGTTGAAGAATACGC	ATTCCTTGACTGGAAGCATTGT
260	cgd6_5220	ATTTGGCAGAATTTCATGGTTT	CAAGTGTACCTCCATTTTCCAAG
260	cgd6_5230	GATAATTTTGGGATGAATTTCTCAA	GGACCGTTAACTGCTTCATTTT
260	cgd6_5270	CGAAATTCTGCACACTTCTTTG	AAGTACTCGGATGCAGCTTGTT
260	cgd6_5430	AAATCATCTCGCATTTCCAAGT	AAAGTTCACGAATAGCCGATGT
260	cgd6_5470	TCCTCCAAGTAATCGCAGAAAT	TTAAGGCATTCCGAACACTTTT
260	cgd6_550	TCCCAATTTCTGTTGAAGTGTG	CTTCAAATGACTGGATTGGGTAG
260	cgd6_5500	CCCCTGTTAAAACCAATGATGT	TTCTTGAATGGAAAGTTCAGCA
260	cgd6_630	AACAAATTTCCCAGGTGGATAC	TGAAAATGATAACTTGCTTGCC
260	cgd6_660	TTGGTATTGCCAAAGCTAAACA	AGCTTCATCATCATCAGGAGGT
260	cgd6_700	CCTTTTAGATGGGTTTTATTTCCA	TGCAAAGGCCATTTCAATATAA
260	cgd6_750	GGAAGAATTGGTGAAATCGAAA	TTGGTAAAGCTCCAAAGTTTTCA
261	cgd6_810	TTTCAAATAGGCCAAAAAGGAA	ACTTGTTGGGGTACATCCAGTC
261	cgd6_830	CAACCATCCGGGTGTATTAACT	GTTTCCACACGTGTTACAGCAT
261	cgd6_860	AAAAGTTGGCTTTACCTGTGGA	TTATTAACTTTGCTTCCTCCCG
261	cgd6_880	TCCCAAGCTCAGGAATTTGTAT	TCAAATTTTGGCTTTGGTCTTT
261	cgd7_1030	TGATTGTGAAATGGTAGGATGC	TCCCAAGTAAGTCCACTCCATT

261	cgd7_1040	TGGGATTAGAAAACTTCCTCCA	TTCTACAGAAAGCTGAAGCTGAAA
261	cgd7_1050	TAAGGACGGAATTCCCTATGAA	GCATGTCCTTTTCTAAATCAAGC
261	cgd7_1070	TGGTAACAGTGGAGCTCAAGAA	GATCCCAAATGAATAAAACTCTCG
261	cgd7_1080	TGTTGTGGATCCTTCATTTTCA	TTTCACATGGACTTGCAACAAT
261	cgd7_1100	AAAAGTGGAGTTGGTGGAAAAA	TTCCAAAGAATTGGATTTCACC
261	cgd7_1120	AGATTTGAGTTTTGGCGATGAT	GACAATTATGTCCTGTGGCTGA
261	cgd7_1170	GCTTTGGGTGAACATCTTTTTC	TATTGGAGGCTTGAGGAACATT
262	cgd7_1210	TGTTGACGAAGATTTCTTGGAA	ATTTGAGGGAGATTGCAGTGTT
262	cgd7_1310	GGCCTAAAGAAAATTGGATTGA	ATTTACCAGATTCTTCCCACCA
262	cgd7_1330	ATACTGGAAAAACTGGAGCCAA	TTCTGCTGTGTCATTTGCTTCT
262	cgd7_1340	GGCTGTTATTTTAACTGGTGGC	AGCCAACTTTTGACCAATAGGA
262	cgd7_1360	AGAACAAATTGGACGCAAGATT	AAGCTCATTTAAAGCGGTCAAG
262	cgd7_1380	GATTTCAGTTTCGATTCTTGGG	AAGCTTTCGCTGATTAAGCTGT
262	cgd7_150	AGCTGAGGTATGGACCGAACTA	ACAATGTCCCGAAGCTTTGTAT
262	cgd7_1520	AACTTCAAGAGTACAAATCCGTTCTT	CTTCATCCGGATCATCGAGT
262	cgd7_1530	GAAGTTCAGTCAGGGGAAGATG	TATTTGCCTTGGTAATCCATCC
262	cgd7_1560	CAATCCACCTTGCTCCATAGAT	GATACATGTTCCTACCCAGGGA
262	cgd7_1570	TGAAAAACTTGCAAAGACTGGA	ATGGTCTAAATCGCTGAGAGGA
262	cgd7_1600	GCGGGGAAAGAGGTCTTATATT	CCTTCTCTACCAAATTCCATCG
263	cgd7_1620	TTCCATTTGGATTATTTGAGCC	ATCTGAGCCACTTCCATTGATT
263	cgd7_1660	GCAGTGGGAACTACCAACTTTC	AAGTCCCACTTTCGATAGTCCA
263	cgd7_170	CAAAAATTGTATGTGGAACGGA	TTTTCAAACCCATCAACAAATG
263	cgd7_1740	TTGAAATGACAAATTTGCTTCG	CCTCAATTTTTGGACTACCAGC
263	cgd7_1870	GCAAAACGAGACAAGATCACAA	CAGAACCATTAAAAGGGAACGA
263	cgd7_190	CTGTTAGAGCTGGAGAGAAGGG	AAATTCGTTCGCTTCTTTGATT
263	cgd7_20	ATAAATGGTCGCCACTTCATTC	CGCACTCATGAATATTGCAGTT
263	cgd7_2060	TTAAAAATTCCCTACGGAGCAA	TGCAGAGTCCAAGTCTCCACTA
263	cgd7_2150	TTTCGATCGACACAGGTAACAT	TGCTTGCTTTAAAAACATCTCCT
263	cgd7_2180	TACCTAACAATGCAGGAGCCTT	GTCGAAAGGAGTTTTTGGAGAA
263	cgd7_220	GGAGGCGTTGGTAAAACTACAC	TGCAGTATCCCAAACATTGAAG
263	cgd7_2220	AAACAGCAGGCTCTCCATTTAG	TCTCTGATTCCATCGGGAGTAT
264	cgd7_2230	TAAATCGCATGGAAACACAAAG	TCTGGAAGTTCATTATCACCCC
264	cgd7_2240	TAAAAAGAAATCTTACGGGCCA	ACGATTCTTCGGTATTGTTGCT
264	cgd7_2250	GGCACCTCAGTTAATTTCCAAG	TCTTACTTCAACGCCTGCATAA
264	cgd7_2350	TGAAGTAGTGCTTTCCTCTCCC	AATGTGTGCAACTTACCTGGTG
264	cgd7_2390	ATTCAACAGGACTGGGAGCTAA	GCTCAGCCAAAAATCTTTCAGT
264	cgd7_2420	TGTATTGAGCGAAGTTCCAATG	TCACTCTTCACCTGCTTCGTTA
264	cgd7_2630	AGGCCAGATTTCCCTAAACTTC	AGGCTTCCATTGCTTCATACAT
264	cgd7_2730	AAATGCAGAAAATGTTGCAATG	CTCCTGAAATAACCGCAATAGG
264	cgd7_2800	GACACCGATTTTTCATCCAAAT	CTCCGCATCTTAAAATGTTTCC
264	cgd7_2810	TTTAAATGCCACAGGATGTCTG	CCAAATTGTCGAGTTCATGTGT
264	cgd7_2890	TTGCCAAAAGTAATGGATTGTG	CCATATCAGCATGCCTTTGTAA
264	cgd7_2940	AACTGAAGACCCCAGATTTCAA	TCCATTTAACAAGTGGCATCAG
265	cgd7_3020	GGACCTCCTCCTCAGGTAGTTT	AAATCCAGTGTGAAGCCAAAAT
265	cgd7_310	CAGATCTCTGCTTTCCCTGACT	TTAGGCCTTCAAGAAATGCTTC
265	cgd7_3150	ATATCGCAGGTTCATTATTGGG	TCCAGGAGAATAGGAAAAACCA
265	cgd7_3210	TGTGAATGCTCCTTCGAGACTA	TCCTCCGTAAATGAAGGTTTGT
265	cgd7_3240	CATTATTTAGAATGCGGTGCTG	TGAATCATCTTTCTCGTCCTCTT
265	cgd7_3260	AAACATATTGGGACAGACTGGG	TCAAGTTGCAGCTGCTCATAAT
265	cgd7_3300	TGTTGGATTTCAAGAGCTGGTA	TAATTGGAGGTGAGCTGGATTT
265	cgd7_3310	AGACAGGGTCAAAGATCAAACC	CTTAAAGCGTAATTTTCTCCGC
265	cgd7_3330	GCCACATCCAGACACTGTTAAA	GTTTCCAAAACCGATTGATGAT
265	cgd7_3430	GCCTAGGTATATGGCACCTGAG	TATAGAGGGGTGGAACCATGAC
265	cgd7_3500	ATTCTGATCTTTTTGGCGAGAG	TCCCGAAATAATTTGTAGGCAG
265	cgd7_3550	CGCTGTCTTGGATGAAGATACA	TCAAGTGGCATACCAATCTCAC

266	cgd7_3570	TCAGGTATTTCATCATTCACGC	CCAGTAATAAATGAAGAGGCGG
266	cgd7_3610	GATGTAATTTGCGCGTTATCTG	CGGATGTATAATGTGCCTGAGA
266	cgd7_3690	AAGAAAACTACGATTTTGCCCA	ATGAGCGACGTGGGTACTTTAT
266	cgd7_3720	TGTGGGCTTATTTGTATTGCAG	TGACATTGCCATTAACTCTTGG
266	cgd7_3740	ATTCGAAGCAAAATCACTCCAT	CAATGGATCAATATATCCGCCT
266	cgd7_390	GATTTCAACAAAGAAGCAAGGG	TCAATGTTGTTGGAGGTGTTGT
266	cgd7_4000	TGAACTGCAGCAAATGAAACTT	TGCAATTGAATTAACTTGGTGG
266	cgd7_4060	GGTGGTTACCGAAAGAACAGAG	GGCTTGGCTTCAATATTACTGG
266	cgd7_4270	GGACGTAATTGCTCCTTCAATC	AATACAAGTGGACATGCTGTGG
266	cgd7_440	ATTTGGAATTCAGGTCATGGAG	TCCTCTTGAGTTGAATCGTCCT
266	cgd7_4430	TCTTTCCCGGCAATCTTTAATA	TCATTCTTGGGCTCATCTTTTT
266	cgd7_4510	GAAACTGGCCATATCAAGGAAG	TCAGCTTCCACAAGTCTCAAAA
267	cgd7_4550	TTTGAGCCTGAAAATACGGTTT	TATCGTGCATCCAGCTGTTACT
267	cgd7_4770	TAAACCAAATGAAGGTCCTCGT	TTCATCAATCTTCCAGTCCTCA
267	cgd7_4800	ATCTTTTTGCCAGGAACGTTTA	CTTCCCCAAACTCTCTCTGCTA
267	cgd7_4850	AAAGGCGAATCTGAGAGCATAG	CCCTAAAAGCCTTTCTGGACTT
267	cgd7_4890	CCTATTCAAATGATCCAGCCTT	GTGGGAAAGAATCGAAAACATT
267	cgd7_4990	TCAGGATCAATTGCTGCTTCTA	TGTGAAACCGATACTGGTTGAG
267	cgd7_5070	CATATTCGTTTTCTTCAAGCCC	AGGATTTTCTAGTGCAGCGTGT
267	cgd7_510	TGTCCAAAGTGCTCATTTTCTG	TCCTTCCATTTGAAGTCACATTT
267	cgd7_5160	CCCATATAGAGGCCAAGCATT	TGTATCCCGAGCTCTTTTTCTC
267	cgd7_5320	ATGTATGCCAATCATGCGTAAG	ATTCTCCATGAAATGATCCCTG
267	cgd7_540	TGGATTCTTGAAAATTCTTTGGA	TAACTTTATCCGGATTTGGGG
267	cgd7_5400	AGGATCTACAAACCCAGCTGAA	TGCATCATATCTGGGAAACTTG
268	cgd7_550	TGTATTTTAGCATTGGCTGTGG	CATGAAGTGCTTTCTCCAAACA
268	cgd7_570	GTTTTGGGAGAAGCAATTTACG	ATAAACTCGCATAGCATTGGCT
268	cgd7_590	GTTTCCCCTGAATTGAGGTATG	AACAAGCTCTCCTTGACAAAGC
268	cgd7_600	ATTCAAAAGCAATGGGATGATT	CATTTGACTTGGATCCCTTTTT
268	cgd7_610	ACCAACTAGAATCATCATGGGG	AGTCAATATTCATTGCGGCTTT
268	cgd7_800	GGAGTCCATGTATTAACCCAGC	ATTTACGTCGTCGGAATCTTGT
268	cgd7_810	TGGATGGATATCAATGGTCTCA	CAAAATAGTCCGGAACTGGAAG
268	cgd7_850	GGAGAATTAGAGGCTTGGAGGT	ATAAACAAGGGGCTGTAAGCAA
268	cgd7_870	ACAGAATGGGGAAGTAAATGGA	CAATTTGAGTTTGAGCTCGTGT
268	cgd7_890	CCCTTCAAAAGGTTCATACTCG	CTGATATGGATGAGAATTGCGA
268	cgd7_990	TCTCTCAGAGTTTATTGGCGAAC	ACTCATGGGAGTGGAGATCATT
268	cgd8_1070	AATACCAGAACCAAACCATTCG	CTTCTTTCAAAACCTGGTACGG
269	cgd8_1100	TGGGAGCTTTGCCTTATATTGT	ATTTTCTCCCAACAATCCTTGA
269	cgd8_1110	TCAATCTGGCTTGAATCTGTTG	GGCCATACCATTTACTCATTGC
269	cgd8_1130	AGAGTTAGCATTGGATGATTGG	TGTTCGATCAGGTTTATATTGATTT
269	cgd8_1140	GGGAGTGGTATTGGTCTTAAACA	TTTTTCTAATTCCCAAACAATCAA
269	cgd8_1190	AGCAGGAAATGAACAATTGGA	AATCAGAAAGATCATCAATAATATCGTC
269	cgd8_1210	GTAGGTGGAATTTCTGCCACA	TAAATGCAAATCCAAATGCTTG
269	cgd8_1250	CCAAATTCCAAGTCAACACTCA	CCATTTTGAGAAACGCATGTAA
269	cgd8_130	TTAAATTTTGGGGCCATGATAC	TGGGTCCTTATACTTTCCGCTA
269	cgd8_1300	CCCAAAACTTGATGATGAAATG	ATTTTGTTTCCAGGTTGATCGT
269	cgd8_1310	AAGGGATATTCTTAAGCTGGGG	TGATTCATTTCCAGGTCCTTCT
269	cgd8_1340	TTTCTACCCCAAGAAACTTCCA	TGTTTTCAAAGTTGTGTTTGCC
269	cgd8_1390	CTGGTACTTTTTCCCAGAGCAA	TGACCAGGAACTACGTCATCAG
270	cgd8_140	CAGGAAGGGTATCAGGAGTCAC	ATTTGACCTGCTCCAACTTGAT
270	cgd8_1410	TGGAAGTCACTTTGGACTGATG	TTCTTTTTCCTTCTTGCCCATA
270	cgd8_1420	TGACAATTCGAATGAGACGAAA	CCAATTTTTCTGGATTTACCGA
270	cgd8_1460	CAAGTCTTGGAGGTTCTATGGG	GCTCTTGGACTGATAATGGACC
270	cgd8_1470	TTAGTAAGGGAATTGAAGGAGCC	AAAGAATATTCCACCTGGGCTT
270	cgd8_1480	AGAGAATGCTCAATATTCCCGA	TAAAGATTCAATTGGGGAGGAA
270	cgd8_1490	GCGTCAGAATCTGGCTTACTTT	ATTTCCGAACTCCTCCTTCATT

270	cgd8_1550	AATTTGAAATGGAGGATGGAAA	AACCCCAACCTTCAAATTAACA
270	cgd8_1640	TGGTCCAACAGTACAGCAGATT	CGAAGATTCCTTTCAAGTCGAT
270	cgd8_1810	AATTGCTATGGAAAAGGCAATG	GAAATTTCAGCCCATTTCTGTC
270	cgd8_1820	CAAGAATGCTCCAGTCTCACAG	GTTATAAGGTTGATTGCGAGCC
270	cgd8_1920	TTGGAATTATCAGCCCAATTTT	ATATGAATGAATGGTTCCAGCA
271	cgd8_1950	AAGTCTGATCAAAGAGGGCATT	CTCCAGCTCAAGCTCTAATTCA
271	cgd8_2230	AAGAGCCATGGAAACTTCAAAT	AAAGTTGGTGTCAGGAAGAAATG
271	cgd8_2240	CAAAATGCGAGAAACAATTCAA	CATGGTACTTTCAGCATGTCGT
271	cgd8_2280	TCATTAACAATGATAGCGGGAA	AATAGCAATCCAAGATCCTCCA
271	cgd8_2290	TAAATTGCTCAATGAACGGTTG	CAGATTCATAAATGTGCTCCGA
271	cgd8_2310	TTGCAATTTTTAGATTGGTGGTT	GACCTAAAAGGAATATTGAAGGAGG
271	cgd8_2350	TTGGTTCAAACGTATGATCAGG	AGGACATTTTCTGGAGGTTTCA
271	cgd8_2400	AAAGGATTCCCAAGGAAAAAGTT	GCAGCCTCTGCTTGAAGTAAAT
271	cgd8_250	CCCAAGACACAAATAATGGGAT	CAACCTACGATCTGTTCCTTCC
271	cgd8_2570	GCCAATTCCAGATTGTTTTAGC	TCTTGGAAATCCACCATTATCC
271	cgd8_2590	TCTTCTGTTTCTTCTTTCGTCC	TCACTTACCTTGGTTTTGTTCA
271	cgd8_2600	GAGCTTAACCAGAGGTGATGAAA	TCAAACAAAGGTGAATCATTGC
272	cgd8_2610	AGGTTTAGGGATATCTGCGACA	TAGTCTTTGGGTGACGGGTACT
272	cgd8_2680	TACAAGCGGAATGAGAATAGCA	ATGGAATGAGCATTTCAGGTTT
272	cgd8_2690	TTGTTCGTTGGAATGTTTTCAG	TCATTTCCACTAATCTCGGGTT
272	cgd8_2740	CCCGGATAAAGAAATGAATCAC	CCATTAATAACCGTTCCTGCTC
272	cgd8_2900	TAATGGCAAATTTATTCCCGAC	CAGAAAATGCAAAAATTCCTCC
272	cgd8_2920	TTAAATTCTGGCCCATTGAAGT	AAAGTCCCAATTATTGAGCGAA
272	cgd8_300	AGAAAACAAACGGTTGAGGAAA	CCGAGTCTGTGAGCTAAAACCT
272	cgd8_3010	CAATGCAAAACACCACTTGTCT	CAGCCAAATGTTTCCATGAGTA
272	cgd8_320	ATTGATCCCAACCCAACTAATG	CTAACAAAAACAGGATGGCTCC
272	cgd8_330	CAGGTTTAATGAGACCAGAGGG	TACGTATTGGAAGGAGCCAGAT
272	cgd8_3340	TCTGAAAAGAAAAGCGAAAGGT	CAACGGATTTATATCGGGAAAA
272	cgd8_3400	ATGATGAGCCTCTATTACCCGA	TGGGTAAATTCCATTTTTCCAG
273	cgd8_3550	TTATGGAGAAGCAGGGTTTTGT	TCCTACGATTGAATTCGCTTTT
273	cgd8_3570	CGAGCCTAATTATTCCAGGATG	CTTGGTATCCATATTCGGGAAA
273	cgd8_3630	GGGAAGGAGTTTGTTGGTACAC	CATTTTCAAAGTTTTCCCATCC
273	cgd8_370	AGTTTTTGCAGGTCAGGACAAT	TTAGCTGGATTGAAGGGACAAT
273	cgd8_3700	TACTCAGTGGTATCTTTGGGCA	GCTCAACGTTCTGGATTTCTTT
273	cgd8_3810	TCCTCCAACTGGATTAATTTCG	AATTCCAAACTTTGCTGAATCG
273	cgd8_390	CTTGGGACGAAGAAACTATTGC	GATTCATCAATTCGGTTGGAAT
273	cgd8_3990	GCCATCATAATACTCACCGTTCT	TGTGACGATGATGAATGAGATTT
273	cgd8_400	CAATCAAGAACATGATGGTTGG	ACAACAACTCCTGGAAGCATCT
273	cgd8_4060	GGAAGACTCCACAGACAAAAGG	AAGCTCAAGATATGGCACCAGT
273	cgd8_4250	CGTAGAATTTCCCCATTTTCAA	CAATTATTACGGTTGCTCCACA
273	cgd8_4310	TGCATTTGCTCCAAATAGTGAA	TAAAGCCAATACATCCTGGAGC
274	cgd8_4350	GGTTCCTCCAAGTGAGTCAAAG	ATTTCAATGCCTCGATCTTAGC
274	cgd8_440	GGATCGTATTCTTGGAAAGCAC	CTAACGTATTGGGACTTCTCGG
274	cgd8_4510	TATAGGAAGCCAACAGCCAAAT	CTCGTTTGATCCCCCATTATTA
274	cgd8_4560	ATGGGGTTTCCAGTATTTTCAG	TTCCATAGTGCTTTGAGATGGA
274	cgd8_4580	AGGGAAAACAACAAGATGGTTC	TTTCTGTTTCAAAGTTTAACGCAT
274	cgd8_4590	TTTAGGACCTTTGGAAAATTGGT	CAAATCCTTCAATCTTTTCCCA
274	cgd8_4600	AGCAAGAACAAGAGCAAGAACA	AGTTCCACGTGTATTTGGTGAA
274	cgd8_4610	ATGGCATTTTTCAATTACCAGG	GGCAAATCAAATTTCAATGGTT
274	cgd8_4670	AAGGATTTCATACCAAATTTCAACA	TTTCGCTCACAATACTGACCAC
274	cgd8_4680	AAGGTTCAAAATGCAAGGAAAA	CTACCCATGGAGAAGTAATCGC
274	cgd8_4690	ACGAGGGTATTGAATTGAAGGA	AGTTCCAAGGCTGTCAAAATGT
274	cgd8_4700	CAAGGAGCAGAGGCTGTAAGTT	TTGGGCAATTTATTCCTTTTTG
275	cgd8_4720	AGGCCATCGTGGTTACTTTTTA	TATTTGAATCAACCTCGCCTTT
275	cgd8_4750	AGCTGCAGCATTACCACTCATA	TTACAACATCCTGGTTTTGACG

275	cgd8_500	GTGCTTCCAGACTTGGTCTTTC	AAATGGCATTATCTCCATCACC
275	cgd8_520	AATTCAGGAATTCAAGACGCAT	TTCTTCGCATCTTTCTTGTTCA
275	cgd8_540	ATTCGCTTTTCATCTGGTCATT	TTTTCTGATAAGCTCCACCACA
275	cgd8_550	AGAAGCTTCGTTCTGCAGACTT	CGAACTGTCGAACTGTACAAGC
275	cgd8_560	GGAGATATTTCTGGTCATTGGC	TAAAATCGGCCACATTTCTTCT
275	cgd8_580	TCAGAAAGGGCAAGCTACTGAT	CAAAAATAGTACACGCCCAGGT
275	cgd8_650	CTGCAACTAAGGCAAATGCTAA	AAAGTTTCAACCATCTTCATTGC
275	cgd8_670	AATTTCCAGGATTTAGCCAGAA	TTCAGCAGTCAATTTTTCGATT
275	cgd8_680	TAGTTCGTCGGGAGATTTGATT	GAACTACTGGTTTTGCTGGTCC
275	cgd8_700	CTCCCACGATAAGCAATAAAGC	CTCCTCCATTTTAGCATCCAAC
276	cgd8_750	GCAAGGAGCATCTGGATTTTAC	TAAGCGCTAATTCTCCAAAAGC
276	cgd8_760	TGTTAGTTCCAAAAGGGTCGTT	GGGAGGGAGCATATAGTCATTG
276	cgd8_790	TGTTGATGATACATGCGTTGAA	TCCTGTTCTATGGTGTGGAGTG
276	cgd8_830	GAAGAGAAAAAGGATGAGCCAA	TCCTAGGATCCTCACCTTCTGA
1 & 46	cgd2_1300	TCGGACTCTTCTCCAAACTCTC	CTGCGCTCCATAAATCACATAA
103 &	cgd1_3660	CATTTTCCAAGCTCATTCACAA	TTGGTTTCGATTCATACACTGC
103 &	cgd1_3680	AAACTCAACTTAGCATGGGACG	TCCATTTTGGCATGGATTATTT
103 &	cgd1_3690	TCTCAAGGGTTGAAGCAAGAAT	CAATAAGAATGGCATGGGACTT
103 &	cgd1_3700	TCATCTCGCTGTTGGAACTAGA	GTATGATCCCTTCCTCCTGTTG
103 &	cgd1_3710	TTTGGATGTGCTGGAGATATTG	TGGAGCAGCGTAAAAATGAGTA
103 &	cgd1_3720	TGTTTTTGGTTGCATTATCAGG	TAAGAATCGGGGTAAATGATGC
103 &	cgd1_3730	TACGACCAACCACATGCTTTAG	TCCATCAAAAGCTGAGCAAATA
103 &	cgd1_3740	GATGGAAGATGATGGAATGGTT	TCTGGGTGATTGTTTTTGATTG
104 &	cgd1_3760	TCTCTACCTTTGGACGTGGAAT	CCATATCATCGGCTCCTCTAAC
104 &	cgd1_3770	GTGAGTTCAGCAGGTACATTGG	ACTGGGTTGCTATTGATGTGAA
104 &	cgd1_3780	ATTTTGGGGTTTTTCCTCATTT	GATTCCGCACTATTATCCTTGG
104 &	cgd1_3800	GATGGCATGGAGAATTAGAAGC	TACAGGCGGCTTTTTCTGTAGT
2 & 46	cgd2_1400	TCGCAATTCAAGTCAGAGGTT	GTTCTGCAAGGTTGTTTGCTATT
2 & 46	cgd2_1480	TTCGACATTAGACTCGGTTAGGT	CCTGCTCCACTACAGCTATGAA
2 &47	cgd2_1600	CAGCTATGCAATGTTTGGAGAA	TGCTTGGATGATTTGATCTTTG
3 &47	cgd2_1650	AGCAACATTTACTAGCGACGG	GCTGTCATTCAGCTTTCTGTGT
3 &47	cgd2_1660	ACCGCAACTTAATGGAGAGTATG	GTCAGCAATAGGGTTTGAATGA
3 &47	cgd2_1960	CATGGTTCCAATCAAACTAGCA	TATTCACTCCAACTCCAGACGA
6 & 48	cgd2_2080	TCGAATGGTAAGTTTGGTAATAGGA	TGACTTCTTCTGAGCGAGACAA
