# Supplementary material for: The Cryptosporidium Parvum Transcriptome during In Vitro Development
Source: PLoS One. 2012 Mar 15;7(3):e31715. doi: 10.1371/journal.pone.0031715 (PMC3305300; doi:10.1371/journal.pone.0031715)
Supplement: Table S3 — C. parvum genes with >80% of total expression at a single time point. (PDF) [file pone.0031715.s006.pdf]

Supplemental Table 3. 107 Genes with a Peak Expression <80%

*For genes with differing annotations between cryptogenome.umn.edu (CG) and www.cryptodb.org (CDB) both are listed.

Peak hr	Gene ID	Annotation	Funtional Designation	Peak %	Peak magnitude
6	cgd1_780	hypothetical protein	hypothetical	83.85%	1.78E-04
6	cgd5_2640	hypothetical secreted protein, signal peptide	interacting	89.44%	2.76E-02
12	cgd1_1870	CG: possible domain similar to KOG4364, pFam02166, COG3064, KOG2897, KOG1029 CDB: hypothetical protein	apicomplexan	87.43%	2.53E-04
12	cgd6_4460	CG: protein kinase CDB: Ser/Thr protein kinase	apicomplexan	86.53%	2.28E-04
12	cgd1_140	CG: phosphoglucomutase [EC:5.4.2.2] CDB: phosphoglucomutase, tandemly duplicated gene	Cryptosporidium specific	86.11%	4.00E-06
12	cgd7_4020	CG: Protein kinase, CAMK/CAMK1 family regulated by Ca and calmodulin CDB: calcium/calmodulin dependent protein kinase with a kinase domain and 4 calmodulin like EF hands	Cryptosporidium specific	85.58%	3.97E-03
12	cgd1_110	CG: Possible Zn binding motif (Znf_RBZ), transcripts identified by EST CDB: little finger (CxxC...CxxC) domain containing protein	hypothetical	91.42%	1.43E-04
12	cgd1_130	hypothetical protein, Possible 2 transmembrane domains	hypothetical	81.90%	4.80E-03
12	cgd2_3310	predicted secreted protein, signal peptide, paralogs	hypothetical	91.30%	1.43E-05
12	cgd4_1020	hypothetical protein	hypothetical	93.84%	1.85E-04
12	cgd4_2340	CG: possible Ca binding protein, limited similarity to myosin tail CDB: myosin regulatory light chain	hypothetical	81.97%	3.00E-05
12	cgd5_1520	CG: myosin A tail domain interacting protein (MTIP), EF-hands CDB: EF-hands domain containing protein	hypothetical	83.54%	4.33E-05
12	cgd5_2740	hypothetical protein	hypothetical	84.59%	5.00E-05
12	cgd5_2760	CG: similar to hypothetical protein, signal peptide, transmembrane domain at C-terminus CDB: hypothetical protein possibly conserved in apicomplexa, signal peptide, transmembrane domain at C-terminus	hypothetical	87.89%	7.77E-05
12	cgd6_3130	CG: Signal peptide, Predicted membrane associated protein paralog of the Cryptosporidium-specific SKSR family CDB: conserved hypothetical protein	hypothetical	92.41%	2.67E-03
12	cgd6_3510	CG: Cryptopsoridial mucin, large thr stretch, signal peptide sequence, transcripts identified by EST CDB: cryptopsoridial mucin, large thr stretch, signal peptide sequence	hypothetical	91.97%	3.00E-05
12	cgd6_4000	hypothetical protein	hypothetical	81.63%	2.00E-06
12	cgd6_4950	CG: hypothetical protein, signal peptide, secreted CDB: conserved hypothetical protein	hypothetical	82.30%	7.00E-07
12	cgd7_1960	CG: hypothetical low complexity protein CDB: Low complexity protein with coiled coil regions	hypothetical	80.66%	1.02E-03
12	cgd7_2780	hypothetical protein	hypothetical	81.03%	1.07E-04
12	cgd7_3830	unconventional myosin	hypothetical	84.83%	9.00E-06
12	cgd7_4300	CG: similar to hypothetical protein, 3 transmembrane domain Membrane associated RING finger domain CDB: conserved 3 transmembrane domain membrane associated RING finger domain (shared by plants and apicomplexans)	hypothetical	84.43%	1.42E-02
12	cgd7_4720	hypothetical protein	hypothetical	85.23%	6.11E-04
12	cgd8_1130	signal peptide, predicted secreted protein with a cysteine cluster at the C-terminus	hypothetical	80.50%	5.20E-05
12	cgd8_3120	hypothetical protein having a signal peptide	hypothetical	85.22%	1.00E-05
12	cgd8_3830	uncharacterized low complexity protein	hypothetical	84.03%	3.00E-06

12	cgd7_4910	hypothetical protein	interacting	82.31%	9.01E-05
12	cgd2_3270	hypothetical protein	metabolism	99.61%	8.26E-04
12	cgd6_4160	hypothetical protein	other	89.67%	2.00E-05
12	cgd5_820	CG: highly similar to hypothetical protein from Plasmodium, large Low comp protein,armadillo repeat CDB: large protein with ARM repeats	phosphorylation	83.27%	1.00E-05
12	cgd7_1190	CG: hypothetical protein, signal peptide, WYLE family CDB: conserved hypothetical protein	phosphorylation	85.14%	2.00E-05
12	cgd1_3570	CG: transcripts identified by EST CDB: hypothetical protein	structural	90.36%	1.62E-03
12	cgd7_3790	hypothetical protein	structural	83.38%	3.22E-04
24	cgd3_2600	CG: domain similar to KOG2002, TPR-containing nuclear phosphoprotein that regulates K(+) uptake, Membrane protein with multiple cysteines in the N-terminus. No homologs in other organisms, signal peptide plus 4 transmembrane domains CDB: membrane protein with multiple cysteines at the N-terminus.	hypothetical	87.70%	8.41E-05
36	cgd7_4740	hypothetical protein	protein transport/modificati	83.54%	7.70E-05
48	cgd2_2510	CG: domainKOG0260, RNA polymeraseII, large subunit[Transcription], signal peptide,Transmembrane CDB: uncharacterized secreted protein with thr rich regions, possible mucin	conserved hypothetical	92.28%	7.92E-05
48	cgd8_4830	CG: possible domain similar to KOG0921, KOG1596, KOG3973, KOG3091, signal peptide, possible ER retention motif CDB: signal peptide, possible ER retention motif	conserved hypothetical	91.04%	1.23E-04
48	cgd2_790	CG: hypothetical protein, signal peptide, HCD family paralog CDB: conserved hypothetical protein	Cryptosporidium specific	85.54%	2.99E-05
48	cgd3_720	CG: domain similar to KOG1144, KOG1015, KOG1059, signal peptide CDB: hypothetical protein containing a signal peptide	Cryptosporidium specific	93.02%	4.00E-06
48	cgd4_1300	CG: translation initiation factor if-3 subunit 10 CDB: RRM domain and KH domain protein (SPAC30D11.14-like KH)	Cryptosporidium specific	98.88%	3.00E-07
48	cgd4_3090	CG: domain similar to COG3889, predicted solute binding protein, Signal peptide plus Thr stretch, possible mucin CDB: protein with signal peptide plus Thr stretch, possible mucin	Cryptosporidium specific	81.59%	7.00E-06
48	cgd5_1210	CG: Protein Kinase CAMK CAMK1 or CAMK3 group, Kinase regulated by Ca plus calmodulin CDB: calcium/calmodulin dependent protein kinase with a kinase domain and 4 calmodulin like EF hands	Cryptosporidium specific	85.91%	5.00E-05
48	cgd6_710	CG: similar to scavenger receptor protein TgSR1 precursor [Toxoplasma gondii] CDB: CpCCp3, multidomain extracellular protein with a signal peptide and the following architecture: LH2+LCCL+2xSR+LCCL+pentraxin+2xLCCL	Cryptosporidium specific	88.67%	4.00E-05
48	cgd8_1160	CG: Peptidase,insl domain, signal peptide CDB: secreted insulinase like peptidase, signal peptide	Cryptosporidium specific	94.07%	7.54E-05
48	cgd2_1230	hypothetical protein	hypothetical	86.36%	3.00E-07
48	cgd3_1370	signal peptide plus His and thr repeats, signal peptide plus transmembrane domain or GPI anchor, predicted mucin	hypothetical	82.08%	6.00E-06
48	cgd3_220	CG: transcripts identified by EST CDB: hypothetical protein	hypothetical	85.27%	2.00E-06
48	cgd3_2490	CG: probable mucin, very large 11700 aa thr repeat, signal peptide sequence CDB: very large probable mucin, 11700 aa long protein with signal peptide and pronounced Thr repeat (308 aa long)	hypothetical	84.77%	1.39E-04
48	cgd3_3450	CG: similar to glucose-methanol-choline (GMC) oxidoreductase family CDB: conserved hypothetical protein	hypothetical	92.27%	3.00E-05

48	cgd4_2410	hypothetical protein	hypothetical	89.09%	8.00E-06
48	cgd4_3700	hypothetical protein	hypothetical	86.43%	6.05E-05
48	cgd5_3810	hypothetical protein with a signal peptide	hypothetical	86.67%	2.73E-04
48	cgd5_80	hypothetical protein	hypothetical	93.32%	6.00E-07
48	cgd6_2680	hypothetical protein	hypothetical	81.57%	3.00E-06
48	cgd6_3060	hypothetical protein with Thr stretches, possible mucin	hypothetical	88.15%	3.00E-05
48	cgd6_5340	CG: domainKOG4372,Predicted alpha/beta hydrolase, possible transmembrane domain near N CDB: conserved hypothetical protein	hypothetical	95.47%	1.16E-04
48	cgd6_770	hypothetical protein	hypothetical	81.16%	3.04E-05
48	cgd7_1290	CG: Protein kinase CDB: Ser/Thr protein kinase	hypothetical	91.61%	2.95E-03
48	cgd7_4680	hypothetical protein	hypothetical	89.64%	2.14E-03
48	cgd8_1750	CG: similar to NLI interacting factor CDB: possible NLI interacting factor CTD-like phosphatase	hypothetical	81.63%	2.00E-05
48	cgd8_2680	hypothetical protein, transmembrane domain or GPI anchor at C-terminus	hypothetical	80.52%	2.00E-06
48	cgd8_5330	hypothetical protein	hypothetical	86.96%	1.00E-05
48	cgd4_1910	CG: hypothetical protein, signal peptide, secreted CDB: hypothetical secreted protein	other	85.77%	9.12E-04
48	cgd2_1300	Rab GDP dissociation inhibitor	phosphorylation	86.67%	1.00E-06
48	cgd4_3240	hypothetical protein	phosphorylation	84.00%	1.79E-04
48	cgd8_5320	Cryptosporidium oocyst wall protein 6, CpCOWP6	phosphorylation	84.99%	1.00E-06
48	cgd3_4200	hypothetical protein	protease related	84.62%	2.00E-07
48	cgd4_130	CG: similar to hypothetical protein, with proteinase inhibitor Kazal domain CDB: extracellular protein with a signal peptide, kazal domain and mucin like low complexity repeats	translation	89.76%	2.00E-05
72	cgd2_3810	CG: similar to uncharacterized expressed protein, contains 3 CCCH like RNA binding domain involved in RNA Metabolism CDB: F11M21.28-like protein with 3 CCCH RNA binding domains involved in RNA metabolism	apicomplexan	79.90%	4.00E-07
72	cgd2_2920	CG: hypothetical protein CDB: [not in database]	conserved hypothetical	89.69%	1.00E-06
72	cgd2_3600	ARF GAP-like zinc finger-containing protein	conserved hypothetical	93.52%	8.00E-06
72	cgd2_4050	CG: similar to er lumen protein retaining receptor, 8 transmembrane domain protein CDB: conserved hypothetical protein	conserved hypothetical	80.52%	8.94E-05
72	cgd3_1540	CG: cyclinA CDB: cyclin	Cryptosporidium specific	90.89%	1.00E-05
72	cgd4_500	hypothetical protein	Cryptosporidium specific	87.75%	3.00E-06
72	cgd7_1690	hypothetical protein	DNA-associated	82.46%	3.00E-05
72	cgd2_1380	2x WD domain containing protein	hypothetical	81.13%	4.00E-07
72	cgd2_2100	CG: similar to crm1 protein CDB: conserved hypothetical protein	hypothetical	91.55%	1.00E-06
72	cgd2_2110	DnaJ domain, possible zf-CSL following	hypothetical	98.41%	2.00E-05
72	cgd2_3620	CG: domain similar to Tryp_mucin, Mucin-like glycoprotein, threonin stretch at COOH region CDB: large protein with signal peptide. cysteine-rich, threonine-rich, possible mucin	hypothetical	91.11%	2.06E-04
72	cgd3_1520	3x TPR domain-containing protein	hypothetical	90.48%	1.17E-06
72	cgd3_190	Cryptosporidium oocyst wall protein 7,CpCOWP7	hypothetical	92.94%	4.00E-06
72	cgd4_1050	hypothetical protein	hypothetical	93.06%	5.00E-09

72	cgd4_1310	CG: possible domain similar to Podocalyxin, COG3889, KOG4701, all secreted protein domains CDB: uncharacterized protein with threonine repeats	hypothetical	98.69%	8.00E-08
72	cgd4_4260	CG: subtilisin-like protease CDB: serine protease, subtilase family, signal peptide, putative	hypothetical	82.12%	2.00E-07
72	cgd4_680	hypothetical protein	hypothetical	82.41%	5.00E-06
72	cgd4_730	hypothetical protein	hypothetical	82.10%	5.36E-08
72	cgd5_2660	CG: DMC1 CDB: meiotic recombination protein DMC1-like protein	hypothetical	85.46%	7.53E-05
72	cgd6_3200	CG: Hypothetical protein with possible central bZIP transcription factor domain CDB: putative transcription factor with central bZIP transcription factor domain	hypothetical	81.55%	5.07E-04
72	cgd6_4800	hypothetical protein	hypothetical	84.01%	2.00E-06
72	cgd6_5050	hypothetical protein	hypothetical	87.25%	1.00E-06
72	cgd6_5170	CG: domain similar to KOG0161, Smc, KOG0612 CDB: hypothetical protein	hypothetical	80.71%	5.00E-06
72	cgd6_5230	PHD finger containing protein	hypothetical	81.45%	5.28E-06
72	cgd6_740	CG: similar to leucine, glutamic acid, lysine family 1 protein CDB: hypothetical protein	hypothetical	82.45%	5.23E-04
72	cgd7_4700	hypothetical protein	hypothetical	87.27%	1.48E-04
72	cgd8_1000	CG: pyruvate decarboxylase [EC:4.1.1.1] CDB: pyruvate decarboxylase	hypothetical	82.75%	3.73E-04
72	cgd8_1220	conserved hypothetical protein, signal peptide, 2-3 transmembrane domain at N-terminus	hypothetical	85.64%	2.00E-06
72	cgd8_5080	peptide synthase like condensation domain	hypothetical	85.83%	4.24E-05
72	cgd1_1690	hypothetical protein with transmembrane domain	interacting	81.81%	1.00E-07
72	cgd2_3240	CG: mucin like protein CDB: latent transforming growth factor beta binding protein like (3F865)	interacting	91.12%	3.00E-06
72	cgd2_3610	hypothetical protein	interacting	95.57%	7.00E-07
72	cgd3_930	CG: hypothetical protein, limited similarity to PF07_0076 pfal CDB: hypothetical protein	interacting	93.40%	3.00E-05
72	cgd7_3120	hypothetical protein	metabolism	80.64%	4.07E-03
72	cgd1_3120	hypothetical protein, possible signal peptide	other	79.95%	8.00E-07
72	cgd2_3660	hypothetical protein with transmembrane or GPI anchor sequence at carboxy terminus	protease related	85.58%	6.00E-08
72	cgd2_3250	hypothetical protein, possible signal peptide	protein transport/modificati	89.29%	2.00E-06
72	cgd3_4050	ring finger domain protein and 2 transmembrane domains	protein transport/modificati	88.05%	1.00E-06
72	cgd8_780	CG: similar to Plasma membrane Sodium Response 2; Psr2p, NLI-interacting factor domain CDB: hypothetical protein	RNA-associated	90.25%	5.00E-07
